# The Dual Prey-Inactivation Strategy of Spiders—In-Depth Venomic Analysis of *Cupiennius salei*

**DOI:** 10.3390/toxins11030167

**Published:** 2019-03-19

**Authors:** Lucia Kuhn-Nentwig, Nicolas Langenegger, Manfred Heller, Dominique Koua, Wolfgang Nentwig

**Affiliations:** 1 Institute of Ecology and Evolution, University of Bern, Baltzerstrasse 6, CH-3012 Bern, Switzerland; nicolas.langenegger@iee.unibe.ch (N.L.); dominique.koua@iee.unibe.ch (D.K.); wolfgang.nentwig@iee.unibe.ch (W.N.); 2 Proteomics and Mass Spectrometry Core Facility, Department for BioMedical Research (DBMR), University of Bern, Freiburgstrasse 15, CH-3010 Bern, Switzerland; manfred.heller@dbmr.unibe.ch; 3 Institut National Polytechnique Félix Houphouet-Boigny, BP 1093 Yamoussoukro, Cote d’Ivoire

**Keywords:** in depth transcriptomics, proteomics, venom, enzymes, neurotoxins, α-amylase

## Abstract

Most knowledge of spider venom concerns neurotoxins acting on ion channels, whereas proteins and their significance for the envenomation process are neglected. The here presented comprehensive analysis of the venom gland transcriptome and proteome of *Cupiennius salei* focusses on proteins and cysteine-containing peptides and offers new insight into the structure and function of spider venom, here described as the dual prey-inactivation strategy. After venom injection, many enzymes and proteins, dominated by α-amylase, angiotensin-converting enzyme, and cysteine-rich secretory proteins, interact with main metabolic pathways, leading to a major disturbance of the cellular homeostasis. Hyaluronidase and cytolytic peptides destroy tissue and membranes, thus supporting the spread of other venom compounds. We detected 81 transcripts of neurotoxins from 13 peptide families, whereof two families comprise 93.7% of all cysteine-containing peptides. This raises the question of the importance of the other low-expressed peptide families. The identification of a venom gland-specific defensin-like peptide and an aga-toxin-like peptide in the hemocytes offers an important clue on the recruitment and neofunctionalization of body proteins and peptides as the origin of toxins.

## 1. Introduction

With more than 47,700 species [1], spiders are the most species-rich terrestrial invertebrate group after insects. They occur in all ecosystems, often in high densities. As general or specialized predators, spiders primarily prey on arthropods, thus contributing to the important regulation of other species, e.g., pest insects in agroecosystems [2]. Spider venoms provide a huge variety of venomous compounds to subdue their prey and to defend against aggressors [3], thus representing an important source for the development of human therapeutics and pest control [4,5]. In the last few years, the appearance of affordable transcriptome analyses and mass spectrometric proteome analyses [6,7,8] dramatically accelerated venom research, which has, for many years, mainly been based on the identification and isolation of single venom components. Thereby, most publications mainly investigated only small organic compounds, neurotoxins, and their interaction with a variety of ion channels and other targets. Other venom components, such as cytolytic peptides or proteins [3] were either only reported to occur in few spider venoms or have, with a few exceptions, not been as deeply investigated.

The role of enzymes in spider venom was controversially discussed, and only recently hyaluronidase was found to have an important role in supporting the venomous effect in most spider families [9]. Another enzyme, phospholipase D, has been well investigated due to its severe effects on humans. The presence of this cell-membrane destroying enzyme, however, is restricted to species of the sicariid family [10]. Modern transcriptomic and proteomic techniques allow the efficient analysis of all amino acid-based venom components. Analyses of the majority of spider venom transcriptomes are focused on cysteine-containing, putatively neurotoxic-acting peptides [7,8] and they rarely cover proteins and enzymes [11].

In this study, we analyze the venom gland transcriptome and proteome of *Cupiennius salei* [12,13], one of the best-investigated spider species [14]. With a holistic view on the transcriptomic data and our long-term experience in venom research, we searched for peptides and proteins influencing the homeostasis of the prey and/or aggressor, as well as for recruited compounds so far not identified in the venom gland. We provide evidence that the venom of *C. salei* interacts with many regulatory and metabolic pathways, types of tissue, and specific receptors. This disturbs the homeostasis of the targeted organism in many ways, usually leading to its death or to non-lethal effects. The present in-depth analysis provides a new understanding of spider venom functionality, presented here as the dual prey-inactivation strategy.

## 2. Results and Discussion

### 2.1. Overview of Venom Gland Composition

**Figure 1:**
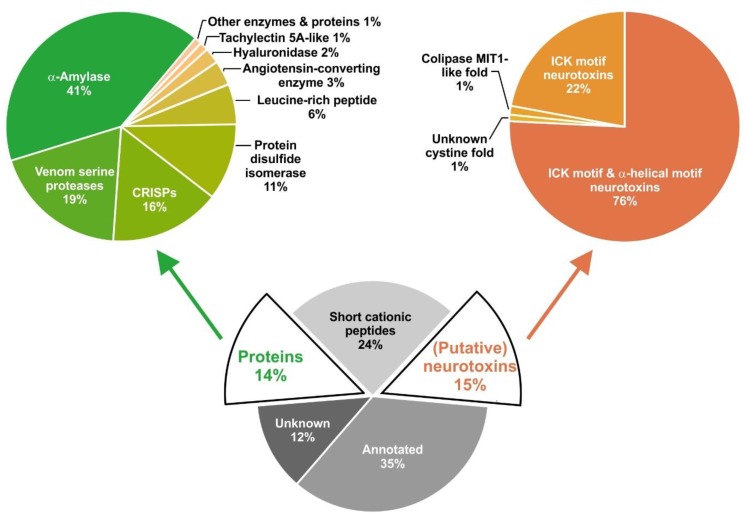
Functional profile of venom gland-specific transcripts of proteins and (putative) neurotoxins of *C. salei*. Contigs were blasted against UniProt KB (E = 10^−5^) and searched against Pfam-A.hmm [16]. The relative abundancy of functional groups is given in % of normalized read counts per contig (TPM), and specified for proteins (green) and cysteine-containing (putative) neurotoxins (orange). Proteins and neurotoxin-like structures are annotated based on their similarity to known proteins or structural motifs (α-helical motif, inhibitor cystine knot motif (ICK), and colipase MIT1-like fold).

The annotation of the *C. salei* venom gland transcriptome by 454-sequencing resulted in 34,107 contigs as described earlier [15]. In-depth transcriptomic data analysis is supported by top-down and bottom-up proteomics of venom and by data from previous work [12]. Of all contigs, 38.2% refer to venom gland-specific peptides and proteins, an additional 39.4% were identified as annotated sequences, and 22.4% could not be annotated. However, summing up normalized read counts per contig (TPM) showed that 53% of all expressed sequences belong to venom gland-specific peptides and proteins, an additional 35% to annotated sequences, and only 12% to unknown sequences. All venom gland-specific peptides and proteins were manually annotated and divided into three functional groups: proteins (14%), cysteine-containing (putative) neurotoxic peptides (15%), and short cationic peptides (24%, not further analyzed here) (Figure 1).

### 2.2. Most Abundant Proteins in the Venom Gland Transcriptome and in the Venom of C. salei

Venom gland proteins were grouped into three functional categories: (1) proteins involved in the protein and peptide-processing machinery, (2) proteins possibly recruited and neofunctionalized in the venom gland, and (3) proteins with putative functions in the innate immune system of the spider. Eighteen out of nineteen identified protein groups exhibit a signal peptide showing that these proteins are synthesized in the endoplasmic reticulum (ER), and may act as enzymes within the ER or are synthesized for the excretion process in the venom gland [17]. Five of these proteins are enzymes, belonging to the protein-/peptide-processing machinery. These are a signal peptidase (SP), specific serine proteases (VSP), protein disulfide isomerases (PDI), carboxypeptidase (CPA), and peptidylglycine α-amidating monooxygenase (PAM), together representing 30.0% of all expressed venom gland-specific protein transcripts (Figure 1). Several protein groups are thought to be recruited and neofunctionalized: amylases (α-AMY, 40.9%), cysteine-rich secretory proteins (CRISPs, 15.7%), angiotensin-converting enzyme (ACE, 3.4%), hyaluronidase (HYAL, 2.2%), cystatin (CST, 0.4%), thyroglobulin type-1-like protein (TT1LP, 0.3%), insulin-like growth factor-binding protein-related protein 1 (IGFBP-rP1, 0.1%) [18], Kunitz domain-containing protein (KCP, 0.06%), and phospholipase (PLA2, 0.04%). Immune-relevant proteins might be tachylectin 5A (TL5A, 1.2%), and leucine-rich repeat protein (5.7%) (Table 1).

### 2.3. Proteins of the Protein- and Peptide-Processing Machinery

#### 2.3.1. Signal Peptidase (SPase)

Removal of the signal peptide of protein and peptide precursors by SPase is the initial step in the translocation of excretory and secretory proteins across the ER membrane [19]. Although the identified SPase is a housekeeping enzyme complex, the enzyme is crucial for the processing of venom gland-specific toxic proteins and peptides. So far, no information is available about protein structure and subunit composition of arachnid SPase. The catalytic subunit belongs to the serine endoprotease S26B (S26.023) family (IPR001733). Active site residues Ser 54 and His 94 could be identified when compared with arthropod peptidase subunit SEC11 (*Drosophila melanogaster* sp_Q3YMT4) (http://merops.sanger.ac.uk) [20]. The N-terminal region exhibits a cytoplasmic domain (1–19 aa). The protein is composed of 177 aa (20 kDa) and shows a high positive charge (pI of 9.21). The *C. salei* SPase sequence is highly identical to other known spider SPases (identities > 95.4%). A remarkably high sequence identity of 91.5% was calculated between the *C. salei* SPase and the horseshoe crab (*Limulus polyphemus*) enzyme, and 78.5% identity is shared with the above-mentioned arthropod peptidase of *D. melanogaster* (Table 1, Supplementary Figure S1.1).

#### 2.3.2. Protein Disulfide-Isomerase (PDI)

This enzyme, located in the ER, catalyzes the formation and breakage of disulfide bonds during the folding of proteins and peptides. The PDI may be involved in the folding of neurotoxin precursors [21] (Figure 1, Table 1). PDI was identified based on similarities with sequences from *L. polyphemus* (68.3% identity) and the mite *Tetranychus urticae* (70.5% identity). The two mature forms of PDI (PDI_1a/1b and PDI_2) from *C. salei* differ by eleven mutations in a restricted area of the C-terminus, resulting in 97.8% identity between both enzymes. These enzymes (IPR005792) exhibit detailed signature matches as the thioredoxin-like fold (IPR012336), the thioredoxin domain (IPR013766), and the disulfide isomerase domain (IPR005788) with the redox-active disulphide region motif “APWCGHCK” in its N-terminal, as well as in its C-terminal part (amino acid residues: 48–55 and 389–396).

So far, no sequence data for PDI identified from other spider venom gland transcriptomes are available. In our venom gland transcriptome of *Alopecosa marikovskyi* [22] we identified a corresponding sequence with 94.9% identity to PDI_1ab, and in the venom gland of *Viridasius fasciatus* we found a protein with an identity of 91.9% toward PDI_2. This points toward a strongly conserved enzyme, which is most probably essential for the proper folding of cysteine-rich venom peptides (Supplementary Figure S1.2).

#### 2.3.3. Venom Serine Proteases (VSPs)

Most biologically active spider venom peptides comprise a pro-peptide that is removed during the maturation process. Different amino acid motifs present at the C-terminal end of the pro-peptide have been described to serve as protease recognition sites for pro-peptide removal. Among them, the dibasic motif (KR, RR, KK, or RK) [23], and the processing quadruplet motif (PQM; XXXR, where at least one X = Glu) are the most common motifs. An additional cleavage motif, the inverted PQM (iPQM; RXXX, where at least one X = Glu), has been described to occur together with the PQM at the predicted cleavage sites of heterodimeric neurotoxins and cytolytic peptide precursors [24].

Recently, we purified the venom gland-specific serine protease VSP1 that specifically cleaves PQMs. According to its target motif, we named this enzyme PQM protease [25]. The active PQM protease exhibits a heterodimeric structure and is responsible for the specific cleavage of the pro-peptide to activate mature peptide toxins in the venom. Moreover, this type of protease is involved in the heterodimerization process of neurotoxins [26] by the cleavage of PQMs and inverted PQMs [12]. Overall, two groups of isoform (VSP1_a1,2 and VSP1_b1,2) have been identified with high-sequence identities of 98.9% between VSP1_a isoforms, and of 99.6% between VSP1_b isoforms. VSP1_a isoforms exhibit one silent mutation in the nucleotide sequence (G576T), and three mutations in the amino acid sequence (F98Y, I100L, and D176N). Sequence identities between 93.3% and 94.3% were calculated for VSP1_a and VSP1_b isoforms. VSP1_b isoforms differ from VSP1_a in an additional Asn residue at position 205, and 16 further non-silent mutations. Besides two silent mutations, the most remarkable difference between the VSP1_b isoforms is a point mutation resulting in amino acid exchange C219R, because the mutation affects the disulfide bond C5-C6 [25]. This may influence the three-dimensional structure and the substrate specificity of the protease. The two VSP1s isoforms mentioned here exhibit identical signal peptides and light chains, and seem to be a product of gene duplication (Table 1, Supplementary Figure S1.3). The different isoforms might be an adaptation to variations in the cleavage motifs of neurotoxins and cytolytic peptide precursors. We identified multiple venom peptide precursors with predicted cleavage motifs featuring an Arg residue at position P1, but no Glu residue in position 1, 2, and 3 after/before an Arg residue (Kuhn-Nentwig, unpublished results). These cleavage motifs are not classical PQM/iPQM and do not follow the Glu to Arg or/and Glu after Arg-processing rules [24].

Another VSP2 (Table 1, Supplementary Figure S1.4) is less expressed in the venom glands, but also belongs to the peptidase S1A chymotrypsin family (IPR001314) with the typical serine protease, trypsin domain (IPR001254, ~aa 43–285). The active site is the typical catalytic triad of His97, Asp144, and Ser237. Additionally, amino acid residues Asp231, Ser256, and Gly258 are supposed to be involved in substrate binding. A cleavage site before Val44 is annotated (cd00190 Tryp_SPc). This cleavage site supports our hypothesis that VSP2 might be activated by cleavage in the N-terminal part, which then leads to its heterodimeric structure, comparable to VSP1. Here, the hypothesized light chain (aa 17–43) might also be connected to the heavy chain by a disulfide bridge, as verified for VSP1. For VSP2, we identified three transcript variants with the following silent mutations: G414A, C543T, A654G, C882T, and G888A. Alignment of VSP1 and VSP2 resulted in only 29.3% identity. High identities of 56.9% to 77.6% were found in the MSA of the VSP2 of different spider species (Supplementary Figure S1.4). The isoelectric point of VSP2 is two units higher than the one of VSP1, which might be indicative of a different pH optimum of the two enzymes. In contrast to all other venom gland-specific proteins here described, VSP1 and VSP2 are characterized by a twofold stop signal. VSP1 shows identities of 59%–98% to putative protease homologs from 18 spider species of nine families from the retrolateral tibial apophysis (RTA) clade, as recently reported [25].

Besides their involvement in the maturation process of venom peptides and proteins, VSPs may also cleave unknown targets in the prey. However, VSPs are thought to have high substrate specificity, as protease activity tests with the unspecific protease substrate Azocoll^TM^ only showed very low proteolytic venom activity after 24 h of incubation [27].

#### 2.3.4. Carboxypeptidase A-Like Protein (CPA)

Within the maturation process of precursors, the next step requires a carboxypeptidase for the removal of the C-terminal Arg in heterodimeric neurotoxins and the C-terminus of cytolytic peptide precursors [25,26,28]. We identified a peptidase M14, metallo carboxypeptidase A-like protein (IPR000834) with the typical Zn^2+^ binding site motif HXXE + H (His68, Glu71, His175), and the M14_CP_N/E_like active site (cd03858) (Table 1, Supplementary Figure S1.5). This enzyme may be responsible for removal of C-terminal Arg-residues from immature venom peptides. The enzyme exhibits high identities towards CPA proteins identified in the venom gland of *A. marikovskyi* (82.1%), *V. fasciatus* (79.0%), and *Stegodyphus mimosarum* (65.1% genomic DNA).

#### 2.3.5. Peptidylglycine α-Amidating Monooxygenase (PAM)

Besides further post-translational modifications, C-terminal amidation of the short chain of VSP1 as well as of some neurotoxins and cytolytic peptides in the venom of *C. salei* is the final step in protein and peptide maturation. PAMs are proteins with two different enzymatic activities. First, the production of a peptidyl-α-hydroxyglycine intermediate is catalyzed by the peptidylglycine α-hydroxylating monooxygenase (PHM), followed by the formation of the α-amidated peptide by the peptidyl-α-hydroxyglycine α-amidating lyase (PAL) [29]. Transcriptome data analysis revealed C-terminal Gly-residues as a prerequisite for the amidation of the short chain of VSP1 and diverse neurotoxins and cytolytic peptides. Amidation of these peptides was confirmed by proteome analysis [12].

The PAM exhibits the copper type II ascorbate-dependent monooxygenase N-terminal (IPR000323) and C-terminal (IPR024548) domains with the histidine cluster 2 H-x-F-x(4)-H-T-H-x(2)-G. MSA with homologs of other spiders shows partially high-sequence identities with the enzyme from *A. marikovskyi* (90.9%), *V. fasciatus* (83.8%), and *Parasteatoda tepidariorum* (72.7%). Lower identities were obtained with a scorpion (*Tityus obscurus*, 64.1%) and a horseshoe crab (*L. polyphemus*, 61.2%) (Table 1, Supplementary Figure S1.6).

### 2.4. Recruited and Neofunctionalized Proteins

Recruitment of genes in the venom glands and neofunctionalization after gene duplication is thought to be the origin of venomous peptides and proteins in venom glands of not only arthropods but also other animals. Importantly, these proteins often participate in key regulatory processes in animals [30,31,32].

#### 2.4.1. α-Amylase (α-AMY)

α-amylases are widely distributed in bacteria, archaea, fungi, and plants [33,34]. Moreover, many invertebrates and vertebrates express theses enzymes in their salivary glands and in the digestive liquid [35,36]. In spiders, α-amylases have been detected in the digestive liquid of *C. salei*, *Eratigena atrica* [37], and *Nephilingis cruentata* [38]. The enzymes act on the α (1 → 4) glycosidic linkages in starch and glycogen and belong to Family 13 of the glycosyl hydrolases. To the best of our knowledge, this is the first time that α-amylase is described as a spider venom gland component. Estimated from transcript abundance, α-AMY is one of the most abundant protein groups identified in the transcriptome of *C. salei*, accounting for 41% of all venom gland-specific proteins (Figure 1, Table 1).

Besides one main sequence encoding the α-AMY precursor, we identified different fragments with non-silent and silent mutations that may point to different mature isoforms. Two mutations were detected in the signal peptide (G110V and S12C) and five in the mature protein (P66S, N204I, I206L, R401K, and A511V). The protein exhibited a high molecular mass of 57 kDa, and the putative isoforms did not vary in length. Ca^2+^-binding sites and catalytic amino acid residues are shown in the supporting material (Supplementary Figure S1.7). Unfortunately, no amino acid sequence data from the α-AMY from the digestive liquid of *C. salei* is available but there is a 75.2% identity with the α-AMY from the digestive liquid of *Nephilingis cruentata*. Venom α-AMYs are possibly widespread within araneomorph spiders, e.g., in *A. marikovskyi* (78.5% identity) and *V. fasciatus* (88% identity). Interestingly, we could identify an α-AMY precursor in the venom gland transcriptome of *Atypus piceus* (69.1% identity), one the most ancient mygalomorph spiders.

What could be the reason for the neofunctionalization of amylases in spider venom? Glycogen is identified in arthropods (and most animals) as a carbohydrate storage form [39,40]. In insects, glycogen is present in the hemolymph, fat body and gut tissue, but also in smaller amounts, in the muscles [41]. Controlled glycogen degradation in insects is mediated by glycogen phosphorylase. This enzyme has to be activated by the hypertrehalosemic hormone AKH/HTH, Ca^2+^, and cAMP, resulting in the release of glucose-1-phosphate, thus avoiding free glucose [42,43,44]. Furthermore, this pathway is coupled with the insulin-signaling system, one of the highest conserved endocrine systems in invertebrates and vertebrates. In some decapods and arthropods, insulin-like proteins have been described instead of insulin, where the mature proteins are characterized as two-chain proteins, comparable to mature insulin [42,45]. Besides glycogen, the main metabolic carbohydrate storage in insects is the disaccharide trehalose [41]. Glucose is only in the order of 1/100 of trehalose available in the hemolymph, and it is hypothesized that the nonreducing trehalose is favored over the reducing glucose, which might prevent undesirable effects in the hemolymph [46]. Injected into an insect, α–amylases may release immediately high amounts of glucose from glycogen in the hemolymph and muscles, which could end in decoupled energy homeostasis, changes in osmotic conditions, and other symptoms of hyperglycemia with a potentially fatal outcome.

#### 2.4.2. Cysteine–Rich Secretory Proteins (CRISPs)

The second frequently recruited protein group identified in the transcriptome of *C. salei* consists of CRISPs (15.7%) belonging to the CAP superfamily (cysteine-rich secretory proteins, antigen 5, and pathogenesis-related 1 protein), and comprises CRISP1 (14.2%) and CRISP2 (1.5%). We identified three transcripts of CRISP1, resulting in two protein isoforms. They differ in positions F52Y, A205T, and S351N. Additionally, three silent mutations were identified in the nucleotide sequences at positions G45A, A267G, and A161G. An InterPro scan revealed a CAP domain-like structure (IPR014044) in the N-terminal part of the precursor proteins (aa: 60–222). Furthermore, the following domains, related to cysteine-rich secretory proteins, were identified: allergen V5/Tpx-like (IPR001283; PR00837), and V5 allergen (IPR002413; PR00838) (aa: 52–68; 112–128; 173–192). Comparable to CRISP1, the precursor protein of CRISP2 shows the CAP domain-like structure (IPR014044, aa: 56–217), four detailed signature matches (aa: 89–107; 146–159, 174–190, 204–217) related to allergen V5/Tpx-1(IPR001283; PR00837) and three signature matches related to V5 allergen (IPR002413; PR00838) (aa: 52–68; 112–128; 173–192) (Supplementary Figure S1.8). CRISP1 and CRISP2 only share 31.1% sequence identity, possibly reflecting different protein subfamilies and functions.

The MSA of CRISP1 with CRISPs of other spiders revealed 54.7% identity with venom allergen 5 from *Lycosa singoriensis*, 51.8% with CRISP1 from *V. fasciatus*, and 37.4% identity with two further CRISPs identified in the spider *S. mimosarum* (Supplementary Figure S1.8). An MSA of CRISP2 and arthropod venomous CRISPs resulted in identities of 70.1% with the CRISP2 of *V. fasciatus*, 55.0% with a CRISP of *S. mimosarum*, and 44.7% to 46.4% with three mygalomorph spiders: *Hadronyche infensa*, *Grammostola rosa*, and *Trittame loki* (Supplementary Figure S1.9).

The MSA of CRISP1 and CRISP2 with scorpions *T. obscurus*, *Tityus bahiensis*, *Lychas buchari*, and *Isometroides vescus* resulted in identities between 30.7% and 32.6% for CRISP1 and between 39.4% and 41.9% for CRISP2. MSA with ticks showed identities between 27.8% and 29.2% for CRISP1 and between 30.1% and 35.5% for CRISP2. Comparably, lower identities were found between CRISP1 (26.6% to 30.5%) and CRISP2 (32.4% to 33.3%) with different CRISPs (e.g., venom allergens 5), identified in the venom glands of stinging insects such as wasps and fire ants (Figure 1, Table 1) (Supplementary Figure S1.10).

In contrast with venom allergen-5, first reported in wasp ([P35786], 23.9 kDa) and fire ant venoms ([P35778], 26.4 kDa), *C. salei* CRISP1 and CRISP2 possess a C-terminal extension leading to molecular masses twice as high. This C-terminal extension of CRISP1 and CRISP2 might be arachnid-specific as we also identified it in comparable cysteine-rich venom proteins from other spiders, scorpions, and from the salivary glands of ticks. Due to this important C-terminal difference between insect and arachnid CRISPs, we generally propose to name such peptides, identified in arachnid venoms, CRISPs rather than venom allergens. In the venom of cone snails, comparable cysteine-rich proteins exhibiting the CAP domain were also identified: Tex31 ([Q7YT83], *Conus textile*, 31.5 kDa) a calcium-dependent substrate specific protease, MR30 ([A1BQQ5], *Conus marmoreus*, 30.1 kDa), and GlaCrisp [DQ647193.1]. So far, the substrate-specific proteolytic activity of Tex31 [47] could not be verified in further studies [48,49]. More recently, functional studies of antigen-5/CAP family members, identified in the salivary glands of blood-sucking insects, revealed that these proteins are Cu^2+^-dependent antioxidant enzymes involved in the removal of radical superoxide and inhibit platelet aggregation by collagen and neutrophil oxidative burst [50].

#### 2.4.3. Angiotensin-Converting Enzyme (ACE)

ACE-like enzymes are known from the venom gland transcriptome of *Phoneutria nigriventer* [11] and of scorpions [51,52], but also from the salivary glands of hematophagous insects [53]. Moreover, ACE plays a crucial role in the regulation of peptide hormones present in various types of insect tissue [54]. We identified at least four ACE isoforms, featuring the following amino acid polymorphisms: A10T (signal peptide) and L315I (mature protein). ACE belongs to the protein domain family of peptidyl-dipeptidase A, M2 metalloprotease group (IPR001548). The metal-binding domain might be formed by the HEXXH +E motif. The ACE identities between different spiders vary between 83.6% (*C. salei*/*A. marikovskyi*), 74.6% (*V. fasciatus*), 63.8% (*S. mimosarum*) and 64.9% (*P. tepidariorum*). Remarkably, an ACE-like peptidase purified from the venom of the *T. serrulatus* scorpion converted angiotensin I into angiotensin II. The crude venom of this scorpion hydrolyzed natural substrates such as angiotensin I, bradykinin, and hemopressin [51]. Insect ACE is likewise able to convert angiotensin I into angiotensin II, and to remove Phe-Arg from the C-terminus of bradykinin in vitro, both vertebrate substrates that have not yet been identified in insects.

So far, no information on whether this enzyme is involved into venomous peptide processing and modification, or if it acts after injecting venom into the prey’s body in a toxic or synergistic manner, e.g., by destabilization of the target homeostasis through hypertensive effects, is available. The latter could be mediated by C-terminal hydrolyzation of regulatory peptide hormones in arthropods (e.g., tachykinin-related insect peptides and, adipokinetic hormone (AKH) peptides) and in small vertebrates (e.g., angiotensin I, bradykinin, hemopressin) [51,55]. AKH and tachykinin-related insect hormone families are known to be targets for insect ACE [54]. The MSA of spider venom ACE and scorpion venom ACE-like peptidases show identities between 55.4% and 59.2% and even more interestingly, 55.5% identity toward insect ACE (Supplementary Figure S1.11). It is tempting to speculate that spider venom ACE may act on comparable ACE substrates in prey with dramatic physiological effects.

#### 2.4.4. Hyaluronidase (HYAL)

The structure, N-glycosylation, and function of the hyaluronidase-like enzyme (IPR018155) in the venom of *C. salei* have recently been published by our group [9]. Interestingly, most but not all investigated spider species exhibit enzymatic activity in their venom, which degrades hyaluronan and chondroitin-4-sulfate in the prey’s tissue, thus acting as a spreading factor for venomous compounds. HYAL sequences of other RTA clade spiders show identities between 77.3% (*V. fasciatus*) and 79.9% (*A. marikovskyi*) with the *C. salei* HYAL. Mygalomorph spider HYALs are 49.7% (*Brachypelma vagans*) to 50.4% (*H. infensa*) identical (Supplementary Figure S1.12).

#### 2.4.5. Phospholipase A2 (PLA2)

PLA2 activity was identified in the venom of different spider families, such as eresids, miturgids, lycosids, and hexathelids, indicating a widespread existence of the enzyme [3]. In opposition to honeybee and scorpion venom phospholipase A2 [56,57,58], PLA2 sequence data and investigations concerning its mode of action are very limited for spiders. PLA2 hydrolyzes the sn-2 acyl ester bond of phospholipids in a Ca2^+^ -dependent reaction. The hydrolyzation products are lysophospholipids and free fatty acids. PLA2s may have a heterodimeric structure, as reported for some scorpion PLA2s, in which a small subunit is linked by a disulfide bridge to the main protein. However, honeybee PLA2 is composed of two subunits that are not covalently linked [57,58].

PLA2 is much lower expressed in the *C. salei* venom gland compared to most other venom proteins (Table 1), which begs the question if it functions as a house-keeping or venomous enzyme. InterPro sequence classification resulted in the identification of the protein as a member of the phospholipase A2 family (IPR001211), belonging to the cl05417 superfamily, subfamily cd00125. Honeybee PLA2 belongs to the same superfamily, but to the cd 4704 subfamily. The MSA of *C. salei* PLA2_CUPSA with predicted genomic and transcriptomic PLA2 sequences from other spiders shows identities between 88.8% (*A. marikovskyi*), 63.0% (*S. mimosarum*), and 58.0% (*V. fasciatus*). The low identity of 37.4% with *P. tepidariorum* points to the problem of differentiating between PLA2 as toxic component in the venom and PLA2 sequences, which are part of the housekeeping genes. The pro-peptide structure of honeybee PLA2 and the N-terminal amino acid residues of spider PLA2 feature a possible cleavage site for a specific protease. Cleavage may result in a heterodimeric structure, comparable to the one of the recently published PQM-protease of *C. salei* (Supplementary Figure S1.13). The biological activities of venomous PLA2s are mainly known from snake PLA2s, with a broad scale of effects, such as hemolytic, neurotoxic, cardiotoxic, anti-inflammatory, and myonecrotic, and the blocking of ion channels [58].

#### 2.4.6. Cystatin (CST)

CSTs occur in prokaryotes and eukaryotes [59], and have been identified in the venom glands of invertebrates and vertebrates [31,60]. Their main function is the partial or total inhibition of cysteine-type endopeptidases, such as peptidase families C1 (papain family) and C13 (legumain family), in which CSTs are less selective inhibitors showing low discrimination between endopeptidases and exopeptidases [61]. *C. salei* CSTs belong to the cystatin type 2 family (IPR027214) with a cystatin conserved site (IPR018073). CST_2 exhibits two mutations in the mature protein as R130E and R132E. Sequence alignments of CST_1,2 with the predicted cystatins identified in *A. marikovskyi* and *S. mimosarum* show identities of 54% and 48.3% with *C. salei*, and even lower identities (38.2% and 34.3%) with *V. fasciatus* and *P. tepidariorum* (Supplementary Figure S1.14). Cystatin may play essential housekeeping and regulatory roles, and may protect toxic proteins and peptides in the glands from proteolytic degradation. However, a specific function in the prey’s tissue after injection should not be excluded. It is reported that hematophagous ticks express cystatins besides other peptidase inhibitors, in their salivary glands to escape the immune defense of the host organism during blood feeding [62].

#### 2.4.7. Kunitz Domain-Containing Protein (KCP)

KCPs have been identified at a very low expression level (Table 1), and may act as serine protease inhibitors inside the venom glands. The proteins belong to the pancreatic trypsin inhibitor Kunitz domain superfamily (IPR036880). Interestingly, two pancreatic trypsin inhibitor Kunitz domains (IPR002223) are identified within the protein (amino acid residues 28–83 and 111–164, respectively). The isoforms (KCP_1a and KCP_1b) differ in amino acid residues D107G, K109Q, D112A, and Q116L. Sequence alignments of different hypothetical spider KCPs with KCP1ab from *C. salei* show identities between 50.3% and 69.7% for araneomorph spider proteins (*A. marikovskyi*, *V. fasciatus*, *S. mimosarum*) and of only 36.3% for the mygalomorph spider *T. loki* (Supplementary Figure S1.15).

Kunitz-type serine protease inhibitors are widely spread in many types of plant and animals tissue. Furthermore, similar proteins with inhibitory activity against serine proteases were identified in the venom glands of insects [63], scorpions [64], pseudoscorpions [65], and spiders [66]. The fact that blood-sucking arthropods, such as ticks (salivary glands, midgut) [62,67] and black flies (salivary glands) [68], also possess Kunitz domain-containing proteins suggests a double function of these structures inside the glands and the targeted organism (prey, aggressor, host). The biological function in the glands might be regulatory in terms of storage and allocation of premature toxic peptides and proteins. After injection, such compounds may act as an antifibrinolytic, antielastolytic [66], and antihemostatic factor [69], regulating clotting and inflammatory responses [68], and inhibiting ion channels [70].

#### 2.4.8. Thyroglobulin Type-1 Domain-Like Protein (TT1LP)

We identified a protein, which exhibits two thyroglobulin type-1 domains (IPR036857). A second isoform is present with only one mutation (T31M). The identification of these domains points to a possible role as a protease inhibitor because the thyroglobulin type-1 domains belong to MEROPS protease inhibitor familyI31 (http://merops.sanger.ac.uk). MSA with other spiders shows an identity of 76.8% with a protein from *V. fasciatus* and of 49.7% with a protein from the mygalomorph spider *Haplopelma schmidti* (tr_B5M6G6). Lower identities of 28.5% and 33.3% were obtained by MSA with the ctenid *P. nigriventer* and the theridiid *Latrodectus hesperus* (Supplementary Figure S1.16). TT1LPs have been detected in the venom and salivary gland transcriptomes of arachnids, and seem to be a fixed constituent of the venomous cocktail of spiders, scorpions [71], pseudoscorpions [65], and blood-sucking ticks [72]. Proteins, exhibiting the thyroglobulin type-1 domain, may act as specific cysteine protease inhibitors [73] within the venom glands and/or in the prey.

#### 2.4.9. Insulin-Like Growth Factor-Binding Protein-Related Protein 1 (IGFBP-rP1)

IGFBP-rP1 was previously identified in the venom glands of *C. salei* [18]. This protein is characterized by four overlapping domains. An N-terminal highly structured globular insulin-like growth factor-binding domain (IPR000867) is followed by a Kazal-domain (IPR002350), acting as linker to the two C-terminal immunoglobulin-like/immunoglobulin subtype domains (IRP007110/IPR003599). High sequence identities of 72%–92% were observed between different spider IGFBPrPs, ticks (62%), and the spiny lobster, *Sagmariasus verreauxi*, a decapod crustacean (39%) (Supplementary Figure S1.17). Decapod IGFBP-related proteins are involved in the insulin-signaling pathway and are able to bind insulin like peptides (ILPs) [74]. In parasitic ticks, similar proteins are involved in their blood feeding activity [75]. It is tempting to speculate that, besides α-AMY, IGFBP-rP1 is another protein that interacts with the insulin-signaling pathway with undesired effects for the bitten prey or aggressor.

### 2.5. Proteins Belonging to the Innate Immune System

#### 2.5.1. Tachylectin 5A-Like Protein (TL5A)

Two TL5A-like proteins from the venom gland of *C. salei* exhibit the fibrinogen, α/β/γ chain, C-terminal globular domain (IPR002181). Two mutations occur at positions T61A and D193N. Additionally, a silent mutation was identified at position T579C. Tachylectin-5A, a lectin, was first described as a component of the innate immune system of the horseshoe crab *Tachypleus tridentatus* (Q9U8W8) that agglutinates a great variety of acetyl group-containing molecules and therefore plays an important role as first-line defense against microbes [76,77]. TL5A may acts as a first defense against microbial infections of the venom gland or it may be active in venom gland homeostasis. TL5A-like proteins were identified in the venom glands of theridiids (51.6% identity), eresids (59.7%), ctenids (67.8%), and lycosids (62.3%) (Supplementary Figure S1.18), but also in the hemocytes of *C. salei* (unpublished results L. Kuhn-Nentwig).

#### 2.5.2. Leucine-Rich Repeat Domain-Containing Protein (LRR)

We identified a protein, classified by InterPro as a possible member of the leucine-rich repeat domain superfamily (IPR032675), which exhibits five typical subtype LRR_typ_2 motifs of 20–23 amino acids. This protein was also identified in the transcriptomes of other spiders, and shows identities between 70.8% and 85.9% with *V. fasciatus*, *Oxyopes lineatus* and *A. marikovskyi* and of 68% with *S. mimosarum* LRRs. Leucine-rich repeats are known to often be involved in protein-protein interactions. Some ticks are well known to be infected with parasites and, subsequently, for transmitting parasites into the hosts. It could be shown that LRR domain-containing proteins were upregulated in the salivary gland and midgut of the infected tick *Haemaphysalis longicornis* during blood feeding. It is stated that LRRs play a key role in the tick innate immunity in controlling *Babesia* parasites [78]. Analogous to this, a possible function of LRRs in venom glands could be protection against undesired microbes that might invade spiders after injecting venom into an organism (Supplementary Figure S1.19).

### 2.6. Cysteine-Containing (Putative) Neurotoxins

To obtain comparable expression data of different neurotoxins, besides real-time PCR, two possibilities exist. (A) Normalizing and counting sequencing reads that map to contigs of a given neurotoxin, or (B) counting only reads that map to the mature peptide sequences within the contigs. For a given contig, we often observed a great imbalance of the number of normalized reads mapping to signal, pro-peptide or mature peptides. For quantification, therefore, we only considered reads mapping to the full mature peptide sequences of contigs (n_(full reads)_ = 2420) related to venom neurotoxins. For two peptides (CsTx-39 and CsTx-20a, b), and 13 further isoforms of different peptides, no full reads were available, and therefore overlapping reads (n_(no full reads)_ = 15) were used and counted each as one read.

Identified (putative) neurotoxins were classified into peptide families based on an updated version of HMMs [79]. Peptides were named according to the present valid nomenclature for spider peptide toxins [80]. Most of the toxins exhibit the inhibitor cystine knot (ICK) fold [81], the second most abundant fold is the colipase MIT1-like fold [82]. Two sequences show unknown cystine-folding patterns (Table 2). For a venom gland-specific defensin, the conserved cystine-stabilized α/β structural fold is supposed [83].

**Figure 2:**
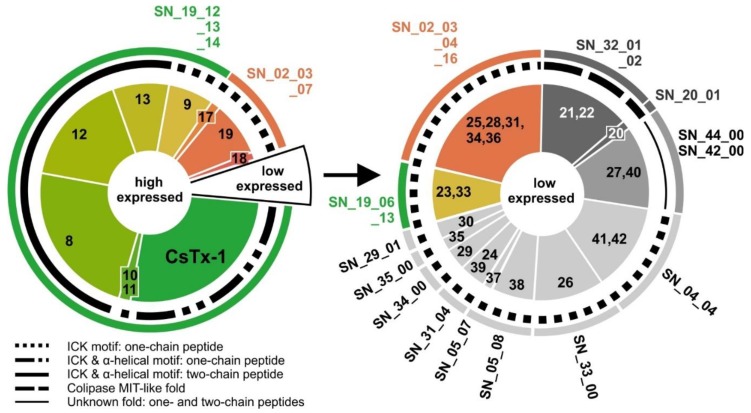
Classification and characterization of high- and low-expressed (putative) neurotoxins identified in the transcriptome of *C. salei*. Toxin names, following the CsTx numbering system, are indicated by numbers in the slices and slice size represents the expression level as estimated by full read counts of relevant contigs. Structural motifs (detailed in the figure legend) and family annotations of toxins according to reference [79] are detailed in black and colored lines on the outer side of the respective slices.

To our astonishment, 93.7% of all expressed neurotoxin-like transcripts are classified in only two different peptide families. These are the SN_19 family (83.1%), with subgroups SN_19_06, 12, 13 and 14, and the SN_02 family (10.6%) with subgroups SN_02_03, 04, 07 and 16. In contrast, the lower-expressed (putative) neurotoxins (6.3%) belong to 13 different peptide families and several subgroups (Figure 2). The transcripts of these peptides are all composed of a signal peptide, a pro-peptide with a C-terminal processing motif (PQM, in rare cases a dibasic “KR” motif), and the mature peptide. Some mature peptides feature a C-terminal glycine residue for PAM-mediated amidation [24] (Supplementary Table S1). Astonishingly, transcripts that belong to the SN_02 family were likewise identified in the transcriptome of the pseudoscorpion *Synsphyronus apimelus* [65]. The majority of *C. salei*’s (putative) neurotoxins exhibit similarities to neurotoxins of other araneomorph spiders, but not mygalomorph spiders. The only toxin family found in *C. salei* and in mygalomorph spiders, scorpions, and pseudoscorpions is the SN_32 family (MIT1-like AcTx family) [86]. Besides the SN_32 family, transcripts of the related SN_20 family were identified in *C. salei* and other araneomorph spiders. Peptides of these families are composed of a signal peptide, directly followed by the mature peptide and a stop signal. Although nothing is known about the target of these peptides, they might represent, besides enzymes and protease inhibitors, one of the phylogenetically first peptides recruited into spider venom glands.

In total, we identified 81 transcripts of (putative) neurotoxins resulting in 66 different precursors and 54 mature variants. The majority of the mature peptide variants were confirmed by sequence analysis via Edman sequencing and/or top-down/bottom-up proteomics. Two further peptides, which we have not identified in the transcriptome, have been formerly determined by Edman degradation (CsTx-11b and CsTx-18b); thus, the total number of neurotoxins is 56.

### 2.7. Signal Peptides and Pro-peptides of (Putative) Neurotoxins

The lengths of the signal peptides of the precursors described here vary only between 16 and 22 amino acid residues, and are independent from the lengths of pro-peptides and mature peptides. The pro-peptide lengths vary between 6 and 29 amino acid residues and, for 2/3 of all pro-peptides, a partial α-helical secondary structure is predicted. We found no length relationship between the proposed α-helices of the pro-peptides and C-terminal α-helices of mature peptides. However, longer mature peptides possess longer pro-peptides. In general, pro-peptides exhibit negative net charges, reaching from −2 to −11. The charge increases with the length of the pro-peptide. These highly negatively charged pro-peptides might influence folding, processing, and/or storage of the precursors (Supplementary Table S1). Further investigations, including more neurotoxin precursors from different spider species, are necessary to shed light on these structures.

### 2.8. C-terminal Modifications of Mature (Putative) Neurotoxins

C-terminal amidation of mature (putative) neurotoxins occur in the SN_19, SN_02, SN_04, and SN_05 families. However, in the SN_19 and SN_02 families, we also identified peptides with free C-termini. Only 30% of all transcripts code for peptides featuring a C-terminal Gly residue before the STOP codon. So far, C-terminal amidation is thought to influence the biological activity of mature peptides [87]. We have identified a further C-terminal post-translational modification of neurotoxins in the proteolytic elimination of C-terminal Arg by an unknown carboxypeptidase. This elimination was observed for the C-terminal Arg of the α-chain of CsTx-8, 12, and 13 [25], and for the C-terminal Arg of the monomer CsTx-27. Furthermore, the proteomic data provides evidence for removal of the C-terminal Lys from monomers CsTx-41 and 42 (Supplementary Table S1).

### 2.9. SN_19 Family

**Figure 3:**
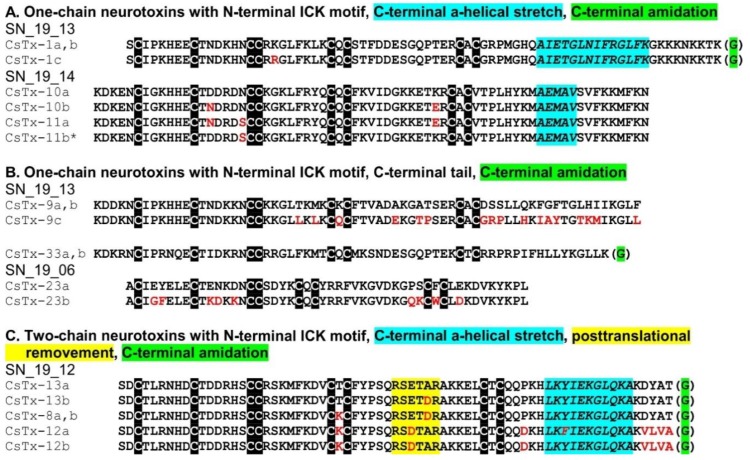
Multiple sequence alignment of mature peptides belonging to the SN_19 family. Amino acid residue differences within the different subgroups are given in red characters, possible C-terminal α-helical parts are marked in blue and italic, glycine residues for C-terminal amidation are highlighted in green and in brackets, and cysteines are highlighted in black. Post-translational removal of amino acid residues is given in yellow. Not shown are identical amino acid sequences with silent mutations and visible mutations within signal and pro-peptide. CsTx-11b * was sequenced by Edman degradation [13].

The SN_19 family is the most abundant peptide family (83.6%) identified in the venom and venom gland transcriptome of *C. salei* [12]. This family combines neurotoxins of different structural motifs (Table 2, Figure 3). The main neurotoxin CsTx-1 (26.4%), is characterized by an N-terminal ICK motif and a C-terminal α-helix. This α-helical part acts in a cytolytic manner [15], whereas the N-terminally located ICK-fold seems to be responsible for the inhibition of L-type Ca^2+^ channels [88]. The presence of two different motifs within CsTx-1 enhances insecticidal activity when compared with the C-terminally truncated form [27]. Such a motif combination is also present in CsTx-10a, b, and 11a, b (1.7%) with a shorter C-terminal α-helical part. No C-terminal α-helix, but an ICK motif is predicted for CsTx-9a, b, c (6.7%), as well as for CsTx-33a,b (0.4%).

CsTx-1 exhibits identities between 39.4% and 52.3% with peptides from two distant spider species within the RTA clade: *V. fasciatus* (VIRFA_DN65866_c0_g1_i1_4) and *L. singoriensis* (B6DCP0). A comparable peptide is identified in *Nephila pilipes* (NEPPI_DN30656_c0_g6_i1_5, 42.9%), belonging to the Araneoidea. All these related peptides are composed of two structural motifs, an N-terminal ICK motif and a C-terminal α-helix. Of interest is the high identity of CsTx-1 with the C-terminal domain of δ-miturgitoxin-Cp2a (A0A059T2H4, 44.6%) from *Cheiracanthium punctorium*, which exhibits N-terminally the ICK motif and C-terminally a short α-helical structure. So far, three different peptide groups have been mainly described from the venom of *C. punctorium* and all belong to the SN_19 family [89]. These peptides, in which two neurotoxins succeed each other, might be interpreted as a specific further development of the SN_19 toxin family. The N-terminal domain is characterized by an ICK motif followed by a short-extended strand, randomly coiled, or an α-helical region that connects to the C-terminal domain. This domain is again characterized first by an ICK motif, followed by differently pronounced α-helical tails comparable to CsTx-1, 10, and 11 (Supplementary Figure S2.1/S2.2).

Up to now, CsTx-1 has been the only known neurotoxin that exhibits such a long cationic C-terminal tail of 30 amino acid residues, and therein an α-helical region composed of 14 amino acid residues. However, *L. singoriensis* expresses several two-motif neurotoxins in which the C-terminal tail is composed of 21 and 28 amino acid residues with an α-helix buildup of six to 12 amino acid residues [90]. It is supposed that such C-terminal structures are acting as possible anchors, attracting negatively charged lipid rafts or glycoproteins on different membrane types [15,91]. Dependent on the length and charge of their C-terminal α-helices, the neurotoxic activity of such peptides is enhanced by the cytolytic activity towards different cell types. Missing a specific target (e.g., specific ion channels), the cytolytic-acting C-terminus can still harm a prey [92].

The highest-expressed mature peptides belong to the two-chain peptides CsTx-8, 12, and 13 (48.3%) that exhibit, besides the N-terminal ICK motif, a C-terminal α-helical motif of 11 amino acid residues [26]. The main difference to other peptides from the SN_19 family is a specific post-translational modification. Here, the PQM-protease, which is typically responsible for cutting pro-peptides from mature peptides, recognizes a PQM, as well as an inverted PQM within the mature peptide chain. As a result, the loop, defined by the disulfide bridge between C6 and C7, is opened by cutting out a six amino acid polypeptide and two short peptide chains remain [25]. These heterodimeric peptides are alone less insecticidal than the main toxin CsTx-1, but in combination with other monomeric peptides from the SN_19 family, (e.g., CsTx-9 or CsTx-1), a synergistic increase of toxicity is observed [26]. With this, *C. salei* exhibits a strategy to enhance the insecticidal activity of the SN_19 peptide family by a so far unknown peptide-peptide interaction between two-chain peptides and single-chain peptides [26]. From an evolutionary point of view, it can be assumed that the synergistic action and in this respect, the production of low toxic heterodimers (e.g., CsTx-13), provide greater benefit than higher production rates of a more toxic monomer (e.g., CsTx-1).

In-depth transcript analysis of the SN_19_00 family discloses a recombination and/or splicing process concerning signal peptides, pro-peptides, and mature peptides between the different genes of the two-chain peptides CsTx-8, 12, 13, and the single chain peptides 10, 11a, and 9. All these neurotoxins exhibit the unique sequence “EVQR” as PQM. In multiple cases, heterodimeric toxins exhibit identical coding sequences for signal and pro-peptide with monomeric toxins. This holds true for CsTx-13a, b; CsTx-10a, b, and CsTx-11a, CsTx-8a and CsTx-9c, CsTx-12b and CsTx-9a, and CsTx-12a and CsTx-9b. It is thereby astonishing that some peptides with identical signal and pro-peptides show higher sequence differences in their mature chain (e.g., heterodimers CsTx-8 and 9c), than other peptides with different signal and pro-peptides (e.g., heterodimers CsTx-8, 12, and 13) (Figure 3, Supplementary Figures S3.1–S3.3).

CsTx-23a,b precursors (0.08%) are characterized by an unusually short pro-peptide part composed of six amino acid residues including the PQM, followed by mature peptides. Compared to other low abundant precursors identified in *C. salei*, these peptides show higher identities with peptides so far described in sparassids (36.0%–52.0%), lycosids (60.0%–62.0%), and viridasiids (46.0%–48.0%) (Supplementary Figure S2.3). Interestingly, the length of the pro-peptide for the related precursors (*C. salei* and *L. singoriensis*) is conserved with four or six amino acid residues.

### 2.10. SN_02 Family

**Figure 4:**
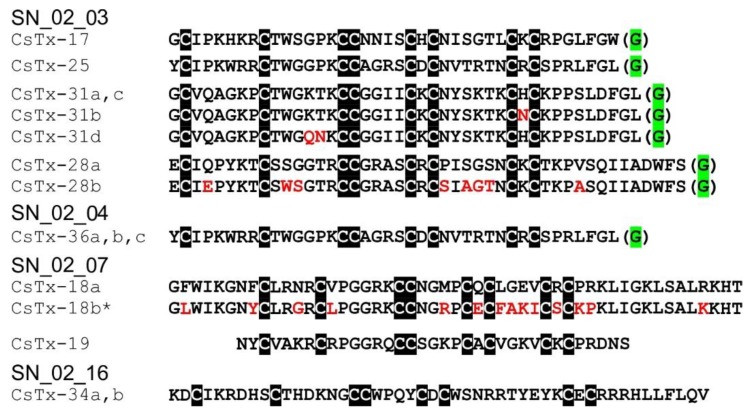
Multiple sequence alignment of mature peptides belonging to the SN_02 family. Amino acid residue differences within the different subgroups are given in red characters, C-terminal amidation is highlighted in green, and cysteines are highlighted in black. Not shown are identical amino acid sequences with silent and visible mutations within the signal and pro-peptide. CsTx-18b* was sequenced by Edman degradation [13].

The SN_02 family includes the shortest neurotoxin-like peptides identified in the venom of *C. salei*. The (putative) neurotoxins are characterized N-terminally by an ICK motif, a fourth disulfide bridge (C6–C7), and a C-terminal tail composed of five to 15 amino acid residues. CsTx-19 (8.0%) is the most abundant peptide of this family. Its signal and pro-peptide sequence exhibits a high identity (94.1%) with the corresponding sequence of CsTx-18a (1.4%). The mature peptides show a lower identity of 57.1% due to N- and C-terminal elongations of CsTx-18a. So far, similar peptides are not reported in UniProtKB (BLAST against with an e-value cut off of 10^−5^), providing evidence that CsTx-18 and CsTx-19 might represent a specific development within the genus *Cupiennius* (Figure 4).

Further peptide groups of the SN_02 family, which were identified in low abundance in the venom of *C. salei*, are widespread among araneomorph spiders, especially within the RTA clade. CsTx-36 (0.37%) and CsTx-28 (0.08%) show identities with peptides from the ctenid *P. nigriventer* (52.9%) and the lycosid *L. singoriensis* (42.6%). For CsTx-17 (1.1%) and CSTx-31 (0.57%), related peptides were identified in *Agelenopsis aperta* (identities of 39.5–53.7%) and *V. fasciatus* (identities of 32.5–42.1%) (Supplementary Figures S2.4 and S2.5).

A peptide similar to CsTx-34 (0.25%) was identified in *P. nigriventer* (TX3-5_PHONI) showing 40.5% identity. This peptide inhibits L-type calcium channels [93], produces paralysis in the posterior limbs and decreases movements after intracerebro-ventricular injection in mice [94]. Moreover, an identity of 48.8% has been found between CsTx-34 and ω-lycotoxin-Gsp2671g from *A. marikovskyi* [95], which modulates P-type voltage-gated calcium channels in vertebrate cerebellar Purkinje cells [96] (Supplementary Figure S2.6). CsTx-25 (0.08%) shows identities between 47.4% and 53.7%, with a fragment identified in the transcriptome of *V. fasciatus* and ω-agatoxin Iva,b, from the agelenid *A. aperta*, a P-type calcium channel antagonist of insects and vertebrates [96,97], pointing to a broad appearance of this peptide type within the RTA clade. The identity towards CsTx-17 is very high (60.1%) (Supplementary Figure S2.7).

**Figure 5:**
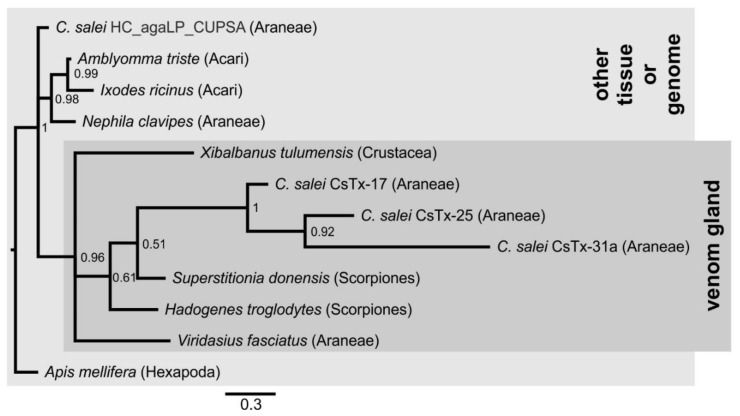
Sequence relationship of peptides of the SN_02 family. Sequence relationship was calculated using Bayesian inference methods. Sequences derived from venom gland transcriptomes are highlighted in dark gray, sequences from transcriptomes of other tissues or genomes in light gray. Nodes are labeled with posterior probability values. The peptide sequences used for analysis are accessible online with the following sequence identifiers: *C. salei* HC_agaLP_CUPSA (MH754628), *Amblyomma triste* (tr|A0A023G4R1|), *Ixodes ricinus* (tr|A0A131YAX3|), *Nephila clavipes* (tr|A0A2P6L7U6|), *Xibalbanus tulumensis* (contig00124SPETU, [99]), *C. salei* CsTx-17 (MH754572), *C. salei* CsTx-25 (MH754577), *C. salei* CsTx-31a (MH754573), *Superstitionia donensis* (tr|A0A1V1WBV1|), *Hadogenes troglodytes* (tr|A0A1B3IJ31|), *Viridasius fasciatus* (tr|A0A1V0FWF9|), *Apis mellifera* (XP_003249808).

In our in-house hemocyte transcriptome of *C. salei*, one peptide fragment was identified that we classified into the SN_02 family. Could this peptide be an evolutionary precursor of the SN_02 family neurotoxins that was recruited into spider venom glands? The agatoxin-like peptide from hemocytes exhibits 35.9% identity to CsTx-25, but possesses a dibasic KR motif instead of the PQM as cleavage motif. Surprisingly, the agatoxin-like peptide shows identities between 44.2% and 90.7% to peptides identified in the genome or in transcriptomes of the honeybee *Apis mellifera* [98], the remipede crustacean *Xibalbanus tulumensis* [99], the tick *Ixodes ricinus* (A0A147BFN0_IXORI), and the spider *Agelena orientalis* [100] (Figure 5, Supplementary Figure S2.7). Recently, the above-mentioned agatoxin-like peptide from the honeybee was shown to be located in the neuroendocrine tissue (glandular part of the corpora cardiaca) and might have a function as a neuropeptide and/or ion channel modulator [98]. It may be possible that this widespread peptide from the neuronal tissue of several major arthropod groups was convergently recruited into the venom glands of different venomous arthropods [98].

Recruitment of agatoxin-like tissue peptides occurs not only in the venom glands of spiders, but also in the venom glands of pseudoscorpions. In such a venom gland transcriptome of *Synsphyronus apimelus*, 11 transcripts have been identified that exhibit high identities with precursors from different spiders [65]. Based on HMMs [79], we classified ten of these peptides as belonging to the SN_02 family. The precursors are composed of a signal peptide, a pro-peptide with the dibasic “KR” motif in place of a PQM as cutting site, and the mature peptide. For such a peptide (Sapi_DN110686_c0_gl_i1), a high identity of 74.4% was found towards the hemocyte-derived agatoxin-like peptide of *C. salei* and 38.5% towards CsTx-25 (Supplementary Figure S2.7).

### 2.11. Low Abundant (Putative) Neurotoxins

Besides the above-mentioned low abundant (putative) neurotoxins of the SN_02 and SN_19 families, a high variety of low abundant transcripts belong to ten more different peptide families. Most of them exhibit high identities with neurotoxins or precursors from other araneomorph spiders in general, or may even be closer related to those from the Araneoidea and/or RTA clade, thus representing phylogenetically old structures. These “ancient” peptides might not functionally be very important, but should be seen as a reinsurance to preserve a high combinatorial diversity of active neurotoxins against a high diversity of targets in different animals.

### 2.12. SN_04, SN_05, and SN_31 Family

**Figure 6:**
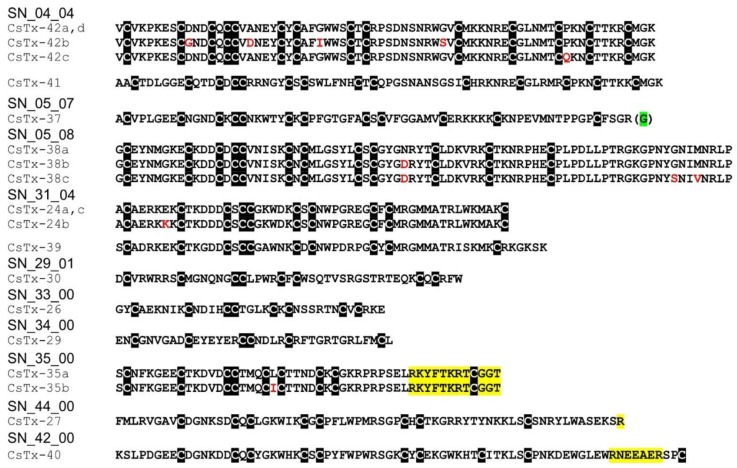
Multiple sequence alignment of mature peptides belonging to different low abundant peptide families. Amino acid residue differences within the different subgroups are given in red characters, C-terminal amidation is highlighted in green, post-translational removal of amino acid residues is given in yellow, and cysteines are highlighted in black. Not shown are identical amino acid sequences with silent and visible mutations within the signal and pro-peptide.

Five different neurotoxin precursor fragments, belonging to the SN_04 family, are present in concentrations between 0.04% (CsTx-41) and 0.78% (CsTx-42a, b, c) in the venom gland of *C. salei*. With seven disulfide bridges, these (putative) neurotoxins exhibit the highest number of cysteines in venomous peptides so far identified in spider venoms. They show identities between 30.3% and 40.6% with neurotoxins from ctenids, such as µ-ctenitoxin-Pn1a (P17727|TXL1_PHONI), a state-dependent inhibitor of neuronal sodium channels [101], and ω-ctenitoxin-Pn3a (P81790|TX34_PHONI) [102,103], an antagonist of calcium channels (Cav2.1 and Cav2.2). Interestingly, according to UniProtKB the last two disulfide bridges for omega-ctenitoxin-Pn3a connect C11–C12 and C13–C14 and, for µ-ctenitoxin-Pn1a, C11–C13 and C12–C14. Identities over 42% were found between CsTx-41 and CsTx-42a, b, c with putative precursors published for the pisaurid *Dolomedes fimbriatus* [104] (Figure 6, Supplementary Figure S2.8).

CsTx-37 (0.08%) was classified into the SN_05 family [79]. The peptide exhibits high identities with transcripts from pisaurids (67.7%), and sparassids (41.5%). Moreover, CsTx-37 exhibits an identity of 56.7% with ω-agatoxin-Aa3a from the agelenid *A. aperta*, which acts as antagonist of synaptosomal Ca^2+^ channels [105] (Figure 6, Supplementary Figure S2.9). Besides CsTx-1, we identified another putative neurotoxin, CsTx-38a, b, c (0.41%), which exhibits a strikingly long C-terminal stretch of 23 amino acid residues mainly composed of five different amino acids: Pro, Leu, Gly, Asn and Arg. This stretch is at minimum twice as long as the longest C-terminal stretch identified in peptides of other spider families and might be an innovation of *C. salei* to enhance toxic activity. Identities of 40.9%–45.5% (ctenids, pisaurids, and oxyopids), and 51.5%–53% (lycosids) were calculated for these (putative) neurotoxins (Figure 6, Supplementary Figure S2.10).

The SN_31 family includes CsTx-24a, b, c (0.21%) and CsTx-39 (0.04%), which are characterized by five disulfide bridges. So far, an Interpro scan could not identify any known protein family memberships for both peptide groups. CsTx-24a, CsTx-24b, CsTx-24c shows high identities with putative neurotoxins from other spiders, such as pisaurids (68.6%–76.5%), viridasiids (52.0%–54.0%), lycosids (40.0%–41.2%), and eresids (52.9%–56.9%), one of the oldest araneomorph spider groups after haplogynes. Therefore, it is tempting to speculate that this family of (putative) neurotoxins could be widespread at least among entelegyne spiders (Supplementary Figure S2.11).

### 2.13. SN_34, SN_29, SN_33, SN_19, and SN_35 Family

The SN_34 family includes peptides that exhibit the ICK motif composed of three disulfide bridges without a fourth disulfide bridge for C6–C7. CsTx-29 (0.2%) is the only *C. salei* venom peptide belonging to this family. The peptide shows identities between 61.8% and 69.4% with peptides of unknown function from viridasiids and pisaurids (Figure 6, Supplementary Figure S2.12). Peptide families SN_29, and _33 are characterized by the ICK fold as cysteine framework, and feature a fourth disulfide bridge between C6 and C7. With CsTx-30 (0.16%), a peptide related to P-type Ca^2+^ channel inhibitor ω-Lsp-IA [95], and to a putative neurotoxin from *Dolomedes fimbriatus* was identified in *C. salei* [104] (Supplementary Figure S2.13).

Peptides similar to CsTx-26 (SN_33_00, 0.66%) were identified in many spider families of the RTA clade (ctenids, lycosids, pisaurids, sparassids) with high sequence identities of between 63.2% and 82.9%, pointing to a functionally highly conserved structure of the mature peptide. First insights into spider venom gland transcriptomes of other spider families support the wide distribution of the conservative peptide family SN_33 (Kuhn-Nentwig and Langenegger, personal communication). Wide distribution is further supported by the high amino acid sequence identity of 80% between CsTx-26 and purotoxin 1 (PT1). PT1, which shows antinociceptive activity by the inhibition of P2X3 receptors of rat dorsal root sensory neurons [106], was first isolated from the lycosid *Alopecosa marikovskyi*. In contrast to the conserved sequences of mature peptides, signal peptide and pro-peptide exhibit lower sequence identities, between 33.3% and 64.9%, and might be more spider family-specific (Figure 6, Supplementary Figure S2.14).

**Figure 7:**
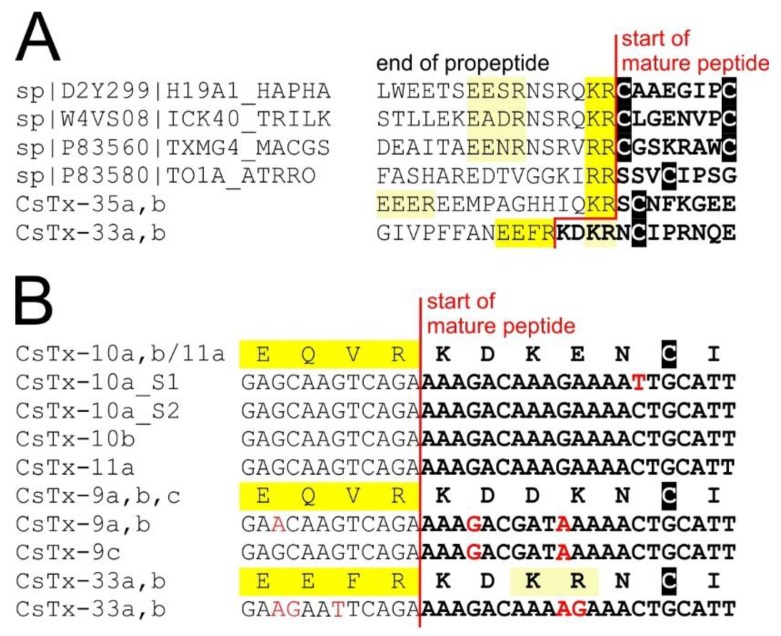
Identification of different pro-peptide cleavage motifs in one precursor. (**A**) Pro-peptide cleavage motifs of CsTx-33, CsTx-35, and other peptides featuring a dibasic motif. The sequence parts shown include the ends of pro-peptides and the beginning of mature peptides. The start of the mature peptide sequences is indicated with a bold red line. Amino acids of the mature peptide are in bold. Potential protease cleavage motifs are displayed in dashed boxes. Red boxes indicate the cleavage motif, which directly locates the N-terminal of the experimentally found start of the mature peptide. (**B**) Sequence comparison of transcripts coding for CsTx-33 and other *C. salei* peptides with a similar N-terminal sequence of the mature peptide. Nucleotides differing between sequences are highlighted in red.

Precursors of CsTx-33 (SN_19_33, 0.4%) (Supplementary Figure S2.15) and CsTx-35a, b (SN_35_00, 0.12%) (Supplementary Table S1, Supplementary Figure S2.16) both exhibit dibasic recognition motif “KR”, and only CsTx-33 exhibits an additional PQM motif between the end of the signal peptide and the first cysteine of the mature peptide. Dibasic motifs have been postulated to serve as pro-peptide cleavage sites in some neurotoxin precursors of mygalomorph spiders. Some peptides of *Trittame loki* (W4VS08) [107] and *Haplopelma hainanum* (D2Y299) show cleavage motif “KR” [108,109], and some of *Macrothele gigas* (P83560) [110] and *Atrax robustus* (P83580) [111] the cleavage motif *“RR”*. However, some toxins also feature a PQM downstream of the dibasic motif (Figure 7A). This presence of multiple known cleavage motifs at possible pro-peptide cleavage sites shows the importance of proteomic data for accurate determination of the actual cleavage site. Proteomic top-down analysis revealed that, in the case of CsTx-33, the PQM motif is used as pro-peptide-cutting site. In contrary, CsTx-35 and some peptides of *H. hainanum* are cleaved after dibasic motif “KR” (Figure 7A) as shown by mass-spectrometry and Edman degradation [112], respectively. Further investigations are needed to explain the observed specificity in pro-peptide cleavage. However, we observed an evident similarity between the nucleotide sequences of the non-dibasic motif containing CsTx-9, -10, -11, and CsTx-33 in the region of the pro-peptide-mature peptide junction (Figure 7B), possibly indicating an evolutionary relationship of these transcript parts. The only mutations within the first 21 N-terminal nucleotides of the mature peptides of CsTx-33, -10, and -11 are two point-mutations causing the dibasic motif in CsTx-33.

Top-down proteomics of CsTx-35 revealed another post-translational modification of the CsTx-35 precursor. The last twelve C-terminal amino acid residues are post-translationally removed. This post-translational modification is comparable to the processing of the precursors of CsTx-8, 12, and 13 by the PQM-protease and a so far unknown carboxypeptidase [25] (Figure 6). Remarkably, mature CsTx-35 showed 92% identity to LDTF-11, a putative neurotoxin from *Dolomedes fimbriatus* [104], whereas their signal peptides and pro-peptides showed only 71.4% identity. The mature chains of CsTx-35 and CsTx-26 are less variable than their signal and pro-peptides when compared with the corresponding peptides of other related spiders. These findings are in contrast to the present opinion that the predominant mutation sites should be in the mature peptides when comparing peptides within a peptide family of one species. However, Kozlov and coworkers showed, for putative neurotoxin precursors of *D. fimbriatus* that the most variable region is the pro-peptide region, followed by the signal peptide and N-terminal parts of the mature peptides [104].

### 2.14. SN_42 and SN_44 Family

A high identity of 70.3% was found between CsTx-40 (0.08%) and omega-agatoxin-1A (agelenids), a heterodimeric neurotoxin and selective L-type calcium channel blocker (Cav/CACNA1) [113,114]. The disulfide bridge pattern for the present 10-Cys-containing peptides has not yet been solved. Interestingly, this cysteine pattern is widespread within spiders of the RTA clade and can be found in pisaurids (73.0% identity), viridasiids (76.8%), thomisids (61.1%), and lycosids (80.0%) (Supplementary Figure S2.17). Comparable to the two-chain neurotoxins CsTx-8, 12, 13 and omega-agatoxin-1A, CsTx-40 exhibits in its C-terminal sequence an inverted PQM as well as a PQM. The post-translational modification of this peptide by a PQM protease produces a heterodimeric structure as shown previously [25]. This also holds true for the related sequences in the above-mentioned spider families. The resulting long chain, C-terminally comprises 10 amino acids after the last Cys residue. This C-terminal part is about two times longer than the corresponding sequence lengths of CsTx-8, -12, and -13. Such long chains might be highly flexible and may interact with other peptides, resulting in increased toxic activity, comparable to CsTx-8, CsTx-12, and CsTx-13.

CsTx-27 (0.7%) is structurally comparable to CsTx-40 but C-terminally misses two cysteine residues. Related precursors have been identified only in lycosids (54.0%–58.1% identity). As a result of top-down proteomics, post-translational modification has been identified for CsTx-27. Here again, the C-terminal Arg residue is removed by an unknown carboxypeptidase [25] (Supplementary Figure S2.18).

### 2.15. SN_20 and SN_32 Family

**Figure 8:**
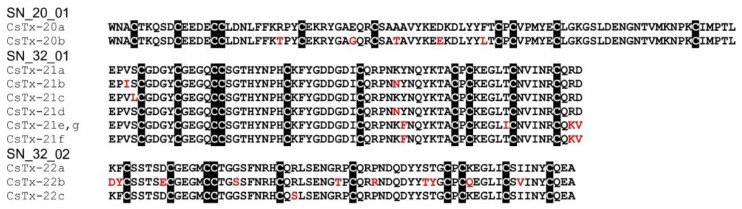
Multiple sequence alignment of mature peptides belonging to the SN_20 and SN_32 peptide families. Amino acid residue differences within the different subgroups are given in red characters, and cysteines are highlighted in black. Not shown are identical amino acid sequences with silent and visible mutations within the signal and pro-peptide.

Precursors corresponding to these families are characterized by the missing pro-peptide region, which seems to be a “requisite” for most so far described (putative) neurotoxins of mygalomorph and araneomorph spiders [115]. CsTx-20 (SN_20_01, 0.08%), CsTx-21a,b,c,d,e,f,g (SN_32_01, 0.62%), and CsTx-22a,b,c (SN_32_02, 0.16%) lack these pro-peptides and are present only in low abundances in the venom. In contrast to (putative) neurotoxins of other peptide families, the mature peptides of the SN_20 and SN_32 family are more anionic peptides with only net charges between –4 and 1. All these peptides possess five disulfide bridges (Figure 8).

With 86 amino acid residues and a molecular mass of 9.9 kDa, CsTx-20 is the largest peptide that we have purified from the venom. Interpro sequence analysis showed no relationship to any protein family and no domain could be identified. Disulfide bridge connectivity was determined as C1–C4, C2–C5, C3–C7, C6–C9, and C8–C10 [85], which corresponds to the disulfide pattern of black mamba intestinal toxin 1 (MIT1) [116]. In contrast to MIT1 (only 23.7% identity), CsTx-20 lacks the N-terminal AVIT sequence, characteristic for a part of the prokineticin domain that is essential for biological activity, e.g., pain sensation and stimulation of smooth muscle contraction [86,117]. Blast results show a broad distribution of CsTx-20 homologs in araneomorph spiders of the RTA clade (pisaurids, 89.5% identity; sparassids, 67.1%) araneids (67.1%), and eresids, (68.2%), but also in scorpions (*Hadrurus spadix,* 31–35.4%). Identifying similar peptides in spider and scorpion venoms points to a common ancient precursor or a convergent evolution in both arachnid orders. So far, no biological activity is described for these peptides isolated from spider and scorpion venom (Supplementary Figure S2.19).

Interpro analysis shows that CsTx-22a, b, c comprise the prokineticin domain (IPR023569) nearly over the whole length of the peptides (amino acid residues 5–59, CsTx-22) but the crucial N-terminal AVIT sequence part, responsible for its biological activity, is lacking. The prokineticin domain is identified in several putative toxin precursors from different araneomorph and mygalomorph spiders, but also, surprisingly, from ticks. Sequence identities between CsTx-22a, b, c and such peptides are medium to high: for araneomorph spiders 43.9%–7.6%, for mygalomorph spiders 41.8%–49.2%, and for ticks 37.5%–39.1%. Sequence alignments even show 22.4%–33.4% identity to the prokineticin Bm8f from the toad *Bombina maxima* and to MIT1 from the elapid black mamba (Supplementary Figure S2.20).

CsTx-21a, b, c, d, e, f, g are classified as belonging to the atracotoxin family (IPR020202). This family classification is based on ACTX–Hvf17 [118] and six more MIT1-like ACTX orthologs isolated from the venom of the mygalomorph funnel web spiders *Hadronyche versuta* and *H. infensa* [86]. They share sequence homologies to the above-mentioned MIT1 and Bm8f, but no pharmacological activity or biological function in the venom is known. Mature CsTx-21 isoforms show amino acid sequence identities to peptides of other araneomorph spider in the range of 51.6–60.0% and with mygalomorph spiders in the 38.1%–45.3% range (Supplementary Figure S2.21).

Taking all arguments into account, it is most likely that CsTx-21a, b, c, d, e, f, g, and CsTx-22a, b, c can be classified as peptides that might exhibit the ancestral disulfide-directed beta-hairpin (DDH) domain as shown for the nontoxic atracotoxin-Hvf17 (ACTX–Hvf17) identified in the atracid *Hadronyche infensa*. The corresponding amino acid consensus sequence is defined as CX_5-9_CX_2_[G or P]X_2_CX_6-19_C, which is in accordance with the amino acid consensus sequence CX_4-5_CX_2_[G]X_2_CX_8_C of isoforms of CsTx-21 and CsTx-22. Furthermore, loop 3 of this domain is highly conserved as C–GXGXC–C, comparable to loop 3 of MIT1-like ACTXs [86]. Together with the determined disulfide bridge pattern of CsTx-20, it seems that CsTx-20, 21, and 22 are the only peptides in the venom of *C. salei* that exhibit a DDH fold (Colipase MIT1-like fold), hypothesized to be the evolutionary precursor of the ICK motif [82]. Identifying related peptides to CsTx-20, 21, and 22, not only in araneomorph and mygalomorph spider venoms [107], but also in the venom of scorpions [119], pseudoscorpions [65], and in the salivary glands of ticks [120], may give a clue that these peptides may be one of the first compounds recruited into venom and salivary glands. Unfortunately, their targets still need to be elucidated.

### 2.16. Defensin-Like Peptide

**Figure 9:**
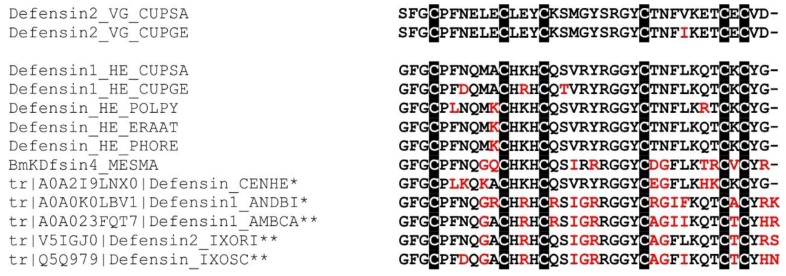
Multiple sequence alignment of mature defensin-like peptides from different arachnids and types of tissue. Amino acid residue differences of defensins within different tissue subgroups (VG, venom gland; HE, hemocytes; * venom glands, ** salivary glands) are given in red characters and cysteines are highlighted in black. Sequences belong to the spiders *C. salei* (defensin2_VG_CUPSA, (CSG_98_27980_30560); defensin1_HE_CUPSA [121]), *Cupiennius getazi* (defensin2_VG_CUPGE, (DN21681_c1_g2_i3_4); defensin1_HE_CUPGE, (DN14943_c0_g1_i1_6)), *Polybetes pythagoricus* (defensin_HE_POLPY [121]), *Eratigena atrica* (defensin_HE_ERAAT [121]), *Phoneutria reidyi* (defensin_HE_PHORE [121]), the scorpions *Mesobuthus martensii* (BmKDfsin4_MESMA [122]), *Centruroides hentzi* (tr|A0A2I9LNX0_defensin_CENHE [123]), *Androctonus bicolor* (tr|A0A0K0LBV1_defensin1_ANDBI [124]), and the ticks *Ixodes scapularis* (tr|V5IGJ0_defensin2_IXORI [125]), *Ixodes ricinus* (tr|Q5Q979_defensin_IXOSC), and *Amblyomma cajennense* (tr|A0A023FQT7_defensin1_AMBCA [120]).

We identified a defensin-like peptide in the venom gland, with a so far unknown function, which we named defensin-2. Transcripts coding for this peptide have not been identified in our *C. salei* hemocyte transcriptome, indicating that defensin-2 is a venom gland-specific peptide. Defensin-2 shows 54% sequence identity to defensin-1, a peptide from *C. salei* that was shown to be expressed in ovaries, subesophageal nerve mass, hepatopancreas, hemocytes, and muscle tissue. Neither reverse-transcriptase-PCR nor 454-sequencing showed any expression in the venom glands of the spider [121]. Illumina sequencing, however, revealed defensin-1 and defensin-2 homolog transcripts in the venom glands of *Cupiennius getazi*, a sister species of *C. salei*. It is tempting to assume that this inconsistency is due to the higher read-depth of Illumina sequencing compared to 454-sequencing, allowing to detect very low-abundant transcripts that may emerge from a few hemocytes present in dissected venom glands. The amino acid differences between hemocyte defensins-1 from both sister species are small (91.9% identity). The same holds true for defensins-2 from venom glands (97.7%) (Figure 9).

Defensins so far identified in other arachnids show higher sequence identities to *C. salei* defensin-1 than to the venom specific defensin-2. BmKDfsin4 [122], a classical defensin identified in the scorpion *Mesobuthus martensii*, exhibits a conserved cystine-stabilized α/β structural fold (C1–C4, C2–C5, C3–C6), which can be likewise assigned to spider defensins. In fact, BmKDfsin4 shows inhibitory activity against Gram-positive bacteria, and potassium channel current-blocking activity. It is hypothesized that scorpion defensins and some scorpion neurotoxins originated from one precursor [83,122]. To the best of our knowledge, it is the first time that a venom gland-specific defensin has been identified in spider venom. Further investigations are necessary to elucidate the recruitment and possible neofunctionalization of defensins in terms of antimicrobial and potassium channel-blocking activities of these spider venom gland peptides.

### 2.17. Proteomics

We used a combined approach of top-down and bottom-up mass spectrometry to validate the sequence of venom-neurotoxins and -proteins identified on the transcriptome level. From a total of 54 (putative) neurotoxins and their mature peptide isoforms identified by transcriptome analysis, we validated the presence of 49 by mass spectrometry of venom fractions (Supplementary Figure S4).

The venom presence of all proteins identified on the transcriptome level, except for the house keeping protein signal peptidase, could be validated with high sequence coverages (Table 1, Supplementary Figures S1.1–S1.19). Proteomic analysis is inevitable for identification or validation of post-translational modifications. Thereby, mass spectrometric analysis of undigested peptides (top-down proteomics) proved to be highly suitable to identify post-translational processing by proteases (as discussed to occur in precursors of CsTx-8, -12, -13, -35, and -27) including the allocation of the cleaved signal peptide (e.g., for CsTx-22) and pro-peptide-processing motif if multiple motifs are present (e.g., CsTx-33). However, very low-expressed peptides or large proteins could not be assessed by top-down proteomics. In such cases, mass spectrometric analysis of digested peptides (bottom-up proteomics), using multiple proteases for digestion, provided a reasonable alternative with high sensitivity and sequence coverage (Table 2, Supplementary Table S1). High sensitivity and sequence coverage are thereby the key to validate low-expressed peptides and highly similar isoforms of peptides. In addition to mature peptides, we also surprisingly identified fragments of pro-peptides of some neurotoxins by bottom-up proteomics. It is tempting to assume that these identifications indicate the presence of immature peptides comprising unprocessed pro- peptides and mature peptides in the venom or trace amounts of cleaved-off intact pro-peptides. The presence of high quantities of intact pro-peptides, however, is not likely as pro-peptides of CsTx-1 and CsTx-12 could not be observed by the anion exchange chromatography of venom (Supplementary Figure S5).

### 2.18. The Dual Prey-Inactivation Strategy of Spiders

**Figure 10:**
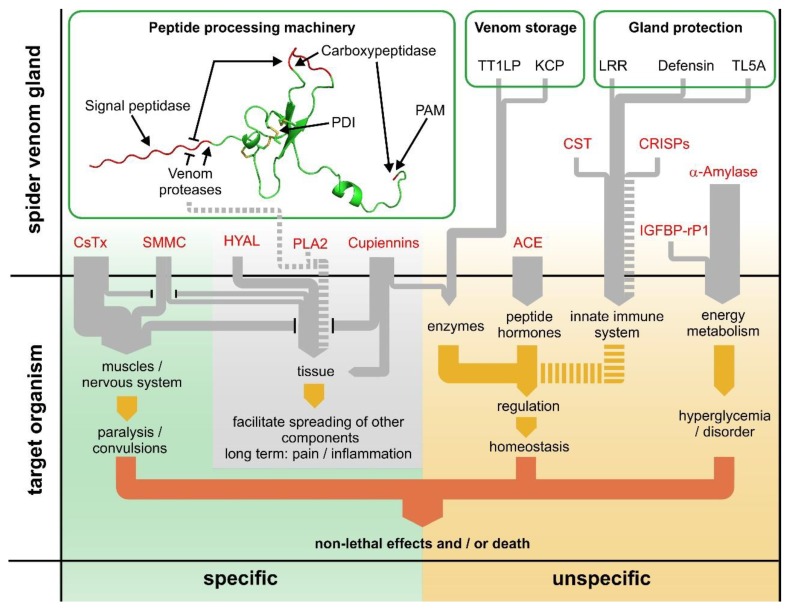
Dual prey-inactivation strategy of the venom of *C. salei* based on specific (left) and unspecific (right) venom pathways. Main interactions of the major venom components are shown in the venom gland (upper half) and, after venom injection, in the target organism (lower half). The specific pathway, mainly based on neurotoxins and other compounds, usually leads to death. The unspecific or metabolic pathway, based on a variety of regulatory elements, disturbs homeostasis or leads to hyperglycemia. The thickness of the gray arrows indicates the estimated impact on the prey. Dashed lines represent vague or uninvestigated connections (for further details compare text). ACE, angiotensin-converting enzyme; CRISPs, Cysteine-rich secretory proteins; CST, cystatin; CsTx, *Cupiennius salei* toxins; HYAL, hyaluronidase; IGFBP-rP1, insulin-like growth factor-binding protein-related protein 1; KCP, Kunitz domain-containing protein; LRR, leucine-rich repeat domain-containing protein; PAM, peptidylglycine α-amidating monooxygenase; PDI, protein disulfide isomerase; PLA2, phospholipase A2; SMMC, small molecular mass compounds; TL5A, tachylectin 5A-like protein; TT1LP, thyroglobulin type-1 domain-like protein.

Previously published data on low molecular mass compounds [92,126] and cytolytic peptides (cupiennins) [127,128], together with the here presented proteins and (putative) neurotoxins open a holistic view on the synergistic mode of action of *C. salei* venom compounds after injection into a prey or aggressor. Analyzing all interacting compounds, we hypothesize a specific and an unspecific prey inactivation pathway, resulting in a dual prey-inactivation strategy (Figure 10).

Compounds of the specific pathway are neurotoxins, low molecular mass compounds, a highly active hyaluronidase, phospholipase A2 and the cupiennins. The unspecific pathway includes α-amylase, CRISPs, angiotensin converting enzyme, cystatin and IGFBP-rP1. In the specific pathway, a great variety of neurotoxins act synergistically [26], but also with small molecular mass compounds and cupiennins, all affecting ion channel targets of the nervous system and in muscle tissues, finally resulting in paralysis, convulsion and death. The spreading of these toxins into the tissue is supported by hyaluronidase, phospholipase A2 and the cupiennins, through destruction of negatively charged membrane types. The unspecific inactivation pathway is characterized by different enzymes, which play a central part by interacting with the regulation of important metabolic pathways, thus unbalancing the homeostasis of an organism. The main actors are α-amylases, CRISPs, and angiotensin-converting enzymes. Furthermore, some of the cupiennins inhibit the formation of nitric oxide by neuronal nitric oxide synthase, which dramatically disturbs numerous processes using nitric oxide as a neurotransmitter [129]. The dual prey-inactivation strategy of spiders reduces the development of resistance against single venom compounds and the risk of losing prey due to escape.

## 3. Conclusions

We roughly divide the proteins mentioned here into three different groups with overlapping functions. (1) Key enzymes, involved in toxin-processing machinery; (2) recruited and neofunctionalized enzymes and proteins, immediately affecting the most conserved endocrine systems of animals, acting as spreading factor, and causing inflammations and indisposition as long-term effect, possibly against vertebrate aggressors, and (3) proteins of the innate immune system of *C. salei*, fighting against microbial invaders in the venom gland and maybe influencing prey homeostasis. Most of these proteins, identified on a transcriptomic and proteomic site in *C. salei*, are also present in the venom glands of other arachnids, especially in the venom of spiders, scorpions, and pseudoscorpions, and in the salivary glands of hematophagous ticks [31,130,131]. This wide distribution may be an example of convergent evolution, especially when the glands, where these substances are expressed, are not homologous.

In contrast with other spider venoms, the number of different neurotoxin families and neurotoxins in *C. salei* is comparatively low. The main actors belong to only two peptide families (SN_19 and SN_02) that have been highly optimized in terms of synergistic interactions [26]. Eleven further neurotoxin families are present in the venom in low concentrations; they may belong to a common heritage of spider toxins, but their evolutionary origin remains unclear.

Our detection of the first spider venom gland-specific defensin and its origin in hemocytes offers a fascinating possibility to track the origin of toxic compounds, the so-called neofunctionalization, and provides insight into the process that leads from a nontoxic to a toxic compound. Moreover, comparable transcriptomic studies of venom glands of different spider families may be a fascinating approach to filter out the essential constellation of venomous components present in each spider venom. Such a constellation might yield a principal composition that is not only realized in spider venoms, but also in the venoms of other arachnids.

## 4. Materials and Methods

### 4.1. Spider Maintenance, Venom Collection, and cDNA Libraries

Breeding of *Cupiennius salei* and venom collection were done as described earlier [132]. The cDNA libraries of venom glands and hemocytes of *C. salei* are based on 454-sequencing and were reported previously [15]. For transcriptomic studies, *Ancylometes rufus, Viridasius fasciatus* and *Cupiennius getazi* were laboratory-bred. *Oxyopes heterophthalmus, Oxyopes lineatus,* and *Cheiracanthium* sp. were collected in France. *Nephila pilipes* was collected in Taiwan, and *Xysticus cristatus* and *Atypus piceus* in Switzerland. No specific permissions for collecting the spiders were required. Collections were done on publicly accessible land without any protection status, such as common land. None of the spiders described here belongs to a protected or endangered species. The cDNA libraries of the venom glands of *Alopecosa marikovskyi, Ancylometes rufus*, *Cheiracanthium* sp., *Nephila pilipes, Oxyopes heterophthalmus*, *Oxyopes lineatus*, *Viridasius fasciatus,* and *Xysticus cristatus*, as well as sequencing with the next-generation sequencing platform of the University of Bern on an Illumina HiSeq3000 were performed as described by Langenegger et al. [25]. Multiple sequence alignments (MSA) of related venom proteins and peptides, as well as the calculation of peptide identities, were performed using ClustalW (https://www.ebi.ac.uk/Tools/msa/clustalo/) and were also partially manually edited. Structural analysis of the protein domains was performed using InterPro [133], and classification of the sequences into different families with HMMcompete [79] that had to be enlarged to cover a wider number of neurotoxins. Information about the secondary structure of the peptides was obtained using the GOR IV secondary-structure prediction method [134]. Measurement of mRNA abundance in the transcriptome (contigs) was done as reported in Reference [135] and given as Transcripts Per kilobase Million (TPM).

### 4.2. Transcriptome Analysis

The assembled reads were analyzed following the workflow described in Supplementary Figure S6 using BlastP against UniProtKB (E-threshold, 0.0001; BLOSUM-62, non filtering and gapped; UniProtKB/Swiss–Prot 2018_06–June 20, 2018), SignalP [136], in-house Hidden Markov Models (HMMs), cysteine pattern search scripts, Getorf (http://www.bioinformatics.nl/cgi-bin/emboss/getorf), and Pfam2go. All obtained contigs and the corresponding reads, referring to venom gland-specific proteins, (putative) neurotoxins, and peptides, were further analyzed on the nucleotide level to detect transcript isoforms. Nucleotide sequence variants that obviously resulted from sequencing errors/frame shifts were excluded.

### 4.3. Tandem Mass Spectrometry

We analyzed the venom proteome of *C. salei* combining bottom-up and top-down proteomics. To reduce sample complexity for further LC-MS^2^ analyses, 50 µL venom was separated into seven fractions by size exclusion chromatography on a Superdex 75 Increase HR 10/300 column (GE Healthcare) using 50 mM NH_4_OAc pH 5.0 supplemented with 500 mM NaCl as the liquid phase (Supplementary Figure S7). All fractions were desalted by RP-HPLC and analyzed by bottom-up proteomics. Peptide-containing fractions (2–7) were additionally analyzed with a top-down proteomic approach. For bottom-up proteomics, 20 µg protein was reduced and alkylated followed by digestion with trypsin and chymotrypsin, respectively, as described elsewhere [137]. Further LC-MS^2^ analysis was done on an Orbitrap Fusion Lumos mass spectrometer (Thermo Fisher Scientific) with a Dionex Ultimate 3000 nano-UPLC system (Thermo Fischer, Bremen, Germany). Protein digests were loaded onto a pre-column (PepMap C18, 5 µm, 100 Å, 300 µm × 5 mm) at a flow rate of 50 µL/min with loading solvent (0.05% TFA in water/acetonitrile 98:2). After loading, peptides were eluted in backflush mode onto the analytical nano-column (C18, 3 µm, 155 Å, 0.075 mm i.d. × 150 mm length, Nikkyo Technos, Tokyo, Japan) using an acetonitrile gradient of 5% to 40% solvent B (0.1% FA in water/acetonitrile 4.9:95) in 60 min at a flow rate of 400 nL/min. The column effluent was directly coupled with the mass spectrometer via a nanoflex electrospray source (Thermo Fischer, Bremen, Germany). Precursor ion scans were recorded in the Fourier transform detector (FT) with a resolution of 120,000 (at m/z = 200), a maximum injection time (mIT) of 50 ms, and an automatic gain control (AGC) setting of 4 × 10^5^. High energy collision-activated (HCD) fragment spectra were acquired parallel to the FT scan with a top-speed fragment spectra acquisition method of the most intense precursor ions in the linear ion trap for a cycle time of 3 s at an mIT of 35 ms, AGC of 1e4, and exclusion from further fragmentation for 30 s, using a relative HCD energy of 30%.

For top-down proteomics, 250 ng of reduced and alkylated protein was analyzed on the same nano-LC-MS^2^ setup as for bottom-up. Proteins were separated by a two-step acetonitrile gradient rising from 5% to 20% within 15 min and then to 60% within 30 min. Precursor and fragment spectra were both recorded in profile mode with the orbitrap detector using a resolution of 120,000 (at m/z = 200). The MS1 full scan range was 500–2000 m/z, the AGC was at 5 × 10^5^, and the maximum injection time was 50 ms, using a declustering potential of 15 V at the source. Data-dependent fragment spectra on the top three most intense precursor ions were produced by electron-transfer dissociation (ETD) fragmentation using a supplemental relative collisional activation of 15% (EThcD). All charge states bigger than 6 were included with an isolation width of 3 Da, and no dynamic exclusion was applied except for isotopes of the same charge state within ±1.5 m/z. ETD reaction time was set to 10 ms with a target of 7 × 10^5^ anions and a maximum injection time of 200 ms. Fragment spectra were recorded within 200–2000 m/z, an AGC of 10^6^, and a maximum injection time of 250 ms by combining 5 microscans.

### 4.4. Tandem Mass Spectrometry Data Analysis

Top-down data were analyzed using the TopPIC suite [138]. First, Thermo RAW files were converted to centroided mzXML files using MSConvert ([139], version 3.0.11781). The resulting files were used for deconvolution with TopFD (version 1.1.2) applying the following settings: maximum charge, 15; maximum monoisotopic mass, 15,000 Da; error tolerance, 0.01 m/z; signal/noise ratio, 1; precursor window size, 3 m/z. The output files were used for identification of proteoforms with TopPIC (version 1.1.0) by searching the spectra against a database containing the sequences of all peptides and proteins identified in the transcriptome of *C. salei* supplemented with all *C. salei* venom peptides on UniprotKB (27 August 2018). We used the program’s default settings with the following exceptions: error tolerance, 10 ppm; N-terminal form, NONE; fixed modification, carbamidomethylation of Cys (unimod: 4); and variable modification, oxidation of Met (unimod: 35), and Gly-loss + Amide (unimod: 822). Protein spectrum matches were filtered applying a false discovery rate (FDR) cut-off of 1%. Protein match tables and detailed analysis results are available as supporting information (Supplementary Dataset EV1, Supplementary Dataset EV2).

For analysis of the bottom-up proteomic data, fragment spectra peak list files were generated as mzXML files with MSConvert (version 3.0.18160) using the vendor centroid option. Interpretation of fragment spectra was done with the search engines Comet (version 2018.01 rev. 2) [140], Xtandem (version Jackhammer TPP (2013.06.15.1)) [141], MSGF+ (version 2018.06.18) [142] and Myrimatch Bumbershoot (release-3_0) [143] against all protein sequences on Uniprot KB matching the search term [taxonomy: “Araneae (spiders) [6893]” venom] (version 27 August 2018) supplemented with sequences of neurotoxins and proteins identified in the transcriptome of *C. salei* and possible contaminants (n_tot_ = 3587). The search parameters were: sample enzyme trypsin with semi-tryptic peptides allowed, and chymotrypsin with semi-chymotryptic peptides allowed, respectively; fixed modification of carbamidomethylation on Cys (unimod: 4); variable modifications of oxidation on Met (unimod: 35), Gly-loss + Amide (unimod: 822) and protein N-terminal acetylation (unimod: 1); maximum missed cleavages of 2; precursor and fragment ion tolerance set to 10 ppm and 0.4 Da, respectively. Statistical validations of peptide identifications were performed using Peptide Prophet [144] implemented in TPP version 5.1 [145], and IProphet [146] to combine the results of the four search engines. Hits were filtered at an FDR of 1% and used for Protein Prophet [147] protein reconstruction. Protein hits were also filtered at an FDR of 1%. Protein and peptide match tables are available as supporting information (Supplementary Dataset EV3).

### 4.5. Sequence Analysis

The Bayesian tree was estimated from a cropped and manually validated amino acid sequence alignment of *C. salei* peptides of the SN_02 family and homologues identified by BLASTP. We ran Mr. Bayes (version 3.2.6) [148] for 5,000,000 generations using the Dayhoff rate matrix and gamma-distributed across-site rate variations (best substitution model as determined using MEGA 7.0.26 [149]). Trees were sampled every 1000 generations. Other parameters were left at default values. Sampled model parameters and trees were summarized using a relative burnin of 50%. Calculations were performed on UBELIX (http://www.id.unibe.ch/hpc), the HPC cluster at the University of Bern.

### 4.6. Sequence Deposition

The nucleotide sequences referred to in this manuscript were deposited in the GenBank database under GenBank Accession Numbers MH754547–MH754628, MH766616–MH766649, and MH795777–MH795789.

### 4.7. Proteomic Data Deposition

The mass spectrometry proteomics data were deposited to the ProteomeXchange Consortium via the PRIDE [150] partner repository with the dataset identifier PXD012886 (top-down data) and PXD012881 (bottom-up data).

## Figures and Tables

**Table 1:** Overview of the main identified proteins in the venom glands of *C. salei*.

Protein	TPM	Transcripts/Precursors (n)/(n)	Length Signal Peptide (aa)	Length Mature Protein (aa)	Molecular Mass * (kDa) (pI) **	InterProScan ID Predicted Functional Role	Proteomic Coverage [%] [Trypsin/Chymo-trypsin]
Signal peptidase catalytic subunit (SPase)	7.97	1/1		177	20.0 (9.21)	IPR001733 Specific peptidase activity	[-/-]
Protein disulfide isomerase (PDI)	14,970.57	3/2	16	479	54.2 (4.77) 54.1 (4.81)	IPR005792 PDI family Isomerase activity	[81.6/60.2] [80.8/56.0]
Venom serine protease PQM protease (VSP1_a) A0A2I5YNR5 A0A2I5YNR1	10,970.57	3/2	18	260	28.0 (7.03) 28.0 (7.66)	IPR001314 IPR001254 PQM serine protease	[91.1/81.8] [91.1/91.1]
Venom serine protease 1_b1/b2 PQM protease (VSP1_b)	12,251.37	2/2	18	261	28.3 (5.84) 28.3 (6.12)	IPR001314 IPR001254 Serine protease	[86.8/84.3] [86.8/77.9]
Venom serine protease 2 (VSP2)	3476.51	3/1	16	281	31.0 (8.03)	IPR001314 IPR001254 Serine protease	[85.2/71.0]
Carboxypeptidase A-like protein (CPA)	72.70	2/1	18	454	53.1 (6.55)	IPR000834 IPR008969 Carboxypeptidase A	[89.4/66.1]
Peptidylglycine alpha-amidating monooxygenase (PAM)	153.60	1/1	25	328	36.9 (5.13)	IPR000720 C-terminal amidating activity	[82.4/79.9]
α-amylase (α-AMY)	57,070.97	>1	21	510	57.6 (6.61)	IPR006046) Alpha amylase	[92.2/89.3]
Cysteine-rich secretory protein (CRISP1)	19,808.52	2/2	26	418	46.6 (7.54) 46.5 (7.54)	IPR001283 IPR002413 CAP domain IPR014044	[94.1/93.2] [94.1/93.2]
Cysteine-rich secretory protein (CRISP2)	2121.07	1/1	19	396	44.0 (5.93)	IPR001283 IPR002413 CAP domain IPR014044	[90.8/82.2]
Angiotensin-converting enzyme (ACE)	4795.97	4/4	20	614	70.9 (5.82)	IPR033591 Metallocarboxy-peptidase activity	[91.3/86.8] [91.3/86.8] [91.3/86.8] [91.3/86.8]
Hyaluronidase-like (HYAL) tr_A0A0S4JYH2	3040.68	1/1	16	378	43.9 (8.56)	IPR018155 O-glycosyl-hydrolase activity	[93.7/85.0]
Phospholipase A2 (PLA2)	60.17	1/1	20	150	16.9 (5.04)	IPR001211 Cell membrane lysis	[62.9/47.6]
Cystatin (CST)	559.42	3/2	20	116	13.2 (8.80) 13.2 (6.90)	IPR027214 Cysteine-type endopeptidase inhibitor activity	[50.7/49.3] [60.3/49.3]
Kunitz domain-containing protein (KCP)	88.19	2/2	16	185	20.6 (4.22) 20.5 (4.22)	IPR002223 Serine-type endopeptidase inhibitor activity	[60.7/44.3] [60.7/27.9]
TT1LP Thyroglobulin type-1-like protein (TT1LP)	392.26	4/2	18	133	14.9 (4.42) 14.9 (4.42)	IPR00716	[90.1/84.1] [88.1/77.5]
Insulin-like growth factor-binding protein-related protein 1 (IGFBP-rP1) G4V4G1	142.67	1/1	17	244	26.7 (5.00)	IPR000867 IGFBP-like IPR002350 Kazal_dom IPR007110 Ig-like_domain	[11.9/-]
Tachylectin 5A-like (TL5A)	1615.50	2/2	18	286	32.4 (5.93) 32.4 (5.93)	IPR002181 Innate immunity	[71.7/53.9] [71.7/53.9]
Leucine-rich repeat protein (LRR)	7994.70	2/1	19	297	34.1 (5.29)	IPR032675Leucine-rich repeat domain; innate immunity	[83.5/78.2]

Proteins belonging to the neurotoxic protein and peptide machinery are highlighted in dark gray, recruited and neofunctionalized proteins are not highlighted, and possible proteins belonging to the innate immune system are in light gray. PQM, processing quadruplet motif, * Molecular mass of the mature peptide (Da), ** Isoelectric point (pI). The proteomic coverage is indicated in % of the full precursor (including signal and pro-peptide, where applicable). Coverage is highlighted in red, if the coverage is not prototypic to the respective protein isoform.

**Table 2:** Overview of the 56 identified (putative) neurotoxins und their cysteine framework in the venom glands of *C. salei*.

SN_Peptide Family_Group	*Cupiennius salei* Neurotoxin	Arachnoserver Nomenclature	Mature Toxins N	Cysteine Framework
***Neurotoxins exhibiting N-terminal ICK fold and C-terminal α-helix***				
SN_19_13 SN_19_14 SN_19_12	CsTx-1a,b,c CsTx-10a,b CsTx-11a,b CsTx-13a,b CsTx-12a,b CsTx-8a,b	Omega-ctenitoxin-Cs1a,,c U_5_-ctenitoxin-Cs1a,b U_6_-ctenitoxin-Cs1a,b U_2_-ctenitoxin-Cs1a,b U_3_-ctenitoxin-Cs1a,b U_4_-ctenitoxin-Cs1a,b	2 [1] 2 [2] 2 * [1] 1 [1] 2 [2] 1 [1]	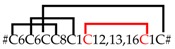
***Neurotoxins exhibiting ICK fold***				
SN_19_13	CsTx-9a,b,c CsTx-33a,b	U_1_-ctenitoxin-Cs1a,b,c U_7_-ctenitoxin-Cs1a,b	2 [2] 1 [1]	#C6C6CC8C1C13C1C#
SN_19_06	CsTx-23a,b	U_8_-ctenitoxin-Cs1a,b	2 [2]	#C6C6CC4C1C13C1C#
SN_02_03	CsTx-17 CsTx-31a,b,c,d CsTx-25 CsTx-28a,b	U_9_-ctenitoxin-Cs1 U_10_-ctenitoxin-Cs1a,b,c,d U_11_-ctenitoxin-Cs1 U_12_-ctenitoxin-Cs1a,b	1 [1] 3 [2] 1 [1] 2 [2]	#C6C6CC4C1C6C1C#
SN_02_04	CsTx-36a,b,c	U_13_-ctenitoxin-Cs1a,b,c	1 [1]	#C6C4CC4C1C6C1C#
SN_02_07	CsTx-18a,b CsTx-19	U_15_-ctenitoxin-Cs1a,b U_16_-ctenitoxin-Cs1	2 * [1] 1 [1]	#C4C6CC4C1C4C1C#
SN_02_16	CsTx-34a,b	U_14_-ctenitoxin-Cs1a,b	1 [1]	#C6C6CC4C1C10C1C#
SN_33_00	CsTx-26	U_17_-ctenitoxin-Cs1	1 [1]	#C6C4CC4C1C6C1C#
SN_29_01	CsTx-30	U_18_-ctenitoxin-Cs1	1 [1]	#C6C6CC4C1C15C1C#
SN_34_00	CsTx-29	U_19_-ctenitoxin-Cs1	1 [1]	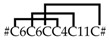
SN_35_00	CsTx-35a,b	U_20_-ctenitoxin-Cs1a,b	2 [2] **	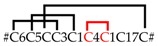
SN_04_04	CsTx-42a,b,c,d CsTx-41	U_21_-ctenitoxin-Cs1a,b,c,d U_22_-ctenitoxin-Cs1	3 [2] 1 [1]	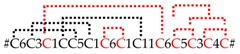
SN_05_07 SN_05_08	CsTx-37 CsTx-38a,b,c	U_23_-ctenitoxin-Cs1 U_24_-ctenitoxin-Cs1a,b,c	1 [1] 3 [3]	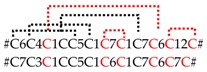
SN_31_04	CsTx-24a,b,c CsTx-39	U_25_-ctenitoxin-Cs1a,b,c U_26_-ctenitoxin-Cs1	2 [1] 1 [1]	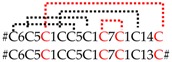
***Unknown cystine fold***				
SN_44_00	CsTx-27	U_27_-ctenitoxin-Cs1	1 [1]	#C6C1C6C1C10C1C13C#
SN_42_00	CsTx-40	U_28_-ctenitoxin-Cs1	1 [1]	#C6C1C6C1C10C1C7C5C19C
***Colipase MIT1-like fold***				
SN_20_01	CsTx-20a,b	U_29_-ctenitoxin-Cs1a,b	2 [2]	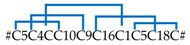
SN_32_01 SN_32_02	CsTx-21a,b,c,d, e,f,g CsTx-22a,b,c	U_30_-ctenitoxin-Cs1a,b,c,d,e,f,g U_31_-ctenitoxin-Cs1a,b,c	6 [5] 3 [3]	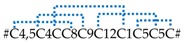

Disulfide bonds involved in the inhibitor cysteine knot (ICK) fold are in black, and additional disulfide bonds are given in red. Predicted cysteine frame works are in dash lines [84]. Colipase MIT1-like fold is given in blue and the predicted fold is given in homology [85]. * one mature sequence only identified by Edman degradation. ** isoforms differ in I<->L only, indistinguishable by MS. [] verified by top-down mass spectrometry.

## Supplementary Materials

The following are available online at https://www.mdpi.com/2072-6651/11/3/167/s1, Supplementary Table S1: Overview on precursors and mature (putative) neurotoxins identified in the transcriptome and proteome of *Cupiennius salei* venom glands; Supplementary Figure S1 (S1.1–S1.19): Multiple sequence alignment of identified venom proteins; Supplementary Figure S2 (S2.1–S2.21): Multiple sequence alignment of identified neurotoxin-like peptides; Supplementary Figure S3 (S3.1–S3.3): Multiple sequence (cDNA) alignment of signal peptides and pro-peptides of SN_19 family peptides; Supplementary Figure S4: Venn diagram of identified (putative) neurotoxins; Supplementary Figure S5: Anion exchange chromatography of *C. salei* venom and synthetic pro-peptides of the neurotoxins CsTx-1 (EIEDDFLEDESFEAEDIIPFFENEQA) and CsTx-12 (ETEDDFLEDESFEADDVIPFLAREQVR); Supplementary Figure S6: Venomics workflow; Supplementary Figure S7: Separation of *C. salei* venom for proteomics; Supplementary Table S1: Overview on precursors and mature (putative) neurotoxins identified in the transcriptome and proteome of *Cupiennius salei* venom glands; Supplementary Dataset EV1: Top-Down Proteomics html result files; Supplementary Dataset EV2: Top-Down proteomics protein and spectrum match tables; Supplementary Dataset EV3: Bottom-up proteomics peptide spectrum match and protein hit tables.
