# Supplementary material for: The Dual Prey-Inactivation Strategy of Spiders—In-Depth Venomic Analysis of Cupiennius salei
Source: Toxins (Basel). 2019 Mar 19;11(3):167. doi: 10.3390/toxins11030167 (PMC6468893; doi:10.3390/toxins11030167)
Supplement: Supplementary file 1 [file toxins-11-00167-s001.zip › Supplementary Table S1/Supplementary Table S1.pdf]

Supplementary Table S1. Overview on precursors and mature (putative) neurotoxins identified in the transcriptome and proteome of *Cupiennius salei* venom glands

						Mature peptide<br><br>STOP CODON	variants			Proteomic coverage [% mature peptide]<br>*= proteotypic in mature peptide not possible (non-silent mutation in signal/pro or I<->L in mature peptide)<br>red= non-proteotypic in mature peptide<br>C-termainally removed residues (e.g. Gly for amidation) do not count for coverage			
Family--group	CSTX-family variants	Signal Peptide (SP)	Propeptide (PP)	cleavage motif	Mature toxin (MT)	MP n	Precursor	Transcript	Counts Reads MP	trypsin	chymotrypsin	combined	Top-down
	1				6 6 8 1 13 1	2	∑=3	∑=5	∑=643				
SN19_13	CSTX-1a_S1	MKVLIISAVLFITIFSNISA	EIEDDFLEDESFEAEDIIPFFEN	EQAR	SCIPKHEECTNDKHNCCRKGLFKLKCQCSTFDDESGQPTERCACGRPMGHQ*AIETGLNIFRGLFK*GKKKNKKTKG (31)	TAA	1	1	151	87.8*	100*	100*	X*
SN19_13	CSTX-1a_S2	MKVLIISAVLFITIFSNISA	EIEDDFLEDESFEAEDIIPFFEN	EQAR	SCIPKHEECTNDKHNCCRKGLFKLKCQCSTFDDESGQPTERCACGRPMGHQ*AIETGLNIFRGLFK*GKKKNKKTKG	TAA		1	159				
		top-down			SCIPKHEECTNDKHNCCRKGLFKLKCQCSTFDDESGQPTERCACGRPMGHQAIETGLNIFRGLFKGKKKNKKTK-NH2<br>SCIPKHEECTNDKHNCCRKGLFKLKCQCSTFDDESGQPTERCACGRPMGHQAIETGLNIFR (CsTx-2a)<br>SCIPKHEECTNDKHNCCRKGLFKLKCQCSTFDDESGQPTERCACGRPMGHQAIETGLNIF (CsTx-2b)								
		trypsin	EIEDDFLEDESFEAEDIIPFFEN	EQAR	SCIPKHEECTNDKHNCCRKGLFKLKCQCSTFDDESGQPTERCACGRPMGHQAIETGLNIFRGLFKGKKKNKKTKG								
		chymotrypsin	EIEDDFLEDESFEAEDIIPFFEN	EQAR	SCIPKHEECTNDKHNCCRKGLFKLKCQCSTFDDESGQPTERCACGRPMGHQAIETGLNIFRGLFKGKKKNKKTK-NH2								
SN19_13	CSTX-1b	MKVLIISAVLFITIFSNISA	EIEDDFLEDESFEAEDIIPFLEN	EQAR	SCIPKHEECTNDKHNCCRKGLFKLKCQCSTFDDESGQPTERCACGRPMGHQ*AIETGLNIFRGLFK*GKKKNKKTKG	TAA	1	1	331	87.8*	100*	100*	X*
		top-down			SCIPKHEECTNDKHNCCRKGLFKLKCQCSTFDDESGQPTERCACGRPMGHQAIETGLNIFRGLFKGKKKNKKTK-NH2<br>SCIPKHEECTNDKHNCCRKGLFKLKCQCSTFDDESGQPTERCACGRPMGHQAIETGLNIFR (CsTx-2a)<br>SCIPKHEECTNDKHNCCRKGLFKLKCQCSTFDDESGQPTERCACGRPMGHQAIETGLNIF (CsTx-2b)								
		trypsin	EIEDDFLEDESFEAEDIIPFFEN	EQAR	SCIPKHEECTNDKHNCCRKGLFKLKCQCSTFDDESGQPTERCACGRPMGHQAIETGLNIFRGLFKGKKKNKKTKG								
		chymotrypsin	EIEDDFLEDESFEAEDIIPFLEN	EQAR	SCIPKHEECTNDKHNCCRKGLFKLKCQCSTFDDESGQPTERCACGRPMGHQAIETGLNIFRGLFKGKKKNKKTK-NH2								
SN19_13	CSTX-1c_S1				SCIPKHEECTNDKHNCCRRGLFKLKCQCSTFDDESGQPTERCACGRPMGHQ*AIETGLNIFRGLFK*GKKKNKKTKG	TAA	1	1	1	86.5	94.6	98.6	-
SN19_13	CSTX-1c_S2				SCIPKHEECTNDKHNCCRRGLFKLKCQCSTFDDESGQPTERCACGRPMGHQ*AIETGLNIFRGLFK*GKKKNKKTKG	TAA		1	1				
		top-down			--								
		trypsin			SCIPKHEECTNDKHNCCRRGLFKLKCQCSTFDDESGQPTERCACGRPMGHQAIETGLNIFRGLFKGKKKNKKTKG								
		chymotrypsin			SCIPKHEECTNDKHNCCRRGLFKLKCQCSTFDDESGQPTERCACGRPMGHQAIETGLNIFRGLFKGKKKNKKTK-NH2								
	13/8/12				6 6 8 1 16 1	4(5)	∑=6	∑=8	∑=1176				
SN19_12	CSTX-13a	MKVLVIFAVLSLVIFSNCSA	ETDEDFFGEESFEADDIIPFIAK	EQVR	SDCTLRNHDCTDDRHSCCRSKMFKDVCTCFYPSQRSETARAKKELCTCQQPKH*LKYIEKGLQKA*KDYATG (22)	TAA	1	1	157	84.1	100	100	X
		top-down			SDCTLRNHDCTDDRHSCCRSKMFKDVCTCFYPSQ<br>AKKELCTCQQPKH*LKYIEKGLQKA*KDYAT-NH2								
		trypsin	ETDEDFFGEESFEADDIIPFIAK	EQVR	SDCTLRNHDCTDDRHSCCRSKMFKDVCTCFYPSQRSETARAKKELCTCQQPKH*LKYIEKGLQKA*KDYATG								
		chymotrypsin	ETDEDFFGEESFEADDIIPFIAK	EQVR	SDCTLRNHDCTDDRHSCCRSKMFKDVCTCFYPSQRSETARAKKELCTCQQPKH*LKYIEKGLQKA*KDYAT-NH2								
SN19_12	CSTX-13b	MKVLVIFAVLSLVIFSNCSA	ETDEDFFGEESFEADDIIPFIAK	EQVR	SDCTLRNHDCTDDRHSCCRSKMFKDVCTCFYPSQRSETDRAKKELCTCQQPKH*LKYIEKGLQKA*KDYATG	TAA	1	1	45	84.1	98.6	98.6	X
		top-down			SDCTLRNHDCTDDRHSCCRSKMFKDVCTCFYPSQ<br>AKKELCTCQQPKH*LKYIEKGLQKA*KDYAT-NH2<br>SETDRAKKELCTCQQPKH*LKYIEKGLQKA*KDYAT-NH2								
		trypsin	ETDEDFFGEESFEADDIIPFIAK	EQVR	SDCTLRNHDCTDDRHSCCRSKMFKDVCTCFYPSQRSETDRAKKELCTCQQPKH*LKYIEKGLQKA*KDYATG								
		chymotrypsin	ETDEDFFGEESFEADDIIPFIAK	EQVR	SDCTLRNHDCTDDRHSCCRSKMFKDVCTCFYPSQRSETDRAKKELCTCQQPKH*LKYIEKGLQKA*KDYAT-NH2								
SN19_12	CSTX-8a_S2	MKVLVICAVLFLAIFSNSSA	ETEDDFLEDESFQADDVIPFLAS	EQVR	SDCTLRNHDCTDDRHSCCRSKMFKDVCKCFYPSQRSETDRAKKELCTCQQPKH*LKYIEKGLQKA*KDYATG (22)	TAA	1	1	27	84.1*	98.6*	98.6*	X*
SN19_12	CSTX-8a_S1	MKVLVICAVLFLAIFSNSSA	ETEDDFLEDESFQADDVIPFLAS	EQVR	SDCTLRNHDCTDDRHSCCRSKMFKDVCKCFYPSQRSETDRAKKELCTCQQPKH*LKYIEKGLQKA*KDYATG	TAA		1	541				
SN19_12	CSTX-8b	MKVLVICAVLFLSIFSNSSA	ETEDDFLEDESFQADDVIPFLAS	EQVR	SDCTLRNHDCTDDRHSCCRSKMFKDVCKCFYPSQRSETDRAKKELCTCQQPKH*LKYIEKGLQKA*KDYATG	TAA	1	1	s.o.				
		top-down			SDCTLRNHDCTDDRHSCCRSKMFKDVCKCFYPSQ<br>AKKELCTCQQPKH*LKYIEKGLQKA*KDYAT-NH2<br>SETDRAKKELCTCQQPKH*LKYIEKGLQKA*KDYAT-NH2								
		trypsin	ETEDDFLEDESFQADDVIPFLAS	EQVR	SDCTLRNHDCTDDRHSCCRSKMFKDVCKCFYPSQRSETDRAKKELCTCQQPKHLK*YIEKGLQK*AKDYATG								
		chymotrypsin	ETEDDFLEDESFQADDVIPFLAS	EQVR	SDCTLRNHDCTDDRHSCCRSKMFKDVCKCFYPSQRSETDRAKKELCTCQQPKH*LKYIEKGLQKA*KDYAT-NH2								
SN19_12	CSTX-12a_S1	MKVLVICAVLFLTIFSNSSA	ETEDDFLEDESFEADDVIPFLAR	EQVR	SDCTLRNHDCTDDRHSCCRSKMFKDVCKCFYPSQRSDTARAKKELCTCQQDKH*LKFIEKGLQKA*KVLVAG	TAA	1	1	14	81.2	98.6	98.6	X
SN19_12	CSTX-12a_S2	MKVLVICAVLFLTIFSNSSA	ETEDDFLEDESFEADDVIPFLAR	EQVR	SDCTLRNHDCTDDRHSCCRSKMFKDVCKCFYPSQRSDTARAKKELCTCQQDKH*LKFIEKGLQKA*KVLVAG	TAA		1	169				
		top-down			SDCTLRNHDCTDDRHSCCRSKMFKDVCKCFYPSQ<br>AKKELCTCQQDKH*LKFIEKGLQKA*KVLVA-NH2								
		trypsin	ETEDDFLEDESFEADDVIPFLAR	EQVR	SDCTLRNHDCTDDRHSCCRSKMFKDVCKCFYPSQRSDTARAKKELCTCQQDKH*LKFIEKGLQK*AKVLVAG								
		chymotrypsin	ETEDDFLEDESFEADDVIPFLAR	EQVR	SDCTLRNHDCTDDRHSCCRSKMFKDVCKCFYPSQRSDTARAKKELCTCQQDKH*LKFIEKGLQKA*KVLVA-NH2								
SN19_12	CSTX-12b	MKVLVICAVLFLAIFSNSSA	ETEDDFLEDESFEADDVIPFLAR	EQVR	SDCTLRNHDCTDDRHSCCRSKMFKDVCKCFYPSQRSDTARAKKELCTCQQDKH*LKYIEKGLQKA*KVLVAG	TAA	1	1	223	81.2	98.6	98.6	X
		top-down			SDCTLRNHDCTDDRHSCCRSKMFKDVCKCFYPSQ<br>AKKELCTCQQDKH*LKYIEKGLQKA*KVLVA-NH2								
		trypsin	ETEDDFLEDESFEADDVIPFLAR	EQVR	SDCTLRNHDCTDDRHSCCRSKMFKDVCKCFYPSQRSDTARAKKELCTCQQDKHLK*YIEKGLQK*AKVLVAG								
		chymotrypsin	ETEDDFLEDESFEADDVIPFLAR	EQVR	SDCTLRNHDCTDDRHSCCRSKMFKDVCKCFYPSQRSDTARAKKELCTCQQDKHLK*YIEKGLQKA*KVLVA-NH2								
	10/11				6 6 8 1 12 1	3	∑=3	∑=4	∑=42				
SN19_14	CSTX-10a_S1	MKVLVIFAVLSLVIFSNCSA	ETDEDFFGEESFEADDIIPFIAK	EQVR	KDKENCIGKHHECTDDRDNCCKGKLFRYQCQCFKVIDGKKETKRCACVTPLHYKM*AEMAV*SVFKKMFKN (22)	TGA	1	1	18	50.7	91.3	94.2	X
SN19_14	CSTX-10a_S2	MKVLVIFAVLSLVIFSNCSA	ETDEDFFGEESFEADDIIPFIAK	EQVR	KDKENCIGKHHECTDDRDNCCKGKLFRYQCQCFKVIDGKKETKRCACVTPLHYKM*AEMAV*SVFKKMFKN	TGA		1	15				
		top-down			KDKENCIGKHHECTDDRDNCCKGKLFRYQCQCFKVIDGKKETKRCACVTPLHYKMAEMAVSVFKKMFKN								
		trypsin	ETDEDFFGEESFEADDIIPFIAK	EQVR	KDKENCIGKHHECTDDRDNCCKGKLFRYQCQCFKVIDGKKETKRCACVTPLHYKMAEMAVSVFKKMFKN								
		chymotrypsin	ETDEDFFGEESFEADDIIPFIAK	EQVR	KDKENCIGKHHECTDDRDNCCKGKLFRYQCQCFKVIDGKKETKRCACVTPLHYKMAEMAVSVFKKMFKN								
SN19_14	CsTx-10b	MKVLVIFAVLSLVIFSNCSA	ETDEDFFGEESFEADDIIPFIAK	EQVR	KDKENCIGKHHECTNDRDNCCKGKLFRYQCQCFKVIDGKKETERCACVTPLHYKM*AEMAV*SVFKKMFKN	TGA	1	1	3	50.7	91.3	94.2	-
		top-down			--								
		trypsin	ETDEDFFGEESFEADDIIPFIAK	EQVR	KDKENCIGKHHECTNDRDNCCKGKLFRYQCQCFKVIDGKKETERCACVTPLHYKMAEMAVSVFKKMFKN								
		chymotrypsin	ETDEDFFGEESFEADDIIPFIAK	EQVR	KDKENCIGKHHECTNDRDNCCKGKLFRYQCQCFKVIDGKKETERCACVTPLHYKMAEMAVSVFKKMFKN								
SN19_14	CSTX-11a	MKVLVIFAVLSLVIFSNCSA	ETDEDFFGEESFEADDIIPFIAK	EQVR	KDKENCIGKHHECTNDRDSCCKGKLFRYQCQCFKVIDGKKETERCACVTPLHYKM*AEMAV*SVFKKMFKN	TGA	1	1	6	50.7	91.3	94.2	X
		top-down			KDKENCIGKHHECTNDRDSCCKGKLFRYQCQCFKVIDGKKETERCACVTPLHYKM*AEMAV*SVFKKMFKN								

		trypsin	ETDEDFFGEESFEADDIIPFIAK	EQVR	KDKENCIGKHHECTNDRDSCCKGKLFRYQCQCFKVIDGKKETERCACVTPLHYKMAEMAVSVFKKMFKN								
		chymotrypsin	ETDEDFFGEESFEADDIIPFIAK	EQVR	KDKENCIGKHHECTNDRDSCCKGKLFRYQCQCFKVIDGKKETERCACVTPLHYKMAEMAVSVFKKMFKN								
	9				6 6 8 1 13 1	2	∑=3	∑=4	∑=163				
SN19_13	CSTX-9a	MKVLVICAVLFLAIFSNSSA	ETEDDFLEDESFEADDVIPFLAR	EQVR	KDDKNCIPKHHECTNDKKNCCKKGLTKMKCKCFTVADAKGATSERCACDSSLLQKFGFTGLHIIKGLF (20)	TAA	1	1	20				
SN19_13	CSTX-9b	MKVLVICAVLFLTIFSNSSA	ETEDDFLEDESFEADDVIPFLAR	EQVR	KDDKNCIPKHHECTNDKKNCCKKGLTKMKCKCFTVADAKGATSERCACDSSLLQKFGFTGLHIIKGLF	TAA	1	1	102	73.5*	100*	100*	X*
	CsTx-7				KDDKNCIPKHHECTNDKKNCCKKGLTKMKCKCFTVADAKGATSERCACDSSLLQKFGFTGLHIIKGL								
		top-down			KDDKNCIPKHHECTNDKKNCCKKGLTKMKCKCFTVADAKGATSERCACDSSLLQKFGFTGLHIIKGLF								
		trypsin	ETEDDFLEDESFEADDVIPFLAR	EQVR	KDDKNCIPKHHECTNDKKNCCKKGLTKMKCKCFTVADAKGATSERCACDSSLLQKFGFTGLHIIKGLF								
		chymotrypsin	ETEDDFLEDESFEADDVIPFLAR	EQVR	KDDKNCIPKHHECTNDKKNCCKKGLTKMKCKCFTVADAKGATSERCACDSSLLQKFGFTGLHIIKGLF								
SN19_13	CSTX-9c_S1	MKVLVICAVLFLAIFSNSSA	ETEDDFLEDESFQADDVIPFLAS	EQVR	KDDKNCIPKHHECTNDKKNCCKKGLLKLKCQCFTVADEKGTPSERCACGRPLLHKIAYTGTKMIKGLL	TAA	1	1	41	57.3	100	100	X
SN19_13	CSTX-9c_S2	MKVLVICAVLFLAIFSNSSA	ETEDDFLEDESFQADDVIPFLAS	EQVR	KDDKNCIPKHHECTNDKKNCCKKGLLKLKCQCFTVADEKGTPSERCACGRPLLHKIAYTGTKMIKGLL	TAA		1	s.o.				
		top-down			KDDKNCIPKHHECTNDKKNCCKKGLLKLKCQCFTVADEKGTPSERCACGRPLLHKIAYTGTKMIK								
		trypsin	ETEDDFLEDESFQADDVIPFLAS	EQVR	KDDKNCIPKHHECTNDKKNCCKKGLLKLKCQCFTVADEKGTPSERCACGRPLLHKIAYTGTKMIKGLL								
		chymotrypsin	ETEDDFLEDESFQADDVIPFLAS	EQVR	KDDKNCIPKHHECTNDKKNCCKKGLLKLKCQCFTVADEKGTPSERCACGRPLLHKIAYTGTKMIKGLL								
	33				6 6 8 1 13 1	1	∑=2	∑=2	∑=10				
SN19_13	CSTX-33a	MKILVICAVLLTTICSKSSA	EIDEDFLKDESFEADGIVPFFAN	EEFR	KDKRNCIPRNQECTIDKRNCCRRGLFKMTCQCMKSNDESGQPTEKCTCRRPRPIFHLLYKGLLKG (17)	TAGTAA	1	1	10	42.1*	34.4*	65.6*	X*
SN19_13	CSTX-33b	MKVLVICAVLLTTICSKSSA	EIDEDFLKDESFEADGIVPFFAN	EEFR	KDKRNCIPRNQECTIDKRNCCRRGLFKMTCQCMKSNDESGQPTEKCTCRRPRPIFHLLYKGLLKG	TAGTAA	1	1					
		top-down			KDKRNCIPRNQECTIDKRNCCRRGLFKMTCQCMKSNDESGQPTEKCTCRRPRPIFHLLYKGLLK-NH2								
		trypsin	EIDEDFLKDESFEADGIVPFFAN	EEFR	KDKRNCIPRNQECTIDKRNCCRRGLFKMTCQCMKSNDESGQPTEKCTCRRPRPIFHLLYKGLLKG								
		chymotrypsin			KDKRNCIPRNQECTIDKRNCCRRGLFKMTCQCMKSNDESGQPTEKCTCRRPRPIFHLLYKGLLKG								
	23				6 6 4 1 13 1	2	∑=2	∑=2	∑=2				
SN19_06	CSTX-23a	MKFLLFTGLVFIVLASLIEA	EA	ESER	ACIEYELECTENKDNCCSDYKCQCYRRFVKGVDKGPSCFCLEKDVKYKPL (10)	TAA	1	1	1	74.0	62.0	82.0	-
		top-down			--								
		trypsin			ACIEYELECTENKDNCCSDYKCQCYRRFVKGVDKGPSCFCLEKDVKYKPL								
		chymotrypsin			ACIEYELECTENKDNCCSDYKCQCYRRFVKGVDKGPSCFCLEKDVKYKPL								
SN19_06	CSTX-23b	MKLLLLTGLFFIVVVSMIEA	EA	ENER	ACIGFELECTKDKKNCCSDYKCQCYRRFVKGVDKGQKCWCLDKDVKYKPL	TAA	1	1	1*	46.0	50.0	66.0	-
		top-down			--								
		trypsin			ACIGFELECTKDKKNCCSDYKCQCYRRFVKGVDKGQKCWCLDKDVKYKPL								
		chymotrypsin			ACIGFELECTKDKKNCCSDYKCQCYRRFVKGVDKGQKCWCLDKDVKYKPL								
2	17				6 6 4 1 6 1	1	∑=1	∑=1	∑=28				
SN02_03	CSTX-17	MWLKVQVLLFVFGLIMLLGVQA	EMDSREIQ	EEAR	GCIPKHKRCTWSGPKCCNNISCHCNISGTLCKCRPGLFGWG (8)	TAA	1	1	28	100	100	100	X
		top-down			GCIPKHKRCTWSGPKCCNNISCHCNISGTLCKCRPGLFGW-NH2								
		trypsin			GCIPKHKRCTWSGPKCCNNISCHCNISGTLCKCRPGLFGW-NH2								
		chymotrypsin	EMDSREIQ	EEAR	GCIPKHKRCTWSGPKCCNNISCHCNISGTLCKCRPGLFGW								
	31				6 6 4 1 6 1	3	∑=4	∑=4	∑=14				
SN02_03	CSTX-31a	MLLKVQVLLFLVGFTMLLGVHA	ETDSSEIT	EEAR	GCVQAGKPCTWGKTKCCGGIICKCNYSKTKCHCKPPSLDFGLG (10)	TAA	1	1	8	88.1*	100*	100*	X*
		top-down			GCVQAGKPCTWGKTKCCGGIICKCNYSKTKCHCKPPSLDFGL-NH2								
		trypsin	ETDSSEIT	EEAR	GCVQAGKPCTWGKTKCCGGIICKCNYSKTKCHCKPPSLDFGL-NH2								
		chymotrypsin	ETDSSEIT	EEAR	GCVQAGKPCTWGKTKCCGGIICKCNYSKTKCHCKPPSLDFGL								
SN02_03	CSTX-31b	MLLKVQVLLFLVGFTMLLGVHA	ETDSSEIT	EEAR	GCVQAGKPCTWGKTKCCGGIICKCNYSKTKCNCKPPSLDFGLG	TAA	1	1	2	88.1	64.2	97.6	X
		top-down			GCVQAGKPCTWGKTKCCGGIICKCNYSKTKCNCKPPSLDFGL-NH2								
		trypsin	ETDSSEIT	EEAR	GCVQAGKPCTWGKTKCCGGIICKCNYSKTKCNCKPPSLDFGL-NH2								
		chymotrypsin	ETDSSEIT	EEAR	GCVQAGKPCTWGKTKCCGGIICKCNYSKTKCNCKPPSLDFGLG								
SN02_03	CSTX-31c	MLLKVLVLLFLVGFNMLLGVHA	ETDSSEIT	EEAR	GCVQAGKPCTWGKTKCCGGIICKCNYSKTKCHCKPPSLDFGLG	TAA	1	1	3	88.1*	100*	100*	X*
		top-down			GCVQAGKPCTWGKTKCCGGIICKCNYSKTKCHCKPPSLDFGL-NH2								
		trypsin	ETDSSEIT	EEAR	GCVQAGKPCTWGKTKCCGGIICKCNYSKTKCHCKPPSLDFGL-NH2								
		chymotrypsin	ETDSSEIT	EEAR	GCVQAGKPCTWGKTKCCGGIICKCNYSKTKCHCKPPSLDFGL								
SN02_03	CSTX-31d	MLLKVQVLLFLVGFTMLLGVHA	ETDSSEIT	EEAR	GCVQAGKPCTWGQNKCCGGIICKCNYSKTKCHCKPPSLDFGLG	TAA	1	1	1*	78.6	90.5	90.5	-
		top-down			--								
		trypsin	ETDSSEIT	EEAR	GCVQAGKPCTWGQNKCCGGIICKCNYSKTKCHCKPPSLDFGL-NH2								
		chymotrypsin	ETDSSEIT	EEAR	GCVQAGKPCTWGQNKCCGGIICKCNYSKTKCHCKPPSLDFGL								
0	25				6 6 4 1 6 1	1	∑=1	∑=1	∑=2				
SN02_03	CSTX-25	MWPKVQVLLVLVGLIMFLGVHA	DTESSEIT	EESR	YCIPKWRRCTWGGPKCCAGRSCDCNVTRTNCRCSPRLFGLG (8)	TAA	1	1	2	20.0	70.0	82.5	X
		top-down			YCIPKWRRCTWGGPKCCAGRSCDCNVTRTNCRCSPRLFGL-NH2								
		trypsin	DTESSEIT	EESR	YCIPKWRRCTWGGPKCCAGRSCDCNVTRTNCRCSPRLFGLG								
		chymotrypsin	DTESSEIT	EESR	YCIPKWRRCTWGGPKCCAGRSCDCNVTRTNCRCSPRLFGLG								
0	28				6 6 4 1 6 1	2	∑=2	∑=2	∑=2				
SN02_03	CSTX-28a	MLHKVQVFLFVVGLIMLLGVDA	EIDSSENL	EEAR	ECIQPYKTCSSGGTRCCGRASCRCPISGSNCKCTKPVSQIIADWFSG (14)	TGA	1	1	1	100	100	100	X
		top-down			ECIQPYKTCSSGGTRCCGRASCRCPISGSNCKCTKPVSQIIADWFS-NH2								
		trypsin	EIDSSENL	EEAR	ECIQPYKTCSSGGTRCCGRASCRCPISGSNCKCTKPVSQIIADWFS-NH2								
		chymotrypsin	EIDSSENL	EEAR	ECIQPYKTCSSGGTRCCGRASCRCPISGSNCKCTKPVSQIIADWFS-NH2								
SN02_03	CSTX-28b	MLHKVQAFLFVFGLIMLLGVNA	EIDSSENL	EEAR	ECIEPYKTCSWSGTRCCGRASCKCSIAGTNCKCTKPASQIIADWFSG	TGA	1	1	1*	100	91.3	100	X
		top-down			ECIEPYKTCSWSGTRCCGRASCKCSIAGTNCKCTKPASQIIADWFS-NH2								
		trypsin	EIDSSENL	EEAR	ECIEPYKTCSWSGTRCCGRASCKCSIAGTNCKCTKPASQIIADWFS-NH2								
		chymotrypsin	EIDSSENL	EEAR	ECIEPYKTCSWSGTRCCGRASCKCSIAGTNCKCTKPASQIIADWFS-NH2								
	36				6 4 4 1 6 1	1	∑=3	∑=4	∑=9				
SN02_04	CSTX-36a_S1	MRLLFPILMVVVAFIAVIGVHG	TAYSNENFENDPEGNR	EGNR	SCAEAYQTCDSIPCCNERSCVCNWLGKECKCKKSLGELIDTLLGS (14)	TAA	1	1	2				
	CSTX-36a_S2	MRLLFPILMVVVAFIAVIGVHG	TAYSNENFENDPEGNR	EGNR	SCAEAYQTCDSIPCCNERSCVCNWLGKECKCKKSLGELIDTLLGS	TAA		1	1	95.6*	53.3*	95.6*	X*
SN02_04	CSTX-36b	MRLLIPILMVVVAFIAVIGVHA	TAYSNENFENDPEGNR	EGNR	SCAEAYQTCDSIPCCNERSCVCNWLGKECKCKKSLGELIDTLLGS	TAA	1	1	2				
		top-down			SCAEAYQTCDSIPCCNERSCVCNWLGKECKCKKSLGELIDTLLGS								
		trypsin	TAYSNENFENDPEGNR	EGNR	SCAEAYQTCDSIPCCNERSCVCNWLGKECKCKKSLGELIDTLLGS								
		chymotrypsin	TAYSNENFENDPEGNR	EGNR	SCAEAYQTCDSIPCCNERSCVCNWLGKECKCKKSLGELIDTLLGS								
SN02_04	CSTX-36c	MRLLIPILMVVVAFIAVIGVHA	TAYSNDNFENDPEGNR	EGNR	SCAEAYQTCDSIPCCNERSCVCNWLGKECKCKKSLGELIDTLLGS	TAA	1	1	4	95.6*	53.3*	95.6*	X*
		top-down			SCAEAYQTCDSIPCCNERSCVCNWLGKECKCKKSLGELIDTLLGS								
		trypsin	TAYSNDNFENDPEGNR	EGNR	SCAEAYQTCDSIPCCNERSCVCNWLGKECKCKKSLGELIDTLLGS								
		chymotrypsin	TAYSNDNFENDPEGNR	EGNR	SCAEAYQTCDSIPCCNERSCVCNWLGKECKCKKSLGELIDTLLGS								
	34				6 6 4 1 10 1	1	∑=2	∑=4	∑=6				
SN02_16	CSTX-34a_S1	MMKFCHFFALHLVTIVCCSK	DEILENEANAEDTLPVVQG	ENAR	KDCIKRDHSCTHDKNGCCWPQYCDCWSNRRTYEYKCECRRRHLLFLQV (10)	TGA	1	1	1	66.7*	66.7*	83.3*	X*

SN02_16	CSTX-34a_S2	MMKFCHFFALHLVTIVCCSK	DEILENEANAEDTLPVVQG	ENAR	KDCIKRDHSCTHDKNGCCWPQYCDCWSNRRTYEYKCECRRRHLLFLQV	TGA		1	3				
SN02_16	CSTX-34b_S1	MMKFCIFFALHLVTIVCCSK	DEILENEANAEDTLPVVQG	ENAR	KDCIKRDHSCTHDKNGCCWPQYCDCWSNRRTYEYKCECRRRHLLFLQV	TGA	1	1	1*				
SN02_16	CSTX-34b_S2	MMKFCIFFALHLVTIVCCSK	DEILENEANAEDTLPVVQG	ENAR	KDCIKRDHSCTHDKNGCCWPQYCDCWSNRRTYEYKCECRRRHLLFLQV	TGA		1	1*				
		top-down			KDCIKRDHSCTHDKNGCCWPQYCDCWSNRRTYEYKCECRRRHLLFLQV								
		trypsin	DEILENEANAEDTLPVVQG	ENAR	KDCIKRDHSCTHDKNGCCWPQYCDCWSNRRTYEYKCECRRRHLLFLQV								
		chymotrypsin	DEILENEANAEDTLPVVQG	ENAR	KDCIKRDHSCTHDKNGCCWPQYCDCWSNRRTYEYKCECRRRHLLFLQV								
	18				4 6 4 1 4 1	1	∑=1	∑=2	∑=34				
SN02_07	CSTX-18a_S1	MWPKMQVLLFVVGLIMLLGVFA	ETDSSEIT	EETR	GFWIKGNFCLRNRCVPGGRKCCNGMPCQCLGEVCRCPRKLIGKLSALRKHT (15)	TAA	1	1	33	58.8	100	100	X
SN02_07	CSTX-18a_S2	MWPKMQVLLFVVGLIMLLGVFA	ETDSSEIT	EETR	GFWIKGNFCLRNRCVPGGRKCCNGMPCQCLGEVCRCPRKLIGKLSALRKHT	TAA		1	1				
		top-down			GFWIKGNFCLRNRCVPGGRKCCNGMPCQCLGEVCRCPRKLIGKLSALRKHT								
		trypsin	ETDSSEIT	EETR	GFWIKGNFCLRNRCVPGGRKCCNGMPCQCLGEVCRCPRKLIGKLSALRKHT								
		chymotrypsin	ETDSSEIT	EETR	GFWIKGNFCLRNRCVPGGRKCCNGMPCQCLGEVCRCPRKLIGKLSALRKHT								
	19				4 6 4 1 4 1	1	∑=1	∑=2	∑=196				
SN02_07	CSTX-19_S1	MCPKMQVLLLVVGLIMLLGVFA	ETDSSEIT	EETR	NYCVAKRCRPGGRQCCSGKPCACVGKVCKCPRDNS (5)	TAA	1	1	194	65.7	100	100	X
SN02_07	CSTX-19_S2	MCPKMQVLLLVVGLIMLLGVFA	ETDSSEIT	EETR	NYCVAKRCRPGGRQCCSGKPCACVGKVCKCPRDNS	TAA		1	2				
		top-down			NYCVAKRCRPGGRQCCSGKPCACVGKVCKCPRDNS								
		trypsin	ETDSSEIT	EETR	NYCVAKRCRPGGRQCCSGKPCACVGKVCKCPRDNS								
		chymotrypsin	ETDSSEIT	EETR	NYCVAKRCRPGGRQCCSGKPCACVGKVCKCPRDNS								
3	42				6 3 1 5 1 6 1 11 6 5 3 4	3	∑=4	∑=4	∑=19				
SN04_04	CSTX-42a	XXXXXXXXXXXXXXXXXXXXXX	XXXXXXXVIEEGEEALQAEESSRE	TTER	VCVKPKESCDNDCQCCVANEYCYCAFGWWSCTCRPSDNSNRWGVCMKKNRECGLNMTCPKNCTTKRCMGK	TAA	1	1	9	79.4*	79.4*	100*	-
		top-down			--								
		trypsin	VIEEGEEALQAEESSRE	TTER	VCVKPKESCDNDCQCCVANEYCYCAFGWWSCTCRPSDNSNRWGVCMKKNRECGLNMTCPKNCTTKRCMGK								
		chymotrypsin	VIEEGEEALQAEESSRE	TTER	VCVKPKESCDNDCQCCVANEYCYCAFGWWSCTCRPSDNSNRWGVCMKKNRECGLNMTCPKNCTTKRCM-NH2								
SN04_04	CSTX-42d	XXXXXXXXXXXXXXXXXXXXXX	XXXXXXXVIEKGEEALQAEESSRE	TTER	VCVKPKESCDNDCQCCVANEYCYCAFGWWSCTCRPSDNSNRWGVCMKKNRECGLNMTCPKNCTTKRCMGK	TAA	1	1	8	79.4*	79.4*	100*	-
		top-down			--								
		trypsin	VIEKGEEALQAEESSRE	TTER	VCVKPKESCDNDCQCCVANEYCYCAFGWWSCTCRPSDNSNRWGVCMKKNRECGLNMTCPKNCTTKRCMGK								
		chymotrypsin	VIEKGEEALQAEESSRE	TTER	VCVKPKESCDNDCQCCVANEYCYCAFGWWSCTCRPSDNSNRWGVCMKKNRECGLNMTCPKNCTTKRCM-NH2								
SN04_04	CSTX-42b	XXXXXXXXXXXXXXXXXXXXXX	XXXXXXXXXXXXXEAVQAEESSRE	TKER	VCVKPKESCGNDCQCCVDNEYCYCAFIWWSCTCRPSDNSNRWSVCMKKNRECGLNMTCPKNCTTKRCMGK	TAA	1	1	1*	35.7	39.7	48.6	-
		top-down			--								
		trypsin	EAVQAEESSRE	TKER	VCVKPKESCGNDCQCCVDNEYCYCAFIWWSCTCRPSDNSNRWSVCMKKNRECGLNMTCPKNCTTKRCMGK								
		chymotrypsin	EAVQAEESSRE	TKER	VCVKPKESCGNDCQCCVDNEYCYCAFIWWSCTCRPSDNSNRWSVCMKKNRECGLNMTCPKNCTTKRCM-NH2								
SN04_04	CSTX-42c	XXXXXXXXXXXXXXXXXXXXXX	XXXXXXXXXEEGEEALQAEESSRE	TTER	VCVKPKESCDNDCQCCVANEYCYCAFGWWSCTCRPSDNSNRWGVCMKKNRECGLNMTCQKNCTTKRCMGK	TAA	1	1	1*	79.4	58.8	85.7	-
		top-down			--								
		trypsin	EEGEEALQAEESSRE	TTER	VCVKPKESCDNDCQCCVANEYCYCAFGWWSCTCRPSDNSNRWGVCMKKNRECGLNMTCQKNCTTKRCMGK								
		chymotrypsin	EEGEEALQAEESSRE	TTER	VCVKPKESCDNDCQCCVANEYCYCAFGWWSCTCRPSDNSNRWGVCMKKNRECGLNMTCQKNCTTKRCMGK								
	41				6 3 1 5 1 6 1 11 6 5 3 4	1	∑=1	∑=1	∑=1				
SN04_04	CSTX-41	XXXXXXXXXXXXXXXXXXXXXX	XXXXXXXXXXXXXXXXXXXXXX	EMKR	AACTDLGGECQTDCDCCRRNGYCSCSWLFNHCTCQPGSNANSGSICHRKNRECGLRMRCPKNCTTKKCMGK (3)	TAA	1	1	1	71.0	81.2	91.3	X
		top-down			AACTDLGGECQTDCDCCRRNGYCSCSWLFNHCTCQPGSNANSGSICHRKNRECGLRMRCPKNCTTKKCM								
		trypsin		EMKR	AACTDLGGECQTDCDCCRRNGYCSCSWLFNHCTCQPGSNANSGSICHRKNRECGLRMRCPKNCTTKKCMGK								
		chymotrypsin		EMKR	AACTDLGGECQTDCDCCRRNGYCSCSWLFNHCTCQPGSNANSGSICHRKNRECGLRMRCPKNCTTKKCM-NH2								
4	37				6 4 1 5 1 7 1 7 6 12	1	∑=1	∑=1	∑=2				
SN05_07	CSTX-37	MKTALIFLCFLAVVYS	ETESKDTSVREELAPK	EEER	ACVPLGEECNGNDCKCCNKWTYCKCPFGTGFACSCVFGGAMVCERKKKKCKNPEVMNTPPGPCFSGRG (5)	TAA	1	1	2	79.4	16.2	85.3	-
		top-down			--								
		trypsin	ETESKDTSVREELAPK	EEER	ACVPLGEECNGNDCKCCNKWTYCKCPFGTGFACSCVFGGAMVCERKKKKCKNPEVMNTPPGPCFSGRG								
		chymotrypsin			ACVPLGEECNGNDCKCCNKWTYCKCPFGTGFACSCVFGGAMVCERKKKKCKNPEVMNTPPGPCFSGRG								
5	38				7 3 1 5 1 6 1 7 6 7	3	∑=3	∑=5	∑=10				
SN05_08	CSTX-38a		PFEDPDPDLFQAEDSAM	EEPR	GCEYNMGKECKDDCDCCVNISKCNCMLGSYLCSCGYGNRYTCLDKVRKCTKNRPHECPLPDLLPTRGKGPNYGNIMNRLP (23)	TAA	1	1	1	96.3	88.6	100	-
		top-down			--								
		trypsin	PFEDPDPDLFQAEDSAM	EEPR	GCEYNMGKECKDDCDCCVNISKCNCMLGSYLCSCGYGNRYTCLDKVRKCTKNRPHECPLPDLLPTRGKGPNYGNIMNRLP								
		chymotrypsin	PFEDPDPDLFQAEDSAM	EEPR	GCEYNMGKECKDDCDCCVNISKCNCMLGSYLCSCGYGNRYTCLDKVRKCTKNRPHECPLPDLLPTRGKGPNYGNIMNRLP								
SN05_08	CSTX-38b_S1	MKISILFLGVICFFSLQETEG	AGLLNDVGPFEDPDPDLFQAEDSAM	EEPR	GCEYNMGKECKDDCDCCVNISKCNCMLGSYLCSCGYGDRYTCLDKVRKCTKNRPHECPLPDLLPTRGKGPNYGNIMNRLP	TAA	1	1	5	96.3	100	100	X
SN05_08	CsTx-38b_S2	MKISILFLGVICFFSLQETEG	AGLLNDVGPFEDPDPDLFQAEDSAM	EEPR	GCEYNMGKECKDDCDCCVNISKCNCMLGSYLCSCGYGDRYTCLDKVRKCTKNRPHECPLPDLLPTRGKGPNYGNIMNRLP	TAA		1	1				
SN05_08	CsTx-38b_S3	MKISILFLGVICFFSLQETEG	AGLLNDVGPFEDPDPDLFQAEDSAM	EEPR	GCEYNMGKECKDDCDCCVNISKCNCMLGSYLCSCGYGDRYTCLDKVRKCTKNRPHECPLPDLLPTRGKGPNYGNIMNRLP	TAA		1	1				
		top-down			GCEYNMGKECKDDCDCCVNISKCNCMLGSYLCSCGYGDRYTCLDKVRKCTKNRPHECPLPDLLPTRGKGPNYGNIMNRLP								
		trypsin	AGLLNDVGPFEDPDPDLFQAEDSAM	EEPR	GCEYNMGKECKDDCDCCVNISKCNCMLGSYLCSCGYGDRYTCLDKVRKCTKNRPHECPLPDLLPTRGKGPNYGNIMNRLP								
		chymotrypsin	AGLLNDVGPFEDPDPDLFQAEDSAM	EEPR	GCEYNMGKECKDDCDCCVNISKCNCMLGSYLCSCGYGDRYTCLDKVRKCTKNRPHECPLPDLLPTRGKGPNYGNIMNRLP								
SN05_08	CSTX-38c	MKISILFLGVICFFSLQETEG	AGLLNDVGPFEDPDPDLFQAEDSAM	EEPR	GCEYNMGKECKDDCDCCVNISKCNCMLGSYLCSCGYGDRYTCLDKVRKCTKNRPHECPLPDLLPTRGKGPNYSNIVNRLP	TAA	1	1	2	96.3	100	100	-
		top-down			--								
		trypsin	AGLLNDVGPFEDPDPDLFQAEDSAM	EEPR	GCEYNMGKECKDDCDCCVNISKCNCMLGSYLCSCGYGDRYTCLDKVRKCTKNRPHECPLPDLLPTRGKGPNYSNIVNRLP								
		chymotrypsin	AGLLNDVGPFEDPDPDLFQAEDSAM	EEPR	GCEYNMGKECKDDCDCCVNISKCNCMLGSYLCSCGYGDRYTCLDKVRKCTKNRPHECPLPDLLPTRGKGPNYSNIVNRLP								
6	30				6 6 4 1 15 1	1	∑=1	∑=2	∑=4				
SN29_01	CSTX-30_S1	MKLFIFLVLLFITIVHC	EDEIPENEADAEEKSPVAQE	EIAR	DCVRWRRSCMGNQNGCCLPWRCFCWSQTVSRGSTRTEQKCQCRFW (3)	TAA	1	1	2	55.6	93.3	100	-
	CSTX-30_S2	MKLFIFLVLLFITIVHC	EDEIPENEADAEEKSPVAQE	EIAR	DCVRWRRSCMGNQNGCCLPWRCFCWSQTVSRGSTRTEQKCQCRFW	TAA		1	2				
		top-down			--								
		trypsin	EDEIPENEADAEEKSPVAQE	EIAR	DCVRWRRSCMGNQNGCCLPWRCFCWSQTVSRGSTRTEQKCQCRFW								
		chymotrypsin	EDEIPENEADAEEKSPVAQE	EIAR	DCVRWRRSCMGNQNGCCLPWRCFCWSQTVSRGSTRTEQKCQCRFW								
7	24				6 5 1 5 1 7 1 14	2	∑=3	∑=3	∑=5				
SN31_04	CSTX-24a	MKLAIIALFSLVVLAVA	SESVEENQEGEFL	EQQR	ACAERKEKCTKDDDCSCCGKWDKCSCNWPGREGCFCMRGMMATRLWKMAKC	TGA	1	1	3	58.9*	90.2*	90.2*	X*
		top-down			ACAERKEKCTKDDDCSCCGKWDKCSCNWPGREGCFCMRGMMATRLWKMAKC								
		trypsin	SESVEENQEGEFL	EQQR	ACAERKEKCTKDDDCSCCGKWDKCSCNWPGREGCFCMRGMMATRLWKMAKC								
		chymotrypsin	SESVEENQEGEFL	EQQR	ACAERKEKCTKDDDCSCCGKWDKCSCNWPGREGCFCMRGMMATRLWKMAKC								
SN31_04	CSTX-24b	MKLAIIALFSLVVLAVA	SESVEENQEGEFL	EQQR	ACAERKKKCTKDDDCSCCGKWDKCSCNWPGREGCFCMRGMMATRLWKMAKC	TGA	1	1	1	58.9	74.5	74.5	-
		top-down			--								
		trypsin	SESVEENQEGEFL	EQQR	ACAERKKKCTKDDDCSCCGKWDKCSCNWPGREGCFCMRGMMATRLWKMAKC								
		chymotrypsin	SESVEENQEGEFL	EQQR	ACAERKKKCTKDDDCSCCGKWDKCSCNWPGREGCFCMRGMMATRLWKMAKC								
SN31_04	CSTX-24c	MKLAIIALFSIVVLAVA	SESVEENQEGEFL	EQQR	ACAERKEKCTKDDDCSCCGKWDKCSCNWPGREGCFCMRGMMATRLWKMAKC	TGA	1	1	1	58.2*	90.2*	90.2*	X*
		top-down			ACAERKEKCTKDDDCSCCGKWDKCSCNWPGREGCFCMRGMMATRLWKMAKC								
		trypsin	SESVEENQEGEFL	EQQR	ACAERKEKCTKDDDCSCCGKWDKCSCNWPGREGCFCMRGMMATRLWKMAKC								

		chymotrypsin	SESVEENQEGEFL	EQQR	ACAERKEKCTKDDDCSCCGKWDKCSCNWPGREGCFCMRGMMATRLWKMAKC								
	39				6 5 1 5 1 7 1 13	1	Σ=1	Σ=1	Σ=1				
SN31_04	CSTX-39	MKLAIIMLFSLVLLAVA	SESIEESRKEEYP	EEVR	SCADRKEKCTKGDDCSCCGAWNKCDCNWPDRPGCYCMRGMMATRISKMKCRKGKSK	TGA	1	1	1*	58.2	87.3	87.3	X
		top-down			SCADRKEKCTKGDDCSCCGAWNKCDCNWPDRPGCYCMRGMMATRISKMKCRKGKS								
		trypsin	SESIEESRKEEYP	EEVR	SCADRKEKCTKGDDCSCCGAWNKCDCNWPDRPGCYCMRGMMATRISKMKCRKGKSK								
		chymotrypsin	SESIEESRKEEYP	EEVR	SCADRKEKCTKGDDCSCCGAWNKCDCNWPDRPGCYCMRGMMATRISKMKCRKGKSK								
					6 4 4 1 6 1	1	Σ=1	Σ=1	Σ=16				
SN33_00	CSTX-26	MKVSTVFGLCIVAFLLLSIVDVSDA	EEIDSEKA	TEER	GYCAEKNIKCNDIHCCTGLKCKCNSSRTNCVCRKE (3)	TAA	1	1	16	31.4	54.3	57.1	X
		top-down		¤	GYCAEKNIKCNDIHCCTGLKCKCNSSRTNCVCRKE								
		trypsin	EEIDSEKA	TEER	GYCAEKNIKCNDIHCCTGLKCKCNSSRTNCVCRKE								
		chymotrypsin	EEIDSEKA	TEER	GYCAEKNIKCNDIHCCTGLKCKCNSSRTNCVCRKE								
9	29				6 6 4 11	1	Σ=1	Σ=2	Σ=5				
SN34_00	CSTX-29_S1	MQNFHGAFFIVLVIVYSVYG	DEADIPEVTVEEV	ESNR	ENCGNVGADCEYEYERCCNDLRCRFTGRTGRLFMCL	TGA	1	1	5	100	91.6	100	X
	CSTX-29_S2	MQNFHGAFFIVLVIVYSVYG	DEADIPEVTVEEV	ESNR	ENCGNVGADCEYEYERCCNDLRCRFTGRTGRLFMCL			1					
		top-down			ENCGNVGADCEYEYERCCNDLRCRFTGRTGRLFMCL								
		trypsin	DEADIPEVTVEEV	ESNR	ENCGNVGADCEYEYERCCNDLRCRFTGRTGRLFMCL								
		chymotrypsin	DEADIPEVTVEEV	ESNR	ENCGNVGADCEYEYERCCNDLRCRFTGRTGRLFMCL								
10	35				6 5 3 1 4 1 17	2	Σ=2	Σ=2	Σ=3				
SN35_00	CSTX-35a	MKSIAYVAALLLLASFTRG	EEEREEMPAGHHIQ	KR	SCNFKGEECTKDVDCCTMQCLCTTNDCKCGKRPRPSELRKYFTKRTCGGT (3)	TGA	1	1	2	78.0*	84.0*	84.0*	X*
SN35_00	CSTX-35b	MKSIAYVAALLLLASFTRG	EEEREEMPAGHHIQ	KR	SCNFKGEECTKDVDCCTMQCICTTNDCKCGKRPRPSELRKYFTKRTCGGT	TGA	1	1	1				
		top-down			SCNFKGEECTKDVDCCTMQCICTTNDCKCGKRPRPSELRKYFTKRTCGGT								
		trypsin	EEEREEMPAGHHIQ	KR	SCNFKGEECTKDVDCCTMQCICTTNDCKCGKRPRPSELRKYFTKRTCGGT								
		chymotrypsin	EEEREEMPAGHHIQ	KR	SCNFKGEECTKDVDCCTMQCICTTNDCKCGKRPRPSELRKYFTKRTCGGT								
11	27				6 1 6 1 10 1 13	1	Σ=1	Σ=1	Σ=17.0				
SN44_00	CSTX-27	MIKYILIFALLIAAVYS	FTIEQDEDLLSEDAEEEVVP	EEAR	FMLRVGAVCDGNKSDCQCLGKWIKCGCPFLWPMRSGPCHCTKGRRYTYNKKLSCSNRYLWASEKSR (12)	TGA	1	1	17	100	92.3	100	X
		top-down			FMLRVGAVCDGNKSDCQCLGKWIKCGCPFLWPMRSGPCHCTKGRRYTYNKKLSCSNRYLWASEKS								
57		trypsin	FTIEQDEDLLSEDAEEEVVP	EEAR	FMLRVGAVCDGNKSDCQCLGKWIKCGCPFLWPMRSGPCHCTKGRRYTYNKKLSCSNRYLWASEKSR								
39		chymotrypsin	FTIEQDEDLLSEDAEEEVVP	EEAR	FMLRVGAVCDGNKSDCQCLGKWIKCGCPFLWPMRSGPCHCTKGRRYTYNKKLSCSNRYLWASEKSR								
	40				6 1 6 1 10 1 7 5 19	1	Σ=1	Σ=1	Σ=2				
SN42_00	CSTX-40	MIKYILISALFISAVYS	FTVEEDEALMPEAAEEEIIP	EEAR	KSLPDGEECDGNKDDCQCYGKWHKCSCPYFWPWRSGKCYCEKGWKHTCITKLSCPNKDEWGLEWRNEEAERSPC	TAA	1	1	2	41.9	-	41.9	-
		top-down			--								
	1	trypsin	FTVEEDEALMPEAAEEEIIP	EEAR	KSLPDGEECDGNKDDCQCYGKWHKCSCPYFWPWRSGKCYCEKGWKHTCITKLSCPNKDEWGLEWRNEEAERSPC								
		chymotrypsin	--	--	--								
12	20				5 4 10 9 16 1 5 18	2	Σ=2	Σ=2	Σ=2				
SN20_01	CSTX-20a	MKVTLAFLVLFGILGVSLA			WNACTKQSDCEEDECCLDNLFFKRPYCEKRYGAEQRCSAAAVYKEDKDLYYFTCPCVPMYECLGKGSLDENGNTVMKNPKCIMPTL	TAA	1	1	1*	80.2	100	100	-
		top-down			--								
76		trypsin			WNACTKQSDCEEDECCLDNLFFKRPYCEKRYGAEQRCSAAAVYKEDKDLYYFTCPCVPMYECLGKGSLDENGNTVMKNPKCIMPTL								
5		chymotrypsin			WNACTKQSDCEEDECCLDNLFFKRPYCEKRYGAEQRCSAAAVYKEDKDLYYFTCPCVPMYECLGKGSLDENGNTVMKNPKCIMPTL								
SN20_01	CSTX-20b	MKVTLAFLVLFGILGVSLA			WNACTKQSDCEEDECCLDNLFFKTPYCEKRYGAGQRCSATAVYKEEKDLYYLTCPCVPMYECLGKGSLDENGNTVMKNPKCIMPTL	TAA	1	1	1*	80.2	100	100	-
		top-down			--								
45		trypsin			WNACTKQSDCEEDECCLDNLFFKTPYCEKRYGAGQRCSATAVYKEEKDLYYLTCPCVPMYECLGKGSLDENGNTVMKNPKCIMPTL								
4		chymotrypsin			WNACTKQSDCEEDECCLDNLFFKTPYCEKRYGAGQRCSATAVYKEEKDLYYLTCPCVPMYECLGKGSLDENGNTVMKNPKCIMPTL								
13	22				5 4 8 9 12 1 5 5	3	Σ=3	Σ=3	Σ=4.0				
SN32_02	CSTX-22a	MKCFIVLAVLATLVLAIQG			KFCSSTSDCGEGMCCTGGSFNRHCQRLSENGRPCQRPNDQDYYSTGCPCKEGLICSIINYCQEA	TAA	1	1	1	100	100	100	-
		top-down			--								
3		trypsin			KFCSSTSDCGEGMCCTGGSFNRHCQRLSENGRPCQRPNDQDYYSTGCPCKEGLICSIINYCQEA								
3		chymotrypsin			KFCSSTSDCGEGMCCTGGSFNRHCQRLSENGRPCQRPNDQDYYSTGCPCKEGLICSIINYCQEA								
SN32_02	CSTX-22b	MKYFIVLAVLATLVLAIQG			DYCSSTSECGEGMCCTGSSFNRHCQRLSENGTPCQRRNDQDYYTYGCPCQEGLICSVINYCQEA	TAA	1	1	2	100	100	100	X
		top-down			DYCSSTSECGEGMCCTGSSFNRHCQRLSENGTPCQRRNDQDYYTYGCPCQEGLICSVINYCQEA								
3		trypsin			DYCSSTSECGEGMCCTGSSFNRHCQRLSENGTPCQRRNDQDYYTYGCPCQEGLICSVINYCQEA								
2		chymotrypsin			DYCSSTSECGEGMCCTGSSFNRHCQRLSENGTPCQRRNDQDYYTYGCPCQEGLICSVINYCQEA								
SN32_02	CSTX-22c	MKCFIVLAVLATLVLAIQG			KFCSSTSDCGEGMCCTGGSFNRHCQSLSENGRPCQRPNDQDYYSTGCPCKEGLICSIINYCQEA	TAA	1	1	1*	100	100	100	X
		top-down			KFCSSTSDCGEGMCCTGGSFNRHCQSLSENGRPCQRPNDQDYYSTGCPCKEGLICSIINYCQEA								
3		trypsin			KFCSSTSDCGEGMCCTGGSFNRHCQSLSENGRPCQRPNDQDYYSTGCPCKEGLICSIINYCQEA								
3		chymotrypsin			KFCSSTSDCGEGMCCTGGSFNRHCQSLSENGRPCQRPNDQDYYSTGCPCKEGLICSIINYCQEA								
	21				4 4 8 9 12 1 5 5	6	Σ=7	Σ=7	Σ=17				
SN32_01	CSTX-21a	MKVTVAFIVLASLMCLVYSASS			EPVSCGDGYCGEGQCCSGTHYNPHCKFYGDDGDICQRPNKYNQYKTACPCKEGLTCNVINRCQRD	TGA	1	1	12	93.8	100	100	X
		top-down			EPVSCGDGYCGEGQCCSGTHYNPHCKFYGDDGDICQRPNKYNQYKTACPCKEGLTCNVINRCQRD								
49		trypsin			EPVSCGDGYCGEGQCCSGTHYNPHCKFYGDDGDICQRPNKYNQYKTACPCKEGLTCNVINRCQRD								
2		chymotrypsin			EPVSCGDGYCGEGQCCSGTHYNPHCKFYGDDGDICQRPNKYNQYKTACPCKEGLTCNVINRCQRD								
SN32_01	CSTX-21b	MKVTVAFIVLASLMCLVYSASS			EPISCGDGYCGEGQCCSGTHYNPHCKFYGDDGDICQRPNNYNQYKTACPCKEGLTCNVINRCQRD	TGA	1	1	1*	93.8	100	100	-
		top-down			--								
42		trypsin			EPISCGDGYCGEGQCCSGTHYNPHCKFYGDDGDICQRPNNYNQYKTACPCKEGLTCNVINRCQRD								
9		chymotrypsin			EPISCGDGYCGEGQCCSGTHYNPHCKFYGDDGDICQRPNNYNQYKTACPCKEGLTCNVINRCQRD								
SN32_01	CSTX-21c	MKVTVAFIVLASLMCLVYSASS			EPVLCGDGYCGEGQCCSGTHYNPHCKFYGDDGDICQRPNKYNQYKTACPCKEGLTCNVINRCQRD	TGA	1	1	1	89.2	73.8	93.8	-
		top-down			--								
		trypsin			EPVLCGDGYCGEGQCCSGTHYNPHCKFYGDDGDICQRPNKYNQYKTACPCKEGLTCNVINRCQRD								
2		chymotrypsin			EPVLCGDGYCGEGQCCSGTHYNPHCKFYGDDGDICQRPNKYNQYKTACPCKEGLTCNVINRCQRD								
SN32_01	CSTX-21d	MKVTVAFIVLASLMCLVYSASS			EPVSCGDGYCGEGQCCSGTHYNPHCKFYGDDGDICQRPNNYNQYKTACPCKEGLTCNVINRCQRD	TGA	1	1	1*	93.8	100	100	X
		top-down			EPVSCGDGYCGEGQCCSGTHYNPHCKFYGDDGDICQRPNNYNQYKTACPCKEGLTCNVINRCQRD								
49		trypsin			EPVSCGDGYCGEGQCCSGTHYNPHCKFYGDDGDICQRPNNYNQYKTACPCKEGLTCNVINRCQRD								
9		chymotrypsin			EPVSCGDGYCGEGQCCSGTHYNPHCKFYGDDGDICQRPNNYNQYKTACPCKEGLTCNVINRCQRD								
SN32_01	CSTX-21e	MKVTVAFIVLASLMCLVYSASS			EPVSCGGEYCREGECCAGGSYHRNCRSYGDPGDICQKPNKFNEYRTACPCKEGLICSVINRCQKV	TGA	1	1	1*	89.2*	100*	100*	X*
		top-down			EPVSCGGEYCREGECCAGGSYHRNCRSYGDPGDICQKPNKFNEYRTACPCKEGLICSVINRCQKV								
		trypsin			EPVSCGGEYCREGECCAGGSYHRNCRSYGDPGDICQKPNKFNEYRTACPCKEGLICSVINRCQKV								
		chymotrypsin			EPVSCGGEYCREGECCAGGSYHRNCRSYGDPGDICQKPNKFNEYRTACPCKEGLICSVINRCQKV								
SN32_01	CSTX-21f	MKVTVAFIVLASLMCLVYSASS			EPVSCGGEYCREGECCAGGSYHRNCRSYGDPGDICQKPNKFNEYRTACPCKEGLTCSVINRCQKV	TGA	1	1	1*	89.2	100	100	-
		top-down											

		**trypsin**			**EPVSCGGEYCREGECCAGGSYHRN**CR**SYGDPGDICQKPNKFNEYRTACPCKEGLTCSVINR**CQ**KV**								
		**chymotrypsin**			**EPVSCGGEYCREGECCAGGSYHRNCRSYGDPGDICQKPNKFNEYRTACPCKEGLTCSVINRCQKV**								
**SN32_01**	CSTX-21g	MKVTVAFIALASLMCLVYSASS			EPVSCGGEYCREGECCAGGSYHRNCRSYGDPGDICQKPNKFNEYRTACPCKEGLICSVINRCQKV	**TGA**	1	1	1*	**98.2***	**100***	**100***	**X***
		**top-down**			**EPVSCGGEYCREGECCAGGSYHRNCRSYGDPGDICQKPNKFNEYRTACPCKEGLICSVINRCQKV**								
		**trypsin**			**EPVSCGGEYCREGECCAGGSYHRN**CR**SYGDPGDICQKPNKFNEYRTACPCKEGLICSVINR**CQ**KV**								
		**chymotrypsin**			**EPVSCGGEYCREGECCAGGSYHRNCRSYGDPGDICQKPNKFNEYRTACPCKEGLICSVINRCQKV**								

Definition of a precursor variant: SP/ PP/ MP (AA-sequences)
Transcript variants: including silent mutations in SP/PP/MP
Naming silent mutations: CSTX_1a_S1_ S: silent mutation in the signal peptide (SP); propeptide (PP); mature peptide (MP)
MP Counts: complete mature peptide (n); 1*: sequence of mature peptide is based of at least 2 overlapping sequences
Proteomic coverage: red highlighted: non proteotypic
yellow highlighted: confirmed sequences by MS/MS

Results:

- **2435 analysed single sequences (therefrom 15 sequences are based on 2 and more overlapping reads)**
- **81 transcripts**
- **66 precursors**
- **54 mature peptides**

+ 2 mature peptides only Edman degradation (sp|B3EWT3|TX27A_CUPSA Toxin CSTX-18; sp|B3EWT1|TXC11_CUPSA Toxin CSTX-11)
