# Supplementary material for: The Dual Prey-Inactivation Strategy of Spiders—In-Depth Venomic Analysis of Cupiennius salei
Source: Toxins (Basel). 2019 Mar 19;11(3):167. doi: 10.3390/toxins11030167 (PMC6468893; doi:10.3390/toxins11030167)
Supplement: Supplementary file 1 [file toxins-11-00167-s001.zip › Supplementary Dataset EV1/20180328_f2_topdown_OTMS2_EThcD_NL_i02_ms2_proteoform_cutoff_html/proteins.html]

21 proteins were identified


## 21 proteins were identified.

  

CsTx-9a Cupiennius salei toxin 9 isoform a

The best PrSM has an E-value 5.41e-56. There
are 3 proteoforms.

CsTx-11a Cupiennius salei toxin 11 isoform a

The best PrSM has an E-value 5.58e-51. There
is 1 proteoform.

CsTx-10a\_S1 Cupiennius salei toxin 10 isoform a S1^ACsTx-10a\_S2 Cupiennius salei toxin 10 isoform a S2

The best PrSM has an E-value 4.63e-49. There
is 1 proteoform.

CsTx-1a\_S1 Cupiennius salei toxin 1 isoform a S1^ACsTx-1a\_S2 Cupiennius salei toxin 1 isoform a S2

The best PrSM has an E-value 6.75e-44. There
are 7 proteoforms.

CsTx-1b Cupiennius salei toxin 1 isoform b

The best PrSM has an E-value 1.99e-36. There
are 4 proteoforms.

CsTx-33a Cupiennius salei toxin 33 isoform a

The best PrSM has an E-value 9.29e-36. There
are 3 proteoforms.

CsTx-12a\_S1 Cupiennius salei toxin 12 isoform a S1^ACsTx-12a\_S2 Cupiennius salei toxin 12 isoform a S2

The best PrSM has an E-value 3.07e-29. There
are 10 proteoforms.

sp|B3EWT6|TXC2A\_CUPSA Cupiennin-2a OS=Cupiennius salei OX=6928 PE=1 SV=1

The best PrSM has an E-value 2.01e-27. There
are 4 proteoforms.

CsTx-13a Cupiennius salei toxin 13 isoform a

The best PrSM has an E-value 3.41e-27. There
are 14 proteoforms.

sp|B3EWU1|TXS1A\_CUPSA Short cationic peptide-1a OS=Cupiennius salei OX=6928 PE=1 SV=1

The best PrSM has an E-value 3.81e-26. There
is 1 proteoform.

sp|B3EWT7|TXC2B\_CUPSA Cupiennin-2b OS=Cupiennius salei OX=6928 PE=1 SV=1

The best PrSM has an E-value 4.00e-25. There
are 4 proteoforms.

sp|B3EWU0|TXC2E\_CUPSA Cupiennin-2e OS=Cupiennius salei OX=6928 PE=1 SV=1

The best PrSM has an E-value 9.14e-25. There
are 5 proteoforms.

sp|B3EWT9|TXC2D\_CUPSA Cupiennin-2d OS=Cupiennius salei OX=6928 PE=1 SV=1

The best PrSM has an E-value 5.55e-24. There
are 5 proteoforms.

sp|B3EWT8|TXC2C\_CUPSA Cupiennin-2c OS=Cupiennius salei OX=6928 PE=1 SV=1

The best PrSM has an E-value 1.86e-23. There
are 4 proteoforms.

CsTx-12b Cupiennius salei toxin 12 isoform b

The best PrSM has an E-value 1.02e-22. There
are 5 proteoforms.

CsTx-9c\_S1 Cupiennius salei toxin 9 isoform c S1^ACsTx-9c\_S2 Cupiennius salei toxin 9 isoform c S2

The best PrSM has an E-value 3.40e-22. There
is 1 proteoform.

sp|B3EWV7|TXC4B\_CUPSA Cupiennin-4b OS=Cupiennius salei OX=6928 PE=1 SV=1

The best PrSM has an E-value 3.95e-21. There
is 1 proteoform.

CsTx-13b Cupiennius salei toxin 13 isoform b

The best PrSM has an E-value 2.25e-19. There
is 1 proteoform.

CsTx-8a\_S1 Cupiennius salei toxin 8 isoform a S1^ACsTx-8a\_S2 Cupiennius salei toxin 8 isoform a S2

The best PrSM has an E-value 5.59e-17. There
are 2 proteoforms.

sp|P83619|TXC1A\_CUPSA Cupiennin-1a OS=Cupiennius salei OX=6928 PE=1 SV=1

The best PrSM has an E-value 1.19e-11. There
is 1 proteoform.

CsTx-8b Cupiennius salei toxin 8 isoform b

The best PrSM has an E-value 2.08e-09. There
is 1 proteoform.
