# Supplementary material for: The Dual Prey-Inactivation Strategy of Spiders—In-Depth Venomic Analysis of Cupiennius salei
Source: Toxins (Basel). 2019 Mar 19;11(3):167. doi: 10.3390/toxins11030167 (PMC6468893; doi:10.3390/toxins11030167)
Supplement: Supplementary file 1 [file toxins-11-00167-s001.zip › Supplementary Dataset EV1/20180328_f2_topdown_OTMS2_EThcD_NL_i02_ms2_proteoform_cutoff_html/proteins/protein13.html]

Proteoforms for protein sp|B3EWU1|TXS1A\_CUPSA Short cationic peptide-1a OS=Cupiennius salei OX=6928 PE=1 SV=1


All proteins

1 proteoforms for protein sp|B3EWU1|TXS1A\_CUPSA Short cationic peptide-1a OS=Cupiennius salei OX=6928 PE=1 SV=1

## Proteoform #23

The best PrSM has an E-value 3.81e-26
and a precursor mass .
There are 2 PrSMs in total.

|  |  |  |  |  |  |  |  |  |  |  |  |  |  |  |  |  |  |  |  |  |  |  |  |  |  |  |  |  |  |  |  |  |  |  |  |  |  |  |  |  |  |  |  |  |  |  |  |  |  |  |  |  |  |  |  |  |  |  |  |  |  |  |  |  |  |  |
| --- | --- | --- | --- | --- | --- | --- | --- | --- | --- | --- | --- | --- | --- | --- | --- | --- | --- | --- | --- | --- | --- | --- | --- | --- | --- | --- | --- | --- | --- | --- | --- | --- | --- | --- | --- | --- | --- | --- | --- | --- | --- | --- | --- | --- | --- | --- | --- | --- | --- | --- | --- | --- | --- | --- | --- | --- | --- | --- | --- | --- | --- | --- | --- | --- | --- | --- |
|  | |  | | | | | | | | | | | | | | | | | | | | | | | | | | | | | | | | | | | | | | | | | | | | | | | | | | | | | | | | | -0.99 | | | | | | | |
| 1 |  |  | F |  | L |  | A |  | K |  | K |  | V |  | A |  | K |  | T |  | V |  |  | A |  | K |  | Q |  | A |  | A |  | K |  | Q |  | G |  | A |  | K |  |  | Y |  | V |  | V |  | N |  | K |  | Q |  | M |  | E |  | | 28 |  | | | |

  
  

All proteins
