# Supplementary material for: The Dual Prey-Inactivation Strategy of Spiders—In-Depth Venomic Analysis of Cupiennius salei
Source: Toxins (Basel). 2019 Mar 19;11(3):167. doi: 10.3390/toxins11030167 (PMC6468893; doi:10.3390/toxins11030167)
Supplement: Supplementary file 1 [file toxins-11-00167-s001.zip › Supplementary Dataset EV1/20180328_f2_topdown_OTMS2_EThcD_NL_i02_ms2_proteoform_cutoff_html/proteins/protein2.html]

Proteoforms for protein CsTx-10a\_S1 Cupiennius salei toxin 10 isoform a S1^ACsTx-10a\_S2 Cupiennius salei toxin 10 isoform a S2


All proteins

1 proteoforms for protein CsTx-10a\_S1 Cupiennius salei toxin 10 isoform a S1^ACsTx-10a\_S2 Cupiennius salei toxin 10 isoform a S2

## Proteoform #2

The best PrSM has an E-value 4.63e-49
and a precursor mass .
There are 4 PrSMs in total.

|  |  |  |  |  |  |  |  |  |  |  |  |  |  |  |  |  |  |  |  |  |  |  |  |  |  |  |  |  |  |  |  |  |  |  |  |  |  |  |  |  |  |  |  |  |  |  |  |  |  |  |  |  |  |  |  |  |  |  |  |  |  |  |  |  |  |  |  |  |  |
| --- | --- | --- | --- | --- | --- | --- | --- | --- | --- | --- | --- | --- | --- | --- | --- | --- | --- | --- | --- | --- | --- | --- | --- | --- | --- | --- | --- | --- | --- | --- | --- | --- | --- | --- | --- | --- | --- | --- | --- | --- | --- | --- | --- | --- | --- | --- | --- | --- | --- | --- | --- | --- | --- | --- | --- | --- | --- | --- | --- | --- | --- | --- | --- | --- | --- | --- | --- | --- | --- |
|  | |  | | | | | | | | | | | | | | | | | | | | | | | | | | | | | | | | | | | | | | | | | | | | | | | | | | | | | | | | | | | | | | | | | | | |
| 1 |  |  | M |  | K |  | V |  | L |  | V |  | I |  | F |  | A |  | V |  | L |  |  | S |  | L |  | V |  | I |  | F |  | S |  | N |  | C |  | S |  | A |  |  | E |  | T |  | D |  | E |  | D |  | F |  | F |  | G |  | E |  | E |  | 30 |  |
|  | |  | | | | | | | | | | | | | | | | | | | | | | | | | | | | | | | | | | | | | | | | | | | | | | | | | | | | | | | | | | | | | | | | | | | |
| 31 |  |  | S |  | F |  | E |  | A |  | D |  | D |  | I |  | I |  | P |  | F |  |  | I |  | A |  | K |  | E |  | Q |  | V |  | R | ] | K |  | D |  | K |  |  | E |  | N |  | C |  | I |  | G |  | K |  | H |  | H |  | E |  | C |  | 60 |  |
|  | |  | | | | | | | | | | | | | | | | | | | | | | | | | | | | | | | | | | | | | | | | | | | | | | | | | | | | | | | | | | | | | | | | | | | |
| 61 |  |  | T |  | D |  | D |  | R |  | D |  | N |  | C |  | C |  | K |  | G |  |  | K |  | L |  | F |  | R |  | Y |  | Q |  | C |  | Q |  | C |  | F |  |  | K |  | V |  | I |  | D |  | G |  | K |  | K |  | E |  | T |  | K |  | 90 |  |
|  | |  | | | | | | | | | | | | | | | | | | | | | | | | | | | | | | | | | | | | | | | | | | | | | | | | | | | | | | | | | | | | | | | | | | | |
| 91 |  |  | R |  | C |  | A |  | C |  | V |  | T |  | P |  | L |  | H |  | Y |  |  | K |  | M |  | A |  | E |  | M |  | A |  | V |  | S |  | V |  | F |  |  | K |  | K |  | M |  | F |  | K |  | N |  | | 116 |  | | | | | | | |

  
  

All proteins
