# Supplementary material for: The Dual Prey-Inactivation Strategy of Spiders—In-Depth Venomic Analysis of Cupiennius salei
Source: Toxins (Basel). 2019 Mar 19;11(3):167. doi: 10.3390/toxins11030167 (PMC6468893; doi:10.3390/toxins11030167)
Supplement: Supplementary file 1 [file toxins-11-00167-s001.zip › Supplementary Dataset EV1/20180328_f2_topdown_OTMS2_EThcD_NL_i02_ms2_proteoform_cutoff_html/proteins/protein24.html]

Proteoforms for protein CsTx-8b Cupiennius salei toxin 8 isoform b


All proteins

1 proteoforms for protein CsTx-8b Cupiennius salei toxin 8 isoform b

## Proteoform #101

There is only 1 PrSM
with an E-value 2.08e-09 and a precursor mass .

|  |  |  |  |  |  |  |  |  |  |  |  |  |  |  |  |  |  |  |  |  |  |  |  |  |  |  |  |  |  |  |  |  |  |  |  |  |  |  |  |  |  |  |  |  |  |  |  |  |  |  |  |  |  |  |  |  |  |  |  |  |  |  |  |  |  |  |  |  |  |
| --- | --- | --- | --- | --- | --- | --- | --- | --- | --- | --- | --- | --- | --- | --- | --- | --- | --- | --- | --- | --- | --- | --- | --- | --- | --- | --- | --- | --- | --- | --- | --- | --- | --- | --- | --- | --- | --- | --- | --- | --- | --- | --- | --- | --- | --- | --- | --- | --- | --- | --- | --- | --- | --- | --- | --- | --- | --- | --- | --- | --- | --- | --- | --- | --- | --- | --- | --- | --- | --- |
|  | |  | | | | | | | | | | | | | | | | | | | | | | | | | | | | | | | | | | | | | | | | | | | | | | | | | | | | | | | | | | | | | | | | | | | |
| 1 |  |  | M |  | K |  | V |  | L |  | V |  | I |  | C |  | A |  | V |  | L |  |  | F |  | L |  | S |  | I |  | F |  | S |  | N |  | S |  | S |  | A |  |  | E |  | T |  | E |  | D |  | D |  | F |  | L |  | E |  | D |  | E |  | 30 |  |
|  | |  | | | | | | | | | | | | | | | | | | | | | | | | | | | | | | | | | | | | | | | | | | | | | | | | | | | | | | | | | | | | | | | | | | | |
| 31 |  |  | S |  | F |  | Q |  | A |  | D |  | D |  | V |  | I |  | P |  | F |  |  | L |  | A |  | S |  | E |  | Q |  | V |  | R | ] | S |  | D |  | C |  |  | T |  | L |  | R |  | N |  | H |  | D |  | C |  | T |  | D |  | D |  | 60 |  |
|  | |  | | | | | | | | | | | | | | | | | -342.13 | | | | | | | | | | | | | | | | | | | | | | | | | | | | | | | | | | | | | | | | | | | | | | | |
| 61 |  |  | R |  | H |  | S |  | C |  | C |  | R |  | S |  | K |  | M |  | F |  |  | K |  | D |  | V |  | C |  | K |  | C |  | F |  | Y |  | P |  | S |  |  | Q |  | R |  | S |  | E | [ | T |  | D |  | R |  | A |  | K |  | K |  | 90 |  |
|  | |  | | | | | | | | | | | | | | | | | | | | | | | | | | | | | | | | | | | | | | | | | | | | | | | | | | | | | | | | | | | | | | | | | | | |
| 91 |  |  | E |  | L |  | C |  | T |  | C |  | Q |  | Q |  | P |  | K |  | H |  |  | L |  | K |  | Y |  | I |  | E |  | K |  | G |  | L |  | Q |  | K |  |  | A |  | K |  | D |  | Y |  | A |  | T |  | G |  | | 117 |  | | | | | |

  
  

All proteins
