# Supplementary material for: The Dual Prey-Inactivation Strategy of Spiders—In-Depth Venomic Analysis of Cupiennius salei
Source: Toxins (Basel). 2019 Mar 19;11(3):167. doi: 10.3390/toxins11030167 (PMC6468893; doi:10.3390/toxins11030167)
Supplement: Supplementary file 1 [file toxins-11-00167-s001.zip › Supplementary Dataset EV1/20180328_f2_topdown_OTMS2_EThcD_NL_i02_ms2_proteoform_cutoff_html/proteoforms/proteoform0.html]

Proteoform #0 from CsTx-9a Cupiennius salei toxin 9 isoform a


All proteins /
CsTx-9a Cupiennius salei toxin 9 isoform a

## Proteoform #0

26 PrSMs for this proteoform

| Scan | Protein | E-value | # all peaks | # matched peaks | # matched fragment ions | Link |
| --- | --- | --- | --- | --- | --- | --- |
| 704 | CsTx-9a | 5.41e-56 | 133 | 82 | 65 | See PrSM>> |
| 697 | CsTx-9a | 5.56e-55 | 131 | 74 | 64 | See PrSM>> |
| 677 | CsTx-9a | 9.81e-54 | 133 | 72 | 63 | See PrSM>> |
| 700 | CsTx-9a | 8.89e-52 | 133 | 76 | 61 | See PrSM>> |
| 683 | CsTx-9a | 1.78e-51 | 133 | 77 | 61 | See PrSM>> |
| 705 | CsTx-9a | 1.20e-50 | 133 | 77 | 60 | See PrSM>> |
| 691 | CsTx-9a | 2.39e-50 | 133 | 77 | 60 | See PrSM>> |
| 693 | CsTx-9a | 4.29e-50 | 133 | 71 | 59 | See PrSM>> |
| 685 | CsTx-9a | 4.29e-50 | 133 | 73 | 59 | See PrSM>> |
| 692 | CsTx-9a | 4.29e-50 | 133 | 73 | 59 | See PrSM>> |
| 699 | CsTx-9a | 7.70e-50 | 133 | 76 | 58 | See PrSM>> |
| 681 | CsTx-9a | 2.48e-49 | 133 | 77 | 56 | See PrSM>> |
| 680 | CsTx-9a | 4.28e-49 | 131 | 70 | 57 | See PrSM>> |
| 688 | CsTx-9a | 1.57e-47 | 133 | 71 | 53 | See PrSM>> |
| 695 | CsTx-9a | 1.57e-47 | 133 | 72 | 53 | See PrSM>> |
| 687 | CsTx-9a | 1.87e-47 | 118 | 58 | 53 | See PrSM>> |
| 689 | CsTx-9a | 4.79e-47 | 130 | 66 | 53 | See PrSM>> |
| 676 | CsTx-9a | 9.32e-47 | 133 | 59 | 52 | See PrSM>> |
| 684 | CsTx-9a | 5.54e-46 | 133 | 65 | 51 | See PrSM>> |
| 675 | CsTx-9a | 1.18e-44 | 133 | 53 | 49 | See PrSM>> |
| 793 | CsTx-9a | 1.51e-43 | 133 | 58 | 47 | See PrSM>> |
| 679 | CsTx-9a | 2.08e-41 | 105 | 49 | 46 | See PrSM>> |
| 1731 | CsTx-9a | 1.13e-36 | 110 | 43 | 37 | See PrSM>> |
| 1732 | CsTx-9a | 5.81e-32 | 97 | 32 | 31 | See PrSM>> |
| 775 | CsTx-9a | 8.78e-32 | 133 | 35 | 32 | See PrSM>> |
| 1737 | CsTx-9a | 2.61e-14 | 36 | 12 | 12 | See PrSM>> |

All proteins /
CsTx-9a Cupiennius salei toxin 9 isoform a
