# Supplementary material for: The Dual Prey-Inactivation Strategy of Spiders—In-Depth Venomic Analysis of Cupiennius salei
Source: Toxins (Basel). 2019 Mar 19;11(3):167. doi: 10.3390/toxins11030167 (PMC6468893; doi:10.3390/toxins11030167)
Supplement: Supplementary file 1 [file toxins-11-00167-s001.zip › Supplementary Dataset EV1/20180328_f2_topdown_OTMS2_EThcD_NL_i02_ms2_proteoform_cutoff_html/proteoforms/proteoform1.html]

Proteoform #1 from CsTx-11a Cupiennius salei toxin 11 isoform a


All proteins /
CsTx-11a Cupiennius salei toxin 11 isoform a

## Proteoform #1

5 PrSMs for this proteoform

| Scan | Protein | E-value | # all peaks | # matched peaks | # matched fragment ions | Link |
| --- | --- | --- | --- | --- | --- | --- |
| 660 | CsTx-11a | 5.58e-51 | 142 | 72 | 55 | See PrSM>> |
| 665 | CsTx-11a | 4.27e-48 | 142 | 70 | 51 | See PrSM>> |
| 661 | CsTx-11a | 2.25e-47 | 142 | 66 | 50 | See PrSM>> |
| 659 | CsTx-11a | 2.07e-42 | 142 | 64 | 44 | See PrSM>> |
| 669 | CsTx-11a | 1.21e-29 | 88 | 31 | 27 | See PrSM>> |

All proteins /
CsTx-11a Cupiennius salei toxin 11 isoform a
