# Supplementary material for: The Dual Prey-Inactivation Strategy of Spiders—In-Depth Venomic Analysis of Cupiennius salei
Source: Toxins (Basel). 2019 Mar 19;11(3):167. doi: 10.3390/toxins11030167 (PMC6468893; doi:10.3390/toxins11030167)
Supplement: Supplementary file 1 [file toxins-11-00167-s001.zip › Supplementary Dataset EV1/20180328_f2_topdown_OTMS2_EThcD_NL_i02_ms2_proteoform_cutoff_html/proteoforms/proteoform10.html]

Proteoform #10 from CsTx-9a Cupiennius salei toxin 9 isoform a


All proteins /
CsTx-9a Cupiennius salei toxin 9 isoform a

## Proteoform #10

5 PrSMs for this proteoform

| Scan | Protein | E-value | # all peaks | # matched peaks | # matched fragment ions | Link |
| --- | --- | --- | --- | --- | --- | --- |
| 536 | CsTx-9a | 4.07e-39 | 127 | 60 | 47 | See PrSM>> |
| 540 | CsTx-9a | 4.07e-39 | 127 | 58 | 47 | See PrSM>> |
| 535 | CsTx-9a | 8.37e-36 | 127 | 48 | 39 | See PrSM>> |
| 537 | CsTx-9a | 1.59e-26 | 126 | 39 | 28 | See PrSM>> |
| 541 | CsTx-9a | 2.44e-26 | 127 | 35 | 30 | See PrSM>> |

All proteins /
CsTx-9a Cupiennius salei toxin 9 isoform a
