# Supplementary material for: The Dual Prey-Inactivation Strategy of Spiders—In-Depth Venomic Analysis of Cupiennius salei
Source: Toxins (Basel). 2019 Mar 19;11(3):167. doi: 10.3390/toxins11030167 (PMC6468893; doi:10.3390/toxins11030167)
Supplement: Supplementary file 1 [file toxins-11-00167-s001.zip › Supplementary Dataset EV1/20180328_f2_topdown_OTMS2_EThcD_NL_i02_ms2_proteoform_cutoff_html/proteoforms/proteoform12.html]

Proteoform #12 from CsTx-33a Cupiennius salei toxin 33 isoform a


All proteins /
CsTx-33a Cupiennius salei toxin 33 isoform a

## Proteoform #12

1 PrSM for this proteoform

| Scan | Protein | E-value | # all peaks | # matched peaks | # matched fragment ions | Link |
| --- | --- | --- | --- | --- | --- | --- |
| 544 | CsTx-33a | 9.29e-36 | 134 | 56 | 38 | See PrSM>> |

All proteins /
CsTx-33a Cupiennius salei toxin 33 isoform a
