# Supplementary material for: The Dual Prey-Inactivation Strategy of Spiders—In-Depth Venomic Analysis of Cupiennius salei
Source: Toxins (Basel). 2019 Mar 19;11(3):167. doi: 10.3390/toxins11030167 (PMC6468893; doi:10.3390/toxins11030167)
Supplement: Supplementary file 1 [file toxins-11-00167-s001.zip › Supplementary Dataset EV1/20180328_f2_topdown_OTMS2_EThcD_NL_i02_ms2_proteoform_cutoff_html/proteoforms/proteoform15.html]

Proteoform #15 from CsTx-1a\_S1 Cupiennius salei toxin 1 isoform a S1^ACsTx-1a\_S2 Cupiennius salei toxin 1 isoform a S2


All proteins /
CsTx-1a\_S1 Cupiennius salei toxin 1 isoform a S1^ACsTx-1a\_S2 Cupiennius salei toxin 1 isoform a S2

## Proteoform #15

10 PrSMs for this proteoform

| Scan | Protein | E-value | # all peaks | # matched peaks | # matched fragment ions | Link |
| --- | --- | --- | --- | --- | --- | --- |
| 549 | CsTx-1a\_S1 | 7.56e-33 | 147 | 59 | 40 | See PrSM>> |
| 560 | CsTx-1a\_S1 | 2.28e-32 | 147 | 54 | 39 | See PrSM>> |
| 545 | CsTx-1a\_S1 | 2.28e-32 | 147 | 51 | 39 | See PrSM>> |
| 548 | CsTx-1a\_S1 | 2.08e-31 | 147 | 47 | 37 | See PrSM>> |
| 551 | CsTx-1a\_S1 | 6.28e-31 | 147 | 53 | 36 | See PrSM>> |
| 543 | CsTx-1a\_S1 | 6.29e-31 | 147 | 46 | 36 | See PrSM>> |
| 561 | CsTx-1a\_S1 | 4.90e-29 | 147 | 47 | 36 | See PrSM>> |
| 556 | CsTx-1a\_S1 | 9.97e-29 | 145 | 43 | 35 | See PrSM>> |
| 552 | CsTx-1a\_S1 | 1.48e-28 | 147 | 50 | 35 | See PrSM>> |
| 555 | CsTx-1a\_S1 | 1.48e-28 | 147 | 44 | 35 | See PrSM>> |

All proteins /
CsTx-1a\_S1 Cupiennius salei toxin 1 isoform a S1^ACsTx-1a\_S2 Cupiennius salei toxin 1 isoform a S2
