# Supplementary material for: The Dual Prey-Inactivation Strategy of Spiders—In-Depth Venomic Analysis of Cupiennius salei
Source: Toxins (Basel). 2019 Mar 19;11(3):167. doi: 10.3390/toxins11030167 (PMC6468893; doi:10.3390/toxins11030167)
Supplement: Supplementary file 1 [file toxins-11-00167-s001.zip › Supplementary Dataset EV1/20180328_f2_topdown_OTMS2_EThcD_NL_i02_ms2_proteoform_cutoff_html/proteoforms/proteoform18.html]

Proteoform #18 from CsTx-12a\_S1 Cupiennius salei toxin 12 isoform a S1^ACsTx-12a\_S2 Cupiennius salei toxin 12 isoform a S2


All proteins /
CsTx-12a\_S1 Cupiennius salei toxin 12 isoform a S1^ACsTx-12a\_S2 Cupiennius salei toxin 12 isoform a S2

## Proteoform #18

29 PrSMs for this proteoform

| Scan | Protein | E-value | # all peaks | # matched peaks | # matched fragment ions | Link |
| --- | --- | --- | --- | --- | --- | --- |
| 457 | CsTx-12a\_S1 | 3.07e-29 | 72 | 34 | 31 | See PrSM>> |
| 448 | CsTx-12a\_S1 | 3.07e-29 | 72 | 34 | 31 | See PrSM>> |
| 379 | CsTx-12a\_S1 | 3.07e-29 | 72 | 32 | 31 | See PrSM>> |
| 387 | CsTx-12a\_S1 | 3.07e-29 | 72 | 33 | 31 | See PrSM>> |
| 464 | CsTx-12a\_S1 | 6.17e-29 | 72 | 32 | 30 | See PrSM>> |
| 371 | CsTx-12a\_S1 | 6.17e-29 | 72 | 34 | 30 | See PrSM>> |
| 440 | CsTx-12a\_S1 | 6.17e-29 | 72 | 32 | 30 | See PrSM>> |
| 417 | CsTx-12a\_S1 | 6.17e-29 | 72 | 34 | 30 | See PrSM>> |
| 405 | CsTx-12a\_S1 | 6.17e-29 | 72 | 33 | 30 | See PrSM>> |
| 409 | CsTx-12a\_S1 | 6.17e-29 | 72 | 32 | 30 | See PrSM>> |
| 475 | CsTx-12a\_S1 | 2.38e-28 | 72 | 33 | 29 | See PrSM>> |
| 433 | CsTx-12a\_S1 | 2.38e-28 | 72 | 31 | 29 | See PrSM>> |
| 425 | CsTx-12a\_S1 | 2.38e-28 | 72 | 32 | 29 | See PrSM>> |
| 363 | CsTx-12a\_S1 | 9.17e-28 | 72 | 31 | 28 | See PrSM>> |
| 395 | CsTx-12a\_S1 | 3.54e-27 | 72 | 28 | 27 | See PrSM>> |
| 480 | CsTx-12a\_S1 | 1.36e-26 | 72 | 27 | 26 | See PrSM>> |
| 380 | CsTx-12a\_S1 | 3.12e-24 | 72 | 26 | 23 | See PrSM>> |
| 372 | CsTx-12a\_S1 | 2.41e-23 | 72 | 26 | 22 | See PrSM>> |
| 443 | CsTx-12a\_S1 | 2.41e-23 | 72 | 26 | 22 | See PrSM>> |
| 429 | CsTx-12a\_S1 | 2.41e-23 | 72 | 25 | 22 | See PrSM>> |
| 388 | CsTx-12a\_S1 | 1.86e-22 | 72 | 27 | 21 | See PrSM>> |
| 364 | CsTx-12a\_S1 | 1.86e-22 | 72 | 25 | 21 | See PrSM>> |
| 419 | CsTx-12a\_S1 | 1.32e-20 | 72 | 25 | 19 | See PrSM>> |
| 359 | CsTx-12a\_S1 | 1.13e-18 | 72 | 18 | 17 | See PrSM>> |
| 383 | CsTx-12a\_S1 | 1.05e-17 | 72 | 21 | 16 | See PrSM>> |
| 367 | CsTx-12a\_S1 | 1.05e-17 | 72 | 20 | 16 | See PrSM>> |
| 376 | CsTx-12a\_S1 | 9.73e-17 | 72 | 19 | 15 | See PrSM>> |
| 360 | CsTx-12a\_S1 | 9.73e-17 | 72 | 18 | 15 | See PrSM>> |
| 357 | CsTx-12a\_S1 | 1.10e-15 | 72 | 14 | 14 | See PrSM>> |

All proteins /
CsTx-12a\_S1 Cupiennius salei toxin 12 isoform a S1^ACsTx-12a\_S2 Cupiennius salei toxin 12 isoform a S2
