# Supplementary material for: The Dual Prey-Inactivation Strategy of Spiders—In-Depth Venomic Analysis of Cupiennius salei
Source: Toxins (Basel). 2019 Mar 19;11(3):167. doi: 10.3390/toxins11030167 (PMC6468893; doi:10.3390/toxins11030167)
Supplement: Supplementary file 1 [file toxins-11-00167-s001.zip › Supplementary Dataset EV1/20180328_f2_topdown_OTMS2_EThcD_NL_i02_ms2_proteoform_cutoff_html/proteoforms/proteoform2.html]

Proteoform #2 from CsTx-10a\_S1 Cupiennius salei toxin 10 isoform a S1^ACsTx-10a\_S2 Cupiennius salei toxin 10 isoform a S2


All proteins /
CsTx-10a\_S1 Cupiennius salei toxin 10 isoform a S1^ACsTx-10a\_S2 Cupiennius salei toxin 10 isoform a S2

## Proteoform #2

4 PrSMs for this proteoform

| Scan | Protein | E-value | # all peaks | # matched peaks | # matched fragment ions | Link |
| --- | --- | --- | --- | --- | --- | --- |
| 651 | CsTx-10a\_S1 | 4.63e-49 | 142 | 76 | 53 | See PrSM>> |
| 656 | CsTx-10a\_S1 | 6.03e-41 | 142 | 58 | 43 | See PrSM>> |
| 657 | CsTx-10a\_S1 | 6.03e-41 | 142 | 60 | 43 | See PrSM>> |
| 655 | CsTx-10a\_S1 | 3.89e-39 | 142 | 61 | 48 | See PrSM>> |

All proteins /
CsTx-10a\_S1 Cupiennius salei toxin 10 isoform a S1^ACsTx-10a\_S2 Cupiennius salei toxin 10 isoform a S2
