# Supplementary material for: The Dual Prey-Inactivation Strategy of Spiders—In-Depth Venomic Analysis of Cupiennius salei
Source: Toxins (Basel). 2019 Mar 19;11(3):167. doi: 10.3390/toxins11030167 (PMC6468893; doi:10.3390/toxins11030167)
Supplement: Supplementary file 1 [file toxins-11-00167-s001.zip › Supplementary Dataset EV1/20180328_f2_topdown_OTMS2_EThcD_NL_i02_ms2_proteoform_cutoff_html/proteoforms/proteoform21.html]

Proteoform #21 from sp|B3EWT6|TXC2A\_CUPSA Cupiennin-2a OS=Cupiennius salei OX=6928 PE=1 SV=1


All proteins /
sp|B3EWT6|TXC2A\_CUPSA Cupiennin-2a OS=Cupiennius salei OX=6928 PE=1 SV=1

## Proteoform #21

109 PrSMs for this proteoform

| Scan | Protein | E-value | # all peaks | # matched peaks | # matched fragment ions | Link |
| --- | --- | --- | --- | --- | --- | --- |
| 1709 | sp|B3EWT6|TXC2A\_CUPSA | 2.01e-27 | 61 | 42 | 33 | See PrSM>> |
| 852 | sp|B3EWT6|TXC2A\_CUPSA | 2.01e-27 | 61 | 40 | 33 | See PrSM>> |
| 996 | sp|B3EWT6|TXC2A\_CUPSA | 6.08e-27 | 61 | 39 | 32 | See PrSM>> |
| 1245 | sp|B3EWT6|TXC2A\_CUPSA | 6.08e-27 | 61 | 41 | 32 | See PrSM>> |
| 1189 | sp|B3EWT6|TXC2A\_CUPSA | 1.84e-26 | 61 | 37 | 31 | See PrSM>> |
| 1197 | sp|B3EWT6|TXC2A\_CUPSA | 1.84e-26 | 61 | 37 | 31 | See PrSM>> |
| 1068 | sp|B3EWT6|TXC2A\_CUPSA | 1.84e-26 | 61 | 36 | 31 | See PrSM>> |
| 1076 | sp|B3EWT6|TXC2A\_CUPSA | 1.84e-26 | 61 | 38 | 31 | See PrSM>> |
| 868 | sp|B3EWT6|TXC2A\_CUPSA | 1.84e-26 | 61 | 36 | 31 | See PrSM>> |
| 1228 | sp|B3EWT6|TXC2A\_CUPSA | 1.84e-26 | 61 | 38 | 31 | See PrSM>> |
| 1148 | sp|B3EWT6|TXC2A\_CUPSA | 5.58e-26 | 61 | 38 | 30 | See PrSM>> |
| 1325 | sp|B3EWT6|TXC2A\_CUPSA | 5.58e-26 | 61 | 37 | 30 | See PrSM>> |
| 1124 | sp|B3EWT6|TXC2A\_CUPSA | 5.58e-26 | 61 | 35 | 30 | See PrSM>> |
| 1109 | sp|B3EWT6|TXC2A\_CUPSA | 5.58e-26 | 61 | 37 | 30 | See PrSM>> |
| 1100 | sp|B3EWT6|TXC2A\_CUPSA | 5.58e-26 | 61 | 38 | 30 | See PrSM>> |
| 1084 | sp|B3EWT6|TXC2A\_CUPSA | 5.58e-26 | 61 | 39 | 30 | See PrSM>> |
| 1044 | sp|B3EWT6|TXC2A\_CUPSA | 5.58e-26 | 61 | 37 | 30 | See PrSM>> |
| 1004 | sp|B3EWT6|TXC2A\_CUPSA | 5.58e-26 | 61 | 34 | 30 | See PrSM>> |
| 1012 | sp|B3EWT6|TXC2A\_CUPSA | 5.58e-26 | 61 | 35 | 30 | See PrSM>> |
| 1163 | sp|B3EWT6|TXC2A\_CUPSA | 5.58e-26 | 61 | 35 | 30 | See PrSM>> |
| 1173 | sp|B3EWT6|TXC2A\_CUPSA | 5.58e-26 | 61 | 35 | 30 | See PrSM>> |
| 1205 | sp|B3EWT6|TXC2A\_CUPSA | 5.58e-26 | 61 | 38 | 30 | See PrSM>> |
| 1276 | sp|B3EWT6|TXC2A\_CUPSA | 5.58e-26 | 61 | 37 | 30 | See PrSM>> |
| 1211 | sp|B3EWT6|TXC2A\_CUPSA | 5.58e-26 | 61 | 36 | 30 | See PrSM>> |
| 876 | sp|B3EWT6|TXC2A\_CUPSA | 5.58e-26 | 61 | 34 | 30 | See PrSM>> |
| 1252 | sp|B3EWT6|TXC2A\_CUPSA | 5.58e-26 | 61 | 37 | 30 | See PrSM>> |
| 1259 | sp|B3EWT6|TXC2A\_CUPSA | 5.58e-26 | 61 | 37 | 30 | See PrSM>> |
| 1723 | sp|B3EWT6|TXC2A\_CUPSA | 2.26e-25 | 61 | 37 | 29 | See PrSM>> |
| 1285 | sp|B3EWT6|TXC2A\_CUPSA | 2.26e-25 | 61 | 37 | 29 | See PrSM>> |
| 1020 | sp|B3EWT6|TXC2A\_CUPSA | 2.26e-25 | 61 | 34 | 29 | See PrSM>> |
| 1028 | sp|B3EWT6|TXC2A\_CUPSA | 2.26e-25 | 61 | 35 | 29 | See PrSM>> |
| 1036 | sp|B3EWT6|TXC2A\_CUPSA | 2.26e-25 | 61 | 35 | 29 | See PrSM>> |
| 1268 | sp|B3EWT6|TXC2A\_CUPSA | 2.26e-25 | 61 | 38 | 29 | See PrSM>> |
| 1051 | sp|B3EWT6|TXC2A\_CUPSA | 2.26e-25 | 61 | 34 | 29 | See PrSM>> |
| 1060 | sp|B3EWT6|TXC2A\_CUPSA | 2.26e-25 | 61 | 35 | 29 | See PrSM>> |
| 1092 | sp|B3EWT6|TXC2A\_CUPSA | 2.26e-25 | 61 | 35 | 29 | See PrSM>> |
| 1237 | sp|B3EWT6|TXC2A\_CUPSA | 2.26e-25 | 61 | 39 | 29 | See PrSM>> |
| 1116 | sp|B3EWT6|TXC2A\_CUPSA | 2.26e-25 | 61 | 37 | 29 | See PrSM>> |
| 1132 | sp|B3EWT6|TXC2A\_CUPSA | 2.26e-25 | 61 | 35 | 29 | See PrSM>> |
| 1139 | sp|B3EWT6|TXC2A\_CUPSA | 2.26e-25 | 61 | 34 | 29 | See PrSM>> |
| 1156 | sp|B3EWT6|TXC2A\_CUPSA | 2.26e-25 | 61 | 35 | 29 | See PrSM>> |
| 916 | sp|B3EWT6|TXC2A\_CUPSA | 2.26e-25 | 61 | 34 | 29 | See PrSM>> |
| 924 | sp|B3EWT6|TXC2A\_CUPSA | 2.26e-25 | 61 | 34 | 29 | See PrSM>> |
| 972 | sp|B3EWT6|TXC2A\_CUPSA | 2.26e-25 | 61 | 33 | 29 | See PrSM>> |
| 940 | sp|B3EWT6|TXC2A\_CUPSA | 2.26e-25 | 61 | 33 | 29 | See PrSM>> |
| 1715 | sp|B3EWT6|TXC2A\_CUPSA | 2.26e-25 | 61 | 39 | 29 | See PrSM>> |
| 1316 | sp|B3EWT6|TXC2A\_CUPSA | 9.14e-25 | 61 | 34 | 28 | See PrSM>> |
| 1341 | sp|B3EWT6|TXC2A\_CUPSA | 9.14e-25 | 61 | 33 | 28 | See PrSM>> |
| 1292 | sp|B3EWT6|TXC2A\_CUPSA | 9.14e-25 | 61 | 35 | 28 | See PrSM>> |
| 1357 | sp|B3EWT6|TXC2A\_CUPSA | 9.14e-25 | 61 | 36 | 28 | See PrSM>> |
| 1180 | sp|B3EWT6|TXC2A\_CUPSA | 9.14e-25 | 61 | 35 | 28 | See PrSM>> |
| 948 | sp|B3EWT6|TXC2A\_CUPSA | 9.14e-25 | 61 | 31 | 28 | See PrSM>> |
| 1389 | sp|B3EWT6|TXC2A\_CUPSA | 9.14e-25 | 61 | 34 | 28 | See PrSM>> |
| 843 | sp|B3EWT6|TXC2A\_CUPSA | 9.14e-25 | 61 | 29 | 28 | See PrSM>> |
| 1397 | sp|B3EWT6|TXC2A\_CUPSA | 9.14e-25 | 61 | 34 | 28 | See PrSM>> |
| 980 | sp|B3EWT6|TXC2A\_CUPSA | 9.14e-25 | 61 | 32 | 28 | See PrSM>> |
| 900 | sp|B3EWT6|TXC2A\_CUPSA | 9.14e-25 | 61 | 34 | 28 | See PrSM>> |
| 884 | sp|B3EWT6|TXC2A\_CUPSA | 9.14e-25 | 61 | 32 | 28 | See PrSM>> |
| 1221 | sp|B3EWT6|TXC2A\_CUPSA | 9.14e-25 | 61 | 37 | 28 | See PrSM>> |
| 1365 | sp|B3EWT6|TXC2A\_CUPSA | 3.70e-24 | 61 | 35 | 27 | See PrSM>> |
| 1309 | sp|B3EWT6|TXC2A\_CUPSA | 3.70e-24 | 61 | 34 | 27 | See PrSM>> |
| 1456 | sp|B3EWT6|TXC2A\_CUPSA | 3.70e-24 | 61 | 32 | 27 | See PrSM>> |
| 1301 | sp|B3EWT6|TXC2A\_CUPSA | 3.70e-24 | 61 | 33 | 27 | See PrSM>> |
| 1333 | sp|B3EWT6|TXC2A\_CUPSA | 3.70e-24 | 61 | 34 | 27 | See PrSM>> |
| 1349 | sp|B3EWT6|TXC2A\_CUPSA | 3.70e-24 | 61 | 31 | 27 | See PrSM>> |
| 1381 | sp|B3EWT6|TXC2A\_CUPSA | 3.70e-24 | 61 | 32 | 27 | See PrSM>> |
| 1405 | sp|B3EWT6|TXC2A\_CUPSA | 3.70e-24 | 61 | 33 | 27 | See PrSM>> |
| 988 | sp|B3EWT6|TXC2A\_CUPSA | 3.70e-24 | 61 | 31 | 27 | See PrSM>> |
| 1421 | sp|B3EWT6|TXC2A\_CUPSA | 3.70e-24 | 61 | 34 | 27 | See PrSM>> |
| 1429 | sp|B3EWT6|TXC2A\_CUPSA | 3.70e-24 | 61 | 33 | 27 | See PrSM>> |
| 1413 | sp|B3EWT6|TXC2A\_CUPSA | 1.02e-23 | 61 | 25 | 21 | See PrSM>> |
| 1703 | sp|B3EWT6|TXC2A\_CUPSA | 1.50e-23 | 61 | 34 | 26 | See PrSM>> |
| 964 | sp|B3EWT6|TXC2A\_CUPSA | 1.50e-23 | 61 | 28 | 26 | See PrSM>> |
| 851 | sp|B3EWT6|TXC2A\_CUPSA | 1.86e-23 | 61 | 25 | 20 | See PrSM>> |
| 892 | sp|B3EWT6|TXC2A\_CUPSA | 1.62e-22 | 61 | 25 | 24 | See PrSM>> |
| 1444 | sp|B3EWT6|TXC2A\_CUPSA | 1.62e-22 | 61 | 32 | 24 | See PrSM>> |
| 1373 | sp|B3EWT6|TXC2A\_CUPSA | 1.62e-22 | 61 | 30 | 24 | See PrSM>> |
| 1003 | sp|B3EWT6|TXC2A\_CUPSA | 4.34e-22 | 61 | 27 | 23 | See PrSM>> |
| 1229 | sp|B3EWT6|TXC2A\_CUPSA | 4.34e-22 | 61 | 29 | 23 | See PrSM>> |
| 1019 | sp|B3EWT6|TXC2A\_CUPSA | 1.16e-21 | 61 | 27 | 22 | See PrSM>> |
| 1027 | sp|B3EWT6|TXC2A\_CUPSA | 1.16e-21 | 61 | 26 | 22 | See PrSM>> |
| 1235 | sp|B3EWT6|TXC2A\_CUPSA | 1.16e-21 | 61 | 27 | 22 | See PrSM>> |
| 995 | sp|B3EWT6|TXC2A\_CUPSA | 1.16e-21 | 61 | 29 | 22 | See PrSM>> |
| 1471 | sp|B3EWT6|TXC2A\_CUPSA | 1.16e-21 | 61 | 27 | 22 | See PrSM>> |
| 971 | sp|B3EWT6|TXC2A\_CUPSA | 1.16e-21 | 61 | 25 | 22 | See PrSM>> |
| 923 | sp|B3EWT6|TXC2A\_CUPSA | 1.16e-21 | 61 | 27 | 22 | See PrSM>> |
| 1131 | sp|B3EWT6|TXC2A\_CUPSA | 1.16e-21 | 61 | 29 | 22 | See PrSM>> |
| 939 | sp|B3EWT6|TXC2A\_CUPSA | 1.16e-21 | 61 | 26 | 22 | See PrSM>> |
| 956 | sp|B3EWT6|TXC2A\_CUPSA | 1.16e-21 | 61 | 23 | 22 | See PrSM>> |
| 1195 | sp|B3EWT6|TXC2A\_CUPSA | 3.12e-21 | 61 | 27 | 21 | See PrSM>> |
| 1140 | sp|B3EWT6|TXC2A\_CUPSA | 3.12e-21 | 61 | 29 | 21 | See PrSM>> |
| 963 | sp|B3EWT6|TXC2A\_CUPSA | 3.12e-21 | 61 | 22 | 21 | See PrSM>> |
| 947 | sp|B3EWT6|TXC2A\_CUPSA | 3.12e-21 | 61 | 25 | 21 | See PrSM>> |
| 932 | sp|B3EWT6|TXC2A\_CUPSA | 8.34e-21 | 61 | 21 | 20 | See PrSM>> |
| 891 | sp|B3EWT6|TXC2A\_CUPSA | 8.34e-21 | 61 | 23 | 20 | See PrSM>> |
| 883 | sp|B3EWT6|TXC2A\_CUPSA | 8.34e-21 | 61 | 24 | 20 | See PrSM>> |
| 1707 | sp|B3EWT6|TXC2A\_CUPSA | 6.13e-20 | 61 | 26 | 19 | See PrSM>> |
| 987 | sp|B3EWT6|TXC2A\_CUPSA | 6.13e-20 | 61 | 20 | 19 | See PrSM>> |
| 875 | sp|B3EWT6|TXC2A\_CUPSA | 6.13e-20 | 61 | 26 | 19 | See PrSM>> |
| 1724 | sp|B3EWT6|TXC2A\_CUPSA | 8.34e-20 | 55 | 27 | 22 | See PrSM>> |
| 931 | sp|B3EWT6|TXC2A\_CUPSA | 4.50e-19 | 61 | 18 | 18 | See PrSM>> |
| 908 | sp|B3EWT6|TXC2A\_CUPSA | 4.50e-19 | 61 | 18 | 18 | See PrSM>> |
| 907 | sp|B3EWT6|TXC2A\_CUPSA | 4.50e-19 | 61 | 20 | 18 | See PrSM>> |
| 844 | sp|B3EWT6|TXC2A\_CUPSA | 4.50e-19 | 61 | 19 | 18 | See PrSM>> |
| 859 | sp|B3EWT6|TXC2A\_CUPSA | 2.43e-17 | 61 | 16 | 16 | See PrSM>> |
| 833 | sp|B3EWT6|TXC2A\_CUPSA | 1.52e-15 | 61 | 15 | 14 | See PrSM>> |
| 837 | sp|B3EWT6|TXC2A\_CUPSA | 1.29e-14 | 61 | 14 | 13 | See PrSM>> |
| 860 | sp|B3EWT6|TXC2A\_CUPSA | 3.83e-11 | 61 | 8 | 8 | See PrSM>> |
| 835 | sp|B3EWT6|TXC2A\_CUPSA | 3.90e-10 | 61 | 7 | 7 | See PrSM>> |

All proteins /
sp|B3EWT6|TXC2A\_CUPSA Cupiennin-2a OS=Cupiennius salei OX=6928 PE=1 SV=1
