# Supplementary material for: The Dual Prey-Inactivation Strategy of Spiders—In-Depth Venomic Analysis of Cupiennius salei
Source: Toxins (Basel). 2019 Mar 19;11(3):167. doi: 10.3390/toxins11030167 (PMC6468893; doi:10.3390/toxins11030167)
Supplement: Supplementary file 1 [file toxins-11-00167-s001.zip › Supplementary Dataset EV1/20180328_f2_topdown_OTMS2_EThcD_NL_i02_ms2_proteoform_cutoff_html/proteoforms/proteoform22.html]

Proteoform #22 from CsTx-13a Cupiennius salei toxin 13 isoform a


All proteins /
CsTx-13a Cupiennius salei toxin 13 isoform a

## Proteoform #22

12 PrSMs for this proteoform

| Scan | Protein | E-value | # all peaks | # matched peaks | # matched fragment ions | Link |
| --- | --- | --- | --- | --- | --- | --- |
| 399 | CsTx-13a | 3.41e-27 | 72 | 29 | 27 | See PrSM>> |
| 424 | CsTx-13a | 5.27e-26 | 72 | 28 | 25 | See PrSM>> |
| 432 | CsTx-13a | 5.27e-26 | 72 | 26 | 25 | See PrSM>> |
| 461 | CsTx-13a | 6.36e-26 | 68 | 28 | 26 | See PrSM>> |
| 465 | CsTx-13a | 1.18e-25 | 67 | 27 | 26 | See PrSM>> |
| 408 | CsTx-13a | 4.06e-25 | 72 | 27 | 24 | See PrSM>> |
| 416 | CsTx-13a | 4.06e-25 | 72 | 26 | 24 | See PrSM>> |
| 441 | CsTx-13a | 4.06e-25 | 72 | 26 | 24 | See PrSM>> |
| 449 | CsTx-13a | 2.80e-24 | 71 | 23 | 23 | See PrSM>> |
| 401 | CsTx-13a | 2.41e-23 | 72 | 26 | 22 | See PrSM>> |
| 392 | CsTx-13a | 1.14e-18 | 72 | 19 | 17 | See PrSM>> |
| 391 | CsTx-13a | 1.60e-12 | 72 | 11 | 11 | See PrSM>> |

All proteins /
CsTx-13a Cupiennius salei toxin 13 isoform a
