# Supplementary material for: The Dual Prey-Inactivation Strategy of Spiders—In-Depth Venomic Analysis of Cupiennius salei
Source: Toxins (Basel). 2019 Mar 19;11(3):167. doi: 10.3390/toxins11030167 (PMC6468893; doi:10.3390/toxins11030167)
Supplement: Supplementary file 1 [file toxins-11-00167-s001.zip › Supplementary Dataset EV1/20180328_f2_topdown_OTMS2_EThcD_NL_i02_ms2_proteoform_cutoff_html/proteoforms/proteoform24.html]

Proteoform #24 from CsTx-12a\_S1 Cupiennius salei toxin 12 isoform a S1^ACsTx-12a\_S2 Cupiennius salei toxin 12 isoform a S2


All proteins /
CsTx-12a\_S1 Cupiennius salei toxin 12 isoform a S1^ACsTx-12a\_S2 Cupiennius salei toxin 12 isoform a S2

## Proteoform #24

5 PrSMs for this proteoform

| Scan | Protein | E-value | # all peaks | # matched peaks | # matched fragment ions | Link |
| --- | --- | --- | --- | --- | --- | --- |
| 281 | CsTx-12a\_S1 | 1.94e-25 | 72 | 37 | 35 | See PrSM>> |
| 280 | CsTx-12a\_S1 | 2.06e-22 | 72 | 33 | 28 | See PrSM>> |
| 289 | CsTx-12a\_S1 | 2.70e-20 | 61 | 27 | 27 | See PrSM>> |
| 288 | CsTx-12a\_S1 | 9.81e-19 | 68 | 26 | 23 | See PrSM>> |
| 297 | CsTx-12a\_S1 | 5.89e-10 | 40 | 14 | 14 | See PrSM>> |

All proteins /
CsTx-12a\_S1 Cupiennius salei toxin 12 isoform a S1^ACsTx-12a\_S2 Cupiennius salei toxin 12 isoform a S2
