# Supplementary material for: The Dual Prey-Inactivation Strategy of Spiders—In-Depth Venomic Analysis of Cupiennius salei
Source: Toxins (Basel). 2019 Mar 19;11(3):167. doi: 10.3390/toxins11030167 (PMC6468893; doi:10.3390/toxins11030167)
Supplement: Supplementary file 1 [file toxins-11-00167-s001.zip › Supplementary Dataset EV1/20180328_f2_topdown_OTMS2_EThcD_NL_i02_ms2_proteoform_cutoff_html/proteoforms/proteoform25.html]

Proteoform #25 from CsTx-12a\_S1 Cupiennius salei toxin 12 isoform a S1^ACsTx-12a\_S2 Cupiennius salei toxin 12 isoform a S2


All proteins /
CsTx-12a\_S1 Cupiennius salei toxin 12 isoform a S1^ACsTx-12a\_S2 Cupiennius salei toxin 12 isoform a S2

## Proteoform #25

13 PrSMs for this proteoform

| Scan | Protein | E-value | # all peaks | # matched peaks | # matched fragment ions | Link |
| --- | --- | --- | --- | --- | --- | --- |
| 331 | CsTx-12a\_S1 | 2.32e-25 | 73 | 37 | 35 | See PrSM>> |
| 341 | CsTx-12a\_S1 | 5.84e-25 | 73 | 36 | 34 | See PrSM>> |
| 315 | CsTx-12a\_S1 | 3.68e-24 | 73 | 35 | 32 | See PrSM>> |
| 323 | CsTx-12a\_S1 | 9.24e-24 | 73 | 34 | 31 | See PrSM>> |
| 309 | CsTx-12a\_S1 | 2.32e-23 | 73 | 31 | 30 | See PrSM>> |
| 348 | CsTx-12a\_S1 | 2.32e-23 | 73 | 32 | 30 | See PrSM>> |
| 361 | CsTx-12a\_S1 | 2.32e-23 | 73 | 33 | 30 | See PrSM>> |
| 377 | CsTx-12a\_S1 | 1.61e-22 | 71 | 29 | 28 | See PrSM>> |
| 355 | CsTx-12a\_S1 | 8.79e-22 | 73 | 29 | 27 | See PrSM>> |
| 311 | CsTx-12a\_S1 | 9.92e-21 | 73 | 28 | 25 | See PrSM>> |
| 316 | CsTx-12a\_S1 | 1.77e-18 | 73 | 25 | 22 | See PrSM>> |
| 349 | CsTx-12a\_S1 | 5.59e-17 | 73 | 23 | 20 | See PrSM>> |
| 324 | CsTx-12a\_S1 | 2.15e-14 | 73 | 20 | 17 | See PrSM>> |

All proteins /
CsTx-12a\_S1 Cupiennius salei toxin 12 isoform a S1^ACsTx-12a\_S2 Cupiennius salei toxin 12 isoform a S2
