# Supplementary material for: The Dual Prey-Inactivation Strategy of Spiders—In-Depth Venomic Analysis of Cupiennius salei
Source: Toxins (Basel). 2019 Mar 19;11(3):167. doi: 10.3390/toxins11030167 (PMC6468893; doi:10.3390/toxins11030167)
Supplement: Supplementary file 1 [file toxins-11-00167-s001.zip › Supplementary Dataset EV1/20180328_f2_topdown_OTMS2_EThcD_NL_i02_ms2_proteoform_cutoff_html/proteoforms/proteoform27.html]

Proteoform #27 from sp|B3EWT7|TXC2B\_CUPSA Cupiennin-2b OS=Cupiennius salei OX=6928 PE=1 SV=1


All proteins /
sp|B3EWT7|TXC2B\_CUPSA Cupiennin-2b OS=Cupiennius salei OX=6928 PE=1 SV=1

## Proteoform #27

115 PrSMs for this proteoform

| Scan | Protein | E-value | # all peaks | # matched peaks | # matched fragment ions | Link |
| --- | --- | --- | --- | --- | --- | --- |
| 1077 | sp|B3EWT7|TXC2B\_CUPSA | 4.00e-25 | 62 | 34 | 28 | See PrSM>> |
| 1220 | sp|B3EWT7|TXC2B\_CUPSA | 4.00e-25 | 62 | 34 | 28 | See PrSM>> |
| 1069 | sp|B3EWT7|TXC2B\_CUPSA | 2.02e-24 | 62 | 34 | 27 | See PrSM>> |
| 1085 | sp|B3EWT7|TXC2B\_CUPSA | 2.02e-24 | 62 | 36 | 27 | See PrSM>> |
| 1284 | sp|B3EWT7|TXC2B\_CUPSA | 2.02e-24 | 62 | 34 | 27 | See PrSM>> |
| 1104 | sp|B3EWT7|TXC2B\_CUPSA | 2.02e-24 | 62 | 35 | 27 | See PrSM>> |
| 1108 | sp|B3EWT7|TXC2B\_CUPSA | 2.02e-24 | 62 | 36 | 27 | See PrSM>> |
| 1213 | sp|B3EWT7|TXC2B\_CUPSA | 2.02e-24 | 62 | 36 | 27 | See PrSM>> |
| 1261 | sp|B3EWT7|TXC2B\_CUPSA | 1.02e-23 | 62 | 35 | 26 | See PrSM>> |
| 1141 | sp|B3EWT7|TXC2B\_CUPSA | 1.02e-23 | 62 | 34 | 26 | See PrSM>> |
| 1149 | sp|B3EWT7|TXC2B\_CUPSA | 1.02e-23 | 62 | 34 | 26 | See PrSM>> |
| 1157 | sp|B3EWT7|TXC2B\_CUPSA | 1.02e-23 | 62 | 34 | 26 | See PrSM>> |
| 1168 | sp|B3EWT7|TXC2B\_CUPSA | 1.02e-23 | 62 | 33 | 26 | See PrSM>> |
| 1181 | sp|B3EWT7|TXC2B\_CUPSA | 1.02e-23 | 62 | 34 | 26 | See PrSM>> |
| 1209 | sp|B3EWT7|TXC2B\_CUPSA | 1.02e-23 | 62 | 34 | 26 | See PrSM>> |
| 1063 | sp|B3EWT7|TXC2B\_CUPSA | 1.02e-23 | 62 | 34 | 26 | See PrSM>> |
| 1269 | sp|B3EWT7|TXC2B\_CUPSA | 1.02e-23 | 62 | 34 | 26 | See PrSM>> |
| 1288 | sp|B3EWT7|TXC2B\_CUPSA | 1.02e-23 | 62 | 34 | 26 | See PrSM>> |
| 1307 | sp|B3EWT7|TXC2B\_CUPSA | 1.02e-23 | 62 | 34 | 26 | See PrSM>> |
| 1323 | sp|B3EWT7|TXC2B\_CUPSA | 1.02e-23 | 62 | 33 | 26 | See PrSM>> |
| 1331 | sp|B3EWT7|TXC2B\_CUPSA | 1.02e-23 | 62 | 33 | 26 | See PrSM>> |
| 1117 | sp|B3EWT7|TXC2B\_CUPSA | 1.02e-23 | 62 | 34 | 26 | See PrSM>> |
| 1101 | sp|B3EWT7|TXC2B\_CUPSA | 1.02e-23 | 62 | 34 | 26 | See PrSM>> |
| 1093 | sp|B3EWT7|TXC2B\_CUPSA | 1.02e-23 | 62 | 35 | 26 | See PrSM>> |
| 1057 | sp|B3EWT7|TXC2B\_CUPSA | 1.02e-23 | 62 | 34 | 26 | See PrSM>> |
| 1125 | sp|B3EWT7|TXC2B\_CUPSA | 1.02e-23 | 62 | 33 | 26 | See PrSM>> |
| 1253 | sp|B3EWT7|TXC2B\_CUPSA | 5.16e-23 | 62 | 35 | 25 | See PrSM>> |
| 1112 | sp|B3EWT7|TXC2B\_CUPSA | 5.16e-23 | 62 | 33 | 25 | See PrSM>> |
| 1371 | sp|B3EWT7|TXC2B\_CUPSA | 5.16e-23 | 62 | 32 | 25 | See PrSM>> |
| 1352 | sp|B3EWT7|TXC2B\_CUPSA | 5.16e-23 | 62 | 32 | 25 | See PrSM>> |
| 1236 | sp|B3EWT7|TXC2B\_CUPSA | 5.16e-23 | 62 | 32 | 25 | See PrSM>> |
| 1244 | sp|B3EWT7|TXC2B\_CUPSA | 5.16e-23 | 62 | 32 | 25 | See PrSM>> |
| 1344 | sp|B3EWT7|TXC2B\_CUPSA | 5.16e-23 | 62 | 32 | 25 | See PrSM>> |
| 1296 | sp|B3EWT7|TXC2B\_CUPSA | 5.16e-23 | 62 | 32 | 25 | See PrSM>> |
| 1315 | sp|B3EWT7|TXC2B\_CUPSA | 5.16e-23 | 62 | 32 | 25 | See PrSM>> |
| 1277 | sp|B3EWT7|TXC2B\_CUPSA | 5.16e-23 | 62 | 32 | 25 | See PrSM>> |
| 1299 | sp|B3EWT7|TXC2B\_CUPSA | 5.16e-23 | 62 | 33 | 25 | See PrSM>> |
| 1293 | sp|B3EWT7|TXC2B\_CUPSA | 5.16e-23 | 62 | 33 | 25 | See PrSM>> |
| 1384 | sp|B3EWT7|TXC2B\_CUPSA | 5.16e-23 | 62 | 33 | 25 | See PrSM>> |
| 1145 | sp|B3EWT7|TXC2B\_CUPSA | 5.16e-23 | 62 | 34 | 25 | See PrSM>> |
| 1133 | sp|B3EWT7|TXC2B\_CUPSA | 5.16e-23 | 62 | 34 | 25 | See PrSM>> |
| 1708 | sp|B3EWT7|TXC2B\_CUPSA | 5.16e-23 | 62 | 32 | 25 | See PrSM>> |
| 1419 | sp|B3EWT7|TXC2B\_CUPSA | 5.16e-23 | 62 | 31 | 25 | See PrSM>> |
| 1165 | sp|B3EWT7|TXC2B\_CUPSA | 5.16e-23 | 62 | 33 | 25 | See PrSM>> |
| 1172 | sp|B3EWT7|TXC2B\_CUPSA | 5.16e-23 | 62 | 34 | 25 | See PrSM>> |
| 1176 | sp|B3EWT7|TXC2B\_CUPSA | 5.16e-23 | 62 | 34 | 25 | See PrSM>> |
| 1120 | sp|B3EWT7|TXC2B\_CUPSA | 5.16e-23 | 62 | 36 | 25 | See PrSM>> |
| 1184 | sp|B3EWT7|TXC2B\_CUPSA | 5.16e-23 | 62 | 33 | 25 | See PrSM>> |
| 1188 | sp|B3EWT7|TXC2B\_CUPSA | 5.16e-23 | 62 | 32 | 25 | See PrSM>> |
| 1196 | sp|B3EWT7|TXC2B\_CUPSA | 5.16e-23 | 62 | 33 | 25 | See PrSM>> |
| 1204 | sp|B3EWT7|TXC2B\_CUPSA | 5.16e-23 | 62 | 33 | 25 | See PrSM>> |
| 1435 | sp|B3EWT7|TXC2B\_CUPSA | 6.06e-23 | 61 | 32 | 25 | See PrSM>> |
| 1347 | sp|B3EWT7|TXC2B\_CUPSA | 1.52e-22 | 62 | 31 | 24 | See PrSM>> |
| 1339 | sp|B3EWT7|TXC2B\_CUPSA | 1.52e-22 | 62 | 31 | 24 | See PrSM>> |
| 1363 | sp|B3EWT7|TXC2B\_CUPSA | 1.52e-22 | 62 | 34 | 24 | See PrSM>> |
| 1320 | sp|B3EWT7|TXC2B\_CUPSA | 1.52e-22 | 62 | 30 | 24 | See PrSM>> |
| 1368 | sp|B3EWT7|TXC2B\_CUPSA | 1.52e-22 | 62 | 32 | 24 | See PrSM>> |
| 1408 | sp|B3EWT7|TXC2B\_CUPSA | 1.52e-22 | 62 | 31 | 24 | See PrSM>> |
| 1073 | sp|B3EWT7|TXC2B\_CUPSA | 1.52e-22 | 62 | 32 | 24 | See PrSM>> |
| 1304 | sp|B3EWT7|TXC2B\_CUPSA | 1.52e-22 | 62 | 33 | 24 | See PrSM>> |
| 1328 | sp|B3EWT7|TXC2B\_CUPSA | 1.52e-22 | 62 | 33 | 24 | See PrSM>> |
| 1080 | sp|B3EWT7|TXC2B\_CUPSA | 1.52e-22 | 62 | 32 | 24 | See PrSM>> |
| 1152 | sp|B3EWT7|TXC2B\_CUPSA | 1.52e-22 | 62 | 32 | 24 | See PrSM>> |
| 1160 | sp|B3EWT7|TXC2B\_CUPSA | 1.52e-22 | 62 | 33 | 24 | See PrSM>> |
| 1193 | sp|B3EWT7|TXC2B\_CUPSA | 1.52e-22 | 62 | 34 | 24 | See PrSM>> |
| 1256 | sp|B3EWT7|TXC2B\_CUPSA | 1.52e-22 | 62 | 31 | 24 | See PrSM>> |
| 1216 | sp|B3EWT7|TXC2B\_CUPSA | 1.52e-22 | 62 | 33 | 24 | See PrSM>> |
| 1227 | sp|B3EWT7|TXC2B\_CUPSA | 1.52e-22 | 62 | 33 | 24 | See PrSM>> |
| 1427 | sp|B3EWT7|TXC2B\_CUPSA | 4.34e-22 | 61 | 28 | 23 | See PrSM>> |
| 1355 | sp|B3EWT7|TXC2B\_CUPSA | 4.34e-22 | 61 | 32 | 23 | See PrSM>> |
| 1088 | sp|B3EWT7|TXC2B\_CUPSA | 4.46e-22 | 62 | 31 | 23 | See PrSM>> |
| 1128 | sp|B3EWT7|TXC2B\_CUPSA | 4.46e-22 | 62 | 31 | 23 | See PrSM>> |
| 1424 | sp|B3EWT7|TXC2B\_CUPSA | 4.46e-22 | 62 | 29 | 23 | See PrSM>> |
| 1392 | sp|B3EWT7|TXC2B\_CUPSA | 4.46e-22 | 62 | 30 | 23 | See PrSM>> |
| 1387 | sp|B3EWT7|TXC2B\_CUPSA | 4.46e-22 | 62 | 30 | 23 | See PrSM>> |
| 1200 | sp|B3EWT7|TXC2B\_CUPSA | 4.46e-22 | 62 | 33 | 23 | See PrSM>> |
| 1379 | sp|B3EWT7|TXC2B\_CUPSA | 4.46e-22 | 62 | 30 | 23 | See PrSM>> |
| 1232 | sp|B3EWT7|TXC2B\_CUPSA | 4.46e-22 | 62 | 29 | 23 | See PrSM>> |
| 1281 | sp|B3EWT7|TXC2B\_CUPSA | 4.46e-22 | 62 | 31 | 23 | See PrSM>> |
| 1241 | sp|B3EWT7|TXC2B\_CUPSA | 4.46e-22 | 62 | 30 | 23 | See PrSM>> |
| 1336 | sp|B3EWT7|TXC2B\_CUPSA | 4.46e-22 | 62 | 31 | 23 | See PrSM>> |
| 1096 | sp|B3EWT7|TXC2B\_CUPSA | 4.46e-22 | 62 | 32 | 23 | See PrSM>> |
| 1136 | sp|B3EWT7|TXC2B\_CUPSA | 4.46e-22 | 62 | 33 | 23 | See PrSM>> |
| 1265 | sp|B3EWT7|TXC2B\_CUPSA | 4.46e-22 | 62 | 31 | 23 | See PrSM>> |
| 1395 | sp|B3EWT7|TXC2B\_CUPSA | 9.75e-22 | 59 | 30 | 23 | See PrSM>> |
| 1416 | sp|B3EWT7|TXC2B\_CUPSA | 9.75e-22 | 59 | 32 | 23 | See PrSM>> |
| 1432 | sp|B3EWT7|TXC2B\_CUPSA | 1.03e-21 | 60 | 27 | 22 | See PrSM>> |
| 1224 | sp|B3EWT7|TXC2B\_CUPSA | 1.31e-21 | 62 | 32 | 22 | See PrSM>> |
| 1704 | sp|B3EWT7|TXC2B\_CUPSA | 1.31e-21 | 62 | 28 | 22 | See PrSM>> |
| 1457 | sp|B3EWT7|TXC2B\_CUPSA | 1.31e-21 | 62 | 28 | 22 | See PrSM>> |
| 1360 | sp|B3EWT7|TXC2B\_CUPSA | 1.31e-21 | 62 | 30 | 22 | See PrSM>> |
| 1312 | sp|B3EWT7|TXC2B\_CUPSA | 1.31e-21 | 62 | 29 | 22 | See PrSM>> |
| 1273 | sp|B3EWT7|TXC2B\_CUPSA | 1.31e-21 | 62 | 29 | 22 | See PrSM>> |
| 1403 | sp|B3EWT7|TXC2B\_CUPSA | 1.31e-21 | 62 | 29 | 22 | See PrSM>> |
| 1401 | sp|B3EWT7|TXC2B\_CUPSA | 1.31e-21 | 62 | 30 | 22 | See PrSM>> |
| 1376 | sp|B3EWT7|TXC2B\_CUPSA | 1.31e-21 | 62 | 29 | 22 | See PrSM>> |
| 1249 | sp|B3EWT7|TXC2B\_CUPSA | 1.31e-21 | 62 | 29 | 22 | See PrSM>> |
| 1412 | sp|B3EWT7|TXC2B\_CUPSA | 2.25e-21 | 58 | 30 | 23 | See PrSM>> |
| 1443 | sp|B3EWT7|TXC2B\_CUPSA | 2.49e-21 | 59 | 29 | 22 | See PrSM>> |
| 1473 | sp|B3EWT7|TXC2B\_CUPSA | 2.49e-21 | 59 | 25 | 22 | See PrSM>> |
| 1465 | sp|B3EWT7|TXC2B\_CUPSA | 3.85e-21 | 62 | 26 | 21 | See PrSM>> |
| 1459 | sp|B3EWT7|TXC2B\_CUPSA | 9.04e-21 | 55 | 29 | 24 | See PrSM>> |
| 1699 | sp|B3EWT7|TXC2B\_CUPSA | 1.13e-20 | 62 | 27 | 20 | See PrSM>> |
| 1065 | sp|B3EWT7|TXC2B\_CUPSA | 1.13e-20 | 62 | 27 | 20 | See PrSM>> |
| 1461 | sp|B3EWT7|TXC2B\_CUPSA | 4.01e-20 | 57 | 26 | 21 | See PrSM>> |
| 1448 | sp|B3EWT7|TXC2B\_CUPSA | 4.01e-20 | 57 | 28 | 21 | See PrSM>> |
| 1475 | sp|B3EWT7|TXC2B\_CUPSA | 8.99e-20 | 52 | 27 | 22 | See PrSM>> |
| 1467 | sp|B3EWT7|TXC2B\_CUPSA | 2.69e-19 | 54 | 26 | 21 | See PrSM>> |
| 1451 | sp|B3EWT7|TXC2B\_CUPSA | 1.04e-18 | 52 | 26 | 20 | See PrSM>> |
| 1483 | sp|B3EWT7|TXC2B\_CUPSA | 1.10e-17 | 50 | 21 | 17 | See PrSM>> |
| 1440 | sp|B3EWT7|TXC2B\_CUPSA | 1.88e-17 | 55 | 24 | 18 | See PrSM>> |
| 1491 | sp|B3EWT7|TXC2B\_CUPSA | 1.32e-15 | 46 | 19 | 16 | See PrSM>> |
| 1721 | sp|B3EWT7|TXC2B\_CUPSA | 3.25e-14 | 53 | 14 | 13 | See PrSM>> |
| 1499 | sp|B3EWT7|TXC2B\_CUPSA | 1.18e-13 | 34 | 15 | 14 | See PrSM>> |
| 1484 | sp|B3EWT7|TXC2B\_CUPSA | 3.94e-11 | 34 | 10 | 10 | See PrSM>> |

All proteins /
sp|B3EWT7|TXC2B\_CUPSA Cupiennin-2b OS=Cupiennius salei OX=6928 PE=1 SV=1
