# Supplementary material for: The Dual Prey-Inactivation Strategy of Spiders—In-Depth Venomic Analysis of Cupiennius salei
Source: Toxins (Basel). 2019 Mar 19;11(3):167. doi: 10.3390/toxins11030167 (PMC6468893; doi:10.3390/toxins11030167)
Supplement: Supplementary file 1 [file toxins-11-00167-s001.zip › Supplementary Dataset EV1/20180328_f2_topdown_OTMS2_EThcD_NL_i02_ms2_proteoform_cutoff_html/proteoforms/proteoform29.html]

Proteoform #29 from sp|B3EWU0|TXC2E\_CUPSA Cupiennin-2e OS=Cupiennius salei OX=6928 PE=1 SV=1


All proteins /
sp|B3EWU0|TXC2E\_CUPSA Cupiennin-2e OS=Cupiennius salei OX=6928 PE=1 SV=1

## Proteoform #29

33 PrSMs for this proteoform

| Scan | Protein | E-value | # all peaks | # matched peaks | # matched fragment ions | Link |
| --- | --- | --- | --- | --- | --- | --- |
| 904 | sp|B3EWU0|TXC2E\_CUPSA | 9.14e-25 | 61 | 35 | 28 | See PrSM>> |
| 953 | sp|B3EWU0|TXC2E\_CUPSA | 3.70e-24 | 61 | 34 | 27 | See PrSM>> |
| 936 | sp|B3EWU0|TXC2E\_CUPSA | 1.50e-23 | 61 | 31 | 26 | See PrSM>> |
| 929 | sp|B3EWU0|TXC2E\_CUPSA | 1.50e-23 | 61 | 30 | 26 | See PrSM>> |
| 920 | sp|B3EWU0|TXC2E\_CUPSA | 1.50e-23 | 61 | 33 | 26 | See PrSM>> |
| 952 | sp|B3EWU0|TXC2E\_CUPSA | 6.06e-23 | 61 | 30 | 25 | See PrSM>> |
| 937 | sp|B3EWU0|TXC2E\_CUPSA | 6.06e-23 | 61 | 31 | 25 | See PrSM>> |
| 928 | sp|B3EWU0|TXC2E\_CUPSA | 6.06e-23 | 61 | 31 | 25 | See PrSM>> |
| 921 | sp|B3EWU0|TXC2E\_CUPSA | 6.06e-23 | 61 | 30 | 25 | See PrSM>> |
| 911 | sp|B3EWU0|TXC2E\_CUPSA | 6.06e-23 | 61 | 30 | 25 | See PrSM>> |
| 905 | sp|B3EWU0|TXC2E\_CUPSA | 6.06e-23 | 61 | 29 | 25 | See PrSM>> |
| 960 | sp|B3EWU0|TXC2E\_CUPSA | 6.06e-23 | 61 | 31 | 25 | See PrSM>> |
| 896 | sp|B3EWU0|TXC2E\_CUPSA | 6.06e-23 | 61 | 30 | 25 | See PrSM>> |
| 985 | sp|B3EWU0|TXC2E\_CUPSA | 6.06e-23 | 61 | 30 | 25 | See PrSM>> |
| 877 | sp|B3EWU0|TXC2E\_CUPSA | 6.06e-23 | 61 | 30 | 25 | See PrSM>> |
| 977 | sp|B3EWU0|TXC2E\_CUPSA | 1.62e-22 | 61 | 29 | 24 | See PrSM>> |
| 880 | sp|B3EWU0|TXC2E\_CUPSA | 1.62e-22 | 61 | 28 | 24 | See PrSM>> |
| 993 | sp|B3EWU0|TXC2E\_CUPSA | 1.62e-22 | 61 | 28 | 24 | See PrSM>> |
| 872 | sp|B3EWU0|TXC2E\_CUPSA | 1.62e-22 | 61 | 29 | 24 | See PrSM>> |
| 1025 | sp|B3EWU0|TXC2E\_CUPSA | 1.62e-22 | 61 | 30 | 24 | See PrSM>> |
| 864 | sp|B3EWU0|TXC2E\_CUPSA | 4.34e-22 | 61 | 28 | 23 | See PrSM>> |
| 889 | sp|B3EWU0|TXC2E\_CUPSA | 4.34e-22 | 61 | 29 | 23 | See PrSM>> |
| 913 | sp|B3EWU0|TXC2E\_CUPSA | 1.16e-21 | 61 | 31 | 22 | See PrSM>> |
| 897 | sp|B3EWU0|TXC2E\_CUPSA | 1.16e-21 | 61 | 30 | 22 | See PrSM>> |
| 961 | sp|B3EWU0|TXC2E\_CUPSA | 1.16e-21 | 61 | 26 | 22 | See PrSM>> |
| 887 | sp|B3EWU0|TXC2E\_CUPSA | 1.16e-21 | 61 | 26 | 22 | See PrSM>> |
| 873 | sp|B3EWU0|TXC2E\_CUPSA | 1.16e-21 | 61 | 27 | 22 | See PrSM>> |
| 1001 | sp|B3EWU0|TXC2E\_CUPSA | 1.16e-21 | 61 | 26 | 22 | See PrSM>> |
| 1009 | sp|B3EWU0|TXC2E\_CUPSA | 1.16e-21 | 61 | 26 | 22 | See PrSM>> |
| 1041 | sp|B3EWU0|TXC2E\_CUPSA | 3.12e-21 | 61 | 27 | 21 | See PrSM>> |
| 1049 | sp|B3EWU0|TXC2E\_CUPSA | 8.34e-21 | 61 | 26 | 20 | See PrSM>> |
| 863 | sp|B3EWU0|TXC2E\_CUPSA | 3.31e-18 | 61 | 20 | 17 | See PrSM>> |
| 1329 | sp|B3EWU0|TXC2E\_CUPSA | 8.46e-11 | 41 | 12 | 11 | See PrSM>> |

All proteins /
sp|B3EWU0|TXC2E\_CUPSA Cupiennin-2e OS=Cupiennius salei OX=6928 PE=1 SV=1
