# Supplementary material for: The Dual Prey-Inactivation Strategy of Spiders—In-Depth Venomic Analysis of Cupiennius salei
Source: Toxins (Basel). 2019 Mar 19;11(3):167. doi: 10.3390/toxins11030167 (PMC6468893; doi:10.3390/toxins11030167)
Supplement: Supplementary file 1 [file toxins-11-00167-s001.zip › Supplementary Dataset EV1/20180328_f2_topdown_OTMS2_EThcD_NL_i02_ms2_proteoform_cutoff_html/proteoforms/proteoform30.html]

Proteoform #30 from sp|B3EWT9|TXC2D\_CUPSA Cupiennin-2d OS=Cupiennius salei OX=6928 PE=1 SV=1


All proteins /
sp|B3EWT9|TXC2D\_CUPSA Cupiennin-2d OS=Cupiennius salei OX=6928 PE=1 SV=1

## Proteoform #30

56 PrSMs for this proteoform

| Scan | Protein | E-value | # all peaks | # matched peaks | # matched fragment ions | Link |
| --- | --- | --- | --- | --- | --- | --- |
| 1340 | sp|B3EWT9|TXC2D\_CUPSA | 5.55e-24 | 61 | 27 | 22 | See PrSM>> |
| 1308 | sp|B3EWT9|TXC2D\_CUPSA | 1.02e-23 | 61 | 27 | 21 | See PrSM>> |
| 1035 | sp|B3EWT9|TXC2D\_CUPSA | 1.02e-23 | 61 | 25 | 21 | See PrSM>> |
| 1380 | sp|B3EWT9|TXC2D\_CUPSA | 1.02e-23 | 61 | 26 | 21 | See PrSM>> |
| 1411 | sp|B3EWT9|TXC2D\_CUPSA | 1.02e-23 | 61 | 26 | 21 | See PrSM>> |
| 1716 | sp|B3EWT9|TXC2D\_CUPSA | 1.86e-23 | 61 | 24 | 20 | See PrSM>> |
| 1317 | sp|B3EWT9|TXC2D\_CUPSA | 1.86e-23 | 61 | 26 | 20 | See PrSM>> |
| 1332 | sp|B3EWT9|TXC2D\_CUPSA | 1.86e-23 | 61 | 25 | 20 | See PrSM>> |
| 1164 | sp|B3EWT9|TXC2D\_CUPSA | 1.86e-23 | 61 | 26 | 20 | See PrSM>> |
| 1396 | sp|B3EWT9|TXC2D\_CUPSA | 1.86e-23 | 61 | 24 | 20 | See PrSM>> |
| 1404 | sp|B3EWT9|TXC2D\_CUPSA | 1.86e-23 | 61 | 26 | 20 | See PrSM>> |
| 1436 | sp|B3EWT9|TXC2D\_CUPSA | 1.86e-23 | 61 | 23 | 20 | See PrSM>> |
| 1083 | sp|B3EWT9|TXC2D\_CUPSA | 1.86e-23 | 61 | 25 | 20 | See PrSM>> |
| 1075 | sp|B3EWT9|TXC2D\_CUPSA | 1.86e-23 | 61 | 26 | 20 | See PrSM>> |
| 1059 | sp|B3EWT9|TXC2D\_CUPSA | 1.86e-23 | 61 | 25 | 20 | See PrSM>> |
| 1052 | sp|B3EWT9|TXC2D\_CUPSA | 1.86e-23 | 61 | 24 | 20 | See PrSM>> |
| 1043 | sp|B3EWT9|TXC2D\_CUPSA | 1.86e-23 | 61 | 25 | 20 | See PrSM>> |
| 1011 | sp|B3EWT9|TXC2D\_CUPSA | 1.86e-23 | 61 | 24 | 20 | See PrSM>> |
| 915 | sp|B3EWT9|TXC2D\_CUPSA | 1.86e-23 | 61 | 26 | 20 | See PrSM>> |
| 1463 | sp|B3EWT9|TXC2D\_CUPSA | 1.62e-22 | 61 | 28 | 24 | See PrSM>> |
| 1437 | sp|B3EWT9|TXC2D\_CUPSA | 1.62e-22 | 61 | 25 | 19 | See PrSM>> |
| 1445 | sp|B3EWT9|TXC2D\_CUPSA | 1.62e-22 | 61 | 23 | 19 | See PrSM>> |
| 1388 | sp|B3EWT9|TXC2D\_CUPSA | 1.62e-22 | 61 | 23 | 19 | See PrSM>> |
| 1356 | sp|B3EWT9|TXC2D\_CUPSA | 1.62e-22 | 61 | 24 | 19 | See PrSM>> |
| 1348 | sp|B3EWT9|TXC2D\_CUPSA | 1.62e-22 | 61 | 25 | 19 | See PrSM>> |
| 899 | sp|B3EWT9|TXC2D\_CUPSA | 1.62e-22 | 61 | 24 | 19 | See PrSM>> |
| 1283 | sp|B3EWT9|TXC2D\_CUPSA | 1.62e-22 | 61 | 25 | 19 | See PrSM>> |
| 1275 | sp|B3EWT9|TXC2D\_CUPSA | 1.62e-22 | 61 | 24 | 19 | See PrSM>> |
| 1251 | sp|B3EWT9|TXC2D\_CUPSA | 1.62e-22 | 61 | 26 | 19 | See PrSM>> |
| 1067 | sp|B3EWT9|TXC2D\_CUPSA | 1.62e-22 | 61 | 24 | 19 | See PrSM>> |
| 1091 | sp|B3EWT9|TXC2D\_CUPSA | 1.62e-22 | 61 | 24 | 19 | See PrSM>> |
| 1219 | sp|B3EWT9|TXC2D\_CUPSA | 1.62e-22 | 61 | 25 | 19 | See PrSM>> |
| 1099 | sp|B3EWT9|TXC2D\_CUPSA | 1.62e-22 | 61 | 25 | 19 | See PrSM>> |
| 1179 | sp|B3EWT9|TXC2D\_CUPSA | 1.62e-22 | 61 | 24 | 19 | See PrSM>> |
| 1171 | sp|B3EWT9|TXC2D\_CUPSA | 1.62e-22 | 61 | 25 | 19 | See PrSM>> |
| 1107 | sp|B3EWT9|TXC2D\_CUPSA | 1.62e-22 | 61 | 25 | 19 | See PrSM>> |
| 1155 | sp|B3EWT9|TXC2D\_CUPSA | 1.62e-22 | 61 | 25 | 19 | See PrSM>> |
| 1115 | sp|B3EWT9|TXC2D\_CUPSA | 1.62e-22 | 61 | 26 | 19 | See PrSM>> |
| 1123 | sp|B3EWT9|TXC2D\_CUPSA | 1.62e-22 | 61 | 25 | 19 | See PrSM>> |
| 867 | sp|B3EWT9|TXC2D\_CUPSA | 1.41e-21 | 61 | 24 | 18 | See PrSM>> |
| 1260 | sp|B3EWT9|TXC2D\_CUPSA | 1.41e-21 | 61 | 24 | 18 | See PrSM>> |
| 1428 | sp|B3EWT9|TXC2D\_CUPSA | 1.41e-21 | 61 | 21 | 18 | See PrSM>> |
| 1420 | sp|B3EWT9|TXC2D\_CUPSA | 1.41e-21 | 61 | 24 | 18 | See PrSM>> |
| 1147 | sp|B3EWT9|TXC2D\_CUPSA | 1.41e-21 | 61 | 23 | 18 | See PrSM>> |
| 1372 | sp|B3EWT9|TXC2D\_CUPSA | 1.41e-21 | 61 | 23 | 18 | See PrSM>> |
| 1187 | sp|B3EWT9|TXC2D\_CUPSA | 1.41e-21 | 61 | 24 | 18 | See PrSM>> |
| 1203 | sp|B3EWT9|TXC2D\_CUPSA | 1.41e-21 | 61 | 24 | 18 | See PrSM>> |
| 1212 | sp|B3EWT9|TXC2D\_CUPSA | 1.41e-21 | 61 | 25 | 18 | See PrSM>> |
| 1243 | sp|B3EWT9|TXC2D\_CUPSA | 1.41e-21 | 61 | 25 | 18 | See PrSM>> |
| 1267 | sp|B3EWT9|TXC2D\_CUPSA | 1.41e-21 | 61 | 24 | 18 | See PrSM>> |
| 1460 | sp|B3EWT9|TXC2D\_CUPSA | 8.75e-21 | 54 | 22 | 20 | See PrSM>> |
| 1291 | sp|B3EWT9|TXC2D\_CUPSA | 1.23e-20 | 61 | 23 | 17 | See PrSM>> |
| 1700 | sp|B3EWT9|TXC2D\_CUPSA | 1.23e-20 | 61 | 20 | 17 | See PrSM>> |
| 1452 | sp|B3EWT9|TXC2D\_CUPSA | 3.09e-20 | 56 | 25 | 19 | See PrSM>> |
| 1453 | sp|B3EWT9|TXC2D\_CUPSA | 1.07e-19 | 61 | 22 | 16 | See PrSM>> |
| 955 | sp|B3EWT9|TXC2D\_CUPSA | 1.07e-19 | 61 | 18 | 16 | See PrSM>> |

All proteins /
sp|B3EWT9|TXC2D\_CUPSA Cupiennin-2d OS=Cupiennius salei OX=6928 PE=1 SV=1
