# Supplementary material for: The Dual Prey-Inactivation Strategy of Spiders—In-Depth Venomic Analysis of Cupiennius salei
Source: Toxins (Basel). 2019 Mar 19;11(3):167. doi: 10.3390/toxins11030167 (PMC6468893; doi:10.3390/toxins11030167)
Supplement: Supplementary file 1 [file toxins-11-00167-s001.zip › Supplementary Dataset EV1/20180328_f2_topdown_OTMS2_EThcD_NL_i02_ms2_proteoform_cutoff_html/proteoforms/proteoform33.html]

Proteoform #33 from sp|B3EWT8|TXC2C\_CUPSA Cupiennin-2c OS=Cupiennius salei OX=6928 PE=1 SV=1


All proteins /
sp|B3EWT8|TXC2C\_CUPSA Cupiennin-2c OS=Cupiennius salei OX=6928 PE=1 SV=1

## Proteoform #33

134 PrSMs for this proteoform

| Scan | Protein | E-value | # all peaks | # matched peaks | # matched fragment ions | Link |
| --- | --- | --- | --- | --- | --- | --- |
| 1005 | sp|B3EWT8|TXC2C\_CUPSA | 1.86e-23 | 61 | 24 | 20 | See PrSM>> |
| 1029 | sp|B3EWT8|TXC2C\_CUPSA | 1.86e-23 | 61 | 26 | 20 | See PrSM>> |
| 901 | sp|B3EWT8|TXC2C\_CUPSA | 6.06e-23 | 61 | 29 | 25 | See PrSM>> |
| 989 | sp|B3EWT8|TXC2C\_CUPSA | 1.62e-22 | 61 | 29 | 24 | See PrSM>> |
| 957 | sp|B3EWT8|TXC2C\_CUPSA | 1.62e-22 | 61 | 27 | 24 | See PrSM>> |
| 1383 | sp|B3EWT8|TXC2C\_CUPSA | 1.62e-22 | 61 | 30 | 24 | See PrSM>> |
| 1319 | sp|B3EWT8|TXC2C\_CUPSA | 1.62e-22 | 61 | 25 | 19 | See PrSM>> |
| 991 | sp|B3EWT8|TXC2C\_CUPSA | 1.62e-22 | 61 | 22 | 19 | See PrSM>> |
| 1135 | sp|B3EWT8|TXC2C\_CUPSA | 1.62e-22 | 61 | 23 | 19 | See PrSM>> |
| 973 | sp|B3EWT8|TXC2C\_CUPSA | 4.34e-22 | 61 | 27 | 23 | See PrSM>> |
| 1071 | sp|B3EWT8|TXC2C\_CUPSA | 4.34e-22 | 61 | 26 | 23 | See PrSM>> |
| 949 | sp|B3EWT8|TXC2C\_CUPSA | 4.34e-22 | 61 | 26 | 23 | See PrSM>> |
| 981 | sp|B3EWT8|TXC2C\_CUPSA | 4.34e-22 | 61 | 26 | 23 | See PrSM>> |
| 1053 | sp|B3EWT8|TXC2C\_CUPSA | 1.16e-21 | 61 | 26 | 22 | See PrSM>> |
| 925 | sp|B3EWT8|TXC2C\_CUPSA | 1.16e-21 | 61 | 25 | 22 | See PrSM>> |
| 1013 | sp|B3EWT8|TXC2C\_CUPSA | 1.16e-21 | 61 | 27 | 22 | See PrSM>> |
| 1303 | sp|B3EWT8|TXC2C\_CUPSA | 1.16e-21 | 61 | 28 | 22 | See PrSM>> |
| 1271 | sp|B3EWT8|TXC2C\_CUPSA | 1.41e-21 | 61 | 23 | 18 | See PrSM>> |
| 1287 | sp|B3EWT8|TXC2C\_CUPSA | 1.41e-21 | 61 | 21 | 18 | See PrSM>> |
| 1215 | sp|B3EWT8|TXC2C\_CUPSA | 1.41e-21 | 61 | 23 | 18 | See PrSM>> |
| 1021 | sp|B3EWT8|TXC2C\_CUPSA | 1.41e-21 | 61 | 23 | 18 | See PrSM>> |
| 1199 | sp|B3EWT8|TXC2C\_CUPSA | 1.41e-21 | 61 | 23 | 18 | See PrSM>> |
| 1263 | sp|B3EWT8|TXC2C\_CUPSA | 1.41e-21 | 61 | 23 | 18 | See PrSM>> |
| 1037 | sp|B3EWT8|TXC2C\_CUPSA | 1.41e-21 | 61 | 24 | 18 | See PrSM>> |
| 1127 | sp|B3EWT8|TXC2C\_CUPSA | 1.41e-21 | 61 | 23 | 18 | See PrSM>> |
| 967 | sp|B3EWT8|TXC2C\_CUPSA | 1.41e-21 | 61 | 21 | 18 | See PrSM>> |
| 1327 | sp|B3EWT8|TXC2C\_CUPSA | 1.41e-21 | 61 | 24 | 18 | See PrSM>> |
| 917 | sp|B3EWT8|TXC2C\_CUPSA | 1.41e-21 | 61 | 22 | 18 | See PrSM>> |
| 1191 | sp|B3EWT8|TXC2C\_CUPSA | 1.41e-21 | 61 | 22 | 18 | See PrSM>> |
| 941 | sp|B3EWT8|TXC2C\_CUPSA | 1.41e-21 | 61 | 21 | 18 | See PrSM>> |
| 1183 | sp|B3EWT8|TXC2C\_CUPSA | 1.41e-21 | 61 | 22 | 18 | See PrSM>> |
| 1045 | sp|B3EWT8|TXC2C\_CUPSA | 3.12e-21 | 61 | 26 | 21 | See PrSM>> |
| 1391 | sp|B3EWT8|TXC2C\_CUPSA | 3.12e-21 | 61 | 25 | 21 | See PrSM>> |
| 1111 | sp|B3EWT8|TXC2C\_CUPSA | 8.34e-21 | 61 | 25 | 20 | See PrSM>> |
| 1407 | sp|B3EWT8|TXC2C\_CUPSA | 8.34e-21 | 61 | 25 | 20 | See PrSM>> |
| 1415 | sp|B3EWT8|TXC2C\_CUPSA | 8.34e-21 | 61 | 25 | 20 | See PrSM>> |
| 1143 | sp|B3EWT8|TXC2C\_CUPSA | 1.23e-20 | 61 | 21 | 17 | See PrSM>> |
| 1061 | sp|B3EWT8|TXC2C\_CUPSA | 1.23e-20 | 61 | 22 | 17 | See PrSM>> |
| 1079 | sp|B3EWT8|TXC2C\_CUPSA | 1.23e-20 | 61 | 22 | 17 | See PrSM>> |
| 1095 | sp|B3EWT8|TXC2C\_CUPSA | 1.23e-20 | 61 | 21 | 17 | See PrSM>> |
| 1119 | sp|B3EWT8|TXC2C\_CUPSA | 1.23e-20 | 61 | 20 | 17 | See PrSM>> |
| 912 | sp|B3EWT8|TXC2C\_CUPSA | 1.23e-20 | 61 | 20 | 17 | See PrSM>> |
| 909 | sp|B3EWT8|TXC2C\_CUPSA | 1.23e-20 | 61 | 20 | 17 | See PrSM>> |
| 1295 | sp|B3EWT8|TXC2C\_CUPSA | 1.23e-20 | 61 | 22 | 17 | See PrSM>> |
| 1311 | sp|B3EWT8|TXC2C\_CUPSA | 1.23e-20 | 61 | 22 | 17 | See PrSM>> |
| 1279 | sp|B3EWT8|TXC2C\_CUPSA | 1.23e-20 | 61 | 23 | 17 | See PrSM>> |
| 1399 | sp|B3EWT8|TXC2C\_CUPSA | 1.23e-20 | 61 | 21 | 17 | See PrSM>> |
| 1167 | sp|B3EWT8|TXC2C\_CUPSA | 1.23e-20 | 61 | 20 | 17 | See PrSM>> |
| 1159 | sp|B3EWT8|TXC2C\_CUPSA | 1.23e-20 | 61 | 22 | 17 | See PrSM>> |
| 959 | sp|B3EWT8|TXC2C\_CUPSA | 1.23e-20 | 61 | 19 | 17 | See PrSM>> |
| 965 | sp|B3EWT8|TXC2C\_CUPSA | 1.23e-20 | 61 | 20 | 17 | See PrSM>> |
| 1367 | sp|B3EWT8|TXC2C\_CUPSA | 6.13e-20 | 61 | 24 | 19 | See PrSM>> |
| 1175 | sp|B3EWT8|TXC2C\_CUPSA | 1.07e-19 | 61 | 20 | 16 | See PrSM>> |
| 1207 | sp|B3EWT8|TXC2C\_CUPSA | 1.07e-19 | 61 | 21 | 16 | See PrSM>> |
| 933 | sp|B3EWT8|TXC2C\_CUPSA | 1.07e-19 | 61 | 18 | 16 | See PrSM>> |
| 1103 | sp|B3EWT8|TXC2C\_CUPSA | 1.07e-19 | 61 | 21 | 16 | See PrSM>> |
| 1231 | sp|B3EWT8|TXC2C\_CUPSA | 1.07e-19 | 61 | 18 | 16 | See PrSM>> |
| 1087 | sp|B3EWT8|TXC2C\_CUPSA | 1.07e-19 | 61 | 20 | 16 | See PrSM>> |
| 935 | sp|B3EWT8|TXC2C\_CUPSA | 1.07e-19 | 61 | 19 | 16 | See PrSM>> |
| 1255 | sp|B3EWT8|TXC2C\_CUPSA | 1.07e-19 | 61 | 21 | 16 | See PrSM>> |
| 1039 | sp|B3EWT8|TXC2C\_CUPSA | 1.07e-19 | 61 | 18 | 16 | See PrSM>> |
| 1359 | sp|B3EWT8|TXC2C\_CUPSA | 1.07e-19 | 61 | 19 | 16 | See PrSM>> |
| 1000 | sp|B3EWT8|TXC2C\_CUPSA | 1.07e-19 | 61 | 17 | 16 | See PrSM>> |
| 1423 | sp|B3EWT8|TXC2C\_CUPSA | 4.50e-19 | 61 | 24 | 18 | See PrSM>> |
| 1431 | sp|B3EWT8|TXC2C\_CUPSA | 7.29e-19 | 59 | 20 | 15 | See PrSM>> |
| 1375 | sp|B3EWT8|TXC2C\_CUPSA | 8.42e-19 | 60 | 21 | 15 | See PrSM>> |
| 1223 | sp|B3EWT8|TXC2C\_CUPSA | 9.31e-19 | 61 | 19 | 15 | See PrSM>> |
| 1313 | sp|B3EWT8|TXC2C\_CUPSA | 9.31e-19 | 61 | 18 | 15 | See PrSM>> |
| 1335 | sp|B3EWT8|TXC2C\_CUPSA | 9.31e-19 | 61 | 20 | 15 | See PrSM>> |
| 1343 | sp|B3EWT8|TXC2C\_CUPSA | 9.31e-19 | 61 | 20 | 15 | See PrSM>> |
| 1345 | sp|B3EWT8|TXC2C\_CUPSA | 9.31e-19 | 61 | 16 | 15 | See PrSM>> |
| 885 | sp|B3EWT8|TXC2C\_CUPSA | 9.31e-19 | 61 | 18 | 15 | See PrSM>> |
| 1151 | sp|B3EWT8|TXC2C\_CUPSA | 9.31e-19 | 61 | 17 | 15 | See PrSM>> |
| 893 | sp|B3EWT8|TXC2C\_CUPSA | 9.31e-19 | 61 | 17 | 15 | See PrSM>> |
| 895 | sp|B3EWT8|TXC2C\_CUPSA | 9.31e-19 | 61 | 18 | 15 | See PrSM>> |
| 976 | sp|B3EWT8|TXC2C\_CUPSA | 9.31e-19 | 61 | 17 | 15 | See PrSM>> |
| 983 | sp|B3EWT8|TXC2C\_CUPSA | 9.31e-19 | 61 | 17 | 15 | See PrSM>> |
| 997 | sp|B3EWT8|TXC2C\_CUPSA | 9.31e-19 | 61 | 19 | 15 | See PrSM>> |
| 1007 | sp|B3EWT8|TXC2C\_CUPSA | 9.31e-19 | 61 | 17 | 15 | See PrSM>> |
| 1016 | sp|B3EWT8|TXC2C\_CUPSA | 9.31e-19 | 61 | 17 | 15 | See PrSM>> |
| 1032 | sp|B3EWT8|TXC2C\_CUPSA | 9.31e-19 | 61 | 15 | 15 | See PrSM>> |
| 1455 | sp|B3EWT8|TXC2C\_CUPSA | 2.07e-18 | 60 | 21 | 17 | See PrSM>> |
| 1447 | sp|B3EWT8|TXC2C\_CUPSA | 3.27e-18 | 53 | 21 | 19 | See PrSM>> |
| 1023 | sp|B3EWT8|TXC2C\_CUPSA | 3.31e-18 | 61 | 20 | 17 | See PrSM>> |
| 1239 | sp|B3EWT8|TXC2C\_CUPSA | 3.31e-18 | 61 | 20 | 17 | See PrSM>> |
| 1481 | sp|B3EWT8|TXC2C\_CUPSA | 6.09e-18 | 51 | 17 | 14 | See PrSM>> |
| 1439 | sp|B3EWT8|TXC2C\_CUPSA | 6.18e-18 | 56 | 17 | 14 | See PrSM>> |
| 1351 | sp|B3EWT8|TXC2C\_CUPSA | 1.23e-17 | 61 | 18 | 14 | See PrSM>> |
| 1217 | sp|B3EWT8|TXC2C\_CUPSA | 1.23e-17 | 61 | 15 | 14 | See PrSM>> |
| 1321 | sp|B3EWT8|TXC2C\_CUPSA | 1.23e-17 | 61 | 16 | 14 | See PrSM>> |
| 943 | sp|B3EWT8|TXC2C\_CUPSA | 1.23e-17 | 61 | 15 | 14 | See PrSM>> |
| 1409 | sp|B3EWT8|TXC2C\_CUPSA | 1.23e-17 | 61 | 15 | 14 | See PrSM>> |
| 1247 | sp|B3EWT8|TXC2C\_CUPSA | 1.23e-17 | 61 | 18 | 14 | See PrSM>> |
| 1272 | sp|B3EWT8|TXC2C\_CUPSA | 1.23e-17 | 61 | 15 | 14 | See PrSM>> |
| 1701 | sp|B3EWT8|TXC2C\_CUPSA | 1.44e-17 | 60 | 21 | 16 | See PrSM>> |
| 1469 | sp|B3EWT8|TXC2C\_CUPSA | 4.35e-17 | 56 | 21 | 17 | See PrSM>> |
| 1472 | sp|B3EWT8|TXC2C\_CUPSA | 9.31e-17 | 55 | 19 | 17 | See PrSM>> |
| 1477 | sp|B3EWT8|TXC2C\_CUPSA | 1.36e-16 | 49 | 19 | 15 | See PrSM>> |
| 1177 | sp|B3EWT8|TXC2C\_CUPSA | 1.62e-16 | 61 | 14 | 13 | See PrSM>> |
| 1393 | sp|B3EWT8|TXC2C\_CUPSA | 1.62e-16 | 61 | 16 | 13 | See PrSM>> |
| 1353 | sp|B3EWT8|TXC2C\_CUPSA | 1.62e-16 | 61 | 15 | 13 | See PrSM>> |
| 951 | sp|B3EWT8|TXC2C\_CUPSA | 1.62e-16 | 61 | 15 | 13 | See PrSM>> |
| 919 | sp|B3EWT8|TXC2C\_CUPSA | 1.62e-16 | 61 | 16 | 13 | See PrSM>> |
| 888 | sp|B3EWT8|TXC2C\_CUPSA | 1.62e-16 | 61 | 14 | 13 | See PrSM>> |
| 1449 | sp|B3EWT8|TXC2C\_CUPSA | 1.84e-15 | 60 | 16 | 12 | See PrSM>> |
| 1305 | sp|B3EWT8|TXC2C\_CUPSA | 2.13e-15 | 61 | 14 | 12 | See PrSM>> |
| 1097 | sp|B3EWT8|TXC2C\_CUPSA | 2.13e-15 | 61 | 13 | 12 | See PrSM>> |
| 1064 | sp|B3EWT8|TXC2C\_CUPSA | 2.13e-15 | 61 | 13 | 12 | See PrSM>> |
| 903 | sp|B3EWT8|TXC2C\_CUPSA | 2.13e-15 | 61 | 14 | 12 | See PrSM>> |
| 1280 | sp|B3EWT8|TXC2C\_CUPSA | 2.13e-15 | 61 | 12 | 12 | See PrSM>> |
| 1369 | sp|B3EWT8|TXC2C\_CUPSA | 2.13e-15 | 61 | 14 | 12 | See PrSM>> |
| 1289 | sp|B3EWT8|TXC2C\_CUPSA | 2.13e-15 | 61 | 13 | 12 | See PrSM>> |
| 1297 | sp|B3EWT8|TXC2C\_CUPSA | 2.13e-15 | 61 | 15 | 12 | See PrSM>> |
| 1464 | sp|B3EWT8|TXC2C\_CUPSA | 1.16e-14 | 50 | 17 | 13 | See PrSM>> |
| 1257 | sp|B3EWT8|TXC2C\_CUPSA | 2.81e-14 | 61 | 13 | 11 | See PrSM>> |
| 1385 | sp|B3EWT8|TXC2C\_CUPSA | 2.81e-14 | 61 | 13 | 11 | See PrSM>> |
| 1377 | sp|B3EWT8|TXC2C\_CUPSA | 2.81e-14 | 61 | 12 | 11 | See PrSM>> |
| 879 | sp|B3EWT8|TXC2C\_CUPSA | 2.81e-14 | 61 | 13 | 11 | See PrSM>> |
| 1400 | sp|B3EWT8|TXC2C\_CUPSA | 2.81e-14 | 61 | 12 | 11 | See PrSM>> |
| 927 | sp|B3EWT8|TXC2C\_CUPSA | 2.81e-14 | 61 | 12 | 11 | See PrSM>> |
| 1337 | sp|B3EWT8|TXC2C\_CUPSA | 2.81e-14 | 61 | 12 | 11 | See PrSM>> |
| 1417 | sp|B3EWT8|TXC2C\_CUPSA | 1.10e-13 | 61 | 14 | 12 | See PrSM>> |
| 1711 | sp|B3EWT8|TXC2C\_CUPSA | 3.10e-13 | 60 | 13 | 10 | See PrSM>> |
| 1433 | sp|B3EWT8|TXC2C\_CUPSA | 3.70e-13 | 61 | 13 | 10 | See PrSM>> |
| 1105 | sp|B3EWT8|TXC2C\_CUPSA | 3.70e-13 | 61 | 11 | 10 | See PrSM>> |
| 1056 | sp|B3EWT8|TXC2C\_CUPSA | 3.70e-13 | 61 | 11 | 10 | See PrSM>> |
| 1121 | sp|B3EWT8|TXC2C\_CUPSA | 3.70e-13 | 61 | 11 | 10 | See PrSM>> |
| 1129 | sp|B3EWT8|TXC2C\_CUPSA | 3.70e-13 | 61 | 11 | 10 | See PrSM>> |
| 1169 | sp|B3EWT8|TXC2C\_CUPSA | 3.70e-13 | 61 | 11 | 10 | See PrSM>> |
| 1361 | sp|B3EWT8|TXC2C\_CUPSA | 3.70e-13 | 61 | 11 | 10 | See PrSM>> |
| 1137 | sp|B3EWT8|TXC2C\_CUPSA | 3.76e-12 | 61 | 10 | 9 | See PrSM>> |
| 1144 | sp|B3EWT8|TXC2C\_CUPSA | 3.76e-12 | 61 | 10 | 9 | See PrSM>> |
| 1208 | sp|B3EWT8|TXC2C\_CUPSA | 3.83e-11 | 61 | 9 | 8 | See PrSM>> |
| 1488 | sp|B3EWT8|TXC2C\_CUPSA | 6.47e-10 | 33 | 9 | 7 | See PrSM>> |

All proteins /
sp|B3EWT8|TXC2C\_CUPSA Cupiennin-2c OS=Cupiennius salei OX=6928 PE=1 SV=1
