# Supplementary material for: The Dual Prey-Inactivation Strategy of Spiders—In-Depth Venomic Analysis of Cupiennius salei
Source: Toxins (Basel). 2019 Mar 19;11(3):167. doi: 10.3390/toxins11030167 (PMC6468893; doi:10.3390/toxins11030167)
Supplement: Supplementary file 1 [file toxins-11-00167-s001.zip › Supplementary Dataset EV1/20180328_f2_topdown_OTMS2_EThcD_NL_i02_ms2_proteoform_cutoff_html/proteoforms/proteoform37.html]

Proteoform #37 from sp|B3EWT9|TXC2D\_CUPSA Cupiennin-2d OS=Cupiennius salei OX=6928 PE=1 SV=1


All proteins /
sp|B3EWT9|TXC2D\_CUPSA Cupiennin-2d OS=Cupiennius salei OX=6928 PE=1 SV=1

## Proteoform #37

12 PrSMs for this proteoform

| Scan | Protein | E-value | # all peaks | # matched peaks | # matched fragment ions | Link |
| --- | --- | --- | --- | --- | --- | --- |
| 832 | sp|B3EWT9|TXC2D\_CUPSA | 6.06e-23 | 61 | 30 | 25 | See PrSM>> |
| 865 | sp|B3EWT9|TXC2D\_CUPSA | 3.12e-21 | 61 | 26 | 21 | See PrSM>> |
| 1712 | sp|B3EWT9|TXC2D\_CUPSA | 3.12e-21 | 61 | 25 | 21 | See PrSM>> |
| 823 | sp|B3EWT9|TXC2D\_CUPSA | 8.34e-21 | 61 | 22 | 20 | See PrSM>> |
| 845 | sp|B3EWT9|TXC2D\_CUPSA | 8.34e-21 | 61 | 24 | 20 | See PrSM>> |
| 861 | sp|B3EWT9|TXC2D\_CUPSA | 6.13e-20 | 61 | 23 | 19 | See PrSM>> |
| 839 | sp|B3EWT9|TXC2D\_CUPSA | 3.31e-18 | 61 | 19 | 17 | See PrSM>> |
| 869 | sp|B3EWT9|TXC2D\_CUPSA | 1.52e-15 | 61 | 17 | 14 | See PrSM>> |
| 824 | sp|B3EWT9|TXC2D\_CUPSA | 1.29e-14 | 61 | 14 | 13 | See PrSM>> |
| 1717 | sp|B3EWT9|TXC2D\_CUPSA | 9.36e-13 | 61 | 13 | 11 | See PrSM>> |
| 1725 | sp|B3EWT9|TXC2D\_CUPSA | 4.62e-12 | 45 | 11 | 11 | See PrSM>> |
| 1720 | sp|B3EWT9|TXC2D\_CUPSA | 8.35e-12 | 57 | 12 | 10 | See PrSM>> |

All proteins /
sp|B3EWT9|TXC2D\_CUPSA Cupiennin-2d OS=Cupiennius salei OX=6928 PE=1 SV=1
