# Supplementary material for: The Dual Prey-Inactivation Strategy of Spiders—In-Depth Venomic Analysis of Cupiennius salei
Source: Toxins (Basel). 2019 Mar 19;11(3):167. doi: 10.3390/toxins11030167 (PMC6468893; doi:10.3390/toxins11030167)
Supplement: Supplementary file 1 [file toxins-11-00167-s001.zip › Supplementary Dataset EV1/20180328_f2_topdown_OTMS2_EThcD_NL_i02_ms2_proteoform_cutoff_html/proteoforms/proteoform38.html]

Proteoform #38 from sp|B3EWT6|TXC2A\_CUPSA Cupiennin-2a OS=Cupiennius salei OX=6928 PE=1 SV=1


All proteins /
sp|B3EWT6|TXC2A\_CUPSA Cupiennin-2a OS=Cupiennius salei OX=6928 PE=1 SV=1

## Proteoform #38

5 PrSMs for this proteoform

| Scan | Protein | E-value | # all peaks | # matched peaks | # matched fragment ions | Link |
| --- | --- | --- | --- | --- | --- | --- |
| 815 | sp|B3EWT6|TXC2A\_CUPSA | 7.12e-23 | 60 | 32 | 25 | See PrSM>> |
| 825 | sp|B3EWT6|TXC2A\_CUPSA | 2.52e-21 | 60 | 28 | 21 | See PrSM>> |
| 808 | sp|B3EWT6|TXC2A\_CUPSA | 2.97e-19 | 60 | 24 | 18 | See PrSM>> |
| 821 | sp|B3EWT6|TXC2A\_CUPSA | 9.19e-16 | 60 | 17 | 14 | See PrSM>> |
| 829 | sp|B3EWT6|TXC2A\_CUPSA | 8.44e-15 | 60 | 17 | 13 | See PrSM>> |

All proteins /
sp|B3EWT6|TXC2A\_CUPSA Cupiennin-2a OS=Cupiennius salei OX=6928 PE=1 SV=1
