# Supplementary material for: The Dual Prey-Inactivation Strategy of Spiders—In-Depth Venomic Analysis of Cupiennius salei
Source: Toxins (Basel). 2019 Mar 19;11(3):167. doi: 10.3390/toxins11030167 (PMC6468893; doi:10.3390/toxins11030167)
Supplement: Supplementary file 1 [file toxins-11-00167-s001.zip › Supplementary Dataset EV1/20180328_f2_topdown_OTMS2_EThcD_NL_i02_ms2_proteoform_cutoff_html/proteoforms/proteoform4.html]

Proteoform #4 from CsTx-9a Cupiennius salei toxin 9 isoform a


All proteins /
CsTx-9a Cupiennius salei toxin 9 isoform a

## Proteoform #4

3 PrSMs for this proteoform

| Scan | Protein | E-value | # all peaks | # matched peaks | # matched fragment ions | Link |
| --- | --- | --- | --- | --- | --- | --- |
| 611 | CsTx-9a | 2.36e-46 | 130 | 68 | 54 | See PrSM>> |
| 613 | CsTx-9a | 7.48e-44 | 127 | 57 | 50 | See PrSM>> |
| 612 | CsTx-9a | 8.11e-41 | 130 | 58 | 45 | See PrSM>> |

All proteins /
CsTx-9a Cupiennius salei toxin 9 isoform a
