# Supplementary material for: The Dual Prey-Inactivation Strategy of Spiders—In-Depth Venomic Analysis of Cupiennius salei
Source: Toxins (Basel). 2019 Mar 19;11(3):167. doi: 10.3390/toxins11030167 (PMC6468893; doi:10.3390/toxins11030167)
Supplement: Supplementary file 1 [file toxins-11-00167-s001.zip › Supplementary Dataset EV1/20180328_f2_topdown_OTMS2_EThcD_NL_i02_ms2_proteoform_cutoff_html/proteoforms/proteoform40.html]

Proteoform #40 from CsTx-13a Cupiennius salei toxin 13 isoform a


All proteins /
CsTx-13a Cupiennius salei toxin 13 isoform a

## Proteoform #40

17 PrSMs for this proteoform

| Scan | Protein | E-value | # all peaks | # matched peaks | # matched fragment ions | Link |
| --- | --- | --- | --- | --- | --- | --- |
| 415 | CsTx-13a | 1.69e-22 | 57 | 35 | 30 | See PrSM>> |
| 407 | CsTx-13a | 5.33e-22 | 57 | 35 | 29 | See PrSM>> |
| 423 | CsTx-13a | 5.33e-22 | 57 | 35 | 29 | See PrSM>> |
| 472 | CsTx-13a | 5.33e-22 | 57 | 34 | 29 | See PrSM>> |
| 512 | CsTx-13a | 5.33e-22 | 57 | 34 | 29 | See PrSM>> |
| 308 | CsTx-13a | 1.68e-21 | 57 | 34 | 28 | See PrSM>> |
| 479 | CsTx-13a | 1.68e-21 | 57 | 33 | 28 | See PrSM>> |
| 431 | CsTx-13a | 5.31e-21 | 57 | 33 | 27 | See PrSM>> |
| 520 | CsTx-13a | 5.31e-21 | 57 | 31 | 27 | See PrSM>> |
| 463 | CsTx-13a | 5.31e-21 | 57 | 33 | 27 | See PrSM>> |
| 507 | CsTx-13a | 5.31e-21 | 57 | 32 | 27 | See PrSM>> |
| 439 | CsTx-13a | 1.68e-20 | 57 | 32 | 26 | See PrSM>> |
| 447 | CsTx-13a | 1.68e-20 | 57 | 32 | 26 | See PrSM>> |
| 400 | CsTx-13a | 5.30e-20 | 57 | 29 | 25 | See PrSM>> |
| 455 | CsTx-13a | 5.30e-20 | 57 | 31 | 25 | See PrSM>> |
| 529 | CsTx-13a | 3.18e-18 | 57 | 26 | 21 | See PrSM>> |
| 1744 | CsTx-13a | 1.64e-08 | 31 | 8 | 7 | See PrSM>> |

All proteins /
CsTx-13a Cupiennius salei toxin 13 isoform a
