# Supplementary material for: The Dual Prey-Inactivation Strategy of Spiders—In-Depth Venomic Analysis of Cupiennius salei
Source: Toxins (Basel). 2019 Mar 19;11(3):167. doi: 10.3390/toxins11030167 (PMC6468893; doi:10.3390/toxins11030167)
Supplement: Supplementary file 1 [file toxins-11-00167-s001.zip › Supplementary Dataset EV1/20180328_f2_topdown_OTMS2_EThcD_NL_i02_ms2_proteoform_cutoff_html/proteoforms/proteoform41.html]

Proteoform #41 from CsTx-13a Cupiennius salei toxin 13 isoform a


All proteins /
CsTx-13a Cupiennius salei toxin 13 isoform a

## Proteoform #41

7 PrSMs for this proteoform

| Scan | Protein | E-value | # all peaks | # matched peaks | # matched fragment ions | Link |
| --- | --- | --- | --- | --- | --- | --- |
| 375 | CsTx-13a | 2.06e-22 | 72 | 29 | 28 | See PrSM>> |
| 339 | CsTx-13a | 8.00e-22 | 72 | 27 | 27 | See PrSM>> |
| 347 | CsTx-13a | 3.11e-21 | 72 | 28 | 26 | See PrSM>> |
| 381 | CsTx-13a | 6.47e-20 | 72 | 25 | 24 | See PrSM>> |
| 340 | CsTx-13a | 5.29e-17 | 72 | 21 | 20 | See PrSM>> |
| 389 | CsTx-13a | 4.00e-16 | 72 | 19 | 19 | See PrSM>> |
| 393 | CsTx-13a | 1.59e-10 | 72 | 15 | 13 | See PrSM>> |

All proteins /
CsTx-13a Cupiennius salei toxin 13 isoform a
