# Supplementary material for: The Dual Prey-Inactivation Strategy of Spiders—In-Depth Venomic Analysis of Cupiennius salei
Source: Toxins (Basel). 2019 Mar 19;11(3):167. doi: 10.3390/toxins11030167 (PMC6468893; doi:10.3390/toxins11030167)
Supplement: Supplementary file 1 [file toxins-11-00167-s001.zip › Supplementary Dataset EV1/20180328_f2_topdown_OTMS2_EThcD_NL_i02_ms2_proteoform_cutoff_html/proteoforms/proteoform43.html]

Proteoform #43 from CsTx-9c\_S1 Cupiennius salei toxin 9 isoform c S1^ACsTx-9c\_S2 Cupiennius salei toxin 9 isoform c S2


All proteins /
CsTx-9c\_S1 Cupiennius salei toxin 9 isoform c S1^ACsTx-9c\_S2 Cupiennius salei toxin 9 isoform c S2

## Proteoform #43

1 PrSM for this proteoform

| Scan | Protein | E-value | # all peaks | # matched peaks | # matched fragment ions | Link |
| --- | --- | --- | --- | --- | --- | --- |
| 453 | CsTx-9c\_S1 | 3.40e-22 | 80 | 22 | 21 | See PrSM>> |

All proteins /
CsTx-9c\_S1 Cupiennius salei toxin 9 isoform c S1^ACsTx-9c\_S2 Cupiennius salei toxin 9 isoform c S2
