# Supplementary material for: The Dual Prey-Inactivation Strategy of Spiders—In-Depth Venomic Analysis of Cupiennius salei
Source: Toxins (Basel). 2019 Mar 19;11(3):167. doi: 10.3390/toxins11030167 (PMC6468893; doi:10.3390/toxins11030167)
Supplement: Supplementary file 1 [file toxins-11-00167-s001.zip › Supplementary Dataset EV1/20180328_f2_topdown_OTMS2_EThcD_NL_i02_ms2_proteoform_cutoff_html/proteoforms/proteoform47.html]

Proteoform #47 from CsTx-12b Cupiennius salei toxin 12 isoform b


All proteins /
CsTx-12b Cupiennius salei toxin 12 isoform b

## Proteoform #47

10 PrSMs for this proteoform

| Scan | Protein | E-value | # all peaks | # matched peaks | # matched fragment ions | Link |
| --- | --- | --- | --- | --- | --- | --- |
| 519 | CsTx-12b | 1.69e-21 | 57 | 33 | 28 | See PrSM>> |
| 511 | CsTx-12b | 5.33e-21 | 57 | 32 | 27 | See PrSM>> |
| 527 | CsTx-12b | 1.68e-20 | 57 | 28 | 26 | See PrSM>> |
| 533 | CsTx-12b | 1.68e-20 | 57 | 28 | 26 | See PrSM>> |
| 495 | CsTx-12b | 5.31e-20 | 57 | 29 | 25 | See PrSM>> |
| 503 | CsTx-12b | 5.31e-20 | 57 | 30 | 25 | See PrSM>> |
| 557 | CsTx-12b | 5.31e-20 | 57 | 27 | 25 | See PrSM>> |
| 629 | CsTx-12b | 5.31e-20 | 57 | 27 | 25 | See PrSM>> |
| 565 | CsTx-12b | 2.14e-19 | 57 | 18 | 18 | See PrSM>> |
| 487 | CsTx-12b | 2.85e-16 | 57 | 19 | 18 | See PrSM>> |

All proteins /
CsTx-12b Cupiennius salei toxin 12 isoform b
