# Supplementary material for: The Dual Prey-Inactivation Strategy of Spiders—In-Depth Venomic Analysis of Cupiennius salei
Source: Toxins (Basel). 2019 Mar 19;11(3):167. doi: 10.3390/toxins11030167 (PMC6468893; doi:10.3390/toxins11030167)
Supplement: Supplementary file 1 [file toxins-11-00167-s001.zip › Supplementary Dataset EV1/20180328_f2_topdown_OTMS2_EThcD_NL_i02_ms2_proteoform_cutoff_html/proteoforms/proteoform48.html]

Proteoform #48 from sp|B3EWT6|TXC2A\_CUPSA Cupiennin-2a OS=Cupiennius salei OX=6928 PE=1 SV=1


All proteins /
sp|B3EWT6|TXC2A\_CUPSA Cupiennin-2a OS=Cupiennius salei OX=6928 PE=1 SV=1

## Proteoform #48

20 PrSMs for this proteoform

| Scan | Protein | E-value | # all peaks | # matched peaks | # matched fragment ions | Link |
| --- | --- | --- | --- | --- | --- | --- |
| 1719 | sp|B3EWT6|TXC2A\_CUPSA | 2.49e-21 | 59 | 27 | 22 | See PrSM>> |
| 847 | sp|B3EWT6|TXC2A\_CUPSA | 3.12e-21 | 61 | 27 | 21 | See PrSM>> |
| 831 | sp|B3EWT6|TXC2A\_CUPSA | 3.12e-21 | 61 | 23 | 21 | See PrSM>> |
| 871 | sp|B3EWT6|TXC2A\_CUPSA | 8.34e-21 | 61 | 23 | 20 | See PrSM>> |
| 1225 | sp|B3EWT6|TXC2A\_CUPSA | 4.50e-19 | 61 | 21 | 18 | See PrSM>> |
| 881 | sp|B3EWT6|TXC2A\_CUPSA | 3.31e-18 | 61 | 20 | 17 | See PrSM>> |
| 1047 | sp|B3EWT6|TXC2A\_CUPSA | 3.31e-18 | 61 | 19 | 17 | See PrSM>> |
| 1072 | sp|B3EWT6|TXC2A\_CUPSA | 3.31e-18 | 61 | 18 | 17 | See PrSM>> |
| 1089 | sp|B3EWT6|TXC2A\_CUPSA | 2.43e-17 | 61 | 17 | 16 | See PrSM>> |
| 1264 | sp|B3EWT6|TXC2A\_CUPSA | 2.43e-17 | 61 | 18 | 16 | See PrSM>> |
| 1248 | sp|B3EWT6|TXC2A\_CUPSA | 2.43e-17 | 61 | 18 | 16 | See PrSM>> |
| 1240 | sp|B3EWT6|TXC2A\_CUPSA | 2.43e-17 | 61 | 18 | 16 | See PrSM>> |
| 1201 | sp|B3EWT6|TXC2A\_CUPSA | 1.78e-16 | 61 | 17 | 15 | See PrSM>> |
| 1185 | sp|B3EWT6|TXC2A\_CUPSA | 1.78e-16 | 61 | 17 | 15 | See PrSM>> |
| 841 | sp|B3EWT6|TXC2A\_CUPSA | 1.78e-16 | 61 | 16 | 15 | See PrSM>> |
| 1233 | sp|B3EWT6|TXC2A\_CUPSA | 1.52e-15 | 61 | 15 | 14 | See PrSM>> |
| 1192 | sp|B3EWT6|TXC2A\_CUPSA | 9.36e-13 | 61 | 12 | 11 | See PrSM>> |
| 1713 | sp|B3EWT6|TXC2A\_CUPSA | 9.36e-13 | 61 | 12 | 11 | See PrSM>> |
| 856 | sp|B3EWT6|TXC2A\_CUPSA | 9.36e-13 | 61 | 11 | 11 | See PrSM>> |
| 1729 | sp|B3EWT6|TXC2A\_CUPSA | 1.86e-06 | 24 | 6 | 6 | See PrSM>> |

All proteins /
sp|B3EWT6|TXC2A\_CUPSA Cupiennin-2a OS=Cupiennius salei OX=6928 PE=1 SV=1
