# Supplementary material for: The Dual Prey-Inactivation Strategy of Spiders—In-Depth Venomic Analysis of Cupiennius salei
Source: Toxins (Basel). 2019 Mar 19;11(3):167. doi: 10.3390/toxins11030167 (PMC6468893; doi:10.3390/toxins11030167)
Supplement: Supplementary file 1 [file toxins-11-00167-s001.zip › Supplementary Dataset EV1/20180328_f2_topdown_OTMS2_EThcD_NL_i02_ms2_proteoform_cutoff_html/proteoforms/proteoform51.html]

Proteoform #51 from sp|B3EWV7|TXC4B\_CUPSA Cupiennin-4b OS=Cupiennius salei OX=6928 PE=1 SV=1


All proteins /
sp|B3EWV7|TXC4B\_CUPSA Cupiennin-4b OS=Cupiennius salei OX=6928 PE=1 SV=1

## Proteoform #51

2 PrSMs for this proteoform

| Scan | Protein | E-value | # all peaks | # matched peaks | # matched fragment ions | Link |
| --- | --- | --- | --- | --- | --- | --- |
| 776 | sp|B3EWV7|TXC4B\_CUPSA | 3.95e-21 | 51 | 27 | 25 | See PrSM>> |
| 783 | sp|B3EWV7|TXC4B\_CUPSA | 1.29e-17 | 51 | 22 | 18 | See PrSM>> |

All proteins /
sp|B3EWV7|TXC4B\_CUPSA Cupiennin-4b OS=Cupiennius salei OX=6928 PE=1 SV=1
