# Supplementary material for: The Dual Prey-Inactivation Strategy of Spiders—In-Depth Venomic Analysis of Cupiennius salei
Source: Toxins (Basel). 2019 Mar 19;11(3):167. doi: 10.3390/toxins11030167 (PMC6468893; doi:10.3390/toxins11030167)
Supplement: Supplementary file 1 [file toxins-11-00167-s001.zip › Supplementary Dataset EV1/20180328_f2_topdown_OTMS2_EThcD_NL_i02_ms2_proteoform_cutoff_html/proteoforms/proteoform56.html]

Proteoform #56 from sp|B3EWT7|TXC2B\_CUPSA Cupiennin-2b OS=Cupiennius salei OX=6928 PE=1 SV=1


All proteins /
sp|B3EWT7|TXC2B\_CUPSA Cupiennin-2b OS=Cupiennius salei OX=6928 PE=1 SV=1

## Proteoform #56

16 PrSMs for this proteoform

| Scan | Protein | E-value | # all peaks | # matched peaks | # matched fragment ions | Link |
| --- | --- | --- | --- | --- | --- | --- |
| 1033 | sp|B3EWT7|TXC2B\_CUPSA | 8.78e-20 | 62 | 21 | 19 | See PrSM>> |
| 969 | sp|B3EWT7|TXC2B\_CUPSA | 6.81e-19 | 62 | 20 | 18 | See PrSM>> |
| 968 | sp|B3EWT7|TXC2B\_CUPSA | 5.29e-18 | 62 | 21 | 17 | See PrSM>> |
| 1040 | sp|B3EWT7|TXC2B\_CUPSA | 5.29e-18 | 62 | 21 | 17 | See PrSM>> |
| 975 | sp|B3EWT7|TXC2B\_CUPSA | 4.10e-17 | 62 | 19 | 16 | See PrSM>> |
| 1008 | sp|B3EWT7|TXC2B\_CUPSA | 4.10e-17 | 62 | 19 | 16 | See PrSM>> |
| 992 | sp|B3EWT7|TXC2B\_CUPSA | 3.18e-16 | 62 | 19 | 15 | See PrSM>> |
| 1015 | sp|B3EWT7|TXC2B\_CUPSA | 3.18e-16 | 62 | 19 | 15 | See PrSM>> |
| 1024 | sp|B3EWT7|TXC2B\_CUPSA | 3.18e-16 | 62 | 19 | 15 | See PrSM>> |
| 1031 | sp|B3EWT7|TXC2B\_CUPSA | 3.18e-16 | 62 | 18 | 15 | See PrSM>> |
| 1055 | sp|B3EWT7|TXC2B\_CUPSA | 3.18e-16 | 62 | 18 | 15 | See PrSM>> |
| 999 | sp|B3EWT7|TXC2B\_CUPSA | 2.51e-15 | 62 | 18 | 14 | See PrSM>> |
| 1048 | sp|B3EWT7|TXC2B\_CUPSA | 2.51e-15 | 62 | 18 | 14 | See PrSM>> |
| 1705 | sp|B3EWT7|TXC2B\_CUPSA | 2.08e-14 | 49 | 14 | 13 | See PrSM>> |
| 945 | sp|B3EWT7|TXC2B\_CUPSA | 1.56e-13 | 62 | 15 | 12 | See PrSM>> |
| 984 | sp|B3EWT7|TXC2B\_CUPSA | 1.56e-13 | 62 | 15 | 12 | See PrSM>> |

All proteins /
sp|B3EWT7|TXC2B\_CUPSA Cupiennin-2b OS=Cupiennius salei OX=6928 PE=1 SV=1
