# Supplementary material for: The Dual Prey-Inactivation Strategy of Spiders—In-Depth Venomic Analysis of Cupiennius salei
Source: Toxins (Basel). 2019 Mar 19;11(3):167. doi: 10.3390/toxins11030167 (PMC6468893; doi:10.3390/toxins11030167)
Supplement: Supplementary file 1 [file toxins-11-00167-s001.zip › Supplementary Dataset EV1/20180328_f2_topdown_OTMS2_EThcD_NL_i02_ms2_proteoform_cutoff_html/proteoforms/proteoform6.html]

Proteoform #6 from CsTx-1a\_S1 Cupiennius salei toxin 1 isoform a S1^ACsTx-1a\_S2 Cupiennius salei toxin 1 isoform a S2


All proteins /
CsTx-1a\_S1 Cupiennius salei toxin 1 isoform a S1^ACsTx-1a\_S2 Cupiennius salei toxin 1 isoform a S2

## Proteoform #6

65 PrSMs for this proteoform

| Scan | Protein | E-value | # all peaks | # matched peaks | # matched fragment ions | Link |
| --- | --- | --- | --- | --- | --- | --- |
| 663 | CsTx-1a\_S1 | 6.75e-44 | 146 | 73 | 55 | See PrSM>> |
| 616 | CsTx-1a\_S1 | 2.67e-43 | 146 | 77 | 54 | See PrSM>> |
| 603 | CsTx-1a\_S1 | 2.67e-43 | 146 | 73 | 54 | See PrSM>> |
| 648 | CsTx-1a\_S1 | 1.05e-42 | 146 | 71 | 53 | See PrSM>> |
| 595 | CsTx-1a\_S1 | 1.05e-42 | 146 | 75 | 53 | See PrSM>> |
| 664 | CsTx-1a\_S1 | 4.16e-42 | 146 | 69 | 52 | See PrSM>> |
| 653 | CsTx-1a\_S1 | 4.16e-42 | 146 | 72 | 52 | See PrSM>> |
| 639 | CsTx-1a\_S1 | 4.16e-42 | 146 | 74 | 52 | See PrSM>> |
| 585 | CsTx-1a\_S1 | 4.16e-42 | 146 | 61 | 52 | See PrSM>> |
| 564 | CsTx-1a\_S1 | 4.42e-42 | 146 | 57 | 46 | See PrSM>> |
| 633 | CsTx-1a\_S1 | 1.64e-41 | 146 | 67 | 51 | See PrSM>> |
| 647 | CsTx-1a\_S1 | 6.49e-41 | 146 | 67 | 50 | See PrSM>> |
| 632 | CsTx-1a\_S1 | 6.49e-41 | 146 | 71 | 50 | See PrSM>> |
| 625 | CsTx-1a\_S1 | 6.49e-41 | 146 | 62 | 50 | See PrSM>> |
| 624 | CsTx-1a\_S1 | 6.49e-41 | 146 | 70 | 50 | See PrSM>> |
| 623 | CsTx-1a\_S1 | 6.49e-41 | 146 | 63 | 50 | See PrSM>> |
| 615 | CsTx-1a\_S1 | 6.49e-41 | 146 | 68 | 50 | See PrSM>> |
| 640 | CsTx-1a\_S1 | 3.80e-40 | 146 | 65 | 49 | See PrSM>> |
| 580 | CsTx-1a\_S1 | 2.23e-39 | 146 | 64 | 48 | See PrSM>> |
| 649 | CsTx-1a\_S1 | 2.23e-39 | 146 | 65 | 48 | See PrSM>> |
| 588 | CsTx-1a\_S1 | 2.23e-39 | 146 | 66 | 48 | See PrSM>> |
| 593 | CsTx-1a\_S1 | 7.00e-39 | 146 | 48 | 43 | See PrSM>> |
| 605 | CsTx-1a\_S1 | 7.00e-39 | 146 | 59 | 43 | See PrSM>> |
| 701 | CsTx-1a\_S1 | 7.14e-39 | 146 | 54 | 43 | See PrSM>> |
| 576 | CsTx-1a\_S1 | 1.31e-38 | 146 | 64 | 47 | See PrSM>> |
| 597 | CsTx-1a\_S1 | 7.67e-38 | 146 | 61 | 46 | See PrSM>> |
| 563 | CsTx-1a\_S1 | 7.67e-38 | 146 | 65 | 46 | See PrSM>> |
| 575 | CsTx-1a\_S1 | 9.29e-38 | 146 | 58 | 42 | See PrSM>> |
| 577 | CsTx-1a\_S1 | 4.49e-37 | 146 | 59 | 45 | See PrSM>> |
| 579 | CsTx-1a\_S1 | 4.49e-37 | 146 | 58 | 45 | See PrSM>> |
| 652 | CsTx-1a\_S1 | 4.49e-37 | 146 | 63 | 45 | See PrSM>> |
| 567 | CsTx-1a\_S1 | 4.49e-37 | 146 | 57 | 45 | See PrSM>> |
| 600 | CsTx-1a\_S1 | 4.49e-37 | 146 | 59 | 45 | See PrSM>> |
| 617 | CsTx-1a\_S1 | 1.21e-36 | 146 | 54 | 41 | See PrSM>> |
| 583 | CsTx-1a\_S1 | 2.66e-36 | 146 | 62 | 44 | See PrSM>> |
| 627 | CsTx-1a\_S1 | 2.66e-36 | 146 | 58 | 44 | See PrSM>> |
| 620 | CsTx-1a\_S1 | 3.92e-36 | 142 | 46 | 39 | See PrSM>> |
| 571 | CsTx-1a\_S1 | 1.54e-35 | 146 | 52 | 40 | See PrSM>> |
| 671 | CsTx-1a\_S1 | 1.57e-35 | 146 | 58 | 40 | See PrSM>> |
| 584 | CsTx-1a\_S1 | 1.58e-35 | 146 | 58 | 43 | See PrSM>> |
| 596 | CsTx-1a\_S1 | 6.57e-35 | 146 | 56 | 39 | See PrSM>> |
| 572 | CsTx-1a\_S1 | 9.36e-35 | 146 | 53 | 42 | See PrSM>> |
| 619 | CsTx-1a\_S1 | 9.36e-35 | 146 | 59 | 42 | See PrSM>> |
| 587 | CsTx-1a\_S1 | 9.36e-35 | 146 | 56 | 42 | See PrSM>> |
| 599 | CsTx-1a\_S1 | 9.36e-35 | 146 | 59 | 42 | See PrSM>> |
| 589 | CsTx-1a\_S1 | 2.69e-34 | 146 | 50 | 38 | See PrSM>> |
| 592 | CsTx-1a\_S1 | 5.55e-34 | 146 | 54 | 41 | See PrSM>> |
| 601 | CsTx-1a\_S1 | 5.55e-34 | 146 | 56 | 41 | See PrSM>> |
| 581 | CsTx-1a\_S1 | 1.15e-33 | 146 | 49 | 37 | See PrSM>> |
| 643 | CsTx-1a\_S1 | 1.15e-33 | 146 | 50 | 37 | See PrSM>> |
| 636 | CsTx-1a\_S1 | 1.43e-33 | 145 | 49 | 40 | See PrSM>> |
| 609 | CsTx-1a\_S1 | 3.29e-33 | 146 | 53 | 40 | See PrSM>> |
| 791 | CsTx-1a\_S1 | 3.99e-33 | 141 | 46 | 40 | See PrSM>> |
| 591 | CsTx-1a\_S1 | 1.13e-32 | 146 | 53 | 39 | See PrSM>> |
| 608 | CsTx-1a\_S1 | 2.17e-32 | 142 | 48 | 37 | See PrSM>> |
| 628 | CsTx-1a\_S1 | 3.86e-32 | 146 | 49 | 38 | See PrSM>> |
| 573 | CsTx-1a\_S1 | 1.32e-31 | 146 | 51 | 37 | See PrSM>> |
| 644 | CsTx-1a\_S1 | 4.54e-31 | 146 | 44 | 36 | See PrSM>> |
| 672 | CsTx-1a\_S1 | 1.56e-30 | 146 | 44 | 35 | See PrSM>> |
| 725 | CsTx-1a\_S1 | 1.39e-28 | 146 | 42 | 33 | See PrSM>> |
| 621 | CsTx-1a\_S1 | 1.92e-24 | 102 | 31 | 27 | See PrSM>> |
| 637 | CsTx-1a\_S1 | 1.36e-22 | 146 | 40 | 29 | See PrSM>> |
| 1735 | CsTx-1a\_S1 | 1.71e-22 | 62 | 25 | 21 | See PrSM>> |
| 1736 | CsTx-1a\_S1 | 8.92e-14 | 45 | 17 | 17 | See PrSM>> |
| 668 | CsTx-1a\_S1 | 1.36e-11 | 146 | 21 | 17 | See PrSM>> |

All proteins /
CsTx-1a\_S1 Cupiennius salei toxin 1 isoform a S1^ACsTx-1a\_S2 Cupiennius salei toxin 1 isoform a S2
