# Supplementary material for: The Dual Prey-Inactivation Strategy of Spiders—In-Depth Venomic Analysis of Cupiennius salei
Source: Toxins (Basel). 2019 Mar 19;11(3):167. doi: 10.3390/toxins11030167 (PMC6468893; doi:10.3390/toxins11030167)
Supplement: Supplementary file 1 [file toxins-11-00167-s001.zip › Supplementary Dataset EV1/20180328_f2_topdown_OTMS2_EThcD_NL_i02_ms2_proteoform_cutoff_html/proteoforms/proteoform61.html]

Proteoform #61 from CsTx-12a\_S1 Cupiennius salei toxin 12 isoform a S1^ACsTx-12a\_S2 Cupiennius salei toxin 12 isoform a S2


All proteins /
CsTx-12a\_S1 Cupiennius salei toxin 12 isoform a S1^ACsTx-12a\_S2 Cupiennius salei toxin 12 isoform a S2

## Proteoform #61

7 PrSMs for this proteoform

| Scan | Protein | E-value | # all peaks | # matched peaks | # matched fragment ions | Link |
| --- | --- | --- | --- | --- | --- | --- |
| 369 | CsTx-12a\_S1 | 3.13e-19 | 73 | 23 | 23 | See PrSM>> |
| 287 | CsTx-12a\_S1 | 2.95e-15 | 73 | 21 | 18 | See PrSM>> |
| 279 | CsTx-12a\_S1 | 2.14e-14 | 73 | 18 | 17 | See PrSM>> |
| 295 | CsTx-12a\_S1 | 1.52e-12 | 71 | 16 | 15 | See PrSM>> |
| 283 | CsTx-12a\_S1 | 1.70e-10 | 73 | 16 | 13 | See PrSM>> |
| 327 | CsTx-12a\_S1 | 7.97e-10 | 61 | 13 | 12 | See PrSM>> |
| 336 | CsTx-12a\_S1 | 1.40e-08 | 68 | 11 | 11 | See PrSM>> |

All proteins /
CsTx-12a\_S1 Cupiennius salei toxin 12 isoform a S1^ACsTx-12a\_S2 Cupiennius salei toxin 12 isoform a S2
