# Supplementary material for: The Dual Prey-Inactivation Strategy of Spiders—In-Depth Venomic Analysis of Cupiennius salei
Source: Toxins (Basel). 2019 Mar 19;11(3):167. doi: 10.3390/toxins11030167 (PMC6468893; doi:10.3390/toxins11030167)
Supplement: Supplementary file 1 [file toxins-11-00167-s001.zip › Supplementary Dataset EV1/20180328_f2_topdown_OTMS2_EThcD_NL_i02_ms2_proteoform_cutoff_html/proteoforms/proteoform63.html]

Proteoform #63 from sp|B3EWT9|TXC2D\_CUPSA Cupiennin-2d OS=Cupiennius salei OX=6928 PE=1 SV=1


All proteins /
sp|B3EWT9|TXC2D\_CUPSA Cupiennin-2d OS=Cupiennius salei OX=6928 PE=1 SV=1

## Proteoform #63

5 PrSMs for this proteoform

| Scan | Protein | E-value | # all peaks | # matched peaks | # matched fragment ions | Link |
| --- | --- | --- | --- | --- | --- | --- |
| 849 | sp|B3EWT9|TXC2D\_CUPSA | 6.81e-19 | 62 | 18 | 18 | See PrSM>> |
| 853 | sp|B3EWT9|TXC2D\_CUPSA | 1.98e-14 | 62 | 16 | 13 | See PrSM>> |
| 805 | sp|B3EWT9|TXC2D\_CUPSA | 1.23e-12 | 62 | 12 | 11 | See PrSM>> |
| 809 | sp|B3EWT9|TXC2D\_CUPSA | 1.23e-12 | 62 | 15 | 11 | See PrSM>> |
| 804 | sp|B3EWT9|TXC2D\_CUPSA | 9.72e-12 | 62 | 11 | 10 | See PrSM>> |

All proteins /
sp|B3EWT9|TXC2D\_CUPSA Cupiennin-2d OS=Cupiennius salei OX=6928 PE=1 SV=1
