# Supplementary material for: The Dual Prey-Inactivation Strategy of Spiders—In-Depth Venomic Analysis of Cupiennius salei
Source: Toxins (Basel). 2019 Mar 19;11(3):167. doi: 10.3390/toxins11030167 (PMC6468893; doi:10.3390/toxins11030167)
Supplement: Supplementary file 1 [file toxins-11-00167-s001.zip › Supplementary Dataset EV1/20180328_f2_topdown_OTMS2_EThcD_NL_i02_ms2_proteoform_cutoff_html/proteoforms/proteoform72.html]

Proteoform #72 from CsTx-8a\_S1 Cupiennius salei toxin 8 isoform a S1^ACsTx-8a\_S2 Cupiennius salei toxin 8 isoform a S2


All proteins /
CsTx-8a\_S1 Cupiennius salei toxin 8 isoform a S1^ACsTx-8a\_S2 Cupiennius salei toxin 8 isoform a S2

## Proteoform #72

3 PrSMs for this proteoform

| Scan | Protein | E-value | # all peaks | # matched peaks | # matched fragment ions | Link |
| --- | --- | --- | --- | --- | --- | --- |
| 333 | CsTx-8a\_S1 | 5.59e-17 | 73 | 22 | 20 | See PrSM>> |
| 317 | CsTx-8a\_S1 | 1.56e-13 | 73 | 17 | 16 | See PrSM>> |
| 321 | CsTx-8a\_S1 | 2.09e-09 | 73 | 12 | 12 | See PrSM>> |

All proteins /
CsTx-8a\_S1 Cupiennius salei toxin 8 isoform a S1^ACsTx-8a\_S2 Cupiennius salei toxin 8 isoform a S2
