# Supplementary material for: The Dual Prey-Inactivation Strategy of Spiders—In-Depth Venomic Analysis of Cupiennius salei
Source: Toxins (Basel). 2019 Mar 19;11(3):167. doi: 10.3390/toxins11030167 (PMC6468893; doi:10.3390/toxins11030167)
Supplement: Supplementary file 1 [file toxins-11-00167-s001.zip › Supplementary Dataset EV1/20180328_f2_topdown_OTMS2_EThcD_NL_i02_ms2_proteoform_cutoff_html/proteoforms/proteoform8.html]

Proteoform #8 from CsTx-1a\_S1 Cupiennius salei toxin 1 isoform a S1^ACsTx-1a\_S2 Cupiennius salei toxin 1 isoform a S2


All proteins /
CsTx-1a\_S1 Cupiennius salei toxin 1 isoform a S1^ACsTx-1a\_S2 Cupiennius salei toxin 1 isoform a S2

## Proteoform #8

15 PrSMs for this proteoform

| Scan | Protein | E-value | # all peaks | # matched peaks | # matched fragment ions | Link |
| --- | --- | --- | --- | --- | --- | --- |
| 488 | CsTx-1a\_S1 | 1.90e-41 | 113 | 47 | 46 | See PrSM>> |
| 496 | CsTx-1a\_S1 | 7.86e-41 | 111 | 48 | 46 | See PrSM>> |
| 499 | CsTx-1a\_S1 | 1.87e-38 | 109 | 51 | 42 | See PrSM>> |
| 491 | CsTx-1b | 1.99e-36 | 115 | 48 | 40 | See PrSM>> |
| 492 | CsTx-1a\_S1 | 1.19e-33 | 115 | 42 | 36 | See PrSM>> |
| 489 | CsTx-1b | 1.73e-30 | 69 | 35 | 35 | See PrSM>> |
| 509 | CsTx-1a\_S1 | 4.70e-27 | 72 | 28 | 27 | See PrSM>> |
| 504 | CsTx-1a\_S1 | 1.27e-23 | 61 | 26 | 25 | See PrSM>> |
| 516 | CsTx-1a\_S1 | 1.03e-21 | 51 | 19 | 19 | See PrSM>> |
| 497 | CsTx-1a\_S1 | 4.15e-20 | 43 | 22 | 22 | See PrSM>> |
| 513 | CsTx-1a\_S1 | 1.23e-19 | 54 | 20 | 20 | See PrSM>> |
| 517 | CsTx-1a\_S1 | 1.91e-18 | 63 | 16 | 16 | See PrSM>> |
| 501 | CsTx-1a\_S1 | 1.53e-14 | 32 | 15 | 15 | See PrSM>> |
| 508 | CsTx-1a\_S1 | 3.30e-09 | 22 | 10 | 10 | See PrSM>> |
| 505 | CsTx-1a\_S1 | 7.27e-07 | 15 | 7 | 7 | See PrSM>> |

All proteins /
CsTx-1a\_S1 Cupiennius salei toxin 1 isoform a S1^ACsTx-1a\_S2 Cupiennius salei toxin 1 isoform a S2
