# Supplementary material for: The Dual Prey-Inactivation Strategy of Spiders—In-Depth Venomic Analysis of Cupiennius salei
Source: Toxins (Basel). 2019 Mar 19;11(3):167. doi: 10.3390/toxins11030167 (PMC6468893; doi:10.3390/toxins11030167)
Supplement: Supplementary file 1 [file toxins-11-00167-s001.zip › Supplementary Dataset EV1/20180328_f2_topdown_OTMS2_EThcD_NL_i02_ms2_proteoform_cutoff_html/proteoforms/proteoform88.html]

Proteoform #88 from CsTx-1b Cupiennius salei toxin 1 isoform b


All proteins /
CsTx-1b Cupiennius salei toxin 1 isoform b

## Proteoform #88

1 PrSM for this proteoform

| Scan | Protein | E-value | # all peaks | # matched peaks | # matched fragment ions | Link |
| --- | --- | --- | --- | --- | --- | --- |
| 459 | CsTx-1b | 1.10e-11 | 74 | 15 | 14 | See PrSM>> |

All proteins /
CsTx-1b Cupiennius salei toxin 1 isoform b
