# Supplementary material for: The Dual Prey-Inactivation Strategy of Spiders—In-Depth Venomic Analysis of Cupiennius salei
Source: Toxins (Basel). 2019 Mar 19;11(3):167. doi: 10.3390/toxins11030167 (PMC6468893; doi:10.3390/toxins11030167)
Supplement: Supplementary file 1 [file toxins-11-00167-s001.zip › Supplementary Dataset EV1/20180328_f2_topdown_OTMS2_EThcD_NL_i02_ms2_proteoform_cutoff_html/prsms/prsm0.html]

Protein-Spectrum-Match for Spectrum #201


All proteins /
CsTx-13a Cupiennius salei toxin 13 isoform a /
Proteoform #28

## Protein-Spectrum-Match #0 for Spectrum #201

|  |  |  |  |  |  |
| --- | --- | --- | --- | --- | --- |
| PrSM ID: | 0 | Scan(s): | 271 | Precursor charge: | 6 |
| Precursor m/z: | 524.6204 | Precursor mass: | 3141.6785 | Proteoform mass: | 3141.6791 |
| # matched peaks: | 29 | # matched fragment ions: | 26 | # unexpected modifications: | 0 |
| E-value: | 5.20e-25 | P-value: | 5.20e-25 | Q-value (Spectral FDR): | 0 |

  

|  |  |  |  |  |  |  |  |  |  |  |  |  |  |  |  |  |  |  |  |  |  |  |  |  |  |  |  |  |  |  |  |  |  |  |  |  |  |  |  |  |  |  |  |  |  |  |  |  |  |  |  |  |  |  |  |  |  |  |  |  |  |  |  |  |  |  |
| --- | --- | --- | --- | --- | --- | --- | --- | --- | --- | --- | --- | --- | --- | --- | --- | --- | --- | --- | --- | --- | --- | --- | --- | --- | --- | --- | --- | --- | --- | --- | --- | --- | --- | --- | --- | --- | --- | --- | --- | --- | --- | --- | --- | --- | --- | --- | --- | --- | --- | --- | --- | --- | --- | --- | --- | --- | --- | --- | --- | --- | --- | --- | --- | --- | --- | --- |
|  | | ... 30 amino acid residues are skipped at the N-terminus ... | | | | | | | | | | | | | | | | | | | | | | | | | | | | | | | | | | | | | | | | | | | | | | | | | | | | | | | | | | | | | |  | | |
|  | |  | | | | | | | | | | | | | | | | | | | | | | | | | | | | | | | | | | | | | | | | | | | | | | | | | | | | | | | | | | | | | | | | | | | |
| 31 |  |  | S |  | F |  | E |  | A |  | D |  | D |  | I |  | I |  | P |  | F |  |  | I |  | A |  | K |  | E |  | Q |  | V |  | R |  | S |  | D |  | C |  |  | T |  | L |  | R |  | N |  | H |  | D |  | C |  | T |  | D |  | D |  | 60 |  |
|  | |  | | | | | | | | | | | | | | | | | | | | | | | | | | | | | | | | | | | | | | | | | | | | | | | | | | | | | | | | | | | | | | | | | | | |
| 61 |  |  | R |  | H |  | S |  | C |  | C |  | R |  | S |  | K |  | M |  | F |  |  | K |  | D |  | V |  | C |  | T |  | C |  | F |  | Y |  | P |  | S |  |  | Q |  | R |  | S |  | E |  | T |  | A |  | R | ] | A | ⎩ | K | ⎩ | K |  | 90 |  |
|  | |  | | | | | | | | | | | | | | | | | | | | | | | | | | | | | | | | | | | | | | | | | | | | | | | | | | | | | | | | | | | | | | | | | | | |
| 91 |  |  | E | ⎱ | L | ⎫ | C |  | T | ⎫ | C | ⎫ | Q | ⎱ | Q |  | P | ⎫ | K | ⎱ | H |  | ⎫ | L |  | K | ⎩ | Y |  | I | ⎫ | E | ⎱ | K | ⎱ | G | ⎱ | L | ⎫ | Q | ⎫ | K |  | ⎫ | A | ⎫ | K | ⎫ | D | [ | Y |  | A |  | T |  | G |  | | 117 |  | | | | | |

Fixed PTMs: Carbamidomethylation [C93 C95 ]

  

All peaks (52)  Matched peaks (29)  Not matched peaks (23)

  

| Scan | Peak | Mono mass | Mono m/z | Intensity | Charge | Theoretical mass | Ion | Pos | Mass error | PPM error |
| --- | --- | --- | --- | --- | --- | --- | --- | --- | --- | --- |
| 271 | 1 | 3141.6606 | 524.6174 | 112398.58 | 6 |  |  |  |  |  |
| 271 | 2 | 3084.6366 | 617.9346 | 27920.87 | 5 |  |  |  |  |  |
| 271 | 3 | 3141.6576 | 629.3388 | 50738.55 | 5 |  |  |  |  |  |
| 271 | 4 | 3025.6486 | 606.1370 | 12708.47 | 5 | 3025.6680 | C25 | 25 | -0.0195 | -6.43 |
| 271 | 5 | 2007.1263 | 670.0494 | 10203.57 | 3 | 2007.1391 | Z\_DOT17 | 9 | -0.0128 | -6.40 |
| 271 | 6 | 3125.6388 | 626.1350 | 8447.40 | 5 |  |  |  |  |  |
| 271 | 7 | 2457.2818 | 615.3277 | 11501.42 | 4 | 2457.2984 | C20 | 20 | -0.0166 | -6.75 |
| 271 | 8 | 2143.1255 | 715.3824 | 11598.29 | 3 | 2143.1394 | C17 | 17 | -0.0139 | -6.48 |
| 271 | 9 | 2826.5174 | 707.6366 | 10882.13 | 4 | 2826.5360 | C23 | 23 | -0.0186 | -6.58 |
| 271 | 10 | 2897.5540 | 725.3958 | 8677.18 | 4 | 2897.5731 | C24 | 24 | -0.0191 | -6.58 |
| 271 | 11 | 2400.2603 | 601.0724 | 6799.82 | 4 | 2400.2769 | C19 | 19 | -0.0166 | -6.92 |
| 271 | 12 | 2272.1665 | 758.3961 | 5167.90 | 3 | 2272.1820 | C18 | 18 | -0.0154 | -6.80 |
| 271 | 13 | 2093.7699 | 524.4498 | 57712.04 | 4 |  |  |  |  |  |
| 271 | 14 | 1135.5408 | 568.7777 | 8627.82 | 2 | 1135.5477 | C9 | 9 | -6.94e-03 | -6.11 |
| 271 | 15 | 1488.7446 | 497.2555 | 6283.14 | 3 | 1488.7540 | C12 | 12 | -9.49e-03 | -6.37 |
| 271 | 16 | 3054.6040 | 764.6583 | 5961.59 | 4 | 3054.6233 | Z\_DOT25 | 1 | -0.0192 | -6.30 |
| 271 | 17 | 1625.8026 | 542.9415 | 6178.76 | 3 | 1625.8130 | C13 | 13 | -0.0104 | -6.39 |
| 271 | 18 | 2926.5079 | 732.6342 | 6731.91 | 4 | 2926.5283 | Z\_DOT24 | 2 | -0.0205 | -6.99 |
| 271 | 19 | 3084.6380 | 772.1668 | 7905.37 | 4 |  |  |  |  |  |
| 271 | 20 | 2698.4239 | 675.6132 | 7842.87 | 4 | 2698.4410 | C22 | 22 | -0.0171 | -6.35 |
| 271 | 21 | 2670.3806 | 891.1341 | 4353.85 | 3 |  |  |  |  |  |
| 271 | 22 | 628.7334 | 629.7407 | 20958.95 | 1 |  |  |  |  |  |
| 271 | 23 | 2626.3182 | 657.5868 | 4420.73 | 4 |  |  |  |  |  |
| 271 | 24 | 1653.9233 | 827.9689 | 5817.94 | 2 | 1653.9328 | Z\_DOT14 | 12 | -9.59e-03 | -5.80 |
| 271 | 25 | 2272.1658 | 569.0487 | 3993.39 | 4 | 2272.1820 | C18 | 18 | -0.0161 | -7.10 |
| 271 | 26 | 2570.3655 | 643.5987 | 4941.59 | 4 | 2570.3824 | C21 | 21 | -0.0169 | -6.58 |
| 271 | 27 | 2400.2618 | 801.0945 | 3179.08 | 3 | 2400.2769 | C19 | 19 | -0.0151 | -6.31 |
| 271 | 28 | 1571.8350 | 786.9248 | 7890.04 | 2 |  |  |  |  |  |
| 271 | 29 | 2204.2103 | 552.0599 | 7345.12 | 4 |  |  |  |  |  |
| 271 | 30 | 2669.3724 | 668.3504 | 3479.33 | 4 | 2669.3908 | Z\_DOT22 | 4 | -0.0184 | -6.89 |
| 271 | 31 | 1291.7054 | 646.8600 | 3230.09 | 2 |  |  |  |  |  |
| 271 | 32 | 1275.6868 | 638.8507 | 4437.29 | 2 | 1275.6949 | Z\_DOT11 | 15 | -8.11e-03 | -6.36 |
| 271 | 33 | 473.2937 | 474.3010 | 3899.10 | 1 | 473.2961 | C4 | 4 | -2.40e-03 | -5.07 |
| 271 | 34 | 847.4534 | 848.4607 | 3031.00 | 1 | 847.4585 | C7 | 7 | -5.10e-03 | -6.01 |
| 271 | 35 | 1360.6492 | 681.3319 | 2101.74 | 2 | 1360.6591 | C11 | 11 | -9.88e-03 | -7.26 |
| 271 | 36 | 870.4993 | 871.5066 | 2151.69 | 1 | 870.5049 | Z\_DOT8 | 18 | -5.61e-03 | -6.45 |
| 271 | 37 | 685.3843 | 686.3915 | 2646.57 | 1 | 685.3885 | Z\_DOT6 | 20 | -4.25e-03 | -6.19 |
| 271 | 38 | 490.0419 | 491.0492 | 2734.70 | 1 |  |  |  |  |  |
| 271 | 39 | 1007.4830 | 504.7488 | 2775.07 | 2 | 1007.4892 | C8 | 8 | -6.16e-03 | -6.12 |
| 271 | 40 | 742.4059 | 743.4132 | 2553.00 | 1 | 742.4100 | Z\_DOT7 | 19 | -4.04e-03 | -5.44 |
| 271 | 41 | 282.0396 | 283.0469 | 1808.22 | 1 |  |  |  |  |  |
| 271 | 42 | 1135.5415 | 1136.5488 | 2046.92 | 1 | 1135.5477 | C9 | 9 | -6.26e-03 | -5.51 |
| 271 | 43 | 1484.3138 | 743.1642 | 1267.94 | 2 |  |  |  |  |  |
| 271 | 44 | 586.3770 | 587.3843 | 3013.78 | 1 | 586.3802 | C5 | 5 | -3.18e-03 | -5.42 |
| 271 | 45 | 300.0501 | 301.0574 | 1532.14 | 1 |  |  |  |  |  |
| 271 | 46 | 506.0736 | 507.0809 | 1485.72 | 1 |  |  |  |  |  |
| 271 | 47 | 940.5289 | 471.2717 | 1779.69 | 2 |  |  |  |  |  |
| 271 | 48 | 1103.6100 | 552.8123 | 995.35 | 2 |  |  |  |  |  |
| 271 | 49 | 758.4248 | 380.2197 | 924.01 | 2 |  |  |  |  |  |
| 271 | 50 | 1153.1309 | 577.5727 | 584.33 | 2 |  |  |  |  |  |
| 271 | 51 | 933.1411 | 934.1484 | 655.32 | 1 |  |  |  |  |  |
| 271 | 52 | 1048.2262 | 1049.2335 | 764.67 | 1 |  |  |  |  |  |

  

All proteins /
CsTx-13a Cupiennius salei toxin 13 isoform a /
Proteoform #28
