# Supplementary material for: The Dual Prey-Inactivation Strategy of Spiders—In-Depth Venomic Analysis of Cupiennius salei
Source: Toxins (Basel). 2019 Mar 19;11(3):167. doi: 10.3390/toxins11030167 (PMC6468893; doi:10.3390/toxins11030167)
Supplement: Supplementary file 1 [file toxins-11-00167-s001.zip › Supplementary Dataset EV1/20180328_f2_topdown_OTMS2_EThcD_NL_i02_ms2_proteoform_cutoff_html/prsms/prsm1.html]

Protein-Spectrum-Match for Spectrum #207


All proteins /
CsTx-12a\_S1 Cupiennius salei toxin 12 isoform a S1^ACsTx-12a\_S2 Cupiennius salei toxin 12 isoform a S2 /
Proteoform #61

## Protein-Spectrum-Match #1 for Spectrum #207

|  |  |  |  |  |  |
| --- | --- | --- | --- | --- | --- |
| PrSM ID: | 1 | Scan(s): | 279 | Precursor charge: | 6 |
| Precursor m/z: | 738.8122 | Precursor mass: | 4426.8293 | Proteoform mass: | 4426.9143 |
| # matched peaks: | 18 | # matched fragment ions: | 17 | # unexpected modifications: | 1 |
| E-value: | 2.14e-14 | P-value: | 2.14e-14 | Q-value (Spectral FDR): | 0 |

  

|  |  |  |  |  |  |  |  |  |  |  |  |  |  |  |  |  |  |  |  |  |  |  |  |  |  |  |  |  |  |  |  |  |  |  |  |  |  |  |  |  |  |  |  |  |  |  |  |  |  |  |  |  |  |  |  |  |  |  |  |  |  |  |  |  |  |  |  |  |  |
| --- | --- | --- | --- | --- | --- | --- | --- | --- | --- | --- | --- | --- | --- | --- | --- | --- | --- | --- | --- | --- | --- | --- | --- | --- | --- | --- | --- | --- | --- | --- | --- | --- | --- | --- | --- | --- | --- | --- | --- | --- | --- | --- | --- | --- | --- | --- | --- | --- | --- | --- | --- | --- | --- | --- | --- | --- | --- | --- | --- | --- | --- | --- | --- | --- | --- | --- | --- | --- | --- |
|  | |  | | | | | | | | | | | | | | | | | | | | | | | | | | | | | | | | | | | | | | | | | | | | | | | | | | | | | | | | | | | | | | | | | | | |
| 1 |  |  | M |  | K |  | V |  | L |  | V |  | I |  | C |  | A |  | V |  | L |  |  | F |  | L |  | T |  | I |  | F |  | S |  | N |  | S |  | S |  | A |  |  | E |  | T |  | E |  | D |  | D |  | F |  | L |  | E |  | D |  | E |  | 30 |  |
|  | |  | | | | | | | | | | | | | | | | | | | | | | | | | | | | | | | | | | | | | | | | | | | | | | | | | | | | | | | | | | | | | | | | | | | |
| 31 |  |  | S |  | F |  | E |  | A |  | D |  | D |  | V |  | I |  | P |  | F |  |  | L |  | A |  | R |  | E |  | Q |  | V |  | R | ] | S |  | D |  | C |  |  | T | ⎫ | L | ⎫ | R | ⎫ | N |  | H | ⎫ | D | ⎫ | C | ⎫ | T | ⎫ | D | ⎫ | D |  | 60 |  |
|  | |  | | | | | | | | | | | | | | | | | 57.11 | | | | | | | | | | | | | | | | | | | | | | | | | | | | | | | | | | | | | | | | | | | | | | | |
| 61 |  | ⎫ | R |  | H |  | S | ⎫ | C | ⎫ | C |  | R | ⎫ | S |  | K | ⎫ | M | ⎩ | F |  |  | K | ⎩ | D | ⎩ | V |  | C | ⎩ | K |  | C |  | F |  | Y |  | P |  | S |  |  | Q | [ | R |  | S |  | D |  | T |  | A |  | R |  | A |  | K |  | K |  | 90 |  |
|  | |  | | | | | | | | | | | | | | | | | | | | | | | | | | | | | | | | | | | | | | | | | | | | | | | | | | | | | | | | | | | | | | | | | | | |
| 91 |  |  | E |  | L |  | C |  | T |  | C |  | Q |  | Q |  | D |  | K |  | H |  |  | L |  | K |  | F |  | I |  | E |  | K |  | G |  | L |  | Q |  | K |  |  | A |  | K |  | V |  | L |  | V |  | A |  | G |  | | 117 |  | | | | | |

Fixed PTMs: Carbamidomethylation [C50 C57 C64 C65 C74 C76 ]   
  
     Unexpected modifications:   Unknown [57.11]

  

All peaks (73)  Matched peaks (18)  Not matched peaks (55)

  

| Scan | Peak | Mono mass | Mono m/z | Intensity | Charge | Theoretical mass | Ion | Pos | Mass error | PPM error |
| --- | --- | --- | --- | --- | --- | --- | --- | --- | --- | --- |
| 279 | 1 | 4321.7983 | 721.3070 | 4622669.90 | 6 |  |  |  |  |  |
| 279 | 2 | 4264.7661 | 853.9605 | 891272.36 | 5 |  |  |  |  |  |
| 279 | 3 | 4369.7881 | 874.9649 | 712900.19 | 5 |  |  |  |  |  |
| 279 | 4 | 4322.7886 | 865.5650 | 658199.34 | 5 |  |  |  |  |  |
| 279 | 5 | 3538.4970 | 885.6315 | 115298.67 | 4 |  |  |  |  |  |
| 279 | 6 | 4412.7924 | 883.5658 | 89323.58 | 5 |  |  |  |  |  |
| 279 | 7 | 4013.7078 | 803.7488 | 96810.68 | 5 |  |  |  |  |  |
| 279 | 8 | 3845.5965 | 962.4064 | 85850.85 | 4 |  |  |  |  |  |
| 279 | 9 | 3036.2803 | 760.0774 | 95968.42 | 4 |  |  |  |  |  |
| 279 | 10 | 2847.2420 | 712.8178 | 95252.39 | 4 |  |  |  |  |  |
| 279 | 11 | 4305.7708 | 862.1614 | 83414.09 | 5 |  |  |  |  |  |
| 279 | 12 | 2213.9038 | 738.9752 | 169485.17 | 3 |  |  |  |  |  |
| 279 | 13 | 4192.7440 | 839.5561 | 80427.14 | 5 |  |  |  |  |  |
| 279 | 14 | 2761.1187 | 921.3802 | 88128.35 | 3 |  |  |  |  |  |
| 279 | 15 | 4232.7947 | 847.5662 | 62754.86 | 5 |  |  |  |  |  |
| 279 | 16 | 4303.7870 | 718.3051 | 82780.07 | 6 |  |  |  |  |  |
| 279 | 17 | 3991.6323 | 799.3337 | 49639.37 | 5 |  |  |  |  |  |
| 279 | 18 | 3698.5291 | 925.6396 | 60319.68 | 4 |  |  |  |  |  |
| 279 | 19 | 4278.7716 | 856.7616 | 51664.34 | 5 |  |  |  |  |  |
| 279 | 20 | 1491.5775 | 746.7961 | 77530.30 | 2 | 1491.5830 | C12 | 12 | -5.48e-03 | -3.67 |
| 279 | 21 | 3151.3072 | 788.8341 | 47066.47 | 4 |  |  |  |  |  |
| 279 | 22 | 3250.3753 | 813.6011 | 49004.70 | 4 |  |  |  |  |  |
| 279 | 23 | 4175.7183 | 836.1509 | 63777.24 | 5 |  |  |  |  |  |
| 279 | 24 | 4215.7644 | 844.1602 | 47977.90 | 5 |  |  |  |  |  |
| 279 | 25 | 4118.7307 | 824.7534 | 38799.65 | 5 |  |  |  |  |  |
| 279 | 26 | 4264.7661 | 1067.1988 | 35151.37 | 4 |  |  |  |  |  |
| 279 | 27 | 4013.7105 | 1004.4349 | 29109.28 | 4 |  |  |  |  |  |
| 279 | 28 | 3117.3186 | 780.3369 | 30213.55 | 4 |  |  |  |  |  |
| 279 | 29 | 3573.4474 | 894.3691 | 32746.45 | 4 |  |  |  |  |  |
| 279 | 30 | 4409.7814 | 735.9708 | 30471.75 | 6 |  |  |  |  |  |
| 279 | 31 | 2831.2186 | 944.7468 | 36329.98 | 3 |  |  |  |  |  |
| 279 | 32 | 2678.0804 | 893.7007 | 39281.13 | 3 | 2678.0914 | C21 | 21 | -0.0110 | -4.12 |
| 279 | 33 | 2423.0319 | 808.6846 | 27363.85 | 3 |  |  |  |  |  |
| 279 | 34 | 4323.8033 | 1081.9581 | 26770.82 | 4 |  |  |  |  |  |
| 279 | 35 | 1986.7955 | 994.4050 | 31531.88 | 2 | 1986.8020 | C16 | 16 | -6.53e-03 | -3.29 |
| 279 | 36 | 4105.7094 | 822.1492 | 24935.01 | 5 |  |  |  |  |  |
| 279 | 37 | 4160.7076 | 833.1488 | 25556.95 | 5 |  |  |  |  |  |
| 279 | 38 | 2085.9695 | 696.3304 | 29661.19 | 3 |  |  |  |  |  |
| 279 | 39 | 2716.1907 | 906.4042 | 22978.48 | 3 |  |  |  |  |  |
| 279 | 40 | 2245.9980 | 749.6733 | 43685.08 | 3 |  |  |  |  |  |
| 279 | 41 | 3729.5494 | 933.3946 | 22570.59 | 4 |  |  |  |  |  |
| 279 | 42 | 1606.6048 | 804.3097 | 38323.33 | 2 | 1606.6100 | C13 | 13 | -5.20e-03 | -3.24 |
| 279 | 43 | 3858.6507 | 965.6700 | 20239.90 | 4 |  |  |  |  |  |
| 279 | 44 | 1561.6763 | 781.8454 | 23100.02 | 2 | 1561.6820 | Z\_DOT12 | 22 | -5.69e-03 | -3.65 |
| 279 | 45 | 3410.4126 | 853.6104 | 69797.25 | 4 |  |  |  |  |  |
| 279 | 46 | 2462.9560 | 821.9926 | 17364.80 | 3 | 2462.9644 | C19 | 19 | -8.44e-03 | -3.43 |
| 279 | 47 | 721.2873 | 722.2946 | 172434.72 | 1 |  |  |  |  |  |
| 279 | 48 | 1376.5511 | 689.2828 | 20257.09 | 2 | 1376.5561 | C11 | 11 | -5.03e-03 | -3.65 |
| 279 | 49 | 1644.7132 | 823.3639 | 22190.63 | 2 |  |  |  |  |  |
| 279 | 50 | 2146.8261 | 1074.4203 | 15984.50 | 2 | 2146.8327 | C17 | 17 | -6.55e-03 | -3.05 |
| 279 | 51 | 4370.7934 | 1093.7056 | 16593.57 | 4 |  |  |  |  |  |
| 279 | 52 | 749.3469 | 750.3542 | 18225.30 | 1 | 749.3490 | C6 | 6 | -2.06e-03 | -2.75 |
| 279 | 53 | 885.5630 | 886.5703 | 21563.72 | 1 |  |  |  |  |  |
| 279 | 54 | 1275.5048 | 638.7597 | 10280.09 | 2 | 1275.5084 | C10 | 10 | -3.61e-03 | -2.83 |
| 279 | 55 | 694.2946 | 695.3019 | 9569.59 | 1 |  |  |  |  |  |
| 279 | 56 | 912.3899 | 913.3972 | 11051.23 | 1 | 912.3926 | Z\_DOT7 | 27 | -2.74e-03 | -3.01 |
| 279 | 57 | 822.3889 | 823.3962 | 6307.87 | 1 |  |  |  |  |  |
| 279 | 58 | 330.1533 | 331.1606 | 14247.76 | 1 |  |  |  |  |  |
| 279 | 59 | 1286.5148 | 1287.5220 | 4466.18 | 1 | 1286.5186 | Z\_DOT10 | 24 | -3.87e-03 | -3.01 |
| 279 | 60 | 738.1321 | 739.1394 | 93698.34 | 1 |  |  |  |  |  |
| 279 | 61 | 1115.4742 | 558.7444 | 8233.43 | 2 | 1115.4778 | C9 | 9 | -3.60e-03 | -3.23 |
| 279 | 62 | 1115.4744 | 1116.4816 | 5968.72 | 1 | 1115.4778 | C9 | 9 | -3.40e-03 | -3.05 |
| 279 | 63 | 1171.4890 | 1172.4963 | 3346.08 | 1 | 1171.4917 | Z\_DOT9 | 25 | -2.69e-03 | -2.29 |
| 279 | 64 | 1000.4474 | 501.2310 | 4836.25 | 2 | 1000.4508 | C8 | 8 | -3.39e-03 | -3.39 |
| 279 | 65 | 493.2160 | 494.2233 | 4660.85 | 1 |  |  |  |  |  |
| 279 | 66 | 593.2463 | 594.2535 | 6024.94 | 1 | 593.2479 | C5 | 5 | -1.60e-03 | -2.70 |
| 279 | 67 | 361.1265 | 362.1338 | 3529.50 | 1 |  |  |  |  |  |
| 279 | 68 | 1324.6051 | 663.3098 | 3495.37 | 2 |  |  |  |  |  |
| 279 | 69 | 480.1626 | 481.1699 | 3783.85 | 1 | 480.1638 | C4 | 4 | -1.19e-03 | -2.48 |
| 279 | 70 | 576.2200 | 577.2272 | 3344.26 | 1 |  |  |  |  |  |
| 279 | 71 | 1098.4471 | 550.2308 | 1730.19 | 2 |  |  |  |  |  |
| 279 | 72 | 1243.5239 | 1244.5312 | 1810.86 | 1 |  |  |  |  |  |
| 279 | 73 | 1098.4496 | 1099.4568 | 1672.79 | 1 |  |  |  |  |  |

  

All proteins /
CsTx-12a\_S1 Cupiennius salei toxin 12 isoform a S1^ACsTx-12a\_S2 Cupiennius salei toxin 12 isoform a S2 /
Proteoform #61
