# Supplementary material for: The Dual Prey-Inactivation Strategy of Spiders—In-Depth Venomic Analysis of Cupiennius salei
Source: Toxins (Basel). 2019 Mar 19;11(3):167. doi: 10.3390/toxins11030167 (PMC6468893; doi:10.3390/toxins11030167)
Supplement: Supplementary file 1 [file toxins-11-00167-s001.zip › Supplementary Dataset EV1/20180328_f2_topdown_OTMS2_EThcD_NL_i02_ms2_proteoform_cutoff_html/prsms/prsm10.html]

Protein-Spectrum-Match for Spectrum #219


All proteins /
CsTx-12a\_S1 Cupiennius salei toxin 12 isoform a S1^ACsTx-12a\_S2 Cupiennius salei toxin 12 isoform a S2 /
Proteoform #61

## Protein-Spectrum-Match #10 for Spectrum #219

|  |  |  |  |  |  |
| --- | --- | --- | --- | --- | --- |
| PrSM ID: | 10 | Scan(s): | 295 | Precursor charge: | 6 |
| Precursor m/z: | 738.8117 | Precursor mass: | 4426.8263 | Proteoform mass: | 4426.9113 |
| # matched peaks: | 16 | # matched fragment ions: | 15 | # unexpected modifications: | 1 |
| E-value: | 1.52e-12 | P-value: | 1.52e-12 | Q-value (Spectral FDR): | 0 |

  

|  |  |  |  |  |  |  |  |  |  |  |  |  |  |  |  |  |  |  |  |  |  |  |  |  |  |  |  |  |  |  |  |  |  |  |  |  |  |  |  |  |  |  |  |  |  |  |  |  |  |  |  |  |  |  |  |  |  |  |  |  |  |  |  |  |  |  |  |  |  |
| --- | --- | --- | --- | --- | --- | --- | --- | --- | --- | --- | --- | --- | --- | --- | --- | --- | --- | --- | --- | --- | --- | --- | --- | --- | --- | --- | --- | --- | --- | --- | --- | --- | --- | --- | --- | --- | --- | --- | --- | --- | --- | --- | --- | --- | --- | --- | --- | --- | --- | --- | --- | --- | --- | --- | --- | --- | --- | --- | --- | --- | --- | --- | --- | --- | --- | --- | --- | --- | --- |
|  | |  | | | | | | | | | | | | | | | | | | | | | | | | | | | | | | | | | | | | | | | | | | | | | | | | | | | | | | | | | | | | | | | | | | | |
| 1 |  |  | M |  | K |  | V |  | L |  | V |  | I |  | C |  | A |  | V |  | L |  |  | F |  | L |  | T |  | I |  | F |  | S |  | N |  | S |  | S |  | A |  |  | E |  | T |  | E |  | D |  | D |  | F |  | L |  | E |  | D |  | E |  | 30 |  |
|  | |  | | | | | | | | | | | | | | | | | | | | | | | | | | | | | | | | | | | | | | | | | | | | | | | | | | | | | | | | | | | | | | | | | | | |
| 31 |  |  | S |  | F |  | E |  | A |  | D |  | D |  | V |  | I |  | P |  | F |  |  | L |  | A |  | R |  | E |  | Q |  | V |  | R | ] | S |  | D |  | C |  |  | T |  | L | ⎫ | R | ⎫ | N | ⎫ | H | ⎫ | D | ⎫ | C | ⎫ | T | ⎫ | D | ⎫ | D |  | 60 |  |
|  | |  | | | | | | | | | | | | | | | | | 57.11 | | | | | | | | | | | | | | | | | | | | | | | | | | | | | | | | | | | | | | | | | | | | | | | |
| 61 |  | ⎫ | R |  | H | ⎫ | S | ⎫ | C | ⎫ | C |  | R | ⎫ | S |  | K | ⎫ | M |  | F |  |  | K |  | D |  | V |  | C | ⎩ | K |  | C |  | F |  | Y |  | P |  | S |  |  | Q | [ | R |  | S |  | D |  | T |  | A |  | R |  | A |  | K |  | K |  | 90 |  |
|  | |  | | | | | | | | | | | | | | | | | | | | | | | | | | | | | | | | | | | | | | | | | | | | | | | | | | | | | | | | | | | | | | | | | | | |
| 91 |  |  | E |  | L |  | C |  | T |  | C |  | Q |  | Q |  | D |  | K |  | H |  |  | L |  | K |  | F |  | I |  | E |  | K |  | G |  | L |  | Q |  | K |  |  | A |  | K |  | V |  | L |  | V |  | A |  | G |  | | 117 |  | | | | | |

Fixed PTMs: Carbamidomethylation [C50 C57 C64 C65 C74 C76 ]   
  
     Unexpected modifications:   Unknown [57.11]

  

All peaks (71)  Matched peaks (16)  Not matched peaks (55)

  

| Scan | Peak | Mono mass | Mono m/z | Intensity | Charge | Theoretical mass | Ion | Pos | Mass error | PPM error |
| --- | --- | --- | --- | --- | --- | --- | --- | --- | --- | --- |
| 295 | 1 | 4321.7764 | 721.3033 | 383395.17 | 6 |  |  |  |  |  |
| 295 | 2 | 4264.7518 | 853.9576 | 96357.67 | 5 |  |  |  |  |  |
| 295 | 3 | 4369.7771 | 874.9627 | 95583.45 | 5 |  |  |  |  |  |
| 295 | 4 | 4322.7788 | 865.5630 | 71871.49 | 5 |  |  |  |  |  |
| 295 | 5 | 4323.7891 | 1081.9545 | 16534.37 | 4 |  |  |  |  |  |
| 295 | 6 | 4264.7539 | 1067.1958 | 20177.83 | 4 |  |  |  |  |  |
| 295 | 7 | 2761.1110 | 921.3776 | 15764.49 | 3 |  |  |  |  |  |
| 295 | 8 | 4412.7829 | 883.5639 | 11281.08 | 5 |  |  |  |  |  |
| 295 | 9 | 3538.4887 | 885.6294 | 14179.10 | 4 |  |  |  |  |  |
| 295 | 10 | 4305.7558 | 862.1584 | 10427.49 | 5 |  |  |  |  |  |
| 295 | 11 | 3698.5186 | 925.6369 | 9020.27 | 4 |  |  |  |  |  |
| 295 | 12 | 1491.5743 | 746.7944 | 14201.34 | 2 | 1491.5830 | C12 | 12 | -8.77e-03 | -5.88 |
| 295 | 13 | 3845.5848 | 962.4035 | 12149.17 | 4 |  |  |  |  |  |
| 295 | 14 | 4278.7649 | 714.1348 | 10884.65 | 6 |  |  |  |  |  |
| 295 | 15 | 4370.7837 | 1093.7032 | 11029.97 | 4 |  |  |  |  |  |
| 295 | 16 | 4192.7334 | 839.5539 | 10221.97 | 5 |  |  |  |  |  |
| 295 | 17 | 3036.2723 | 760.0753 | 10800.39 | 4 |  |  |  |  |  |
| 295 | 18 | 4013.6953 | 803.7463 | 9985.37 | 5 |  |  |  |  |  |
| 295 | 19 | 4417.7571 | 737.3001 | 8223.78 | 6 |  |  |  |  |  |
| 295 | 20 | 4207.7364 | 1052.9414 | 6650.13 | 4 |  |  |  |  |  |
| 295 | 21 | 4278.7679 | 856.7609 | 7449.72 | 5 |  |  |  |  |  |
| 295 | 22 | 2831.2108 | 944.7442 | 6578.12 | 3 |  |  |  |  |  |
| 295 | 23 | 4014.7003 | 1004.6823 | 6296.27 | 4 |  |  |  |  |  |
| 295 | 24 | 1986.7906 | 994.4026 | 7944.25 | 2 | 1986.8020 | C16 | 16 | -0.0114 | -5.75 |
| 295 | 25 | 3151.2991 | 788.8321 | 6257.14 | 4 |  |  |  |  |  |
| 295 | 26 | 2847.2291 | 712.8145 | 5710.02 | 4 |  |  |  |  |  |
| 295 | 27 | 4294.7167 | 716.7934 | 7257.49 | 6 |  |  |  |  |  |
| 295 | 28 | 4219.7403 | 1055.9423 | 5258.15 | 4 |  |  |  |  |  |
| 295 | 29 | 1899.7562 | 950.8854 | 7517.48 | 2 | 1899.7700 | C15 | 15 | -0.0138 | -7.27 |
| 295 | 30 | 3250.3689 | 813.5995 | 6016.08 | 4 |  |  |  |  |  |
| 295 | 31 | 1474.5476 | 738.2811 | 7628.26 | 2 |  |  |  |  |  |
| 295 | 32 | 2213.8996 | 738.9738 | 11406.61 | 3 |  |  |  |  |  |
| 295 | 33 | 3573.4374 | 894.3666 | 5488.38 | 4 |  |  |  |  |  |
| 295 | 34 | 1606.6007 | 804.3076 | 7886.41 | 2 | 1606.6100 | C13 | 13 | -9.23e-03 | -5.74 |
| 295 | 35 | 2678.0756 | 893.6992 | 7359.45 | 3 | 2678.0914 | C21 | 21 | -0.0158 | -5.90 |
| 295 | 36 | 4279.7720 | 1070.9503 | 4441.86 | 4 |  |  |  |  |  |
| 295 | 37 | 2085.9625 | 696.3281 | 4986.51 | 3 |  |  |  |  |  |
| 295 | 38 | 4232.7834 | 847.5640 | 6616.62 | 5 |  |  |  |  |  |
| 295 | 39 | 2423.0256 | 808.6825 | 4407.71 | 3 |  |  |  |  |  |
| 295 | 40 | 4119.7152 | 824.9503 | 3904.19 | 5 |  |  |  |  |  |
| 295 | 41 | 3117.3150 | 780.3360 | 4421.74 | 4 |  |  |  |  |  |
| 295 | 42 | 2716.1850 | 906.4023 | 4349.16 | 3 |  |  |  |  |  |
| 295 | 43 | 1644.7100 | 823.3623 | 3670.40 | 2 |  |  |  |  |  |
| 295 | 44 | 3982.5744 | 797.5222 | 5498.31 | 5 |  |  |  |  |  |
| 295 | 45 | 2146.8195 | 1074.4170 | 4360.41 | 2 | 2146.8327 | C17 | 17 | -0.0131 | -6.12 |
| 295 | 46 | 2245.9934 | 749.6717 | 6103.93 | 3 |  |  |  |  |  |
| 295 | 47 | 4354.7593 | 1089.6971 | 2893.39 | 4 |  |  |  |  |  |
| 295 | 48 | 3858.6412 | 965.6676 | 2878.12 | 4 |  |  |  |  |  |
| 295 | 49 | 3729.5443 | 933.3933 | 3657.00 | 4 |  |  |  |  |  |
| 295 | 50 | 2462.9505 | 821.9908 | 3791.61 | 3 | 2462.9644 | C19 | 19 | -0.0139 | -5.66 |
| 295 | 51 | 749.3473 | 750.3546 | 5196.36 | 1 | 749.3490 | C6 | 6 | -1.70e-03 | -2.27 |
| 295 | 52 | 863.3869 | 864.3942 | 4982.05 | 1 | 863.3919 | C7 | 7 | -4.95e-03 | -5.74 |
| 295 | 53 | 1000.4454 | 1001.4526 | 3118.39 | 1 | 1000.4508 | C8 | 8 | -5.45e-03 | -5.45 |
| 295 | 54 | 982.4148 | 983.4221 | 3341.85 | 1 |  |  |  |  |  |
| 295 | 55 | 1376.5485 | 689.2815 | 3800.03 | 2 | 1376.5561 | C11 | 11 | -7.59e-03 | -5.51 |
| 295 | 56 | 912.3869 | 913.3942 | 2628.33 | 1 | 912.3926 | Z\_DOT7 | 27 | -5.67e-03 | -6.22 |
| 295 | 57 | 330.1526 | 331.1599 | 2645.41 | 1 |  |  |  |  |  |
| 295 | 58 | 1275.5016 | 638.7581 | 2148.72 | 2 | 1275.5084 | C10 | 10 | -6.79e-03 | -5.32 |
| 295 | 59 | 694.2925 | 695.2998 | 1579.42 | 1 |  |  |  |  |  |
| 295 | 60 | 885.5616 | 886.5688 | 2904.78 | 1 |  |  |  |  |  |
| 295 | 61 | 500.2230 | 501.2303 | 1184.40 | 1 |  |  |  |  |  |
| 295 | 62 | 1115.4716 | 558.7431 | 2454.96 | 2 | 1115.4778 | C9 | 9 | -6.16e-03 | -5.52 |
| 295 | 63 | 1115.4741 | 1116.4814 | 1139.84 | 1 | 1115.4778 | C9 | 9 | -3.65e-03 | -3.27 |
| 295 | 64 | 822.3873 | 823.3946 | 1175.34 | 1 |  |  |  |  |  |
| 295 | 65 | 593.2449 | 594.2522 | 1599.12 | 1 | 593.2479 | C5 | 5 | -2.94e-03 | -4.96 |
| 295 | 66 | 1037.9304 | 1038.9377 | 622.82 | 1 |  |  |  |  |  |
| 295 | 67 | 1287.5154 | 1288.5226 | 773.43 | 1 |  |  |  |  |  |
| 295 | 68 | 1056.6874 | 1057.6946 | 671.00 | 1 |  |  |  |  |  |
| 295 | 69 | 1422.2536 | 1423.2609 | 564.86 | 1 |  |  |  |  |  |
| 295 | 70 | 1103.1988 | 1104.2061 | 674.97 | 1 |  |  |  |  |  |
| 295 | 71 | 1402.9101 | 702.4623 | 614.81 | 2 |  |  |  |  |  |

  

All proteins /
CsTx-12a\_S1 Cupiennius salei toxin 12 isoform a S1^ACsTx-12a\_S2 Cupiennius salei toxin 12 isoform a S2 /
Proteoform #61
