# Supplementary material for: The Dual Prey-Inactivation Strategy of Spiders—In-Depth Venomic Analysis of Cupiennius salei
Source: Toxins (Basel). 2019 Mar 19;11(3):167. doi: 10.3390/toxins11030167 (PMC6468893; doi:10.3390/toxins11030167)
Supplement: Supplementary file 1 [file toxins-11-00167-s001.zip › Supplementary Dataset EV1/20180328_f2_topdown_OTMS2_EThcD_NL_i02_ms2_proteoform_cutoff_html/prsms/prsm100.html]

Protein-Spectrum-Match for Spectrum #329


All proteins /
CsTx-13a Cupiennius salei toxin 13 isoform a /
Proteoform #22

## Protein-Spectrum-Match #100 for Spectrum #329

|  |  |  |  |  |  |
| --- | --- | --- | --- | --- | --- |
| PrSM ID: | 100 | Scan(s): | 441 | Precursor charge: | 6 |
| Precursor m/z: | 724.7997 | Precursor mass: | 4342.7548 | Proteoform mass: | 4342.7588 |
| # matched peaks: | 26 | # matched fragment ions: | 24 | # unexpected modifications: | 0 |
| E-value: | 4.06e-25 | P-value: | 4.06e-25 | Q-value (Spectral FDR): | 0 |

  

|  |  |  |  |  |  |  |  |  |  |  |  |  |  |  |  |  |  |  |  |  |  |  |  |  |  |  |  |  |  |  |  |  |  |  |  |  |  |  |  |  |  |  |  |  |  |  |  |  |  |  |  |  |  |  |  |  |  |  |  |  |  |  |  |  |  |  |  |  |  |
| --- | --- | --- | --- | --- | --- | --- | --- | --- | --- | --- | --- | --- | --- | --- | --- | --- | --- | --- | --- | --- | --- | --- | --- | --- | --- | --- | --- | --- | --- | --- | --- | --- | --- | --- | --- | --- | --- | --- | --- | --- | --- | --- | --- | --- | --- | --- | --- | --- | --- | --- | --- | --- | --- | --- | --- | --- | --- | --- | --- | --- | --- | --- | --- | --- | --- | --- | --- | --- | --- |
|  | |  | | | | | | | | | | | | | | | | | | | | | | | | | | | | | | | | | | | | | | | | | | | | | | | | | | | | | | | | | | | | | | | | | | | |
| 1 |  |  | M |  | K |  | V |  | L |  | V |  | I |  | F |  | A |  | V |  | L |  |  | S |  | L |  | V |  | I |  | F |  | S |  | N |  | C |  | S |  | A |  |  | E |  | T |  | D |  | E |  | D |  | F |  | F |  | G |  | E |  | E |  | 30 |  |
|  | |  | | | | | | | | | | | | | | | | | | | | | | | | | | | | | | | | | | | | | | | | | | | | | | | | | | | | | | | | | | | | | | | | | | | |
| 31 |  |  | S |  | F |  | E |  | A |  | D |  | D |  | I |  | I |  | P |  | F |  |  | I |  | A |  | K |  | E |  | Q |  | V |  | R | ] | S |  | D |  | C |  |  | T |  | L | ⎫ | R | ⎱ | N |  | H | ⎫ | D | ⎫ | C | ⎫ | T | ⎫ | D | ⎫ | D |  | 60 |  |
|  | |  | | | | | | | | | | | | | | | | | | | | | | | | | | | | | | | | | | | | | | | | | | | | | | | | | | | | | | | | | | | | | | | | | | | |
| 61 |  | ⎩ | R |  | H |  | S | ⎫ | C |  | C |  | R | ⎫ | S | ⎫ | K | ⎫ | M | ⎫ | F |  | ⎱ | K | ⎱ | D |  | V | ⎫ | C | ⎫ | T | ⎫ | C | ⎫ | F | ⎫ | Y |  | P |  | S |  | ⎫ | Q | [ | R |  | S |  | E |  | T |  | A |  | R |  | A |  | K |  | K |  | 90 |  |
|  | |  | | | | | | | | | | | | | | | | | | | | | | | | | | | | | | | | | | | | | | | | | | | | | | | | | | | | | | | | | | | | | | | | | | | |
| 91 |  |  | E |  | L |  | C |  | T |  | C |  | Q |  | Q |  | P |  | K |  | H |  |  | L |  | K |  | Y |  | I |  | E |  | K |  | G |  | L |  | Q |  | K |  |  | A |  | K |  | D |  | Y |  | A |  | T |  | G |  | | 117 |  | | | | | |

Fixed PTMs: Carbamidomethylation [C50 C57 C64 C65 C74 C76 ]

  

All peaks (72)  Matched peaks (26)  Not matched peaks (46)

  

| Scan | Peak | Mono mass | Mono m/z | Intensity | Charge | Theoretical mass | Ion | Pos | Mass error | PPM error |
| --- | --- | --- | --- | --- | --- | --- | --- | --- | --- | --- |
| 441 | 1 | 4285.7079 | 858.1489 | 99393.40 | 5 |  |  |  |  |  |
| 441 | 2 | 4324.7207 | 721.7941 | 112801.95 | 6 |  |  |  |  |  |
| 441 | 3 | 4012.5774 | 803.5228 | 90043.47 | 5 |  |  |  |  |  |
| 441 | 4 | 4196.6610 | 840.3395 | 47556.86 | 5 |  |  |  |  |  |
| 441 | 5 | 4285.7095 | 1072.4347 | 28956.90 | 4 |  |  |  |  |  |
| 441 | 6 | 4326.7138 | 866.3500 | 22838.18 | 5 |  |  |  |  |  |
| 441 | 7 | 4034.6548 | 1009.6710 | 21837.04 | 4 |  |  |  |  |  |
| 441 | 8 | 4213.6877 | 843.7448 | 16212.59 | 5 | 4213.7162 | C33 | 33 | -0.0285 | -6.76 |
| 441 | 9 | 1447.9109 | 724.9627 | 51401.00 | 2 |  |  |  |  |  |
| 441 | 10 | 4327.7251 | 1082.9386 | 16276.77 | 4 |  |  |  |  |  |
| 441 | 11 | 3084.2747 | 772.0760 | 15443.35 | 4 | 3084.2953 | C24 | 24 | -0.0206 | -6.67 |
| 441 | 12 | 3719.4730 | 930.8755 | 11022.58 | 4 | 3719.4996 | C29 | 29 | -0.0266 | -7.16 |
| 441 | 13 | 3529.7394 | 883.4421 | 8354.60 | 4 |  |  |  |  |  |
| 441 | 14 | 2956.1807 | 740.0525 | 8945.13 | 4 | 2956.2003 | C23 | 23 | -0.0196 | -6.64 |
| 441 | 15 | 3866.5389 | 967.6420 | 10482.15 | 4 | 3866.5680 | C30 | 30 | -0.0291 | -7.54 |
| 441 | 16 | 4179.6489 | 836.9371 | 9642.94 | 5 |  |  |  |  |  |
| 441 | 17 | 2172.8728 | 1087.4437 | 26999.38 | 2 |  |  |  |  |  |
| 441 | 18 | 4253.7374 | 851.7548 | 8853.33 | 5 |  |  |  |  |  |
| 441 | 19 | 3559.4452 | 890.8686 | 8116.91 | 4 | 3559.4690 | C28 | 28 | -0.0238 | -6.68 |
| 441 | 20 | 1491.5740 | 746.7943 | 11468.42 | 2 | 1491.5830 | C12 | 12 | -9.02e-03 | -6.05 |
| 441 | 21 | 4267.6958 | 854.5464 | 9192.93 | 5 |  |  |  |  |  |
| 441 | 22 | 2678.0727 | 893.6982 | 10459.01 | 3 | 2678.0914 | C21 | 21 | -0.0187 | -6.99 |
| 441 | 23 | 4228.6871 | 1058.1790 | 7211.71 | 4 |  |  |  |  |  |
| 441 | 24 | 4012.5811 | 1004.1526 | 7827.59 | 4 |  |  |  |  |  |
| 441 | 25 | 3594.3922 | 899.6053 | 7150.08 | 4 | 3594.4177 | Z\_DOT28 | 6 | -0.0255 | -7.08 |
| 441 | 26 | 868.9483 | 869.9556 | 35042.79 | 1 |  |  |  |  |  |
| 441 | 27 | 2549.9767 | 850.9995 | 9816.26 | 3 | 2549.9965 | C20 | 20 | -0.0198 | -7.76 |
| 441 | 28 | 4238.7136 | 848.7500 | 7169.76 | 5 |  |  |  |  |  |
| 441 | 29 | 4299.7173 | 860.9507 | 6553.41 | 5 |  |  |  |  |  |
| 441 | 30 | 2895.4874 | 724.8791 | 42211.82 | 4 |  |  |  |  |  |
| 441 | 31 | 2809.1133 | 703.2856 | 9748.24 | 4 | 2809.1319 | C22 | 22 | -0.0186 | -6.62 |
| 441 | 32 | 2462.9483 | 821.9900 | 7770.05 | 3 | 2462.9644 | C19 | 19 | -0.0161 | -6.55 |
| 441 | 33 | 1376.5478 | 689.2812 | 6882.09 | 2 | 1376.5561 | C11 | 11 | -8.32e-03 | -6.05 |
| 441 | 34 | 3298.3702 | 825.5998 | 6473.85 | 4 | 3298.3906 | C26 | 26 | -0.0205 | -6.21 |
| 441 | 35 | 4268.6929 | 1068.1805 | 5440.50 | 4 |  |  |  |  |  |
| 441 | 36 | 3849.5120 | 770.9097 | 5691.87 | 5 |  |  |  |  |  |
| 441 | 37 | 3984.5843 | 797.9241 | 4360.07 | 5 |  |  |  |  |  |
| 441 | 38 | 2737.1383 | 913.3867 | 4803.84 | 3 | 2737.1567 | Z\_DOT21 | 13 | -0.0184 | -6.71 |
| 441 | 39 | 3524.7394 | 705.9551 | 5251.57 | 5 |  |  |  |  |  |
| 441 | 40 | 4034.6505 | 807.9374 | 4741.02 | 5 |  |  |  |  |  |
| 441 | 41 | 2266.9489 | 756.6569 | 6325.17 | 3 |  |  |  |  |  |
| 441 | 42 | 4195.7007 | 1049.9325 | 6843.52 | 4 |  |  |  |  |  |
| 441 | 43 | 1986.7901 | 994.4023 | 7287.17 | 2 | 1986.8020 | C16 | 16 | -0.0119 | -5.99 |
| 441 | 44 | 4309.7198 | 1078.4372 | 4685.44 | 4 |  |  |  |  |  |
| 441 | 45 | 4306.7028 | 718.7911 | 6926.42 | 6 |  |  |  |  |  |
| 441 | 46 | 4213.6875 | 1054.4292 | 5105.67 | 4 | 4213.7162 | C33 | 33 | -0.0286 | -6.79 |
| 441 | 47 | 3344.2916 | 1115.7711 | 4392.30 | 3 |  |  |  |  |  |
| 441 | 48 | 3458.3986 | 865.6069 | 9520.42 | 4 | 3458.4213 | C27 | 27 | -0.0227 | -6.57 |
| 441 | 49 | 3138.2656 | 1047.0958 | 5189.01 | 3 |  |  |  |  |  |
| 441 | 50 | 4241.6972 | 1061.4316 | 5493.88 | 4 |  |  |  |  |  |
| 441 | 51 | 2809.1125 | 937.3781 | 5761.48 | 3 | 2809.1319 | C22 | 22 | -0.0194 | -6.92 |
| 441 | 52 | 330.1525 | 331.1597 | 8170.49 | 1 |  |  |  |  |  |
| 441 | 53 | 749.3447 | 750.3520 | 6045.84 | 1 | 749.3490 | C6 | 6 | -4.26e-03 | -5.69 |
| 441 | 54 | 1000.4450 | 501.2298 | 5241.46 | 2 | 1000.4508 | C8 | 8 | -5.83e-03 | -5.83 |
| 441 | 55 | 1115.4715 | 558.7430 | 3163.16 | 2 | 1115.4778 | C9 | 9 | -6.28e-03 | -5.63 |
| 441 | 56 | 1474.5469 | 738.2807 | 3939.53 | 2 |  |  |  |  |  |
| 441 | 57 | 724.1247 | 725.1320 | 13868.34 | 1 |  |  |  |  |  |
| 441 | 58 | 512.0754 | 513.0826 | 2745.54 | 1 |  |  |  |  |  |
| 441 | 59 | 1086.6847 | 1087.6920 | 12685.03 | 1 |  |  |  |  |  |
| 441 | 60 | 1275.4987 | 638.7566 | 2236.14 | 2 | 1275.5084 | C10 | 10 | -9.72e-03 | -7.62 |
| 441 | 61 | 843.9418 | 844.9491 | 1251.20 | 1 |  |  |  |  |  |
| 441 | 62 | 1275.4947 | 1276.5020 | 835.37 | 1 |  |  |  |  |  |
| 441 | 63 | 593.2447 | 594.2520 | 779.02 | 1 | 593.2479 | C5 | 5 | -3.13e-03 | -5.27 |
| 441 | 64 | 1058.6738 | 1059.6811 | 990.47 | 1 |  |  |  |  |  |
| 441 | 65 | 1387.5578 | 1388.5651 | 840.74 | 1 | 1387.5663 | Z\_DOT11 | 23 | -8.46e-03 | -6.10 |
| 441 | 66 | 1133.9805 | 1134.9877 | 996.39 | 1 |  |  |  |  |  |
| 441 | 67 | 1442.9142 | 1443.9214 | 448.62 | 1 |  |  |  |  |  |
| 441 | 68 | 1345.5538 | 1346.5611 | 547.08 | 1 |  |  |  |  |  |
| 441 | 69 | 1259.4646 | 1260.4719 | 811.08 | 1 | 1259.4713 | Z\_DOT10 | 24 | -6.76e-03 | -5.37 |
| 441 | 70 | 1409.5675 | 1410.5748 | 531.66 | 1 |  |  |  |  |  |
| 441 | 71 | 822.7317 | 823.7390 | 555.04 | 1 |  |  |  |  |  |
| 441 | 72 | 625.2655 | 626.2727 | 600.43 | 1 |  |  |  |  |  |

  

All proteins /
CsTx-13a Cupiennius salei toxin 13 isoform a /
Proteoform #22
