# Supplementary material for: The Dual Prey-Inactivation Strategy of Spiders—In-Depth Venomic Analysis of Cupiennius salei
Source: Toxins (Basel). 2019 Mar 19;11(3):167. doi: 10.3390/toxins11030167 (PMC6468893; doi:10.3390/toxins11030167)
Supplement: Supplementary file 1 [file toxins-11-00167-s001.zip › Supplementary Dataset EV1/20180328_f2_topdown_OTMS2_EThcD_NL_i02_ms2_proteoform_cutoff_html/prsms/prsm101.html]

Protein-Spectrum-Match for Spectrum #330


All proteins /
CsTx-12a\_S1 Cupiennius salei toxin 12 isoform a S1^ACsTx-12a\_S2 Cupiennius salei toxin 12 isoform a S2 /
Proteoform #18

## Protein-Spectrum-Match #101 for Spectrum #330

|  |  |  |  |  |  |
| --- | --- | --- | --- | --- | --- |
| PrSM ID: | 101 | Scan(s): | 443 | Precursor charge: | 7 |
| Precursor m/z: | 625.2650 | Precursor mass: | 4369.8038 | Proteoform mass: | 4369.8061 |
| # matched peaks: | 26 | # matched fragment ions: | 22 | # unexpected modifications: | 0 |
| E-value: | 2.41e-23 | P-value: | 2.41e-23 | Q-value (Spectral FDR): | 0 |

  

|  |  |  |  |  |  |  |  |  |  |  |  |  |  |  |  |  |  |  |  |  |  |  |  |  |  |  |  |  |  |  |  |  |  |  |  |  |  |  |  |  |  |  |  |  |  |  |  |  |  |  |  |  |  |  |  |  |  |  |  |  |  |  |  |  |  |  |  |  |  |
| --- | --- | --- | --- | --- | --- | --- | --- | --- | --- | --- | --- | --- | --- | --- | --- | --- | --- | --- | --- | --- | --- | --- | --- | --- | --- | --- | --- | --- | --- | --- | --- | --- | --- | --- | --- | --- | --- | --- | --- | --- | --- | --- | --- | --- | --- | --- | --- | --- | --- | --- | --- | --- | --- | --- | --- | --- | --- | --- | --- | --- | --- | --- | --- | --- | --- | --- | --- | --- | --- |
|  | |  | | | | | | | | | | | | | | | | | | | | | | | | | | | | | | | | | | | | | | | | | | | | | | | | | | | | | | | | | | | | | | | | | | | |
| 1 |  |  | M |  | K |  | V |  | L |  | V |  | I |  | C |  | A |  | V |  | L |  |  | F |  | L |  | T |  | I |  | F |  | S |  | N |  | S |  | S |  | A |  |  | E |  | T |  | E |  | D |  | D |  | F |  | L |  | E |  | D |  | E |  | 30 |  |
|  | |  | | | | | | | | | | | | | | | | | | | | | | | | | | | | | | | | | | | | | | | | | | | | | | | | | | | | | | | | | | | | | | | | | | | |
| 31 |  |  | S |  | F |  | E |  | A |  | D |  | D |  | V |  | I |  | P |  | F |  |  | L |  | A |  | R |  | E |  | Q |  | V |  | R | ] | S |  | D |  | C |  |  | T |  | L |  | R | ⎱ | N |  | H | ⎫ | D | ⎫ | C |  | T | ⎫ | D | ⎫ | D |  | 60 |  |
|  | |  | | | | | | | | | | | | | | | | | | | | | | | | | | | | | | | | | | | | | | | | | | | | | | | | | | | | | | | | | | | | | | | | | | | |
| 61 |  | ⎫ | R |  | H |  | S | ⎫ | C | ⎫ | C |  | R | ⎫ | S | ⎱ | K | ⎫ | M | ⎫ | F |  | ⎫ | K | ⎫ | D |  | V | ⎫ | C | ⎫ | K | ⎫ | C | ⎫ | F | ⎫ | Y |  | P |  | S |  | ⎫ | Q | [ | R |  | S |  | D |  | T |  | A |  | R |  | A |  | K |  | K |  | 90 |  |
|  | |  | | | | | | | | | | | | | | | | | | | | | | | | | | | | | | | | | | | | | | | | | | | | | | | | | | | | | | | | | | | | | | | | | | | |
| 91 |  |  | E |  | L |  | C |  | T |  | C |  | Q |  | Q |  | D |  | K |  | H |  |  | L |  | K |  | F |  | I |  | E |  | K |  | G |  | L |  | Q |  | K |  |  | A |  | K |  | V |  | L |  | V |  | A |  | G |  | | 117 |  | | | | | |

Fixed PTMs: Carbamidomethylation [C50 C57 C64 C65 C74 C76 ]

  

All peaks (72)  Matched peaks (26)  Not matched peaks (46)

  

| Scan | Peak | Mono mass | Mono m/z | Intensity | Charge | Theoretical mass | Ion | Pos | Mass error | PPM error |
| --- | --- | --- | --- | --- | --- | --- | --- | --- | --- | --- |
| 443 | 1 | 4312.7548 | 719.7997 | 47604.32 | 6 |  |  |  |  |  |
| 443 | 2 | 4223.7088 | 704.9587 | 20692.69 | 6 |  |  |  |  |  |
| 443 | 3 | 4312.7565 | 863.5586 | 21648.49 | 5 |  |  |  |  |  |
| 443 | 4 | 623.2258 | 624.2331 | 126051.57 | 1 |  |  |  |  |  |
| 443 | 5 | 4351.7671 | 622.6883 | 14917.46 | 7 |  |  |  |  |  |
| 443 | 6 | 2185.3904 | 729.4708 | 33324.65 | 3 |  |  |  |  |  |
| 443 | 7 | 4240.7324 | 707.7960 | 12149.50 | 6 | 4240.7634 | C33 | 33 | -0.0310 | -7.31 |
| 443 | 8 | 1246.5724 | 624.2935 | 18691.84 | 2 |  |  |  |  |  |
| 443 | 9 | 4353.7557 | 726.6332 | 10836.12 | 6 |  |  |  |  |  |
| 443 | 10 | 4061.6990 | 813.3471 | 10854.82 | 5 |  |  |  |  |  |
| 443 | 11 | 4354.7661 | 871.9605 | 9125.92 | 5 |  |  |  |  |  |
| 443 | 12 | 1457.2618 | 729.6382 | 22842.48 | 2 |  |  |  |  |  |
| 443 | 13 | 220.0764 | 221.0836 | 28513.89 | 1 |  |  |  |  |  |
| 443 | 14 | 4240.7360 | 849.1545 | 8143.15 | 5 | 4240.7634 | C33 | 33 | -0.0275 | -6.47 |
| 443 | 15 | 4205.7007 | 701.9574 | 6146.40 | 6 |  |  |  |  |  |
| 443 | 16 | 3084.2750 | 772.0760 | 7993.08 | 4 | 3084.2953 | C24 | 24 | -0.0203 | -6.59 |
| 443 | 17 | 294.0947 | 295.1020 | 27194.84 | 1 |  |  |  |  |  |
| 443 | 18 | 3586.4914 | 718.3056 | 7611.47 | 5 | 3586.5162 | C28 | 28 | -0.0249 | -6.94 |
| 443 | 19 | 4254.7323 | 851.9537 | 7554.87 | 5 |  |  |  |  |  |
| 443 | 20 | 368.1130 | 369.1202 | 25613.54 | 1 |  |  |  |  |  |
| 443 | 21 | 4295.7400 | 860.1553 | 3908.90 | 5 |  |  |  |  |  |
| 443 | 22 | 2678.0735 | 670.5257 | 6303.92 | 4 | 2678.0914 | C21 | 21 | -0.0179 | -6.68 |
| 443 | 23 | 3458.3960 | 692.6865 | 3591.39 | 5 | 3458.4213 | C27 | 27 | -0.0253 | -7.32 |
| 443 | 24 | 1986.7889 | 994.4017 | 3860.37 | 2 | 1986.8020 | C16 | 16 | -0.0131 | -6.61 |
| 443 | 25 | 606.1991 | 607.2064 | 18372.91 | 1 |  |  |  |  |  |
| 443 | 26 | 4334.7563 | 620.2582 | 6087.55 | 7 |  |  |  |  |  |
| 443 | 27 | 3746.5203 | 750.3113 | 3705.29 | 5 | 3746.5469 | C29 | 29 | -0.0266 | -7.09 |
| 443 | 28 | 3298.3706 | 825.5999 | 4056.53 | 4 | 3298.3906 | C26 | 26 | -0.0200 | -6.06 |
| 443 | 29 | 2498.3016 | 625.5827 | 18468.75 | 4 |  |  |  |  |  |
| 443 | 30 | 2293.9984 | 765.6734 | 3623.99 | 3 |  |  |  |  |  |
| 443 | 31 | 1491.5738 | 746.7942 | 6233.74 | 2 | 1491.5830 | C12 | 12 | -9.26e-03 | -6.21 |
| 443 | 32 | 2133.9660 | 712.3293 | 4483.41 | 3 |  |  |  |  |  |
| 443 | 33 | 2549.9805 | 851.0008 | 3887.60 | 3 | 2549.9965 | C20 | 20 | -0.0159 | -6.25 |
| 443 | 34 | 3747.0948 | 625.5231 | 15479.16 | 6 |  |  |  |  |  |
| 443 | 35 | 1376.5468 | 689.2807 | 4253.48 | 2 | 1376.5561 | C11 | 11 | -9.30e-03 | -6.76 |
| 443 | 36 | 4314.7681 | 1079.6993 | 3879.71 | 4 |  |  |  |  |  |
| 443 | 37 | 3586.4947 | 897.6310 | 3223.06 | 4 | 3586.5162 | C28 | 28 | -0.0215 | -6.00 |
| 443 | 38 | 2956.1819 | 740.0528 | 3773.85 | 4 | 2956.2003 | C23 | 23 | -0.0184 | -6.22 |
| 443 | 39 | 2809.1136 | 703.2857 | 3978.13 | 4 | 2809.1319 | C22 | 22 | -0.0183 | -6.53 |
| 443 | 40 | 4368.7711 | 625.1174 | 19519.88 | 7 |  |  |  |  |  |
| 443 | 41 | 3621.4477 | 906.3692 | 2887.82 | 4 | 3621.4649 | Z\_DOT28 | 6 | -0.0172 | -4.76 |
| 443 | 42 | 2462.9483 | 821.9900 | 2768.08 | 3 | 2462.9644 | C19 | 19 | -0.0161 | -6.55 |
| 443 | 43 | 4262.7575 | 711.4669 | 4010.58 | 6 |  |  |  |  |  |
| 443 | 44 | 4222.7337 | 845.5540 | 4064.50 | 5 |  |  |  |  |  |
| 443 | 45 | 413.0439 | 414.0512 | 10389.45 | 1 |  |  |  |  |  |
| 443 | 46 | 4327.7614 | 722.3008 | 2839.98 | 6 |  |  |  |  |  |
| 443 | 47 | 2549.9810 | 638.5025 | 4568.59 | 4 | 2549.9965 | C20 | 20 | -0.0155 | -6.06 |
| 443 | 48 | 3165.3116 | 792.3352 | 3319.18 | 4 |  |  |  |  |  |
| 443 | 49 | 1000.4451 | 501.2298 | 4167.80 | 2 | 1000.4508 | C8 | 8 | -5.71e-03 | -5.71 |
| 443 | 50 | 2146.8182 | 716.6133 | 3311.16 | 3 | 2146.8327 | C17 | 17 | -0.0145 | -6.75 |
| 443 | 51 | 874.5595 | 875.5668 | 4889.36 | 1 |  |  |  |  |  |
| 443 | 52 | 4278.7166 | 714.1267 | 3152.47 | 6 |  |  |  |  |  |
| 443 | 53 | 4062.7056 | 1016.6837 | 2820.16 | 4 |  |  |  |  |  |
| 443 | 54 | 4005.6836 | 1002.4282 | 2336.38 | 4 |  |  |  |  |  |
| 443 | 55 | 1606.6001 | 804.3073 | 5431.21 | 2 | 1606.6100 | C13 | 13 | -9.84e-03 | -6.12 |
| 443 | 56 | 2678.0771 | 893.6996 | 1760.48 | 3 | 2678.0914 | C21 | 21 | -0.0143 | -5.35 |
| 443 | 57 | 1820.8071 | 911.4108 | 3227.71 | 2 | 1820.8174 | Z\_DOT14 | 20 | -0.0104 | -5.69 |
| 443 | 58 | 3893.5881 | 779.7249 | 3469.64 | 5 | 3893.6153 | C30 | 30 | -0.0273 | -7.00 |
| 443 | 59 | 1474.5480 | 738.2813 | 2939.22 | 2 |  |  |  |  |  |
| 443 | 60 | 1875.1706 | 626.0642 | 8639.16 | 3 |  |  |  |  |  |
| 443 | 61 | 442.1312 | 443.1385 | 3901.72 | 1 |  |  |  |  |  |
| 443 | 62 | 1115.4709 | 558.7427 | 2265.04 | 2 | 1115.4778 | C9 | 9 | -6.89e-03 | -6.18 |
| 443 | 63 | 749.3445 | 750.3517 | 2942.76 | 1 | 749.3490 | C6 | 6 | -4.51e-03 | -6.01 |
| 443 | 64 | 470.0651 | 471.0724 | 3878.52 | 1 |  |  |  |  |  |
| 443 | 65 | 238.0868 | 239.0941 | 2216.17 | 1 |  |  |  |  |  |
| 443 | 66 | 516.1496 | 517.1569 | 2320.89 | 1 |  |  |  |  |  |
| 443 | 67 | 1093.4477 | 1094.4550 | 702.46 | 1 |  |  |  |  |  |
| 443 | 68 | 493.2149 | 494.2221 | 798.14 | 1 |  |  |  |  |  |
| 443 | 69 | 1045.9285 | 1046.9357 | 489.64 | 1 |  |  |  |  |  |
| 443 | 70 | 1231.6875 | 616.8510 | 693.48 | 2 |  |  |  |  |  |
| 443 | 71 | 1415.6076 | 708.8111 | 483.17 | 2 |  |  |  |  |  |
| 443 | 72 | 588.0550 | 589.0623 | 751.89 | 1 |  |  |  |  |  |

  

All proteins /
CsTx-12a\_S1 Cupiennius salei toxin 12 isoform a S1^ACsTx-12a\_S2 Cupiennius salei toxin 12 isoform a S2 /
Proteoform #18
