# Supplementary material for: The Dual Prey-Inactivation Strategy of Spiders—In-Depth Venomic Analysis of Cupiennius salei
Source: Toxins (Basel). 2019 Mar 19;11(3):167. doi: 10.3390/toxins11030167 (PMC6468893; doi:10.3390/toxins11030167)
Supplement: Supplementary file 1 [file toxins-11-00167-s001.zip › Supplementary Dataset EV1/20180328_f2_topdown_OTMS2_EThcD_NL_i02_ms2_proteoform_cutoff_html/prsms/prsm102.html]

Protein-Spectrum-Match for Spectrum #331


All proteins /
CsTx-13b Cupiennius salei toxin 13 isoform b /
Proteoform #60

## Protein-Spectrum-Match #102 for Spectrum #331

|  |  |  |  |  |  |
| --- | --- | --- | --- | --- | --- |
| PrSM ID: | 102 | Scan(s): | 444 | Precursor charge: | 6 |
| Precursor m/z: | 678.3566 | Precursor mass: | 4064.0961 | Proteoform mass: | 4064.0811 |
| # matched peaks: | 27 | # matched fragment ions: | 25 | # unexpected modifications: | 1 |
| E-value: | 2.25e-19 | P-value: | 2.25e-19 | Q-value (Spectral FDR): | 0 |

  

|  |  |  |  |  |  |  |  |  |  |  |  |  |  |  |  |  |  |  |  |  |  |  |  |  |  |  |  |  |  |  |  |  |  |  |  |  |  |  |  |  |  |  |  |  |  |  |  |  |  |  |  |  |  |  |  |  |  |  |  |  |  |  |  |  |  |  |
| --- | --- | --- | --- | --- | --- | --- | --- | --- | --- | --- | --- | --- | --- | --- | --- | --- | --- | --- | --- | --- | --- | --- | --- | --- | --- | --- | --- | --- | --- | --- | --- | --- | --- | --- | --- | --- | --- | --- | --- | --- | --- | --- | --- | --- | --- | --- | --- | --- | --- | --- | --- | --- | --- | --- | --- | --- | --- | --- | --- | --- | --- | --- | --- | --- | --- | --- |
|  | | ... 30 amino acid residues are skipped at the N-terminus ... | | | | | | | | | | | | | | | | | | | | | | | | | | | | | | | | | | | | | | | | | | | | | | | | | | | | | | | | | | | | | |  | | |
|  | |  | | | | | | | | | | | | | | | | | | | | | | | | | | | | | | | | | | | | | | | | | | | | | | | | | | | | | | | | | | | | | | | | | | | |
| 31 |  |  | S |  | F |  | E |  | A |  | D |  | D |  | I |  | I |  | P |  | F |  |  | I |  | A |  | K |  | E |  | Q |  | V |  | R |  | S |  | D |  | C |  |  | T |  | L |  | R |  | N |  | H |  | D |  | C |  | T |  | D |  | D |  | 60 |  |
|  | |  | | | | | | | | | | | | | | | | | | | | | | | | | | | | | | | | | | | | | | | | | | | | | | | | | | | | | | | | | | | | | | | | | | | |
| 61 |  |  | R |  | H |  | S |  | C |  | C |  | R |  | S |  | K |  | M |  | F |  |  | K |  | D |  | V |  | C |  | T |  | C |  | F |  | Y |  | P |  | S |  |  | Q |  | R | ] | S |  | E |  | T |  | D |  | R | ⎱ | A | ⎩ | K |  | K |  | 90 |  |
|  | |  | | | | | | | | | | | | | | | | | | | | | | | | | | | | | | | | | | | | | | | | | | | | | | | | | | | | | -58.02 | | | | | | | | | | | |
| 91 |  |  | E |  | L | ⎫ | C |  | T | ⎫ | C | ⎱ | Q | ⎱ | Q |  | P | ⎱ | K | ⎱ | H |  |  | L | ⎱ | K | ⎱ | Y |  | I |  | E | ⎩ | K |  | G |  | L |  | Q | ⎫ | K |  | ⎱ | A |  | K |  | D | ⎫ | Y | ⎫ | A | ⎫ | T | ⎫ | G |  | | 117 |  | | | | | |

Fixed PTMs: Carbamidomethylation [C93 C95 ]   
  
     Unexpected modifications:   Unknown [-58.02]

  

All peaks (55)  Matched peaks (27)  Not matched peaks (28)

  

| Scan | Peak | Mono mass | Mono m/z | Intensity | Charge | Theoretical mass | Ion | Pos | Mass error | PPM error |
| --- | --- | --- | --- | --- | --- | --- | --- | --- | --- | --- |
| 444 | 1 | 2032.5333 | 678.5184 | 81042.44 | 3 |  |  |  |  |  |
| 444 | 2 | 3384.8111 | 677.9695 | 32438.42 | 5 |  |  |  |  |  |
| 444 | 3 | 3614.8976 | 904.7317 | 10759.39 | 4 |  |  |  |  |  |
| 444 | 4 | 4048.0477 | 810.6168 | 11205.94 | 5 |  |  |  |  |  |
| 444 | 5 | 4006.0415 | 802.2156 | 19124.90 | 5 | 4006.0756 | C34 | 34 | -0.0341 | -8.51 |
| 444 | 6 | 3729.9242 | 933.4883 | 11272.25 | 4 |  |  |  |  |  |
| 444 | 7 | 4007.0433 | 1002.7681 | 10121.55 | 4 |  |  |  |  |  |
| 444 | 8 | 2710.0540 | 678.5208 | 74866.55 | 4 |  |  |  |  |  |
| 444 | 9 | 3384.8136 | 847.2107 | 13865.86 | 4 |  |  |  |  |  |
| 444 | 10 | 677.3454 | 678.3527 | 20999.86 | 1 |  |  |  |  |  |
| 444 | 11 | 1625.8666 | 813.9406 | 11358.29 | 2 |  |  |  |  |  |
| 444 | 12 | 3728.9163 | 746.7905 | 5489.23 | 5 | 3728.9453 | C31 | 31 | -0.0291 | -7.79 |
| 444 | 13 | 3414.7628 | 854.6980 | 5136.11 | 4 | 3414.7863 | C28 | 28 | -0.0235 | -6.88 |
| 444 | 14 | 2327.1320 | 776.7179 | 6935.17 | 3 | 2327.1474 | C19 | 19 | -0.0154 | -6.60 |
| 444 | 15 | 3891.9833 | 779.4039 | 4752.47 | 5 | 3892.0087 | C32 | 32 | -0.0254 | -6.52 |
| 444 | 16 | 2033.0406 | 1017.5276 | 8618.72 | 2 |  |  |  |  |  |
| 444 | 17 | 4049.0530 | 1013.2705 | 5530.80 | 4 |  |  |  |  |  |
| 444 | 18 | 2455.2260 | 819.4159 | 6042.95 | 3 | 2455.2423 | C20 | 20 | -0.0163 | -6.64 |
| 444 | 19 | 2076.9918 | 693.3379 | 4637.46 | 3 | 2077.0044 | C17 | 17 | -0.0126 | -6.06 |
| 444 | 20 | 3832.9791 | 959.2520 | 5095.16 | 4 |  |  |  |  |  |
| 444 | 21 | 3962.0205 | 793.4114 | 3569.91 | 5 |  |  |  |  |  |
| 444 | 22 | 1737.9446 | 869.9796 | 6103.41 | 2 | 1737.9416 | Z\_DOT16 | 19 | 3.04e-03 | 1.75 |
| 444 | 23 | 1609.8485 | 805.9315 | 4769.95 | 2 | 1609.8466 | Z\_DOT15 | 20 | 1.93e-03 | 1.20 |
| 444 | 24 | 2116.1775 | 706.3998 | 4840.88 | 3 | 2116.1795 | Z\_DOT19 | 16 | -2.00e-03 | -0.94 |
| 444 | 25 | 1948.8969 | 975.4557 | 4315.73 | 2 | 1948.9094 | C16 | 16 | -0.0125 | -6.41 |
| 444 | 26 | 3286.6700 | 1096.5640 | 2788.33 | 3 | 3286.6914 | C27 | 27 | -0.0213 | -6.49 |
| 444 | 27 | 1988.0853 | 995.0499 | 3117.48 | 2 | 1988.0845 | Z\_DOT18 | 17 | 7.40e-04 | 0.37 |
| 444 | 28 | 813.4156 | 814.4229 | 18302.75 | 1 |  |  |  |  |  |
| 444 | 29 | 3963.0264 | 991.7639 | 3529.51 | 4 | 3963.0458 | C33 | 33 | -0.0194 | -4.90 |
| 444 | 30 | 1723.7856 | 862.9001 | 3451.70 | 2 | 1723.7981 | C14 | 14 | -0.0125 | -7.28 |
| 444 | 31 | 1220.6801 | 611.3473 | 4992.15 | 2 |  |  |  |  |  |
| 444 | 32 | 3975.0692 | 796.0211 | 3276.64 | 5 |  |  |  |  |  |
| 444 | 33 | 1595.7276 | 798.8711 | 2413.61 | 2 | 1595.7395 | C13 | 13 | -0.0119 | -7.49 |
| 444 | 34 | 3459.8016 | 865.9577 | 2173.29 | 4 | 3459.8121 | Z\_DOT30 | 5 | -0.0105 | -3.02 |
| 444 | 35 | 4022.0571 | 805.4187 | 3568.92 | 5 |  |  |  |  |  |
| 444 | 36 | 2469.3462 | 824.1227 | 2604.40 | 3 | 2469.3494 | Z\_DOT22 | 13 | -3.23e-03 | -1.31 |
| 444 | 37 | 2816.4004 | 939.8074 | 3317.52 | 3 |  |  |  |  |  |
| 444 | 38 | 2411.2127 | 804.7449 | 5204.02 | 3 |  |  |  |  |  |
| 444 | 39 | 3388.7712 | 1130.5977 | 1971.00 | 3 | 3388.7750 | Z\_DOT29 | 6 | -3.76e-03 | -1.11 |
| 444 | 40 | 3891.9830 | 974.0030 | 2881.00 | 4 | 3892.0087 | C32 | 32 | -0.0257 | -6.60 |
| 444 | 41 | 2341.2879 | 781.4366 | 2666.77 | 3 | 2341.2908 | Z\_DOT21 | 14 | -2.93e-03 | -1.25 |
| 444 | 42 | 3684.9173 | 922.2366 | 2572.76 | 4 |  |  |  |  |  |
| 444 | 43 | 4021.0633 | 1006.2731 | 4619.27 | 4 |  |  |  |  |  |
| 444 | 44 | 3286.6675 | 822.6742 | 2498.00 | 4 | 3286.6914 | C27 | 27 | -0.0238 | -7.25 |
| 444 | 45 | 3930.0550 | 787.0183 | 2783.85 | 5 |  |  |  |  |  |
| 444 | 46 | 605.2737 | 606.2809 | 1123.84 | 1 | 605.2768 | C5 | 5 | -3.18e-03 | -5.26 |
| 444 | 47 | 1435.6986 | 718.8566 | 1999.02 | 2 | 1435.7089 | C12 | 12 | -0.0102 | -7.12 |
| 444 | 48 | 650.3108 | 651.3181 | 1186.69 | 1 | 650.3026 | Z\_DOT7 | 28 | 8.23e-03 | 12.65 |
| 444 | 49 | 1174.6239 | 1175.6312 | 648.30 | 1 | 1174.6305 | C10 | 10 | -6.64e-03 | -5.66 |
| 444 | 50 | 1087.5597 | 1088.5670 | 733.54 | 1 |  |  |  |  |  |
| 444 | 51 | 1204.6614 | 603.3380 | 683.11 | 2 | 1204.6566 | Z\_DOT12 | 23 | 4.78e-03 | 3.97 |
| 444 | 52 | 1220.6797 | 1221.6870 | 427.06 | 1 |  |  |  |  |  |
| 444 | 53 | 1336.0156 | 1337.0229 | 322.41 | 1 |  |  |  |  |  |
| 444 | 54 | 1356.0312 | 1357.0385 | 511.93 | 1 |  |  |  |  |  |
| 444 | 55 | 1006.5179 | 1007.5251 | 821.16 | 1 |  |  |  |  |  |

  

All proteins /
CsTx-13b Cupiennius salei toxin 13 isoform b /
Proteoform #60
