# Supplementary material for: The Dual Prey-Inactivation Strategy of Spiders—In-Depth Venomic Analysis of Cupiennius salei
Source: Toxins (Basel). 2019 Mar 19;11(3):167. doi: 10.3390/toxins11030167 (PMC6468893; doi:10.3390/toxins11030167)
Supplement: Supplementary file 1 [file toxins-11-00167-s001.zip › Supplementary Dataset EV1/20180328_f2_topdown_OTMS2_EThcD_NL_i02_ms2_proteoform_cutoff_html/prsms/prsm103.html]

Protein-Spectrum-Match for Spectrum #333


All proteins /
CsTx-13a Cupiennius salei toxin 13 isoform a /
Proteoform #40

## Protein-Spectrum-Match #103 for Spectrum #333

|  |  |  |  |  |  |
| --- | --- | --- | --- | --- | --- |
| PrSM ID: | 103 | Scan(s): | 447 | Precursor charge: | 6 |
| Precursor m/z: | 580.3143 | Precursor mass: | 3475.8420 | Proteoform mass: | 3475.8370 |
| # matched peaks: | 32 | # matched fragment ions: | 26 | # unexpected modifications: | 1 |
| E-value: | 1.68e-20 | P-value: | 1.68e-20 | Q-value (Spectral FDR): | 0 |

  

|  |  |  |  |  |  |  |  |  |  |  |  |  |  |  |  |  |  |  |  |  |  |  |  |  |  |  |  |  |  |  |  |  |  |  |  |  |  |  |  |  |  |  |  |  |  |  |  |  |  |  |  |  |  |  |  |  |  |  |  |  |  |  |  |  |  |  |
| --- | --- | --- | --- | --- | --- | --- | --- | --- | --- | --- | --- | --- | --- | --- | --- | --- | --- | --- | --- | --- | --- | --- | --- | --- | --- | --- | --- | --- | --- | --- | --- | --- | --- | --- | --- | --- | --- | --- | --- | --- | --- | --- | --- | --- | --- | --- | --- | --- | --- | --- | --- | --- | --- | --- | --- | --- | --- | --- | --- | --- | --- | --- | --- | --- | --- | --- |
|  | | ... 30 amino acid residues are skipped at the N-terminus ... | | | | | | | | | | | | | | | | | | | | | | | | | | | | | | | | | | | | | | | | | | | | | | | | | | | | | | | | | | | | | |  | | |
|  | |  | | | | | | | | | | | | | | | | | | | | | | | | | | | | | | | | | | | | | | | | | | | | | | | | | | | | | | | | | | | | | | | | | | | |
| 31 |  |  | S |  | F |  | E |  | A |  | D |  | D |  | I |  | I |  | P |  | F |  |  | I |  | A |  | K |  | E |  | Q |  | V |  | R |  | S |  | D |  | C |  |  | T |  | L |  | R |  | N |  | H |  | D |  | C |  | T |  | D |  | D |  | 60 |  |
|  | |  | | | | | | | | | | | | | | | | | | | | | | | | | | | | | | | | | | | | | | | | | | | | | | | | | | | | | | | | | | | | | | | | | | | |
| 61 |  |  | R |  | H |  | S |  | C |  | C |  | R |  | S |  | K |  | M |  | F |  |  | K |  | D |  | V |  | C |  | T |  | C |  | F |  | Y |  | P |  | S |  |  | Q |  | R |  | S |  | E |  | T |  | A |  | R | ] | A | ⎩ | K | ⎩ | K |  | 90 |  |
|  | |  | | | | | | | | | | | | | | | | | | | | | | | | | | | | | | | | | | | | | | | | | | | | | | | | | | | -58.01 | | | | | | | | | | | | | |
| 91 |  |  | E | ⎱ | L |  | C |  | T | ⎫ | C | ⎫ | Q | ⎱ | Q |  | P | ⎱ | K | ⎫ | H |  |  | L |  | K | ⎱ | Y |  | I | ⎱ | E | ⎱ | K | ⎱ | G |  | L |  | Q | ⎱ | K |  | ⎱ | A |  | K | ⎫ | D | ⎫ | Y | ⎫ | A |  | T |  | G |  | | 117 |  | | | | | |

Fixed PTMs: Carbamidomethylation [C93 C95 ]   
  
     Unexpected modifications:   Unknown [-58.01]

  

All peaks (57)  Matched peaks (32)  Not matched peaks (25)

  

| Scan | Peak | Mono mass | Mono m/z | Intensity | Charge | Theoretical mass | Ion | Pos | Mass error | PPM error |
| --- | --- | --- | --- | --- | --- | --- | --- | --- | --- | --- |
| 447 | 1 | 3418.7991 | 684.7671 | 163959.04 | 5 |  |  |  |  |  |
| 447 | 2 | 1738.4163 | 580.4794 | 393462.31 | 3 |  |  |  |  |  |
| 447 | 3 | 3025.6504 | 757.4199 | 72074.07 | 4 | 3025.6680 | C25 | 25 | -0.0176 | -5.82 |
| 447 | 4 | 3140.6763 | 786.1764 | 71514.84 | 4 | 3140.6950 | C26 | 26 | -0.0187 | -5.95 |
| 447 | 5 | 3474.8342 | 580.1463 | 293402.19 | 6 |  |  |  |  |  |
| 447 | 6 | 2272.1689 | 758.3969 | 58105.66 | 3 | 2272.1820 | C18 | 18 | -0.0131 | -5.75 |
| 447 | 7 | 3418.8016 | 855.7077 | 52962.23 | 4 |  |  |  |  |  |
| 447 | 8 | 2698.4263 | 900.4827 | 43762.65 | 3 | 2698.4410 | C22 | 22 | -0.0147 | -5.44 |
| 447 | 9 | 2143.1270 | 715.3829 | 47655.69 | 3 | 2143.1394 | C17 | 17 | -0.0124 | -5.79 |
| 447 | 10 | 2826.5191 | 707.6370 | 42016.40 | 4 | 2826.5360 | C23 | 23 | -0.0169 | -5.97 |
| 447 | 11 | 3459.8053 | 692.9683 | 37986.26 | 5 |  |  |  |  |  |
| 447 | 12 | 1866.9814 | 623.3344 | 65271.14 | 3 | 1866.9920 | C15 | 15 | -0.0106 | -5.67 |
| 447 | 13 | 1609.8504 | 805.9325 | 54905.61 | 2 | 1609.8529 | Z\_DOT15 | 15 | -2.51e-03 | -1.56 |
| 447 | 14 | 3260.6727 | 816.1754 | 33290.57 | 4 | 3260.6863 | Z\_DOT28 | 2 | -0.0136 | -4.18 |
| 447 | 15 | 2116.1801 | 706.4006 | 47430.42 | 3 | 2116.1858 | Z\_DOT19 | 11 | -5.71e-03 | -2.70 |
| 447 | 16 | 579.6381 | 580.6454 | 241353.82 | 1 |  |  |  |  |  |
| 447 | 17 | 3303.7400 | 826.9423 | 29372.33 | 4 | 3303.7583 | C27 | 27 | -0.0183 | -5.53 |
| 447 | 18 | 2539.3770 | 635.8515 | 33919.58 | 4 |  |  |  |  |  |
| 447 | 19 | 2800.4517 | 701.1202 | 38133.52 | 4 |  |  |  |  |  |
| 447 | 20 | 2341.2907 | 781.4375 | 35626.21 | 3 | 2341.2971 | Z\_DOT21 | 9 | -6.45e-03 | -2.76 |
| 447 | 21 | 1360.6519 | 681.3332 | 37072.21 | 2 | 1360.6591 | C11 | 11 | -7.20e-03 | -5.29 |
| 447 | 22 | 3303.7394 | 661.7552 | 25588.16 | 5 | 3303.7583 | C27 | 27 | -0.0189 | -5.72 |
| 447 | 23 | 1625.8688 | 813.9417 | 38582.17 | 2 |  |  |  |  |  |
| 447 | 24 | 3432.8173 | 859.2116 | 23772.06 | 4 |  |  |  |  |  |
| 447 | 25 | 3388.7644 | 678.7601 | 23996.24 | 5 | 3388.7813 | Z\_DOT29 | 1 | -0.0169 | -4.99 |
| 447 | 26 | 3025.6508 | 1009.5575 | 23469.81 | 3 | 3025.6680 | C25 | 25 | -0.0172 | -5.70 |
| 447 | 27 | 3458.8033 | 577.4745 | 25263.13 | 6 |  |  |  |  |  |
| 447 | 28 | 2960.4837 | 741.1282 | 20136.01 | 4 |  |  |  |  |  |
| 447 | 29 | 1204.6624 | 603.3385 | 27260.37 | 2 | 1204.6629 | Z\_DOT12 | 18 | -5.15e-04 | -0.43 |
| 447 | 30 | 3460.8146 | 866.2109 | 23651.29 | 4 |  |  |  |  |  |
| 447 | 31 | 3474.8238 | 695.9720 | 106733.06 | 5 |  |  |  |  |  |
| 447 | 32 | 3003.5376 | 751.8917 | 19230.32 | 4 | 3003.5487 | Z\_DOT26 | 4 | -0.0111 | -3.69 |
| 447 | 33 | 3431.8091 | 687.3691 | 17808.10 | 5 |  |  |  |  |  |
| 447 | 34 | 695.5665 | 696.5738 | 119069.33 | 1 |  |  |  |  |  |
| 447 | 35 | 2698.4261 | 675.6138 | 24991.67 | 4 | 2698.4410 | C22 | 22 | -0.0149 | -5.54 |
| 447 | 36 | 2400.2631 | 801.0950 | 17210.72 | 3 | 2400.2769 | C19 | 19 | -0.0139 | -5.78 |
| 447 | 37 | 1390.7323 | 696.3734 | 108574.53 | 2 |  |  |  |  |  |
| 447 | 38 | 1488.7463 | 745.3804 | 18032.94 | 2 | 1488.7540 | C12 | 12 | -7.80e-03 | -5.24 |
| 447 | 39 | 1333.7044 | 667.8595 | 23865.23 | 2 | 1333.7055 | Z\_DOT13 | 17 | -1.11e-03 | -0.83 |
| 447 | 40 | 1135.5420 | 568.7783 | 22759.83 | 2 | 1135.5477 | C9 | 9 | -5.72e-03 | -5.04 |
| 447 | 41 | 869.4602 | 870.4675 | 16745.89 | 1 |  |  |  |  |  |
| 447 | 42 | 1220.6814 | 611.3480 | 9756.27 | 2 |  |  |  |  |  |
| 447 | 43 | 473.2941 | 474.3013 | 17822.10 | 1 | 473.2961 | C4 | 4 | -2.07e-03 | -4.36 |
| 447 | 44 | 650.3119 | 651.3192 | 14217.94 | 1 | 650.3089 | Z\_DOT7 | 23 | 2.99e-03 | 4.60 |
| 447 | 45 | 1135.5421 | 1136.5494 | 9925.08 | 1 | 1135.5477 | C9 | 9 | -5.65e-03 | -4.97 |
| 447 | 46 | 526.2964 | 527.3037 | 12057.26 | 1 |  |  |  |  |  |
| 447 | 47 | 1488.7459 | 497.2559 | 9288.24 | 3 | 1488.7540 | C12 | 12 | -8.12e-03 | -5.45 |
| 447 | 48 | 847.4552 | 848.4625 | 11118.72 | 1 | 847.4585 | C7 | 7 | -3.27e-03 | -3.85 |
| 447 | 49 | 778.4060 | 779.4133 | 8202.66 | 1 | 778.4038 | Z\_DOT8 | 22 | 2.15e-03 | 2.76 |
| 447 | 50 | 1007.4836 | 1008.4909 | 6080.81 | 1 | 1007.4892 | C8 | 8 | -5.54e-03 | -5.50 |
| 447 | 51 | 1274.6912 | 638.3529 | 6396.66 | 2 |  |  |  |  |  |
| 447 | 52 | 976.4926 | 489.2536 | 6994.04 | 2 |  |  |  |  |  |
| 447 | 53 | 564.0609 | 565.0682 | 8184.19 | 1 |  |  |  |  |  |
| 447 | 54 | 1007.4842 | 504.7494 | 4388.75 | 2 | 1007.4892 | C8 | 8 | -5.00e-03 | -4.97 |
| 447 | 55 | 1417.7488 | 709.8817 | 2836.22 | 2 |  |  |  |  |  |
| 447 | 56 | 1076.5673 | 539.2909 | 2487.86 | 2 | 1076.5679 | Z\_DOT11 | 19 | -6.48e-04 | -0.60 |
| 447 | 57 | 1205.6702 | 1206.6774 | 2482.11 | 1 |  |  |  |  |  |

  

All proteins /
CsTx-13a Cupiennius salei toxin 13 isoform a /
Proteoform #40
