# Supplementary material for: The Dual Prey-Inactivation Strategy of Spiders—In-Depth Venomic Analysis of Cupiennius salei
Source: Toxins (Basel). 2019 Mar 19;11(3):167. doi: 10.3390/toxins11030167 (PMC6468893; doi:10.3390/toxins11030167)
Supplement: Supplementary file 1 [file toxins-11-00167-s001.zip › Supplementary Dataset EV1/20180328_f2_topdown_OTMS2_EThcD_NL_i02_ms2_proteoform_cutoff_html/prsms/prsm104.html]

Protein-Spectrum-Match for Spectrum #334


All proteins /
CsTx-12a\_S1 Cupiennius salei toxin 12 isoform a S1^ACsTx-12a\_S2 Cupiennius salei toxin 12 isoform a S2 /
Proteoform #18

## Protein-Spectrum-Match #104 for Spectrum #334

|  |  |  |  |  |  |
| --- | --- | --- | --- | --- | --- |
| PrSM ID: | 104 | Scan(s): | 448 | Precursor charge: | 6 |
| Precursor m/z: | 729.3078 | Precursor mass: | 4369.8031 | Proteoform mass: | 4369.8061 |
| # matched peaks: | 34 | # matched fragment ions: | 31 | # unexpected modifications: | 0 |
| E-value: | 3.07e-29 | P-value: | 3.07e-29 | Q-value (Spectral FDR): | 0 |

  

|  |  |  |  |  |  |  |  |  |  |  |  |  |  |  |  |  |  |  |  |  |  |  |  |  |  |  |  |  |  |  |  |  |  |  |  |  |  |  |  |  |  |  |  |  |  |  |  |  |  |  |  |  |  |  |  |  |  |  |  |  |  |  |  |  |  |  |  |  |  |
| --- | --- | --- | --- | --- | --- | --- | --- | --- | --- | --- | --- | --- | --- | --- | --- | --- | --- | --- | --- | --- | --- | --- | --- | --- | --- | --- | --- | --- | --- | --- | --- | --- | --- | --- | --- | --- | --- | --- | --- | --- | --- | --- | --- | --- | --- | --- | --- | --- | --- | --- | --- | --- | --- | --- | --- | --- | --- | --- | --- | --- | --- | --- | --- | --- | --- | --- | --- | --- | --- |
|  | |  | | | | | | | | | | | | | | | | | | | | | | | | | | | | | | | | | | | | | | | | | | | | | | | | | | | | | | | | | | | | | | | | | | | |
| 1 |  |  | M |  | K |  | V |  | L |  | V |  | I |  | C |  | A |  | V |  | L |  |  | F |  | L |  | T |  | I |  | F |  | S |  | N |  | S |  | S |  | A |  |  | E |  | T |  | E |  | D |  | D |  | F |  | L |  | E |  | D |  | E |  | 30 |  |
|  | |  | | | | | | | | | | | | | | | | | | | | | | | | | | | | | | | | | | | | | | | | | | | | | | | | | | | | | | | | | | | | | | | | | | | |
| 31 |  |  | S |  | F |  | E |  | A |  | D |  | D |  | V |  | I |  | P |  | F |  |  | L |  | A |  | R |  | E |  | Q |  | V |  | R | ] | S |  | D |  | C |  |  | T | ⎫ | L | ⎫ | R | ⎱ | N | ⎩ | H | ⎱ | D | ⎫ | C | ⎫ | T | ⎫ | D | ⎱ | D |  | 60 |  |
|  | |  | | | | | | | | | | | | | | | | | | | | | | | | | | | | | | | | | | | | | | | | | | | | | | | | | | | | | | | | | | | | | | | | | | | |
| 61 |  | ⎱ | R |  | H |  | S | ⎫ | C |  | C | ⎫ | R | ⎱ | S | ⎱ | K | ⎫ | M |  | F |  |  | K | ⎫ | D | ⎱ | V | ⎫ | C | ⎫ | K | ⎫ | C | ⎫ | F | ⎫ | Y |  | P | ⎫ | S |  | ⎫ | Q | [ | R |  | S |  | D |  | T |  | A |  | R |  | A |  | K |  | K |  | 90 |  |
|  | |  | | | | | | | | | | | | | | | | | | | | | | | | | | | | | | | | | | | | | | | | | | | | | | | | | | | | | | | | | | | | | | | | | | | |
| 91 |  |  | E |  | L |  | C |  | T |  | C |  | Q |  | Q |  | D |  | K |  | H |  |  | L |  | K |  | F |  | I |  | E |  | K |  | G |  | L |  | Q |  | K |  |  | A |  | K |  | V |  | L |  | V |  | A |  | G |  | | 117 |  | | | | | |

Fixed PTMs: Carbamidomethylation [C50 C57 C64 C65 C74 C76 ]

  

All peaks (72)  Matched peaks (34)  Not matched peaks (38)

  

| Scan | Peak | Mono mass | Mono m/z | Intensity | Charge | Theoretical mass | Ion | Pos | Mass error | PPM error |
| --- | --- | --- | --- | --- | --- | --- | --- | --- | --- | --- |
| 448 | 1 | 4312.7575 | 863.5588 | 250768.66 | 5 |  |  |  |  |  |
| 448 | 2 | 2185.3890 | 729.4703 | 413680.33 | 3 |  |  |  |  |  |
| 448 | 3 | 4312.7574 | 1079.1966 | 47478.91 | 4 |  |  |  |  |  |
| 448 | 4 | 3586.4933 | 897.6306 | 34412.97 | 4 | 3586.5162 | C28 | 28 | -0.0230 | -6.40 |
| 448 | 5 | 2293.9965 | 765.6728 | 40176.62 | 3 |  |  |  |  |  |
| 448 | 6 | 4353.7621 | 871.7597 | 30716.51 | 5 |  |  |  |  |  |
| 448 | 7 | 2462.9496 | 821.9905 | 31836.30 | 3 | 2462.9644 | C19 | 19 | -0.0148 | -6.03 |
| 448 | 8 | 3893.5899 | 974.4048 | 23695.07 | 4 | 3893.6153 | C30 | 30 | -0.0254 | -6.52 |
| 448 | 9 | 4240.7357 | 849.1544 | 29399.62 | 5 | 4240.7634 | C33 | 33 | -0.0278 | -6.55 |
| 448 | 10 | 1986.7907 | 994.4026 | 31104.61 | 2 | 1986.8020 | C16 | 16 | -0.0113 | -5.68 |
| 448 | 11 | 4061.7020 | 1016.4328 | 24183.19 | 4 |  |  |  |  |  |
| 448 | 12 | 3084.2762 | 772.0763 | 22764.76 | 4 | 3084.2953 | C24 | 24 | -0.0191 | -6.20 |
| 448 | 13 | 3746.5235 | 937.6382 | 19188.34 | 4 | 3746.5469 | C29 | 29 | -0.0234 | -6.24 |
| 448 | 14 | 4255.7364 | 1064.9414 | 17477.76 | 4 |  |  |  |  |  |
| 448 | 15 | 4221.7495 | 845.3572 | 16172.12 | 5 |  |  |  |  |  |
| 448 | 16 | 3458.3969 | 865.6065 | 21981.26 | 4 | 3458.4213 | C27 | 27 | -0.0244 | -7.07 |
| 448 | 17 | 2549.9811 | 851.0010 | 18886.06 | 3 | 2549.9965 | C20 | 20 | -0.0154 | -6.03 |
| 448 | 18 | 1491.5743 | 746.7944 | 21520.05 | 2 | 1491.5830 | C12 | 12 | -8.77e-03 | -5.88 |
| 448 | 19 | 4327.7596 | 866.5592 | 13788.69 | 5 |  |  |  |  |  |
| 448 | 20 | 4354.7744 | 1089.7009 | 13168.22 | 4 |  |  |  |  |  |
| 448 | 21 | 2879.2133 | 960.7450 | 12817.83 | 3 | 2879.2309 | Z\_DOT22 | 12 | -0.0176 | -6.10 |
| 448 | 22 | 874.1570 | 875.1643 | 35714.66 | 1 |  |  |  |  |  |
| 448 | 23 | 1907.8381 | 954.9263 | 15822.83 | 2 | 1907.8495 | Z\_DOT15 | 19 | -0.0114 | -5.97 |
| 448 | 24 | 3298.3699 | 825.5998 | 10593.15 | 4 | 3298.3906 | C26 | 26 | -0.0207 | -6.28 |
| 448 | 25 | 3199.3025 | 800.8329 | 10800.18 | 4 | 3199.3222 | C25 | 25 | -0.0197 | -6.16 |
| 448 | 26 | 4267.7388 | 1067.9420 | 11446.22 | 4 |  |  |  |  |  |
| 448 | 27 | 3621.4425 | 906.3679 | 9636.64 | 4 | 3621.4649 | Z\_DOT28 | 6 | -0.0225 | -6.20 |
| 448 | 28 | 4061.7004 | 813.3473 | 9423.49 | 5 |  |  |  |  |  |
| 448 | 29 | 2133.9658 | 712.3292 | 9409.87 | 3 |  |  |  |  |  |
| 448 | 30 | 4263.7612 | 853.7595 | 10339.41 | 5 |  |  |  |  |  |
| 448 | 31 | 4326.7740 | 1082.7008 | 6955.97 | 4 |  |  |  |  |  |
| 448 | 32 | 2185.3946 | 1093.7046 | 15624.97 | 2 |  |  |  |  |  |
| 448 | 33 | 2678.0749 | 893.6989 | 8031.47 | 3 | 2678.0914 | C21 | 21 | -0.0165 | -6.17 |
| 448 | 34 | 2895.2335 | 724.8156 | 9297.37 | 4 |  |  |  |  |  |
| 448 | 35 | 1820.8068 | 911.4107 | 9445.24 | 2 | 1820.8174 | Z\_DOT14 | 20 | -0.0106 | -5.83 |
| 448 | 36 | 4280.7883 | 857.1649 | 10424.66 | 5 |  |  |  |  |  |
| 448 | 37 | 1606.6005 | 804.3075 | 12124.99 | 2 | 1606.6100 | C13 | 13 | -9.47e-03 | -5.89 |
| 448 | 38 | 2764.1876 | 922.4031 | 8406.99 | 3 | 2764.2039 | Z\_DOT21 | 13 | -0.0164 | -5.93 |
| 448 | 39 | 729.2964 | 730.3037 | 63114.05 | 1 |  |  |  |  |  |
| 448 | 40 | 1376.5481 | 689.2813 | 8413.19 | 2 | 1376.5561 | C11 | 11 | -7.96e-03 | -5.78 |
| 448 | 41 | 4153.7047 | 831.7482 | 6206.86 | 5 | 4153.7314 | C32 | 32 | -0.0267 | -6.42 |
| 448 | 42 | 2306.8503 | 1154.4324 | 8190.62 | 2 | 2306.8633 | C18 | 18 | -0.0130 | -5.65 |
| 448 | 43 | 3507.3978 | 877.8567 | 5475.59 | 4 | 3507.4220 | Z\_DOT27 | 7 | -0.0242 | -6.89 |
| 448 | 44 | 3370.3475 | 1124.4564 | 5763.65 | 3 | 3370.3631 | Z\_DOT26 | 8 | -0.0156 | -4.63 |
| 448 | 45 | 4222.7486 | 1056.6944 | 9014.48 | 4 |  |  |  |  |  |
| 448 | 46 | 4240.7408 | 1061.1925 | 8567.60 | 4 | 4240.7634 | C33 | 33 | -0.0227 | -5.35 |
| 448 | 47 | 4351.7690 | 726.3021 | 6760.51 | 6 |  |  |  |  |  |
| 448 | 48 | 3165.3129 | 1056.1116 | 5814.93 | 3 |  |  |  |  |  |
| 448 | 49 | 3849.5731 | 963.4005 | 4328.98 | 4 |  |  |  |  |  |
| 448 | 50 | 4295.7439 | 860.1561 | 5850.35 | 5 |  |  |  |  |  |
| 448 | 51 | 3084.2744 | 1029.0987 | 6747.23 | 3 | 3084.2953 | C24 | 24 | -0.0209 | -6.77 |
| 448 | 52 | 1474.5470 | 738.2808 | 6809.43 | 2 |  |  |  |  |  |
| 448 | 53 | 1115.4716 | 558.7431 | 3507.69 | 2 | 1115.4778 | C9 | 9 | -6.16e-03 | -5.52 |
| 448 | 54 | 749.3447 | 750.3520 | 8055.33 | 1 | 749.3490 | C6 | 6 | -4.26e-03 | -5.69 |
| 448 | 55 | 1275.5011 | 638.7578 | 3211.64 | 2 | 1275.5084 | C10 | 10 | -7.28e-03 | -5.70 |
| 448 | 56 | 694.2926 | 695.2999 | 3496.68 | 1 |  |  |  |  |  |
| 448 | 57 | 1000.4452 | 501.2299 | 4231.58 | 2 | 1000.4508 | C8 | 8 | -5.59e-03 | -5.59 |
| 448 | 58 | 846.3557 | 847.3630 | 6779.29 | 1 |  |  |  |  |  |
| 448 | 59 | 330.1525 | 331.1598 | 3834.41 | 1 |  |  |  |  |  |
| 448 | 60 | 493.2149 | 494.2222 | 1703.73 | 1 |  |  |  |  |  |
| 448 | 61 | 1093.4480 | 1094.4553 | 4405.90 | 1 |  |  |  |  |  |
| 448 | 62 | 822.3864 | 823.3937 | 1762.47 | 1 |  |  |  |  |  |
| 448 | 63 | 1287.5173 | 1288.5246 | 2071.00 | 1 |  |  |  |  |  |
| 448 | 64 | 1171.4866 | 1172.4938 | 1387.42 | 1 | 1171.4917 | Z\_DOT9 | 25 | -5.13e-03 | -4.38 |
| 448 | 65 | 480.1617 | 481.1690 | 1061.02 | 1 | 480.1638 | C4 | 4 | -2.10e-03 | -4.38 |
| 448 | 66 | 712.0798 | 713.0870 | 1279.01 | 1 |  |  |  |  |  |
| 448 | 67 | 361.1258 | 362.1331 | 1047.34 | 1 |  |  |  |  |  |
| 448 | 68 | 593.2449 | 594.2522 | 1976.10 | 1 | 593.2479 | C5 | 5 | -2.94e-03 | -4.96 |
| 448 | 69 | 1115.4728 | 1116.4800 | 991.60 | 1 | 1115.4778 | C9 | 9 | -4.99e-03 | -4.47 |
| 448 | 70 | 1231.9775 | 1232.9848 | 756.58 | 1 |  |  |  |  |  |
| 448 | 71 | 1457.9333 | 1458.9406 | 733.32 | 1 |  |  |  |  |  |
| 448 | 72 | 983.4181 | 492.7163 | 708.64 | 2 |  |  |  |  |  |

  

All proteins /
CsTx-12a\_S1 Cupiennius salei toxin 12 isoform a S1^ACsTx-12a\_S2 Cupiennius salei toxin 12 isoform a S2 /
Proteoform #18
