# Supplementary material for: The Dual Prey-Inactivation Strategy of Spiders—In-Depth Venomic Analysis of Cupiennius salei
Source: Toxins (Basel). 2019 Mar 19;11(3):167. doi: 10.3390/toxins11030167 (PMC6468893; doi:10.3390/toxins11030167)
Supplement: Supplementary file 1 [file toxins-11-00167-s001.zip › Supplementary Dataset EV1/20180328_f2_topdown_OTMS2_EThcD_NL_i02_ms2_proteoform_cutoff_html/prsms/prsm105.html]

Protein-Spectrum-Match for Spectrum #335


All proteins /
CsTx-13a Cupiennius salei toxin 13 isoform a /
Proteoform #22

## Protein-Spectrum-Match #105 for Spectrum #335

|  |  |  |  |  |  |
| --- | --- | --- | --- | --- | --- |
| PrSM ID: | 105 | Scan(s): | 449 | Precursor charge: | 6 |
| Precursor m/z: | 724.7999 | Precursor mass: | 4342.7555 | Proteoform mass: | 4342.7588 |
| # matched peaks: | 23 | # matched fragment ions: | 23 | # unexpected modifications: | 0 |
| E-value: | 2.80e-24 | P-value: | 2.80e-24 | Q-value (Spectral FDR): | 0 |

  

|  |  |  |  |  |  |  |  |  |  |  |  |  |  |  |  |  |  |  |  |  |  |  |  |  |  |  |  |  |  |  |  |  |  |  |  |  |  |  |  |  |  |  |  |  |  |  |  |  |  |  |  |  |  |  |  |  |  |  |  |  |  |  |  |  |  |  |  |  |  |
| --- | --- | --- | --- | --- | --- | --- | --- | --- | --- | --- | --- | --- | --- | --- | --- | --- | --- | --- | --- | --- | --- | --- | --- | --- | --- | --- | --- | --- | --- | --- | --- | --- | --- | --- | --- | --- | --- | --- | --- | --- | --- | --- | --- | --- | --- | --- | --- | --- | --- | --- | --- | --- | --- | --- | --- | --- | --- | --- | --- | --- | --- | --- | --- | --- | --- | --- | --- | --- | --- |
|  | |  | | | | | | | | | | | | | | | | | | | | | | | | | | | | | | | | | | | | | | | | | | | | | | | | | | | | | | | | | | | | | | | | | | | |
| 1 |  |  | M |  | K |  | V |  | L |  | V |  | I |  | F |  | A |  | V |  | L |  |  | S |  | L |  | V |  | I |  | F |  | S |  | N |  | C |  | S |  | A |  |  | E |  | T |  | D |  | E |  | D |  | F |  | F |  | G |  | E |  | E |  | 30 |  |
|  | |  | | | | | | | | | | | | | | | | | | | | | | | | | | | | | | | | | | | | | | | | | | | | | | | | | | | | | | | | | | | | | | | | | | | |
| 31 |  |  | S |  | F |  | E |  | A |  | D |  | D |  | I |  | I |  | P |  | F |  |  | I |  | A |  | K |  | E |  | Q |  | V |  | R | ] | S |  | D |  | C |  |  | T |  | L |  | R | ⎱ | N |  | H | ⎫ | D | ⎫ | C |  | T | ⎫ | D | ⎱ | D |  | 60 |  |
|  | |  | | | | | | | | | | | | | | | | | | | | | | | | | | | | | | | | | | | | | | | | | | | | | | | | | | | | | | | | | | | | | | | | | | | |
| 61 |  | ⎫ | R |  | H |  | S | ⎫ | C |  | C | ⎫ | R | ⎫ | S | ⎫ | K | ⎫ | M | ⎫ | F |  | ⎫ | K | ⎱ | D |  | V | ⎫ | C |  | T | ⎫ | C | ⎫ | F | ⎫ | Y |  | P | ⎫ | S |  | ⎫ | Q | [ | R |  | S |  | E |  | T |  | A |  | R |  | A |  | K |  | K |  | 90 |  |
|  | |  | | | | | | | | | | | | | | | | | | | | | | | | | | | | | | | | | | | | | | | | | | | | | | | | | | | | | | | | | | | | | | | | | | | |
| 91 |  |  | E |  | L |  | C |  | T |  | C |  | Q |  | Q |  | P |  | K |  | H |  |  | L |  | K |  | Y |  | I |  | E |  | K |  | G |  | L |  | Q |  | K |  |  | A |  | K |  | D |  | Y |  | A |  | T |  | G |  | | 117 |  | | | | | |

Fixed PTMs: Carbamidomethylation [C50 C57 C64 C65 C74 C76 ]

  

All peaks (71)  Matched peaks (23)  Not matched peaks (48)

  

| Scan | Peak | Mono mass | Mono m/z | Intensity | Charge | Theoretical mass | Ion | Pos | Mass error | PPM error |
| --- | --- | --- | --- | --- | --- | --- | --- | --- | --- | --- |
| 449 | 1 | 1447.9101 | 724.9623 | 149351.61 | 2 |  |  |  |  |  |
| 449 | 2 | 4285.7076 | 858.1488 | 95284.63 | 5 |  |  |  |  |  |
| 449 | 3 | 4324.7211 | 721.7941 | 105432.47 | 6 |  |  |  |  |  |
| 449 | 4 | 4012.5787 | 803.5230 | 78222.35 | 5 |  |  |  |  |  |
| 449 | 5 | 4196.6619 | 840.3397 | 40788.64 | 5 |  |  |  |  |  |
| 449 | 6 | 4285.7100 | 1072.4348 | 27249.18 | 4 |  |  |  |  |  |
| 449 | 7 | 4326.7144 | 866.3502 | 22412.26 | 5 |  |  |  |  |  |
| 449 | 8 | 4034.6524 | 1009.6704 | 19922.43 | 4 |  |  |  |  |  |
| 449 | 9 | 4213.6867 | 843.7446 | 13815.98 | 5 | 4213.7162 | C33 | 33 | -0.0294 | -6.98 |
| 449 | 10 | 4327.7242 | 1082.9383 | 13261.67 | 4 |  |  |  |  |  |
| 449 | 11 | 3084.2762 | 772.0763 | 13939.95 | 4 | 3084.2953 | C24 | 24 | -0.0191 | -6.20 |
| 449 | 12 | 2172.3723 | 1087.1934 | 22922.26 | 2 |  |  |  |  |  |
| 449 | 13 | 4267.6943 | 854.5461 | 11352.65 | 5 |  |  |  |  |  |
| 449 | 14 | 2956.1812 | 740.0526 | 9442.92 | 4 | 2956.2003 | C23 | 23 | -0.0191 | -6.47 |
| 449 | 15 | 3866.5416 | 967.6427 | 10139.04 | 4 | 3866.5680 | C30 | 30 | -0.0265 | -6.84 |
| 449 | 16 | 4179.6471 | 836.9367 | 8102.41 | 5 |  |  |  |  |  |
| 449 | 17 | 2678.0743 | 893.6987 | 8549.98 | 3 | 2678.0914 | C21 | 21 | -0.0171 | -6.38 |
| 449 | 18 | 3559.4476 | 890.8692 | 8227.80 | 4 | 3559.4690 | C28 | 28 | -0.0213 | -6.00 |
| 449 | 19 | 2549.9785 | 851.0001 | 7677.09 | 3 | 2549.9965 | C20 | 20 | -0.0180 | -7.04 |
| 449 | 20 | 3719.4750 | 930.8760 | 7994.55 | 4 | 3719.4996 | C29 | 29 | -0.0247 | -6.63 |
| 449 | 21 | 4228.6866 | 1058.1789 | 7058.16 | 4 |  |  |  |  |  |
| 449 | 22 | 1737.8960 | 869.9553 | 41774.60 | 2 |  |  |  |  |  |
| 449 | 23 | 868.7476 | 869.7549 | 21045.04 | 1 |  |  |  |  |  |
| 449 | 24 | 4013.5814 | 1004.4026 | 8867.47 | 4 |  |  |  |  |  |
| 449 | 25 | 4306.7028 | 718.7911 | 6467.08 | 6 |  |  |  |  |  |
| 449 | 26 | 2809.1141 | 703.2858 | 8308.99 | 4 | 2809.1319 | C22 | 22 | -0.0179 | -6.36 |
| 449 | 27 | 2462.9485 | 821.9901 | 7176.81 | 3 | 2462.9644 | C19 | 19 | -0.0159 | -6.48 |
| 449 | 28 | 2896.1589 | 725.0470 | 55574.43 | 4 |  |  |  |  |  |
| 449 | 29 | 1376.5484 | 689.2815 | 6239.70 | 2 | 1376.5561 | C11 | 11 | -7.71e-03 | -5.60 |
| 449 | 30 | 3298.3699 | 825.5998 | 5572.73 | 4 | 3298.3906 | C26 | 26 | -0.0207 | -6.28 |
| 449 | 31 | 4252.7335 | 851.5540 | 7170.12 | 5 |  |  |  |  |  |
| 449 | 32 | 4240.6875 | 1061.1792 | 5209.35 | 4 |  |  |  |  |  |
| 449 | 33 | 4236.7174 | 848.3508 | 6630.05 | 5 |  |  |  |  |  |
| 449 | 34 | 1606.5990 | 804.3068 | 6198.31 | 2 | 1606.6100 | C13 | 13 | -0.0109 | -6.81 |
| 449 | 35 | 3594.3959 | 899.6062 | 4942.08 | 4 | 3594.4177 | Z\_DOT28 | 6 | -0.0218 | -6.06 |
| 449 | 36 | 4126.6578 | 1032.6717 | 5214.00 | 4 | 4126.6841 | C32 | 32 | -0.0264 | -6.39 |
| 449 | 37 | 2306.8487 | 769.9568 | 4631.05 | 3 | 2306.8633 | C18 | 18 | -0.0146 | -6.34 |
| 449 | 38 | 4109.6320 | 822.9337 | 5825.93 | 5 |  |  |  |  |  |
| 449 | 39 | 1986.7899 | 994.4022 | 6199.67 | 2 | 1986.8020 | C16 | 16 | -0.0121 | -6.11 |
| 449 | 40 | 1491.5735 | 746.7940 | 10249.26 | 2 | 1491.5830 | C12 | 12 | -9.51e-03 | -6.37 |
| 449 | 41 | 4195.6924 | 1049.9304 | 4897.51 | 4 |  |  |  |  |  |
| 449 | 42 | 4267.6978 | 1067.9317 | 4920.56 | 4 |  |  |  |  |  |
| 449 | 43 | 2852.1653 | 951.7291 | 5368.72 | 3 | 2852.1836 | Z\_DOT22 | 12 | -0.0183 | -6.40 |
| 449 | 44 | 3138.2653 | 1047.0957 | 4524.72 | 3 |  |  |  |  |  |
| 449 | 45 | 3344.2975 | 1115.7731 | 4069.07 | 3 |  |  |  |  |  |
| 449 | 46 | 2266.9506 | 756.6575 | 5337.32 | 3 |  |  |  |  |  |
| 449 | 47 | 3984.5837 | 797.9240 | 3517.16 | 5 |  |  |  |  |  |
| 449 | 48 | 4341.7255 | 869.3524 | 22498.04 | 5 |  |  |  |  |  |
| 449 | 49 | 4034.6481 | 807.9369 | 4406.19 | 5 |  |  |  |  |  |
| 449 | 50 | 3445.5817 | 690.1236 | 2881.55 | 5 |  |  |  |  |  |
| 449 | 51 | 330.1525 | 331.1598 | 7570.74 | 1 |  |  |  |  |  |
| 449 | 52 | 4300.7242 | 861.1521 | 8157.62 | 5 |  |  |  |  |  |
| 449 | 53 | 1000.4449 | 501.2297 | 4552.30 | 2 | 1000.4508 | C8 | 8 | -5.89e-03 | -5.89 |
| 449 | 54 | 1474.5489 | 738.2817 | 2495.76 | 2 |  |  |  |  |  |
| 449 | 55 | 749.3448 | 750.3521 | 5914.97 | 1 | 749.3490 | C6 | 6 | -4.14e-03 | -5.52 |
| 449 | 56 | 1086.9369 | 1087.9442 | 13487.23 | 1 |  |  |  |  |  |
| 449 | 57 | 1115.4712 | 558.7429 | 3165.13 | 2 | 1115.4778 | C9 | 9 | -6.53e-03 | -5.85 |
| 449 | 58 | 1447.9094 | 1448.9167 | 2973.11 | 1 |  |  |  |  |  |
| 449 | 59 | 1017.1641 | 1018.1714 | 989.60 | 1 |  |  |  |  |  |
| 449 | 60 | 983.4188 | 492.7167 | 741.40 | 2 |  |  |  |  |  |
| 449 | 61 | 493.2151 | 494.2224 | 879.55 | 1 |  |  |  |  |  |
| 449 | 62 | 1190.4846 | 1191.4919 | 733.07 | 1 |  |  |  |  |  |
| 449 | 63 | 1258.5362 | 630.2754 | 719.73 | 2 |  |  |  |  |  |
| 449 | 64 | 1410.1101 | 706.0623 | 3739.07 | 2 |  |  |  |  |  |
| 449 | 65 | 1222.4944 | 1223.5016 | 673.46 | 1 |  |  |  |  |  |
| 449 | 66 | 1275.9923 | 639.0034 | 442.58 | 2 |  |  |  |  |  |
| 449 | 67 | 686.7894 | 687.7966 | 840.33 | 1 |  |  |  |  |  |
| 449 | 68 | 1425.5900 | 713.8023 | 701.46 | 2 |  |  |  |  |  |
| 449 | 69 | 1388.5643 | 1389.5716 | 637.18 | 1 |  |  |  |  |  |
| 449 | 70 | 1259.4636 | 1260.4709 | 566.91 | 1 | 1259.4713 | Z\_DOT10 | 24 | -7.74e-03 | -6.14 |
| 449 | 71 | 772.5714 | 773.5787 | 845.90 | 1 |  |  |  |  |  |

  

All proteins /
CsTx-13a Cupiennius salei toxin 13 isoform a /
Proteoform #22
