# Supplementary material for: The Dual Prey-Inactivation Strategy of Spiders—In-Depth Venomic Analysis of Cupiennius salei
Source: Toxins (Basel). 2019 Mar 19;11(3):167. doi: 10.3390/toxins11030167 (PMC6468893; doi:10.3390/toxins11030167)
Supplement: Supplementary file 1 [file toxins-11-00167-s001.zip › Supplementary Dataset EV1/20180328_f2_topdown_OTMS2_EThcD_NL_i02_ms2_proteoform_cutoff_html/prsms/prsm106.html]

Protein-Spectrum-Match for Spectrum #337


All proteins /
CsTx-1b Cupiennius salei toxin 1 isoform b /
Proteoform #92

## Protein-Spectrum-Match #106 for Spectrum #337

|  |  |  |  |  |  |
| --- | --- | --- | --- | --- | --- |
| PrSM ID: | 106 | Scan(s): | 452 | Precursor charge: | 10 |
| Precursor m/z: | 739.1383 | Precursor mass: | 7381.3103 | Proteoform mass: | 7382.3082 |
| # matched peaks: | 12 | # matched fragment ions: | 10 | # unexpected modifications: | 0 |
| E-value: | 2.68e-11 | P-value: | 2.68e-11 | Q-value (Spectral FDR): | 0 |

  

|  |  |  |  |  |  |  |  |  |  |  |  |  |  |  |  |  |  |  |  |  |  |  |  |  |  |  |  |  |  |  |  |  |  |  |  |  |  |  |  |  |  |  |  |  |  |  |  |  |  |  |  |  |  |  |  |  |  |  |  |  |  |  |  |  |  |  |  |  |  |
| --- | --- | --- | --- | --- | --- | --- | --- | --- | --- | --- | --- | --- | --- | --- | --- | --- | --- | --- | --- | --- | --- | --- | --- | --- | --- | --- | --- | --- | --- | --- | --- | --- | --- | --- | --- | --- | --- | --- | --- | --- | --- | --- | --- | --- | --- | --- | --- | --- | --- | --- | --- | --- | --- | --- | --- | --- | --- | --- | --- | --- | --- | --- | --- | --- | --- | --- | --- | --- | --- |
|  | |  | | | | | | | | | | | | | | | | | | | | | | | | | | | | | | | | | | | | | | | | | | | | | | | | | | | | | | | | | | | | | | | | | | | |
| 1 |  |  | M |  | K |  | V |  | L |  | I |  | I |  | S |  | A |  | V |  | L |  |  | F |  | I |  | T |  | I |  | F |  | S |  | N |  | I |  | S |  | A |  |  | E |  | I |  | E |  | D |  | D |  | F |  | L |  | E |  | D |  | E |  | 30 |  |
|  | |  | | | | | | | | | | | | | | | | | | | | | | | | | | | | | | | | | | | | | | | | | | | | | | | | | | | | | | | | | | | | | | | | | | | |
| 31 |  |  | S |  | F |  | E |  | A |  | E |  | D |  | I |  | I |  | P |  | F |  |  | L |  | E |  | N |  | E |  | Q |  | A |  | R | ] | S | ⎩ | C |  | I |  |  | P |  | K |  | H |  | E | ⎫ | E |  | C |  | T | ⎫ | N | ⎫ | D |  | K |  | 60 |  |
|  | |  | | | | | | | | | | | | | | | | | | | | | | | | | | | | | | | | | | | | | | | | | | | | | | | | | | | | | | | | | | | | | | | | | | | |
| 61 |  |  | H | ⎫ | N | ⎫ | C |  | C |  | R |  | K |  | G |  | L |  | F | ⎫ | K |  |  | L |  | K | ⎫ | C |  | Q |  | C |  | S |  | T |  | F |  | D |  | D |  |  | E |  | S |  | G |  | Q |  | P |  | T |  | E |  | R |  | C |  | A |  | 90 |  |
|  | |  | | | | | | | | | | | | | | | | | | | | | | | | | | | | | | | | | | | | | | | | | | | | | | | | | | | | | | | | | | | | | | | | | | | |
| 91 |  |  | C |  | G |  | R |  | P |  | M |  | G |  | H |  | Q |  | A |  | I |  |  | E |  | T |  | G |  | L | ⎫ | N |  | I |  | F |  | R | ⎫ | G | [ | L |  |  | F |  | K |  | G |  | K |  | K |  | K |  | N |  | K |  | K |  | T |  | 120 |  |
|  | |  | | | | | | | | | | | | | | | | | | | | | | | | | | | | | | | | | | | | | | | | | | | | | | | | | | | | | | | | | | | | | | | | | | | |
| 121 |  |  | K |  | G |  | | | | 122 |  | | | | | | | | | | | | | | | | | | | | | | | | | | | | | | | | | | | | | | | | | | | | | | | | | | | | | | | |

Fixed PTMs: Carbamidomethylation [C49 C56 C63 C64 C73 C75 C89 C91 ]

  

All peaks (74)  Matched peaks (12)  Not matched peaks (62)

  

| Scan | Peak | Mono mass | Mono m/z | Intensity | Charge | Theoretical mass | Ion | Pos | Mass error | PPM error |
| --- | --- | --- | --- | --- | --- | --- | --- | --- | --- | --- |
| 452 | 1 | 3684.8370 | 737.9747 | 86739.71 | 5 |  |  |  |  |  |
| 452 | 2 | 4321.7734 | 721.3028 | 50825.39 | 6 |  |  |  |  |  |
| 452 | 3 | 4369.7750 | 874.9623 | 20337.96 | 5 |  |  |  |  |  |
| 452 | 4 | 4426.7926 | 738.8060 | 34844.58 | 6 |  |  |  |  |  |
| 452 | 5 | 3627.8131 | 907.9605 | 13891.59 | 4 |  |  |  |  |  |
| 452 | 6 | 4264.7509 | 853.9575 | 13678.13 | 5 |  |  |  |  |  |
| 452 | 7 | 3685.8407 | 922.4674 | 13306.27 | 4 |  |  |  |  |  |
| 452 | 8 | 4322.7794 | 865.5632 | 9914.25 | 5 |  |  |  |  |  |
| 452 | 9 | 7267.2332 | 1039.1834 | 5983.04 | 7 |  |  |  |  |  |
| 452 | 10 | 7324.2495 | 916.5385 | 4952.87 | 8 | 7324.3027 | C61 | 61 | -0.0532 | -7.27 |
| 452 | 11 | 4427.8003 | 886.5673 | 6136.57 | 5 |  |  |  |  |  |
| 452 | 12 | 7267.2317 | 909.4112 | 5061.05 | 8 |  |  |  |  |  |
| 452 | 13 | 7278.2606 | 910.7898 | 4565.06 | 8 | 7279.2575 | Z\_DOT61 | 1 | 5.42e-03 | 0.74 |
| 452 | 14 | 738.1179 | 739.1252 | 5160.43 | 1 |  |  |  |  |  |
| 452 | 15 | 4369.7847 | 1093.4535 | 2705.99 | 4 |  |  |  |  |  |
| 452 | 16 | 1625.8677 | 813.9411 | 4044.98 | 2 |  |  |  |  |  |
| 452 | 17 | 7325.2496 | 814.9239 | 3497.21 | 9 | 7324.3027 | C61 | 61 | -0.0554 | -7.57 |
| 452 | 18 | 1752.7558 | 877.3852 | 2642.31 | 2 | 1752.7671 | C14 | 14 | -0.0114 | -6.48 |
| 452 | 19 | 7220.2187 | 903.5346 | 3985.22 | 8 |  |  |  |  |  |
| 452 | 20 | 7275.2550 | 809.3689 | 2783.25 | 9 |  |  |  |  |  |
| 452 | 21 | 7220.2218 | 803.2541 | 2467.37 | 9 |  |  |  |  |  |
| 452 | 22 | 3640.8251 | 911.2136 | 2165.61 | 4 |  |  |  |  |  |
| 452 | 23 | 7325.2421 | 1047.4704 | 3264.97 | 7 | 7324.3027 | C61 | 61 | -0.0629 | -8.59 |
| 452 | 24 | 7311.2421 | 1045.4704 | 2010.84 | 7 |  |  |  |  |  |
| 452 | 25 | 4410.7782 | 883.1629 | 1582.71 | 5 |  |  |  |  |  |
| 452 | 26 | 5523.2191 | 921.5438 | 4970.03 | 6 |  |  |  |  |  |
| 452 | 27 | 7220.2302 | 1032.4687 | 2337.92 | 7 |  |  |  |  |  |
| 452 | 28 | 4323.7799 | 1081.9523 | 2574.26 | 4 |  |  |  |  |  |
| 452 | 29 | 3514.7586 | 879.6969 | 1716.42 | 4 |  |  |  |  |  |
| 452 | 30 | 2788.2214 | 930.4144 | 2123.01 | 3 | 2788.2414 | C22 | 22 | -0.0199 | -7.15 |
| 452 | 31 | 1491.5729 | 746.7937 | 1810.16 | 2 |  |  |  |  |  |
| 452 | 32 | 1947.9055 | 974.9600 | 2253.95 | 2 |  |  |  |  |  |
| 452 | 33 | 3349.6920 | 838.4303 | 3006.27 | 4 |  |  |  |  |  |
| 452 | 34 | 4264.7554 | 1067.1961 | 2007.89 | 4 |  |  |  |  |  |
| 452 | 35 | 721.0921 | 722.0994 | 2599.40 | 1 |  |  |  |  |  |
| 452 | 36 | 2075.9970 | 1039.0058 | 1597.12 | 2 |  |  |  |  |  |
| 452 | 37 | 1609.8506 | 805.9326 | 1383.01 | 2 |  |  |  |  |  |
| 452 | 38 | 1866.7971 | 934.4058 | 1911.64 | 2 | 1866.8101 | C15 | 15 | -0.0130 | -6.95 |
| 452 | 39 | 1986.7893 | 994.4019 | 1286.86 | 2 |  |  |  |  |  |
| 452 | 40 | 4444.9123 | 889.9897 | 1856.99 | 5 |  |  |  |  |  |
| 452 | 41 | 5164.3489 | 738.7714 | 2116.34 | 7 |  |  |  |  |  |
| 452 | 42 | 4191.7335 | 839.3540 | 1478.53 | 5 |  |  |  |  |  |
| 452 | 43 | 2915.3178 | 729.8367 | 1373.46 | 4 |  |  |  |  |  |
| 452 | 44 | 1372.5778 | 687.2962 | 1537.29 | 2 | 1372.5863 | C11 | 11 | -8.52e-03 | -6.21 |
| 452 | 45 | 2775.3272 | 694.8391 | 1465.65 | 4 |  |  |  |  |  |
| 452 | 46 | 3035.2672 | 759.8241 | 1507.11 | 4 |  |  |  |  |  |
| 452 | 47 | 2217.2426 | 740.0881 | 5270.49 | 3 |  |  |  |  |  |
| 452 | 48 | 3634.1245 | 1212.3821 | 1139.70 | 3 |  |  |  |  |  |
| 452 | 49 | 3233.6591 | 1078.8937 | 1414.84 | 3 |  |  |  |  |  |
| 452 | 50 | 3636.7340 | 728.3541 | 1002.90 | 5 |  |  |  |  |  |
| 452 | 51 | 2677.0720 | 893.3646 | 1390.33 | 3 |  |  |  |  |  |
| 452 | 52 | 868.4189 | 869.4261 | 1210.99 | 1 | 868.4225 | C7 | 7 | -3.59e-03 | -4.14 |
| 452 | 53 | 4278.7659 | 856.7605 | 1356.54 | 5 |  |  |  |  |  |
| 452 | 54 | 6793.9621 | 971.5733 | 2396.46 | 7 | 6794.0062 | C57 | 57 | -0.0440 | -6.48 |
| 452 | 55 | 6640.4717 | 738.8375 | 2462.17 | 9 |  |  |  |  |  |
| 452 | 56 | 4303.7551 | 718.2998 | 842.39 | 6 |  |  |  |  |  |
| 452 | 57 | 2908.4359 | 970.4859 | 876.60 | 3 |  |  |  |  |  |
| 452 | 58 | 1737.9427 | 869.9786 | 2016.67 | 2 |  |  |  |  |  |
| 452 | 59 | 3698.5167 | 925.6364 | 978.97 | 4 |  |  |  |  |  |
| 452 | 60 | 2155.3820 | 1078.6983 | 890.91 | 2 |  |  |  |  |  |
| 452 | 61 | 4769.0648 | 954.8202 | 1196.19 | 5 |  |  |  |  |  |
| 452 | 62 | 764.6654 | 765.6727 | 689.56 | 1 |  |  |  |  |  |
| 452 | 63 | 3844.5871 | 962.1540 | 1084.88 | 4 |  |  |  |  |  |
| 452 | 64 | 1115.6145 | 1116.6218 | 581.77 | 1 |  |  |  |  |  |
| 452 | 65 | 1416.6784 | 709.3465 | 529.36 | 2 |  |  |  |  |  |
| 452 | 66 | 950.3807 | 951.3880 | 747.85 | 1 |  |  |  |  |  |
| 452 | 67 | 4304.7576 | 861.9588 | 1458.94 | 5 |  |  |  |  |  |
| 452 | 68 | 1258.5370 | 1259.5442 | 506.58 | 1 | 1258.5434 | C10 | 10 | -6.41e-03 | -5.10 |
| 452 | 69 | 4011.6938 | 803.3460 | 1256.52 | 5 |  |  |  |  |  |
| 452 | 70 | 3687.8527 | 1230.2915 | 1131.37 | 3 |  |  |  |  |  |
| 452 | 71 | 1394.6133 | 698.3139 | 539.57 | 2 |  |  |  |  |  |
| 452 | 72 | 1434.9214 | 718.4680 | 404.94 | 2 |  |  |  |  |  |
| 452 | 73 | 3157.4958 | 790.3812 | 1674.18 | 4 | 3157.5153 | C25 | 25 | -0.0195 | -6.19 |
| 452 | 74 | 4379.6589 | 730.9504 | 1290.68 | 6 |  |  |  |  |  |

  

All proteins /
CsTx-1b Cupiennius salei toxin 1 isoform b /
Proteoform #92
