# Supplementary material for: The Dual Prey-Inactivation Strategy of Spiders—In-Depth Venomic Analysis of Cupiennius salei
Source: Toxins (Basel). 2019 Mar 19;11(3):167. doi: 10.3390/toxins11030167 (PMC6468893; doi:10.3390/toxins11030167)
Supplement: Supplementary file 1 [file toxins-11-00167-s001.zip › Supplementary Dataset EV1/20180328_f2_topdown_OTMS2_EThcD_NL_i02_ms2_proteoform_cutoff_html/prsms/prsm107.html]

Protein-Spectrum-Match for Spectrum #338


All proteins /
CsTx-9c\_S1 Cupiennius salei toxin 9 isoform c S1^ACsTx-9c\_S2 Cupiennius salei toxin 9 isoform c S2 /
Proteoform #43

## Protein-Spectrum-Match #107 for Spectrum #338

|  |  |  |  |  |  |
| --- | --- | --- | --- | --- | --- |
| PrSM ID: | 107 | Scan(s): | 453 | Precursor charge: | 13 |
| Precursor m/z: | 598.9174 | Precursor mass: | 7772.8319 | Proteoform mass: | 7773.8311 |
| # matched peaks: | 22 | # matched fragment ions: | 21 | # unexpected modifications: | 0 |
| E-value: | 3.40e-22 | P-value: | 3.40e-22 | Q-value (Spectral FDR): | 0 |

  

|  |  |  |  |  |  |  |  |  |  |  |  |  |  |  |  |  |  |  |  |  |  |  |  |  |  |  |  |  |  |  |  |  |  |  |  |  |  |  |  |  |  |  |  |  |  |  |  |  |  |  |  |  |  |  |  |  |  |  |  |  |  |  |  |  |  |  |  |  |  |
| --- | --- | --- | --- | --- | --- | --- | --- | --- | --- | --- | --- | --- | --- | --- | --- | --- | --- | --- | --- | --- | --- | --- | --- | --- | --- | --- | --- | --- | --- | --- | --- | --- | --- | --- | --- | --- | --- | --- | --- | --- | --- | --- | --- | --- | --- | --- | --- | --- | --- | --- | --- | --- | --- | --- | --- | --- | --- | --- | --- | --- | --- | --- | --- | --- | --- | --- | --- | --- | --- |
|  | |  | | | | | | | | | | | | | | | | | | | | | | | | | | | | | | | | | | | | | | | | | | | | | | | | | | | | | | | | | | | | | | | | | | | |
| 1 |  |  | M |  | K |  | V |  | L |  | V |  | I |  | C |  | A |  | V |  | L |  |  | F |  | L |  | A |  | I |  | F |  | S |  | N |  | S |  | S |  | A |  |  | E |  | T |  | E |  | D |  | D |  | F |  | L |  | E |  | D |  | E |  | 30 |  |
|  | |  | | | | | | | | | | | | | | | | | | | | | | | | | | | | | | | | | | | | | | | | | | | | | | | | | | | | | | | | | | | | | | | | | | | |
| 31 |  |  | S |  | F |  | Q |  | A |  | D |  | D |  | V |  | I |  | P |  | F |  |  | L |  | A |  | S |  | E |  | Q |  | V |  | R | ] | K | ⎩ | D |  | D |  |  | K |  | N | ⎫ | C |  | I |  | P |  | K |  | H |  | H | ⎫ | E | ⎫ | C |  | 60 |  |
|  | |  | | | | | | | | | | | | | | | | | | | | | | | | | | | | | | | | | | | | | | | | | | | | | | | | | | | | | | | | | | | | | | | | | | | |
| 61 |  |  | T | ⎫ | N | ⎫ | D | ⎫ | K |  | K | ⎫ | N | ⎫ | C | ⎫ | C |  | K |  | K |  | ⎱ | G | ⎫ | L | ⎫ | L |  | K |  | L |  | K | ⎫ | C | ⎩ | Q |  | C |  | F |  |  | T |  | V |  | A |  | D | ⎫ | E | ⎱ | K |  | G |  | T |  | P |  | S |  | 90 |  |
|  | |  | | | | | | | | | | | | | | | | | | | | | | | | | | | | | | | | | | | | | | | | | | | | | | | | | | | | | | | | | | | | | | | | | | | |
| 91 |  | ⎩ | E |  | R |  | C |  | A |  | C |  | G | ⎩ | R |  | P |  | L |  | L |  |  | H |  | K |  | I |  | A |  | Y |  | T |  | G |  | T |  | K |  | M |  |  | I |  | K | [ | G |  | L |  | L |  | | 115 |  | | | | | | | | | |

Fixed PTMs: Carbamidomethylation [C53 C60 C67 C68 C77 C79 C93 C95 ]

  

All peaks (80)  Matched peaks (22)  Not matched peaks (58)

  

| Scan | Peak | Mono mass | Mono m/z | Intensity | Charge | Theoretical mass | Ion | Pos | Mass error | PPM error |
| --- | --- | --- | --- | --- | --- | --- | --- | --- | --- | --- |
| 453 | 1 | 1197.6907 | 599.8526 | 66582.55 | 2 |  |  |  |  |  |
| 453 | 2 | 597.2516 | 598.2588 | 52463.84 | 1 |  |  |  |  |  |
| 453 | 3 | 3579.8855 | 597.6549 | 19303.97 | 6 |  |  |  |  |  |
| 453 | 4 | 3580.8847 | 717.1842 | 5554.33 | 5 |  |  |  |  |  |
| 453 | 5 | 3565.8670 | 714.1807 | 3727.53 | 5 |  |  |  |  |  |
| 453 | 6 | 1794.9351 | 599.3190 | 5621.99 | 3 |  |  |  |  |  |
| 453 | 7 | 3522.8616 | 705.5796 | 3453.83 | 5 |  |  |  |  |  |
| 453 | 8 | 2955.3492 | 592.0771 | 5968.14 | 5 | 2955.3684 | C23 | 23 | -0.0191 | -6.48 |
| 453 | 9 | 3581.8983 | 896.4819 | 5372.95 | 4 |  |  |  |  |  |
| 453 | 10 | 2008.8711 | 670.6310 | 3798.44 | 3 | 2008.8843 | C16 | 16 | -0.0132 | -6.56 |
| 453 | 11 | 2379.1014 | 794.0411 | 2992.99 | 3 | 2379.1171 | C19 | 19 | -0.0157 | -6.62 |
| 453 | 12 | 7716.7897 | 858.4284 | 3086.23 | 9 |  |  |  |  |  |
| 453 | 13 | 7715.7871 | 772.5860 | 2371.97 | 10 |  |  |  |  |  |
| 453 | 14 | 1893.8441 | 947.9293 | 2127.24 | 2 | 1893.8573 | C15 | 15 | -0.0133 | -7.00 |
| 453 | 15 | 3125.4542 | 626.0981 | 1635.39 | 5 | 3125.4739 | C25 | 25 | -0.0197 | -6.30 |
| 453 | 16 | 1198.6977 | 1199.7050 | 3777.61 | 1 |  |  |  |  |  |
| 453 | 17 | 3459.8026 | 865.9579 | 3269.76 | 4 |  |  |  |  |  |
| 453 | 18 | 2265.0593 | 756.0270 | 2043.12 | 3 | 2265.0742 | C18 | 18 | -0.0149 | -6.59 |
| 453 | 19 | 1136.5827 | 569.2986 | 1572.83 | 2 |  |  |  |  |  |
| 453 | 20 | 1779.8036 | 890.9091 | 2693.80 | 2 | 1779.8144 | C14 | 14 | -0.0109 | -6.10 |
| 453 | 21 | 1892.8423 | 631.9547 | 2860.29 | 3 |  |  |  |  |  |
| 453 | 22 | 5986.7968 | 599.6870 | 7952.74 | 10 |  |  |  |  |  |
| 453 | 23 | 3607.8091 | 722.5691 | 1874.48 | 5 | 3607.8319 | C29 | 29 | -0.0228 | -6.32 |
| 453 | 24 | 4762.3871 | 681.3483 | 1837.47 | 7 |  |  |  |  |  |
| 453 | 25 | 4588.1642 | 656.4593 | 1741.93 | 7 |  |  |  |  |  |
| 453 | 26 | 3369.6959 | 674.9465 | 2092.61 | 5 |  |  |  |  |  |
| 453 | 27 | 4819.4689 | 804.2521 | 2332.83 | 6 | 4819.4706 | Z\_DOT42 | 23 | -1.72e-03 | -0.36 |
| 453 | 28 | 522.2199 | 523.2272 | 2480.68 | 1 |  |  |  |  |  |
| 453 | 29 | 2954.3446 | 739.5934 | 2143.60 | 4 |  |  |  |  |  |
| 453 | 30 | 2870.4778 | 718.6267 | 1806.83 | 4 |  |  |  |  |  |
| 453 | 31 | 7184.5250 | 599.7177 | 3274.39 | 12 |  |  |  |  |  |
| 453 | 32 | 3012.3725 | 603.4818 | 1692.94 | 5 | 3012.3898 | C24 | 24 | -0.0173 | -5.74 |
| 453 | 33 | 3056.5993 | 765.1571 | 1339.90 | 4 | 3056.5960 | Z\_DOT27 | 38 | 3.23e-03 | 1.06 |
| 453 | 34 | 3459.7943 | 692.9661 | 2182.35 | 5 |  |  |  |  |  |
| 453 | 35 | 1389.6842 | 695.8494 | 2084.27 | 2 | 1389.6935 | C11 | 11 | -9.24e-03 | -6.65 |
| 453 | 36 | 1485.8532 | 743.9339 | 1175.12 | 2 |  |  |  |  |  |
| 453 | 37 | 1592.8206 | 797.4176 | 1274.82 | 2 |  |  |  |  |  |
| 453 | 38 | 1852.0905 | 618.3708 | 1273.24 | 3 |  |  |  |  |  |
| 453 | 39 | 1853.0935 | 927.5540 | 1393.58 | 2 | 1853.0836 | Z\_DOT16 | 49 | 9.92e-03 | 5.36 |
| 453 | 40 | 3596.8864 | 600.4883 | 1661.56 | 6 |  |  |  |  |  |
| 453 | 41 | 7172.7400 | 598.7356 | 2652.39 | 12 |  |  |  |  |  |
| 453 | 42 | 1625.8702 | 813.9424 | 1323.76 | 2 |  |  |  |  |  |
| 453 | 43 | 4589.1698 | 765.8689 | 1413.75 | 6 | 4589.2004 | C37 | 37 | -0.0305 | -6.66 |
| 453 | 44 | 2586.3508 | 863.1242 | 986.75 | 3 | 2586.3471 | Z\_DOT22 | 43 | 3.65e-03 | 1.41 |
| 453 | 45 | 1609.8485 | 805.9315 | 1216.96 | 2 |  |  |  |  |  |
| 453 | 46 | 1442.7981 | 722.4063 | 1272.61 | 2 |  |  |  |  |  |
| 453 | 47 | 3534.8764 | 884.7264 | 943.57 | 4 |  |  |  |  |  |
| 453 | 48 | 4076.9985 | 680.5070 | 1364.49 | 6 |  |  |  |  |  |
| 453 | 49 | 2854.4615 | 714.6226 | 1055.98 | 4 |  |  |  |  |  |
| 453 | 50 | 4718.2090 | 787.3754 | 1244.90 | 6 | 4718.2429 | C38 | 38 | -0.0340 | -7.20 |
| 453 | 51 | 2116.1810 | 706.4009 | 1208.27 | 3 |  |  |  |  |  |
| 453 | 52 | 7630.6994 | 954.8447 | 811.26 | 8 | 7629.7175 | Z\_DOT64 | 1 | -0.0205 | -2.68 |
| 453 | 53 | 4718.2136 | 675.0378 | 1001.77 | 7 | 4718.2429 | C38 | 38 | -0.0293 | -6.21 |
| 453 | 54 | 3129.7056 | 783.4337 | 1165.77 | 4 |  |  |  |  |  |
| 453 | 55 | 4006.9680 | 802.4009 | 819.88 | 5 | 4006.9764 | Z\_DOT35 | 30 | -8.39e-03 | -2.09 |
| 453 | 56 | 564.0603 | 565.0676 | 1627.55 | 1 |  |  |  |  |  |
| 453 | 57 | 1181.6730 | 1182.6802 | 749.43 | 1 |  |  |  |  |  |
| 453 | 58 | 1464.7129 | 733.3637 | 649.54 | 2 |  |  |  |  |  |
| 453 | 59 | 7673.7779 | 853.6493 | 1431.17 | 9 |  |  |  |  |  |
| 453 | 60 | 3765.8298 | 942.4647 | 799.03 | 4 |  |  |  |  |  |
| 453 | 61 | 1011.5691 | 1012.5764 | 812.27 | 1 |  |  |  |  |  |
| 453 | 62 | 7658.7567 | 766.8829 | 997.51 | 10 |  |  |  |  |  |
| 453 | 63 | 4074.9927 | 816.0058 | 1623.88 | 5 |  |  |  |  |  |
| 453 | 64 | 617.3098 | 618.3171 | 1629.86 | 1 | 617.3132 | C5 | 5 | -3.43e-03 | -5.55 |
| 453 | 65 | 1139.6515 | 1140.6588 | 658.61 | 1 |  |  |  |  |  |
| 453 | 66 | 3245.7388 | 812.4420 | 1224.87 | 4 |  |  |  |  |  |
| 453 | 67 | 3370.7030 | 843.6830 | 955.91 | 4 |  |  |  |  |  |
| 453 | 68 | 1220.6813 | 611.3479 | 525.37 | 2 |  |  |  |  |  |
| 453 | 69 | 3896.9000 | 650.4906 | 928.58 | 6 |  |  |  |  |  |
| 453 | 70 | 1928.9477 | 965.4811 | 947.32 | 2 |  |  |  |  |  |
| 453 | 71 | 2324.6784 | 775.9001 | 787.52 | 3 |  |  |  |  |  |
| 453 | 72 | 1263.1675 | 632.5910 | 623.47 | 2 |  |  |  |  |  |
| 453 | 73 | 2539.1308 | 847.3842 | 786.93 | 3 | 2539.1478 | C20 | 20 | -0.0170 | -6.70 |
| 453 | 74 | 580.0928 | 581.1000 | 1736.54 | 1 |  |  |  |  |  |
| 453 | 75 | 852.2007 | 853.2079 | 499.44 | 1 |  |  |  |  |  |
| 453 | 76 | 1372.7691 | 687.3918 | 1641.24 | 2 |  |  |  |  |  |
| 453 | 77 | 1977.1367 | 660.0528 | 1222.90 | 3 |  |  |  |  |  |
| 453 | 78 | 896.4765 | 897.4838 | 694.65 | 1 |  |  |  |  |  |
| 453 | 79 | 1413.7941 | 707.9043 | 608.95 | 2 |  |  |  |  |  |
| 453 | 80 | 1518.7430 | 760.3788 | 2125.31 | 2 | 1518.7361 | C12 | 12 | 6.91e-03 | 4.55 |

  

All proteins /
CsTx-9c\_S1 Cupiennius salei toxin 9 isoform c S1^ACsTx-9c\_S2 Cupiennius salei toxin 9 isoform c S2 /
Proteoform #43
