# Supplementary material for: The Dual Prey-Inactivation Strategy of Spiders—In-Depth Venomic Analysis of Cupiennius salei
Source: Toxins (Basel). 2019 Mar 19;11(3):167. doi: 10.3390/toxins11030167 (PMC6468893; doi:10.3390/toxins11030167)
Supplement: Supplementary file 1 [file toxins-11-00167-s001.zip › Supplementary Dataset EV1/20180328_f2_topdown_OTMS2_EThcD_NL_i02_ms2_proteoform_cutoff_html/prsms/prsm108.html]

Protein-Spectrum-Match for Spectrum #339


All proteins /
CsTx-13a Cupiennius salei toxin 13 isoform a /
Proteoform #40

## Protein-Spectrum-Match #108 for Spectrum #339

|  |  |  |  |  |  |
| --- | --- | --- | --- | --- | --- |
| PrSM ID: | 108 | Scan(s): | 455 | Precursor charge: | 6 |
| Precursor m/z: | 580.3139 | Precursor mass: | 3475.8398 | Proteoform mass: | 3475.8348 |
| # matched peaks: | 31 | # matched fragment ions: | 25 | # unexpected modifications: | 1 |
| E-value: | 5.30e-20 | P-value: | 5.30e-20 | Q-value (Spectral FDR): | 0 |

  

|  |  |  |  |  |  |  |  |  |  |  |  |  |  |  |  |  |  |  |  |  |  |  |  |  |  |  |  |  |  |  |  |  |  |  |  |  |  |  |  |  |  |  |  |  |  |  |  |  |  |  |  |  |  |  |  |  |  |  |  |  |  |  |  |  |  |  |
| --- | --- | --- | --- | --- | --- | --- | --- | --- | --- | --- | --- | --- | --- | --- | --- | --- | --- | --- | --- | --- | --- | --- | --- | --- | --- | --- | --- | --- | --- | --- | --- | --- | --- | --- | --- | --- | --- | --- | --- | --- | --- | --- | --- | --- | --- | --- | --- | --- | --- | --- | --- | --- | --- | --- | --- | --- | --- | --- | --- | --- | --- | --- | --- | --- | --- | --- |
|  | | ... 30 amino acid residues are skipped at the N-terminus ... | | | | | | | | | | | | | | | | | | | | | | | | | | | | | | | | | | | | | | | | | | | | | | | | | | | | | | | | | | | | | |  | | |
|  | |  | | | | | | | | | | | | | | | | | | | | | | | | | | | | | | | | | | | | | | | | | | | | | | | | | | | | | | | | | | | | | | | | | | | |
| 31 |  |  | S |  | F |  | E |  | A |  | D |  | D |  | I |  | I |  | P |  | F |  |  | I |  | A |  | K |  | E |  | Q |  | V |  | R |  | S |  | D |  | C |  |  | T |  | L |  | R |  | N |  | H |  | D |  | C |  | T |  | D |  | D |  | 60 |  |
|  | |  | | | | | | | | | | | | | | | | | | | | | | | | | | | | | | | | | | | | | | | | | | | | | | | | | | | | | | | | | | | | | | | | | | | |
| 61 |  |  | R |  | H |  | S |  | C |  | C |  | R |  | S |  | K |  | M |  | F |  |  | K |  | D |  | V |  | C |  | T |  | C |  | F |  | Y |  | P |  | S |  |  | Q |  | R |  | S |  | E |  | T |  | A |  | R | ] | A | ⎩ | K | ⎩ | K |  | 90 |  |
|  | |  | | | | | | | | | | | | | | | | | | | | | | | | | | | | | | | | | | | | | | | | | | | | | | | | | | | -58.01 | | | | | | | | | | | | | |
| 91 |  |  | E | ⎱ | L |  | C |  | T | ⎫ | C | ⎫ | Q | ⎱ | Q |  | P | ⎱ | K | ⎫ | H |  |  | L |  | K | ⎱ | Y | ⎫ | I | ⎱ | E | ⎱ | K |  | G |  | L |  | Q | ⎱ | K |  | ⎱ | A |  | K | ⎫ | D | ⎫ | Y | ⎫ | A |  | T |  | G |  | | 117 |  | | | | | |

Fixed PTMs: Carbamidomethylation [C93 C95 ]   
  
     Unexpected modifications:   Unknown [-58.01]

  

All peaks (57)  Matched peaks (31)  Not matched peaks (26)

  

| Scan | Peak | Mono mass | Mono m/z | Intensity | Charge | Theoretical mass | Ion | Pos | Mass error | PPM error |
| --- | --- | --- | --- | --- | --- | --- | --- | --- | --- | --- |
| 455 | 1 | 3418.7991 | 684.7671 | 149145.63 | 5 |  |  |  |  |  |
| 455 | 2 | 1738.4161 | 580.4793 | 357284.26 | 3 |  |  |  |  |  |
| 455 | 3 | 3474.8320 | 580.1460 | 290458.67 | 6 |  |  |  |  |  |
| 455 | 4 | 3140.6753 | 786.1761 | 64590.77 | 4 | 3140.6950 | C26 | 26 | -0.0197 | -6.26 |
| 455 | 5 | 3418.8019 | 855.7077 | 51354.18 | 4 |  |  |  |  |  |
| 455 | 6 | 3025.6497 | 757.4197 | 67387.53 | 4 | 3025.6680 | C25 | 25 | -0.0183 | -6.06 |
| 455 | 7 | 2272.1689 | 758.3969 | 54782.55 | 3 | 2272.1820 | C18 | 18 | -0.0131 | -5.75 |
| 455 | 8 | 2116.1799 | 706.4006 | 43615.59 | 3 | 2116.1836 | Z\_DOT19 | 11 | -3.69e-03 | -1.75 |
| 455 | 9 | 2143.1271 | 715.3830 | 44277.47 | 3 | 2143.1394 | C17 | 17 | -0.0122 | -5.71 |
| 455 | 10 | 3459.8059 | 692.9685 | 35663.56 | 5 |  |  |  |  |  |
| 455 | 11 | 1866.9810 | 623.3343 | 55533.30 | 3 | 1866.9920 | C15 | 15 | -0.0109 | -5.86 |
| 455 | 12 | 2698.4252 | 900.4824 | 39147.97 | 3 | 2698.4410 | C22 | 22 | -0.0158 | -5.85 |
| 455 | 13 | 1609.8500 | 805.9323 | 53014.09 | 2 | 1609.8507 | Z\_DOT15 | 15 | -6.76e-04 | -0.42 |
| 455 | 14 | 2826.5184 | 707.6369 | 40048.37 | 4 | 2826.5360 | C23 | 23 | -0.0176 | -6.23 |
| 455 | 15 | 2800.4517 | 701.1202 | 35547.17 | 4 |  |  |  |  |  |
| 455 | 16 | 579.6382 | 580.6454 | 221908.63 | 1 |  |  |  |  |  |
| 455 | 17 | 3303.7391 | 826.9420 | 31034.32 | 4 | 3303.7583 | C27 | 27 | -0.0193 | -5.83 |
| 455 | 18 | 2341.2903 | 781.4374 | 33804.81 | 3 | 2341.2949 | Z\_DOT21 | 9 | -4.62e-03 | -1.97 |
| 455 | 19 | 3260.6727 | 816.1754 | 30426.51 | 4 | 3260.6841 | Z\_DOT28 | 2 | -0.0114 | -3.51 |
| 455 | 20 | 3303.7388 | 661.7550 | 25378.92 | 5 | 3303.7583 | C27 | 27 | -0.0195 | -5.91 |
| 455 | 21 | 3388.7637 | 678.7600 | 25111.20 | 5 | 3388.7791 | Z\_DOT29 | 1 | -0.0153 | -4.52 |
| 455 | 22 | 1625.8688 | 813.9417 | 36061.48 | 2 |  |  |  |  |  |
| 455 | 23 | 1360.6518 | 681.3332 | 35625.23 | 2 | 1360.6591 | C11 | 11 | -7.32e-03 | -5.38 |
| 455 | 24 | 3432.8151 | 859.2110 | 23849.00 | 4 |  |  |  |  |  |
| 455 | 25 | 2539.3763 | 635.8513 | 34312.83 | 4 |  |  |  |  |  |
| 455 | 26 | 3458.8041 | 577.4746 | 22595.63 | 6 |  |  |  |  |  |
| 455 | 27 | 3460.8129 | 866.2105 | 20315.89 | 4 |  |  |  |  |  |
| 455 | 28 | 3025.6503 | 1009.5574 | 21563.07 | 3 | 3025.6680 | C25 | 25 | -0.0178 | -5.88 |
| 455 | 29 | 1204.6623 | 603.3384 | 28549.89 | 2 | 1204.6607 | Z\_DOT12 | 18 | 1.56e-03 | 1.29 |
| 455 | 30 | 3003.5369 | 751.8915 | 20562.08 | 4 | 3003.5465 | Z\_DOT26 | 4 | -9.63e-03 | -3.21 |
| 455 | 31 | 695.5665 | 696.5737 | 119282.79 | 1 |  |  |  |  |  |
| 455 | 32 | 2030.0441 | 677.6887 | 16255.00 | 3 | 2030.0553 | C16 | 16 | -0.0112 | -5.50 |
| 455 | 33 | 1333.7041 | 667.8593 | 22510.89 | 2 | 1333.7033 | Z\_DOT13 | 17 | 8.41e-04 | 0.63 |
| 455 | 34 | 3474.8235 | 695.9720 | 98933.28 | 5 |  |  |  |  |  |
| 455 | 35 | 3004.5450 | 1002.5223 | 19073.68 | 3 |  |  |  |  |  |
| 455 | 36 | 2698.4256 | 675.6137 | 17639.33 | 4 | 2698.4410 | C22 | 22 | -0.0154 | -5.72 |
| 455 | 37 | 2960.4837 | 741.1282 | 18900.45 | 4 |  |  |  |  |  |
| 455 | 38 | 1135.5418 | 568.7782 | 21665.56 | 2 | 1135.5477 | C9 | 9 | -5.96e-03 | -5.25 |
| 455 | 39 | 1488.7458 | 745.3802 | 17140.62 | 2 | 1488.7540 | C12 | 12 | -8.29e-03 | -5.57 |
| 455 | 40 | 1390.7320 | 696.3733 | 104719.60 | 2 |  |  |  |  |  |
| 455 | 41 | 847.4562 | 848.4634 | 9602.42 | 1 | 847.4585 | C7 | 7 | -2.35e-03 | -2.77 |
| 455 | 42 | 1488.7458 | 497.2559 | 9270.86 | 3 | 1488.7540 | C12 | 12 | -8.21e-03 | -5.51 |
| 455 | 43 | 650.3118 | 651.3191 | 12571.67 | 1 | 650.3067 | Z\_DOT7 | 23 | 5.13e-03 | 7.89 |
| 455 | 44 | 869.4603 | 870.4675 | 13580.21 | 1 |  |  |  |  |  |
| 455 | 45 | 473.2940 | 474.3013 | 16619.44 | 1 | 473.2961 | C4 | 4 | -2.13e-03 | -4.49 |
| 455 | 46 | 1220.6809 | 611.3477 | 9708.45 | 2 |  |  |  |  |  |
| 455 | 47 | 1135.5421 | 1136.5494 | 8623.04 | 1 | 1135.5477 | C9 | 9 | -5.65e-03 | -4.97 |
| 455 | 48 | 778.4059 | 779.4131 | 7479.45 | 1 | 778.4016 | Z\_DOT8 | 22 | 4.22e-03 | 5.43 |
| 455 | 49 | 526.2963 | 527.3036 | 9259.67 | 1 |  |  |  |  |  |
| 455 | 50 | 1007.4838 | 1008.4910 | 6203.54 | 1 | 1007.4892 | C8 | 8 | -5.42e-03 | -5.38 |
| 455 | 51 | 976.4926 | 489.2536 | 5834.27 | 2 |  |  |  |  |  |
| 455 | 52 | 1274.6913 | 638.3529 | 7214.00 | 2 |  |  |  |  |  |
| 455 | 53 | 564.0610 | 565.0682 | 8043.83 | 1 |  |  |  |  |  |
| 455 | 54 | 1092.5865 | 547.3005 | 2843.00 | 2 |  |  |  |  |  |
| 455 | 55 | 1417.7481 | 709.8813 | 3369.64 | 2 |  |  |  |  |  |
| 455 | 56 | 1007.4840 | 504.7493 | 4175.67 | 2 | 1007.4892 | C8 | 8 | -5.19e-03 | -5.15 |
| 455 | 57 | 1205.6709 | 1206.6782 | 2330.32 | 1 |  |  |  |  |  |

  

All proteins /
CsTx-13a Cupiennius salei toxin 13 isoform a /
Proteoform #40
