# Supplementary material for: The Dual Prey-Inactivation Strategy of Spiders—In-Depth Venomic Analysis of Cupiennius salei
Source: Toxins (Basel). 2019 Mar 19;11(3):167. doi: 10.3390/toxins11030167 (PMC6468893; doi:10.3390/toxins11030167)
Supplement: Supplementary file 1 [file toxins-11-00167-s001.zip › Supplementary Dataset EV1/20180328_f2_topdown_OTMS2_EThcD_NL_i02_ms2_proteoform_cutoff_html/prsms/prsm109.html]

Protein-Spectrum-Match for Spectrum #340


All proteins /
CsTx-1a\_S1 Cupiennius salei toxin 1 isoform a S1^ACsTx-1a\_S2 Cupiennius salei toxin 1 isoform a S2 /
Proteoform #106

## Protein-Spectrum-Match #109 for Spectrum #340

|  |  |  |  |  |  |
| --- | --- | --- | --- | --- | --- |
| PrSM ID: | 109 | Scan(s): | 456 | Precursor charge: | 7 |
| Precursor m/z: | 1033.3220 | Precursor mass: | 7226.2031 | Proteoform mass: | 7226.1931 |
| # matched peaks: | 12 | # matched fragment ions: | 12 | # unexpected modifications: | 1 |
| E-value: | 1.68e-08 | P-value: | 1.68e-08 | Q-value (Spectral FDR): | 0 |

  

|  |  |  |  |  |  |  |  |  |  |  |  |  |  |  |  |  |  |  |  |  |  |  |  |  |  |  |  |  |  |  |  |  |  |  |  |  |  |  |  |  |  |  |  |  |  |  |  |  |  |  |  |  |  |  |  |  |  |  |  |  |  |  |  |  |  |  |  |  |  |
| --- | --- | --- | --- | --- | --- | --- | --- | --- | --- | --- | --- | --- | --- | --- | --- | --- | --- | --- | --- | --- | --- | --- | --- | --- | --- | --- | --- | --- | --- | --- | --- | --- | --- | --- | --- | --- | --- | --- | --- | --- | --- | --- | --- | --- | --- | --- | --- | --- | --- | --- | --- | --- | --- | --- | --- | --- | --- | --- | --- | --- | --- | --- | --- | --- | --- | --- | --- | --- | --- |
|  | |  | | | | | | | | | | | | | | | | | | | | | | | | | | | | | | | | | | | | | | | | | | | | | | | | | | | | | | | | | | | | | | | | | | | |
| 1 |  |  | M |  | K |  | V |  | L |  | I |  | I |  | S |  | A |  | V |  | L |  |  | F |  | I |  | T |  | I |  | F |  | S |  | N |  | I |  | S |  | A |  |  | E |  | I |  | E |  | D |  | D |  | F |  | L |  | E |  | D |  | E |  | 30 |  |
|  | |  | | | | | | | | | | | | | | | | | | | | | | | | | | | | | | | | | | | | | | | | | | | | | | | | | | | | | | | | | | | | | | | | | | | |
| 31 |  |  | S |  | F |  | E |  | A |  | E |  | D |  | I |  | I |  | P |  | F |  |  | F |  | E |  | N |  | E |  | Q |  | A |  | R | ] | S | ⎩ | C |  | I |  |  | P |  | K | ⎫ | H | ⎫ | E | ⎫ | E | ⎫ | C |  | T |  | N |  | D |  | K |  | 60 |  |
|  | |  | | | | | | | | | | | | | | | | | | | | | | | | | | | | | | 57.01 | | | | | | | | | | | | | | | | | | | | | | | | | | | | | | | | | | |
| 61 |  |  | H | ⎫ | N | ⎫ | C |  | C |  | R |  | K | ⎫ | G |  | L |  | F |  | K |  | ⎫ | L |  | K | ⎫ | C |  | Q | ⎫ | C |  | S |  | T |  | F |  | D |  | D |  |  | E |  | S |  | G |  | Q |  | P |  | T |  | E |  | R |  | C |  | A |  | 90 |  |
|  | |  | | | | | | | | | | | | | | | | | | | | | | | | | | | | | | | | | | | | | | | | | | | | | | | | | | | | | | | | | | | | | | | | | | | |
| 91 |  |  | C |  | G |  | R |  | P |  | M |  | G |  | H |  | Q |  | A |  | I |  |  | E |  | T |  | G |  | L |  | N |  | I | ⎫ | F | [ | R |  | G |  | L |  |  | F |  | K |  | G |  | K |  | K |  | K |  | N |  | K |  | K |  | T |  | 120 |  |
|  | |  | | | | | | | | | | | | | | | | | | | | | | | | | | | | | | | | | | | | | | | | | | | | | | | | | | | | | | | | | | | | | | | | | | | |
| 121 |  |  | K |  | G |  | | | | 122 |  | | | | | | | | | | | | | | | | | | | | | | | | | | | | | | | | | | | | | | | | | | | | | | | | | | | | | | | |

Fixed PTMs: Carbamidomethylation [C49 C56 C63 C64 C73 C75 C89 C91 ]   
  
     Unexpected modifications:   Unknown [57.01]

  

All peaks (43)  Matched peaks (12)  Not matched peaks (31)

  

| Scan | Peak | Mono mass | Mono m/z | Intensity | Charge | Theoretical mass | Ion | Pos | Mass error | PPM error |
| --- | --- | --- | --- | --- | --- | --- | --- | --- | --- | --- |
| 456 | 1 | 7121.1296 | 1018.3115 | 169498.67 | 7 |  |  |  |  |  |
| 456 | 2 | 7064.1102 | 1178.3590 | 43969.64 | 6 |  |  |  |  |  |
| 456 | 3 | 7169.1328 | 1195.8627 | 38574.04 | 6 |  |  |  |  |  |
| 456 | 4 | 7121.1324 | 1187.8627 | 40932.10 | 6 |  |  |  |  |  |
| 456 | 5 | 7077.1158 | 1180.5266 | 9718.55 | 6 | 7078.1406 | C59 | 59 | -0.0225 | -3.18 |
| 456 | 6 | 7170.1373 | 1435.0347 | 3685.50 | 5 |  |  |  |  |  |
| 456 | 7 | 3157.4961 | 1053.5060 | 4622.24 | 3 | 3157.5153 | C25 | 25 | -0.0192 | -6.10 |
| 456 | 8 | 7032.1282 | 1173.0286 | 5274.07 | 6 |  |  |  |  |  |
| 456 | 9 | 7064.1026 | 1413.8278 | 4199.04 | 5 |  |  |  |  |  |
| 456 | 10 | 7105.1221 | 1185.1943 | 3896.14 | 6 |  |  |  |  |  |
| 456 | 11 | 2782.3044 | 928.4421 | 3336.82 | 3 |  |  |  |  |  |
| 456 | 12 | 6973.0980 | 1163.1903 | 4350.45 | 6 |  |  |  |  |  |
| 456 | 13 | 1752.7563 | 877.3854 | 3588.26 | 2 | 1752.7671 | C14 | 14 | -0.0109 | -6.20 |
| 456 | 14 | 2872.3024 | 958.4414 | 3166.37 | 3 |  |  |  |  |  |
| 456 | 15 | 1866.7966 | 934.4056 | 2971.43 | 2 | 1866.8101 | C15 | 15 | -0.0135 | -7.21 |
| 456 | 16 | 868.4173 | 869.4246 | 4569.10 | 1 | 868.4225 | C7 | 7 | -5.18e-03 | -5.96 |
| 456 | 17 | 3614.0825 | 1205.7014 | 4837.58 | 3 |  |  |  |  |  |
| 456 | 18 | 7122.1380 | 1425.4349 | 2806.64 | 5 | 7123.1424 | Z\_DOT59 | 1 | -1.99e-03 | -0.28 |
| 456 | 19 | 6928.0665 | 1155.6850 | 5251.60 | 6 |  |  |  |  |  |
| 456 | 20 | 7211.1369 | 1202.8634 | 2883.26 | 6 |  |  |  |  |  |
| 456 | 21 | 3445.5789 | 1149.5336 | 1593.34 | 3 | 3445.6046 | C27 | 27 | -0.0256 | -7.44 |
| 456 | 22 | 6162.6433 | 1233.5359 | 2291.22 | 5 |  |  |  |  |  |
| 456 | 23 | 739.3756 | 740.3829 | 2792.16 | 1 | 739.3799 | C6 | 6 | -4.28e-03 | -5.78 |
| 456 | 24 | 6986.1146 | 1165.3597 | 2031.74 | 6 |  |  |  |  |  |
| 456 | 25 | 602.3174 | 603.3246 | 4146.31 | 1 | 602.3210 | C5 | 5 | -3.59e-03 | -5.97 |
| 456 | 26 | 6743.9452 | 1124.9981 | 1464.74 | 6 |  |  |  |  |  |
| 456 | 27 | 3318.5271 | 1107.1830 | 1773.44 | 3 |  |  |  |  |  |
| 456 | 28 | 6254.7220 | 1251.9517 | 839.08 | 5 |  |  |  |  |  |
| 456 | 29 | 2916.3171 | 973.1130 | 1748.84 | 3 | 2916.3363 | C23 | 23 | -0.0192 | -6.59 |
| 456 | 30 | 6033.5889 | 1207.7250 | 2173.10 | 5 |  |  |  |  |  |
| 456 | 31 | 3093.7483 | 1032.2567 | 2354.10 | 3 |  |  |  |  |  |
| 456 | 32 | 997.4583 | 998.4656 | 996.54 | 1 | 997.4651 | C8 | 8 | -6.76e-03 | -6.77 |
| 456 | 33 | 3183.5139 | 1062.1786 | 1169.57 | 3 |  |  |  |  |  |
| 456 | 34 | 5837.5119 | 1168.5097 | 1040.92 | 5 |  |  |  |  |  |
| 456 | 35 | 2743.2013 | 915.4077 | 874.83 | 3 |  |  |  |  |  |
| 456 | 36 | 1032.7381 | 1033.7454 | 3307.85 | 1 |  |  |  |  |  |
| 456 | 37 | 2471.0463 | 1236.5304 | 652.10 | 2 | 2471.0674 | C19 | 19 | -0.0211 | -8.54 |
| 456 | 38 | 1251.1457 | 1252.1530 | 434.68 | 1 |  |  |  |  |  |
| 456 | 39 | 3373.4756 | 1125.4992 | 2051.16 | 3 |  |  |  |  |  |
| 456 | 40 | 3587.5655 | 1196.8624 | 9558.38 | 3 |  |  |  |  |  |
| 456 | 41 | 1445.8332 | 1446.8405 | 641.04 | 1 |  |  |  |  |  |
| 456 | 42 | 1170.1880 | 1171.1953 | 429.14 | 1 |  |  |  |  |  |
| 456 | 43 | 1206.7184 | 1207.7256 | 426.81 | 1 |  |  |  |  |  |

  

All proteins /
CsTx-1a\_S1 Cupiennius salei toxin 1 isoform a S1^ACsTx-1a\_S2 Cupiennius salei toxin 1 isoform a S2 /
Proteoform #106
