# Supplementary material for: The Dual Prey-Inactivation Strategy of Spiders—In-Depth Venomic Analysis of Cupiennius salei
Source: Toxins (Basel). 2019 Mar 19;11(3):167. doi: 10.3390/toxins11030167 (PMC6468893; doi:10.3390/toxins11030167)
Supplement: Supplementary file 1 [file toxins-11-00167-s001.zip › Supplementary Dataset EV1/20180328_f2_topdown_OTMS2_EThcD_NL_i02_ms2_proteoform_cutoff_html/prsms/prsm11.html]

Protein-Spectrum-Match for Spectrum #221


All proteins /
CsTx-12a\_S1 Cupiennius salei toxin 12 isoform a S1^ACsTx-12a\_S2 Cupiennius salei toxin 12 isoform a S2 /
Proteoform #24

## Protein-Spectrum-Match #11 for Spectrum #221

|  |  |  |  |  |  |
| --- | --- | --- | --- | --- | --- |
| PrSM ID: | 11 | Scan(s): | 297 | Precursor charge: | 6 |
| Precursor m/z: | 721.3075 | Precursor mass: | 4321.8016 | Proteoform mass: | 4321.7866 |
| # matched peaks: | 14 | # matched fragment ions: | 14 | # unexpected modifications: | 1 |
| E-value: | 5.89e-10 | P-value: | 5.89e-10 | Q-value (Spectral FDR): | 0 |

  

|  |  |  |  |  |  |  |  |  |  |  |  |  |  |  |  |  |  |  |  |  |  |  |  |  |  |  |  |  |  |  |  |  |  |  |  |  |  |  |  |  |  |  |  |  |  |  |  |  |  |  |  |  |  |  |  |  |  |  |  |  |  |  |  |  |  |  |  |  |  |
| --- | --- | --- | --- | --- | --- | --- | --- | --- | --- | --- | --- | --- | --- | --- | --- | --- | --- | --- | --- | --- | --- | --- | --- | --- | --- | --- | --- | --- | --- | --- | --- | --- | --- | --- | --- | --- | --- | --- | --- | --- | --- | --- | --- | --- | --- | --- | --- | --- | --- | --- | --- | --- | --- | --- | --- | --- | --- | --- | --- | --- | --- | --- | --- | --- | --- | --- | --- | --- | --- |
|  | |  | | | | | | | | | | | | | | | | | | | | | | | | | | | | | | | | | | | | | | | | | | | | | | | | | | | | | | | | | | | | | | | | | | | |
| 1 |  |  | M |  | K |  | V |  | L |  | V |  | I |  | C |  | A |  | V |  | L |  |  | F |  | L |  | T |  | I |  | F |  | S |  | N |  | S |  | S |  | A |  |  | E |  | T |  | E |  | D |  | D |  | F |  | L |  | E |  | D |  | E |  | 30 |  |
|  | |  | | | | | | | | | | | | | | | | | | | | | | | | | | | | | | | | | | | | | | | | | | | | | | | | | | | | | | | | | | | | | | | | | | | |
| 31 |  |  | S |  | F |  | E |  | A |  | D |  | D |  | V |  | I |  | P |  | F |  |  | L |  | A |  | R |  | E |  | Q |  | V |  | R | ] | S |  | D |  | C |  |  | T |  | L |  | R | ⎫ | N |  | H | ⎫ | D | ⎫ | C |  | T | ⎫ | D | ⎫ | D |  | 60 |  |
|  | |  | | | | | | | | | -48.02 | | | | | | | | | | | | | | | | | | | | | | | | | | | | | | | | | | | | | | | | | | | | | | | | | | | | | | | |
| 61 |  | ⎫ | R |  | H |  | S | ⎫ | C | ⎫ | C |  | R |  | S |  | K |  | M | ⎱ | F |  |  | K | ⎫ | D |  | V |  | C | ⎫ | K | ⎫ | C |  | F |  | Y |  | P |  | S |  | ⎫ | Q | [ | R |  | S |  | D |  | T |  | A |  | R |  | A |  | K |  | K |  | 90 |  |
|  | |  | | | | | | | | | | | | | | | | | | | | | | | | | | | | | | | | | | | | | | | | | | | | | | | | | | | | | | | | | | | | | | | | | | | |
| 91 |  |  | E |  | L |  | C |  | T |  | C |  | Q |  | Q |  | D |  | K |  | H |  |  | L |  | K |  | F |  | I |  | E |  | K |  | G |  | L |  | Q |  | K |  |  | A |  | K |  | V |  | L |  | V |  | A |  | G |  | | 117 |  | | | | | |

Fixed PTMs: Carbamidomethylation [C50 C57 C64 C65 C74 C76 ]   
  
     Unexpected modifications:   Unknown [-48.02]

  

All peaks (40)  Matched peaks (14)  Not matched peaks (26)

  

| Scan | Peak | Mono mass | Mono m/z | Intensity | Charge | Theoretical mass | Ion | Pos | Mass error | PPM error |
| --- | --- | --- | --- | --- | --- | --- | --- | --- | --- | --- |
| 297 | 1 | 2161.3873 | 721.4697 | 32226.59 | 3 |  |  |  |  |  |
| 297 | 2 | 4264.7530 | 853.9579 | 11889.56 | 5 |  |  |  |  |  |
| 297 | 3 | 720.9644 | 721.9717 | 12504.31 | 1 |  |  |  |  |  |
| 297 | 4 | 2761.1108 | 921.3776 | 4342.72 | 3 | 2761.1125 | C22 | 22 | -1.63e-03 | -0.59 |
| 297 | 5 | 4264.7652 | 1067.1986 | 3302.95 | 4 |  |  |  |  |  |
| 297 | 6 | 1491.5740 | 746.7943 | 2449.49 | 2 | 1491.5830 | C12 | 12 | -9.02e-03 | -6.05 |
| 297 | 7 | 4222.6900 | 704.7889 | 2242.62 | 6 |  |  |  |  |  |
| 297 | 8 | 4237.7358 | 848.5544 | 2528.62 | 5 |  |  |  |  |  |
| 297 | 9 | 4165.6698 | 834.1412 | 2452.21 | 5 |  |  |  |  |  |
| 297 | 10 | 4300.6793 | 717.7872 | 2408.38 | 6 |  |  |  |  |  |
| 297 | 11 | 2882.3536 | 721.5957 | 3091.15 | 4 |  |  |  |  |  |
| 297 | 12 | 1606.5994 | 804.3070 | 1402.67 | 2 | 1606.6100 | C13 | 13 | -0.0106 | -6.58 |
| 297 | 13 | 3410.3946 | 853.6059 | 1894.28 | 4 | 3410.4019 | C27 | 27 | -7.27e-03 | -2.13 |
| 297 | 14 | 3036.2752 | 760.0761 | 1458.72 | 4 | 3036.2758 | C24 | 24 | -6.61e-04 | -0.22 |
| 297 | 15 | 3538.4869 | 885.6290 | 1523.81 | 4 | 3538.4968 | C28 | 28 | -9.87e-03 | -2.79 |
| 297 | 16 | 4192.7332 | 839.5539 | 1463.31 | 5 | 4192.7440 | C33 | 33 | -0.0108 | -2.57 |
| 297 | 17 | 2164.8761 | 1083.4453 | 2231.07 | 2 |  |  |  |  |  |
| 297 | 18 | 3846.5826 | 962.6529 | 2139.75 | 4 |  |  |  |  |  |
| 297 | 19 | 4311.7328 | 863.3538 | 2766.28 | 5 |  |  |  |  |  |
| 297 | 20 | 2146.8186 | 1074.4166 | 882.98 | 2 | 2146.8327 | C17 | 17 | -0.0141 | -6.57 |
| 297 | 21 | 2677.0718 | 893.3645 | 1736.57 | 3 |  |  |  |  |  |
| 297 | 22 | 4215.7487 | 844.1570 | 1061.21 | 5 |  |  |  |  |  |
| 297 | 23 | 3604.4299 | 721.8933 | 4120.35 | 5 |  |  |  |  |  |
| 297 | 24 | 3988.5296 | 798.7132 | 688.74 | 5 |  |  |  |  |  |
| 297 | 25 | 1471.6726 | 736.8436 | 668.01 | 2 |  |  |  |  |  |
| 297 | 26 | 1986.7891 | 994.4018 | 1206.89 | 2 | 1986.8020 | C16 | 16 | -0.0129 | -6.48 |
| 297 | 27 | 1376.5486 | 689.2816 | 1231.85 | 2 | 1376.5561 | C11 | 11 | -7.47e-03 | -5.43 |
| 297 | 28 | 1561.6718 | 781.8432 | 1302.29 | 2 | 1561.6820 | Z\_DOT12 | 22 | -0.0102 | -6.54 |
| 297 | 29 | 950.3804 | 951.3877 | 596.55 | 1 |  |  |  |  |  |
| 297 | 30 | 4201.6969 | 701.2901 | 689.09 | 6 |  |  |  |  |  |
| 297 | 31 | 1000.4458 | 1001.4531 | 535.59 | 1 | 1000.4508 | C8 | 8 | -5.03e-03 | -5.03 |
| 297 | 32 | 1424.1158 | 713.0652 | 552.05 | 2 |  |  |  |  |  |
| 297 | 33 | 749.3454 | 750.3527 | 1245.24 | 1 | 749.3490 | C6 | 6 | -3.59e-03 | -4.79 |
| 297 | 34 | 1115.4715 | 558.7430 | 535.53 | 2 | 1115.4778 | C9 | 9 | -6.28e-03 | -5.63 |
| 297 | 35 | 4282.7381 | 857.5549 | 1008.13 | 5 |  |  |  |  |  |
| 297 | 36 | 2103.8419 | 702.2879 | 769.12 | 3 |  |  |  |  |  |
| 297 | 37 | 683.6083 | 684.6156 | 873.36 | 1 |  |  |  |  |  |
| 297 | 38 | 4020.5885 | 805.1250 | 1331.05 | 5 |  |  |  |  |  |
| 297 | 39 | 1052.9215 | 1053.9288 | 565.65 | 1 |  |  |  |  |  |
| 297 | 40 | 702.6164 | 703.6237 | 1020.68 | 1 |  |  |  |  |  |

  

All proteins /
CsTx-12a\_S1 Cupiennius salei toxin 12 isoform a S1^ACsTx-12a\_S2 Cupiennius salei toxin 12 isoform a S2 /
Proteoform #24
