# Supplementary material for: The Dual Prey-Inactivation Strategy of Spiders—In-Depth Venomic Analysis of Cupiennius salei
Source: Toxins (Basel). 2019 Mar 19;11(3):167. doi: 10.3390/toxins11030167 (PMC6468893; doi:10.3390/toxins11030167)
Supplement: Supplementary file 1 [file toxins-11-00167-s001.zip › Supplementary Dataset EV1/20180328_f2_topdown_OTMS2_EThcD_NL_i02_ms2_proteoform_cutoff_html/prsms/prsm110.html]

Protein-Spectrum-Match for Spectrum #341


All proteins /
CsTx-12a\_S1 Cupiennius salei toxin 12 isoform a S1^ACsTx-12a\_S2 Cupiennius salei toxin 12 isoform a S2 /
Proteoform #18

## Protein-Spectrum-Match #110 for Spectrum #341

|  |  |  |  |  |  |
| --- | --- | --- | --- | --- | --- |
| PrSM ID: | 110 | Scan(s): | 457 | Precursor charge: | 6 |
| Precursor m/z: | 729.3076 | Precursor mass: | 4369.8020 | Proteoform mass: | 4369.8061 |
| # matched peaks: | 34 | # matched fragment ions: | 31 | # unexpected modifications: | 0 |
| E-value: | 3.07e-29 | P-value: | 3.07e-29 | Q-value (Spectral FDR): | 0 |

  

|  |  |  |  |  |  |  |  |  |  |  |  |  |  |  |  |  |  |  |  |  |  |  |  |  |  |  |  |  |  |  |  |  |  |  |  |  |  |  |  |  |  |  |  |  |  |  |  |  |  |  |  |  |  |  |  |  |  |  |  |  |  |  |  |  |  |  |  |  |  |
| --- | --- | --- | --- | --- | --- | --- | --- | --- | --- | --- | --- | --- | --- | --- | --- | --- | --- | --- | --- | --- | --- | --- | --- | --- | --- | --- | --- | --- | --- | --- | --- | --- | --- | --- | --- | --- | --- | --- | --- | --- | --- | --- | --- | --- | --- | --- | --- | --- | --- | --- | --- | --- | --- | --- | --- | --- | --- | --- | --- | --- | --- | --- | --- | --- | --- | --- | --- | --- | --- |
|  | |  | | | | | | | | | | | | | | | | | | | | | | | | | | | | | | | | | | | | | | | | | | | | | | | | | | | | | | | | | | | | | | | | | | | |
| 1 |  |  | M |  | K |  | V |  | L |  | V |  | I |  | C |  | A |  | V |  | L |  |  | F |  | L |  | T |  | I |  | F |  | S |  | N |  | S |  | S |  | A |  |  | E |  | T |  | E |  | D |  | D |  | F |  | L |  | E |  | D |  | E |  | 30 |  |
|  | |  | | | | | | | | | | | | | | | | | | | | | | | | | | | | | | | | | | | | | | | | | | | | | | | | | | | | | | | | | | | | | | | | | | | |
| 31 |  |  | S |  | F |  | E |  | A |  | D |  | D |  | V |  | I |  | P |  | F |  |  | L |  | A |  | R |  | E |  | Q |  | V |  | R | ] | S |  | D |  | C |  |  | T | ⎫ | L | ⎫ | R | ⎱ | N | ⎩ | H | ⎱ | D | ⎫ | C | ⎫ | T | ⎫ | D | ⎱ | D |  | 60 |  |
|  | |  | | | | | | | | | | | | | | | | | | | | | | | | | | | | | | | | | | | | | | | | | | | | | | | | | | | | | | | | | | | | | | | | | | | |
| 61 |  | ⎱ | R |  | H |  | S | ⎫ | C | ⎫ | C | ⎫ | R | ⎱ | S | ⎱ | K | ⎫ | M |  | F |  |  | K | ⎫ | D | ⎫ | V | ⎫ | C | ⎫ | K | ⎫ | C | ⎫ | F | ⎫ | Y |  | P | ⎫ | S |  | ⎫ | Q | [ | R |  | S |  | D |  | T |  | A |  | R |  | A |  | K |  | K |  | 90 |  |
|  | |  | | | | | | | | | | | | | | | | | | | | | | | | | | | | | | | | | | | | | | | | | | | | | | | | | | | | | | | | | | | | | | | | | | | |
| 91 |  |  | E |  | L |  | C |  | T |  | C |  | Q |  | Q |  | D |  | K |  | H |  |  | L |  | K |  | F |  | I |  | E |  | K |  | G |  | L |  | Q |  | K |  |  | A |  | K |  | V |  | L |  | V |  | A |  | G |  | | 117 |  | | | | | |

Fixed PTMs: Carbamidomethylation [C50 C57 C64 C65 C74 C76 ]

  

All peaks (72)  Matched peaks (34)  Not matched peaks (38)

  

| Scan | Peak | Mono mass | Mono m/z | Intensity | Charge | Theoretical mass | Ion | Pos | Mass error | PPM error |
| --- | --- | --- | --- | --- | --- | --- | --- | --- | --- | --- |
| 457 | 1 | 4312.7572 | 863.5587 | 265799.31 | 5 |  |  |  |  |  |
| 457 | 2 | 2185.3888 | 729.4702 | 432104.03 | 3 |  |  |  |  |  |
| 457 | 3 | 4312.7569 | 1079.1965 | 51933.45 | 4 |  |  |  |  |  |
| 457 | 4 | 2293.9967 | 765.6728 | 43507.11 | 3 |  |  |  |  |  |
| 457 | 5 | 3586.4928 | 897.6305 | 36160.02 | 4 | 3586.5162 | C28 | 28 | -0.0235 | -6.54 |
| 457 | 6 | 4353.7633 | 871.7599 | 31938.41 | 5 |  |  |  |  |  |
| 457 | 7 | 4240.7366 | 849.1546 | 32104.09 | 5 | 4240.7634 | C33 | 33 | -0.0268 | -6.33 |
| 457 | 8 | 2462.9492 | 821.9903 | 31281.23 | 3 | 2462.9644 | C19 | 19 | -0.0152 | -6.18 |
| 457 | 9 | 4061.7007 | 1016.4325 | 25299.21 | 4 |  |  |  |  |  |
| 457 | 10 | 1986.7904 | 994.4025 | 33582.19 | 2 | 1986.8020 | C16 | 16 | -0.0117 | -5.87 |
| 457 | 11 | 3084.2762 | 772.0763 | 25017.29 | 4 | 3084.2953 | C24 | 24 | -0.0191 | -6.20 |
| 457 | 12 | 3893.5889 | 974.4045 | 24519.81 | 4 | 3893.6153 | C30 | 30 | -0.0264 | -6.77 |
| 457 | 13 | 4255.7359 | 1064.9412 | 20371.49 | 4 |  |  |  |  |  |
| 457 | 14 | 3458.3969 | 865.6065 | 19799.30 | 4 | 3458.4213 | C27 | 27 | -0.0244 | -7.07 |
| 457 | 15 | 4221.7489 | 845.3570 | 15708.17 | 5 |  |  |  |  |  |
| 457 | 16 | 4354.7686 | 1089.6994 | 16472.34 | 4 |  |  |  |  |  |
| 457 | 17 | 1491.5743 | 746.7944 | 22096.54 | 2 | 1491.5830 | C12 | 12 | -8.77e-03 | -5.88 |
| 457 | 18 | 3746.5202 | 937.6373 | 16252.97 | 4 | 3746.5469 | C29 | 29 | -0.0267 | -7.13 |
| 457 | 19 | 874.1570 | 875.1643 | 38020.02 | 1 |  |  |  |  |  |
| 457 | 20 | 2549.9807 | 851.0008 | 19139.01 | 3 | 2549.9965 | C20 | 20 | -0.0158 | -6.18 |
| 457 | 21 | 3199.3016 | 800.8327 | 12569.72 | 4 | 3199.3222 | C25 | 25 | -0.0207 | -6.46 |
| 457 | 22 | 1907.8382 | 954.9264 | 16237.53 | 2 | 1907.8495 | Z\_DOT15 | 19 | -0.0113 | -5.91 |
| 457 | 23 | 2879.2139 | 960.7452 | 11833.57 | 3 | 2879.2309 | Z\_DOT22 | 12 | -0.0170 | -5.91 |
| 457 | 24 | 3298.3704 | 825.5999 | 10490.68 | 4 | 3298.3906 | C26 | 26 | -0.0202 | -6.14 |
| 457 | 25 | 4326.7644 | 866.3602 | 14268.53 | 5 |  |  |  |  |  |
| 457 | 26 | 2895.2327 | 724.8155 | 10410.14 | 4 |  |  |  |  |  |
| 457 | 27 | 4264.7616 | 853.9596 | 10957.53 | 5 |  |  |  |  |  |
| 457 | 28 | 3621.4415 | 906.3677 | 10152.60 | 4 | 3621.4649 | Z\_DOT28 | 6 | -0.0234 | -6.47 |
| 457 | 29 | 2186.3972 | 1094.2059 | 17176.77 | 2 |  |  |  |  |  |
| 457 | 30 | 4280.7781 | 857.1629 | 11206.61 | 5 |  |  |  |  |  |
| 457 | 31 | 2764.1872 | 922.4030 | 7968.85 | 3 | 2764.2039 | Z\_DOT21 | 13 | -0.0167 | -6.06 |
| 457 | 32 | 4153.7023 | 831.7477 | 7933.88 | 5 | 4153.7314 | C32 | 32 | -0.0291 | -7.01 |
| 457 | 33 | 4061.6975 | 813.3468 | 8536.38 | 5 |  |  |  |  |  |
| 457 | 34 | 2133.9667 | 712.3295 | 10154.35 | 3 |  |  |  |  |  |
| 457 | 35 | 4351.7676 | 726.3019 | 7018.54 | 6 |  |  |  |  |  |
| 457 | 36 | 1820.8071 | 911.4108 | 9866.55 | 2 | 1820.8174 | Z\_DOT14 | 20 | -0.0104 | -5.69 |
| 457 | 37 | 1376.5478 | 689.2812 | 8161.75 | 2 | 1376.5561 | C11 | 11 | -8.32e-03 | -6.05 |
| 457 | 38 | 2306.8498 | 1154.4322 | 8258.46 | 2 | 2306.8633 | C18 | 18 | -0.0135 | -5.86 |
| 457 | 39 | 1606.6004 | 804.3075 | 13119.16 | 2 | 1606.6100 | C13 | 13 | -9.59e-03 | -5.97 |
| 457 | 40 | 3370.3456 | 1124.4558 | 6370.18 | 3 | 3370.3631 | Z\_DOT26 | 8 | -0.0175 | -5.18 |
| 457 | 41 | 4240.7427 | 1061.1930 | 7740.51 | 4 | 4240.7634 | C33 | 33 | -0.0207 | -4.89 |
| 457 | 42 | 4267.7350 | 1067.9410 | 9465.41 | 4 |  |  |  |  |  |
| 457 | 43 | 3166.3159 | 1056.4459 | 11261.16 | 3 |  |  |  |  |  |
| 457 | 44 | 4296.7408 | 1075.1925 | 4935.16 | 4 |  |  |  |  |  |
| 457 | 45 | 4326.7740 | 1082.7008 | 7072.38 | 4 |  |  |  |  |  |
| 457 | 46 | 2678.0743 | 893.6987 | 6886.22 | 3 | 2678.0914 | C21 | 21 | -0.0171 | -6.38 |
| 457 | 47 | 2146.8193 | 1074.4169 | 8189.49 | 2 | 2146.8327 | C17 | 17 | -0.0134 | -6.23 |
| 457 | 48 | 3084.2715 | 1029.0978 | 6114.06 | 3 | 3084.2953 | C24 | 24 | -0.0238 | -7.72 |
| 457 | 49 | 1474.5474 | 738.2810 | 5771.31 | 2 |  |  |  |  |  |
| 457 | 50 | 3849.5762 | 963.4013 | 5040.74 | 4 |  |  |  |  |  |
| 457 | 51 | 3507.3954 | 877.8561 | 4866.70 | 4 | 3507.4220 | Z\_DOT27 | 7 | -0.0266 | -7.59 |
| 457 | 52 | 728.6293 | 729.6366 | 242003.63 | 1 |  |  |  |  |  |
| 457 | 53 | 1275.5009 | 638.7577 | 4376.36 | 2 | 1275.5084 | C10 | 10 | -7.52e-03 | -5.90 |
| 457 | 54 | 749.3448 | 750.3520 | 8592.10 | 1 | 749.3490 | C6 | 6 | -4.20e-03 | -5.61 |
| 457 | 55 | 1000.4452 | 501.2299 | 3721.73 | 2 | 1000.4508 | C8 | 8 | -5.59e-03 | -5.59 |
| 457 | 56 | 330.1525 | 331.1598 | 3457.38 | 1 |  |  |  |  |  |
| 457 | 57 | 694.2924 | 695.2997 | 3948.89 | 1 |  |  |  |  |  |
| 457 | 58 | 1287.5170 | 1288.5242 | 2081.53 | 1 |  |  |  |  |  |
| 457 | 59 | 493.2148 | 494.2220 | 1878.82 | 1 |  |  |  |  |  |
| 457 | 60 | 1115.4716 | 558.7431 | 3748.52 | 2 | 1115.4778 | C9 | 9 | -6.16e-03 | -5.52 |
| 457 | 61 | 822.3864 | 823.3937 | 1938.71 | 1 |  |  |  |  |  |
| 457 | 62 | 593.2448 | 594.2521 | 1938.06 | 1 | 593.2479 | C5 | 5 | -3.07e-03 | -5.17 |
| 457 | 63 | 361.1259 | 362.1331 | 1127.61 | 1 |  |  |  |  |  |
| 457 | 64 | 1230.1676 | 1231.1749 | 1450.71 | 1 |  |  |  |  |  |
| 457 | 65 | 1115.4712 | 1116.4785 | 1181.85 | 1 | 1115.4778 | C9 | 9 | -6.58e-03 | -5.90 |
| 457 | 66 | 576.2188 | 577.2261 | 853.40 | 1 |  |  |  |  |  |
| 457 | 67 | 712.0801 | 713.0873 | 1387.83 | 1 |  |  |  |  |  |
| 457 | 68 | 1049.4321 | 1050.4394 | 809.86 | 1 |  |  |  |  |  |
| 457 | 69 | 1202.9641 | 1203.9714 | 639.03 | 1 |  |  |  |  |  |
| 457 | 70 | 480.1615 | 481.1688 | 1570.14 | 1 | 480.1638 | C4 | 4 | -2.26e-03 | -4.70 |
| 457 | 71 | 1426.9218 | 714.4682 | 595.36 | 2 |  |  |  |  |  |
| 457 | 72 | 1458.2679 | 1459.2752 | 565.32 | 1 |  |  |  |  |  |

  

All proteins /
CsTx-12a\_S1 Cupiennius salei toxin 12 isoform a S1^ACsTx-12a\_S2 Cupiennius salei toxin 12 isoform a S2 /
Proteoform #18
