# Supplementary material for: The Dual Prey-Inactivation Strategy of Spiders—In-Depth Venomic Analysis of Cupiennius salei
Source: Toxins (Basel). 2019 Mar 19;11(3):167. doi: 10.3390/toxins11030167 (PMC6468893; doi:10.3390/toxins11030167)
Supplement: Supplementary file 1 [file toxins-11-00167-s001.zip › Supplementary Dataset EV1/20180328_f2_topdown_OTMS2_EThcD_NL_i02_ms2_proteoform_cutoff_html/prsms/prsm111.html]

Protein-Spectrum-Match for Spectrum #342


All proteins /
CsTx-1b Cupiennius salei toxin 1 isoform b /
Proteoform #88

## Protein-Spectrum-Match #111 for Spectrum #342

|  |  |  |  |  |  |
| --- | --- | --- | --- | --- | --- |
| PrSM ID: | 111 | Scan(s): | 459 | Precursor charge: | 9 |
| Precursor m/z: | 804.0298 | Precursor mass: | 7227.2023 | Proteoform mass: | 7227.1823 |
| # matched peaks: | 15 | # matched fragment ions: | 14 | # unexpected modifications: | 1 |
| E-value: | 1.10e-11 | P-value: | 1.10e-11 | Q-value (Spectral FDR): | 0 |

  

|  |  |  |  |  |  |  |  |  |  |  |  |  |  |  |  |  |  |  |  |  |  |  |  |  |  |  |  |  |  |  |  |  |  |  |  |  |  |  |  |  |  |  |  |  |  |  |  |  |  |  |  |  |  |  |  |  |  |  |  |  |  |  |  |  |  |  |  |  |  |
| --- | --- | --- | --- | --- | --- | --- | --- | --- | --- | --- | --- | --- | --- | --- | --- | --- | --- | --- | --- | --- | --- | --- | --- | --- | --- | --- | --- | --- | --- | --- | --- | --- | --- | --- | --- | --- | --- | --- | --- | --- | --- | --- | --- | --- | --- | --- | --- | --- | --- | --- | --- | --- | --- | --- | --- | --- | --- | --- | --- | --- | --- | --- | --- | --- | --- | --- | --- | --- | --- |
|  | |  | | | | | | | | | | | | | | | | | | | | | | | | | | | | | | | | | | | | | | | | | | | | | | | | | | | | | | | | | | | | | | | | | | | |
| 1 |  |  | M |  | K |  | V |  | L |  | I |  | I |  | S |  | A |  | V |  | L |  |  | F |  | I |  | T |  | I |  | F |  | S |  | N |  | I |  | S |  | A |  |  | E |  | I |  | E |  | D |  | D |  | F |  | L |  | E |  | D |  | E |  | 30 |  |
|  | |  | | | | | | | | | | | | | | | | | | | | | | | | | | | | | | | | | | | | | | | | | | | | | | | | | | | | | | | | | | | | | | | | | | | |
| 31 |  |  | S |  | F |  | E |  | A |  | E |  | D |  | I |  | I |  | P |  | F |  |  | L |  | E |  | N |  | E |  | Q |  | A |  | R | ] | S | ⎩ | C |  | I |  |  | P |  | K |  | H | ⎫ | E | ⎫ | E |  | C |  | T |  | N |  | D |  | K |  | 60 |  |
|  | |  | | | | | | | | | | | | | | | | | | | | | | | | | | | | | | | | | | | | | | | | | | | | | | | | | | | | | | | | | | | | | | | | | | | |
| 61 |  |  | H | ⎫ | N | ⎫ | C |  | C |  | R |  | K |  | G | ⎫ | L | ⎫ | F | ⎫ | K |  |  | L |  | K | ⎫ | C | ⎫ | Q |  | C |  | S |  | T |  | F | ⎫ | D |  | D |  |  | E |  | S |  | G | ⎫ | Q |  | P |  | T |  | E |  | R |  | C |  | A |  | 90 |  |
|  | |  | | | | | | | | | | | | | | | | | | | | | | | | | | | | | | | | | | | | -155.13 | | | | | | | | | | | | | | | | | | | | | | | | | | | | |
| 91 |  |  | C |  | G |  | R |  | P |  | M |  | G |  | H |  | Q |  | A |  | I |  |  | E |  | T |  | G |  | L |  | N |  | I |  | F | ⎫ | R | ⎫ | G | [ | L |  |  | F |  | K |  | G |  | K |  | K |  | K |  | N |  | K |  | K |  | T |  | 120 |  |
|  | |  | | | | | | | | | | | | | | | | | | | | | | | | | | | | | | | | | | | | | | | | | | | | | | | | | | | | | | | | | | | | | | | | | | | |
| 121 |  |  | K |  | G |  | | | | 122 |  | | | | | | | | | | | | | | | | | | | | | | | | | | | | | | | | | | | | | | | | | | | | | | | | | | | | | | | |

Fixed PTMs: Carbamidomethylation [C49 C56 C63 C64 C73 C75 C89 C91 ]   
  
     Unexpected modifications:   Unknown [-155.13]

  

All peaks (74)  Matched peaks (15)  Not matched peaks (59)

  

| Scan | Peak | Mono mass | Mono m/z | Intensity | Charge | Theoretical mass | Ion | Pos | Mass error | PPM error |
| --- | --- | --- | --- | --- | --- | --- | --- | --- | --- | --- |
| 459 | 1 | 7169.1341 | 1025.1693 | 15341.13 | 7 | 7168.2016 | C60 | 60 | -0.0698 | -9.74 |
| 459 | 1 | 7169.1341 | 1025.1693 | 15341.13 | 7 | 7169.1768 | C61 | 61 | -0.0427 | -5.95 |
| 459 | 2 | 7169.1283 | 897.1483 | 14783.32 | 8 | 7169.1768 | C61 | 61 | -0.0485 | -6.76 |
| 459 | 3 | 7112.1100 | 1017.0230 | 10996.82 | 7 |  |  |  |  |  |
| 459 | 4 | 6842.9623 | 856.3776 | 10173.62 | 8 |  |  |  |  |  |
| 459 | 5 | 6900.0354 | 986.7266 | 6730.58 | 7 |  |  |  |  |  |
| 459 | 6 | 7064.1102 | 1010.1659 | 9056.90 | 7 |  |  |  |  |  |
| 459 | 7 | 7124.1353 | 1018.7409 | 7388.31 | 7 | 7124.1316 | Z\_DOT61 | 1 | 3.64e-03 | 0.51 |
| 459 | 8 | 7122.1355 | 891.2742 | 5391.44 | 8 |  |  |  |  |  |
| 459 | 9 | 6956.0498 | 994.7287 | 5403.87 | 7 |  |  |  |  |  |
| 459 | 10 | 6956.0522 | 870.5138 | 5921.66 | 8 |  |  |  |  |  |
| 459 | 11 | 7171.1415 | 1196.1975 | 4324.26 | 6 |  |  |  |  |  |
| 459 | 12 | 7005.0733 | 1001.7320 | 4343.37 | 7 |  |  |  |  |  |
| 459 | 13 | 6842.9766 | 978.5754 | 3998.38 | 7 |  |  |  |  |  |
| 459 | 14 | 7111.1144 | 1186.1930 | 4819.44 | 6 |  |  |  |  |  |
| 459 | 15 | 6785.9448 | 970.4280 | 4102.87 | 7 |  |  |  |  |  |
| 459 | 16 | 7064.1020 | 884.0200 | 5625.40 | 8 |  |  |  |  |  |
| 459 | 17 | 7211.1478 | 1031.1712 | 3053.27 | 7 |  |  |  |  |  |
| 459 | 18 | 802.0842 | 803.0915 | 8591.18 | 1 |  |  |  |  |  |
| 459 | 19 | 4013.5812 | 803.7235 | 4485.40 | 5 |  |  |  |  |  |
| 459 | 20 | 3157.4898 | 790.3797 | 3032.42 | 4 | 3157.5153 | C25 | 25 | -0.0255 | -8.09 |
| 459 | 21 | 6900.0312 | 863.5112 | 2623.14 | 8 |  |  |  |  |  |
| 459 | 22 | 7154.1185 | 1023.0242 | 4355.27 | 7 |  |  |  |  |  |
| 459 | 23 | 7154.1178 | 1193.3602 | 2807.20 | 6 |  |  |  |  |  |
| 459 | 24 | 4444.9053 | 889.9883 | 3637.39 | 5 |  |  |  |  |  |
| 459 | 25 | 1752.7555 | 877.3850 | 4470.80 | 2 | 1752.7671 | C14 | 14 | -0.0116 | -6.62 |
| 459 | 26 | 3614.0813 | 904.5276 | 2125.07 | 4 |  |  |  |  |  |
| 459 | 27 | 6956.0537 | 773.9021 | 2860.34 | 9 |  |  |  |  |  |
| 459 | 28 | 2788.2222 | 930.4147 | 2476.04 | 3 | 2788.2414 | C22 | 22 | -0.0192 | -6.89 |
| 459 | 29 | 3874.6497 | 969.6697 | 2170.15 | 4 |  |  |  |  |  |
| 459 | 30 | 2678.2410 | 893.7543 | 1788.27 | 3 |  |  |  |  |  |
| 459 | 31 | 7124.1321 | 1188.3626 | 2741.95 | 6 | 7124.1316 | Z\_DOT61 | 1 | 4.81e-04 | 0.07 |
| 459 | 32 | 4443.9020 | 1111.9828 | 1928.03 | 4 | 4443.9333 | C36 | 36 | -0.0313 | -7.04 |
| 459 | 33 | 3446.5822 | 862.6528 | 1860.98 | 4 |  |  |  |  |  |
| 459 | 34 | 7212.1342 | 902.5241 | 2480.01 | 8 |  |  |  |  |  |
| 459 | 35 | 4056.7798 | 1015.2022 | 2106.98 | 4 |  |  |  |  |  |
| 459 | 36 | 7103.1030 | 888.8952 | 1671.29 | 8 |  |  |  |  |  |
| 459 | 37 | 2025.8249 | 1013.9197 | 1695.90 | 2 |  |  |  |  |  |
| 459 | 38 | 2528.0689 | 843.6969 | 1330.82 | 3 | 2528.0889 | C20 | 20 | -0.0200 | -7.93 |
| 459 | 39 | 1866.7939 | 934.4042 | 2604.02 | 2 | 1866.8101 | C15 | 15 | -0.0161 | -8.65 |
| 459 | 40 | 1195.3579 | 1196.3652 | 1173.54 | 1 |  |  |  |  |  |
| 459 | 41 | 3215.4790 | 804.8770 | 2153.06 | 4 |  |  |  |  |  |
| 459 | 42 | 2641.1548 | 881.3922 | 1951.72 | 3 | 2641.1730 | C21 | 21 | -0.0182 | -6.88 |
| 459 | 43 | 5166.3452 | 862.0648 | 2235.70 | 6 |  |  |  |  |  |
| 459 | 44 | 1009.3030 | 1010.3103 | 2001.42 | 1 |  |  |  |  |  |
| 459 | 45 | 2472.0479 | 825.0232 | 1135.09 | 3 |  |  |  |  |  |
| 459 | 46 | 2408.1474 | 803.7231 | 5381.97 | 3 |  |  |  |  |  |
| 459 | 47 | 1607.8186 | 804.9166 | 2622.09 | 2 |  |  |  |  |  |
| 459 | 48 | 3940.7489 | 986.1945 | 1814.90 | 4 | 3940.7834 | C31 | 31 | -0.0345 | -8.75 |
| 459 | 49 | 868.4177 | 869.4249 | 1554.12 | 1 | 868.4225 | C7 | 7 | -4.81e-03 | -5.54 |
| 459 | 50 | 3317.5186 | 830.3869 | 1580.57 | 4 | 3317.5460 | C26 | 26 | -0.0274 | -8.26 |
| 459 | 51 | 4222.8403 | 1056.7173 | 1058.31 | 4 |  |  |  |  |  |
| 459 | 52 | 4385.8751 | 1097.4761 | 2126.94 | 4 |  |  |  |  |  |
| 459 | 53 | 2165.0108 | 722.6775 | 1098.31 | 3 |  |  |  |  |  |
| 459 | 54 | 3820.6526 | 956.1704 | 1458.52 | 4 |  |  |  |  |  |
| 459 | 55 | 7033.1216 | 1005.7389 | 1961.14 | 7 |  |  |  |  |  |
| 459 | 56 | 739.3754 | 740.3826 | 728.03 | 1 | 739.3799 | C6 | 6 | -4.52e-03 | -6.11 |
| 459 | 57 | 2352.0300 | 1177.0223 | 1201.55 | 2 |  |  |  |  |  |
| 459 | 58 | 2335.3616 | 1168.6881 | 909.93 | 2 |  |  |  |  |  |
| 459 | 59 | 2932.2676 | 978.4298 | 709.41 | 3 |  |  |  |  |  |
| 459 | 60 | 957.7694 | 958.7767 | 671.30 | 1 |  |  |  |  |  |
| 459 | 61 | 1082.2184 | 1083.2256 | 649.74 | 1 |  |  |  |  |  |
| 459 | 62 | 834.3605 | 835.3677 | 643.85 | 1 |  |  |  |  |  |
| 459 | 63 | 3532.0688 | 1178.3635 | 1248.85 | 3 |  |  |  |  |  |
| 459 | 64 | 2085.9112 | 1043.9629 | 1587.83 | 2 |  |  |  |  |  |
| 459 | 65 | 977.7117 | 978.7189 | 619.45 | 1 |  |  |  |  |  |
| 459 | 66 | 686.2890 | 687.2963 | 773.82 | 1 |  |  |  |  |  |
| 459 | 67 | 3016.3027 | 1006.4415 | 1079.05 | 3 |  |  |  |  |  |
| 459 | 68 | 770.0661 | 771.0734 | 518.88 | 1 |  |  |  |  |  |
| 459 | 69 | 1150.3401 | 1151.3473 | 616.94 | 1 |  |  |  |  |  |
| 459 | 70 | 1682.9816 | 842.4981 | 602.55 | 2 |  |  |  |  |  |
| 459 | 71 | 2876.2946 | 959.7721 | 806.25 | 3 |  |  |  |  |  |
| 459 | 72 | 3071.3424 | 1024.7881 | 726.04 | 3 |  |  |  |  |  |
| 459 | 73 | 1737.7531 | 869.8838 | 873.39 | 2 |  |  |  |  |  |
| 459 | 74 | 1041.4578 | 1042.4650 | 457.02 | 1 |  |  |  |  |  |

  

All proteins /
CsTx-1b Cupiennius salei toxin 1 isoform b /
Proteoform #88
