# Supplementary material for: The Dual Prey-Inactivation Strategy of Spiders—In-Depth Venomic Analysis of Cupiennius salei
Source: Toxins (Basel). 2019 Mar 19;11(3):167. doi: 10.3390/toxins11030167 (PMC6468893; doi:10.3390/toxins11030167)
Supplement: Supplementary file 1 [file toxins-11-00167-s001.zip › Supplementary Dataset EV1/20180328_f2_topdown_OTMS2_EThcD_NL_i02_ms2_proteoform_cutoff_html/prsms/prsm112.html]

Protein-Spectrum-Match for Spectrum #343


All proteins /
CsTx-1a\_S1 Cupiennius salei toxin 1 isoform a S1^ACsTx-1a\_S2 Cupiennius salei toxin 1 isoform a S2 /
Proteoform #106

## Protein-Spectrum-Match #112 for Spectrum #343

|  |  |  |  |  |  |
| --- | --- | --- | --- | --- | --- |
| PrSM ID: | 112 | Scan(s): | 460 | Precursor charge: | 7 |
| Precursor m/z: | 1033.3214 | Precursor mass: | 7226.1988 | Proteoform mass: | 7226.1888 |
| # matched peaks: | 11 | # matched fragment ions: | 10 | # unexpected modifications: | 1 |
| E-value: | 2.35e-07 | P-value: | 2.35e-07 | Q-value (Spectral FDR): | 3.61e-03 |

  

|  |  |  |  |  |  |  |  |  |  |  |  |  |  |  |  |  |  |  |  |  |  |  |  |  |  |  |  |  |  |  |  |  |  |  |  |  |  |  |  |  |  |  |  |  |  |  |  |  |  |  |  |  |  |  |  |  |  |  |  |  |  |  |  |  |  |  |  |  |  |
| --- | --- | --- | --- | --- | --- | --- | --- | --- | --- | --- | --- | --- | --- | --- | --- | --- | --- | --- | --- | --- | --- | --- | --- | --- | --- | --- | --- | --- | --- | --- | --- | --- | --- | --- | --- | --- | --- | --- | --- | --- | --- | --- | --- | --- | --- | --- | --- | --- | --- | --- | --- | --- | --- | --- | --- | --- | --- | --- | --- | --- | --- | --- | --- | --- | --- | --- | --- | --- | --- |
|  | |  | | | | | | | | | | | | | | | | | | | | | | | | | | | | | | | | | | | | | | | | | | | | | | | | | | | | | | | | | | | | | | | | | | | |
| 1 |  |  | M |  | K |  | V |  | L |  | I |  | I |  | S |  | A |  | V |  | L |  |  | F |  | I |  | T |  | I |  | F |  | S |  | N |  | I |  | S |  | A |  |  | E |  | I |  | E |  | D |  | D |  | F |  | L |  | E |  | D |  | E |  | 30 |  |
|  | |  | | | | | | | | | | | | | | | | | | | | | | | | | | | | | | | | | | | | | | | | | | | | | | | | | | | | | | | | | | | | | | | | | | | |
| 31 |  |  | S |  | F |  | E |  | A |  | E |  | D |  | I |  | I |  | P |  | F |  |  | F |  | E |  | N |  | E |  | Q |  | A |  | R | ] | S | ⎩ | C |  | I |  |  | P |  | K | ⎫ | H | ⎫ | E | ⎫ | E | ⎫ | C |  | T |  | N |  | D |  | K |  | 60 |  |
|  | |  | | | | | | | | | | | | | | | | | | | | | | | | | | | | | | 57.00 | | | | | | | | | | | | | | | | | | | | | | | | | | | | | | | | | | |
| 61 |  |  | H | ⎫ | N | ⎫ | C |  | C |  | R |  | K |  | G |  | L |  | F |  | K |  |  | L |  | K | ⎫ | C |  | Q | ⎫ | C |  | S |  | T |  | F |  | D |  | D |  |  | E |  | S |  | G |  | Q |  | P |  | T |  | E |  | R |  | C |  | A |  | 90 |  |
|  | |  | | | | | | | | | | | | | | | | | | | | | | | | | | | | | | | | | | | | | | | | | | | | | | | | | | | | | | | | | | | | | | | | | | | |
| 91 |  |  | C |  | G |  | R |  | P |  | M |  | G |  | H |  | Q |  | A |  | I |  |  | E |  | T |  | G |  | L |  | N |  | I | ⎫ | F | [ | R |  | G |  | L |  |  | F |  | K |  | G |  | K |  | K |  | K |  | N |  | K |  | K |  | T |  | 120 |  |
|  | |  | | | | | | | | | | | | | | | | | | | | | | | | | | | | | | | | | | | | | | | | | | | | | | | | | | | | | | | | | | | | | | | | | | | |
| 121 |  |  | K |  | G |  | | | | 122 |  | | | | | | | | | | | | | | | | | | | | | | | | | | | | | | | | | | | | | | | | | | | | | | | | | | | | | | | |

Fixed PTMs: Carbamidomethylation [C49 C56 C63 C64 C73 C75 C89 C91 ]   
  
     Unexpected modifications:   Unknown [57.00]

  

All peaks (34)  Matched peaks (11)  Not matched peaks (23)

  

| Scan | Peak | Mono mass | Mono m/z | Intensity | Charge | Theoretical mass | Ion | Pos | Mass error | PPM error |
| --- | --- | --- | --- | --- | --- | --- | --- | --- | --- | --- |
| 460 | 1 | 7121.1274 | 1018.3112 | 110265.68 | 7 |  |  |  |  |  |
| 460 | 2 | 7064.1087 | 1178.3587 | 28411.14 | 6 |  |  |  |  |  |
| 460 | 3 | 7170.1311 | 1196.0291 | 23914.92 | 6 |  |  |  |  |  |
| 460 | 4 | 7122.1341 | 1188.0296 | 25966.98 | 6 | 7123.1381 | Z\_DOT59 | 1 | -1.70e-03 | -0.24 |
| 460 | 5 | 7078.1206 | 1180.6940 | 5199.42 | 6 | 7078.1363 | C59 | 59 | -0.0157 | -2.22 |
| 460 | 6 | 3092.7493 | 1031.9237 | 6229.12 | 3 |  |  |  |  |  |
| 460 | 7 | 3157.4924 | 1053.5048 | 3388.35 | 3 | 3157.5153 | C25 | 25 | -0.0229 | -7.26 |
| 460 | 8 | 7169.1362 | 1434.8345 | 3076.69 | 5 |  |  |  |  |  |
| 460 | 9 | 7105.1118 | 1185.1926 | 3015.57 | 6 |  |  |  |  |  |
| 460 | 10 | 7065.1190 | 1414.0311 | 3242.62 | 5 |  |  |  |  |  |
| 460 | 11 | 7032.1140 | 1173.0263 | 2532.76 | 6 |  |  |  |  |  |
| 460 | 12 | 6973.0921 | 1163.1893 | 5137.70 | 6 |  |  |  |  |  |
| 460 | 13 | 1752.7544 | 877.3845 | 3158.49 | 2 | 1752.7671 | C14 | 14 | -0.0127 | -7.25 |
| 460 | 14 | 6928.0866 | 1155.6884 | 2692.25 | 6 |  |  |  |  |  |
| 460 | 15 | 868.4170 | 869.4243 | 2714.32 | 1 | 868.4225 | C7 | 7 | -5.48e-03 | -6.32 |
| 460 | 16 | 3614.0744 | 1205.6987 | 4442.10 | 3 |  |  |  |  |  |
| 460 | 17 | 2782.3069 | 928.4429 | 1835.86 | 3 |  |  |  |  |  |
| 460 | 18 | 602.3176 | 603.3249 | 2368.83 | 1 | 602.3210 | C5 | 5 | -3.35e-03 | -5.56 |
| 460 | 19 | 1866.7989 | 934.4067 | 1768.75 | 2 | 1866.8101 | C15 | 15 | -0.0111 | -5.97 |
| 460 | 20 | 2872.3023 | 958.4414 | 2022.89 | 3 |  |  |  |  |  |
| 460 | 21 | 3445.5837 | 1149.5352 | 1413.25 | 3 | 3445.6046 | C27 | 27 | -0.0209 | -6.06 |
| 460 | 22 | 6160.6564 | 1233.1386 | 1783.16 | 5 |  |  |  |  |  |
| 460 | 23 | 7124.1365 | 1425.8346 | 1489.52 | 5 | 7123.1381 | Z\_DOT59 | 1 | -3.98e-03 | -0.56 |
| 460 | 24 | 1976.9410 | 989.4778 | 643.17 | 2 |  |  |  |  |  |
| 460 | 25 | 1032.5912 | 1033.5984 | 1133.78 | 1 |  |  |  |  |  |
| 460 | 26 | 2915.3151 | 972.7790 | 1175.45 | 3 |  |  |  |  |  |
| 460 | 27 | 6033.5828 | 1207.7238 | 1401.50 | 5 |  |  |  |  |  |
| 460 | 28 | 1195.3538 | 1196.3611 | 2269.84 | 1 |  |  |  |  |  |
| 460 | 29 | 997.4590 | 998.4663 | 685.82 | 1 | 997.4651 | C8 | 8 | -6.02e-03 | -6.04 |
| 460 | 30 | 739.3753 | 740.3826 | 1507.84 | 1 | 739.3799 | C6 | 6 | -4.58e-03 | -6.20 |
| 460 | 31 | 1415.8265 | 1416.8338 | 584.65 | 1 |  |  |  |  |  |
| 460 | 32 | 5165.6752 | 1034.1423 | 796.23 | 5 |  |  |  |  |  |
| 460 | 33 | 4127.6100 | 1032.9098 | 902.15 | 4 |  |  |  |  |  |
| 460 | 34 | 1111.2281 | 1112.2354 | 405.21 | 1 |  |  |  |  |  |

  

All proteins /
CsTx-1a\_S1 Cupiennius salei toxin 1 isoform a S1^ACsTx-1a\_S2 Cupiennius salei toxin 1 isoform a S2 /
Proteoform #106
