# Supplementary material for: The Dual Prey-Inactivation Strategy of Spiders—In-Depth Venomic Analysis of Cupiennius salei
Source: Toxins (Basel). 2019 Mar 19;11(3):167. doi: 10.3390/toxins11030167 (PMC6468893; doi:10.3390/toxins11030167)
Supplement: Supplementary file 1 [file toxins-11-00167-s001.zip › Supplementary Dataset EV1/20180328_f2_topdown_OTMS2_EThcD_NL_i02_ms2_proteoform_cutoff_html/prsms/prsm113.html]

Protein-Spectrum-Match for Spectrum #344


All proteins /
CsTx-13a Cupiennius salei toxin 13 isoform a /
Proteoform #22

## Protein-Spectrum-Match #113 for Spectrum #344

|  |  |  |  |  |  |
| --- | --- | --- | --- | --- | --- |
| PrSM ID: | 113 | Scan(s): | 461 | Precursor charge: | 6 |
| Precursor m/z: | 724.7997 | Precursor mass: | 4342.7548 | Proteoform mass: | 4342.7588 |
| # matched peaks: | 28 | # matched fragment ions: | 26 | # unexpected modifications: | 0 |
| E-value: | 6.36e-26 | P-value: | 6.36e-26 | Q-value (Spectral FDR): | 0 |

  

|  |  |  |  |  |  |  |  |  |  |  |  |  |  |  |  |  |  |  |  |  |  |  |  |  |  |  |  |  |  |  |  |  |  |  |  |  |  |  |  |  |  |  |  |  |  |  |  |  |  |  |  |  |  |  |  |  |  |  |  |  |  |  |  |  |  |  |  |  |  |
| --- | --- | --- | --- | --- | --- | --- | --- | --- | --- | --- | --- | --- | --- | --- | --- | --- | --- | --- | --- | --- | --- | --- | --- | --- | --- | --- | --- | --- | --- | --- | --- | --- | --- | --- | --- | --- | --- | --- | --- | --- | --- | --- | --- | --- | --- | --- | --- | --- | --- | --- | --- | --- | --- | --- | --- | --- | --- | --- | --- | --- | --- | --- | --- | --- | --- | --- | --- | --- | --- |
|  | |  | | | | | | | | | | | | | | | | | | | | | | | | | | | | | | | | | | | | | | | | | | | | | | | | | | | | | | | | | | | | | | | | | | | |
| 1 |  |  | M |  | K |  | V |  | L |  | V |  | I |  | F |  | A |  | V |  | L |  |  | S |  | L |  | V |  | I |  | F |  | S |  | N |  | C |  | S |  | A |  |  | E |  | T |  | D |  | E |  | D |  | F |  | F |  | G |  | E |  | E |  | 30 |  |
|  | |  | | | | | | | | | | | | | | | | | | | | | | | | | | | | | | | | | | | | | | | | | | | | | | | | | | | | | | | | | | | | | | | | | | | |
| 31 |  |  | S |  | F |  | E |  | A |  | D |  | D |  | I |  | I |  | P |  | F |  |  | I |  | A |  | K |  | E |  | Q |  | V |  | R | ] | S |  | D |  | C |  |  | T |  | L |  | R | ⎱ | N |  | H | ⎱ | D | ⎫ | C | ⎫ | T | ⎫ | D | ⎱ | D |  | 60 |  |
|  | |  | | | | | | | | | | | | | | | | | | | | | | | | | | | | | | | | | | | | | | | | | | | | | | | | | | | | | | | | | | | | | | | | | | | |
| 61 |  | ⎩ | R | ⎫ | H |  | S |  | C |  | C | ⎩ | R | ⎫ | S | ⎫ | K | ⎫ | M | ⎫ | F |  | ⎱ | K | ⎫ | D | ⎫ | V | ⎫ | C |  | T | ⎫ | C | ⎫ | F | ⎫ | Y |  | P | ⎫ | S |  | ⎫ | Q | [ | R |  | S |  | E |  | T |  | A |  | R |  | A |  | K |  | K |  | 90 |  |
|  | |  | | | | | | | | | | | | | | | | | | | | | | | | | | | | | | | | | | | | | | | | | | | | | | | | | | | | | | | | | | | | | | | | | | | |
| 91 |  |  | E |  | L |  | C |  | T |  | C |  | Q |  | Q |  | P |  | K |  | H |  |  | L |  | K |  | Y |  | I |  | E |  | K |  | G |  | L |  | Q |  | K |  |  | A |  | K |  | D |  | Y |  | A |  | T |  | G |  | | 117 |  | | | | | |

Fixed PTMs: Carbamidomethylation [C50 C57 C64 C65 C74 C76 ]

  

All peaks (68)  Matched peaks (28)  Not matched peaks (40)

  

| Scan | Peak | Mono mass | Mono m/z | Intensity | Charge | Theoretical mass | Ion | Pos | Mass error | PPM error |
| --- | --- | --- | --- | --- | --- | --- | --- | --- | --- | --- |
| 461 | 1 | 4324.7207 | 721.7941 | 111266.85 | 6 |  |  |  |  |  |
| 461 | 2 | 4285.7082 | 858.1489 | 93285.37 | 5 |  |  |  |  |  |
| 461 | 3 | 4012.5780 | 803.5229 | 78556.29 | 5 |  |  |  |  |  |
| 461 | 4 | 4196.6604 | 840.3393 | 43860.01 | 5 |  |  |  |  |  |
| 461 | 5 | 4034.6536 | 1009.6707 | 22654.18 | 4 |  |  |  |  |  |
| 461 | 6 | 4285.7100 | 1072.4348 | 25543.49 | 4 |  |  |  |  |  |
| 461 | 7 | 4326.7156 | 866.3504 | 20483.62 | 5 |  |  |  |  |  |
| 461 | 8 | 4327.7237 | 1082.9382 | 14416.97 | 4 |  |  |  |  |  |
| 461 | 9 | 4213.6898 | 843.7452 | 14488.52 | 5 | 4213.7162 | C33 | 33 | -0.0264 | -6.26 |
| 461 | 10 | 1447.9109 | 724.9627 | 41541.71 | 2 |  |  |  |  |  |
| 461 | 11 | 3866.5426 | 967.6429 | 10947.54 | 4 | 3866.5680 | C30 | 30 | -0.0255 | -6.59 |
| 461 | 12 | 3084.2762 | 772.0763 | 13779.71 | 4 | 3084.2953 | C24 | 24 | -0.0191 | -6.20 |
| 461 | 13 | 2172.3728 | 1087.1937 | 22030.15 | 2 |  |  |  |  |  |
| 461 | 14 | 3559.4449 | 890.8685 | 8525.85 | 4 | 3559.4690 | C28 | 28 | -0.0240 | -6.75 |
| 461 | 15 | 2956.1800 | 740.0523 | 9939.21 | 4 | 2956.2003 | C23 | 23 | -0.0204 | -6.88 |
| 461 | 16 | 1491.5740 | 746.7943 | 10454.21 | 2 | 1491.5830 | C12 | 12 | -9.02e-03 | -6.05 |
| 461 | 17 | 4267.6980 | 854.5469 | 9402.39 | 5 |  |  |  |  |  |
| 461 | 18 | 4178.6513 | 836.7375 | 7713.47 | 5 |  |  |  |  |  |
| 461 | 19 | 3719.4747 | 930.8760 | 9235.55 | 4 | 3719.4996 | C29 | 29 | -0.0249 | -6.70 |
| 461 | 20 | 2678.0743 | 893.6987 | 8308.68 | 3 | 2678.0914 | C21 | 21 | -0.0171 | -6.38 |
| 461 | 21 | 2549.9778 | 850.9999 | 8454.18 | 3 | 2549.9965 | C20 | 20 | -0.0187 | -7.33 |
| 461 | 22 | 4238.7154 | 848.7504 | 6744.82 | 5 |  |  |  |  |  |
| 461 | 23 | 2809.1131 | 703.2855 | 8015.80 | 4 | 2809.1319 | C22 | 22 | -0.0188 | -6.71 |
| 461 | 24 | 2266.9486 | 756.6568 | 6208.32 | 3 |  |  |  |  |  |
| 461 | 25 | 868.9482 | 869.9555 | 31469.27 | 1 |  |  |  |  |  |
| 461 | 26 | 4109.6281 | 822.9329 | 5532.41 | 5 |  |  |  |  |  |
| 461 | 27 | 2462.9492 | 821.9903 | 7883.54 | 3 | 2462.9644 | C19 | 19 | -0.0152 | -6.18 |
| 461 | 28 | 3138.2693 | 1047.0970 | 4914.95 | 3 |  |  |  |  |  |
| 461 | 29 | 4253.7356 | 851.7544 | 8847.19 | 5 |  |  |  |  |  |
| 461 | 30 | 3594.3942 | 899.6058 | 6513.52 | 4 | 3594.4177 | Z\_DOT28 | 6 | -0.0235 | -6.54 |
| 461 | 31 | 4012.5794 | 1004.1521 | 7366.12 | 4 |  |  |  |  |  |
| 461 | 32 | 3298.3699 | 825.5998 | 5395.69 | 4 | 3298.3906 | C26 | 26 | -0.0207 | -6.28 |
| 461 | 33 | 3343.2918 | 1115.4379 | 5095.95 | 3 | 3343.3158 | Z\_DOT26 | 8 | -0.0240 | -7.17 |
| 461 | 34 | 4240.6924 | 1061.1804 | 5493.55 | 4 |  |  |  |  |  |
| 461 | 35 | 4268.6924 | 1068.1804 | 5611.94 | 4 |  |  |  |  |  |
| 461 | 36 | 1762.7005 | 882.3575 | 5633.55 | 2 | 1762.7111 | C14 | 14 | -0.0106 | -6.02 |
| 461 | 37 | 3199.3023 | 800.8328 | 5410.26 | 4 | 3199.3222 | C25 | 25 | -0.0199 | -6.23 |
| 461 | 38 | 4299.7225 | 860.9518 | 6099.16 | 5 |  |  |  |  |  |
| 461 | 39 | 4195.6993 | 1049.9321 | 5443.40 | 4 |  |  |  |  |  |
| 461 | 40 | 4213.6905 | 1054.4299 | 4442.58 | 4 | 4213.7162 | C33 | 33 | -0.0257 | -6.09 |
| 461 | 41 | 2896.1650 | 725.0485 | 52896.87 | 4 |  |  |  |  |  |
| 461 | 42 | 2036.8913 | 1019.4529 | 4348.99 | 2 | 2036.9033 | Z\_DOT16 | 18 | -0.0120 | -5.89 |
| 461 | 43 | 4228.6910 | 1058.1800 | 5581.29 | 4 |  |  |  |  |  |
| 461 | 44 | 4034.6481 | 807.9369 | 4632.10 | 5 |  |  |  |  |  |
| 461 | 45 | 330.1525 | 331.1598 | 7208.26 | 1 |  |  |  |  |  |
| 461 | 46 | 2737.1409 | 913.3876 | 3333.73 | 3 | 2737.1567 | Z\_DOT21 | 13 | -0.0158 | -5.77 |
| 461 | 47 | 4309.7183 | 1078.4368 | 3650.80 | 4 |  |  |  |  |  |
| 461 | 48 | 2678.0740 | 670.5258 | 3653.13 | 4 | 2678.0914 | C21 | 21 | -0.0174 | -6.50 |
| 461 | 49 | 4307.7026 | 718.9577 | 5640.24 | 6 |  |  |  |  |  |
| 461 | 50 | 1376.5476 | 689.2811 | 6123.82 | 2 | 1376.5561 | C11 | 11 | -8.45e-03 | -6.14 |
| 461 | 51 | 4126.6544 | 1032.6709 | 4758.19 | 4 | 4126.6841 | C32 | 32 | -0.0298 | -7.21 |
| 461 | 52 | 2852.1655 | 951.7291 | 5387.13 | 3 | 2852.1836 | Z\_DOT22 | 12 | -0.0181 | -6.34 |
| 461 | 53 | 749.3448 | 750.3520 | 5793.84 | 1 | 749.3490 | C6 | 6 | -4.20e-03 | -5.61 |
| 461 | 54 | 1474.5474 | 738.2810 | 2782.88 | 2 |  |  |  |  |  |
| 461 | 55 | 993.3940 | 994.4012 | 3312.80 | 1 |  |  |  |  |  |
| 461 | 56 | 1000.4452 | 501.2299 | 5487.93 | 2 | 1000.4508 | C8 | 8 | -5.65e-03 | -5.65 |
| 461 | 57 | 1086.9370 | 1087.9443 | 12277.97 | 1 |  |  |  |  |  |
| 461 | 58 | 1115.4717 | 558.7431 | 3120.85 | 2 | 1115.4778 | C9 | 9 | -6.04e-03 | -5.41 |
| 461 | 59 | 883.2655 | 884.2728 | 1031.61 | 1 |  |  |  |  |  |
| 461 | 60 | 1032.6672 | 1033.6745 | 654.68 | 1 |  |  |  |  |  |
| 461 | 61 | 1387.5582 | 1388.5655 | 652.23 | 1 | 1387.5663 | Z\_DOT11 | 23 | -8.09e-03 | -5.83 |
| 461 | 62 | 1222.4936 | 1223.5009 | 592.34 | 1 |  |  |  |  |  |
| 461 | 63 | 1057.4237 | 1058.4310 | 1807.80 | 1 |  |  |  |  |  |
| 461 | 64 | 1133.9756 | 1134.9828 | 1035.34 | 1 |  |  |  |  |  |
| 461 | 65 | 1275.4999 | 638.7572 | 2085.18 | 2 | 1275.5084 | C10 | 10 | -8.50e-03 | -6.66 |
| 461 | 66 | 1448.5832 | 1449.5905 | 842.38 | 1 |  |  |  |  |  |
| 461 | 67 | 1337.5281 | 669.7713 | 523.57 | 2 |  |  |  |  |  |
| 461 | 68 | 704.9479 | 705.9552 | 802.60 | 1 |  |  |  |  |  |

  

All proteins /
CsTx-13a Cupiennius salei toxin 13 isoform a /
Proteoform #22
