# Supplementary material for: The Dual Prey-Inactivation Strategy of Spiders—In-Depth Venomic Analysis of Cupiennius salei
Source: Toxins (Basel). 2019 Mar 19;11(3):167. doi: 10.3390/toxins11030167 (PMC6468893; doi:10.3390/toxins11030167)
Supplement: Supplementary file 1 [file toxins-11-00167-s001.zip › Supplementary Dataset EV1/20180328_f2_topdown_OTMS2_EThcD_NL_i02_ms2_proteoform_cutoff_html/prsms/prsm114.html]

Protein-Spectrum-Match for Spectrum #345


All proteins /
CsTx-13a Cupiennius salei toxin 13 isoform a /
Proteoform #40

## Protein-Spectrum-Match #114 for Spectrum #345

|  |  |  |  |  |  |
| --- | --- | --- | --- | --- | --- |
| PrSM ID: | 114 | Scan(s): | 463 | Precursor charge: | 6 |
| Precursor m/z: | 580.3142 | Precursor mass: | 3475.8413 | Proteoform mass: | 3475.8313 |
| # matched peaks: | 33 | # matched fragment ions: | 27 | # unexpected modifications: | 1 |
| E-value: | 5.31e-21 | P-value: | 5.31e-21 | Q-value (Spectral FDR): | 0 |

  

|  |  |  |  |  |  |  |  |  |  |  |  |  |  |  |  |  |  |  |  |  |  |  |  |  |  |  |  |  |  |  |  |  |  |  |  |  |  |  |  |  |  |  |  |  |  |  |  |  |  |  |  |  |  |  |  |  |  |  |  |  |  |  |  |  |  |  |
| --- | --- | --- | --- | --- | --- | --- | --- | --- | --- | --- | --- | --- | --- | --- | --- | --- | --- | --- | --- | --- | --- | --- | --- | --- | --- | --- | --- | --- | --- | --- | --- | --- | --- | --- | --- | --- | --- | --- | --- | --- | --- | --- | --- | --- | --- | --- | --- | --- | --- | --- | --- | --- | --- | --- | --- | --- | --- | --- | --- | --- | --- | --- | --- | --- | --- | --- |
|  | | ... 30 amino acid residues are skipped at the N-terminus ... | | | | | | | | | | | | | | | | | | | | | | | | | | | | | | | | | | | | | | | | | | | | | | | | | | | | | | | | | | | | | |  | | |
|  | |  | | | | | | | | | | | | | | | | | | | | | | | | | | | | | | | | | | | | | | | | | | | | | | | | | | | | | | | | | | | | | | | | | | | |
| 31 |  |  | S |  | F |  | E |  | A |  | D |  | D |  | I |  | I |  | P |  | F |  |  | I |  | A |  | K |  | E |  | Q |  | V |  | R |  | S |  | D |  | C |  |  | T |  | L |  | R |  | N |  | H |  | D |  | C |  | T |  | D |  | D |  | 60 |  |
|  | |  | | | | | | | | | | | | | | | | | | | | | | | | | | | | | | | | | | | | | | | | | | | | | | | | | | | | | | | | | | | | | | | | | | | |
| 61 |  |  | R |  | H |  | S |  | C |  | C |  | R |  | S |  | K |  | M |  | F |  |  | K |  | D |  | V |  | C |  | T |  | C |  | F |  | Y |  | P |  | S |  |  | Q |  | R |  | S |  | E |  | T |  | A |  | R | ] | A | ⎩ | K | ⎩ | K |  | 90 |  |
|  | |  | | | | | | | | | | | | | | | | | | | | | | | | | | | | | | | | | | | | | | | | | | | | | | | | | | | -58.02 | | | | | | | | | | | | | |
| 91 |  | ⎫ | E | ⎱ | L |  | C |  | T | ⎫ | C | ⎫ | Q | ⎱ | Q |  | P | ⎱ | K | ⎱ | H |  |  | L |  | K | ⎱ | Y |  | I | ⎱ | E | ⎱ | K | ⎫ | G |  | L |  | Q | ⎱ | K |  | ⎱ | A |  | K | ⎫ | D | ⎫ | Y | ⎫ | A |  | T |  | G |  | | 117 |  | | | | | |

Fixed PTMs: Carbamidomethylation [C93 C95 ]   
  
     Unexpected modifications:   Unknown [-58.02]

  

All peaks (57)  Matched peaks (33)  Not matched peaks (24)

  

| Scan | Peak | Mono mass | Mono m/z | Intensity | Charge | Theoretical mass | Ion | Pos | Mass error | PPM error |
| --- | --- | --- | --- | --- | --- | --- | --- | --- | --- | --- |
| 463 | 1 | 3418.7991 | 684.7671 | 125253.33 | 5 |  |  |  |  |  |
| 463 | 2 | 3140.6744 | 786.1759 | 60626.85 | 4 | 3140.6950 | C26 | 26 | -0.0206 | -6.57 |
| 463 | 3 | 3474.8295 | 580.1455 | 246967.31 | 6 |  |  |  |  |  |
| 463 | 4 | 3025.6492 | 757.4196 | 58750.31 | 4 | 3025.6680 | C25 | 25 | -0.0188 | -6.22 |
| 463 | 5 | 2272.1683 | 758.3967 | 47001.38 | 3 | 2272.1820 | C18 | 18 | -0.0136 | -5.99 |
| 463 | 6 | 3418.8007 | 855.7074 | 45553.18 | 4 |  |  |  |  |  |
| 463 | 7 | 1158.9438 | 580.4792 | 220266.89 | 2 |  |  |  |  |  |
| 463 | 8 | 2698.4254 | 900.4824 | 35426.53 | 3 | 2698.4410 | C22 | 22 | -0.0156 | -5.78 |
| 463 | 9 | 2826.5181 | 707.6368 | 34659.81 | 4 | 2826.5360 | C23 | 23 | -0.0179 | -6.32 |
| 463 | 10 | 3303.7383 | 826.9419 | 27906.77 | 4 | 3303.7583 | C27 | 27 | -0.0200 | -6.05 |
| 463 | 11 | 1609.8494 | 805.9320 | 46352.02 | 2 | 1609.8472 | Z\_DOT15 | 15 | 2.25e-03 | 1.40 |
| 463 | 12 | 1866.9809 | 623.3342 | 47691.95 | 3 | 1866.9920 | C15 | 15 | -0.0111 | -5.96 |
| 463 | 13 | 2143.1262 | 715.3827 | 37496.88 | 3 | 2143.1394 | C17 | 17 | -0.0131 | -6.13 |
| 463 | 14 | 3260.6722 | 816.1753 | 26204.22 | 4 | 3260.6806 | Z\_DOT28 | 2 | -8.40e-03 | -2.58 |
| 463 | 15 | 579.6381 | 580.6454 | 188977.81 | 1 |  |  |  |  |  |
| 463 | 16 | 2116.1790 | 706.4003 | 38936.38 | 3 | 2116.1800 | Z\_DOT19 | 11 | -1.07e-03 | -0.51 |
| 463 | 17 | 2800.4507 | 701.1200 | 32254.79 | 4 |  |  |  |  |  |
| 463 | 18 | 3459.8047 | 692.9682 | 32669.10 | 5 |  |  |  |  |  |
| 463 | 19 | 1360.6513 | 681.3329 | 32064.36 | 2 | 1360.6591 | C11 | 11 | -7.81e-03 | -5.74 |
| 463 | 20 | 3303.7379 | 661.7549 | 21667.28 | 5 | 3303.7583 | C27 | 27 | -0.0204 | -6.18 |
| 463 | 21 | 2341.2894 | 781.4371 | 30423.89 | 3 | 2341.2914 | Z\_DOT21 | 9 | -2.00e-03 | -0.86 |
| 463 | 22 | 3388.7644 | 678.7601 | 20371.13 | 5 | 3388.7755 | Z\_DOT29 | 1 | -0.0112 | -3.29 |
| 463 | 23 | 3432.8160 | 859.2113 | 21091.49 | 4 |  |  |  |  |  |
| 463 | 24 | 2539.3753 | 635.8511 | 28336.09 | 4 |  |  |  |  |  |
| 463 | 25 | 3458.8037 | 577.4746 | 19462.59 | 6 |  |  |  |  |  |
| 463 | 26 | 1625.8678 | 813.9412 | 32818.96 | 2 |  |  |  |  |  |
| 463 | 27 | 3025.6503 | 1009.5574 | 18892.38 | 3 | 3025.6680 | C25 | 25 | -0.0178 | -5.88 |
| 463 | 28 | 1204.6620 | 603.3383 | 24559.79 | 2 | 1204.6572 | Z\_DOT12 | 18 | 4.85e-03 | 4.03 |
| 463 | 29 | 2960.4820 | 741.1278 | 15657.73 | 4 |  |  |  |  |  |
| 463 | 30 | 1333.7039 | 667.8592 | 18742.92 | 2 | 1333.6998 | Z\_DOT13 | 17 | 4.13e-03 | 3.10 |
| 463 | 31 | 3003.5359 | 751.8913 | 15025.56 | 4 | 3003.5430 | Z\_DOT26 | 4 | -7.07e-03 | -2.35 |
| 463 | 32 | 2698.4256 | 675.6137 | 15389.75 | 4 | 2698.4410 | C22 | 22 | -0.0154 | -5.72 |
| 463 | 33 | 3474.8216 | 695.9716 | 309180.44 | 5 |  |  |  |  |  |
| 463 | 34 | 1988.0866 | 995.0506 | 13067.55 | 2 | 1988.0851 | Z\_DOT18 | 12 | 1.54e-03 | 0.78 |
| 463 | 35 | 3431.8066 | 687.3686 | 14090.01 | 5 |  |  |  |  |  |
| 463 | 36 | 3004.5426 | 1002.5215 | 15370.40 | 3 |  |  |  |  |  |
| 463 | 37 | 1488.7454 | 745.3800 | 14354.64 | 2 | 1488.7540 | C12 | 12 | -8.65e-03 | -5.81 |
| 463 | 38 | 2400.2618 | 801.0945 | 13251.04 | 3 | 2400.2769 | C19 | 19 | -0.0151 | -6.31 |
| 463 | 39 | 1390.7315 | 696.3730 | 97276.54 | 2 |  |  |  |  |  |
| 463 | 40 | 1135.5414 | 568.7780 | 19460.25 | 2 | 1135.5477 | C9 | 9 | -6.33e-03 | -5.58 |
| 463 | 41 | 847.4546 | 848.4619 | 8171.93 | 1 | 847.4585 | C7 | 7 | -3.94e-03 | -4.65 |
| 463 | 42 | 650.3116 | 651.3188 | 11999.07 | 1 | 650.3032 | Z\_DOT7 | 23 | 8.42e-03 | 12.95 |
| 463 | 43 | 473.2939 | 474.3011 | 14609.07 | 1 | 473.2961 | C4 | 4 | -2.25e-03 | -4.75 |
| 463 | 44 | 1220.6806 | 611.3476 | 8409.04 | 2 |  |  |  |  |  |
| 463 | 45 | 1488.7455 | 497.2558 | 8530.30 | 3 | 1488.7540 | C12 | 12 | -8.57e-03 | -5.76 |
| 463 | 46 | 1135.5415 | 1136.5488 | 7793.16 | 1 | 1135.5477 | C9 | 9 | -6.26e-03 | -5.51 |
| 463 | 47 | 1274.6912 | 638.3529 | 5553.28 | 2 |  |  |  |  |  |
| 463 | 48 | 564.0606 | 565.0679 | 7849.41 | 1 |  |  |  |  |  |
| 463 | 49 | 976.4924 | 489.2535 | 5543.44 | 2 |  |  |  |  |  |
| 463 | 50 | 1007.4835 | 1008.4908 | 5919.06 | 1 | 1007.4892 | C8 | 8 | -5.66e-03 | -5.62 |
| 463 | 51 | 778.4056 | 779.4128 | 6593.89 | 1 | 778.3981 | Z\_DOT8 | 22 | 7.45e-03 | 9.58 |
| 463 | 52 | 526.2963 | 527.3035 | 5649.55 | 1 |  |  |  |  |  |
| 463 | 53 | 933.8193 | 934.8265 | 3776.61 | 1 |  |  |  |  |  |
| 463 | 54 | 1007.4839 | 504.7492 | 3782.49 | 2 | 1007.4892 | C8 | 8 | -5.25e-03 | -5.21 |
| 463 | 55 | 1092.5856 | 547.3001 | 2769.06 | 2 |  |  |  |  |  |
| 463 | 56 | 344.2521 | 345.2594 | 2551.40 | 1 | 344.2535 | C3 | 3 | -1.44e-03 | -4.18 |
| 463 | 57 | 683.7622 | 684.7694 | 16014.14 | 1 |  |  |  |  |  |

  

All proteins /
CsTx-13a Cupiennius salei toxin 13 isoform a /
Proteoform #40
