# Supplementary material for: The Dual Prey-Inactivation Strategy of Spiders—In-Depth Venomic Analysis of Cupiennius salei
Source: Toxins (Basel). 2019 Mar 19;11(3):167. doi: 10.3390/toxins11030167 (PMC6468893; doi:10.3390/toxins11030167)
Supplement: Supplementary file 1 [file toxins-11-00167-s001.zip › Supplementary Dataset EV1/20180328_f2_topdown_OTMS2_EThcD_NL_i02_ms2_proteoform_cutoff_html/prsms/prsm115.html]

Protein-Spectrum-Match for Spectrum #346


All proteins /
CsTx-12a\_S1 Cupiennius salei toxin 12 isoform a S1^ACsTx-12a\_S2 Cupiennius salei toxin 12 isoform a S2 /
Proteoform #18

## Protein-Spectrum-Match #115 for Spectrum #346

|  |  |  |  |  |  |
| --- | --- | --- | --- | --- | --- |
| PrSM ID: | 115 | Scan(s): | 464 | Precursor charge: | 6 |
| Precursor m/z: | 729.3075 | Precursor mass: | 4369.8016 | Proteoform mass: | 4369.8061 |
| # matched peaks: | 32 | # matched fragment ions: | 30 | # unexpected modifications: | 0 |
| E-value: | 6.17e-29 | P-value: | 6.17e-29 | Q-value (Spectral FDR): | 0 |

  

|  |  |  |  |  |  |  |  |  |  |  |  |  |  |  |  |  |  |  |  |  |  |  |  |  |  |  |  |  |  |  |  |  |  |  |  |  |  |  |  |  |  |  |  |  |  |  |  |  |  |  |  |  |  |  |  |  |  |  |  |  |  |  |  |  |  |  |  |  |  |
| --- | --- | --- | --- | --- | --- | --- | --- | --- | --- | --- | --- | --- | --- | --- | --- | --- | --- | --- | --- | --- | --- | --- | --- | --- | --- | --- | --- | --- | --- | --- | --- | --- | --- | --- | --- | --- | --- | --- | --- | --- | --- | --- | --- | --- | --- | --- | --- | --- | --- | --- | --- | --- | --- | --- | --- | --- | --- | --- | --- | --- | --- | --- | --- | --- | --- | --- | --- | --- | --- |
|  | |  | | | | | | | | | | | | | | | | | | | | | | | | | | | | | | | | | | | | | | | | | | | | | | | | | | | | | | | | | | | | | | | | | | | |
| 1 |  |  | M |  | K |  | V |  | L |  | V |  | I |  | C |  | A |  | V |  | L |  |  | F |  | L |  | T |  | I |  | F |  | S |  | N |  | S |  | S |  | A |  |  | E |  | T |  | E |  | D |  | D |  | F |  | L |  | E |  | D |  | E |  | 30 |  |
|  | |  | | | | | | | | | | | | | | | | | | | | | | | | | | | | | | | | | | | | | | | | | | | | | | | | | | | | | | | | | | | | | | | | | | | |
| 31 |  |  | S |  | F |  | E |  | A |  | D |  | D |  | V |  | I |  | P |  | F |  |  | L |  | A |  | R |  | E |  | Q |  | V |  | R | ] | S |  | D |  | C |  |  | T | ⎫ | L | ⎱ | R | ⎱ | N | ⎩ | H | ⎫ | D | ⎫ | C | ⎫ | T | ⎫ | D | ⎱ | D |  | 60 |  |
|  | |  | | | | | | | | | | | | | | | | | | | | | | | | | | | | | | | | | | | | | | | | | | | | | | | | | | | | | | | | | | | | | | | | | | | |
| 61 |  | ⎱ | R |  | H |  | S | ⎫ | C |  | C | ⎫ | R | ⎱ | S | ⎱ | K |  | M |  | F |  |  | K | ⎫ | D | ⎫ | V | ⎫ | C | ⎱ | K | ⎫ | C | ⎫ | F | ⎫ | Y |  | P | ⎫ | S |  | ⎫ | Q | [ | R |  | S |  | D |  | T |  | A |  | R |  | A |  | K |  | K |  | 90 |  |
|  | |  | | | | | | | | | | | | | | | | | | | | | | | | | | | | | | | | | | | | | | | | | | | | | | | | | | | | | | | | | | | | | | | | | | | |
| 91 |  |  | E |  | L |  | C |  | T |  | C |  | Q |  | Q |  | D |  | K |  | H |  |  | L |  | K |  | F |  | I |  | E |  | K |  | G |  | L |  | Q |  | K |  |  | A |  | K |  | V |  | L |  | V |  | A |  | G |  | | 117 |  | | | | | |

Fixed PTMs: Carbamidomethylation [C50 C57 C64 C65 C74 C76 ]

  

All peaks (72)  Matched peaks (32)  Not matched peaks (40)

  

| Scan | Peak | Mono mass | Mono m/z | Intensity | Charge | Theoretical mass | Ion | Pos | Mass error | PPM error |
| --- | --- | --- | --- | --- | --- | --- | --- | --- | --- | --- |
| 464 | 1 | 4312.7562 | 863.5585 | 235473.84 | 5 |  |  |  |  |  |
| 464 | 2 | 1456.9300 | 729.4723 | 255756.02 | 2 |  |  |  |  |  |
| 464 | 3 | 4368.7854 | 729.1382 | 490207.24 | 6 |  |  |  |  |  |
| 464 | 4 | 4312.7574 | 1079.1966 | 45778.06 | 4 |  |  |  |  |  |
| 464 | 5 | 2293.9956 | 765.6725 | 36067.09 | 3 |  |  |  |  |  |
| 464 | 6 | 3586.4911 | 897.6300 | 30130.26 | 4 | 3586.5162 | C28 | 28 | -0.0252 | -7.02 |
| 464 | 7 | 4353.7609 | 871.7595 | 26841.17 | 5 |  |  |  |  |  |
| 464 | 8 | 2462.9496 | 821.9905 | 28991.48 | 3 | 2462.9644 | C19 | 19 | -0.0148 | -6.03 |
| 464 | 9 | 4240.7363 | 849.1545 | 26531.54 | 5 | 4240.7634 | C33 | 33 | -0.0271 | -6.40 |
| 464 | 10 | 3893.5877 | 974.4042 | 21489.68 | 4 | 3893.6153 | C30 | 30 | -0.0276 | -7.08 |
| 464 | 11 | 2914.1851 | 729.5536 | 303898.39 | 4 |  |  |  |  |  |
| 464 | 12 | 1986.7896 | 994.4021 | 29970.50 | 2 | 1986.8020 | C16 | 16 | -0.0124 | -6.24 |
| 464 | 13 | 3084.2752 | 772.0761 | 22120.49 | 4 | 3084.2953 | C24 | 24 | -0.0201 | -6.51 |
| 464 | 14 | 4061.7015 | 1016.4326 | 20235.53 | 4 |  |  |  |  |  |
| 464 | 15 | 3746.5208 | 937.6375 | 17058.66 | 4 | 3746.5469 | C29 | 29 | -0.0261 | -6.96 |
| 464 | 16 | 4255.7349 | 1064.9410 | 17143.97 | 4 |  |  |  |  |  |
| 464 | 17 | 3458.3964 | 865.6064 | 20678.16 | 4 | 3458.4213 | C27 | 27 | -0.0249 | -7.21 |
| 464 | 18 | 4221.7495 | 845.3572 | 14132.55 | 5 |  |  |  |  |  |
| 464 | 19 | 2549.9792 | 851.0004 | 15245.90 | 3 | 2549.9965 | C20 | 20 | -0.0172 | -6.75 |
| 464 | 20 | 1491.5738 | 746.7942 | 20172.35 | 2 | 1491.5830 | C12 | 12 | -9.26e-03 | -6.21 |
| 464 | 21 | 4354.7676 | 1089.6992 | 13976.52 | 4 |  |  |  |  |  |
| 464 | 22 | 874.1568 | 875.1641 | 33912.64 | 1 |  |  |  |  |  |
| 464 | 23 | 4327.7550 | 866.5583 | 12703.66 | 5 |  |  |  |  |  |
| 464 | 24 | 4280.7799 | 857.1633 | 9809.39 | 5 |  |  |  |  |  |
| 464 | 25 | 3298.3680 | 825.5993 | 9411.38 | 4 | 3298.3906 | C26 | 26 | -0.0227 | -6.88 |
| 464 | 26 | 1907.8375 | 954.9260 | 14548.55 | 2 | 1907.8495 | Z\_DOT15 | 19 | -0.0120 | -6.29 |
| 464 | 27 | 3199.3023 | 800.8328 | 10253.51 | 4 | 3199.3222 | C25 | 25 | -0.0199 | -6.23 |
| 464 | 28 | 2879.2128 | 960.7449 | 10352.72 | 3 | 2879.2309 | Z\_DOT22 | 12 | -0.0181 | -6.30 |
| 464 | 29 | 4263.7609 | 853.7595 | 10410.67 | 5 |  |  |  |  |  |
| 464 | 30 | 4267.7393 | 1067.9421 | 10097.35 | 4 |  |  |  |  |  |
| 464 | 31 | 3621.4393 | 906.3671 | 8609.52 | 4 | 3621.4649 | Z\_DOT28 | 6 | -0.0256 | -7.08 |
| 464 | 32 | 2186.3959 | 1094.2052 | 15414.07 | 2 |  |  |  |  |  |
| 464 | 33 | 4061.7025 | 813.3478 | 8256.27 | 5 |  |  |  |  |  |
| 464 | 34 | 2895.2339 | 724.8158 | 8999.88 | 4 |  |  |  |  |  |
| 464 | 35 | 1606.6006 | 804.3076 | 11800.84 | 2 | 1606.6100 | C13 | 13 | -9.35e-03 | -5.82 |
| 464 | 36 | 3084.2751 | 1029.0990 | 6685.03 | 3 | 3084.2953 | C24 | 24 | -0.0201 | -6.53 |
| 464 | 37 | 2764.1861 | 922.4026 | 7290.62 | 3 | 2764.2039 | Z\_DOT21 | 13 | -0.0178 | -6.46 |
| 464 | 38 | 1820.8059 | 911.4102 | 8106.11 | 2 | 1820.8174 | Z\_DOT14 | 20 | -0.0116 | -6.36 |
| 464 | 39 | 4326.7705 | 1082.6999 | 6075.93 | 4 |  |  |  |  |  |
| 464 | 40 | 2133.9655 | 712.3291 | 8034.11 | 3 |  |  |  |  |  |
| 464 | 41 | 4240.7339 | 1061.1908 | 6287.39 | 4 | 4240.7634 | C33 | 33 | -0.0295 | -6.96 |
| 464 | 42 | 1376.5483 | 689.2814 | 7171.45 | 2 | 1376.5561 | C11 | 11 | -7.84e-03 | -5.69 |
| 464 | 43 | 4223.7549 | 1056.9460 | 8218.86 | 4 |  |  |  |  |  |
| 464 | 44 | 2306.8486 | 1154.4316 | 7386.22 | 2 | 2306.8633 | C18 | 18 | -0.0147 | -6.39 |
| 464 | 45 | 3507.3969 | 877.8565 | 5142.04 | 4 | 3507.4220 | Z\_DOT27 | 7 | -0.0252 | -7.17 |
| 464 | 46 | 4153.7032 | 831.7479 | 5498.68 | 5 | 4153.7314 | C32 | 32 | -0.0282 | -6.79 |
| 464 | 47 | 3165.3114 | 1056.1111 | 6119.17 | 3 |  |  |  |  |  |
| 464 | 48 | 4352.7725 | 726.4694 | 7317.86 | 6 |  |  |  |  |  |
| 464 | 49 | 4295.7421 | 860.1557 | 4157.55 | 5 |  |  |  |  |  |
| 464 | 50 | 3907.6441 | 977.9183 | 4063.03 | 4 |  |  |  |  |  |
| 464 | 51 | 3777.5408 | 945.3925 | 5933.19 | 4 | 3777.5660 | Z\_DOT29 | 5 | -0.0252 | -6.67 |
| 464 | 52 | 1474.5465 | 738.2805 | 5149.56 | 2 |  |  |  |  |  |
| 464 | 53 | 912.3866 | 913.3939 | 3621.58 | 1 | 912.3926 | Z\_DOT7 | 27 | -6.04e-03 | -6.62 |
| 464 | 54 | 749.3447 | 750.3519 | 7699.37 | 1 | 749.3490 | C6 | 6 | -4.32e-03 | -5.77 |
| 464 | 55 | 694.2922 | 695.2994 | 3551.49 | 1 |  |  |  |  |  |
| 464 | 56 | 1147.5013 | 1148.5086 | 2163.82 | 1 |  |  |  |  |  |
| 464 | 57 | 1000.4450 | 501.2298 | 3406.86 | 2 | 1000.4508 | C8 | 8 | -5.83e-03 | -5.83 |
| 464 | 58 | 1275.5013 | 638.7579 | 3175.53 | 2 | 1275.5084 | C10 | 10 | -7.15e-03 | -5.61 |
| 464 | 59 | 330.1525 | 331.1598 | 3383.24 | 1 |  |  |  |  |  |
| 464 | 60 | 1032.4278 | 1033.4351 | 1532.71 | 1 |  |  |  |  |  |
| 464 | 61 | 493.2150 | 494.2223 | 1573.18 | 1 |  |  |  |  |  |
| 464 | 62 | 1115.4716 | 558.7431 | 2967.80 | 2 | 1115.4778 | C9 | 9 | -6.16e-03 | -5.52 |
| 464 | 63 | 593.2446 | 594.2519 | 1901.49 | 1 | 593.2479 | C5 | 5 | -3.25e-03 | -5.48 |
| 464 | 64 | 822.3866 | 823.3939 | 1924.33 | 1 |  |  |  |  |  |
| 464 | 65 | 1172.4891 | 1173.4964 | 1169.07 | 1 |  |  |  |  |  |
| 464 | 66 | 1287.5172 | 1288.5245 | 1876.15 | 1 |  |  |  |  |  |
| 464 | 67 | 361.1256 | 362.1329 | 1092.42 | 1 |  |  |  |  |  |
| 464 | 68 | 1093.4457 | 1094.4530 | 4463.21 | 1 |  |  |  |  |  |
| 464 | 69 | 576.2184 | 577.2257 | 836.04 | 1 |  |  |  |  |  |
| 464 | 70 | 712.0799 | 713.0872 | 1399.51 | 1 |  |  |  |  |  |
| 464 | 71 | 480.1615 | 481.1688 | 1009.14 | 1 | 480.1638 | C4 | 4 | -2.26e-03 | -4.70 |
| 464 | 72 | 1457.6007 | 1458.6080 | 978.56 | 1 |  |  |  |  |  |

  

All proteins /
CsTx-12a\_S1 Cupiennius salei toxin 12 isoform a S1^ACsTx-12a\_S2 Cupiennius salei toxin 12 isoform a S2 /
Proteoform #18
