# Supplementary material for: The Dual Prey-Inactivation Strategy of Spiders—In-Depth Venomic Analysis of Cupiennius salei
Source: Toxins (Basel). 2019 Mar 19;11(3):167. doi: 10.3390/toxins11030167 (PMC6468893; doi:10.3390/toxins11030167)
Supplement: Supplementary file 1 [file toxins-11-00167-s001.zip › Supplementary Dataset EV1/20180328_f2_topdown_OTMS2_EThcD_NL_i02_ms2_proteoform_cutoff_html/prsms/prsm116.html]

Protein-Spectrum-Match for Spectrum #347


All proteins /
CsTx-13a Cupiennius salei toxin 13 isoform a /
Proteoform #22

## Protein-Spectrum-Match #116 for Spectrum #347

|  |  |  |  |  |  |
| --- | --- | --- | --- | --- | --- |
| PrSM ID: | 116 | Scan(s): | 465 | Precursor charge: | 6 |
| Precursor m/z: | 724.9664 | Precursor mass: | 4343.7545 | Proteoform mass: | 4342.7588 |
| # matched peaks: | 27 | # matched fragment ions: | 26 | # unexpected modifications: | 0 |
| E-value: | 1.18e-25 | P-value: | 1.18e-25 | Q-value (Spectral FDR): | 0 |

  

|  |  |  |  |  |  |  |  |  |  |  |  |  |  |  |  |  |  |  |  |  |  |  |  |  |  |  |  |  |  |  |  |  |  |  |  |  |  |  |  |  |  |  |  |  |  |  |  |  |  |  |  |  |  |  |  |  |  |  |  |  |  |  |  |  |  |  |  |  |  |
| --- | --- | --- | --- | --- | --- | --- | --- | --- | --- | --- | --- | --- | --- | --- | --- | --- | --- | --- | --- | --- | --- | --- | --- | --- | --- | --- | --- | --- | --- | --- | --- | --- | --- | --- | --- | --- | --- | --- | --- | --- | --- | --- | --- | --- | --- | --- | --- | --- | --- | --- | --- | --- | --- | --- | --- | --- | --- | --- | --- | --- | --- | --- | --- | --- | --- | --- | --- | --- | --- |
|  | |  | | | | | | | | | | | | | | | | | | | | | | | | | | | | | | | | | | | | | | | | | | | | | | | | | | | | | | | | | | | | | | | | | | | |
| 1 |  |  | M |  | K |  | V |  | L |  | V |  | I |  | F |  | A |  | V |  | L |  |  | S |  | L |  | V |  | I |  | F |  | S |  | N |  | C |  | S |  | A |  |  | E |  | T |  | D |  | E |  | D |  | F |  | F |  | G |  | E |  | E |  | 30 |  |
|  | |  | | | | | | | | | | | | | | | | | | | | | | | | | | | | | | | | | | | | | | | | | | | | | | | | | | | | | | | | | | | | | | | | | | | |
| 31 |  |  | S |  | F |  | E |  | A |  | D |  | D |  | I |  | I |  | P |  | F |  |  | I |  | A |  | K |  | E |  | Q |  | V |  | R | ] | S |  | D |  | C |  |  | T |  | L |  | R | ⎱ | N |  | H | ⎱ | D | ⎫ | C |  | T | ⎫ | D | ⎫ | D |  | 60 |  |
|  | |  | | | | | | | | | | | | | | | | | | | | | | | | | | | | | | | | | | | | | | | | | | | | | | | | | | | | | | | | | | | | | | | | | | | |
| 61 |  | ⎩ | R | ⎫ | H |  | S | ⎫ | C |  | C | ⎱ | R | ⎫ | S | ⎫ | K | ⎫ | M | ⎫ | F |  | ⎱ | K | ⎫ | D | ⎫ | V | ⎫ | C |  | T | ⎫ | C | ⎫ | F | ⎫ | Y |  | P | ⎫ | S |  | ⎫ | Q | [ | R |  | S |  | E |  | T |  | A |  | R |  | A |  | K |  | K |  | 90 |  |
|  | |  | | | | | | | | | | | | | | | | | | | | | | | | | | | | | | | | | | | | | | | | | | | | | | | | | | | | | | | | | | | | | | | | | | | |
| 91 |  |  | E |  | L |  | C |  | T |  | C |  | Q |  | Q |  | P |  | K |  | H |  |  | L |  | K |  | Y |  | I |  | E |  | K |  | G |  | L |  | Q |  | K |  |  | A |  | K |  | D |  | Y |  | A |  | T |  | G |  | | 117 |  | | | | | |

Fixed PTMs: Carbamidomethylation [C50 C57 C64 C65 C74 C76 ]

  

All peaks (67)  Matched peaks (27)  Not matched peaks (40)

  

| Scan | Peak | Mono mass | Mono m/z | Intensity | Charge | Theoretical mass | Ion | Pos | Mass error | PPM error |
| --- | --- | --- | --- | --- | --- | --- | --- | --- | --- | --- |
| 465 | 1 | 4324.7211 | 721.7941 | 87540.83 | 6 |  |  |  |  |  |
| 465 | 2 | 4285.7082 | 858.1489 | 72385.22 | 5 |  |  |  |  |  |
| 465 | 3 | 4012.5768 | 803.5226 | 65187.47 | 5 |  |  |  |  |  |
| 465 | 4 | 4196.6601 | 840.3393 | 34045.70 | 5 |  |  |  |  |  |
| 465 | 5 | 2170.8630 | 724.6283 | 41315.14 | 3 |  |  |  |  |  |
| 465 | 6 | 4285.7100 | 1072.4348 | 23399.62 | 4 |  |  |  |  |  |
| 465 | 7 | 4326.7141 | 866.3501 | 16653.08 | 5 |  |  |  |  |  |
| 465 | 8 | 4034.6514 | 1009.6701 | 16371.58 | 4 |  |  |  |  |  |
| 465 | 9 | 4213.6855 | 843.7444 | 13905.05 | 5 | 4213.7162 | C33 | 33 | -0.0306 | -7.27 |
| 465 | 10 | 3615.9039 | 724.1881 | 20906.21 | 5 |  |  |  |  |  |
| 465 | 11 | 4342.7323 | 869.5537 | 83796.00 | 5 |  |  |  |  |  |
| 465 | 12 | 3084.2754 | 772.0761 | 11221.43 | 4 | 3084.2953 | C24 | 24 | -0.0198 | -6.43 |
| 465 | 13 | 2172.3720 | 1087.1933 | 17409.26 | 2 |  |  |  |  |  |
| 465 | 14 | 4327.7217 | 1082.9377 | 10224.53 | 4 |  |  |  |  |  |
| 465 | 15 | 2678.0751 | 893.6990 | 8175.05 | 3 | 2678.0914 | C21 | 21 | -0.0163 | -6.10 |
| 465 | 16 | 2956.1807 | 740.0525 | 7214.99 | 4 | 2956.2003 | C23 | 23 | -0.0196 | -6.64 |
| 465 | 17 | 2549.9781 | 851.0000 | 7063.22 | 3 | 2549.9965 | C20 | 20 | -0.0183 | -7.18 |
| 465 | 18 | 3866.5389 | 967.6420 | 8336.93 | 4 | 3866.5680 | C30 | 30 | -0.0291 | -7.54 |
| 465 | 19 | 3719.4732 | 930.8756 | 6965.95 | 4 | 3719.4996 | C29 | 29 | -0.0264 | -7.09 |
| 465 | 20 | 2809.1141 | 703.2858 | 6575.15 | 4 | 2809.1319 | C22 | 22 | -0.0179 | -6.36 |
| 465 | 21 | 1448.2454 | 725.1300 | 32555.47 | 2 |  |  |  |  |  |
| 465 | 22 | 4267.6915 | 854.5456 | 7025.30 | 5 |  |  |  |  |  |
| 465 | 23 | 4179.6468 | 836.9366 | 6315.31 | 5 |  |  |  |  |  |
| 465 | 24 | 4237.7144 | 848.5502 | 7016.21 | 5 |  |  |  |  |  |
| 465 | 25 | 4012.5775 | 1004.1517 | 6271.72 | 4 |  |  |  |  |  |
| 465 | 26 | 2462.9487 | 821.9902 | 5712.55 | 3 | 2462.9644 | C19 | 19 | -0.0158 | -6.40 |
| 465 | 27 | 4228.6867 | 1058.1789 | 5333.81 | 4 |  |  |  |  |  |
| 465 | 28 | 4253.7417 | 851.7556 | 5429.04 | 5 |  |  |  |  |  |
| 465 | 29 | 3298.3719 | 825.6002 | 5319.15 | 4 | 3298.3906 | C26 | 26 | -0.0188 | -5.69 |
| 465 | 30 | 1376.5473 | 689.2809 | 5165.85 | 2 | 1376.5561 | C11 | 11 | -8.81e-03 | -6.40 |
| 465 | 31 | 1491.5739 | 746.7942 | 8075.88 | 2 | 1491.5830 | C12 | 12 | -9.14e-03 | -6.13 |
| 465 | 32 | 3559.4447 | 890.8685 | 6115.42 | 4 | 3559.4690 | C28 | 28 | -0.0243 | -6.82 |
| 465 | 33 | 3594.3889 | 899.6045 | 4034.13 | 4 | 3594.4177 | Z\_DOT28 | 6 | -0.0288 | -8.01 |
| 465 | 34 | 2266.9471 | 756.6563 | 3853.83 | 3 |  |  |  |  |  |
| 465 | 35 | 1762.7001 | 882.3573 | 3715.25 | 2 | 1762.7111 | C14 | 14 | -0.0110 | -6.22 |
| 465 | 36 | 2306.8492 | 769.9570 | 3651.06 | 3 | 2306.8633 | C18 | 18 | -0.0141 | -6.10 |
| 465 | 37 | 1986.7904 | 994.4025 | 4823.61 | 2 | 1986.8020 | C16 | 16 | -0.0117 | -5.87 |
| 465 | 38 | 3199.3018 | 800.8327 | 3271.73 | 4 | 3199.3222 | C25 | 25 | -0.0204 | -6.38 |
| 465 | 39 | 4306.7028 | 718.7911 | 4584.70 | 6 |  |  |  |  |  |
| 465 | 40 | 3616.9083 | 905.2343 | 5603.93 | 4 |  |  |  |  |  |
| 465 | 41 | 4194.6905 | 1049.6799 | 4470.79 | 4 |  |  |  |  |  |
| 465 | 42 | 3343.2992 | 1115.4403 | 3216.18 | 3 | 3343.3158 | Z\_DOT26 | 8 | -0.0167 | -4.98 |
| 465 | 43 | 4300.7218 | 861.1516 | 4619.18 | 5 |  |  |  |  |  |
| 465 | 44 | 2036.8912 | 1019.4529 | 3165.98 | 2 | 2036.9033 | Z\_DOT16 | 18 | -0.0121 | -5.95 |
| 465 | 45 | 3138.2671 | 1047.0963 | 4284.48 | 3 |  |  |  |  |  |
| 465 | 46 | 4034.6429 | 807.9358 | 3407.94 | 5 |  |  |  |  |  |
| 465 | 47 | 4126.6583 | 1032.6718 | 4958.00 | 4 | 4126.6841 | C32 | 32 | -0.0259 | -6.27 |
| 465 | 48 | 330.1525 | 331.1598 | 6164.62 | 1 |  |  |  |  |  |
| 465 | 49 | 4268.7051 | 1068.1836 | 4439.48 | 4 |  |  |  |  |  |
| 465 | 50 | 2737.1392 | 913.3870 | 2946.78 | 3 | 2737.1567 | Z\_DOT21 | 13 | -0.0175 | -6.38 |
| 465 | 51 | 4109.6407 | 822.9354 | 4706.84 | 5 |  |  |  |  |  |
| 465 | 52 | 1115.4716 | 558.7431 | 2184.85 | 2 | 1115.4778 | C9 | 9 | -6.16e-03 | -5.52 |
| 465 | 53 | 1000.4447 | 501.2296 | 4247.89 | 2 | 1000.4508 | C8 | 8 | -6.08e-03 | -6.08 |
| 465 | 54 | 1474.5472 | 738.2809 | 2010.01 | 2 |  |  |  |  |  |
| 465 | 55 | 1000.4451 | 1001.4524 | 2169.33 | 1 | 1000.4508 | C8 | 8 | -5.70e-03 | -5.70 |
| 465 | 56 | 749.3444 | 750.3517 | 4617.29 | 1 | 749.3490 | C6 | 6 | -4.57e-03 | -6.09 |
| 465 | 57 | 1086.9367 | 1087.9439 | 11346.29 | 1 |  |  |  |  |  |
| 465 | 58 | 502.0978 | 503.1051 | 1072.57 | 1 |  |  |  |  |  |
| 465 | 59 | 1017.1620 | 1018.1693 | 729.59 | 1 |  |  |  |  |  |
| 465 | 60 | 1058.6741 | 1059.6813 | 841.66 | 1 |  |  |  |  |  |
| 465 | 61 | 1387.5592 | 1388.5665 | 592.50 | 1 | 1387.5663 | Z\_DOT11 | 23 | -7.12e-03 | -5.13 |
| 465 | 62 | 1099.4563 | 1100.4636 | 717.68 | 1 |  |  |  |  |  |
| 465 | 63 | 1398.8931 | 700.4538 | 401.45 | 2 |  |  |  |  |  |
| 465 | 64 | 1290.5148 | 1291.5220 | 513.04 | 1 |  |  |  |  |  |
| 465 | 65 | 1038.4186 | 1039.4258 | 566.74 | 1 |  |  |  |  |  |
| 465 | 66 | 1199.1345 | 1200.1418 | 649.76 | 1 |  |  |  |  |  |
| 465 | 67 | 1449.5892 | 1450.5965 | 493.55 | 1 |  |  |  |  |  |

  

All proteins /
CsTx-13a Cupiennius salei toxin 13 isoform a /
Proteoform #22
