# Supplementary material for: The Dual Prey-Inactivation Strategy of Spiders—In-Depth Venomic Analysis of Cupiennius salei
Source: Toxins (Basel). 2019 Mar 19;11(3):167. doi: 10.3390/toxins11030167 (PMC6468893; doi:10.3390/toxins11030167)
Supplement: Supplementary file 1 [file toxins-11-00167-s001.zip › Supplementary Dataset EV1/20180328_f2_topdown_OTMS2_EThcD_NL_i02_ms2_proteoform_cutoff_html/prsms/prsm117.html]

Protein-Spectrum-Match for Spectrum #351


All proteins /
CsTx-1a\_S1 Cupiennius salei toxin 1 isoform a S1^ACsTx-1a\_S2 Cupiennius salei toxin 1 isoform a S2 /
Proteoform #42

## Protein-Spectrum-Match #117 for Spectrum #351

|  |  |  |  |  |  |
| --- | --- | --- | --- | --- | --- |
| PrSM ID: | 117 | Scan(s): | 471 | Precursor charge: | 7 |
| Precursor m/z: | 1027.4619 | Precursor mass: | 7185.1823 | Proteoform mass: | 7185.1423 |
| # matched peaks: | 28 | # matched fragment ions: | 28 | # unexpected modifications: | 1 |
| E-value: | 2.15e-22 | P-value: | 2.15e-22 | Q-value (Spectral FDR): | 0 |

  

|  |  |  |  |  |  |  |  |  |  |  |  |  |  |  |  |  |  |  |  |  |  |  |  |  |  |  |  |  |  |  |  |  |  |  |  |  |  |  |  |  |  |  |  |  |  |  |  |  |  |  |  |  |  |  |  |  |  |  |  |  |  |  |  |  |  |  |  |  |  |
| --- | --- | --- | --- | --- | --- | --- | --- | --- | --- | --- | --- | --- | --- | --- | --- | --- | --- | --- | --- | --- | --- | --- | --- | --- | --- | --- | --- | --- | --- | --- | --- | --- | --- | --- | --- | --- | --- | --- | --- | --- | --- | --- | --- | --- | --- | --- | --- | --- | --- | --- | --- | --- | --- | --- | --- | --- | --- | --- | --- | --- | --- | --- | --- | --- | --- | --- | --- | --- | --- |
|  | |  | | | | | | | | | | | | | | | | | | | | | | | | | | | | | | | | | | | | | | | | | | | | | | | | | | | | | | | | | | | | | | | | | | | |
| 1 |  |  | M |  | K |  | V |  | L |  | I |  | I |  | S |  | A |  | V |  | L |  |  | F |  | I |  | T |  | I |  | F |  | S |  | N |  | I |  | S |  | A |  |  | E |  | I |  | E |  | D |  | D |  | F |  | L |  | E |  | D |  | E |  | 30 |  |
|  | |  | | | | | | | | | | | | | | | | | | | | | | | | | | | | | | | | | | | | | | | | | | | | | | | | | | | | | | | | | | | | | | | | | | | |
| 31 |  |  | S |  | F |  | E |  | A |  | E |  | D |  | I |  | I |  | P |  | F |  |  | F |  | E |  | N |  | E |  | Q |  | A |  | R | ] | S |  | C |  | I |  |  | P | ⎫ | K | ⎱ | H | ⎱ | E | ⎱ | E | ⎫ | C | ⎫ | T |  | N |  | D |  | K |  | 60 |  |
|  | |  | | | | | | | | | | | | | | | | | | | | | | | | | | | | | | | | | | | | | | | | | | | | | | | | | | | | | | | | | | | | | | | | | | | |
| 61 |  |  | H | ⎫ | N | ⎫ | C | ⎫ | C |  | R |  | K |  | G | ⎩ | L |  | F |  | K |  | ⎫ | L |  | K | ⎫ | C | ⎫ | Q | ⎫ | C |  | S |  | T |  | F |  | D |  | D |  |  | E |  | S |  | G | ⎱ | Q |  | P |  | T |  | E | ⎫ | R |  | C |  | A |  | 90 |  |
|  | |  | | | | | | | | | 15.96 | | | | | | | | | | | | | | | | | | | | | | | | | | | | | | | | | | | | | | | | | | | | | | | | | | | | | | | |
| 91 |  |  | C |  | G | ⎫ | R |  | P | ⎫ | M | ⎱ | G | ⎫ | H | ⎫ | Q | ⎫ | A |  | I |  |  | E |  | T |  | G |  | L |  | N |  | I | ⎫ | F | [ | R |  | G |  | L |  |  | F |  | K |  | G |  | K |  | K |  | K |  | N |  | K |  | K |  | T |  | 120 |  |
|  | |  | | | | | | | | | | | | | | | | | | | | | | | | | | | | | | | | | | | | | | | | | | | | | | | | | | | | | | | | | | | | | | | | | | | |
| 121 |  |  | K |  | G |  | | | | 122 |  | | | | | | | | | | | | | | | | | | | | | | | | | | | | | | | | | | | | | | | | | | | | | | | | | | | | | | | |

Fixed PTMs: Carbamidomethylation [C49 C56 C63 C64 C73 C75 C89 C91 ]   
  
     Unexpected modifications:   Unknown [15.96]

  

All peaks (73)  Matched peaks (28)  Not matched peaks (45)

  

| Scan | Peak | Mono mass | Mono m/z | Intensity | Charge | Theoretical mass | Ion | Pos | Mass error | PPM error |
| --- | --- | --- | --- | --- | --- | --- | --- | --- | --- | --- |
| 471 | 1 | 7128.1070 | 1189.0251 | 98449.98 | 6 |  |  |  |  |  |
| 471 | 2 | 3593.5666 | 1198.8628 | 63264.26 | 3 |  |  |  |  |  |
| 471 | 3 | 7141.1199 | 1191.1939 | 22536.13 | 6 |  |  |  |  |  |
| 471 | 4 | 2396.0465 | 1199.0305 | 50356.09 | 2 |  |  |  |  |  |
| 471 | 5 | 7169.1182 | 1195.8603 | 15317.62 | 6 |  |  |  |  |  |
| 471 | 6 | 7128.1079 | 1426.6289 | 13601.12 | 5 |  |  |  |  |  |
| 471 | 7 | 1026.7350 | 1027.7422 | 58940.59 | 1 |  |  |  |  |  |
| 471 | 8 | 3157.4950 | 1053.5056 | 10966.58 | 3 | 3157.5153 | C25 | 25 | -0.0203 | -6.44 |
| 471 | 9 | 2782.3016 | 928.4412 | 9594.75 | 3 |  |  |  |  |  |
| 471 | 10 | 7096.1302 | 1183.6956 | 9820.79 | 6 |  |  |  |  |  |
| 471 | 11 | 7182.1301 | 1027.0259 | 68570.83 | 7 |  |  |  |  |  |
| 471 | 12 | 7037.1028 | 1173.8577 | 7674.07 | 6 | 7037.0898 | C59 | 59 | 0.0130 | 1.85 |
| 471 | 13 | 6992.0783 | 1166.3537 | 7835.38 | 6 |  |  |  |  |  |
| 471 | 14 | 1752.7553 | 877.3849 | 8785.47 | 2 | 1752.7671 | C14 | 14 | -0.0118 | -6.76 |
| 471 | 15 | 6225.6462 | 1246.1365 | 7561.41 | 5 | 6225.6459 | C51 | 51 | 2.90e-04 | 0.05 |
| 471 | 16 | 4443.9020 | 1111.9828 | 6928.97 | 4 | 4443.9333 | C36 | 36 | -0.0313 | -7.04 |
| 471 | 17 | 868.4173 | 869.4246 | 9457.43 | 1 | 868.4225 | C7 | 7 | -5.18e-03 | -5.96 |
| 471 | 18 | 6317.7195 | 1264.5512 | 4596.50 | 5 | 6317.7277 | Z\_DOT53 | 7 | -8.16e-03 | -1.29 |
| 471 | 19 | 2872.3024 | 958.4414 | 6250.03 | 3 |  |  |  |  |  |
| 471 | 20 | 6096.5628 | 1220.3198 | 5663.58 | 5 | 6097.5873 | C50 | 50 | -0.0222 | -3.63 |
| 471 | 21 | 5961.5282 | 1193.3129 | 4135.89 | 5 | 5960.5284 | C49 | 49 | -2.58e-03 | -0.43 |
| 471 | 22 | 5903.4996 | 1181.7072 | 6485.84 | 5 | 5903.5069 | C48 | 48 | -7.32e-03 | -1.24 |
| 471 | 23 | 1866.7968 | 934.4057 | 5306.75 | 2 | 1866.8101 | C15 | 15 | -0.0132 | -7.08 |
| 471 | 24 | 7078.1232 | 1180.6945 | 5479.52 | 6 |  |  |  |  |  |
| 471 | 25 | 7112.0889 | 1186.3554 | 5909.57 | 6 |  |  |  |  |  |
| 471 | 26 | 3445.5786 | 1149.5335 | 3535.49 | 3 | 3445.6046 | C27 | 27 | -0.0260 | -7.55 |
| 471 | 27 | 5504.3169 | 1101.8707 | 2652.92 | 5 | 5503.3559 | C45 | 45 | -0.0413 | -7.51 |
| 471 | 28 | 7170.1257 | 1435.0324 | 4162.13 | 5 |  |  |  |  |  |
| 471 | 29 | 5756.4611 | 1152.2995 | 3523.90 | 5 | 5756.5098 | C47 | 47 | -0.0487 | -8.46 |
| 471 | 30 | 6583.8307 | 1317.7734 | 2964.58 | 5 | 6583.8292 | Z\_DOT55 | 5 | 1.48e-03 | 0.22 |
| 471 | 31 | 3938.6442 | 1313.8887 | 2784.08 | 3 |  |  |  |  |  |
| 471 | 32 | 7051.1173 | 1176.1935 | 4992.68 | 6 |  |  |  |  |  |
| 471 | 33 | 739.3753 | 740.3826 | 4512.75 | 1 | 739.3799 | C6 | 6 | -4.58e-03 | -6.20 |
| 471 | 34 | 3650.5541 | 1217.8587 | 2224.11 | 3 |  |  |  |  |  |
| 471 | 35 | 602.3176 | 603.3248 | 7669.23 | 1 | 602.3210 | C5 | 5 | -3.41e-03 | -5.66 |
| 471 | 36 | 7020.0470 | 1003.8711 | 3367.69 | 7 |  |  |  |  |  |
| 471 | 37 | 2916.3142 | 973.1120 | 3483.17 | 3 | 2916.3363 | C23 | 23 | -0.0222 | -7.60 |
| 471 | 38 | 3183.5117 | 1062.1778 | 2975.42 | 3 |  |  |  |  |  |
| 471 | 39 | 6600.8383 | 1321.1749 | 2449.25 | 5 |  |  |  |  |  |
| 471 | 40 | 2026.8276 | 1014.4211 | 1994.34 | 2 | 2026.8407 | C16 | 16 | -0.0131 | -6.47 |
| 471 | 41 | 997.4584 | 998.4657 | 2443.33 | 1 | 997.4651 | C8 | 8 | -6.63e-03 | -6.65 |
| 471 | 42 | 4285.8480 | 1429.6233 | 2595.21 | 3 |  |  |  |  |  |
| 471 | 43 | 7168.1044 | 1025.0222 | 3655.64 | 7 |  |  |  |  |  |
| 471 | 44 | 3317.5233 | 1106.8484 | 1931.20 | 3 | 3317.5460 | C26 | 26 | -0.0227 | -6.84 |
| 471 | 45 | 7072.0905 | 1415.4254 | 2985.67 | 5 |  |  |  |  |  |
| 471 | 46 | 6259.6916 | 1252.9456 | 2423.36 | 5 |  |  |  |  |  |
| 471 | 47 | 7142.1246 | 1429.4322 | 4162.13 | 5 |  |  |  |  |  |
| 471 | 48 | 3404.9746 | 1135.9988 | 1956.88 | 3 |  |  |  |  |  |
| 471 | 49 | 474.2236 | 475.2309 | 1457.70 | 1 | 474.2260 | C4 | 4 | -2.41e-03 | -5.09 |
| 471 | 50 | 6446.7608 | 1290.3594 | 1991.97 | 5 | 6446.7703 | Z\_DOT54 | 6 | -9.47e-03 | -1.47 |
| 471 | 51 | 7022.0658 | 1171.3516 | 1591.95 | 6 |  |  |  |  |  |
| 471 | 52 | 1682.8222 | 842.4184 | 1091.57 | 2 |  |  |  |  |  |
| 471 | 53 | 6493.7971 | 1299.7667 | 1103.53 | 5 |  |  |  |  |  |
| 471 | 54 | 4056.7829 | 1015.2030 | 1060.82 | 4 |  |  |  |  |  |
| 471 | 55 | 4899.0939 | 1225.7808 | 1636.73 | 4 | 4899.1349 | C40 | 40 | -0.0410 | -8.37 |
| 471 | 56 | 5068.3151 | 1268.0861 | 1527.71 | 4 |  |  |  |  |  |
| 471 | 57 | 1100.8663 | 1101.8736 | 889.92 | 1 |  |  |  |  |  |
| 471 | 58 | 2287.0390 | 1144.5268 | 728.18 | 2 |  |  |  |  |  |
| 471 | 59 | 2085.9019 | 1043.9582 | 725.36 | 2 |  |  |  |  |  |
| 471 | 60 | 1176.6843 | 1177.6916 | 629.19 | 1 |  |  |  |  |  |
| 471 | 61 | 1135.4933 | 1136.5005 | 1300.73 | 1 |  |  |  |  |  |
| 471 | 62 | 2743.2323 | 1372.6234 | 1158.78 | 2 |  |  |  |  |  |
| 471 | 63 | 7127.0850 | 1019.1623 | 1682.68 | 7 |  |  |  |  |  |
| 471 | 64 | 2742.2349 | 915.0856 | 583.07 | 3 | 2742.2168 | Z\_DOT24 | 36 | 0.0180 | 6.58 |
| 471 | 65 | 1157.4879 | 1158.4952 | 686.98 | 1 | 1157.4957 | C9 | 9 | -7.81e-03 | -6.74 |
| 471 | 66 | 4658.0154 | 1165.5111 | 1200.89 | 4 | 4658.0613 | Z\_DOT40 | 20 | -0.0459 | -9.85 |
| 471 | 67 | 1299.1648 | 1300.1721 | 481.90 | 1 |  |  |  |  |  |
| 471 | 68 | 7114.0968 | 1423.8266 | 1244.78 | 5 |  |  |  |  |  |
| 471 | 69 | 3884.6284 | 1295.8834 | 674.06 | 3 |  |  |  |  |  |
| 471 | 70 | 3573.0573 | 1192.0264 | 3724.72 | 3 |  |  |  |  |  |
| 471 | 71 | 1399.2204 | 1400.2277 | 498.71 | 1 |  |  |  |  |  |
| 471 | 72 | 1351.9278 | 1352.9351 | 472.09 | 1 |  |  |  |  |  |
| 471 | 73 | 1282.6354 | 1283.6426 | 636.52 | 1 | 1282.6432 | Z\_DOT12 | 48 | -7.85e-03 | -6.12 |

  

All proteins /
CsTx-1a\_S1 Cupiennius salei toxin 1 isoform a S1^ACsTx-1a\_S2 Cupiennius salei toxin 1 isoform a S2 /
Proteoform #42
