# Supplementary material for: The Dual Prey-Inactivation Strategy of Spiders—In-Depth Venomic Analysis of Cupiennius salei
Source: Toxins (Basel). 2019 Mar 19;11(3):167. doi: 10.3390/toxins11030167 (PMC6468893; doi:10.3390/toxins11030167)
Supplement: Supplementary file 1 [file toxins-11-00167-s001.zip › Supplementary Dataset EV1/20180328_f2_topdown_OTMS2_EThcD_NL_i02_ms2_proteoform_cutoff_html/prsms/prsm118.html]

Protein-Spectrum-Match for Spectrum #352


All proteins /
CsTx-13a Cupiennius salei toxin 13 isoform a /
Proteoform #40

## Protein-Spectrum-Match #118 for Spectrum #352

|  |  |  |  |  |  |
| --- | --- | --- | --- | --- | --- |
| PrSM ID: | 118 | Scan(s): | 472 | Precursor charge: | 6 |
| Precursor m/z: | 580.3144 | Precursor mass: | 3475.8428 | Proteoform mass: | 3475.8378 |
| # matched peaks: | 34 | # matched fragment ions: | 29 | # unexpected modifications: | 1 |
| E-value: | 5.33e-22 | P-value: | 5.33e-22 | Q-value (Spectral FDR): | 0 |

  

|  |  |  |  |  |  |  |  |  |  |  |  |  |  |  |  |  |  |  |  |  |  |  |  |  |  |  |  |  |  |  |  |  |  |  |  |  |  |  |  |  |  |  |  |  |  |  |  |  |  |  |  |  |  |  |  |  |  |  |  |  |  |  |  |  |  |  |
| --- | --- | --- | --- | --- | --- | --- | --- | --- | --- | --- | --- | --- | --- | --- | --- | --- | --- | --- | --- | --- | --- | --- | --- | --- | --- | --- | --- | --- | --- | --- | --- | --- | --- | --- | --- | --- | --- | --- | --- | --- | --- | --- | --- | --- | --- | --- | --- | --- | --- | --- | --- | --- | --- | --- | --- | --- | --- | --- | --- | --- | --- | --- | --- | --- | --- | --- |
|  | | ... 30 amino acid residues are skipped at the N-terminus ... | | | | | | | | | | | | | | | | | | | | | | | | | | | | | | | | | | | | | | | | | | | | | | | | | | | | | | | | | | | | | |  | | |
|  | |  | | | | | | | | | | | | | | | | | | | | | | | | | | | | | | | | | | | | | | | | | | | | | | | | | | | | | | | | | | | | | | | | | | | |
| 31 |  |  | S |  | F |  | E |  | A |  | D |  | D |  | I |  | I |  | P |  | F |  |  | I |  | A |  | K |  | E |  | Q |  | V |  | R |  | S |  | D |  | C |  |  | T |  | L |  | R |  | N |  | H |  | D |  | C |  | T |  | D |  | D |  | 60 |  |
|  | |  | | | | | | | | | | | | | | | | | | | | | | | | | | | | | | | | | | | | | | | | | | | | | | | | | | | | | | | | | | | | | | | | | | | |
| 61 |  |  | R |  | H |  | S |  | C |  | C |  | R |  | S |  | K |  | M |  | F |  |  | K |  | D |  | V |  | C |  | T |  | C |  | F |  | Y |  | P |  | S |  |  | Q |  | R |  | S |  | E |  | T |  | A |  | R | ] | A | ⎩ | K | ⎩ | K |  | 90 |  |
|  | |  | | | | | | | | | | | | | | | | | | | | | | | | | | | | | | | | | | | | | | | | | | | | | | | | | | | -58.01 | | | | | | | | | | | | | |
| 91 |  | ⎫ | E | ⎱ | L |  | C |  | T | ⎫ | C | ⎫ | Q | ⎱ | Q |  | P | ⎱ | K | ⎫ | H |  |  | L | ⎫ | K | ⎱ | Y | ⎱ | I | ⎱ | E | ⎱ | K | ⎫ | G |  | L |  | Q | ⎱ | K |  | ⎱ | A |  | K | ⎫ | D | ⎫ | Y | ⎫ | A |  | T |  | G |  | | 117 |  | | | | | |

Fixed PTMs: Carbamidomethylation [C93 C95 ]   
  
     Unexpected modifications:   Unknown [-58.01]

  

All peaks (57)  Matched peaks (34)  Not matched peaks (23)

  

| Scan | Peak | Mono mass | Mono m/z | Intensity | Charge | Theoretical mass | Ion | Pos | Mass error | PPM error |
| --- | --- | --- | --- | --- | --- | --- | --- | --- | --- | --- |
| 472 | 1 | 3418.7997 | 684.7672 | 126772.74 | 5 |  |  |  |  |  |
| 472 | 2 | 3474.8295 | 580.1455 | 228705.88 | 6 |  |  |  |  |  |
| 472 | 3 | 3140.6751 | 786.1760 | 53195.26 | 4 | 3140.6950 | C26 | 26 | -0.0199 | -6.34 |
| 472 | 4 | 3025.6502 | 757.4198 | 52681.39 | 4 | 3025.6680 | C25 | 25 | -0.0179 | -5.90 |
| 472 | 5 | 3418.8014 | 855.7076 | 40467.45 | 4 |  |  |  |  |  |
| 472 | 6 | 2272.1683 | 758.3967 | 46791.28 | 3 | 2272.1820 | C18 | 18 | -0.0136 | -5.99 |
| 472 | 7 | 3459.8053 | 692.9683 | 32901.54 | 5 |  |  |  |  |  |
| 472 | 8 | 1866.9814 | 623.3344 | 46240.98 | 3 | 1866.9920 | C15 | 15 | -0.0106 | -5.67 |
| 472 | 9 | 1158.9441 | 580.4793 | 195067.70 | 2 |  |  |  |  |  |
| 472 | 10 | 2116.1797 | 706.4005 | 36615.57 | 3 | 2116.1865 | Z\_DOT19 | 11 | -6.81e-03 | -3.22 |
| 472 | 11 | 2698.4258 | 900.4825 | 33889.03 | 3 | 2698.4410 | C22 | 22 | -0.0152 | -5.65 |
| 472 | 12 | 1609.8503 | 805.9324 | 41198.23 | 2 | 1609.8536 | Z\_DOT15 | 15 | -3.36e-03 | -2.09 |
| 472 | 13 | 2826.5189 | 707.6370 | 30262.89 | 4 | 2826.5360 | C23 | 23 | -0.0171 | -6.06 |
| 472 | 14 | 2143.1271 | 715.3830 | 38241.26 | 3 | 2143.1394 | C17 | 17 | -0.0122 | -5.71 |
| 472 | 15 | 3303.7391 | 826.9420 | 24358.89 | 4 | 3303.7583 | C27 | 27 | -0.0193 | -5.83 |
| 472 | 16 | 579.6384 | 580.6456 | 175905.28 | 1 |  |  |  |  |  |
| 472 | 17 | 2800.4524 | 701.1204 | 28477.69 | 4 |  |  |  |  |  |
| 472 | 18 | 3260.6727 | 816.1754 | 26800.47 | 4 | 3260.6870 | Z\_DOT28 | 2 | -0.0144 | -4.41 |
| 472 | 19 | 2341.2907 | 781.4375 | 28020.49 | 3 | 2341.2978 | Z\_DOT21 | 9 | -7.19e-03 | -3.07 |
| 472 | 20 | 3432.8173 | 859.2116 | 23109.65 | 4 |  |  |  |  |  |
| 472 | 21 | 2539.3758 | 635.8512 | 28231.37 | 4 |  |  |  |  |  |
| 472 | 22 | 1625.8683 | 813.9414 | 30195.73 | 2 |  |  |  |  |  |
| 472 | 23 | 1360.6515 | 681.3330 | 27881.06 | 2 | 1360.6591 | C11 | 11 | -7.56e-03 | -5.56 |
| 472 | 24 | 3388.7641 | 678.7601 | 21620.92 | 5 | 3388.7820 | Z\_DOT29 | 1 | -0.0179 | -5.29 |
| 472 | 25 | 3458.8030 | 577.4744 | 18239.02 | 6 |  |  |  |  |  |
| 472 | 26 | 3303.7385 | 661.7550 | 19735.08 | 5 | 3303.7583 | C27 | 27 | -0.0198 | -6.00 |
| 472 | 27 | 3025.6506 | 1009.5575 | 18288.77 | 3 | 3025.6680 | C25 | 25 | -0.0174 | -5.76 |
| 472 | 28 | 3003.5367 | 751.8914 | 16346.03 | 4 | 3003.5495 | Z\_DOT26 | 4 | -0.0128 | -4.26 |
| 472 | 29 | 3004.5446 | 1002.5221 | 15287.52 | 3 |  |  |  |  |  |
| 472 | 30 | 695.5665 | 696.5737 | 122550.53 | 1 |  |  |  |  |  |
| 472 | 31 | 1204.6624 | 603.3385 | 21384.04 | 2 | 1204.6636 | Z\_DOT12 | 18 | -1.25e-03 | -1.04 |
| 472 | 32 | 3460.8136 | 866.2107 | 17188.91 | 4 |  |  |  |  |  |
| 472 | 33 | 2698.4268 | 675.6140 | 15142.47 | 4 | 2698.4410 | C22 | 22 | -0.0142 | -5.27 |
| 472 | 34 | 2960.4832 | 741.1281 | 14392.47 | 4 |  |  |  |  |  |
| 472 | 35 | 1333.7041 | 667.8593 | 18120.07 | 2 | 1333.7062 | Z\_DOT13 | 17 | -2.09e-03 | -1.57 |
| 472 | 36 | 2400.2623 | 801.0947 | 14029.29 | 3 | 2400.2769 | C19 | 19 | -0.0146 | -6.08 |
| 472 | 37 | 1738.8846 | 870.4496 | 16147.09 | 2 | 1738.8970 | C14 | 14 | -0.0125 | -7.17 |
| 472 | 38 | 2030.0436 | 677.6885 | 12556.09 | 3 | 2030.0553 | C16 | 16 | -0.0117 | -5.77 |
| 472 | 39 | 1135.5418 | 568.7782 | 16209.53 | 2 | 1135.5477 | C9 | 9 | -5.96e-03 | -5.25 |
| 472 | 40 | 1488.7459 | 745.3802 | 14692.89 | 2 | 1488.7540 | C12 | 12 | -8.16e-03 | -5.48 |
| 472 | 41 | 1390.7318 | 696.3732 | 141144.81 | 2 |  |  |  |  |  |
| 472 | 42 | 650.3118 | 651.3191 | 11245.76 | 1 | 650.3096 | Z\_DOT7 | 23 | 2.20e-03 | 3.38 |
| 472 | 43 | 847.4552 | 848.4625 | 7558.40 | 1 | 847.4585 | C7 | 7 | -3.33e-03 | -3.93 |
| 472 | 44 | 1220.6811 | 611.3478 | 8479.58 | 2 |  |  |  |  |  |
| 472 | 45 | 1135.5419 | 1136.5491 | 7841.87 | 1 | 1135.5477 | C9 | 9 | -5.89e-03 | -5.19 |
| 472 | 46 | 1274.6911 | 638.3528 | 4841.85 | 2 |  |  |  |  |  |
| 472 | 47 | 473.2940 | 474.3013 | 14385.33 | 1 | 473.2961 | C4 | 4 | -2.13e-03 | -4.49 |
| 472 | 48 | 1007.4841 | 1008.4914 | 5247.82 | 1 | 1007.4892 | C8 | 8 | -5.05e-03 | -5.01 |
| 472 | 49 | 1488.7457 | 497.2559 | 7202.45 | 3 | 1488.7540 | C12 | 12 | -8.30e-03 | -5.57 |
| 472 | 50 | 1446.7909 | 724.4027 | 8517.58 | 2 | 1446.7903 | Z\_DOT14 | 16 | 6.42e-04 | 0.44 |
| 472 | 51 | 564.0607 | 565.0679 | 7828.04 | 1 |  |  |  |  |  |
| 472 | 52 | 870.2116 | 871.2189 | 22784.10 | 1 |  |  |  |  |  |
| 472 | 53 | 778.4058 | 779.4131 | 6065.69 | 1 | 778.4046 | Z\_DOT8 | 22 | 1.23e-03 | 1.59 |
| 472 | 54 | 976.4926 | 489.2536 | 4497.26 | 2 |  |  |  |  |  |
| 472 | 55 | 1417.7487 | 709.8816 | 2289.40 | 2 |  |  |  |  |  |
| 472 | 56 | 344.2522 | 345.2595 | 2571.70 | 1 | 344.2535 | C3 | 3 | -1.35e-03 | -3.91 |
| 472 | 57 | 724.3884 | 725.3956 | 2640.80 | 1 |  |  |  |  |  |

  

All proteins /
CsTx-13a Cupiennius salei toxin 13 isoform a /
Proteoform #40
