# Supplementary material for: The Dual Prey-Inactivation Strategy of Spiders—In-Depth Venomic Analysis of Cupiennius salei
Source: Toxins (Basel). 2019 Mar 19;11(3):167. doi: 10.3390/toxins11030167 (PMC6468893; doi:10.3390/toxins11030167)
Supplement: Supplementary file 1 [file toxins-11-00167-s001.zip › Supplementary Dataset EV1/20180328_f2_topdown_OTMS2_EThcD_NL_i02_ms2_proteoform_cutoff_html/prsms/prsm119.html]

Protein-Spectrum-Match for Spectrum #354


All proteins /
CsTx-12a\_S1 Cupiennius salei toxin 12 isoform a S1^ACsTx-12a\_S2 Cupiennius salei toxin 12 isoform a S2 /
Proteoform #18

## Protein-Spectrum-Match #119 for Spectrum #354

|  |  |  |  |  |  |
| --- | --- | --- | --- | --- | --- |
| PrSM ID: | 119 | Scan(s): | 475 | Precursor charge: | 6 |
| Precursor m/z: | 729.3079 | Precursor mass: | 4369.8038 | Proteoform mass: | 4369.8061 |
| # matched peaks: | 33 | # matched fragment ions: | 29 | # unexpected modifications: | 0 |
| E-value: | 2.38e-28 | P-value: | 2.38e-28 | Q-value (Spectral FDR): | 0 |

  

|  |  |  |  |  |  |  |  |  |  |  |  |  |  |  |  |  |  |  |  |  |  |  |  |  |  |  |  |  |  |  |  |  |  |  |  |  |  |  |  |  |  |  |  |  |  |  |  |  |  |  |  |  |  |  |  |  |  |  |  |  |  |  |  |  |  |  |  |  |  |
| --- | --- | --- | --- | --- | --- | --- | --- | --- | --- | --- | --- | --- | --- | --- | --- | --- | --- | --- | --- | --- | --- | --- | --- | --- | --- | --- | --- | --- | --- | --- | --- | --- | --- | --- | --- | --- | --- | --- | --- | --- | --- | --- | --- | --- | --- | --- | --- | --- | --- | --- | --- | --- | --- | --- | --- | --- | --- | --- | --- | --- | --- | --- | --- | --- | --- | --- | --- | --- | --- |
|  | |  | | | | | | | | | | | | | | | | | | | | | | | | | | | | | | | | | | | | | | | | | | | | | | | | | | | | | | | | | | | | | | | | | | | |
| 1 |  |  | M |  | K |  | V |  | L |  | V |  | I |  | C |  | A |  | V |  | L |  |  | F |  | L |  | T |  | I |  | F |  | S |  | N |  | S |  | S |  | A |  |  | E |  | T |  | E |  | D |  | D |  | F |  | L |  | E |  | D |  | E |  | 30 |  |
|  | |  | | | | | | | | | | | | | | | | | | | | | | | | | | | | | | | | | | | | | | | | | | | | | | | | | | | | | | | | | | | | | | | | | | | |
| 31 |  |  | S |  | F |  | E |  | A |  | D |  | D |  | V |  | I |  | P |  | F |  |  | L |  | A |  | R |  | E |  | Q |  | V |  | R | ] | S |  | D |  | C |  |  | T |  | L |  | R | ⎱ | N | ⎩ | H | ⎫ | D | ⎫ | C | ⎫ | T | ⎫ | D | ⎱ | D |  | 60 |  |
|  | |  | | | | | | | | | | | | | | | | | | | | | | | | | | | | | | | | | | | | | | | | | | | | | | | | | | | | | | | | | | | | | | | | | | | |
| 61 |  | ⎱ | R |  | H |  | S | ⎫ | C |  | C | ⎫ | R | ⎱ | S | ⎱ | K | ⎫ | M |  | F |  |  | K | ⎱ | D | ⎱ | V | ⎫ | C | ⎫ | K | ⎫ | C | ⎫ | F | ⎫ | Y |  | P | ⎫ | S |  | ⎫ | Q | [ | R |  | S |  | D |  | T |  | A |  | R |  | A |  | K |  | K |  | 90 |  |
|  | |  | | | | | | | | | | | | | | | | | | | | | | | | | | | | | | | | | | | | | | | | | | | | | | | | | | | | | | | | | | | | | | | | | | | |
| 91 |  |  | E |  | L |  | C |  | T |  | C |  | Q |  | Q |  | D |  | K |  | H |  |  | L |  | K |  | F |  | I |  | E |  | K |  | G |  | L |  | Q |  | K |  |  | A |  | K |  | V |  | L |  | V |  | A |  | G |  | | 117 |  | | | | | |

Fixed PTMs: Carbamidomethylation [C50 C57 C64 C65 C74 C76 ]

  

All peaks (72)  Matched peaks (33)  Not matched peaks (39)

  

| Scan | Peak | Mono mass | Mono m/z | Intensity | Charge | Theoretical mass | Ion | Pos | Mass error | PPM error |
| --- | --- | --- | --- | --- | --- | --- | --- | --- | --- | --- |
| 475 | 1 | 4312.7572 | 863.5587 | 210682.76 | 5 |  |  |  |  |  |
| 475 | 2 | 1456.9301 | 729.4723 | 261329.50 | 2 |  |  |  |  |  |
| 475 | 3 | 4312.7578 | 1079.1967 | 40080.90 | 4 |  |  |  |  |  |
| 475 | 4 | 3586.4935 | 897.6307 | 29969.27 | 4 | 3586.5162 | C28 | 28 | -0.0227 | -6.34 |
| 475 | 5 | 2293.9965 | 765.6728 | 33672.06 | 3 |  |  |  |  |  |
| 475 | 6 | 4353.7627 | 871.7598 | 26694.14 | 5 |  |  |  |  |  |
| 475 | 7 | 3893.5882 | 974.4043 | 19762.98 | 4 | 3893.6153 | C30 | 30 | -0.0271 | -6.96 |
| 475 | 8 | 4240.7357 | 849.1544 | 21643.01 | 5 | 4240.7634 | C33 | 33 | -0.0278 | -6.55 |
| 475 | 9 | 2462.9489 | 821.9902 | 25619.90 | 3 | 2462.9644 | C19 | 19 | -0.0156 | -6.33 |
| 475 | 10 | 2914.1863 | 729.5539 | 369112.25 | 4 |  |  |  |  |  |
| 475 | 11 | 1986.7902 | 994.4024 | 27899.13 | 2 | 1986.8020 | C16 | 16 | -0.0118 | -5.93 |
| 475 | 12 | 4061.7025 | 1016.4329 | 17559.63 | 4 |  |  |  |  |  |
| 475 | 13 | 3084.2752 | 772.0761 | 20617.77 | 4 | 3084.2953 | C24 | 24 | -0.0201 | -6.51 |
| 475 | 14 | 4221.7507 | 845.3574 | 14915.21 | 5 |  |  |  |  |  |
| 475 | 15 | 4255.7334 | 1064.9406 | 15482.72 | 4 |  |  |  |  |  |
| 475 | 16 | 2549.9803 | 851.0007 | 16446.54 | 3 | 2549.9965 | C20 | 20 | -0.0161 | -6.32 |
| 475 | 17 | 3458.3971 | 865.6065 | 18616.29 | 4 | 3458.4213 | C27 | 27 | -0.0242 | -7.00 |
| 475 | 18 | 3746.5216 | 937.6377 | 15288.63 | 4 | 3746.5469 | C29 | 29 | -0.0253 | -6.76 |
| 475 | 19 | 874.1570 | 875.1643 | 30191.61 | 1 |  |  |  |  |  |
| 475 | 20 | 1491.5743 | 746.7944 | 16401.64 | 2 | 1491.5830 | C12 | 12 | -8.77e-03 | -5.88 |
| 475 | 21 | 1907.8382 | 954.9264 | 13169.99 | 2 | 1907.8495 | Z\_DOT15 | 19 | -0.0113 | -5.91 |
| 475 | 22 | 4354.7710 | 1089.7000 | 11830.29 | 4 |  |  |  |  |  |
| 475 | 23 | 4263.7594 | 853.7591 | 9802.94 | 5 |  |  |  |  |  |
| 475 | 24 | 4326.7635 | 866.3600 | 10266.08 | 5 |  |  |  |  |  |
| 475 | 25 | 4280.7815 | 857.1636 | 9369.58 | 5 |  |  |  |  |  |
| 475 | 26 | 1606.6010 | 804.3078 | 11124.60 | 2 | 1606.6100 | C13 | 13 | -8.98e-03 | -5.59 |
| 475 | 27 | 2879.2140 | 960.7453 | 9085.01 | 3 | 2879.2309 | Z\_DOT22 | 12 | -0.0168 | -5.85 |
| 475 | 28 | 3621.4391 | 906.3670 | 7348.53 | 4 | 3621.4649 | Z\_DOT28 | 6 | -0.0259 | -7.15 |
| 475 | 29 | 3199.3018 | 800.8327 | 10611.61 | 4 | 3199.3222 | C25 | 25 | -0.0204 | -6.38 |
| 475 | 30 | 2895.2349 | 724.8160 | 8678.87 | 4 |  |  |  |  |  |
| 475 | 31 | 2185.8955 | 1093.9550 | 15755.24 | 2 |  |  |  |  |  |
| 475 | 32 | 3298.3697 | 825.5997 | 8245.17 | 4 | 3298.3906 | C26 | 26 | -0.0210 | -6.36 |
| 475 | 33 | 2133.9660 | 712.3293 | 8051.73 | 3 |  |  |  |  |  |
| 475 | 34 | 4267.7398 | 1067.9422 | 7164.77 | 4 |  |  |  |  |  |
| 475 | 35 | 2306.8510 | 1154.4328 | 7117.30 | 2 | 2306.8633 | C18 | 18 | -0.0123 | -5.33 |
| 475 | 36 | 4240.7354 | 1061.1911 | 5719.20 | 4 | 4240.7634 | C33 | 33 | -0.0280 | -6.61 |
| 475 | 37 | 2678.0740 | 893.6986 | 5965.35 | 3 | 2678.0914 | C21 | 21 | -0.0174 | -6.51 |
| 475 | 38 | 4296.7456 | 1075.1937 | 5384.71 | 4 |  |  |  |  |  |
| 475 | 39 | 1376.5480 | 689.2813 | 5939.90 | 2 | 1376.5561 | C11 | 11 | -8.08e-03 | -5.87 |
| 475 | 40 | 4351.7679 | 726.3019 | 6753.54 | 6 |  |  |  |  |  |
| 475 | 41 | 1820.8073 | 911.4109 | 7054.59 | 2 | 1820.8174 | Z\_DOT14 | 20 | -0.0101 | -5.56 |
| 475 | 42 | 3371.3483 | 1124.7901 | 4461.11 | 3 |  |  |  |  |  |
| 475 | 43 | 3084.2766 | 1029.0995 | 5622.73 | 3 | 3084.2953 | C24 | 24 | -0.0187 | -6.05 |
| 475 | 44 | 4061.7001 | 813.3473 | 7424.21 | 5 |  |  |  |  |  |
| 475 | 45 | 728.0745 | 729.0818 | 29796.85 | 1 |  |  |  |  |  |
| 475 | 46 | 4326.7754 | 1082.7011 | 4715.03 | 4 |  |  |  |  |  |
| 475 | 47 | 1474.5478 | 738.2812 | 4441.82 | 2 |  |  |  |  |  |
| 475 | 48 | 4004.6806 | 1002.1774 | 4918.27 | 4 |  |  |  |  |  |
| 475 | 49 | 3507.3993 | 877.8571 | 4630.53 | 4 | 3507.4220 | Z\_DOT27 | 7 | -0.0227 | -6.48 |
| 475 | 50 | 4222.7442 | 1056.6933 | 7212.13 | 4 |  |  |  |  |  |
| 475 | 51 | 2764.1885 | 922.4034 | 7264.52 | 3 | 2764.2039 | Z\_DOT21 | 13 | -0.0155 | -5.59 |
| 475 | 52 | 4313.7679 | 1438.9299 | 3367.30 | 3 |  |  |  |  |  |
| 475 | 53 | 4153.7083 | 831.7489 | 4839.52 | 5 | 4153.7314 | C32 | 32 | -0.0232 | -5.57 |
| 475 | 54 | 982.4162 | 983.4235 | 4145.65 | 1 |  |  |  |  |  |
| 475 | 55 | 1000.4452 | 1001.4525 | 4273.17 | 1 | 1000.4508 | C8 | 8 | -5.64e-03 | -5.64 |
| 475 | 56 | 749.3449 | 750.3522 | 7225.83 | 1 | 749.3490 | C6 | 6 | -4.08e-03 | -5.44 |
| 475 | 57 | 1275.5009 | 638.7577 | 2960.59 | 2 | 1275.5084 | C10 | 10 | -7.52e-03 | -5.90 |
| 475 | 58 | 1115.4717 | 558.7431 | 3170.43 | 2 | 1115.4778 | C9 | 9 | -6.04e-03 | -5.41 |
| 475 | 59 | 694.2922 | 695.2994 | 3208.30 | 1 |  |  |  |  |  |
| 475 | 60 | 822.3862 | 823.3935 | 1663.29 | 1 |  |  |  |  |  |
| 475 | 61 | 330.1526 | 331.1599 | 3064.07 | 1 |  |  |  |  |  |
| 475 | 62 | 710.1128 | 711.1201 | 1513.37 | 1 |  |  |  |  |  |
| 475 | 63 | 1286.5123 | 1287.5196 | 2080.08 | 1 | 1286.5186 | Z\_DOT10 | 24 | -6.31e-03 | -4.91 |
| 475 | 64 | 1065.4363 | 1066.4435 | 1703.73 | 1 |  |  |  |  |  |
| 475 | 65 | 1115.4740 | 1116.4813 | 1302.47 | 1 | 1115.4778 | C9 | 9 | -3.77e-03 | -3.38 |
| 475 | 66 | 1000.4453 | 501.2299 | 3596.51 | 2 | 1000.4508 | C8 | 8 | -5.53e-03 | -5.53 |
| 475 | 67 | 766.3353 | 767.3425 | 1596.96 | 1 |  |  |  |  |  |
| 475 | 68 | 576.2183 | 577.2256 | 880.30 | 1 |  |  |  |  |  |
| 475 | 69 | 1171.4822 | 1172.4894 | 1614.59 | 1 | 1171.4917 | Z\_DOT9 | 25 | -9.52e-03 | -8.13 |
| 475 | 70 | 1093.6963 | 1094.7036 | 7499.42 | 1 |  |  |  |  |  |
| 475 | 71 | 493.2146 | 494.2219 | 1366.17 | 1 |  |  |  |  |  |
| 475 | 72 | 517.0908 | 518.0981 | 1240.73 | 1 |  |  |  |  |  |

  

All proteins /
CsTx-12a\_S1 Cupiennius salei toxin 12 isoform a S1^ACsTx-12a\_S2 Cupiennius salei toxin 12 isoform a S2 /
Proteoform #18
