# Supplementary material for: The Dual Prey-Inactivation Strategy of Spiders—In-Depth Venomic Analysis of Cupiennius salei
Source: Toxins (Basel). 2019 Mar 19;11(3):167. doi: 10.3390/toxins11030167 (PMC6468893; doi:10.3390/toxins11030167)
Supplement: Supplementary file 1 [file toxins-11-00167-s001.zip › Supplementary Dataset EV1/20180328_f2_topdown_OTMS2_EThcD_NL_i02_ms2_proteoform_cutoff_html/prsms/prsm12.html]

Protein-Spectrum-Match for Spectrum #222


All proteins /
CsTx-12b Cupiennius salei toxin 12 isoform b /
Proteoform #100

## Protein-Spectrum-Match #12 for Spectrum #222

|  |  |  |  |  |  |
| --- | --- | --- | --- | --- | --- |
| PrSM ID: | 12 | Scan(s): | 299 | Precursor charge: | 6 |
| Precursor m/z: | 734.3052 | Precursor mass: | 4399.7874 | Proteoform mass: | 4399.8474 |
| # matched peaks: | 14 | # matched fragment ions: | 12 | # unexpected modifications: | 1 |
| E-value: | 2.08e-09 | P-value: | 2.08e-09 | Q-value (Spectral FDR): | 0 |

  

|  |  |  |  |  |  |  |  |  |  |  |  |  |  |  |  |  |  |  |  |  |  |  |  |  |  |  |  |  |  |  |  |  |  |  |  |  |  |  |  |  |  |  |  |  |  |  |  |  |  |  |  |  |  |  |  |  |  |  |  |  |  |  |  |  |  |  |  |  |  |
| --- | --- | --- | --- | --- | --- | --- | --- | --- | --- | --- | --- | --- | --- | --- | --- | --- | --- | --- | --- | --- | --- | --- | --- | --- | --- | --- | --- | --- | --- | --- | --- | --- | --- | --- | --- | --- | --- | --- | --- | --- | --- | --- | --- | --- | --- | --- | --- | --- | --- | --- | --- | --- | --- | --- | --- | --- | --- | --- | --- | --- | --- | --- | --- | --- | --- | --- | --- | --- | --- |
|  | |  | | | | | | | | | | | | | | | | | | | | | | | | | | | | | | | | | | | | | | | | | | | | | | | | | | | | | | | | | | | | | | | | | | | |
| 1 |  |  | M |  | K |  | V |  | L |  | V |  | I |  | C |  | A |  | V |  | L |  |  | F |  | L |  | A |  | I |  | F |  | S |  | N |  | S |  | S |  | A |  |  | E |  | T |  | E |  | D |  | D |  | F |  | L |  | E |  | D |  | E |  | 30 |  |
|  | |  | | | | | | | | | | | | | | | | | | | | | | | | | | | | | | | | | | | | | | | | | | | | | | | | | | | | | | | | | | | | | | | | | | | |
| 31 |  |  | S |  | F |  | E |  | A |  | D |  | D |  | V |  | I |  | P |  | F |  |  | L |  | A |  | R |  | E |  | Q |  | V |  | R | ] | S | ⎩ | D |  | C |  |  | T | ⎫ | L | ⎫ | R | ⎫ | N |  | H | ⎫ | D | ⎫ | C | ⎫ | T | ⎫ | D | ⎫ | D |  | 60 |  |
|  | |  | | | | | | | | | | | | | | | | | 342.18 | | | | | | | | | | | | | | | | | | | | | | | | | | | | | | | | | | | | | | | | | | | | | | | |
| 61 |  | ⎫ | R |  | H |  | S |  | C |  | C |  | R |  | S |  | K | ⎫ | M | ⎫ | F |  |  | K |  | D |  | V |  | C |  | K |  | C |  | F |  | Y | [ | P |  | S |  |  | Q |  | R |  | S |  | D |  | T |  | A |  | R |  | A |  | K |  | K |  | 90 |  |
|  | |  | | | | | | | | | | | | | | | | | | | | | | | | | | | | | | | | | | | | | | | | | | | | | | | | | | | | | | | | | | | | | | | | | | | |
| 91 |  |  | E |  | L |  | C |  | T |  | C |  | Q |  | Q |  | D |  | K |  | H |  |  | L |  | K |  | Y |  | I |  | E |  | K |  | G |  | L |  | Q |  | K |  |  | A |  | K |  | V |  | L |  | V |  | A |  | G |  | | 117 |  | | | | | |

Fixed PTMs: Carbamidomethylation [C50 C57 C64 C65 C74 C76 ]   
  
     Unexpected modifications:   Unknown [342.18]

  

All peaks (73)  Matched peaks (14)  Not matched peaks (59)

  

| Scan | Peak | Mono mass | Mono m/z | Intensity | Charge | Theoretical mass | Ion | Pos | Mass error | PPM error |
| --- | --- | --- | --- | --- | --- | --- | --- | --- | --- | --- |
| 299 | 1 | 4276.7500 | 713.7989 | 1506479.22 | 6 |  |  |  |  |  |
| 299 | 2 | 4294.7460 | 716.7983 | 2100025.59 | 6 |  |  |  |  |  |
| 299 | 3 | 3964.5959 | 793.9265 | 1614750.75 | 5 |  |  |  |  |  |
| 299 | 4 | 4237.7230 | 848.5519 | 795993.79 | 5 |  |  |  |  |  |
| 299 | 5 | 4295.7485 | 860.1570 | 836835.11 | 5 |  |  |  |  |  |
| 299 | 6 | 4148.6770 | 830.7427 | 837109.70 | 5 |  |  |  |  |  |
| 299 | 7 | 4342.7459 | 869.5564 | 716097.99 | 5 |  |  |  |  |  |
| 299 | 8 | 4130.6673 | 827.1407 | 154447.76 | 5 |  |  |  |  |  |
| 299 | 9 | 4278.7295 | 856.7532 | 122027.79 | 5 |  |  |  |  |  |
| 299 | 10 | 4061.6433 | 813.3359 | 120973.33 | 5 |  |  |  |  |  |
| 299 | 11 | 3801.5258 | 761.3124 | 113025.33 | 5 |  |  |  |  |  |
| 299 | 12 | 4385.7500 | 878.1573 | 112479.22 | 5 |  |  |  |  |  |
| 299 | 13 | 4259.7285 | 710.9620 | 104486.30 | 6 |  |  |  |  |  |
| 299 | 14 | 2200.3846 | 734.4688 | 149970.79 | 3 |  |  |  |  |  |
| 299 | 15 | 3936.6018 | 788.3276 | 95897.22 | 5 |  |  |  |  |  |
| 299 | 16 | 4205.7500 | 842.1573 | 82926.80 | 5 |  |  |  |  |  |
| 299 | 17 | 3964.5933 | 992.1556 | 84932.71 | 4 |  |  |  |  |  |
| 299 | 18 | 2761.1214 | 691.2876 | 86693.62 | 4 |  |  |  |  |  |
| 299 | 19 | 3986.6564 | 798.3386 | 75606.66 | 5 |  |  |  |  |  |
| 299 | 20 | 2820.1976 | 706.0567 | 69850.27 | 4 |  |  |  |  |  |
| 299 | 21 | 3250.3787 | 813.6020 | 76449.27 | 4 |  |  |  |  |  |
| 299 | 22 | 3986.6685 | 997.6744 | 58868.47 | 4 |  |  |  |  |  |
| 299 | 23 | 3818.5553 | 955.6461 | 65709.26 | 4 |  |  |  |  |  |
| 299 | 24 | 1474.5525 | 738.2835 | 85785.94 | 2 |  |  |  |  |  |
| 299 | 25 | 4165.6960 | 834.1465 | 42399.58 | 5 |  |  |  |  |  |
| 299 | 26 | 4219.7136 | 844.9500 | 52765.70 | 5 |  |  |  |  |  |
| 299 | 27 | 3801.5296 | 951.3897 | 68458.74 | 4 |  |  |  |  |  |
| 299 | 28 | 4188.7243 | 838.7521 | 47153.15 | 5 |  |  |  |  |  |
| 299 | 29 | 3511.4496 | 878.8697 | 53624.19 | 4 |  |  |  |  |  |
| 299 | 30 | 3671.4859 | 918.8788 | 45047.87 | 4 |  |  |  |  |  |
| 299 | 31 | 3036.2828 | 760.0780 | 42997.95 | 4 |  |  |  |  |  |
| 299 | 32 | 4091.6878 | 819.3448 | 42048.87 | 5 |  |  |  |  |  |
| 299 | 33 | 4061.6440 | 677.9479 | 37079.92 | 6 |  |  |  |  |  |
| 299 | 34 | 4148.6754 | 692.4532 | 38826.92 | 6 |  |  |  |  |  |
| 299 | 35 | 4382.7472 | 731.4651 | 36308.09 | 6 |  |  |  |  |  |
| 299 | 36 | 4251.7251 | 851.3523 | 42920.30 | 5 |  |  |  |  |  |
| 299 | 37 | 330.1537 | 331.1610 | 116167.59 | 1 |  |  |  |  |  |
| 299 | 38 | 3410.4076 | 853.6092 | 35814.84 | 4 |  |  |  |  |  |
| 299 | 39 | 1491.5796 | 746.7971 | 47024.97 | 2 | 1491.5830 | C12 | 12 | -3.40e-03 | -2.28 |
| 299 | 40 | 3964.5882 | 661.7720 | 24276.28 | 6 |  |  |  |  |  |
| 299 | 41 | 2218.9535 | 740.6584 | 26523.97 | 3 |  |  |  |  |  |
| 299 | 42 | 3546.4022 | 887.6078 | 28906.17 | 4 |  |  |  |  |  |
| 299 | 43 | 1376.5533 | 689.2839 | 36864.94 | 2 | 1376.5561 | C11 | 11 | -2.83e-03 | -2.06 |
| 299 | 44 | 3151.3179 | 788.8368 | 31881.26 | 4 | 3151.3166 | C22 | 22 | 1.36e-03 | 0.43 |
| 299 | 45 | 880.3555 | 881.3628 | 42690.30 | 1 |  |  |  |  |  |
| 299 | 46 | 4296.7598 | 1075.1972 | 21479.91 | 4 | 4296.7967 | Z\_DOT30 | 1 | -0.0369 | -8.58 |
| 299 | 47 | 2802.1822 | 701.5528 | 22639.47 | 4 |  |  |  |  |  |
| 299 | 48 | 1606.6056 | 804.3101 | 27016.34 | 2 | 1606.6100 | C13 | 13 | -4.34e-03 | -2.70 |
| 299 | 49 | 4325.7290 | 866.1531 | 21968.53 | 5 |  |  |  |  |  |
| 299 | 50 | 2908.1858 | 728.0537 | 28152.70 | 4 |  |  |  |  |  |
| 299 | 51 | 2058.9232 | 687.3150 | 18844.99 | 3 |  |  |  |  |  |
| 299 | 52 | 2804.1728 | 935.7316 | 19633.95 | 3 |  |  |  |  |  |
| 299 | 53 | 2678.0831 | 893.7017 | 20413.48 | 3 | 2678.0914 | C21 | 21 | -8.29e-03 | -3.10 |
| 299 | 54 | 1000.4488 | 501.2317 | 19317.11 | 2 | 1000.4508 | C8 | 8 | -1.99e-03 | -1.99 |
| 299 | 55 | 1115.4755 | 558.7450 | 15339.60 | 2 | 1115.4778 | C9 | 9 | -2.26e-03 | -2.02 |
| 299 | 56 | 749.3473 | 750.3546 | 26569.87 | 1 | 749.3490 | C6 | 6 | -1.64e-03 | -2.18 |
| 299 | 57 | 1275.5049 | 638.7597 | 10396.58 | 2 | 1275.5084 | C10 | 10 | -3.49e-03 | -2.74 |
| 299 | 58 | 1000.4487 | 1001.4560 | 6725.96 | 1 | 1000.4508 | C8 | 8 | -2.10e-03 | -2.10 |
| 299 | 59 | 830.0135 | 831.0208 | 11039.53 | 1 |  |  |  |  |  |
| 299 | 60 | 493.2165 | 494.2238 | 7817.30 | 1 |  |  |  |  |  |
| 299 | 61 | 312.1432 | 313.1505 | 9235.45 | 1 |  |  |  |  |  |
| 299 | 62 | 694.2951 | 695.3024 | 4513.99 | 1 |  |  |  |  |  |
| 299 | 63 | 712.7958 | 713.8031 | 222192.90 | 1 |  |  |  |  |  |
| 299 | 64 | 955.4279 | 478.7212 | 2916.82 | 2 |  |  |  |  |  |
| 299 | 65 | 983.4222 | 492.7184 | 4124.77 | 2 |  |  |  |  |  |
| 299 | 66 | 576.2201 | 577.2274 | 2221.22 | 1 |  |  |  |  |  |
| 299 | 67 | 480.1633 | 481.1706 | 3671.87 | 1 | 480.1638 | C4 | 4 | -4.57e-04 | -0.95 |
| 299 | 68 | 233.1011 | 234.1084 | 4265.25 | 1 |  |  |  |  |  |
| 299 | 69 | 593.2472 | 594.2545 | 2877.08 | 1 | 593.2479 | C5 | 5 | -6.85e-04 | -1.15 |
| 299 | 70 | 1141.4927 | 571.7536 | 1663.61 | 2 |  |  |  |  |  |
| 299 | 71 | 1115.4781 | 1116.4854 | 2035.79 | 1 | 1115.4778 | C9 | 9 | 3.81e-04 | 0.34 |
| 299 | 72 | 361.1270 | 362.1343 | 3015.17 | 1 |  |  |  |  |  |
| 299 | 73 | 1387.5638 | 1388.5711 | 1180.55 | 1 |  |  |  |  |  |

  

All proteins /
CsTx-12b Cupiennius salei toxin 12 isoform b /
Proteoform #100
