# Supplementary material for: The Dual Prey-Inactivation Strategy of Spiders—In-Depth Venomic Analysis of Cupiennius salei
Source: Toxins (Basel). 2019 Mar 19;11(3):167. doi: 10.3390/toxins11030167 (PMC6468893; doi:10.3390/toxins11030167)
Supplement: Supplementary file 1 [file toxins-11-00167-s001.zip › Supplementary Dataset EV1/20180328_f2_topdown_OTMS2_EThcD_NL_i02_ms2_proteoform_cutoff_html/prsms/prsm120.html]

Protein-Spectrum-Match for Spectrum #355


All proteins /
CsTx-1a\_S1 Cupiennius salei toxin 1 isoform a S1^ACsTx-1a\_S2 Cupiennius salei toxin 1 isoform a S2 /
Proteoform #42

## Protein-Spectrum-Match #120 for Spectrum #355

|  |  |  |  |  |  |
| --- | --- | --- | --- | --- | --- |
| PrSM ID: | 120 | Scan(s): | 476 | Precursor charge: | 8 |
| Precursor m/z: | 899.0295 | Precursor mass: | 7184.1781 | Proteoform mass: | 7184.1281 |
| # matched peaks: | 29 | # matched fragment ions: | 26 | # unexpected modifications: | 1 |
| E-value: | 9.49e-22 | P-value: | 9.49e-22 | Q-value (Spectral FDR): | 0 |

  

|  |  |  |  |  |  |  |  |  |  |  |  |  |  |  |  |  |  |  |  |  |  |  |  |  |  |  |  |  |  |  |  |  |  |  |  |  |  |  |  |  |  |  |  |  |  |  |  |  |  |  |  |  |  |  |  |  |  |  |  |  |  |  |  |  |  |  |  |  |  |
| --- | --- | --- | --- | --- | --- | --- | --- | --- | --- | --- | --- | --- | --- | --- | --- | --- | --- | --- | --- | --- | --- | --- | --- | --- | --- | --- | --- | --- | --- | --- | --- | --- | --- | --- | --- | --- | --- | --- | --- | --- | --- | --- | --- | --- | --- | --- | --- | --- | --- | --- | --- | --- | --- | --- | --- | --- | --- | --- | --- | --- | --- | --- | --- | --- | --- | --- | --- | --- | --- |
|  | |  | | | | | | | | | | | | | | | | | | | | | | | | | | | | | | | | | | | | | | | | | | | | | | | | | | | | | | | | | | | | | | | | | | | |
| 1 |  |  | M |  | K |  | V |  | L |  | I |  | I |  | S |  | A |  | V |  | L |  |  | F |  | I |  | T |  | I |  | F |  | S |  | N |  | I |  | S |  | A |  |  | E |  | I |  | E |  | D |  | D |  | F |  | L |  | E |  | D |  | E |  | 30 |  |
|  | |  | | | | | | | | | | | | | | | | | | | | | | | | | | | | | | | | | | | | | | | | | | | | | | | | | | | | | | | | | | | | | | | | | | | |
| 31 |  |  | S |  | F |  | E |  | A |  | E |  | D |  | I |  | I |  | P |  | F |  |  | F |  | E |  | N |  | E |  | Q |  | A |  | R | ] | S | ⎩ | C |  | I |  |  | P |  | K | ⎫ | H | ⎫ | E | ⎩ | E | ⎫ | C |  | T | ⎫ | N | ⎱ | D |  | K |  | 60 |  |
|  | |  | | | | | | | | | | | | | | | | | | | | | | | | | | | | | | | | | | | | | | | | | | | | | | | | | | | | | | | | | | | | | | | | | | | |
| 61 |  |  | H | ⎫ | N | ⎫ | C | ⎫ | C |  | R |  | K |  | G |  | L | ⎫ | F | ⎫ | K |  | ⎫ | L |  | K | ⎫ | C | ⎫ | Q | ⎫ | C |  | S |  | T |  | F | ⎫ | D |  | D |  | ⎫ | E |  | S |  | G |  | Q |  | P |  | T |  | E |  | R |  | C |  | A |  | 90 |  |
|  | |  | | | | | | | | | 14.94 | | | | | | | | | | | | | | | | | | | | | | | | | | | | | | | | | | | | | | | | | | | | | | | | | | | | | | | |
| 91 |  |  | C |  | G | ⎫ | R |  | P | ⎫ | M | ⎫ | G | ⎫ | H |  | Q | ⎫ | A |  | I |  |  | E |  | T |  | G |  | L |  | N | ⎫ | I | ⎫ | F | [ | R |  | G |  | L |  |  | F |  | K |  | G |  | K |  | K |  | K |  | N |  | K |  | K |  | T |  | 120 |  |
|  | |  | | | | | | | | | | | | | | | | | | | | | | | | | | | | | | | | | | | | | | | | | | | | | | | | | | | | | | | | | | | | | | | | | | | |
| 121 |  |  | K |  | G |  | | | | 122 |  | | | | | | | | | | | | | | | | | | | | | | | | | | | | | | | | | | | | | | | | | | | | | | | | | | | | | | | |

Fixed PTMs: Carbamidomethylation [C49 C56 C63 C64 C73 C75 C89 C91 ]   
  
     Unexpected modifications:   Unknown [14.94]

  

All peaks (101)  Matched peaks (29)  Not matched peaks (72)

  

| Scan | Peak | Mono mass | Mono m/z | Intensity | Charge | Theoretical mass | Ion | Pos | Mass error | PPM error |
| --- | --- | --- | --- | --- | --- | --- | --- | --- | --- | --- |
| 476 | 1 | 7128.1069 | 1019.3083 | 56649.81 | 7 |  |  |  |  |  |
| 476 | 2 | 7128.1077 | 1189.0252 | 26279.35 | 6 |  |  |  |  |  |
| 476 | 3 | 7169.1162 | 1025.1667 | 11122.73 | 7 |  |  |  |  |  |
| 476 | 4 | 3592.5633 | 899.1481 | 34957.49 | 4 |  |  |  |  |  |
| 476 | 5 | 7141.1162 | 1021.1667 | 9550.73 | 7 |  |  |  |  |  |
| 476 | 6 | 3593.5714 | 1198.8644 | 10768.65 | 3 |  |  |  |  |  |
| 476 | 7 | 6907.9642 | 987.8593 | 8198.76 | 7 |  |  |  |  |  |
| 476 | 8 | 7170.1165 | 1196.0267 | 8398.50 | 6 |  |  |  |  |  |
| 476 | 9 | 7020.0466 | 1003.8711 | 9953.77 | 7 |  |  |  |  |  |
| 476 | 10 | 4495.8240 | 900.1721 | 14493.78 | 5 |  |  |  |  |  |
| 476 | 11 | 7072.0829 | 1179.6878 | 7088.54 | 6 |  |  |  |  |  |
| 476 | 12 | 7021.0536 | 878.6390 | 5901.72 | 8 |  |  |  |  |  |
| 476 | 13 | 7143.1249 | 1191.5281 | 6378.34 | 6 |  |  |  |  |  |
| 476 | 14 | 1796.0290 | 899.0218 | 16197.51 | 2 |  |  |  |  |  |
| 476 | 15 | 2788.2233 | 930.4150 | 4822.44 | 3 | 2788.2414 | C22 | 22 | -0.0181 | -6.49 |
| 476 | 16 | 3940.7540 | 986.1958 | 3599.45 | 4 | 3940.7834 | C31 | 31 | -0.0294 | -7.45 |
| 476 | 17 | 7113.0798 | 1017.1615 | 4679.57 | 7 |  |  |  |  |  |
| 476 | 18 | 1026.7346 | 1027.7419 | 10679.53 | 1 |  |  |  |  |  |
| 476 | 19 | 7112.0984 | 1186.3570 | 4318.51 | 6 |  |  |  |  |  |
| 476 | 20 | 3445.5797 | 862.4022 | 3712.23 | 4 | 3445.6046 | C27 | 27 | -0.0249 | -7.21 |
| 476 | 21 | 5229.3451 | 1046.8763 | 3512.96 | 5 |  |  |  |  |  |
| 476 | 22 | 6600.8505 | 1101.1490 | 3502.86 | 6 |  |  |  |  |  |
| 476 | 23 | 6204.6925 | 1035.1227 | 4633.64 | 6 |  |  |  |  |  |
| 476 | 24 | 4444.9045 | 1112.2334 | 3839.98 | 4 |  |  |  |  |  |
| 476 | 25 | 5903.4972 | 984.9235 | 3283.36 | 6 | 5902.4927 | C48 | 48 | 2.16e-03 | 0.37 |
| 476 | 26 | 7038.0961 | 1006.4496 | 3802.29 | 7 |  |  |  |  |  |
| 476 | 27 | 5961.5190 | 994.5938 | 3003.20 | 6 |  |  |  |  |  |
| 476 | 28 | 3157.4942 | 790.3808 | 4474.11 | 4 | 3157.5153 | C25 | 25 | -0.0212 | -6.70 |
| 476 | 29 | 1866.7973 | 934.4059 | 3796.17 | 2 | 1866.8101 | C15 | 15 | -0.0127 | -6.82 |
| 476 | 30 | 1752.7571 | 877.3858 | 5935.37 | 2 | 1752.7671 | C14 | 14 | -0.0100 | -5.71 |
| 476 | 31 | 2641.1493 | 881.3904 | 3313.13 | 3 | 2641.1730 | C21 | 21 | -0.0237 | -8.96 |
| 476 | 32 | 7130.1141 | 1427.0301 | 3511.32 | 5 |  |  |  |  |  |
| 476 | 33 | 3510.5305 | 1171.1841 | 2761.70 | 3 |  |  |  |  |  |
| 476 | 34 | 898.2673 | 899.2746 | 5131.28 | 1 |  |  |  |  |  |
| 476 | 35 | 3938.6460 | 1313.8893 | 2034.19 | 3 |  |  |  |  |  |
| 476 | 36 | 7168.1142 | 897.0216 | 3537.67 | 8 |  |  |  |  |  |
| 476 | 37 | 2026.8286 | 1014.4216 | 2269.92 | 2 | 2026.8407 | C16 | 16 | -0.0121 | -5.99 |
| 476 | 38 | 7097.1252 | 1183.8615 | 2034.32 | 6 |  |  |  |  |  |
| 476 | 39 | 2916.3107 | 973.1108 | 3026.31 | 3 | 2916.3363 | C23 | 23 | -0.0257 | -8.80 |
| 476 | 40 | 6043.6732 | 1008.2861 | 2613.50 | 6 |  |  |  |  |  |
| 476 | 41 | 3650.5541 | 1217.8587 | 1902.23 | 3 |  |  |  |  |  |
| 476 | 42 | 3157.4976 | 1053.5065 | 2578.21 | 3 | 3157.5153 | C25 | 25 | -0.0178 | -5.63 |
| 476 | 43 | 6225.6462 | 1246.1365 | 1616.85 | 5 | 6224.6317 | C51 | 51 | 0.0122 | 1.95 |
| 476 | 44 | 5814.5677 | 1163.9208 | 2122.46 | 5 |  |  |  |  |  |
| 476 | 45 | 3317.5236 | 830.3882 | 1995.09 | 4 | 3317.5460 | C26 | 26 | -0.0224 | -6.76 |
| 476 | 46 | 6259.6929 | 1044.2894 | 3171.85 | 6 |  |  |  |  |  |
| 476 | 47 | 997.4597 | 998.4670 | 2399.42 | 1 | 997.4651 | C8 | 8 | -5.35e-03 | -5.36 |
| 476 | 48 | 4056.7709 | 1015.2000 | 6447.60 | 4 |  |  |  |  |  |
| 476 | 49 | 6993.0766 | 1166.5200 | 1751.05 | 6 |  |  |  |  |  |
| 476 | 50 | 6792.9162 | 971.4239 | 1474.18 | 7 |  |  |  |  |  |
| 476 | 51 | 2470.0465 | 824.3561 | 2277.18 | 3 |  |  |  |  |  |
| 476 | 52 | 4170.8063 | 1043.7088 | 2993.59 | 4 | 4170.8372 | C33 | 33 | -0.0309 | -7.42 |
| 476 | 53 | 6318.7173 | 1264.7507 | 1500.81 | 5 |  |  |  |  |  |
| 476 | 54 | 6099.6694 | 1017.6189 | 4848.04 | 6 |  |  |  |  |  |
| 476 | 55 | 6224.6330 | 1038.4461 | 3201.69 | 6 | 6224.6317 | C51 | 51 | 1.38e-03 | 0.22 |
| 476 | 56 | 7052.1264 | 1008.4539 | 1515.91 | 7 |  |  |  |  |  |
| 476 | 57 | 4495.8220 | 1124.9628 | 1798.05 | 4 |  |  |  |  |  |
| 476 | 58 | 2149.9235 | 1075.9690 | 1177.81 | 2 |  |  |  |  |  |
| 476 | 59 | 2527.0696 | 843.3638 | 1239.72 | 3 |  |  |  |  |  |
| 476 | 60 | 739.3752 | 740.3824 | 1235.66 | 1 | 739.3799 | C6 | 6 | -4.70e-03 | -6.36 |
| 476 | 61 | 7080.1024 | 1012.4505 | 3280.49 | 7 | 7081.0774 | Z\_DOT59 | 1 | 0.0274 | 3.87 |
| 476 | 62 | 2742.2400 | 915.0873 | 1863.84 | 3 |  |  |  |  |  |
| 476 | 63 | 2885.2898 | 962.7705 | 1353.36 | 3 |  |  |  |  |  |
| 476 | 64 | 5343.3844 | 1069.6841 | 1912.74 | 5 |  |  |  |  |  |
| 476 | 65 | 6029.6532 | 1005.9495 | 1692.30 | 6 |  |  |  |  |  |
| 476 | 66 | 2321.3427 | 1161.6786 | 1107.25 | 2 |  |  |  |  |  |
| 476 | 67 | 1809.8700 | 905.9423 | 927.06 | 2 |  |  |  |  |  |
| 476 | 68 | 5503.3149 | 1101.6703 | 1455.89 | 5 | 5503.3559 | C45 | 45 | -0.0410 | -7.45 |
| 476 | 69 | 5435.3845 | 1088.0842 | 1676.79 | 5 |  |  |  |  |  |
| 476 | 70 | 6962.0278 | 995.5827 | 1639.33 | 7 |  |  |  |  |  |
| 476 | 71 | 4659.0469 | 1165.7690 | 1135.16 | 4 |  |  |  |  |  |
| 476 | 72 | 5813.5518 | 969.9326 | 1591.48 | 6 | 5812.5496 | Z\_DOT49 | 11 | -1.83e-04 | -0.03 |
| 476 | 73 | 4467.6804 | 894.5434 | 654.03 | 5 |  |  |  |  |  |
| 476 | 74 | 2684.2058 | 895.7425 | 2463.55 | 3 |  |  |  |  |  |
| 476 | 75 | 4544.9835 | 1137.2531 | 980.87 | 4 |  |  |  |  |  |
| 476 | 76 | 3691.6467 | 923.9189 | 833.80 | 4 |  |  |  |  |  |
| 476 | 77 | 5756.4669 | 960.4184 | 1356.12 | 6 | 5756.5098 | C47 | 47 | -0.0429 | -7.45 |
| 476 | 78 | 3567.0617 | 1190.0278 | 4934.36 | 3 |  |  |  |  |  |
| 476 | 79 | 6922.9874 | 990.0055 | 655.76 | 7 | 6922.9916 | C58 | 58 | -4.13e-03 | -0.60 |
| 476 | 80 | 6317.7117 | 1053.9592 | 1139.27 | 6 | 6316.7135 | Z\_DOT53 | 7 | -4.08e-03 | -0.65 |
| 476 | 81 | 2758.2584 | 920.4268 | 737.17 | 3 |  |  |  |  |  |
| 476 | 82 | 6992.0928 | 999.8777 | 2152.41 | 7 |  |  |  |  |  |
| 476 | 83 | 4900.1177 | 1226.0367 | 1045.54 | 4 |  |  |  |  |  |
| 476 | 84 | 5714.5588 | 1143.9190 | 652.43 | 5 |  |  |  |  |  |
| 476 | 85 | 3488.5289 | 1163.8502 | 1137.97 | 3 |  |  |  |  |  |
| 476 | 86 | 1181.8522 | 1182.8594 | 622.96 | 1 |  |  |  |  |  |
| 476 | 87 | 2873.3086 | 958.7768 | 1280.89 | 3 |  |  |  |  |  |
| 476 | 88 | 602.3175 | 603.3248 | 1694.79 | 1 | 602.3210 | C5 | 5 | -3.47e-03 | -5.76 |
| 476 | 89 | 7037.0725 | 1173.8527 | 1740.09 | 6 | 7036.0756 | C59 | 59 | -5.51e-03 | -0.78 |
| 476 | 90 | 1258.5340 | 1259.5413 | 613.36 | 1 | 1258.5434 | C10 | 10 | -9.34e-03 | -7.42 |
| 476 | 91 | 5960.5199 | 1193.1113 | 647.18 | 5 | 5959.5142 | C49 | 49 | 3.35e-03 | 0.56 |
| 476 | 92 | 1082.4632 | 1083.4705 | 650.24 | 1 |  |  |  |  |  |
| 476 | 93 | 2193.9389 | 1097.9767 | 664.63 | 2 |  |  |  |  |  |
| 476 | 94 | 824.3520 | 825.3593 | 479.36 | 1 |  |  |  |  |  |
| 476 | 95 | 1131.4847 | 1132.4920 | 574.16 | 1 |  |  |  |  |  |
| 476 | 96 | 1372.5782 | 687.2964 | 606.28 | 2 | 1372.5863 | C11 | 11 | -8.16e-03 | -5.94 |
| 476 | 97 | 971.1590 | 972.1663 | 510.86 | 1 |  |  |  |  |  |
| 476 | 98 | 1372.5776 | 1373.5849 | 836.17 | 1 | 1372.5863 | C11 | 11 | -8.69e-03 | -6.33 |
| 476 | 99 | 1316.9645 | 1317.9717 | 600.26 | 1 |  |  |  |  |  |
| 476 | 100 | 1101.8683 | 1102.8756 | 320.92 | 1 |  |  |  |  |  |
| 476 | 101 | 3453.4770 | 1152.1663 | 1147.47 | 3 |  |  |  |  |  |

  

All proteins /
CsTx-1a\_S1 Cupiennius salei toxin 1 isoform a S1^ACsTx-1a\_S2 Cupiennius salei toxin 1 isoform a S2 /
Proteoform #42
