# Supplementary material for: The Dual Prey-Inactivation Strategy of Spiders—In-Depth Venomic Analysis of Cupiennius salei
Source: Toxins (Basel). 2019 Mar 19;11(3):167. doi: 10.3390/toxins11030167 (PMC6468893; doi:10.3390/toxins11030167)
Supplement: Supplementary file 1 [file toxins-11-00167-s001.zip › Supplementary Dataset EV1/20180328_f2_topdown_OTMS2_EThcD_NL_i02_ms2_proteoform_cutoff_html/prsms/prsm121.html]

Protein-Spectrum-Match for Spectrum #356


All proteins /
CsTx-12b Cupiennius salei toxin 12 isoform b /
Proteoform #39

## Protein-Spectrum-Match #121 for Spectrum #356

|  |  |  |  |  |  |
| --- | --- | --- | --- | --- | --- |
| PrSM ID: | 121 | Scan(s): | 477 | Precursor charge: | 6 |
| Precursor m/z: | 560.3153 | Precursor mass: | 3355.8479 | Proteoform mass: | 3355.8472 |
| # matched peaks: | 30 | # matched fragment ions: | 26 | # unexpected modifications: | 0 |
| E-value: | 1.02e-22 | P-value: | 1.02e-22 | Q-value (Spectral FDR): | 0 |

  

|  |  |  |  |  |  |  |  |  |  |  |  |  |  |  |  |  |  |  |  |  |  |  |  |  |  |  |  |  |  |  |  |  |  |  |  |  |  |  |  |  |  |  |  |  |  |  |  |  |  |  |  |  |  |  |  |  |  |  |  |  |  |  |  |  |  |  |
| --- | --- | --- | --- | --- | --- | --- | --- | --- | --- | --- | --- | --- | --- | --- | --- | --- | --- | --- | --- | --- | --- | --- | --- | --- | --- | --- | --- | --- | --- | --- | --- | --- | --- | --- | --- | --- | --- | --- | --- | --- | --- | --- | --- | --- | --- | --- | --- | --- | --- | --- | --- | --- | --- | --- | --- | --- | --- | --- | --- | --- | --- | --- | --- | --- | --- | --- |
|  | | ... 30 amino acid residues are skipped at the N-terminus ... | | | | | | | | | | | | | | | | | | | | | | | | | | | | | | | | | | | | | | | | | | | | | | | | | | | | | | | | | | | | | |  | | |
|  | |  | | | | | | | | | | | | | | | | | | | | | | | | | | | | | | | | | | | | | | | | | | | | | | | | | | | | | | | | | | | | | | | | | | | |
| 31 |  |  | S |  | F |  | E |  | A |  | D |  | D |  | V |  | I |  | P |  | F |  |  | L |  | A |  | R |  | E |  | Q |  | V |  | R |  | S |  | D |  | C |  |  | T |  | L |  | R |  | N |  | H |  | D |  | C |  | T |  | D |  | D |  | 60 |  |
|  | |  | | | | | | | | | | | | | | | | | | | | | | | | | | | | | | | | | | | | | | | | | | | | | | | | | | | | | | | | | | | | | | | | | | | |
| 61 |  |  | R |  | H |  | S |  | C |  | C |  | R |  | S |  | K |  | M |  | F |  |  | K |  | D |  | V |  | C |  | K |  | C |  | F |  | Y |  | P |  | S |  |  | Q |  | R |  | S |  | D |  | T |  | A |  | R | ] | A | ⎩ | K |  | K |  | 90 |  |
|  | |  | | | | | | | | | | | | | | | | | | | | | | | | | | | | | | | | | | | | | | | | | | | | | | | | | | | | | | | | | | | | | | | | | | | |
| 91 |  |  | E | ⎫ | L |  | C |  | T |  | C | ⎫ | Q | ⎱ | Q | ⎱ | D | ⎱ | K |  | H |  |  | L | ⎫ | K | ⎱ | Y | ⎱ | I | ⎱ | E | ⎱ | K | ⎫ | G | ⎱ | L | ⎫ | Q | ⎫ | K |  |  | A |  | K | ⎫ | V | ⎫ | L | ⎫ | V | [ | A |  | G |  | | 117 |  | | | | | |

Fixed PTMs: Carbamidomethylation [C93 C95 ]

  

All peaks (55)  Matched peaks (30)  Not matched peaks (25)

  

| Scan | Peak | Mono mass | Mono m/z | Intensity | Charge | Theoretical mass | Ion | Pos | Mass error | PPM error |
| --- | --- | --- | --- | --- | --- | --- | --- | --- | --- | --- |
| 477 | 1 | 1681.9861 | 561.6693 | 63682.66 | 3 |  |  |  |  |  |
| 477 | 2 | 3298.8029 | 660.7679 | 27193.63 | 5 |  |  |  |  |  |
| 477 | 3 | 3354.8247 | 560.1447 | 124884.67 | 6 |  |  |  |  |  |
| 477 | 4 | 1118.5160 | 560.2653 | 30663.87 | 2 |  |  |  |  |  |
| 477 | 5 | 3142.6858 | 786.6787 | 12932.43 | 4 | 3142.7106 | C26 | 26 | -0.0248 | -7.89 |
| 477 | 6 | 3298.8050 | 825.7085 | 8589.22 | 4 |  |  |  |  |  |
| 477 | 7 | 2161.0993 | 721.3737 | 8279.43 | 3 | 2161.1135 | C17 | 17 | -0.0142 | -6.59 |
| 477 | 8 | 1471.8794 | 736.9470 | 7496.28 | 2 | 1471.8888 | Z\_DOT13 | 15 | -9.42e-03 | -6.40 |
| 477 | 9 | 3255.7719 | 652.1617 | 5558.73 | 5 | 3255.7947 | C27 | 27 | -0.0228 | -7.01 |
| 477 | 10 | 1884.9547 | 629.3255 | 8089.52 | 3 | 1884.9662 | C15 | 15 | -0.0115 | -6.10 |
| 477 | 11 | 2048.0178 | 683.6799 | 5471.94 | 3 | 2048.0295 | C16 | 16 | -0.0117 | -5.72 |
| 477 | 12 | 2290.1423 | 764.3881 | 6084.67 | 3 | 2290.1561 | C18 | 18 | -0.0138 | -6.03 |
| 477 | 13 | 3043.6216 | 761.9127 | 5196.83 | 4 | 3043.6422 | C25 | 25 | -0.0206 | -6.77 |
| 477 | 14 | 3339.8087 | 668.9690 | 4109.51 | 5 |  |  |  |  |  |
| 477 | 15 | 2237.8859 | 560.4788 | 48551.24 | 4 |  |  |  |  |  |
| 477 | 16 | 2475.2568 | 826.0929 | 4619.04 | 3 | 2475.2726 | C20 | 20 | -0.0157 | -6.35 |
| 477 | 17 | 3268.7671 | 818.1991 | 4059.97 | 4 | 3268.7914 | Z\_DOT27 | 1 | -0.0243 | -7.42 |
| 477 | 18 | 1487.8982 | 744.9564 | 4559.22 | 2 |  |  |  |  |  |
| 477 | 19 | 1978.2095 | 660.4105 | 5515.66 | 3 | 1978.2217 | Z\_DOT17 | 11 | -0.0122 | -6.17 |
| 477 | 20 | 2221.2949 | 741.4389 | 3576.15 | 3 | 2221.3073 | Z\_DOT19 | 9 | -0.0124 | -5.58 |
| 477 | 21 | 559.3176 | 560.3249 | 10521.50 | 1 |  |  |  |  |  |
| 477 | 22 | 1378.6249 | 690.3197 | 5654.82 | 2 | 1378.6333 | C11 | 11 | -8.35e-03 | -6.06 |
| 477 | 23 | 542.2910 | 543.2983 | 7376.59 | 1 |  |  |  |  |  |
| 477 | 24 | 1195.7338 | 598.8742 | 3658.97 | 2 | 1195.7415 | Z\_DOT11 | 17 | -7.66e-03 | -6.41 |
| 477 | 25 | 3142.6914 | 629.5456 | 3876.54 | 5 | 3142.7106 | C26 | 26 | -0.0192 | -6.11 |
| 477 | 26 | 1756.8615 | 879.4380 | 3458.29 | 2 | 1756.8712 | C14 | 14 | -9.71e-03 | -5.53 |
| 477 | 27 | 671.1653 | 672.1726 | 5916.67 | 1 |  |  |  |  |  |
| 477 | 28 | 1308.8172 | 655.4159 | 2582.39 | 2 | 1308.8255 | Z\_DOT12 | 16 | -8.35e-03 | -6.38 |
| 477 | 29 | 2093.2338 | 698.7519 | 2976.56 | 3 | 2093.2487 | Z\_DOT18 | 10 | -0.0149 | -7.10 |
| 477 | 30 | 2418.2376 | 807.0865 | 2821.89 | 3 | 2418.2511 | C19 | 19 | -0.0135 | -5.58 |
| 477 | 31 | 3220.8067 | 645.1686 | 2460.85 | 5 |  |  |  |  |  |
| 477 | 32 | 3339.8087 | 835.9595 | 3114.79 | 4 |  |  |  |  |  |
| 477 | 33 | 3141.6816 | 1048.2345 | 2762.63 | 3 |  |  |  |  |  |
| 477 | 34 | 2716.4012 | 680.1076 | 2259.31 | 4 | 2716.4152 | C22 | 22 | -0.0140 | -5.17 |
| 477 | 35 | 1263.5985 | 632.8065 | 3380.78 | 2 | 1263.6063 | C10 | 10 | -7.78e-03 | -6.16 |
| 477 | 36 | 2588.3401 | 648.0923 | 2124.76 | 4 | 2588.3566 | C21 | 21 | -0.0165 | -6.37 |
| 477 | 37 | 3268.7678 | 654.7608 | 2729.34 | 5 | 3268.7914 | Z\_DOT27 | 1 | -0.0236 | -7.22 |
| 477 | 38 | 2365.3682 | 592.3493 | 2586.25 | 4 |  |  |  |  |  |
| 477 | 39 | 2350.3557 | 784.4592 | 2764.95 | 3 |  |  |  |  |  |
| 477 | 40 | 2291.3228 | 573.8380 | 2642.30 | 4 |  |  |  |  |  |
| 477 | 41 | 838.5227 | 420.2686 | 2179.56 | 2 |  |  |  |  |  |
| 477 | 42 | 1135.5411 | 568.7778 | 2231.67 | 2 | 1135.5477 | C9 | 9 | -6.70e-03 | -5.90 |
| 477 | 43 | 1007.4832 | 1008.4905 | 2049.46 | 1 | 1007.4892 | C8 | 8 | -5.97e-03 | -5.92 |
| 477 | 44 | 1066.6922 | 534.3534 | 1862.00 | 2 | 1066.6989 | Z\_DOT10 | 18 | -6.70e-03 | -6.28 |
| 477 | 45 | 473.2936 | 474.3009 | 945.18 | 1 | 473.2961 | C4 | 4 | -2.52e-03 | -5.33 |
| 477 | 46 | 920.9719 | 921.9792 | 790.04 | 1 |  |  |  |  |  |
| 477 | 47 | 1342.7385 | 672.3765 | 18290.60 | 2 |  |  |  |  |  |
| 477 | 48 | 1422.7995 | 712.4070 | 834.82 | 2 |  |  |  |  |  |
| 477 | 49 | 881.5770 | 882.5842 | 1115.88 | 1 | 881.5824 | Z\_DOT8 | 20 | -5.50e-03 | -6.24 |
| 477 | 50 | 1239.6328 | 620.8237 | 334.20 | 2 |  |  |  |  |  |
| 477 | 51 | 1196.7413 | 1197.7486 | 769.37 | 1 |  |  |  |  |  |
| 477 | 52 | 1007.4826 | 504.7486 | 806.68 | 2 | 1007.4892 | C8 | 8 | -6.53e-03 | -6.48 |
| 477 | 53 | 1135.5399 | 1136.5472 | 804.36 | 1 | 1135.5477 | C9 | 9 | -7.84e-03 | -6.91 |
| 477 | 54 | 1455.8199 | 728.9172 | 1352.57 | 2 |  |  |  |  |  |
| 477 | 55 | 1279.7781 | 640.8963 | 930.95 | 2 |  |  |  |  |  |

  

All proteins /
CsTx-12b Cupiennius salei toxin 12 isoform b /
Proteoform #39
