# Supplementary material for: The Dual Prey-Inactivation Strategy of Spiders—In-Depth Venomic Analysis of Cupiennius salei
Source: Toxins (Basel). 2019 Mar 19;11(3):167. doi: 10.3390/toxins11030167 (PMC6468893; doi:10.3390/toxins11030167)
Supplement: Supplementary file 1 [file toxins-11-00167-s001.zip › Supplementary Dataset EV1/20180328_f2_topdown_OTMS2_EThcD_NL_i02_ms2_proteoform_cutoff_html/prsms/prsm122.html]

Protein-Spectrum-Match for Spectrum #357


All proteins /
CsTx-13a Cupiennius salei toxin 13 isoform a /
Proteoform #40

## Protein-Spectrum-Match #122 for Spectrum #357

|  |  |  |  |  |  |
| --- | --- | --- | --- | --- | --- |
| PrSM ID: | 122 | Scan(s): | 479 | Precursor charge: | 6 |
| Precursor m/z: | 580.3142 | Precursor mass: | 3475.8417 | Proteoform mass: | 3475.8317 |
| # matched peaks: | 33 | # matched fragment ions: | 28 | # unexpected modifications: | 1 |
| E-value: | 1.68e-21 | P-value: | 1.68e-21 | Q-value (Spectral FDR): | 0 |

  

|  |  |  |  |  |  |  |  |  |  |  |  |  |  |  |  |  |  |  |  |  |  |  |  |  |  |  |  |  |  |  |  |  |  |  |  |  |  |  |  |  |  |  |  |  |  |  |  |  |  |  |  |  |  |  |  |  |  |  |  |  |  |  |  |  |  |  |
| --- | --- | --- | --- | --- | --- | --- | --- | --- | --- | --- | --- | --- | --- | --- | --- | --- | --- | --- | --- | --- | --- | --- | --- | --- | --- | --- | --- | --- | --- | --- | --- | --- | --- | --- | --- | --- | --- | --- | --- | --- | --- | --- | --- | --- | --- | --- | --- | --- | --- | --- | --- | --- | --- | --- | --- | --- | --- | --- | --- | --- | --- | --- | --- | --- | --- | --- |
|  | | ... 30 amino acid residues are skipped at the N-terminus ... | | | | | | | | | | | | | | | | | | | | | | | | | | | | | | | | | | | | | | | | | | | | | | | | | | | | | | | | | | | | | |  | | |
|  | |  | | | | | | | | | | | | | | | | | | | | | | | | | | | | | | | | | | | | | | | | | | | | | | | | | | | | | | | | | | | | | | | | | | | |
| 31 |  |  | S |  | F |  | E |  | A |  | D |  | D |  | I |  | I |  | P |  | F |  |  | I |  | A |  | K |  | E |  | Q |  | V |  | R |  | S |  | D |  | C |  |  | T |  | L |  | R |  | N |  | H |  | D |  | C |  | T |  | D |  | D |  | 60 |  |
|  | |  | | | | | | | | | | | | | | | | | | | | | | | | | | | | | | | | | | | | | | | | | | | | | | | | | | | | | | | | | | | | | | | | | | | |
| 61 |  |  | R |  | H |  | S |  | C |  | C |  | R |  | S |  | K |  | M |  | F |  |  | K |  | D |  | V |  | C |  | T |  | C |  | F |  | Y |  | P |  | S |  |  | Q |  | R |  | S |  | E |  | T |  | A |  | R | ] | A | ⎩ | K | ⎩ | K |  | 90 |  |
|  | |  | | | | | | | | | | | | | | | | | | | | | | | | | | | | | | | | | | | | | | | | | | | | | | | | | | | -58.02 | | | | | | | | | | | | | |
| 91 |  | ⎫ | E | ⎱ | L |  | C | ⎫ | T | ⎫ | C | ⎫ | Q | ⎱ | Q |  | P | ⎱ | K | ⎫ | H |  |  | L |  | K | ⎱ | Y | ⎫ | I | ⎱ | E | ⎱ | K | ⎫ | G |  | L |  | Q | ⎱ | K |  | ⎱ | A |  | K | ⎫ | D | ⎫ | Y | ⎫ | A |  | T |  | G |  | | 117 |  | | | | | |

Fixed PTMs: Carbamidomethylation [C93 C95 ]   
  
     Unexpected modifications:   Unknown [-58.02]

  

All peaks (57)  Matched peaks (33)  Not matched peaks (24)

  

| Scan | Peak | Mono mass | Mono m/z | Intensity | Charge | Theoretical mass | Ion | Pos | Mass error | PPM error |
| --- | --- | --- | --- | --- | --- | --- | --- | --- | --- | --- |
| 479 | 1 | 3418.7994 | 684.7672 | 108250.97 | 5 |  |  |  |  |  |
| 479 | 2 | 3474.8273 | 580.1452 | 211378.89 | 6 |  |  |  |  |  |
| 479 | 3 | 3025.6499 | 757.4198 | 51229.47 | 4 | 3025.6680 | C25 | 25 | -0.0181 | -5.98 |
| 479 | 4 | 3140.6753 | 786.1761 | 47402.97 | 4 | 3140.6950 | C26 | 26 | -0.0197 | -6.26 |
| 479 | 5 | 3418.8021 | 855.7078 | 40764.26 | 4 |  |  |  |  |  |
| 479 | 6 | 2272.1683 | 758.3967 | 42258.47 | 3 | 2272.1820 | C18 | 18 | -0.0136 | -5.99 |
| 479 | 7 | 3459.8035 | 692.9680 | 27696.03 | 5 |  |  |  |  |  |
| 479 | 8 | 1866.9812 | 623.3343 | 45213.61 | 3 | 1866.9920 | C15 | 15 | -0.0108 | -5.76 |
| 479 | 9 | 2698.4254 | 900.4824 | 30219.26 | 3 | 2698.4410 | C22 | 22 | -0.0156 | -5.78 |
| 479 | 10 | 2143.1268 | 715.3829 | 32181.67 | 3 | 2143.1394 | C17 | 17 | -0.0126 | -5.88 |
| 479 | 11 | 2826.5186 | 707.6369 | 27496.69 | 4 | 2826.5360 | C23 | 23 | -0.0174 | -6.15 |
| 479 | 12 | 1158.9438 | 580.4792 | 177900.50 | 2 |  |  |  |  |  |
| 479 | 13 | 3303.7391 | 826.9420 | 22452.40 | 4 | 3303.7583 | C27 | 27 | -0.0193 | -5.83 |
| 479 | 14 | 579.6383 | 580.6456 | 165463.74 | 1 |  |  |  |  |  |
| 479 | 15 | 1609.8499 | 805.9322 | 37916.42 | 2 | 1609.8475 | Z\_DOT15 | 15 | 2.37e-03 | 1.47 |
| 479 | 16 | 2116.1797 | 706.4005 | 32931.59 | 3 | 2116.1804 | Z\_DOT19 | 11 | -7.07e-04 | -0.33 |
| 479 | 17 | 3260.6734 | 816.1756 | 23050.09 | 4 | 3260.6809 | Z\_DOT28 | 2 | -7.54e-03 | -2.31 |
| 479 | 18 | 2800.4522 | 701.1203 | 26594.31 | 4 |  |  |  |  |  |
| 479 | 19 | 2341.2897 | 781.4372 | 24039.08 | 3 | 2341.2917 | Z\_DOT21 | 9 | -2.00e-03 | -0.86 |
| 479 | 20 | 3303.7379 | 661.7549 | 18002.20 | 5 | 3303.7583 | C27 | 27 | -0.0204 | -6.18 |
| 479 | 21 | 2539.3763 | 635.8513 | 25909.14 | 4 |  |  |  |  |  |
| 479 | 22 | 3432.8165 | 859.2114 | 19181.82 | 4 |  |  |  |  |  |
| 479 | 23 | 3458.8044 | 577.4747 | 16575.47 | 6 |  |  |  |  |  |
| 479 | 24 | 1625.8681 | 813.9413 | 28774.11 | 2 |  |  |  |  |  |
| 479 | 25 | 1360.6516 | 681.3331 | 28356.05 | 2 | 1360.6591 | C11 | 11 | -7.44e-03 | -5.47 |
| 479 | 26 | 3025.6495 | 1009.5571 | 17563.45 | 3 | 3025.6680 | C25 | 25 | -0.0185 | -6.12 |
| 479 | 27 | 3388.7644 | 678.7601 | 20230.22 | 5 | 3388.7759 | Z\_DOT29 | 1 | -0.0115 | -3.40 |
| 479 | 28 | 3004.5444 | 1002.5221 | 15677.26 | 3 |  |  |  |  |  |
| 479 | 29 | 3460.8129 | 866.2105 | 16476.78 | 4 |  |  |  |  |  |
| 479 | 30 | 1204.6621 | 603.3383 | 18542.97 | 2 | 1204.6575 | Z\_DOT12 | 18 | 4.61e-03 | 3.82 |
| 479 | 31 | 3474.8216 | 695.9716 | 245354.82 | 5 |  |  |  |  |  |
| 479 | 32 | 1488.7456 | 745.3801 | 12875.74 | 2 | 1488.7540 | C12 | 12 | -8.41e-03 | -5.65 |
| 479 | 33 | 1390.7315 | 696.3730 | 75033.84 | 2 |  |  |  |  |  |
| 479 | 34 | 2030.0434 | 677.6884 | 13696.09 | 3 | 2030.0553 | C16 | 16 | -0.0119 | -5.86 |
| 479 | 35 | 3003.5364 | 751.8914 | 15674.05 | 4 | 3003.5434 | Z\_DOT26 | 4 | -6.95e-03 | -2.31 |
| 479 | 36 | 2400.2631 | 801.0950 | 10988.98 | 3 | 2400.2769 | C19 | 19 | -0.0139 | -5.78 |
| 479 | 37 | 1135.5417 | 568.7781 | 15728.28 | 2 | 1135.5477 | C9 | 9 | -6.09e-03 | -5.36 |
| 479 | 38 | 2960.4822 | 741.1278 | 14289.43 | 4 |  |  |  |  |  |
| 479 | 39 | 3431.8106 | 687.3694 | 11483.42 | 5 |  |  |  |  |  |
| 479 | 40 | 1333.7040 | 667.8593 | 15910.54 | 2 | 1333.7001 | Z\_DOT13 | 17 | 3.89e-03 | 2.92 |
| 479 | 41 | 562.0643 | 563.0715 | 29555.32 | 1 |  |  |  |  |  |
| 479 | 42 | 847.4549 | 848.4622 | 7016.61 | 1 | 847.4585 | C7 | 7 | -3.57e-03 | -4.21 |
| 479 | 43 | 1220.6812 | 611.3479 | 6839.38 | 2 |  |  |  |  |  |
| 479 | 44 | 1135.5419 | 1136.5491 | 7147.70 | 1 | 1135.5477 | C9 | 9 | -5.89e-03 | -5.19 |
| 479 | 45 | 1488.7457 | 497.2558 | 6221.79 | 3 | 1488.7540 | C12 | 12 | -8.39e-03 | -5.64 |
| 479 | 46 | 1274.6908 | 638.3527 | 4718.78 | 2 |  |  |  |  |  |
| 479 | 47 | 650.3116 | 651.3189 | 9364.27 | 1 | 650.3035 | Z\_DOT7 | 23 | 8.12e-03 | 12.48 |
| 479 | 48 | 473.2939 | 474.3012 | 12422.18 | 1 | 473.2961 | C4 | 4 | -2.19e-03 | -4.62 |
| 479 | 49 | 1007.4838 | 1008.4910 | 4295.31 | 1 | 1007.4892 | C8 | 8 | -5.42e-03 | -5.38 |
| 479 | 50 | 778.4060 | 779.4133 | 5546.33 | 1 | 778.3985 | Z\_DOT8 | 22 | 7.52e-03 | 9.66 |
| 479 | 51 | 869.9610 | 870.9683 | 19590.99 | 1 |  |  |  |  |  |
| 479 | 52 | 976.4924 | 489.2535 | 4791.63 | 2 |  |  |  |  |  |
| 479 | 53 | 1417.7484 | 709.8815 | 1929.27 | 2 |  |  |  |  |  |
| 479 | 54 | 1007.4840 | 504.7493 | 3325.21 | 2 | 1007.4892 | C8 | 8 | -5.19e-03 | -5.15 |
| 479 | 55 | 1159.6141 | 1160.6214 | 2600.70 | 1 |  |  |  |  |  |
| 479 | 56 | 746.4070 | 747.4143 | 3161.29 | 1 | 746.4108 | C6 | 6 | -3.80e-03 | -5.10 |
| 479 | 57 | 344.2522 | 345.2595 | 2567.71 | 1 | 344.2535 | C3 | 3 | -1.35e-03 | -3.91 |

  

All proteins /
CsTx-13a Cupiennius salei toxin 13 isoform a /
Proteoform #40
