# Supplementary material for: The Dual Prey-Inactivation Strategy of Spiders—In-Depth Venomic Analysis of Cupiennius salei
Source: Toxins (Basel). 2019 Mar 19;11(3):167. doi: 10.3390/toxins11030167 (PMC6468893; doi:10.3390/toxins11030167)
Supplement: Supplementary file 1 [file toxins-11-00167-s001.zip › Supplementary Dataset EV1/20180328_f2_topdown_OTMS2_EThcD_NL_i02_ms2_proteoform_cutoff_html/prsms/prsm123.html]

Protein-Spectrum-Match for Spectrum #358


All proteins /
CsTx-12a\_S1 Cupiennius salei toxin 12 isoform a S1^ACsTx-12a\_S2 Cupiennius salei toxin 12 isoform a S2 /
Proteoform #18

## Protein-Spectrum-Match #123 for Spectrum #358

|  |  |  |  |  |  |
| --- | --- | --- | --- | --- | --- |
| PrSM ID: | 123 | Scan(s): | 480 | Precursor charge: | 6 |
| Precursor m/z: | 729.3077 | Precursor mass: | 4369.8024 | Proteoform mass: | 4369.8061 |
| # matched peaks: | 27 | # matched fragment ions: | 26 | # unexpected modifications: | 0 |
| E-value: | 1.36e-26 | P-value: | 1.36e-26 | Q-value (Spectral FDR): | 0 |

  

|  |  |  |  |  |  |  |  |  |  |  |  |  |  |  |  |  |  |  |  |  |  |  |  |  |  |  |  |  |  |  |  |  |  |  |  |  |  |  |  |  |  |  |  |  |  |  |  |  |  |  |  |  |  |  |  |  |  |  |  |  |  |  |  |  |  |  |  |  |  |
| --- | --- | --- | --- | --- | --- | --- | --- | --- | --- | --- | --- | --- | --- | --- | --- | --- | --- | --- | --- | --- | --- | --- | --- | --- | --- | --- | --- | --- | --- | --- | --- | --- | --- | --- | --- | --- | --- | --- | --- | --- | --- | --- | --- | --- | --- | --- | --- | --- | --- | --- | --- | --- | --- | --- | --- | --- | --- | --- | --- | --- | --- | --- | --- | --- | --- | --- | --- | --- | --- |
|  | |  | | | | | | | | | | | | | | | | | | | | | | | | | | | | | | | | | | | | | | | | | | | | | | | | | | | | | | | | | | | | | | | | | | | |
| 1 |  |  | M |  | K |  | V |  | L |  | V |  | I |  | C |  | A |  | V |  | L |  |  | F |  | L |  | T |  | I |  | F |  | S |  | N |  | S |  | S |  | A |  |  | E |  | T |  | E |  | D |  | D |  | F |  | L |  | E |  | D |  | E |  | 30 |  |
|  | |  | | | | | | | | | | | | | | | | | | | | | | | | | | | | | | | | | | | | | | | | | | | | | | | | | | | | | | | | | | | | | | | | | | | |
| 31 |  |  | S |  | F |  | E |  | A |  | D |  | D |  | V |  | I |  | P |  | F |  |  | L |  | A |  | R |  | E |  | Q |  | V |  | R | ] | S |  | D |  | C |  |  | T |  | L | ⎫ | R | ⎱ | N |  | H | ⎫ | D | ⎫ | C | ⎫ | T | ⎫ | D | ⎱ | D |  | 60 |  |
|  | |  | | | | | | | | | | | | | | | | | | | | | | | | | | | | | | | | | | | | | | | | | | | | | | | | | | | | | | | | | | | | | | | | | | | |
| 61 |  | ⎱ | R |  | H |  | S | ⎫ | C |  | C | ⎫ | R | ⎱ | S | ⎱ | K | ⎫ | M |  | F |  |  | K | ⎫ | D | ⎱ | V |  | C |  | K | ⎫ | C | ⎫ | F | ⎫ | Y |  | P | ⎫ | S |  | ⎫ | Q | [ | R |  | S |  | D |  | T |  | A |  | R |  | A |  | K |  | K |  | 90 |  |
|  | |  | | | | | | | | | | | | | | | | | | | | | | | | | | | | | | | | | | | | | | | | | | | | | | | | | | | | | | | | | | | | | | | | | | | |
| 91 |  |  | E |  | L |  | C |  | T |  | C |  | Q |  | Q |  | D |  | K |  | H |  |  | L |  | K |  | F |  | I |  | E |  | K |  | G |  | L |  | Q |  | K |  |  | A |  | K |  | V |  | L |  | V |  | A |  | G |  | | 117 |  | | | | | |

Fixed PTMs: Carbamidomethylation [C50 C57 C64 C65 C74 C76 ]

  

All peaks (72)  Matched peaks (27)  Not matched peaks (45)

  

| Scan | Peak | Mono mass | Mono m/z | Intensity | Charge | Theoretical mass | Ion | Pos | Mass error | PPM error |
| --- | --- | --- | --- | --- | --- | --- | --- | --- | --- | --- |
| 480 | 1 | 4312.7569 | 863.5586 | 184313.33 | 5 |  |  |  |  |  |
| 480 | 2 | 1456.9294 | 729.4720 | 210211.61 | 2 |  |  |  |  |  |
| 480 | 3 | 4368.7840 | 729.1379 | 191922.41 | 6 |  |  |  |  |  |
| 480 | 4 | 4312.7574 | 1079.1966 | 38152.94 | 4 |  |  |  |  |  |
| 480 | 5 | 2293.9958 | 765.6725 | 30686.64 | 3 |  |  |  |  |  |
| 480 | 6 | 3586.4933 | 897.6306 | 25828.25 | 4 | 3586.5162 | C28 | 28 | -0.0230 | -6.40 |
| 480 | 7 | 4353.7624 | 871.7598 | 25221.17 | 5 |  |  |  |  |  |
| 480 | 8 | 3634.9016 | 727.9876 | 29206.97 | 5 |  |  |  |  |  |
| 480 | 9 | 4240.7342 | 849.1541 | 20715.32 | 5 | 4240.7634 | C33 | 33 | -0.0293 | -6.91 |
| 480 | 10 | 2462.9492 | 821.9903 | 22775.89 | 3 | 2462.9644 | C19 | 19 | -0.0152 | -6.18 |
| 480 | 11 | 2914.1854 | 729.5536 | 239914.13 | 4 |  |  |  |  |  |
| 480 | 12 | 4061.7017 | 1016.4327 | 18757.57 | 4 |  |  |  |  |  |
| 480 | 13 | 3893.5907 | 974.4049 | 18293.26 | 4 | 3893.6153 | C30 | 30 | -0.0247 | -6.33 |
| 480 | 14 | 1986.7900 | 994.4023 | 24287.87 | 2 | 1986.8020 | C16 | 16 | -0.0120 | -6.05 |
| 480 | 15 | 3084.2747 | 772.0760 | 18702.71 | 4 | 3084.2953 | C24 | 24 | -0.0206 | -6.67 |
| 480 | 16 | 3746.5223 | 937.6379 | 14103.11 | 4 | 3746.5469 | C29 | 29 | -0.0246 | -6.57 |
| 480 | 17 | 1491.5738 | 746.7942 | 17054.07 | 2 | 1491.5830 | C12 | 12 | -9.26e-03 | -6.21 |
| 480 | 18 | 4221.7498 | 845.3572 | 12224.08 | 5 |  |  |  |  |  |
| 480 | 19 | 3459.4003 | 865.8573 | 15279.94 | 4 |  |  |  |  |  |
| 480 | 20 | 2549.9802 | 851.0007 | 13706.50 | 3 | 2549.9965 | C20 | 20 | -0.0163 | -6.39 |
| 480 | 21 | 4255.7349 | 1064.9410 | 14268.84 | 4 |  |  |  |  |  |
| 480 | 22 | 4354.7696 | 1089.6997 | 10656.20 | 4 |  |  |  |  |  |
| 480 | 23 | 1907.8382 | 954.9264 | 12273.26 | 2 | 1907.8495 | Z\_DOT15 | 19 | -0.0113 | -5.91 |
| 480 | 24 | 2879.2135 | 960.7451 | 9194.83 | 3 | 2879.2309 | Z\_DOT22 | 12 | -0.0174 | -6.04 |
| 480 | 25 | 3199.3023 | 800.8328 | 8931.95 | 4 | 3199.3222 | C25 | 25 | -0.0199 | -6.23 |
| 480 | 26 | 874.1568 | 875.1641 | 24947.55 | 1 |  |  |  |  |  |
| 480 | 27 | 2186.3962 | 1094.2054 | 15514.76 | 2 |  |  |  |  |  |
| 480 | 28 | 4327.7572 | 866.5587 | 9990.56 | 5 |  |  |  |  |  |
| 480 | 29 | 4267.7408 | 1067.9425 | 7535.00 | 4 |  |  |  |  |  |
| 480 | 30 | 4265.7595 | 854.1592 | 7227.35 | 5 |  |  |  |  |  |
| 480 | 31 | 1606.6003 | 804.3074 | 8941.85 | 2 | 1606.6100 | C13 | 13 | -9.71e-03 | -6.05 |
| 480 | 32 | 3621.4381 | 906.3668 | 5816.41 | 4 | 3621.4649 | Z\_DOT28 | 6 | -0.0269 | -7.42 |
| 480 | 33 | 3635.8978 | 909.9817 | 5115.78 | 4 |  |  |  |  |  |
| 480 | 34 | 4222.7544 | 1056.6959 | 6974.58 | 4 |  |  |  |  |  |
| 480 | 35 | 2306.8503 | 1154.4324 | 6028.84 | 2 | 2306.8633 | C18 | 18 | -0.0130 | -5.65 |
| 480 | 36 | 728.6267 | 729.6340 | 84921.18 | 1 |  |  |  |  |  |
| 480 | 37 | 2895.2330 | 724.8155 | 7167.27 | 4 |  |  |  |  |  |
| 480 | 38 | 2764.1854 | 922.4024 | 6936.45 | 3 | 2764.2039 | Z\_DOT21 | 13 | -0.0186 | -6.72 |
| 480 | 39 | 2133.9662 | 712.3293 | 5993.91 | 3 |  |  |  |  |  |
| 480 | 40 | 4279.7834 | 856.9640 | 8530.52 | 5 |  |  |  |  |  |
| 480 | 41 | 4352.7728 | 726.4694 | 5664.87 | 6 |  |  |  |  |  |
| 480 | 42 | 3165.3121 | 1056.1113 | 4720.14 | 3 |  |  |  |  |  |
| 480 | 43 | 1820.8056 | 911.4101 | 6369.92 | 2 | 1820.8174 | Z\_DOT14 | 20 | -0.0118 | -6.50 |
| 480 | 44 | 2678.0738 | 893.6985 | 5341.10 | 3 | 2678.0914 | C21 | 21 | -0.0176 | -6.58 |
| 480 | 45 | 4061.7019 | 813.3477 | 6573.42 | 5 |  |  |  |  |  |
| 480 | 46 | 4153.6988 | 1039.4320 | 4433.56 | 4 | 4153.7314 | C32 | 32 | -0.0326 | -7.86 |
| 480 | 47 | 4326.7701 | 1082.6998 | 5245.18 | 4 |  |  |  |  |  |
| 480 | 48 | 4005.6783 | 1002.4268 | 3961.83 | 4 |  |  |  |  |  |
| 480 | 49 | 3906.6483 | 977.6693 | 4232.87 | 4 |  |  |  |  |  |
| 480 | 50 | 4239.7369 | 1060.9415 | 5053.65 | 4 |  |  |  |  |  |
| 480 | 51 | 1376.5479 | 689.2812 | 5478.64 | 2 | 1376.5561 | C11 | 11 | -8.20e-03 | -5.96 |
| 480 | 52 | 3084.2759 | 1029.0992 | 5222.67 | 3 | 3084.2953 | C24 | 24 | -0.0194 | -6.29 |
| 480 | 53 | 1474.5463 | 738.2804 | 3454.38 | 2 |  |  |  |  |  |
| 480 | 54 | 1275.5006 | 638.7576 | 2879.21 | 2 | 1275.5084 | C10 | 10 | -7.76e-03 | -6.09 |
| 480 | 55 | 694.2922 | 695.2994 | 2871.92 | 1 |  |  |  |  |  |
| 480 | 56 | 749.3448 | 750.3520 | 6335.40 | 1 | 749.3490 | C6 | 6 | -4.20e-03 | -5.61 |
| 480 | 57 | 1115.4712 | 558.7429 | 2873.19 | 2 | 1115.4778 | C9 | 9 | -6.53e-03 | -5.85 |
| 480 | 58 | 1123.7827 | 1124.7900 | 1911.49 | 1 |  |  |  |  |  |
| 480 | 59 | 1000.4451 | 501.2298 | 2887.96 | 2 | 1000.4508 | C8 | 8 | -5.71e-03 | -5.71 |
| 480 | 60 | 710.1124 | 711.1196 | 1757.65 | 1 |  |  |  |  |  |
| 480 | 61 | 330.1526 | 331.1599 | 2851.73 | 1 |  |  |  |  |  |
| 480 | 62 | 1171.4855 | 1172.4927 | 1441.85 | 1 | 1171.4917 | Z\_DOT9 | 25 | -6.23e-03 | -5.32 |
| 480 | 63 | 1457.9343 | 1458.9416 | 1335.18 | 1 |  |  |  |  |  |
| 480 | 64 | 830.7406 | 831.7479 | 1276.59 | 1 |  |  |  |  |  |
| 480 | 65 | 593.2446 | 594.2519 | 1489.66 | 1 | 593.2479 | C5 | 5 | -3.25e-03 | -5.48 |
| 480 | 66 | 517.0906 | 518.0978 | 1917.96 | 1 |  |  |  |  |  |
| 480 | 67 | 625.2710 | 626.2783 | 708.53 | 1 |  |  |  |  |  |
| 480 | 68 | 493.2148 | 494.2221 | 1196.23 | 1 |  |  |  |  |  |
| 480 | 69 | 1287.5163 | 1288.5236 | 1407.53 | 1 |  |  |  |  |  |
| 480 | 70 | 576.2182 | 577.2255 | 686.57 | 1 |  |  |  |  |  |
| 480 | 71 | 1242.5207 | 1243.5280 | 526.34 | 1 |  |  |  |  |  |
| 480 | 72 | 1439.9242 | 1440.9315 | 489.67 | 1 |  |  |  |  |  |

  

All proteins /
CsTx-12a\_S1 Cupiennius salei toxin 12 isoform a S1^ACsTx-12a\_S2 Cupiennius salei toxin 12 isoform a S2 /
Proteoform #18
