# Supplementary material for: The Dual Prey-Inactivation Strategy of Spiders—In-Depth Venomic Analysis of Cupiennius salei
Source: Toxins (Basel). 2019 Mar 19;11(3):167. doi: 10.3390/toxins11030167 (PMC6468893; doi:10.3390/toxins11030167)
Supplement: Supplementary file 1 [file toxins-11-00167-s001.zip › Supplementary Dataset EV1/20180328_f2_topdown_OTMS2_EThcD_NL_i02_ms2_proteoform_cutoff_html/prsms/prsm124.html]

Protein-Spectrum-Match for Spectrum #359


All proteins /
CsTx-12b Cupiennius salei toxin 12 isoform b /
Proteoform #39

## Protein-Spectrum-Match #124 for Spectrum #359

|  |  |  |  |  |  |
| --- | --- | --- | --- | --- | --- |
| PrSM ID: | 124 | Scan(s): | 481 | Precursor charge: | 6 |
| Precursor m/z: | 560.3144 | Precursor mass: | 3355.8428 | Proteoform mass: | 3355.8472 |
| # matched peaks: | 22 | # matched fragment ions: | 20 | # unexpected modifications: | 0 |
| E-value: | 8.09e-22 | P-value: | 8.09e-22 | Q-value (Spectral FDR): | 0 |

  

|  |  |  |  |  |  |  |  |  |  |  |  |  |  |  |  |  |  |  |  |  |  |  |  |  |  |  |  |  |  |  |  |  |  |  |  |  |  |  |  |  |  |  |  |  |  |  |  |  |  |  |  |  |  |  |  |  |  |  |  |  |  |  |  |  |  |  |
| --- | --- | --- | --- | --- | --- | --- | --- | --- | --- | --- | --- | --- | --- | --- | --- | --- | --- | --- | --- | --- | --- | --- | --- | --- | --- | --- | --- | --- | --- | --- | --- | --- | --- | --- | --- | --- | --- | --- | --- | --- | --- | --- | --- | --- | --- | --- | --- | --- | --- | --- | --- | --- | --- | --- | --- | --- | --- | --- | --- | --- | --- | --- | --- | --- | --- | --- |
|  | | ... 30 amino acid residues are skipped at the N-terminus ... | | | | | | | | | | | | | | | | | | | | | | | | | | | | | | | | | | | | | | | | | | | | | | | | | | | | | | | | | | | | | |  | | |
|  | |  | | | | | | | | | | | | | | | | | | | | | | | | | | | | | | | | | | | | | | | | | | | | | | | | | | | | | | | | | | | | | | | | | | | |
| 31 |  |  | S |  | F |  | E |  | A |  | D |  | D |  | V |  | I |  | P |  | F |  |  | L |  | A |  | R |  | E |  | Q |  | V |  | R |  | S |  | D |  | C |  |  | T |  | L |  | R |  | N |  | H |  | D |  | C |  | T |  | D |  | D |  | 60 |  |
|  | |  | | | | | | | | | | | | | | | | | | | | | | | | | | | | | | | | | | | | | | | | | | | | | | | | | | | | | | | | | | | | | | | | | | | |
| 61 |  |  | R |  | H |  | S |  | C |  | C |  | R |  | S |  | K |  | M |  | F |  |  | K |  | D |  | V |  | C |  | K |  | C |  | F |  | Y |  | P |  | S |  |  | Q |  | R |  | S |  | D |  | T |  | A |  | R | ] | A | ⎩ | K |  | K |  | 90 |  |
|  | |  | | | | | | | | | | | | | | | | | | | | | | | | | | | | | | | | | | | | | | | | | | | | | | | | | | | | | | | | | | | | | | | | | | | |
| 91 |  |  | E |  | L |  | C |  | T |  | C | ⎫ | Q | ⎫ | Q | ⎫ | D | ⎱ | K |  | H |  | ⎫ | L | ⎱ | K | ⎩ | Y | ⎱ | I | ⎱ | E | ⎱ | K |  | G | ⎫ | L |  | Q |  | K |  |  | A |  | K | ⎫ | V | ⎫ | L | ⎫ | V | [ | A |  | G |  | | 117 |  | | | | | |

Fixed PTMs: Carbamidomethylation [C93 C95 ]

  

All peaks (52)  Matched peaks (22)  Not matched peaks (30)

  

| Scan | Peak | Mono mass | Mono m/z | Intensity | Charge | Theoretical mass | Ion | Pos | Mass error | PPM error |
| --- | --- | --- | --- | --- | --- | --- | --- | --- | --- | --- |
| 481 | 1 | 558.2884 | 559.2957 | 21312.02 | 1 |  |  |  |  |  |
| 481 | 2 | 1678.4139 | 560.4786 | 24310.78 | 3 |  |  |  |  |  |
| 481 | 3 | 1118.5164 | 560.2655 | 48067.65 | 2 |  |  |  |  |  |
| 481 | 4 | 3297.8005 | 660.5674 | 10415.06 | 5 |  |  |  |  |  |
| 481 | 5 | 3354.8247 | 560.1447 | 20699.23 | 6 |  |  |  |  |  |
| 481 | 6 | 3142.6868 | 786.6790 | 5593.75 | 4 | 3142.7106 | C26 | 26 | -0.0238 | -7.58 |
| 481 | 7 | 3298.8006 | 825.7074 | 4023.87 | 4 |  |  |  |  |  |
| 481 | 8 | 2797.6429 | 560.5359 | 5840.60 | 5 |  |  |  |  |  |
| 481 | 9 | 2290.1400 | 764.3873 | 3201.14 | 3 | 2290.1561 | C18 | 18 | -0.0162 | -7.07 |
| 481 | 10 | 2048.0154 | 683.6791 | 2800.64 | 3 | 2048.0295 | C16 | 16 | -0.0141 | -6.88 |
| 481 | 11 | 2237.5503 | 560.3949 | 28861.28 | 4 |  |  |  |  |  |
| 481 | 12 | 1471.8789 | 736.9467 | 3272.25 | 2 | 1471.8888 | Z\_DOT13 | 15 | -9.91e-03 | -6.73 |
| 481 | 13 | 3255.7710 | 652.1615 | 3613.85 | 5 | 3255.7947 | C27 | 27 | -0.0237 | -7.29 |
| 481 | 14 | 3043.6224 | 761.9129 | 2834.47 | 4 | 3043.6422 | C25 | 25 | -0.0199 | -6.53 |
| 481 | 15 | 1883.9527 | 628.9915 | 3895.22 | 3 |  |  |  |  |  |
| 481 | 16 | 2475.2559 | 826.0926 | 2261.30 | 3 | 2475.2726 | C20 | 20 | -0.0166 | -6.72 |
| 481 | 17 | 3354.8204 | 671.9714 | 17915.23 | 5 |  |  |  |  |  |
| 481 | 18 | 3268.7710 | 818.2000 | 2857.41 | 4 | 3268.7914 | Z\_DOT27 | 1 | -0.0204 | -6.23 |
| 481 | 19 | 542.2910 | 543.2983 | 3472.61 | 1 |  |  |  |  |  |
| 481 | 20 | 2161.0997 | 721.3738 | 3329.54 | 3 | 2161.1135 | C17 | 17 | -0.0139 | -6.42 |
| 481 | 21 | 1487.8973 | 744.9559 | 2157.66 | 2 |  |  |  |  |  |
| 481 | 22 | 1978.2094 | 660.4104 | 2032.56 | 3 | 1978.2217 | Z\_DOT17 | 11 | -0.0124 | -6.26 |
| 481 | 23 | 1378.6242 | 690.3194 | 2221.09 | 2 | 1378.6333 | C11 | 11 | -9.09e-03 | -6.59 |
| 481 | 24 | 3142.6890 | 629.5451 | 1589.05 | 5 | 3142.7106 | C26 | 26 | -0.0216 | -6.89 |
| 481 | 25 | 1599.9735 | 800.9940 | 1407.43 | 2 | 1599.9838 | Z\_DOT14 | 14 | -0.0103 | -6.42 |
| 481 | 26 | 1007.4830 | 1008.4903 | 1638.42 | 1 | 1007.4892 | C8 | 8 | -6.15e-03 | -6.10 |
| 481 | 27 | 1756.8597 | 879.4371 | 1337.78 | 2 | 1756.8712 | C14 | 14 | -0.0115 | -6.57 |
| 481 | 28 | 3312.8169 | 829.2115 | 1518.57 | 4 |  |  |  |  |  |
| 481 | 29 | 1756.8599 | 586.6273 | 920.11 | 3 | 1756.8712 | C14 | 14 | -0.0113 | -6.40 |
| 481 | 30 | 2290.3178 | 573.5867 | 1309.39 | 4 |  |  |  |  |  |
| 481 | 31 | 1643.7770 | 822.8958 | 963.07 | 2 | 1643.7871 | C13 | 13 | -0.0101 | -6.16 |
| 481 | 32 | 2222.2983 | 741.7734 | 1622.00 | 3 |  |  |  |  |  |
| 481 | 33 | 2798.6469 | 700.6690 | 1335.98 | 4 |  |  |  |  |  |
| 481 | 34 | 2680.4581 | 671.1218 | 1547.14 | 4 |  |  |  |  |  |
| 481 | 35 | 1637.6867 | 546.9028 | 2162.45 | 3 |  |  |  |  |  |
| 481 | 36 | 2419.3802 | 605.8523 | 840.33 | 4 |  |  |  |  |  |
| 481 | 37 | 3340.8158 | 836.2112 | 707.60 | 4 |  |  |  |  |  |
| 481 | 38 | 1195.7331 | 598.8738 | 1305.95 | 2 | 1195.7415 | Z\_DOT11 | 17 | -8.39e-03 | -7.02 |
| 481 | 39 | 1119.5209 | 1120.5281 | 875.05 | 1 |  |  |  |  |  |
| 481 | 40 | 1263.5978 | 632.8062 | 855.01 | 2 | 1263.6063 | C10 | 10 | -8.52e-03 | -6.74 |
| 481 | 41 | 1308.8169 | 655.4157 | 1239.32 | 2 | 1308.8255 | Z\_DOT12 | 16 | -8.59e-03 | -6.56 |
| 481 | 42 | 1135.5406 | 568.7776 | 997.58 | 2 | 1135.5477 | C9 | 9 | -7.19e-03 | -6.33 |
| 481 | 43 | 697.7452 | 698.7525 | 727.69 | 1 |  |  |  |  |  |
| 481 | 44 | 839.4577 | 840.4650 | 696.64 | 1 |  |  |  |  |  |
| 481 | 45 | 1100.5049 | 551.2597 | 701.75 | 2 |  |  |  |  |  |
| 481 | 46 | 897.5949 | 449.8047 | 563.81 | 2 |  |  |  |  |  |
| 481 | 47 | 838.5229 | 420.2687 | 913.25 | 2 |  |  |  |  |  |
| 481 | 48 | 1066.6923 | 534.3534 | 628.55 | 2 | 1066.6989 | Z\_DOT10 | 18 | -6.57e-03 | -6.16 |
| 481 | 49 | 1342.7352 | 672.3749 | 4289.38 | 2 |  |  |  |  |  |
| 481 | 50 | 1238.1299 | 620.0722 | 567.22 | 2 |  |  |  |  |  |
| 481 | 51 | 1083.4768 | 542.7457 | 522.61 | 2 |  |  |  |  |  |
| 481 | 52 | 783.1158 | 784.1230 | 782.02 | 1 |  |  |  |  |  |

  

All proteins /
CsTx-12b Cupiennius salei toxin 12 isoform b /
Proteoform #39
