# Supplementary material for: The Dual Prey-Inactivation Strategy of Spiders—In-Depth Venomic Analysis of Cupiennius salei
Source: Toxins (Basel). 2019 Mar 19;11(3):167. doi: 10.3390/toxins11030167 (PMC6468893; doi:10.3390/toxins11030167)
Supplement: Supplementary file 1 [file toxins-11-00167-s001.zip › Supplementary Dataset EV1/20180328_f2_topdown_OTMS2_EThcD_NL_i02_ms2_proteoform_cutoff_html/prsms/prsm125.html]

Protein-Spectrum-Match for Spectrum #360


All proteins /
CsTx-1a\_S1 Cupiennius salei toxin 1 isoform a S1^ACsTx-1a\_S2 Cupiennius salei toxin 1 isoform a S2 /
Proteoform #42

## Protein-Spectrum-Match #125 for Spectrum #360

|  |  |  |  |  |  |
| --- | --- | --- | --- | --- | --- |
| PrSM ID: | 125 | Scan(s): | 483 | Precursor charge: | 7 |
| Precursor m/z: | 1027.4605 | Precursor mass: | 7185.1729 | Proteoform mass: | 7185.1379 |
| # matched peaks: | 19 | # matched fragment ions: | 20 | # unexpected modifications: | 1 |
| E-value: | 9.37e-17 | P-value: | 9.37e-17 | Q-value (Spectral FDR): | 0 |

  

|  |  |  |  |  |  |  |  |  |  |  |  |  |  |  |  |  |  |  |  |  |  |  |  |  |  |  |  |  |  |  |  |  |  |  |  |  |  |  |  |  |  |  |  |  |  |  |  |  |  |  |  |  |  |  |  |  |  |  |  |  |  |  |  |  |  |  |  |  |  |
| --- | --- | --- | --- | --- | --- | --- | --- | --- | --- | --- | --- | --- | --- | --- | --- | --- | --- | --- | --- | --- | --- | --- | --- | --- | --- | --- | --- | --- | --- | --- | --- | --- | --- | --- | --- | --- | --- | --- | --- | --- | --- | --- | --- | --- | --- | --- | --- | --- | --- | --- | --- | --- | --- | --- | --- | --- | --- | --- | --- | --- | --- | --- | --- | --- | --- | --- | --- | --- | --- |
|  | |  | | | | | | | | | | | | | | | | | | | | | | | | | | | | | | | | | | | | | | | | | | | | | | | | | | | | | | | | | | | | | | | | | | | |
| 1 |  |  | M |  | K |  | V |  | L |  | I |  | I |  | S |  | A |  | V |  | L |  |  | F |  | I |  | T |  | I |  | F |  | S |  | N |  | I |  | S |  | A |  |  | E |  | I |  | E |  | D |  | D |  | F |  | L |  | E |  | D |  | E |  | 30 |  |
|  | |  | | | | | | | | | | | | | | | | | | | | | | | | | | | | | | | | | | | | | | | | | | | | | | | | | | | | | | | | | | | | | | | | | | | |
| 31 |  |  | S |  | F |  | E |  | A |  | E |  | D |  | I |  | I |  | P |  | F |  |  | F |  | E |  | N |  | E |  | Q |  | A |  | R | ] | S |  | C |  | I |  | ⎩ | P |  | K | ⎫ | H | ⎫ | E | ⎱ | E | ⎫ | C |  | T |  | N |  | D |  | K |  | 60 |  |
|  | |  | | | | | | | | | | | | | | | | | | | | | | | | | | | | | | | | | | 15.95 | | | | | | | | | | | | | | | | | | | | | | | | | | | | | | |
| 61 |  |  | H | ⎫ | N | ⎫ | C |  | C |  | R |  | K |  | G |  | L |  | F |  | K |  | ⎫ | L |  | K | ⎫ | C | ⎫ | Q |  | C |  | S | ⎫ | T |  | F |  | D |  | D |  |  | E |  | S |  | G |  | Q |  | P |  | T |  | E |  | R |  | C |  | A |  | 90 |  |
|  | |  | | | | | | | | | | | | | | | | | | | | | | | | | | | | | | | | | | | | | | | | | | | | | | | | | | | | | | | | | | | | | | | | | | | |
| 91 |  |  | C |  | G |  | R |  | P | ⎫ | M |  | G | ⎱ | H | ⎫ | Q | ⎫ | A |  | I |  |  | E | ⎫ | T |  | G |  | L | ⎫ | N |  | I | ⎫ | F | [ | R |  | G |  | L |  |  | F |  | K |  | G |  | K |  | K |  | K |  | N |  | K |  | K |  | T |  | 120 |  |
|  | |  | | | | | | | | | | | | | | | | | | | | | | | | | | | | | | | | | | | | | | | | | | | | | | | | | | | | | | | | | | | | | | | | | | | |
| 121 |  |  | K |  | G |  | | | | 122 |  | | | | | | | | | | | | | | | | | | | | | | | | | | | | | | | | | | | | | | | | | | | | | | | | | | | | | | | |

Fixed PTMs: Carbamidomethylation [C49 C56 C63 C64 C73 C75 C89 C91 ]   
  
     Unexpected modifications:   Unknown [15.95]

  

All peaks (67)  Matched peaks (19)  Not matched peaks (48)

  

| Scan | Peak | Mono mass | Mono m/z | Intensity | Charge | Theoretical mass | Ion | Pos | Mass error | PPM error |
| --- | --- | --- | --- | --- | --- | --- | --- | --- | --- | --- |
| 483 | 1 | 7128.1077 | 1189.0252 | 80812.22 | 6 |  |  |  |  |  |
| 483 | 2 | 7175.1472 | 1026.0283 | 185609.83 | 7 |  |  |  |  |  |
| 483 | 3 | 3593.0674 | 1198.6964 | 42439.24 | 3 |  |  |  |  |  |
| 483 | 4 | 7176.1465 | 1197.0317 | 64448.72 | 6 |  |  |  |  |  |
| 483 | 5 | 7078.1273 | 1012.1683 | 17390.05 | 7 |  |  |  |  |  |
| 483 | 6 | 2395.7126 | 1198.8636 | 27835.68 | 2 |  |  |  |  |  |
| 483 | 7 | 1026.7347 | 1027.7420 | 50508.60 | 1 |  |  |  |  |  |
| 483 | 8 | 7141.1166 | 1191.1934 | 15624.37 | 6 |  |  |  |  |  |
| 483 | 9 | 7129.1137 | 1426.8300 | 10130.53 | 5 |  |  |  |  |  |
| 483 | 10 | 7078.1280 | 1180.6953 | 7672.85 | 6 |  |  |  |  |  |
| 483 | 11 | 3157.4950 | 1053.5056 | 8579.94 | 3 | 3157.5153 | C25 | 25 | -0.0203 | -6.44 |
| 483 | 12 | 7096.1236 | 1183.6945 | 7583.83 | 6 |  |  |  |  |  |
| 483 | 13 | 2782.3040 | 928.4419 | 7218.19 | 3 |  |  |  |  |  |
| 483 | 14 | 868.4173 | 869.4246 | 9304.54 | 1 | 868.4225 | C7 | 7 | -5.12e-03 | -5.89 |
| 483 | 15 | 7037.0970 | 1173.8568 | 7454.17 | 6 | 7037.0854 | C59 | 59 | 0.0115 | 1.64 |
| 483 | 16 | 1752.7563 | 877.3854 | 7331.96 | 2 | 1752.7671 | C14 | 14 | -0.0109 | -6.20 |
| 483 | 17 | 1866.7972 | 934.4059 | 5112.75 | 2 | 1866.8101 | C15 | 15 | -0.0128 | -6.88 |
| 483 | 18 | 2872.3025 | 958.4415 | 4513.91 | 3 |  |  |  |  |  |
| 483 | 19 | 6993.0711 | 1166.5191 | 5361.93 | 6 |  |  |  |  |  |
| 483 | 20 | 6225.6358 | 1246.1344 | 4113.76 | 5 | 6225.6415 | C51 | 51 | -5.69e-03 | -0.91 |
| 483 | 21 | 602.3177 | 603.3250 | 6574.34 | 1 | 602.3210 | C5 | 5 | -3.29e-03 | -5.46 |
| 483 | 22 | 4445.9059 | 1112.4838 | 3359.26 | 4 |  |  |  |  |  |
| 483 | 23 | 2916.3173 | 973.1130 | 3083.44 | 3 | 2916.3363 | C23 | 23 | -0.0191 | -6.54 |
| 483 | 24 | 739.3758 | 740.3831 | 4926.36 | 1 | 739.3799 | C6 | 6 | -4.03e-03 | -5.45 |
| 483 | 25 | 6097.5775 | 1220.5228 | 3822.33 | 5 | 6097.5829 | C50 | 50 | -5.40e-03 | -0.88 |
| 483 | 26 | 5960.5394 | 1193.1152 | 4069.14 | 5 | 5960.5240 | C49 | 49 | 0.0154 | 2.58 |
| 483 | 27 | 3184.5148 | 1062.5122 | 1990.12 | 3 |  |  |  |  |  |
| 483 | 28 | 7070.0879 | 1415.0249 | 2495.51 | 5 |  |  |  |  |  |
| 483 | 29 | 997.4591 | 998.4664 | 2885.12 | 1 | 997.4651 | C8 | 8 | -5.96e-03 | -5.98 |
| 483 | 30 | 6318.7255 | 1264.7524 | 2125.36 | 5 | 6317.7233 | Z\_DOT53 | 7 | -9.49e-05 | -0.02 |
| 483 | 31 | 3446.5838 | 1149.8686 | 2482.24 | 3 |  |  |  |  |  |
| 483 | 32 | 4723.4040 | 1181.8583 | 4958.51 | 4 |  |  |  |  |  |
| 483 | 33 | 7143.1270 | 1429.6327 | 2335.43 | 5 |  |  |  |  |  |
| 483 | 34 | 2027.8291 | 1014.9218 | 1368.77 | 2 |  |  |  |  |  |
| 483 | 35 | 5772.4663 | 1155.5005 | 2394.77 | 5 | 5772.4620 | C47 | 47 | 4.27e-03 | 0.74 |
| 483 | 36 | 7149.0878 | 1022.3055 | 1913.71 | 7 |  |  |  |  |  |
| 483 | 37 | 7176.1250 | 1436.2323 | 4695.84 | 5 |  |  |  |  |  |
| 483 | 38 | 4271.8525 | 1424.9581 | 2385.83 | 3 |  |  |  |  |  |
| 483 | 39 | 5507.3229 | 1102.4719 | 1352.96 | 5 |  |  |  |  |  |
| 483 | 40 | 2744.2042 | 915.7420 | 1347.48 | 3 |  |  |  |  |  |
| 483 | 41 | 1438.2300 | 1439.2372 | 2200.58 | 1 |  |  |  |  |  |
| 483 | 42 | 2888.2983 | 963.7734 | 1218.39 | 3 |  |  |  |  |  |
| 483 | 43 | 3692.6405 | 924.1674 | 842.21 | 4 | 3692.6673 | C29 | 29 | -0.0268 | -7.26 |
| 483 | 44 | 4460.9015 | 1116.2327 | 1249.92 | 4 |  |  |  |  |  |
| 483 | 45 | 1317.5648 | 1318.5721 | 1061.18 | 1 |  |  |  |  |  |
| 483 | 46 | 3923.6654 | 1308.8958 | 1139.75 | 3 |  |  |  |  |  |
| 483 | 47 | 3317.5218 | 1106.8479 | 1339.99 | 3 | 3317.5460 | C26 | 26 | -0.0242 | -7.29 |
| 483 | 48 | 6808.9458 | 1135.8316 | 929.16 | 6 | 6808.9725 | Z\_DOT57 | 3 | -0.0267 | -3.92 |
| 483 | 48 | 6808.9458 | 1135.8316 | 929.16 | 6 | 6809.9584 | C57 | 57 | -0.0103 | -1.51 |
| 483 | 49 | 7019.0395 | 1003.7272 | 2036.60 | 7 |  |  |  |  |  |
| 483 | 50 | 6597.8430 | 1320.5759 | 1306.86 | 5 |  |  |  |  |  |
| 483 | 51 | 7039.1084 | 1408.8290 | 685.80 | 5 |  |  |  |  |  |
| 483 | 52 | 1768.7503 | 885.3824 | 1075.79 | 2 |  |  |  |  |  |
| 483 | 53 | 3636.5722 | 1213.1980 | 827.52 | 3 |  |  |  |  |  |
| 483 | 54 | 6538.8018 | 1308.7676 | 1053.98 | 5 | 6538.8052 | C54 | 54 | -3.43e-03 | -0.53 |
| 483 | 55 | 1422.8231 | 1423.8304 | 518.40 | 1 |  |  |  |  |  |
| 483 | 56 | 1174.8474 | 1175.8547 | 821.71 | 1 |  |  |  |  |  |
| 483 | 57 | 1343.3867 | 1344.3940 | 624.87 | 1 |  |  |  |  |  |
| 483 | 58 | 1970.3762 | 986.1954 | 942.16 | 2 |  |  |  |  |  |
| 483 | 59 | 1290.5548 | 1291.5621 | 824.03 | 1 |  |  |  |  |  |
| 483 | 60 | 1212.1902 | 1213.1974 | 415.86 | 1 |  |  |  |  |  |
| 483 | 61 | 1225.6156 | 1226.6229 | 561.11 | 1 | 1225.6217 | Z\_DOT11 | 49 | -6.16e-03 | -5.03 |
| 483 | 62 | 4709.4109 | 1178.3600 | 1073.05 | 4 |  |  |  |  |  |
| 483 | 63 | 1329.5776 | 1330.5849 | 701.74 | 1 |  |  |  |  |  |
| 483 | 64 | 6247.7316 | 1250.5536 | 1248.73 | 5 |  |  |  |  |  |
| 483 | 65 | 2327.3561 | 1164.6853 | 1902.97 | 2 |  |  |  |  |  |
| 483 | 66 | 1263.5449 | 1264.5522 | 631.31 | 1 |  |  |  |  |  |
| 483 | 67 | 1242.1400 | 1243.1473 | 935.50 | 1 |  |  |  |  |  |

  

All proteins /
CsTx-1a\_S1 Cupiennius salei toxin 1 isoform a S1^ACsTx-1a\_S2 Cupiennius salei toxin 1 isoform a S2 /
Proteoform #42
