# Supplementary material for: The Dual Prey-Inactivation Strategy of Spiders—In-Depth Venomic Analysis of Cupiennius salei
Source: Toxins (Basel). 2019 Mar 19;11(3):167. doi: 10.3390/toxins11030167 (PMC6468893; doi:10.3390/toxins11030167)
Supplement: Supplementary file 1 [file toxins-11-00167-s001.zip › Supplementary Dataset EV1/20180328_f2_topdown_OTMS2_EThcD_NL_i02_ms2_proteoform_cutoff_html/prsms/prsm126.html]

Protein-Spectrum-Match for Spectrum #363


All proteins /
CsTx-12b Cupiennius salei toxin 12 isoform b /
Proteoform #47

## Protein-Spectrum-Match #126 for Spectrum #363

|  |  |  |  |  |  |
| --- | --- | --- | --- | --- | --- |
| PrSM ID: | 126 | Scan(s): | 487 | Precursor charge: | 6 |
| Precursor m/z: | 571.9902 | Precursor mass: | 3425.8978 | Proteoform mass: | 3425.8978 |
| # matched peaks: | 19 | # matched fragment ions: | 18 | # unexpected modifications: | 1 |
| E-value: | 2.85e-16 | P-value: | 2.85e-16 | Q-value (Spectral FDR): | 0 |

  

|  |  |  |  |  |  |  |  |  |  |  |  |  |  |  |  |  |  |  |  |  |  |  |  |  |  |  |  |  |  |  |  |  |  |  |  |  |  |  |  |  |  |  |  |  |  |  |  |  |  |  |  |  |  |  |  |  |  |  |  |  |  |  |  |  |  |  |
| --- | --- | --- | --- | --- | --- | --- | --- | --- | --- | --- | --- | --- | --- | --- | --- | --- | --- | --- | --- | --- | --- | --- | --- | --- | --- | --- | --- | --- | --- | --- | --- | --- | --- | --- | --- | --- | --- | --- | --- | --- | --- | --- | --- | --- | --- | --- | --- | --- | --- | --- | --- | --- | --- | --- | --- | --- | --- | --- | --- | --- | --- | --- | --- | --- | --- | --- |
|  | | ... 30 amino acid residues are skipped at the N-terminus ... | | | | | | | | | | | | | | | | | | | | | | | | | | | | | | | | | | | | | | | | | | | | | | | | | | | | | | | | | | | | | |  | | |
|  | |  | | | | | | | | | | | | | | | | | | | | | | | | | | | | | | | | | | | | | | | | | | | | | | | | | | | | | | | | | | | | | | | | | | | |
| 31 |  |  | S |  | F |  | E |  | A |  | D |  | D |  | V |  | I |  | P |  | F |  |  | L |  | A |  | R |  | E |  | Q |  | V |  | R |  | S |  | D |  | C |  |  | T |  | L |  | R |  | N |  | H |  | D |  | C |  | T |  | D |  | D |  | 60 |  |
|  | |  | | | | | | | | | | | | | | | | | | | | | | | | | | | | | | | | | | | | | | | | | | | | | | | | | | | | | | | | | | | | | | | | | | | |
| 61 |  |  | R |  | H |  | S |  | C |  | C |  | R |  | S |  | K |  | M |  | F |  |  | K |  | D |  | V |  | C |  | K |  | C |  | F |  | Y |  | P |  | S |  |  | Q |  | R |  | S |  | D |  | T |  | A |  | R | ] | A |  | K | ⎩ | K |  | 90 |  |
|  | |  | | | | | | | | | | | | | | | | | | | | | | | | | | | | | | | | | | | | | | | | | | | | | | | | | | | | | -58.01 | | | | | | | | | | | |
| 91 |  |  | E |  | L |  | C |  | T |  | C | ⎫ | Q | ⎫ | Q |  | D | ⎱ | K |  | H |  |  | L |  | K | ⎱ | Y |  | I | ⎱ | E | ⎫ | K |  | G | ⎫ | L |  | Q | ⎱ | K |  | ⎫ | A | ⎫ | K | ⎫ | V | ⎫ | L |  | V | ⎫ | A |  | G |  | | 117 |  | | | | | |

Fixed PTMs: Carbamidomethylation [C93 C95 ]   
  
     Unexpected modifications:   Unknown [-58.01]

  

All peaks (57)  Matched peaks (19)  Not matched peaks (38)

  

| Scan | Peak | Mono mass | Mono m/z | Intensity | Charge | Theoretical mass | Ion | Pos | Mass error | PPM error |
| --- | --- | --- | --- | --- | --- | --- | --- | --- | --- | --- |
| 487 | 1 | 3337.8269 | 668.5726 | 895081.49 | 5 |  |  |  |  |  |
| 487 | 2 | 3408.8785 | 569.1537 | 636673.56 | 6 |  |  |  |  |  |
| 487 | 3 | 3368.8663 | 674.7805 | 507218.33 | 5 |  |  |  |  |  |
| 487 | 4 | 571.3158 | 572.3231 | 2527202.42 | 1 |  |  |  |  |  |
| 487 | 5 | 3309.8020 | 662.9677 | 344741.00 | 5 |  |  |  |  |  |
| 487 | 6 | 3354.8526 | 671.9778 | 143291.13 | 5 | 3354.8631 | C28 | 28 | -0.0105 | -3.13 |
| 487 | 7 | 1142.9569 | 572.4857 | 2465411.05 | 2 |  |  |  |  |  |
| 487 | 8 | 3142.7034 | 786.6831 | 136073.47 | 4 | 3142.7106 | C26 | 26 | -7.23e-03 | -2.30 |
| 487 | 9 | 3238.7596 | 648.7592 | 128869.67 | 5 |  |  |  |  |  |
| 487 | 10 | 3380.8802 | 564.4873 | 117041.74 | 6 |  |  |  |  |  |
| 487 | 11 | 3408.8661 | 682.7805 | 149125.74 | 5 |  |  |  |  |  |
| 487 | 12 | 2161.1085 | 721.3768 | 81928.71 | 3 | 2161.1135 | C17 | 17 | -5.09e-03 | -2.36 |
| 487 | 13 | 3337.8314 | 557.3125 | 62567.40 | 6 |  |  |  |  |  |
| 487 | 14 | 3210.7410 | 803.6925 | 63923.88 | 4 | 3210.7471 | Z\_DOT28 | 2 | -6.07e-03 | -1.89 |
| 487 | 15 | 3281.8022 | 657.3677 | 73665.57 | 5 |  |  |  |  |  |
| 487 | 16 | 3226.7591 | 646.3591 | 79668.31 | 5 |  |  |  |  |  |
| 487 | 17 | 2048.2692 | 683.7637 | 76178.27 | 3 | 2048.2724 | Z\_DOT19 | 11 | -3.20e-03 | -1.56 |
| 487 | 18 | 2475.2669 | 826.0962 | 55489.24 | 3 | 2475.2726 | C20 | 20 | -5.65e-03 | -2.28 |
| 487 | 19 | 1884.9613 | 629.3277 | 77190.74 | 3 | 1884.9662 | C15 | 15 | -4.90e-03 | -2.60 |
| 487 | 20 | 3381.8753 | 677.3823 | 54506.55 | 5 |  |  |  |  |  |
| 487 | 21 | 2290.1511 | 764.3910 | 54601.21 | 3 | 2290.1561 | C18 | 18 | -5.01e-03 | -2.19 |
| 487 | 22 | 3196.7118 | 640.3496 | 72063.11 | 5 |  |  |  |  |  |
| 487 | 23 | 2915.5398 | 729.8922 | 53762.86 | 4 | 2915.5473 | C24 | 24 | -7.42e-03 | -2.54 |
| 487 | 24 | 2844.5020 | 712.1328 | 42659.39 | 4 | 2844.5102 | C23 | 23 | -8.15e-03 | -2.87 |
| 487 | 25 | 2361.3829 | 591.3530 | 42345.99 | 4 |  |  |  |  |  |
| 487 | 26 | 3043.6350 | 761.9160 | 45683.19 | 4 | 3043.6422 | C25 | 25 | -7.18e-03 | -2.36 |
| 487 | 27 | 3320.8054 | 665.1684 | 43116.52 | 5 |  |  |  |  |  |
| 487 | 28 | 1867.9341 | 623.6520 | 51969.75 | 3 |  |  |  |  |  |
| 487 | 29 | 1378.6292 | 690.3219 | 52300.51 | 2 | 1378.6333 | C11 | 11 | -4.08e-03 | -2.96 |
| 487 | 30 | 3209.7321 | 642.9537 | 51166.56 | 5 |  |  |  |  |  |
| 487 | 31 | 1361.6036 | 681.8091 | 81147.65 | 2 |  |  |  |  |  |
| 487 | 32 | 1541.9368 | 771.9757 | 68161.11 | 2 | 1541.9395 | Z\_DOT15 | 15 | -2.68e-03 | -1.74 |
| 487 | 33 | 685.5796 | 686.5869 | 290298.94 | 1 |  |  |  |  |  |
| 487 | 34 | 2716.4075 | 680.1092 | 36156.59 | 4 | 2716.4152 | C22 | 22 | -7.68e-03 | -2.83 |
| 487 | 35 | 3110.6631 | 778.6731 | 39169.76 | 4 |  |  |  |  |  |
| 487 | 36 | 3181.7417 | 637.3556 | 35173.94 | 5 |  |  |  |  |  |
| 487 | 37 | 3423.9133 | 571.6595 | 6294976.91 | 6 |  |  |  |  |  |
| 487 | 38 | 1265.7908 | 633.9027 | 32686.26 | 2 | 1265.7921 | Z\_DOT13 | 17 | -1.29e-03 | -1.02 |
| 487 | 39 | 908.5789 | 455.2967 | 32850.83 | 2 |  |  |  |  |  |
| 487 | 40 | 662.9614 | 663.9686 | 20226.31 | 1 |  |  |  |  |  |
| 487 | 41 | 1469.8940 | 735.9543 | 10805.44 | 2 |  |  |  |  |  |
| 487 | 42 | 1007.4878 | 1008.4951 | 13656.22 | 1 | 1007.4892 | C8 | 8 | -1.39e-03 | -1.38 |
| 487 | 43 | 1206.7790 | 604.3968 | 11388.52 | 2 |  |  |  |  |  |
| 487 | 44 | 1489.6984 | 745.8565 | 11198.04 | 2 |  |  |  |  |  |
| 487 | 45 | 1455.2746 | 728.6446 | 10492.64 | 2 |  |  |  |  |  |
| 487 | 46 | 936.6106 | 937.6179 | 8197.26 | 1 |  |  |  |  |  |
| 487 | 47 | 1394.8933 | 698.4539 | 7390.88 | 2 |  |  |  |  |  |
| 487 | 48 | 1135.5461 | 1136.5534 | 5696.41 | 1 | 1135.5477 | C9 | 9 | -1.62e-03 | -1.43 |
| 487 | 49 | 710.4915 | 711.4988 | 6620.72 | 1 | 710.4904 | Z\_DOT8 | 22 | 1.06e-03 | 1.49 |
| 487 | 50 | 1007.4875 | 504.7510 | 6971.25 | 2 | 1007.4892 | C8 | 8 | -1.65e-03 | -1.63 |
| 487 | 51 | 873.4724 | 874.4797 | 5060.10 | 1 |  |  |  |  |  |
| 487 | 52 | 726.5104 | 727.5176 | 4825.98 | 1 |  |  |  |  |  |
| 487 | 53 | 1281.8087 | 641.9117 | 6606.74 | 2 |  |  |  |  |  |
| 487 | 54 | 837.5420 | 838.5493 | 4742.43 | 1 |  |  |  |  |  |
| 487 | 55 | 1441.8623 | 721.9384 | 27248.47 | 2 |  |  |  |  |  |
| 487 | 56 | 1162.4728 | 1163.4800 | 4094.69 | 1 |  |  |  |  |  |
| 487 | 57 | 598.4153 | 599.4225 | 6270.38 | 1 |  |  |  |  |  |

  

All proteins /
CsTx-12b Cupiennius salei toxin 12 isoform b /
Proteoform #47
