# Supplementary material for: The Dual Prey-Inactivation Strategy of Spiders—In-Depth Venomic Analysis of Cupiennius salei
Source: Toxins (Basel). 2019 Mar 19;11(3):167. doi: 10.3390/toxins11030167 (PMC6468893; doi:10.3390/toxins11030167)
Supplement: Supplementary file 1 [file toxins-11-00167-s001.zip › Supplementary Dataset EV1/20180328_f2_topdown_OTMS2_EThcD_NL_i02_ms2_proteoform_cutoff_html/prsms/prsm127.html]

Protein-Spectrum-Match for Spectrum #364


All proteins /
CsTx-1a\_S1 Cupiennius salei toxin 1 isoform a S1^ACsTx-1a\_S2 Cupiennius salei toxin 1 isoform a S2 /
Proteoform #8

## Protein-Spectrum-Match #127 for Spectrum #364

|  |  |  |  |  |  |
| --- | --- | --- | --- | --- | --- |
| PrSM ID: | 127 | Scan(s): | 488 | Precursor charge: | 7 |
| Precursor m/z: | 1025.1762 | Precursor mass: | 7169.1828 | Proteoform mass: | 7169.1856 |
| # matched peaks: | 47 | # matched fragment ions: | 46 | # unexpected modifications: | 0 |
| E-value: | 1.90e-41 | P-value: | 1.90e-41 | Q-value (Spectral FDR): | 0 |

  

|  |  |  |  |  |  |  |  |  |  |  |  |  |  |  |  |  |  |  |  |  |  |  |  |  |  |  |  |  |  |  |  |  |  |  |  |  |  |  |  |  |  |  |  |  |  |  |  |  |  |  |  |  |  |  |  |  |  |  |  |  |  |  |  |  |  |  |  |  |  |
| --- | --- | --- | --- | --- | --- | --- | --- | --- | --- | --- | --- | --- | --- | --- | --- | --- | --- | --- | --- | --- | --- | --- | --- | --- | --- | --- | --- | --- | --- | --- | --- | --- | --- | --- | --- | --- | --- | --- | --- | --- | --- | --- | --- | --- | --- | --- | --- | --- | --- | --- | --- | --- | --- | --- | --- | --- | --- | --- | --- | --- | --- | --- | --- | --- | --- | --- | --- | --- | --- |
|  | |  | | | | | | | | | | | | | | | | | | | | | | | | | | | | | | | | | | | | | | | | | | | | | | | | | | | | | | | | | | | | | | | | | | | |
| 1 |  |  | M |  | K |  | V |  | L |  | I |  | I |  | S |  | A |  | V |  | L |  |  | F |  | I |  | T |  | I |  | F |  | S |  | N |  | I |  | S |  | A |  |  | E |  | I |  | E |  | D |  | D |  | F |  | L |  | E |  | D |  | E |  | 30 |  |
|  | |  | | | | | | | | | | | | | | | | | | | | | | | | | | | | | | | | | | | | | | | | | | | | | | | | | | | | | | | | | | | | | | | | | | | |
| 31 |  |  | S |  | F |  | E |  | A |  | E |  | D |  | I |  | I |  | P |  | F |  |  | F |  | E |  | N |  | E |  | Q |  | A |  | R | ] | S |  | C |  | I |  | ⎩ | P | ⎱ | K | ⎱ | H | ⎱ | E | ⎱ | E | ⎫ | C | ⎩ | T | ⎱ | N | ⎩ | D |  | K |  | 60 |  |
|  | |  | | | | | | | | | | | | | | | | | | | | | | | | | | | | | | | | | | | | | | | | | | | | | | | | | | | | | | | | | | | | | | | | | | | |
| 61 |  |  | H | ⎱ | N | ⎫ | C | ⎫ | C |  | R |  | K | ⎫ | G |  | L |  | F |  | K |  | ⎫ | L |  | K | ⎫ | C | ⎫ | Q | ⎫ | C | ⎱ | S | ⎫ | T |  | F | ⎫ | D | ⎫ | D |  | ⎫ | E |  | S |  | G | ⎱ | Q |  | P |  | T |  | E | ⎱ | R |  | C |  | A |  | 90 |  |
|  | |  | | | | | | | | | | | | | | | | | | | | | | | | | | | | | | | | | | | | | | | | | | | | | | | | | | | | | | | | | | | | | | | | | | | |
| 91 |  |  | C |  | G | ⎱ | R |  | P | ⎫ | M | ⎫ | G | ⎫ | H | ⎱ | Q | ⎱ | A |  | I |  |  | E | ⎫ | T | ⎫ | G |  | L | ⎫ | N |  | I | ⎫ | F | [ | R |  | G |  | L |  |  | F |  | K |  | G |  | K |  | K |  | K |  | N |  | K |  | K |  | T |  | 120 |  |
|  | |  | | | | | | | | | | | | | | | | | | | | | | | | | | | | | | | | | | | | | | | | | | | | | | | | | | | | | | | | | | | | | | | | | | | |
| 121 |  |  | K |  | G |  | | | | 122 |  | | | | | | | | | | | | | | | | | | | | | | | | | | | | | | | | | | | | | | | | | | | | | | | | | | | | | | | |

Fixed PTMs: Carbamidomethylation [C49 C56 C63 C64 C73 C75 C89 C91 ]

  

All peaks (113)  Matched peaks (47)  Not matched peaks (66)

  

| Scan | Peak | Mono mass | Mono m/z | Intensity | Charge | Theoretical mass | Ion | Pos | Mass error | PPM error |
| --- | --- | --- | --- | --- | --- | --- | --- | --- | --- | --- |
| 488 | 1 | 7112.1233 | 1186.3612 | 454090.05 | 6 |  |  |  |  |  |
| 488 | 2 | 3585.0727 | 1196.0315 | 297274.55 | 3 |  |  |  |  |  |
| 488 | 3 | 7125.1297 | 1188.5289 | 85657.22 | 6 |  |  |  |  |  |
| 488 | 4 | 7153.1346 | 1193.1964 | 50006.85 | 6 |  |  |  |  |  |
| 488 | 5 | 7080.1429 | 1181.0311 | 41703.67 | 6 |  |  |  |  |  |
| 488 | 6 | 7021.1053 | 1171.1915 | 38690.91 | 6 | 7021.1332 | C59 | 59 | -0.0279 | -3.97 |
| 488 | 7 | 1024.5958 | 1025.6031 | 555143.32 | 1 |  |  |  |  |  |
| 488 | 8 | 6976.0873 | 1163.6885 | 38492.55 | 6 |  |  |  |  |  |
| 488 | 9 | 7113.1238 | 1423.6320 | 43674.59 | 5 |  |  |  |  |  |
| 488 | 10 | 3157.4979 | 1053.5066 | 35181.79 | 3 | 3157.5153 | C25 | 25 | -0.0174 | -5.52 |
| 488 | 11 | 6209.6470 | 1242.9367 | 23537.19 | 5 | 6209.6892 | C51 | 51 | -0.0422 | -6.79 |
| 488 | 12 | 7097.1131 | 1183.8595 | 24616.26 | 6 |  |  |  |  |  |
| 488 | 13 | 6081.5733 | 1217.3219 | 29253.32 | 5 | 6081.6306 | C50 | 50 | -0.0573 | -9.43 |
| 488 | 14 | 5944.5336 | 1189.9140 | 19916.09 | 5 | 5944.5717 | C49 | 49 | -0.0382 | -6.42 |
| 488 | 15 | 2782.3051 | 928.4423 | 24202.60 | 3 |  |  |  |  |  |
| 488 | 16 | 7035.1322 | 1173.5293 | 22009.89 | 6 |  |  |  |  |  |
| 488 | 17 | 4443.9030 | 1111.9830 | 20024.87 | 4 | 4443.9333 | C36 | 36 | -0.0303 | -6.82 |
| 488 | 18 | 7005.0571 | 1001.7297 | 21075.78 | 7 |  |  |  |  |  |
| 488 | 19 | 1752.7574 | 877.3860 | 29252.59 | 2 | 1752.7671 | C14 | 14 | -9.77e-03 | -5.57 |
| 488 | 20 | 7063.1302 | 1178.1956 | 21830.63 | 6 |  |  |  |  |  |
| 488 | 21 | 2872.3071 | 958.4430 | 17658.22 | 3 |  |  |  |  |  |
| 488 | 22 | 7126.1358 | 1426.2344 | 12042.60 | 5 |  |  |  |  |  |
| 488 | 23 | 1866.8004 | 934.4075 | 18260.14 | 2 | 1866.8101 | C15 | 15 | -9.67e-03 | -5.18 |
| 488 | 24 | 1195.5246 | 1196.5318 | 136841.56 | 1 |  |  |  |  |  |
| 488 | 25 | 3183.5102 | 1062.1774 | 11391.76 | 3 |  |  |  |  |  |
| 488 | 26 | 5887.5212 | 1178.5115 | 23000.67 | 5 | 5887.5503 | C48 | 48 | -0.0291 | -4.93 |
| 488 | 27 | 868.4182 | 869.4255 | 28785.78 | 1 | 868.4225 | C7 | 7 | -4.26e-03 | -4.91 |
| 488 | 28 | 5756.4678 | 1152.3008 | 13101.94 | 5 | 5756.5098 | C47 | 47 | -0.0420 | -7.30 |
| 488 | 29 | 7152.1327 | 1022.7405 | 13903.20 | 7 |  |  |  |  |  |
| 488 | 30 | 3922.6514 | 1308.5577 | 8346.40 | 3 |  |  |  |  |  |
| 488 | 31 | 7154.1412 | 1431.8355 | 9052.67 | 5 |  |  |  |  |  |
| 488 | 32 | 6301.7430 | 1261.3559 | 11654.16 | 5 | 6301.7710 | Z\_DOT53 | 7 | -0.0281 | -4.45 |
| 488 | 33 | 7055.1038 | 1412.0280 | 9657.60 | 5 |  |  |  |  |  |
| 488 | 34 | 3445.5881 | 1149.5366 | 11667.54 | 3 | 3445.6046 | C27 | 27 | -0.0165 | -4.78 |
| 488 | 35 | 602.3182 | 603.3254 | 19611.56 | 1 | 602.3210 | C5 | 5 | -2.80e-03 | -4.65 |
| 488 | 36 | 5503.3228 | 1101.6718 | 10355.54 | 5 | 5503.3559 | C45 | 45 | -0.0331 | -6.01 |
| 488 | 37 | 6583.8545 | 1317.7782 | 8566.19 | 5 |  |  |  |  |  |
| 488 | 38 | 6696.9312 | 1340.3935 | 7916.10 | 5 | 6695.9675 | Z\_DOT56 | 4 | -0.0386 | -5.77 |
| 488 | 39 | 2916.3213 | 973.1144 | 10743.19 | 3 | 2916.3363 | C23 | 23 | -0.0150 | -5.15 |
| 488 | 40 | 6567.8247 | 1314.5722 | 8207.26 | 5 | 6567.8725 | Z\_DOT55 | 5 | -0.0479 | -7.29 |
| 488 | 41 | 3317.5222 | 1106.8480 | 6563.22 | 3 | 3317.5460 | C26 | 26 | -0.0238 | -7.17 |
| 488 | 42 | 739.3763 | 740.3835 | 13353.59 | 1 | 739.3799 | C6 | 6 | -3.61e-03 | -4.88 |
| 488 | 43 | 997.4604 | 998.4677 | 7294.05 | 1 | 997.4651 | C8 | 8 | -4.68e-03 | -4.69 |
| 488 | 44 | 2026.8354 | 1014.4250 | 6990.09 | 2 | 2026.8407 | C16 | 16 | -5.30e-03 | -2.61 |
| 488 | 45 | 5213.3574 | 1304.3466 | 6202.93 | 4 |  |  |  |  |  |
| 488 | 46 | 3634.5613 | 1212.5277 | 6773.76 | 3 |  |  |  |  |  |
| 488 | 47 | 6242.7153 | 1249.5503 | 8143.86 | 5 |  |  |  |  |  |
| 488 | 48 | 6430.7792 | 1287.1631 | 6613.33 | 5 | 6430.8136 | Z\_DOT54 | 6 | -0.0344 | -5.35 |
| 488 | 49 | 4270.8531 | 1424.6250 | 9770.80 | 3 |  |  |  |  |  |
| 488 | 50 | 6793.9557 | 1133.3332 | 8610.67 | 6 | 6793.0202 | Z\_DOT57 | 3 | -0.0669 | -9.85 |
| 488 | 50 | 6793.9557 | 1133.3332 | 8610.67 | 6 | 6794.0062 | C57 | 57 | -0.0505 | -7.44 |
| 488 | 51 | 6012.6708 | 1203.5414 | 4404.89 | 5 | 6012.6978 | Z\_DOT51 | 9 | -0.0270 | -4.49 |
| 488 | 52 | 2726.2496 | 909.7571 | 4713.90 | 3 | 2726.2602 | Z\_DOT24 | 36 | -0.0106 | -3.89 |
| 488 | 53 | 7068.1181 | 1414.6309 | 4465.25 | 5 |  |  |  |  |  |
| 488 | 54 | 3868.6408 | 1290.5542 | 3791.28 | 3 |  |  |  |  |  |
| 488 | 55 | 5418.3915 | 1355.6052 | 4994.14 | 4 | 5417.4264 | Z\_DOT46 | 14 | -0.0372 | -6.86 |
| 488 | 56 | 2726.2460 | 1364.1303 | 4458.30 | 2 | 2726.2602 | Z\_DOT24 | 36 | -0.0141 | -5.18 |
| 488 | 57 | 3940.7620 | 986.1978 | 4417.35 | 4 | 3940.7834 | C31 | 31 | -0.0213 | -5.41 |
| 488 | 58 | 6977.0885 | 1396.4250 | 3742.93 | 5 |  |  |  |  |  |
| 488 | 59 | 7005.0700 | 1168.5189 | 5522.14 | 6 |  |  |  |  |  |
| 488 | 60 | 6479.8269 | 1296.9727 | 3158.62 | 5 |  |  |  |  |  |
| 488 | 61 | 7098.1164 | 1420.6306 | 2483.80 | 5 |  |  |  |  |  |
| 488 | 62 | 5797.5751 | 1160.5223 | 4718.09 | 5 | 5797.6072 | Z\_DOT49 | 11 | -0.0321 | -5.53 |
| 488 | 63 | 3564.5616 | 1189.1945 | 16321.82 | 3 | 3564.5582 | Z\_DOT32 | 28 | 3.31e-03 | 0.93 |
| 488 | 64 | 3284.5116 | 1095.8445 | 3270.12 | 3 |  |  |  |  |  |
| 488 | 65 | 6521.8347 | 1305.3742 | 5983.18 | 5 | 6522.8530 | C54 | 54 | -0.0160 | -2.45 |
| 488 | 66 | 4170.8096 | 1043.7097 | 3210.25 | 4 | 4170.8372 | C33 | 33 | -0.0276 | -6.62 |
| 488 | 67 | 3391.4684 | 1131.4967 | 4706.40 | 3 |  |  |  |  |  |
| 488 | 68 | 3471.5971 | 1158.2063 | 3909.68 | 3 |  |  |  |  |  |
| 488 | 69 | 5505.3217 | 1377.3377 | 3318.54 | 4 |  |  |  |  |  |
| 488 | 70 | 474.2238 | 475.2310 | 4938.70 | 1 | 474.2260 | C4 | 4 | -2.23e-03 | -4.71 |
| 488 | 71 | 1793.8764 | 897.9455 | 3018.70 | 2 |  |  |  |  |  |
| 488 | 72 | 2743.1997 | 915.4072 | 4012.80 | 3 |  |  |  |  |  |
| 488 | 73 | 3552.5627 | 1185.1949 | 8185.57 | 3 |  |  |  |  |  |
| 488 | 74 | 7124.1323 | 1018.7405 | 5574.24 | 7 |  |  |  |  |  |
| 488 | 75 | 2271.0534 | 1136.5340 | 3688.65 | 2 | 2271.0586 | Z\_DOT20 | 40 | -5.13e-03 | -2.26 |
| 488 | 76 | 4300.8706 | 1076.2249 | 2648.62 | 4 |  |  |  |  |  |
| 488 | 77 | 7080.1195 | 1012.4529 | 3261.91 | 7 |  |  |  |  |  |
| 488 | 78 | 7023.1236 | 1405.6320 | 2662.79 | 5 |  |  |  |  |  |
| 488 | 79 | 7110.1271 | 1016.7397 | 4070.57 | 7 |  |  |  |  |  |
| 488 | 80 | 360.1454 | 361.1527 | 3608.57 | 1 |  |  |  |  |  |
| 488 | 81 | 6623.8912 | 1325.7855 | 2537.63 | 5 | 6623.9007 | C55 | 55 | -9.44e-03 | -1.43 |
| 488 | 82 | 1666.8258 | 834.4202 | 3915.10 | 2 | 1666.8376 | Z\_DOT15 | 45 | -0.0117 | -7.04 |
| 488 | 83 | 6919.0782 | 1384.8229 | 2370.86 | 5 |  |  |  |  |  |
| 488 | 84 | 6608.9340 | 1322.7941 | 1941.07 | 5 |  |  |  |  |  |
| 488 | 85 | 6028.6642 | 1206.7401 | 4848.34 | 5 |  |  |  |  |  |
| 488 | 86 | 7080.1457 | 1417.0364 | 3082.61 | 5 |  |  |  |  |  |
| 488 | 87 | 4642.0198 | 1161.5122 | 3445.19 | 4 |  |  |  |  |  |
| 488 | 88 | 2502.1386 | 1252.0766 | 1428.61 | 2 |  |  |  |  |  |
| 488 | 89 | 1088.5605 | 1089.5678 | 1701.77 | 1 | 1088.5628 | Z\_DOT10 | 50 | -2.31e-03 | -2.12 |
| 488 | 90 | 5910.6461 | 1183.1365 | 2275.64 | 5 | 5911.6501 | Z\_DOT50 | 10 | -1.68e-03 | -0.28 |
| 488 | 91 | 663.3557 | 664.3630 | 2080.71 | 1 |  |  |  |  |  |
| 488 | 92 | 6924.0913 | 1155.0225 | 1704.32 | 6 |  |  |  |  |  |
| 488 | 93 | 5284.2730 | 1057.8619 | 1363.65 | 5 |  |  |  |  |  |
| 488 | 94 | 2999.3234 | 1000.7817 | 2638.98 | 3 |  |  |  |  |  |
| 488 | 95 | 1258.5345 | 1259.5418 | 1343.18 | 1 | 1258.5434 | C10 | 10 | -8.86e-03 | -7.04 |
| 488 | 96 | 4899.1270 | 1225.7890 | 3628.50 | 4 | 4899.1349 | C40 | 40 | -7.91e-03 | -1.61 |
| 488 | 97 | 3692.6534 | 1231.8917 | 1664.45 | 3 | 3692.6673 | C29 | 29 | -0.0139 | -3.77 |
| 488 | 98 | 5053.3356 | 1264.3412 | 3701.37 | 4 |  |  |  |  |  |
| 488 | 99 | 1226.6222 | 1227.6295 | 2256.47 | 1 |  |  |  |  |  |
| 488 | 100 | 2471.0556 | 1236.5351 | 2891.28 | 2 | 2471.0674 | C19 | 19 | -0.0118 | -4.78 |
| 488 | 101 | 2307.0251 | 1154.5198 | 1485.58 | 2 |  |  |  |  |  |
| 488 | 102 | 3115.3635 | 1039.4618 | 2100.36 | 3 |  |  |  |  |  |
| 488 | 103 | 1283.6410 | 1284.6483 | 1832.32 | 1 |  |  |  |  |  |
| 488 | 104 | 4055.7811 | 1352.9343 | 2201.90 | 3 | 4055.8103 | C32 | 32 | -0.0292 | -7.20 |
| 488 | 105 | 4055.7879 | 1014.9543 | 1769.39 | 4 | 4055.8103 | C32 | 32 | -0.0224 | -5.52 |
| 488 | 106 | 3605.6285 | 1202.8834 | 3457.36 | 3 | 3605.6352 | C28 | 28 | -6.73e-03 | -1.87 |
| 488 | 107 | 1381.6145 | 1382.6218 | 911.12 | 1 |  |  |  |  |  |
| 488 | 108 | 960.4986 | 961.5059 | 1435.79 | 1 | 960.5043 | Z\_DOT9 | 51 | -5.62e-03 | -5.85 |
| 488 | 109 | 2814.2587 | 939.0935 | 1584.02 | 3 |  |  |  |  |  |
| 488 | 110 | 1076.4615 | 1077.4688 | 1264.04 | 1 |  |  |  |  |  |
| 488 | 111 | 1481.6368 | 1482.6441 | 1008.13 | 1 |  |  |  |  |  |
| 488 | 112 | 1332.0976 | 1333.1049 | 837.07 | 1 |  |  |  |  |  |
| 488 | 113 | 1436.6533 | 1437.6606 | 842.16 | 1 |  |  |  |  |  |

  

All proteins /
CsTx-1a\_S1 Cupiennius salei toxin 1 isoform a S1^ACsTx-1a\_S2 Cupiennius salei toxin 1 isoform a S2 /
Proteoform #8
