# Supplementary material for: The Dual Prey-Inactivation Strategy of Spiders—In-Depth Venomic Analysis of Cupiennius salei
Source: Toxins (Basel). 2019 Mar 19;11(3):167. doi: 10.3390/toxins11030167 (PMC6468893; doi:10.3390/toxins11030167)
Supplement: Supplementary file 1 [file toxins-11-00167-s001.zip › Supplementary Dataset EV1/20180328_f2_topdown_OTMS2_EThcD_NL_i02_ms2_proteoform_cutoff_html/prsms/prsm128.html]

Protein-Spectrum-Match for Spectrum #365


All proteins /
CsTx-1b Cupiennius salei toxin 1 isoform b /
Proteoform #8

## Protein-Spectrum-Match #128 for Spectrum #365

|  |  |  |  |  |  |
| --- | --- | --- | --- | --- | --- |
| PrSM ID: | 128 | Scan(s): | 489 | Precursor charge: | 6 |
| Precursor m/z: | 1195.8713 | Precursor mass: | 7169.1841 | Proteoform mass: | 7169.1856 |
| # matched peaks: | 35 | # matched fragment ions: | 35 | # unexpected modifications: | 0 |
| E-value: | 1.73e-30 | P-value: | 1.73e-30 | Q-value (Spectral FDR): | 0 |

  

|  |  |  |  |  |  |  |  |  |  |  |  |  |  |  |  |  |  |  |  |  |  |  |  |  |  |  |  |  |  |  |  |  |  |  |  |  |  |  |  |  |  |  |  |  |  |  |  |  |  |  |  |  |  |  |  |  |  |  |  |  |  |  |  |  |  |  |  |  |  |
| --- | --- | --- | --- | --- | --- | --- | --- | --- | --- | --- | --- | --- | --- | --- | --- | --- | --- | --- | --- | --- | --- | --- | --- | --- | --- | --- | --- | --- | --- | --- | --- | --- | --- | --- | --- | --- | --- | --- | --- | --- | --- | --- | --- | --- | --- | --- | --- | --- | --- | --- | --- | --- | --- | --- | --- | --- | --- | --- | --- | --- | --- | --- | --- | --- | --- | --- | --- | --- | --- |
|  | |  | | | | | | | | | | | | | | | | | | | | | | | | | | | | | | | | | | | | | | | | | | | | | | | | | | | | | | | | | | | | | | | | | | | |
| 1 |  |  | M |  | K |  | V |  | L |  | I |  | I |  | S |  | A |  | V |  | L |  |  | F |  | I |  | T |  | I |  | F |  | S |  | N |  | I |  | S |  | A |  |  | E |  | I |  | E |  | D |  | D |  | F |  | L |  | E |  | D |  | E |  | 30 |  |
|  | |  | | | | | | | | | | | | | | | | | | | | | | | | | | | | | | | | | | | | | | | | | | | | | | | | | | | | | | | | | | | | | | | | | | | |
| 31 |  |  | S |  | F |  | E |  | A |  | E |  | D |  | I |  | I |  | P |  | F |  |  | L |  | E |  | N |  | E |  | Q |  | A |  | R | ] | S |  | C |  | I |  | ⎩ | P | ⎫ | K | ⎫ | H | ⎫ | E | ⎫ | E | ⎫ | C |  | T | ⎫ | N | ⎫ | D |  | K |  | 60 |  |
|  | |  | | | | | | | | | | | | | | | | | | | | | | | | | | | | | | | | | | | | | | | | | | | | | | | | | | | | | | | | | | | | | | | | | | | |
| 61 |  | ⎫ | H | ⎫ | N | ⎫ | C | ⎫ | C |  | R |  | K | ⎫ | G |  | L |  | F | ⎫ | K |  | ⎫ | L | ⎫ | K | ⎫ | C | ⎫ | Q | ⎫ | C |  | S | ⎫ | T | ⎫ | F | ⎫ | D |  | D |  |  | E |  | S |  | G | ⎱ | Q |  | P |  | T |  | E | ⎩ | R |  | C |  | A |  | 90 |  |
|  | |  | | | | | | | | | | | | | | | | | | | | | | | | | | | | | | | | | | | | | | | | | | | | | | | | | | | | | | | | | | | | | | | | | | | |
| 91 |  | ⎫ | C |  | G | ⎫ | R |  | P |  | M | ⎫ | G | ⎱ | H |  | Q | ⎱ | A |  | I |  |  | E |  | T |  | G |  | L | ⎫ | N | ⎫ | I | ⎫ | F | [ | R |  | G |  | L |  |  | F |  | K |  | G |  | K |  | K |  | K |  | N |  | K |  | K |  | T |  | 120 |  |
|  | |  | | | | | | | | | | | | | | | | | | | | | | | | | | | | | | | | | | | | | | | | | | | | | | | | | | | | | | | | | | | | | | | | | | | |
| 121 |  |  | K |  | G |  | | | | 122 |  | | | | | | | | | | | | | | | | | | | | | | | | | | | | | | | | | | | | | | | | | | | | | | | | | | | | | | | |

Fixed PTMs: Carbamidomethylation [C49 C56 C63 C64 C73 C75 C89 C91 ]

  

All peaks (69)  Matched peaks (35)  Not matched peaks (34)

  

| Scan | Peak | Mono mass | Mono m/z | Intensity | Charge | Theoretical mass | Ion | Pos | Mass error | PPM error |
| --- | --- | --- | --- | --- | --- | --- | --- | --- | --- | --- |
| 489 | 1 | 3585.0672 | 1196.0297 | 381141.40 | 3 |  |  |  |  |  |
| 489 | 2 | 7112.1148 | 1423.4302 | 117029.24 | 5 |  |  |  |  |  |
| 489 | 3 | 2390.3827 | 1196.1986 | 197415.50 | 2 |  |  |  |  |  |
| 489 | 4 | 7125.1300 | 1426.0333 | 24522.02 | 5 |  |  |  |  |  |
| 489 | 5 | 6976.0828 | 1396.2238 | 14266.90 | 5 |  |  |  |  |  |
| 489 | 6 | 7080.1392 | 1417.0351 | 10036.49 | 5 |  |  |  |  |  |
| 489 | 7 | 7021.1011 | 1405.2275 | 10492.04 | 5 | 7021.1332 | C59 | 59 | -0.0321 | -4.57 |
| 489 | 8 | 1752.7552 | 877.3849 | 20680.69 | 2 | 1752.7671 | C14 | 14 | -0.0120 | -6.83 |
| 489 | 9 | 7153.1232 | 1431.6319 | 11183.09 | 5 |  |  |  |  |  |
| 489 | 10 | 1866.7976 | 934.4061 | 16207.47 | 2 | 1866.8101 | C15 | 15 | -0.0125 | -6.69 |
| 489 | 11 | 1434.2296 | 1435.2369 | 41118.54 | 1 |  |  |  |  |  |
| 489 | 12 | 7096.1011 | 1420.2275 | 6693.73 | 5 |  |  |  |  |  |
| 489 | 13 | 602.3175 | 603.3248 | 16317.40 | 1 | 602.3210 | C5 | 5 | -3.47e-03 | -5.76 |
| 489 | 14 | 7035.1294 | 1408.0332 | 7747.50 | 5 |  |  |  |  |  |
| 489 | 15 | 868.4170 | 869.4243 | 8361.73 | 1 | 868.4225 | C7 | 7 | -5.48e-03 | -6.32 |
| 489 | 16 | 739.3754 | 740.3826 | 10881.84 | 1 | 739.3799 | C6 | 6 | -4.52e-03 | -6.11 |
| 489 | 17 | 7063.1195 | 1413.6312 | 4305.36 | 5 |  |  |  |  |  |
| 489 | 18 | 2726.2394 | 1364.1270 | 3719.01 | 2 | 2726.2602 | Z\_DOT24 | 36 | -0.0207 | -7.60 |
| 489 | 19 | 3157.4943 | 1053.5054 | 6174.97 | 3 | 3157.5153 | C25 | 25 | -0.0211 | -6.68 |
| 489 | 20 | 3445.5800 | 1149.5340 | 4045.76 | 3 | 3445.6046 | C27 | 27 | -0.0245 | -7.12 |
| 489 | 21 | 997.4588 | 998.4661 | 5283.28 | 1 | 997.4651 | C8 | 8 | -6.21e-03 | -6.22 |
| 489 | 22 | 2916.3154 | 973.1124 | 4462.71 | 3 | 2916.3363 | C23 | 23 | -0.0209 | -7.16 |
| 489 | 23 | 6907.0349 | 1382.4143 | 3781.72 | 5 | 6908.0491 | C58 | 58 | -0.0118 | -1.71 |
| 489 | 24 | 6209.6420 | 1553.4178 | 2824.97 | 4 | 6209.6892 | C51 | 51 | -0.0472 | -7.61 |
| 489 | 25 | 6792.9572 | 1359.5987 | 3646.25 | 5 | 6793.0202 | Z\_DOT57 | 3 | -0.0630 | -9.28 |
| 489 | 25 | 6792.9572 | 1359.5987 | 3646.25 | 5 | 6794.0062 | C57 | 57 | -0.0466 | -6.87 |
| 489 | 26 | 4443.9066 | 1482.3095 | 3811.85 | 3 | 4443.9333 | C36 | 36 | -0.0267 | -6.01 |
| 489 | 27 | 3940.7558 | 1314.5925 | 2266.72 | 3 | 3940.7834 | C31 | 31 | -0.0276 | -7.00 |
| 489 | 28 | 7152.1201 | 1193.0273 | 3858.42 | 6 |  |  |  |  |  |
| 489 | 29 | 5944.5350 | 1487.1410 | 3908.95 | 4 | 5944.5717 | C49 | 49 | -0.0367 | -6.18 |
| 489 | 30 | 5503.3194 | 1376.8371 | 3812.31 | 4 | 5503.3559 | C45 | 45 | -0.0365 | -6.63 |
| 489 | 31 | 5286.2663 | 1322.5738 | 2239.13 | 4 | 5286.3038 | C43 | 43 | -0.0375 | -7.10 |
| 489 | 32 | 7112.1135 | 1779.0356 | 2242.48 | 4 |  |  |  |  |  |
| 489 | 33 | 3317.5200 | 1106.8473 | 4462.27 | 3 | 3317.5460 | C26 | 26 | -0.0260 | -7.83 |
| 489 | 34 | 3692.6394 | 1231.8871 | 3609.46 | 3 | 3692.6673 | C29 | 29 | -0.0279 | -7.54 |
| 489 | 35 | 2026.8282 | 1014.4214 | 3108.38 | 2 | 2026.8407 | C16 | 16 | -0.0125 | -6.17 |
| 489 | 36 | 5887.5087 | 1472.8845 | 1932.14 | 4 | 5887.5503 | C48 | 48 | -0.0416 | -7.06 |
| 489 | 37 | 2271.0422 | 1136.5284 | 1827.37 | 2 | 2271.0586 | Z\_DOT20 | 40 | -0.0164 | -7.21 |
| 489 | 38 | 3793.6899 | 1265.5706 | 2410.96 | 3 | 3793.7149 | C30 | 30 | -0.0251 | -6.61 |
| 489 | 39 | 7077.1080 | 1180.5253 | 1794.66 | 6 |  |  |  |  |  |
| 489 | 40 | 2872.3040 | 958.4419 | 1997.78 | 3 |  |  |  |  |  |
| 489 | 41 | 2788.2244 | 930.4154 | 2486.01 | 3 | 2788.2414 | C22 | 22 | -0.0170 | -6.10 |
| 489 | 42 | 474.2234 | 475.2307 | 3270.21 | 1 | 474.2260 | C4 | 4 | -2.57e-03 | -5.41 |
| 489 | 43 | 4056.7820 | 1353.2679 | 1332.43 | 3 |  |  |  |  |  |
| 489 | 44 | 3585.0687 | 1793.5416 | 2366.31 | 2 |  |  |  |  |  |
| 489 | 45 | 3029.4015 | 1010.8078 | 1174.59 | 3 | 3029.4204 | C24 | 24 | -0.0188 | -6.22 |
| 489 | 46 | 7126.1290 | 1188.6954 | 2155.26 | 6 |  |  |  |  |  |
| 489 | 47 | 2788.2285 | 1395.1215 | 1714.00 | 2 | 2788.2414 | C22 | 22 | -0.0129 | -4.63 |
| 489 | 48 | 1615.6982 | 808.8564 | 1971.99 | 2 | 1615.7082 | C13 | 13 | -0.0101 | -6.23 |
| 489 | 49 | 2187.8566 | 1094.9356 | 850.47 | 2 |  |  |  |  |  |
| 489 | 50 | 1225.6144 | 1226.6216 | 2473.97 | 1 | 1225.6217 | Z\_DOT11 | 49 | -7.38e-03 | -6.02 |
| 489 | 51 | 1372.5776 | 1373.5849 | 1147.77 | 1 | 1372.5863 | C11 | 11 | -8.69e-03 | -6.33 |
| 489 | 52 | 2899.2893 | 1450.6519 | 1175.40 | 2 |  |  |  |  |  |
| 489 | 53 | 663.3554 | 664.3627 | 1428.38 | 1 |  |  |  |  |  |
| 489 | 54 | 360.1450 | 361.1523 | 1990.25 | 1 |  |  |  |  |  |
| 489 | 55 | 6922.0690 | 1385.4211 | 2486.90 | 5 |  |  |  |  |  |
| 489 | 56 | 1012.4720 | 1013.4793 | 851.63 | 1 |  |  |  |  |  |
| 489 | 57 | 1258.5366 | 1259.5439 | 1238.00 | 1 | 1258.5434 | C10 | 10 | -6.78e-03 | -5.39 |
| 489 | 58 | 3041.7936 | 1521.9041 | 1667.56 | 2 |  |  |  |  |  |
| 489 | 59 | 3183.5113 | 1062.1777 | 1516.15 | 3 |  |  |  |  |  |
| 489 | 60 | 1352.2624 | 1353.2697 | 802.46 | 1 |  |  |  |  |  |
| 489 | 61 | 3606.6066 | 1203.2095 | 696.68 | 3 |  |  |  |  |  |
| 489 | 62 | 1385.2181 | 1386.2254 | 518.86 | 1 |  |  |  |  |  |
| 489 | 63 | 1283.6409 | 1284.6481 | 807.17 | 1 |  |  |  |  |  |
| 489 | 64 | 911.3924 | 912.3997 | 480.96 | 1 |  |  |  |  |  |
| 489 | 65 | 2471.0517 | 1236.5331 | 697.83 | 2 | 2471.0674 | C19 | 19 | -0.0157 | -6.37 |
| 489 | 66 | 4254.8494 | 1419.2904 | 1392.76 | 3 |  |  |  |  |  |
| 489 | 67 | 853.8785 | 854.8858 | 508.41 | 1 |  |  |  |  |  |
| 489 | 68 | 960.4985 | 961.5058 | 398.10 | 1 | 960.5043 | Z\_DOT9 | 51 | -5.74e-03 | -5.97 |
| 489 | 69 | 1328.0734 | 1329.0806 | 612.25 | 1 |  |  |  |  |  |

  

All proteins /
CsTx-1b Cupiennius salei toxin 1 isoform b /
Proteoform #8
