# Supplementary material for: The Dual Prey-Inactivation Strategy of Spiders—In-Depth Venomic Analysis of Cupiennius salei
Source: Toxins (Basel). 2019 Mar 19;11(3):167. doi: 10.3390/toxins11030167 (PMC6468893; doi:10.3390/toxins11030167)
Supplement: Supplementary file 1 [file toxins-11-00167-s001.zip › Supplementary Dataset EV1/20180328_f2_topdown_OTMS2_EThcD_NL_i02_ms2_proteoform_cutoff_html/prsms/prsm129.html]

Protein-Spectrum-Match for Spectrum #366


All proteins /
CsTx-1b Cupiennius salei toxin 1 isoform b /
Proteoform #8

## Protein-Spectrum-Match #129 for Spectrum #366

|  |  |  |  |  |  |
| --- | --- | --- | --- | --- | --- |
| PrSM ID: | 129 | Scan(s): | 491 | Precursor charge: | 8 |
| Precursor m/z: | 897.1552 | Precursor mass: | 7169.1835 | Proteoform mass: | 7169.1856 |
| # matched peaks: | 48 | # matched fragment ions: | 40 | # unexpected modifications: | 0 |
| E-value: | 1.99e-36 | P-value: | 1.99e-36 | Q-value (Spectral FDR): | 0 |

  

|  |  |  |  |  |  |  |  |  |  |  |  |  |  |  |  |  |  |  |  |  |  |  |  |  |  |  |  |  |  |  |  |  |  |  |  |  |  |  |  |  |  |  |  |  |  |  |  |  |  |  |  |  |  |  |  |  |  |  |  |  |  |  |  |  |  |  |  |  |  |
| --- | --- | --- | --- | --- | --- | --- | --- | --- | --- | --- | --- | --- | --- | --- | --- | --- | --- | --- | --- | --- | --- | --- | --- | --- | --- | --- | --- | --- | --- | --- | --- | --- | --- | --- | --- | --- | --- | --- | --- | --- | --- | --- | --- | --- | --- | --- | --- | --- | --- | --- | --- | --- | --- | --- | --- | --- | --- | --- | --- | --- | --- | --- | --- | --- | --- | --- | --- | --- | --- |
|  | |  | | | | | | | | | | | | | | | | | | | | | | | | | | | | | | | | | | | | | | | | | | | | | | | | | | | | | | | | | | | | | | | | | | | |
| 1 |  |  | M |  | K |  | V |  | L |  | I |  | I |  | S |  | A |  | V |  | L |  |  | F |  | I |  | T |  | I |  | F |  | S |  | N |  | I |  | S |  | A |  |  | E |  | I |  | E |  | D |  | D |  | F |  | L |  | E |  | D |  | E |  | 30 |  |
|  | |  | | | | | | | | | | | | | | | | | | | | | | | | | | | | | | | | | | | | | | | | | | | | | | | | | | | | | | | | | | | | | | | | | | | |
| 31 |  |  | S |  | F |  | E |  | A |  | E |  | D |  | I |  | I |  | P |  | F |  |  | L |  | E |  | N |  | E |  | Q |  | A |  | R | ] | S | ⎩ | C |  | I |  |  | P | ⎫ | K | ⎫ | H | ⎫ | E | ⎱ | E | ⎫ | C |  | T | ⎫ | N | ⎱ | D | ⎫ | K |  | 60 |  |
|  | |  | | | | | | | | | | | | | | | | | | | | | | | | | | | | | | | | | | | | | | | | | | | | | | | | | | | | | | | | | | | | | | | | | | | |
| 61 |  |  | H | ⎱ | N | ⎫ | C |  | C |  | R |  | K | ⎫ | G | ⎫ | L | ⎱ | F | ⎱ | K |  | ⎫ | L |  | K | ⎫ | C | ⎫ | Q | ⎫ | C |  | S | ⎫ | T | ⎫ | F | ⎩ | D | ⎩ | D |  | ⎫ | E | ⎫ | S |  | G | ⎱ | Q |  | P |  | T |  | E | ⎫ | R |  | C |  | A |  | 90 |  |
|  | |  | | | | | | | | | | | | | | | | | | | | | | | | | | | | | | | | | | | | | | | | | | | | | | | | | | | | | | | | | | | | | | | | | | | |
| 91 |  |  | C |  | G | ⎫ | R |  | P | ⎫ | M | ⎫ | G | ⎫ | H | ⎫ | Q | ⎫ | A |  | I |  |  | E |  | T |  | G |  | L |  | N |  | I | ⎫ | F | [ | R |  | G |  | L |  |  | F |  | K |  | G |  | K |  | K |  | K |  | N |  | K |  | K |  | T |  | 120 |  |
|  | |  | | | | | | | | | | | | | | | | | | | | | | | | | | | | | | | | | | | | | | | | | | | | | | | | | | | | | | | | | | | | | | | | | | | |
| 121 |  |  | K |  | G |  | | | | 122 |  | | | | | | | | | | | | | | | | | | | | | | | | | | | | | | | | | | | | | | | | | | | | | | | | | | | | | | | |

Fixed PTMs: Carbamidomethylation [C49 C56 C63 C64 C73 C75 C89 C91 ]

  

All peaks (115)  Matched peaks (48)  Not matched peaks (67)

  

| Scan | Peak | Mono mass | Mono m/z | Intensity | Charge | Theoretical mass | Ion | Pos | Mass error | PPM error |
| --- | --- | --- | --- | --- | --- | --- | --- | --- | --- | --- |
| 491 | 1 | 7112.1185 | 1017.0242 | 228564.17 | 7 |  |  |  |  |  |
| 491 | 2 | 7112.1185 | 1186.3604 | 102186.48 | 6 |  |  |  |  |  |
| 491 | 3 | 3584.5707 | 897.1499 | 167508.32 | 4 |  |  |  |  |  |
| 491 | 4 | 7153.1261 | 1022.8824 | 39800.11 | 7 |  |  |  |  |  |
| 491 | 5 | 7125.1260 | 1018.8824 | 36312.35 | 7 |  |  |  |  |  |
| 491 | 6 | 7055.1006 | 1176.8574 | 27034.22 | 6 |  |  |  |  |  |
| 491 | 7 | 7154.1328 | 1193.3627 | 31201.13 | 6 |  |  |  |  |  |
| 491 | 8 | 7126.1360 | 1188.6966 | 23807.26 | 6 |  |  |  |  |  |
| 491 | 9 | 7004.0574 | 1001.5869 | 24668.96 | 7 |  |  |  |  |  |
| 491 | 10 | 3585.5759 | 1196.1992 | 43303.84 | 3 |  |  |  |  |  |
| 491 | 11 | 6890.9773 | 985.4326 | 23582.35 | 7 |  |  |  |  |  |
| 491 | 12 | 6188.7070 | 1032.4584 | 18123.02 | 6 |  |  |  |  |  |
| 491 | 13 | 3157.4961 | 790.3813 | 16734.15 | 4 | 3157.5153 | C25 | 25 | -0.0192 | -6.08 |
| 491 | 14 | 2788.2249 | 930.4156 | 17355.14 | 3 | 2788.2414 | C22 | 22 | -0.0165 | -5.90 |
| 491 | 15 | 1024.4507 | 1025.4579 | 40957.48 | 1 |  |  |  |  |  |
| 491 | 16 | 7004.0566 | 876.5143 | 17560.03 | 8 |  |  |  |  |  |
| 491 | 17 | 7095.1132 | 1183.5261 | 14586.23 | 6 |  |  |  |  |  |
| 491 | 18 | 5213.3508 | 1043.6774 | 13350.35 | 5 |  |  |  |  |  |
| 491 | 19 | 1752.7576 | 877.3861 | 20997.05 | 2 | 1752.7671 | C14 | 14 | -9.53e-03 | -5.43 |
| 491 | 20 | 3445.5824 | 862.4029 | 13157.20 | 4 | 3445.6046 | C27 | 27 | -0.0222 | -6.44 |
| 491 | 21 | 7021.0998 | 1004.0215 | 13770.39 | 7 | 7021.1332 | C59 | 59 | -0.0334 | -4.76 |
| 491 | 22 | 7080.1328 | 1012.4548 | 13748.45 | 7 |  |  |  |  |  |
| 491 | 23 | 4443.9001 | 1111.9823 | 11758.31 | 4 | 4443.9333 | C36 | 36 | -0.0332 | -7.48 |
| 491 | 24 | 6209.6433 | 1035.9478 | 10995.52 | 6 | 6209.6892 | C51 | 51 | -0.0459 | -7.40 |
| 491 | 25 | 6583.8451 | 1098.3148 | 13414.07 | 6 |  |  |  |  |  |
| 491 | 26 | 7065.1108 | 1010.3088 | 10388.85 | 7 | 7066.1350 | Z\_DOT59 | 1 | -0.0218 | -3.08 |
| 491 | 27 | 7113.1280 | 1423.6329 | 10180.58 | 5 |  |  |  |  |  |
| 491 | 28 | 3941.7550 | 986.4460 | 13425.15 | 4 |  |  |  |  |  |
| 491 | 29 | 5887.5161 | 982.2600 | 12142.82 | 6 | 5887.5503 | C48 | 48 | -0.0341 | -5.80 |
| 491 | 30 | 1866.7984 | 934.4065 | 15693.53 | 2 | 1866.8101 | C15 | 15 | -0.0116 | -6.23 |
| 491 | 31 | 7068.1095 | 1179.0255 | 14668.78 | 6 |  |  |  |  |  |
| 491 | 32 | 2641.1564 | 881.3928 | 11546.96 | 3 | 2641.1730 | C21 | 21 | -0.0165 | -6.26 |
| 491 | 33 | 5944.5341 | 991.7630 | 9877.57 | 6 | 5944.5717 | C49 | 49 | -0.0376 | -6.33 |
| 491 | 34 | 2674.1319 | 892.3846 | 17993.56 | 3 |  |  |  |  |  |
| 491 | 35 | 3317.5274 | 830.3891 | 7439.53 | 4 | 3317.5460 | C26 | 26 | -0.0186 | -5.61 |
| 491 | 36 | 3157.4972 | 1053.5063 | 7539.36 | 3 | 3157.5153 | C25 | 25 | -0.0182 | -5.75 |
| 491 | 37 | 5757.4626 | 960.5844 | 6042.26 | 6 | 5756.5098 | C47 | 47 | -0.0495 | -8.61 |
| 491 | 38 | 6209.6501 | 1242.9373 | 5412.32 | 5 | 6209.6892 | C51 | 51 | -0.0391 | -6.30 |
| 491 | 39 | 3634.5635 | 1212.5285 | 7159.20 | 3 |  |  |  |  |  |
| 491 | 40 | 6302.7409 | 1261.5555 | 6196.68 | 5 | 6301.7710 | Z\_DOT53 | 7 | -0.0325 | -5.15 |
| 491 | 41 | 3922.6536 | 1308.5585 | 7368.44 | 3 |  |  |  |  |  |
| 491 | 42 | 2471.0529 | 824.6916 | 9582.27 | 3 | 2471.0674 | C19 | 19 | -0.0145 | -5.88 |
| 491 | 43 | 7022.1061 | 1171.3583 | 8761.59 | 6 | 7021.1332 | C59 | 59 | -0.0294 | -4.19 |
| 491 | 44 | 7151.1279 | 894.8983 | 8304.15 | 8 |  |  |  |  |  |
| 491 | 45 | 5417.3925 | 1084.4858 | 7173.29 | 5 | 5417.4264 | Z\_DOT46 | 14 | -0.0338 | -6.24 |
| 491 | 46 | 896.2693 | 897.2766 | 29540.55 | 1 |  |  |  |  |  |
| 491 | 47 | 4170.8101 | 1043.7098 | 6366.43 | 4 | 4170.8372 | C33 | 33 | -0.0271 | -6.51 |
| 491 | 48 | 6977.0911 | 1163.8558 | 6598.60 | 6 |  |  |  |  |  |
| 491 | 49 | 2871.2999 | 958.1072 | 6735.97 | 3 |  |  |  |  |  |
| 491 | 50 | 5503.3251 | 1101.6723 | 6265.01 | 5 | 5503.3559 | C45 | 45 | -0.0308 | -5.59 |
| 491 | 51 | 4554.9381 | 911.9949 | 6277.80 | 5 |  |  |  |  |  |
| 491 | 52 | 5053.3233 | 1011.6719 | 9456.21 | 5 |  |  |  |  |  |
| 491 | 53 | 868.4183 | 869.4256 | 10961.03 | 1 | 868.4225 | C7 | 7 | -4.14e-03 | -4.77 |
| 491 | 54 | 2528.0751 | 843.6990 | 6347.05 | 3 | 2528.0889 | C20 | 20 | -0.0138 | -5.47 |
| 491 | 55 | 3692.6410 | 924.1675 | 5037.43 | 4 | 3692.6673 | C29 | 29 | -0.0262 | -7.10 |
| 491 | 56 | 2916.3196 | 973.1138 | 7169.39 | 3 | 2916.3363 | C23 | 23 | -0.0167 | -5.72 |
| 491 | 57 | 5327.3919 | 1066.4857 | 7950.64 | 5 |  |  |  |  |  |
| 491 | 58 | 6948.0458 | 993.5853 | 4916.64 | 7 |  |  |  |  |  |
| 491 | 59 | 6028.6824 | 1005.7877 | 7375.73 | 6 |  |  |  |  |  |
| 491 | 60 | 7093.0945 | 1014.3065 | 8618.65 | 7 |  |  |  |  |  |
| 491 | 61 | 4381.9234 | 1096.4881 | 6285.47 | 4 | 4381.9521 | Z\_DOT38 | 22 | -0.0287 | -6.56 |
| 491 | 62 | 2028.3940 | 1015.2043 | 7859.67 | 2 |  |  |  |  |  |
| 491 | 63 | 5797.5729 | 967.2694 | 5598.89 | 6 | 5797.6072 | Z\_DOT49 | 11 | -0.0342 | -5.90 |
| 491 | 64 | 3793.6917 | 949.4302 | 5238.69 | 4 | 3793.7149 | C30 | 30 | -0.0232 | -6.12 |
| 491 | 65 | 7055.0885 | 1412.0250 | 5185.66 | 5 |  |  |  |  |  |
| 491 | 66 | 997.4613 | 998.4686 | 7518.56 | 1 | 997.4651 | C8 | 8 | -3.76e-03 | -3.77 |
| 491 | 67 | 6776.9312 | 969.1403 | 3805.85 | 7 |  |  |  |  |  |
| 491 | 68 | 5798.5674 | 1160.7208 | 7991.49 | 5 | 5797.6072 | Z\_DOT49 | 11 | -0.0421 | -7.27 |
| 491 | 69 | 4899.1037 | 1225.7832 | 4354.38 | 4 | 4899.1349 | C40 | 40 | -0.0313 | -6.38 |
| 491 | 70 | 6243.7148 | 1041.6264 | 4378.28 | 6 |  |  |  |  |  |
| 491 | 71 | 4299.8487 | 1075.9694 | 6792.66 | 4 | 4299.8798 | C34 | 34 | -0.0312 | -7.25 |
| 491 | 72 | 7006.0826 | 1168.6877 | 4646.18 | 6 |  |  |  |  |  |
| 491 | 73 | 6083.6876 | 1014.9552 | 19131.05 | 6 |  |  |  |  |  |
| 491 | 74 | 6583.8549 | 1317.7783 | 4285.21 | 5 |  |  |  |  |  |
| 491 | 75 | 3245.4080 | 1082.8099 | 3198.96 | 3 |  |  |  |  |  |
| 491 | 76 | 2726.2410 | 909.7543 | 5017.04 | 3 | 2726.2602 | Z\_DOT24 | 36 | -0.0192 | -7.05 |
| 491 | 77 | 5944.5317 | 1189.9136 | 5511.66 | 5 | 5944.5717 | C49 | 49 | -0.0400 | -6.73 |
| 491 | 78 | 7037.1245 | 1006.3108 | 6478.12 | 7 |  |  |  |  |  |
| 491 | 79 | 3868.6353 | 968.1661 | 3878.43 | 4 |  |  |  |  |  |
| 491 | 80 | 2614.2046 | 872.4088 | 7883.67 | 3 |  |  |  |  |  |
| 491 | 81 | 6947.0333 | 1158.8462 | 3825.46 | 6 |  |  |  |  |  |
| 491 | 82 | 7037.1123 | 1173.8593 | 4132.89 | 6 |  |  |  |  |  |
| 491 | 83 | 4528.9811 | 1133.2526 | 6743.75 | 4 | 4529.0205 | Z\_DOT39 | 21 | -0.0394 | -8.70 |
| 491 | 84 | 6081.5953 | 1217.3263 | 5706.92 | 5 | 6081.6306 | C50 | 50 | -0.0353 | -5.81 |
| 491 | 85 | 3229.3958 | 1077.4725 | 4266.56 | 3 | 3229.4101 | Z\_DOT29 | 31 | -0.0144 | -4.45 |
| 491 | 86 | 6300.7327 | 1051.1294 | 5005.73 | 6 | 6301.7710 | Z\_DOT53 | 7 | -0.0359 | -5.70 |
| 491 | 87 | 6919.0548 | 1154.1831 | 3465.25 | 6 |  |  |  |  |  |
| 491 | 88 | 3114.3656 | 1039.1291 | 4817.78 | 3 | 3114.3832 | Z\_DOT28 | 32 | -0.0176 | -5.65 |
| 491 | 89 | 5707.5740 | 952.2696 | 3195.93 | 6 |  |  |  |  |  |
| 491 | 90 | 4642.0514 | 1161.5201 | 5760.80 | 4 |  |  |  |  |  |
| 491 | 91 | 5419.4101 | 1355.8598 | 3646.98 | 4 |  |  |  |  |  |
| 491 | 92 | 5831.4829 | 1167.3038 | 3379.34 | 5 |  |  |  |  |  |
| 491 | 93 | 2886.3101 | 963.1106 | 3180.38 | 3 |  |  |  |  |  |
| 491 | 94 | 6525.8332 | 1306.1739 | 4406.56 | 5 |  |  |  |  |  |
| 491 | 95 | 6712.9549 | 1119.8331 | 4721.79 | 6 |  |  |  |  |  |
| 491 | 96 | 6188.7010 | 1238.7475 | 3943.61 | 5 |  |  |  |  |  |
| 491 | 97 | 5503.3188 | 918.2271 | 4650.95 | 6 | 5503.3559 | C45 | 45 | -0.0371 | -6.74 |
| 491 | 98 | 6976.0770 | 997.5897 | 6550.81 | 7 |  |  |  |  |  |
| 491 | 99 | 1372.5790 | 1373.5862 | 2732.14 | 1 | 1372.5863 | C11 | 11 | -7.35e-03 | -5.36 |
| 491 | 100 | 602.3180 | 603.3253 | 5556.13 | 1 | 602.3210 | C5 | 5 | -2.98e-03 | -4.95 |
| 491 | 101 | 1372.5784 | 687.2965 | 3291.31 | 2 | 1372.5863 | C11 | 11 | -7.91e-03 | -5.77 |
| 491 | 102 | 739.3759 | 740.3832 | 4161.90 | 1 | 739.3799 | C6 | 6 | -3.97e-03 | -5.37 |
| 491 | 103 | 1258.5370 | 1259.5442 | 2259.76 | 1 | 1258.5434 | C10 | 10 | -6.41e-03 | -5.10 |
| 491 | 104 | 502.2424 | 503.2496 | 1274.19 | 1 |  |  |  |  |  |
| 491 | 105 | 953.4473 | 954.4546 | 1196.21 | 1 |  |  |  |  |  |
| 491 | 106 | 1434.8334 | 1435.8406 | 1397.59 | 1 |  |  |  |  |  |
| 491 | 107 | 663.3556 | 664.3629 | 838.39 | 1 |  |  |  |  |  |
| 491 | 108 | 1166.5034 | 1167.5107 | 917.94 | 1 |  |  |  |  |  |
| 491 | 109 | 474.2240 | 475.2313 | 1198.51 | 1 | 474.2260 | C4 | 4 | -2.02e-03 | -4.26 |
| 491 | 110 | 771.6855 | 772.6927 | 1029.51 | 1 |  |  |  |  |  |
| 491 | 111 | 1314.1704 | 1315.1777 | 964.10 | 1 |  |  |  |  |  |
| 491 | 112 | 1487.6029 | 744.8087 | 656.99 | 2 | 1487.6133 | C12 | 12 | -0.0103 | -6.94 |
| 491 | 113 | 1080.1357 | 1081.1430 | 680.26 | 1 |  |  |  |  |  |
| 491 | 114 | 1142.5498 | 572.2822 | 1384.68 | 2 |  |  |  |  |  |
| 491 | 115 | 1352.6853 | 677.3499 | 927.62 | 2 |  |  |  |  |  |

  

All proteins /
CsTx-1b Cupiennius salei toxin 1 isoform b /
Proteoform #8
