# Supplementary material for: The Dual Prey-Inactivation Strategy of Spiders—In-Depth Venomic Analysis of Cupiennius salei
Source: Toxins (Basel). 2019 Mar 19;11(3):167. doi: 10.3390/toxins11030167 (PMC6468893; doi:10.3390/toxins11030167)
Supplement: Supplementary file 1 [file toxins-11-00167-s001.zip › Supplementary Dataset EV1/20180328_f2_topdown_OTMS2_EThcD_NL_i02_ms2_proteoform_cutoff_html/prsms/prsm13.html]

Protein-Spectrum-Match for Spectrum #225


All proteins /
CsTx-13a Cupiennius salei toxin 13 isoform a /
Proteoform #80

## Protein-Spectrum-Match #13 for Spectrum #225

|  |  |  |  |  |  |
| --- | --- | --- | --- | --- | --- |
| PrSM ID: | 13 | Scan(s): | 303 | Precursor charge: | 7 |
| Precursor m/z: | 614.5436 | Precursor mass: | 4294.7541 | Proteoform mass: | 4294.7391 |
| # matched peaks: | 14 | # matched fragment ions: | 11 | # unexpected modifications: | 1 |
| E-value: | 5.20e-09 | P-value: | 5.20e-09 | Q-value (Spectral FDR): | 0 |

  

|  |  |  |  |  |  |  |  |  |  |  |  |  |  |  |  |  |  |  |  |  |  |  |  |  |  |  |  |  |  |  |  |  |  |  |  |  |  |  |  |  |  |  |  |  |  |  |  |  |  |  |  |  |  |  |  |  |  |  |  |  |  |  |  |  |  |  |  |  |  |
| --- | --- | --- | --- | --- | --- | --- | --- | --- | --- | --- | --- | --- | --- | --- | --- | --- | --- | --- | --- | --- | --- | --- | --- | --- | --- | --- | --- | --- | --- | --- | --- | --- | --- | --- | --- | --- | --- | --- | --- | --- | --- | --- | --- | --- | --- | --- | --- | --- | --- | --- | --- | --- | --- | --- | --- | --- | --- | --- | --- | --- | --- | --- | --- | --- | --- | --- | --- | --- | --- |
|  | |  | | | | | | | | | | | | | | | | | | | | | | | | | | | | | | | | | | | | | | | | | | | | | | | | | | | | | | | | | | | | | | | | | | | |
| 1 |  |  | M |  | K |  | V |  | L |  | V |  | I |  | F |  | A |  | V |  | L |  |  | S |  | L |  | V |  | I |  | F |  | S |  | N |  | C |  | S |  | A |  |  | E |  | T |  | D |  | E |  | D |  | F |  | F |  | G |  | E |  | E |  | 30 |  |
|  | |  | | | | | | | | | | | | | | | | | | | | | | | | | | | | | | | | | | | | | | | | | | | | | | | | | | | | | | | | | | | | | | | | | | | |
| 31 |  |  | S |  | F |  | E |  | A |  | D |  | D |  | I |  | I |  | P |  | F |  |  | I |  | A |  | K |  | E |  | Q |  | V |  | R | ] | S |  | D |  | C |  |  | T |  | L |  | R |  | N |  | H | ⎫ | D | ⎫ | C |  | T |  | D | ⎫ | D |  | 60 |  |
|  | |  | | | | | | | | | | | | | | | | | -48.02 | | | | | | | | | | | | | | | | | | | | | | | | | | | | | | | | | | | | | | | | | | | | | | | |
| 61 |  |  | R |  | H | ⎫ | S |  | C |  | C |  | R |  | S |  | K | ⎱ | M | ⎫ | F |  | ⎫ | K | ⎫ | D |  | V |  | C |  | T |  | C |  | F |  | Y |  | P | ⎫ | S |  | ⎫ | Q | [ | R |  | S |  | E |  | T |  | A |  | R |  | A |  | K |  | K |  | 90 |  |
|  | |  | | | | | | | | | | | | | | | | | | | | | | | | | | | | | | | | | | | | | | | | | | | | | | | | | | | | | | | | | | | | | | | | | | | |
| 91 |  |  | E |  | L |  | C |  | T |  | C |  | Q |  | Q |  | P |  | K |  | H |  |  | L |  | K |  | Y |  | I |  | E |  | K |  | G |  | L |  | Q |  | K |  |  | A |  | K |  | D |  | Y |  | A |  | T |  | G |  | | 117 |  | | | | | |

Fixed PTMs: Carbamidomethylation [C50 C57 C64 C65 C74 C76 ]   
  
     Unexpected modifications:   Unknown [-48.02]

  

All peaks (60)  Matched peaks (14)  Not matched peaks (46)

  

| Scan | Peak | Mono mass | Mono m/z | Intensity | Charge | Theoretical mass | Ion | Pos | Mass error | PPM error |
| --- | --- | --- | --- | --- | --- | --- | --- | --- | --- | --- |
| 303 | 1 | 1226.5348 | 614.2747 | 33462.57 | 2 |  |  |  |  |  |
| 303 | 2 | 613.1679 | 614.1752 | 47235.42 | 1 |  |  |  |  |  |
| 303 | 3 | 4236.7023 | 707.1243 | 13605.71 | 6 |  |  |  |  |  |
| 303 | 4 | 4276.7162 | 611.9667 | 12670.67 | 7 |  |  |  |  |  |
| 303 | 5 | 2147.8661 | 716.9626 | 12693.71 | 3 |  |  |  |  |  |
| 303 | 6 | 3964.5745 | 793.9222 | 8300.86 | 5 |  |  |  |  |  |
| 303 | 7 | 4165.6829 | 834.1439 | 8069.54 | 5 | 4165.6965 | C33 | 33 | -0.0136 | -3.27 |
| 303 | 8 | 4278.7191 | 714.1271 | 8066.53 | 6 |  |  |  |  |  |
| 303 | 9 | 2678.0738 | 670.5257 | 8866.87 | 4 | 2678.0914 | C21 | 21 | -0.0176 | -6.59 |
| 303 | 10 | 4237.7093 | 848.5491 | 8716.24 | 5 |  |  |  |  |  |
| 303 | 11 | 4148.6586 | 692.4504 | 7012.51 | 6 |  |  |  |  |  |
| 303 | 12 | 3684.8342 | 615.1463 | 11197.82 | 6 |  |  |  |  |  |
| 303 | 13 | 4279.7179 | 856.9509 | 6525.48 | 5 |  |  |  |  |  |
| 303 | 14 | 3986.6503 | 798.3373 | 7049.01 | 5 |  |  |  |  |  |
| 303 | 15 | 4078.6513 | 816.7375 | 3221.91 | 5 | 4078.6645 | C32 | 32 | -0.0131 | -3.22 |
| 303 | 16 | 4148.6629 | 830.7399 | 3954.61 | 5 |  |  |  |  |  |
| 303 | 17 | 2908.1761 | 728.0513 | 3412.35 | 4 | 2908.1807 | C23 | 23 | -4.60e-03 | -1.58 |
| 303 | 18 | 3685.8383 | 738.1749 | 2968.49 | 5 |  |  |  |  |  |
| 303 | 19 | 3801.5090 | 761.3091 | 3149.02 | 5 |  |  |  |  |  |
| 303 | 20 | 3036.2730 | 608.2619 | 3055.94 | 5 | 3036.2756 | C24 | 24 | -2.66e-03 | -0.88 |
| 303 | 21 | 2761.1102 | 691.2848 | 5341.17 | 4 | 2761.1123 | C22 | 22 | -2.10e-03 | -0.76 |
| 303 | 22 | 4238.7119 | 1060.6853 | 2657.19 | 4 |  |  |  |  |  |
| 303 | 23 | 1491.5734 | 746.7940 | 3614.04 | 2 | 1491.5830 | C12 | 12 | -9.63e-03 | -6.46 |
| 303 | 24 | 4219.6933 | 704.2895 | 4853.90 | 6 |  |  |  |  |  |
| 303 | 25 | 3626.8116 | 726.3696 | 2951.12 | 5 |  |  |  |  |  |
| 303 | 26 | 3409.3918 | 682.8856 | 3029.54 | 5 |  |  |  |  |  |
| 303 | 27 | 4131.6448 | 689.6147 | 3485.76 | 6 |  |  |  |  |  |
| 303 | 28 | 4179.6787 | 836.9430 | 3011.15 | 5 |  |  |  |  |  |
| 303 | 29 | 2082.3407 | 695.1208 | 2380.68 | 3 |  |  |  |  |  |
| 303 | 30 | 4221.6962 | 845.3465 | 2722.87 | 5 |  |  |  |  |  |
| 303 | 31 | 2058.9160 | 687.3126 | 1848.39 | 3 |  |  |  |  |  |
| 303 | 32 | 3547.3939 | 887.8558 | 1842.94 | 4 |  |  |  |  |  |
| 303 | 33 | 4091.6347 | 819.3342 | 2460.83 | 5 |  |  |  |  |  |
| 303 | 34 | 3036.2754 | 760.0761 | 2615.35 | 4 | 3036.2756 | C24 | 24 | -2.04e-04 | -0.07 |
| 303 | 35 | 1617.6620 | 809.8383 | 3311.19 | 2 | 1617.6556 | Z\_DOT13 | 21 | 6.47e-03 | 4.00 |
| 303 | 36 | 859.5490 | 860.5563 | 4876.07 | 1 |  |  |  |  |  |
| 303 | 37 | 4260.7065 | 609.6796 | 5150.08 | 7 |  |  |  |  |  |
| 303 | 38 | 2119.3509 | 707.4576 | 3901.13 | 3 |  |  |  |  |  |
| 303 | 39 | 3249.3636 | 650.8800 | 2200.51 | 5 |  |  |  |  |  |
| 303 | 40 | 2761.1129 | 921.3782 | 1774.76 | 3 | 2761.1123 | C22 | 22 | 5.95e-04 | 0.22 |
| 303 | 41 | 2678.0745 | 893.6988 | 2558.80 | 3 | 2678.0914 | C21 | 21 | -0.0169 | -6.31 |
| 303 | 42 | 3964.5761 | 661.7700 | 2685.00 | 6 |  |  |  |  |  |
| 303 | 43 | 3296.2981 | 1099.7733 | 1442.86 | 3 |  |  |  |  |  |
| 303 | 44 | 2550.9786 | 851.3335 | 1766.20 | 3 |  |  |  |  |  |
| 303 | 45 | 1000.4450 | 501.2298 | 2341.11 | 2 | 1000.4508 | C8 | 8 | -5.77e-03 | -5.77 |
| 303 | 46 | 4132.6504 | 827.5374 | 2051.32 | 5 |  |  |  |  |  |
| 303 | 47 | 3906.5467 | 782.3166 | 2138.88 | 5 |  |  |  |  |  |
| 303 | 48 | 4108.6520 | 822.7377 | 1300.58 | 5 |  |  |  |  |  |
| 303 | 49 | 3937.5765 | 657.2700 | 1574.64 | 6 |  |  |  |  |  |
| 303 | 50 | 4192.7116 | 699.7925 | 1687.50 | 6 |  |  |  |  |  |
| 303 | 51 | 1899.7583 | 950.8864 | 1888.52 | 2 | 1899.7700 | C15 | 15 | -0.0117 | -6.18 |
| 303 | 52 | 356.0579 | 357.0652 | 2423.89 | 1 |  |  |  |  |  |
| 303 | 53 | 1474.5494 | 738.2820 | 1476.85 | 2 |  |  |  |  |  |
| 303 | 54 | 595.6957 | 596.7030 | 2183.87 | 1 |  |  |  |  |  |
| 303 | 55 | 881.3508 | 882.3581 | 1633.39 | 1 |  |  |  |  |  |
| 303 | 56 | 1115.4704 | 558.7425 | 875.22 | 2 | 1115.4778 | C9 | 9 | -7.38e-03 | -6.62 |
| 303 | 57 | 1266.5038 | 634.2592 | 496.32 | 2 |  |  |  |  |  |
| 303 | 58 | 1210.5171 | 1211.5244 | 569.92 | 1 |  |  |  |  |  |
| 303 | 59 | 725.9657 | 726.9730 | 371.73 | 1 |  |  |  |  |  |
| 303 | 60 | 710.2827 | 711.2899 | 707.23 | 1 |  |  |  |  |  |

  

All proteins /
CsTx-13a Cupiennius salei toxin 13 isoform a /
Proteoform #80
