# Supplementary material for: The Dual Prey-Inactivation Strategy of Spiders—In-Depth Venomic Analysis of Cupiennius salei
Source: Toxins (Basel). 2019 Mar 19;11(3):167. doi: 10.3390/toxins11030167 (PMC6468893; doi:10.3390/toxins11030167)
Supplement: Supplementary file 1 [file toxins-11-00167-s001.zip › Supplementary Dataset EV1/20180328_f2_topdown_OTMS2_EThcD_NL_i02_ms2_proteoform_cutoff_html/prsms/prsm130.html]

Protein-Spectrum-Match for Spectrum #367


All proteins /
CsTx-1a\_S1 Cupiennius salei toxin 1 isoform a S1^ACsTx-1a\_S2 Cupiennius salei toxin 1 isoform a S2 /
Proteoform #8

## Protein-Spectrum-Match #130 for Spectrum #367

|  |  |  |  |  |  |
| --- | --- | --- | --- | --- | --- |
| PrSM ID: | 130 | Scan(s): | 492 | Precursor charge: | 9 |
| Precursor m/z: | 797.5832 | Precursor mass: | 7169.1830 | Proteoform mass: | 7169.1856 |
| # matched peaks: | 42 | # matched fragment ions: | 36 | # unexpected modifications: | 0 |
| E-value: | 1.19e-33 | P-value: | 1.19e-33 | Q-value (Spectral FDR): | 0 |

  

|  |  |  |  |  |  |  |  |  |  |  |  |  |  |  |  |  |  |  |  |  |  |  |  |  |  |  |  |  |  |  |  |  |  |  |  |  |  |  |  |  |  |  |  |  |  |  |  |  |  |  |  |  |  |  |  |  |  |  |  |  |  |  |  |  |  |  |  |  |  |
| --- | --- | --- | --- | --- | --- | --- | --- | --- | --- | --- | --- | --- | --- | --- | --- | --- | --- | --- | --- | --- | --- | --- | --- | --- | --- | --- | --- | --- | --- | --- | --- | --- | --- | --- | --- | --- | --- | --- | --- | --- | --- | --- | --- | --- | --- | --- | --- | --- | --- | --- | --- | --- | --- | --- | --- | --- | --- | --- | --- | --- | --- | --- | --- | --- | --- | --- | --- | --- | --- |
|  | |  | | | | | | | | | | | | | | | | | | | | | | | | | | | | | | | | | | | | | | | | | | | | | | | | | | | | | | | | | | | | | | | | | | | |
| 1 |  |  | M |  | K |  | V |  | L |  | I |  | I |  | S |  | A |  | V |  | L |  |  | F |  | I |  | T |  | I |  | F |  | S |  | N |  | I |  | S |  | A |  |  | E |  | I |  | E |  | D |  | D |  | F |  | L |  | E |  | D |  | E |  | 30 |  |
|  | |  | | | | | | | | | | | | | | | | | | | | | | | | | | | | | | | | | | | | | | | | | | | | | | | | | | | | | | | | | | | | | | | | | | | |
| 31 |  |  | S |  | F |  | E |  | A |  | E |  | D |  | I |  | I |  | P |  | F |  |  | F |  | E |  | N |  | E |  | Q |  | A |  | R | ] | S |  | C |  | I |  | ⎩ | P |  | K | ⎫ | H | ⎫ | E | ⎱ | E | ⎫ | C |  | T | ⎫ | N | ⎱ | D |  | K |  | 60 |  |
|  | |  | | | | | | | | | | | | | | | | | | | | | | | | | | | | | | | | | | | | | | | | | | | | | | | | | | | | | | | | | | | | | | | | | | | |
| 61 |  | ⎫ | H | ⎩ | N | ⎫ | C |  | C |  | R |  | K | ⎫ | G |  | L | ⎫ | F | ⎫ | K |  |  | L |  | K | ⎫ | C | ⎫ | Q | ⎫ | C |  | S |  | T |  | F | ⎩ | D | ⎫ | D |  | ⎫ | E | ⎫ | S |  | G | ⎱ | Q |  | P |  | T |  | E |  | R |  | C |  | A |  | 90 |  |
|  | |  | | | | | | | | | | | | | | | | | | | | | | | | | | | | | | | | | | | | | | | | | | | | | | | | | | | | | | | | | | | | | | | | | | | |
| 91 |  |  | C | ⎫ | G | ⎱ | R |  | P |  | M |  | G | ⎱ | H | ⎫ | Q | ⎫ | A |  | I |  |  | E |  | T | ⎫ | G | ⎫ | L | ⎫ | N | ⎫ | I | ⎫ | F | [ | R |  | G |  | L |  |  | F |  | K |  | G |  | K |  | K |  | K |  | N |  | K |  | K |  | T |  | 120 |  |
|  | |  | | | | | | | | | | | | | | | | | | | | | | | | | | | | | | | | | | | | | | | | | | | | | | | | | | | | | | | | | | | | | | | | | | | |
| 121 |  |  | K |  | G |  | | | | 122 |  | | | | | | | | | | | | | | | | | | | | | | | | | | | | | | | | | | | | | | | | | | | | | | | | | | | | | | | |

Fixed PTMs: Carbamidomethylation [C49 C56 C63 C64 C73 C75 C89 C91 ]

  

All peaks (115)  Matched peaks (42)  Not matched peaks (73)

  

| Scan | Peak | Mono mass | Mono m/z | Intensity | Charge | Theoretical mass | Ion | Pos | Mass error | PPM error |
| --- | --- | --- | --- | --- | --- | --- | --- | --- | --- | --- |
| 492 | 1 | 7112.1190 | 1017.0243 | 54972.03 | 7 |  |  |  |  |  |
| 492 | 2 | 6890.9750 | 862.3792 | 38211.28 | 8 |  |  |  |  |  |
| 492 | 3 | 6947.0392 | 993.4415 | 35074.52 | 7 |  |  |  |  |  |
| 492 | 4 | 6890.9824 | 985.4333 | 32098.60 | 7 |  |  |  |  |  |
| 492 | 5 | 7005.0652 | 876.6404 | 41840.27 | 8 |  |  |  |  |  |
| 492 | 6 | 2614.2046 | 872.4088 | 41782.96 | 3 |  |  |  |  |  |
| 492 | 7 | 7005.0644 | 1001.7308 | 31506.79 | 7 |  |  |  |  |  |
| 492 | 8 | 7055.0932 | 1008.8777 | 18162.21 | 7 |  |  |  |  |  |
| 492 | 9 | 7153.1295 | 1022.8829 | 19373.84 | 7 |  |  |  |  |  |
| 492 | 10 | 6833.9553 | 977.2866 | 21301.51 | 7 |  |  |  |  |  |
| 492 | 11 | 7112.1161 | 890.0218 | 23736.41 | 8 |  |  |  |  |  |
| 492 | 12 | 7113.1275 | 1186.5285 | 18211.58 | 6 |  |  |  |  |  |
| 492 | 13 | 7126.1267 | 1019.0254 | 13143.08 | 7 |  |  |  |  |  |
| 492 | 14 | 4554.9372 | 911.9947 | 12344.59 | 5 |  |  |  |  |  |
| 492 | 15 | 6947.0375 | 869.3870 | 12012.66 | 8 |  |  |  |  |  |
| 492 | 16 | 3157.4969 | 790.3815 | 10950.59 | 4 | 3157.5153 | C25 | 25 | -0.0185 | -5.85 |
| 492 | 17 | 4554.9346 | 760.1630 | 13663.78 | 6 |  |  |  |  |  |
| 492 | 18 | 6947.0442 | 1158.8480 | 11615.71 | 6 |  |  |  |  |  |
| 492 | 19 | 3585.5704 | 897.3999 | 20054.10 | 4 |  |  |  |  |  |
| 492 | 20 | 7055.0992 | 1176.8571 | 12976.56 | 6 |  |  |  |  |  |
| 492 | 21 | 7152.1239 | 895.0228 | 9985.00 | 8 |  |  |  |  |  |
| 492 | 22 | 7096.1122 | 1014.7376 | 14564.78 | 7 |  |  |  |  |  |
| 492 | 23 | 2788.2253 | 930.4157 | 10672.02 | 3 | 2788.2414 | C22 | 22 | -0.0161 | -5.77 |
| 492 | 24 | 1866.7997 | 934.4071 | 11010.76 | 2 | 1866.8101 | C15 | 15 | -0.0104 | -5.57 |
| 492 | 25 | 7068.1071 | 1010.7369 | 9365.02 | 7 |  |  |  |  |  |
| 492 | 26 | 6978.0796 | 997.8758 | 8984.13 | 7 |  |  |  |  |  |
| 492 | 27 | 6962.0552 | 995.5866 | 7594.91 | 7 |  |  |  |  |  |
| 492 | 28 | 5503.3199 | 918.2273 | 7931.85 | 6 | 5503.3559 | C45 | 45 | -0.0360 | -6.54 |
| 492 | 29 | 3445.5836 | 862.4032 | 7373.03 | 4 | 3445.6046 | C27 | 27 | -0.0210 | -6.08 |
| 492 | 30 | 7096.1060 | 1183.6916 | 8215.88 | 6 |  |  |  |  |  |
| 492 | 31 | 6524.8244 | 1088.4780 | 6437.59 | 6 |  |  |  |  |  |
| 492 | 32 | 4443.9094 | 1111.9846 | 6474.88 | 4 | 4443.9333 | C36 | 36 | -0.0240 | -5.39 |
| 492 | 33 | 2641.1559 | 881.3926 | 7879.71 | 3 | 2641.1730 | C21 | 21 | -0.0171 | -6.47 |
| 492 | 34 | 4269.8496 | 1068.4697 | 5044.84 | 4 |  |  |  |  |  |
| 492 | 35 | 6918.0472 | 989.2997 | 6754.32 | 7 |  |  |  |  |  |
| 492 | 36 | 6890.9884 | 1149.5053 | 6858.80 | 6 |  |  |  |  |  |
| 492 | 37 | 4443.9023 | 889.7877 | 11684.75 | 5 | 4443.9333 | C36 | 36 | -0.0310 | -6.97 |
| 492 | 38 | 2726.2408 | 909.7542 | 6571.49 | 3 | 2726.2602 | Z\_DOT24 | 36 | -0.0194 | -7.11 |
| 492 | 39 | 6989.0495 | 999.4429 | 7001.96 | 7 |  |  |  |  |  |
| 492 | 40 | 5798.5763 | 967.4367 | 3903.14 | 6 | 5797.6072 | Z\_DOT49 | 11 | -0.0332 | -5.72 |
| 492 | 41 | 7005.0700 | 1168.5189 | 6312.51 | 6 |  |  |  |  |  |
| 492 | 42 | 7095.1078 | 887.8958 | 7925.13 | 8 |  |  |  |  |  |
| 492 | 43 | 3317.5254 | 830.3886 | 5860.46 | 4 | 3317.5460 | C26 | 26 | -0.0206 | -6.20 |
| 492 | 44 | 7069.1027 | 1179.1911 | 6171.22 | 6 |  |  |  |  |  |
| 492 | 45 | 6921.0623 | 1154.5177 | 4856.56 | 6 |  |  |  |  |  |
| 492 | 46 | 7125.1185 | 891.6471 | 7519.63 | 8 |  |  |  |  |  |
| 492 | 47 | 5447.3040 | 1090.4681 | 3741.31 | 5 | 5446.3345 | C44 | 44 | -0.0328 | -6.02 |
| 492 | 48 | 4299.8472 | 1075.9691 | 4848.04 | 4 | 4299.8798 | C34 | 34 | -0.0326 | -7.59 |
| 492 | 49 | 6987.0521 | 874.3888 | 3862.31 | 8 |  |  |  |  |  |
| 492 | 50 | 4497.9206 | 900.5914 | 5226.97 | 5 |  |  |  |  |  |
| 492 | 51 | 6908.0092 | 864.5084 | 4321.56 | 8 | 6908.0491 | C58 | 58 | -0.0399 | -5.78 |
| 492 | 52 | 1666.8278 | 834.4212 | 5456.59 | 2 | 1666.8376 | Z\_DOT15 | 45 | -9.78e-03 | -5.87 |
| 492 | 53 | 7022.1170 | 1004.1669 | 4613.47 | 7 | 7021.1332 | C59 | 59 | -0.0185 | -2.63 |
| 492 | 54 | 3229.3895 | 1077.4705 | 3998.17 | 3 | 3229.4101 | Z\_DOT29 | 31 | -0.0206 | -6.38 |
| 492 | 55 | 6833.9686 | 1140.0020 | 6435.22 | 6 |  |  |  |  |  |
| 492 | 56 | 1592.8128 | 797.4137 | 6190.52 | 2 |  |  |  |  |  |
| 492 | 57 | 2471.0527 | 824.6915 | 5133.62 | 3 | 2471.0674 | C19 | 19 | -0.0147 | -5.95 |
| 492 | 58 | 3157.4972 | 1053.5063 | 3715.21 | 3 | 3157.5153 | C25 | 25 | -0.0182 | -5.75 |
| 492 | 59 | 6209.6477 | 1035.9486 | 5926.33 | 6 | 6209.6892 | C51 | 51 | -0.0415 | -6.69 |
| 492 | 60 | 6793.9698 | 971.5744 | 8182.00 | 7 | 6793.0202 | Z\_DOT57 | 3 | -0.0528 | -7.78 |
| 492 | 60 | 6793.9698 | 971.5744 | 8182.00 | 7 | 6794.0062 | C57 | 57 | -0.0364 | -5.36 |
| 492 | 61 | 7154.1197 | 1193.3606 | 6070.13 | 6 |  |  |  |  |  |
| 492 | 62 | 4055.7880 | 812.1649 | 4145.00 | 5 | 4055.8103 | C32 | 32 | -0.0223 | -5.49 |
| 492 | 63 | 7081.1150 | 1012.5951 | 4588.36 | 7 |  |  |  |  |  |
| 492 | 64 | 4170.8038 | 1043.7082 | 3932.48 | 4 | 4170.8372 | C33 | 33 | -0.0335 | -8.03 |
| 492 | 65 | 868.4187 | 869.4260 | 6233.34 | 1 | 868.4225 | C7 | 7 | -3.71e-03 | -4.28 |
| 492 | 66 | 6681.8831 | 955.5620 | 5297.62 | 7 | 6680.9221 | C56 | 56 | -0.0413 | -6.19 |
| 492 | 67 | 2871.3021 | 958.1080 | 5476.15 | 3 |  |  |  |  |  |
| 492 | 68 | 6776.9385 | 969.1413 | 5272.37 | 7 |  |  |  |  |  |
| 492 | 69 | 4900.0968 | 981.0266 | 3695.98 | 5 |  |  |  |  |  |
| 492 | 70 | 6931.0231 | 991.1534 | 4994.73 | 7 |  |  |  |  |  |
| 492 | 71 | 6792.9694 | 1133.1688 | 4885.69 | 6 | 6793.0202 | Z\_DOT57 | 3 | -0.0508 | -7.48 |
| 492 | 71 | 6792.9694 | 1133.1688 | 4885.69 | 6 | 6794.0062 | C57 | 57 | -0.0344 | -5.07 |
| 492 | 72 | 6623.8544 | 1104.9830 | 3692.64 | 6 | 6623.9007 | C55 | 55 | -0.0463 | -6.99 |
| 492 | 73 | 7004.0603 | 779.2362 | 4190.98 | 9 |  |  |  |  |  |
| 492 | 74 | 4170.8098 | 835.1692 | 3226.03 | 5 | 4170.8372 | C33 | 33 | -0.0274 | -6.57 |
| 492 | 75 | 5503.3155 | 1101.6704 | 4898.13 | 5 | 5503.3559 | C45 | 45 | -0.0404 | -7.34 |
| 492 | 76 | 6990.0437 | 1166.0146 | 3703.47 | 6 |  |  |  |  |  |
| 492 | 77 | 4299.8523 | 860.9777 | 4268.13 | 5 | 4299.8798 | C34 | 34 | -0.0275 | -6.39 |
| 492 | 78 | 997.4598 | 998.4670 | 4260.55 | 1 | 997.4651 | C8 | 8 | -5.29e-03 | -5.30 |
| 492 | 79 | 5417.3907 | 903.9057 | 2315.43 | 6 | 5417.4264 | Z\_DOT46 | 14 | -0.0356 | -6.57 |
| 492 | 80 | 6420.7712 | 1071.1358 | 4120.65 | 6 |  |  |  |  |  |
| 492 | 81 | 6301.7446 | 1051.2980 | 3438.12 | 6 | 6301.7710 | Z\_DOT53 | 7 | -0.0264 | -4.19 |
| 492 | 82 | 5944.5297 | 991.7622 | 3239.05 | 6 | 5944.5717 | C49 | 49 | -0.0420 | -7.07 |
| 492 | 83 | 1615.6988 | 808.8567 | 3371.30 | 2 | 1615.7082 | C13 | 13 | -9.45e-03 | -5.85 |
| 492 | 84 | 1793.8785 | 897.9465 | 3313.17 | 2 |  |  |  |  |  |
| 492 | 85 | 3941.7623 | 986.4478 | 7199.64 | 4 |  |  |  |  |  |
| 492 | 86 | 5419.4152 | 1084.8903 | 4178.16 | 5 |  |  |  |  |  |
| 492 | 87 | 2917.3210 | 973.4476 | 3734.41 | 3 |  |  |  |  |  |
| 492 | 88 | 4643.0390 | 929.6151 | 2133.79 | 5 |  |  |  |  |  |
| 492 | 89 | 3757.5670 | 940.3990 | 2643.71 | 4 |  |  |  |  |  |
| 492 | 90 | 2668.2130 | 890.4116 | 3851.74 | 3 |  |  |  |  |  |
| 492 | 91 | 1372.5783 | 687.2964 | 3889.27 | 2 | 1372.5863 | C11 | 11 | -8.04e-03 | -5.85 |
| 492 | 92 | 3693.6414 | 924.4176 | 2632.02 | 4 |  |  |  |  |  |
| 492 | 93 | 4382.9326 | 1096.7404 | 3258.85 | 4 |  |  |  |  |  |
| 492 | 94 | 2025.8278 | 1013.9212 | 3696.89 | 2 |  |  |  |  |  |
| 492 | 95 | 6779.9404 | 848.4998 | 1923.33 | 8 |  |  |  |  |  |
| 492 | 96 | 6082.5942 | 869.9493 | 4148.10 | 7 | 6081.6306 | C50 | 50 | -0.0388 | -6.37 |
| 492 | 97 | 3183.5121 | 1062.1780 | 1682.10 | 3 |  |  |  |  |  |
| 492 | 98 | 602.3180 | 603.3253 | 3340.24 | 1 | 602.3210 | C5 | 5 | -2.98e-03 | -4.95 |
| 492 | 99 | 842.6905 | 843.6978 | 1931.05 | 1 |  |  |  |  |  |
| 492 | 100 | 739.3755 | 740.3828 | 2468.36 | 1 | 739.3799 | C6 | 6 | -4.34e-03 | -5.87 |
| 492 | 101 | 1372.5768 | 1373.5840 | 1970.52 | 1 | 1372.5863 | C11 | 11 | -9.55e-03 | -6.96 |
| 492 | 102 | 1258.5364 | 1259.5436 | 1401.55 | 1 | 1258.5434 | C10 | 10 | -7.03e-03 | -5.58 |
| 492 | 103 | 1057.4565 | 1058.4638 | 1031.61 | 1 |  |  |  |  |  |
| 492 | 104 | 917.3856 | 918.3929 | 850.56 | 1 |  |  |  |  |  |
| 492 | 105 | 1160.6787 | 1161.6860 | 1101.65 | 1 |  |  |  |  |  |
| 492 | 106 | 1459.1564 | 730.5855 | 687.65 | 2 |  |  |  |  |  |
| 492 | 107 | 1042.8727 | 1043.8799 | 1004.34 | 1 |  |  |  |  |  |
| 492 | 108 | 1140.4922 | 1141.4994 | 1233.68 | 1 |  |  |  |  |  |
| 492 | 109 | 757.3503 | 758.3575 | 753.18 | 1 |  |  |  |  |  |
| 492 | 110 | 953.4501 | 954.4573 | 1010.83 | 1 |  |  |  |  |  |
| 492 | 111 | 816.3754 | 817.3826 | 822.75 | 1 |  |  |  |  |  |
| 492 | 112 | 1411.4214 | 1412.4286 | 662.19 | 1 |  |  |  |  |  |
| 492 | 113 | 1396.6173 | 1397.6246 | 583.39 | 1 |  |  |  |  |  |
| 492 | 114 | 1225.6156 | 1226.6229 | 737.70 | 1 | 1225.6217 | Z\_DOT11 | 49 | -6.16e-03 | -5.03 |
| 492 | 115 | 1423.6272 | 1424.6345 | 1085.37 | 1 |  |  |  |  |  |

  

All proteins /
CsTx-1a\_S1 Cupiennius salei toxin 1 isoform a S1^ACsTx-1a\_S2 Cupiennius salei toxin 1 isoform a S2 /
Proteoform #8
