# Supplementary material for: The Dual Prey-Inactivation Strategy of Spiders—In-Depth Venomic Analysis of Cupiennius salei
Source: Toxins (Basel). 2019 Mar 19;11(3):167. doi: 10.3390/toxins11030167 (PMC6468893; doi:10.3390/toxins11030167)
Supplement: Supplementary file 1 [file toxins-11-00167-s001.zip › Supplementary Dataset EV1/20180328_f2_topdown_OTMS2_EThcD_NL_i02_ms2_proteoform_cutoff_html/prsms/prsm131.html]

Protein-Spectrum-Match for Spectrum #368


All proteins /
CsTx-1a\_S1 Cupiennius salei toxin 1 isoform a S1^ACsTx-1a\_S2 Cupiennius salei toxin 1 isoform a S2 /
Proteoform #16

## Protein-Spectrum-Match #131 for Spectrum #368

|  |  |  |  |  |  |
| --- | --- | --- | --- | --- | --- |
| PrSM ID: | 131 | Scan(s): | 493 | Precursor charge: | 7 |
| Precursor m/z: | 1047.3362 | Precursor mass: | 7324.3022 | Proteoform mass: | 7325.2868 |
| # matched peaks: | 31 | # matched fragment ions: | 30 | # unexpected modifications: | 0 |
| E-value: | 6.48e-31 | P-value: | 6.48e-31 | Q-value (Spectral FDR): | 0 |

  

|  |  |  |  |  |  |  |  |  |  |  |  |  |  |  |  |  |  |  |  |  |  |  |  |  |  |  |  |  |  |  |  |  |  |  |  |  |  |  |  |  |  |  |  |  |  |  |  |  |  |  |  |  |  |  |  |  |  |  |  |  |  |  |  |  |  |  |  |  |  |
| --- | --- | --- | --- | --- | --- | --- | --- | --- | --- | --- | --- | --- | --- | --- | --- | --- | --- | --- | --- | --- | --- | --- | --- | --- | --- | --- | --- | --- | --- | --- | --- | --- | --- | --- | --- | --- | --- | --- | --- | --- | --- | --- | --- | --- | --- | --- | --- | --- | --- | --- | --- | --- | --- | --- | --- | --- | --- | --- | --- | --- | --- | --- | --- | --- | --- | --- | --- | --- | --- |
|  | |  | | | | | | | | | | | | | | | | | | | | | | | | | | | | | | | | | | | | | | | | | | | | | | | | | | | | | | | | | | | | | | | | | | | |
| 1 |  |  | M |  | K |  | V |  | L |  | I |  | I |  | S |  | A |  | V |  | L |  |  | F |  | I |  | T |  | I |  | F |  | S |  | N |  | I |  | S |  | A |  |  | E |  | I |  | E |  | D |  | D |  | F |  | L |  | E |  | D |  | E |  | 30 |  |
|  | |  | | | | | | | | | | | | | | | | | | | | | | | | | | | | | | | | | | | | | | | | | | | | | | | | | | | | | | | | | | | | | | | | | | | |
| 31 |  |  | S |  | F |  | E |  | A |  | E |  | D |  | I |  | I |  | P |  | F |  |  | F |  | E |  | N |  | E |  | Q |  | A |  | R | ] | S | ⎩ | C | ⎩ | I |  |  | P | ⎱ | K | ⎫ | H | ⎱ | E | ⎱ | E | ⎫ | C | ⎫ | T |  | N | ⎩ | D |  | K |  | 60 |  |
|  | |  | | | | | | | | | | | | | | | | | | | | | | | | | | | | | | | | | | | | | | | | | | | | | | | | | | | | | | | | | | | | | | | | | | | |
| 61 |  |  | H | ⎫ | N | ⎫ | C | ⎫ | C |  | R |  | K | ⎫ | G |  | L |  | F |  | K |  | ⎫ | L |  | K | ⎫ | C | ⎫ | Q |  | C |  | S |  | T | ⎫ | F |  | D |  | D |  |  | E |  | S |  | G | ⎫ | Q |  | P |  | T |  | E |  | R |  | C |  | A |  | 90 |  |
|  | |  | | | | | | | | | | | | | | | | | | | | | | | | | | | | | | | | | | | | | | | | | | | | | | | | | | | | | | | | | | | | | | | | | | | |
| 91 |  |  | C |  | G | ⎫ | R |  | P | ⎫ | M | ⎫ | G |  | H | ⎱ | Q | ⎫ | A |  | I |  |  | E | ⎫ | T |  | G |  | L | ⎫ | N |  | I |  | F | ⎫ | R | [ | G |  | L |  |  | F |  | K |  | G |  | K |  | K |  | K |  | N |  | K |  | K |  | T |  | 120 |  |
|  | |  | | | | | | | | | | | | | | | | | | | | | | | | | | | | | | | | | | | | | | | | | | | | | | | | | | | | | | | | | | | | | | | | | | | |
| 121 |  |  | K |  | G |  | | | | 122 |  | | | | | | | | | | | | | | | | | | | | | | | | | | | | | | | | | | | | | | | | | | | | | | | | | | | | | | | |

Fixed PTMs: Carbamidomethylation [C49 C56 C63 C64 C73 C75 C89 C91 ]

  

All peaks (81)  Matched peaks (31)  Not matched peaks (50)

  

| Scan | Peak | Mono mass | Mono m/z | Intensity | Charge | Theoretical mass | Ion | Pos | Mass error | PPM error |
| --- | --- | --- | --- | --- | --- | --- | --- | --- | --- | --- |
| 493 | 1 | 7267.2305 | 1212.2124 | 137199.93 | 6 |  |  |  |  |  |
| 493 | 2 | 3662.6277 | 1221.8832 | 90011.50 | 3 |  |  |  |  |  |
| 493 | 3 | 7307.2260 | 1044.8967 | 26237.74 | 7 |  |  |  |  |  |
| 493 | 4 | 7280.2419 | 1214.3809 | 27386.87 | 6 |  |  |  |  |  |
| 493 | 5 | 7308.2380 | 1219.0469 | 16644.16 | 6 |  |  |  |  |  |
| 493 | 6 | 7267.2338 | 1454.4540 | 15639.33 | 5 |  |  |  |  |  |
| 493 | 7 | 7251.2142 | 1209.5430 | 13143.05 | 6 |  |  |  |  |  |
| 493 | 8 | 5887.5115 | 1178.5096 | 10609.09 | 5 | 5887.5503 | C48 | 48 | -0.0388 | -6.59 |
| 493 | 9 | 1046.6097 | 1047.6170 | 78486.47 | 1 |  |  |  |  |  |
| 493 | 10 | 7235.2482 | 1206.8820 | 10095.20 | 6 |  |  |  |  |  |
| 493 | 11 | 7132.2049 | 1189.7081 | 12243.01 | 6 |  |  |  |  |  |
| 493 | 12 | 1821.9443 | 911.9794 | 11147.17 | 2 |  |  |  |  |  |
| 493 | 13 | 7221.2186 | 1204.5437 | 7929.96 | 6 | 7222.2361 | Z\_DOT60 | 1 | -0.0151 | -2.09 |
| 493 | 14 | 1752.7559 | 877.3852 | 8528.98 | 2 | 1752.7671 | C14 | 14 | -0.0112 | -6.41 |
| 493 | 15 | 7176.2252 | 1197.0448 | 5725.61 | 6 |  |  |  |  |  |
| 493 | 16 | 7190.2361 | 1199.3800 | 6547.15 | 6 |  |  |  |  |  |
| 493 | 17 | 6238.8092 | 1248.7691 | 5385.02 | 5 |  |  |  |  |  |
| 493 | 18 | 1866.7987 | 934.4066 | 7414.61 | 2 | 1866.8101 | C15 | 15 | -0.0114 | -6.10 |
| 493 | 19 | 7282.2467 | 1457.4566 | 4487.43 | 5 |  |  |  |  |  |
| 493 | 20 | 7210.2044 | 1443.0482 | 5630.08 | 5 |  |  |  |  |  |
| 493 | 21 | 2782.3046 | 928.4421 | 5291.08 | 3 |  |  |  |  |  |
| 493 | 22 | 602.3176 | 603.3249 | 9114.65 | 1 | 602.3210 | C5 | 5 | -3.35e-03 | -5.56 |
| 493 | 23 | 1948.9926 | 975.5036 | 5140.03 | 2 |  |  |  |  |  |
| 493 | 24 | 6585.8814 | 1318.1836 | 2664.32 | 5 | 6586.9147 | Z\_DOT55 | 6 | -0.0309 | -4.69 |
| 493 | 25 | 6082.5822 | 1217.5237 | 3941.69 | 5 | 6081.6306 | C50 | 50 | -0.0508 | -8.35 |
| 493 | 26 | 2881.3597 | 961.4605 | 5131.14 | 3 |  |  |  |  |  |
| 493 | 27 | 7168.1492 | 1434.6371 | 3365.60 | 5 | 7168.2016 | C60 | 60 | -0.0523 | -7.30 |
| 493 | 28 | 868.4174 | 869.4247 | 6165.97 | 1 | 868.4225 | C7 | 7 | -5.06e-03 | -5.82 |
| 493 | 29 | 739.3756 | 740.3829 | 6389.51 | 1 | 739.3799 | C6 | 6 | -4.28e-03 | -5.78 |
| 493 | 30 | 5953.6870 | 1191.7447 | 3179.57 | 5 | 5953.7083 | Z\_DOT50 | 11 | -0.0213 | -3.57 |
| 493 | 31 | 5756.4775 | 1152.3028 | 2752.06 | 5 | 5756.5098 | C47 | 47 | -0.0322 | -5.60 |
| 493 | 32 | 7208.2189 | 1202.3771 | 3611.49 | 6 |  |  |  |  |  |
| 493 | 33 | 3157.4913 | 1053.5044 | 2785.67 | 3 | 3157.5153 | C25 | 25 | -0.0240 | -7.60 |
| 493 | 34 | 2916.3132 | 973.1117 | 2654.87 | 3 | 2916.3363 | C23 | 23 | -0.0231 | -7.92 |
| 493 | 35 | 6457.8546 | 1292.5782 | 3204.39 | 5 | 6457.8721 | Z\_DOT54 | 7 | -0.0176 | -2.72 |
| 493 | 36 | 6793.9656 | 1359.8004 | 4118.43 | 5 | 6794.0062 | C57 | 57 | -0.0405 | -5.97 |
| 493 | 37 | 6208.6540 | 1242.7381 | 2584.89 | 5 | 6209.6892 | C51 | 51 | -0.0329 | -5.30 |
| 493 | 38 | 2180.0576 | 1091.0361 | 1900.77 | 2 |  |  |  |  |  |
| 493 | 39 | 2426.1619 | 809.7279 | 3216.25 | 3 |  |  |  |  |  |
| 493 | 40 | 7222.2217 | 1445.4516 | 2595.70 | 5 | 7222.2361 | Z\_DOT60 | 1 | -0.0144 | -2.00 |
| 493 | 41 | 7310.2423 | 1463.0557 | 2734.41 | 5 |  |  |  |  |  |
| 493 | 42 | 1221.7106 | 1222.7179 | 42231.79 | 1 |  |  |  |  |  |
| 493 | 43 | 3317.5226 | 1106.8481 | 2863.04 | 3 | 3317.5460 | C26 | 26 | -0.0234 | -7.06 |
| 493 | 44 | 5368.4790 | 1343.1270 | 3320.31 | 4 |  |  |  |  |  |
| 493 | 45 | 997.4592 | 998.4664 | 2961.22 | 1 | 997.4651 | C8 | 8 | -5.90e-03 | -5.92 |
| 493 | 46 | 532.3096 | 533.3168 | 2537.49 | 1 |  |  |  |  |  |
| 493 | 47 | 6852.0442 | 1371.4161 | 2470.96 | 5 | 6852.0686 | Z\_DOT57 | 4 | -0.0243 | -3.55 |
| 493 | 48 | 7132.2223 | 1427.4517 | 2240.87 | 5 |  |  |  |  |  |
| 493 | 49 | 6398.8247 | 1280.7722 | 1292.59 | 5 |  |  |  |  |  |
| 493 | 50 | 6183.7887 | 1237.7650 | 2353.05 | 5 |  |  |  |  |  |
| 493 | 51 | 803.4606 | 804.4679 | 1743.94 | 1 |  |  |  |  |  |
| 493 | 52 | 7250.2024 | 1451.0478 | 2087.61 | 5 |  |  |  |  |  |
| 493 | 53 | 5504.3110 | 1377.0850 | 1956.70 | 4 | 5503.3559 | C45 | 45 | -0.0472 | -8.58 |
| 493 | 54 | 474.2236 | 475.2308 | 1879.55 | 1 | 474.2260 | C4 | 4 | -2.45e-03 | -5.16 |
| 493 | 55 | 4423.9642 | 1475.6620 | 1099.82 | 3 |  |  |  |  |  |
| 493 | 56 | 3029.7542 | 1010.9253 | 2530.79 | 3 |  |  |  |  |  |
| 493 | 57 | 1878.9669 | 940.4907 | 1907.09 | 2 |  |  |  |  |  |
| 493 | 58 | 6523.8156 | 1305.7704 | 3687.61 | 5 | 6522.8530 | C54 | 54 | -0.0398 | -6.10 |
| 493 | 59 | 7076.1821 | 1416.2437 | 1485.29 | 5 |  |  |  |  |  |
| 493 | 60 | 7077.1857 | 1180.5382 | 1828.88 | 6 |  |  |  |  |  |
| 493 | 61 | 4443.9037 | 1482.3085 | 1178.50 | 3 | 4443.9333 | C36 | 36 | -0.0296 | -6.67 |
| 493 | 62 | 2471.0495 | 1236.5320 | 864.49 | 2 | 2471.0674 | C19 | 19 | -0.0179 | -7.25 |
| 493 | 63 | 2026.8296 | 1014.4221 | 1063.83 | 2 | 2026.8407 | C16 | 16 | -0.0112 | -5.50 |
| 493 | 64 | 702.4137 | 703.4210 | 1105.81 | 1 |  |  |  |  |  |
| 493 | 65 | 7233.2279 | 1034.3255 | 1437.67 | 7 |  |  |  |  |  |
| 493 | 66 | 7062.1858 | 1413.4444 | 1541.09 | 5 | 7062.2054 | Z\_DOT59 | 2 | -0.0196 | -2.77 |
| 493 | 67 | 1116.6173 | 1117.6246 | 1033.14 | 1 |  |  |  |  |  |
| 493 | 68 | 2369.1335 | 790.7184 | 751.76 | 3 |  |  |  |  |  |
| 493 | 69 | 1466.2562 | 1467.2635 | 3101.77 | 1 |  |  |  |  |  |
| 493 | 70 | 2823.3272 | 942.1163 | 1588.80 | 3 |  |  |  |  |  |
| 493 | 71 | 1244.6763 | 1245.6835 | 745.84 | 1 | 1244.6639 | Z\_DOT11 | 50 | 0.0123 | 9.89 |
| 493 | 72 | 487.2882 | 488.2954 | 689.49 | 1 |  |  |  |  |  |
| 493 | 73 | 3793.6925 | 1265.5714 | 800.82 | 3 | 3793.7149 | C30 | 30 | -0.0225 | -5.93 |
| 493 | 74 | 1157.4888 | 1158.4960 | 432.12 | 1 | 1157.4957 | C9 | 9 | -6.95e-03 | -6.01 |
| 493 | 75 | 1263.8940 | 1264.9013 | 390.42 | 1 |  |  |  |  |  |
| 493 | 76 | 1192.8578 | 1193.8651 | 700.68 | 1 |  |  |  |  |  |
| 493 | 77 | 1325.1745 | 1326.1818 | 378.56 | 1 |  |  |  |  |  |
| 493 | 78 | 502.2628 | 503.2700 | 658.61 | 1 |  |  |  |  |  |
| 493 | 79 | 7279.2456 | 1040.8995 | 1083.05 | 7 |  |  |  |  |  |
| 493 | 80 | 1345.1932 | 1346.2005 | 1394.93 | 1 |  |  |  |  |  |
| 493 | 81 | 4408.9479 | 1103.2443 | 996.22 | 4 |  |  |  |  |  |

  

All proteins /
CsTx-1a\_S1 Cupiennius salei toxin 1 isoform a S1^ACsTx-1a\_S2 Cupiennius salei toxin 1 isoform a S2 /
Proteoform #16
