# Supplementary material for: The Dual Prey-Inactivation Strategy of Spiders—In-Depth Venomic Analysis of Cupiennius salei
Source: Toxins (Basel). 2019 Mar 19;11(3):167. doi: 10.3390/toxins11030167 (PMC6468893; doi:10.3390/toxins11030167)
Supplement: Supplementary file 1 [file toxins-11-00167-s001.zip › Supplementary Dataset EV1/20180328_f2_topdown_OTMS2_EThcD_NL_i02_ms2_proteoform_cutoff_html/prsms/prsm132.html]

Protein-Spectrum-Match for Spectrum #369


All proteins /
CsTx-12b Cupiennius salei toxin 12 isoform b /
Proteoform #47

## Protein-Spectrum-Match #132 for Spectrum #369

|  |  |  |  |  |  |
| --- | --- | --- | --- | --- | --- |
| PrSM ID: | 132 | Scan(s): | 495 | Precursor charge: | 6 |
| Precursor m/z: | 571.9905 | Precursor mass: | 3425.8993 | Proteoform mass: | 3425.8943 |
| # matched peaks: | 29 | # matched fragment ions: | 25 | # unexpected modifications: | 1 |
| E-value: | 5.31e-20 | P-value: | 5.31e-20 | Q-value (Spectral FDR): | 0 |

  

|  |  |  |  |  |  |  |  |  |  |  |  |  |  |  |  |  |  |  |  |  |  |  |  |  |  |  |  |  |  |  |  |  |  |  |  |  |  |  |  |  |  |  |  |  |  |  |  |  |  |  |  |  |  |  |  |  |  |  |  |  |  |  |  |  |  |  |
| --- | --- | --- | --- | --- | --- | --- | --- | --- | --- | --- | --- | --- | --- | --- | --- | --- | --- | --- | --- | --- | --- | --- | --- | --- | --- | --- | --- | --- | --- | --- | --- | --- | --- | --- | --- | --- | --- | --- | --- | --- | --- | --- | --- | --- | --- | --- | --- | --- | --- | --- | --- | --- | --- | --- | --- | --- | --- | --- | --- | --- | --- | --- | --- | --- | --- | --- |
|  | | ... 30 amino acid residues are skipped at the N-terminus ... | | | | | | | | | | | | | | | | | | | | | | | | | | | | | | | | | | | | | | | | | | | | | | | | | | | | | | | | | | | | | |  | | |
|  | |  | | | | | | | | | | | | | | | | | | | | | | | | | | | | | | | | | | | | | | | | | | | | | | | | | | | | | | | | | | | | | | | | | | | |
| 31 |  |  | S |  | F |  | E |  | A |  | D |  | D |  | V |  | I |  | P |  | F |  |  | L |  | A |  | R |  | E |  | Q |  | V |  | R |  | S |  | D |  | C |  |  | T |  | L |  | R |  | N |  | H |  | D |  | C |  | T |  | D |  | D |  | 60 |  |
|  | |  | | | | | | | | | | | | | | | | | | | | | | | | | | | | | | | | | | | | | | | | | | | | | | | | | | | | | | | | | | | | | | | | | | | |
| 61 |  |  | R |  | H |  | S |  | C |  | C |  | R |  | S |  | K |  | M |  | F |  |  | K |  | D |  | V |  | C |  | K |  | C |  | F |  | Y |  | P |  | S |  |  | Q |  | R |  | S |  | D |  | T |  | A |  | R | ] | A | ⎩ | K | ⎩ | K |  | 90 |  |
|  | |  | | | | | | | | | | | | | | | | | | | | | | | | | | | | | | | | | | | | | | | | | | | | | | | | | | | | | -58.01 | | | | | | | | | | | |
| 91 |  | ⎫ | E | ⎫ | L | ⎫ | C |  | T | ⎫ | C | ⎫ | Q | ⎫ | Q |  | D | ⎱ | K |  | H |  |  | L |  | K | ⎱ | Y |  | I | ⎱ | E | ⎫ | K |  | G | ⎫ | L |  | Q | ⎱ | K |  | ⎫ | A | ⎱ | K | ⎫ | V | ⎫ | L | ⎫ | V | ⎫ | A |  | G |  | | 117 |  | | | | | |

Fixed PTMs: Carbamidomethylation [C93 C95 ]   
  
     Unexpected modifications:   Unknown [-58.01]

  

All peaks (57)  Matched peaks (29)  Not matched peaks (28)

  

| Scan | Peak | Mono mass | Mono m/z | Intensity | Charge | Theoretical mass | Ion | Pos | Mass error | PPM error |
| --- | --- | --- | --- | --- | --- | --- | --- | --- | --- | --- |
| 495 | 1 | 3368.8614 | 674.7796 | 553179.73 | 5 |  |  |  |  |  |
| 495 | 2 | 3142.6978 | 786.6817 | 185614.86 | 4 | 3142.7106 | C26 | 26 | -0.0128 | -4.09 |
| 495 | 3 | 3354.8511 | 671.9775 | 160101.37 | 5 | 3354.8631 | C28 | 28 | -0.0120 | -3.58 |
| 495 | 4 | 3368.8654 | 843.2236 | 130949.87 | 4 |  |  |  |  |  |
| 495 | 5 | 2161.1046 | 721.3755 | 142285.28 | 3 | 2161.1135 | C17 | 17 | -8.94e-03 | -4.14 |
| 495 | 6 | 1142.3012 | 572.1579 | 1032501.51 | 2 |  |  |  |  |  |
| 495 | 7 | 2048.2672 | 683.7630 | 143043.65 | 3 | 2048.2688 | Z\_DOT19 | 11 | -1.68e-03 | -0.82 |
| 495 | 8 | 571.3149 | 572.3222 | 918422.13 | 1 |  |  |  |  |  |
| 495 | 9 | 3408.8683 | 569.1520 | 82634.24 | 6 |  |  |  |  |  |
| 495 | 10 | 3210.7366 | 803.6914 | 91296.07 | 4 | 3210.7435 | Z\_DOT28 | 2 | -6.93e-03 | -2.16 |
| 495 | 11 | 2475.2629 | 826.0949 | 108921.93 | 3 | 2475.2726 | C20 | 20 | -9.68e-03 | -3.91 |
| 495 | 12 | 1884.9587 | 629.3268 | 128199.22 | 3 | 1884.9662 | C15 | 15 | -7.47e-03 | -3.96 |
| 495 | 13 | 2290.1473 | 764.3897 | 96869.57 | 3 | 2290.1561 | C18 | 18 | -8.86e-03 | -3.87 |
| 495 | 14 | 3409.8691 | 682.9811 | 84864.81 | 5 |  |  |  |  |  |
| 495 | 15 | 1541.9361 | 771.9753 | 112051.96 | 2 | 1541.9360 | Z\_DOT15 | 15 | 1.19e-04 | 0.08 |
| 495 | 16 | 2915.5350 | 729.8910 | 78329.32 | 4 | 2915.5473 | C24 | 24 | -0.0123 | -4.22 |
| 495 | 17 | 3338.8300 | 668.7733 | 89191.70 | 5 | 3338.8385 | Z\_DOT29 | 1 | -8.53e-03 | -2.56 |
| 495 | 18 | 1378.6270 | 690.3208 | 105816.70 | 2 | 1378.6333 | C11 | 11 | -6.28e-03 | -4.56 |
| 495 | 19 | 2844.4983 | 712.1319 | 68055.61 | 4 | 2844.5102 | C23 | 23 | -0.0118 | -4.15 |
| 495 | 20 | 3381.8719 | 677.3817 | 56844.39 | 5 |  |  |  |  |  |
| 495 | 21 | 3338.8314 | 835.7151 | 78481.63 | 4 | 3338.8385 | Z\_DOT29 | 1 | -7.05e-03 | -2.11 |
| 495 | 22 | 2716.4051 | 906.4756 | 57179.99 | 3 | 2716.4152 | C22 | 22 | -0.0101 | -3.72 |
| 495 | 23 | 2361.3799 | 591.3523 | 59253.31 | 4 |  |  |  |  |  |
| 495 | 24 | 3382.8800 | 846.7273 | 52898.58 | 4 |  |  |  |  |  |
| 495 | 25 | 3043.6299 | 761.9148 | 56673.33 | 4 | 3043.6422 | C25 | 25 | -0.0123 | -4.04 |
| 495 | 26 | 3255.7806 | 814.9524 | 46655.30 | 4 | 3255.7947 | C27 | 27 | -0.0141 | -4.34 |
| 495 | 27 | 1265.7896 | 633.9021 | 66976.49 | 2 | 1265.7886 | Z\_DOT13 | 17 | 1.02e-03 | 0.81 |
| 495 | 28 | 3226.7549 | 807.6960 | 40723.04 | 4 |  |  |  |  |  |
| 495 | 29 | 2435.4271 | 609.8640 | 50806.10 | 4 |  |  |  |  |  |
| 495 | 30 | 3424.8891 | 685.9851 | 426232.47 | 5 |  |  |  |  |  |
| 495 | 31 | 2489.4380 | 623.3668 | 38533.52 | 4 |  |  |  |  |  |
| 495 | 32 | 3353.8472 | 839.4691 | 37679.04 | 4 |  |  |  |  |  |
| 495 | 33 | 1557.9549 | 779.9847 | 52604.37 | 2 |  |  |  |  |  |
| 495 | 34 | 3410.8764 | 853.7264 | 42683.11 | 4 |  |  |  |  |  |
| 495 | 35 | 3290.8701 | 659.1813 | 46652.21 | 5 |  |  |  |  |  |
| 495 | 36 | 2716.4041 | 680.1083 | 41095.75 | 4 | 2716.4152 | C22 | 22 | -0.0111 | -4.09 |
| 495 | 37 | 685.5788 | 686.5861 | 231111.93 | 1 |  |  |  |  |  |
| 495 | 38 | 908.5775 | 455.2961 | 53905.35 | 2 |  |  |  |  |  |
| 495 | 39 | 1007.4860 | 1008.4933 | 33254.55 | 1 | 1007.4892 | C8 | 8 | -3.16e-03 | -3.14 |
| 495 | 40 | 710.4905 | 711.4977 | 20806.30 | 1 | 710.4869 | Z\_DOT8 | 22 | 3.55e-03 | 5.00 |
| 495 | 41 | 1206.7768 | 604.3957 | 21138.60 | 2 |  |  |  |  |  |
| 495 | 42 | 1135.5442 | 1136.5515 | 15011.54 | 1 | 1135.5477 | C9 | 9 | -3.57e-03 | -3.15 |
| 495 | 43 | 847.4556 | 848.4628 | 14445.15 | 1 | 847.4585 | C7 | 7 | -2.96e-03 | -3.49 |
| 495 | 44 | 967.6509 | 484.8327 | 7173.52 | 2 |  |  |  |  |  |
| 495 | 45 | 473.2948 | 474.3021 | 13611.45 | 1 | 473.2961 | C4 | 4 | -1.27e-03 | -2.69 |
| 495 | 46 | 1007.4858 | 504.7502 | 9546.99 | 2 | 1007.4892 | C8 | 8 | -3.42e-03 | -3.39 |
| 495 | 47 | 780.5193 | 391.2669 | 7248.92 | 2 |  |  |  |  |  |
| 495 | 48 | 873.4712 | 874.4785 | 9424.76 | 1 |  |  |  |  |  |
| 495 | 49 | 511.3593 | 512.3666 | 11366.17 | 1 | 511.3548 | Z\_DOT6 | 24 | 4.46e-03 | 8.72 |
| 495 | 50 | 1078.6827 | 540.3486 | 6895.65 | 2 |  |  |  |  |  |
| 495 | 51 | 586.3782 | 587.3855 | 9975.86 | 1 | 586.3802 | C5 | 5 | -1.96e-03 | -3.34 |
| 495 | 52 | 726.5091 | 727.5164 | 6093.86 | 1 |  |  |  |  |  |
| 495 | 53 | 344.2527 | 345.2600 | 7169.43 | 1 | 344.2535 | C3 | 3 | -7.97e-04 | -2.31 |
| 495 | 54 | 1266.7981 | 1267.8054 | 3518.30 | 1 |  |  |  |  |  |
| 495 | 55 | 1378.6291 | 1379.6364 | 3227.53 | 1 | 1378.6333 | C11 | 11 | -4.13e-03 | -3.00 |
| 495 | 56 | 1024.6719 | 513.3432 | 4497.78 | 2 |  |  |  |  |  |
| 495 | 57 | 891.9619 | 892.9692 | 4925.41 | 1 |  |  |  |  |  |

  

All proteins /
CsTx-12b Cupiennius salei toxin 12 isoform b /
Proteoform #47
