# Supplementary material for: The Dual Prey-Inactivation Strategy of Spiders—In-Depth Venomic Analysis of Cupiennius salei
Source: Toxins (Basel). 2019 Mar 19;11(3):167. doi: 10.3390/toxins11030167 (PMC6468893; doi:10.3390/toxins11030167)
Supplement: Supplementary file 1 [file toxins-11-00167-s001.zip › Supplementary Dataset EV1/20180328_f2_topdown_OTMS2_EThcD_NL_i02_ms2_proteoform_cutoff_html/prsms/prsm133.html]

Protein-Spectrum-Match for Spectrum #370


All proteins /
CsTx-1a\_S1 Cupiennius salei toxin 1 isoform a S1^ACsTx-1a\_S2 Cupiennius salei toxin 1 isoform a S2 /
Proteoform #8

## Protein-Spectrum-Match #133 for Spectrum #370

|  |  |  |  |  |  |
| --- | --- | --- | --- | --- | --- |
| PrSM ID: | 133 | Scan(s): | 496 | Precursor charge: | 7 |
| Precursor m/z: | 1025.3197 | Precursor mass: | 7170.1868 | Proteoform mass: | 7169.1856 |
| # matched peaks: | 48 | # matched fragment ions: | 46 | # unexpected modifications: | 0 |
| E-value: | 7.86e-41 | P-value: | 7.86e-41 | Q-value (Spectral FDR): | 0 |

  

|  |  |  |  |  |  |  |  |  |  |  |  |  |  |  |  |  |  |  |  |  |  |  |  |  |  |  |  |  |  |  |  |  |  |  |  |  |  |  |  |  |  |  |  |  |  |  |  |  |  |  |  |  |  |  |  |  |  |  |  |  |  |  |  |  |  |  |  |  |  |
| --- | --- | --- | --- | --- | --- | --- | --- | --- | --- | --- | --- | --- | --- | --- | --- | --- | --- | --- | --- | --- | --- | --- | --- | --- | --- | --- | --- | --- | --- | --- | --- | --- | --- | --- | --- | --- | --- | --- | --- | --- | --- | --- | --- | --- | --- | --- | --- | --- | --- | --- | --- | --- | --- | --- | --- | --- | --- | --- | --- | --- | --- | --- | --- | --- | --- | --- | --- | --- | --- |
|  | |  | | | | | | | | | | | | | | | | | | | | | | | | | | | | | | | | | | | | | | | | | | | | | | | | | | | | | | | | | | | | | | | | | | | |
| 1 |  |  | M |  | K |  | V |  | L |  | I |  | I |  | S |  | A |  | V |  | L |  |  | F |  | I |  | T |  | I |  | F |  | S |  | N |  | I |  | S |  | A |  |  | E |  | I |  | E |  | D |  | D |  | F |  | L |  | E |  | D |  | E |  | 30 |  |
|  | |  | | | | | | | | | | | | | | | | | | | | | | | | | | | | | | | | | | | | | | | | | | | | | | | | | | | | | | | | | | | | | | | | | | | |
| 31 |  |  | S |  | F |  | E |  | A |  | E |  | D |  | I |  | I |  | P |  | F |  |  | F |  | E |  | N |  | E |  | Q |  | A |  | R | ] | S | ⎩ | C | ⎩ | I |  | ⎩ | P | ⎱ | K | ⎱ | H | ⎱ | E | ⎱ | E | ⎫ | C | ⎱ | T | ⎩ | N | ⎱ | D | ⎩ | K |  | 60 |  |
|  | |  | | | | | | | | | | | | | | | | | | | | | | | | | | | | | | | | | | | | | | | | | | | | | | | | | | | | | | | | | | | | | | | | | | | |
| 61 |  |  | H | ⎱ | N | ⎫ | C |  | C |  | R |  | K | ⎫ | G |  | L |  | F |  | K |  | ⎫ | L |  | K | ⎫ | C | ⎫ | Q | ⎫ | C |  | S |  | T |  | F | ⎫ | D | ⎫ | D |  | ⎱ | E |  | S |  | G | ⎱ | Q |  | P |  | T |  | E | ⎱ | R |  | C |  | A |  | 90 |  |
|  | |  | | | | | | | | | | | | | | | | | | | | | | | | | | | | | | | | | | | | | | | | | | | | | | | | | | | | | | | | | | | | | | | | | | | |
| 91 |  | ⎫ | C |  | G | ⎱ | R |  | P | ⎫ | M | ⎱ | G | ⎫ | H |  | Q | ⎫ | A |  | I |  |  | E | ⎫ | T |  | G |  | L | ⎫ | N | ⎫ | I | ⎫ | F | [ | R |  | G |  | L |  |  | F |  | K |  | G |  | K |  | K |  | K |  | N |  | K |  | K |  | T |  | 120 |  |
|  | |  | | | | | | | | | | | | | | | | | | | | | | | | | | | | | | | | | | | | | | | | | | | | | | | | | | | | | | | | | | | | | | | | | | | |
| 121 |  |  | K |  | G |  | | | | 122 |  | | | | | | | | | | | | | | | | | | | | | | | | | | | | | | | | | | | | | | | | | | | | | | | | | | | | | | | |

Fixed PTMs: Carbamidomethylation [C49 C56 C63 C64 C73 C75 C89 C91 ]

  

All peaks (111)  Matched peaks (48)  Not matched peaks (63)

  

| Scan | Peak | Mono mass | Mono m/z | Intensity | Charge | Theoretical mass | Ion | Pos | Mass error | PPM error |
| --- | --- | --- | --- | --- | --- | --- | --- | --- | --- | --- |
| 496 | 1 | 7112.1182 | 1186.3603 | 216699.77 | 6 |  |  |  |  |  |
| 496 | 2 | 3585.0709 | 1196.0309 | 145471.67 | 3 |  |  |  |  |  |
| 496 | 3 | 7125.1304 | 1188.5290 | 43081.39 | 6 |  |  |  |  |  |
| 496 | 4 | 7167.1474 | 1024.8855 | 593699.13 | 7 |  |  |  |  |  |
| 496 | 5 | 7153.1287 | 1193.1954 | 26602.76 | 6 |  |  |  |  |  |
| 496 | 6 | 7113.1201 | 1423.6313 | 23398.43 | 5 |  |  |  |  |  |
| 496 | 7 | 7080.1421 | 1181.0310 | 17160.45 | 6 |  |  |  |  |  |
| 496 | 8 | 3157.4976 | 1053.5065 | 19134.57 | 3 | 3157.5153 | C25 | 25 | -0.0178 | -5.63 |
| 496 | 9 | 7021.0979 | 1171.1903 | 15001.58 | 6 | 7021.1332 | C59 | 59 | -0.0352 | -5.02 |
| 496 | 10 | 6976.0884 | 1163.6887 | 15195.23 | 6 |  |  |  |  |  |
| 496 | 11 | 7096.0987 | 1183.6904 | 12252.18 | 6 |  |  |  |  |  |
| 496 | 12 | 2782.3042 | 928.4420 | 15540.74 | 3 |  |  |  |  |  |
| 496 | 13 | 1752.7563 | 877.3854 | 15138.80 | 2 | 1752.7671 | C14 | 14 | -0.0109 | -6.20 |
| 496 | 14 | 6209.6452 | 1242.9363 | 14192.87 | 5 | 6209.6892 | C51 | 51 | -0.0440 | -7.09 |
| 496 | 15 | 4443.9045 | 1111.9834 | 10712.25 | 4 | 4443.9333 | C36 | 36 | -0.0288 | -6.49 |
| 496 | 16 | 5887.5133 | 1178.5099 | 13620.03 | 5 | 5887.5503 | C48 | 48 | -0.0370 | -6.28 |
| 496 | 17 | 5944.5238 | 1189.9120 | 10895.44 | 5 | 5944.5717 | C49 | 49 | -0.0479 | -8.06 |
| 496 | 18 | 7034.1233 | 1173.3612 | 9352.89 | 6 |  |  |  |  |  |
| 496 | 19 | 6081.5684 | 1217.3210 | 14919.67 | 5 |  |  |  |  |  |
| 496 | 20 | 2872.3066 | 958.4428 | 10299.79 | 3 |  |  |  |  |  |
| 496 | 21 | 6301.7326 | 1261.3538 | 7961.61 | 5 | 6301.7710 | Z\_DOT53 | 7 | -0.0384 | -6.10 |
| 496 | 22 | 868.4178 | 869.4250 | 16376.13 | 1 | 868.4225 | C7 | 7 | -4.69e-03 | -5.40 |
| 496 | 23 | 5503.3202 | 1101.6713 | 6111.23 | 5 | 5503.3559 | C45 | 45 | -0.0357 | -6.48 |
| 496 | 24 | 7126.1346 | 1426.2342 | 6247.04 | 5 |  |  |  |  |  |
| 496 | 25 | 1866.7986 | 934.4066 | 9655.19 | 2 | 1866.8101 | C15 | 15 | -0.0115 | -6.16 |
| 496 | 26 | 7054.0921 | 1176.6893 | 7309.34 | 6 |  |  |  |  |  |
| 496 | 27 | 7055.1007 | 1412.0274 | 5524.57 | 5 |  |  |  |  |  |
| 496 | 28 | 1195.5226 | 1196.5299 | 67407.14 | 1 |  |  |  |  |  |
| 496 | 29 | 6567.8266 | 1314.5726 | 5296.42 | 5 | 6567.8725 | Z\_DOT55 | 5 | -0.0459 | -6.99 |
| 496 | 30 | 3922.6514 | 1308.5577 | 5649.12 | 3 |  |  |  |  |  |
| 496 | 31 | 7154.1479 | 1431.8369 | 5445.88 | 5 |  |  |  |  |  |
| 496 | 32 | 3445.5830 | 1149.5349 | 7262.69 | 3 | 3445.6046 | C27 | 27 | -0.0216 | -6.27 |
| 496 | 33 | 5756.4646 | 1152.3002 | 5636.93 | 5 | 5756.5098 | C47 | 47 | -0.0452 | -7.85 |
| 496 | 34 | 602.3179 | 603.3251 | 12379.48 | 1 | 602.3210 | C5 | 5 | -3.11e-03 | -5.16 |
| 496 | 35 | 3317.5237 | 1106.8485 | 4315.14 | 3 | 3317.5460 | C26 | 26 | -0.0223 | -6.73 |
| 496 | 36 | 6430.7774 | 1287.1628 | 4781.02 | 5 | 6430.8136 | Z\_DOT54 | 6 | -0.0362 | -5.63 |
| 496 | 37 | 7003.0636 | 1001.4449 | 5665.96 | 7 |  |  |  |  |  |
| 496 | 38 | 3183.5113 | 1062.1777 | 5061.65 | 3 |  |  |  |  |  |
| 496 | 39 | 3634.5617 | 1212.5278 | 4134.76 | 3 |  |  |  |  |  |
| 496 | 40 | 739.3760 | 740.3832 | 8289.48 | 1 | 739.3799 | C6 | 6 | -3.91e-03 | -5.29 |
| 496 | 41 | 6583.8467 | 1317.7766 | 3207.30 | 5 |  |  |  |  |  |
| 496 | 42 | 4269.8471 | 1424.2896 | 4787.39 | 3 |  |  |  |  |  |
| 496 | 43 | 2916.3215 | 973.1144 | 5770.42 | 3 | 2916.3363 | C23 | 23 | -0.0148 | -5.09 |
| 496 | 44 | 6243.7101 | 1249.7493 | 4193.12 | 5 |  |  |  |  |  |
| 496 | 45 | 997.4604 | 998.4677 | 4644.95 | 1 | 997.4651 | C8 | 8 | -4.68e-03 | -4.69 |
| 496 | 46 | 7153.1235 | 1022.8821 | 5905.88 | 7 |  |  |  |  |  |
| 496 | 47 | 6792.9584 | 1133.1670 | 4516.06 | 6 | 6793.0202 | Z\_DOT57 | 3 | -0.0618 | -9.10 |
| 496 | 47 | 6792.9584 | 1133.1670 | 4516.06 | 6 | 6794.0062 | C57 | 57 | -0.0454 | -6.68 |
| 496 | 48 | 2743.2026 | 915.4081 | 2600.19 | 3 |  |  |  |  |  |
| 496 | 49 | 2025.8299 | 1013.9222 | 3740.55 | 2 |  |  |  |  |  |
| 496 | 50 | 7095.1166 | 1420.0306 | 2447.69 | 5 |  |  |  |  |  |
| 496 | 51 | 7065.1000 | 1178.5239 | 8828.42 | 6 | 7066.1350 | Z\_DOT59 | 1 | -0.0326 | -4.61 |
| 496 | 52 | 5213.3458 | 1304.3437 | 3220.38 | 4 |  |  |  |  |  |
| 496 | 53 | 3471.5953 | 1158.2057 | 2146.08 | 3 |  |  |  |  |  |
| 496 | 54 | 6695.9180 | 1340.1909 | 3951.17 | 5 | 6695.9675 | Z\_DOT56 | 4 | -0.0494 | -7.38 |
| 496 | 55 | 6523.8069 | 1305.7686 | 2915.33 | 5 | 6522.8530 | C54 | 54 | -0.0485 | -7.43 |
| 496 | 56 | 6977.0996 | 1396.4272 | 2854.78 | 5 |  |  |  |  |  |
| 496 | 57 | 5417.3989 | 1355.3570 | 3394.25 | 4 | 5417.4264 | Z\_DOT46 | 14 | -0.0275 | -5.08 |
| 496 | 58 | 6012.6622 | 1203.5397 | 2642.96 | 5 | 6012.6978 | Z\_DOT51 | 9 | -0.0356 | -5.91 |
| 496 | 59 | 7068.1160 | 1414.6305 | 3291.19 | 5 |  |  |  |  |  |
| 496 | 60 | 3868.6411 | 1290.5543 | 2330.78 | 3 |  |  |  |  |  |
| 496 | 61 | 6027.6724 | 1206.5418 | 2219.90 | 5 |  |  |  |  |  |
| 496 | 62 | 5796.5659 | 1160.3205 | 2157.50 | 5 | 5797.6072 | Z\_DOT49 | 11 | -0.0389 | -6.71 |
| 496 | 63 | 2725.2419 | 909.4212 | 2096.20 | 3 |  |  |  |  |  |
| 496 | 64 | 2726.2451 | 1364.1298 | 1962.50 | 2 | 2726.2602 | Z\_DOT24 | 36 | -0.0151 | -5.54 |
| 496 | 65 | 2271.0468 | 1136.5307 | 1732.54 | 2 | 2271.0586 | Z\_DOT20 | 40 | -0.0117 | -5.16 |
| 496 | 66 | 3606.6096 | 1203.2105 | 1864.31 | 3 |  |  |  |  |  |
| 496 | 67 | 1666.8262 | 834.4204 | 1726.49 | 2 | 1666.8376 | Z\_DOT15 | 45 | -0.0114 | -6.82 |
| 496 | 68 | 6479.8095 | 1296.9692 | 1499.31 | 5 |  |  |  |  |  |
| 496 | 69 | 6189.7073 | 1238.9487 | 2334.43 | 5 |  |  |  |  |  |
| 496 | 70 | 2471.0563 | 1236.5354 | 1739.43 | 2 | 2471.0674 | C19 | 19 | -0.0111 | -4.49 |
| 496 | 71 | 7063.1101 | 1010.0230 | 2158.21 | 7 |  |  |  |  |  |
| 496 | 72 | 3940.7543 | 986.1959 | 2165.39 | 4 | 3940.7834 | C31 | 31 | -0.0290 | -7.37 |
| 496 | 73 | 3283.5008 | 1095.5075 | 1523.71 | 3 |  |  |  |  |  |
| 496 | 74 | 6907.9981 | 1152.3403 | 4064.44 | 6 | 6908.0491 | C58 | 58 | -0.0510 | -7.38 |
| 496 | 75 | 4170.8131 | 1043.7106 | 2331.63 | 4 | 4170.8372 | C33 | 33 | -0.0241 | -5.78 |
| 496 | 76 | 4899.1041 | 1225.7833 | 2950.46 | 4 | 4899.1349 | C40 | 40 | -0.0309 | -6.30 |
| 496 | 77 | 4055.7794 | 1014.9521 | 2025.29 | 4 | 4055.8103 | C32 | 32 | -0.0309 | -7.63 |
| 496 | 78 | 1793.8746 | 897.9446 | 1741.40 | 2 |  |  |  |  |  |
| 496 | 79 | 6906.0655 | 1382.2204 | 2156.17 | 5 | 6906.1043 | Z\_DOT58 | 2 | -0.0388 | -5.61 |
| 496 | 80 | 7126.1249 | 1019.0251 | 3056.79 | 7 |  |  |  |  |  |
| 496 | 81 | 5708.5857 | 1142.7244 | 1191.04 | 5 |  |  |  |  |  |
| 496 | 82 | 2999.3393 | 1000.7870 | 1763.88 | 3 | 2999.3562 | Z\_DOT27 | 33 | -0.0170 | -5.65 |
| 496 | 83 | 1434.4337 | 1435.4410 | 4217.94 | 1 |  |  |  |  |  |
| 496 | 84 | 5287.2773 | 1322.8266 | 1411.10 | 4 | 5286.3038 | C43 | 43 | -0.0288 | -5.46 |
| 496 | 85 | 4641.0705 | 1161.2749 | 1320.11 | 4 |  |  |  |  |  |
| 496 | 86 | 5910.6082 | 1183.1289 | 2672.07 | 5 | 5911.6501 | Z\_DOT50 | 10 | -0.0395 | -6.68 |
| 496 | 87 | 474.2237 | 475.2310 | 2475.25 | 1 | 474.2260 | C4 | 4 | -2.26e-03 | -4.77 |
| 496 | 88 | 1282.6326 | 1283.6398 | 1315.55 | 1 | 1282.6432 | Z\_DOT12 | 48 | -0.0107 | -8.31 |
| 496 | 89 | 7077.0908 | 1012.0202 | 2396.43 | 7 |  |  |  |  |  |
| 496 | 90 | 7114.1355 | 1779.5412 | 1900.96 | 4 |  |  |  |  |  |
| 496 | 91 | 2178.1189 | 1090.0667 | 1210.35 | 2 |  |  |  |  |  |
| 496 | 92 | 4170.8036 | 1391.2751 | 786.07 | 3 | 4170.8372 | C33 | 33 | -0.0337 | -8.08 |
| 496 | 93 | 4300.8458 | 1076.2187 | 1061.73 | 4 |  |  |  |  |  |
| 496 | 94 | 663.3553 | 664.3625 | 1137.62 | 1 |  |  |  |  |  |
| 496 | 95 | 5052.3352 | 1264.0911 | 1734.39 | 4 |  |  |  |  |  |
| 496 | 96 | 3501.5180 | 1168.1799 | 1610.48 | 3 |  |  |  |  |  |
| 496 | 97 | 2667.2092 | 890.0770 | 1005.53 | 3 |  |  |  |  |  |
| 496 | 98 | 1157.4917 | 1158.4990 | 1519.34 | 1 | 1157.4957 | C9 | 9 | -4.02e-03 | -3.48 |
| 496 | 99 | 5683.5407 | 1137.7154 | 1191.89 | 5 | 5682.5802 | Z\_DOT48 | 12 | -0.0419 | -7.37 |
| 496 | 100 | 1130.8279 | 1131.8351 | 654.11 | 1 |  |  |  |  |  |
| 496 | 101 | 5888.5125 | 1473.1354 | 1143.65 | 4 | 5887.5503 | C48 | 48 | -0.0401 | -6.81 |
| 496 | 102 | 3604.6054 | 902.1586 | 801.33 | 4 |  |  |  |  |  |
| 496 | 103 | 5813.5752 | 1163.7223 | 1525.04 | 5 |  |  |  |  |  |
| 496 | 104 | 1038.1218 | 1039.1291 | 542.05 | 1 |  |  |  |  |  |
| 496 | 105 | 1394.6144 | 1395.6216 | 605.55 | 1 |  |  |  |  |  |
| 496 | 106 | 795.4211 | 796.4283 | 463.15 | 1 |  |  |  |  |  |
| 496 | 107 | 4443.9253 | 1482.3157 | 1316.87 | 3 | 4443.9333 | C36 | 36 | -7.99e-03 | -1.80 |
| 496 | 108 | 1372.5772 | 687.2959 | 413.83 | 2 | 1372.5863 | C11 | 11 | -9.13e-03 | -6.65 |
| 496 | 109 | 1264.1526 | 1265.1598 | 426.04 | 1 |  |  |  |  |  |
| 496 | 110 | 1112.9824 | 1113.9897 | 581.57 | 1 |  |  |  |  |  |
| 496 | 111 | 4671.0290 | 1168.7645 | 1488.89 | 4 |  |  |  |  |  |

  

All proteins /
CsTx-1a\_S1 Cupiennius salei toxin 1 isoform a S1^ACsTx-1a\_S2 Cupiennius salei toxin 1 isoform a S2 /
Proteoform #8
