# Supplementary material for: The Dual Prey-Inactivation Strategy of Spiders—In-Depth Venomic Analysis of Cupiennius salei
Source: Toxins (Basel). 2019 Mar 19;11(3):167. doi: 10.3390/toxins11030167 (PMC6468893; doi:10.3390/toxins11030167)
Supplement: Supplementary file 1 [file toxins-11-00167-s001.zip › Supplementary Dataset EV1/20180328_f2_topdown_OTMS2_EThcD_NL_i02_ms2_proteoform_cutoff_html/prsms/prsm134.html]

Protein-Spectrum-Match for Spectrum #371


All proteins /
CsTx-1a\_S1 Cupiennius salei toxin 1 isoform a S1^ACsTx-1a\_S2 Cupiennius salei toxin 1 isoform a S2 /
Proteoform #8

## Protein-Spectrum-Match #134 for Spectrum #371

|  |  |  |  |  |  |
| --- | --- | --- | --- | --- | --- |
| PrSM ID: | 134 | Scan(s): | 497 | Precursor charge: | 6 |
| Precursor m/z: | 1195.8717 | Precursor mass: | 7169.1863 | Proteoform mass: | 7169.1856 |
| # matched peaks: | 22 | # matched fragment ions: | 22 | # unexpected modifications: | 0 |
| E-value: | 4.15e-20 | P-value: | 4.15e-20 | Q-value (Spectral FDR): | 0 |

  

|  |  |  |  |  |  |  |  |  |  |  |  |  |  |  |  |  |  |  |  |  |  |  |  |  |  |  |  |  |  |  |  |  |  |  |  |  |  |  |  |  |  |  |  |  |  |  |  |  |  |  |  |  |  |  |  |  |  |  |  |  |  |  |  |  |  |  |  |  |  |
| --- | --- | --- | --- | --- | --- | --- | --- | --- | --- | --- | --- | --- | --- | --- | --- | --- | --- | --- | --- | --- | --- | --- | --- | --- | --- | --- | --- | --- | --- | --- | --- | --- | --- | --- | --- | --- | --- | --- | --- | --- | --- | --- | --- | --- | --- | --- | --- | --- | --- | --- | --- | --- | --- | --- | --- | --- | --- | --- | --- | --- | --- | --- | --- | --- | --- | --- | --- | --- | --- |
|  | |  | | | | | | | | | | | | | | | | | | | | | | | | | | | | | | | | | | | | | | | | | | | | | | | | | | | | | | | | | | | | | | | | | | | |
| 1 |  |  | M |  | K |  | V |  | L |  | I |  | I |  | S |  | A |  | V |  | L |  |  | F |  | I |  | T |  | I |  | F |  | S |  | N |  | I |  | S |  | A |  |  | E |  | I |  | E |  | D |  | D |  | F |  | L |  | E |  | D |  | E |  | 30 |  |
|  | |  | | | | | | | | | | | | | | | | | | | | | | | | | | | | | | | | | | | | | | | | | | | | | | | | | | | | | | | | | | | | | | | | | | | |
| 31 |  |  | S |  | F |  | E |  | A |  | E |  | D |  | I |  | I |  | P |  | F |  |  | F |  | E |  | N |  | E |  | Q |  | A |  | R | ] | S |  | C |  | I |  |  | P |  | K | ⎫ | H | ⎫ | E | ⎫ | E | ⎫ | C |  | T |  | N |  | D |  | K |  | 60 |  |
|  | |  | | | | | | | | | | | | | | | | | | | | | | | | | | | | | | | | | | | | | | | | | | | | | | | | | | | | | | | | | | | | | | | | | | | |
| 61 |  |  | H | ⎫ | N | ⎫ | C |  | C |  | R |  | K |  | G |  | L |  | F | ⎫ | K |  | ⎫ | L |  | K | ⎫ | C | ⎫ | Q | ⎫ | C |  | S | ⎫ | T | ⎫ | F |  | D | ⎫ | D |  |  | E |  | S |  | G | ⎩ | Q |  | P |  | T |  | E | ⎩ | R |  | C |  | A |  | 90 |  |
|  | |  | | | | | | | | | | | | | | | | | | | | | | | | | | | | | | | | | | | | | | | | | | | | | | | | | | | | | | | | | | | | | | | | | | | |
| 91 |  | ⎫ | C |  | G | ⎫ | R |  | P |  | M |  | G | ⎩ | H | ⎫ | Q |  | A |  | I |  |  | E |  | T |  | G |  | L | ⎫ | N |  | I | ⎫ | F | [ | R |  | G |  | L |  |  | F |  | K |  | G |  | K |  | K |  | K |  | N |  | K |  | K |  | T |  | 120 |  |
|  | |  | | | | | | | | | | | | | | | | | | | | | | | | | | | | | | | | | | | | | | | | | | | | | | | | | | | | | | | | | | | | | | | | | | | |
| 121 |  |  | K |  | G |  | | | | 122 |  | | | | | | | | | | | | | | | | | | | | | | | | | | | | | | | | | | | | | | | | | | | | | | | | | | | | | | | |

Fixed PTMs: Carbamidomethylation [C49 C56 C63 C64 C73 C75 C89 C91 ]

  

All peaks (43)  Matched peaks (22)  Not matched peaks (21)

  

| Scan | Peak | Mono mass | Mono m/z | Intensity | Charge | Theoretical mass | Ion | Pos | Mass error | PPM error |
| --- | --- | --- | --- | --- | --- | --- | --- | --- | --- | --- |
| 497 | 1 | 3585.0683 | 1196.0300 | 188943.12 | 3 |  |  |  |  |  |
| 497 | 2 | 7112.1173 | 1423.4307 | 58664.16 | 5 |  |  |  |  |  |
| 497 | 3 | 2390.3817 | 1196.1981 | 97393.81 | 2 |  |  |  |  |  |
| 497 | 4 | 7125.1257 | 1426.0324 | 11066.26 | 5 |  |  |  |  |  |
| 497 | 5 | 1434.4303 | 1435.4376 | 21931.03 | 1 |  |  |  |  |  |
| 497 | 6 | 6977.0864 | 1396.4245 | 6297.92 | 5 |  |  |  |  |  |
| 497 | 7 | 1752.7555 | 877.3850 | 10147.34 | 2 | 1752.7671 | C14 | 14 | -0.0116 | -6.62 |
| 497 | 8 | 1195.5244 | 1196.5317 | 103991.94 | 1 |  |  |  |  |  |
| 497 | 9 | 7152.1342 | 1431.4341 | 4701.70 | 5 |  |  |  |  |  |
| 497 | 10 | 7020.0908 | 1405.0254 | 4210.01 | 5 | 7021.1332 | C59 | 59 | -0.0400 | -5.70 |
| 497 | 11 | 1866.7992 | 934.4069 | 8009.84 | 2 | 1866.8101 | C15 | 15 | -0.0109 | -5.84 |
| 497 | 12 | 7034.1197 | 1407.8312 | 3505.63 | 5 |  |  |  |  |  |
| 497 | 13 | 602.3177 | 603.3250 | 8691.99 | 1 | 602.3210 | C5 | 5 | -3.23e-03 | -5.36 |
| 497 | 14 | 7080.1445 | 1417.0362 | 4529.34 | 5 |  |  |  |  |  |
| 497 | 15 | 739.3757 | 740.3829 | 5383.81 | 1 | 739.3799 | C6 | 6 | -4.22e-03 | -5.70 |
| 497 | 16 | 997.4592 | 998.4665 | 2840.30 | 1 | 997.4651 | C8 | 8 | -5.84e-03 | -5.85 |
| 497 | 17 | 868.4173 | 869.4246 | 4462.51 | 1 | 868.4225 | C7 | 7 | -5.18e-03 | -5.96 |
| 497 | 18 | 3317.5273 | 1106.8497 | 2078.91 | 3 | 3317.5460 | C26 | 26 | -0.0187 | -5.62 |
| 497 | 19 | 2726.2414 | 1364.1280 | 1877.59 | 2 | 2726.2602 | Z\_DOT24 | 36 | -0.0188 | -6.88 |
| 497 | 20 | 2916.3198 | 973.1139 | 1923.44 | 3 | 2916.3363 | C23 | 23 | -0.0165 | -5.66 |
| 497 | 21 | 3445.5800 | 1149.5339 | 2386.60 | 3 | 3445.6046 | C27 | 27 | -0.0246 | -7.13 |
| 497 | 22 | 2872.3062 | 958.4427 | 1377.95 | 3 |  |  |  |  |  |
| 497 | 23 | 6081.6108 | 1521.4100 | 1710.19 | 4 | 6081.6306 | C50 | 50 | -0.0198 | -3.26 |
| 497 | 24 | 6793.9534 | 1359.7980 | 1735.55 | 5 | 6794.0062 | C57 | 57 | -0.0527 | -7.76 |
| 497 | 25 | 3692.6442 | 1231.8887 | 1672.81 | 3 | 3692.6673 | C29 | 29 | -0.0230 | -6.24 |
| 497 | 26 | 3157.4954 | 1053.5057 | 2560.36 | 3 | 3157.5153 | C25 | 25 | -0.0200 | -6.32 |
| 497 | 27 | 2025.8279 | 1013.9212 | 1208.90 | 2 |  |  |  |  |  |
| 497 | 28 | 7064.1112 | 1413.8295 | 2310.94 | 5 |  |  |  |  |  |
| 497 | 29 | 7095.0825 | 1420.0238 | 2088.03 | 5 |  |  |  |  |  |
| 497 | 30 | 2271.0437 | 1136.5291 | 1431.97 | 2 | 2271.0586 | Z\_DOT20 | 40 | -0.0149 | -6.56 |
| 497 | 31 | 5503.3170 | 1376.8365 | 1430.85 | 4 | 5503.3559 | C45 | 45 | -0.0389 | -7.07 |
| 497 | 32 | 1225.6157 | 1226.6230 | 1477.16 | 1 | 1225.6217 | Z\_DOT11 | 49 | -6.04e-03 | -4.93 |
| 497 | 33 | 360.1450 | 361.1523 | 1374.43 | 1 |  |  |  |  |  |
| 497 | 34 | 3793.6847 | 1265.5689 | 653.28 | 3 | 3793.7149 | C30 | 30 | -0.0302 | -7.96 |
| 497 | 35 | 3104.8189 | 1553.4167 | 1172.72 | 2 |  |  |  |  |  |
| 497 | 36 | 2787.2237 | 1394.6191 | 779.45 | 2 |  |  |  |  |  |
| 497 | 37 | 7126.1437 | 1188.6979 | 1059.63 | 6 |  |  |  |  |  |
| 497 | 38 | 4444.9057 | 1482.6425 | 1015.99 | 3 |  |  |  |  |  |
| 497 | 39 | 4055.7804 | 1352.9341 | 959.47 | 3 | 4055.8103 | C32 | 32 | -0.0299 | -7.38 |
| 497 | 40 | 2788.2172 | 930.4130 | 985.70 | 3 | 2788.2414 | C22 | 22 | -0.0241 | -8.66 |
| 497 | 41 | 663.3556 | 664.3629 | 603.82 | 1 |  |  |  |  |  |
| 497 | 42 | 1481.3021 | 1482.3094 | 379.69 | 1 |  |  |  |  |  |
| 497 | 43 | 5286.2623 | 1322.5728 | 1318.57 | 4 | 5286.3038 | C43 | 43 | -0.0415 | -7.86 |

  

All proteins /
CsTx-1a\_S1 Cupiennius salei toxin 1 isoform a S1^ACsTx-1a\_S2 Cupiennius salei toxin 1 isoform a S2 /
Proteoform #8
