# Supplementary material for: The Dual Prey-Inactivation Strategy of Spiders—In-Depth Venomic Analysis of Cupiennius salei
Source: Toxins (Basel). 2019 Mar 19;11(3):167. doi: 10.3390/toxins11030167 (PMC6468893; doi:10.3390/toxins11030167)
Supplement: Supplementary file 1 [file toxins-11-00167-s001.zip › Supplementary Dataset EV1/20180328_f2_topdown_OTMS2_EThcD_NL_i02_ms2_proteoform_cutoff_html/prsms/prsm135.html]

Protein-Spectrum-Match for Spectrum #372


All proteins /
CsTx-1a\_S1 Cupiennius salei toxin 1 isoform a S1^ACsTx-1a\_S2 Cupiennius salei toxin 1 isoform a S2 /
Proteoform #8

## Protein-Spectrum-Match #135 for Spectrum #372

|  |  |  |  |  |  |
| --- | --- | --- | --- | --- | --- |
| PrSM ID: | 135 | Scan(s): | 499 | Precursor charge: | 8 |
| Precursor m/z: | 897.1558 | Precursor mass: | 7169.1883 | Proteoform mass: | 7169.1856 |
| # matched peaks: | 51 | # matched fragment ions: | 42 | # unexpected modifications: | 0 |
| E-value: | 1.87e-38 | P-value: | 1.87e-38 | Q-value (Spectral FDR): | 0 |

  

|  |  |  |  |  |  |  |  |  |  |  |  |  |  |  |  |  |  |  |  |  |  |  |  |  |  |  |  |  |  |  |  |  |  |  |  |  |  |  |  |  |  |  |  |  |  |  |  |  |  |  |  |  |  |  |  |  |  |  |  |  |  |  |  |  |  |  |  |  |  |
| --- | --- | --- | --- | --- | --- | --- | --- | --- | --- | --- | --- | --- | --- | --- | --- | --- | --- | --- | --- | --- | --- | --- | --- | --- | --- | --- | --- | --- | --- | --- | --- | --- | --- | --- | --- | --- | --- | --- | --- | --- | --- | --- | --- | --- | --- | --- | --- | --- | --- | --- | --- | --- | --- | --- | --- | --- | --- | --- | --- | --- | --- | --- | --- | --- | --- | --- | --- | --- | --- |
|  | |  | | | | | | | | | | | | | | | | | | | | | | | | | | | | | | | | | | | | | | | | | | | | | | | | | | | | | | | | | | | | | | | | | | | |
| 1 |  |  | M |  | K |  | V |  | L |  | I |  | I |  | S |  | A |  | V |  | L |  |  | F |  | I |  | T |  | I |  | F |  | S |  | N |  | I |  | S |  | A |  |  | E |  | I |  | E |  | D |  | D |  | F |  | L |  | E |  | D |  | E |  | 30 |  |
|  | |  | | | | | | | | | | | | | | | | | | | | | | | | | | | | | | | | | | | | | | | | | | | | | | | | | | | | | | | | | | | | | | | | | | | |
| 31 |  |  | S |  | F |  | E |  | A |  | E |  | D |  | I |  | I |  | P |  | F |  |  | F |  | E |  | N |  | E |  | Q |  | A |  | R | ] | S | ⎩ | C |  | I |  |  | P |  | K | ⎫ | H | ⎫ | E | ⎱ | E | ⎫ | C |  | T | ⎫ | N | ⎱ | D |  | K |  | 60 |  |
|  | |  | | | | | | | | | | | | | | | | | | | | | | | | | | | | | | | | | | | | | | | | | | | | | | | | | | | | | | | | | | | | | | | | | | | |
| 61 |  |  | H | ⎱ | N | ⎫ | C |  | C |  | R |  | K | ⎫ | G | ⎱ | L | ⎱ | F | ⎱ | K |  | ⎫ | L |  | K | ⎫ | C | ⎫ | Q | ⎫ | C |  | S | ⎫ | T |  | F | ⎱ | D | ⎱ | D |  | ⎫ | E | ⎫ | S |  | G | ⎱ | Q |  | P |  | T |  | E | ⎫ | R |  | C |  | A |  | 90 |  |
|  | |  | | | | | | | | | | | | | | | | | | | | | | | | | | | | | | | | | | | | | | | | | | | | | | | | | | | | | | | | | | | | | | | | | | | |
| 91 |  |  | C | ⎫ | G | ⎫ | R |  | P | ⎫ | M | ⎫ | G | ⎫ | H | ⎫ | Q | ⎱ | A |  | I |  |  | E |  | T |  | G |  | L |  | N |  | I | ⎫ | F | [ | R |  | G |  | L |  |  | F |  | K |  | G |  | K |  | K |  | K |  | N |  | K |  | K |  | T |  | 120 |  |
|  | |  | | | | | | | | | | | | | | | | | | | | | | | | | | | | | | | | | | | | | | | | | | | | | | | | | | | | | | | | | | | | | | | | | | | |
| 121 |  |  | K |  | G |  | | | | 122 |  | | | | | | | | | | | | | | | | | | | | | | | | | | | | | | | | | | | | | | | | | | | | | | | | | | | | | | | |

Fixed PTMs: Carbamidomethylation [C49 C56 C63 C64 C73 C75 C89 C91 ]

  

All peaks (109)  Matched peaks (51)  Not matched peaks (58)

  

| Scan | Peak | Mono mass | Mono m/z | Intensity | Charge | Theoretical mass | Ion | Pos | Mass error | PPM error |
| --- | --- | --- | --- | --- | --- | --- | --- | --- | --- | --- |
| 499 | 1 | 7112.1160 | 1017.0238 | 181042.87 | 7 |  |  |  |  |  |
| 499 | 2 | 7112.1171 | 1186.3601 | 86450.64 | 6 |  |  |  |  |  |
| 499 | 3 | 3585.0695 | 897.2746 | 134574.81 | 4 |  |  |  |  |  |
| 499 | 4 | 7125.1260 | 1018.8824 | 27478.58 | 7 |  |  |  |  |  |
| 499 | 5 | 7153.1265 | 1022.8825 | 29319.10 | 7 |  |  |  |  |  |
| 499 | 6 | 7004.0569 | 1001.5868 | 22989.48 | 7 |  |  |  |  |  |
| 499 | 7 | 7154.1321 | 1193.3626 | 23373.43 | 6 |  |  |  |  |  |
| 499 | 8 | 7055.0911 | 1176.8558 | 21499.67 | 6 |  |  |  |  |  |
| 499 | 9 | 7126.1294 | 1188.6955 | 16331.60 | 6 |  |  |  |  |  |
| 499 | 10 | 3585.5748 | 1196.1989 | 29720.60 | 3 |  |  |  |  |  |
| 499 | 11 | 6890.9739 | 985.4321 | 19860.38 | 7 |  |  |  |  |  |
| 499 | 12 | 7021.1023 | 1004.0219 | 13836.72 | 7 | 7021.1332 | C59 | 59 | -0.0309 | -4.39 |
| 499 | 13 | 2788.2236 | 930.4152 | 14405.63 | 3 | 2788.2414 | C22 | 22 | -0.0177 | -6.36 |
| 499 | 14 | 7080.1336 | 1012.4549 | 12942.26 | 7 |  |  |  |  |  |
| 499 | 15 | 6583.8473 | 1098.3152 | 12216.10 | 6 |  |  |  |  |  |
| 499 | 16 | 7095.1026 | 1183.5244 | 13643.33 | 6 |  |  |  |  |  |
| 499 | 17 | 3445.5805 | 862.4024 | 10723.71 | 4 | 3445.6046 | C27 | 27 | -0.0241 | -7.00 |
| 499 | 18 | 5887.5161 | 982.2600 | 11627.13 | 6 | 5887.5503 | C48 | 48 | -0.0341 | -5.80 |
| 499 | 19 | 6188.7070 | 1032.4584 | 10993.44 | 6 |  |  |  |  |  |
| 499 | 20 | 7004.0585 | 876.5146 | 11473.33 | 8 |  |  |  |  |  |
| 499 | 21 | 4443.9030 | 1111.9830 | 10309.97 | 4 | 4443.9333 | C36 | 36 | -0.0303 | -6.82 |
| 499 | 22 | 1024.4501 | 1025.4573 | 30927.65 | 1 |  |  |  |  |  |
| 499 | 23 | 7112.1234 | 1423.4320 | 8346.88 | 5 |  |  |  |  |  |
| 499 | 24 | 3157.4947 | 790.3809 | 11906.73 | 4 | 3157.5153 | C25 | 25 | -0.0207 | -6.55 |
| 499 | 25 | 2641.1553 | 881.3924 | 9521.95 | 3 | 2641.1730 | C21 | 21 | -0.0176 | -6.67 |
| 499 | 26 | 5213.3460 | 1043.6765 | 9168.62 | 5 |  |  |  |  |  |
| 499 | 27 | 1752.7578 | 877.3862 | 16920.13 | 2 | 1752.7671 | C14 | 14 | -9.28e-03 | -5.30 |
| 499 | 28 | 1866.7982 | 934.4064 | 11755.02 | 2 | 1866.8101 | C15 | 15 | -0.0119 | -6.36 |
| 499 | 29 | 3940.7559 | 986.1963 | 10322.71 | 4 | 3940.7834 | C31 | 31 | -0.0274 | -6.96 |
| 499 | 30 | 4554.9347 | 911.9942 | 7166.82 | 5 |  |  |  |  |  |
| 499 | 31 | 6209.6455 | 1035.9482 | 10561.67 | 6 | 6209.6892 | C51 | 51 | -0.0437 | -7.04 |
| 499 | 32 | 7068.0916 | 1179.0225 | 12336.32 | 6 |  |  |  |  |  |
| 499 | 33 | 7055.0952 | 1412.0263 | 5039.36 | 5 |  |  |  |  |  |
| 499 | 34 | 7065.1189 | 1010.3100 | 6764.92 | 7 | 7066.1350 | Z\_DOT59 | 1 | -0.0137 | -1.93 |
| 499 | 35 | 7152.1355 | 895.0242 | 8525.28 | 8 |  |  |  |  |  |
| 499 | 36 | 7053.0646 | 1008.5879 | 7620.87 | 7 |  |  |  |  |  |
| 499 | 37 | 7022.1054 | 1171.3582 | 5544.98 | 6 | 7021.1332 | C59 | 59 | -0.0302 | -4.30 |
| 499 | 38 | 2471.0525 | 824.6915 | 6387.30 | 3 | 2471.0674 | C19 | 19 | -0.0149 | -6.02 |
| 499 | 39 | 5417.3889 | 1084.4850 | 6432.76 | 5 | 5417.4264 | Z\_DOT46 | 14 | -0.0375 | -6.92 |
| 499 | 40 | 4381.9200 | 1096.4873 | 5299.02 | 4 | 4381.9521 | Z\_DOT38 | 22 | -0.0322 | -7.34 |
| 499 | 41 | 2668.2143 | 890.4120 | 5465.72 | 3 |  |  |  |  |  |
| 499 | 42 | 2916.3184 | 973.1134 | 5427.02 | 3 | 2916.3363 | C23 | 23 | -0.0180 | -6.16 |
| 499 | 43 | 2872.3058 | 958.4426 | 5940.09 | 3 |  |  |  |  |  |
| 499 | 44 | 5944.5308 | 991.7624 | 7169.56 | 6 | 5944.5717 | C49 | 49 | -0.0409 | -6.89 |
| 499 | 45 | 5327.3907 | 1066.4854 | 5167.00 | 5 |  |  |  |  |  |
| 499 | 46 | 7093.0954 | 1014.3066 | 6784.55 | 7 |  |  |  |  |  |
| 499 | 47 | 5798.5779 | 1160.7229 | 6754.60 | 5 | 5797.6072 | Z\_DOT49 | 11 | -0.0316 | -5.45 |
| 499 | 48 | 4055.7942 | 1014.9558 | 16711.52 | 4 | 4055.8103 | C32 | 32 | -0.0160 | -3.96 |
| 499 | 49 | 3317.5254 | 830.3886 | 6678.96 | 4 | 3317.5460 | C26 | 26 | -0.0206 | -6.20 |
| 499 | 50 | 868.4185 | 869.4258 | 8606.28 | 1 | 868.4225 | C7 | 7 | -3.96e-03 | -4.56 |
| 499 | 51 | 6302.7360 | 1261.5545 | 4192.79 | 5 | 6301.7710 | Z\_DOT53 | 7 | -0.0374 | -5.93 |
| 499 | 52 | 3922.6547 | 1308.5588 | 5460.31 | 3 |  |  |  |  |  |
| 499 | 53 | 3634.5665 | 1212.5294 | 6074.98 | 3 |  |  |  |  |  |
| 499 | 54 | 3692.6422 | 924.1678 | 4167.99 | 4 | 3692.6673 | C29 | 29 | -0.0251 | -6.79 |
| 499 | 55 | 6209.6521 | 1242.9377 | 4705.48 | 5 | 6209.6892 | C51 | 51 | -0.0372 | -5.98 |
| 499 | 56 | 2614.2029 | 872.4083 | 6625.22 | 3 |  |  |  |  |  |
| 499 | 57 | 5503.3123 | 1101.6697 | 5089.48 | 5 | 5503.3559 | C45 | 45 | -0.0436 | -7.92 |
| 499 | 58 | 6978.0953 | 1164.0232 | 5843.33 | 6 |  |  |  |  |  |
| 499 | 59 | 2528.0736 | 843.6985 | 4818.55 | 3 | 2528.0889 | C20 | 20 | -0.0153 | -6.05 |
| 499 | 60 | 4299.8536 | 1075.9707 | 4666.09 | 4 | 4299.8798 | C34 | 34 | -0.0263 | -6.11 |
| 499 | 61 | 5756.4595 | 960.4172 | 6211.72 | 6 | 5756.5098 | C47 | 47 | -0.0503 | -8.73 |
| 499 | 62 | 7035.1172 | 1006.0240 | 5524.39 | 7 |  |  |  |  |  |
| 499 | 63 | 7005.0858 | 1168.5216 | 5256.87 | 6 |  |  |  |  |  |
| 499 | 64 | 3157.4961 | 1053.5060 | 6052.26 | 3 | 3157.5153 | C25 | 25 | -0.0192 | -6.10 |
| 499 | 65 | 997.4618 | 998.4691 | 6296.61 | 1 | 997.4651 | C8 | 8 | -3.28e-03 | -3.28 |
| 499 | 66 | 4528.9904 | 1133.2549 | 4972.58 | 4 | 4529.0205 | Z\_DOT39 | 21 | -0.0301 | -6.65 |
| 499 | 67 | 6711.9383 | 1119.6637 | 3388.97 | 6 |  |  |  |  |  |
| 499 | 68 | 4170.8116 | 1043.7102 | 5986.36 | 4 | 4170.8372 | C33 | 33 | -0.0257 | -6.15 |
| 499 | 69 | 2726.2413 | 909.7544 | 3736.98 | 3 | 2726.2602 | Z\_DOT24 | 36 | -0.0188 | -6.91 |
| 499 | 70 | 3794.6921 | 949.6803 | 3231.96 | 4 |  |  |  |  |  |
| 499 | 71 | 6976.0757 | 997.5895 | 5561.43 | 7 |  |  |  |  |  |
| 499 | 72 | 3015.3429 | 1006.1216 | 3938.80 | 3 |  |  |  |  |  |
| 499 | 73 | 5797.5759 | 967.2699 | 4520.31 | 6 | 5797.6072 | Z\_DOT49 | 11 | -0.0313 | -5.40 |
| 499 | 74 | 6301.7263 | 1051.2950 | 3379.29 | 6 | 6301.7710 | Z\_DOT53 | 7 | -0.0447 | -7.10 |
| 499 | 75 | 5887.5049 | 1178.5083 | 5897.18 | 5 | 5887.5503 | C48 | 48 | -0.0454 | -7.71 |
| 499 | 76 | 5418.4009 | 1355.6075 | 3606.82 | 4 | 5417.4264 | Z\_DOT46 | 14 | -0.0278 | -5.13 |
| 499 | 77 | 6189.7032 | 1238.9479 | 2948.23 | 5 |  |  |  |  |  |
| 499 | 78 | 6947.0375 | 993.4412 | 3402.03 | 7 |  |  |  |  |  |
| 499 | 79 | 3868.6392 | 968.1671 | 2987.27 | 4 |  |  |  |  |  |
| 499 | 80 | 5053.3182 | 1011.6709 | 6145.04 | 5 |  |  |  |  |  |
| 499 | 81 | 4899.1159 | 1225.7862 | 3104.94 | 4 | 4899.1349 | C40 | 40 | -0.0190 | -3.89 |
| 499 | 82 | 5446.3026 | 1090.2678 | 2970.75 | 5 | 5446.3345 | C44 | 44 | -0.0319 | -5.85 |
| 499 | 83 | 6919.0603 | 1154.1840 | 2925.31 | 6 |  |  |  |  |  |
| 499 | 84 | 3392.4780 | 1131.8333 | 2105.53 | 3 |  |  |  |  |  |
| 499 | 85 | 6946.0364 | 1158.6800 | 2702.36 | 6 |  |  |  |  |  |
| 499 | 86 | 4642.0583 | 1161.5218 | 3424.51 | 4 | 4642.1046 | Z\_DOT40 | 20 | -0.0463 | -9.98 |
| 499 | 87 | 3229.3917 | 1077.4712 | 3288.75 | 3 | 3229.4101 | Z\_DOT29 | 31 | -0.0184 | -5.69 |
| 499 | 88 | 3183.5117 | 1062.1778 | 2703.63 | 3 |  |  |  |  |  |
| 499 | 89 | 602.3179 | 603.3251 | 5107.83 | 1 | 602.3210 | C5 | 5 | -3.11e-03 | -5.16 |
| 499 | 90 | 5503.3196 | 918.2272 | 2921.83 | 6 | 5503.3559 | C45 | 45 | -0.0364 | -6.61 |
| 499 | 91 | 7069.1212 | 1414.8315 | 3289.45 | 5 |  |  |  |  |  |
| 499 | 92 | 6833.9547 | 1139.9997 | 3055.40 | 6 |  |  |  |  |  |
| 499 | 93 | 6242.7044 | 1041.4580 | 3600.47 | 6 |  |  |  |  |  |
| 499 | 94 | 3114.3671 | 1039.1296 | 3649.94 | 3 | 3114.3832 | Z\_DOT28 | 32 | -0.0161 | -5.18 |
| 499 | 95 | 6152.6292 | 1231.5331 | 2560.18 | 5 |  |  |  |  |  |
| 499 | 96 | 6082.5751 | 1217.5223 | 5031.77 | 5 | 6081.6306 | C50 | 50 | -0.0579 | -9.53 |
| 499 | 97 | 6966.0869 | 1162.0218 | 3171.45 | 6 |  |  |  |  |  |
| 499 | 98 | 5831.4917 | 1167.3056 | 2787.53 | 5 |  |  |  |  |  |
| 499 | 99 | 1372.5754 | 1373.5827 | 2450.77 | 1 | 1372.5863 | C11 | 11 | -0.0109 | -7.94 |
| 499 | 100 | 739.3755 | 740.3828 | 3704.69 | 1 | 739.3799 | C6 | 6 | -4.40e-03 | -5.95 |
| 499 | 101 | 1372.5785 | 687.2965 | 2180.33 | 2 | 1372.5863 | C11 | 11 | -7.79e-03 | -5.68 |
| 499 | 102 | 1258.5366 | 1259.5439 | 1984.02 | 1 | 1258.5434 | C10 | 10 | -6.78e-03 | -5.39 |
| 499 | 103 | 1148.8313 | 1149.8386 | 1067.12 | 1 |  |  |  |  |  |
| 499 | 104 | 1226.6203 | 1227.6276 | 805.77 | 1 |  |  |  |  |  |
| 499 | 105 | 1283.6415 | 1284.6487 | 874.98 | 1 |  |  |  |  |  |
| 499 | 106 | 1206.5421 | 1207.5494 | 752.91 | 1 |  |  |  |  |  |
| 499 | 107 | 1089.5641 | 1090.5713 | 932.48 | 1 |  |  |  |  |  |
| 499 | 108 | 960.4985 | 961.5057 | 809.61 | 1 | 960.5043 | Z\_DOT9 | 51 | -5.80e-03 | -6.04 |
| 499 | 109 | 1063.9606 | 1064.9678 | 913.08 | 1 |  |  |  |  |  |

  

All proteins /
CsTx-1a\_S1 Cupiennius salei toxin 1 isoform a S1^ACsTx-1a\_S2 Cupiennius salei toxin 1 isoform a S2 /
Proteoform #8
