# Supplementary material for: The Dual Prey-Inactivation Strategy of Spiders—In-Depth Venomic Analysis of Cupiennius salei
Source: Toxins (Basel). 2019 Mar 19;11(3):167. doi: 10.3390/toxins11030167 (PMC6468893; doi:10.3390/toxins11030167)
Supplement: Supplementary file 1 [file toxins-11-00167-s001.zip › Supplementary Dataset EV1/20180328_f2_topdown_OTMS2_EThcD_NL_i02_ms2_proteoform_cutoff_html/prsms/prsm136.html]

Protein-Spectrum-Match for Spectrum #373


All proteins /
CsTx-1a\_S1 Cupiennius salei toxin 1 isoform a S1^ACsTx-1a\_S2 Cupiennius salei toxin 1 isoform a S2 /
Proteoform #16

## Protein-Spectrum-Match #136 for Spectrum #373

|  |  |  |  |  |  |
| --- | --- | --- | --- | --- | --- |
| PrSM ID: | 136 | Scan(s): | 500 | Precursor charge: | 7 |
| Precursor m/z: | 1047.3365 | Precursor mass: | 7324.3047 | Proteoform mass: | 7325.2868 |
| # matched peaks: | 12 | # matched fragment ions: | 12 | # unexpected modifications: | 0 |
| E-value: | 4.73e-14 | P-value: | 4.73e-14 | Q-value (Spectral FDR): | 0 |

  

|  |  |  |  |  |  |  |  |  |  |  |  |  |  |  |  |  |  |  |  |  |  |  |  |  |  |  |  |  |  |  |  |  |  |  |  |  |  |  |  |  |  |  |  |  |  |  |  |  |  |  |  |  |  |  |  |  |  |  |  |  |  |  |  |  |  |  |  |  |  |
| --- | --- | --- | --- | --- | --- | --- | --- | --- | --- | --- | --- | --- | --- | --- | --- | --- | --- | --- | --- | --- | --- | --- | --- | --- | --- | --- | --- | --- | --- | --- | --- | --- | --- | --- | --- | --- | --- | --- | --- | --- | --- | --- | --- | --- | --- | --- | --- | --- | --- | --- | --- | --- | --- | --- | --- | --- | --- | --- | --- | --- | --- | --- | --- | --- | --- | --- | --- | --- | --- |
|  | |  | | | | | | | | | | | | | | | | | | | | | | | | | | | | | | | | | | | | | | | | | | | | | | | | | | | | | | | | | | | | | | | | | | | |
| 1 |  |  | M |  | K |  | V |  | L |  | I |  | I |  | S |  | A |  | V |  | L |  |  | F |  | I |  | T |  | I |  | F |  | S |  | N |  | I |  | S |  | A |  |  | E |  | I |  | E |  | D |  | D |  | F |  | L |  | E |  | D |  | E |  | 30 |  |
|  | |  | | | | | | | | | | | | | | | | | | | | | | | | | | | | | | | | | | | | | | | | | | | | | | | | | | | | | | | | | | | | | | | | | | | |
| 31 |  |  | S |  | F |  | E |  | A |  | E |  | D |  | I |  | I |  | P |  | F |  |  | F |  | E |  | N |  | E |  | Q |  | A |  | R | ] | S |  | C |  | I |  |  | P |  | K | ⎫ | H | ⎫ | E | ⎫ | E | ⎫ | C |  | T |  | N | ⎩ | D |  | K |  | 60 |  |
|  | |  | | | | | | | | | | | | | | | | | | | | | | | | | | | | | | | | | | | | | | | | | | | | | | | | | | | | | | | | | | | | | | | | | | | |
| 61 |  |  | H | ⎫ | N | ⎫ | C |  | C |  | R |  | K |  | G |  | L |  | F |  | K |  |  | L |  | K | ⎫ | C | ⎫ | Q |  | C |  | S |  | T |  | F |  | D |  | D |  |  | E |  | S |  | G |  | Q |  | P |  | T |  | E |  | R |  | C |  | A |  | 90 |  |
|  | |  | | | | | | | | | | | | | | | | | | | | | | | | | | | | | | | | | | | | | | | | | | | | | | | | | | | | | | | | | | | | | | | | | | | |
| 91 |  |  | C |  | G |  | R |  | P |  | M | ⎫ | G |  | H | ⎫ | Q |  | A |  | I |  |  | E |  | T |  | G |  | L | ⎫ | N |  | I |  | F |  | R | [ | G |  | L |  |  | F |  | K |  | G |  | K |  | K |  | K |  | N |  | K |  | K |  | T |  | 120 |  |
|  | |  | | | | | | | | | | | | | | | | | | | | | | | | | | | | | | | | | | | | | | | | | | | | | | | | | | | | | | | | | | | | | | | | | | | |
| 121 |  |  | K |  | G |  | | | | 122 |  | | | | | | | | | | | | | | | | | | | | | | | | | | | | | | | | | | | | | | | | | | | | | | | | | | | | | | | |

Fixed PTMs: Carbamidomethylation [C49 C56 C63 C64 C73 C75 C89 C91 ]

  

All peaks (48)  Matched peaks (12)  Not matched peaks (36)

  

| Scan | Peak | Mono mass | Mono m/z | Intensity | Charge | Theoretical mass | Ion | Pos | Mass error | PPM error |
| --- | --- | --- | --- | --- | --- | --- | --- | --- | --- | --- |
| 500 | 1 | 7267.2275 | 1212.2119 | 62135.01 | 6 |  |  |  |  |  |
| 500 | 2 | 3662.6270 | 1221.8829 | 40281.06 | 3 |  |  |  |  |  |
| 500 | 3 | 7307.2277 | 1044.8969 | 12098.94 | 7 |  |  |  |  |  |
| 500 | 4 | 7281.2417 | 1214.5476 | 11534.05 | 6 |  |  |  |  |  |
| 500 | 5 | 1046.6090 | 1047.6163 | 36041.73 | 1 |  |  |  |  |  |
| 500 | 6 | 5887.5078 | 1178.5088 | 5960.56 | 5 | 5887.5503 | C48 | 48 | -0.0425 | -7.22 |
| 500 | 7 | 7309.2327 | 1219.2127 | 9553.72 | 6 |  |  |  |  |  |
| 500 | 8 | 7268.2281 | 1454.6529 | 7247.00 | 5 |  |  |  |  |  |
| 500 | 9 | 7235.2581 | 1206.8836 | 5918.38 | 6 |  |  |  |  |  |
| 500 | 10 | 7131.1966 | 1189.5401 | 6167.27 | 6 |  |  |  |  |  |
| 500 | 11 | 7251.2142 | 1209.5430 | 5350.24 | 6 |  |  |  |  |  |
| 500 | 12 | 1821.9442 | 911.9794 | 5012.62 | 2 |  |  |  |  |  |
| 500 | 13 | 1752.7572 | 877.3859 | 4219.13 | 2 | 1752.7671 | C14 | 14 | -9.89e-03 | -5.64 |
| 500 | 14 | 602.3177 | 603.3250 | 4663.82 | 1 | 602.3210 | C5 | 5 | -3.23e-03 | -5.36 |
| 500 | 15 | 1866.7988 | 934.4067 | 3371.82 | 2 | 1866.8101 | C15 | 15 | -0.0113 | -6.03 |
| 500 | 16 | 7191.2256 | 1199.5449 | 2188.46 | 6 |  |  |  |  |  |
| 500 | 17 | 7176.2135 | 1197.0429 | 3651.61 | 6 |  |  |  |  |  |
| 500 | 18 | 2881.3575 | 961.4598 | 2445.65 | 3 |  |  |  |  |  |
| 500 | 19 | 868.4172 | 869.4244 | 2555.11 | 1 | 868.4225 | C7 | 7 | -5.30e-03 | -6.11 |
| 500 | 20 | 7218.2373 | 1204.0468 | 3477.67 | 6 |  |  |  |  |  |
| 500 | 21 | 1948.9940 | 975.5043 | 2330.91 | 2 |  |  |  |  |  |
| 500 | 22 | 739.3758 | 740.3831 | 2946.02 | 1 | 739.3799 | C6 | 6 | -4.09e-03 | -5.54 |
| 500 | 23 | 2426.1597 | 809.7272 | 1390.62 | 3 |  |  |  |  |  |
| 500 | 24 | 2782.3029 | 928.4416 | 3299.99 | 3 |  |  |  |  |  |
| 500 | 25 | 6081.5881 | 1217.3249 | 1901.44 | 5 | 6081.6306 | C50 | 50 | -0.0425 | -6.99 |
| 500 | 26 | 2423.7457 | 1212.8801 | 5952.12 | 2 |  |  |  |  |  |
| 500 | 27 | 6793.9472 | 1359.7967 | 2064.61 | 5 | 6794.0062 | C57 | 57 | -0.0590 | -8.68 |
| 500 | 28 | 1221.7101 | 1222.7174 | 20869.42 | 1 |  |  |  |  |  |
| 500 | 29 | 532.3094 | 533.3167 | 1144.97 | 1 |  |  |  |  |  |
| 500 | 30 | 5952.6872 | 1191.5447 | 1033.75 | 5 | 5953.7083 | Z\_DOT50 | 11 | -0.0187 | -3.14 |
| 500 | 31 | 3157.4961 | 1053.5060 | 1616.84 | 3 | 3157.5153 | C25 | 25 | -0.0192 | -6.10 |
| 500 | 32 | 3317.5211 | 1106.8476 | 1068.57 | 3 | 3317.5460 | C26 | 26 | -0.0249 | -7.50 |
| 500 | 33 | 7308.2347 | 1462.6542 | 2150.43 | 5 |  |  |  |  |  |
| 500 | 34 | 5367.4577 | 1342.8717 | 1388.00 | 4 |  |  |  |  |  |
| 500 | 35 | 6237.7948 | 1248.5662 | 1780.06 | 5 |  |  |  |  |  |
| 500 | 36 | 2822.3301 | 941.7840 | 1019.55 | 3 |  |  |  |  |  |
| 500 | 37 | 997.4587 | 998.4660 | 969.03 | 1 | 997.4651 | C8 | 8 | -6.33e-03 | -6.34 |
| 500 | 38 | 802.4516 | 803.4589 | 1170.34 | 1 |  |  |  |  |  |
| 500 | 39 | 4424.9574 | 1475.9931 | 676.20 | 3 |  |  |  |  |  |
| 500 | 40 | 1115.6146 | 1116.6219 | 614.24 | 1 |  |  |  |  |  |
| 500 | 41 | 1465.8546 | 1466.8619 | 2582.41 | 1 |  |  |  |  |  |
| 500 | 42 | 1148.8631 | 1149.8704 | 566.37 | 1 |  |  |  |  |  |
| 500 | 43 | 3597.6055 | 1200.2091 | 842.06 | 3 |  |  |  |  |  |
| 500 | 44 | 1304.9643 | 1305.9716 | 542.50 | 1 |  |  |  |  |  |
| 500 | 45 | 1201.3694 | 1202.3766 | 694.03 | 1 |  |  |  |  |  |
| 500 | 46 | 1371.0088 | 1372.0161 | 926.99 | 1 |  |  |  |  |  |
| 500 | 47 | 1291.7720 | 1292.7792 | 553.16 | 1 |  |  |  |  |  |
| 500 | 48 | 1318.5862 | 1319.5935 | 484.54 | 1 |  |  |  |  |  |

  

All proteins /
CsTx-1a\_S1 Cupiennius salei toxin 1 isoform a S1^ACsTx-1a\_S2 Cupiennius salei toxin 1 isoform a S2 /
Proteoform #16
