# Supplementary material for: The Dual Prey-Inactivation Strategy of Spiders—In-Depth Venomic Analysis of Cupiennius salei
Source: Toxins (Basel). 2019 Mar 19;11(3):167. doi: 10.3390/toxins11030167 (PMC6468893; doi:10.3390/toxins11030167)
Supplement: Supplementary file 1 [file toxins-11-00167-s001.zip › Supplementary Dataset EV1/20180328_f2_topdown_OTMS2_EThcD_NL_i02_ms2_proteoform_cutoff_html/prsms/prsm137.html]

Protein-Spectrum-Match for Spectrum #374


All proteins /
CsTx-1a\_S1 Cupiennius salei toxin 1 isoform a S1^ACsTx-1a\_S2 Cupiennius salei toxin 1 isoform a S2 /
Proteoform #8

## Protein-Spectrum-Match #137 for Spectrum #374

|  |  |  |  |  |  |
| --- | --- | --- | --- | --- | --- |
| PrSM ID: | 137 | Scan(s): | 501 | Precursor charge: | 6 |
| Precursor m/z: | 1195.8720 | Precursor mass: | 7169.1885 | Proteoform mass: | 7169.1856 |
| # matched peaks: | 15 | # matched fragment ions: | 15 | # unexpected modifications: | 0 |
| E-value: | 1.53e-14 | P-value: | 1.53e-14 | Q-value (Spectral FDR): | 0 |

  

|  |  |  |  |  |  |  |  |  |  |  |  |  |  |  |  |  |  |  |  |  |  |  |  |  |  |  |  |  |  |  |  |  |  |  |  |  |  |  |  |  |  |  |  |  |  |  |  |  |  |  |  |  |  |  |  |  |  |  |  |  |  |  |  |  |  |  |  |  |  |
| --- | --- | --- | --- | --- | --- | --- | --- | --- | --- | --- | --- | --- | --- | --- | --- | --- | --- | --- | --- | --- | --- | --- | --- | --- | --- | --- | --- | --- | --- | --- | --- | --- | --- | --- | --- | --- | --- | --- | --- | --- | --- | --- | --- | --- | --- | --- | --- | --- | --- | --- | --- | --- | --- | --- | --- | --- | --- | --- | --- | --- | --- | --- | --- | --- | --- | --- | --- | --- | --- |
|  | |  | | | | | | | | | | | | | | | | | | | | | | | | | | | | | | | | | | | | | | | | | | | | | | | | | | | | | | | | | | | | | | | | | | | |
| 1 |  |  | M |  | K |  | V |  | L |  | I |  | I |  | S |  | A |  | V |  | L |  |  | F |  | I |  | T |  | I |  | F |  | S |  | N |  | I |  | S |  | A |  |  | E |  | I |  | E |  | D |  | D |  | F |  | L |  | E |  | D |  | E |  | 30 |  |
|  | |  | | | | | | | | | | | | | | | | | | | | | | | | | | | | | | | | | | | | | | | | | | | | | | | | | | | | | | | | | | | | | | | | | | | |
| 31 |  |  | S |  | F |  | E |  | A |  | E |  | D |  | I |  | I |  | P |  | F |  |  | F |  | E |  | N |  | E |  | Q |  | A |  | R | ] | S |  | C |  | I |  |  | P | ⎫ | K | ⎫ | H | ⎫ | E | ⎫ | E | ⎫ | C | ⎫ | T | ⎫ | N |  | D |  | K |  | 60 |  |
|  | |  | | | | | | | | | | | | | | | | | | | | | | | | | | | | | | | | | | | | | | | | | | | | | | | | | | | | | | | | | | | | | | | | | | | |
| 61 |  |  | H | ⎫ | N | ⎫ | C |  | C |  | R |  | K |  | G |  | L |  | F |  | K |  | ⎫ | L |  | K |  | C | ⎫ | Q | ⎫ | C |  | S |  | T |  | F |  | D | ⎫ | D |  |  | E |  | S |  | G |  | Q |  | P |  | T |  | E |  | R |  | C |  | A |  | 90 |  |
|  | |  | | | | | | | | | | | | | | | | | | | | | | | | | | | | | | | | | | | | | | | | | | | | | | | | | | | | | | | | | | | | | | | | | | | |
| 91 |  |  | C |  | G | ⎫ | R |  | P |  | M |  | G | ⎫ | H |  | Q |  | A |  | I |  |  | E |  | T |  | G |  | L |  | N |  | I |  | F | [ | R |  | G |  | L |  |  | F |  | K |  | G |  | K |  | K |  | K |  | N |  | K |  | K |  | T |  | 120 |  |
|  | |  | | | | | | | | | | | | | | | | | | | | | | | | | | | | | | | | | | | | | | | | | | | | | | | | | | | | | | | | | | | | | | | | | | | |
| 121 |  |  | K |  | G |  | | | | 122 |  | | | | | | | | | | | | | | | | | | | | | | | | | | | | | | | | | | | | | | | | | | | | | | | | | | | | | | | |

Fixed PTMs: Carbamidomethylation [C49 C56 C63 C64 C73 C75 C89 C91 ]

  

All peaks (32)  Matched peaks (15)  Not matched peaks (17)

  

| Scan | Peak | Mono mass | Mono m/z | Intensity | Charge | Theoretical mass | Ion | Pos | Mass error | PPM error |
| --- | --- | --- | --- | --- | --- | --- | --- | --- | --- | --- |
| 501 | 1 | 3585.0672 | 1196.0297 | 123237.92 | 3 |  |  |  |  |  |
| 501 | 2 | 7112.1130 | 1423.4299 | 38334.84 | 5 |  |  |  |  |  |
| 501 | 3 | 2390.3807 | 1196.1976 | 66868.83 | 2 |  |  |  |  |  |
| 501 | 4 | 7125.1239 | 1426.0321 | 8019.39 | 5 |  |  |  |  |  |
| 501 | 5 | 1752.7548 | 877.3847 | 6843.30 | 2 | 1752.7671 | C14 | 14 | -0.0123 | -7.04 |
| 501 | 6 | 1434.4291 | 1435.4363 | 12733.63 | 1 |  |  |  |  |  |
| 501 | 7 | 1866.7976 | 934.4061 | 4415.59 | 2 | 1866.8101 | C15 | 15 | -0.0125 | -6.69 |
| 501 | 8 | 7080.1239 | 1417.0321 | 4870.56 | 5 |  |  |  |  |  |
| 501 | 9 | 7153.1260 | 1431.6325 | 3882.75 | 5 |  |  |  |  |  |
| 501 | 10 | 602.3176 | 603.3249 | 5781.42 | 1 | 602.3210 | C5 | 5 | -3.35e-03 | -5.56 |
| 501 | 11 | 739.3758 | 740.3831 | 3869.02 | 1 | 739.3799 | C6 | 6 | -4.09e-03 | -5.54 |
| 501 | 12 | 2916.3158 | 973.1125 | 1951.65 | 3 | 2916.3363 | C23 | 23 | -0.0205 | -7.04 |
| 501 | 13 | 7023.1013 | 1405.6275 | 3000.19 | 5 |  |  |  |  |  |
| 501 | 14 | 868.4169 | 869.4242 | 2601.69 | 1 | 868.4225 | C7 | 7 | -5.55e-03 | -6.39 |
| 501 | 15 | 6977.0935 | 1396.4260 | 4570.15 | 5 |  |  |  |  |  |
| 501 | 16 | 7061.1010 | 1177.8574 | 1168.80 | 6 |  |  |  |  |  |
| 501 | 17 | 4442.8991 | 1481.9737 | 1292.14 | 3 |  |  |  |  |  |
| 501 | 18 | 3445.5822 | 1149.5347 | 1491.12 | 3 | 3445.6046 | C27 | 27 | -0.0223 | -6.48 |
| 501 | 19 | 997.4593 | 998.4666 | 1882.85 | 1 | 997.4651 | C8 | 8 | -5.78e-03 | -5.79 |
| 501 | 20 | 7036.1259 | 1408.2325 | 2017.17 | 5 |  |  |  |  |  |
| 501 | 21 | 3317.5245 | 1106.8488 | 1708.18 | 3 | 3317.5460 | C26 | 26 | -0.0215 | -6.48 |
| 501 | 22 | 474.2237 | 475.2309 | 1306.43 | 1 | 474.2260 | C4 | 4 | -2.35e-03 | -4.96 |
| 501 | 23 | 7068.1123 | 1414.6297 | 1787.89 | 5 |  |  |  |  |  |
| 501 | 24 | 5503.3125 | 1376.8354 | 1738.48 | 4 | 5503.3559 | C45 | 45 | -0.0434 | -7.89 |
| 501 | 25 | 6791.9457 | 1359.3964 | 1164.30 | 5 |  |  |  |  |  |
| 501 | 26 | 7095.1018 | 1420.0276 | 1632.79 | 5 |  |  |  |  |  |
| 501 | 27 | 2725.2415 | 1363.6280 | 954.62 | 2 |  |  |  |  |  |
| 501 | 28 | 1226.6200 | 1227.6273 | 677.93 | 1 |  |  |  |  |  |
| 501 | 29 | 4055.7822 | 1352.9347 | 576.41 | 3 | 4055.8103 | C32 | 32 | -0.0281 | -6.92 |
| 501 | 30 | 5944.5318 | 1487.1402 | 986.19 | 4 | 5944.5717 | C49 | 49 | -0.0400 | -6.72 |
| 501 | 31 | 1157.4877 | 1158.4949 | 423.95 | 1 | 1157.4957 | C9 | 9 | -8.05e-03 | -6.96 |
| 501 | 32 | 1258.5332 | 1259.5405 | 338.11 | 1 | 1258.5434 | C10 | 10 | -0.0102 | -8.10 |

  

All proteins /
CsTx-1a\_S1 Cupiennius salei toxin 1 isoform a S1^ACsTx-1a\_S2 Cupiennius salei toxin 1 isoform a S2 /
Proteoform #8
