# Supplementary material for: The Dual Prey-Inactivation Strategy of Spiders—In-Depth Venomic Analysis of Cupiennius salei
Source: Toxins (Basel). 2019 Mar 19;11(3):167. doi: 10.3390/toxins11030167 (PMC6468893; doi:10.3390/toxins11030167)
Supplement: Supplementary file 1 [file toxins-11-00167-s001.zip › Supplementary Dataset EV1/20180328_f2_topdown_OTMS2_EThcD_NL_i02_ms2_proteoform_cutoff_html/prsms/prsm138.html]

Protein-Spectrum-Match for Spectrum #375


All proteins /
CsTx-12b Cupiennius salei toxin 12 isoform b /
Proteoform #47

## Protein-Spectrum-Match #138 for Spectrum #375

|  |  |  |  |  |  |
| --- | --- | --- | --- | --- | --- |
| PrSM ID: | 138 | Scan(s): | 503 | Precursor charge: | 6 |
| Precursor m/z: | 571.9905 | Precursor mass: | 3425.8996 | Proteoform mass: | 3425.8946 |
| # matched peaks: | 30 | # matched fragment ions: | 25 | # unexpected modifications: | 1 |
| E-value: | 5.31e-20 | P-value: | 5.31e-20 | Q-value (Spectral FDR): | 0 |

  

|  |  |  |  |  |  |  |  |  |  |  |  |  |  |  |  |  |  |  |  |  |  |  |  |  |  |  |  |  |  |  |  |  |  |  |  |  |  |  |  |  |  |  |  |  |  |  |  |  |  |  |  |  |  |  |  |  |  |  |  |  |  |  |  |  |  |  |
| --- | --- | --- | --- | --- | --- | --- | --- | --- | --- | --- | --- | --- | --- | --- | --- | --- | --- | --- | --- | --- | --- | --- | --- | --- | --- | --- | --- | --- | --- | --- | --- | --- | --- | --- | --- | --- | --- | --- | --- | --- | --- | --- | --- | --- | --- | --- | --- | --- | --- | --- | --- | --- | --- | --- | --- | --- | --- | --- | --- | --- | --- | --- | --- | --- | --- | --- |
|  | | ... 30 amino acid residues are skipped at the N-terminus ... | | | | | | | | | | | | | | | | | | | | | | | | | | | | | | | | | | | | | | | | | | | | | | | | | | | | | | | | | | | | | |  | | |
|  | |  | | | | | | | | | | | | | | | | | | | | | | | | | | | | | | | | | | | | | | | | | | | | | | | | | | | | | | | | | | | | | | | | | | | |
| 31 |  |  | S |  | F |  | E |  | A |  | D |  | D |  | V |  | I |  | P |  | F |  |  | L |  | A |  | R |  | E |  | Q |  | V |  | R |  | S |  | D |  | C |  |  | T |  | L |  | R |  | N |  | H |  | D |  | C |  | T |  | D |  | D |  | 60 |  |
|  | |  | | | | | | | | | | | | | | | | | | | | | | | | | | | | | | | | | | | | | | | | | | | | | | | | | | | | | | | | | | | | | | | | | | | |
| 61 |  |  | R |  | H |  | S |  | C |  | C |  | R |  | S |  | K |  | M |  | F |  |  | K |  | D |  | V |  | C |  | K |  | C |  | F |  | Y |  | P |  | S |  |  | Q |  | R |  | S |  | D |  | T |  | A |  | R | ] | A | ⎩ | K | ⎩ | K |  | 90 |  |
|  | |  | | | | | | | | | | | | | | | | | | | | | | | | | | | | | | | | | | | | | | | | | | | | | | | | | | | | | -58.01 | | | | | | | | | | | |
| 91 |  | ⎫ | E | ⎫ | L | ⎫ | C |  | T | ⎫ | C | ⎫ | Q | ⎫ | Q |  | D | ⎱ | K |  | H |  |  | L |  | K | ⎱ | Y |  | I | ⎱ | E | ⎫ | K |  | G | ⎫ | L |  | Q | ⎱ | K |  | ⎫ | A | ⎱ | K | ⎫ | V | ⎫ | L | ⎫ | V | ⎫ | A |  | G |  | | 117 |  | | | | | |

Fixed PTMs: Carbamidomethylation [C93 C95 ]   
  
     Unexpected modifications:   Unknown [-58.01]

  

All peaks (57)  Matched peaks (30)  Not matched peaks (27)

  

| Scan | Peak | Mono mass | Mono m/z | Intensity | Charge | Theoretical mass | Ion | Pos | Mass error | PPM error |
| --- | --- | --- | --- | --- | --- | --- | --- | --- | --- | --- |
| 503 | 1 | 3368.8614 | 674.7796 | 530634.89 | 5 |  |  |  |  |  |
| 503 | 2 | 1713.4511 | 572.1577 | 1288600.73 | 3 |  |  |  |  |  |
| 503 | 3 | 3142.6973 | 786.6816 | 181286.96 | 4 | 3142.7106 | C26 | 26 | -0.0133 | -4.24 |
| 503 | 4 | 3354.8505 | 671.9774 | 152421.56 | 5 | 3354.8631 | C28 | 28 | -0.0126 | -3.77 |
| 503 | 5 | 3368.8649 | 843.2235 | 127120.59 | 4 |  |  |  |  |  |
| 503 | 6 | 2161.1046 | 721.3755 | 136742.11 | 3 | 2161.1135 | C17 | 17 | -8.94e-03 | -4.14 |
| 503 | 7 | 2048.2670 | 683.7629 | 131216.98 | 3 | 2048.2692 | Z\_DOT19 | 11 | -2.23e-03 | -1.09 |
| 503 | 8 | 3210.7366 | 803.6914 | 88220.71 | 4 | 3210.7439 | Z\_DOT28 | 2 | -7.29e-03 | -2.27 |
| 503 | 9 | 571.3149 | 572.3222 | 870520.77 | 1 |  |  |  |  |  |
| 503 | 10 | 1884.9583 | 629.3267 | 126961.77 | 3 | 1884.9662 | C15 | 15 | -7.83e-03 | -4.16 |
| 503 | 11 | 2475.2625 | 826.0948 | 100682.28 | 3 | 2475.2726 | C20 | 20 | -0.0100 | -4.06 |
| 503 | 12 | 3408.8690 | 569.1521 | 78441.09 | 6 |  |  |  |  |  |
| 503 | 13 | 3338.8300 | 668.7733 | 90108.32 | 5 | 3338.8389 | Z\_DOT29 | 1 | -8.90e-03 | -2.67 |
| 503 | 14 | 3409.8688 | 682.9810 | 83942.55 | 5 |  |  |  |  |  |
| 503 | 15 | 2290.1469 | 764.3896 | 94850.96 | 3 | 2290.1561 | C18 | 18 | -9.22e-03 | -4.03 |
| 503 | 16 | 1541.9358 | 771.9752 | 106252.08 | 2 | 1541.9363 | Z\_DOT15 | 15 | -4.91e-04 | -0.32 |
| 503 | 17 | 2915.5350 | 729.8910 | 71417.90 | 4 | 2915.5473 | C24 | 24 | -0.0123 | -4.22 |
| 503 | 18 | 1378.6269 | 690.3207 | 102864.79 | 2 | 1378.6333 | C11 | 11 | -6.40e-03 | -4.64 |
| 503 | 19 | 3338.8317 | 835.7152 | 77668.41 | 4 | 3338.8389 | Z\_DOT29 | 1 | -7.18e-03 | -2.15 |
| 503 | 20 | 3382.8795 | 846.7272 | 54409.94 | 4 |  |  |  |  |  |
| 503 | 21 | 2844.4983 | 712.1319 | 63506.59 | 4 | 2844.5102 | C23 | 23 | -0.0118 | -4.15 |
| 503 | 22 | 3043.6292 | 761.9146 | 57151.84 | 4 | 3043.6422 | C25 | 25 | -0.0130 | -4.28 |
| 503 | 23 | 2716.4040 | 906.4753 | 56146.90 | 3 | 2716.4152 | C22 | 22 | -0.0112 | -4.13 |
| 503 | 24 | 3381.8713 | 677.3815 | 52910.04 | 5 |  |  |  |  |  |
| 503 | 25 | 2361.3799 | 591.3523 | 53731.20 | 4 |  |  |  |  |  |
| 503 | 26 | 1265.7895 | 633.9020 | 64139.89 | 2 | 1265.7889 | Z\_DOT13 | 17 | 5.36e-04 | 0.42 |
| 503 | 27 | 2716.4041 | 680.1083 | 42805.88 | 4 | 2716.4152 | C22 | 22 | -0.0111 | -4.09 |
| 503 | 28 | 3424.8888 | 685.9850 | 383893.16 | 5 |  |  |  |  |  |
| 503 | 29 | 3255.7808 | 814.9525 | 41022.30 | 4 | 3255.7947 | C27 | 27 | -0.0139 | -4.27 |
| 503 | 30 | 3226.7544 | 807.6959 | 37157.89 | 4 |  |  |  |  |  |
| 503 | 31 | 2435.4271 | 609.8640 | 46548.53 | 4 |  |  |  |  |  |
| 503 | 32 | 1557.9546 | 779.9846 | 50877.66 | 2 |  |  |  |  |  |
| 503 | 33 | 3354.8490 | 839.7195 | 36363.33 | 4 | 3354.8631 | C28 | 28 | -0.0141 | -4.20 |
| 503 | 34 | 685.5788 | 686.5861 | 221171.16 | 1 |  |  |  |  |  |
| 503 | 35 | 2489.4380 | 623.3668 | 37504.41 | 4 |  |  |  |  |  |
| 503 | 36 | 3290.8738 | 659.1820 | 40922.39 | 5 |  |  |  |  |  |
| 503 | 37 | 908.5775 | 455.2961 | 51570.38 | 2 |  |  |  |  |  |
| 503 | 38 | 1007.4858 | 1008.4931 | 33303.17 | 1 | 1007.4892 | C8 | 8 | -3.34e-03 | -3.32 |
| 503 | 39 | 1206.7766 | 604.3956 | 19344.28 | 2 |  |  |  |  |  |
| 503 | 40 | 710.4905 | 711.4977 | 19108.23 | 1 | 710.4873 | Z\_DOT8 | 22 | 3.19e-03 | 4.49 |
| 503 | 41 | 1135.5438 | 1136.5511 | 14059.09 | 1 | 1135.5477 | C9 | 9 | -3.94e-03 | -3.47 |
| 503 | 42 | 511.3592 | 512.3665 | 10360.11 | 1 | 511.3552 | Z\_DOT6 | 24 | 4.03e-03 | 7.88 |
| 503 | 43 | 847.4553 | 848.4626 | 14888.50 | 1 | 847.4585 | C7 | 7 | -3.20e-03 | -3.78 |
| 503 | 44 | 586.3782 | 587.3854 | 10086.52 | 1 | 586.3802 | C5 | 5 | -2.02e-03 | -3.45 |
| 503 | 45 | 473.2948 | 474.3021 | 12857.35 | 1 | 473.2961 | C4 | 4 | -1.33e-03 | -2.82 |
| 503 | 46 | 780.5196 | 391.2671 | 7718.66 | 2 |  |  |  |  |  |
| 503 | 47 | 1007.4858 | 504.7502 | 8979.38 | 2 | 1007.4892 | C8 | 8 | -3.42e-03 | -3.39 |
| 503 | 48 | 632.3024 | 633.3097 | 23563.07 | 1 |  |  |  |  |  |
| 503 | 49 | 1078.6823 | 540.3484 | 6108.19 | 2 |  |  |  |  |  |
| 503 | 50 | 967.6505 | 484.8325 | 6734.78 | 2 |  |  |  |  |  |
| 503 | 51 | 1378.6283 | 1379.6356 | 3806.88 | 1 | 1378.6333 | C11 | 11 | -4.99e-03 | -3.62 |
| 503 | 52 | 1349.8342 | 450.9520 | 4133.35 | 3 |  |  |  |  |  |
| 503 | 53 | 1024.6722 | 513.3434 | 4388.40 | 2 |  |  |  |  |  |
| 503 | 54 | 344.2527 | 345.2600 | 6438.62 | 1 | 344.2535 | C3 | 3 | -7.97e-04 | -2.31 |
| 503 | 55 | 726.5091 | 727.5164 | 5893.05 | 1 |  |  |  |  |  |
| 503 | 56 | 1065.7102 | 533.8624 | 3367.31 | 2 |  |  |  |  |  |
| 503 | 57 | 1266.7972 | 1267.8045 | 3720.28 | 1 |  |  |  |  |  |

  

All proteins /
CsTx-12b Cupiennius salei toxin 12 isoform b /
Proteoform #47
