# Supplementary material for: The Dual Prey-Inactivation Strategy of Spiders—In-Depth Venomic Analysis of Cupiennius salei
Source: Toxins (Basel). 2019 Mar 19;11(3):167. doi: 10.3390/toxins11030167 (PMC6468893; doi:10.3390/toxins11030167)
Supplement: Supplementary file 1 [file toxins-11-00167-s001.zip › Supplementary Dataset EV1/20180328_f2_topdown_OTMS2_EThcD_NL_i02_ms2_proteoform_cutoff_html/prsms/prsm139.html]

Protein-Spectrum-Match for Spectrum #376


All proteins /
CsTx-1a\_S1 Cupiennius salei toxin 1 isoform a S1^ACsTx-1a\_S2 Cupiennius salei toxin 1 isoform a S2 /
Proteoform #8

## Protein-Spectrum-Match #139 for Spectrum #376

|  |  |  |  |  |  |
| --- | --- | --- | --- | --- | --- |
| PrSM ID: | 139 | Scan(s): | 504 | Precursor charge: | 7 |
| Precursor m/z: | 1025.1766 | Precursor mass: | 7169.1854 | Proteoform mass: | 7169.1856 |
| # matched peaks: | 26 | # matched fragment ions: | 25 | # unexpected modifications: | 0 |
| E-value: | 1.27e-23 | P-value: | 1.27e-23 | Q-value (Spectral FDR): | 0 |

  

|  |  |  |  |  |  |  |  |  |  |  |  |  |  |  |  |  |  |  |  |  |  |  |  |  |  |  |  |  |  |  |  |  |  |  |  |  |  |  |  |  |  |  |  |  |  |  |  |  |  |  |  |  |  |  |  |  |  |  |  |  |  |  |  |  |  |  |  |  |  |
| --- | --- | --- | --- | --- | --- | --- | --- | --- | --- | --- | --- | --- | --- | --- | --- | --- | --- | --- | --- | --- | --- | --- | --- | --- | --- | --- | --- | --- | --- | --- | --- | --- | --- | --- | --- | --- | --- | --- | --- | --- | --- | --- | --- | --- | --- | --- | --- | --- | --- | --- | --- | --- | --- | --- | --- | --- | --- | --- | --- | --- | --- | --- | --- | --- | --- | --- | --- | --- | --- |
|  | |  | | | | | | | | | | | | | | | | | | | | | | | | | | | | | | | | | | | | | | | | | | | | | | | | | | | | | | | | | | | | | | | | | | | |
| 1 |  |  | M |  | K |  | V |  | L |  | I |  | I |  | S |  | A |  | V |  | L |  |  | F |  | I |  | T |  | I |  | F |  | S |  | N |  | I |  | S |  | A |  |  | E |  | I |  | E |  | D |  | D |  | F |  | L |  | E |  | D |  | E |  | 30 |  |
|  | |  | | | | | | | | | | | | | | | | | | | | | | | | | | | | | | | | | | | | | | | | | | | | | | | | | | | | | | | | | | | | | | | | | | | |
| 31 |  |  | S |  | F |  | E |  | A |  | E |  | D |  | I |  | I |  | P |  | F |  |  | F |  | E |  | N |  | E |  | Q |  | A |  | R | ] | S |  | C |  | I |  |  | P | ⎩ | K | ⎱ | H | ⎫ | E | ⎱ | E | ⎫ | C | ⎩ | T |  | N |  | D |  | K |  | 60 |  |
|  | |  | | | | | | | | | | | | | | | | | | | | | | | | | | | | | | | | | | | | | | | | | | | | | | | | | | | | | | | | | | | | | | | | | | | |
| 61 |  |  | H | ⎫ | N | ⎫ | C | ⎫ | C |  | R |  | K |  | G |  | L |  | F |  | K |  | ⎫ | L |  | K | ⎫ | C | ⎫ | Q | ⎫ | C |  | S |  | T |  | F |  | D |  | D |  |  | E |  | S |  | G | ⎫ | Q |  | P |  | T |  | E |  | R |  | C |  | A |  | 90 |  |
|  | |  | | | | | | | | | | | | | | | | | | | | | | | | | | | | | | | | | | | | | | | | | | | | | | | | | | | | | | | | | | | | | | | | | | | |
| 91 |  |  | C |  | G | ⎫ | R |  | P | ⎫ | M | ⎫ | G | ⎫ | H |  | Q | ⎫ | A |  | I |  |  | E | ⎫ | T |  | G |  | L | ⎫ | N | ⎫ | I | ⎫ | F | [ | R |  | G |  | L |  |  | F |  | K |  | G |  | K |  | K |  | K |  | N |  | K |  | K |  | T |  | 120 |  |
|  | |  | | | | | | | | | | | | | | | | | | | | | | | | | | | | | | | | | | | | | | | | | | | | | | | | | | | | | | | | | | | | | | | | | | | |
| 121 |  |  | K |  | G |  | | | | 122 |  | | | | | | | | | | | | | | | | | | | | | | | | | | | | | | | | | | | | | | | | | | | | | | | | | | | | | | | |

Fixed PTMs: Carbamidomethylation [C49 C56 C63 C64 C73 C75 C89 C91 ]

  

All peaks (61)  Matched peaks (26)  Not matched peaks (35)

  

| Scan | Peak | Mono mass | Mono m/z | Intensity | Charge | Theoretical mass | Ion | Pos | Mass error | PPM error |
| --- | --- | --- | --- | --- | --- | --- | --- | --- | --- | --- |
| 504 | 1 | 7112.1123 | 1186.3593 | 81045.34 | 6 |  |  |  |  |  |
| 504 | 2 | 3585.5693 | 1196.1970 | 58783.14 | 3 |  |  |  |  |  |
| 504 | 3 | 7125.1267 | 1188.5284 | 15206.38 | 6 |  |  |  |  |  |
| 504 | 4 | 3075.7593 | 1026.2604 | 39937.46 | 3 |  |  |  |  |  |
| 504 | 5 | 1024.5926 | 1025.5999 | 74113.85 | 1 |  |  |  |  |  |
| 504 | 6 | 7153.1206 | 1193.1940 | 10182.27 | 6 |  |  |  |  |  |
| 504 | 7 | 7021.0935 | 1171.1895 | 6917.18 | 6 | 7021.1332 | C59 | 59 | -0.0396 | -5.64 |
| 504 | 8 | 3157.4943 | 1053.5054 | 8078.25 | 3 | 3157.5153 | C25 | 25 | -0.0211 | -6.68 |
| 504 | 9 | 7112.1144 | 1423.4302 | 6916.48 | 5 |  |  |  |  |  |
| 504 | 10 | 5944.5287 | 1189.9130 | 5261.50 | 5 | 5944.5717 | C49 | 49 | -0.0431 | -7.24 |
| 504 | 11 | 7080.1326 | 1181.0294 | 7923.47 | 6 |  |  |  |  |  |
| 504 | 12 | 6976.0841 | 1163.6880 | 6525.36 | 6 |  |  |  |  |  |
| 504 | 13 | 6081.5678 | 1217.3208 | 5086.42 | 5 |  |  |  |  |  |
| 504 | 14 | 4443.8971 | 1111.9816 | 4326.18 | 4 | 4443.9333 | C36 | 36 | -0.0362 | -8.14 |
| 504 | 15 | 2782.3027 | 928.4415 | 6356.51 | 3 |  |  |  |  |  |
| 504 | 16 | 2872.3036 | 958.4418 | 4854.02 | 3 |  |  |  |  |  |
| 504 | 17 | 7063.1258 | 1178.1949 | 4626.86 | 6 |  |  |  |  |  |
| 504 | 18 | 1866.7972 | 934.4059 | 3835.33 | 2 | 1866.8101 | C15 | 15 | -0.0128 | -6.88 |
| 504 | 19 | 7166.1306 | 1024.7402 | 132843.84 | 7 |  |  |  |  |  |
| 504 | 20 | 868.4170 | 869.4243 | 6257.94 | 1 | 868.4225 | C7 | 7 | -5.42e-03 | -6.25 |
| 504 | 21 | 5887.5052 | 1178.5083 | 5502.90 | 5 | 5887.5503 | C48 | 48 | -0.0451 | -7.66 |
| 504 | 22 | 5756.4745 | 1152.3022 | 2965.58 | 5 | 5756.5098 | C47 | 47 | -0.0353 | -6.13 |
| 504 | 23 | 6208.6392 | 1242.7351 | 5048.45 | 5 | 6209.6892 | C51 | 51 | -0.0477 | -7.68 |
| 504 | 24 | 7034.1193 | 1173.3605 | 4269.61 | 6 |  |  |  |  |  |
| 504 | 25 | 1752.7556 | 877.3851 | 6489.33 | 2 | 1752.7671 | C14 | 14 | -0.0115 | -6.55 |
| 504 | 26 | 6300.7269 | 1261.1527 | 2828.87 | 5 | 6301.7710 | Z\_DOT53 | 7 | -0.0418 | -6.63 |
| 504 | 27 | 3445.5771 | 1149.5330 | 2682.20 | 3 | 3445.6046 | C27 | 27 | -0.0275 | -7.97 |
| 504 | 28 | 3182.5050 | 1061.8423 | 2025.62 | 3 |  |  |  |  |  |
| 504 | 29 | 602.3176 | 603.3248 | 4869.56 | 1 | 602.3210 | C5 | 5 | -3.41e-03 | -5.66 |
| 504 | 30 | 7054.0959 | 1411.8265 | 2142.85 | 5 |  |  |  |  |  |
| 504 | 31 | 2916.3200 | 973.1139 | 2617.28 | 3 | 2916.3363 | C23 | 23 | -0.0163 | -5.59 |
| 504 | 32 | 5503.3147 | 1101.6702 | 2708.76 | 5 | 5503.3559 | C45 | 45 | -0.0412 | -7.48 |
| 504 | 33 | 7005.0601 | 1001.7301 | 3172.56 | 7 |  |  |  |  |  |
| 504 | 34 | 7125.1361 | 1426.0345 | 2102.96 | 5 |  |  |  |  |  |
| 504 | 35 | 6583.8464 | 1317.7766 | 1348.52 | 5 |  |  |  |  |  |
| 504 | 36 | 739.3755 | 740.3828 | 3881.51 | 1 | 739.3799 | C6 | 6 | -4.34e-03 | -5.87 |
| 504 | 37 | 3317.5218 | 1106.8479 | 1737.49 | 3 | 3317.5460 | C26 | 26 | -0.0242 | -7.29 |
| 504 | 38 | 6244.7082 | 1249.9489 | 1924.02 | 5 |  |  |  |  |  |
| 504 | 39 | 3922.6543 | 1308.5587 | 2903.06 | 3 |  |  |  |  |  |
| 504 | 40 | 7062.0910 | 1009.8774 | 1633.62 | 7 |  |  |  |  |  |
| 504 | 41 | 2026.8264 | 1014.4205 | 1307.75 | 2 | 2026.8407 | C16 | 16 | -0.0143 | -7.07 |
| 504 | 42 | 6567.8320 | 1314.5737 | 1907.05 | 5 | 6567.8725 | Z\_DOT55 | 5 | -0.0405 | -6.17 |
| 504 | 43 | 3074.7522 | 1538.3834 | 1257.38 | 2 |  |  |  |  |  |
| 504 | 44 | 6793.9546 | 1133.3330 | 1629.70 | 6 | 6794.0062 | C57 | 57 | -0.0516 | -7.59 |
| 504 | 45 | 997.4594 | 998.4667 | 1389.51 | 1 | 997.4651 | C8 | 8 | -5.66e-03 | -5.67 |
| 504 | 46 | 6011.6513 | 1203.3375 | 1062.90 | 5 | 6012.6978 | Z\_DOT51 | 9 | -0.0441 | -7.34 |
| 504 | 47 | 4900.0987 | 1226.0319 | 1063.60 | 4 |  |  |  |  |  |
| 504 | 48 | 6695.9267 | 1340.1926 | 1915.37 | 5 | 6695.9675 | Z\_DOT56 | 4 | -0.0407 | -6.09 |
| 504 | 49 | 4269.8666 | 1424.2961 | 2507.09 | 3 |  |  |  |  |  |
| 504 | 50 | 2744.2016 | 915.7411 | 1090.31 | 3 |  |  |  |  |  |
| 504 | 51 | 6522.7973 | 1305.5667 | 1104.67 | 5 | 6522.8530 | C54 | 54 | -0.0556 | -8.53 |
| 504 | 52 | 6478.7915 | 1296.7656 | 1125.93 | 5 |  |  |  |  |  |
| 504 | 53 | 3938.7504 | 985.6949 | 672.05 | 4 |  |  |  |  |  |
| 504 | 54 | 5503.3203 | 1376.8374 | 763.33 | 4 | 5503.3559 | C45 | 45 | -0.0356 | -6.47 |
| 504 | 55 | 908.7475 | 909.7548 | 627.14 | 1 |  |  |  |  |  |
| 504 | 56 | 6908.0658 | 1382.6204 | 618.46 | 5 | 6908.0491 | C58 | 58 | 0.0167 | 2.42 |
| 504 | 57 | 7080.1413 | 1012.4560 | 626.23 | 7 |  |  |  |  |  |
| 504 | 58 | 1363.6210 | 1364.6282 | 746.16 | 1 |  |  |  |  |  |
| 504 | 59 | 7095.0842 | 1420.0241 | 705.63 | 5 |  |  |  |  |  |
| 504 | 60 | 5053.3003 | 1264.3324 | 720.85 | 4 |  |  |  |  |  |
| 504 | 61 | 1395.8216 | 1396.8289 | 400.44 | 1 |  |  |  |  |  |

  

All proteins /
CsTx-1a\_S1 Cupiennius salei toxin 1 isoform a S1^ACsTx-1a\_S2 Cupiennius salei toxin 1 isoform a S2 /
Proteoform #8
