# Supplementary material for: The Dual Prey-Inactivation Strategy of Spiders—In-Depth Venomic Analysis of Cupiennius salei
Source: Toxins (Basel). 2019 Mar 19;11(3):167. doi: 10.3390/toxins11030167 (PMC6468893; doi:10.3390/toxins11030167)
Supplement: Supplementary file 1 [file toxins-11-00167-s001.zip › Supplementary Dataset EV1/20180328_f2_topdown_OTMS2_EThcD_NL_i02_ms2_proteoform_cutoff_html/prsms/prsm14.html]

Protein-Spectrum-Match for Spectrum #226


All proteins /
CsTx-13a Cupiennius salei toxin 13 isoform a /
Proteoform #80

## Protein-Spectrum-Match #14 for Spectrum #226

|  |  |  |  |  |  |
| --- | --- | --- | --- | --- | --- |
| PrSM ID: | 14 | Scan(s): | 304 | Precursor charge: | 6 |
| Precursor m/z: | 716.8000 | Precursor mass: | 4294.7566 | Proteoform mass: | 4294.7366 |
| # matched peaks: | 20 | # matched fragment ions: | 18 | # unexpected modifications: | 1 |
| E-value: | 1.23e-14 | P-value: | 1.23e-14 | Q-value (Spectral FDR): | 0 |

  

|  |  |  |  |  |  |  |  |  |  |  |  |  |  |  |  |  |  |  |  |  |  |  |  |  |  |  |  |  |  |  |  |  |  |  |  |  |  |  |  |  |  |  |  |  |  |  |  |  |  |  |  |  |  |  |  |  |  |  |  |  |  |  |  |  |  |  |  |  |  |
| --- | --- | --- | --- | --- | --- | --- | --- | --- | --- | --- | --- | --- | --- | --- | --- | --- | --- | --- | --- | --- | --- | --- | --- | --- | --- | --- | --- | --- | --- | --- | --- | --- | --- | --- | --- | --- | --- | --- | --- | --- | --- | --- | --- | --- | --- | --- | --- | --- | --- | --- | --- | --- | --- | --- | --- | --- | --- | --- | --- | --- | --- | --- | --- | --- | --- | --- | --- | --- | --- |
|  | |  | | | | | | | | | | | | | | | | | | | | | | | | | | | | | | | | | | | | | | | | | | | | | | | | | | | | | | | | | | | | | | | | | | | |
| 1 |  |  | M |  | K |  | V |  | L |  | V |  | I |  | F |  | A |  | V |  | L |  |  | S |  | L |  | V |  | I |  | F |  | S |  | N |  | C |  | S |  | A |  |  | E |  | T |  | D |  | E |  | D |  | F |  | F |  | G |  | E |  | E |  | 30 |  |
|  | |  | | | | | | | | | | | | | | | | | | | | | | | | | | | | | | | | | | | | | | | | | | | | | | | | | | | | | | | | | | | | | | | | | | | |
| 31 |  |  | S |  | F |  | E |  | A |  | D |  | D |  | I |  | I |  | P |  | F |  |  | I |  | A |  | K |  | E |  | Q |  | V |  | R | ] | S |  | D |  | C |  |  | T |  | L |  | R | ⎫ | N |  | H | ⎫ | D | ⎫ | C |  | T | ⎫ | D | ⎱ | D |  | 60 |  |
|  | |  | | | | | | | | | | | | | | | | | -48.02 | | | | | | | | | | | | | | | | | | | | | | | | | | | | | | | | | | | | | | | | | | | | | | | |
| 61 |  | ⎫ | R |  | H | ⎫ | S |  | C |  | C | ⎩ | R | ⎱ | S |  | K | ⎫ | M | ⎫ | F |  |  | K |  | D |  | V | ⎫ | C | ⎫ | T |  | C | ⎫ | F | ⎫ | Y |  | P |  | S |  | ⎫ | Q | [ | R |  | S |  | E |  | T |  | A |  | R |  | A |  | K |  | K |  | 90 |  |
|  | |  | | | | | | | | | | | | | | | | | | | | | | | | | | | | | | | | | | | | | | | | | | | | | | | | | | | | | | | | | | | | | | | | | | | |
| 91 |  |  | E |  | L |  | C |  | T |  | C |  | Q |  | Q |  | P |  | K |  | H |  |  | L |  | K |  | Y |  | I |  | E |  | K |  | G |  | L |  | Q |  | K |  |  | A |  | K |  | D |  | Y |  | A |  | T |  | G |  | | 117 |  | | | | | |

Fixed PTMs: Carbamidomethylation [C50 C57 C64 C65 C74 C76 ]   
  
     Unexpected modifications:   Unknown [-48.02]

  

All peaks (66)  Matched peaks (20)  Not matched peaks (46)

  

| Scan | Peak | Mono mass | Mono m/z | Intensity | Charge | Theoretical mass | Ion | Pos | Mass error | PPM error |
| --- | --- | --- | --- | --- | --- | --- | --- | --- | --- | --- |
| 304 | 1 | 4237.7028 | 848.5478 | 34621.89 | 5 |  |  |  |  |  |
| 304 | 2 | 4276.7148 | 713.7931 | 30195.58 | 6 |  |  |  |  |  |
| 304 | 3 | 3964.5745 | 793.9222 | 21881.08 | 5 |  |  |  |  |  |
| 304 | 4 | 4193.7131 | 699.9595 | 11121.63 | 6 |  |  |  |  |  |
| 304 | 5 | 4148.6544 | 830.7382 | 11276.63 | 5 |  |  |  |  |  |
| 304 | 6 | 2761.1099 | 691.2848 | 8706.56 | 4 | 2761.1097 | C22 | 22 | 1.99e-04 | 0.07 |
| 304 | 7 | 1431.9099 | 716.9622 | 17831.81 | 2 |  |  |  |  |  |
| 304 | 8 | 4238.7080 | 1060.6843 | 8073.79 | 4 |  |  |  |  |  |
| 304 | 9 | 3555.7291 | 712.1531 | 6696.35 | 5 |  |  |  |  |  |
| 304 | 10 | 3573.7402 | 715.7553 | 7836.13 | 5 |  |  |  |  |  |
| 304 | 11 | 4293.7225 | 716.6277 | 15429.67 | 6 |  |  |  |  |  |
| 304 | 12 | 2761.1097 | 921.3772 | 5712.55 | 3 | 2761.1097 | C22 | 22 | 2.83e-05 | 0.01 |
| 304 | 13 | 2148.8723 | 1075.4434 | 6588.39 | 2 |  |  |  |  |  |
| 304 | 14 | 3986.6507 | 997.6700 | 4559.89 | 4 |  |  |  |  |  |
| 304 | 15 | 4278.7116 | 856.7496 | 5025.79 | 5 |  |  |  |  |  |
| 304 | 16 | 2678.0736 | 893.6985 | 3998.66 | 3 | 2678.0914 | C21 | 21 | -0.0178 | -6.65 |
| 304 | 17 | 4278.7164 | 1070.6864 | 4322.38 | 4 |  |  |  |  |  |
| 304 | 18 | 859.1464 | 860.1537 | 7492.30 | 1 |  |  |  |  |  |
| 304 | 19 | 3475.8197 | 696.1712 | 3300.00 | 5 |  |  |  |  |  |
| 304 | 20 | 3671.4681 | 918.8743 | 2885.73 | 4 | 3671.4774 | C29 | 29 | -9.31e-03 | -2.54 |
| 304 | 21 | 4194.7164 | 839.9506 | 2946.26 | 5 |  |  |  |  |  |
| 304 | 22 | 3986.6421 | 798.3357 | 3597.07 | 5 |  |  |  |  |  |
| 304 | 23 | 3538.7245 | 885.6884 | 3088.17 | 4 |  |  |  |  |  |
| 304 | 24 | 2462.9463 | 821.9894 | 3347.25 | 3 | 2462.9644 | C19 | 19 | -0.0181 | -7.37 |
| 304 | 25 | 1491.5729 | 746.7937 | 3226.33 | 2 | 1491.5830 | C12 | 12 | -0.0101 | -6.78 |
| 304 | 26 | 3521.7074 | 881.4341 | 2662.84 | 4 |  |  |  |  |  |
| 304 | 27 | 4179.6836 | 1045.9282 | 2611.44 | 4 |  |  |  |  |  |
| 304 | 28 | 2864.4865 | 717.1289 | 23440.49 | 4 |  |  |  |  |  |
| 304 | 29 | 3526.7428 | 706.3558 | 3068.13 | 5 |  |  |  |  |  |
| 304 | 30 | 3818.5335 | 955.6407 | 2556.70 | 4 | 3818.5458 | C30 | 30 | -0.0123 | -3.22 |
| 304 | 31 | 3556.7379 | 890.1918 | 3040.09 | 4 |  |  |  |  |  |
| 304 | 32 | 4061.6244 | 813.3322 | 2062.08 | 5 |  |  |  |  |  |
| 304 | 33 | 3410.3949 | 853.6060 | 1785.66 | 4 | 3410.3991 | C27 | 27 | -4.18e-03 | -1.23 |
| 304 | 34 | 3964.5745 | 992.1509 | 2159.61 | 4 |  |  |  |  |  |
| 304 | 35 | 4206.7309 | 842.3534 | 2603.37 | 5 |  |  |  |  |  |
| 304 | 36 | 3250.3672 | 813.5991 | 2978.13 | 4 | 3250.3684 | C26 | 26 | -1.21e-03 | -0.37 |
| 304 | 37 | 3037.2710 | 760.3250 | 2208.21 | 4 |  |  |  |  |  |
| 304 | 38 | 749.3443 | 750.3516 | 1842.11 | 1 | 749.3490 | C6 | 6 | -4.69e-03 | -6.26 |
| 304 | 39 | 1899.7574 | 950.8860 | 2451.57 | 2 | 1899.7700 | C15 | 15 | -0.0126 | -6.63 |
| 304 | 40 | 4165.6750 | 834.1423 | 2502.77 | 5 | 4165.6940 | C33 | 33 | -0.0190 | -4.56 |
| 304 | 41 | 4147.6959 | 1037.9312 | 1964.05 | 4 |  |  |  |  |  |
| 304 | 42 | 4192.6832 | 1049.1781 | 1642.90 | 4 |  |  |  |  |  |
| 304 | 43 | 3538.7144 | 708.7502 | 1892.92 | 5 |  |  |  |  |  |
| 304 | 44 | 1376.5491 | 689.2818 | 1462.83 | 2 | 1376.5561 | C11 | 11 | -6.98e-03 | -5.07 |
| 304 | 45 | 1606.5994 | 804.3070 | 1762.51 | 2 | 1606.6100 | C13 | 13 | -0.0106 | -6.58 |
| 304 | 46 | 4253.7121 | 851.7497 | 1459.90 | 5 |  |  |  |  |  |
| 304 | 47 | 1832.7864 | 917.4005 | 1365.26 | 2 | 1832.7800 | Z\_DOT15 | 19 | 6.45e-03 | 3.52 |
| 304 | 48 | 3801.5124 | 761.3098 | 1442.33 | 5 |  |  |  |  |  |
| 304 | 49 | 2804.1622 | 935.7280 | 1957.09 | 3 | 2804.1614 | Z\_DOT22 | 12 | 7.85e-04 | 0.28 |
| 304 | 50 | 1755.7204 | 878.8675 | 1200.39 | 2 |  |  |  |  |  |
| 304 | 51 | 1988.8876 | 995.4511 | 1072.74 | 2 | 1988.8811 | Z\_DOT16 | 18 | 6.54e-03 | 3.29 |
| 304 | 52 | 330.1523 | 331.1596 | 2030.26 | 1 |  |  |  |  |  |
| 304 | 53 | 1115.4704 | 558.7425 | 839.09 | 2 | 1115.4778 | C9 | 9 | -7.38e-03 | -6.62 |
| 304 | 54 | 1098.7645 | 1099.7718 | 815.61 | 1 |  |  |  |  |  |
| 304 | 55 | 699.0448 | 700.0521 | 877.84 | 1 |  |  |  |  |  |
| 304 | 56 | 881.3492 | 882.3564 | 763.20 | 1 |  |  |  |  |  |
| 304 | 57 | 1474.5468 | 738.2807 | 1652.28 | 2 |  |  |  |  |  |
| 304 | 58 | 1000.4444 | 501.2295 | 1157.20 | 2 | 1000.4508 | C8 | 8 | -6.38e-03 | -6.38 |
| 304 | 59 | 1000.4436 | 1001.4509 | 745.46 | 1 | 1000.4508 | C8 | 8 | -7.22e-03 | -7.22 |
| 304 | 60 | 1073.4109 | 1074.4182 | 891.27 | 1 |  |  |  |  |  |
| 304 | 61 | 982.4081 | 983.4153 | 589.46 | 1 |  |  |  |  |  |
| 304 | 62 | 1412.9044 | 1413.9117 | 403.55 | 1 |  |  |  |  |  |
| 304 | 63 | 814.3460 | 815.3533 | 491.55 | 1 |  |  |  |  |  |
| 304 | 64 | 1048.1717 | 1049.1790 | 348.54 | 1 |  |  |  |  |  |
| 304 | 65 | 1454.0851 | 728.0498 | 567.03 | 2 |  |  |  |  |  |
| 304 | 66 | 826.7316 | 827.7389 | 531.28 | 1 |  |  |  |  |  |

  

All proteins /
CsTx-13a Cupiennius salei toxin 13 isoform a /
Proteoform #80
