# Supplementary material for: The Dual Prey-Inactivation Strategy of Spiders—In-Depth Venomic Analysis of Cupiennius salei
Source: Toxins (Basel). 2019 Mar 19;11(3):167. doi: 10.3390/toxins11030167 (PMC6468893; doi:10.3390/toxins11030167)
Supplement: Supplementary file 1 [file toxins-11-00167-s001.zip › Supplementary Dataset EV1/20180328_f2_topdown_OTMS2_EThcD_NL_i02_ms2_proteoform_cutoff_html/prsms/prsm140.html]

Protein-Spectrum-Match for Spectrum #377


All proteins /
CsTx-1a\_S1 Cupiennius salei toxin 1 isoform a S1^ACsTx-1a\_S2 Cupiennius salei toxin 1 isoform a S2 /
Proteoform #8

## Protein-Spectrum-Match #140 for Spectrum #377

|  |  |  |  |  |  |
| --- | --- | --- | --- | --- | --- |
| PrSM ID: | 140 | Scan(s): | 505 | Precursor charge: | 6 |
| Precursor m/z: | 1195.8720 | Precursor mass: | 7169.1885 | Proteoform mass: | 7169.1856 |
| # matched peaks: | 7 | # matched fragment ions: | 7 | # unexpected modifications: | 0 |
| E-value: | 7.27e-07 | P-value: | 7.27e-07 | Q-value (Spectral FDR): | 4.80e-03 |

  

|  |  |  |  |  |  |  |  |  |  |  |  |  |  |  |  |  |  |  |  |  |  |  |  |  |  |  |  |  |  |  |  |  |  |  |  |  |  |  |  |  |  |  |  |  |  |  |  |  |  |  |  |  |  |  |  |  |  |  |  |  |  |  |  |  |  |  |  |  |  |
| --- | --- | --- | --- | --- | --- | --- | --- | --- | --- | --- | --- | --- | --- | --- | --- | --- | --- | --- | --- | --- | --- | --- | --- | --- | --- | --- | --- | --- | --- | --- | --- | --- | --- | --- | --- | --- | --- | --- | --- | --- | --- | --- | --- | --- | --- | --- | --- | --- | --- | --- | --- | --- | --- | --- | --- | --- | --- | --- | --- | --- | --- | --- | --- | --- | --- | --- | --- | --- | --- |
|  | |  | | | | | | | | | | | | | | | | | | | | | | | | | | | | | | | | | | | | | | | | | | | | | | | | | | | | | | | | | | | | | | | | | | | |
| 1 |  |  | M |  | K |  | V |  | L |  | I |  | I |  | S |  | A |  | V |  | L |  |  | F |  | I |  | T |  | I |  | F |  | S |  | N |  | I |  | S |  | A |  |  | E |  | I |  | E |  | D |  | D |  | F |  | L |  | E |  | D |  | E |  | 30 |  |
|  | |  | | | | | | | | | | | | | | | | | | | | | | | | | | | | | | | | | | | | | | | | | | | | | | | | | | | | | | | | | | | | | | | | | | | |
| 31 |  |  | S |  | F |  | E |  | A |  | E |  | D |  | I |  | I |  | P |  | F |  |  | F |  | E |  | N |  | E |  | Q |  | A |  | R | ] | S |  | C |  | I |  |  | P |  | K | ⎫ | H | ⎫ | E | ⎫ | E | ⎫ | C |  | T |  | N |  | D |  | K |  | 60 |  |
|  | |  | | | | | | | | | | | | | | | | | | | | | | | | | | | | | | | | | | | | | | | | | | | | | | | | | | | | | | | | | | | | | | | | | | | |
| 61 |  |  | H | ⎫ | N | ⎫ | C |  | C |  | R |  | K |  | G |  | L |  | F |  | K |  |  | L |  | K |  | C |  | Q |  | C |  | S |  | T |  | F |  | D |  | D |  |  | E |  | S |  | G |  | Q |  | P |  | T |  | E |  | R |  | C |  | A |  | 90 |  |
|  | |  | | | | | | | | | | | | | | | | | | | | | | | | | | | | | | | | | | | | | | | | | | | | | | | | | | | | | | | | | | | | | | | | | | | |
| 91 |  |  | C |  | G |  | R |  | P |  | M |  | G |  | H |  | Q |  | A |  | I |  |  | E |  | T |  | G |  | L |  | N |  | I | ⎫ | F | [ | R |  | G |  | L |  |  | F |  | K |  | G |  | K |  | K |  | K |  | N |  | K |  | K |  | T |  | 120 |  |
|  | |  | | | | | | | | | | | | | | | | | | | | | | | | | | | | | | | | | | | | | | | | | | | | | | | | | | | | | | | | | | | | | | | | | | | |
| 121 |  |  | K |  | G |  | | | | 122 |  | | | | | | | | | | | | | | | | | | | | | | | | | | | | | | | | | | | | | | | | | | | | | | | | | | | | | | | |

Fixed PTMs: Carbamidomethylation [C49 C56 C63 C64 C73 C75 C89 C91 ]

  

All peaks (15)  Matched peaks (7)  Not matched peaks (8)

  

| Scan | Peak | Mono mass | Mono m/z | Intensity | Charge | Theoretical mass | Ion | Pos | Mass error | PPM error |
| --- | --- | --- | --- | --- | --- | --- | --- | --- | --- | --- |
| 505 | 1 | 3585.0657 | 1196.0292 | 76553.24 | 3 |  |  |  |  |  |
| 505 | 2 | 7112.1106 | 1423.4294 | 20717.24 | 5 |  |  |  |  |  |
| 505 | 3 | 2390.3795 | 1196.1970 | 41492.22 | 2 |  |  |  |  |  |
| 505 | 4 | 7125.1286 | 1426.0330 | 5091.90 | 5 |  |  |  |  |  |
| 505 | 5 | 1752.7554 | 877.3850 | 3707.98 | 2 | 1752.7671 | C14 | 14 | -0.0117 | -6.69 |
| 505 | 6 | 602.3176 | 603.3249 | 3482.07 | 1 | 602.3210 | C5 | 5 | -3.35e-03 | -5.56 |
| 505 | 7 | 1866.7981 | 934.4063 | 2810.85 | 2 | 1866.8101 | C15 | 15 | -0.0120 | -6.42 |
| 505 | 8 | 7079.1325 | 1416.8338 | 2278.34 | 5 |  |  |  |  |  |
| 505 | 9 | 739.3753 | 740.3826 | 2378.92 | 1 | 739.3799 | C6 | 6 | -4.58e-03 | -6.20 |
| 505 | 10 | 2915.3092 | 972.7770 | 1064.57 | 3 |  |  |  |  |  |
| 505 | 11 | 997.4576 | 998.4649 | 1093.41 | 1 | 997.4651 | C8 | 8 | -7.43e-03 | -7.45 |
| 505 | 12 | 6977.0879 | 1396.4249 | 1451.43 | 5 |  |  |  |  |  |
| 505 | 13 | 868.4172 | 869.4245 | 1822.73 | 1 | 868.4225 | C7 | 7 | -5.24e-03 | -6.03 |
| 505 | 14 | 7022.0872 | 1405.4247 | 1670.06 | 5 | 7021.1332 | C59 | 59 | -0.0483 | -6.88 |
| 505 | 15 | 1363.1216 | 1364.1288 | 493.62 | 1 |  |  |  |  |  |

  

All proteins /
CsTx-1a\_S1 Cupiennius salei toxin 1 isoform a S1^ACsTx-1a\_S2 Cupiennius salei toxin 1 isoform a S2 /
Proteoform #8
