# Supplementary material for: The Dual Prey-Inactivation Strategy of Spiders—In-Depth Venomic Analysis of Cupiennius salei
Source: Toxins (Basel). 2019 Mar 19;11(3):167. doi: 10.3390/toxins11030167 (PMC6468893; doi:10.3390/toxins11030167)
Supplement: Supplementary file 1 [file toxins-11-00167-s001.zip › Supplementary Dataset EV1/20180328_f2_topdown_OTMS2_EThcD_NL_i02_ms2_proteoform_cutoff_html/prsms/prsm141.html]

Protein-Spectrum-Match for Spectrum #378


All proteins /
CsTx-13a Cupiennius salei toxin 13 isoform a /
Proteoform #40

## Protein-Spectrum-Match #141 for Spectrum #378

|  |  |  |  |  |  |
| --- | --- | --- | --- | --- | --- |
| PrSM ID: | 141 | Scan(s): | 507 | Precursor charge: | 6 |
| Precursor m/z: | 580.3143 | Precursor mass: | 3475.8420 | Proteoform mass: | 3475.8320 |
| # matched peaks: | 32 | # matched fragment ions: | 27 | # unexpected modifications: | 1 |
| E-value: | 5.31e-21 | P-value: | 5.31e-21 | Q-value (Spectral FDR): | 0 |

  

|  |  |  |  |  |  |  |  |  |  |  |  |  |  |  |  |  |  |  |  |  |  |  |  |  |  |  |  |  |  |  |  |  |  |  |  |  |  |  |  |  |  |  |  |  |  |  |  |  |  |  |  |  |  |  |  |  |  |  |  |  |  |  |  |  |  |  |
| --- | --- | --- | --- | --- | --- | --- | --- | --- | --- | --- | --- | --- | --- | --- | --- | --- | --- | --- | --- | --- | --- | --- | --- | --- | --- | --- | --- | --- | --- | --- | --- | --- | --- | --- | --- | --- | --- | --- | --- | --- | --- | --- | --- | --- | --- | --- | --- | --- | --- | --- | --- | --- | --- | --- | --- | --- | --- | --- | --- | --- | --- | --- | --- | --- | --- | --- |
|  | | ... 30 amino acid residues are skipped at the N-terminus ... | | | | | | | | | | | | | | | | | | | | | | | | | | | | | | | | | | | | | | | | | | | | | | | | | | | | | | | | | | | | | |  | | |
|  | |  | | | | | | | | | | | | | | | | | | | | | | | | | | | | | | | | | | | | | | | | | | | | | | | | | | | | | | | | | | | | | | | | | | | |
| 31 |  |  | S |  | F |  | E |  | A |  | D |  | D |  | I |  | I |  | P |  | F |  |  | I |  | A |  | K |  | E |  | Q |  | V |  | R |  | S |  | D |  | C |  |  | T |  | L |  | R |  | N |  | H |  | D |  | C |  | T |  | D |  | D |  | 60 |  |
|  | |  | | | | | | | | | | | | | | | | | | | | | | | | | | | | | | | | | | | | | | | | | | | | | | | | | | | | | | | | | | | | | | | | | | | |
| 61 |  |  | R |  | H |  | S |  | C |  | C |  | R |  | S |  | K |  | M |  | F |  |  | K |  | D |  | V |  | C |  | T |  | C |  | F |  | Y |  | P |  | S |  |  | Q |  | R |  | S |  | E |  | T |  | A |  | R | ] | A | ⎩ | K | ⎩ | K |  | 90 |  |
|  | |  | | | | | | | | | | | | | | | | | | | | | | | | | | | | | | | | | | | | | | | | | | | | | | | | | | | -58.02 | | | | | | | | | | | | | |
| 91 |  |  | E | ⎱ | L |  | C | ⎫ | T | ⎫ | C | ⎫ | Q | ⎱ | Q |  | P | ⎱ | K | ⎫ | H |  |  | L | ⎫ | K | ⎱ | Y | ⎫ | I | ⎱ | E | ⎱ | K |  | G |  | L |  | Q | ⎱ | K |  | ⎱ | A |  | K | ⎫ | D | ⎫ | Y | ⎫ | A |  | T |  | G |  | | 117 |  | | | | | |

Fixed PTMs: Carbamidomethylation [C93 C95 ]   
  
     Unexpected modifications:   Unknown [-58.02]

  

All peaks (57)  Matched peaks (32)  Not matched peaks (25)

  

| Scan | Peak | Mono mass | Mono m/z | Intensity | Charge | Theoretical mass | Ion | Pos | Mass error | PPM error |
| --- | --- | --- | --- | --- | --- | --- | --- | --- | --- | --- |
| 507 | 1 | 3418.8003 | 684.7673 | 91089.70 | 5 |  |  |  |  |  |
| 507 | 2 | 3474.8273 | 580.1452 | 411275.36 | 6 |  |  |  |  |  |
| 507 | 3 | 3140.6751 | 786.1760 | 41876.04 | 4 | 3140.6950 | C26 | 26 | -0.0199 | -6.34 |
| 507 | 4 | 3025.6507 | 757.4199 | 35989.78 | 4 | 3025.6680 | C25 | 25 | -0.0174 | -5.74 |
| 507 | 5 | 2272.1682 | 758.3967 | 32641.40 | 3 | 2272.1820 | C18 | 18 | -0.0138 | -6.07 |
| 507 | 6 | 3418.8021 | 855.7078 | 31075.27 | 4 |  |  |  |  |  |
| 507 | 7 | 1158.9435 | 580.4790 | 149401.31 | 2 |  |  |  |  |  |
| 507 | 8 | 578.3324 | 579.3397 | 51442.95 | 1 |  |  |  |  |  |
| 507 | 9 | 1866.9814 | 623.3344 | 36104.68 | 3 | 1866.9920 | C15 | 15 | -0.0106 | -5.67 |
| 507 | 10 | 2143.1271 | 715.3830 | 26828.56 | 3 | 2143.1394 | C17 | 17 | -0.0122 | -5.71 |
| 507 | 11 | 3459.8056 | 692.9684 | 22878.38 | 5 |  |  |  |  |  |
| 507 | 12 | 2698.4254 | 900.4824 | 24358.61 | 3 | 2698.4410 | C22 | 22 | -0.0156 | -5.78 |
| 507 | 13 | 2826.5191 | 707.6370 | 23320.05 | 4 | 2826.5360 | C23 | 23 | -0.0169 | -5.97 |
| 507 | 14 | 2800.4517 | 701.1202 | 20790.32 | 4 |  |  |  |  |  |
| 507 | 15 | 3303.7386 | 826.9419 | 16657.38 | 4 | 3303.7583 | C27 | 27 | -0.0197 | -5.98 |
| 507 | 16 | 3260.6724 | 816.1754 | 18024.35 | 4 | 3260.6813 | Z\_DOT28 | 2 | -8.88e-03 | -2.72 |
| 507 | 17 | 2116.1793 | 706.4004 | 26169.34 | 3 | 2116.1808 | Z\_DOT19 | 11 | -1.44e-03 | -0.68 |
| 507 | 18 | 2539.3758 | 635.8512 | 18927.76 | 4 |  |  |  |  |  |
| 507 | 19 | 3388.7631 | 678.7599 | 14890.62 | 5 | 3388.7763 | Z\_DOT29 | 1 | -0.0131 | -3.87 |
| 507 | 20 | 1609.8499 | 805.9322 | 29703.93 | 2 | 1609.8479 | Z\_DOT15 | 15 | 2.00e-03 | 1.25 |
| 507 | 21 | 3432.8158 | 859.2112 | 15103.32 | 4 |  |  |  |  |  |
| 507 | 22 | 2341.2901 | 781.4373 | 20196.96 | 3 | 2341.2921 | Z\_DOT21 | 9 | -2.00e-03 | -0.86 |
| 507 | 23 | 3303.7376 | 661.7548 | 17136.66 | 5 | 3303.7583 | C27 | 27 | -0.0207 | -6.28 |
| 507 | 24 | 1360.6518 | 681.3332 | 21936.43 | 2 | 1360.6591 | C11 | 11 | -7.32e-03 | -5.38 |
| 507 | 25 | 3458.8022 | 577.4743 | 14011.60 | 6 |  |  |  |  |  |
| 507 | 26 | 3460.8158 | 866.2112 | 12608.30 | 4 |  |  |  |  |  |
| 507 | 27 | 1625.8684 | 813.9415 | 22801.63 | 2 |  |  |  |  |  |
| 507 | 28 | 3004.5443 | 1002.5220 | 12121.82 | 3 |  |  |  |  |  |
| 507 | 29 | 2960.4832 | 741.1281 | 10520.47 | 4 |  |  |  |  |  |
| 507 | 30 | 3025.6497 | 1009.5572 | 13390.45 | 3 | 3025.6680 | C25 | 25 | -0.0183 | -6.06 |
| 507 | 31 | 3431.8069 | 687.3687 | 10200.47 | 5 |  |  |  |  |  |
| 507 | 32 | 1204.6624 | 603.3385 | 15903.87 | 2 | 1204.6579 | Z\_DOT12 | 18 | 4.48e-03 | 3.72 |
| 507 | 33 | 695.5665 | 696.5738 | 73045.22 | 1 |  |  |  |  |  |
| 507 | 34 | 2030.0443 | 677.6887 | 9873.35 | 3 | 2030.0553 | C16 | 16 | -0.0110 | -5.41 |
| 507 | 35 | 3474.8216 | 695.9716 | 120986.72 | 5 |  |  |  |  |  |
| 507 | 36 | 1738.8846 | 870.4496 | 11838.00 | 2 | 1738.8970 | C14 | 14 | -0.0125 | -7.17 |
| 507 | 37 | 3003.5369 | 751.8915 | 12465.59 | 4 | 3003.5437 | Z\_DOT26 | 4 | -6.82e-03 | -2.27 |
| 507 | 38 | 1488.7458 | 745.3802 | 10149.97 | 2 | 1488.7540 | C12 | 12 | -8.29e-03 | -5.57 |
| 507 | 39 | 1333.7041 | 667.8593 | 13386.24 | 2 | 1333.7005 | Z\_DOT13 | 17 | 3.64e-03 | 2.73 |
| 507 | 40 | 1135.5417 | 568.7781 | 12960.59 | 2 | 1135.5477 | C9 | 9 | -6.09e-03 | -5.36 |
| 507 | 41 | 561.3051 | 562.3124 | 11781.58 | 1 |  |  |  |  |  |
| 507 | 42 | 1488.7459 | 497.2559 | 5520.40 | 3 | 1488.7540 | C12 | 12 | -8.12e-03 | -5.45 |
| 507 | 43 | 1220.6809 | 611.3477 | 6075.57 | 2 |  |  |  |  |  |
| 507 | 44 | 1135.5414 | 1136.5486 | 5599.88 | 1 | 1135.5477 | C9 | 9 | -6.38e-03 | -5.62 |
| 507 | 45 | 847.4584 | 848.4657 | 5637.62 | 1 | 847.4585 | C7 | 7 | -9.21e-05 | -0.11 |
| 507 | 46 | 650.3117 | 651.3190 | 7570.55 | 1 | 650.3039 | Z\_DOT7 | 23 | 7.81e-03 | 12.01 |
| 507 | 47 | 473.2941 | 474.3013 | 10707.04 | 1 | 473.2961 | C4 | 4 | -2.07e-03 | -4.36 |
| 507 | 48 | 1274.6914 | 638.3530 | 4105.45 | 2 |  |  |  |  |  |
| 507 | 49 | 778.4060 | 779.4133 | 4715.71 | 1 | 778.3988 | Z\_DOT8 | 22 | 7.15e-03 | 9.19 |
| 507 | 50 | 976.4926 | 489.2536 | 3461.24 | 2 |  |  |  |  |  |
| 507 | 51 | 1007.4840 | 504.7493 | 2833.18 | 2 | 1007.4892 | C8 | 8 | -5.19e-03 | -5.15 |
| 507 | 52 | 870.2118 | 871.2191 | 15553.78 | 1 |  |  |  |  |  |
| 507 | 53 | 1007.4836 | 1008.4908 | 3817.70 | 1 | 1007.4892 | C8 | 8 | -5.60e-03 | -5.56 |
| 507 | 54 | 746.4073 | 747.4146 | 2387.92 | 1 | 746.4108 | C6 | 6 | -3.50e-03 | -4.69 |
| 507 | 55 | 356.0581 | 357.0653 | 2206.08 | 1 |  |  |  |  |  |
| 507 | 56 | 723.0475 | 724.0547 | 1902.07 | 1 |  |  |  |  |  |
| 507 | 57 | 1098.6210 | 550.3178 | 2162.24 | 2 |  |  |  |  |  |

  

All proteins /
CsTx-13a Cupiennius salei toxin 13 isoform a /
Proteoform #40
