# Supplementary material for: The Dual Prey-Inactivation Strategy of Spiders—In-Depth Venomic Analysis of Cupiennius salei
Source: Toxins (Basel). 2019 Mar 19;11(3):167. doi: 10.3390/toxins11030167 (PMC6468893; doi:10.3390/toxins11030167)
Supplement: Supplementary file 1 [file toxins-11-00167-s001.zip › Supplementary Dataset EV1/20180328_f2_topdown_OTMS2_EThcD_NL_i02_ms2_proteoform_cutoff_html/prsms/prsm142.html]

Protein-Spectrum-Match for Spectrum #379


All proteins /
CsTx-1a\_S1 Cupiennius salei toxin 1 isoform a S1^ACsTx-1a\_S2 Cupiennius salei toxin 1 isoform a S2 /
Proteoform #8

## Protein-Spectrum-Match #142 for Spectrum #379

|  |  |  |  |  |  |
| --- | --- | --- | --- | --- | --- |
| PrSM ID: | 142 | Scan(s): | 508 | Precursor charge: | 6 |
| Precursor m/z: | 1195.8721 | Precursor mass: | 7169.1892 | Proteoform mass: | 7169.1856 |
| # matched peaks: | 10 | # matched fragment ions: | 10 | # unexpected modifications: | 0 |
| E-value: | 3.30e-09 | P-value: | 3.30e-09 | Q-value (Spectral FDR): | 0 |

  

|  |  |  |  |  |  |  |  |  |  |  |  |  |  |  |  |  |  |  |  |  |  |  |  |  |  |  |  |  |  |  |  |  |  |  |  |  |  |  |  |  |  |  |  |  |  |  |  |  |  |  |  |  |  |  |  |  |  |  |  |  |  |  |  |  |  |  |  |  |  |
| --- | --- | --- | --- | --- | --- | --- | --- | --- | --- | --- | --- | --- | --- | --- | --- | --- | --- | --- | --- | --- | --- | --- | --- | --- | --- | --- | --- | --- | --- | --- | --- | --- | --- | --- | --- | --- | --- | --- | --- | --- | --- | --- | --- | --- | --- | --- | --- | --- | --- | --- | --- | --- | --- | --- | --- | --- | --- | --- | --- | --- | --- | --- | --- | --- | --- | --- | --- | --- | --- |
|  | |  | | | | | | | | | | | | | | | | | | | | | | | | | | | | | | | | | | | | | | | | | | | | | | | | | | | | | | | | | | | | | | | | | | | |
| 1 |  |  | M |  | K |  | V |  | L |  | I |  | I |  | S |  | A |  | V |  | L |  |  | F |  | I |  | T |  | I |  | F |  | S |  | N |  | I |  | S |  | A |  |  | E |  | I |  | E |  | D |  | D |  | F |  | L |  | E |  | D |  | E |  | 30 |  |
|  | |  | | | | | | | | | | | | | | | | | | | | | | | | | | | | | | | | | | | | | | | | | | | | | | | | | | | | | | | | | | | | | | | | | | | |
| 31 |  |  | S |  | F |  | E |  | A |  | E |  | D |  | I |  | I |  | P |  | F |  |  | F |  | E |  | N |  | E |  | Q |  | A |  | R | ] | S |  | C |  | I |  |  | P | ⎫ | K | ⎫ | H | ⎫ | E | ⎫ | E | ⎫ | C |  | T |  | N |  | D |  | K |  | 60 |  |
|  | |  | | | | | | | | | | | | | | | | | | | | | | | | | | | | | | | | | | | | | | | | | | | | | | | | | | | | | | | | | | | | | | | | | | | |
| 61 |  |  | H | ⎫ | N | ⎫ | C |  | C |  | R |  | K |  | G |  | L |  | F |  | K |  | ⎫ | L |  | K | ⎫ | C |  | Q |  | C |  | S |  | T |  | F |  | D |  | D |  |  | E |  | S |  | G |  | Q |  | P |  | T |  | E |  | R |  | C |  | A |  | 90 |  |
|  | |  | | | | | | | | | | | | | | | | | | | | | | | | | | | | | | | | | | | | | | | | | | | | | | | | | | | | | | | | | | | | | | | | | | | |
| 91 |  |  | C |  | G |  | R |  | P |  | M |  | G | ⎩ | H |  | Q |  | A |  | I |  |  | E |  | T |  | G |  | L |  | N |  | I |  | F | [ | R |  | G |  | L |  |  | F |  | K |  | G |  | K |  | K |  | K |  | N |  | K |  | K |  | T |  | 120 |  |
|  | |  | | | | | | | | | | | | | | | | | | | | | | | | | | | | | | | | | | | | | | | | | | | | | | | | | | | | | | | | | | | | | | | | | | | |
| 121 |  |  | K |  | G |  | | | | 122 |  | | | | | | | | | | | | | | | | | | | | | | | | | | | | | | | | | | | | | | | | | | | | | | | | | | | | | | | |

Fixed PTMs: Carbamidomethylation [C49 C56 C63 C64 C73 C75 C89 C91 ]

  

All peaks (22)  Matched peaks (10)  Not matched peaks (12)

  

| Scan | Peak | Mono mass | Mono m/z | Intensity | Charge | Theoretical mass | Ion | Pos | Mass error | PPM error |
| --- | --- | --- | --- | --- | --- | --- | --- | --- | --- | --- |
| 508 | 1 | 3585.0668 | 1196.0296 | 96129.38 | 3 |  |  |  |  |  |
| 508 | 2 | 7112.1179 | 1423.4309 | 23941.10 | 5 |  |  |  |  |  |
| 508 | 3 | 7125.1233 | 1426.0319 | 5033.56 | 5 |  |  |  |  |  |
| 508 | 4 | 6976.0858 | 1396.2244 | 2321.25 | 5 |  |  |  |  |  |
| 508 | 5 | 1866.7978 | 934.4062 | 3463.43 | 2 | 1866.8101 | C15 | 15 | -0.0122 | -6.56 |
| 508 | 6 | 1752.7550 | 877.3848 | 4065.04 | 2 | 1752.7671 | C14 | 14 | -0.0121 | -6.90 |
| 508 | 7 | 7153.1268 | 1431.6326 | 2985.85 | 5 |  |  |  |  |  |
| 508 | 8 | 7079.1264 | 1416.8326 | 2623.58 | 5 |  |  |  |  |  |
| 508 | 9 | 602.3175 | 603.3248 | 3891.94 | 1 | 602.3210 | C5 | 5 | -3.47e-03 | -5.76 |
| 508 | 10 | 7097.0985 | 1420.4270 | 1895.72 | 5 |  |  |  |  |  |
| 508 | 11 | 739.3755 | 740.3828 | 2564.33 | 1 | 739.3799 | C6 | 6 | -4.34e-03 | -5.87 |
| 508 | 12 | 3157.4946 | 1053.5055 | 797.77 | 3 | 3157.5153 | C25 | 25 | -0.0207 | -6.56 |
| 508 | 13 | 868.4172 | 869.4245 | 1816.92 | 1 | 868.4225 | C7 | 7 | -5.24e-03 | -6.03 |
| 508 | 14 | 2916.3178 | 973.1132 | 1448.10 | 3 | 2916.3363 | C23 | 23 | -0.0185 | -6.35 |
| 508 | 15 | 7035.1161 | 1408.0305 | 1459.07 | 5 |  |  |  |  |  |
| 508 | 16 | 997.4579 | 998.4652 | 993.77 | 1 | 997.4651 | C8 | 8 | -7.12e-03 | -7.14 |
| 508 | 17 | 474.2234 | 475.2307 | 760.01 | 1 | 474.2260 | C4 | 4 | -2.63e-03 | -5.54 |
| 508 | 18 | 1148.8598 | 1149.8671 | 358.45 | 1 |  |  |  |  |  |
| 508 | 19 | 1405.0212 | 1406.0285 | 899.13 | 1 |  |  |  |  |  |
| 508 | 20 | 1225.6134 | 1226.6207 | 537.75 | 1 | 1225.6217 | Z\_DOT11 | 49 | -8.36e-03 | -6.82 |
| 508 | 21 | 1194.8590 | 1195.8663 | 50372.46 | 1 |  |  |  |  |  |
| 508 | 22 | 1486.3839 | 1487.3912 | 356.83 | 1 |  |  |  |  |  |

  

All proteins /
CsTx-1a\_S1 Cupiennius salei toxin 1 isoform a S1^ACsTx-1a\_S2 Cupiennius salei toxin 1 isoform a S2 /
Proteoform #8
