# Supplementary material for: The Dual Prey-Inactivation Strategy of Spiders—In-Depth Venomic Analysis of Cupiennius salei
Source: Toxins (Basel). 2019 Mar 19;11(3):167. doi: 10.3390/toxins11030167 (PMC6468893; doi:10.3390/toxins11030167)
Supplement: Supplementary file 1 [file toxins-11-00167-s001.zip › Supplementary Dataset EV1/20180328_f2_topdown_OTMS2_EThcD_NL_i02_ms2_proteoform_cutoff_html/prsms/prsm143.html]

Protein-Spectrum-Match for Spectrum #380


All proteins /
CsTx-1a\_S1 Cupiennius salei toxin 1 isoform a S1^ACsTx-1a\_S2 Cupiennius salei toxin 1 isoform a S2 /
Proteoform #8

## Protein-Spectrum-Match #143 for Spectrum #380

|  |  |  |  |  |  |
| --- | --- | --- | --- | --- | --- |
| PrSM ID: | 143 | Scan(s): | 509 | Precursor charge: | 7 |
| Precursor m/z: | 1025.1769 | Precursor mass: | 7169.1871 | Proteoform mass: | 7169.1856 |
| # matched peaks: | 28 | # matched fragment ions: | 27 | # unexpected modifications: | 0 |
| E-value: | 4.70e-27 | P-value: | 4.70e-27 | Q-value (Spectral FDR): | 0 |

  

|  |  |  |  |  |  |  |  |  |  |  |  |  |  |  |  |  |  |  |  |  |  |  |  |  |  |  |  |  |  |  |  |  |  |  |  |  |  |  |  |  |  |  |  |  |  |  |  |  |  |  |  |  |  |  |  |  |  |  |  |  |  |  |  |  |  |  |  |  |  |
| --- | --- | --- | --- | --- | --- | --- | --- | --- | --- | --- | --- | --- | --- | --- | --- | --- | --- | --- | --- | --- | --- | --- | --- | --- | --- | --- | --- | --- | --- | --- | --- | --- | --- | --- | --- | --- | --- | --- | --- | --- | --- | --- | --- | --- | --- | --- | --- | --- | --- | --- | --- | --- | --- | --- | --- | --- | --- | --- | --- | --- | --- | --- | --- | --- | --- | --- | --- | --- | --- |
|  | |  | | | | | | | | | | | | | | | | | | | | | | | | | | | | | | | | | | | | | | | | | | | | | | | | | | | | | | | | | | | | | | | | | | | |
| 1 |  |  | M |  | K |  | V |  | L |  | I |  | I |  | S |  | A |  | V |  | L |  |  | F |  | I |  | T |  | I |  | F |  | S |  | N |  | I |  | S |  | A |  |  | E |  | I |  | E |  | D |  | D |  | F |  | L |  | E |  | D |  | E |  | 30 |  |
|  | |  | | | | | | | | | | | | | | | | | | | | | | | | | | | | | | | | | | | | | | | | | | | | | | | | | | | | | | | | | | | | | | | | | | | |
| 31 |  |  | S |  | F |  | E |  | A |  | E |  | D |  | I |  | I |  | P |  | F |  |  | F |  | E |  | N |  | E |  | Q |  | A |  | R | ] | S | ⎩ | C |  | I |  | ⎩ | P | ⎱ | K | ⎱ | H | ⎫ | E | ⎱ | E | ⎫ | C | ⎩ | T |  | N |  | D |  | K |  | 60 |  |
|  | |  | | | | | | | | | | | | | | | | | | | | | | | | | | | | | | | | | | | | | | | | | | | | | | | | | | | | | | | | | | | | | | | | | | | |
| 61 |  |  | H | ⎫ | N |  | C |  | C |  | R |  | K |  | G |  | L |  | F |  | K |  | ⎫ | L |  | K | ⎫ | C |  | Q | ⎫ | C |  | S |  | T |  | F | ⎫ | D | ⎩ | D |  |  | E |  | S |  | G | ⎱ | Q |  | P |  | T |  | E |  | R |  | C |  | A |  | 90 |  |
|  | |  | | | | | | | | | | | | | | | | | | | | | | | | | | | | | | | | | | | | | | | | | | | | | | | | | | | | | | | | | | | | | | | | | | | |
| 91 |  |  | C |  | G | ⎱ | R |  | P | ⎫ | M | ⎫ | G | ⎫ | H | ⎫ | Q | ⎫ | A |  | I |  |  | E |  | T |  | G |  | L |  | N |  | I | ⎫ | F | [ | R |  | G |  | L |  |  | F |  | K |  | G |  | K |  | K |  | K |  | N |  | K |  | K |  | T |  | 120 |  |
|  | |  | | | | | | | | | | | | | | | | | | | | | | | | | | | | | | | | | | | | | | | | | | | | | | | | | | | | | | | | | | | | | | | | | | | |
| 121 |  |  | K |  | G |  | | | | 122 |  | | | | | | | | | | | | | | | | | | | | | | | | | | | | | | | | | | | | | | | | | | | | | | | | | | | | | | | |

Fixed PTMs: Carbamidomethylation [C49 C56 C63 C64 C73 C75 C89 C91 ]

  

All peaks (72)  Matched peaks (28)  Not matched peaks (44)

  

| Scan | Peak | Mono mass | Mono m/z | Intensity | Charge | Theoretical mass | Ion | Pos | Mass error | PPM error |
| --- | --- | --- | --- | --- | --- | --- | --- | --- | --- | --- |
| 509 | 1 | 7112.1138 | 1186.3596 | 94363.26 | 6 |  |  |  |  |  |
| 509 | 2 | 3584.5684 | 1195.8634 | 59042.09 | 3 |  |  |  |  |  |
| 509 | 3 | 3074.7533 | 1025.9250 | 52320.34 | 3 |  |  |  |  |  |
| 509 | 4 | 7125.1297 | 1188.5289 | 21180.32 | 6 |  |  |  |  |  |
| 509 | 5 | 2390.7159 | 1196.3652 | 42951.49 | 2 |  |  |  |  |  |
| 509 | 6 | 1024.5932 | 1025.6005 | 106455.39 | 1 |  |  |  |  |  |
| 509 | 7 | 7152.1267 | 1193.0284 | 13006.14 | 6 |  |  |  |  |  |
| 509 | 8 | 7112.1222 | 1423.4317 | 10888.78 | 5 |  |  |  |  |  |
| 509 | 9 | 3157.4957 | 1053.5059 | 10501.84 | 3 | 3157.5153 | C25 | 25 | -0.0196 | -6.21 |
| 509 | 10 | 7080.1249 | 1181.0281 | 9697.43 | 6 |  |  |  |  |  |
| 509 | 11 | 2782.3033 | 928.4417 | 8023.29 | 3 |  |  |  |  |  |
| 509 | 12 | 1752.7561 | 877.3853 | 7334.39 | 2 | 1752.7671 | C14 | 14 | -0.0110 | -6.27 |
| 509 | 13 | 7096.0825 | 1183.6877 | 5362.91 | 6 |  |  |  |  |  |
| 509 | 14 | 6977.0985 | 1163.8570 | 6481.65 | 6 |  |  |  |  |  |
| 509 | 15 | 5944.5275 | 1189.9128 | 5768.41 | 5 | 5944.5717 | C49 | 49 | -0.0443 | -7.45 |
| 509 | 16 | 7035.1198 | 1173.5272 | 5765.80 | 6 |  |  |  |  |  |
| 509 | 17 | 4443.9025 | 1111.9829 | 4727.59 | 4 | 4443.9333 | C36 | 36 | -0.0308 | -6.93 |
| 509 | 18 | 6302.7276 | 1261.5528 | 4449.94 | 5 | 6301.7710 | Z\_DOT53 | 7 | -0.0458 | -7.26 |
| 509 | 19 | 868.4176 | 869.4249 | 8087.71 | 1 | 868.4225 | C7 | 7 | -4.87e-03 | -5.61 |
| 509 | 20 | 6080.5911 | 1217.1255 | 6743.97 | 5 | 6081.6306 | C50 | 50 | -0.0372 | -6.11 |
| 509 | 21 | 2872.3033 | 958.4417 | 5497.98 | 3 |  |  |  |  |  |
| 509 | 22 | 7021.1119 | 1171.1926 | 7753.34 | 6 | 7021.1332 | C59 | 59 | -0.0213 | -3.04 |
| 509 | 23 | 6209.6426 | 1242.9358 | 5649.57 | 5 | 6209.6892 | C51 | 51 | -0.0466 | -7.51 |
| 509 | 24 | 7004.0569 | 1001.5868 | 3989.26 | 7 |  |  |  |  |  |
| 509 | 25 | 5886.5079 | 1178.3089 | 7444.52 | 5 | 5887.5503 | C48 | 48 | -0.0400 | -6.80 |
| 509 | 26 | 3445.5822 | 1149.5347 | 3354.16 | 3 | 3445.6046 | C27 | 27 | -0.0223 | -6.48 |
| 509 | 27 | 6695.9174 | 1340.1908 | 2572.29 | 5 | 6695.9675 | Z\_DOT56 | 4 | -0.0501 | -7.48 |
| 509 | 28 | 6568.8333 | 1314.7739 | 3295.11 | 5 | 6567.8725 | Z\_DOT55 | 5 | -0.0416 | -6.34 |
| 509 | 29 | 2916.3200 | 973.1139 | 3684.26 | 3 | 2916.3363 | C23 | 23 | -0.0163 | -5.59 |
| 509 | 30 | 1865.7955 | 933.9050 | 4499.41 | 2 |  |  |  |  |  |
| 509 | 31 | 7065.1110 | 1178.5258 | 5640.82 | 6 | 7066.1350 | Z\_DOT59 | 1 | -0.0216 | -3.06 |
| 509 | 32 | 602.3177 | 603.3250 | 7441.43 | 1 | 602.3210 | C5 | 5 | -3.23e-03 | -5.36 |
| 509 | 33 | 739.3757 | 740.3829 | 4085.27 | 1 | 739.3799 | C6 | 6 | -4.22e-03 | -5.70 |
| 509 | 34 | 3922.6539 | 1308.5586 | 2606.08 | 3 |  |  |  |  |  |
| 509 | 35 | 7054.0904 | 1411.8254 | 3726.18 | 5 |  |  |  |  |  |
| 509 | 36 | 5756.4725 | 1152.3018 | 2509.71 | 5 | 5756.5098 | C47 | 47 | -0.0373 | -6.47 |
| 509 | 37 | 7126.1317 | 1426.2336 | 3685.90 | 5 |  |  |  |  |  |
| 509 | 38 | 3183.5143 | 1062.1787 | 2400.44 | 3 |  |  |  |  |  |
| 509 | 39 | 7154.1209 | 1431.8315 | 2837.44 | 5 |  |  |  |  |  |
| 509 | 40 | 3318.5260 | 1107.1826 | 2217.65 | 3 |  |  |  |  |  |
| 509 | 41 | 6243.7088 | 1249.7490 | 2052.17 | 5 |  |  |  |  |  |
| 509 | 42 | 2025.8262 | 1013.9204 | 1840.51 | 2 |  |  |  |  |  |
| 509 | 43 | 5503.3204 | 1101.6714 | 2134.53 | 5 | 5503.3559 | C45 | 45 | -0.0355 | -6.45 |
| 509 | 44 | 3634.5617 | 1212.5278 | 2244.87 | 3 |  |  |  |  |  |
| 509 | 45 | 4269.8699 | 1424.2972 | 3667.11 | 3 |  |  |  |  |  |
| 509 | 46 | 2743.2015 | 915.4078 | 1440.08 | 3 |  |  |  |  |  |
| 509 | 47 | 3074.7513 | 1538.3829 | 2189.69 | 2 |  |  |  |  |  |
| 509 | 48 | 997.4593 | 998.4666 | 2732.27 | 1 | 997.4651 | C8 | 8 | -5.72e-03 | -5.73 |
| 509 | 49 | 6791.9506 | 1132.9990 | 1549.32 | 6 | 6793.0202 | Z\_DOT57 | 3 | -0.0673 | -9.90 |
| 509 | 50 | 3527.5226 | 1176.8481 | 2380.15 | 3 |  |  |  |  |  |
| 509 | 51 | 3867.6377 | 1290.2198 | 893.65 | 3 |  |  |  |  |  |
| 509 | 52 | 7151.1108 | 1022.5945 | 1524.13 | 7 |  |  |  |  |  |
| 509 | 53 | 2726.2404 | 909.7541 | 836.30 | 3 | 2726.2602 | Z\_DOT24 | 36 | -0.0198 | -7.26 |
| 509 | 54 | 2726.2456 | 1364.1301 | 1364.38 | 2 | 2726.2602 | Z\_DOT24 | 36 | -0.0145 | -5.33 |
| 509 | 55 | 7165.1172 | 1024.5954 | 210876.77 | 7 |  |  |  |  |  |
| 509 | 56 | 474.2235 | 475.2307 | 995.69 | 1 | 474.2260 | C4 | 4 | -2.54e-03 | -5.35 |
| 509 | 57 | 3940.7540 | 986.1958 | 1211.52 | 4 | 3940.7834 | C31 | 31 | -0.0294 | -7.45 |
| 509 | 58 | 3114.3652 | 1039.1290 | 698.80 | 3 | 3114.3832 | Z\_DOT28 | 32 | -0.0180 | -5.78 |
| 509 | 59 | 6011.6577 | 1203.3388 | 1134.62 | 5 | 6012.6978 | Z\_DOT51 | 9 | -0.0377 | -6.27 |
| 509 | 60 | 1248.9444 | 1249.9517 | 875.41 | 1 |  |  |  |  |  |
| 509 | 61 | 1666.8252 | 834.4199 | 962.11 | 2 | 1666.8376 | Z\_DOT15 | 45 | -0.0123 | -7.40 |
| 509 | 62 | 1151.4907 | 1152.4980 | 1163.82 | 1 |  |  |  |  |  |
| 509 | 63 | 7112.1105 | 1017.0231 | 1358.09 | 7 |  |  |  |  |  |
| 509 | 64 | 1135.5209 | 1136.5281 | 499.14 | 1 |  |  |  |  |  |
| 509 | 65 | 1226.6217 | 1227.6290 | 517.62 | 1 |  |  |  |  |  |
| 509 | 66 | 3392.4674 | 1131.8297 | 888.29 | 3 |  |  |  |  |  |
| 509 | 67 | 663.3556 | 664.3629 | 491.71 | 1 |  |  |  |  |  |
| 509 | 68 | 1435.2339 | 1436.2411 | 2439.08 | 1 |  |  |  |  |  |
| 509 | 69 | 1286.7551 | 1287.7624 | 452.75 | 1 |  |  |  |  |  |
| 509 | 70 | 1793.8764 | 897.9455 | 858.31 | 2 |  |  |  |  |  |
| 509 | 71 | 6029.6930 | 1206.9459 | 1149.29 | 5 |  |  |  |  |  |
| 509 | 72 | 1263.3341 | 1264.3414 | 479.61 | 1 |  |  |  |  |  |

  

All proteins /
CsTx-1a\_S1 Cupiennius salei toxin 1 isoform a S1^ACsTx-1a\_S2 Cupiennius salei toxin 1 isoform a S2 /
Proteoform #8
