# Supplementary material for: The Dual Prey-Inactivation Strategy of Spiders—In-Depth Venomic Analysis of Cupiennius salei
Source: Toxins (Basel). 2019 Mar 19;11(3):167. doi: 10.3390/toxins11030167 (PMC6468893; doi:10.3390/toxins11030167)
Supplement: Supplementary file 1 [file toxins-11-00167-s001.zip › Supplementary Dataset EV1/20180328_f2_topdown_OTMS2_EThcD_NL_i02_ms2_proteoform_cutoff_html/prsms/prsm144.html]

Protein-Spectrum-Match for Spectrum #381


All proteins /
CsTx-12b Cupiennius salei toxin 12 isoform b /
Proteoform #47

## Protein-Spectrum-Match #144 for Spectrum #381

|  |  |  |  |  |  |
| --- | --- | --- | --- | --- | --- |
| PrSM ID: | 144 | Scan(s): | 511 | Precursor charge: | 6 |
| Precursor m/z: | 571.9898 | Precursor mass: | 3425.8952 | Proteoform mass: | 3425.8952 |
| # matched peaks: | 32 | # matched fragment ions: | 27 | # unexpected modifications: | 1 |
| E-value: | 5.33e-21 | P-value: | 5.33e-21 | Q-value (Spectral FDR): | 0 |

  

|  |  |  |  |  |  |  |  |  |  |  |  |  |  |  |  |  |  |  |  |  |  |  |  |  |  |  |  |  |  |  |  |  |  |  |  |  |  |  |  |  |  |  |  |  |  |  |  |  |  |  |  |  |  |  |  |  |  |  |  |  |  |  |  |  |  |  |
| --- | --- | --- | --- | --- | --- | --- | --- | --- | --- | --- | --- | --- | --- | --- | --- | --- | --- | --- | --- | --- | --- | --- | --- | --- | --- | --- | --- | --- | --- | --- | --- | --- | --- | --- | --- | --- | --- | --- | --- | --- | --- | --- | --- | --- | --- | --- | --- | --- | --- | --- | --- | --- | --- | --- | --- | --- | --- | --- | --- | --- | --- | --- | --- | --- | --- | --- |
|  | | ... 30 amino acid residues are skipped at the N-terminus ... | | | | | | | | | | | | | | | | | | | | | | | | | | | | | | | | | | | | | | | | | | | | | | | | | | | | | | | | | | | | | |  | | |
|  | |  | | | | | | | | | | | | | | | | | | | | | | | | | | | | | | | | | | | | | | | | | | | | | | | | | | | | | | | | | | | | | | | | | | | |
| 31 |  |  | S |  | F |  | E |  | A |  | D |  | D |  | V |  | I |  | P |  | F |  |  | L |  | A |  | R |  | E |  | Q |  | V |  | R |  | S |  | D |  | C |  |  | T |  | L |  | R |  | N |  | H |  | D |  | C |  | T |  | D |  | D |  | 60 |  |
|  | |  | | | | | | | | | | | | | | | | | | | | | | | | | | | | | | | | | | | | | | | | | | | | | | | | | | | | | | | | | | | | | | | | | | | |
| 61 |  |  | R |  | H |  | S |  | C |  | C |  | R |  | S |  | K |  | M |  | F |  |  | K |  | D |  | V |  | C |  | K |  | C |  | F |  | Y |  | P |  | S |  |  | Q |  | R |  | S |  | D |  | T |  | A |  | R | ] | A | ⎩ | K | ⎩ | K |  | 90 |  |
|  | |  | | | | | | | | | | | | | | | | | | | | | | | | | | | | | | | | | | | | | | | | | | | | | | | | | | | | | -58.01 | | | | | | | | | | | |
| 91 |  | ⎫ | E | ⎫ | L | ⎫ | C |  | T | ⎫ | C | ⎫ | Q | ⎫ | Q | ⎫ | D | ⎱ | K |  | H |  |  | L | ⎫ | K | ⎱ | Y |  | I | ⎱ | E | ⎫ | K |  | G | ⎫ | L |  | Q | ⎱ | K |  | ⎫ | A | ⎱ | K | ⎫ | V | ⎫ | L | ⎫ | V | ⎫ | A |  | G |  | | 117 |  | | | | | |

Fixed PTMs: Carbamidomethylation [C93 C95 ]   
  
     Unexpected modifications:   Unknown [-58.01]

  

All peaks (57)  Matched peaks (32)  Not matched peaks (25)

  

| Scan | Peak | Mono mass | Mono m/z | Intensity | Charge | Theoretical mass | Ion | Pos | Mass error | PPM error |
| --- | --- | --- | --- | --- | --- | --- | --- | --- | --- | --- |
| 511 | 1 | 3368.8608 | 674.7794 | 401104.84 | 5 |  |  |  |  |  |
| 511 | 2 | 1713.4489 | 572.1569 | 967808.97 | 3 |  |  |  |  |  |
| 511 | 3 | 3142.6966 | 786.6814 | 144806.02 | 4 | 3142.7106 | C26 | 26 | -0.0141 | -4.48 |
| 511 | 4 | 3354.8486 | 671.9770 | 130024.59 | 5 | 3354.8631 | C28 | 28 | -0.0145 | -4.31 |
| 511 | 5 | 3368.8634 | 843.2231 | 108354.72 | 4 |  |  |  |  |  |
| 511 | 6 | 2161.1039 | 721.3752 | 112317.75 | 3 | 2161.1135 | C17 | 17 | -9.67e-03 | -4.47 |
| 511 | 7 | 2048.2662 | 683.7627 | 117704.99 | 3 | 2048.2698 | Z\_DOT19 | 11 | -3.57e-03 | -1.74 |
| 511 | 8 | 571.3143 | 572.3216 | 703202.03 | 1 |  |  |  |  |  |
| 511 | 9 | 2475.2620 | 826.0946 | 81297.52 | 3 | 2475.2726 | C20 | 20 | -0.0106 | -4.28 |
| 511 | 10 | 3409.8669 | 682.9807 | 69433.14 | 5 |  |  |  |  |  |
| 511 | 11 | 3210.7351 | 803.6911 | 70038.27 | 4 | 3210.7445 | Z\_DOT28 | 2 | -9.36e-03 | -2.92 |
| 511 | 12 | 2290.1460 | 764.3893 | 79602.15 | 3 | 2290.1561 | C18 | 18 | -0.0101 | -4.43 |
| 511 | 13 | 3408.8657 | 569.1516 | 64816.31 | 6 |  |  |  |  |  |
| 511 | 14 | 1884.9578 | 629.3265 | 104212.47 | 3 | 1884.9662 | C15 | 15 | -8.38e-03 | -4.45 |
| 511 | 15 | 3338.8281 | 668.7729 | 69218.60 | 5 | 3338.8395 | Z\_DOT29 | 1 | -0.0113 | -3.39 |
| 511 | 16 | 2915.5335 | 729.8907 | 58883.51 | 4 | 2915.5473 | C24 | 24 | -0.0138 | -4.72 |
| 511 | 17 | 1541.9353 | 771.9749 | 90248.14 | 2 | 1541.9369 | Z\_DOT15 | 15 | -1.58e-03 | -1.03 |
| 511 | 18 | 1378.6266 | 690.3206 | 83636.62 | 2 | 1378.6333 | C11 | 11 | -6.65e-03 | -4.82 |
| 511 | 19 | 3381.8710 | 677.3815 | 41201.91 | 5 |  |  |  |  |  |
| 511 | 20 | 2844.4966 | 712.1314 | 49812.11 | 4 | 2844.5102 | C23 | 23 | -0.0135 | -4.75 |
| 511 | 21 | 3338.8305 | 835.7149 | 67978.50 | 4 | 3338.8395 | Z\_DOT29 | 1 | -9.00e-03 | -2.70 |
| 511 | 22 | 2361.3792 | 591.3521 | 43241.44 | 4 |  |  |  |  |  |
| 511 | 23 | 3382.8778 | 846.7267 | 44609.72 | 4 |  |  |  |  |  |
| 511 | 24 | 2716.4036 | 906.4751 | 45033.32 | 3 | 2716.4152 | C22 | 22 | -0.0116 | -4.26 |
| 511 | 25 | 3255.7796 | 814.9522 | 32887.18 | 4 | 3255.7947 | C27 | 27 | -0.0151 | -4.64 |
| 511 | 26 | 3424.8860 | 685.9845 | 508085.84 | 5 |  |  |  |  |  |
| 511 | 27 | 2716.4029 | 680.1080 | 33817.91 | 4 | 2716.4152 | C22 | 22 | -0.0123 | -4.54 |
| 511 | 28 | 3410.8747 | 853.7259 | 38505.53 | 4 |  |  |  |  |  |
| 511 | 29 | 1265.7890 | 633.9018 | 53302.54 | 2 | 1265.7895 | Z\_DOT13 | 17 | -5.58e-04 | -0.44 |
| 511 | 30 | 2435.4261 | 609.8638 | 33020.72 | 4 |  |  |  |  |  |
| 511 | 31 | 3354.8485 | 839.7194 | 29236.86 | 4 | 3354.8631 | C28 | 28 | -0.0146 | -4.34 |
| 511 | 32 | 3043.6282 | 761.9143 | 45292.02 | 4 | 3043.6422 | C25 | 25 | -0.0140 | -4.60 |
| 511 | 33 | 3226.7535 | 807.6956 | 29371.24 | 4 |  |  |  |  |  |
| 511 | 34 | 1756.8636 | 879.4391 | 39721.67 | 2 | 1756.8712 | C14 | 14 | -7.64e-03 | -4.35 |
| 511 | 35 | 1557.9540 | 779.9843 | 45324.60 | 2 |  |  |  |  |  |
| 511 | 36 | 685.3787 | 686.3860 | 116239.65 | 1 |  |  |  |  |  |
| 511 | 37 | 908.5772 | 455.2959 | 41765.70 | 2 |  |  |  |  |  |
| 511 | 38 | 1007.4855 | 1008.4927 | 26539.70 | 1 | 1007.4892 | C8 | 8 | -3.71e-03 | -3.68 |
| 511 | 39 | 710.4903 | 711.4976 | 17953.36 | 1 | 710.4879 | Z\_DOT8 | 22 | 2.46e-03 | 3.46 |
| 511 | 40 | 1206.7763 | 604.3954 | 16426.99 | 2 |  |  |  |  |  |
| 511 | 41 | 1349.8327 | 675.9236 | 9909.40 | 2 |  |  |  |  |  |
| 511 | 42 | 1135.5437 | 1136.5510 | 12210.35 | 1 | 1135.5477 | C9 | 9 | -4.06e-03 | -3.58 |
| 511 | 43 | 847.4551 | 848.4623 | 14511.31 | 1 | 847.4585 | C7 | 7 | -3.45e-03 | -4.07 |
| 511 | 44 | 473.2947 | 474.3019 | 11979.86 | 1 | 473.2961 | C4 | 4 | -1.46e-03 | -3.08 |
| 511 | 45 | 780.5192 | 391.2669 | 5589.40 | 2 |  |  |  |  |  |
| 511 | 46 | 873.4707 | 874.4780 | 6901.43 | 1 |  |  |  |  |  |
| 511 | 47 | 586.3780 | 587.3853 | 8743.99 | 1 | 586.3802 | C5 | 5 | -2.14e-03 | -3.65 |
| 511 | 48 | 511.3591 | 512.3664 | 10104.82 | 1 | 511.3558 | Z\_DOT6 | 24 | 3.30e-03 | 6.46 |
| 511 | 49 | 967.6502 | 484.8324 | 5342.42 | 2 |  |  |  |  |  |
| 511 | 50 | 726.5090 | 727.5162 | 5474.10 | 1 |  |  |  |  |  |
| 511 | 51 | 1007.4852 | 504.7499 | 6858.48 | 2 | 1007.4892 | C8 | 8 | -3.97e-03 | -3.94 |
| 511 | 52 | 1078.6824 | 540.3485 | 4831.78 | 2 |  |  |  |  |  |
| 511 | 53 | 1024.6714 | 513.3430 | 4003.93 | 2 |  |  |  |  |  |
| 511 | 54 | 344.2526 | 345.2599 | 5727.49 | 1 | 344.2535 | C3 | 3 | -9.19e-04 | -2.67 |
| 511 | 55 | 1263.6025 | 1264.6098 | 3521.61 | 1 | 1263.6063 | C10 | 10 | -3.80e-03 | -3.01 |
| 511 | 56 | 1378.6289 | 1379.6362 | 2667.61 | 1 | 1378.6333 | C11 | 11 | -4.37e-03 | -3.17 |
| 511 | 57 | 891.9626 | 892.9698 | 3465.71 | 1 |  |  |  |  |  |

  

All proteins /
CsTx-12b Cupiennius salei toxin 12 isoform b /
Proteoform #47
