# Supplementary material for: The Dual Prey-Inactivation Strategy of Spiders—In-Depth Venomic Analysis of Cupiennius salei
Source: Toxins (Basel). 2019 Mar 19;11(3):167. doi: 10.3390/toxins11030167 (PMC6468893; doi:10.3390/toxins11030167)
Supplement: Supplementary file 1 [file toxins-11-00167-s001.zip › Supplementary Dataset EV1/20180328_f2_topdown_OTMS2_EThcD_NL_i02_ms2_proteoform_cutoff_html/prsms/prsm145.html]

Protein-Spectrum-Match for Spectrum #382


All proteins /
CsTx-13a Cupiennius salei toxin 13 isoform a /
Proteoform #40

## Protein-Spectrum-Match #145 for Spectrum #382

|  |  |  |  |  |  |
| --- | --- | --- | --- | --- | --- |
| PrSM ID: | 145 | Scan(s): | 512 | Precursor charge: | 6 |
| Precursor m/z: | 580.3139 | Precursor mass: | 3475.8398 | Proteoform mass: | 3475.8348 |
| # matched peaks: | 34 | # matched fragment ions: | 29 | # unexpected modifications: | 1 |
| E-value: | 5.33e-22 | P-value: | 5.33e-22 | Q-value (Spectral FDR): | 0 |

  

|  |  |  |  |  |  |  |  |  |  |  |  |  |  |  |  |  |  |  |  |  |  |  |  |  |  |  |  |  |  |  |  |  |  |  |  |  |  |  |  |  |  |  |  |  |  |  |  |  |  |  |  |  |  |  |  |  |  |  |  |  |  |  |  |  |  |  |
| --- | --- | --- | --- | --- | --- | --- | --- | --- | --- | --- | --- | --- | --- | --- | --- | --- | --- | --- | --- | --- | --- | --- | --- | --- | --- | --- | --- | --- | --- | --- | --- | --- | --- | --- | --- | --- | --- | --- | --- | --- | --- | --- | --- | --- | --- | --- | --- | --- | --- | --- | --- | --- | --- | --- | --- | --- | --- | --- | --- | --- | --- | --- | --- | --- | --- | --- |
|  | | ... 30 amino acid residues are skipped at the N-terminus ... | | | | | | | | | | | | | | | | | | | | | | | | | | | | | | | | | | | | | | | | | | | | | | | | | | | | | | | | | | | | | |  | | |
|  | |  | | | | | | | | | | | | | | | | | | | | | | | | | | | | | | | | | | | | | | | | | | | | | | | | | | | | | | | | | | | | | | | | | | | |
| 31 |  |  | S |  | F |  | E |  | A |  | D |  | D |  | I |  | I |  | P |  | F |  |  | I |  | A |  | K |  | E |  | Q |  | V |  | R |  | S |  | D |  | C |  |  | T |  | L |  | R |  | N |  | H |  | D |  | C |  | T |  | D |  | D |  | 60 |  |
|  | |  | | | | | | | | | | | | | | | | | | | | | | | | | | | | | | | | | | | | | | | | | | | | | | | | | | | | | | | | | | | | | | | | | | | |
| 61 |  |  | R |  | H |  | S |  | C |  | C |  | R |  | S |  | K |  | M |  | F |  |  | K |  | D |  | V |  | C |  | T |  | C |  | F |  | Y |  | P |  | S |  |  | Q |  | R |  | S |  | E |  | T |  | A |  | R | ] | A | ⎩ | K | ⎩ | K |  | 90 |  |
|  | |  | | | | | | | | | | | | | | | | | | | | | | | | | | | | | | | | | | | | | | | | | | | | | | | | | | | -58.01 | | | | | | | | | | | | | |
| 91 |  | ⎫ | E | ⎱ | L |  | C | ⎫ | T |  | C | ⎫ | Q | ⎱ | Q |  | P | ⎱ | K | ⎫ | H |  |  | L | ⎫ | K | ⎱ | Y |  | I | ⎱ | E | ⎱ | K | ⎩ | G | ⎫ | L |  | Q | ⎱ | K |  | ⎱ | A | ⎫ | K | ⎫ | D | ⎫ | Y | ⎫ | A |  | T |  | G |  | | 117 |  | | | | | |

Fixed PTMs: Carbamidomethylation [C93 C95 ]   
  
     Unexpected modifications:   Unknown [-58.01]

  

All peaks (57)  Matched peaks (34)  Not matched peaks (23)

  

| Scan | Peak | Mono mass | Mono m/z | Intensity | Charge | Theoretical mass | Ion | Pos | Mass error | PPM error |
| --- | --- | --- | --- | --- | --- | --- | --- | --- | --- | --- |
| 512 | 1 | 3418.8003 | 684.7673 | 90725.94 | 5 |  |  |  |  |  |
| 512 | 2 | 3474.8266 | 580.1450 | 165877.69 | 6 |  |  |  |  |  |
| 512 | 3 | 3140.6751 | 786.1760 | 39768.34 | 4 | 3140.6950 | C26 | 26 | -0.0199 | -6.34 |
| 512 | 4 | 2272.1687 | 758.3968 | 31966.24 | 3 | 2272.1820 | C18 | 18 | -0.0132 | -5.83 |
| 512 | 5 | 3025.6497 | 757.4197 | 38351.07 | 4 | 3025.6680 | C25 | 25 | -0.0183 | -6.06 |
| 512 | 6 | 3418.8019 | 855.7077 | 28315.02 | 4 |  |  |  |  |  |
| 512 | 7 | 2698.4258 | 900.4825 | 25038.94 | 3 | 2698.4410 | C22 | 22 | -0.0152 | -5.65 |
| 512 | 8 | 1158.9435 | 580.4790 | 136101.63 | 2 |  |  |  |  |  |
| 512 | 9 | 1866.9814 | 623.3344 | 33674.79 | 3 | 1866.9920 | C15 | 15 | -0.0106 | -5.67 |
| 512 | 10 | 1609.8500 | 805.9323 | 30154.76 | 2 | 1609.8507 | Z\_DOT15 | 15 | -6.76e-04 | -0.42 |
| 512 | 11 | 3260.6714 | 816.1751 | 17762.49 | 4 | 3260.6841 | Z\_DOT28 | 2 | -0.0127 | -3.88 |
| 512 | 12 | 2116.1795 | 706.4004 | 25156.77 | 3 | 2116.1836 | Z\_DOT19 | 11 | -4.06e-03 | -1.92 |
| 512 | 13 | 2143.1271 | 715.3830 | 24686.99 | 3 | 2143.1394 | C17 | 17 | -0.0122 | -5.71 |
| 512 | 14 | 3459.8044 | 692.9681 | 21785.31 | 5 |  |  |  |  |  |
| 512 | 15 | 579.6384 | 580.6457 | 119287.93 | 1 |  |  |  |  |  |
| 512 | 16 | 2800.4512 | 701.1201 | 18878.10 | 4 |  |  |  |  |  |
| 512 | 17 | 2826.5193 | 707.6371 | 20649.40 | 4 | 2826.5360 | C23 | 23 | -0.0166 | -5.89 |
| 512 | 18 | 3303.7393 | 826.9421 | 17169.02 | 4 | 3303.7583 | C27 | 27 | -0.0190 | -5.75 |
| 512 | 19 | 2341.2899 | 781.4373 | 19703.23 | 3 | 2341.2949 | Z\_DOT21 | 9 | -4.99e-03 | -2.13 |
| 512 | 20 | 3388.7637 | 678.7600 | 14717.99 | 5 | 3388.7791 | Z\_DOT29 | 1 | -0.0153 | -4.52 |
| 512 | 21 | 3432.8173 | 859.2116 | 15266.84 | 4 |  |  |  |  |  |
| 512 | 22 | 2539.3760 | 635.8513 | 19449.16 | 4 |  |  |  |  |  |
| 512 | 23 | 3303.7382 | 661.7549 | 14036.98 | 5 | 3303.7583 | C27 | 27 | -0.0201 | -6.09 |
| 512 | 24 | 1625.8689 | 813.9417 | 20203.44 | 2 |  |  |  |  |  |
| 512 | 25 | 3460.8136 | 866.2107 | 11452.03 | 4 |  |  |  |  |  |
| 512 | 26 | 1204.6624 | 603.3385 | 16259.69 | 2 | 1204.6607 | Z\_DOT12 | 18 | 1.68e-03 | 1.40 |
| 512 | 27 | 3458.8037 | 577.4746 | 12801.52 | 6 |  |  |  |  |  |
| 512 | 28 | 3004.5454 | 1002.5224 | 10856.46 | 3 |  |  |  |  |  |
| 512 | 29 | 695.5665 | 696.5737 | 86978.56 | 1 |  |  |  |  |  |
| 512 | 30 | 1360.6515 | 681.3330 | 20330.53 | 2 | 1360.6591 | C11 | 11 | -7.56e-03 | -5.56 |
| 512 | 31 | 2897.5560 | 725.3963 | 8939.00 | 4 | 2897.5731 | C24 | 24 | -0.0171 | -5.91 |
| 512 | 32 | 2960.4820 | 741.1278 | 10509.26 | 4 |  |  |  |  |  |
| 512 | 33 | 1333.7043 | 667.8594 | 14247.01 | 2 | 1333.7033 | Z\_DOT13 | 17 | 9.63e-04 | 0.72 |
| 512 | 34 | 3003.5372 | 751.8916 | 11598.87 | 4 | 3003.5465 | Z\_DOT26 | 4 | -9.38e-03 | -3.12 |
| 512 | 35 | 2457.2841 | 820.1020 | 8688.68 | 3 | 2457.2984 | C20 | 20 | -0.0143 | -5.80 |
| 512 | 36 | 1738.8847 | 870.4496 | 10089.58 | 2 | 1738.8970 | C14 | 14 | -0.0123 | -7.10 |
| 512 | 37 | 3389.7692 | 848.4496 | 14979.60 | 4 |  |  |  |  |  |
| 512 | 38 | 2698.4258 | 675.6137 | 10905.09 | 4 | 2698.4410 | C22 | 22 | -0.0152 | -5.63 |
| 512 | 39 | 1488.7460 | 745.3803 | 9623.82 | 2 | 1488.7540 | C12 | 12 | -8.04e-03 | -5.40 |
| 512 | 40 | 562.0643 | 563.0716 | 27844.94 | 1 |  |  |  |  |  |
| 512 | 41 | 1135.5417 | 568.7781 | 12927.55 | 2 | 1135.5477 | C9 | 9 | -6.09e-03 | -5.36 |
| 512 | 42 | 473.2940 | 474.3013 | 9504.70 | 1 | 473.2961 | C4 | 4 | -2.10e-03 | -4.43 |
| 512 | 43 | 1488.7462 | 497.2560 | 4930.65 | 3 | 1488.7540 | C12 | 12 | -7.84e-03 | -5.27 |
| 512 | 44 | 1220.6811 | 611.3478 | 5492.16 | 2 |  |  |  |  |  |
| 512 | 45 | 778.4064 | 779.4136 | 4297.33 | 1 | 778.4016 | Z\_DOT8 | 22 | 4.71e-03 | 6.05 |
| 512 | 46 | 1135.5421 | 1136.5494 | 5805.69 | 1 | 1135.5477 | C9 | 9 | -5.65e-03 | -4.97 |
| 512 | 47 | 650.3118 | 651.3190 | 7991.19 | 1 | 650.3067 | Z\_DOT7 | 23 | 5.07e-03 | 7.79 |
| 512 | 48 | 1007.4838 | 1008.4910 | 4140.38 | 1 | 1007.4892 | C8 | 8 | -5.42e-03 | -5.38 |
| 512 | 49 | 1274.6916 | 638.3531 | 3290.33 | 2 |  |  |  |  |  |
| 512 | 50 | 976.4925 | 489.2535 | 2989.51 | 2 |  |  |  |  |  |
| 512 | 51 | 344.2523 | 345.2596 | 1850.33 | 1 | 344.2535 | C3 | 3 | -1.22e-03 | -3.55 |
| 512 | 52 | 733.3765 | 734.3838 | 1606.41 | 1 |  |  |  |  |  |
| 512 | 53 | 1007.4838 | 504.7492 | 2848.31 | 2 | 1007.4892 | C8 | 8 | -5.37e-03 | -5.33 |
| 512 | 54 | 1417.7493 | 709.8819 | 1561.47 | 2 |  |  |  |  |  |
| 512 | 55 | 746.4083 | 747.4155 | 2051.27 | 1 | 746.4108 | C6 | 6 | -2.58e-03 | -3.46 |
| 512 | 56 | 1076.5683 | 539.2914 | 1169.73 | 2 | 1076.5657 | Z\_DOT11 | 19 | 2.53e-03 | 2.35 |
| 512 | 57 | 1092.5866 | 547.3006 | 1416.86 | 2 |  |  |  |  |  |

  

All proteins /
CsTx-13a Cupiennius salei toxin 13 isoform a /
Proteoform #40
