# Supplementary material for: The Dual Prey-Inactivation Strategy of Spiders—In-Depth Venomic Analysis of Cupiennius salei
Source: Toxins (Basel). 2019 Mar 19;11(3):167. doi: 10.3390/toxins11030167 (PMC6468893; doi:10.3390/toxins11030167)
Supplement: Supplementary file 1 [file toxins-11-00167-s001.zip › Supplementary Dataset EV1/20180328_f2_topdown_OTMS2_EThcD_NL_i02_ms2_proteoform_cutoff_html/prsms/prsm146.html]

Protein-Spectrum-Match for Spectrum #383


All proteins /
CsTx-1a\_S1 Cupiennius salei toxin 1 isoform a S1^ACsTx-1a\_S2 Cupiennius salei toxin 1 isoform a S2 /
Proteoform #8

## Protein-Spectrum-Match #146 for Spectrum #383

|  |  |  |  |  |  |
| --- | --- | --- | --- | --- | --- |
| PrSM ID: | 146 | Scan(s): | 513 | Precursor charge: | 7 |
| Precursor m/z: | 1025.1766 | Precursor mass: | 7169.1854 | Proteoform mass: | 7169.1856 |
| # matched peaks: | 20 | # matched fragment ions: | 20 | # unexpected modifications: | 0 |
| E-value: | 1.23e-19 | P-value: | 1.23e-19 | Q-value (Spectral FDR): | 0 |

  

|  |  |  |  |  |  |  |  |  |  |  |  |  |  |  |  |  |  |  |  |  |  |  |  |  |  |  |  |  |  |  |  |  |  |  |  |  |  |  |  |  |  |  |  |  |  |  |  |  |  |  |  |  |  |  |  |  |  |  |  |  |  |  |  |  |  |  |  |  |  |
| --- | --- | --- | --- | --- | --- | --- | --- | --- | --- | --- | --- | --- | --- | --- | --- | --- | --- | --- | --- | --- | --- | --- | --- | --- | --- | --- | --- | --- | --- | --- | --- | --- | --- | --- | --- | --- | --- | --- | --- | --- | --- | --- | --- | --- | --- | --- | --- | --- | --- | --- | --- | --- | --- | --- | --- | --- | --- | --- | --- | --- | --- | --- | --- | --- | --- | --- | --- | --- | --- |
|  | |  | | | | | | | | | | | | | | | | | | | | | | | | | | | | | | | | | | | | | | | | | | | | | | | | | | | | | | | | | | | | | | | | | | | |
| 1 |  |  | M |  | K |  | V |  | L |  | I |  | I |  | S |  | A |  | V |  | L |  |  | F |  | I |  | T |  | I |  | F |  | S |  | N |  | I |  | S |  | A |  |  | E |  | I |  | E |  | D |  | D |  | F |  | L |  | E |  | D |  | E |  | 30 |  |
|  | |  | | | | | | | | | | | | | | | | | | | | | | | | | | | | | | | | | | | | | | | | | | | | | | | | | | | | | | | | | | | | | | | | | | | |
| 31 |  |  | S |  | F |  | E |  | A |  | E |  | D |  | I |  | I |  | P |  | F |  |  | F |  | E |  | N |  | E |  | Q |  | A |  | R | ] | S | ⎩ | C |  | I |  |  | P | ⎫ | K | ⎱ | H | ⎫ | E | ⎫ | E | ⎫ | C |  | T |  | N |  | D |  | K |  | 60 |  |
|  | |  | | | | | | | | | | | | | | | | | | | | | | | | | | | | | | | | | | | | | | | | | | | | | | | | | | | | | | | | | | | | | | | | | | | |
| 61 |  |  | H | ⎫ | N | ⎫ | C | ⎫ | C |  | R |  | K |  | G |  | L |  | F |  | K |  |  | L |  | K | ⎫ | C | ⎫ | Q | ⎫ | C |  | S |  | T |  | F |  | D |  | D |  |  | E |  | S |  | G | ⎫ | Q |  | P |  | T |  | E |  | R |  | C |  | A |  | 90 |  |
|  | |  | | | | | | | | | | | | | | | | | | | | | | | | | | | | | | | | | | | | | | | | | | | | | | | | | | | | | | | | | | | | | | | | | | | |
| 91 |  |  | C |  | G |  | R |  | P | ⎫ | M | ⎫ | G | ⎫ | H | ⎫ | Q | ⎫ | A |  | I |  |  | E |  | T |  | G |  | L |  | N |  | I | ⎫ | F | [ | R |  | G |  | L |  |  | F |  | K |  | G |  | K |  | K |  | K |  | N |  | K |  | K |  | T |  | 120 |  |
|  | |  | | | | | | | | | | | | | | | | | | | | | | | | | | | | | | | | | | | | | | | | | | | | | | | | | | | | | | | | | | | | | | | | | | | |
| 121 |  |  | K |  | G |  | | | | 122 |  | | | | | | | | | | | | | | | | | | | | | | | | | | | | | | | | | | | | | | | | | | | | | | | | | | | | | | | |

Fixed PTMs: Carbamidomethylation [C49 C56 C63 C64 C73 C75 C89 C91 ]

  

All peaks (54)  Matched peaks (20)  Not matched peaks (34)

  

| Scan | Peak | Mono mass | Mono m/z | Intensity | Charge | Theoretical mass | Ion | Pos | Mass error | PPM error |
| --- | --- | --- | --- | --- | --- | --- | --- | --- | --- | --- |
| 513 | 1 | 3075.7542 | 1026.2587 | 133744.43 | 3 |  |  |  |  |  |
| 513 | 2 | 7112.1145 | 1186.3597 | 54822.62 | 6 |  |  |  |  |  |
| 513 | 3 | 3584.5677 | 1195.8632 | 32735.59 | 3 |  |  |  |  |  |
| 513 | 4 | 7125.1241 | 1188.5280 | 11090.04 | 6 |  |  |  |  |  |
| 513 | 5 | 2389.7133 | 1195.8639 | 25115.40 | 2 |  |  |  |  |  |
| 513 | 6 | 7112.1197 | 1423.4312 | 7124.54 | 5 |  |  |  |  |  |
| 513 | 7 | 7153.1228 | 1193.1944 | 8188.51 | 6 |  |  |  |  |  |
| 513 | 8 | 7080.1355 | 1181.0299 | 7186.52 | 6 |  |  |  |  |  |
| 513 | 9 | 1024.4499 | 1025.4572 | 39096.89 | 1 |  |  |  |  |  |
| 513 | 10 | 3157.4961 | 1053.5060 | 6371.83 | 3 | 3157.5153 | C25 | 25 | -0.0192 | -6.10 |
| 513 | 11 | 2782.3058 | 928.4426 | 5091.69 | 3 |  |  |  |  |  |
| 513 | 12 | 7097.1035 | 1183.8579 | 3842.37 | 6 |  |  |  |  |  |
| 513 | 13 | 3075.7575 | 1538.8860 | 4416.62 | 2 |  |  |  |  |  |
| 513 | 14 | 5888.5169 | 1178.7106 | 3374.16 | 5 | 5887.5503 | C48 | 48 | -0.0358 | -6.07 |
| 513 | 15 | 6976.0826 | 1163.6877 | 3851.37 | 6 |  |  |  |  |  |
| 513 | 16 | 7021.0965 | 1171.1900 | 4662.83 | 6 | 7021.1332 | C59 | 59 | -0.0367 | -5.23 |
| 513 | 17 | 1752.7564 | 877.3855 | 4579.07 | 2 | 1752.7671 | C14 | 14 | -0.0107 | -6.13 |
| 513 | 18 | 868.4172 | 869.4244 | 4428.34 | 1 | 868.4225 | C7 | 7 | -5.30e-03 | -6.11 |
| 513 | 19 | 7064.1172 | 1178.3601 | 3531.43 | 6 |  |  |  |  |  |
| 513 | 20 | 3445.5826 | 1149.5348 | 2225.75 | 3 | 3445.6046 | C27 | 27 | -0.0220 | -6.38 |
| 513 | 21 | 4443.8913 | 1111.9801 | 3612.24 | 4 | 4443.9333 | C36 | 36 | -0.0420 | -9.46 |
| 513 | 22 | 6081.5721 | 1217.3217 | 4528.04 | 5 | 6081.6306 | C50 | 50 | -0.0585 | -9.63 |
| 513 | 23 | 602.3176 | 603.3248 | 3390.62 | 1 | 602.3210 | C5 | 5 | -3.41e-03 | -5.66 |
| 513 | 24 | 2871.3029 | 958.1083 | 3309.90 | 3 |  |  |  |  |  |
| 513 | 25 | 7054.0868 | 1411.8246 | 1755.73 | 5 |  |  |  |  |  |
| 513 | 26 | 6566.8275 | 1314.3728 | 1385.61 | 5 | 6567.8725 | Z\_DOT55 | 5 | -0.0427 | -6.50 |
| 513 | 27 | 1866.7960 | 934.4053 | 2469.72 | 2 | 1866.8101 | C15 | 15 | -0.0141 | -7.54 |
| 513 | 28 | 3923.6537 | 1308.8919 | 1458.86 | 3 |  |  |  |  |  |
| 513 | 29 | 739.3755 | 740.3828 | 2441.06 | 1 | 739.3799 | C6 | 6 | -4.40e-03 | -5.95 |
| 513 | 30 | 5944.5255 | 1189.9124 | 2516.55 | 5 | 5944.5717 | C49 | 49 | -0.0463 | -7.78 |
| 513 | 31 | 6209.6466 | 1242.9366 | 3139.43 | 5 | 6209.6892 | C51 | 51 | -0.0426 | -6.87 |
| 513 | 32 | 6583.8421 | 1317.7757 | 1984.67 | 5 |  |  |  |  |  |
| 513 | 33 | 7036.1357 | 1173.6966 | 2564.87 | 6 |  |  |  |  |  |
| 513 | 34 | 3183.5092 | 1062.1770 | 1648.07 | 3 |  |  |  |  |  |
| 513 | 35 | 3528.5407 | 1177.1875 | 1442.63 | 3 |  |  |  |  |  |
| 513 | 36 | 1434.6329 | 1435.6402 | 2365.76 | 1 |  |  |  |  |  |
| 513 | 37 | 2917.3225 | 973.4481 | 2133.52 | 3 |  |  |  |  |  |
| 513 | 38 | 2026.8292 | 1014.4219 | 1154.57 | 2 | 2026.8407 | C16 | 16 | -0.0115 | -5.68 |
| 513 | 39 | 3317.5233 | 1106.8484 | 1079.38 | 3 | 3317.5460 | C26 | 26 | -0.0227 | -6.83 |
| 513 | 40 | 474.2236 | 475.2309 | 898.29 | 1 | 474.2260 | C4 | 4 | -2.38e-03 | -5.03 |
| 513 | 41 | 997.4589 | 998.4662 | 1030.67 | 1 | 997.4651 | C8 | 8 | -6.14e-03 | -6.16 |
| 513 | 42 | 2743.2059 | 915.4092 | 1023.25 | 3 |  |  |  |  |  |
| 513 | 43 | 6625.8930 | 1326.1859 | 521.34 | 5 |  |  |  |  |  |
| 513 | 44 | 7066.1028 | 1414.2278 | 1231.16 | 5 | 7066.1350 | Z\_DOT59 | 1 | -0.0322 | -4.56 |
| 513 | 45 | 3635.5607 | 1212.8609 | 783.73 | 3 |  |  |  |  |  |
| 513 | 46 | 6029.6762 | 1206.9425 | 736.41 | 5 |  |  |  |  |  |
| 513 | 47 | 1164.0183 | 1165.0256 | 347.02 | 1 |  |  |  |  |  |
| 513 | 48 | 2272.0478 | 1137.0312 | 587.31 | 2 |  |  |  |  |  |
| 513 | 49 | 1263.3307 | 1264.3380 | 486.77 | 1 |  |  |  |  |  |
| 513 | 50 | 1303.5886 | 1304.5959 | 609.13 | 1 |  |  |  |  |  |
| 513 | 51 | 5755.4505 | 1152.0974 | 1234.31 | 5 | 5756.5098 | C47 | 47 | -0.0570 | -9.89 |
| 513 | 52 | 4269.8482 | 1424.2900 | 1926.47 | 3 |  |  |  |  |  |
| 513 | 53 | 1339.5838 | 1340.5911 | 490.22 | 1 |  |  |  |  |  |
| 513 | 54 | 1074.9623 | 1075.9695 | 462.97 | 1 |  |  |  |  |  |

  

All proteins /
CsTx-1a\_S1 Cupiennius salei toxin 1 isoform a S1^ACsTx-1a\_S2 Cupiennius salei toxin 1 isoform a S2 /
Proteoform #8
