# Supplementary material for: The Dual Prey-Inactivation Strategy of Spiders—In-Depth Venomic Analysis of Cupiennius salei
Source: Toxins (Basel). 2019 Mar 19;11(3):167. doi: 10.3390/toxins11030167 (PMC6468893; doi:10.3390/toxins11030167)
Supplement: Supplementary file 1 [file toxins-11-00167-s001.zip › Supplementary Dataset EV1/20180328_f2_topdown_OTMS2_EThcD_NL_i02_ms2_proteoform_cutoff_html/prsms/prsm147.html]

Protein-Spectrum-Match for Spectrum #385


All proteins /
CsTx-1a\_S1 Cupiennius salei toxin 1 isoform a S1^ACsTx-1a\_S2 Cupiennius salei toxin 1 isoform a S2 /
Proteoform #8

## Protein-Spectrum-Match #147 for Spectrum #385

|  |  |  |  |  |  |
| --- | --- | --- | --- | --- | --- |
| PrSM ID: | 147 | Scan(s): | 516 | Precursor charge: | 7 |
| Precursor m/z: | 1025.3189 | Precursor mass: | 7170.1817 | Proteoform mass: | 7169.1856 |
| # matched peaks: | 19 | # matched fragment ions: | 19 | # unexpected modifications: | 0 |
| E-value: | 1.03e-21 | P-value: | 1.03e-21 | Q-value (Spectral FDR): | 0 |

  

|  |  |  |  |  |  |  |  |  |  |  |  |  |  |  |  |  |  |  |  |  |  |  |  |  |  |  |  |  |  |  |  |  |  |  |  |  |  |  |  |  |  |  |  |  |  |  |  |  |  |  |  |  |  |  |  |  |  |  |  |  |  |  |  |  |  |  |  |  |  |
| --- | --- | --- | --- | --- | --- | --- | --- | --- | --- | --- | --- | --- | --- | --- | --- | --- | --- | --- | --- | --- | --- | --- | --- | --- | --- | --- | --- | --- | --- | --- | --- | --- | --- | --- | --- | --- | --- | --- | --- | --- | --- | --- | --- | --- | --- | --- | --- | --- | --- | --- | --- | --- | --- | --- | --- | --- | --- | --- | --- | --- | --- | --- | --- | --- | --- | --- | --- | --- | --- |
|  | |  | | | | | | | | | | | | | | | | | | | | | | | | | | | | | | | | | | | | | | | | | | | | | | | | | | | | | | | | | | | | | | | | | | | |
| 1 |  |  | M |  | K |  | V |  | L |  | I |  | I |  | S |  | A |  | V |  | L |  |  | F |  | I |  | T |  | I |  | F |  | S |  | N |  | I |  | S |  | A |  |  | E |  | I |  | E |  | D |  | D |  | F |  | L |  | E |  | D |  | E |  | 30 |  |
|  | |  | | | | | | | | | | | | | | | | | | | | | | | | | | | | | | | | | | | | | | | | | | | | | | | | | | | | | | | | | | | | | | | | | | | |
| 31 |  |  | S |  | F |  | E |  | A |  | E |  | D |  | I |  | I |  | P |  | F |  |  | F |  | E |  | N |  | E |  | Q |  | A |  | R | ] | S |  | C |  | I |  |  | P | ⎫ | K | ⎫ | H | ⎫ | E | ⎱ | E | ⎫ | C |  | T |  | N |  | D |  | K |  | 60 |  |
|  | |  | | | | | | | | | | | | | | | | | | | | | | | | | | | | | | | | | | | | | | | | | | | | | | | | | | | | | | | | | | | | | | | | | | | |
| 61 |  |  | H | ⎫ | N | ⎫ | C | ⎫ | C |  | R |  | K |  | G |  | L |  | F |  | K |  | ⎫ | L |  | K | ⎫ | C |  | Q | ⎫ | C |  | S |  | T |  | F |  | D |  | D |  |  | E |  | S |  | G | ⎫ | Q |  | P |  | T |  | E |  | R |  | C |  | A |  | 90 |  |
|  | |  | | | | | | | | | | | | | | | | | | | | | | | | | | | | | | | | | | | | | | | | | | | | | | | | | | | | | | | | | | | | | | | | | | | |
| 91 |  |  | C |  | G | ⎫ | R |  | P | ⎫ | M | ⎫ | G | ⎫ | H |  | Q | ⎫ | A |  | I |  |  | E |  | T |  | G |  | L |  | N |  | I | ⎫ | F | [ | R |  | G |  | L |  |  | F |  | K |  | G |  | K |  | K |  | K |  | N |  | K |  | K |  | T |  | 120 |  |
|  | |  | | | | | | | | | | | | | | | | | | | | | | | | | | | | | | | | | | | | | | | | | | | | | | | | | | | | | | | | | | | | | | | | | | | |
| 121 |  |  | K |  | G |  | | | | 122 |  | | | | | | | | | | | | | | | | | | | | | | | | | | | | | | | | | | | | | | | | | | | | | | | | | | | | | | | |

Fixed PTMs: Carbamidomethylation [C49 C56 C63 C64 C73 C75 C89 C91 ]

  

All peaks (51)  Matched peaks (19)  Not matched peaks (32)

  

| Scan | Peak | Mono mass | Mono m/z | Intensity | Charge | Theoretical mass | Ion | Pos | Mass error | PPM error |
| --- | --- | --- | --- | --- | --- | --- | --- | --- | --- | --- |
| 516 | 1 | 3074.7514 | 1025.9244 | 188214.36 | 3 |  |  |  |  |  |
| 516 | 2 | 7112.1131 | 1186.3595 | 51654.74 | 6 |  |  |  |  |  |
| 516 | 3 | 3585.0698 | 1196.0305 | 34845.41 | 3 |  |  |  |  |  |
| 516 | 4 | 7167.1338 | 1024.8835 | 150099.37 | 7 |  |  |  |  |  |
| 516 | 5 | 7125.1227 | 1188.5277 | 10374.13 | 6 |  |  |  |  |  |
| 516 | 6 | 7113.1176 | 1423.6308 | 5351.24 | 5 |  |  |  |  |  |
| 516 | 7 | 2782.3027 | 928.4415 | 4959.40 | 3 |  |  |  |  |  |
| 516 | 8 | 7153.1218 | 1193.1942 | 6605.33 | 6 |  |  |  |  |  |
| 516 | 9 | 3075.7575 | 1538.8860 | 5750.85 | 2 |  |  |  |  |  |
| 516 | 10 | 7021.1064 | 1171.1917 | 4766.81 | 6 | 7021.1332 | C59 | 59 | -0.0268 | -3.81 |
| 516 | 11 | 1752.7549 | 877.3847 | 4269.13 | 2 | 1752.7671 | C14 | 14 | -0.0122 | -6.97 |
| 516 | 12 | 7080.1256 | 1181.0282 | 4755.32 | 6 |  |  |  |  |  |
| 516 | 13 | 6977.0794 | 1163.8538 | 3508.21 | 6 |  |  |  |  |  |
| 516 | 14 | 6209.6397 | 1242.9352 | 3051.93 | 5 | 6209.6892 | C51 | 51 | -0.0495 | -7.97 |
| 516 | 15 | 7095.0945 | 1183.5230 | 3040.23 | 6 |  |  |  |  |  |
| 516 | 16 | 3157.4950 | 1053.5056 | 6021.30 | 3 | 3157.5153 | C25 | 25 | -0.0203 | -6.44 |
| 516 | 17 | 1866.7983 | 934.4064 | 2907.77 | 2 | 1866.8101 | C15 | 15 | -0.0117 | -6.29 |
| 516 | 18 | 5887.5060 | 1178.5085 | 3124.88 | 5 | 5887.5503 | C48 | 48 | -0.0443 | -7.53 |
| 516 | 19 | 6301.7208 | 1261.3514 | 2833.42 | 5 | 6301.7710 | Z\_DOT53 | 7 | -0.0502 | -7.96 |
| 516 | 20 | 7064.1175 | 1178.3602 | 2419.16 | 6 |  |  |  |  |  |
| 516 | 21 | 868.4173 | 869.4246 | 4352.17 | 1 | 868.4225 | C7 | 7 | -5.18e-03 | -5.96 |
| 516 | 22 | 6081.5660 | 1217.3205 | 2979.09 | 5 |  |  |  |  |  |
| 516 | 23 | 602.3176 | 603.3249 | 3391.42 | 1 | 602.3210 | C5 | 5 | -3.35e-03 | -5.56 |
| 516 | 24 | 4443.9026 | 1111.9829 | 2440.47 | 4 | 4443.9333 | C36 | 36 | -0.0307 | -6.91 |
| 516 | 25 | 5756.4689 | 1152.3010 | 2128.15 | 5 | 5756.5098 | C47 | 47 | -0.0409 | -7.11 |
| 516 | 26 | 3183.5062 | 1062.1760 | 1466.45 | 3 |  |  |  |  |  |
| 516 | 27 | 2872.3020 | 958.4413 | 3269.93 | 3 |  |  |  |  |  |
| 516 | 28 | 3445.5866 | 1149.5362 | 2086.71 | 3 | 3445.6046 | C27 | 27 | -0.0179 | -5.21 |
| 516 | 29 | 7003.0628 | 1001.4448 | 2048.97 | 7 |  |  |  |  |  |
| 516 | 30 | 739.3757 | 740.3829 | 2529.22 | 1 | 739.3799 | C6 | 6 | -4.22e-03 | -5.70 |
| 516 | 31 | 2916.3145 | 973.1121 | 1991.00 | 3 | 2916.3363 | C23 | 23 | -0.0218 | -7.48 |
| 516 | 32 | 5944.5288 | 1189.9130 | 2695.33 | 5 | 5944.5717 | C49 | 49 | -0.0429 | -7.22 |
| 516 | 33 | 3518.0568 | 1173.6929 | 1702.88 | 3 |  |  |  |  |  |
| 516 | 34 | 3030.7371 | 1516.3758 | 1378.32 | 2 |  |  |  |  |  |
| 516 | 35 | 1170.3483 | 1171.3555 | 1130.72 | 1 |  |  |  |  |  |
| 516 | 36 | 1195.1908 | 1196.1981 | 10856.76 | 1 |  |  |  |  |  |
| 516 | 37 | 4269.8457 | 1424.2892 | 1171.46 | 3 |  |  |  |  |  |
| 516 | 38 | 4898.0940 | 1225.5308 | 1199.01 | 4 |  |  |  |  |  |
| 516 | 39 | 997.4604 | 998.4677 | 990.56 | 1 | 997.4651 | C8 | 8 | -4.68e-03 | -4.69 |
| 516 | 40 | 5502.3191 | 1101.4711 | 700.40 | 5 | 5503.3559 | C45 | 45 | -0.0345 | -6.26 |
| 516 | 41 | 6782.9426 | 1131.4977 | 633.49 | 6 |  |  |  |  |  |
| 516 | 42 | 474.2234 | 475.2307 | 729.81 | 1 | 474.2260 | C4 | 4 | -2.63e-03 | -5.54 |
| 516 | 43 | 2026.8308 | 1014.4227 | 952.60 | 2 | 2026.8407 | C16 | 16 | -9.93e-03 | -4.90 |
| 516 | 44 | 2708.1936 | 1355.1041 | 816.81 | 2 |  |  |  |  |  |
| 516 | 45 | 1313.7655 | 1314.7728 | 498.82 | 1 |  |  |  |  |  |
| 516 | 46 | 2743.2029 | 915.4082 | 542.53 | 3 |  |  |  |  |  |
| 516 | 47 | 2945.7132 | 1473.8639 | 732.09 | 2 |  |  |  |  |  |
| 516 | 48 | 1226.6205 | 1227.6277 | 463.86 | 1 |  |  |  |  |  |
| 516 | 49 | 3922.6474 | 1308.5564 | 1226.48 | 3 |  |  |  |  |  |
| 516 | 50 | 1434.8310 | 1435.8383 | 1458.38 | 1 |  |  |  |  |  |
| 516 | 51 | 1100.6598 | 1101.6671 | 310.27 | 1 |  |  |  |  |  |

  

All proteins /
CsTx-1a\_S1 Cupiennius salei toxin 1 isoform a S1^ACsTx-1a\_S2 Cupiennius salei toxin 1 isoform a S2 /
Proteoform #8
