# Supplementary material for: The Dual Prey-Inactivation Strategy of Spiders—In-Depth Venomic Analysis of Cupiennius salei
Source: Toxins (Basel). 2019 Mar 19;11(3):167. doi: 10.3390/toxins11030167 (PMC6468893; doi:10.3390/toxins11030167)
Supplement: Supplementary file 1 [file toxins-11-00167-s001.zip › Supplementary Dataset EV1/20180328_f2_topdown_OTMS2_EThcD_NL_i02_ms2_proteoform_cutoff_html/prsms/prsm148.html]

Protein-Spectrum-Match for Spectrum #386


All proteins /
CsTx-1a\_S1 Cupiennius salei toxin 1 isoform a S1^ACsTx-1a\_S2 Cupiennius salei toxin 1 isoform a S2 /
Proteoform #8

## Protein-Spectrum-Match #148 for Spectrum #386

|  |  |  |  |  |  |
| --- | --- | --- | --- | --- | --- |
| PrSM ID: | 148 | Scan(s): | 517 | Precursor charge: | 8 |
| Precursor m/z: | 897.1546 | Precursor mass: | 7169.1786 | Proteoform mass: | 7169.1856 |
| # matched peaks: | 16 | # matched fragment ions: | 16 | # unexpected modifications: | 0 |
| E-value: | 1.91e-18 | P-value: | 1.91e-18 | Q-value (Spectral FDR): | 0 |

  

|  |  |  |  |  |  |  |  |  |  |  |  |  |  |  |  |  |  |  |  |  |  |  |  |  |  |  |  |  |  |  |  |  |  |  |  |  |  |  |  |  |  |  |  |  |  |  |  |  |  |  |  |  |  |  |  |  |  |  |  |  |  |  |  |  |  |  |  |  |  |
| --- | --- | --- | --- | --- | --- | --- | --- | --- | --- | --- | --- | --- | --- | --- | --- | --- | --- | --- | --- | --- | --- | --- | --- | --- | --- | --- | --- | --- | --- | --- | --- | --- | --- | --- | --- | --- | --- | --- | --- | --- | --- | --- | --- | --- | --- | --- | --- | --- | --- | --- | --- | --- | --- | --- | --- | --- | --- | --- | --- | --- | --- | --- | --- | --- | --- | --- | --- | --- | --- |
|  | |  | | | | | | | | | | | | | | | | | | | | | | | | | | | | | | | | | | | | | | | | | | | | | | | | | | | | | | | | | | | | | | | | | | | |
| 1 |  |  | M |  | K |  | V |  | L |  | I |  | I |  | S |  | A |  | V |  | L |  |  | F |  | I |  | T |  | I |  | F |  | S |  | N |  | I |  | S |  | A |  |  | E |  | I |  | E |  | D |  | D |  | F |  | L |  | E |  | D |  | E |  | 30 |  |
|  | |  | | | | | | | | | | | | | | | | | | | | | | | | | | | | | | | | | | | | | | | | | | | | | | | | | | | | | | | | | | | | | | | | | | | |
| 31 |  |  | S |  | F |  | E |  | A |  | E |  | D |  | I |  | I |  | P |  | F |  |  | F |  | E |  | N |  | E |  | Q |  | A |  | R | ] | S | ⎩ | C |  | I |  |  | P |  | K |  | H |  | E | ⎫ | E | ⎫ | C |  | T |  | N | ⎩ | D |  | K |  | 60 |  |
|  | |  | | | | | | | | | | | | | | | | | | | | | | | | | | | | | | | | | | | | | | | | | | | | | | | | | | | | | | | | | | | | | | | | | | | |
| 61 |  |  | H | ⎫ | N | ⎫ | C |  | C |  | R |  | K | ⎫ | G |  | L |  | F | ⎫ | K |  | ⎫ | L |  | K | ⎫ | C |  | Q | ⎫ | C |  | S |  | T |  | F |  | D | ⎫ | D |  |  | E | ⎫ | S |  | G | ⎫ | Q |  | P |  | T |  | E |  | R |  | C |  | A |  | 90 |  |
|  | |  | | | | | | | | | | | | | | | | | | | | | | | | | | | | | | | | | | | | | | | | | | | | | | | | | | | | | | | | | | | | | | | | | | | |
| 91 |  |  | C |  | G |  | R |  | P |  | M | ⎫ | G |  | H |  | Q | ⎫ | A |  | I |  |  | E |  | T |  | G |  | L |  | N |  | I |  | F | [ | R |  | G |  | L |  |  | F |  | K |  | G |  | K |  | K |  | K |  | N |  | K |  | K |  | T |  | 120 |  |
|  | |  | | | | | | | | | | | | | | | | | | | | | | | | | | | | | | | | | | | | | | | | | | | | | | | | | | | | | | | | | | | | | | | | | | | |
| 121 |  |  | K |  | G |  | | | | 122 |  | | | | | | | | | | | | | | | | | | | | | | | | | | | | | | | | | | | | | | | | | | | | | | | | | | | | | | | |

Fixed PTMs: Carbamidomethylation [C49 C56 C63 C64 C73 C75 C89 C91 ]

  

All peaks (63)  Matched peaks (16)  Not matched peaks (47)

  

| Scan | Peak | Mono mass | Mono m/z | Intensity | Charge | Theoretical mass | Ion | Pos | Mass error | PPM error |
| --- | --- | --- | --- | --- | --- | --- | --- | --- | --- | --- |
| 517 | 1 | 7112.1130 | 1017.0234 | 45064.98 | 7 |  |  |  |  |  |
| 517 | 2 | 7112.1175 | 1186.3602 | 25454.93 | 6 |  |  |  |  |  |
| 517 | 3 | 3585.5682 | 897.3993 | 49899.02 | 4 |  |  |  |  |  |
| 517 | 4 | 7153.1252 | 1022.8823 | 8415.76 | 7 |  |  |  |  |  |
| 517 | 5 | 6890.9786 | 985.4328 | 5898.33 | 7 |  |  |  |  |  |
| 517 | 6 | 3585.5751 | 1196.1990 | 11291.65 | 3 |  |  |  |  |  |
| 517 | 7 | 7126.1245 | 1019.0251 | 7064.92 | 7 |  |  |  |  |  |
| 517 | 8 | 7055.0849 | 1176.8548 | 5910.21 | 6 |  |  |  |  |  |
| 517 | 9 | 7154.1211 | 1193.3608 | 6954.56 | 6 |  |  |  |  |  |
| 517 | 10 | 4029.9393 | 1008.4921 | 5245.52 | 4 |  |  |  |  |  |
| 517 | 11 | 7004.0518 | 1001.5861 | 7566.65 | 7 |  |  |  |  |  |
| 517 | 12 | 1752.7571 | 877.3858 | 5098.21 | 2 | 1752.7671 | C14 | 14 | -0.0100 | -5.71 |
| 517 | 13 | 1024.4499 | 1025.4572 | 10477.92 | 1 |  |  |  |  |  |
| 517 | 14 | 3157.4910 | 790.3800 | 3067.45 | 4 | 3157.5153 | C25 | 25 | -0.0243 | -7.70 |
| 517 | 15 | 6209.6492 | 1035.9488 | 2999.63 | 6 | 6209.6892 | C51 | 51 | -0.0401 | -6.45 |
| 517 | 16 | 4000.9251 | 1001.2386 | 5886.32 | 4 |  |  |  |  |  |
| 517 | 17 | 6187.7036 | 1032.2912 | 3436.53 | 6 |  |  |  |  |  |
| 517 | 18 | 2686.2938 | 896.4385 | 4602.71 | 3 |  |  |  |  |  |
| 517 | 19 | 2788.2222 | 930.4147 | 4241.30 | 3 | 2788.2414 | C22 | 22 | -0.0192 | -6.89 |
| 517 | 20 | 4443.8986 | 1111.9819 | 2170.85 | 4 | 4443.9333 | C36 | 36 | -0.0347 | -7.81 |
| 517 | 21 | 7023.1023 | 1004.3076 | 3699.92 | 7 |  |  |  |  |  |
| 517 | 22 | 7125.1359 | 1188.5299 | 3698.67 | 6 |  |  |  |  |  |
| 517 | 23 | 7081.1274 | 1012.5969 | 3563.18 | 7 |  |  |  |  |  |
| 517 | 24 | 4299.8531 | 1075.9705 | 1996.93 | 4 | 4299.8798 | C34 | 34 | -0.0268 | -6.22 |
| 517 | 25 | 3445.5824 | 862.4029 | 3022.96 | 4 | 3445.6046 | C27 | 27 | -0.0222 | -6.44 |
| 517 | 26 | 7005.0638 | 876.6402 | 3010.18 | 8 |  |  |  |  |  |
| 517 | 27 | 5888.5133 | 982.4262 | 1772.35 | 6 | 5887.5503 | C48 | 48 | -0.0393 | -6.67 |
| 517 | 28 | 3941.7547 | 986.4460 | 2181.81 | 4 |  |  |  |  |  |
| 517 | 29 | 7006.0698 | 1168.6856 | 1805.74 | 6 |  |  |  |  |  |
| 517 | 30 | 1866.8000 | 934.4073 | 3160.52 | 2 | 1866.8101 | C15 | 15 | -0.0100 | -5.38 |
| 517 | 31 | 2015.2213 | 1008.6179 | 3163.78 | 2 |  |  |  |  |  |
| 517 | 32 | 5377.6005 | 897.2740 | 32333.73 | 6 |  |  |  |  |  |
| 517 | 33 | 7067.1142 | 1178.8596 | 3001.17 | 6 | 7066.1350 | Z\_DOT59 | 1 | -0.0231 | -3.28 |
| 517 | 34 | 2872.3018 | 958.4412 | 1890.15 | 3 |  |  |  |  |  |
| 517 | 35 | 868.4184 | 869.4257 | 1883.54 | 1 | 868.4225 | C7 | 7 | -4.02e-03 | -4.63 |
| 517 | 36 | 3922.6525 | 1308.5581 | 1346.32 | 3 |  |  |  |  |  |
| 517 | 37 | 4554.9378 | 911.9948 | 1415.58 | 5 |  |  |  |  |  |
| 517 | 38 | 4055.7942 | 1014.9558 | 4699.80 | 4 | 4055.8103 | C32 | 32 | -0.0160 | -3.96 |
| 517 | 39 | 5797.5646 | 1160.5202 | 1015.06 | 5 | 5797.6072 | Z\_DOT49 | 11 | -0.0426 | -7.35 |
| 517 | 40 | 2471.0511 | 824.6910 | 1219.07 | 3 | 2471.0674 | C19 | 19 | -0.0164 | -6.62 |
| 517 | 41 | 2916.3184 | 973.1134 | 1957.57 | 3 | 2916.3363 | C23 | 23 | -0.0180 | -6.16 |
| 517 | 42 | 5052.3162 | 1011.4705 | 1899.68 | 5 |  |  |  |  |  |
| 517 | 43 | 2640.1477 | 881.0565 | 2467.13 | 3 |  |  |  |  |  |
| 517 | 44 | 4472.8344 | 895.5741 | 1625.00 | 5 |  |  |  |  |  |
| 517 | 45 | 6028.6743 | 1005.7863 | 1763.97 | 6 |  |  |  |  |  |
| 517 | 46 | 3634.5613 | 1212.5277 | 1098.35 | 3 |  |  |  |  |  |
| 517 | 47 | 7024.0961 | 1171.6900 | 2072.51 | 6 |  |  |  |  |  |
| 517 | 48 | 6303.7228 | 1261.7518 | 1524.13 | 5 |  |  |  |  |  |
| 517 | 49 | 6084.6881 | 1015.1220 | 1354.79 | 6 |  |  |  |  |  |
| 517 | 50 | 3158.5010 | 1053.8409 | 1795.37 | 3 |  |  |  |  |  |
| 517 | 51 | 5328.3929 | 1066.6859 | 1148.68 | 5 |  |  |  |  |  |
| 517 | 52 | 1579.9742 | 790.9944 | 1616.97 | 2 |  |  |  |  |  |
| 517 | 53 | 1143.2666 | 1144.2739 | 1355.84 | 1 |  |  |  |  |  |
| 517 | 54 | 1833.1275 | 917.5710 | 1442.18 | 2 |  |  |  |  |  |
| 517 | 55 | 4380.9225 | 1096.2379 | 1429.99 | 4 |  |  |  |  |  |
| 517 | 56 | 997.4591 | 998.4664 | 2066.87 | 1 | 997.4651 | C8 | 8 | -5.96e-03 | -5.98 |
| 517 | 57 | 4169.7995 | 1043.4571 | 1231.00 | 4 |  |  |  |  |  |
| 517 | 58 | 896.2084 | 897.2156 | 1516.50 | 1 |  |  |  |  |  |
| 517 | 59 | 1790.0819 | 896.0482 | 2364.21 | 2 |  |  |  |  |  |
| 517 | 60 | 4000.9359 | 1334.6526 | 1282.75 | 3 |  |  |  |  |  |
| 517 | 61 | 1193.8559 | 1194.8632 | 884.39 | 1 |  |  |  |  |  |
| 517 | 62 | 2527.0685 | 843.3634 | 1200.32 | 3 |  |  |  |  |  |
| 517 | 63 | 1110.9774 | 1111.9847 | 752.72 | 1 |  |  |  |  |  |

  

All proteins /
CsTx-1a\_S1 Cupiennius salei toxin 1 isoform a S1^ACsTx-1a\_S2 Cupiennius salei toxin 1 isoform a S2 /
Proteoform #8
