# Supplementary material for: The Dual Prey-Inactivation Strategy of Spiders—In-Depth Venomic Analysis of Cupiennius salei
Source: Toxins (Basel). 2019 Mar 19;11(3):167. doi: 10.3390/toxins11030167 (PMC6468893; doi:10.3390/toxins11030167)
Supplement: Supplementary file 1 [file toxins-11-00167-s001.zip › Supplementary Dataset EV1/20180328_f2_topdown_OTMS2_EThcD_NL_i02_ms2_proteoform_cutoff_html/prsms/prsm149.html]

Protein-Spectrum-Match for Spectrum #387


All proteins /
CsTx-12b Cupiennius salei toxin 12 isoform b /
Proteoform #47

## Protein-Spectrum-Match #149 for Spectrum #387

|  |  |  |  |  |  |
| --- | --- | --- | --- | --- | --- |
| PrSM ID: | 149 | Scan(s): | 519 | Precursor charge: | 6 |
| Precursor m/z: | 571.9903 | Precursor mass: | 3425.8982 | Proteoform mass: | 3425.8932 |
| # matched peaks: | 33 | # matched fragment ions: | 28 | # unexpected modifications: | 1 |
| E-value: | 1.69e-21 | P-value: | 1.69e-21 | Q-value (Spectral FDR): | 0 |

  

|  |  |  |  |  |  |  |  |  |  |  |  |  |  |  |  |  |  |  |  |  |  |  |  |  |  |  |  |  |  |  |  |  |  |  |  |  |  |  |  |  |  |  |  |  |  |  |  |  |  |  |  |  |  |  |  |  |  |  |  |  |  |  |  |  |  |  |
| --- | --- | --- | --- | --- | --- | --- | --- | --- | --- | --- | --- | --- | --- | --- | --- | --- | --- | --- | --- | --- | --- | --- | --- | --- | --- | --- | --- | --- | --- | --- | --- | --- | --- | --- | --- | --- | --- | --- | --- | --- | --- | --- | --- | --- | --- | --- | --- | --- | --- | --- | --- | --- | --- | --- | --- | --- | --- | --- | --- | --- | --- | --- | --- | --- | --- | --- |
|  | | ... 30 amino acid residues are skipped at the N-terminus ... | | | | | | | | | | | | | | | | | | | | | | | | | | | | | | | | | | | | | | | | | | | | | | | | | | | | | | | | | | | | | |  | | |
|  | |  | | | | | | | | | | | | | | | | | | | | | | | | | | | | | | | | | | | | | | | | | | | | | | | | | | | | | | | | | | | | | | | | | | | |
| 31 |  |  | S |  | F |  | E |  | A |  | D |  | D |  | V |  | I |  | P |  | F |  |  | L |  | A |  | R |  | E |  | Q |  | V |  | R |  | S |  | D |  | C |  |  | T |  | L |  | R |  | N |  | H |  | D |  | C |  | T |  | D |  | D |  | 60 |  |
|  | |  | | | | | | | | | | | | | | | | | | | | | | | | | | | | | | | | | | | | | | | | | | | | | | | | | | | | | | | | | | | | | | | | | | | |
| 61 |  |  | R |  | H |  | S |  | C |  | C |  | R |  | S |  | K |  | M |  | F |  |  | K |  | D |  | V |  | C |  | K |  | C |  | F |  | Y |  | P |  | S |  |  | Q |  | R |  | S |  | D |  | T |  | A |  | R | ] | A | ⎩ | K | ⎩ | K |  | 90 |  |
|  | |  | | | | | | | | | | | | | | | | | | | | | | | | | | | | | | | | | | | | | | | | | | | | | | | | | | | | | -58.01 | | | | | | | | | | | |
| 91 |  | ⎫ | E | ⎫ | L | ⎫ | C |  | T | ⎫ | C | ⎫ | Q | ⎫ | Q | ⎫ | D | ⎱ | K |  | H |  | ⎫ | L | ⎫ | K | ⎱ | Y |  | I | ⎱ | E | ⎫ | K |  | G | ⎫ | L |  | Q | ⎱ | K |  | ⎫ | A | ⎱ | K | ⎫ | V | ⎫ | L | ⎫ | V | ⎫ | A |  | G |  | | 117 |  | | | | | |

Fixed PTMs: Carbamidomethylation [C93 C95 ]   
  
     Unexpected modifications:   Unknown [-58.01]

  

All peaks (57)  Matched peaks (33)  Not matched peaks (24)

  

| Scan | Peak | Mono mass | Mono m/z | Intensity | Charge | Theoretical mass | Ion | Pos | Mass error | PPM error |
| --- | --- | --- | --- | --- | --- | --- | --- | --- | --- | --- |
| 519 | 1 | 3368.8586 | 674.7790 | 195573.08 | 5 |  |  |  |  |  |
| 519 | 2 | 1713.4453 | 572.1557 | 350645.25 | 3 |  |  |  |  |  |
| 519 | 3 | 3142.6934 | 786.6806 | 66547.55 | 4 | 3142.7106 | C26 | 26 | -0.0172 | -5.49 |
| 519 | 4 | 3424.8900 | 571.8223 | 270347.99 | 6 |  |  |  |  |  |
| 519 | 5 | 3368.8597 | 843.2222 | 60680.79 | 4 |  |  |  |  |  |
| 519 | 6 | 2048.2642 | 683.7620 | 56259.74 | 3 | 2048.2677 | Z\_DOT19 | 11 | -3.51e-03 | -1.71 |
| 519 | 7 | 2161.1022 | 721.3747 | 53366.31 | 3 | 2161.1135 | C17 | 17 | -0.0113 | -5.24 |
| 519 | 8 | 3354.8459 | 671.9765 | 55709.25 | 5 | 3354.8631 | C28 | 28 | -0.0172 | -5.13 |
| 519 | 9 | 1884.9563 | 629.3260 | 52205.70 | 3 | 1884.9662 | C15 | 15 | -9.85e-03 | -5.22 |
| 519 | 10 | 3210.7317 | 803.6902 | 33144.13 | 4 | 3210.7424 | Z\_DOT28 | 2 | -0.0107 | -3.34 |
| 519 | 11 | 3409.8627 | 682.9798 | 31662.59 | 5 |  |  |  |  |  |
| 519 | 12 | 2475.2596 | 826.0938 | 41905.88 | 3 | 2475.2726 | C20 | 20 | -0.0130 | -5.24 |
| 519 | 13 | 571.3144 | 572.3217 | 219764.78 | 1 |  |  |  |  |  |
| 519 | 14 | 2290.1436 | 764.3885 | 35904.46 | 3 | 2290.1561 | C18 | 18 | -0.0125 | -5.47 |
| 519 | 15 | 3338.8248 | 668.7722 | 28828.50 | 5 | 3338.8374 | Z\_DOT29 | 1 | -0.0126 | -3.78 |
| 519 | 16 | 1541.9339 | 771.9742 | 44129.05 | 2 | 1541.9349 | Z\_DOT15 | 15 | -9.79e-04 | -0.64 |
| 519 | 17 | 1378.6259 | 690.3202 | 45221.46 | 2 | 1378.6333 | C11 | 11 | -7.38e-03 | -5.35 |
| 519 | 18 | 2915.5308 | 729.8900 | 29945.22 | 4 | 2915.5473 | C24 | 24 | -0.0165 | -5.64 |
| 519 | 19 | 3408.8584 | 569.1503 | 23746.73 | 6 |  |  |  |  |  |
| 519 | 20 | 3338.8270 | 835.7140 | 32910.82 | 4 | 3338.8374 | Z\_DOT29 | 1 | -0.0104 | -3.10 |
| 519 | 21 | 2716.4005 | 906.4741 | 26200.03 | 3 | 2716.4152 | C22 | 22 | -0.0147 | -5.41 |
| 519 | 22 | 2844.4942 | 712.1308 | 23473.63 | 4 | 2844.5102 | C23 | 23 | -0.0160 | -5.61 |
| 519 | 23 | 3381.8680 | 677.3809 | 20109.37 | 5 |  |  |  |  |  |
| 519 | 24 | 3410.8712 | 853.7251 | 21483.87 | 4 |  |  |  |  |  |
| 519 | 25 | 3043.6253 | 761.9136 | 19742.47 | 4 | 3043.6422 | C25 | 25 | -0.0169 | -5.57 |
| 519 | 26 | 3354.8446 | 839.7184 | 17264.57 | 4 | 3354.8631 | C28 | 28 | -0.0185 | -5.51 |
| 519 | 27 | 1265.7881 | 633.9013 | 26390.42 | 2 | 1265.7875 | Z\_DOT13 | 17 | 6.58e-04 | 0.52 |
| 519 | 28 | 3255.7769 | 814.9515 | 15632.20 | 4 | 3255.7947 | C27 | 27 | -0.0178 | -5.47 |
| 519 | 29 | 2716.4002 | 680.1073 | 16446.82 | 4 | 2716.4152 | C22 | 22 | -0.0150 | -5.53 |
| 519 | 30 | 3382.8746 | 846.7259 | 24423.59 | 4 |  |  |  |  |  |
| 519 | 31 | 1557.9524 | 779.9835 | 23105.10 | 2 |  |  |  |  |  |
| 519 | 32 | 1756.8621 | 879.4383 | 20480.38 | 2 | 1756.8712 | C14 | 14 | -9.10e-03 | -5.18 |
| 519 | 33 | 1643.7788 | 822.8967 | 18932.26 | 2 | 1643.7871 | C13 | 13 | -8.29e-03 | -5.05 |
| 519 | 34 | 3226.7508 | 807.6950 | 14965.31 | 4 |  |  |  |  |  |
| 519 | 35 | 1713.9462 | 857.9804 | 26583.98 | 2 |  |  |  |  |  |
| 519 | 36 | 685.5779 | 686.5851 | 81477.03 | 1 |  |  |  |  |  |
| 519 | 37 | 1007.4844 | 1008.4916 | 15780.83 | 1 | 1007.4892 | C8 | 8 | -4.81e-03 | -4.77 |
| 519 | 38 | 908.5764 | 455.2955 | 20541.88 | 2 |  |  |  |  |  |
| 519 | 39 | 710.4895 | 711.4968 | 10579.94 | 1 | 710.4858 | Z\_DOT8 | 22 | 3.74e-03 | 5.26 |
| 519 | 40 | 1206.7749 | 604.3947 | 7004.33 | 2 |  |  |  |  |  |
| 519 | 41 | 1135.5427 | 1136.5500 | 6585.68 | 1 | 1135.5477 | C9 | 9 | -5.04e-03 | -4.44 |
| 519 | 42 | 586.3775 | 587.3848 | 5208.69 | 1 | 586.3802 | C5 | 5 | -2.63e-03 | -4.49 |
| 519 | 43 | 1007.4842 | 504.7494 | 4213.12 | 2 | 1007.4892 | C8 | 8 | -4.94e-03 | -4.91 |
| 519 | 44 | 511.3587 | 512.3660 | 5133.46 | 1 | 511.3537 | Z\_DOT6 | 24 | 4.95e-03 | 9.67 |
| 519 | 45 | 553.0763 | 554.0836 | 6508.18 | 1 |  |  |  |  |  |
| 519 | 46 | 873.4698 | 874.4771 | 3889.25 | 1 |  |  |  |  |  |
| 519 | 47 | 473.2942 | 474.3015 | 6018.43 | 1 | 473.2961 | C4 | 4 | -1.91e-03 | -4.04 |
| 519 | 48 | 967.6495 | 484.8320 | 3234.05 | 2 |  |  |  |  |  |
| 519 | 49 | 1378.6285 | 1379.6358 | 2139.45 | 1 | 1378.6333 | C11 | 11 | -4.74e-03 | -3.44 |
| 519 | 50 | 847.4541 | 848.4614 | 7913.14 | 1 | 847.4585 | C7 | 7 | -4.36e-03 | -5.15 |
| 519 | 51 | 1263.5994 | 1264.6066 | 2381.77 | 1 | 1263.6063 | C10 | 10 | -6.98e-03 | -5.52 |
| 519 | 52 | 344.2522 | 345.2595 | 3352.09 | 1 | 344.2535 | C3 | 3 | -1.28e-03 | -3.73 |
| 519 | 53 | 780.5186 | 391.2666 | 3381.82 | 2 |  |  |  |  |  |
| 519 | 54 | 726.5085 | 727.5158 | 2364.45 | 1 |  |  |  |  |  |
| 519 | 55 | 1485.8713 | 496.2977 | 1545.47 | 3 |  |  |  |  |  |
| 519 | 56 | 417.0757 | 418.0829 | 1581.59 | 1 |  |  |  |  |  |
| 519 | 57 | 1024.6701 | 513.3423 | 1621.29 | 2 |  |  |  |  |  |

  

All proteins /
CsTx-12b Cupiennius salei toxin 12 isoform b /
Proteoform #47
