# Supplementary material for: The Dual Prey-Inactivation Strategy of Spiders—In-Depth Venomic Analysis of Cupiennius salei
Source: Toxins (Basel). 2019 Mar 19;11(3):167. doi: 10.3390/toxins11030167 (PMC6468893; doi:10.3390/toxins11030167)
Supplement: Supplementary file 1 [file toxins-11-00167-s001.zip › Supplementary Dataset EV1/20180328_f2_topdown_OTMS2_EThcD_NL_i02_ms2_proteoform_cutoff_html/prsms/prsm15.html]

Protein-Spectrum-Match for Spectrum #227


All proteins /
CsTx-13a Cupiennius salei toxin 13 isoform a /
Proteoform #91

## Protein-Spectrum-Match #15 for Spectrum #227

|  |  |  |  |  |  |
| --- | --- | --- | --- | --- | --- |
| PrSM ID: | 15 | Scan(s): | 305 | Precursor charge: | 7 |
| Precursor m/z: | 629.5471 | Precursor mass: | 4399.7789 | Proteoform mass: | 4399.8639 |
| # matched peaks: | 15 | # matched fragment ions: | 14 | # unexpected modifications: | 1 |
| E-value: | 1.39e-11 | P-value: | 1.39e-11 | Q-value (Spectral FDR): | 0 |

  

|  |  |  |  |  |  |  |  |  |  |  |  |  |  |  |  |  |  |  |  |  |  |  |  |  |  |  |  |  |  |  |  |  |  |  |  |  |  |  |  |  |  |  |  |  |  |  |  |  |  |  |  |  |  |  |  |  |  |  |  |  |  |  |  |  |  |  |  |  |  |
| --- | --- | --- | --- | --- | --- | --- | --- | --- | --- | --- | --- | --- | --- | --- | --- | --- | --- | --- | --- | --- | --- | --- | --- | --- | --- | --- | --- | --- | --- | --- | --- | --- | --- | --- | --- | --- | --- | --- | --- | --- | --- | --- | --- | --- | --- | --- | --- | --- | --- | --- | --- | --- | --- | --- | --- | --- | --- | --- | --- | --- | --- | --- | --- | --- | --- | --- | --- | --- | --- |
|  | |  | | | | | | | | | | | | | | | | | | | | | | | | | | | | | | | | | | | | | | | | | | | | | | | | | | | | | | | | | | | | | | | | | | | |
| 1 |  |  | M |  | K |  | V |  | L |  | V |  | I |  | F |  | A |  | V |  | L |  |  | S |  | L |  | V |  | I |  | F |  | S |  | N |  | C |  | S |  | A |  |  | E |  | T |  | D |  | E |  | D |  | F |  | F |  | G |  | E |  | E |  | 30 |  |
|  | |  | | | | | | | | | | | | | | | | | | | | | | | | | | | | | | | | | | | | | | | | | | | | | | | | | | | | | | | | | | | | | | | | | | | |
| 31 |  |  | S |  | F |  | E |  | A |  | D |  | D |  | I |  | I |  | P |  | F |  |  | I |  | A |  | K |  | E |  | Q |  | V |  | R | ] | S |  | D |  | C |  |  | T |  | L | ⎫ | R | ⎫ | N | ⎫ | H | ⎫ | D | ⎫ | C |  | T | ⎫ | D | ⎫ | D |  | 60 |  |
|  | |  | | | | | | | | | | | | | | | | | 369.25 | | | | | | | | | | | | | | | | | | | | | | | | | | | | | | | | | | | | | | | | | | | | | | | |
| 61 |  | ⎫ | R | ⎫ | H | ⎫ | S | ⎫ | C |  | C | ⎫ | R |  | S |  | K | ⎫ | M |  | F |  | ⎩ | K |  | D |  | V |  | C |  | T |  | C |  | F |  | Y | [ | P |  | S |  |  | Q |  | R |  | S |  | E |  | T |  | A |  | R |  | A |  | K |  | K |  | 90 |  |
|  | |  | | | | | | | | | | | | | | | | | | | | | | | | | | | | | | | | | | | | | | | | | | | | | | | | | | | | | | | | | | | | | | | | | | | |
| 91 |  |  | E |  | L |  | C |  | T |  | C |  | Q |  | Q |  | P |  | K |  | H |  |  | L |  | K |  | Y |  | I |  | E |  | K |  | G |  | L |  | Q |  | K |  |  | A |  | K |  | D |  | Y |  | A |  | T |  | G |  | | 117 |  | | | | | |

Fixed PTMs: Carbamidomethylation [C50 C57 C64 C65 C74 C76 ]   
  
     Unexpected modifications:   Unknown [369.25]

  

All peaks (73)  Matched peaks (15)  Not matched peaks (58)

  

| Scan | Peak | Mono mass | Mono m/z | Intensity | Charge | Theoretical mass | Ion | Pos | Mass error | PPM error |
| --- | --- | --- | --- | --- | --- | --- | --- | --- | --- | --- |
| 305 | 1 | 4276.7150 | 611.9666 | 30839.25 | 7 |  |  |  |  |  |
| 305 | 2 | 4295.7326 | 716.9627 | 36215.31 | 6 |  |  |  |  |  |
| 305 | 3 | 4236.7012 | 707.1241 | 30304.60 | 6 |  |  |  |  |  |
| 305 | 4 | 4296.7394 | 860.3552 | 27983.89 | 5 |  |  |  |  |  |
| 305 | 5 | 4343.7358 | 724.9632 | 21216.80 | 6 |  |  |  |  |  |
| 305 | 6 | 4238.7093 | 848.7491 | 17607.42 | 5 |  |  |  |  |  |
| 305 | 7 | 3964.5742 | 793.9221 | 18047.49 | 5 |  |  |  |  |  |
| 305 | 8 | 2678.0731 | 670.5255 | 16407.04 | 4 | 2678.0914 | C21 | 21 | -0.0184 | -6.86 |
| 305 | 9 | 4165.6835 | 834.1440 | 17545.44 | 5 |  |  |  |  |  |
| 305 | 10 | 4277.7194 | 713.9605 | 15253.46 | 6 |  |  |  |  |  |
| 305 | 11 | 4148.6579 | 692.4503 | 11636.57 | 6 |  |  |  |  |  |
| 305 | 12 | 3986.6503 | 798.3373 | 15398.26 | 5 |  |  |  |  |  |
| 305 | 13 | 4278.7200 | 856.7513 | 12860.19 | 5 |  |  |  |  |  |
| 305 | 14 | 4344.7403 | 869.9553 | 12618.12 | 5 |  |  |  |  |  |
| 305 | 15 | 4219.6954 | 704.2899 | 11106.24 | 6 |  |  |  |  |  |
| 305 | 16 | 2761.1089 | 691.2845 | 9153.67 | 4 |  |  |  |  |  |
| 305 | 17 | 4294.7230 | 614.5391 | 12046.68 | 7 |  |  |  |  |  |
| 305 | 18 | 4078.6492 | 816.7371 | 7375.64 | 5 |  |  |  |  |  |
| 305 | 19 | 1491.5738 | 746.7942 | 10098.93 | 2 | 1491.5830 | C12 | 12 | -9.26e-03 | -6.21 |
| 305 | 20 | 4390.7767 | 732.8034 | 14690.22 | 6 |  |  |  |  |  |
| 305 | 21 | 3964.5754 | 661.7698 | 6413.92 | 6 |  |  |  |  |  |
| 305 | 22 | 2908.1771 | 728.0515 | 8018.01 | 4 |  |  |  |  |  |
| 305 | 23 | 4255.7006 | 710.2907 | 5626.00 | 6 |  |  |  |  |  |
| 305 | 24 | 3036.2725 | 760.0754 | 5277.92 | 4 |  |  |  |  |  |
| 305 | 25 | 2201.3847 | 734.8022 | 8303.19 | 3 |  |  |  |  |  |
| 305 | 26 | 2121.3570 | 708.1263 | 11665.34 | 3 |  |  |  |  |  |
| 305 | 27 | 1606.5988 | 804.3067 | 6779.41 | 2 | 1606.6100 | C13 | 13 | -0.0112 | -6.96 |
| 305 | 28 | 1258.4739 | 630.2442 | 10759.51 | 2 |  |  |  |  |  |
| 305 | 29 | 1376.5464 | 689.2805 | 6274.19 | 2 | 1376.5561 | C11 | 11 | -9.67e-03 | -7.02 |
| 305 | 30 | 4389.7685 | 628.1171 | 9341.15 | 7 |  |  |  |  |  |
| 305 | 31 | 4180.6807 | 837.1434 | 6405.31 | 5 |  |  |  |  |  |
| 305 | 32 | 3410.3934 | 683.0860 | 5330.28 | 5 |  |  |  |  |  |
| 305 | 33 | 3801.5109 | 761.3095 | 6928.91 | 5 |  |  |  |  |  |
| 305 | 34 | 2058.9155 | 687.3124 | 4466.01 | 3 |  |  |  |  |  |
| 305 | 35 | 2678.0751 | 893.6990 | 5149.71 | 3 | 2678.0914 | C21 | 21 | -0.0163 | -6.10 |
| 305 | 36 | 4060.6215 | 677.7775 | 4314.71 | 6 |  |  |  |  |  |
| 305 | 37 | 3987.6522 | 997.9203 | 4420.84 | 4 |  |  |  |  |  |
| 305 | 38 | 628.3898 | 629.3970 | 10261.35 | 1 |  |  |  |  |  |
| 305 | 39 | 4372.7639 | 729.8013 | 7188.38 | 6 |  |  |  |  |  |
| 305 | 40 | 2306.8489 | 769.9569 | 3483.66 | 3 | 2306.8633 | C18 | 18 | -0.0144 | -6.26 |
| 305 | 41 | 3907.5509 | 782.5175 | 4277.52 | 5 |  |  |  |  |  |
| 305 | 42 | 1986.7867 | 994.4006 | 4483.79 | 2 | 1986.8020 | C16 | 16 | -0.0153 | -7.71 |
| 305 | 43 | 3036.2721 | 608.2617 | 5615.05 | 5 |  |  |  |  |  |
| 305 | 44 | 1474.5469 | 738.2807 | 4834.70 | 2 |  |  |  |  |  |
| 305 | 45 | 1762.7022 | 882.3584 | 6590.97 | 2 | 1762.7111 | C14 | 14 | -8.90e-03 | -5.05 |
| 305 | 46 | 1899.7568 | 950.8857 | 4279.74 | 2 | 1899.7700 | C15 | 15 | -0.0132 | -6.95 |
| 305 | 47 | 3103.2718 | 776.8252 | 3131.45 | 4 |  |  |  |  |  |
| 305 | 48 | 2761.1101 | 921.3773 | 3448.98 | 3 |  |  |  |  |  |
| 305 | 49 | 3511.4416 | 703.2956 | 4284.25 | 5 |  |  |  |  |  |
| 305 | 50 | 3250.3645 | 651.0802 | 3214.07 | 5 |  |  |  |  |  |
| 305 | 51 | 4261.7295 | 853.3532 | 4764.60 | 5 |  |  |  |  |  |
| 305 | 52 | 3141.6589 | 629.3391 | 4643.17 | 5 |  |  |  |  |  |
| 305 | 53 | 4149.6758 | 830.9424 | 6463.10 | 5 |  |  |  |  |  |
| 305 | 54 | 330.1524 | 331.1597 | 5618.89 | 1 |  |  |  |  |  |
| 305 | 55 | 749.3445 | 750.3517 | 4594.31 | 1 | 749.3490 | C6 | 6 | -4.51e-03 | -6.01 |
| 305 | 56 | 863.3865 | 864.3937 | 2511.48 | 1 | 863.3919 | C7 | 7 | -5.44e-03 | -6.30 |
| 305 | 57 | 1000.4447 | 501.2296 | 7022.82 | 2 | 1000.4508 | C8 | 8 | -6.08e-03 | -6.08 |
| 305 | 58 | 1115.4705 | 558.7425 | 4237.35 | 2 | 1115.4778 | C9 | 9 | -7.26e-03 | -6.51 |
| 305 | 59 | 694.2920 | 695.2993 | 1224.12 | 1 |  |  |  |  |  |
| 305 | 60 | 880.5543 | 881.5616 | 1607.71 | 1 |  |  |  |  |  |
| 305 | 61 | 1357.5816 | 679.7981 | 866.10 | 2 |  |  |  |  |  |
| 305 | 62 | 763.8315 | 764.8387 | 838.59 | 1 |  |  |  |  |  |
| 305 | 63 | 707.9520 | 708.9592 | 1893.59 | 1 |  |  |  |  |  |
| 305 | 64 | 818.3273 | 819.3346 | 632.52 | 1 |  |  |  |  |  |
| 305 | 65 | 662.2635 | 663.2708 | 1078.54 | 1 |  |  |  |  |  |
| 305 | 66 | 1032.1669 | 1033.1741 | 731.07 | 1 |  |  |  |  |  |
| 305 | 67 | 1056.6750 | 1057.6823 | 856.55 | 1 |  |  |  |  |  |
| 305 | 68 | 901.1191 | 902.1263 | 581.26 | 1 |  |  |  |  |  |
| 305 | 69 | 1075.4137 | 1076.4210 | 452.04 | 1 | 1075.4229 | Z\_DOT8 | 23 | -9.23e-03 | -8.58 |
| 305 | 70 | 1260.4690 | 1261.4763 | 744.65 | 1 |  |  |  |  |  |
| 305 | 71 | 1288.5151 | 1289.5224 | 481.97 | 1 |  |  |  |  |  |
| 305 | 72 | 593.2442 | 594.2515 | 775.19 | 1 | 593.2479 | C5 | 5 | -3.68e-03 | -6.20 |
| 305 | 73 | 721.1206 | 722.1279 | 1388.06 | 1 |  |  |  |  |  |

  

All proteins /
CsTx-13a Cupiennius salei toxin 13 isoform a /
Proteoform #91
