# Supplementary material for: The Dual Prey-Inactivation Strategy of Spiders—In-Depth Venomic Analysis of Cupiennius salei
Source: Toxins (Basel). 2019 Mar 19;11(3):167. doi: 10.3390/toxins11030167 (PMC6468893; doi:10.3390/toxins11030167)
Supplement: Supplementary file 1 [file toxins-11-00167-s001.zip › Supplementary Dataset EV1/20180328_f2_topdown_OTMS2_EThcD_NL_i02_ms2_proteoform_cutoff_html/prsms/prsm150.html]

Protein-Spectrum-Match for Spectrum #388


All proteins /
CsTx-13a Cupiennius salei toxin 13 isoform a /
Proteoform #40

## Protein-Spectrum-Match #150 for Spectrum #388

|  |  |  |  |  |  |
| --- | --- | --- | --- | --- | --- |
| PrSM ID: | 150 | Scan(s): | 520 | Precursor charge: | 6 |
| Precursor m/z: | 580.3142 | Precursor mass: | 3475.8413 | Proteoform mass: | 3475.8363 |
| # matched peaks: | 31 | # matched fragment ions: | 27 | # unexpected modifications: | 1 |
| E-value: | 5.31e-21 | P-value: | 5.31e-21 | Q-value (Spectral FDR): | 0 |

  

|  |  |  |  |  |  |  |  |  |  |  |  |  |  |  |  |  |  |  |  |  |  |  |  |  |  |  |  |  |  |  |  |  |  |  |  |  |  |  |  |  |  |  |  |  |  |  |  |  |  |  |  |  |  |  |  |  |  |  |  |  |  |  |  |  |  |  |
| --- | --- | --- | --- | --- | --- | --- | --- | --- | --- | --- | --- | --- | --- | --- | --- | --- | --- | --- | --- | --- | --- | --- | --- | --- | --- | --- | --- | --- | --- | --- | --- | --- | --- | --- | --- | --- | --- | --- | --- | --- | --- | --- | --- | --- | --- | --- | --- | --- | --- | --- | --- | --- | --- | --- | --- | --- | --- | --- | --- | --- | --- | --- | --- | --- | --- | --- |
|  | | ... 30 amino acid residues are skipped at the N-terminus ... | | | | | | | | | | | | | | | | | | | | | | | | | | | | | | | | | | | | | | | | | | | | | | | | | | | | | | | | | | | | | |  | | |
|  | |  | | | | | | | | | | | | | | | | | | | | | | | | | | | | | | | | | | | | | | | | | | | | | | | | | | | | | | | | | | | | | | | | | | | |
| 31 |  |  | S |  | F |  | E |  | A |  | D |  | D |  | I |  | I |  | P |  | F |  |  | I |  | A |  | K |  | E |  | Q |  | V |  | R |  | S |  | D |  | C |  |  | T |  | L |  | R |  | N |  | H |  | D |  | C |  | T |  | D |  | D |  | 60 |  |
|  | |  | | | | | | | | | | | | | | | | | | | | | | | | | | | | | | | | | | | | | | | | | | | | | | | | | | | | | | | | | | | | | | | | | | | |
| 61 |  |  | R |  | H |  | S |  | C |  | C |  | R |  | S |  | K |  | M |  | F |  |  | K |  | D |  | V |  | C |  | T |  | C |  | F |  | Y |  | P |  | S |  |  | Q |  | R |  | S |  | E |  | T |  | A |  | R | ] | A | ⎩ | K | ⎩ | K |  | 90 |  |
|  | |  | | | | | | | | | | | | | | | | | | | | | | | | | | | | | | | | | | | | | | | | | | | | | | | | | | | -58.01 | | | | | | | | | | | | | |
| 91 |  |  | E | ⎱ | L |  | C | ⎫ | T | ⎫ | C | ⎫ | Q | ⎱ | Q |  | P | ⎱ | K | ⎫ | H |  |  | L |  | K | ⎱ | Y | ⎫ | I | ⎱ | E | ⎱ | K | ⎫ | G |  | L |  | Q | ⎱ | K |  | ⎫ | A | ⎫ | K | ⎫ | D | ⎫ | Y | ⎫ | A |  | T |  | G |  | | 117 |  | | | | | |

Fixed PTMs: Carbamidomethylation [C93 C95 ]   
  
     Unexpected modifications:   Unknown [-58.01]

  

All peaks (57)  Matched peaks (31)  Not matched peaks (26)

  

| Scan | Peak | Mono mass | Mono m/z | Intensity | Charge | Theoretical mass | Ion | Pos | Mass error | PPM error |
| --- | --- | --- | --- | --- | --- | --- | --- | --- | --- | --- |
| 520 | 1 | 3418.8013 | 684.7675 | 87698.64 | 5 |  |  |  |  |  |
| 520 | 2 | 3474.8288 | 580.1454 | 182381.47 | 6 |  |  |  |  |  |
| 520 | 3 | 3140.6766 | 786.1764 | 37015.06 | 4 | 3140.6950 | C26 | 26 | -0.0184 | -5.87 |
| 520 | 4 | 3025.6509 | 757.4200 | 39599.07 | 4 | 3025.6680 | C25 | 25 | -0.0171 | -5.66 |
| 520 | 5 | 3418.8014 | 855.7076 | 29682.45 | 4 |  |  |  |  |  |
| 520 | 6 | 2272.1694 | 758.3971 | 34143.76 | 3 | 2272.1820 | C18 | 18 | -0.0125 | -5.51 |
| 520 | 7 | 1158.9435 | 580.4790 | 140949.18 | 2 |  |  |  |  |  |
| 520 | 8 | 2826.5191 | 707.6370 | 23217.63 | 4 | 2826.5360 | C23 | 23 | -0.0169 | -5.97 |
| 520 | 9 | 1866.9816 | 623.3345 | 32077.43 | 3 | 1866.9920 | C15 | 15 | -0.0104 | -5.57 |
| 520 | 10 | 2698.4258 | 900.4825 | 22976.97 | 3 | 2698.4410 | C22 | 22 | -0.0152 | -5.65 |
| 520 | 11 | 3459.8062 | 692.9685 | 23469.94 | 5 |  |  |  |  |  |
| 520 | 12 | 2116.1801 | 706.4006 | 24842.17 | 3 | 2116.1850 | Z\_DOT19 | 11 | -4.97e-03 | -2.35 |
| 520 | 13 | 2143.1273 | 715.3830 | 24570.95 | 3 | 2143.1394 | C17 | 17 | -0.0120 | -5.62 |
| 520 | 14 | 3303.7396 | 826.9422 | 16292.69 | 4 | 3303.7583 | C27 | 27 | -0.0188 | -5.68 |
| 520 | 15 | 2800.4524 | 701.1204 | 20069.81 | 4 |  |  |  |  |  |
| 520 | 16 | 579.6384 | 580.6457 | 120360.29 | 1 |  |  |  |  |  |
| 520 | 17 | 2539.3772 | 635.8516 | 18045.23 | 4 |  |  |  |  |  |
| 520 | 18 | 2341.2910 | 781.4376 | 18937.37 | 3 | 2341.2964 | Z\_DOT21 | 9 | -5.36e-03 | -2.29 |
| 520 | 19 | 1609.8499 | 805.9322 | 30284.11 | 2 | 1609.8522 | Z\_DOT15 | 15 | -2.26e-03 | -1.41 |
| 520 | 20 | 1625.8687 | 813.9416 | 21821.57 | 2 |  |  |  |  |  |
| 520 | 21 | 3432.8182 | 859.2118 | 13779.58 | 4 |  |  |  |  |  |
| 520 | 22 | 3260.6753 | 816.1761 | 18382.06 | 4 | 3260.6856 | Z\_DOT28 | 2 | -0.0102 | -3.14 |
| 520 | 23 | 1360.6519 | 681.3332 | 20247.67 | 2 | 1360.6591 | C11 | 11 | -7.20e-03 | -5.29 |
| 520 | 24 | 3025.6504 | 1009.5574 | 12322.92 | 3 | 3025.6680 | C25 | 25 | -0.0176 | -5.82 |
| 520 | 25 | 3388.7656 | 678.7604 | 14287.68 | 5 | 3388.7805 | Z\_DOT29 | 1 | -0.0149 | -4.41 |
| 520 | 26 | 3303.7379 | 661.7549 | 14124.00 | 5 | 3303.7583 | C27 | 27 | -0.0204 | -6.18 |
| 520 | 27 | 3459.8112 | 865.9601 | 11574.93 | 4 |  |  |  |  |  |
| 520 | 28 | 695.5665 | 696.5738 | 83710.89 | 1 |  |  |  |  |  |
| 520 | 29 | 2960.4817 | 741.1277 | 10246.02 | 4 |  |  |  |  |  |
| 520 | 30 | 1204.6626 | 603.3386 | 15282.88 | 2 | 1204.6622 | Z\_DOT12 | 18 | 4.61e-04 | 0.38 |
| 520 | 31 | 3431.8130 | 687.3699 | 8509.56 | 5 |  |  |  |  |  |
| 520 | 32 | 1333.7045 | 667.8595 | 11656.67 | 2 | 1333.7048 | Z\_DOT13 | 17 | -2.58e-04 | -0.19 |
| 520 | 33 | 3004.5443 | 1002.5220 | 10246.60 | 3 |  |  |  |  |  |
| 520 | 34 | 2030.0441 | 677.6887 | 9089.29 | 3 | 2030.0553 | C16 | 16 | -0.0112 | -5.50 |
| 520 | 35 | 2400.2632 | 801.0950 | 7629.88 | 3 | 2400.2769 | C19 | 19 | -0.0137 | -5.70 |
| 520 | 36 | 3003.5372 | 751.8916 | 10210.94 | 4 | 3003.5480 | Z\_DOT26 | 4 | -0.0108 | -3.61 |
| 520 | 37 | 2897.5569 | 725.3965 | 9452.54 | 4 | 2897.5731 | C24 | 24 | -0.0162 | -5.57 |
| 520 | 38 | 1739.4201 | 870.7173 | 33569.18 | 2 |  |  |  |  |  |
| 520 | 39 | 1135.5421 | 568.7784 | 13540.67 | 2 | 1135.5477 | C9 | 9 | -5.60e-03 | -4.93 |
| 520 | 40 | 1135.5421 | 1136.5494 | 5453.90 | 1 | 1135.5477 | C9 | 9 | -5.65e-03 | -4.97 |
| 520 | 41 | 473.2941 | 474.3014 | 10654.54 | 1 | 473.2961 | C4 | 4 | -2.00e-03 | -4.24 |
| 520 | 42 | 1488.7460 | 745.3803 | 8811.99 | 2 | 1488.7540 | C12 | 12 | -8.04e-03 | -5.40 |
| 520 | 43 | 1488.7464 | 497.2561 | 5141.19 | 3 | 1488.7540 | C12 | 12 | -7.66e-03 | -5.14 |
| 520 | 44 | 869.4615 | 870.4688 | 8861.57 | 1 |  |  |  |  |  |
| 520 | 45 | 1007.4841 | 1008.4913 | 3949.20 | 1 | 1007.4892 | C8 | 8 | -5.11e-03 | -5.07 |
| 520 | 46 | 564.0610 | 565.0682 | 7557.07 | 1 |  |  |  |  |  |
| 520 | 47 | 1220.6809 | 611.3477 | 5879.25 | 2 |  |  |  |  |  |
| 520 | 48 | 847.4556 | 848.4628 | 4452.62 | 1 | 847.4585 | C7 | 7 | -2.96e-03 | -3.49 |
| 520 | 49 | 1274.6916 | 638.3531 | 3174.98 | 2 |  |  |  |  |  |
| 520 | 50 | 1300.6234 | 651.3190 | 9801.99 | 2 |  |  |  |  |  |
| 520 | 51 | 976.4928 | 489.2537 | 3093.28 | 2 |  |  |  |  |  |
| 520 | 52 | 778.4057 | 779.4130 | 3724.23 | 1 | 778.4031 | Z\_DOT8 | 22 | 2.58e-03 | 3.31 |
| 520 | 53 | 1421.6310 | 711.8228 | 2129.81 | 2 |  |  |  |  |  |
| 520 | 54 | 908.5764 | 455.2955 | 2034.11 | 2 |  |  |  |  |  |
| 520 | 55 | 1378.8709 | 690.4427 | 1863.32 | 2 |  |  |  |  |  |
| 520 | 56 | 1098.6214 | 550.3180 | 1588.45 | 2 |  |  |  |  |  |
| 520 | 57 | 746.4067 | 747.4140 | 1967.96 | 1 | 746.4108 | C6 | 6 | -4.11e-03 | -5.51 |

  

All proteins /
CsTx-13a Cupiennius salei toxin 13 isoform a /
Proteoform #40
