# Supplementary material for: The Dual Prey-Inactivation Strategy of Spiders—In-Depth Venomic Analysis of Cupiennius salei
Source: Toxins (Basel). 2019 Mar 19;11(3):167. doi: 10.3390/toxins11030167 (PMC6468893; doi:10.3390/toxins11030167)
Supplement: Supplementary file 1 [file toxins-11-00167-s001.zip › Supplementary Dataset EV1/20180328_f2_topdown_OTMS2_EThcD_NL_i02_ms2_proteoform_cutoff_html/prsms/prsm151.html]

Protein-Spectrum-Match for Spectrum #389


All proteins /
sp|B3EWU1|TXS1A\_CUPSA Short cationic peptide-1a OS=Cupiennius salei OX=6928 PE=1 SV=1 /
Proteoform #23

## Protein-Spectrum-Match #151 for Spectrum #389

|  |  |  |  |  |  |
| --- | --- | --- | --- | --- | --- |
| PrSM ID: | 151 | Scan(s): | 521 | Precursor charge: | 6 |
| Precursor m/z: | 513.6361 | Precursor mass: | 3075.7730 | Proteoform mass: | 3075.7680 |
| # matched peaks: | 39 | # matched fragment ions: | 35 | # unexpected modifications: | 1 |
| E-value: | 3.81e-26 | P-value: | 3.81e-26 | Q-value (Spectral FDR): | 0 |

  

|  |  |  |  |  |  |  |  |  |  |  |  |  |  |  |  |  |  |  |  |  |  |  |  |  |  |  |  |  |  |  |  |  |  |  |  |  |  |  |  |  |  |  |  |  |  |  |  |  |  |  |  |  |  |  |  |  |  |  |  |  |  |  |  |  |  |  |
| --- | --- | --- | --- | --- | --- | --- | --- | --- | --- | --- | --- | --- | --- | --- | --- | --- | --- | --- | --- | --- | --- | --- | --- | --- | --- | --- | --- | --- | --- | --- | --- | --- | --- | --- | --- | --- | --- | --- | --- | --- | --- | --- | --- | --- | --- | --- | --- | --- | --- | --- | --- | --- | --- | --- | --- | --- | --- | --- | --- | --- | --- | --- | --- | --- | --- | --- |
|  | |  | | | | | | | | | | | | | | | | | | | | | | | | | | | | | | | | | | | | | | | | | | | | | | | | | | | | | | | | | -0.99 | | | | | | | |
| 1 |  |  | F | ⎩ | L | ⎩ | A |  | K | ⎱ | K |  | V | ⎱ | A | ⎫ | K | ⎱ | T | ⎱ | V |  | ⎱ | A | ⎫ | K | ⎫ | Q | ⎫ | A |  | A |  | K | ⎱ | Q | ⎱ | G | ⎱ | A | ⎱ | K |  | ⎱ | Y | ⎱ | V | ⎱ | V | ⎫ | N | ⎫ | K | ⎫ | Q | ⎫ | M | ⎫ | E |  | | 28 |  | | | |

Unexpected modifications:   Unknown [-0.99]

  

All peaks (51)  Matched peaks (39)  Not matched peaks (12)

  

| Scan | Peak | Mono mass | Mono m/z | Intensity | Charge | Theoretical mass | Ion | Pos | Mass error | PPM error |
| --- | --- | --- | --- | --- | --- | --- | --- | --- | --- | --- |
| 521 | 1 | 3075.7639 | 513.6346 | 247686.77 | 6 |  |  |  |  |  |
| 521 | 2 | 2174.1633 | 725.7284 | 100645.70 | 3 | 2174.1693 | Z\_DOT20 | 8 | -6.00e-03 | -2.76 |
| 521 | 3 | 3059.7379 | 612.9549 | 84497.81 | 5 |  |  |  |  |  |
| 521 | 4 | 2946.7146 | 737.6859 | 61334.30 | 4 | 2946.7316 | C27 | 27 | -0.0170 | -5.76 |
| 521 | 5 | 2815.6751 | 704.9260 | 53811.92 | 4 | 2815.6911 | C26 | 26 | -0.0160 | -5.70 |
| 521 | 6 | 1885.1520 | 629.3913 | 65288.54 | 3 | 1885.1624 | C18 | 18 | -0.0104 | -5.51 |
| 521 | 7 | 1376.6922 | 689.3534 | 47794.60 | 2 | 1376.6934 | Z\_DOT12 | 16 | -1.24e-03 | -0.90 |
| 521 | 8 | 3075.7565 | 616.1586 | 130611.90 | 5 |  |  |  |  |  |
| 521 | 9 | 1700.0733 | 567.6984 | 67636.37 | 3 | 1700.0824 | C16 | 16 | -9.14e-03 | -5.37 |
| 521 | 10 | 902.6021 | 452.3083 | 62646.18 | 2 | 902.6065 | C8 | 8 | -4.39e-03 | -4.87 |
| 521 | 11 | 2073.1162 | 692.0460 | 30559.18 | 3 | 2073.1217 | Z\_DOT19 | 9 | -5.44e-03 | -2.62 |
| 521 | 12 | 2445.4815 | 612.3776 | 34322.05 | 4 | 2445.4947 | C23 | 23 | -0.0132 | -5.38 |
| 521 | 13 | 3058.7346 | 510.7964 | 30466.43 | 6 |  |  |  |  |  |
| 521 | 14 | 1974.0483 | 659.0234 | 39025.52 | 3 | 1974.0532 | Z\_DOT18 | 10 | -4.95e-03 | -2.51 |
| 521 | 15 | 2687.6172 | 672.9116 | 31210.02 | 4 | 2687.6325 | C25 | 25 | -0.0153 | -5.70 |
| 521 | 16 | 1956.1886 | 653.0701 | 39897.52 | 3 | 1956.1995 | C19 | 19 | -0.0110 | -5.62 |
| 521 | 17 | 512.9605 | 513.9678 | 109163.74 | 1 |  |  |  |  |  |
| 521 | 18 | 2600.4566 | 651.1214 | 26657.69 | 4 | 2600.4648 | Z\_DOT24 | 4 | -8.18e-03 | -3.14 |
| 521 | 19 | 2912.6710 | 729.1750 | 21671.78 | 4 | 2912.6809 | Z\_DOT27 | 1 | -9.95e-03 | -3.42 |
| 521 | 20 | 3017.7296 | 604.5532 | 23133.58 | 5 |  |  |  |  |  |
| 521 | 21 | 3060.7430 | 766.1930 | 31034.87 | 4 |  |  |  |  |  |
| 521 | 22 | 2559.5232 | 640.8881 | 24934.21 | 4 | 2559.5376 | C24 | 24 | -0.0144 | -5.61 |
| 521 | 23 | 2051.1753 | 513.8011 | 153190.56 | 4 |  |  |  |  |  |
| 521 | 24 | 1102.7170 | 552.3658 | 30205.58 | 2 | 1102.7226 | C10 | 10 | -5.62e-03 | -5.09 |
| 521 | 25 | 1828.1312 | 610.3843 | 23580.17 | 3 | 1828.1410 | C17 | 17 | -9.81e-03 | -5.37 |
| 521 | 26 | 1173.7533 | 587.8839 | 22164.90 | 2 | 1173.7597 | C11 | 11 | -6.35e-03 | -5.41 |
| 521 | 27 | 1191.6132 | 596.8139 | 23586.26 | 2 | 1191.6134 | Z\_DOT10 | 18 | -1.76e-04 | -0.15 |
| 521 | 28 | 2084.2820 | 522.0778 | 19527.34 | 4 | 2084.2945 | C20 | 20 | -0.0125 | -5.99 |
| 521 | 29 | 2247.3448 | 562.8435 | 24280.77 | 4 | 2247.3578 | C21 | 21 | -0.0131 | -5.81 |
| 521 | 30 | 2373.2947 | 792.1055 | 13688.03 | 3 | 2373.3014 | Z\_DOT22 | 6 | -6.66e-03 | -2.81 |
| 521 | 31 | 2346.4156 | 587.6112 | 15463.48 | 4 | 2346.4262 | C22 | 22 | -0.0107 | -4.55 |
| 521 | 32 | 1003.6493 | 502.8319 | 17609.96 | 2 | 1003.6542 | C9 | 9 | -4.89e-03 | -4.88 |
| 521 | 33 | 2799.5872 | 700.9041 | 18055.19 | 4 | 2799.5968 | Z\_DOT26 | 2 | -9.64e-03 | -3.44 |
| 521 | 34 | 1429.9054 | 715.9600 | 12414.31 | 2 | 1429.9132 | C13 | 13 | -7.79e-03 | -5.45 |
| 521 | 35 | 1429.9055 | 477.6425 | 10394.61 | 3 | 1429.9132 | C13 | 13 | -7.68e-03 | -5.37 |
| 521 | 36 | 1120.5766 | 561.2956 | 16161.60 | 2 | 1120.5763 | Z\_DOT9 | 19 | 3.12e-04 | 0.28 |
| 521 | 37 | 1301.8477 | 651.9311 | 11075.78 | 2 | 1301.8546 | C12 | 12 | -6.95e-03 | -5.34 |
| 521 | 38 | 902.6019 | 903.6092 | 8623.83 | 1 | 902.6065 | C8 | 8 | -4.56e-03 | -5.06 |
| 521 | 39 | 829.4200 | 830.4273 | 11076.44 | 1 | 829.4180 | Z\_DOT7 | 21 | 2.06e-03 | 2.48 |
| 521 | 40 | 1248.6343 | 625.3244 | 11106.03 | 2 | 1248.6348 | Z\_DOT11 | 17 | -5.19e-04 | -0.42 |
| 521 | 41 | 476.3090 | 477.3163 | 14005.12 | 1 | 476.3110 | C4 | 4 | -2.03e-03 | -4.27 |
| 521 | 42 | 992.4824 | 993.4897 | 7667.40 | 1 | 992.4813 | Z\_DOT8 | 20 | 1.11e-03 | 1.12 |
| 521 | 43 | 703.4709 | 704.4781 | 6489.56 | 1 | 703.4744 | C6 | 6 | -3.54e-03 | -5.03 |
| 521 | 44 | 774.5077 | 388.2611 | 5377.65 | 2 | 774.5115 | C7 | 7 | -3.86e-03 | -4.98 |
| 521 | 45 | 774.5078 | 775.5151 | 4399.63 | 1 | 774.5115 | C7 | 7 | -3.72e-03 | -4.80 |
| 521 | 46 | 1301.8480 | 1302.8553 | 3063.81 | 1 | 1301.8546 | C12 | 12 | -6.63e-03 | -5.09 |
| 521 | 47 | 1284.8205 | 643.4175 | 2916.12 | 2 |  |  |  |  |  |
| 521 | 48 | 730.3521 | 731.3594 | 6029.15 | 1 | 730.3496 | Z\_DOT6 | 22 | 2.54e-03 | 3.47 |
| 521 | 49 | 1377.7005 | 1378.7078 | 2404.50 | 1 |  |  |  |  |  |
| 521 | 50 | 1207.6314 | 604.8230 | 3633.85 | 2 |  |  |  |  |  |
| 521 | 51 | 232.1569 | 233.1642 | 2972.78 | 1 |  |  |  |  |  |

  

All proteins /
sp|B3EWU1|TXS1A\_CUPSA Short cationic peptide-1a OS=Cupiennius salei OX=6928 PE=1 SV=1 /
Proteoform #23
