# Supplementary material for: The Dual Prey-Inactivation Strategy of Spiders—In-Depth Venomic Analysis of Cupiennius salei
Source: Toxins (Basel). 2019 Mar 19;11(3):167. doi: 10.3390/toxins11030167 (PMC6468893; doi:10.3390/toxins11030167)
Supplement: Supplementary file 1 [file toxins-11-00167-s001.zip › Supplementary Dataset EV1/20180328_f2_topdown_OTMS2_EThcD_NL_i02_ms2_proteoform_cutoff_html/prsms/prsm152.html]

Protein-Spectrum-Match for Spectrum #390


All proteins /
CsTx-33a Cupiennius salei toxin 33 isoform a /
Proteoform #20

## Protein-Spectrum-Match #152 for Spectrum #390

|  |  |  |  |  |  |
| --- | --- | --- | --- | --- | --- |
| PrSM ID: | 152 | Scan(s): | 523 | Precursor charge: | 13 |
| Precursor m/z: | 620.8474 | Precursor mass: | 8057.9215 | Proteoform mass: | 8057.9165 |
| # matched peaks: | 47 | # matched fragment ions: | 34 | # unexpected modifications: | 1 |
| E-value: | 1.21e-28 | P-value: | 1.21e-28 | Q-value (Spectral FDR): | 0 |

  

|  |  |  |  |  |  |  |  |  |  |  |  |  |  |  |  |  |  |  |  |  |  |  |  |  |  |  |  |  |  |  |  |  |  |  |  |  |  |  |  |  |  |  |  |  |  |  |  |  |  |  |  |  |  |  |  |  |  |  |  |  |  |  |  |  |  |  |  |  |  |
| --- | --- | --- | --- | --- | --- | --- | --- | --- | --- | --- | --- | --- | --- | --- | --- | --- | --- | --- | --- | --- | --- | --- | --- | --- | --- | --- | --- | --- | --- | --- | --- | --- | --- | --- | --- | --- | --- | --- | --- | --- | --- | --- | --- | --- | --- | --- | --- | --- | --- | --- | --- | --- | --- | --- | --- | --- | --- | --- | --- | --- | --- | --- | --- | --- | --- | --- | --- | --- | --- |
|  | |  | | | | | | | | | | | | | | | | | | | | | | | | | | | | | | | | | | | | | | | | | | | | | | | | | | | | | | | | | | | | | | | | | | | |
| 1 |  |  | M |  | K |  | I |  | L |  | V |  | I |  | C |  | A |  | V |  | L |  |  | L |  | T |  | T |  | I |  | C |  | S |  | K |  | S |  | S |  | A |  |  | E |  | I |  | D |  | E |  | D |  | F |  | L |  | K |  | D |  | E |  | 30 |  |
|  | |  | | | | | | | | | | | | | | | | | | | | | | | | | | | | | | | | | | | | | | | | | | | | | | | | | | | | | | | | | | | | | | | | | | | |
| 31 |  |  | S |  | F |  | E |  | A |  | D |  | G |  | I |  | V |  | P |  | F |  |  | F |  | A |  | N |  | E |  | E |  | F |  | R | ] | K |  | D |  | K |  | ⎫ | R |  | N | ⎫ | C |  | I |  | P | ⎫ | R | ⎫ | N | ⎫ | Q | ⎫ | E | ⎫ | C |  | 60 |  |
|  | |  | | | | | | | | | | | | | | | | | | | | | | | | | | | | | | | | | | | | | | 14.99 | | | | | | | | | | | | | | | | | | | | | | | | | | |
| 61 |  | ⎫ | T | ⎫ | I | ⎫ | D |  | K | ⎫ | R | ⎫ | N | ⎫ | C | ⎫ | C | ⎫ | R | ⎫ | R |  | ⎫ | G | ⎫ | L |  | F | ⎱ | K | ⎱ | M |  | T | ⎫ | C |  | Q | ⎫ | C |  | M |  | ⎩ | K | ⎩ | S | ⎩ | N | ⎩ | D | ⎩ | E |  | S |  | G | ⎱ | Q |  | P |  | T |  | 90 |  |
|  | |  | | | | | | | | | | | | | | | | | | | | | | | | | | | | | | | | | | | | | | | | | | | | | | | | | | | | | | | | | | | | | | | | | | | |
| 91 |  |  | E |  | K |  | C |  | T |  | C | ⎩ | R | ⎩ | R |  | P |  | R |  | P |  |  | I |  | F |  | H |  | L |  | L |  | Y |  | K |  | G |  | L |  | L |  | ⎫ | K | [ | G |  | | 112 |  | | | | | | | | | | | | | | | |

Fixed PTMs: Carbamidomethylation [C53 C60 C67 C68 C77 C79 C93 C95 ]   
  
     Unexpected modifications:   Unknown [14.99]

  

All peaks (134)  Matched peaks (47)  Not matched peaks (87)

  

| Scan | Peak | Mono mass | Mono m/z | Intensity | Charge | Theoretical mass | Ion | Pos | Mass error | PPM error |
| --- | --- | --- | --- | --- | --- | --- | --- | --- | --- | --- |
| 523 | 1 | 2478.3465 | 620.5939 | 48786.72 | 4 |  |  |  |  |  |
| 523 | 2 | 3093.7576 | 619.7588 | 30430.56 | 5 |  |  |  |  |  |
| 523 | 3 | 1990.2350 | 664.4189 | 15693.25 | 3 | 1990.2231 | Z\_DOT16 | 48 | 0.0119 | 5.98 |
| 523 | 4 | 8000.8758 | 801.0949 | 11208.27 | 10 |  |  |  |  |  |
| 523 | 5 | 2993.6280 | 749.4143 | 14186.08 | 4 |  |  |  |  |  |
| 523 | 6 | 1298.6921 | 650.3533 | 11079.18 | 2 | 1298.6989 | C10 | 10 | -6.86e-03 | -5.28 |
| 523 | 7 | 3075.4750 | 616.1023 | 11699.83 | 5 | 3075.4919 | C23 | 23 | -0.0170 | -5.53 |
| 523 | 8 | 2329.1720 | 777.3979 | 11979.97 | 3 | 2329.1855 | C18 | 18 | -0.0135 | -5.79 |
| 523 | 9 | 2320.3335 | 581.0907 | 8298.36 | 4 |  |  |  |  |  |
| 523 | 10 | 7985.8486 | 888.3238 | 8660.64 | 9 |  |  |  |  |  |
| 523 | 11 | 7984.8566 | 799.4929 | 7625.43 | 10 |  |  |  |  |  |
| 523 | 12 | 2603.2428 | 868.7549 | 7475.85 | 3 | 2603.2591 | C20 | 20 | -0.0163 | -6.25 |
| 523 | 13 | 7943.8325 | 883.6553 | 7878.32 | 9 |  |  |  |  |  |
| 523 | 14 | 7928.8366 | 881.9891 | 8565.44 | 9 | 7928.8374 | C63 | 63 | -8.52e-04 | -0.11 |
| 523 | 15 | 3392.6453 | 679.5363 | 7152.61 | 5 | 3392.6659 | C26 | 26 | -0.0206 | -6.07 |
| 523 | 16 | 1929.9512 | 644.3243 | 7980.27 | 3 | 1929.9625 | C15 | 15 | -0.0113 | -5.85 |
| 523 | 17 | 2027.9514 | 676.9911 | 7337.85 | 3 |  |  |  |  |  |
| 523 | 18 | 2603.2432 | 651.8181 | 8225.38 | 4 | 2603.2591 | C20 | 20 | -0.0159 | -6.09 |
| 523 | 19 | 2443.2159 | 815.4126 | 7003.00 | 3 | 2443.2284 | C19 | 19 | -0.0125 | -5.11 |
| 523 | 20 | 8001.8682 | 890.1037 | 7183.80 | 9 |  |  |  |  |  |
| 523 | 21 | 7943.8291 | 795.3902 | 9845.69 | 10 |  |  |  |  |  |
| 523 | 22 | 1579.9754 | 790.9950 | 10544.06 | 2 |  |  |  |  |  |
| 523 | 23 | 1833.1292 | 612.0503 | 9847.83 | 3 |  |  |  |  |  |
| 523 | 24 | 3382.7597 | 846.6972 | 7683.27 | 4 | 3382.7517 | Z\_DOT28 | 36 | 7.99e-03 | 2.36 |
| 523 | 25 | 2763.2730 | 922.0983 | 6546.63 | 3 | 2763.2897 | C21 | 21 | -0.0168 | -6.07 |
| 523 | 26 | 1184.6497 | 593.3321 | 9489.08 | 2 | 1184.6560 | C9 | 9 | -6.29e-03 | -5.31 |
| 523 | 27 | 2763.2732 | 691.8256 | 6694.37 | 4 | 2763.2897 | C21 | 21 | -0.0165 | -5.96 |
| 523 | 28 | 1834.1332 | 918.0739 | 6459.60 | 2 | 1834.1220 | Z\_DOT15 | 49 | 0.0113 | 6.15 |
| 523 | 29 | 1715.8211 | 572.9476 | 10903.88 | 3 | 1715.8307 | C13 | 13 | -9.67e-03 | -5.64 |
| 523 | 30 | 658.3842 | 659.3915 | 12106.87 | 1 | 658.3874 | C5 | 5 | -3.20e-03 | -4.86 |
| 523 | 31 | 3496.7975 | 875.2066 | 5647.86 | 4 | 3496.7946 | Z\_DOT29 | 35 | 2.90e-03 | 0.83 |
| 523 | 32 | 2059.2574 | 515.8216 | 6276.21 | 4 |  |  |  |  |  |
| 523 | 33 | 2173.0729 | 725.3649 | 6301.13 | 3 | 2173.0844 | C17 | 17 | -0.0114 | -5.27 |
| 523 | 34 | 4216.0703 | 703.6857 | 4615.95 | 6 |  |  |  |  |  |
| 523 | 35 | 3132.4937 | 627.5060 | 4715.88 | 5 | 3132.5134 | C24 | 24 | -0.0197 | -6.30 |
| 523 | 36 | 8042.8703 | 732.1773 | 3757.45 | 11 |  |  |  |  |  |
| 523 | 37 | 4217.0693 | 844.4211 | 4451.59 | 5 |  |  |  |  |  |
| 523 | 38 | 2059.2573 | 687.4264 | 5298.10 | 3 |  |  |  |  |  |
| 523 | 39 | 3267.7295 | 817.9397 | 4643.91 | 4 | 3267.7247 | Z\_DOT27 | 37 | 4.82e-03 | 1.47 |
| 523 | 40 | 5065.2633 | 845.2178 | 3214.41 | 6 | 5064.2957 | C40 | 40 | -0.0348 | -6.86 |
| 523 | 41 | 8000.8677 | 728.3589 | 8310.40 | 11 |  |  |  |  |  |
| 523 | 42 | 3520.7406 | 705.1554 | 6364.55 | 5 | 3520.7608 | C27 | 27 | -0.0202 | -5.74 |
| 523 | 43 | 3752.8271 | 751.5727 | 7345.08 | 5 | 3752.8490 | C29 | 29 | -0.0219 | -5.84 |
| 523 | 44 | 6012.6486 | 1003.1154 | 2947.50 | 6 |  |  |  |  |  |
| 523 | 45 | 7943.8530 | 993.9889 | 6737.53 | 8 |  |  |  |  |  |
| 523 | 46 | 2919.3772 | 730.8516 | 5263.90 | 4 | 2919.3908 | C22 | 22 | -0.0136 | -4.65 |
| 523 | 47 | 4040.9146 | 674.4930 | 4271.79 | 6 | 4040.9382 | C31 | 31 | -0.0236 | -5.85 |
| 523 | 48 | 2420.3204 | 807.7807 | 6371.26 | 3 |  |  |  |  |  |
| 523 | 49 | 3911.9842 | 783.4041 | 4227.43 | 5 |  |  |  |  |  |
| 523 | 50 | 3598.8369 | 720.7747 | 3162.17 | 5 |  |  |  |  |  |
| 523 | 51 | 886.5597 | 444.2871 | 4706.84 | 2 |  |  |  |  |  |
| 523 | 52 | 3928.9803 | 786.8033 | 3380.15 | 5 |  |  |  |  |  |
| 523 | 53 | 7870.7969 | 875.5403 | 3721.69 | 9 |  |  |  |  |  |
| 523 | 54 | 7942.8163 | 723.0815 | 4143.46 | 11 |  |  |  |  |  |
| 523 | 55 | 1426.7493 | 714.3819 | 4354.39 | 2 | 1426.7575 | C11 | 11 | -8.19e-03 | -5.74 |
| 523 | 56 | 5065.2702 | 724.6173 | 4828.70 | 7 | 5064.2957 | C40 | 40 | -0.0278 | -5.49 |
| 523 | 57 | 312.2150 | 313.2223 | 5682.05 | 1 |  |  |  |  |  |
| 523 | 58 | 1298.6923 | 433.9047 | 8076.78 | 3 | 1298.6989 | C10 | 10 | -6.66e-03 | -5.13 |
| 523 | 59 | 929.6140 | 930.6213 | 4243.30 | 1 |  |  |  |  |  |
| 523 | 60 | 3018.4588 | 755.6220 | 3212.05 | 4 |  |  |  |  |  |
| 523 | 61 | 3752.8245 | 626.4780 | 5080.58 | 6 | 3752.8490 | C29 | 29 | -0.0245 | -6.52 |
| 523 | 62 | 7928.8427 | 992.1126 | 5139.47 | 8 | 7928.8374 | C63 | 63 | 5.23e-03 | 0.66 |
| 523 | 63 | 4984.4226 | 831.7444 | 2727.18 | 6 |  |  |  |  |  |
| 523 | 64 | 7970.8143 | 798.0887 | 3710.72 | 10 |  |  |  |  |  |
| 523 | 65 | 8042.8733 | 894.6599 | 2958.59 | 9 |  |  |  |  |  |
| 523 | 66 | 3092.7605 | 774.1974 | 6352.12 | 4 |  |  |  |  |  |
| 523 | 67 | 1715.8196 | 858.9171 | 3823.71 | 2 | 1715.8307 | C13 | 13 | -0.0111 | -6.48 |
| 523 | 68 | 4538.1525 | 908.6378 | 3366.01 | 5 | 4538.1635 | Z\_DOT37 | 27 | -0.0110 | -2.42 |
| 523 | 69 | 3075.4756 | 769.8762 | 4626.76 | 4 | 3075.4919 | C23 | 23 | -0.0163 | -5.30 |
| 523 | 70 | 1426.7498 | 476.5905 | 4851.78 | 3 | 1426.7575 | C11 | 11 | -7.69e-03 | -5.39 |
| 523 | 71 | 3711.9230 | 928.9880 | 3922.79 | 4 | 3711.9216 | Z\_DOT31 | 33 | 1.37e-03 | 0.37 |
| 523 | 72 | 816.5307 | 817.5380 | 3384.23 | 1 |  |  |  |  |  |
| 523 | 73 | 4666.2531 | 934.2579 | 2876.78 | 5 | 4666.2584 | Z\_DOT38 | 26 | -5.36e-03 | -1.15 |
| 523 | 74 | 3583.8294 | 896.9646 | 3480.97 | 4 | 3583.8266 | Z\_DOT30 | 34 | 2.75e-03 | 0.77 |
| 523 | 75 | 388.2418 | 389.2491 | 6431.35 | 1 | 388.2434 | C3 | 3 | -1.58e-03 | -4.08 |
| 523 | 76 | 2903.6274 | 581.7328 | 2423.02 | 5 |  |  |  |  |  |
| 523 | 77 | 7900.8017 | 988.6075 | 2980.70 | 8 |  |  |  |  |  |
| 523 | 78 | 1815.8664 | 606.2961 | 4808.34 | 3 |  |  |  |  |  |
| 523 | 79 | 7632.6079 | 955.0833 | 2360.72 | 8 |  |  |  |  |  |
| 523 | 80 | 2538.4313 | 635.6151 | 3020.62 | 4 |  |  |  |  |  |
| 523 | 81 | 2443.2156 | 611.8112 | 5359.50 | 4 | 2443.2284 | C19 | 19 | -0.0128 | -5.24 |
| 523 | 82 | 4041.9125 | 809.3898 | 4919.22 | 5 |  |  |  |  |  |
| 523 | 83 | 7900.8010 | 878.8741 | 2967.13 | 9 |  |  |  |  |  |
| 523 | 84 | 8043.8964 | 805.3969 | 5895.68 | 10 |  |  |  |  |  |
| 523 | 85 | 2994.6330 | 999.2183 | 4964.65 | 3 | 2994.6286 | Z\_DOT24 | 40 | 4.33e-03 | 1.45 |
| 523 | 86 | 7855.7567 | 873.8691 | 5171.15 | 9 |  |  |  |  |  |
| 523 | 87 | 1184.6501 | 395.8907 | 3732.24 | 3 | 1184.6560 | C9 | 9 | -5.85e-03 | -4.93 |
| 523 | 88 | 2478.3486 | 827.1235 | 4283.39 | 3 |  |  |  |  |  |
| 523 | 89 | 1213.7392 | 607.8769 | 2291.75 | 2 |  |  |  |  |  |
| 523 | 90 | 1555.7916 | 519.6045 | 3520.80 | 3 | 1555.8001 | C12 | 12 | -8.44e-03 | -5.42 |
| 523 | 91 | 7926.8187 | 793.6891 | 3143.76 | 10 |  |  |  |  |  |
| 523 | 92 | 1816.8684 | 909.4415 | 4589.44 | 2 | 1816.8784 | C14 | 14 | -0.0101 | -5.53 |
| 523 | 93 | 540.3848 | 541.3920 | 4498.63 | 1 |  |  |  |  |  |
| 523 | 94 | 7647.6198 | 850.7428 | 2924.93 | 9 |  |  |  |  |  |
| 523 | 95 | 7592.5684 | 950.0783 | 2493.62 | 8 |  |  |  |  |  |
| 523 | 96 | 3137.6790 | 785.4270 | 2752.59 | 4 |  |  |  |  |  |
| 523 | 97 | 7957.8447 | 885.2122 | 3070.05 | 9 |  |  |  |  |  |
| 523 | 98 | 2043.9748 | 682.3322 | 3442.91 | 3 |  |  |  |  |  |
| 523 | 99 | 7884.7904 | 877.0951 | 5092.49 | 9 |  |  |  |  |  |
| 523 | 100 | 1929.9509 | 965.9827 | 2939.04 | 2 | 1929.9625 | C15 | 15 | -0.0116 | -6.00 |
| 523 | 101 | 3048.7410 | 763.1925 | 4258.66 | 4 |  |  |  |  |  |
| 523 | 102 | 7957.8507 | 995.7386 | 3236.46 | 8 |  |  |  |  |  |
| 523 | 103 | 1555.7912 | 778.9029 | 3830.72 | 2 | 1555.8001 | C12 | 12 | -8.91e-03 | -5.73 |
| 523 | 104 | 7969.8395 | 997.2372 | 3140.50 | 8 |  |  |  |  |  |
| 523 | 105 | 2918.3740 | 584.6821 | 2357.47 | 5 |  |  |  |  |  |
| 523 | 106 | 2546.2234 | 849.7484 | 2020.97 | 3 |  |  |  |  |  |
| 523 | 107 | 7873.7851 | 985.2304 | 3833.05 | 8 |  |  |  |  |  |
| 523 | 108 | 1213.7410 | 1214.7482 | 2937.73 | 1 |  |  |  |  |  |
| 523 | 109 | 5367.6542 | 895.6163 | 1958.44 | 6 |  |  |  |  |  |
| 523 | 110 | 3335.6111 | 834.9101 | 2721.49 | 4 |  |  |  |  |  |
| 523 | 111 | 1028.5491 | 515.2818 | 2210.55 | 2 | 1028.5549 | C8 | 8 | -5.77e-03 | -5.61 |
| 523 | 112 | 6704.2003 | 958.7502 | 2931.53 | 7 |  |  |  |  |  |
| 523 | 113 | 2935.5999 | 734.9073 | 2498.57 | 4 |  |  |  |  |  |
| 523 | 114 | 4506.0890 | 752.0221 | 1751.91 | 6 |  |  |  |  |  |
| 523 | 115 | 1760.8712 | 587.9644 | 1767.40 | 3 |  |  |  |  |  |
| 523 | 116 | 8043.8888 | 671.3313 | 2713.31 | 12 |  |  |  |  |  |
| 523 | 117 | 2293.2822 | 765.4347 | 1500.83 | 3 |  |  |  |  |  |
| 523 | 118 | 7885.8451 | 986.7379 | 3471.39 | 8 |  |  |  |  |  |
| 523 | 119 | 4909.3904 | 702.3488 | 2794.54 | 7 |  |  |  |  |  |
| 523 | 120 | 2934.6125 | 587.9298 | 3977.61 | 5 |  |  |  |  |  |
| 523 | 121 | 703.4468 | 704.4540 | 2590.31 | 1 |  |  |  |  |  |
| 523 | 122 | 6013.6380 | 860.0984 | 3351.67 | 7 |  |  |  |  |  |
| 523 | 123 | 2903.6235 | 726.9132 | 2468.51 | 4 |  |  |  |  |  |
| 523 | 124 | 5298.6333 | 757.9549 | 2072.09 | 7 |  |  |  |  |  |
| 523 | 125 | 5050.2625 | 842.7177 | 2600.20 | 6 |  |  |  |  |  |
| 523 | 126 | 2622.5962 | 875.2060 | 2334.46 | 3 |  |  |  |  |  |
| 523 | 127 | 1164.5863 | 583.3004 | 1463.30 | 2 |  |  |  |  |  |
| 523 | 128 | 773.4757 | 387.7451 | 1644.92 | 2 |  |  |  |  |  |
| 523 | 129 | 1167.6229 | 390.2149 | 1535.21 | 3 |  |  |  |  |  |
| 523 | 130 | 1000.3603 | 1001.3676 | 1304.41 | 1 |  |  |  |  |  |
| 523 | 131 | 841.4042 | 842.4115 | 1168.43 | 1 |  |  |  |  |  |
| 523 | 132 | 721.8967 | 722.9039 | 1176.83 | 1 |  |  |  |  |  |
| 523 | 133 | 853.8429 | 854.8502 | 1174.43 | 1 |  |  |  |  |  |
| 523 | 134 | 1281.6654 | 428.2291 | 777.18 | 3 |  |  |  |  |  |

  

All proteins /
CsTx-33a Cupiennius salei toxin 33 isoform a /
Proteoform #20
