# Supplementary material for: The Dual Prey-Inactivation Strategy of Spiders—In-Depth Venomic Analysis of Cupiennius salei
Source: Toxins (Basel). 2019 Mar 19;11(3):167. doi: 10.3390/toxins11030167 (PMC6468893; doi:10.3390/toxins11030167)
Supplement: Supplementary file 1 [file toxins-11-00167-s001.zip › Supplementary Dataset EV1/20180328_f2_topdown_OTMS2_EThcD_NL_i02_ms2_proteoform_cutoff_html/prsms/prsm153.html]

Protein-Spectrum-Match for Spectrum #391


All proteins /
CsTx-33a Cupiennius salei toxin 33 isoform a /
Proteoform #20

## Protein-Spectrum-Match #153 for Spectrum #391

|  |  |  |  |  |  |
| --- | --- | --- | --- | --- | --- |
| PrSM ID: | 153 | Scan(s): | 524 | Precursor charge: | 12 |
| Precursor m/z: | 672.5841 | Precursor mass: | 8058.9221 | Proteoform mass: | 8058.9121 |
| # matched peaks: | 38 | # matched fragment ions: | 31 | # unexpected modifications: | 1 |
| E-value: | 1.72e-25 | P-value: | 1.72e-25 | Q-value (Spectral FDR): | 0 |

  

|  |  |  |  |  |  |  |  |  |  |  |  |  |  |  |  |  |  |  |  |  |  |  |  |  |  |  |  |  |  |  |  |  |  |  |  |  |  |  |  |  |  |  |  |  |  |  |  |  |  |  |  |  |  |  |  |  |  |  |  |  |  |  |  |  |  |  |  |  |  |
| --- | --- | --- | --- | --- | --- | --- | --- | --- | --- | --- | --- | --- | --- | --- | --- | --- | --- | --- | --- | --- | --- | --- | --- | --- | --- | --- | --- | --- | --- | --- | --- | --- | --- | --- | --- | --- | --- | --- | --- | --- | --- | --- | --- | --- | --- | --- | --- | --- | --- | --- | --- | --- | --- | --- | --- | --- | --- | --- | --- | --- | --- | --- | --- | --- | --- | --- | --- | --- | --- |
|  | |  | | | | | | | | | | | | | | | | | | | | | | | | | | | | | | | | | | | | | | | | | | | | | | | | | | | | | | | | | | | | | | | | | | | |
| 1 |  |  | M |  | K |  | I |  | L |  | V |  | I |  | C |  | A |  | V |  | L |  |  | L |  | T |  | T |  | I |  | C |  | S |  | K |  | S |  | S |  | A |  |  | E |  | I |  | D |  | E |  | D |  | F |  | L |  | K |  | D |  | E |  | 30 |  |
|  | |  | | | | | | | | | | | | | | | | | | | | | | | | | | | | | | | | | | | | | | | | | | | | | | | | | | | | | | | | | | | | | | | | | | | |
| 31 |  |  | S |  | F |  | E |  | A |  | D |  | G |  | I |  | V |  | P |  | F |  |  | F |  | A |  | N |  | E |  | E |  | F |  | R | ] | K |  | D | ⎩ | K |  | ⎫ | R |  | N | ⎫ | C |  | I |  | P |  | R | ⎫ | N | ⎫ | Q | ⎫ | E |  | C |  | 60 |  |
|  | |  | | | | | | | | | | | | | | | | | | | | | | | | | | | | | | | | | | | | | | 15.99 | | | | | | | | | | | | | | | | | | | | | | | | | | |
| 61 |  | ⎫ | T | ⎫ | I | ⎫ | D |  | K | ⎫ | R | ⎫ | N | ⎫ | C | ⎫ | C | ⎫ | R | ⎫ | R |  | ⎫ | G |  | L |  | F | ⎱ | K | ⎱ | M | ⎫ | T | ⎫ | C |  | Q | ⎫ | C |  | M |  | ⎩ | K |  | S | ⎩ | N | ⎱ | D |  | E |  | S |  | G |  | Q |  | P |  | T |  | 90 |  |
|  | |  | | | | | | | | | | | | | | | | | | | | | | | | | | | | | | | | | | | | | | | | | | | | | | | | | | | | | | | | | | | | | | | | | | | |
| 91 |  |  | E |  | K |  | C |  | T |  | C |  | R | ⎱ | R |  | P |  | R |  | P |  |  | I |  | F |  | H |  | L |  | L |  | Y |  | K | ⎫ | G |  | L |  | L |  | ⎫ | K | [ | G |  | | 112 |  | | | | | | | | | | | | | | | |

Fixed PTMs: Carbamidomethylation [C53 C60 C67 C68 C77 C79 C93 C95 ]   
  
     Unexpected modifications:   Unknown [15.99]

  

All peaks (134)  Matched peaks (38)  Not matched peaks (96)

  

| Scan | Peak | Mono mass | Mono m/z | Intensity | Charge | Theoretical mass | Ion | Pos | Mass error | PPM error |
| --- | --- | --- | --- | --- | --- | --- | --- | --- | --- | --- |
| 524 | 1 | 2329.1711 | 777.3976 | 19946.00 | 3 | 2329.1855 | C18 | 18 | -0.0144 | -6.19 |
| 524 | 2 | 7984.8572 | 888.2136 | 17670.25 | 9 |  |  |  |  |  |
| 524 | 3 | 7943.8394 | 993.9872 | 12282.14 | 8 |  |  |  |  |  |
| 524 | 4 | 3752.8256 | 751.5724 | 17151.48 | 5 | 3752.8490 | C29 | 29 | -0.0234 | -6.24 |
| 524 | 5 | 7985.8668 | 799.5940 | 14955.01 | 10 |  |  |  |  |  |
| 524 | 6 | 3392.6434 | 679.5360 | 16062.13 | 5 | 3392.6659 | C26 | 26 | -0.0224 | -6.61 |
| 524 | 7 | 2603.2433 | 868.7551 | 15468.08 | 3 | 2603.2591 | C20 | 20 | -0.0157 | -6.04 |
| 524 | 8 | 8000.8521 | 889.9908 | 14846.13 | 9 |  |  |  |  |  |
| 524 | 9 | 7942.8244 | 883.5433 | 12381.27 | 9 |  |  |  |  |  |
| 524 | 10 | 1298.6914 | 650.3530 | 17997.14 | 2 | 1298.6989 | C10 | 10 | -7.47e-03 | -5.75 |
| 524 | 11 | 8000.8603 | 728.3582 | 10096.71 | 11 |  |  |  |  |  |
| 524 | 12 | 8001.8623 | 1001.2401 | 9793.53 | 8 |  |  |  |  |  |
| 524 | 13 | 7927.8396 | 881.8783 | 12767.29 | 9 |  |  |  |  |  |
| 524 | 14 | 7999.8572 | 800.9930 | 16492.08 | 10 |  |  |  |  |  |
| 524 | 15 | 3520.7376 | 705.1548 | 11440.23 | 5 | 3520.7608 | C27 | 27 | -0.0232 | -6.60 |
| 524 | 16 | 3355.8243 | 672.1721 | 17917.04 | 5 |  |  |  |  |  |
| 524 | 17 | 1989.2323 | 664.0847 | 14673.75 | 3 |  |  |  |  |  |
| 524 | 18 | 2443.2141 | 815.4120 | 11353.29 | 3 | 2443.2284 | C19 | 19 | -0.0143 | -5.85 |
| 524 | 19 | 7927.8447 | 991.9879 | 13224.47 | 8 |  |  |  |  |  |
| 524 | 20 | 2763.2741 | 922.0986 | 11990.38 | 3 | 2763.2897 | C21 | 21 | -0.0157 | -5.67 |
| 524 | 21 | 8042.8913 | 805.2964 | 10816.44 | 10 |  |  |  |  |  |
| 524 | 22 | 1579.9753 | 790.9949 | 16210.23 | 2 |  |  |  |  |  |
| 524 | 23 | 2993.6263 | 749.4138 | 11762.47 | 4 |  |  |  |  |  |
| 524 | 24 | 4040.9124 | 809.1898 | 10880.11 | 5 | 4040.9382 | C31 | 31 | -0.0258 | -6.39 |
| 524 | 25 | 7985.8595 | 999.2397 | 12033.66 | 8 |  |  |  |  |  |
| 524 | 26 | 7970.8512 | 886.6574 | 8436.81 | 9 |  |  |  |  |  |
| 524 | 27 | 8042.8834 | 894.6610 | 8125.25 | 9 |  |  |  |  |  |
| 524 | 28 | 2027.9502 | 676.9907 | 8088.93 | 3 |  |  |  |  |  |
| 524 | 29 | 1833.1287 | 612.0502 | 8937.75 | 3 |  |  |  |  |  |
| 524 | 30 | 4200.0720 | 701.0193 | 6573.78 | 6 |  |  |  |  |  |
| 524 | 31 | 1184.6496 | 593.3321 | 12435.91 | 2 | 1184.6560 | C9 | 9 | -6.41e-03 | -5.41 |
| 524 | 32 | 3382.7572 | 846.6966 | 7564.66 | 4 | 3382.7517 | Z\_DOT28 | 36 | 5.55e-03 | 1.64 |
| 524 | 33 | 7985.8770 | 726.9961 | 9588.66 | 11 |  |  |  |  |  |
| 524 | 34 | 658.3841 | 659.3914 | 13181.50 | 1 | 658.3874 | C5 | 5 | -3.32e-03 | -5.04 |
| 524 | 35 | 8043.8888 | 732.2699 | 7070.13 | 11 |  |  |  |  |  |
| 524 | 36 | 1816.8682 | 909.4414 | 9020.44 | 2 | 1816.8784 | C14 | 14 | -0.0102 | -5.60 |
| 524 | 37 | 1298.6921 | 433.9046 | 7988.01 | 3 | 1298.6989 | C10 | 10 | -6.84e-03 | -5.27 |
| 524 | 38 | 2059.2568 | 687.4262 | 6493.87 | 3 |  |  |  |  |  |
| 524 | 39 | 7646.5997 | 850.6295 | 5136.63 | 9 | 7646.6435 | C60 | 60 | -0.0438 | -5.73 |
| 524 | 40 | 1715.8202 | 858.9174 | 5994.81 | 2 | 1715.8307 | C13 | 13 | -0.0105 | -6.13 |
| 524 | 41 | 8043.8809 | 1006.4924 | 5661.96 | 8 |  |  |  |  |  |
| 524 | 42 | 4216.0641 | 703.6846 | 5575.29 | 6 |  |  |  |  |  |
| 524 | 43 | 7943.8140 | 795.3887 | 6634.24 | 10 |  |  |  |  |  |
| 524 | 44 | 1426.7489 | 714.3817 | 7563.37 | 2 | 1426.7575 | C11 | 11 | -8.56e-03 | -6.00 |
| 524 | 45 | 4200.0683 | 841.0209 | 5538.31 | 5 |  |  |  |  |  |
| 524 | 46 | 7928.8376 | 1133.6984 | 5228.17 | 7 | 7929.8331 | C63 | 63 | 6.88e-03 | 0.87 |
| 524 | 47 | 1834.1347 | 918.0746 | 9936.20 | 2 | 1834.1220 | Z\_DOT15 | 49 | 0.0127 | 6.95 |
| 524 | 48 | 6747.2022 | 964.8933 | 4520.68 | 7 |  |  |  |  |  |
| 524 | 49 | 3911.9842 | 783.4041 | 8476.71 | 5 |  |  |  |  |  |
| 524 | 50 | 3927.9827 | 786.6038 | 6117.08 | 5 |  |  |  |  |  |
| 524 | 51 | 7957.8343 | 885.2111 | 5634.32 | 9 |  |  |  |  |  |
| 524 | 52 | 7884.7963 | 877.0957 | 5030.47 | 9 |  |  |  |  |  |
| 524 | 53 | 5365.6481 | 767.5284 | 3614.54 | 7 |  |  |  |  |  |
| 524 | 54 | 2013.2547 | 672.0922 | 6477.42 | 3 |  |  |  |  |  |
| 524 | 55 | 4667.2519 | 934.4577 | 5258.03 | 5 | 4667.2541 | Z\_DOT38 | 26 | -2.15e-03 | -0.46 |
| 524 | 56 | 7886.8437 | 986.8627 | 5459.07 | 8 |  |  |  |  |  |
| 524 | 57 | 7958.8438 | 995.8628 | 4450.02 | 8 |  |  |  |  |  |
| 524 | 58 | 7885.7977 | 1127.5498 | 5514.87 | 7 |  |  |  |  |  |
| 524 | 59 | 4677.1384 | 780.5303 | 5842.66 | 6 | 4677.1683 | C36 | 36 | -0.0298 | -6.38 |
| 524 | 60 | 4651.2525 | 931.2578 | 6126.59 | 5 |  |  |  |  |  |
| 524 | 61 | 886.5594 | 444.2870 | 5416.59 | 2 |  |  |  |  |  |
| 524 | 62 | 1554.7865 | 778.4005 | 5175.94 | 2 |  |  |  |  |  |
| 524 | 63 | 3075.4747 | 769.8759 | 5929.52 | 4 | 3075.4919 | C23 | 23 | -0.0173 | -5.62 |
| 524 | 64 | 7943.8237 | 1135.8392 | 4975.30 | 7 |  |  |  |  |  |
| 524 | 65 | 7871.7836 | 875.6499 | 4164.72 | 9 |  |  |  |  |  |
| 524 | 66 | 7572.5807 | 947.5799 | 4813.31 | 8 |  |  |  |  |  |
| 524 | 67 | 3496.7965 | 875.2064 | 5110.48 | 4 | 3496.7946 | Z\_DOT29 | 35 | 1.92e-03 | 0.55 |
| 524 | 68 | 2173.0728 | 725.3649 | 6832.11 | 3 | 2173.0844 | C17 | 17 | -0.0116 | -5.35 |
| 524 | 69 | 7899.8085 | 878.7638 | 5321.30 | 9 |  |  |  |  |  |
| 524 | 70 | 7873.7753 | 985.2292 | 5041.99 | 8 |  |  |  |  |  |
| 524 | 71 | 929.6135 | 930.6208 | 5447.35 | 1 |  |  |  |  |  |
| 524 | 72 | 2320.3335 | 581.0907 | 4911.69 | 4 |  |  |  |  |  |
| 524 | 73 | 6703.1800 | 958.6044 | 5182.65 | 7 |  |  |  |  |  |
| 524 | 74 | 312.2150 | 313.2223 | 6592.56 | 1 |  |  |  |  |  |
| 524 | 75 | 7872.7992 | 1125.6929 | 3361.79 | 7 |  |  |  |  |  |
| 524 | 76 | 2059.2559 | 515.8213 | 3008.55 | 4 |  |  |  |  |  |
| 524 | 77 | 8041.9009 | 671.1657 | 4475.92 | 12 |  |  |  |  |  |
| 524 | 78 | 1815.8656 | 606.2958 | 4593.54 | 3 |  |  |  |  |  |
| 524 | 79 | 3794.7971 | 949.7065 | 2880.62 | 4 |  |  |  |  |  |
| 524 | 80 | 6224.7667 | 779.1031 | 3352.10 | 8 | 6225.7980 | C49 | 49 | -0.0289 | -4.65 |
| 524 | 81 | 7912.8278 | 1131.4112 | 5860.77 | 7 |  |  |  |  |  |
| 524 | 82 | 1747.0587 | 874.5367 | 4266.18 | 2 |  |  |  |  |  |
| 524 | 83 | 4216.0644 | 844.2201 | 5586.20 | 5 |  |  |  |  |  |
| 524 | 84 | 1929.9493 | 965.9819 | 5100.88 | 2 | 1929.9625 | C15 | 15 | -0.0132 | -6.82 |
| 524 | 85 | 7985.8604 | 1141.8445 | 4564.22 | 7 |  |  |  |  |  |
| 524 | 86 | 2919.3768 | 730.8515 | 3662.13 | 4 | 2919.3908 | C22 | 22 | -0.0141 | -4.82 |
| 524 | 87 | 3911.9805 | 653.0040 | 4281.59 | 6 |  |  |  |  |  |
| 524 | 88 | 7899.8036 | 988.4827 | 4536.18 | 8 |  |  |  |  |  |
| 524 | 89 | 2903.6202 | 726.9123 | 4268.97 | 4 |  |  |  |  |  |
| 524 | 90 | 2329.1711 | 1165.5928 | 3657.33 | 2 | 2329.1855 | C18 | 18 | -0.0144 | -6.18 |
| 524 | 91 | 388.2418 | 389.2491 | 6470.48 | 1 | 388.2434 | C3 | 3 | -1.58e-03 | -4.08 |
| 524 | 92 | 7928.8326 | 793.8905 | 5929.31 | 10 | 7929.8331 | C63 | 63 | 1.86e-03 | 0.24 |
| 524 | 93 | 3463.7112 | 866.9351 | 3371.48 | 4 |  |  |  |  |  |
| 524 | 94 | 6152.7359 | 1026.4633 | 2893.39 | 6 |  |  |  |  |  |
| 524 | 95 | 3711.9176 | 928.9867 | 3969.96 | 4 | 3711.9216 | Z\_DOT31 | 33 | -4.00e-03 | -1.08 |
| 524 | 96 | 6169.7537 | 882.4007 | 3543.36 | 7 |  |  |  |  |  |
| 524 | 97 | 2935.5999 | 734.9073 | 4519.38 | 4 |  |  |  |  |  |
| 524 | 98 | 7800.7213 | 976.0974 | 2264.33 | 8 | 7799.7715 | Z\_DOT62 | 2 | -0.0526 | -6.74 |
| 524 | 99 | 3985.9097 | 997.4847 | 3563.29 | 4 |  |  |  |  |  |
| 524 | 100 | 7630.6149 | 954.8341 | 4685.98 | 8 |  |  |  |  |  |
| 524 | 101 | 3912.9896 | 979.2547 | 3878.21 | 4 |  |  |  |  |  |
| 524 | 102 | 7597.6295 | 950.7110 | 2759.67 | 8 |  |  |  |  |  |
| 524 | 103 | 816.5305 | 817.5378 | 3865.17 | 1 |  |  |  |  |  |
| 524 | 104 | 703.4469 | 704.4542 | 4553.56 | 1 |  |  |  |  |  |
| 524 | 105 | 540.3847 | 541.3920 | 4408.83 | 1 |  |  |  |  |  |
| 524 | 106 | 3266.7242 | 817.6883 | 3538.55 | 4 |  |  |  |  |  |
| 524 | 107 | 3333.8619 | 667.7796 | 2518.96 | 5 |  |  |  |  |  |
| 524 | 108 | 3752.8287 | 939.2145 | 3429.04 | 4 | 3752.8490 | C29 | 29 | -0.0203 | -5.41 |
| 524 | 109 | 3598.8379 | 720.7748 | 3748.70 | 5 |  |  |  |  |  |
| 524 | 110 | 4040.9146 | 674.4930 | 2831.11 | 6 | 4040.9382 | C31 | 31 | -0.0236 | -5.85 |
| 524 | 111 | 6012.6614 | 1003.1175 | 2976.53 | 6 |  |  |  |  |  |
| 524 | 112 | 7912.8327 | 880.2109 | 5647.05 | 9 |  |  |  |  |  |
| 524 | 113 | 3334.6245 | 834.6634 | 3917.24 | 4 |  |  |  |  |  |
| 524 | 114 | 3651.7750 | 731.3623 | 2706.19 | 5 | 3651.8013 | C28 | 28 | -0.0263 | -7.20 |
| 524 | 115 | 7898.8159 | 1129.4095 | 2693.88 | 7 |  |  |  |  |  |
| 524 | 116 | 4539.1631 | 908.8399 | 3415.13 | 5 | 4539.1591 | Z\_DOT37 | 27 | 3.99e-03 | 0.88 |
| 524 | 117 | 7309.5102 | 813.1751 | 2801.95 | 9 |  |  |  |  |  |
| 524 | 118 | 4661.1515 | 777.8659 | 4764.76 | 6 |  |  |  |  |  |
| 524 | 119 | 1715.8200 | 572.9473 | 2344.76 | 3 | 1715.8307 | C13 | 13 | -0.0108 | -6.28 |
| 524 | 120 | 8026.8563 | 892.8802 | 5394.12 | 9 |  |  |  |  |  |
| 524 | 121 | 3317.8610 | 664.5795 | 3387.56 | 5 |  |  |  |  |  |
| 524 | 122 | 1213.7385 | 1214.7458 | 4141.86 | 1 |  |  |  |  |  |
| 524 | 123 | 1066.6722 | 1067.6795 | 2264.60 | 1 |  |  |  |  |  |
| 524 | 124 | 732.8999 | 733.9072 | 2375.71 | 1 |  |  |  |  |  |
| 524 | 125 | 1439.8931 | 720.9538 | 1613.07 | 2 |  |  |  |  |  |
| 524 | 126 | 369.2360 | 370.2433 | 1874.47 | 1 |  |  |  |  |  |
| 524 | 127 | 1426.7495 | 476.5905 | 1926.64 | 3 | 1426.7575 | C11 | 11 | -7.96e-03 | -5.58 |
| 524 | 128 | 867.0249 | 868.0322 | 1463.19 | 1 |  |  |  |  |  |
| 524 | 129 | 1213.7395 | 607.8770 | 2078.48 | 2 |  |  |  |  |  |
| 524 | 130 | 1029.1271 | 1030.1343 | 1373.45 | 1 |  |  |  |  |  |
| 524 | 131 | 883.6510 | 884.6583 | 1497.06 | 1 |  |  |  |  |  |
| 524 | 132 | 1326.8252 | 1327.8325 | 1105.02 | 1 |  |  |  |  |  |
| 524 | 133 | 860.5082 | 431.2614 | 860.17 | 2 |  |  |  |  |  |
| 524 | 134 | 795.8848 | 796.8920 | 1066.53 | 1 |  |  |  |  |  |

  

All proteins /
CsTx-33a Cupiennius salei toxin 33 isoform a /
Proteoform #20
