# Supplementary material for: The Dual Prey-Inactivation Strategy of Spiders—In-Depth Venomic Analysis of Cupiennius salei
Source: Toxins (Basel). 2019 Mar 19;11(3):167. doi: 10.3390/toxins11030167 (PMC6468893; doi:10.3390/toxins11030167)
Supplement: Supplementary file 1 [file toxins-11-00167-s001.zip › Supplementary Dataset EV1/20180328_f2_topdown_OTMS2_EThcD_NL_i02_ms2_proteoform_cutoff_html/prsms/prsm154.html]

Protein-Spectrum-Match for Spectrum #392


All proteins /
CsTx-33a Cupiennius salei toxin 33 isoform a /
Proteoform #79

## Protein-Spectrum-Match #154 for Spectrum #392

|  |  |  |  |  |  |
| --- | --- | --- | --- | --- | --- |
| PrSM ID: | 154 | Scan(s): | 525 | Precursor charge: | 13 |
| Precursor m/z: | 624.0809 | Precursor mass: | 8099.9572 | Proteoform mass: | 8099.9446 |
| # matched peaks: | 19 | # matched fragment ions: | 14 | # unexpected modifications: | 0 |
| E-value: | 4.47e-15 | P-value: | 4.47e-15 | Q-value (Spectral FDR): | 0 |

  

|  |  |  |  |  |  |  |  |  |  |  |  |  |  |  |  |  |  |  |  |  |  |  |  |  |  |  |  |  |  |  |  |  |  |  |  |  |  |  |  |  |  |  |  |  |  |  |  |  |  |  |  |  |  |  |  |  |  |  |  |  |  |  |  |  |  |  |  |  |  |
| --- | --- | --- | --- | --- | --- | --- | --- | --- | --- | --- | --- | --- | --- | --- | --- | --- | --- | --- | --- | --- | --- | --- | --- | --- | --- | --- | --- | --- | --- | --- | --- | --- | --- | --- | --- | --- | --- | --- | --- | --- | --- | --- | --- | --- | --- | --- | --- | --- | --- | --- | --- | --- | --- | --- | --- | --- | --- | --- | --- | --- | --- | --- | --- | --- | --- | --- | --- | --- | --- |
|  | |  | | | | | | | | | | | | | | | | | | | | | | | | | | | | | | | | | | | | | | | | | | | | | | | | | | | | | | | | | | | | | | | | | | | |
| 1 |  |  | M |  | K |  | I |  | L |  | V |  | I |  | C |  | A |  | V |  | L |  |  | L |  | T |  | T |  | I |  | C |  | S |  | K |  | S |  | S |  | A |  |  | E |  | I |  | D |  | E |  | D |  | F |  | L |  | K |  | D |  | E |  | 30 |  |
|  | |  | | | | | | | | | | | | | | | | | | | | | | | | | | | | | | | | | | | | | | | | | | | | | | | | | | | | | | | | | | | | | | | | | | | |
| 31 |  |  | S |  | F |  | E |  | A |  | D |  | G |  | I |  | V |  | P |  | F |  |  | F |  | A |  | N |  | E |  | E |  | F |  | R | ] | K |  | D |  | K |  | ⎫ | R |  | N | ⎫ | C |  | I |  | P |  | R | ⎫ | N | ⎫ | Q | ⎫ | E |  | C |  | 60 |  |
|  | |  | | | | | | | | | | | | | | | | | | | | | | | | | | | | | | | | | | | | | | | | | | | | | | | | | | | | | | | | | | | | | | | | | | | |
| 61 |  | ⎫ | T |  | I | ⎫ | D |  | K |  | R | ⎫ | N | ⎫ | C | ⎫ | C | ⎫ | R |  | R |  | ⎫ | G | ⎫ | L |  | F |  | K |  | M |  | T |  | C |  | Q |  | C |  | M |  |  | K |  | S |  | N |  | D |  | E |  | S |  | G |  | Q |  | P |  | T |  | 90 |  |
|  | |  | | | | | | | | | | | | | | | | | | | | | | | | | | | | | | | | | | | | | | | | | | | | | | | | | | | | | | | | | | | | | | | | | | | |
| 91 |  |  | E |  | K |  | C |  | T |  | C |  | R |  | R |  | P |  | R |  | P |  |  | I |  | F |  | H |  | L |  | L |  | Y |  | K |  | G |  | L |  | L |  |  | K | ⎫ | G |  | | 112 |  | | | | | | | | | | | | | | | |

Fixed PTMs: Carbamidomethylation [C53 C60 C67 C68 C77 C79 C93 C95 ]

  

All peaks (103)  Matched peaks (19)  Not matched peaks (84)

  

| Scan | Peak | Mono mass | Mono m/z | Intensity | Charge | Theoretical mass | Ion | Pos | Mass error | PPM error |
| --- | --- | --- | --- | --- | --- | --- | --- | --- | --- | --- |
| 525 | 1 | 1867.9299 | 623.6506 | 52944.73 | 3 |  |  |  |  |  |
| 525 | 2 | 623.2256 | 624.2329 | 113239.26 | 1 |  |  |  |  |  |
| 525 | 3 | 2489.2437 | 623.3182 | 18030.44 | 4 |  |  |  |  |  |
| 525 | 4 | 2471.2317 | 618.8152 | 9531.27 | 4 |  |  |  |  |  |
| 525 | 5 | 4368.7759 | 729.1366 | 11975.99 | 6 |  |  |  |  |  |
| 525 | 6 | 4311.7521 | 719.6326 | 12381.93 | 6 |  |  |  |  |  |
| 525 | 7 | 6226.4110 | 623.6484 | 12718.80 | 10 |  |  |  |  |  |
| 525 | 8 | 2261.1306 | 754.7175 | 7023.34 | 3 |  |  |  |  |  |
| 525 | 9 | 368.1130 | 369.1202 | 20999.00 | 1 |  |  |  |  |  |
| 525 | 10 | 606.1990 | 607.2063 | 17680.02 | 1 |  |  |  |  |  |
| 525 | 11 | 4222.7072 | 704.7918 | 6593.52 | 6 |  |  |  |  |  |
| 525 | 12 | 220.0764 | 221.0836 | 20606.67 | 1 |  |  |  |  |  |
| 525 | 13 | 294.0947 | 295.1020 | 20078.85 | 1 |  |  |  |  |  |
| 525 | 14 | 3409.8846 | 682.9842 | 5831.63 | 5 |  |  |  |  |  |
| 525 | 15 | 3075.4710 | 616.1015 | 3794.47 | 5 | 3075.4919 | C23 | 23 | -0.0210 | -6.82 |
| 525 | 16 | 3046.7106 | 610.3494 | 3855.68 | 5 |  |  |  |  |  |
| 525 | 17 | 4312.7550 | 863.5583 | 4409.76 | 5 |  |  |  |  |  |
| 525 | 18 | 4370.7850 | 875.1643 | 4402.61 | 5 |  |  |  |  |  |
| 525 | 19 | 4353.7728 | 871.7618 | 2965.87 | 5 |  |  |  |  |  |
| 525 | 20 | 3047.7227 | 762.9380 | 3700.29 | 4 |  |  |  |  |  |
| 525 | 21 | 4351.7606 | 726.3007 | 3849.26 | 6 |  |  |  |  |  |
| 525 | 22 | 1579.9733 | 790.9939 | 2699.62 | 2 |  |  |  |  |  |
| 525 | 23 | 1989.2326 | 664.0848 | 3665.20 | 3 |  |  |  |  |  |
| 525 | 24 | 1810.9062 | 906.4604 | 2501.77 | 2 |  |  |  |  |  |
| 525 | 25 | 1715.8200 | 572.9473 | 2163.25 | 3 | 1715.8307 | C13 | 13 | -0.0108 | -6.28 |
| 525 | 26 | 1739.8324 | 870.9235 | 2664.82 | 2 |  |  |  |  |  |
| 525 | 27 | 4240.7372 | 707.7968 | 2661.47 | 6 |  |  |  |  |  |
| 525 | 28 | 3382.7545 | 846.6959 | 2455.67 | 4 |  |  |  |  |  |
| 525 | 29 | 3115.7207 | 779.9375 | 1837.84 | 4 |  |  |  |  |  |
| 525 | 30 | 2489.2428 | 830.7549 | 2072.86 | 3 |  |  |  |  |  |
| 525 | 31 | 1184.6491 | 593.3318 | 2494.94 | 2 | 1184.6560 | C9 | 9 | -6.90e-03 | -5.83 |
| 525 | 32 | 442.1312 | 443.1385 | 3572.50 | 1 |  |  |  |  |  |
| 525 | 33 | 2678.0711 | 670.5251 | 2108.81 | 4 |  |  |  |  |  |
| 525 | 34 | 2043.0422 | 1022.5284 | 1893.29 | 2 |  |  |  |  |  |
| 525 | 35 | 4061.7019 | 813.3477 | 2893.54 | 5 |  |  |  |  |  |
| 525 | 36 | 4255.7331 | 852.1539 | 1500.37 | 5 |  |  |  |  |  |
| 525 | 37 | 2059.2551 | 687.4257 | 1520.13 | 3 |  |  |  |  |  |
| 525 | 38 | 2159.0800 | 720.7006 | 1743.03 | 3 |  |  |  |  |  |
| 525 | 39 | 1426.7483 | 476.5900 | 1212.80 | 3 | 1426.7575 | C11 | 11 | -9.24e-03 | -6.48 |
| 525 | 40 | 8041.8635 | 805.1936 | 1627.43 | 10 | 8041.9391 | C64 | 64 | -0.0756 | -9.40 |
| 525 | 41 | 2993.6258 | 749.4137 | 3398.43 | 4 |  |  |  |  |  |
| 525 | 42 | 1833.1276 | 612.0498 | 2459.35 | 3 |  |  |  |  |  |
| 525 | 43 | 2329.1709 | 777.3976 | 2872.10 | 3 | 2329.1855 | C18 | 18 | -0.0146 | -6.27 |
| 525 | 44 | 2320.3328 | 581.0905 | 2272.60 | 4 |  |  |  |  |  |
| 525 | 45 | 3586.4921 | 718.3057 | 1890.57 | 5 |  |  |  |  |  |
| 525 | 46 | 516.1495 | 517.1567 | 1836.76 | 1 |  |  |  |  |  |
| 525 | 47 | 1498.8170 | 750.4158 | 897.85 | 2 |  |  |  |  |  |
| 525 | 48 | 4333.7373 | 620.1126 | 1659.35 | 7 |  |  |  |  |  |
| 525 | 49 | 3028.7107 | 606.7494 | 1952.25 | 5 |  |  |  |  |  |
| 525 | 50 | 1210.1692 | 606.0919 | 1793.52 | 2 |  |  |  |  |  |
| 525 | 51 | 2430.2303 | 811.0840 | 1779.42 | 3 |  |  |  |  |  |
| 525 | 52 | 1929.9512 | 644.3243 | 1909.11 | 3 | 1929.9625 | C15 | 15 | -0.0113 | -5.85 |
| 525 | 53 | 3079.7158 | 1027.5792 | 1170.25 | 3 |  |  |  |  |  |
| 525 | 54 | 3075.4720 | 769.8753 | 1713.46 | 4 | 3075.4919 | C23 | 23 | -0.0200 | -6.49 |
| 525 | 55 | 312.2148 | 313.2221 | 1659.39 | 1 |  |  |  |  |  |
| 525 | 56 | 1298.6897 | 650.3521 | 2456.78 | 2 | 1298.6989 | C10 | 10 | -9.18e-03 | -7.07 |
| 525 | 57 | 388.2416 | 389.2489 | 1423.89 | 1 | 388.2434 | C3 | 3 | -1.74e-03 | -4.47 |
| 525 | 58 | 2172.0649 | 725.0289 | 1899.38 | 3 |  |  |  |  |  |
| 525 | 59 | 1815.8655 | 606.2958 | 1542.48 | 3 |  |  |  |  |  |
| 525 | 60 | 2763.2750 | 922.0989 | 1279.08 | 3 | 2763.2897 | C21 | 21 | -0.0148 | -5.34 |
| 525 | 61 | 2603.2427 | 651.8180 | 1290.12 | 4 | 2603.2591 | C20 | 20 | -0.0163 | -6.28 |
| 525 | 62 | 534.8055 | 535.8128 | 1039.07 | 1 |  |  |  |  |  |
| 525 | 63 | 658.3837 | 659.3910 | 3036.04 | 1 | 658.3874 | C5 | 5 | -3.69e-03 | -5.60 |
| 525 | 64 | 1715.8190 | 858.9168 | 865.64 | 2 | 1715.8307 | C13 | 13 | -0.0117 | -6.84 |
| 525 | 65 | 886.5589 | 444.2867 | 1073.77 | 2 |  |  |  |  |  |
| 525 | 66 | 2305.8449 | 769.6222 | 685.42 | 3 |  |  |  |  |  |
| 525 | 67 | 1298.6913 | 433.9044 | 1087.77 | 3 | 1298.6989 | C10 | 10 | -7.58e-03 | -5.83 |
| 525 | 68 | 1668.8006 | 835.4076 | 1151.01 | 2 |  |  |  |  |  |
| 525 | 69 | 2444.2120 | 612.0603 | 890.34 | 4 |  |  |  |  |  |
| 525 | 70 | 2026.9485 | 676.6568 | 1357.13 | 3 |  |  |  |  |  |
| 525 | 71 | 6240.5187 | 625.0591 | 4264.44 | 10 |  |  |  |  |  |
| 525 | 72 | 4277.7215 | 713.9609 | 995.33 | 6 |  |  |  |  |  |
| 525 | 73 | 2762.2676 | 691.5742 | 1503.28 | 4 |  |  |  |  |  |
| 525 | 74 | 2443.2148 | 815.4122 | 889.57 | 3 | 2443.2284 | C19 | 19 | -0.0136 | -5.56 |
| 525 | 75 | 2058.2531 | 515.5705 | 994.32 | 4 |  |  |  |  |  |
| 525 | 76 | 1833.1273 | 917.5709 | 2039.41 | 2 |  |  |  |  |  |
| 525 | 77 | 2603.2424 | 868.7548 | 1033.47 | 3 | 2603.2591 | C20 | 20 | -0.0166 | -6.39 |
| 525 | 78 | 2400.2229 | 801.0816 | 1180.51 | 3 |  |  |  |  |  |
| 525 | 79 | 8042.8810 | 1006.3674 | 1205.40 | 8 | 8041.9391 | C64 | 64 | -0.0605 | -7.52 |
| 525 | 80 | 822.3896 | 823.3969 | 728.72 | 1 |  |  |  |  |  |
| 525 | 81 | 713.8758 | 714.8831 | 735.91 | 1 |  |  |  |  |  |
| 525 | 82 | 847.4532 | 848.4605 | 629.28 | 1 |  |  |  |  |  |
| 525 | 83 | 1868.9333 | 935.4740 | 1071.86 | 2 |  |  |  |  |  |
| 525 | 84 | 749.3453 | 750.3526 | 861.62 | 1 |  |  |  |  |  |
| 525 | 85 | 929.6138 | 930.6211 | 1051.34 | 1 |  |  |  |  |  |
| 525 | 86 | 4039.6207 | 674.2774 | 916.26 | 6 |  |  |  |  |  |
| 525 | 87 | 1780.8737 | 891.4441 | 828.67 | 2 |  |  |  |  |  |
| 525 | 88 | 803.4874 | 804.4947 | 482.59 | 1 |  |  |  |  |  |
| 525 | 89 | 4294.7385 | 859.9550 | 1106.46 | 5 |  |  |  |  |  |
| 525 | 90 | 1402.5719 | 702.2932 | 491.38 | 2 |  |  |  |  |  |
| 525 | 91 | 2955.1815 | 739.8027 | 1003.64 | 4 |  |  |  |  |  |
| 525 | 92 | 1652.7810 | 827.3978 | 1336.54 | 2 |  |  |  |  |  |
| 525 | 93 | 7917.8194 | 792.7892 | 761.99 | 10 |  |  |  |  |  |
| 525 | 94 | 3132.4914 | 627.5056 | 993.51 | 5 | 3132.5134 | C24 | 24 | -0.0220 | -7.03 |
| 525 | 95 | 1633.3564 | 817.6855 | 1059.34 | 2 |  |  |  |  |  |
| 525 | 96 | 1525.8246 | 763.9196 | 889.87 | 2 |  |  |  |  |  |
| 525 | 97 | 6437.4072 | 716.2747 | 957.27 | 9 |  |  |  |  |  |
| 525 | 98 | 3969.4203 | 794.8913 | 1020.66 | 5 |  |  |  |  |  |
| 525 | 99 | 4994.2831 | 625.2927 | 819.94 | 8 |  |  |  |  |  |
| 525 | 100 | 5687.5667 | 711.9531 | 888.51 | 8 |  |  |  |  |  |
| 525 | 101 | 2118.3870 | 707.1363 | 686.71 | 3 |  |  |  |  |  |
| 525 | 102 | 7892.8128 | 718.5357 | 610.22 | 11 |  |  |  |  |  |
| 525 | 103 | 4336.7603 | 868.3593 | 751.46 | 5 |  |  |  |  |  |

  

All proteins /
CsTx-33a Cupiennius salei toxin 33 isoform a /
Proteoform #79
