# Supplementary material for: The Dual Prey-Inactivation Strategy of Spiders—In-Depth Venomic Analysis of Cupiennius salei
Source: Toxins (Basel). 2019 Mar 19;11(3):167. doi: 10.3390/toxins11030167 (PMC6468893; doi:10.3390/toxins11030167)
Supplement: Supplementary file 1 [file toxins-11-00167-s001.zip › Supplementary Dataset EV1/20180328_f2_topdown_OTMS2_EThcD_NL_i02_ms2_proteoform_cutoff_html/prsms/prsm155.html]

Protein-Spectrum-Match for Spectrum #393


All proteins /
CsTx-12b Cupiennius salei toxin 12 isoform b /
Proteoform #47

## Protein-Spectrum-Match #155 for Spectrum #393

|  |  |  |  |  |  |
| --- | --- | --- | --- | --- | --- |
| PrSM ID: | 155 | Scan(s): | 527 | Precursor charge: | 6 |
| Precursor m/z: | 571.9887 | Precursor mass: | 3425.8883 | Proteoform mass: | 3425.8883 |
| # matched peaks: | 28 | # matched fragment ions: | 26 | # unexpected modifications: | 1 |
| E-value: | 1.68e-20 | P-value: | 1.68e-20 | Q-value (Spectral FDR): | 0 |

  

|  |  |  |  |  |  |  |  |  |  |  |  |  |  |  |  |  |  |  |  |  |  |  |  |  |  |  |  |  |  |  |  |  |  |  |  |  |  |  |  |  |  |  |  |  |  |  |  |  |  |  |  |  |  |  |  |  |  |  |  |  |  |  |  |  |  |  |
| --- | --- | --- | --- | --- | --- | --- | --- | --- | --- | --- | --- | --- | --- | --- | --- | --- | --- | --- | --- | --- | --- | --- | --- | --- | --- | --- | --- | --- | --- | --- | --- | --- | --- | --- | --- | --- | --- | --- | --- | --- | --- | --- | --- | --- | --- | --- | --- | --- | --- | --- | --- | --- | --- | --- | --- | --- | --- | --- | --- | --- | --- | --- | --- | --- | --- | --- |
|  | | ... 30 amino acid residues are skipped at the N-terminus ... | | | | | | | | | | | | | | | | | | | | | | | | | | | | | | | | | | | | | | | | | | | | | | | | | | | | | | | | | | | | | |  | | |
|  | |  | | | | | | | | | | | | | | | | | | | | | | | | | | | | | | | | | | | | | | | | | | | | | | | | | | | | | | | | | | | | | | | | | | | |
| 31 |  |  | S |  | F |  | E |  | A |  | D |  | D |  | V |  | I |  | P |  | F |  |  | L |  | A |  | R |  | E |  | Q |  | V |  | R |  | S |  | D |  | C |  |  | T |  | L |  | R |  | N |  | H |  | D |  | C |  | T |  | D |  | D |  | 60 |  |
|  | |  | | | | | | | | | | | | | | | | | | | | | | | | | | | | | | | | | | | | | | | | | | | | | | | | | | | | | | | | | | | | | | | | | | | |
| 61 |  |  | R |  | H |  | S |  | C |  | C |  | R |  | S |  | K |  | M |  | F |  |  | K |  | D |  | V |  | C |  | K |  | C |  | F |  | Y |  | P |  | S |  |  | Q |  | R |  | S |  | D |  | T |  | A |  | R | ] | A | ⎩ | K | ⎩ | K |  | 90 |  |
|  | |  | | | | | | | | | | | | | | | | | | | | | | | | | | | | | | | | | | | | | | | | | | | | | | | | | | | | | -58.02 | | | | | | | | | | | |
| 91 |  | ⎫ | E | ⎫ | L | ⎫ | C |  | T | ⎫ | C | ⎫ | Q | ⎫ | Q |  | D | ⎱ | K |  | H |  |  | L | ⎫ | K | ⎱ | Y |  | I | ⎱ | E | ⎫ | K |  | G | ⎫ | L |  | Q | ⎱ | K |  | ⎫ | A | ⎱ | K | ⎫ | V | ⎫ | L | ⎫ | V | ⎫ | A |  | G |  | | 117 |  | | | | | |

Fixed PTMs: Carbamidomethylation [C93 C95 ]   
  
     Unexpected modifications:   Unknown [-58.02]

  

All peaks (57)  Matched peaks (28)  Not matched peaks (29)

  

| Scan | Peak | Mono mass | Mono m/z | Intensity | Charge | Theoretical mass | Ion | Pos | Mass error | PPM error |
| --- | --- | --- | --- | --- | --- | --- | --- | --- | --- | --- |
| 527 | 1 | 3368.8577 | 674.7788 | 167674.46 | 5 |  |  |  |  |  |
| 527 | 2 | 3142.6929 | 786.6805 | 68151.89 | 4 | 3142.7106 | C26 | 26 | -0.0177 | -5.64 |
| 527 | 3 | 3354.8453 | 671.9763 | 56312.58 | 5 | 3354.8631 | C28 | 28 | -0.0178 | -5.31 |
| 527 | 4 | 3368.8597 | 843.2222 | 53277.77 | 4 |  |  |  |  |  |
| 527 | 5 | 2161.1017 | 721.3745 | 51790.47 | 3 | 2161.1135 | C17 | 17 | -0.0119 | -5.49 |
| 527 | 6 | 2048.2639 | 683.7619 | 46541.61 | 3 | 2048.2629 | Z\_DOT19 | 11 | 1.01e-03 | 0.49 |
| 527 | 7 | 2290.1433 | 764.3884 | 40144.94 | 3 | 2290.1561 | C18 | 18 | -0.0129 | -5.63 |
| 527 | 8 | 1142.2963 | 572.1554 | 282962.55 | 2 |  |  |  |  |  |
| 527 | 9 | 2475.2594 | 826.0937 | 35691.70 | 3 | 2475.2726 | C20 | 20 | -0.0132 | -5.32 |
| 527 | 10 | 1884.9558 | 629.3259 | 40700.30 | 3 | 1884.9662 | C15 | 15 | -0.0104 | -5.52 |
| 527 | 11 | 3338.8242 | 668.7721 | 29177.33 | 5 | 3338.8325 | Z\_DOT29 | 1 | -8.34e-03 | -2.50 |
| 527 | 12 | 2915.5308 | 729.8900 | 28237.76 | 4 | 2915.5473 | C24 | 24 | -0.0165 | -5.64 |
| 527 | 13 | 3210.7312 | 803.6901 | 28672.18 | 4 | 3210.7376 | Z\_DOT28 | 2 | -6.31e-03 | -1.97 |
| 527 | 14 | 3408.8606 | 569.1507 | 24498.32 | 6 |  |  |  |  |  |
| 527 | 15 | 2844.4939 | 712.1308 | 25259.98 | 4 | 2844.5102 | C23 | 23 | -0.0162 | -5.70 |
| 527 | 16 | 1378.6255 | 690.3200 | 38009.11 | 2 | 1378.6333 | C11 | 11 | -7.74e-03 | -5.62 |
| 527 | 17 | 571.4802 | 572.4875 | 178840.58 | 1 |  |  |  |  |  |
| 527 | 18 | 3338.8263 | 835.7139 | 30177.48 | 4 | 3338.8325 | Z\_DOT29 | 1 | -6.20e-03 | -1.86 |
| 527 | 19 | 1541.9323 | 771.9734 | 34300.00 | 2 | 1541.9300 | Z\_DOT15 | 15 | 2.32e-03 | 1.51 |
| 527 | 20 | 1265.7878 | 633.9012 | 27488.44 | 2 | 1265.7826 | Z\_DOT13 | 17 | 5.18e-03 | 4.09 |
| 527 | 21 | 3381.8728 | 846.4755 | 21973.76 | 4 |  |  |  |  |  |
| 527 | 22 | 2716.4005 | 906.4741 | 24012.66 | 3 | 2716.4152 | C22 | 22 | -0.0147 | -5.41 |
| 527 | 23 | 3409.8636 | 682.9800 | 22891.23 | 5 |  |  |  |  |  |
| 527 | 24 | 3043.6246 | 761.9134 | 19940.10 | 4 | 3043.6422 | C25 | 25 | -0.0177 | -5.81 |
| 527 | 25 | 3381.8668 | 677.3806 | 19739.81 | 5 |  |  |  |  |  |
| 527 | 26 | 3424.8790 | 685.9831 | 223663.96 | 5 |  |  |  |  |  |
| 527 | 27 | 3255.7740 | 814.9508 | 18040.15 | 4 | 3255.7947 | C27 | 27 | -0.0207 | -6.37 |
| 527 | 28 | 1557.9522 | 779.9834 | 18569.18 | 2 |  |  |  |  |  |
| 527 | 29 | 3353.8433 | 839.4681 | 14616.15 | 4 |  |  |  |  |  |
| 527 | 30 | 2361.3765 | 591.3514 | 17098.77 | 4 |  |  |  |  |  |
| 527 | 31 | 1756.8617 | 879.4381 | 17121.29 | 2 | 1756.8712 | C14 | 14 | -9.47e-03 | -5.39 |
| 527 | 32 | 3409.8689 | 853.4745 | 17317.21 | 4 |  |  |  |  |  |
| 527 | 33 | 2716.4012 | 680.1076 | 15928.71 | 4 | 2716.4152 | C22 | 22 | -0.0140 | -5.17 |
| 527 | 34 | 1740.0564 | 581.0261 | 14852.79 | 3 |  |  |  |  |  |
| 527 | 35 | 3290.8665 | 659.1806 | 13891.35 | 5 |  |  |  |  |  |
| 527 | 36 | 685.3774 | 686.3847 | 52914.97 | 1 |  |  |  |  |  |
| 527 | 37 | 2435.4236 | 609.8632 | 13051.07 | 4 |  |  |  |  |  |
| 527 | 38 | 908.5760 | 455.2953 | 18195.18 | 2 |  |  |  |  |  |
| 527 | 39 | 1007.4841 | 1008.4913 | 12486.53 | 1 | 1007.4892 | C8 | 8 | -5.11e-03 | -5.07 |
| 527 | 40 | 1364.3467 | 683.1806 | 22731.83 | 2 |  |  |  |  |  |
| 527 | 41 | 710.4893 | 711.4966 | 10402.12 | 1 | 710.4809 | Z\_DOT8 | 22 | 8.38e-03 | 11.80 |
| 527 | 42 | 1206.7747 | 604.3946 | 7341.49 | 2 |  |  |  |  |  |
| 527 | 43 | 1349.8322 | 675.9234 | 5356.49 | 2 |  |  |  |  |  |
| 527 | 44 | 847.4538 | 848.4611 | 6825.33 | 1 | 847.4585 | C7 | 7 | -4.73e-03 | -5.58 |
| 527 | 45 | 1135.5425 | 1136.5497 | 5691.54 | 1 | 1135.5477 | C9 | 9 | -5.28e-03 | -4.65 |
| 527 | 46 | 586.3774 | 587.3846 | 5485.52 | 1 | 586.3802 | C5 | 5 | -2.81e-03 | -4.80 |
| 527 | 47 | 511.3586 | 512.3658 | 5248.72 | 1 | 511.3489 | Z\_DOT6 | 24 | 9.71e-03 | 18.99 |
| 527 | 48 | 473.2941 | 474.3013 | 6280.15 | 1 | 473.2961 | C4 | 4 | -2.07e-03 | -4.36 |
| 527 | 49 | 967.6494 | 484.8320 | 2739.04 | 2 |  |  |  |  |  |
| 527 | 50 | 780.5186 | 391.2666 | 2884.32 | 2 |  |  |  |  |  |
| 527 | 51 | 873.4700 | 874.4773 | 3727.16 | 1 |  |  |  |  |  |
| 527 | 52 | 344.2522 | 345.2595 | 3522.50 | 1 | 344.2535 | C3 | 3 | -1.28e-03 | -3.73 |
| 527 | 53 | 553.0762 | 554.0835 | 5638.05 | 1 |  |  |  |  |  |
| 527 | 54 | 1065.6731 | 533.8438 | 1864.48 | 2 |  |  |  |  |  |
| 527 | 55 | 891.9604 | 892.9677 | 2720.07 | 1 |  |  |  |  |  |
| 527 | 56 | 1078.6807 | 540.3476 | 2511.84 | 2 |  |  |  |  |  |
| 527 | 57 | 726.5080 | 727.5153 | 2517.19 | 1 |  |  |  |  |  |

  

All proteins /
CsTx-12b Cupiennius salei toxin 12 isoform b /
Proteoform #47
