# Supplementary material for: The Dual Prey-Inactivation Strategy of Spiders—In-Depth Venomic Analysis of Cupiennius salei
Source: Toxins (Basel). 2019 Mar 19;11(3):167. doi: 10.3390/toxins11030167 (PMC6468893; doi:10.3390/toxins11030167)
Supplement: Supplementary file 1 [file toxins-11-00167-s001.zip › Supplementary Dataset EV1/20180328_f2_topdown_OTMS2_EThcD_NL_i02_ms2_proteoform_cutoff_html/prsms/prsm156.html]

Protein-Spectrum-Match for Spectrum #394


All proteins /
sp|B3EWU1|TXS1A\_CUPSA Short cationic peptide-1a OS=Cupiennius salei OX=6928 PE=1 SV=1 /
Proteoform #23

## Protein-Spectrum-Match #156 for Spectrum #394

|  |  |  |  |  |  |
| --- | --- | --- | --- | --- | --- |
| PrSM ID: | 156 | Scan(s): | 528 | Precursor charge: | 6 |
| Precursor m/z: | 513.6359 | Precursor mass: | 3075.7715 | Proteoform mass: | 3075.7665 |
| # matched peaks: | 37 | # matched fragment ions: | 34 | # unexpected modifications: | 1 |
| E-value: | 7.34e-26 | P-value: | 7.34e-26 | Q-value (Spectral FDR): | 0 |

  

|  |  |  |  |  |  |  |  |  |  |  |  |  |  |  |  |  |  |  |  |  |  |  |  |  |  |  |  |  |  |  |  |  |  |  |  |  |  |  |  |  |  |  |  |  |  |  |  |  |  |  |  |  |  |  |  |  |  |  |  |  |  |  |  |  |  |  |
| --- | --- | --- | --- | --- | --- | --- | --- | --- | --- | --- | --- | --- | --- | --- | --- | --- | --- | --- | --- | --- | --- | --- | --- | --- | --- | --- | --- | --- | --- | --- | --- | --- | --- | --- | --- | --- | --- | --- | --- | --- | --- | --- | --- | --- | --- | --- | --- | --- | --- | --- | --- | --- | --- | --- | --- | --- | --- | --- | --- | --- | --- | --- | --- | --- | --- | --- |
|  | |  | | | | | | | | | | | | | | | | | | | | | | | | | | | | | | | | | | | | | | | | | | | | | | | | | | | | | | | | | -0.99 | | | | | | | |
| 1 |  |  | F | ⎩ | L | ⎩ | A |  | K | ⎱ | K |  | V | ⎱ | A | ⎫ | K | ⎱ | T | ⎱ | V |  | ⎱ | A | ⎫ | K | ⎫ | Q | ⎫ | A |  | A |  | K | ⎱ | Q | ⎱ | G | ⎱ | A | ⎱ | K |  | ⎩ | Y | ⎱ | V | ⎱ | V | ⎫ | N | ⎫ | K | ⎫ | Q | ⎫ | M | ⎫ | E |  | | 28 |  | | | |

Unexpected modifications:   Unknown [-0.99]

  

All peaks (51)  Matched peaks (37)  Not matched peaks (14)

  

| Scan | Peak | Mono mass | Mono m/z | Intensity | Charge | Theoretical mass | Ion | Pos | Mass error | PPM error |
| --- | --- | --- | --- | --- | --- | --- | --- | --- | --- | --- |
| 528 | 1 | 3075.7639 | 513.6346 | 299645.24 | 6 |  |  |  |  |  |
| 528 | 2 | 2174.1639 | 725.7286 | 95530.28 | 3 | 2174.1679 | Z\_DOT20 | 8 | -3.99e-03 | -1.83 |
| 528 | 3 | 3059.7382 | 612.9549 | 78879.77 | 5 |  |  |  |  |  |
| 528 | 4 | 2946.7154 | 737.6861 | 62072.63 | 4 | 2946.7316 | C27 | 27 | -0.0162 | -5.51 |
| 528 | 5 | 2815.6756 | 704.9262 | 51386.98 | 4 | 2815.6911 | C26 | 26 | -0.0156 | -5.52 |
| 528 | 6 | 1700.0734 | 567.6984 | 65158.28 | 3 | 1700.0824 | C16 | 16 | -8.95e-03 | -5.27 |
| 528 | 7 | 1885.1522 | 629.3914 | 60100.33 | 3 | 1885.1624 | C18 | 18 | -0.0102 | -5.41 |
| 528 | 8 | 3075.7571 | 616.1587 | 123497.74 | 5 |  |  |  |  |  |
| 528 | 9 | 902.6023 | 452.3084 | 56405.07 | 2 | 902.6065 | C8 | 8 | -4.21e-03 | -4.67 |
| 528 | 10 | 512.0511 | 513.0583 | 179043.75 | 1 |  |  |  |  |  |
| 528 | 11 | 1376.6925 | 689.3535 | 47641.11 | 2 | 1376.6919 | Z\_DOT12 | 16 | 5.94e-04 | 0.43 |
| 528 | 12 | 3058.7352 | 510.7965 | 31967.00 | 6 |  |  |  |  |  |
| 528 | 13 | 2073.1166 | 692.0461 | 30326.48 | 3 | 2073.1202 | Z\_DOT19 | 9 | -3.61e-03 | -1.74 |
| 528 | 14 | 2445.4815 | 612.3776 | 31249.77 | 4 | 2445.4947 | C23 | 23 | -0.0132 | -5.38 |
| 528 | 15 | 2687.6177 | 672.9117 | 28712.47 | 4 | 2687.6325 | C25 | 25 | -0.0148 | -5.51 |
| 528 | 16 | 1956.1889 | 653.0702 | 38587.05 | 3 | 1956.1995 | C19 | 19 | -0.0106 | -5.44 |
| 528 | 17 | 1974.0488 | 659.0236 | 37970.28 | 3 | 1974.0518 | Z\_DOT18 | 10 | -2.94e-03 | -1.49 |
| 528 | 18 | 2600.4571 | 651.1215 | 24285.52 | 4 | 2600.4633 | Z\_DOT24 | 4 | -6.22e-03 | -2.39 |
| 528 | 19 | 3017.7299 | 604.5533 | 21718.44 | 5 |  |  |  |  |  |
| 528 | 20 | 2247.3460 | 562.8438 | 21350.45 | 4 | 2247.3578 | C21 | 21 | -0.0118 | -5.27 |
| 528 | 21 | 3060.7444 | 766.1934 | 28651.47 | 4 |  |  |  |  |  |
| 528 | 22 | 2051.1757 | 513.8012 | 154118.39 | 4 |  |  |  |  |  |
| 528 | 23 | 1102.7171 | 552.3658 | 28160.78 | 2 | 1102.7226 | C10 | 10 | -5.49e-03 | -4.98 |
| 528 | 24 | 2559.5232 | 640.8881 | 23819.81 | 4 | 2559.5376 | C24 | 24 | -0.0144 | -5.61 |
| 528 | 25 | 2799.5884 | 700.9044 | 16381.15 | 4 | 2799.5954 | Z\_DOT26 | 2 | -6.96e-03 | -2.48 |
| 528 | 26 | 2912.6710 | 729.1750 | 17674.21 | 4 | 2912.6794 | Z\_DOT27 | 1 | -8.48e-03 | -2.91 |
| 528 | 27 | 1191.6136 | 596.8141 | 23937.41 | 2 | 1191.6119 | Z\_DOT10 | 18 | 1.65e-03 | 1.39 |
| 528 | 28 | 1828.1310 | 610.3843 | 21924.92 | 3 | 1828.1410 | C17 | 17 | -1.00e-02 | -5.47 |
| 528 | 29 | 1173.7536 | 587.8841 | 20166.96 | 2 | 1173.7597 | C11 | 11 | -6.11e-03 | -5.20 |
| 528 | 30 | 3031.7442 | 607.3561 | 12195.65 | 5 |  |  |  |  |  |
| 528 | 31 | 2373.2947 | 792.1055 | 12911.60 | 3 | 2373.2999 | Z\_DOT22 | 6 | -5.20e-03 | -2.19 |
| 528 | 32 | 2346.4166 | 587.6114 | 14103.68 | 4 | 2346.4262 | C22 | 22 | -9.69e-03 | -4.13 |
| 528 | 33 | 1429.9059 | 715.9602 | 11062.95 | 2 | 1429.9132 | C13 | 13 | -7.30e-03 | -5.11 |
| 528 | 34 | 1003.6493 | 502.8319 | 16114.17 | 2 | 1003.6542 | C9 | 9 | -4.83e-03 | -4.82 |
| 528 | 35 | 1429.9060 | 477.6426 | 11693.52 | 3 | 1429.9132 | C13 | 13 | -7.22e-03 | -5.05 |
| 528 | 36 | 1301.8477 | 651.9311 | 10355.74 | 2 | 1301.8546 | C12 | 12 | -6.95e-03 | -5.34 |
| 528 | 37 | 1120.5766 | 561.2956 | 14905.65 | 2 | 1120.5748 | Z\_DOT9 | 19 | 1.78e-03 | 1.59 |
| 528 | 38 | 902.6021 | 903.6094 | 7867.09 | 1 | 902.6065 | C8 | 8 | -4.38e-03 | -4.85 |
| 528 | 39 | 476.3091 | 477.3164 | 12871.17 | 1 | 476.3110 | C4 | 4 | -1.94e-03 | -4.07 |
| 528 | 40 | 829.4202 | 830.4274 | 10767.43 | 1 | 829.4165 | Z\_DOT7 | 21 | 3.65e-03 | 4.40 |
| 528 | 41 | 1248.6346 | 625.3246 | 10737.64 | 2 | 1248.6334 | Z\_DOT11 | 17 | 1.19e-03 | 0.95 |
| 528 | 42 | 992.4827 | 993.4899 | 7209.76 | 1 | 992.4798 | Z\_DOT8 | 20 | 2.82e-03 | 2.84 |
| 528 | 43 | 1499.8898 | 500.9706 | 6379.63 | 3 |  |  |  |  |  |
| 528 | 44 | 703.4711 | 704.4783 | 6059.30 | 1 | 703.4744 | C6 | 6 | -3.35e-03 | -4.77 |
| 528 | 45 | 774.5081 | 388.2613 | 5352.76 | 2 | 774.5115 | C7 | 7 | -3.43e-03 | -4.43 |
| 528 | 46 | 774.5075 | 775.5148 | 3773.68 | 1 | 774.5115 | C7 | 7 | -4.03e-03 | -5.20 |
| 528 | 47 | 730.3522 | 731.3594 | 6013.41 | 1 | 730.3481 | Z\_DOT6 | 22 | 4.06e-03 | 5.56 |
| 528 | 48 | 1224.7464 | 613.3805 | 3693.70 | 2 |  |  |  |  |  |
| 528 | 49 | 1207.6316 | 604.8231 | 3151.29 | 2 |  |  |  |  |  |
| 528 | 50 | 1377.7018 | 1378.7090 | 1988.73 | 1 |  |  |  |  |  |
| 528 | 51 | 232.1570 | 233.1642 | 2792.57 | 1 |  |  |  |  |  |

  

All proteins /
sp|B3EWU1|TXS1A\_CUPSA Short cationic peptide-1a OS=Cupiennius salei OX=6928 PE=1 SV=1 /
Proteoform #23
