# Supplementary material for: The Dual Prey-Inactivation Strategy of Spiders—In-Depth Venomic Analysis of Cupiennius salei
Source: Toxins (Basel). 2019 Mar 19;11(3):167. doi: 10.3390/toxins11030167 (PMC6468893; doi:10.3390/toxins11030167)
Supplement: Supplementary file 1 [file toxins-11-00167-s001.zip › Supplementary Dataset EV1/20180328_f2_topdown_OTMS2_EThcD_NL_i02_ms2_proteoform_cutoff_html/prsms/prsm157.html]

Protein-Spectrum-Match for Spectrum #395


All proteins /
CsTx-13a Cupiennius salei toxin 13 isoform a /
Proteoform #40

## Protein-Spectrum-Match #157 for Spectrum #395

|  |  |  |  |  |  |
| --- | --- | --- | --- | --- | --- |
| PrSM ID: | 157 | Scan(s): | 529 | Precursor charge: | 6 |
| Precursor m/z: | 580.3132 | Precursor mass: | 3475.8354 | Proteoform mass: | 3475.8354 |
| # matched peaks: | 26 | # matched fragment ions: | 21 | # unexpected modifications: | 1 |
| E-value: | 3.18e-18 | P-value: | 3.18e-18 | Q-value (Spectral FDR): | 0 |

  

|  |  |  |  |  |  |  |  |  |  |  |  |  |  |  |  |  |  |  |  |  |  |  |  |  |  |  |  |  |  |  |  |  |  |  |  |  |  |  |  |  |  |  |  |  |  |  |  |  |  |  |  |  |  |  |  |  |  |  |  |  |  |  |  |  |  |  |
| --- | --- | --- | --- | --- | --- | --- | --- | --- | --- | --- | --- | --- | --- | --- | --- | --- | --- | --- | --- | --- | --- | --- | --- | --- | --- | --- | --- | --- | --- | --- | --- | --- | --- | --- | --- | --- | --- | --- | --- | --- | --- | --- | --- | --- | --- | --- | --- | --- | --- | --- | --- | --- | --- | --- | --- | --- | --- | --- | --- | --- | --- | --- | --- | --- | --- | --- |
|  | | ... 30 amino acid residues are skipped at the N-terminus ... | | | | | | | | | | | | | | | | | | | | | | | | | | | | | | | | | | | | | | | | | | | | | | | | | | | | | | | | | | | | | |  | | |
|  | |  | | | | | | | | | | | | | | | | | | | | | | | | | | | | | | | | | | | | | | | | | | | | | | | | | | | | | | | | | | | | | | | | | | | |
| 31 |  |  | S |  | F |  | E |  | A |  | D |  | D |  | I |  | I |  | P |  | F |  |  | I |  | A |  | K |  | E |  | Q |  | V |  | R |  | S |  | D |  | C |  |  | T |  | L |  | R |  | N |  | H |  | D |  | C |  | T |  | D |  | D |  | 60 |  |
|  | |  | | | | | | | | | | | | | | | | | | | | | | | | | | | | | | | | | | | | | | | | | | | | | | | | | | | | | | | | | | | | | | | | | | | |
| 61 |  |  | R |  | H |  | S |  | C |  | C |  | R |  | S |  | K |  | M |  | F |  |  | K |  | D |  | V |  | C |  | T |  | C |  | F |  | Y |  | P |  | S |  |  | Q |  | R |  | S |  | E |  | T |  | A |  | R | ] | A |  | K | ⎩ | K |  | 90 |  |
|  | |  | | | | | | | | | | | | | | | | | | | | | | | | | | | | | | | | | | | | | | | | | | | | | | | | | | | -58.01 | | | | | | | | | | | | | |
| 91 |  |  | E | ⎫ | L |  | C |  | T | ⎫ | C | ⎫ | Q | ⎱ | Q |  | P | ⎱ | K | ⎫ | H |  |  | L |  | K | ⎱ | Y |  | I | ⎱ | E | ⎱ | K |  | G |  | L |  | Q | ⎫ | K |  | ⎱ | A |  | K | ⎫ | D | ⎫ | Y | ⎫ | A |  | T |  | G |  | | 117 |  | | | | | |

Fixed PTMs: Carbamidomethylation [C93 C95 ]   
  
     Unexpected modifications:   Unknown [-58.01]

  

All peaks (57)  Matched peaks (26)  Not matched peaks (31)

  

| Scan | Peak | Mono mass | Mono m/z | Intensity | Charge | Theoretical mass | Ion | Pos | Mass error | PPM error |
| --- | --- | --- | --- | --- | --- | --- | --- | --- | --- | --- |
| 529 | 1 | 3466.9075 | 578.8252 | 164755.13 | 6 |  |  |  |  |  |
| 529 | 2 | 3418.8003 | 684.7673 | 68135.34 | 5 |  |  |  |  |  |
| 529 | 3 | 3467.9098 | 694.5892 | 57636.22 | 5 |  |  |  |  |  |
| 529 | 4 | 1738.4137 | 580.4785 | 199057.08 | 3 |  |  |  |  |  |
| 529 | 5 | 3140.6763 | 786.1764 | 29148.97 | 4 | 3140.6950 | C26 | 26 | -0.0187 | -5.95 |
| 529 | 6 | 3025.6499 | 757.4198 | 29651.53 | 4 | 3025.6680 | C25 | 25 | -0.0181 | -5.98 |
| 529 | 7 | 3418.8034 | 855.7081 | 22036.11 | 4 |  |  |  |  |  |
| 529 | 8 | 3395.8710 | 680.1815 | 19806.98 | 5 |  |  |  |  |  |
| 529 | 9 | 2272.1689 | 758.3969 | 23953.47 | 3 | 2272.1820 | C18 | 18 | -0.0131 | -5.75 |
| 529 | 10 | 2698.4258 | 900.4825 | 18110.12 | 3 | 2698.4410 | C22 | 22 | -0.0152 | -5.65 |
| 529 | 11 | 2116.1802 | 706.4007 | 19138.56 | 3 | 2116.1842 | Z\_DOT19 | 11 | -3.93e-03 | -1.86 |
| 529 | 12 | 1866.9818 | 623.3345 | 25304.43 | 3 | 1866.9920 | C15 | 15 | -0.0102 | -5.47 |
| 529 | 13 | 1609.8504 | 805.9325 | 23440.68 | 2 | 1609.8513 | Z\_DOT15 | 15 | -9.15e-04 | -0.57 |
| 529 | 14 | 579.6382 | 580.6455 | 126952.60 | 1 |  |  |  |  |  |
| 529 | 15 | 3260.6736 | 816.1757 | 14432.55 | 4 | 3260.6847 | Z\_DOT28 | 2 | -0.0111 | -3.40 |
| 529 | 16 | 2143.1271 | 715.3830 | 19718.93 | 3 | 2143.1394 | C17 | 17 | -0.0122 | -5.71 |
| 529 | 17 | 2341.2910 | 781.4376 | 16028.41 | 3 | 2341.2955 | Z\_DOT21 | 9 | -4.50e-03 | -1.92 |
| 529 | 18 | 2826.5201 | 707.6373 | 16374.81 | 4 | 2826.5360 | C23 | 23 | -0.0159 | -5.63 |
| 529 | 19 | 3303.7391 | 661.7551 | 12291.68 | 5 | 3303.7583 | C27 | 27 | -0.0192 | -5.81 |
| 529 | 20 | 2800.4524 | 701.1204 | 15129.56 | 4 |  |  |  |  |  |
| 529 | 21 | 3459.8109 | 865.9600 | 12918.43 | 4 |  |  |  |  |  |
| 529 | 22 | 3303.7408 | 826.9425 | 12771.85 | 4 | 3303.7583 | C27 | 27 | -0.0175 | -5.31 |
| 529 | 23 | 3432.8165 | 859.2114 | 10968.59 | 4 |  |  |  |  |  |
| 529 | 24 | 2539.3760 | 635.8513 | 14446.83 | 4 |  |  |  |  |  |
| 529 | 25 | 1625.8691 | 813.9418 | 16842.29 | 2 |  |  |  |  |  |
| 529 | 26 | 3183.7195 | 796.9372 | 13832.93 | 4 |  |  |  |  |  |
| 529 | 27 | 3025.6512 | 1009.5577 | 9587.86 | 3 | 3025.6680 | C25 | 25 | -0.0169 | -5.57 |
| 529 | 28 | 1204.6625 | 603.3385 | 12598.84 | 2 | 1204.6613 | Z\_DOT12 | 18 | 1.20e-03 | 0.99 |
| 529 | 29 | 3431.8106 | 687.3694 | 7301.67 | 5 |  |  |  |  |  |
| 529 | 30 | 2202.1285 | 735.0501 | 8581.00 | 3 |  |  |  |  |  |
| 529 | 31 | 3004.5455 | 1002.5225 | 8824.51 | 3 |  |  |  |  |  |
| 529 | 32 | 695.5664 | 696.5737 | 65556.89 | 1 |  |  |  |  |  |
| 529 | 33 | 2698.4266 | 675.6139 | 8539.42 | 4 | 2698.4410 | C22 | 22 | -0.0145 | -5.36 |
| 529 | 34 | 1941.9766 | 648.3328 | 8920.58 | 3 |  |  |  |  |  |
| 529 | 35 | 2516.2853 | 839.7690 | 8981.23 | 3 |  |  |  |  |  |
| 529 | 36 | 1360.6520 | 681.3333 | 15558.70 | 2 | 1360.6591 | C11 | 11 | -7.08e-03 | -5.20 |
| 529 | 37 | 1333.7045 | 667.8595 | 8705.01 | 2 | 1333.7039 | Z\_DOT13 | 17 | 6.02e-04 | 0.45 |
| 529 | 38 | 1136.7458 | 569.3802 | 7094.29 | 2 |  |  |  |  |  |
| 529 | 39 | 562.0644 | 563.0717 | 25292.29 | 1 |  |  |  |  |  |
| 529 | 40 | 1265.7876 | 633.9011 | 5708.61 | 2 |  |  |  |  |  |
| 529 | 41 | 1378.8715 | 690.4430 | 4351.18 | 2 |  |  |  |  |  |
| 529 | 42 | 869.4617 | 870.4690 | 7527.43 | 1 |  |  |  |  |  |
| 529 | 43 | 1320.6213 | 661.3179 | 4787.61 | 2 |  |  |  |  |  |
| 529 | 44 | 650.3117 | 651.3190 | 5654.28 | 1 | 650.3073 | Z\_DOT7 | 23 | 4.40e-03 | 6.77 |
| 529 | 45 | 473.2941 | 474.3014 | 8246.64 | 1 | 473.2961 | C4 | 4 | -2.00e-03 | -4.24 |
| 529 | 46 | 908.5760 | 455.2953 | 6722.31 | 2 |  |  |  |  |  |
| 529 | 47 | 1488.7458 | 745.3802 | 7394.13 | 2 | 1488.7540 | C12 | 12 | -8.29e-03 | -5.57 |
| 529 | 48 | 1220.6811 | 611.3478 | 4246.17 | 2 |  |  |  |  |  |
| 529 | 49 | 1135.5417 | 1136.5490 | 4571.82 | 1 | 1135.5477 | C9 | 9 | -6.01e-03 | -5.30 |
| 529 | 50 | 681.9775 | 682.9847 | 7541.40 | 1 |  |  |  |  |  |
| 529 | 51 | 1192.5628 | 597.2887 | 3447.79 | 2 |  |  |  |  |  |
| 529 | 52 | 847.4570 | 848.4642 | 4897.51 | 1 | 847.4585 | C7 | 7 | -1.56e-03 | -1.84 |
| 529 | 53 | 1007.4841 | 1008.4914 | 3343.84 | 1 | 1007.4892 | C8 | 8 | -5.05e-03 | -5.01 |
| 529 | 54 | 710.4894 | 711.4967 | 3111.18 | 1 |  |  |  |  |  |
| 529 | 55 | 1488.7461 | 497.2560 | 4870.40 | 3 | 1488.7540 | C12 | 12 | -7.93e-03 | -5.33 |
| 529 | 56 | 1007.4838 | 504.7492 | 2309.20 | 2 | 1007.4892 | C8 | 8 | -5.37e-03 | -5.33 |
| 529 | 57 | 906.9424 | 907.9496 | 2123.69 | 1 |  |  |  |  |  |

  

All proteins /
CsTx-13a Cupiennius salei toxin 13 isoform a /
Proteoform #40
