# Supplementary material for: The Dual Prey-Inactivation Strategy of Spiders—In-Depth Venomic Analysis of Cupiennius salei
Source: Toxins (Basel). 2019 Mar 19;11(3):167. doi: 10.3390/toxins11030167 (PMC6468893; doi:10.3390/toxins11030167)
Supplement: Supplementary file 1 [file toxins-11-00167-s001.zip › Supplementary Dataset EV1/20180328_f2_topdown_OTMS2_EThcD_NL_i02_ms2_proteoform_cutoff_html/prsms/prsm158.html]

Protein-Spectrum-Match for Spectrum #396


All proteins /
CsTx-12a\_S1 Cupiennius salei toxin 12 isoform a S1^ACsTx-12a\_S2 Cupiennius salei toxin 12 isoform a S2 /
Proteoform #53

## Protein-Spectrum-Match #158 for Spectrum #396

|  |  |  |  |  |  |
| --- | --- | --- | --- | --- | --- |
| PrSM ID: | 158 | Scan(s): | 531 | Precursor charge: | 6 |
| Precursor m/z: | 569.3244 | Precursor mass: | 3409.9025 | Proteoform mass: | 3409.9025 |
| # matched peaks: | 26 | # matched fragment ions: | 24 | # unexpected modifications: | 1 |
| E-value: | 3.25e-19 | P-value: | 3.25e-19 | Q-value (Spectral FDR): | 0 |

  

|  |  |  |  |  |  |  |  |  |  |  |  |  |  |  |  |  |  |  |  |  |  |  |  |  |  |  |  |  |  |  |  |  |  |  |  |  |  |  |  |  |  |  |  |  |  |  |  |  |  |  |  |  |  |  |  |  |  |  |  |  |  |  |  |  |  |  |
| --- | --- | --- | --- | --- | --- | --- | --- | --- | --- | --- | --- | --- | --- | --- | --- | --- | --- | --- | --- | --- | --- | --- | --- | --- | --- | --- | --- | --- | --- | --- | --- | --- | --- | --- | --- | --- | --- | --- | --- | --- | --- | --- | --- | --- | --- | --- | --- | --- | --- | --- | --- | --- | --- | --- | --- | --- | --- | --- | --- | --- | --- | --- | --- | --- | --- | --- |
|  | | ... 30 amino acid residues are skipped at the N-terminus ... | | | | | | | | | | | | | | | | | | | | | | | | | | | | | | | | | | | | | | | | | | | | | | | | | | | | | | | | | | | | | |  | | |
|  | |  | | | | | | | | | | | | | | | | | | | | | | | | | | | | | | | | | | | | | | | | | | | | | | | | | | | | | | | | | | | | | | | | | | | |
| 31 |  |  | S |  | F |  | E |  | A |  | D |  | D |  | V |  | I |  | P |  | F |  |  | L |  | A |  | R |  | E |  | Q |  | V |  | R |  | S |  | D |  | C |  |  | T |  | L |  | R |  | N |  | H |  | D |  | C |  | T |  | D |  | D |  | 60 |  |
|  | |  | | | | | | | | | | | | | | | | | | | | | | | | | | | | | | | | | | | | | | | | | | | | | | | | | | | | | | | | | | | | | | | | | | | |
| 61 |  |  | R |  | H |  | S |  | C |  | C |  | R |  | S |  | K |  | M |  | F |  |  | K |  | D |  | V |  | C |  | K |  | C |  | F |  | Y |  | P |  | S |  |  | Q |  | R |  | S |  | D |  | T |  | A |  | R | ] | A | ⎩ | K | ⎩ | K |  | 90 |  |
|  | |  | | | | | | | | | | | | | | | | | | | | | | | | | | | | | | | | | | | | | | | | | | | | | | | | | | | | | -58.01 | | | | | | | | | | | |
| 91 |  |  | E | ⎫ | L |  | C |  | T | ⎫ | C | ⎫ | Q | ⎫ | Q | ⎫ | D |  | K |  | H |  |  | L |  | K | ⎱ | F | ⎱ | I | ⎫ | E | ⎫ | K |  | G | ⎫ | L |  | Q | ⎱ | K |  | ⎱ | A | ⎱ | K | ⎫ | V | ⎫ | L | ⎫ | V | ⎫ | A |  | G |  | | 117 |  | | | | | |

Fixed PTMs: Carbamidomethylation [C93 C95 ]   
  
     Unexpected modifications:   Unknown [-58.01]

  

All peaks (56)  Matched peaks (26)  Not matched peaks (30)

  

| Scan | Peak | Mono mass | Mono m/z | Intensity | Charge | Theoretical mass | Ion | Pos | Mass error | PPM error |
| --- | --- | --- | --- | --- | --- | --- | --- | --- | --- | --- |
| 531 | 1 | 3352.8702 | 671.5813 | 1054124.86 | 5 |  |  |  |  |  |
| 531 | 2 | 3392.8810 | 566.4874 | 794136.43 | 6 |  |  |  |  |  |
| 531 | 3 | 3321.8344 | 665.3742 | 666973.70 | 5 |  |  |  |  |  |
| 531 | 4 | 3126.7068 | 782.6840 | 332017.72 | 4 | 3126.7157 | C26 | 26 | -8.89e-03 | -2.84 |
| 531 | 5 | 3338.8590 | 668.7791 | 394860.09 | 5 | 3338.8682 | C28 | 28 | -9.23e-03 | -2.76 |
| 531 | 6 | 568.4866 | 569.4938 | 2816513.88 | 1 |  |  |  |  |  |
| 531 | 7 | 1705.9477 | 569.6565 | 4670068.64 | 3 |  |  |  |  |  |
| 531 | 8 | 3393.8742 | 679.7821 | 278168.55 | 5 |  |  |  |  |  |
| 531 | 9 | 2274.1554 | 759.0591 | 210687.48 | 3 | 2274.1612 | C18 | 18 | -5.82e-03 | -2.56 |
| 531 | 10 | 2899.5438 | 725.8932 | 202871.70 | 4 | 2899.5523 | C24 | 24 | -8.59e-03 | -2.96 |
| 531 | 11 | 2828.5066 | 708.1339 | 182419.46 | 4 | 2828.5152 | C23 | 23 | -8.59e-03 | -3.04 |
| 531 | 12 | 2145.1126 | 716.0448 | 178439.04 | 3 | 2145.1186 | C17 | 17 | -6.08e-03 | -2.83 |
| 531 | 13 | 1884.9607 | 629.3275 | 204938.75 | 3 | 1884.9662 | C15 | 15 | -5.45e-03 | -2.89 |
| 531 | 14 | 3194.7459 | 799.6937 | 125238.36 | 4 | 3194.7517 | Z\_DOT28 | 2 | -5.83e-03 | -1.83 |
| 531 | 15 | 3365.8792 | 674.1831 | 116272.61 | 5 |  |  |  |  |  |
| 531 | 16 | 3364.8827 | 561.8211 | 102668.39 | 6 |  |  |  |  |  |
| 531 | 17 | 3027.6390 | 757.9170 | 122504.94 | 4 | 3027.6473 | C25 | 25 | -8.35e-03 | -2.76 |
| 531 | 18 | 3322.8412 | 831.7176 | 97641.94 | 4 | 3322.8467 | Z\_DOT29 | 1 | -5.46e-03 | -1.64 |
| 531 | 19 | 2032.0285 | 678.3501 | 115061.43 | 3 | 2032.0346 | C16 | 16 | -6.11e-03 | -3.01 |
| 531 | 20 | 1525.9423 | 763.9784 | 157277.50 | 2 | 1525.9441 | Z\_DOT15 | 15 | -1.84e-03 | -1.20 |
| 531 | 21 | 2700.4117 | 676.1102 | 96189.77 | 4 | 2700.4203 | C22 | 22 | -8.61e-03 | -3.19 |
| 531 | 22 | 2459.2710 | 820.7643 | 103815.48 | 3 | 2459.2776 | C20 | 20 | -6.64e-03 | -2.70 |
| 531 | 23 | 2700.4134 | 901.1451 | 79156.98 | 3 | 2700.4203 | C22 | 22 | -6.89e-03 | -2.55 |
| 531 | 24 | 3408.8969 | 682.7867 | 459795.42 | 5 |  |  |  |  |  |
| 531 | 25 | 3274.8835 | 655.9840 | 83552.56 | 5 |  |  |  |  |  |
| 531 | 26 | 3210.7644 | 803.6984 | 67858.78 | 4 |  |  |  |  |  |
| 531 | 27 | 3210.7630 | 643.1599 | 63818.60 | 5 |  |  |  |  |  |
| 531 | 28 | 3293.8123 | 659.7697 | 92919.38 | 5 |  |  |  |  |  |
| 531 | 29 | 2345.3870 | 587.3540 | 93587.83 | 4 |  |  |  |  |  |
| 531 | 30 | 682.1807 | 683.1880 | 368929.35 | 1 |  |  |  |  |  |
| 531 | 31 | 3222.7647 | 645.5602 | 56671.96 | 5 |  |  |  |  |  |
| 531 | 32 | 3239.7906 | 810.9549 | 62404.30 | 4 | 3239.7998 | C27 | 27 | -9.20e-03 | -2.84 |
| 531 | 33 | 2473.4458 | 619.3687 | 65694.22 | 4 |  |  |  |  |  |
| 531 | 34 | 1263.6029 | 632.8087 | 97480.25 | 2 | 1263.6063 | C10 | 10 | -3.39e-03 | -2.68 |
| 531 | 35 | 1378.8738 | 690.4442 | 56312.39 | 2 | 1378.8757 | Z\_DOT14 | 16 | -1.91e-03 | -1.38 |
| 531 | 36 | 1364.7602 | 683.3874 | 716667.43 | 2 |  |  |  |  |  |
| 531 | 37 | 1341.1515 | 671.5830 | 222415.62 | 2 |  |  |  |  |  |
| 531 | 38 | 664.3675 | 665.3748 | 65538.34 | 1 |  |  |  |  |  |
| 531 | 39 | 908.5785 | 455.2965 | 46161.50 | 2 |  |  |  |  |  |
| 531 | 40 | 1007.4872 | 1008.4945 | 33495.84 | 1 | 1007.4892 | C8 | 8 | -1.94e-03 | -1.92 |
| 531 | 41 | 582.3965 | 583.4037 | 26337.62 | 1 | 582.3951 | Z\_DOT7 | 23 | 1.42e-03 | 2.43 |
| 531 | 42 | 710.4912 | 711.4985 | 19873.57 | 1 | 710.4900 | Z\_DOT8 | 22 | 1.18e-03 | 1.67 |
| 531 | 43 | 1206.7781 | 604.3963 | 24593.08 | 2 |  |  |  |  |  |
| 531 | 44 | 1135.5456 | 1136.5529 | 13495.17 | 1 | 1135.5477 | C9 | 9 | -2.11e-03 | -1.86 |
| 531 | 45 | 1469.8801 | 490.9673 | 8017.50 | 3 |  |  |  |  |  |
| 531 | 46 | 1496.9045 | 499.9754 | 18642.89 | 3 |  |  |  |  |  |
| 531 | 47 | 873.4723 | 874.4796 | 11887.04 | 1 |  |  |  |  |  |
| 531 | 48 | 1349.8358 | 450.9525 | 7981.71 | 3 |  |  |  |  |  |
| 531 | 49 | 1007.4864 | 504.7505 | 14365.89 | 2 | 1007.4892 | C8 | 8 | -2.81e-03 | -2.78 |
| 531 | 50 | 847.4567 | 848.4639 | 16021.67 | 1 | 847.4585 | C7 | 7 | -1.86e-03 | -2.20 |
| 531 | 51 | 473.2953 | 474.3026 | 13753.35 | 1 | 473.2961 | C4 | 4 | -7.84e-04 | -1.66 |
| 531 | 52 | 511.3600 | 512.3672 | 15428.35 | 1 | 511.3579 | Z\_DOT6 | 24 | 2.03e-03 | 3.96 |
| 531 | 53 | 598.4153 | 599.4225 | 9670.97 | 1 |  |  |  |  |  |
| 531 | 54 | 1078.6836 | 540.3491 | 11101.80 | 2 |  |  |  |  |  |
| 531 | 55 | 967.6517 | 484.8331 | 7884.57 | 2 |  |  |  |  |  |
| 531 | 56 | 726.5099 | 727.5171 | 8903.43 | 1 |  |  |  |  |  |

  

All proteins /
CsTx-12a\_S1 Cupiennius salei toxin 12 isoform a S1^ACsTx-12a\_S2 Cupiennius salei toxin 12 isoform a S2 /
Proteoform #53
