# Supplementary material for: The Dual Prey-Inactivation Strategy of Spiders—In-Depth Venomic Analysis of Cupiennius salei
Source: Toxins (Basel). 2019 Mar 19;11(3):167. doi: 10.3390/toxins11030167 (PMC6468893; doi:10.3390/toxins11030167)
Supplement: Supplementary file 1 [file toxins-11-00167-s001.zip › Supplementary Dataset EV1/20180328_f2_topdown_OTMS2_EThcD_NL_i02_ms2_proteoform_cutoff_html/prsms/prsm159.html]

Protein-Spectrum-Match for Spectrum #398


All proteins /
CsTx-12b Cupiennius salei toxin 12 isoform b /
Proteoform #47

## Protein-Spectrum-Match #159 for Spectrum #398

|  |  |  |  |  |  |
| --- | --- | --- | --- | --- | --- |
| PrSM ID: | 159 | Scan(s): | 533 | Precursor charge: | 6 |
| Precursor m/z: | 571.9894 | Precursor mass: | 3425.8927 | Proteoform mass: | 3425.8927 |
| # matched peaks: | 28 | # matched fragment ions: | 26 | # unexpected modifications: | 1 |
| E-value: | 1.68e-20 | P-value: | 1.68e-20 | Q-value (Spectral FDR): | 0 |

  

|  |  |  |  |  |  |  |  |  |  |  |  |  |  |  |  |  |  |  |  |  |  |  |  |  |  |  |  |  |  |  |  |  |  |  |  |  |  |  |  |  |  |  |  |  |  |  |  |  |  |  |  |  |  |  |  |  |  |  |  |  |  |  |  |  |  |  |
| --- | --- | --- | --- | --- | --- | --- | --- | --- | --- | --- | --- | --- | --- | --- | --- | --- | --- | --- | --- | --- | --- | --- | --- | --- | --- | --- | --- | --- | --- | --- | --- | --- | --- | --- | --- | --- | --- | --- | --- | --- | --- | --- | --- | --- | --- | --- | --- | --- | --- | --- | --- | --- | --- | --- | --- | --- | --- | --- | --- | --- | --- | --- | --- | --- | --- | --- |
|  | | ... 30 amino acid residues are skipped at the N-terminus ... | | | | | | | | | | | | | | | | | | | | | | | | | | | | | | | | | | | | | | | | | | | | | | | | | | | | | | | | | | | | | |  | | |
|  | |  | | | | | | | | | | | | | | | | | | | | | | | | | | | | | | | | | | | | | | | | | | | | | | | | | | | | | | | | | | | | | | | | | | | |
| 31 |  |  | S |  | F |  | E |  | A |  | D |  | D |  | V |  | I |  | P |  | F |  |  | L |  | A |  | R |  | E |  | Q |  | V |  | R |  | S |  | D |  | C |  |  | T |  | L |  | R |  | N |  | H |  | D |  | C |  | T |  | D |  | D |  | 60 |  |
|  | |  | | | | | | | | | | | | | | | | | | | | | | | | | | | | | | | | | | | | | | | | | | | | | | | | | | | | | | | | | | | | | | | | | | | |
| 61 |  |  | R |  | H |  | S |  | C |  | C |  | R |  | S |  | K |  | M |  | F |  |  | K |  | D |  | V |  | C |  | K |  | C |  | F |  | Y |  | P |  | S |  |  | Q |  | R |  | S |  | D |  | T |  | A |  | R | ] | A | ⎩ | K | ⎩ | K |  | 90 |  |
|  | |  | | | | | | | | | | | | | | | | | | | | | | | | | | | | | | | | | | | | | | | | | | | | | | | | | | | | | -58.01 | | | | | | | | | | | |
| 91 |  | ⎫ | E | ⎫ | L | ⎫ | C |  | T | ⎫ | C | ⎫ | Q | ⎫ | Q |  | D | ⎱ | K |  | H |  |  | L | ⎫ | K | ⎱ | Y |  | I | ⎱ | E | ⎫ | K |  | G | ⎫ | L |  | Q | ⎱ | K |  | ⎫ | A | ⎱ | K | ⎫ | V | ⎫ | L | ⎫ | V | ⎫ | A |  | G |  | | 117 |  | | | | | |

Fixed PTMs: Carbamidomethylation [C93 C95 ]   
  
     Unexpected modifications:   Unknown [-58.01]

  

All peaks (57)  Matched peaks (28)  Not matched peaks (29)

  

| Scan | Peak | Mono mass | Mono m/z | Intensity | Charge | Theoretical mass | Ion | Pos | Mass error | PPM error |
| --- | --- | --- | --- | --- | --- | --- | --- | --- | --- | --- |
| 533 | 1 | 3368.8571 | 674.7787 | 112993.56 | 5 |  |  |  |  |  |
| 533 | 2 | 3424.8853 | 571.8215 | 201320.59 | 6 |  |  |  |  |  |
| 533 | 3 | 3142.6929 | 786.6805 | 41710.50 | 4 | 3142.7106 | C26 | 26 | -0.0177 | -5.64 |
| 533 | 4 | 3354.8428 | 671.9758 | 39028.05 | 5 | 3354.8631 | C28 | 28 | -0.0203 | -6.04 |
| 533 | 5 | 3368.8590 | 843.2220 | 33124.94 | 4 |  |  |  |  |  |
| 533 | 6 | 2161.1011 | 721.3743 | 33097.74 | 3 | 2161.1135 | C17 | 17 | -0.0124 | -5.75 |
| 533 | 7 | 2048.2633 | 683.7617 | 31240.46 | 3 | 2048.2672 | Z\_DOT19 | 11 | -3.93e-03 | -1.92 |
| 533 | 8 | 3409.8615 | 682.9796 | 21084.42 | 5 |  |  |  |  |  |
| 533 | 9 | 2475.2583 | 826.0934 | 24557.66 | 3 | 2475.2726 | C20 | 20 | -0.0143 | -5.76 |
| 533 | 10 | 1884.9554 | 629.3257 | 27281.88 | 3 | 1884.9662 | C15 | 15 | -0.0108 | -5.71 |
| 533 | 11 | 2284.2527 | 572.0705 | 207478.78 | 4 |  |  |  |  |  |
| 533 | 12 | 2290.1429 | 764.3882 | 22482.54 | 3 | 2290.1561 | C18 | 18 | -0.0133 | -5.79 |
| 533 | 13 | 3210.7310 | 803.6900 | 18141.74 | 4 | 3210.7419 | Z\_DOT28 | 2 | -0.0110 | -3.41 |
| 533 | 14 | 1378.6254 | 690.3200 | 25117.41 | 2 | 1378.6333 | C11 | 11 | -7.87e-03 | -5.71 |
| 533 | 15 | 2915.5306 | 729.8899 | 18894.57 | 4 | 2915.5473 | C24 | 24 | -0.0167 | -5.73 |
| 533 | 16 | 2844.4939 | 712.1308 | 17473.56 | 4 | 2844.5102 | C23 | 23 | -0.0162 | -5.70 |
| 533 | 17 | 3338.8256 | 835.7137 | 18747.34 | 4 | 3338.8369 | Z\_DOT29 | 1 | -0.0113 | -3.39 |
| 533 | 18 | 3408.8580 | 569.1503 | 14006.16 | 6 |  |  |  |  |  |
| 533 | 19 | 571.4806 | 572.4879 | 118604.64 | 1 |  |  |  |  |  |
| 533 | 20 | 1541.9324 | 771.9735 | 23934.55 | 2 | 1541.9344 | Z\_DOT15 | 15 | -1.95e-03 | -1.27 |
| 533 | 21 | 2716.3998 | 906.4739 | 16710.51 | 3 | 2716.4152 | C22 | 22 | -0.0154 | -5.68 |
| 533 | 22 | 3338.8233 | 668.7719 | 18796.74 | 5 | 3338.8369 | Z\_DOT29 | 1 | -0.0137 | -4.09 |
| 533 | 23 | 3043.6241 | 761.9133 | 13044.50 | 4 | 3043.6422 | C25 | 25 | -0.0182 | -5.97 |
| 533 | 24 | 3381.8711 | 846.4750 | 13118.55 | 4 |  |  |  |  |  |
| 533 | 25 | 1265.7875 | 633.9010 | 17698.64 | 2 | 1265.7870 | Z\_DOT13 | 17 | 5.40e-04 | 0.43 |
| 533 | 26 | 3381.8643 | 677.3801 | 11105.38 | 5 |  |  |  |  |  |
| 533 | 27 | 3226.7498 | 807.6947 | 7990.41 | 4 |  |  |  |  |  |
| 533 | 28 | 2361.3763 | 591.3513 | 11798.40 | 4 |  |  |  |  |  |
| 533 | 29 | 3255.7759 | 814.9513 | 10876.29 | 4 | 3255.7947 | C27 | 27 | -0.0188 | -5.77 |
| 533 | 30 | 3409.8668 | 853.4740 | 12187.52 | 4 |  |  |  |  |  |
| 533 | 31 | 2716.3990 | 680.1070 | 10772.92 | 4 | 2716.4152 | C22 | 22 | -0.0162 | -5.97 |
| 533 | 32 | 3424.8769 | 685.9826 | 82295.84 | 5 |  |  |  |  |  |
| 533 | 33 | 2750.5101 | 688.6348 | 7184.14 | 4 |  |  |  |  |  |
| 533 | 34 | 1756.8615 | 879.4380 | 11807.09 | 2 | 1756.8712 | C14 | 14 | -9.71e-03 | -5.53 |
| 533 | 35 | 1740.0558 | 581.0259 | 9835.94 | 3 |  |  |  |  |  |
| 533 | 36 | 685.5778 | 686.5851 | 47370.78 | 1 |  |  |  |  |  |
| 533 | 37 | 908.5760 | 455.2953 | 14879.20 | 2 |  |  |  |  |  |
| 533 | 38 | 1007.4840 | 1008.4913 | 8888.20 | 1 | 1007.4892 | C8 | 8 | -5.17e-03 | -5.13 |
| 533 | 39 | 710.4892 | 711.4965 | 7365.86 | 1 | 710.4853 | Z\_DOT8 | 22 | 3.93e-03 | 5.53 |
| 533 | 40 | 1206.7743 | 604.3944 | 5216.17 | 2 |  |  |  |  |  |
| 533 | 41 | 1135.5426 | 1136.5499 | 4102.52 | 1 | 1135.5477 | C9 | 9 | -5.16e-03 | -4.54 |
| 533 | 42 | 1349.8317 | 675.9231 | 3399.82 | 2 |  |  |  |  |  |
| 533 | 43 | 417.0754 | 418.0827 | 8174.66 | 1 |  |  |  |  |  |
| 533 | 44 | 553.0762 | 554.0834 | 9989.96 | 1 |  |  |  |  |  |
| 533 | 45 | 847.4537 | 848.4610 | 4444.07 | 1 | 847.4585 | C7 | 7 | -4.79e-03 | -5.65 |
| 533 | 46 | 511.3586 | 512.3658 | 3962.70 | 1 | 511.3532 | Z\_DOT6 | 24 | 5.32e-03 | 10.40 |
| 533 | 47 | 473.2940 | 474.3013 | 3910.64 | 1 | 473.2961 | C4 | 4 | -2.10e-03 | -4.43 |
| 533 | 48 | 586.3773 | 587.3846 | 3329.61 | 1 | 586.3802 | C5 | 5 | -2.87e-03 | -4.90 |
| 533 | 49 | 873.4693 | 874.4766 | 2290.54 | 1 |  |  |  |  |  |
| 533 | 50 | 535.0659 | 536.0732 | 2313.71 | 1 |  |  |  |  |  |
| 533 | 51 | 780.5183 | 391.2664 | 1985.56 | 2 |  |  |  |  |  |
| 533 | 52 | 399.0650 | 400.0722 | 3074.71 | 1 |  |  |  |  |  |
| 533 | 53 | 967.6494 | 484.8320 | 2059.67 | 2 |  |  |  |  |  |
| 533 | 54 | 344.2523 | 345.2596 | 1891.50 | 1 | 344.2535 | C3 | 3 | -1.25e-03 | -3.64 |
| 533 | 55 | 1078.6805 | 540.3475 | 1623.95 | 2 |  |  |  |  |  |
| 533 | 56 | 1279.5942 | 640.8044 | 1719.84 | 2 |  |  |  |  |  |
| 533 | 57 | 726.5077 | 727.5150 | 2051.18 | 1 |  |  |  |  |  |

  

All proteins /
CsTx-12b Cupiennius salei toxin 12 isoform b /
Proteoform #47
