# Supplementary material for: The Dual Prey-Inactivation Strategy of Spiders—In-Depth Venomic Analysis of Cupiennius salei
Source: Toxins (Basel). 2019 Mar 19;11(3):167. doi: 10.3390/toxins11030167 (PMC6468893; doi:10.3390/toxins11030167)
Supplement: Supplementary file 1 [file toxins-11-00167-s001.zip › Supplementary Dataset EV1/20180328_f2_topdown_OTMS2_EThcD_NL_i02_ms2_proteoform_cutoff_html/prsms/prsm16.html]

Protein-Spectrum-Match for Spectrum #228


All proteins /
CsTx-8b Cupiennius salei toxin 8 isoform b /
Proteoform #101

## Protein-Spectrum-Match #16 for Spectrum #228

|  |  |  |  |  |  |
| --- | --- | --- | --- | --- | --- |
| PrSM ID: | 16 | Scan(s): | 307 | Precursor charge: | 6 |
| Precursor m/z: | 734.3025 | Precursor mass: | 4399.7716 | Proteoform mass: | 4399.8566 |
| # matched peaks: | 14 | # matched fragment ions: | 12 | # unexpected modifications: | 1 |
| E-value: | 2.08e-09 | P-value: | 2.08e-09 | Q-value (Spectral FDR): | 0 |

  

|  |  |  |  |  |  |  |  |  |  |  |  |  |  |  |  |  |  |  |  |  |  |  |  |  |  |  |  |  |  |  |  |  |  |  |  |  |  |  |  |  |  |  |  |  |  |  |  |  |  |  |  |  |  |  |  |  |  |  |  |  |  |  |  |  |  |  |  |  |  |
| --- | --- | --- | --- | --- | --- | --- | --- | --- | --- | --- | --- | --- | --- | --- | --- | --- | --- | --- | --- | --- | --- | --- | --- | --- | --- | --- | --- | --- | --- | --- | --- | --- | --- | --- | --- | --- | --- | --- | --- | --- | --- | --- | --- | --- | --- | --- | --- | --- | --- | --- | --- | --- | --- | --- | --- | --- | --- | --- | --- | --- | --- | --- | --- | --- | --- | --- | --- | --- | --- |
|  | |  | | | | | | | | | | | | | | | | | | | | | | | | | | | | | | | | | | | | | | | | | | | | | | | | | | | | | | | | | | | | | | | | | | | |
| 1 |  |  | M |  | K |  | V |  | L |  | V |  | I |  | C |  | A |  | V |  | L |  |  | F |  | L |  | S |  | I |  | F |  | S |  | N |  | S |  | S |  | A |  |  | E |  | T |  | E |  | D |  | D |  | F |  | L |  | E |  | D |  | E |  | 30 |  |
|  | |  | | | | | | | | | | | | | | | | | | | | | | | | | | | | | | | | | | | | | | | | | | | | | | | | | | | | | | | | | | | | | | | | | | | |
| 31 |  |  | S |  | F |  | Q |  | A |  | D |  | D |  | V |  | I |  | P |  | F |  |  | L |  | A |  | S |  | E |  | Q |  | V |  | R | ] | S |  | D |  | C |  |  | T | ⎫ | L | ⎫ | R | ⎫ | N | ⎫ | H | ⎫ | D | ⎫ | C | ⎫ | T | ⎫ | D | ⎫ | D |  | 60 |  |
|  | |  | | | | | | | | | | | | | | | | | -342.13 | | | | | | | | | | | | | | | | | | | | | | | | | | | | | | | | | | | | | | | | | | | | | | | |
| 61 |  | ⎫ | R |  | H |  | S |  | C |  | C |  | R |  | S |  | K | ⎫ | M |  | F |  |  | K |  | D |  | V |  | C | ⎫ | K |  | C |  | F |  | Y |  | P |  | S |  |  | Q |  | R |  | S |  | E | [ | T |  | D |  | R |  | A |  | K |  | K |  | 90 |  |
|  | |  | | | | | | | | | | | | | | | | | | | | | | | | | | | | | | | | | | | | | | | | | | | | | | | | | | | | | | | | | | | | | | | | | | | |
| 91 |  |  | E |  | L |  | C |  | T |  | C |  | Q |  | Q |  | P |  | K |  | H |  |  | L |  | K |  | Y |  | I |  | E |  | K |  | G |  | L |  | Q |  | K |  |  | A |  | K |  | D |  | Y |  | A |  | T |  | G |  | | 117 |  | | | | | |

Fixed PTMs: Carbamidomethylation [C50 C57 C64 C65 C74 C76 ]   
  
     Unexpected modifications:   Unknown [-342.13]

  

All peaks (73)  Matched peaks (14)  Not matched peaks (59)

  

| Scan | Peak | Mono mass | Mono m/z | Intensity | Charge | Theoretical mass | Ion | Pos | Mass error | PPM error |
| --- | --- | --- | --- | --- | --- | --- | --- | --- | --- | --- |
| 307 | 1 | 4294.7291 | 716.7955 | 193480.57 | 6 |  |  |  |  |  |
| 307 | 2 | 4343.7359 | 869.7544 | 170005.79 | 5 |  |  |  |  |  |
| 307 | 3 | 4295.7345 | 860.1542 | 120650.93 | 5 |  |  |  |  |  |
| 307 | 4 | 4237.7071 | 848.5487 | 112239.52 | 5 |  |  |  |  |  |
| 307 | 5 | 4389.7839 | 732.6379 | 434742.41 | 6 |  |  |  |  |  |
| 307 | 6 | 4276.7192 | 713.7938 | 84275.91 | 6 |  |  |  |  |  |
| 307 | 7 | 3964.5767 | 793.9226 | 73090.87 | 5 |  |  |  |  |  |
| 307 | 8 | 4148.6584 | 830.7389 | 37840.71 | 5 |  |  |  |  |  |
| 307 | 9 | 4390.7822 | 879.1637 | 76905.88 | 5 |  |  |  |  |  |
| 307 | 10 | 1467.5898 | 734.8022 | 74147.25 | 2 |  |  |  |  |  |
| 307 | 11 | 4286.7154 | 1072.6861 | 21116.85 | 4 |  |  |  |  |  |
| 307 | 12 | 4343.7388 | 1086.9420 | 27933.25 | 4 |  |  |  |  |  |
| 307 | 13 | 4237.7095 | 1060.4347 | 19084.90 | 4 |  |  |  |  |  |
| 307 | 14 | 2311.9938 | 771.6719 | 19912.85 | 3 |  |  |  |  |  |
| 307 | 15 | 4278.7127 | 856.7498 | 15237.51 | 5 |  |  |  |  |  |
| 307 | 16 | 1491.5746 | 746.7946 | 17393.22 | 2 | 1491.5830 | C12 | 12 | -8.41e-03 | -5.64 |
| 307 | 17 | 1987.7921 | 994.9033 | 18165.23 | 2 |  |  |  |  |  |
| 307 | 18 | 3987.6529 | 997.9205 | 14874.87 | 4 |  |  |  |  |  |
| 307 | 19 | 3250.3687 | 813.5995 | 9793.56 | 4 |  |  |  |  |  |
| 307 | 20 | 3103.2737 | 776.8257 | 11908.36 | 4 |  |  |  |  |  |
| 307 | 21 | 2463.9504 | 822.3241 | 14808.46 | 3 |  |  |  |  |  |
| 307 | 22 | 4261.7389 | 853.3551 | 18169.78 | 5 |  |  |  |  |  |
| 307 | 23 | 4205.7342 | 842.1541 | 11194.40 | 5 |  |  |  |  |  |
| 307 | 24 | 3606.4916 | 902.6302 | 14624.35 | 4 |  |  |  |  |  |
| 307 | 25 | 2761.1116 | 691.2852 | 9820.68 | 4 |  |  |  |  |  |
| 307 | 26 | 1606.6009 | 804.3077 | 11147.97 | 2 | 1606.6100 | C13 | 13 | -9.10e-03 | -5.67 |
| 307 | 27 | 3986.6515 | 798.3376 | 9313.62 | 5 |  |  |  |  |  |
| 307 | 28 | 2935.5103 | 734.8849 | 111254.01 | 4 |  |  |  |  |  |
| 307 | 29 | 4091.6764 | 819.3426 | 8907.57 | 5 |  |  |  |  |  |
| 307 | 30 | 2550.9823 | 851.3347 | 10735.84 | 3 |  |  |  |  |  |
| 307 | 31 | 1376.5480 | 689.2813 | 9396.40 | 2 | 1376.5561 | C11 | 11 | -8.08e-03 | -5.87 |
| 307 | 32 | 3818.5401 | 955.6423 | 9358.85 | 4 |  |  |  |  |  |
| 307 | 33 | 4297.7438 | 1075.4432 | 17884.76 | 4 |  |  |  |  |  |
| 307 | 34 | 3116.2666 | 780.0739 | 5745.94 | 4 | 3116.2961 | C27 | 27 | -0.0294 | -9.45 |
| 307 | 35 | 4385.7491 | 1097.4445 | 7653.58 | 4 |  |  |  |  |  |
| 307 | 36 | 4061.6296 | 813.3332 | 8014.13 | 5 |  |  |  |  |  |
| 307 | 37 | 4165.6850 | 834.1443 | 6734.40 | 5 |  |  |  |  |  |
| 307 | 38 | 4093.6934 | 1024.4306 | 6987.58 | 4 |  |  |  |  |  |
| 307 | 39 | 2201.3857 | 1101.7001 | 9296.17 | 2 |  |  |  |  |  |
| 307 | 40 | 3671.4745 | 918.8759 | 7189.19 | 4 |  |  |  |  |  |
| 307 | 41 | 3546.3944 | 887.6059 | 7656.49 | 4 |  |  |  |  |  |
| 307 | 42 | 4260.7305 | 1066.1899 | 5798.74 | 4 |  |  |  |  |  |
| 307 | 43 | 880.3528 | 881.3601 | 16965.28 | 1 |  |  |  |  |  |
| 307 | 44 | 2218.9465 | 740.6561 | 6367.75 | 3 |  |  |  |  |  |
| 307 | 45 | 2678.0769 | 893.6996 | 6085.80 | 3 | 2678.0914 | C21 | 21 | -0.0145 | -5.42 |
| 307 | 46 | 4355.7570 | 872.1587 | 6827.42 | 5 |  |  |  |  |  |
| 307 | 47 | 4180.6866 | 1046.1789 | 6556.35 | 4 |  |  |  |  |  |
| 307 | 48 | 2820.1814 | 706.0526 | 5433.80 | 4 |  |  |  |  |  |
| 307 | 49 | 4189.7109 | 838.9495 | 6202.37 | 5 |  |  |  |  |  |
| 307 | 50 | 4131.6518 | 827.3376 | 6828.91 | 5 |  |  |  |  |  |
| 307 | 51 | 3965.5792 | 992.4021 | 5311.88 | 4 |  |  |  |  |  |
| 307 | 52 | 2898.2134 | 967.0784 | 5132.12 | 3 |  |  |  |  |  |
| 307 | 53 | 1000.4452 | 501.2299 | 6992.17 | 2 | 1000.4508 | C8 | 8 | -5.59e-03 | -5.59 |
| 307 | 54 | 749.3450 | 750.3522 | 11215.31 | 1 | 749.3490 | C6 | 6 | -4.02e-03 | -5.36 |
| 307 | 55 | 863.3871 | 864.3943 | 6534.08 | 1 | 863.3919 | C7 | 7 | -4.83e-03 | -5.60 |
| 307 | 56 | 330.1526 | 331.1599 | 10639.55 | 1 |  |  |  |  |  |
| 307 | 57 | 1275.5005 | 638.7575 | 3944.40 | 2 | 1275.5084 | C10 | 10 | -7.89e-03 | -6.18 |
| 307 | 58 | 732.1306 | 733.1379 | 42346.13 | 1 |  |  |  |  |  |
| 307 | 59 | 1000.4450 | 1001.4523 | 4433.43 | 1 | 1000.4508 | C8 | 8 | -5.82e-03 | -5.82 |
| 307 | 60 | 1115.4718 | 558.7432 | 5054.74 | 2 | 1115.4778 | C9 | 9 | -5.92e-03 | -5.31 |
| 307 | 61 | 694.2926 | 695.2999 | 3060.82 | 1 |  |  |  |  |  |
| 307 | 62 | 982.4170 | 983.4243 | 2696.31 | 1 |  |  |  |  |  |
| 307 | 63 | 1101.4444 | 1102.4516 | 6128.55 | 1 |  |  |  |  |  |
| 307 | 64 | 493.2150 | 494.2223 | 1576.29 | 1 |  |  |  |  |  |
| 307 | 65 | 593.2447 | 594.2520 | 1583.34 | 1 | 593.2479 | C5 | 5 | -3.19e-03 | -5.37 |
| 307 | 66 | 1115.4707 | 1116.4780 | 1644.50 | 1 | 1115.4778 | C9 | 9 | -7.07e-03 | -6.33 |
| 307 | 67 | 576.2187 | 577.2260 | 946.86 | 1 |  |  |  |  |  |
| 307 | 68 | 1038.7567 | 1039.7640 | 791.01 | 1 |  |  |  |  |  |
| 307 | 69 | 1287.5168 | 1288.5241 | 1050.70 | 1 |  |  |  |  |  |
| 307 | 70 | 1443.9150 | 1444.9223 | 666.04 | 1 |  |  |  |  |  |
| 307 | 71 | 640.2825 | 641.2897 | 612.27 | 1 |  |  |  |  |  |
| 307 | 72 | 983.4189 | 492.7167 | 827.92 | 2 |  |  |  |  |  |
| 307 | 73 | 480.1614 | 481.1687 | 1375.57 | 1 | 480.1638 | C4 | 4 | -2.38e-03 | -4.95 |

  

All proteins /
CsTx-8b Cupiennius salei toxin 8 isoform b /
Proteoform #101
