# Supplementary material for: The Dual Prey-Inactivation Strategy of Spiders—In-Depth Venomic Analysis of Cupiennius salei
Source: Toxins (Basel). 2019 Mar 19;11(3):167. doi: 10.3390/toxins11030167 (PMC6468893; doi:10.3390/toxins11030167)
Supplement: Supplementary file 1 [file toxins-11-00167-s001.zip › Supplementary Dataset EV1/20180328_f2_topdown_OTMS2_EThcD_NL_i02_ms2_proteoform_cutoff_html/prsms/prsm160.html]

Protein-Spectrum-Match for Spectrum #399


All proteins /
CsTx-9a Cupiennius salei toxin 9 isoform a /
Proteoform #10

## Protein-Spectrum-Match #160 for Spectrum #399

|  |  |  |  |  |  |
| --- | --- | --- | --- | --- | --- |
| PrSM ID: | 160 | Scan(s): | 535 | Precursor charge: | 12 |
| Precursor m/z: | 640.3990 | Precursor mass: | 7672.7004 | Proteoform mass: | 7672.6994 |
| # matched peaks: | 48 | # matched fragment ions: | 39 | # unexpected modifications: | 0 |
| E-value: | 8.37e-36 | P-value: | 8.37e-36 | Q-value (Spectral FDR): | 0 |

  

|  |  |  |  |  |  |  |  |  |  |  |  |  |  |  |  |  |  |  |  |  |  |  |  |  |  |  |  |  |  |  |  |  |  |  |  |  |  |  |  |  |  |  |  |  |  |  |  |  |  |  |  |  |  |  |  |  |  |  |  |  |  |  |  |  |  |  |  |  |  |
| --- | --- | --- | --- | --- | --- | --- | --- | --- | --- | --- | --- | --- | --- | --- | --- | --- | --- | --- | --- | --- | --- | --- | --- | --- | --- | --- | --- | --- | --- | --- | --- | --- | --- | --- | --- | --- | --- | --- | --- | --- | --- | --- | --- | --- | --- | --- | --- | --- | --- | --- | --- | --- | --- | --- | --- | --- | --- | --- | --- | --- | --- | --- | --- | --- | --- | --- | --- | --- | --- |
|  | |  | | | | | | | | | | | | | | | | | | | | | | | | | | | | | | | | | | | | | | | | | | | | | | | | | | | | | | | | | | | | | | | | | | | |
| 1 |  |  | M |  | K |  | V |  | L |  | V |  | I |  | C |  | A |  | V |  | L |  |  | F |  | L |  | A |  | I |  | F |  | S |  | N |  | S |  | S |  | A |  |  | E |  | T |  | E |  | D |  | D |  | F |  | L |  | E |  | D |  | E |  | 30 |  |
|  | |  | | | | | | | | | | | | | | | | | | | | | | | | | | | | | | | | | | | | | | | | | | | | | | | | | | | | | | | | | | | | | | | | | | | |
| 31 |  |  | S |  | F |  | E |  | A |  | D |  | D |  | V |  | I |  | P |  | F |  |  | L |  | A |  | R |  | E |  | Q |  | V |  | R | ] | K |  | D | ⎩ | D |  | ⎫ | K | ⎫ | N | ⎫ | C | ⎫ | I |  | P |  | K | ⎫ | H | ⎫ | H | ⎫ | E | ⎫ | C |  | 60 |  |
|  | |  | | | | | | | | | | | | | | | | | | | | | | | | | | | | | | | | | | | | | | | | | | | | | | | | | | | | | | | | | | | | | | | | | | | |
| 61 |  |  | T | ⎱ | N | ⎱ | D | ⎱ | K | ⎫ | K | ⎫ | N | ⎫ | C | ⎫ | C |  | K |  | K |  | ⎱ | G | ⎱ | L |  | T | ⎱ | K | ⎫ | M | ⎫ | K |  | C | ⎫ | K | ⎫ | C | ⎫ | F |  |  | T |  | V |  | A | ⎩ | D | ⎱ | A |  | K | ⎱ | G |  | A | ⎫ | T | ⎫ | S |  | 90 |  |
|  | |  | | | | | | | | | | | | | | | | | | | | | | | | | | | | | | | | | | | | | | | | | | | | | | | | | | | | | | | | | | | | | | | | | | | |
| 91 |  |  | E |  | R | ⎫ | C |  | A |  | C |  | D |  | S |  | S | ⎫ | L |  | L |  |  | Q |  | K |  | F |  | G |  | F |  | T |  | G |  | L |  | H |  | I |  |  | I |  | K | [ | G |  | L |  | F |  | | 115 |  | | | | | | | | | |

Fixed PTMs: Carbamidomethylation [C53 C60 C67 C68 C77 C79 C93 C95 ]

  

All peaks (127)  Matched peaks (48)  Not matched peaks (79)

  

| Scan | Peak | Mono mass | Mono m/z | Intensity | Charge | Theoretical mass | Ion | Pos | Mass error | PPM error |
| --- | --- | --- | --- | --- | --- | --- | --- | --- | --- | --- |
| 535 | 1 | 7614.6486 | 847.0793 | 40469.63 | 9 |  |  |  |  |  |
| 535 | 2 | 7614.6519 | 762.4725 | 35361.60 | 10 |  |  |  |  |  |
| 535 | 3 | 2450.4903 | 613.6298 | 30027.49 | 4 |  |  |  |  |  |
| 535 | 4 | 7615.6592 | 952.9647 | 22387.48 | 8 |  |  |  |  |  |
| 535 | 5 | 2187.1382 | 730.0533 | 24764.20 | 3 |  |  |  |  |  |
| 535 | 6 | 3263.6109 | 816.9100 | 15834.97 | 4 |  |  |  |  |  |
| 535 | 7 | 7557.6309 | 945.7111 | 20516.87 | 8 |  |  |  |  |  |
| 535 | 8 | 3226.5034 | 646.3080 | 17268.19 | 5 | 3226.5216 | C26 | 26 | -0.0182 | -5.63 |
| 535 | 9 | 2008.8735 | 670.6318 | 25116.63 | 3 | 2008.8843 | C16 | 16 | -0.0108 | -5.37 |
| 535 | 10 | 3836.8371 | 768.3747 | 14432.40 | 5 |  |  |  |  |  |
| 535 | 11 | 7557.6244 | 840.7433 | 20771.99 | 9 |  |  |  |  |  |
| 535 | 12 | 1893.8477 | 632.2899 | 19656.31 | 3 | 1893.8573 | C15 | 15 | -9.62e-03 | -5.08 |
| 535 | 13 | 3485.6418 | 698.1356 | 13849.79 | 5 | 3485.6570 | C28 | 28 | -0.0152 | -4.37 |
| 535 | 14 | 3077.5467 | 770.3939 | 15167.56 | 4 |  |  |  |  |  |
| 535 | 15 | 1779.8033 | 890.9089 | 18399.03 | 2 | 1779.8144 | C14 | 14 | -0.0111 | -6.23 |
| 535 | 16 | 3354.5981 | 671.9269 | 14303.31 | 5 | 3354.6165 | C27 | 27 | -0.0184 | -5.49 |
| 535 | 17 | 1683.9848 | 842.9997 | 16835.62 | 2 |  |  |  |  |  |
| 535 | 18 | 2379.1039 | 794.0419 | 12831.22 | 3 | 2379.1171 | C19 | 19 | -0.0132 | -5.54 |
| 535 | 19 | 3773.7586 | 629.9670 | 10416.02 | 6 | 3773.7826 | C30 | 30 | -0.0240 | -6.37 |
| 535 | 20 | 7614.6465 | 693.2479 | 16998.40 | 11 |  |  |  |  |  |
| 535 | 21 | 3901.8537 | 651.3162 | 10681.17 | 6 | 3901.8776 | C31 | 31 | -0.0239 | -6.12 |
| 535 | 22 | 2649.3149 | 884.1122 | 12240.74 | 3 |  |  |  |  |  |
| 535 | 23 | 7558.6323 | 1080.8119 | 10145.49 | 7 |  |  |  |  |  |
| 535 | 24 | 4595.1322 | 657.4547 | 10931.12 | 7 | 4595.1568 | C37 | 37 | -0.0246 | -5.36 |
| 535 | 25 | 1596.9538 | 799.4842 | 17987.91 | 2 |  |  |  |  |  |
| 535 | 26 | 3680.8246 | 921.2134 | 10505.79 | 4 |  |  |  |  |  |
| 535 | 27 | 7555.6137 | 756.5686 | 8227.11 | 10 |  |  |  |  |  |
| 535 | 28 | 7571.6373 | 947.4619 | 11769.55 | 8 |  |  |  |  |  |
| 535 | 29 | 1389.6863 | 695.8504 | 9466.32 | 2 | 1389.6935 | C11 | 11 | -7.17e-03 | -5.16 |
| 535 | 30 | 7598.6305 | 845.2996 | 9155.85 | 9 |  |  |  |  |  |
| 535 | 31 | 1893.8471 | 947.9308 | 10712.99 | 2 | 1893.8573 | C15 | 15 | -0.0102 | -5.39 |
| 535 | 32 | 5779.8370 | 826.6983 | 8052.09 | 7 | 5779.8499 | Z\_DOT50 | 15 | -0.0129 | -2.24 |
| 535 | 33 | 3680.8261 | 737.1725 | 7690.99 | 5 |  |  |  |  |  |
| 535 | 34 | 4481.1094 | 747.8588 | 8109.75 | 6 |  |  |  |  |  |
| 535 | 35 | 7629.6619 | 848.7475 | 10017.16 | 9 |  |  |  |  |  |
| 535 | 36 | 2750.3602 | 917.7940 | 10022.09 | 3 |  |  |  |  |  |
| 535 | 37 | 7572.6341 | 842.4111 | 9345.85 | 9 |  |  |  |  |  |
| 535 | 38 | 3898.9250 | 975.7385 | 9616.40 | 4 |  |  |  |  |  |
| 535 | 39 | 2187.1384 | 1094.5765 | 10069.73 | 2 |  |  |  |  |  |
| 535 | 40 | 3192.5762 | 799.1513 | 9140.19 | 4 |  |  |  |  |  |
| 535 | 41 | 4717.3330 | 787.2294 | 5261.72 | 6 |  |  |  |  |  |
| 535 | 42 | 1553.8997 | 777.9571 | 8697.05 | 2 |  |  |  |  |  |
| 535 | 43 | 1518.7288 | 760.3717 | 10401.76 | 2 | 1518.7361 | C12 | 12 | -7.28e-03 | -4.79 |
| 535 | 44 | 2265.0611 | 756.0276 | 12640.65 | 3 | 2265.0742 | C18 | 18 | -0.0131 | -5.78 |
| 535 | 45 | 4661.3104 | 933.2694 | 7239.70 | 5 | 4661.3175 | Z\_DOT41 | 24 | -7.03e-03 | -1.51 |
| 535 | 46 | 4447.1785 | 890.4430 | 9511.81 | 5 | 4447.1857 | Z\_DOT39 | 26 | -7.19e-03 | -1.62 |
| 535 | 47 | 4410.0676 | 631.0169 | 5663.75 | 7 |  |  |  |  |  |
| 535 | 48 | 3463.7262 | 866.9388 | 6251.16 | 4 |  |  |  |  |  |
| 535 | 49 | 5663.8044 | 708.9828 | 4815.61 | 8 | 5664.8230 | Z\_DOT49 | 16 | -0.0163 | -2.88 |
| 535 | 50 | 4718.3293 | 944.6731 | 8748.68 | 5 | 4718.3389 | Z\_DOT42 | 23 | -9.60e-03 | -2.03 |
| 535 | 51 | 5023.3689 | 718.6314 | 6574.71 | 7 | 5023.3951 | C42 | 42 | -0.0262 | -5.22 |
| 535 | 52 | 5395.5361 | 771.7981 | 5729.99 | 7 | 5395.5708 | C45 | 45 | -0.0348 | -6.44 |
| 535 | 53 | 3148.5830 | 788.1530 | 6978.57 | 4 |  |  |  |  |  |
| 535 | 54 | 3227.5054 | 807.8836 | 7298.41 | 4 |  |  |  |  |  |
| 535 | 55 | 638.4450 | 639.4523 | 10923.62 | 1 |  |  |  |  |  |
| 535 | 56 | 2955.3523 | 592.0777 | 10196.34 | 5 | 2955.3684 | C23 | 23 | -0.0161 | -5.45 |
| 535 | 57 | 5203.5745 | 868.2697 | 5471.25 | 6 |  |  |  |  |  |
| 535 | 58 | 5396.5324 | 675.5738 | 4597.90 | 8 | 5395.5708 | C45 | 45 | -0.0407 | -7.55 |
| 535 | 59 | 2955.3514 | 739.8451 | 6695.51 | 4 | 2955.3684 | C23 | 23 | -0.0170 | -5.75 |
| 535 | 60 | 2539.1352 | 847.3857 | 6489.45 | 3 | 2539.1478 | C20 | 20 | -0.0126 | -4.96 |
| 535 | 61 | 2821.3978 | 941.4732 | 5945.99 | 3 |  |  |  |  |  |
| 535 | 62 | 7486.5592 | 936.8272 | 6444.16 | 8 |  |  |  |  |  |
| 535 | 63 | 7599.6490 | 950.9634 | 6822.90 | 8 |  |  |  |  |  |
| 535 | 64 | 2008.8748 | 1005.4447 | 6212.90 | 2 | 2008.8843 | C16 | 16 | -9.43e-03 | -4.70 |
| 535 | 65 | 4794.2589 | 800.0504 | 5369.51 | 6 | 4794.2889 | C39 | 39 | -0.0300 | -6.25 |
| 535 | 66 | 5893.8920 | 842.9919 | 6651.09 | 7 | 5893.8929 | Z\_DOT51 | 14 | -8.29e-04 | -0.14 |
| 535 | 67 | 3354.6000 | 839.6573 | 6616.11 | 4 | 3354.6165 | C27 | 27 | -0.0165 | -4.92 |
| 535 | 68 | 6018.6971 | 860.8211 | 6267.63 | 7 |  |  |  |  |  |
| 535 | 69 | 4595.1281 | 766.8620 | 5035.24 | 6 | 4595.1568 | C37 | 37 | -0.0287 | -6.25 |
| 535 | 70 | 3902.8506 | 781.5774 | 4379.67 | 5 |  |  |  |  |  |
| 535 | 71 | 3968.9474 | 794.7968 | 6795.04 | 5 |  |  |  |  |  |
| 535 | 72 | 3968.9477 | 993.2442 | 6228.97 | 4 |  |  |  |  |  |
| 535 | 73 | 3715.7389 | 744.1551 | 5403.96 | 5 |  |  |  |  |  |
| 535 | 74 | 3078.5575 | 1027.1931 | 6645.84 | 3 | 3078.5505 | Z\_DOT28 | 37 | 7.00e-03 | 2.27 |
| 535 | 75 | 4061.8802 | 677.9873 | 4782.75 | 6 | 4061.9082 | C32 | 32 | -0.0281 | -6.91 |
| 535 | 76 | 3836.3338 | 640.3962 | 7311.53 | 6 |  |  |  |  |  |
| 535 | 77 | 4538.1046 | 757.3580 | 5628.18 | 6 |  |  |  |  |  |
| 535 | 78 | 4922.3198 | 821.3939 | 4710.27 | 6 | 4922.3474 | C41 | 41 | -0.0276 | -5.60 |
| 535 | 79 | 6075.7262 | 760.4730 | 5893.34 | 8 | 6075.7603 | C51 | 51 | -0.0341 | -5.61 |
| 535 | 80 | 3193.5829 | 1065.5349 | 6795.36 | 3 | 3193.5774 | Z\_DOT29 | 36 | 5.44e-03 | 1.70 |
| 535 | 81 | 1920.1911 | 641.0710 | 12024.46 | 3 |  |  |  |  |  |
| 535 | 82 | 1612.9727 | 807.4936 | 6559.02 | 2 |  |  |  |  |  |
| 535 | 83 | 4318.0796 | 864.6232 | 6073.86 | 5 |  |  |  |  |  |
| 535 | 84 | 5146.5482 | 858.7653 | 4142.50 | 6 |  |  |  |  |  |
| 535 | 85 | 617.3104 | 618.3176 | 10124.98 | 1 | 617.3132 | C5 | 5 | -2.88e-03 | -4.66 |
| 535 | 86 | 6018.6999 | 753.3448 | 4489.41 | 8 |  |  |  |  |  |
| 535 | 87 | 4203.0565 | 841.6186 | 5771.75 | 5 |  |  |  |  |  |
| 535 | 88 | 4423.0863 | 885.6245 | 5058.58 | 5 |  |  |  |  |  |
| 535 | 89 | 4187.0461 | 838.4165 | 4611.98 | 5 |  |  |  |  |  |
| 535 | 90 | 4061.8865 | 813.3846 | 3266.64 | 5 | 4061.9082 | C32 | 32 | -0.0217 | -5.35 |
| 535 | 91 | 7541.6042 | 943.7078 | 7069.05 | 8 |  |  |  |  |  |
| 535 | 92 | 3614.7357 | 723.9544 | 4857.92 | 5 |  |  |  |  |  |
| 535 | 93 | 7414.5275 | 927.8232 | 3504.20 | 8 | 7413.5589 | Z\_DOT63 | 2 | -0.0338 | -4.56 |
| 535 | 94 | 5043.5451 | 841.5981 | 4857.32 | 6 |  |  |  |  |  |
| 535 | 95 | 7616.6558 | 1089.1010 | 5911.49 | 7 |  |  |  |  |  |
| 535 | 96 | 5023.3735 | 838.2362 | 5175.86 | 6 | 5023.3951 | C42 | 42 | -0.0216 | -4.30 |
| 535 | 97 | 2558.5629 | 853.8616 | 6940.50 | 3 |  |  |  |  |  |
| 535 | 98 | 4446.1839 | 742.0379 | 5248.24 | 6 |  |  |  |  |  |
| 535 | 99 | 3006.5184 | 752.6369 | 3660.99 | 4 |  |  |  |  |  |
| 535 | 100 | 3441.6252 | 689.3323 | 4469.38 | 5 |  |  |  |  |  |
| 535 | 101 | 2879.4270 | 960.8163 | 4736.88 | 3 | 2879.4184 | Z\_DOT26 | 39 | 8.57e-03 | 2.97 |
| 535 | 102 | 6394.3964 | 640.4469 | 9563.08 | 10 |  |  |  |  |  |
| 535 | 103 | 3012.3710 | 603.4815 | 3707.39 | 5 | 3012.3898 | C24 | 24 | -0.0188 | -6.24 |
| 535 | 104 | 7572.6358 | 1082.8124 | 4383.04 | 7 |  |  |  |  |  |
| 535 | 105 | 2136.9667 | 713.3295 | 3524.61 | 3 | 2136.9792 | C17 | 17 | -0.0125 | -5.85 |
| 535 | 106 | 763.4787 | 764.4860 | 5497.93 | 1 |  |  |  |  |  |
| 535 | 107 | 1252.6282 | 627.3214 | 4370.53 | 2 | 1252.6346 | C10 | 10 | -6.37e-03 | -5.08 |
| 535 | 108 | 503.2681 | 504.2754 | 5873.97 | 1 | 503.2703 | C4 | 4 | -2.16e-03 | -4.28 |
| 535 | 109 | 326.2305 | 327.2378 | 3071.99 | 1 |  |  |  |  |  |
| 535 | 110 | 777.3399 | 778.3472 | 2429.38 | 1 | 777.3439 | C6 | 6 | -3.99e-03 | -5.14 |
| 535 | 111 | 1115.5700 | 558.7923 | 3500.51 | 2 | 1115.5757 | C9 | 9 | -5.68e-03 | -5.09 |
| 535 | 112 | 1071.5568 | 536.7857 | 1609.96 | 2 |  |  |  |  |  |
| 535 | 113 | 891.4049 | 892.4122 | 2319.87 | 1 |  |  |  |  |  |
| 535 | 114 | 959.9626 | 960.9699 | 1729.24 | 1 |  |  |  |  |  |
| 535 | 115 | 375.1739 | 376.1812 | 2817.02 | 1 | 375.1753 | C3 | 3 | -1.43e-03 | -3.82 |
| 535 | 116 | 910.5445 | 911.5518 | 1408.11 | 1 |  |  |  |  |  |
| 535 | 117 | 1115.6409 | 1116.6481 | 1186.49 | 1 |  |  |  |  |  |
| 535 | 118 | 1476.1692 | 739.0919 | 800.24 | 2 |  |  |  |  |  |
| 535 | 119 | 662.4321 | 663.4393 | 1678.05 | 1 |  |  |  |  |  |
| 535 | 120 | 1071.6586 | 1072.6658 | 815.87 | 1 |  |  |  |  |  |
| 535 | 121 | 1243.7363 | 1244.7436 | 839.70 | 1 |  |  |  |  |  |
| 535 | 122 | 1325.1588 | 1326.1661 | 869.89 | 1 |  |  |  |  |  |
| 535 | 123 | 1419.3554 | 710.6850 | 1038.21 | 2 |  |  |  |  |  |
| 535 | 124 | 1012.4858 | 507.2502 | 961.92 | 2 |  |  |  |  |  |
| 535 | 125 | 1389.6854 | 464.2357 | 2079.17 | 3 | 1389.6935 | C11 | 11 | -8.07e-03 | -5.81 |
| 535 | 126 | 981.5563 | 491.7854 | 1076.58 | 2 |  |  |  |  |  |
| 535 | 127 | 1023.4846 | 1024.4919 | 793.78 | 1 |  |  |  |  |  |

  

All proteins /
CsTx-9a Cupiennius salei toxin 9 isoform a /
Proteoform #10
