# Supplementary material for: The Dual Prey-Inactivation Strategy of Spiders—In-Depth Venomic Analysis of Cupiennius salei
Source: Toxins (Basel). 2019 Mar 19;11(3):167. doi: 10.3390/toxins11030167 (PMC6468893; doi:10.3390/toxins11030167)
Supplement: Supplementary file 1 [file toxins-11-00167-s001.zip › Supplementary Dataset EV1/20180328_f2_topdown_OTMS2_EThcD_NL_i02_ms2_proteoform_cutoff_html/prsms/prsm161.html]

Protein-Spectrum-Match for Spectrum #400


All proteins /
CsTx-9a Cupiennius salei toxin 9 isoform a /
Proteoform #10

## Protein-Spectrum-Match #161 for Spectrum #400

|  |  |  |  |  |  |
| --- | --- | --- | --- | --- | --- |
| PrSM ID: | 161 | Scan(s): | 536 | Precursor charge: | 11 |
| Precursor m/z: | 698.4349 | Precursor mass: | 7671.7044 | Proteoform mass: | 7671.6944 |
| # matched peaks: | 60 | # matched fragment ions: | 47 | # unexpected modifications: | 1 |
| E-value: | 4.07e-39 | P-value: | 4.07e-39 | Q-value (Spectral FDR): | 0 |

  

|  |  |  |  |  |  |  |  |  |  |  |  |  |  |  |  |  |  |  |  |  |  |  |  |  |  |  |  |  |  |  |  |  |  |  |  |  |  |  |  |  |  |  |  |  |  |  |  |  |  |  |  |  |  |  |  |  |  |  |  |  |  |  |  |  |  |  |  |  |  |
| --- | --- | --- | --- | --- | --- | --- | --- | --- | --- | --- | --- | --- | --- | --- | --- | --- | --- | --- | --- | --- | --- | --- | --- | --- | --- | --- | --- | --- | --- | --- | --- | --- | --- | --- | --- | --- | --- | --- | --- | --- | --- | --- | --- | --- | --- | --- | --- | --- | --- | --- | --- | --- | --- | --- | --- | --- | --- | --- | --- | --- | --- | --- | --- | --- | --- | --- | --- | --- | --- |
|  | |  | | | | | | | | | | | | | | | | | | | | | | | | | | | | | | | | | | | | | | | | | | | | | | | | | | | | | | | | | | | | | | | | | | | |
| 1 |  |  | M |  | K |  | V |  | L |  | V |  | I |  | C |  | A |  | V |  | L |  |  | F |  | L |  | A |  | I |  | F |  | S |  | N |  | S |  | S |  | A |  |  | E |  | T |  | E |  | D |  | D |  | F |  | L |  | E |  | D |  | E |  | 30 |  |
|  | |  | | | | | | | | | | | | | | | | | | | | | | | | | | | | | | | | | | | | | | | | | | | | | | | | | | | | | | | | | | | | | | | | | | | |
| 31 |  |  | S |  | F |  | E |  | A |  | D |  | D |  | V |  | I |  | P |  | F |  |  | L |  | A |  | R |  | E |  | Q |  | V |  | R | ] | K |  | D |  | D |  |  | K | ⎫ | N | ⎫ | C | ⎫ | I |  | P |  | K | ⎫ | H |  | H | ⎫ | E | ⎫ | C |  | 60 |  |
|  | |  | | | | | | | | | | | | | | | | | | | | | | | | | | | | | | | | | | | | | | | | | | | | | | | | | | | | | | | | | | | | | | | | | | | |
| 61 |  |  | T | ⎱ | N | ⎱ | D | ⎱ | K |  | K | ⎱ | N | ⎫ | C | ⎫ | C |  | K | ⎩ | K |  | ⎱ | G | ⎩ | L |  | T | ⎱ | K | ⎱ | M | ⎩ | K | ⎫ | C | ⎱ | K | ⎫ | C | ⎱ | F |  | ⎩ | T |  | V |  | A | ⎱ | D |  | A |  | K | ⎱ | G | ⎱ | A | ⎱ | T | ⎱ | S |  | 90 |  |
|  | |  | | | | | | | | | | | | | | | | | | | | | | | | | | | | | | | | | | | | | | | | | | | | | -318.18 | | | | | | | | | | | | | | | | | | | |
| 91 |  |  | E |  | R | ⎫ | C |  | A |  | C |  | D |  | S | ⎩ | S | ⎱ | L |  | L |  |  | Q |  | K |  | F |  | G |  | F |  | T |  | G |  | L |  | H |  | I |  |  | I | ⎫ | K |  | G |  | L |  | F |  | | 115 |  | | | | | | | | | |

Fixed PTMs: Carbamidomethylation [C53 C60 C67 C68 C77 C79 C93 C95 ]   
  
     Unexpected modifications:   Unknown [-318.18]

  

All peaks (127)  Matched peaks (60)  Not matched peaks (67)

  

| Scan | Peak | Mono mass | Mono m/z | Intensity | Charge | Theoretical mass | Ion | Pos | Mass error | PPM error |
| --- | --- | --- | --- | --- | --- | --- | --- | --- | --- | --- |
| 536 | 1 | 7614.6464 | 847.0791 | 58009.02 | 9 |  |  |  |  |  |
| 536 | 2 | 3491.8136 | 699.3700 | 62041.13 | 5 |  |  |  |  |  |
| 536 | 3 | 7615.6465 | 952.9631 | 42551.68 | 8 |  |  |  |  |  |
| 536 | 4 | 7614.6421 | 762.4715 | 24050.10 | 10 |  |  |  |  |  |
| 536 | 5 | 7557.6251 | 945.7104 | 22797.18 | 8 |  |  |  |  |  |
| 536 | 6 | 7615.6534 | 1088.9578 | 15861.32 | 7 |  |  |  |  |  |
| 536 | 7 | 7556.6243 | 840.6322 | 15726.52 | 9 |  |  |  |  |  |
| 536 | 8 | 2187.1387 | 730.0535 | 22472.93 | 3 |  |  |  |  |  |
| 536 | 9 | 3680.8224 | 921.2129 | 18690.91 | 4 |  |  |  |  |  |
| 536 | 10 | 7627.6531 | 848.5243 | 11393.52 | 9 |  |  |  |  |  |
| 536 | 11 | 3836.3366 | 768.2746 | 20873.57 | 5 |  |  |  |  |  |
| 536 | 12 | 7557.6275 | 1080.6683 | 15858.58 | 7 |  |  |  |  |  |
| 536 | 13 | 3491.8180 | 873.9618 | 12621.39 | 4 |  |  |  |  |  |
| 536 | 14 | 5395.5399 | 771.7987 | 10317.89 | 7 | 5395.5708 | C45 | 45 | -0.0309 | -5.73 |
| 536 | 15 | 3354.5960 | 671.9265 | 11177.34 | 5 | 3354.6165 | C27 | 27 | -0.0206 | -6.13 |
| 536 | 16 | 2379.1034 | 794.0417 | 12691.99 | 3 | 2379.1171 | C19 | 19 | -0.0137 | -5.77 |
| 536 | 17 | 7570.6305 | 947.3361 | 13151.79 | 8 |  |  |  |  |  |
| 536 | 18 | 1779.8025 | 890.9085 | 15190.02 | 2 | 1779.8144 | C14 | 14 | -0.0120 | -6.71 |
| 536 | 19 | 7598.6343 | 950.8366 | 9543.56 | 8 |  |  |  |  |  |
| 536 | 20 | 3898.9180 | 975.7368 | 10203.06 | 4 | 3898.9196 | Z\_DOT38 | 30 | -1.54e-03 | -0.39 |
| 536 | 21 | 3901.8515 | 651.3159 | 8914.66 | 6 | 3901.8776 | C31 | 31 | -0.0261 | -6.69 |
| 536 | 22 | 2008.8728 | 670.6315 | 13575.01 | 3 | 2008.8843 | C16 | 16 | -0.0115 | -5.73 |
| 536 | 23 | 1596.9535 | 799.4840 | 12636.05 | 2 | 1596.9419 | Z\_DOT17 | 51 | 0.0116 | 7.25 |
| 536 | 24 | 7656.6685 | 851.7482 | 8524.71 | 9 |  |  |  |  |  |
| 536 | 25 | 1683.9864 | 843.0005 | 10690.40 | 2 | 1683.9740 | Z\_DOT18 | 50 | 0.0124 | 7.36 |
| 536 | 26 | 5395.5382 | 900.2636 | 8787.51 | 6 | 5395.5708 | C45 | 45 | -0.0326 | -6.05 |
| 536 | 27 | 2187.1389 | 1094.5767 | 13321.44 | 2 |  |  |  |  |  |
| 536 | 28 | 3192.5732 | 1065.1984 | 8408.05 | 3 | 3192.5724 | Z\_DOT32 | 36 | 8.81e-04 | 0.28 |
| 536 | 29 | 2750.3594 | 917.7938 | 8464.31 | 3 | 2750.3548 | Z\_DOT27 | 41 | 4.67e-03 | 1.70 |
| 536 | 30 | 7629.6583 | 954.7146 | 10221.98 | 8 |  |  |  |  |  |
| 536 | 31 | 1518.7277 | 760.3711 | 9453.65 | 2 | 1518.7361 | C12 | 12 | -8.38e-03 | -5.52 |
| 536 | 32 | 4446.1731 | 890.2419 | 9901.57 | 5 | 4446.1806 | Z\_DOT42 | 26 | -7.51e-03 | -1.69 |
| 536 | 33 | 697.2442 | 698.2515 | 32218.76 | 1 |  |  |  |  |  |
| 536 | 34 | 3968.9425 | 993.2429 | 9433.82 | 4 |  |  |  |  |  |
| 536 | 35 | 3226.5025 | 646.3078 | 8201.31 | 5 | 3226.5216 | C26 | 26 | -0.0191 | -5.92 |
| 536 | 36 | 4794.2534 | 800.0495 | 8003.21 | 6 | 4794.2889 | C39 | 39 | -0.0354 | -7.39 |
| 536 | 37 | 2649.3119 | 884.1113 | 7853.24 | 3 | 2649.3071 | Z\_DOT26 | 42 | 4.85e-03 | 1.83 |
| 536 | 38 | 5893.8750 | 842.9894 | 6200.10 | 7 | 5892.8878 | Z\_DOT54 | 14 | -0.0152 | -2.57 |
| 536 | 39 | 3226.5030 | 807.6330 | 7176.43 | 4 | 3226.5216 | C26 | 26 | -0.0186 | -5.76 |
| 536 | 40 | 3836.3405 | 960.0924 | 10184.02 | 4 |  |  |  |  |  |
| 536 | 41 | 2558.5600 | 853.8606 | 15112.05 | 3 |  |  |  |  |  |
| 536 | 42 | 5204.5770 | 1041.9227 | 5871.45 | 5 |  |  |  |  |  |
| 536 | 43 | 1892.8441 | 631.9553 | 10388.49 | 3 |  |  |  |  |  |
| 536 | 44 | 2539.1323 | 847.3847 | 7935.18 | 3 | 2539.1478 | C20 | 20 | -0.0155 | -6.11 |
| 536 | 45 | 1893.8477 | 947.9311 | 10045.64 | 2 | 1893.8573 | C15 | 15 | -9.60e-03 | -5.07 |
| 536 | 46 | 7499.5934 | 938.4564 | 5807.62 | 8 |  |  |  |  |  |
| 536 | 47 | 3422.8108 | 856.7100 | 16626.58 | 4 |  |  |  |  |  |
| 536 | 48 | 1553.8998 | 777.9572 | 8056.11 | 2 |  |  |  |  |  |
| 536 | 49 | 7570.6431 | 1082.5277 | 7370.30 | 7 |  |  |  |  |  |
| 536 | 50 | 5204.5776 | 868.4369 | 7905.76 | 6 |  |  |  |  |  |
| 536 | 51 | 4480.0989 | 897.0271 | 5513.68 | 5 | 4480.1298 | C36 | 36 | -0.0309 | -6.90 |
| 536 | 52 | 7570.6302 | 842.1884 | 7062.59 | 9 |  |  |  |  |  |
| 536 | 53 | 5778.8334 | 826.5549 | 6095.98 | 7 | 5778.8449 | Z\_DOT53 | 15 | -0.0114 | -1.98 |
| 536 | 54 | 4318.0849 | 864.6243 | 6922.51 | 5 | 4318.0857 | Z\_DOT41 | 27 | -7.64e-04 | -0.18 |
| 536 | 55 | 5407.6173 | 902.2768 | 4729.15 | 6 | 5407.6280 | Z\_DOT50 | 18 | -0.0107 | -1.98 |
| 536 | 56 | 3354.5995 | 839.6572 | 7243.02 | 4 | 3354.6165 | C27 | 27 | -0.0170 | -5.07 |
| 536 | 57 | 4319.0839 | 1080.7783 | 9061.73 | 4 |  |  |  |  |  |
| 536 | 58 | 5043.5492 | 1009.7171 | 5105.63 | 5 |  |  |  |  |  |
| 536 | 59 | 2008.8734 | 1005.4440 | 6208.47 | 2 | 2008.8843 | C16 | 16 | -0.0109 | -5.42 |
| 536 | 60 | 2692.3282 | 898.4500 | 5634.54 | 3 |  |  |  |  |  |
| 536 | 61 | 4922.3198 | 821.3939 | 8963.13 | 6 | 4922.3474 | C41 | 41 | -0.0276 | -5.60 |
| 536 | 62 | 2955.3509 | 739.8450 | 6262.94 | 4 | 2955.3684 | C23 | 23 | -0.0175 | -5.92 |
| 536 | 63 | 7657.6695 | 958.2160 | 7137.08 | 8 |  |  |  |  |  |
| 536 | 64 | 1389.6867 | 695.8506 | 11037.33 | 2 | 1389.6935 | C11 | 11 | -6.80e-03 | -4.90 |
| 536 | 65 | 2821.3956 | 941.4725 | 6836.23 | 3 | 2821.3919 | Z\_DOT28 | 40 | 3.69e-03 | 1.31 |
| 536 | 66 | 5663.8064 | 810.1225 | 8373.00 | 7 | 5663.8179 | Z\_DOT52 | 16 | -0.0115 | -2.04 |
| 536 | 67 | 7597.6295 | 845.1883 | 4756.65 | 9 |  |  |  |  |  |
| 536 | 68 | 617.3102 | 618.3175 | 8870.47 | 1 | 617.3132 | C5 | 5 | -3.00e-03 | -4.86 |
| 536 | 69 | 5663.8016 | 944.9742 | 5782.37 | 6 | 5663.8179 | Z\_DOT52 | 16 | -0.0163 | -2.88 |
| 536 | 70 | 4922.3128 | 704.1948 | 5005.32 | 7 | 4922.3474 | C41 | 41 | -0.0346 | -7.03 |
| 536 | 71 | 4203.0572 | 1051.7716 | 6697.87 | 4 |  |  |  |  |  |
| 536 | 72 | 4187.0454 | 1047.7686 | 4692.13 | 4 | 4187.0452 | Z\_DOT40 | 28 | 2.54e-04 | 0.06 |
| 536 | 73 | 5893.8772 | 983.3201 | 5295.64 | 6 | 5892.8878 | Z\_DOT54 | 14 | -0.0129 | -2.19 |
| 536 | 74 | 2265.0604 | 756.0274 | 8277.59 | 3 | 2265.0742 | C18 | 18 | -0.0138 | -6.10 |
| 536 | 75 | 3192.5760 | 799.1513 | 3940.99 | 4 | 3192.5724 | Z\_DOT32 | 36 | 3.62e-03 | 1.13 |
| 536 | 76 | 6075.7207 | 868.9674 | 5141.97 | 7 | 6075.7603 | C51 | 51 | -0.0396 | -6.52 |
| 536 | 77 | 3773.7590 | 629.9671 | 5719.22 | 6 | 3773.7826 | C30 | 30 | -0.0237 | -6.27 |
| 536 | 78 | 4480.1016 | 747.6909 | 7350.16 | 6 | 4480.1298 | C36 | 36 | -0.0283 | -6.31 |
| 536 | 79 | 3613.7283 | 723.7529 | 3652.49 | 5 | 3613.7520 | C29 | 29 | -0.0237 | -6.55 |
| 536 | 80 | 7558.6401 | 1260.7806 | 4333.22 | 6 |  |  |  |  |  |
| 536 | 81 | 6018.6987 | 860.8214 | 5567.15 | 7 |  |  |  |  |  |
| 536 | 82 | 1612.9726 | 807.4936 | 6093.91 | 2 |  |  |  |  |  |
| 536 | 83 | 7627.6446 | 763.7717 | 5301.87 | 10 |  |  |  |  |  |
| 536 | 84 | 3463.7220 | 866.9378 | 5019.19 | 4 | 3463.7256 | Z\_DOT35 | 33 | -3.51e-03 | -1.01 |
| 536 | 85 | 4793.2549 | 959.6582 | 7938.58 | 5 |  |  |  |  |  |
| 536 | 86 | 5043.5407 | 841.5974 | 6246.36 | 6 |  |  |  |  |  |
| 536 | 87 | 1258.7484 | 630.3815 | 4629.45 | 2 |  |  |  |  |  |
| 536 | 88 | 5146.5546 | 1030.3182 | 5673.11 | 5 |  |  |  |  |  |
| 536 | 89 | 7542.6012 | 1078.5217 | 4666.58 | 7 | 7543.6204 | C64 | 64 | -0.0168 | -2.23 |
| 536 | 90 | 4660.3003 | 933.0673 | 4524.80 | 5 | 4660.3124 | Z\_DOT44 | 24 | -0.0121 | -2.59 |
| 536 | 91 | 6573.1338 | 822.6490 | 3501.07 | 8 |  |  |  |  |  |
| 536 | 92 | 7616.6699 | 1270.4523 | 4012.20 | 6 |  |  |  |  |  |
| 536 | 93 | 4061.8841 | 813.3841 | 3454.86 | 5 | 4061.9082 | C32 | 32 | -0.0242 | -5.95 |
| 536 | 94 | 5778.8317 | 964.1459 | 7920.31 | 6 | 5778.8449 | Z\_DOT53 | 15 | -0.0131 | -2.27 |
| 536 | 95 | 3455.7883 | 692.1649 | 5871.47 | 5 |  |  |  |  |  |
| 536 | 96 | 4851.2853 | 694.0480 | 3601.41 | 7 | 4851.3103 | C40 | 40 | -0.0250 | -5.15 |
| 536 | 97 | 3148.5862 | 788.1538 | 2785.97 | 4 |  |  |  |  |  |
| 536 | 98 | 4852.2827 | 809.7211 | 3356.93 | 6 |  |  |  |  |  |
| 536 | 99 | 3901.8563 | 781.3785 | 5921.53 | 5 | 3901.8776 | C31 | 31 | -0.0213 | -5.45 |
| 536 | 100 | 7599.6480 | 1086.6713 | 6434.71 | 7 |  |  |  |  |  |
| 536 | 101 | 3434.7963 | 859.7063 | 7676.67 | 4 |  |  |  |  |  |
| 536 | 102 | 5023.3676 | 838.2352 | 4602.95 | 6 | 5023.3951 | C42 | 42 | -0.0275 | -5.47 |
| 536 | 103 | 2878.4168 | 960.4795 | 7676.59 | 3 | 2878.4133 | Z\_DOT29 | 39 | 3.43e-03 | 1.19 |
| 536 | 104 | 763.4814 | 764.4887 | 4188.24 | 1 |  |  |  |  |  |
| 536 | 105 | 4717.3216 | 944.4716 | 4536.38 | 5 | 4717.3338 | Z\_DOT45 | 23 | -0.0122 | -2.59 |
| 536 | 106 | 4845.4166 | 970.0906 | 3411.27 | 5 | 4845.4288 | Z\_DOT46 | 22 | -0.0122 | -2.51 |
| 536 | 107 | 3610.7945 | 903.7059 | 4075.52 | 4 | 3610.7940 | Z\_DOT36 | 32 | 5.63e-04 | 0.16 |
| 536 | 108 | 294.0949 | 295.1021 | 3901.43 | 1 |  |  |  |  |  |
| 536 | 109 | 368.1132 | 369.1205 | 4289.00 | 1 |  |  |  |  |  |
| 536 | 110 | 220.0765 | 221.0837 | 3636.96 | 1 |  |  |  |  |  |
| 536 | 111 | 1115.5695 | 558.7920 | 2916.86 | 2 | 1115.5757 | C9 | 9 | -6.17e-03 | -5.53 |
| 536 | 112 | 503.2680 | 504.2753 | 4535.99 | 1 | 503.2703 | C4 | 4 | -2.28e-03 | -4.53 |
| 536 | 113 | 326.2305 | 327.2377 | 1538.60 | 1 |  |  |  |  |  |
| 536 | 114 | 777.3396 | 778.3469 | 1642.54 | 1 | 777.3439 | C6 | 6 | -4.24e-03 | -5.45 |
| 536 | 115 | 680.2178 | 681.2251 | 2773.59 | 1 |  |  |  |  |  |
| 536 | 116 | 1115.5697 | 1116.5770 | 1154.79 | 1 | 1115.5757 | C9 | 9 | -5.98e-03 | -5.36 |
| 536 | 117 | 542.3020 | 543.3093 | 1710.19 | 1 |  |  |  |  |  |
| 536 | 118 | 1062.6542 | 1063.6614 | 1196.12 | 1 |  |  |  |  |  |
| 536 | 119 | 1491.7486 | 746.8816 | 775.76 | 2 |  |  |  |  |  |
| 536 | 120 | 1071.5568 | 536.7857 | 945.31 | 2 |  |  |  |  |  |
| 536 | 121 | 662.4317 | 663.4390 | 1487.30 | 1 |  |  |  |  |  |
| 536 | 122 | 1389.6862 | 464.2360 | 578.47 | 3 | 1389.6935 | C11 | 11 | -7.24e-03 | -5.21 |
| 536 | 123 | 1220.6816 | 611.3481 | 693.26 | 2 |  |  |  |  |  |
| 536 | 124 | 931.2942 | 932.3015 | 395.61 | 1 |  |  |  |  |  |
| 536 | 125 | 1371.7921 | 686.9034 | 701.02 | 2 |  |  |  |  |  |
| 536 | 126 | 1204.6629 | 603.3387 | 543.12 | 2 |  |  |  |  |  |
| 536 | 127 | 976.0629 | 977.0701 | 645.54 | 1 |  |  |  |  |  |

  

All proteins /
CsTx-9a Cupiennius salei toxin 9 isoform a /
Proteoform #10
