# Supplementary material for: The Dual Prey-Inactivation Strategy of Spiders—In-Depth Venomic Analysis of Cupiennius salei
Source: Toxins (Basel). 2019 Mar 19;11(3):167. doi: 10.3390/toxins11030167 (PMC6468893; doi:10.3390/toxins11030167)
Supplement: Supplementary file 1 [file toxins-11-00167-s001.zip › Supplementary Dataset EV1/20180328_f2_topdown_OTMS2_EThcD_NL_i02_ms2_proteoform_cutoff_html/prsms/prsm162.html]

Protein-Spectrum-Match for Spectrum #401


All proteins /
CsTx-9a Cupiennius salei toxin 9 isoform a /
Proteoform #10

## Protein-Spectrum-Match #162 for Spectrum #401

|  |  |  |  |  |  |
| --- | --- | --- | --- | --- | --- |
| PrSM ID: | 162 | Scan(s): | 537 | Precursor charge: | 13 |
| Precursor m/z: | 591.2156 | Precursor mass: | 7672.7087 | Proteoform mass: | 7672.6994 |
| # matched peaks: | 39 | # matched fragment ions: | 28 | # unexpected modifications: | 0 |
| E-value: | 1.59e-26 | P-value: | 1.59e-26 | Q-value (Spectral FDR): | 0 |

  

|  |  |  |  |  |  |  |  |  |  |  |  |  |  |  |  |  |  |  |  |  |  |  |  |  |  |  |  |  |  |  |  |  |  |  |  |  |  |  |  |  |  |  |  |  |  |  |  |  |  |  |  |  |  |  |  |  |  |  |  |  |  |  |  |  |  |  |  |  |  |
| --- | --- | --- | --- | --- | --- | --- | --- | --- | --- | --- | --- | --- | --- | --- | --- | --- | --- | --- | --- | --- | --- | --- | --- | --- | --- | --- | --- | --- | --- | --- | --- | --- | --- | --- | --- | --- | --- | --- | --- | --- | --- | --- | --- | --- | --- | --- | --- | --- | --- | --- | --- | --- | --- | --- | --- | --- | --- | --- | --- | --- | --- | --- | --- | --- | --- | --- | --- | --- | --- |
|  | |  | | | | | | | | | | | | | | | | | | | | | | | | | | | | | | | | | | | | | | | | | | | | | | | | | | | | | | | | | | | | | | | | | | | |
| 1 |  |  | M |  | K |  | V |  | L |  | V |  | I |  | C |  | A |  | V |  | L |  |  | F |  | L |  | A |  | I |  | F |  | S |  | N |  | S |  | S |  | A |  |  | E |  | T |  | E |  | D |  | D |  | F |  | L |  | E |  | D |  | E |  | 30 |  |
|  | |  | | | | | | | | | | | | | | | | | | | | | | | | | | | | | | | | | | | | | | | | | | | | | | | | | | | | | | | | | | | | | | | | | | | |
| 31 |  |  | S |  | F |  | E |  | A |  | D |  | D |  | V |  | I |  | P |  | F |  |  | L |  | A |  | R |  | E |  | Q |  | V |  | R | ] | K |  | D |  | D |  |  | K | ⎫ | N | ⎫ | C |  | I |  | P |  | K | ⎫ | H |  | H | ⎫ | E | ⎫ | C |  | 60 |  |
|  | |  | | | | | | | | | | | | | | | | | | | | | | | | | | | | | | | | | | | | | | | | | | | | | | | | | | | | | | | | | | | | | | | | | | | |
| 61 |  |  | T | ⎱ | N | ⎱ | D | ⎫ | K | ⎫ | K | ⎫ | N | ⎫ | C |  | C |  | K | ⎫ | K |  | ⎩ | G |  | L |  | T | ⎱ | K | ⎩ | M | ⎫ | K |  | C | ⎱ | K | ⎫ | C | ⎫ | F |  |  | T |  | V |  | A | ⎱ | D | ⎩ | A | ⎩ | K | ⎩ | G |  | A |  | T |  | S |  | 90 |  |
|  | |  | | | | | | | | | | | | | | | | | | | | | | | | | | | | | | | | | | | | | | | | | | | | | | | | | | | | | | | | | | | | | | | | | | | |
| 91 |  |  | E |  | R |  | C |  | A |  | C |  | D |  | S |  | S |  | L |  | L |  |  | Q |  | K |  | F |  | G |  | F |  | T |  | G |  | L |  | H |  | I |  |  | I |  | K | [ | G |  | L |  | F |  | | 115 |  | | | | | | | | | |

Fixed PTMs: Carbamidomethylation [C53 C60 C67 C68 C77 C79 C93 C95 ]

  

All peaks (126)  Matched peaks (39)  Not matched peaks (87)

  

| Scan | Peak | Mono mass | Mono m/z | Intensity | Charge | Theoretical mass | Ion | Pos | Mass error | PPM error |
| --- | --- | --- | --- | --- | --- | --- | --- | --- | --- | --- |
| 537 | 1 | 2951.7195 | 591.3512 | 18090.71 | 5 |  |  |  |  |  |
| 537 | 2 | 7558.6236 | 840.8543 | 7436.10 | 9 |  |  |  |  |  |
| 537 | 3 | 1779.8034 | 594.2751 | 11038.29 | 3 | 1779.8144 | C14 | 14 | -0.0110 | -6.17 |
| 537 | 4 | 1596.9535 | 799.4840 | 9723.79 | 2 |  |  |  |  |  |
| 537 | 5 | 2187.1376 | 730.0532 | 9160.73 | 3 |  |  |  |  |  |
| 537 | 6 | 589.0372 | 590.0444 | 13677.60 | 1 |  |  |  |  |  |
| 537 | 7 | 7615.6443 | 847.1900 | 9331.47 | 9 |  |  |  |  |  |
| 537 | 8 | 3077.5467 | 770.3939 | 6849.29 | 4 |  |  |  |  |  |
| 537 | 9 | 1683.9854 | 843.0000 | 7651.12 | 2 |  |  |  |  |  |
| 537 | 10 | 7615.6606 | 762.5733 | 8265.67 | 10 |  |  |  |  |  |
| 537 | 11 | 7557.6202 | 945.7098 | 5760.11 | 8 |  |  |  |  |  |
| 537 | 12 | 2951.7185 | 738.9369 | 7516.01 | 4 |  |  |  |  |  |
| 537 | 13 | 2008.8726 | 670.6315 | 6634.13 | 3 | 2008.8843 | C16 | 16 | -0.0117 | -5.83 |
| 537 | 14 | 4137.3952 | 592.0637 | 73000.43 | 7 |  |  |  |  |  |
| 537 | 15 | 3680.8241 | 921.2133 | 5192.49 | 4 |  |  |  |  |  |
| 537 | 16 | 7557.6208 | 756.7694 | 4383.02 | 10 |  |  |  |  |  |
| 537 | 17 | 1893.8457 | 632.2892 | 5501.54 | 3 | 1893.8573 | C15 | 15 | -0.0116 | -6.14 |
| 537 | 18 | 2649.3138 | 884.1119 | 5497.57 | 3 |  |  |  |  |  |
| 537 | 19 | 3226.5022 | 646.3077 | 4013.82 | 5 | 3226.5216 | C26 | 26 | -0.0194 | -6.01 |
| 537 | 20 | 3680.8218 | 737.1716 | 4218.37 | 5 |  |  |  |  |  |
| 537 | 21 | 7613.6382 | 693.1562 | 6205.06 | 11 |  |  |  |  |  |
| 537 | 22 | 3263.6102 | 816.9098 | 6325.91 | 4 |  |  |  |  |  |
| 537 | 23 | 3078.5560 | 1027.1926 | 3848.78 | 3 | 3078.5505 | Z\_DOT28 | 37 | 5.54e-03 | 1.80 |
| 537 | 24 | 1779.8023 | 890.9084 | 4151.88 | 2 | 1779.8144 | C14 | 14 | -0.0121 | -6.81 |
| 537 | 25 | 7616.6500 | 953.0885 | 5338.99 | 8 |  |  |  |  |  |
| 537 | 26 | 2528.0726 | 633.0254 | 3387.21 | 4 |  |  |  |  |  |
| 537 | 27 | 3899.9207 | 975.9875 | 3458.44 | 4 | 3899.9247 | Z\_DOT35 | 30 | -3.93e-03 | -1.01 |
| 537 | 28 | 1553.8992 | 777.9569 | 3268.80 | 2 |  |  |  |  |  |
| 537 | 29 | 2923.7287 | 585.7530 | 2904.58 | 5 |  |  |  |  |  |
| 537 | 30 | 2379.1038 | 794.0419 | 3281.97 | 3 | 2379.1171 | C19 | 19 | -0.0134 | -5.62 |
| 537 | 31 | 2363.3668 | 591.8490 | 69158.68 | 4 |  |  |  |  |  |
| 537 | 32 | 3192.5712 | 799.1501 | 4365.50 | 4 |  |  |  |  |  |
| 537 | 33 | 3901.8478 | 651.3152 | 3882.32 | 6 | 3901.8776 | C31 | 31 | -0.0298 | -7.63 |
| 537 | 34 | 3533.8436 | 589.9812 | 5536.40 | 6 |  |  |  |  |  |
| 537 | 35 | 3773.7571 | 629.9668 | 3753.71 | 6 | 3773.7826 | C30 | 30 | -0.0255 | -6.75 |
| 537 | 36 | 4319.0810 | 864.8235 | 3337.16 | 5 | 4319.0908 | Z\_DOT38 | 27 | -9.75e-03 | -2.26 |
| 537 | 37 | 3844.8299 | 641.8123 | 3039.06 | 6 |  |  |  |  |  |
| 537 | 38 | 3193.5807 | 1065.5342 | 3124.00 | 3 | 3193.5774 | Z\_DOT29 | 36 | 3.24e-03 | 1.02 |
| 537 | 39 | 3353.5935 | 559.9395 | 2941.11 | 6 |  |  |  |  |  |
| 537 | 40 | 2187.1381 | 1094.5763 | 3595.04 | 2 |  |  |  |  |  |
| 537 | 41 | 2265.0600 | 756.0273 | 4160.87 | 3 | 2265.0742 | C18 | 18 | -0.0142 | -6.26 |
| 537 | 42 | 3546.8832 | 710.3839 | 2876.16 | 5 |  |  |  |  |  |
| 537 | 43 | 3485.6418 | 581.9476 | 3580.27 | 6 | 3485.6570 | C28 | 28 | -0.0152 | -4.35 |
| 537 | 44 | 3968.9385 | 794.7950 | 2255.18 | 5 |  |  |  |  |  |
| 537 | 45 | 1389.6852 | 695.8499 | 2994.09 | 2 | 1389.6935 | C11 | 11 | -8.27e-03 | -5.95 |
| 537 | 46 | 1893.8507 | 947.9326 | 2797.93 | 2 | 1893.8573 | C15 | 15 | -6.67e-03 | -3.52 |
| 537 | 47 | 1612.9699 | 807.4922 | 3038.39 | 2 |  |  |  |  |  |
| 537 | 48 | 4718.3301 | 944.6733 | 1715.12 | 5 | 4718.3389 | Z\_DOT42 | 23 | -8.84e-03 | -1.87 |
| 537 | 49 | 2879.4226 | 960.8148 | 2516.80 | 3 | 2879.4184 | Z\_DOT26 | 39 | 4.17e-03 | 1.45 |
| 537 | 50 | 4447.1769 | 890.4426 | 3209.71 | 5 | 4447.1857 | Z\_DOT39 | 26 | -8.87e-03 | -1.99 |
| 537 | 51 | 2470.0504 | 618.5199 | 3009.09 | 4 |  |  |  |  |  |
| 537 | 52 | 2821.3934 | 941.4717 | 2731.63 | 3 |  |  |  |  |  |
| 537 | 53 | 7499.5851 | 938.4554 | 1577.72 | 8 |  |  |  |  |  |
| 537 | 54 | 3837.8356 | 768.5744 | 4092.84 | 5 |  |  |  |  |  |
| 537 | 55 | 2750.3556 | 917.7925 | 3363.98 | 3 |  |  |  |  |  |
| 537 | 56 | 2562.2773 | 855.0997 | 2196.11 | 3 |  |  |  |  |  |
| 537 | 57 | 4480.0979 | 641.0213 | 2724.18 | 7 | 4480.1298 | C36 | 36 | -0.0319 | -7.13 |
| 537 | 58 | 3148.5773 | 788.1516 | 1883.14 | 4 |  |  |  |  |  |
| 537 | 59 | 763.4808 | 764.4881 | 2695.91 | 1 |  |  |  |  |  |
| 537 | 60 | 617.3101 | 618.3174 | 3697.70 | 1 | 617.3132 | C5 | 5 | -3.12e-03 | -5.06 |
| 537 | 61 | 1372.5774 | 687.2960 | 2092.40 | 2 |  |  |  |  |  |
| 537 | 62 | 5780.8393 | 826.8415 | 2512.15 | 7 | 5779.8499 | Z\_DOT50 | 15 | -0.0129 | -2.24 |
| 537 | 63 | 5894.8711 | 737.8662 | 2482.67 | 8 | 5893.8929 | Z\_DOT51 | 14 | -0.0241 | -4.09 |
| 537 | 64 | 7599.6130 | 760.9686 | 2957.97 | 10 |  |  |  |  |  |
| 537 | 65 | 3226.4986 | 807.6319 | 2016.74 | 4 | 3226.5216 | C26 | 26 | -0.0230 | -7.12 |
| 537 | 66 | 7500.5891 | 834.4061 | 1582.69 | 9 |  |  |  |  |  |
| 537 | 67 | 1518.7251 | 760.3698 | 2828.01 | 2 | 1518.7361 | C12 | 12 | -0.0109 | -7.20 |
| 537 | 68 | 1389.6861 | 464.2360 | 2558.80 | 3 | 1389.6935 | C11 | 11 | -7.43e-03 | -5.34 |
| 537 | 69 | 4660.2960 | 933.0665 | 1871.32 | 5 |  |  |  |  |  |
| 537 | 70 | 2700.1616 | 676.0477 | 1666.22 | 4 |  |  |  |  |  |
| 537 | 71 | 3533.8470 | 707.7767 | 1351.09 | 5 |  |  |  |  |  |
| 537 | 72 | 3485.6384 | 698.1350 | 3859.96 | 5 | 3485.6570 | C28 | 28 | -0.0186 | -5.33 |
| 537 | 73 | 4717.3217 | 787.2276 | 1630.70 | 6 |  |  |  |  |  |
| 537 | 74 | 5837.8357 | 834.9838 | 1411.16 | 7 |  |  |  |  |  |
| 537 | 75 | 1518.7277 | 507.2498 | 1759.76 | 3 | 1518.7361 | C12 | 12 | -8.36e-03 | -5.51 |
| 537 | 76 | 1758.9226 | 880.4686 | 1692.22 | 2 |  |  |  |  |  |
| 537 | 77 | 7014.6100 | 585.5581 | 2203.91 | 12 |  |  |  |  |  |
| 537 | 78 | 2008.8742 | 1005.4444 | 1384.54 | 2 | 2008.8843 | C16 | 16 | -0.0100 | -5.00 |
| 537 | 79 | 1956.0739 | 979.0442 | 2665.60 | 2 |  |  |  |  |  |
| 537 | 80 | 2136.9673 | 535.2491 | 1625.27 | 4 | 2136.9792 | C17 | 17 | -0.0119 | -5.57 |
| 537 | 81 | 7090.6593 | 591.8955 | 15932.73 | 12 |  |  |  |  |  |
| 537 | 82 | 5893.8762 | 842.9896 | 1763.73 | 7 | 5893.8929 | Z\_DOT51 | 14 | -0.0166 | -2.82 |
| 537 | 83 | 3428.6160 | 686.7305 | 1562.79 | 5 |  |  |  |  |  |
| 537 | 84 | 4596.1294 | 657.5972 | 1283.12 | 7 |  |  |  |  |  |
| 537 | 85 | 3609.7901 | 903.4548 | 1538.16 | 4 |  |  |  |  |  |
| 537 | 86 | 2433.2420 | 812.0879 | 1906.90 | 3 |  |  |  |  |  |
| 537 | 87 | 2102.0286 | 701.6835 | 1315.03 | 3 |  |  |  |  |  |
| 537 | 88 | 2293.1544 | 765.3921 | 1364.81 | 3 |  |  |  |  |  |
| 537 | 89 | 4202.0585 | 841.4190 | 1564.35 | 5 |  |  |  |  |  |
| 537 | 90 | 3298.5686 | 825.6494 | 1314.40 | 4 |  |  |  |  |  |
| 537 | 91 | 5259.6930 | 585.4176 | 1641.16 | 9 |  |  |  |  |  |
| 537 | 92 | 3455.7736 | 692.1620 | 1267.04 | 5 |  |  |  |  |  |
| 537 | 93 | 3773.7588 | 540.1157 | 1091.26 | 7 | 3773.7826 | C30 | 30 | -0.0239 | -6.32 |
| 537 | 94 | 3355.6012 | 839.9076 | 1859.90 | 4 |  |  |  |  |  |
| 537 | 95 | 2379.1023 | 595.7829 | 1443.16 | 4 | 2379.1171 | C19 | 19 | -0.0148 | -6.21 |
| 537 | 96 | 3296.5766 | 660.3226 | 1476.60 | 5 |  |  |  |  |  |
| 537 | 97 | 847.0744 | 848.0817 | 1520.78 | 1 |  |  |  |  |  |
| 537 | 98 | 2692.3335 | 898.4518 | 2327.88 | 3 |  |  |  |  |  |
| 537 | 99 | 3007.5225 | 752.8879 | 2128.05 | 4 | 3007.5134 | Z\_DOT27 | 38 | 9.12e-03 | 3.03 |
| 537 | 100 | 556.3067 | 557.3140 | 3558.51 | 1 |  |  |  |  |  |
| 537 | 101 | 4389.1497 | 878.8372 | 1821.87 | 5 |  |  |  |  |  |
| 537 | 102 | 1963.8577 | 655.6265 | 2272.71 | 3 |  |  |  |  |  |
| 537 | 103 | 2827.2574 | 707.8216 | 1853.12 | 4 | 2827.2734 | C22 | 22 | -0.0160 | -5.67 |
| 537 | 104 | 1242.7292 | 622.3719 | 1404.11 | 2 |  |  |  |  |  |
| 537 | 105 | 5203.5767 | 868.2701 | 2063.33 | 6 |  |  |  |  |  |
| 537 | 106 | 6852.1746 | 857.5291 | 1300.09 | 8 |  |  |  |  |  |
| 537 | 107 | 4169.8120 | 695.9759 | 1078.86 | 6 |  |  |  |  |  |
| 537 | 108 | 7598.6342 | 845.3000 | 2354.03 | 9 |  |  |  |  |  |
| 537 | 109 | 503.2679 | 504.2751 | 3112.28 | 1 | 503.2703 | C4 | 4 | -2.43e-03 | -4.83 |
| 537 | 110 | 671.1196 | 672.1269 | 1372.81 | 1 |  |  |  |  |  |
| 537 | 111 | 2136.9664 | 713.3294 | 1703.47 | 3 | 2136.9792 | C17 | 17 | -0.0129 | -6.02 |
| 537 | 112 | 4061.8809 | 677.9874 | 1891.44 | 6 | 4061.9082 | C32 | 32 | -0.0274 | -6.74 |
| 537 | 113 | 1209.8218 | 605.9182 | 1680.67 | 2 |  |  |  |  |  |
| 537 | 114 | 7571.6340 | 842.2999 | 1277.43 | 9 |  |  |  |  |  |
| 537 | 115 | 1115.5691 | 558.7918 | 1000.46 | 2 | 1115.5757 | C9 | 9 | -6.54e-03 | -5.86 |
| 537 | 116 | 1322.9049 | 662.4597 | 907.01 | 2 |  |  |  |  |  |
| 537 | 117 | 1494.9876 | 748.5011 | 1057.71 | 2 |  |  |  |  |  |
| 537 | 118 | 1081.7284 | 541.8715 | 1266.92 | 2 |  |  |  |  |  |
| 537 | 119 | 1114.6346 | 1115.6419 | 1124.52 | 1 |  |  |  |  |  |
| 537 | 120 | 866.4336 | 867.4409 | 919.84 | 1 |  |  |  |  |  |
| 537 | 121 | 1269.5657 | 635.7901 | 714.35 | 2 |  |  |  |  |  |
| 537 | 122 | 1486.9507 | 496.6575 | 1307.74 | 3 |  |  |  |  |  |
| 537 | 123 | 1433.9551 | 717.9848 | 703.32 | 2 |  |  |  |  |  |
| 537 | 124 | 1088.2073 | 1089.2145 | 892.54 | 1 |  |  |  |  |  |
| 537 | 125 | 1132.5297 | 567.2721 | 1206.25 | 2 |  |  |  |  |  |
| 537 | 126 | 685.3243 | 686.3316 | 804.66 | 1 |  |  |  |  |  |

  

All proteins /
CsTx-9a Cupiennius salei toxin 9 isoform a /
Proteoform #10
