# Supplementary material for: The Dual Prey-Inactivation Strategy of Spiders—In-Depth Venomic Analysis of Cupiennius salei
Source: Toxins (Basel). 2019 Mar 19;11(3):167. doi: 10.3390/toxins11030167 (PMC6468893; doi:10.3390/toxins11030167)
Supplement: Supplementary file 1 [file toxins-11-00167-s001.zip › Supplementary Dataset EV1/20180328_f2_topdown_OTMS2_EThcD_NL_i02_ms2_proteoform_cutoff_html/prsms/prsm163.html]

Protein-Spectrum-Match for Spectrum #402


All proteins /
CsTx-12a\_S1 Cupiennius salei toxin 12 isoform a S1^ACsTx-12a\_S2 Cupiennius salei toxin 12 isoform a S2 /
Proteoform #53

## Protein-Spectrum-Match #163 for Spectrum #402

|  |  |  |  |  |  |
| --- | --- | --- | --- | --- | --- |
| PrSM ID: | 163 | Scan(s): | 539 | Precursor charge: | 6 |
| Precursor m/z: | 569.3245 | Precursor mass: | 3409.9036 | Proteoform mass: | 3409.8986 |
| # matched peaks: | 29 | # matched fragment ions: | 24 | # unexpected modifications: | 1 |
| E-value: | 3.25e-19 | P-value: | 3.25e-19 | Q-value (Spectral FDR): | 0 |

  

|  |  |  |  |  |  |  |  |  |  |  |  |  |  |  |  |  |  |  |  |  |  |  |  |  |  |  |  |  |  |  |  |  |  |  |  |  |  |  |  |  |  |  |  |  |  |  |  |  |  |  |  |  |  |  |  |  |  |  |  |  |  |  |  |  |  |  |
| --- | --- | --- | --- | --- | --- | --- | --- | --- | --- | --- | --- | --- | --- | --- | --- | --- | --- | --- | --- | --- | --- | --- | --- | --- | --- | --- | --- | --- | --- | --- | --- | --- | --- | --- | --- | --- | --- | --- | --- | --- | --- | --- | --- | --- | --- | --- | --- | --- | --- | --- | --- | --- | --- | --- | --- | --- | --- | --- | --- | --- | --- | --- | --- | --- | --- | --- |
|  | | ... 30 amino acid residues are skipped at the N-terminus ... | | | | | | | | | | | | | | | | | | | | | | | | | | | | | | | | | | | | | | | | | | | | | | | | | | | | | | | | | | | | | |  | | |
|  | |  | | | | | | | | | | | | | | | | | | | | | | | | | | | | | | | | | | | | | | | | | | | | | | | | | | | | | | | | | | | | | | | | | | | |
| 31 |  |  | S |  | F |  | E |  | A |  | D |  | D |  | V |  | I |  | P |  | F |  |  | L |  | A |  | R |  | E |  | Q |  | V |  | R |  | S |  | D |  | C |  |  | T |  | L |  | R |  | N |  | H |  | D |  | C |  | T |  | D |  | D |  | 60 |  |
|  | |  | | | | | | | | | | | | | | | | | | | | | | | | | | | | | | | | | | | | | | | | | | | | | | | | | | | | | | | | | | | | | | | | | | | |
| 61 |  |  | R |  | H |  | S |  | C |  | C |  | R |  | S |  | K |  | M |  | F |  |  | K |  | D |  | V |  | C |  | K |  | C |  | F |  | Y |  | P |  | S |  |  | Q |  | R |  | S |  | D |  | T |  | A |  | R | ] | A | ⎩ | K | ⎩ | K |  | 90 |  |
|  | |  | | | | | | | | | | | | | | | | | | | | | | | | | | | | | | | | | | | | | | | | | | | | | | | | | | | | | -58.01 | | | | | | | | | | | |
| 91 |  |  | E | ⎫ | L |  | C |  | T | ⎫ | C | ⎫ | Q | ⎫ | Q | ⎫ | D |  | K |  | H |  |  | L |  | K | ⎱ | F | ⎱ | I | ⎫ | E | ⎫ | K |  | G | ⎫ | L |  | Q | ⎱ | K |  | ⎱ | A | ⎱ | K | ⎫ | V | ⎫ | L | ⎫ | V | ⎫ | A |  | G |  | | 117 |  | | | | | |

Fixed PTMs: Carbamidomethylation [C93 C95 ]   
  
     Unexpected modifications:   Unknown [-58.01]

  

All peaks (56)  Matched peaks (29)  Not matched peaks (27)

  

| Scan | Peak | Mono mass | Mono m/z | Intensity | Charge | Theoretical mass | Ion | Pos | Mass error | PPM error |
| --- | --- | --- | --- | --- | --- | --- | --- | --- | --- | --- |
| 539 | 1 | 3352.8653 | 671.5803 | 436424.60 | 5 |  |  |  |  |  |
| 539 | 2 | 3126.7007 | 782.6825 | 160145.15 | 4 | 3126.7157 | C26 | 26 | -0.0150 | -4.79 |
| 539 | 3 | 1705.4516 | 569.4911 | 1065627.21 | 3 |  |  |  |  |  |
| 539 | 4 | 3338.8538 | 668.7780 | 159051.32 | 5 | 3338.8682 | C28 | 28 | -0.0144 | -4.32 |
| 539 | 5 | 3352.8683 | 839.2244 | 133722.39 | 4 |  |  |  |  |  |
| 539 | 6 | 2274.1508 | 759.0576 | 118150.44 | 3 | 2274.1612 | C18 | 18 | -0.0104 | -4.57 |
| 539 | 7 | 2145.1087 | 716.0435 | 105493.41 | 3 | 2145.1186 | C17 | 17 | -9.92e-03 | -4.63 |
| 539 | 8 | 3322.8324 | 665.5737 | 80599.51 | 5 | 3322.8428 | Z\_DOT29 | 1 | -0.0104 | -3.14 |
| 539 | 9 | 2899.5389 | 725.8920 | 95201.63 | 4 | 2899.5523 | C24 | 24 | -0.0135 | -4.65 |
| 539 | 10 | 2828.5020 | 708.1328 | 86976.07 | 4 | 2828.5152 | C23 | 23 | -0.0132 | -4.68 |
| 539 | 11 | 568.6491 | 569.6564 | 719393.85 | 1 |  |  |  |  |  |
| 539 | 12 | 1884.9574 | 629.3264 | 118942.78 | 3 | 1884.9662 | C15 | 15 | -8.75e-03 | -4.64 |
| 539 | 13 | 3392.8707 | 566.4857 | 70881.37 | 6 |  |  |  |  |  |
| 539 | 14 | 3194.7395 | 799.6922 | 60580.33 | 4 | 3194.7478 | Z\_DOT28 | 2 | -8.28e-03 | -2.59 |
| 539 | 15 | 1525.9402 | 763.9774 | 101094.60 | 2 | 1525.9402 | Z\_DOT15 | 15 | -1.18e-05 | -7.74e-03 |
| 539 | 16 | 3393.8696 | 679.7812 | 63374.75 | 5 |  |  |  |  |  |
| 539 | 17 | 2700.4084 | 901.1434 | 59798.70 | 3 | 2700.4203 | C22 | 22 | -0.0118 | -4.38 |
| 539 | 18 | 2459.2666 | 820.7628 | 64330.52 | 3 | 2459.2776 | C20 | 20 | -0.0110 | -4.49 |
| 539 | 19 | 3322.8350 | 831.7160 | 74474.52 | 4 | 3322.8428 | Z\_DOT29 | 1 | -7.82e-03 | -2.35 |
| 539 | 20 | 2032.0255 | 678.3491 | 70426.82 | 3 | 2032.0346 | C16 | 16 | -9.04e-03 | -4.45 |
| 539 | 21 | 3365.8734 | 674.1820 | 43437.24 | 5 |  |  |  |  |  |
| 539 | 22 | 3239.7850 | 810.9535 | 42331.72 | 4 | 3239.7998 | C27 | 27 | -0.0148 | -4.57 |
| 539 | 23 | 1884.9580 | 943.4863 | 59235.93 | 2 | 1884.9662 | C15 | 15 | -8.19e-03 | -4.34 |
| 539 | 24 | 3027.6333 | 757.9156 | 53195.67 | 4 | 3027.6473 | C25 | 25 | -0.0140 | -4.61 |
| 539 | 25 | 3366.8827 | 842.7280 | 41629.46 | 4 |  |  |  |  |  |
| 539 | 26 | 3408.8902 | 682.7853 | 178759.30 | 5 |  |  |  |  |  |
| 539 | 27 | 1263.6009 | 632.8077 | 59507.10 | 2 | 1263.6063 | C10 | 10 | -5.46e-03 | -4.32 |
| 539 | 28 | 2700.4075 | 676.1092 | 41693.62 | 4 | 2700.4203 | C22 | 22 | -0.0128 | -4.73 |
| 539 | 29 | 2345.3838 | 587.3532 | 46303.73 | 4 |  |  |  |  |  |
| 539 | 30 | 3210.7588 | 803.6970 | 32721.54 | 4 |  |  |  |  |  |
| 539 | 31 | 2419.4315 | 605.8651 | 33199.82 | 4 |  |  |  |  |  |
| 539 | 32 | 3274.8771 | 655.9827 | 30330.90 | 5 |  |  |  |  |  |
| 539 | 33 | 1378.8720 | 690.4433 | 37064.61 | 2 | 1378.8718 | Z\_DOT14 | 16 | 1.61e-04 | 0.12 |
| 539 | 34 | 3338.8524 | 835.7204 | 31986.29 | 4 | 3338.8682 | C28 | 28 | -0.0157 | -4.72 |
| 539 | 35 | 682.1796 | 683.1868 | 137447.54 | 1 |  |  |  |  |  |
| 539 | 36 | 1364.7583 | 683.3864 | 260519.45 | 2 |  |  |  |  |  |
| 539 | 37 | 1007.4853 | 1008.4926 | 28271.44 | 1 | 1007.4892 | C8 | 8 | -3.89e-03 | -3.86 |
| 539 | 38 | 908.5771 | 455.2958 | 30898.85 | 2 |  |  |  |  |  |
| 539 | 39 | 710.4900 | 711.4973 | 20764.86 | 1 | 710.4861 | Z\_DOT8 | 22 | 3.93e-03 | 5.53 |
| 539 | 40 | 582.3956 | 583.4029 | 28271.02 | 1 | 582.3912 | Z\_DOT7 | 23 | 4.46e-03 | 7.66 |
| 539 | 41 | 1349.8333 | 675.9239 | 11797.94 | 2 |  |  |  |  |  |
| 539 | 42 | 1206.7759 | 604.3952 | 15222.86 | 2 |  |  |  |  |  |
| 539 | 43 | 1135.5433 | 1136.5506 | 11943.06 | 1 | 1135.5477 | C9 | 9 | -4.43e-03 | -3.90 |
| 539 | 44 | 511.3590 | 512.3663 | 15398.86 | 1 | 511.3540 | Z\_DOT6 | 24 | 4.95e-03 | 9.68 |
| 539 | 45 | 1496.9016 | 499.9745 | 8448.87 | 3 |  |  |  |  |  |
| 539 | 46 | 873.4706 | 874.4779 | 7873.14 | 1 |  |  |  |  |  |
| 539 | 47 | 633.3962 | 634.4034 | 25087.21 | 1 |  |  |  |  |  |
| 539 | 48 | 780.5192 | 391.2669 | 5731.68 | 2 |  |  |  |  |  |
| 539 | 49 | 847.4548 | 848.4621 | 13471.30 | 1 | 847.4585 | C7 | 7 | -3.69e-03 | -4.36 |
| 539 | 50 | 967.6499 | 484.8322 | 5275.56 | 2 |  |  |  |  |  |
| 539 | 51 | 1152.7652 | 577.3899 | 7166.76 | 2 |  |  |  |  |  |
| 539 | 52 | 473.2946 | 474.3019 | 11355.20 | 1 | 473.2961 | C4 | 4 | -1.52e-03 | -3.20 |
| 539 | 53 | 726.5086 | 727.5159 | 5914.68 | 1 |  |  |  |  |  |
| 539 | 54 | 1349.8337 | 450.9518 | 4505.13 | 3 |  |  |  |  |  |
| 539 | 55 | 1078.6822 | 540.3484 | 7655.09 | 2 |  |  |  |  |  |
| 539 | 56 | 1263.6013 | 1264.6086 | 4230.92 | 1 | 1263.6063 | C10 | 10 | -5.02e-03 | -3.98 |

  

All proteins /
CsTx-12a\_S1 Cupiennius salei toxin 12 isoform a S1^ACsTx-12a\_S2 Cupiennius salei toxin 12 isoform a S2 /
Proteoform #53
