# Supplementary material for: The Dual Prey-Inactivation Strategy of Spiders—In-Depth Venomic Analysis of Cupiennius salei
Source: Toxins (Basel). 2019 Mar 19;11(3):167. doi: 10.3390/toxins11030167 (PMC6468893; doi:10.3390/toxins11030167)
Supplement: Supplementary file 1 [file toxins-11-00167-s001.zip › Supplementary Dataset EV1/20180328_f2_topdown_OTMS2_EThcD_NL_i02_ms2_proteoform_cutoff_html/prsms/prsm164.html]

Protein-Spectrum-Match for Spectrum #403


All proteins /
CsTx-9a Cupiennius salei toxin 9 isoform a /
Proteoform #10

## Protein-Spectrum-Match #164 for Spectrum #403

|  |  |  |  |  |  |
| --- | --- | --- | --- | --- | --- |
| PrSM ID: | 164 | Scan(s): | 540 | Precursor charge: | 12 |
| Precursor m/z: | 640.3164 | Precursor mass: | 7671.7095 | Proteoform mass: | 7671.7045 |
| # matched peaks: | 58 | # matched fragment ions: | 47 | # unexpected modifications: | 1 |
| E-value: | 4.07e-39 | P-value: | 4.07e-39 | Q-value (Spectral FDR): | 0 |

  

|  |  |  |  |  |  |  |  |  |  |  |  |  |  |  |  |  |  |  |  |  |  |  |  |  |  |  |  |  |  |  |  |  |  |  |  |  |  |  |  |  |  |  |  |  |  |  |  |  |  |  |  |  |  |  |  |  |  |  |  |  |  |  |  |  |  |  |  |  |  |
| --- | --- | --- | --- | --- | --- | --- | --- | --- | --- | --- | --- | --- | --- | --- | --- | --- | --- | --- | --- | --- | --- | --- | --- | --- | --- | --- | --- | --- | --- | --- | --- | --- | --- | --- | --- | --- | --- | --- | --- | --- | --- | --- | --- | --- | --- | --- | --- | --- | --- | --- | --- | --- | --- | --- | --- | --- | --- | --- | --- | --- | --- | --- | --- | --- | --- | --- | --- | --- | --- |
|  | |  | | | | | | | | | | | | | | | | | | | | | | | | | | | | | | | | | | | | | | | | | | | | | | | | | | | | | | | | | | | | | | | | | | | |
| 1 |  |  | M |  | K |  | V |  | L |  | V |  | I |  | C |  | A |  | V |  | L |  |  | F |  | L |  | A |  | I |  | F |  | S |  | N |  | S |  | S |  | A |  |  | E |  | T |  | E |  | D |  | D |  | F |  | L |  | E |  | D |  | E |  | 30 |  |
|  | |  | | | | | | | | | | | | | | | | | | | | | | | | | | | | | | | | | | | | | | | | | | | | | | | | | | | | | | | | | | | | | | | | | | | |
| 31 |  |  | S |  | F |  | E |  | A |  | D |  | D |  | V |  | I |  | P |  | F |  |  | L |  | A |  | R |  | E |  | Q |  | V |  | R | ] | K |  | D |  | D |  |  | K | ⎫ | N | ⎫ | C | ⎫ | I |  | P |  | K | ⎫ | H |  | H | ⎫ | E | ⎫ | C |  | 60 |  |
|  | |  | | | | | | | | | | | | | | | | | | | | | | | | | | | | | | | | | | | | | | | | | | | | | | | | | | | | | | | | | | | | | | | | | | | |
| 61 |  |  | T | ⎱ | N | ⎫ | D | ⎱ | K |  | K | ⎫ | N | ⎫ | C | ⎫ | C | ⎫ | K |  | K |  | ⎫ | G | ⎫ | L |  | T | ⎫ | K | ⎫ | M | ⎱ | K | ⎫ | C | ⎱ | K | ⎫ | C |  | F |  |  | T |  | V | ⎱ | A | ⎱ | D | ⎱ | A | ⎫ | K | ⎫ | G | ⎩ | A | ⎩ | T | ⎱ | S |  | 90 |  |
|  | |  | | | | | | | | | | | | | | | | | | | | | | | | | | | | | | | | | | | | | | | | | | | | | -318.17 | | | | | | | | | | | | | | | | | | | |
| 91 |  | ⎩ | E |  | R | ⎫ | C |  | A |  | C |  | D |  | S | ⎩ | S | ⎱ | L |  | L |  |  | Q | ⎩ | K | ⎩ | F |  | G | ⎩ | F | ⎩ | T |  | G |  | L |  | H |  | I |  |  | I | ⎫ | K |  | G |  | L |  | F |  | | 115 |  | | | | | | | | | |

Fixed PTMs: Carbamidomethylation [C53 C60 C67 C68 C77 C79 C93 C95 ]   
  
     Unexpected modifications:   Unknown [-318.17]

  

All peaks (127)  Matched peaks (58)  Not matched peaks (69)

  

| Scan | Peak | Mono mass | Mono m/z | Intensity | Charge | Theoretical mass | Ion | Pos | Mass error | PPM error |
| --- | --- | --- | --- | --- | --- | --- | --- | --- | --- | --- |
| 540 | 1 | 7614.6442 | 847.0788 | 19059.10 | 9 |  |  |  |  |  |
| 540 | 2 | 2450.4893 | 613.6296 | 18525.29 | 4 |  |  |  |  |  |
| 540 | 3 | 7615.6488 | 762.5722 | 16521.16 | 10 |  |  |  |  |  |
| 540 | 4 | 2187.1384 | 730.0534 | 11969.91 | 3 |  |  |  |  |  |
| 540 | 5 | 7615.6540 | 952.9640 | 9892.95 | 8 |  |  |  |  |  |
| 540 | 6 | 2008.8729 | 670.6316 | 12196.60 | 3 | 2008.8843 | C16 | 16 | -0.0113 | -5.64 |
| 540 | 7 | 7557.6184 | 840.7427 | 11095.21 | 9 |  |  |  |  |  |
| 540 | 8 | 3263.6104 | 816.9099 | 7723.05 | 4 | 3263.6196 | Z\_DOT33 | 35 | -9.18e-03 | -2.81 |
| 540 | 9 | 7557.6280 | 945.7108 | 10847.43 | 8 |  |  |  |  |  |
| 540 | 10 | 1779.8035 | 890.9090 | 9741.50 | 2 | 1779.8144 | C14 | 14 | -0.0109 | -6.13 |
| 540 | 11 | 2379.1039 | 794.0419 | 6752.11 | 3 | 2379.1171 | C19 | 19 | -0.0132 | -5.54 |
| 540 | 12 | 1893.8466 | 632.2895 | 10072.00 | 3 | 1893.8573 | C15 | 15 | -0.0107 | -5.66 |
| 540 | 13 | 1683.9864 | 843.0005 | 8573.67 | 2 | 1683.9841 | Z\_DOT18 | 50 | 2.27e-03 | 1.35 |
| 540 | 14 | 3680.8224 | 921.2129 | 5656.06 | 4 |  |  |  |  |  |
| 540 | 15 | 3226.5019 | 646.3076 | 7646.62 | 5 | 3226.5216 | C26 | 26 | -0.0197 | -6.10 |
| 540 | 16 | 1919.2162 | 640.7460 | 16377.50 | 3 |  |  |  |  |  |
| 540 | 17 | 1596.9532 | 799.4839 | 9818.01 | 2 | 1596.9521 | Z\_DOT17 | 51 | 1.10e-03 | 0.69 |
| 540 | 18 | 3077.5460 | 770.3938 | 6739.85 | 4 | 3077.5555 | Z\_DOT31 | 37 | -9.58e-03 | -3.11 |
| 540 | 19 | 2649.3125 | 884.1114 | 7008.20 | 3 | 2649.3172 | Z\_DOT26 | 42 | -4.71e-03 | -1.78 |
| 540 | 20 | 3354.5953 | 671.9263 | 6805.82 | 5 | 3354.6165 | C27 | 27 | -0.0212 | -6.31 |
| 540 | 21 | 2187.1406 | 1094.5776 | 6491.57 | 2 |  |  |  |  |  |
| 540 | 22 | 4595.1284 | 657.4542 | 5891.94 | 7 | 4595.1568 | C37 | 37 | -0.0284 | -6.19 |
| 540 | 23 | 3226.5042 | 807.6333 | 3909.60 | 4 | 3226.5216 | C26 | 26 | -0.0174 | -5.38 |
| 540 | 24 | 3485.6375 | 698.1348 | 7069.93 | 5 | 3485.6570 | C28 | 28 | -0.0195 | -5.59 |
| 540 | 25 | 1518.7277 | 760.3711 | 6087.22 | 2 | 1518.7361 | C12 | 12 | -8.38e-03 | -5.52 |
| 540 | 26 | 3837.3396 | 768.4752 | 6683.75 | 5 |  |  |  |  |  |
| 540 | 27 | 5395.5301 | 771.7973 | 3813.61 | 7 | 5395.5708 | C45 | 45 | -0.0408 | -7.55 |
| 540 | 28 | 1893.8466 | 947.9306 | 6001.24 | 2 | 1893.8573 | C15 | 15 | -0.0107 | -5.65 |
| 540 | 29 | 4718.3237 | 944.6720 | 4569.50 | 5 |  |  |  |  |  |
| 540 | 30 | 3901.8519 | 651.3159 | 5580.96 | 6 | 3901.8776 | C31 | 31 | -0.0257 | -6.59 |
| 540 | 31 | 5663.7970 | 810.1211 | 3276.54 | 7 | 5663.8280 | Z\_DOT52 | 16 | -0.0310 | -5.48 |
| 540 | 32 | 638.4447 | 639.4520 | 6035.71 | 1 |  |  |  |  |  |
| 540 | 33 | 4319.0792 | 864.8231 | 3770.40 | 5 |  |  |  |  |  |
| 540 | 34 | 3192.5750 | 799.1510 | 5627.60 | 4 | 3192.5825 | Z\_DOT32 | 36 | -7.48e-03 | -2.34 |
| 540 | 35 | 4794.2534 | 800.0495 | 3381.81 | 6 | 4794.2889 | C39 | 39 | -0.0354 | -7.39 |
| 540 | 36 | 2539.1339 | 847.3852 | 3375.50 | 3 | 2539.1478 | C20 | 20 | -0.0139 | -5.46 |
| 540 | 37 | 2955.3526 | 592.0778 | 4313.95 | 5 | 2955.3684 | C23 | 23 | -0.0158 | -5.34 |
| 540 | 38 | 3773.7612 | 629.9675 | 5964.86 | 6 | 3773.7826 | C30 | 30 | -0.0215 | -5.69 |
| 540 | 39 | 7571.6290 | 947.4609 | 4596.94 | 8 |  |  |  |  |  |
| 540 | 40 | 7598.6212 | 950.8349 | 5382.07 | 8 |  |  |  |  |  |
| 540 | 41 | 4481.1072 | 747.8585 | 4901.57 | 6 |  |  |  |  |  |
| 540 | 42 | 5204.5743 | 868.4363 | 3323.36 | 6 |  |  |  |  |  |
| 540 | 43 | 2879.4178 | 960.8132 | 3342.18 | 3 |  |  |  |  |  |
| 540 | 44 | 1389.6858 | 695.8502 | 6265.07 | 2 | 1389.6935 | C11 | 11 | -7.66e-03 | -5.51 |
| 540 | 45 | 617.3103 | 618.3176 | 5446.28 | 1 | 617.3132 | C5 | 5 | -2.94e-03 | -4.76 |
| 540 | 46 | 4447.1800 | 742.2039 | 2637.92 | 6 |  |  |  |  |  |
| 540 | 47 | 7498.6017 | 938.3325 | 3424.84 | 8 |  |  |  |  |  |
| 540 | 48 | 4447.1686 | 890.4410 | 3924.90 | 5 |  |  |  |  |  |
| 540 | 49 | 2265.0589 | 756.0269 | 6453.70 | 3 | 2265.0742 | C18 | 18 | -0.0153 | -6.75 |
| 540 | 50 | 7629.6524 | 848.7464 | 3589.01 | 9 |  |  |  |  |  |
| 540 | 51 | 2750.3534 | 917.7917 | 5149.24 | 3 | 2750.3649 | Z\_DOT27 | 41 | -0.0115 | -4.18 |
| 540 | 52 | 3968.9490 | 993.2445 | 2180.78 | 4 |  |  |  |  |  |
| 540 | 53 | 5606.7802 | 801.9759 | 2063.11 | 7 |  |  |  |  |  |
| 540 | 54 | 2008.8740 | 1005.4443 | 3686.60 | 2 | 2008.8843 | C16 | 16 | -0.0103 | -5.12 |
| 540 | 55 | 7597.6410 | 845.1896 | 4537.11 | 9 |  |  |  |  |  |
| 540 | 56 | 3898.9255 | 975.7386 | 4558.53 | 4 | 3898.9297 | Z\_DOT38 | 30 | -4.23e-03 | -1.09 |
| 540 | 57 | 1612.9724 | 807.4935 | 3589.96 | 2 |  |  |  |  |  |
| 540 | 58 | 7663.8411 | 639.6607 | 3378.52 | 12 |  |  |  |  |  |
| 540 | 59 | 7542.5900 | 943.8310 | 2995.90 | 8 | 7543.6204 | C64 | 64 | -0.0281 | -3.72 |
| 540 | 60 | 7627.6418 | 763.7715 | 3538.70 | 10 |  |  |  |  |  |
| 540 | 61 | 3192.5806 | 1065.2008 | 3988.61 | 3 | 3192.5825 | Z\_DOT32 | 36 | -1.91e-03 | -0.60 |
| 540 | 62 | 7656.6568 | 851.7469 | 3551.11 | 9 |  |  |  |  |  |
| 540 | 63 | 4667.1709 | 778.8691 | 2389.40 | 6 |  |  |  |  |  |
| 540 | 64 | 3354.5967 | 839.6564 | 3917.43 | 4 | 3354.6165 | C27 | 27 | -0.0198 | -5.92 |
| 540 | 65 | 4480.1036 | 897.0280 | 3019.43 | 5 | 4480.1298 | C36 | 36 | -0.0262 | -5.85 |
| 540 | 66 | 4187.0469 | 838.4166 | 2722.50 | 5 | 4187.0553 | Z\_DOT40 | 28 | -8.44e-03 | -2.02 |
| 540 | 67 | 4661.3085 | 933.2690 | 2446.30 | 5 |  |  |  |  |  |
| 540 | 68 | 7558.6247 | 1080.8108 | 4543.81 | 7 |  |  |  |  |  |
| 540 | 69 | 3077.5541 | 1026.8586 | 3717.38 | 3 | 3077.5555 | Z\_DOT31 | 37 | -1.48e-03 | -0.48 |
| 540 | 70 | 1258.7478 | 630.3812 | 2503.66 | 2 |  |  |  |  |  |
| 540 | 71 | 7038.1260 | 640.8369 | 4309.52 | 11 |  |  |  |  |  |
| 540 | 72 | 4479.1003 | 640.8787 | 2971.24 | 7 |  |  |  |  |  |
| 540 | 73 | 2562.2786 | 855.1001 | 1831.52 | 3 | 2562.2852 | Z\_DOT25 | 43 | -6.56e-03 | -2.56 |
| 540 | 74 | 763.4803 | 764.4876 | 2946.30 | 1 | 763.4721 | Z\_DOT10 | 58 | 8.21e-03 | 10.76 |
| 540 | 75 | 3680.8239 | 737.1721 | 3312.42 | 5 |  |  |  |  |  |
| 540 | 76 | 2821.4005 | 941.4741 | 3023.59 | 3 | 2821.4020 | Z\_DOT28 | 40 | -1.48e-03 | -0.52 |
| 540 | 77 | 7614.6506 | 693.2482 | 4581.11 | 11 |  |  |  |  |  |
| 540 | 78 | 3902.8481 | 781.5769 | 2974.50 | 5 |  |  |  |  |  |
| 540 | 79 | 1779.8038 | 594.2752 | 3034.09 | 3 | 1779.8144 | C14 | 14 | -0.0106 | -5.96 |
| 540 | 80 | 3190.0329 | 639.0139 | 6906.76 | 5 |  |  |  |  |  |
| 540 | 81 | 4409.0629 | 735.8511 | 3442.89 | 6 | 4409.0927 | C35 | 35 | -0.0298 | -6.77 |
| 540 | 82 | 6075.7237 | 760.4727 | 2522.74 | 8 | 6075.7603 | C51 | 51 | -0.0365 | -6.01 |
| 540 | 83 | 2699.1631 | 675.7981 | 1713.59 | 4 | 2699.1784 | C21 | 21 | -0.0153 | -5.67 |
| 540 | 84 | 4846.4138 | 970.2900 | 2228.15 | 5 |  |  |  |  |  |
| 540 | 85 | 5115.4480 | 640.4383 | 3939.11 | 8 |  |  |  |  |  |
| 540 | 86 | 5659.1514 | 944.1992 | 5221.58 | 6 |  |  |  |  |  |
| 540 | 87 | 3613.7289 | 723.7531 | 2660.02 | 5 | 3613.7520 | C29 | 29 | -0.0231 | -6.38 |
| 540 | 88 | 4666.1622 | 667.6019 | 1797.80 | 7 | 4666.1939 | C38 | 38 | -0.0316 | -6.78 |
| 540 | 89 | 1553.9001 | 777.9573 | 4676.16 | 2 |  |  |  |  |  |
| 540 | 90 | 5337.5278 | 890.5952 | 1676.59 | 6 |  |  |  |  |  |
| 540 | 91 | 5780.8303 | 826.8402 | 2321.99 | 7 |  |  |  |  |  |
| 540 | 92 | 3013.3775 | 754.3516 | 2362.54 | 4 |  |  |  |  |  |
| 540 | 93 | 7261.3715 | 908.6787 | 2437.62 | 8 |  |  |  |  |  |
| 540 | 94 | 3148.5834 | 788.1531 | 3637.90 | 4 |  |  |  |  |  |
| 540 | 95 | 2955.3487 | 739.8444 | 3562.28 | 4 | 2955.3684 | C23 | 23 | -0.0197 | -6.66 |
| 540 | 96 | 2692.3242 | 898.4487 | 1945.44 | 3 |  |  |  |  |  |
| 540 | 97 | 2558.9613 | 640.7476 | 3174.39 | 4 |  |  |  |  |  |
| 540 | 98 | 7628.6632 | 954.5902 | 3370.61 | 8 |  |  |  |  |  |
| 540 | 99 | 4865.2788 | 811.8871 | 2226.19 | 6 |  |  |  |  |  |
| 540 | 100 | 4203.0643 | 841.6201 | 2691.80 | 5 |  |  |  |  |  |
| 540 | 101 | 5892.8893 | 842.8486 | 2661.18 | 7 | 5892.8979 | Z\_DOT54 | 14 | -8.64e-03 | -1.47 |
| 540 | 102 | 3485.6383 | 872.4169 | 3057.09 | 4 | 3485.6570 | C28 | 28 | -0.0187 | -5.36 |
| 540 | 103 | 3012.3719 | 603.4817 | 1689.82 | 5 | 3012.3898 | C24 | 24 | -0.0179 | -5.94 |
| 540 | 104 | 5722.8074 | 818.5512 | 1917.26 | 7 |  |  |  |  |  |
| 540 | 105 | 5024.3656 | 718.7738 | 2640.54 | 7 | 5023.3951 | C42 | 42 | -0.0318 | -6.33 |
| 540 | 106 | 3912.9313 | 979.2401 | 1705.28 | 4 |  |  |  |  |  |
| 540 | 107 | 1956.0730 | 979.0438 | 2454.15 | 2 |  |  |  |  |  |
| 540 | 108 | 1242.7284 | 622.3715 | 1961.02 | 2 | 1242.7254 | Z\_DOT14 | 54 | 3.07e-03 | 2.47 |
| 540 | 109 | 503.2680 | 504.2753 | 2886.00 | 1 | 503.2703 | C4 | 4 | -2.28e-03 | -4.53 |
| 540 | 110 | 1115.5694 | 558.7920 | 2606.69 | 2 | 1115.5757 | C9 | 9 | -6.29e-03 | -5.64 |
| 540 | 111 | 885.5093 | 886.5165 | 1993.59 | 1 |  |  |  |  |  |
| 540 | 112 | 777.3393 | 778.3466 | 1335.11 | 1 | 777.3439 | C6 | 6 | -4.54e-03 | -5.84 |
| 540 | 113 | 847.0746 | 848.0819 | 2206.56 | 1 |  |  |  |  |  |
| 540 | 114 | 326.2305 | 327.2377 | 1277.10 | 1 |  |  |  |  |  |
| 540 | 115 | 697.1272 | 698.1345 | 589.09 | 1 |  |  |  |  |  |
| 540 | 116 | 910.5454 | 911.5527 | 1134.10 | 1 | 910.5405 | Z\_DOT11 | 57 | 4.87e-03 | 5.35 |
| 540 | 117 | 1389.6856 | 464.2358 | 1030.74 | 3 | 1389.6935 | C11 | 11 | -7.88e-03 | -5.67 |
| 540 | 118 | 860.3872 | 861.3945 | 846.54 | 1 |  |  |  |  |  |
| 540 | 119 | 535.7782 | 536.7855 | 727.30 | 1 |  |  |  |  |  |
| 540 | 120 | 959.9642 | 960.9715 | 908.10 | 1 |  |  |  |  |  |
| 540 | 121 | 1190.0543 | 1191.0616 | 840.80 | 1 |  |  |  |  |  |
| 540 | 122 | 1204.9115 | 603.4630 | 610.83 | 2 |  |  |  |  |  |
| 540 | 123 | 1242.7294 | 1243.7366 | 731.30 | 1 | 1242.7254 | Z\_DOT14 | 54 | 4.00e-03 | 3.22 |
| 540 | 124 | 1114.6352 | 1115.6425 | 1282.41 | 1 | 1114.6304 | Z\_DOT13 | 55 | 4.84e-03 | 4.34 |
| 540 | 125 | 929.0667 | 930.0740 | 780.44 | 1 |  |  |  |  |  |
| 540 | 126 | 1071.6601 | 1072.6674 | 913.70 | 1 |  |  |  |  |  |
| 540 | 127 | 1036.7601 | 1037.7674 | 1082.68 | 1 |  |  |  |  |  |

  

All proteins /
CsTx-9a Cupiennius salei toxin 9 isoform a /
Proteoform #10
