# Supplementary material for: The Dual Prey-Inactivation Strategy of Spiders—In-Depth Venomic Analysis of Cupiennius salei
Source: Toxins (Basel). 2019 Mar 19;11(3):167. doi: 10.3390/toxins11030167 (PMC6468893; doi:10.3390/toxins11030167)
Supplement: Supplementary file 1 [file toxins-11-00167-s001.zip › Supplementary Dataset EV1/20180328_f2_topdown_OTMS2_EThcD_NL_i02_ms2_proteoform_cutoff_html/prsms/prsm165.html]

Protein-Spectrum-Match for Spectrum #404


All proteins /
CsTx-9a Cupiennius salei toxin 9 isoform a /
Proteoform #10

## Protein-Spectrum-Match #165 for Spectrum #404

|  |  |  |  |  |  |
| --- | --- | --- | --- | --- | --- |
| PrSM ID: | 165 | Scan(s): | 541 | Precursor charge: | 11 |
| Precursor m/z: | 698.4348 | Precursor mass: | 7671.7030 | Proteoform mass: | 7671.6980 |
| # matched peaks: | 35 | # matched fragment ions: | 30 | # unexpected modifications: | 1 |
| E-value: | 2.44e-26 | P-value: | 2.44e-26 | Q-value (Spectral FDR): | 0 |

  

|  |  |  |  |  |  |  |  |  |  |  |  |  |  |  |  |  |  |  |  |  |  |  |  |  |  |  |  |  |  |  |  |  |  |  |  |  |  |  |  |  |  |  |  |  |  |  |  |  |  |  |  |  |  |  |  |  |  |  |  |  |  |  |  |  |  |  |  |  |  |
| --- | --- | --- | --- | --- | --- | --- | --- | --- | --- | --- | --- | --- | --- | --- | --- | --- | --- | --- | --- | --- | --- | --- | --- | --- | --- | --- | --- | --- | --- | --- | --- | --- | --- | --- | --- | --- | --- | --- | --- | --- | --- | --- | --- | --- | --- | --- | --- | --- | --- | --- | --- | --- | --- | --- | --- | --- | --- | --- | --- | --- | --- | --- | --- | --- | --- | --- | --- | --- | --- |
|  | |  | | | | | | | | | | | | | | | | | | | | | | | | | | | | | | | | | | | | | | | | | | | | | | | | | | | | | | | | | | | | | | | | | | | |
| 1 |  |  | M |  | K |  | V |  | L |  | V |  | I |  | C |  | A |  | V |  | L |  |  | F |  | L |  | A |  | I |  | F |  | S |  | N |  | S |  | S |  | A |  |  | E |  | T |  | E |  | D |  | D |  | F |  | L |  | E |  | D |  | E |  | 30 |  |
|  | |  | | | | | | | | | | | | | | | | | | | | | | | | | | | | | | | | | | | | | | | | | | | | | | | | | | | | | | | | | | | | | | | | | | | |
| 31 |  |  | S |  | F |  | E |  | A |  | D |  | D |  | V |  | I |  | P |  | F |  |  | L |  | A |  | R |  | E |  | Q |  | V |  | R | ] | K |  | D |  | D |  |  | K | ⎫ | N | ⎫ | C |  | I |  | P | ⎫ | K | ⎫ | H |  | H |  | E | ⎫ | C |  | 60 |  |
|  | |  | | | | | | | | | | | | | | | | | | | | | | | | | | | | | | | | | | | | | | | | | | | | | | | | | | | | | | | | | | | | | | | | | | | |
| 61 |  |  | T | ⎫ | N | ⎫ | D | ⎱ | K |  | K | ⎫ | N | ⎫ | C | ⎫ | C |  | K | ⎫ | K |  | ⎫ | G |  | L |  | T | ⎱ | K | ⎱ | M | ⎩ | K |  | C |  | K |  | C |  | F |  |  | T |  | V |  | A | ⎩ | D |  | A |  | K | ⎫ | G | ⎩ | A | ⎱ | T | ⎩ | S |  | 90 |  |
|  | |  | | | | | | | | | | | | | | | | | | | | | | | | | | | | | | | | | | | | | | | | | | | | | -318.18 | | | | | | | | | | | | | | | | | | | |
| 91 |  |  | E |  | R | ⎫ | C |  | A |  | C |  | D |  | S |  | S | ⎱ | L |  | L |  |  | Q | ⎩ | K |  | F |  | G |  | F |  | T |  | G |  | L |  | H |  | I |  |  | I | ⎫ | K |  | G |  | L |  | F |  | | 115 |  | | | | | | | | | |

Fixed PTMs: Carbamidomethylation [C53 C60 C67 C68 C77 C79 C93 C95 ]   
  
     Unexpected modifications:   Unknown [-318.18]

  

All peaks (127)  Matched peaks (35)  Not matched peaks (92)

  

| Scan | Peak | Mono mass | Mono m/z | Intensity | Charge | Theoretical mass | Ion | Pos | Mass error | PPM error |
| --- | --- | --- | --- | --- | --- | --- | --- | --- | --- | --- |
| 541 | 1 | 3491.8097 | 699.3692 | 75032.36 | 5 |  |  |  |  |  |
| 541 | 2 | 3479.8335 | 696.9740 | 119966.62 | 5 |  |  |  |  |  |
| 541 | 3 | 7614.6431 | 847.0787 | 16914.60 | 9 |  |  |  |  |  |
| 541 | 4 | 3491.8173 | 873.9616 | 14425.53 | 4 |  |  |  |  |  |
| 541 | 5 | 7557.6207 | 945.7099 | 9817.00 | 8 |  |  |  |  |  |
| 541 | 6 | 7615.6456 | 952.9630 | 14548.75 | 8 |  |  |  |  |  |
| 541 | 7 | 697.2439 | 698.2512 | 36431.08 | 1 |  |  |  |  |  |
| 541 | 8 | 1392.7344 | 697.3745 | 107574.61 | 2 |  |  |  |  |  |
| 541 | 9 | 3422.8079 | 856.7093 | 18664.89 | 4 |  |  |  |  |  |
| 541 | 10 | 3384.8120 | 677.9697 | 5973.21 | 5 |  |  |  |  |  |
| 541 | 11 | 3680.8236 | 921.2132 | 6153.23 | 4 |  |  |  |  |  |
| 541 | 12 | 7557.6189 | 1080.6671 | 6374.43 | 7 |  |  |  |  |  |
| 541 | 13 | 2187.1382 | 730.0533 | 7062.92 | 3 |  |  |  |  |  |
| 541 | 14 | 1779.8017 | 890.9081 | 5959.10 | 2 | 1779.8144 | C14 | 14 | -0.0127 | -7.13 |
| 541 | 15 | 2008.8726 | 670.6315 | 4813.98 | 3 | 2008.8843 | C16 | 16 | -0.0117 | -5.83 |
| 541 | 16 | 7613.6379 | 762.3711 | 7269.78 | 10 |  |  |  |  |  |
| 541 | 17 | 3434.7968 | 859.7065 | 8683.33 | 4 |  |  |  |  |  |
| 541 | 18 | 3354.5917 | 671.9256 | 4950.84 | 5 | 3354.6165 | C27 | 27 | -0.0248 | -7.40 |
| 541 | 19 | 7557.6228 | 840.7431 | 7165.51 | 9 |  |  |  |  |  |
| 541 | 20 | 4446.1695 | 890.2412 | 4390.37 | 5 | 4446.1843 | Z\_DOT42 | 26 | -0.0148 | -3.33 |
| 541 | 21 | 1627.8741 | 814.9443 | 5226.50 | 2 |  |  |  |  |  |
| 541 | 22 | 7630.6446 | 954.8378 | 3921.35 | 8 |  |  |  |  |  |
| 541 | 23 | 3392.7704 | 849.1999 | 5242.10 | 4 |  |  |  |  |  |
| 541 | 24 | 3836.8360 | 960.2163 | 2975.47 | 4 |  |  |  |  |  |
| 541 | 25 | 3355.6024 | 839.9079 | 3077.28 | 4 |  |  |  |  |  |
| 541 | 26 | 1596.9529 | 799.4837 | 4188.75 | 2 | 1596.9456 | Z\_DOT17 | 51 | 7.31e-03 | 4.58 |
| 541 | 27 | 7665.8263 | 697.9006 | 4472.56 | 11 |  |  |  |  |  |
| 541 | 28 | 2187.1372 | 1094.5759 | 4717.76 | 2 |  |  |  |  |  |
| 541 | 29 | 2750.3547 | 917.7922 | 3165.39 | 3 | 2750.3584 | Z\_DOT27 | 41 | -3.75e-03 | -1.36 |
| 541 | 30 | 3835.8335 | 768.1740 | 6996.63 | 5 |  |  |  |  |  |
| 541 | 31 | 1893.8442 | 632.2887 | 3361.02 | 3 | 1893.8573 | C15 | 15 | -0.0131 | -6.91 |
| 541 | 32 | 368.1130 | 369.1203 | 6090.35 | 1 |  |  |  |  |  |
| 541 | 33 | 3968.9459 | 993.2437 | 3791.36 | 4 |  |  |  |  |  |
| 541 | 34 | 7598.6319 | 950.8363 | 3806.81 | 8 |  |  |  |  |  |
| 541 | 35 | 3029.6537 | 1010.8918 | 5019.92 | 3 |  |  |  |  |  |
| 541 | 36 | 3144.6827 | 1049.2349 | 3236.31 | 3 |  |  |  |  |  |
| 541 | 37 | 4318.0826 | 1080.5279 | 4082.27 | 4 | 4318.0893 | Z\_DOT41 | 27 | -6.79e-03 | -1.57 |
| 541 | 38 | 3455.7910 | 692.1655 | 3767.57 | 5 |  |  |  |  |  |
| 541 | 39 | 4885.7009 | 698.9645 | 23103.68 | 7 |  |  |  |  |  |
| 541 | 40 | 3144.6822 | 787.1778 | 5840.35 | 4 |  |  |  |  |  |
| 541 | 41 | 4481.1050 | 747.8581 | 2805.80 | 6 |  |  |  |  |  |
| 541 | 42 | 1610.8537 | 806.4341 | 3143.97 | 2 |  |  |  |  |  |
| 541 | 43 | 4794.2600 | 800.0506 | 3309.77 | 6 | 4794.2889 | C39 | 39 | -0.0289 | -6.02 |
| 541 | 44 | 2379.1023 | 794.0414 | 4749.51 | 3 | 2379.1171 | C19 | 19 | -0.0148 | -6.23 |
| 541 | 45 | 5395.5283 | 900.2620 | 3193.60 | 6 | 5395.5708 | C45 | 45 | -0.0425 | -7.88 |
| 541 | 46 | 5202.5704 | 868.1023 | 2615.53 | 6 |  |  |  |  |  |
| 541 | 47 | 1892.8433 | 947.4289 | 4019.74 | 2 |  |  |  |  |  |
| 541 | 48 | 2265.0604 | 756.0274 | 2902.35 | 3 | 2265.0742 | C18 | 18 | -0.0138 | -6.10 |
| 541 | 49 | 1518.7268 | 760.3707 | 3473.31 | 2 | 1518.7361 | C12 | 12 | -9.23e-03 | -6.08 |
| 541 | 50 | 7570.6504 | 947.3386 | 4795.04 | 8 |  |  |  |  |  |
| 541 | 51 | 6280.8763 | 698.8824 | 1835.56 | 9 |  |  |  |  |  |
| 541 | 52 | 3226.5012 | 646.3075 | 2577.11 | 5 | 3226.5216 | C26 | 26 | -0.0203 | -6.29 |
| 541 | 53 | 2649.3108 | 884.1109 | 2542.62 | 3 | 2649.3108 | Z\_DOT26 | 42 | 9.41e-05 | 0.04 |
| 541 | 54 | 3899.9216 | 975.9877 | 3270.85 | 4 |  |  |  |  |  |
| 541 | 55 | 5395.5442 | 771.7993 | 2471.40 | 7 | 5395.5708 | C45 | 45 | -0.0267 | -4.94 |
| 541 | 56 | 5663.8012 | 944.9741 | 2470.94 | 6 | 5663.8216 | Z\_DOT52 | 16 | -0.0204 | -3.60 |
| 541 | 57 | 2692.3269 | 898.4496 | 1847.84 | 3 |  |  |  |  |  |
| 541 | 58 | 2821.3936 | 941.4718 | 2240.88 | 3 | 2821.3955 | Z\_DOT28 | 40 | -1.98e-03 | -0.70 |
| 541 | 59 | 7571.6284 | 1082.6685 | 2712.98 | 7 |  |  |  |  |  |
| 541 | 60 | 2539.1257 | 847.3825 | 2505.90 | 3 | 2539.1478 | C20 | 20 | -0.0221 | -8.71 |
| 541 | 61 | 7629.6515 | 848.7463 | 4097.54 | 9 |  |  |  |  |  |
| 541 | 62 | 2558.5572 | 853.8597 | 3310.38 | 3 |  |  |  |  |  |
| 541 | 63 | 2879.4191 | 960.8136 | 3083.54 | 3 |  |  |  |  |  |
| 541 | 64 | 2785.2640 | 697.3233 | 27119.87 | 4 |  |  |  |  |  |
| 541 | 65 | 2803.4606 | 935.4941 | 3519.56 | 3 |  |  |  |  |  |
| 541 | 66 | 3456.7968 | 865.2065 | 2056.47 | 4 |  |  |  |  |  |
| 541 | 67 | 1740.4168 | 871.2157 | 21441.30 | 2 |  |  |  |  |  |
| 541 | 68 | 2901.5637 | 968.1952 | 1432.54 | 3 |  |  |  |  |  |
| 541 | 69 | 3492.8234 | 1165.2818 | 2278.73 | 3 |  |  |  |  |  |
| 541 | 70 | 1553.8987 | 777.9566 | 2694.91 | 2 |  |  |  |  |  |
| 541 | 71 | 220.0764 | 221.0837 | 5015.35 | 1 |  |  |  |  |  |
| 541 | 72 | 7543.5888 | 839.1838 | 2196.88 | 9 | 7543.6204 | C64 | 64 | -0.0316 | -4.19 |
| 541 | 73 | 680.2175 | 681.2248 | 3139.95 | 1 |  |  |  |  |  |
| 541 | 74 | 2008.8719 | 1005.4432 | 2316.35 | 2 | 2008.8843 | C16 | 16 | -0.0124 | -6.15 |
| 541 | 75 | 3404.7600 | 852.1973 | 2007.03 | 4 |  |  |  |  |  |
| 541 | 76 | 5576.0466 | 698.0131 | 7120.01 | 8 |  |  |  |  |  |
| 541 | 77 | 4922.3120 | 704.1947 | 1600.52 | 7 | 4922.3474 | C41 | 41 | -0.0354 | -7.20 |
| 541 | 78 | 3226.4992 | 807.6321 | 1973.92 | 4 | 3226.5216 | C26 | 26 | -0.0224 | -6.94 |
| 541 | 79 | 3900.8455 | 651.1482 | 2886.59 | 6 |  |  |  |  |  |
| 541 | 80 | 3319.7323 | 830.9404 | 1370.13 | 4 |  |  |  |  |  |
| 541 | 81 | 294.0947 | 295.1020 | 4516.31 | 1 |  |  |  |  |  |
| 541 | 82 | 1221.6839 | 611.8492 | 1289.31 | 2 |  |  |  |  |  |
| 541 | 83 | 3422.8050 | 1141.9423 | 2943.48 | 3 |  |  |  |  |  |
| 541 | 84 | 3902.8585 | 781.5790 | 1984.78 | 5 |  |  |  |  |  |
| 541 | 85 | 3192.5784 | 1065.2001 | 3010.12 | 3 | 3192.5760 | Z\_DOT32 | 36 | 2.35e-03 | 0.74 |
| 541 | 86 | 4852.2761 | 809.7200 | 1120.35 | 6 |  |  |  |  |  |
| 541 | 87 | 1258.7477 | 630.3811 | 1890.17 | 2 |  |  |  |  |  |
| 541 | 88 | 3614.7234 | 904.6881 | 1447.38 | 4 |  |  |  |  |  |
| 541 | 89 | 763.4800 | 764.4872 | 1747.01 | 1 |  |  |  |  |  |
| 541 | 90 | 2955.3479 | 739.8443 | 2283.83 | 4 | 2955.3684 | C23 | 23 | -0.0204 | -6.91 |
| 541 | 91 | 5607.7812 | 935.6375 | 1584.33 | 6 |  |  |  |  |  |
| 541 | 92 | 2344.2962 | 782.4393 | 1303.91 | 3 |  |  |  |  |  |
| 541 | 93 | 1869.9802 | 935.9974 | 3190.85 | 2 |  |  |  |  |  |
| 541 | 94 | 3156.6659 | 1053.2292 | 1266.06 | 3 |  |  |  |  |  |
| 541 | 95 | 2118.1840 | 1060.0993 | 1302.46 | 2 |  |  |  |  |  |
| 541 | 96 | 3157.6714 | 790.4251 | 1786.10 | 4 |  |  |  |  |  |
| 541 | 97 | 4318.0726 | 864.6218 | 2832.65 | 5 | 4318.0893 | Z\_DOT41 | 27 | -0.0168 | -3.89 |
| 541 | 98 | 503.2677 | 504.2750 | 1314.62 | 1 | 503.2703 | C4 | 4 | -2.55e-03 | -5.07 |
| 541 | 99 | 1826.9725 | 914.4935 | 1562.20 | 2 |  |  |  |  |  |
| 541 | 100 | 1489.7478 | 745.8812 | 1601.10 | 2 |  |  |  |  |  |
| 541 | 101 | 2827.2508 | 707.8200 | 1534.40 | 4 | 2827.2734 | C22 | 22 | -0.0226 | -8.00 |
| 541 | 102 | 4203.0591 | 1051.7721 | 1750.00 | 4 |  |  |  |  |  |
| 541 | 103 | 617.3097 | 618.3170 | 3346.21 | 1 | 617.3132 | C5 | 5 | -3.49e-03 | -5.65 |
| 541 | 104 | 4187.0386 | 1047.7669 | 1305.73 | 4 | 4187.0488 | Z\_DOT40 | 28 | -0.0102 | -2.45 |
| 541 | 105 | 3008.8516 | 1003.9578 | 1466.89 | 3 |  |  |  |  |  |
| 541 | 106 | 542.3024 | 543.3096 | 1155.56 | 1 |  |  |  |  |  |
| 541 | 107 | 5781.8254 | 826.9823 | 1580.06 | 7 |  |  |  |  |  |
| 541 | 108 | 3192.5718 | 799.1502 | 1189.63 | 4 | 3192.5760 | Z\_DOT32 | 36 | -4.19e-03 | -1.31 |
| 541 | 109 | 2966.8355 | 989.9525 | 1265.27 | 3 |  |  |  |  |  |
| 541 | 110 | 2461.1564 | 821.3928 | 733.28 | 3 |  |  |  |  |  |
| 541 | 111 | 6076.7222 | 869.1104 | 1486.14 | 7 | 6075.7603 | C51 | 51 | -0.0404 | -6.65 |
| 541 | 112 | 3734.6070 | 934.6590 | 794.31 | 4 |  |  |  |  |  |
| 541 | 113 | 4661.2972 | 933.2667 | 1252.07 | 5 |  |  |  |  |  |
| 541 | 114 | 2767.3766 | 923.4661 | 1366.54 | 3 |  |  |  |  |  |
| 541 | 115 | 586.3769 | 587.3842 | 1027.74 | 1 |  |  |  |  |  |
| 541 | 116 | 3041.6443 | 1014.8887 | 1892.62 | 3 |  |  |  |  |  |
| 541 | 117 | 841.9603 | 842.9676 | 728.82 | 1 |  |  |  |  |  |
| 541 | 118 | 473.2937 | 474.3010 | 772.55 | 1 |  |  |  |  |  |
| 541 | 119 | 987.4778 | 988.4850 | 892.30 | 1 | 987.4807 | C8 | 8 | -2.93e-03 | -2.97 |
| 541 | 120 | 827.4386 | 828.4459 | 2112.33 | 1 |  |  |  |  |  |
| 541 | 121 | 1077.8003 | 1078.8076 | 576.32 | 1 |  |  |  |  |  |
| 541 | 122 | 868.0990 | 869.1063 | 421.86 | 1 |  |  |  |  |  |
| 541 | 123 | 1115.5696 | 558.7921 | 731.31 | 2 | 1115.5757 | C9 | 9 | -6.05e-03 | -5.42 |
| 541 | 124 | 1242.7292 | 622.3719 | 776.18 | 2 | 1242.7189 | Z\_DOT14 | 54 | 0.0103 | 8.25 |
| 541 | 125 | 1009.4834 | 1010.4907 | 544.19 | 1 |  |  |  |  |  |
| 541 | 126 | 909.8442 | 910.8514 | 549.36 | 1 |  |  |  |  |  |
| 541 | 127 | 1139.9336 | 1140.9409 | 399.16 | 1 |  |  |  |  |  |

  

All proteins /
CsTx-9a Cupiennius salei toxin 9 isoform a /
Proteoform #10
