# Supplementary material for: The Dual Prey-Inactivation Strategy of Spiders—In-Depth Venomic Analysis of Cupiennius salei
Source: Toxins (Basel). 2019 Mar 19;11(3):167. doi: 10.3390/toxins11030167 (PMC6468893; doi:10.3390/toxins11030167)
Supplement: Supplementary file 1 [file toxins-11-00167-s001.zip › Supplementary Dataset EV1/20180328_f2_topdown_OTMS2_EThcD_NL_i02_ms2_proteoform_cutoff_html/prsms/prsm166.html]

Protein-Spectrum-Match for Spectrum #405


All proteins /
CsTx-1a\_S1 Cupiennius salei toxin 1 isoform a S1^ACsTx-1a\_S2 Cupiennius salei toxin 1 isoform a S2 /
Proteoform #15

## Protein-Spectrum-Match #166 for Spectrum #405

|  |  |  |  |  |  |
| --- | --- | --- | --- | --- | --- |
| PrSM ID: | 166 | Scan(s): | 543 | Precursor charge: | 12 |
| Precursor m/z: | 736.5274 | Precursor mass: | 8826.2415 | Proteoform mass: | 8826.2115 |
| # matched peaks: | 46 | # matched fragment ions: | 36 | # unexpected modifications: | 1 |
| E-value: | 6.29e-31 | P-value: | 6.29e-31 | Q-value (Spectral FDR): | 0 |

  

|  |  |  |  |  |  |  |  |  |  |  |  |  |  |  |  |  |  |  |  |  |  |  |  |  |  |  |  |  |  |  |  |  |  |  |  |  |  |  |  |  |  |  |  |  |  |  |  |  |  |  |  |  |  |  |  |  |  |  |  |  |  |  |  |  |  |  |  |  |  |
| --- | --- | --- | --- | --- | --- | --- | --- | --- | --- | --- | --- | --- | --- | --- | --- | --- | --- | --- | --- | --- | --- | --- | --- | --- | --- | --- | --- | --- | --- | --- | --- | --- | --- | --- | --- | --- | --- | --- | --- | --- | --- | --- | --- | --- | --- | --- | --- | --- | --- | --- | --- | --- | --- | --- | --- | --- | --- | --- | --- | --- | --- | --- | --- | --- | --- | --- | --- | --- | --- |
|  | |  | | | | | | | | | | | | | | | | | | | | | | | | | | | | | | | | | | | | | | | | | | | | | | | | | | | | | | | | | | | | | | | | | | | |
| 1 |  |  | M |  | K |  | V |  | L |  | I |  | I |  | S |  | A |  | V |  | L |  |  | F |  | I |  | T |  | I |  | F |  | S |  | N |  | I |  | S |  | A |  |  | E |  | I |  | E |  | D |  | D |  | F |  | L |  | E |  | D |  | E |  | 30 |  |
|  | |  | | | | | | | | | | | | | | | | | | | | | | | | | | | | | | | | | | | | | | | | | | | | | | | | | | | | | | | | | | | | | | | | | | | |
| 31 |  |  | S |  | F |  | E |  | A |  | E |  | D |  | I |  | I |  | P |  | F |  |  | F |  | E |  | N |  | E |  | Q |  | A |  | R | ] | S | ⎩ | C |  | I |  |  | P | ⎫ | K |  | H |  | E |  | E | ⎫ | C |  | T | ⎫ | N | ⎱ | D |  | K |  | 60 |  |
|  | |  | | | | | | | | | | | | | | | | | | | | | | | | | | | | | | | | | | | | | | | | | | | | | | | | | | | | | | | | | | | | | | | | | | | |
| 61 |  |  | H | ⎱ | N | ⎫ | C | ⎫ | C |  | R |  | K |  | G | ⎫ | L | ⎫ | F | ⎱ | K |  |  | L |  | K | ⎫ | C |  | Q | ⎫ | C |  | S |  | T |  | F | ⎫ | D | ⎫ | D |  | ⎫ | E | ⎱ | S |  | G | ⎱ | Q |  | P |  | T | ⎫ | E | ⎫ | R |  | C |  | A |  | 90 |  |
|  | |  | | | | | -42.05 | | | | | | | | | | | | | | | | | | | | | | | | | | | | | | | | | | | | | | | | | | | | | | | | | | | | | | | | | | | |
| 91 |  |  | C | ⎩ | G | ⎱ | R |  | P |  | M |  | G |  | H |  | Q |  | A |  | I |  |  | E |  | T |  | G |  | L |  | N |  | I |  | F |  | R |  | G | ⎫ | L |  |  | F |  | K |  | G | ⎫ | K | ⎫ | K | ⎫ | K | ⎫ | N | ⎫ | K |  | K |  | T |  | 120 |  |
|  | |  | | | | | | | | | | | | | | | | | | | | | | | | | | | | | | | | | | | | | | | | | | | | | | | | | | | | | | | | | | | | | | | | | | | |
| 121 |  | ⎫ | K | ⎫ | G |  | | | | 122 |  | | | | | | | | | | | | | | | | | | | | | | | | | | | | | | | | | | | | | | | | | | | | | | | | | | | | | | | |

Fixed PTMs: Carbamidomethylation [C49 C56 C63 C64 C73 C75 C89 C91 ]   
  
     Unexpected modifications:   Unknown [-42.05]

  

All peaks (147)  Matched peaks (46)  Not matched peaks (101)

  

| Scan | Peak | Mono mass | Mono m/z | Intensity | Charge | Theoretical mass | Ion | Pos | Mass error | PPM error |
| --- | --- | --- | --- | --- | --- | --- | --- | --- | --- | --- |
| 543 | 1 | 8769.1833 | 877.9256 | 165608.10 | 10 | 8768.2060 | C74 | 74 | -0.0250 | -2.85 |
| 543 | 2 | 8770.1838 | 975.4721 | 124394.41 | 9 |  |  |  |  |  |
| 543 | 3 | 8769.1790 | 798.2054 | 99838.97 | 11 | 8768.2060 | C74 | 74 | -0.0293 | -3.34 |
| 543 | 4 | 8811.1913 | 882.1264 | 64391.04 | 10 |  |  |  |  |  |
| 543 | 5 | 8783.1878 | 879.3261 | 65548.68 | 10 |  |  |  |  |  |
| 543 | 6 | 8712.1551 | 969.0245 | 48372.44 | 9 |  |  |  |  |  |
| 543 | 7 | 8770.1937 | 1097.2815 | 42677.79 | 8 |  |  |  |  |  |
| 543 | 8 | 8754.1745 | 973.6933 | 41613.77 | 9 |  |  |  |  |  |
| 543 | 9 | 4414.1034 | 883.8280 | 100438.36 | 5 |  |  |  |  |  |
| 543 | 10 | 8811.1897 | 980.0284 | 38580.54 | 9 |  |  |  |  |  |
| 543 | 11 | 8784.1934 | 977.0288 | 41012.77 | 9 |  |  |  |  |  |
| 543 | 12 | 8726.1621 | 970.5808 | 39570.17 | 9 |  |  |  |  |  |
| 543 | 13 | 8711.1547 | 872.1228 | 38994.27 | 10 |  |  |  |  |  |
| 543 | 14 | 4443.9106 | 889.7894 | 40381.62 | 5 | 4443.9333 | C36 | 36 | -0.0228 | -5.12 |
| 543 | 15 | 8810.1833 | 801.9330 | 27608.44 | 11 |  |  |  |  |  |
| 543 | 16 | 8782.1878 | 799.3880 | 28910.71 | 11 |  |  |  |  |  |
| 543 | 17 | 8753.1683 | 876.3241 | 31576.04 | 10 |  |  |  |  |  |
| 543 | 18 | 8726.1703 | 1091.7786 | 24481.33 | 8 |  |  |  |  |  |
| 543 | 19 | 4413.0981 | 736.5236 | 37270.75 | 6 |  |  |  |  |  |
| 543 | 20 | 8697.1118 | 870.7185 | 26897.77 | 10 |  |  |  |  |  |
| 543 | 21 | 8725.1806 | 873.5253 | 26647.70 | 10 |  |  |  |  |  |
| 543 | 22 | 8754.1703 | 1095.2786 | 23749.44 | 8 |  |  |  |  |  |
| 543 | 23 | 4770.0711 | 955.0215 | 21996.96 | 5 | 4770.0923 | C39 | 39 | -0.0212 | -4.45 |
| 543 | 24 | 8697.1199 | 967.3539 | 24765.95 | 9 |  |  |  |  |  |
| 543 | 25 | 5926.9054 | 847.7080 | 17220.17 | 7 |  |  |  |  |  |
| 543 | 26 | 3157.5020 | 790.3828 | 23033.15 | 4 | 3157.5153 | C25 | 25 | -0.0133 | -4.23 |
| 543 | 27 | 8712.1541 | 1090.0265 | 29211.47 | 8 |  |  |  |  |  |
| 543 | 28 | 4325.2730 | 721.8861 | 22740.60 | 6 |  |  |  |  |  |
| 543 | 29 | 3323.8864 | 831.9789 | 24804.10 | 4 | 3323.8634 | Z\_DOT30 | 45 | 0.0230 | 6.92 |
| 543 | 30 | 3998.1291 | 667.3621 | 15448.30 | 6 |  |  |  |  |  |
| 543 | 31 | 8341.8808 | 927.8829 | 21012.07 | 9 |  |  |  |  |  |
| 543 | 32 | 8784.1953 | 1099.0317 | 15989.82 | 8 |  |  |  |  |  |
| 543 | 33 | 8738.1801 | 971.9162 | 16052.44 | 9 |  |  |  |  |  |
| 543 | 34 | 2788.2289 | 930.4169 | 19444.71 | 3 | 2788.2414 | C22 | 22 | -0.0124 | -4.46 |
| 543 | 35 | 8812.2083 | 1102.5333 | 14566.30 | 8 |  |  |  |  |  |
| 543 | 36 | 8226.8195 | 915.0983 | 13489.05 | 9 |  |  |  |  |  |
| 543 | 37 | 2942.0689 | 736.5245 | 27900.41 | 4 |  |  |  |  |  |
| 543 | 38 | 6870.4032 | 859.8077 | 14094.85 | 8 |  |  |  |  |  |
| 543 | 39 | 6226.6530 | 890.5291 | 13450.63 | 7 |  |  |  |  |  |
| 543 | 40 | 7970.6320 | 886.6331 | 14786.07 | 9 |  |  |  |  |  |
| 543 | 41 | 3445.5868 | 862.4040 | 15765.46 | 4 | 3445.6046 | C27 | 27 | -0.0178 | -5.16 |
| 543 | 42 | 1752.7603 | 877.3874 | 30204.27 | 2 | 1752.7671 | C14 | 14 | -6.84e-03 | -3.90 |
| 543 | 43 | 5503.3287 | 918.2287 | 16804.95 | 6 | 5503.3559 | C45 | 45 | -0.0272 | -4.94 |
| 543 | 44 | 4384.3035 | 877.8680 | 21977.49 | 5 |  |  |  |  |  |
| 543 | 45 | 8751.1650 | 796.5677 | 15151.46 | 11 |  |  |  |  |  |
| 543 | 46 | 2203.3696 | 735.4638 | 21292.61 | 3 |  |  |  |  |  |
| 543 | 47 | 5579.7093 | 930.9588 | 18593.09 | 6 |  |  |  |  |  |
| 543 | 48 | 6097.5952 | 1017.2731 | 13104.56 | 6 |  |  |  |  |  |
| 543 | 49 | 7341.2643 | 918.6653 | 13232.39 | 8 |  |  |  |  |  |
| 543 | 50 | 5926.9109 | 988.8258 | 14433.32 | 6 |  |  |  |  |  |
| 543 | 51 | 4554.9412 | 760.1641 | 10022.62 | 6 |  |  |  |  |  |
| 543 | 52 | 4299.8574 | 860.9788 | 13757.38 | 5 | 4299.8798 | C34 | 34 | -0.0224 | -5.22 |
| 543 | 53 | 2943.0726 | 982.0315 | 24764.07 | 3 |  |  |  |  |  |
| 543 | 54 | 8226.8593 | 1029.3647 | 12887.94 | 8 |  |  |  |  |  |
| 543 | 55 | 4271.2612 | 712.8841 | 11508.57 | 6 |  |  |  |  |  |
| 543 | 56 | 2288.3959 | 763.8059 | 14293.86 | 3 |  |  |  |  |  |
| 543 | 57 | 4170.8153 | 835.1703 | 9622.42 | 5 | 4170.8372 | C33 | 33 | -0.0219 | -5.25 |
| 543 | 58 | 8226.8360 | 823.6909 | 11240.88 | 10 |  |  |  |  |  |
| 543 | 59 | 4383.3035 | 731.5579 | 11546.80 | 6 | 4383.2860 | Z\_DOT39 | 36 | 0.0175 | 3.99 |
| 543 | 60 | 4443.9123 | 1111.9853 | 10995.81 | 4 | 4443.9333 | C36 | 36 | -0.0210 | -4.73 |
| 543 | 61 | 7912.6060 | 990.0830 | 11692.11 | 8 | 7912.6405 | C67 | 67 | -0.0346 | -4.37 |
| 543 | 62 | 1470.5808 | 736.2977 | 18455.47 | 2 |  |  |  |  |  |
| 543 | 63 | 8098.7353 | 1013.3492 | 12980.46 | 8 |  |  |  |  |  |
| 543 | 64 | 8340.8747 | 835.0947 | 12592.68 | 10 |  |  |  |  |  |
| 543 | 65 | 8668.1307 | 964.1329 | 9710.70 | 9 |  |  |  |  |  |
| 543 | 66 | 8283.8506 | 1036.4886 | 10854.17 | 8 | 8282.8734 | C70 | 70 | -0.0251 | -3.03 |
| 543 | 67 | 8668.1322 | 1084.5238 | 9278.27 | 8 |  |  |  |  |  |
| 543 | 68 | 3450.9354 | 691.1943 | 9580.21 | 5 |  |  |  |  |  |
| 543 | 69 | 5291.6310 | 882.9458 | 12028.08 | 6 |  |  |  |  |  |
| 543 | 70 | 8098.7390 | 900.8672 | 17622.58 | 9 |  |  |  |  |  |
| 543 | 71 | 8641.0980 | 1081.1445 | 11765.64 | 8 | 8640.1110 | C73 | 73 | -0.0153 | -1.77 |
| 543 | 72 | 3323.8840 | 665.7841 | 11599.02 | 5 | 3323.8634 | Z\_DOT30 | 45 | 0.0206 | 6.20 |
| 543 | 73 | 2528.0727 | 843.6982 | 12161.85 | 3 | 2528.0889 | C20 | 20 | -0.0162 | -6.41 |
| 543 | 74 | 3683.0004 | 737.6074 | 12692.97 | 5 |  |  |  |  |  |
| 543 | 75 | 6870.4087 | 764.3860 | 8736.12 | 9 |  |  |  |  |  |
| 543 | 76 | 1866.8007 | 934.4077 | 19032.20 | 2 | 1866.8101 | C15 | 15 | -9.31e-03 | -4.99 |
| 543 | 77 | 6538.8108 | 935.1231 | 9298.11 | 7 |  |  |  |  |  |
| 543 | 78 | 4055.7909 | 812.1655 | 10283.78 | 5 | 4055.8103 | C32 | 32 | -0.0194 | -4.77 |
| 543 | 79 | 7340.2652 | 1049.6166 | 10544.02 | 7 | 7339.2767 | C62 | 62 | -0.0138 | -1.88 |
| 543 | 80 | 2617.5782 | 655.4018 | 14504.21 | 4 |  |  |  |  |  |
| 543 | 81 | 5503.3251 | 1101.6723 | 9621.17 | 5 | 5503.3559 | C45 | 45 | -0.0308 | -5.59 |
| 543 | 82 | 8640.0822 | 961.0164 | 12978.58 | 9 | 8640.1110 | C73 | 73 | -0.0288 | -3.33 |
| 543 | 83 | 6710.3754 | 839.8042 | 11236.94 | 8 |  |  |  |  |  |
| 543 | 84 | 8041.7110 | 1006.2212 | 10321.05 | 8 | 8040.7355 | C68 | 68 | -0.0268 | -3.34 |
| 543 | 85 | 8655.1156 | 1082.8967 | 8085.70 | 8 |  |  |  |  |  |
| 543 | 86 | 2641.1584 | 881.3934 | 11104.79 | 3 | 2641.1730 | C21 | 21 | -0.0145 | -5.50 |
| 543 | 87 | 2671.5894 | 668.9046 | 10538.02 | 4 |  |  |  |  |  |
| 543 | 88 | 4456.3411 | 743.7308 | 8184.12 | 6 |  |  |  |  |  |
| 543 | 89 | 8097.7191 | 810.7792 | 7315.80 | 10 |  |  |  |  |  |
| 543 | 90 | 7454.6446 | 932.8379 | 12292.45 | 8 | 7454.6330 | Z\_DOT64 | 11 | 0.0116 | 1.56 |
| 543 | 91 | 7740.7525 | 775.0825 | 7992.50 | 10 |  |  |  |  |  |
| 543 | 92 | 8241.9155 | 916.7757 | 11741.44 | 9 |  |  |  |  |  |
| 543 | 93 | 2187.3530 | 730.1249 | 9287.95 | 3 |  |  |  |  |  |
| 543 | 94 | 1169.7796 | 585.8971 | 14964.04 | 2 |  |  |  |  |  |
| 543 | 95 | 6038.9848 | 863.7194 | 10735.63 | 7 | 6038.9779 | Z\_DOT53 | 22 | 6.86e-03 | 1.14 |
| 543 | 96 | 4899.1074 | 980.8288 | 9240.64 | 5 | 4899.1349 | C40 | 40 | -0.0275 | -5.61 |
| 543 | 97 | 7075.4493 | 1011.7857 | 9123.07 | 7 | 7074.4522 | Z\_DOT61 | 14 | -5.22e-03 | -0.74 |
| 543 | 98 | 4414.1125 | 1104.5354 | 9267.45 | 4 |  |  |  |  |  |
| 543 | 99 | 856.5696 | 857.5769 | 11652.74 | 1 |  |  |  |  |  |
| 543 | 100 | 8681.1510 | 965.5796 | 12965.48 | 9 |  |  |  |  |  |
| 543 | 101 | 3940.7596 | 986.1972 | 9265.79 | 4 | 3940.7834 | C31 | 31 | -0.0238 | -6.03 |
| 543 | 102 | 8597.0625 | 1075.6401 | 8638.02 | 8 |  |  |  |  |  |
| 543 | 103 | 3157.4979 | 1053.5066 | 10239.39 | 3 | 3157.5153 | C25 | 25 | -0.0174 | -5.52 |
| 543 | 104 | 8168.8232 | 1022.1102 | 10036.87 | 8 | 8168.8305 | C69 | 69 | -7.24e-03 | -0.89 |
| 543 | 105 | 7076.4589 | 885.5646 | 10683.09 | 8 |  |  |  |  |  |
| 543 | 106 | 4055.7928 | 1014.9555 | 9166.51 | 4 | 4055.8103 | C32 | 32 | -0.0175 | -4.32 |
| 543 | 107 | 8284.8050 | 921.5412 | 10667.66 | 9 |  |  |  |  |  |
| 543 | 108 | 7785.5215 | 866.0652 | 7232.25 | 9 | 7784.5456 | C66 | 66 | -0.0265 | -3.40 |
| 543 | 109 | 2470.0540 | 824.3586 | 10157.36 | 3 |  |  |  |  |  |
| 543 | 110 | 8739.1742 | 1093.4040 | 11155.40 | 8 |  |  |  |  |  |
| 543 | 111 | 7684.7412 | 854.8674 | 9868.90 | 9 |  |  |  |  |  |
| 543 | 112 | 5525.7004 | 790.3931 | 6470.38 | 7 |  |  |  |  |  |
| 543 | 113 | 1372.5800 | 687.2973 | 10609.46 | 2 | 1372.5863 | C11 | 11 | -6.33e-03 | -4.61 |
| 543 | 114 | 6870.3998 | 982.4930 | 25631.95 | 7 |  |  |  |  |  |
| 543 | 115 | 8681.1560 | 1086.1518 | 9748.75 | 8 |  |  |  |  |  |
| 543 | 116 | 1486.9517 | 744.4831 | 9730.77 | 2 |  |  |  |  |  |
| 543 | 117 | 8723.1850 | 794.0241 | 6923.56 | 11 | 8723.1608 | Z\_DOT74 | 1 | 0.0242 | 2.77 |
| 543 | 118 | 4299.8550 | 1075.9710 | 6714.18 | 4 | 4299.8798 | C34 | 34 | -0.0248 | -5.77 |
| 543 | 119 | 2866.6827 | 956.5682 | 8658.88 | 3 |  |  |  |  |  |
| 543 | 120 | 6710.3830 | 746.6054 | 5595.56 | 9 |  |  |  |  |  |
| 543 | 121 | 3380.9059 | 846.2337 | 8325.26 | 4 | 3380.8849 | Z\_DOT31 | 44 | 0.0210 | 6.22 |
| 543 | 122 | 7455.6348 | 829.4111 | 6740.65 | 9 | 7454.6330 | Z\_DOT64 | 11 | -5.26e-04 | -0.07 |
| 543 | 123 | 4527.3530 | 755.5661 | 7358.03 | 6 | 4527.3395 | Z\_DOT41 | 34 | 0.0136 | 2.99 |
| 543 | 124 | 6809.9710 | 852.2537 | 6382.63 | 8 |  |  |  |  |  |
| 543 | 125 | 2026.8324 | 1014.4235 | 10470.79 | 2 | 2026.8407 | C16 | 16 | -8.35e-03 | -4.12 |
| 543 | 126 | 8469.9770 | 942.1158 | 9118.72 | 9 |  |  |  |  |  |
| 543 | 127 | 5524.6971 | 921.7901 | 7886.85 | 6 |  |  |  |  |  |
| 543 | 128 | 997.4593 | 998.4666 | 9771.40 | 1 | 997.4651 | C8 | 8 | -5.72e-03 | -5.73 |
| 543 | 129 | 1428.8865 | 477.3028 | 5069.05 | 3 |  |  |  |  |  |
| 543 | 130 | 802.8382 | 803.8455 | 46004.20 | 1 |  |  |  |  |  |
| 543 | 131 | 728.4754 | 729.4827 | 9518.76 | 1 |  |  |  |  |  |
| 543 | 132 | 1372.5803 | 1373.5876 | 3221.16 | 1 | 1372.5863 | C11 | 11 | -6.01e-03 | -4.38 |
| 543 | 133 | 600.3811 | 601.3884 | 6825.26 | 1 |  |  |  |  |  |
| 543 | 134 | 1258.5372 | 1259.5445 | 3673.82 | 1 | 1258.5434 | C10 | 10 | -6.17e-03 | -4.90 |
| 543 | 135 | 1098.7061 | 550.3603 | 2245.67 | 2 |  |  |  |  |  |
| 543 | 136 | 953.4463 | 954.4536 | 4043.12 | 1 |  |  |  |  |  |
| 543 | 137 | 428.2732 | 429.2805 | 2961.10 | 1 |  |  |  |  |  |
| 543 | 138 | 1185.7980 | 593.9063 | 4307.07 | 2 |  |  |  |  |  |
| 543 | 139 | 502.2430 | 503.2502 | 3364.06 | 1 |  |  |  |  |  |
| 543 | 140 | 894.4352 | 895.4425 | 3983.70 | 1 |  |  |  |  |  |
| 543 | 141 | 1386.8754 | 463.2991 | 1841.88 | 3 |  |  |  |  |  |
| 543 | 142 | 1316.8520 | 659.4333 | 4363.56 | 2 |  |  |  |  |  |
| 543 | 143 | 486.3386 | 487.3459 | 3924.29 | 1 |  |  |  |  |  |
| 543 | 144 | 1086.1472 | 1087.1545 | 2442.61 | 1 |  |  |  |  |  |
| 543 | 145 | 360.1456 | 361.1529 | 1500.92 | 1 |  |  |  |  |  |
| 543 | 146 | 474.2244 | 475.2317 | 1400.81 | 1 | 474.2260 | C4 | 4 | -1.62e-03 | -3.42 |
| 543 | 147 | 542.3157 | 543.3229 | 2274.28 | 1 |  |  |  |  |  |

  

All proteins /
CsTx-1a\_S1 Cupiennius salei toxin 1 isoform a S1^ACsTx-1a\_S2 Cupiennius salei toxin 1 isoform a S2 /
Proteoform #15
