# Supplementary material for: The Dual Prey-Inactivation Strategy of Spiders—In-Depth Venomic Analysis of Cupiennius salei
Source: Toxins (Basel). 2019 Mar 19;11(3):167. doi: 10.3390/toxins11030167 (PMC6468893; doi:10.3390/toxins11030167)
Supplement: Supplementary file 1 [file toxins-11-00167-s001.zip › Supplementary Dataset EV1/20180328_f2_topdown_OTMS2_EThcD_NL_i02_ms2_proteoform_cutoff_html/prsms/prsm167.html]

Protein-Spectrum-Match for Spectrum #406


All proteins /
CsTx-33a Cupiennius salei toxin 33 isoform a /
Proteoform #12

## Protein-Spectrum-Match #167 for Spectrum #406

|  |  |  |  |  |  |
| --- | --- | --- | --- | --- | --- |
| PrSM ID: | 167 | Scan(s): | 544 | Precursor charge: | 13 |
| Precursor m/z: | 619.6169 | Precursor mass: | 8041.9246 | Proteoform mass: | 8042.9231 |
| # matched peaks: | 56 | # matched fragment ions: | 38 | # unexpected modifications: | 0 |
| E-value: | 9.29e-36 | P-value: | 9.29e-36 | Q-value (Spectral FDR): | 0 |

  

|  |  |  |  |  |  |  |  |  |  |  |  |  |  |  |  |  |  |  |  |  |  |  |  |  |  |  |  |  |  |  |  |  |  |  |  |  |  |  |  |  |  |  |  |  |  |  |  |  |  |  |  |  |  |  |  |  |  |  |  |  |  |  |  |  |  |  |  |  |  |
| --- | --- | --- | --- | --- | --- | --- | --- | --- | --- | --- | --- | --- | --- | --- | --- | --- | --- | --- | --- | --- | --- | --- | --- | --- | --- | --- | --- | --- | --- | --- | --- | --- | --- | --- | --- | --- | --- | --- | --- | --- | --- | --- | --- | --- | --- | --- | --- | --- | --- | --- | --- | --- | --- | --- | --- | --- | --- | --- | --- | --- | --- | --- | --- | --- | --- | --- | --- | --- | --- |
|  | |  | | | | | | | | | | | | | | | | | | | | | | | | | | | | | | | | | | | | | | | | | | | | | | | | | | | | | | | | | | | | | | | | | | | |
| 1 |  |  | M |  | K |  | I |  | L |  | V |  | I |  | C |  | A |  | V |  | L |  |  | L |  | T |  | T |  | I |  | C |  | S |  | K |  | S |  | S |  | A |  |  | E |  | I |  | D |  | E |  | D |  | F |  | L |  | K |  | D |  | E |  | 30 |  |
|  | |  | | | | | | | | | | | | | | | | | | | | | | | | | | | | | | | | | | | | | | | | | | | | | | | | | | | | | | | | | | | | | | | | | | | |
| 31 |  |  | S |  | F |  | E |  | A |  | D |  | G |  | I |  | V |  | P |  | F |  |  | F |  | A |  | N |  | E |  | E |  | F |  | R | ] | K | ⎩ | D |  | K |  | ⎫ | R |  | N | ⎫ | C |  | I |  | P | ⎫ | R | ⎫ | N | ⎱ | Q | ⎫ | E | ⎫ | C |  | 60 |  |
|  | |  | | | | | | | | | | | | | | | | | | | | | | | | | | | | | | | | | | | | | | | | | | | | | | | | | | | | | | | | | | | | | | | | | | | |
| 61 |  | ⎫ | T |  | I | ⎫ | D | ⎫ | K | ⎫ | R | ⎱ | N | ⎫ | C | ⎫ | C | ⎫ | R | ⎫ | R |  | ⎱ | G | ⎫ | L |  | F | ⎫ | K | ⎱ | M |  | T | ⎫ | C |  | Q | ⎫ | C |  | M |  | ⎩ | K | ⎩ | S | ⎩ | N | ⎱ | D | ⎩ | E |  | S |  | G | ⎫ | Q |  | P |  | T |  | 90 |  |
|  | |  | | | | | | | | | | | | | | | | | | | | | | | | | | | | | | | | | | | | | | | | | | | | | | | | | | | | | | | | | | | | | | | | | | | |
| 91 |  |  | E |  | K |  | C |  | T |  | C | ⎫ | R | ⎩ | R |  | P |  | R |  | P |  |  | I |  | F |  | H |  | L |  | L |  | Y |  | K | ⎫ | G |  | L |  | L |  | ⎫ | K | [ | G |  | | 112 |  | | | | | | | | | | | | | | | |

Fixed PTMs: Carbamidomethylation [C53 C60 C67 C68 C77 C79 C93 C95 ]

  

All peaks (134)  Matched peaks (56)  Not matched peaks (78)

  

| Scan | Peak | Mono mass | Mono m/z | Intensity | Charge | Theoretical mass | Ion | Pos | Mass error | PPM error |
| --- | --- | --- | --- | --- | --- | --- | --- | --- | --- | --- |
| 544 | 1 | 3091.7509 | 619.3575 | 30890.20 | 5 |  |  |  |  |  |
| 544 | 2 | 7985.8650 | 799.5938 | 13956.29 | 10 |  |  |  |  |  |
| 544 | 3 | 7927.8324 | 881.8775 | 13717.42 | 9 |  |  |  |  |  |
| 544 | 4 | 7984.8699 | 726.9045 | 12774.44 | 11 |  |  |  |  |  |
| 544 | 5 | 3075.4740 | 616.1021 | 11231.30 | 5 | 3075.4919 | C23 | 23 | -0.0179 | -5.82 |
| 544 | 6 | 2329.1700 | 777.3973 | 14126.86 | 3 | 2329.1855 | C18 | 18 | -0.0155 | -6.66 |
| 544 | 7 | 2478.3436 | 620.5932 | 10626.48 | 4 |  |  |  |  |  |
| 544 | 8 | 3752.8222 | 751.5717 | 10733.14 | 5 | 3752.8490 | C29 | 29 | -0.0268 | -7.14 |
| 544 | 9 | 3392.6431 | 679.5359 | 9484.27 | 5 | 3392.6659 | C26 | 26 | -0.0227 | -6.70 |
| 544 | 10 | 7985.8644 | 888.3255 | 9728.83 | 9 |  |  |  |  |  |
| 544 | 11 | 1298.6916 | 650.3531 | 12162.22 | 2 | 1298.6989 | C10 | 10 | -7.35e-03 | -5.66 |
| 544 | 12 | 7970.8587 | 886.6582 | 8062.24 | 9 |  |  |  |  |  |
| 544 | 13 | 2443.2139 | 815.4119 | 8915.98 | 3 | 2443.2284 | C19 | 19 | -0.0145 | -5.93 |
| 544 | 14 | 2993.6272 | 749.4141 | 16020.38 | 4 |  |  |  |  |  |
| 544 | 15 | 4040.9103 | 809.1893 | 6839.24 | 5 | 4040.9382 | C31 | 31 | -0.0280 | -6.92 |
| 544 | 16 | 2320.3331 | 581.0905 | 9094.98 | 4 |  |  |  |  |  |
| 544 | 17 | 3911.9817 | 783.4036 | 6312.68 | 5 |  |  |  |  |  |
| 544 | 18 | 3382.7533 | 846.6956 | 7845.38 | 4 | 3382.7517 | Z\_DOT28 | 36 | 1.64e-03 | 0.49 |
| 544 | 19 | 2763.2735 | 691.8256 | 6314.07 | 4 | 2763.2897 | C21 | 21 | -0.0162 | -5.88 |
| 544 | 20 | 2603.2433 | 868.7551 | 8737.90 | 3 | 2603.2591 | C20 | 20 | -0.0157 | -6.04 |
| 544 | 21 | 4200.0687 | 701.0187 | 7948.50 | 6 |  |  |  |  |  |
| 544 | 22 | 1579.9750 | 790.9948 | 11033.61 | 2 |  |  |  |  |  |
| 544 | 23 | 2173.0720 | 725.3646 | 7264.31 | 3 | 2173.0844 | C17 | 17 | -0.0124 | -5.69 |
| 544 | 24 | 7926.8454 | 793.6918 | 11883.42 | 10 |  |  |  |  |  |
| 544 | 25 | 8025.8815 | 803.5954 | 4148.23 | 10 |  |  |  |  |  |
| 544 | 26 | 2763.2746 | 922.0988 | 7619.27 | 3 | 2763.2897 | C21 | 21 | -0.0151 | -5.47 |
| 544 | 27 | 1929.9508 | 644.3242 | 8621.58 | 3 | 1929.9625 | C15 | 15 | -0.0117 | -6.04 |
| 544 | 28 | 1834.1306 | 612.3842 | 8298.21 | 3 | 1834.1220 | Z\_DOT15 | 49 | 8.67e-03 | 4.73 |
| 544 | 29 | 1989.2301 | 664.0840 | 16181.27 | 3 |  |  |  |  |  |
| 544 | 30 | 4523.1650 | 905.6403 | 7471.51 | 5 | 4523.1701 | Z\_DOT37 | 27 | -5.12e-03 | -1.13 |
| 544 | 31 | 2603.2422 | 651.8178 | 7339.81 | 4 | 2603.2591 | C20 | 20 | -0.0168 | -6.47 |
| 544 | 32 | 2059.2555 | 687.4258 | 5823.41 | 3 |  |  |  |  |  |
| 544 | 33 | 4040.9091 | 674.4921 | 4805.53 | 6 | 4040.9382 | C31 | 31 | -0.0291 | -7.21 |
| 544 | 34 | 3911.9823 | 653.0043 | 5823.56 | 6 |  |  |  |  |  |
| 544 | 35 | 1184.6494 | 593.3320 | 10621.95 | 2 | 1184.6560 | C9 | 9 | -6.53e-03 | -5.52 |
| 544 | 36 | 658.3841 | 659.3914 | 12679.12 | 1 | 658.3874 | C5 | 5 | -3.32e-03 | -5.04 |
| 544 | 37 | 2059.2562 | 515.8213 | 7022.67 | 4 |  |  |  |  |  |
| 544 | 38 | 3520.7376 | 705.1548 | 9150.20 | 5 | 3520.7608 | C27 | 27 | -0.0232 | -6.60 |
| 544 | 39 | 7912.8384 | 990.1121 | 5214.53 | 8 | 7913.8441 | C63 | 63 | -3.36e-03 | -0.42 |
| 544 | 40 | 1715.8209 | 572.9476 | 10790.48 | 3 | 1715.8307 | C13 | 13 | -9.85e-03 | -5.74 |
| 544 | 41 | 5050.2692 | 842.7188 | 4598.61 | 6 | 5049.3023 | C40 | 40 | -0.0355 | -7.03 |
| 544 | 42 | 4661.1500 | 777.8656 | 4897.20 | 6 | 4661.1793 | C36 | 36 | -0.0293 | -6.28 |
| 544 | 43 | 7927.8441 | 991.9878 | 6873.30 | 8 |  |  |  |  |  |
| 544 | 44 | 7968.8468 | 997.1131 | 5229.56 | 8 |  |  |  |  |  |
| 544 | 45 | 6054.6660 | 865.9596 | 4297.33 | 7 | 6053.7079 | C48 | 48 | -0.0442 | -7.30 |
| 544 | 46 | 7631.6209 | 848.9652 | 4605.87 | 9 | 7630.6545 | C60 | 60 | -0.0359 | -4.71 |
| 544 | 47 | 4993.2439 | 833.2146 | 5896.39 | 6 |  |  |  |  |  |
| 544 | 48 | 7968.8350 | 797.8908 | 6197.29 | 10 |  |  |  |  |  |
| 544 | 49 | 7998.8691 | 800.8942 | 4223.15 | 10 |  |  |  |  |  |
| 544 | 50 | 6152.7463 | 879.9710 | 5232.16 | 7 |  |  |  |  |  |
| 544 | 51 | 4200.0673 | 841.0207 | 6396.62 | 5 |  |  |  |  |  |
| 544 | 52 | 3267.7259 | 817.9387 | 6200.01 | 4 | 3267.7247 | Z\_DOT27 | 37 | 1.16e-03 | 0.35 |
| 544 | 53 | 1426.7497 | 714.3821 | 4890.57 | 2 | 1426.7575 | C11 | 11 | -7.83e-03 | -5.48 |
| 544 | 54 | 3752.8264 | 626.4783 | 5073.99 | 6 | 3752.8490 | C29 | 29 | -0.0226 | -6.03 |
| 544 | 55 | 7912.8206 | 880.2096 | 4627.82 | 9 | 7913.8441 | C63 | 63 | -0.0212 | -2.67 |
| 544 | 56 | 3496.7990 | 875.2070 | 6195.17 | 4 | 3496.7946 | Z\_DOT29 | 35 | 4.36e-03 | 1.25 |
| 544 | 57 | 1555.7915 | 519.6044 | 4829.18 | 3 | 1555.8001 | C12 | 12 | -8.62e-03 | -5.54 |
| 544 | 58 | 5049.2660 | 722.3310 | 6471.73 | 7 | 5049.3023 | C40 | 40 | -0.0363 | -7.19 |
| 544 | 59 | 1426.7494 | 476.5904 | 4895.72 | 3 | 1426.7575 | C11 | 11 | -8.05e-03 | -5.64 |
| 544 | 60 | 1298.6919 | 433.9046 | 7720.50 | 3 | 1298.6989 | C10 | 10 | -7.03e-03 | -5.41 |
| 544 | 61 | 7854.8009 | 982.8574 | 4198.69 | 8 |  |  |  |  |  |
| 544 | 62 | 5511.6930 | 919.6228 | 3926.34 | 6 |  |  |  |  |  |
| 544 | 63 | 7574.5943 | 947.8316 | 4410.18 | 8 |  |  |  |  |  |
| 544 | 64 | 2443.2156 | 611.8112 | 4708.98 | 4 | 2443.2284 | C19 | 19 | -0.0128 | -5.24 |
| 544 | 65 | 7984.8563 | 666.4120 | 5497.25 | 12 |  |  |  |  |  |
| 544 | 66 | 886.5593 | 444.2869 | 5068.41 | 2 |  |  |  |  |  |
| 544 | 67 | 388.2417 | 389.2490 | 7685.79 | 1 | 388.2434 | C3 | 3 | -1.61e-03 | -4.16 |
| 544 | 68 | 1815.8674 | 908.9410 | 4189.32 | 2 |  |  |  |  |  |
| 544 | 69 | 2935.5989 | 734.9070 | 4106.39 | 4 |  |  |  |  |  |
| 544 | 70 | 2027.9512 | 676.9910 | 4983.82 | 3 |  |  |  |  |  |
| 544 | 71 | 3713.8897 | 619.9889 | 11653.96 | 6 |  |  |  |  |  |
| 544 | 72 | 7941.8454 | 795.1918 | 3952.99 | 10 |  |  |  |  |  |
| 544 | 73 | 3132.4915 | 627.5056 | 4539.66 | 5 | 3132.5134 | C24 | 24 | -0.0219 | -6.98 |
| 544 | 74 | 4650.2419 | 931.0556 | 5699.58 | 5 |  |  |  |  |  |
| 544 | 75 | 1815.8649 | 606.2956 | 6052.22 | 3 |  |  |  |  |  |
| 544 | 76 | 4991.2537 | 999.2580 | 4412.55 | 5 |  |  |  |  |  |
| 544 | 77 | 1555.7920 | 778.9033 | 3850.71 | 2 | 1555.8001 | C12 | 12 | -8.06e-03 | -5.18 |
| 544 | 78 | 3520.7292 | 587.7955 | 3786.51 | 6 | 3520.7608 | C27 | 27 | -0.0316 | -8.98 |
| 544 | 79 | 1929.9522 | 965.9834 | 3954.51 | 2 | 1929.9625 | C15 | 15 | -0.0102 | -5.31 |
| 544 | 80 | 2993.6297 | 998.8838 | 5095.92 | 3 |  |  |  |  |  |
| 544 | 81 | 2935.6019 | 588.1277 | 4085.68 | 5 |  |  |  |  |  |
| 544 | 82 | 4022.4455 | 671.4149 | 3466.78 | 6 |  |  |  |  |  |
| 544 | 83 | 1851.9607 | 618.3275 | 3319.16 | 3 |  |  |  |  |  |
| 544 | 84 | 3091.7513 | 773.9451 | 6018.99 | 4 |  |  |  |  |  |
| 544 | 85 | 312.2149 | 313.2222 | 6208.16 | 1 |  |  |  |  |  |
| 544 | 86 | 6745.2002 | 844.1573 | 5458.29 | 8 | 6745.2320 | Z\_DOT54 | 10 | -0.0318 | -4.72 |
| 544 | 87 | 3583.8203 | 896.9624 | 3162.69 | 4 | 3583.8266 | Z\_DOT30 | 34 | -6.28e-03 | -1.75 |
| 544 | 88 | 3075.4759 | 769.8762 | 6406.44 | 4 | 3075.4919 | C23 | 23 | -0.0161 | -5.22 |
| 544 | 89 | 929.6138 | 930.6211 | 4289.47 | 1 |  |  |  |  |  |
| 544 | 90 | 2538.4325 | 635.6154 | 3393.08 | 4 |  |  |  |  |  |
| 544 | 91 | 7855.7700 | 873.8706 | 4944.94 | 9 |  |  |  |  |  |
| 544 | 92 | 4549.1093 | 759.1922 | 2117.30 | 6 |  |  |  |  |  |
| 544 | 93 | 3711.9173 | 928.9866 | 3323.00 | 4 | 3711.9216 | Z\_DOT31 | 33 | -4.24e-03 | -1.14 |
| 544 | 94 | 5996.6473 | 1000.4485 | 3307.26 | 6 |  |  |  |  |  |
| 544 | 95 | 7910.8263 | 792.0899 | 4828.10 | 10 |  |  |  |  |  |
| 544 | 96 | 5509.6872 | 788.1055 | 6124.62 | 7 |  |  |  |  |  |
| 544 | 97 | 611.0427 | 612.0500 | 2344.42 | 1 |  |  |  |  |  |
| 544 | 98 | 7941.8318 | 883.4330 | 3762.06 | 9 |  |  |  |  |  |
| 544 | 99 | 5997.6577 | 857.8155 | 3598.75 | 7 |  |  |  |  |  |
| 544 | 100 | 4603.1261 | 768.1950 | 2761.11 | 6 |  |  |  |  |  |
| 544 | 101 | 3137.6823 | 785.4278 | 4348.07 | 4 |  |  |  |  |  |
| 544 | 102 | 3047.7373 | 762.9416 | 3065.50 | 4 |  |  |  |  |  |
| 544 | 103 | 4661.1477 | 666.8855 | 3358.94 | 7 | 4661.1793 | C36 | 36 | -0.0316 | -6.78 |
| 544 | 104 | 7556.5865 | 945.5806 | 2486.46 | 8 |  |  |  |  |  |
| 544 | 105 | 3695.8192 | 924.9621 | 2819.58 | 4 |  |  |  |  |  |
| 544 | 106 | 1833.1332 | 917.5739 | 5358.86 | 2 |  |  |  |  |  |
| 544 | 107 | 1213.7400 | 1214.7473 | 3124.86 | 1 |  |  |  |  |  |
| 544 | 108 | 540.3848 | 541.3920 | 4076.02 | 1 |  |  |  |  |  |
| 544 | 109 | 6745.2053 | 964.6080 | 2766.13 | 7 | 6745.2320 | Z\_DOT54 | 10 | -0.0268 | -3.97 |
| 544 | 110 | 5350.6637 | 765.3878 | 5283.21 | 7 |  |  |  |  |  |
| 544 | 111 | 2044.9769 | 682.6663 | 3081.61 | 3 | 2044.9894 | C16 | 16 | -0.0125 | -6.10 |
| 544 | 112 | 4968.4265 | 829.0784 | 2863.26 | 6 | 4968.4390 | Z\_DOT41 | 23 | -0.0125 | -2.52 |
| 544 | 113 | 2919.3792 | 730.8521 | 4449.58 | 4 | 2919.3908 | C22 | 22 | -0.0116 | -3.99 |
| 544 | 114 | 1715.8188 | 858.9167 | 4923.48 | 2 | 1715.8307 | C13 | 13 | -0.0120 | -6.98 |
| 544 | 115 | 3652.7813 | 731.5635 | 3545.74 | 5 |  |  |  |  |  |
| 544 | 116 | 4893.3952 | 700.0637 | 3384.71 | 7 |  |  |  |  |  |
| 544 | 117 | 6801.2154 | 972.6095 | 3414.13 | 7 |  |  |  |  |  |
| 544 | 118 | 3520.7379 | 881.1918 | 3001.79 | 4 | 3520.7608 | C27 | 27 | -0.0229 | -6.51 |
| 544 | 119 | 3464.7168 | 693.9506 | 2280.79 | 5 |  |  |  |  |  |
| 544 | 120 | 5714.7358 | 817.3981 | 3362.57 | 7 | 5714.7455 | Z\_DOT46 | 18 | -9.64e-03 | -1.69 |
| 544 | 121 | 7897.7818 | 988.2300 | 2831.44 | 8 | 7898.8095 | Z\_DOT63 | 1 | -0.0254 | -3.22 |
| 544 | 122 | 773.4760 | 387.7453 | 2745.63 | 2 |  |  |  |  |  |
| 544 | 123 | 4894.4109 | 816.7424 | 2511.76 | 6 |  |  |  |  |  |
| 544 | 124 | 816.5302 | 817.5375 | 3829.74 | 1 |  |  |  |  |  |
| 544 | 125 | 1028.5492 | 515.2819 | 2409.38 | 2 | 1028.5549 | C8 | 8 | -5.65e-03 | -5.49 |
| 544 | 126 | 1184.6498 | 395.8905 | 4898.00 | 3 | 1184.6560 | C9 | 9 | -6.21e-03 | -5.24 |
| 544 | 127 | 703.4471 | 704.4543 | 3494.16 | 1 |  |  |  |  |  |
| 544 | 128 | 1167.6232 | 390.2150 | 1558.93 | 3 |  |  |  |  |  |
| 544 | 129 | 1010.6097 | 506.3121 | 1096.31 | 2 |  |  |  |  |  |
| 544 | 130 | 1213.7395 | 607.8770 | 2533.57 | 2 |  |  |  |  |  |
| 544 | 131 | 998.8800 | 999.8873 | 974.88 | 1 |  |  |  |  |  |
| 544 | 132 | 887.4316 | 888.4389 | 846.31 | 1 |  |  |  |  |  |
| 544 | 133 | 1165.0878 | 1166.0950 | 948.77 | 1 |  |  |  |  |  |
| 544 | 134 | 986.4803 | 987.4876 | 1403.24 | 1 |  |  |  |  |  |

  

All proteins /
CsTx-33a Cupiennius salei toxin 33 isoform a /
Proteoform #12
