# Supplementary material for: The Dual Prey-Inactivation Strategy of Spiders—In-Depth Venomic Analysis of Cupiennius salei
Source: Toxins (Basel). 2019 Mar 19;11(3):167. doi: 10.3390/toxins11030167 (PMC6468893; doi:10.3390/toxins11030167)
Supplement: Supplementary file 1 [file toxins-11-00167-s001.zip › Supplementary Dataset EV1/20180328_f2_topdown_OTMS2_EThcD_NL_i02_ms2_proteoform_cutoff_html/prsms/prsm168.html]

Protein-Spectrum-Match for Spectrum #407


All proteins /
CsTx-1a\_S1 Cupiennius salei toxin 1 isoform a S1^ACsTx-1a\_S2 Cupiennius salei toxin 1 isoform a S2 /
Proteoform #15

## Protein-Spectrum-Match #168 for Spectrum #407

|  |  |  |  |  |  |
| --- | --- | --- | --- | --- | --- |
| PrSM ID: | 168 | Scan(s): | 545 | Precursor charge: | 13 |
| Precursor m/z: | 679.9494 | Precursor mass: | 8826.2479 | Proteoform mass: | 8826.2179 |
| # matched peaks: | 51 | # matched fragment ions: | 39 | # unexpected modifications: | 1 |
| E-value: | 2.28e-32 | P-value: | 2.28e-32 | Q-value (Spectral FDR): | 0 |

  

|  |  |  |  |  |  |  |  |  |  |  |  |  |  |  |  |  |  |  |  |  |  |  |  |  |  |  |  |  |  |  |  |  |  |  |  |  |  |  |  |  |  |  |  |  |  |  |  |  |  |  |  |  |  |  |  |  |  |  |  |  |  |  |  |  |  |  |  |  |  |
| --- | --- | --- | --- | --- | --- | --- | --- | --- | --- | --- | --- | --- | --- | --- | --- | --- | --- | --- | --- | --- | --- | --- | --- | --- | --- | --- | --- | --- | --- | --- | --- | --- | --- | --- | --- | --- | --- | --- | --- | --- | --- | --- | --- | --- | --- | --- | --- | --- | --- | --- | --- | --- | --- | --- | --- | --- | --- | --- | --- | --- | --- | --- | --- | --- | --- | --- | --- | --- | --- |
|  | |  | | | | | | | | | | | | | | | | | | | | | | | | | | | | | | | | | | | | | | | | | | | | | | | | | | | | | | | | | | | | | | | | | | | |
| 1 |  |  | M |  | K |  | V |  | L |  | I |  | I |  | S |  | A |  | V |  | L |  |  | F |  | I |  | T |  | I |  | F |  | S |  | N |  | I |  | S |  | A |  |  | E |  | I |  | E |  | D |  | D |  | F |  | L |  | E |  | D |  | E |  | 30 |  |
|  | |  | | | | | | | | | | | | | | | | | | | | | | | | | | | | | | | | | | | | | | | | | | | | | | | | | | | | | | | | | | | | | | | | | | | |
| 31 |  |  | S |  | F |  | E |  | A |  | E |  | D |  | I |  | I |  | P |  | F |  |  | F |  | E |  | N |  | E |  | Q |  | A |  | R | ] | S | ⎩ | C |  | I |  |  | P |  | K |  | H |  | E |  | E | ⎫ | C |  | T | ⎫ | N | ⎱ | D |  | K |  | 60 |  |
|  | |  | | | | | | | | | | | | | | | | | | | | | | | | | | | | | | | | | | | | | | | | | | | | | | | | | | | | | | | | | | | | | | | | | | | |
| 61 |  | ⎫ | H | ⎫ | N | ⎫ | C | ⎫ | C |  | R | ⎫ | K | ⎱ | G | ⎱ | L | ⎫ | F | ⎱ | K |  | ⎫ | L | ⎫ | K | ⎫ | C |  | Q | ⎫ | C |  | S |  | T |  | F | ⎱ | D | ⎫ | D |  | ⎫ | E | ⎱ | S |  | G | ⎱ | Q |  | P |  | T | ⎫ | E | ⎫ | R |  | C |  | A |  | 90 |  |
|  | |  | | | | | -42.04 | | | | | | | | | | | | | | | | | | | | | | | | | | | | | | | | | | | | | | | | | | | | | | | | | | | | | | | | | | | |
| 91 |  |  | C |  | G | ⎱ | R |  | P |  | M |  | G |  | H |  | Q |  | A |  | I |  |  | E |  | T |  | G |  | L |  | N |  | I |  | F |  | R | ⎫ | G | ⎫ | L |  |  | F |  | K |  | G |  | K | ⎫ | K | ⎫ | K |  | N |  | K |  | K |  | T |  | 120 |  |
|  | |  | | | | | | | | | | | | | | | | | | | | | | | | | | | | | | | | | | | | | | | | | | | | | | | | | | | | | | | | | | | | | | | | | | | |
| 121 |  | ⎫ | K | ⎫ | G |  | | | | 122 |  | | | | | | | | | | | | | | | | | | | | | | | | | | | | | | | | | | | | | | | | | | | | | | | | | | | | | | | |

Fixed PTMs: Carbamidomethylation [C49 C56 C63 C64 C73 C75 C89 C91 ]   
  
     Unexpected modifications:   Unknown [-42.04]

  

All peaks (147)  Matched peaks (51)  Not matched peaks (96)

  

| Scan | Peak | Mono mass | Mono m/z | Intensity | Charge | Theoretical mass | Ion | Pos | Mass error | PPM error |
| --- | --- | --- | --- | --- | --- | --- | --- | --- | --- | --- |
| 545 | 1 | 8770.1830 | 878.0256 | 99902.54 | 10 |  |  |  |  |  |
| 545 | 2 | 8769.1709 | 798.2046 | 97758.87 | 11 | 8768.2124 | C74 | 74 | -0.0438 | -5.00 |
| 545 | 3 | 8770.1909 | 975.4729 | 45638.34 | 9 |  |  |  |  |  |
| 545 | 4 | 8784.1932 | 799.5703 | 37754.23 | 11 |  |  |  |  |  |
| 545 | 5 | 8811.1999 | 882.1273 | 38302.37 | 10 |  |  |  |  |  |
| 545 | 6 | 8712.1590 | 969.0249 | 31371.79 | 9 |  |  |  |  |  |
| 545 | 7 | 8810.1887 | 801.9335 | 30409.48 | 11 |  |  |  |  |  |
| 545 | 8 | 8753.1768 | 876.3250 | 34209.08 | 10 |  |  |  |  |  |
| 545 | 9 | 8784.1961 | 879.4269 | 35546.38 | 10 |  |  |  |  |  |
| 545 | 10 | 8711.1552 | 872.1228 | 39783.70 | 10 |  |  |  |  |  |
| 545 | 11 | 8770.1827 | 731.8558 | 31407.15 | 12 |  |  |  |  |  |
| 545 | 12 | 8726.1687 | 970.5816 | 24969.86 | 9 |  |  |  |  |  |
| 545 | 13 | 4443.9109 | 889.7894 | 29698.26 | 5 | 4443.9333 | C36 | 36 | -0.0224 | -5.05 |
| 545 | 14 | 4443.9108 | 741.6591 | 25887.19 | 6 | 4443.9333 | C36 | 36 | -0.0225 | -5.07 |
| 545 | 15 | 8754.1696 | 973.6928 | 27089.13 | 9 |  |  |  |  |  |
| 545 | 16 | 8725.1695 | 873.5242 | 28455.37 | 10 |  |  |  |  |  |
| 545 | 17 | 4414.1038 | 736.6912 | 47014.21 | 6 |  |  |  |  |  |
| 545 | 18 | 3395.8725 | 680.1818 | 30667.24 | 5 |  |  |  |  |  |
| 545 | 19 | 8697.1238 | 870.7197 | 20583.00 | 10 |  |  |  |  |  |
| 545 | 20 | 8712.1521 | 793.0211 | 20224.01 | 11 |  |  |  |  |  |
| 545 | 21 | 4414.1056 | 883.8284 | 32486.74 | 5 |  |  |  |  |  |
| 545 | 22 | 8699.1303 | 967.5773 | 16465.53 | 9 |  |  |  |  |  |
| 545 | 23 | 8784.1886 | 977.0282 | 17694.00 | 9 |  |  |  |  |  |
| 545 | 24 | 2528.0772 | 633.0266 | 23765.10 | 4 | 2528.0889 | C20 | 20 | -0.0117 | -4.63 |
| 545 | 25 | 4325.2704 | 721.8857 | 19575.59 | 6 |  |  |  |  |  |
| 545 | 26 | 3157.5004 | 632.5074 | 16646.68 | 5 | 3157.5153 | C25 | 25 | -0.0149 | -4.73 |
| 545 | 27 | 4384.3071 | 877.8687 | 18086.70 | 5 |  |  |  |  |  |
| 545 | 28 | 8697.1184 | 791.6544 | 17107.28 | 11 |  |  |  |  |  |
| 545 | 29 | 1372.5800 | 687.2973 | 19746.35 | 2 | 1372.5863 | C11 | 11 | -6.33e-03 | -4.61 |
| 545 | 30 | 1752.7597 | 877.3871 | 27244.32 | 2 | 1752.7671 | C14 | 14 | -7.45e-03 | -4.25 |
| 545 | 31 | 3157.5008 | 790.3825 | 19365.36 | 4 | 3157.5153 | C25 | 25 | -0.0146 | -4.61 |
| 545 | 32 | 4055.7888 | 812.1650 | 15057.33 | 5 | 4055.8103 | C32 | 32 | -0.0215 | -5.30 |
| 545 | 33 | 3323.8852 | 665.7843 | 17063.43 | 5 | 3323.8699 | Z\_DOT30 | 45 | 0.0154 | 4.62 |
| 545 | 34 | 8724.1699 | 794.1136 | 15516.38 | 11 | 8723.1672 | Z\_DOT74 | 1 | 3.38e-04 | 0.04 |
| 545 | 35 | 2471.0560 | 618.7713 | 19308.67 | 4 | 2471.0674 | C19 | 19 | -0.0115 | -4.65 |
| 545 | 36 | 5579.7203 | 930.9607 | 13497.00 | 6 |  |  |  |  |  |
| 545 | 37 | 2528.0716 | 843.6978 | 18829.01 | 3 | 2528.0889 | C20 | 20 | -0.0173 | -6.84 |
| 545 | 38 | 4770.0677 | 955.0208 | 17377.50 | 5 | 4770.0923 | C39 | 39 | -0.0246 | -5.15 |
| 545 | 39 | 8770.1859 | 1097.2805 | 12400.68 | 8 |  |  |  |  |  |
| 545 | 40 | 5503.3298 | 918.2289 | 15834.12 | 6 | 5503.3559 | C45 | 45 | -0.0261 | -4.74 |
| 545 | 41 | 8812.1951 | 980.1401 | 16169.25 | 9 |  |  |  |  |  |
| 545 | 42 | 8668.1373 | 964.1336 | 11968.71 | 9 |  |  |  |  |  |
| 545 | 43 | 3445.5856 | 690.1244 | 14019.91 | 5 | 3445.6046 | C27 | 27 | -0.0190 | -5.50 |
| 545 | 44 | 4383.3031 | 731.5578 | 17545.26 | 6 | 4383.2925 | Z\_DOT39 | 36 | 0.0106 | 2.43 |
| 545 | 45 | 8753.1644 | 796.7495 | 19809.54 | 11 |  |  |  |  |  |
| 545 | 46 | 6300.1094 | 788.5210 | 13802.31 | 8 | 6299.1369 | Z\_DOT55 | 20 | -0.0298 | -4.73 |
| 545 | 47 | 3324.8940 | 832.2308 | 16277.74 | 4 |  |  |  |  |  |
| 545 | 48 | 4554.9408 | 760.1641 | 14653.49 | 6 |  |  |  |  |  |
| 545 | 49 | 7340.2497 | 816.5906 | 10347.95 | 9 | 7339.2831 | C62 | 62 | -0.0358 | -4.88 |
| 545 | 50 | 2788.2295 | 930.4171 | 13199.19 | 3 | 2788.2414 | C22 | 22 | -0.0119 | -4.26 |
| 545 | 51 | 8739.1661 | 972.0257 | 11872.14 | 9 |  |  |  |  |  |
| 545 | 52 | 2916.3231 | 730.0880 | 11960.55 | 4 | 2916.3363 | C23 | 23 | -0.0133 | -4.55 |
| 545 | 53 | 4170.8159 | 835.1705 | 12685.87 | 5 | 4170.8372 | C33 | 33 | -0.0213 | -5.10 |
| 545 | 54 | 2471.0544 | 824.6921 | 12832.70 | 3 | 2471.0674 | C19 | 19 | -0.0131 | -5.28 |
| 545 | 55 | 4299.8583 | 860.9789 | 13793.98 | 5 | 4299.8798 | C34 | 34 | -0.0215 | -5.01 |
| 545 | 56 | 2617.5789 | 655.4020 | 11948.54 | 4 |  |  |  |  |  |
| 545 | 57 | 8640.0883 | 961.0171 | 13521.90 | 9 | 8640.1174 | C73 | 73 | -0.0292 | -3.38 |
| 545 | 58 | 8810.2004 | 735.1906 | 14968.59 | 12 |  |  |  |  |  |
| 545 | 59 | 1866.8009 | 934.4077 | 18259.60 | 2 | 1866.8101 | C15 | 15 | -9.19e-03 | -4.92 |
| 545 | 60 | 6300.1368 | 901.0268 | 14472.75 | 7 | 6299.1369 | Z\_DOT55 | 20 | -2.39e-03 | -0.38 |
| 545 | 61 | 6041.5763 | 1007.9367 | 8586.77 | 6 |  |  |  |  |  |
| 545 | 62 | 2641.1612 | 661.2976 | 13943.05 | 4 | 2641.1730 | C21 | 21 | -0.0118 | -4.46 |
| 545 | 63 | 8726.1732 | 1091.7789 | 12041.15 | 8 |  |  |  |  |  |
| 545 | 64 | 4527.3596 | 755.5672 | 9537.08 | 6 | 4527.3459 | Z\_DOT41 | 34 | 0.0137 | 3.02 |
| 545 | 65 | 6256.0841 | 783.0178 | 11913.24 | 8 |  |  |  |  |  |
| 545 | 66 | 8226.8378 | 823.6911 | 9775.15 | 10 |  |  |  |  |  |
| 545 | 67 | 7971.6389 | 886.7449 | 11013.55 | 9 |  |  |  |  |  |
| 545 | 68 | 2288.3975 | 763.8065 | 12038.32 | 3 |  |  |  |  |  |
| 545 | 69 | 3445.5895 | 862.4046 | 10834.63 | 4 | 3445.6046 | C27 | 27 | -0.0151 | -4.38 |
| 545 | 70 | 3029.4048 | 758.3585 | 10465.75 | 4 | 3029.4204 | C24 | 24 | -0.0156 | -5.14 |
| 545 | 71 | 8698.1537 | 1088.2765 | 8100.91 | 8 |  |  |  |  |  |
| 545 | 72 | 4058.1474 | 677.3652 | 10563.73 | 6 |  |  |  |  |  |
| 545 | 73 | 1169.7797 | 585.8971 | 13583.51 | 2 |  |  |  |  |  |
| 545 | 74 | 6539.8174 | 935.2669 | 9610.66 | 7 |  |  |  |  |  |
| 545 | 75 | 2671.5894 | 668.9046 | 11417.98 | 4 |  |  |  |  |  |
| 545 | 76 | 4770.0692 | 796.0188 | 10929.57 | 6 | 4770.0923 | C39 | 39 | -0.0231 | -4.84 |
| 545 | 77 | 6225.6546 | 890.3865 | 8370.43 | 7 |  |  |  |  |  |
| 545 | 78 | 8737.1814 | 795.2965 | 9031.53 | 11 |  |  |  |  |  |
| 545 | 79 | 8226.8435 | 915.1010 | 10108.31 | 9 |  |  |  |  |  |
| 545 | 80 | 3999.1284 | 667.5287 | 11682.51 | 6 |  |  |  |  |  |
| 545 | 81 | 8682.1212 | 965.6874 | 10803.58 | 9 |  |  |  |  |  |
| 545 | 82 | 8041.7129 | 894.5309 | 9548.42 | 9 | 8040.7420 | C68 | 68 | -0.0314 | -3.90 |
| 545 | 83 | 8098.7342 | 810.8807 | 10342.04 | 10 |  |  |  |  |  |
| 545 | 84 | 2203.3703 | 735.4640 | 9865.89 | 3 |  |  |  |  |  |
| 545 | 85 | 4271.2590 | 712.8838 | 7365.29 | 6 |  |  |  |  |  |
| 545 | 86 | 6098.5899 | 872.2344 | 15760.81 | 7 |  |  |  |  |  |
| 545 | 87 | 2788.2278 | 698.0642 | 11806.06 | 4 | 2788.2414 | C22 | 22 | -0.0135 | -4.85 |
| 545 | 88 | 7341.2560 | 918.6643 | 10849.91 | 8 |  |  |  |  |  |
| 545 | 89 | 1615.7008 | 808.8577 | 14055.78 | 2 | 1615.7082 | C13 | 13 | -7.38e-03 | -4.57 |
| 545 | 90 | 5926.9079 | 847.7084 | 8788.31 | 7 |  |  |  |  |  |
| 545 | 91 | 7912.6308 | 880.1885 | 12110.04 | 9 | 7912.6470 | C67 | 67 | -0.0162 | -2.04 |
| 545 | 92 | 1486.9512 | 744.4829 | 9708.34 | 2 |  |  |  |  |  |
| 545 | 93 | 3940.7617 | 789.1596 | 10816.64 | 5 | 3940.7834 | C31 | 31 | -0.0217 | -5.50 |
| 545 | 94 | 8041.7262 | 1006.2230 | 8713.79 | 8 | 8040.7420 | C68 | 68 | -0.0181 | -2.26 |
| 545 | 95 | 6357.1600 | 909.1730 | 7650.20 | 7 | 6356.1583 | Z\_DOT56 | 19 | -6.36e-04 | -0.10 |
| 545 | 96 | 8641.0949 | 865.1168 | 13160.13 | 10 | 8640.1174 | C73 | 73 | -0.0249 | -2.88 |
| 545 | 97 | 8341.8933 | 835.1966 | 9093.55 | 10 |  |  |  |  |  |
| 545 | 98 | 2017.2471 | 673.4230 | 8156.51 | 3 |  |  |  |  |  |
| 545 | 99 | 8713.1722 | 1090.1538 | 12367.31 | 8 |  |  |  |  |  |
| 545 | 100 | 8668.1366 | 867.8209 | 8444.42 | 10 |  |  |  |  |  |
| 545 | 101 | 8783.1773 | 732.9387 | 10999.05 | 12 |  |  |  |  |  |
| 545 | 102 | 6870.4075 | 764.3859 | 7088.24 | 9 |  |  |  |  |  |
| 545 | 103 | 6039.9888 | 863.8628 | 9533.08 | 7 | 6038.9844 | Z\_DOT53 | 22 | 2.05e-03 | 0.34 |
| 545 | 104 | 6098.6026 | 1017.4411 | 8568.89 | 6 |  |  |  |  |  |
| 545 | 105 | 997.4604 | 998.4677 | 8864.52 | 1 | 997.4651 | C8 | 8 | -4.68e-03 | -4.69 |
| 545 | 106 | 8653.1323 | 962.4664 | 11131.71 | 9 |  |  |  |  |  |
| 545 | 107 | 7282.2368 | 911.2869 | 7416.92 | 8 | 7282.2617 | C61 | 61 | -0.0249 | -3.42 |
| 545 | 108 | 2026.8322 | 1014.4234 | 7852.83 | 2 | 2026.8407 | C16 | 16 | -8.47e-03 | -4.18 |
| 545 | 109 | 4899.1073 | 980.8287 | 7086.88 | 5 | 4899.1349 | C40 | 40 | -0.0276 | -5.64 |
| 545 | 110 | 8655.1365 | 1082.8993 | 6956.34 | 8 |  |  |  |  |  |
| 545 | 111 | 856.5697 | 857.5770 | 10008.12 | 1 |  |  |  |  |  |
| 545 | 112 | 8753.1708 | 1095.1536 | 12223.42 | 8 |  |  |  |  |  |
| 545 | 113 | 2203.3699 | 551.8498 | 9324.48 | 4 |  |  |  |  |  |
| 545 | 114 | 2729.6249 | 910.8823 | 7897.13 | 3 |  |  |  |  |  |
| 545 | 115 | 7076.4550 | 885.5641 | 7299.45 | 8 |  |  |  |  |  |
| 545 | 116 | 2342.9619 | 781.9946 | 6965.71 | 3 | 2342.9725 | C18 | 18 | -0.0106 | -4.53 |
| 545 | 117 | 8668.1599 | 1084.5273 | 7933.91 | 8 |  |  |  |  |  |
| 545 | 118 | 8341.8864 | 927.8835 | 8369.19 | 9 |  |  |  |  |  |
| 545 | 119 | 5291.6299 | 882.9456 | 8468.64 | 6 |  |  |  |  |  |
| 545 | 120 | 8468.9613 | 942.0030 | 7169.92 | 9 |  |  |  |  |  |
| 545 | 121 | 8796.1918 | 880.6265 | 6887.94 | 10 |  |  |  |  |  |
| 545 | 122 | 6711.3793 | 746.7161 | 6048.25 | 9 |  |  |  |  |  |
| 545 | 123 | 4886.4586 | 815.4170 | 5380.46 | 6 | 4886.4424 | Z\_DOT44 | 31 | 0.0162 | 3.31 |
| 545 | 124 | 6871.4242 | 859.9353 | 8460.10 | 8 |  |  |  |  |  |
| 545 | 125 | 2187.3520 | 730.1246 | 8086.87 | 3 |  |  |  |  |  |
| 545 | 126 | 7455.6385 | 829.4116 | 8602.15 | 9 | 7454.6395 | Z\_DOT64 | 11 | -3.29e-03 | -0.44 |
| 545 | 127 | 3681.9983 | 737.4069 | 7728.89 | 5 |  |  |  |  |  |
| 545 | 128 | 1386.8766 | 463.2995 | 5162.70 | 3 |  |  |  |  |  |
| 545 | 129 | 600.3812 | 601.3884 | 8232.26 | 1 |  |  |  |  |  |
| 545 | 130 | 1258.5383 | 1259.5456 | 4138.28 | 1 | 1258.5434 | C10 | 10 | -5.07e-03 | -4.03 |
| 545 | 131 | 1428.8866 | 477.3028 | 6323.32 | 3 |  |  |  |  |  |
| 545 | 132 | 1357.9447 | 679.9796 | 5870.45 | 2 |  |  |  |  |  |
| 545 | 133 | 1185.7986 | 593.9066 | 3710.05 | 2 |  |  |  |  |  |
| 545 | 134 | 1472.2037 | 737.1091 | 20284.90 | 2 |  |  |  |  |  |
| 545 | 135 | 1372.5799 | 1373.5872 | 3256.25 | 1 | 1372.5863 | C11 | 11 | -6.38e-03 | -4.64 |
| 545 | 136 | 798.5044 | 400.2595 | 3511.03 | 2 |  |  |  |  |  |
| 545 | 137 | 802.9299 | 803.9372 | 19091.41 | 1 |  |  |  |  |  |
| 545 | 138 | 1486.9512 | 496.6577 | 2627.55 | 3 |  |  |  |  |  |
| 545 | 139 | 1258.5385 | 630.2765 | 4812.37 | 2 | 1258.5434 | C10 | 10 | -4.90e-03 | -3.89 |
| 545 | 140 | 680.0466 | 681.0538 | 12373.35 | 1 |  |  |  |  |  |
| 545 | 141 | 1456.9505 | 729.4825 | 8825.02 | 2 |  |  |  |  |  |
| 545 | 142 | 1316.8469 | 439.9562 | 2246.08 | 3 |  |  |  |  |  |
| 545 | 143 | 1317.8525 | 659.9335 | 4503.29 | 2 |  |  |  |  |  |
| 545 | 144 | 1057.7044 | 529.8595 | 2249.58 | 2 |  |  |  |  |  |
| 545 | 145 | 880.1819 | 881.1891 | 1794.08 | 1 |  |  |  |  |  |
| 545 | 146 | 1429.9286 | 715.9716 | 2086.79 | 2 |  |  |  |  |  |
| 545 | 147 | 486.3386 | 487.3459 | 4626.24 | 1 |  |  |  |  |  |

  

All proteins /
CsTx-1a\_S1 Cupiennius salei toxin 1 isoform a S1^ACsTx-1a\_S2 Cupiennius salei toxin 1 isoform a S2 /
Proteoform #15
