# Supplementary material for: The Dual Prey-Inactivation Strategy of Spiders—In-Depth Venomic Analysis of Cupiennius salei
Source: Toxins (Basel). 2019 Mar 19;11(3):167. doi: 10.3390/toxins11030167 (PMC6468893; doi:10.3390/toxins11030167)
Supplement: Supplementary file 1 [file toxins-11-00167-s001.zip › Supplementary Dataset EV1/20180328_f2_topdown_OTMS2_EThcD_NL_i02_ms2_proteoform_cutoff_html/prsms/prsm169.html]

Protein-Spectrum-Match for Spectrum #408


All proteins /
CsTx-12a\_S1 Cupiennius salei toxin 12 isoform a S1^ACsTx-12a\_S2 Cupiennius salei toxin 12 isoform a S2 /
Proteoform #53

## Protein-Spectrum-Match #169 for Spectrum #408

|  |  |  |  |  |  |
| --- | --- | --- | --- | --- | --- |
| PrSM ID: | 169 | Scan(s): | 547 | Precursor charge: | 6 |
| Precursor m/z: | 569.3244 | Precursor mass: | 3409.9028 | Proteoform mass: | 3409.8978 |
| # matched peaks: | 30 | # matched fragment ions: | 25 | # unexpected modifications: | 1 |
| E-value: | 1.12e-19 | P-value: | 1.12e-19 | Q-value (Spectral FDR): | 0 |

  

|  |  |  |  |  |  |  |  |  |  |  |  |  |  |  |  |  |  |  |  |  |  |  |  |  |  |  |  |  |  |  |  |  |  |  |  |  |  |  |  |  |  |  |  |  |  |  |  |  |  |  |  |  |  |  |  |  |  |  |  |  |  |  |  |  |  |  |
| --- | --- | --- | --- | --- | --- | --- | --- | --- | --- | --- | --- | --- | --- | --- | --- | --- | --- | --- | --- | --- | --- | --- | --- | --- | --- | --- | --- | --- | --- | --- | --- | --- | --- | --- | --- | --- | --- | --- | --- | --- | --- | --- | --- | --- | --- | --- | --- | --- | --- | --- | --- | --- | --- | --- | --- | --- | --- | --- | --- | --- | --- | --- | --- | --- | --- | --- |
|  | | ... 30 amino acid residues are skipped at the N-terminus ... | | | | | | | | | | | | | | | | | | | | | | | | | | | | | | | | | | | | | | | | | | | | | | | | | | | | | | | | | | | | | |  | | |
|  | |  | | | | | | | | | | | | | | | | | | | | | | | | | | | | | | | | | | | | | | | | | | | | | | | | | | | | | | | | | | | | | | | | | | | |
| 31 |  |  | S |  | F |  | E |  | A |  | D |  | D |  | V |  | I |  | P |  | F |  |  | L |  | A |  | R |  | E |  | Q |  | V |  | R |  | S |  | D |  | C |  |  | T |  | L |  | R |  | N |  | H |  | D |  | C |  | T |  | D |  | D |  | 60 |  |
|  | |  | | | | | | | | | | | | | | | | | | | | | | | | | | | | | | | | | | | | | | | | | | | | | | | | | | | | | | | | | | | | | | | | | | | |
| 61 |  |  | R |  | H |  | S |  | C |  | C |  | R |  | S |  | K |  | M |  | F |  |  | K |  | D |  | V |  | C |  | K |  | C |  | F |  | Y |  | P |  | S |  |  | Q |  | R |  | S |  | D |  | T |  | A |  | R | ] | A | ⎩ | K | ⎩ | K |  | 90 |  |
|  | |  | | | | | | | | | | | | | | | | | | | | | | | | | | | | | | | | | | | | | | | | | | | | | | | | | | | | | -58.01 | | | | | | | | | | | |
| 91 |  | ⎫ | E | ⎫ | L |  | C |  | T | ⎫ | C | ⎫ | Q | ⎫ | Q | ⎫ | D |  | K |  | H |  |  | L |  | K | ⎱ | F | ⎱ | I | ⎫ | E | ⎫ | K |  | G | ⎫ | L |  | Q | ⎱ | K |  | ⎱ | A | ⎱ | K | ⎫ | V | ⎫ | L | ⎫ | V | ⎫ | A |  | G |  | | 117 |  | | | | | |

Fixed PTMs: Carbamidomethylation [C93 C95 ]   
  
     Unexpected modifications:   Unknown [-58.01]

  

All peaks (56)  Matched peaks (30)  Not matched peaks (26)

  

| Scan | Peak | Mono mass | Mono m/z | Intensity | Charge | Theoretical mass | Ion | Pos | Mass error | PPM error |
| --- | --- | --- | --- | --- | --- | --- | --- | --- | --- | --- |
| 547 | 1 | 3352.8647 | 671.5802 | 220532.28 | 5 |  |  |  |  |  |
| 547 | 2 | 3126.6993 | 782.6821 | 91915.74 | 4 | 3126.7157 | C26 | 26 | -0.0165 | -5.26 |
| 547 | 3 | 3408.8980 | 569.1569 | 350467.06 | 6 |  |  |  |  |  |
| 547 | 4 | 3338.8513 | 668.7775 | 78159.07 | 5 | 3338.8682 | C28 | 28 | -0.0169 | -5.05 |
| 547 | 5 | 3352.8668 | 839.2240 | 72045.41 | 4 |  |  |  |  |  |
| 547 | 6 | 2274.1497 | 759.0572 | 65755.08 | 3 | 2274.1612 | C18 | 18 | -0.0115 | -5.05 |
| 547 | 7 | 2145.1078 | 716.0432 | 62032.59 | 3 | 2145.1186 | C17 | 17 | -0.0108 | -5.05 |
| 547 | 8 | 2899.5369 | 725.8915 | 56172.60 | 4 | 2899.5523 | C24 | 24 | -0.0154 | -5.32 |
| 547 | 9 | 1136.9662 | 569.4904 | 304833.78 | 2 |  |  |  |  |  |
| 547 | 10 | 3322.8308 | 665.5734 | 44717.75 | 5 | 3322.8420 | Z\_DOT29 | 1 | -0.0112 | -3.38 |
| 547 | 11 | 1884.9567 | 629.3262 | 70504.01 | 3 | 1884.9662 | C15 | 15 | -9.48e-03 | -5.03 |
| 547 | 12 | 1525.9395 | 763.9770 | 62303.69 | 2 | 1525.9395 | Z\_DOT15 | 15 | -1.18e-05 | -7.74e-03 |
| 547 | 13 | 2828.5005 | 708.1324 | 51485.82 | 4 | 2828.5152 | C23 | 23 | -0.0147 | -5.20 |
| 547 | 14 | 568.6490 | 569.6562 | 275040.67 | 1 |  |  |  |  |  |
| 547 | 15 | 3392.8674 | 566.4852 | 36642.25 | 6 |  |  |  |  |  |
| 547 | 16 | 3194.7378 | 799.6917 | 34518.10 | 4 | 3194.7471 | Z\_DOT28 | 2 | -9.25e-03 | -2.90 |
| 547 | 17 | 3393.8684 | 679.7810 | 34503.86 | 5 |  |  |  |  |  |
| 547 | 18 | 2032.0246 | 678.3488 | 35314.06 | 3 | 2032.0346 | C16 | 16 | -9.96e-03 | -4.90 |
| 547 | 19 | 3322.8337 | 831.7157 | 45205.25 | 4 | 3322.8420 | Z\_DOT29 | 1 | -8.40e-03 | -2.53 |
| 547 | 20 | 2459.2655 | 820.7624 | 33527.00 | 3 | 2459.2776 | C20 | 20 | -0.0121 | -4.93 |
| 547 | 21 | 3365.8740 | 674.1821 | 21878.18 | 5 |  |  |  |  |  |
| 547 | 22 | 2700.4075 | 901.1431 | 31845.49 | 3 | 2700.4203 | C22 | 22 | -0.0127 | -4.72 |
| 547 | 23 | 3027.6319 | 757.9152 | 29420.08 | 4 | 3027.6473 | C25 | 25 | -0.0154 | -5.10 |
| 547 | 24 | 3239.7825 | 810.9529 | 23651.53 | 4 | 3239.7998 | C27 | 27 | -0.0173 | -5.33 |
| 547 | 25 | 3366.8803 | 842.7273 | 24540.10 | 4 |  |  |  |  |  |
| 547 | 26 | 2700.4058 | 676.1087 | 24280.68 | 4 | 2700.4203 | C22 | 22 | -0.0145 | -5.36 |
| 547 | 27 | 3394.8776 | 849.7267 | 26860.10 | 4 |  |  |  |  |  |
| 547 | 28 | 1884.9569 | 943.4857 | 31777.50 | 2 | 1884.9662 | C15 | 15 | -9.29e-03 | -4.93 |
| 547 | 29 | 2345.3831 | 587.3531 | 23817.30 | 4 |  |  |  |  |  |
| 547 | 30 | 1263.6003 | 632.8074 | 34245.48 | 2 | 1263.6063 | C10 | 10 | -6.07e-03 | -4.81 |
| 547 | 31 | 2473.4405 | 619.3674 | 22118.38 | 4 |  |  |  |  |  |
| 547 | 32 | 3408.8866 | 682.7846 | 93780.93 | 5 |  |  |  |  |  |
| 547 | 33 | 3338.8505 | 835.7199 | 19160.35 | 4 | 3338.8682 | C28 | 28 | -0.0177 | -5.30 |
| 547 | 34 | 3210.7549 | 803.6960 | 17190.59 | 4 |  |  |  |  |  |
| 547 | 35 | 1378.8716 | 690.4431 | 21869.11 | 2 | 1378.8711 | Z\_DOT14 | 16 | 5.27e-04 | 0.38 |
| 547 | 36 | 682.1789 | 683.1862 | 73163.62 | 1 |  |  |  |  |  |
| 547 | 37 | 1364.7577 | 683.3861 | 139077.72 | 2 |  |  |  |  |  |
| 547 | 38 | 1007.4847 | 1008.4919 | 16580.03 | 1 | 1007.4892 | C8 | 8 | -4.50e-03 | -4.47 |
| 547 | 39 | 582.3954 | 583.4026 | 16737.81 | 1 | 582.3904 | Z\_DOT7 | 23 | 4.95e-03 | 8.50 |
| 547 | 40 | 710.4898 | 711.4971 | 13053.00 | 1 | 710.4854 | Z\_DOT8 | 22 | 4.41e-03 | 6.21 |
| 547 | 41 | 908.5765 | 455.2955 | 16831.26 | 2 |  |  |  |  |  |
| 547 | 42 | 1206.7752 | 604.3949 | 8194.65 | 2 |  |  |  |  |  |
| 547 | 43 | 847.4547 | 848.4620 | 9904.53 | 1 | 847.4585 | C7 | 7 | -3.82e-03 | -4.50 |
| 547 | 44 | 1135.5427 | 1136.5500 | 7246.30 | 1 | 1135.5477 | C9 | 9 | -5.04e-03 | -4.44 |
| 547 | 45 | 1152.7648 | 577.3897 | 3980.88 | 2 |  |  |  |  |  |
| 547 | 46 | 473.2943 | 474.3016 | 6856.55 | 1 | 473.2961 | C4 | 4 | -1.82e-03 | -3.85 |
| 547 | 47 | 511.3588 | 512.3660 | 8789.08 | 1 | 511.3533 | Z\_DOT6 | 24 | 5.44e-03 | 10.64 |
| 547 | 48 | 1298.6791 | 650.3468 | 3770.53 | 2 |  |  |  |  |  |
| 547 | 49 | 1496.9006 | 499.9742 | 4226.34 | 3 |  |  |  |  |  |
| 547 | 50 | 633.3960 | 634.4033 | 14246.44 | 1 |  |  |  |  |  |
| 547 | 51 | 967.6499 | 484.8322 | 2867.82 | 2 |  |  |  |  |  |
| 547 | 52 | 873.4700 | 874.4773 | 4141.42 | 1 |  |  |  |  |  |
| 547 | 53 | 1078.6818 | 540.3482 | 3966.68 | 2 |  |  |  |  |  |
| 547 | 54 | 550.0771 | 551.0843 | 3461.19 | 1 |  |  |  |  |  |
| 547 | 55 | 344.2525 | 345.2597 | 4100.26 | 1 | 344.2535 | C3 | 3 | -1.07e-03 | -3.11 |
| 547 | 56 | 1007.4847 | 504.7496 | 4186.37 | 2 | 1007.4892 | C8 | 8 | -4.51e-03 | -4.48 |

  

All proteins /
CsTx-12a\_S1 Cupiennius salei toxin 12 isoform a S1^ACsTx-12a\_S2 Cupiennius salei toxin 12 isoform a S2 /
Proteoform #53
