# Supplementary material for: The Dual Prey-Inactivation Strategy of Spiders—In-Depth Venomic Analysis of Cupiennius salei
Source: Toxins (Basel). 2019 Mar 19;11(3):167. doi: 10.3390/toxins11030167 (PMC6468893; doi:10.3390/toxins11030167)
Supplement: Supplementary file 1 [file toxins-11-00167-s001.zip › Supplementary Dataset EV1/20180328_f2_topdown_OTMS2_EThcD_NL_i02_ms2_proteoform_cutoff_html/prsms/prsm17.html]

Protein-Spectrum-Match for Spectrum #229


All proteins /
CsTx-13a Cupiennius salei toxin 13 isoform a /
Proteoform #40

## Protein-Spectrum-Match #17 for Spectrum #229

|  |  |  |  |  |  |
| --- | --- | --- | --- | --- | --- |
| PrSM ID: | 17 | Scan(s): | 308 | Precursor charge: | 6 |
| Precursor m/z: | 580.3140 | Precursor mass: | 3475.8402 | Proteoform mass: | 3475.8352 |
| # matched peaks: | 34 | # matched fragment ions: | 28 | # unexpected modifications: | 1 |
| E-value: | 1.68e-21 | P-value: | 1.68e-21 | Q-value (Spectral FDR): | 0 |

  

|  |  |  |  |  |  |  |  |  |  |  |  |  |  |  |  |  |  |  |  |  |  |  |  |  |  |  |  |  |  |  |  |  |  |  |  |  |  |  |  |  |  |  |  |  |  |  |  |  |  |  |  |  |  |  |  |  |  |  |  |  |  |  |  |  |  |  |
| --- | --- | --- | --- | --- | --- | --- | --- | --- | --- | --- | --- | --- | --- | --- | --- | --- | --- | --- | --- | --- | --- | --- | --- | --- | --- | --- | --- | --- | --- | --- | --- | --- | --- | --- | --- | --- | --- | --- | --- | --- | --- | --- | --- | --- | --- | --- | --- | --- | --- | --- | --- | --- | --- | --- | --- | --- | --- | --- | --- | --- | --- | --- | --- | --- | --- | --- |
|  | | ... 30 amino acid residues are skipped at the N-terminus ... | | | | | | | | | | | | | | | | | | | | | | | | | | | | | | | | | | | | | | | | | | | | | | | | | | | | | | | | | | | | | |  | | |
|  | |  | | | | | | | | | | | | | | | | | | | | | | | | | | | | | | | | | | | | | | | | | | | | | | | | | | | | | | | | | | | | | | | | | | | |
| 31 |  |  | S |  | F |  | E |  | A |  | D |  | D |  | I |  | I |  | P |  | F |  |  | I |  | A |  | K |  | E |  | Q |  | V |  | R |  | S |  | D |  | C |  |  | T |  | L |  | R |  | N |  | H |  | D |  | C |  | T |  | D |  | D |  | 60 |  |
|  | |  | | | | | | | | | | | | | | | | | | | | | | | | | | | | | | | | | | | | | | | | | | | | | | | | | | | | | | | | | | | | | | | | | | | |
| 61 |  |  | R |  | H |  | S |  | C |  | C |  | R |  | S |  | K |  | M |  | F |  |  | K |  | D |  | V |  | C |  | T |  | C |  | F |  | Y |  | P |  | S |  |  | Q |  | R |  | S |  | E |  | T |  | A |  | R | ] | A | ⎩ | K | ⎩ | K |  | 90 |  |
|  | |  | | | | | | | | | | | | | | | | | | | | | | | | | | | | | | | | | | | | | | | | | | | | | | | | | | | | | -58.01 | | | | | | | | | | | |
| 91 |  |  | E | ⎫ | L |  | C |  | T | ⎫ | C | ⎫ | Q | ⎱ | Q |  | P | ⎱ | K | ⎫ | H |  |  | L |  | K | ⎱ | Y | ⎫ | I | ⎱ | E | ⎱ | K | ⎱ | G |  | L |  | Q | ⎱ | K |  | ⎱ | A | ⎫ | K | ⎫ | D | ⎫ | Y | ⎫ | A | ⎫ | T |  | G |  | | 117 |  | | | | | |

Fixed PTMs: Carbamidomethylation [C93 C95 ]   
  
     Unexpected modifications:   Unknown [-58.01]

  

All peaks (57)  Matched peaks (34)  Not matched peaks (23)

  

| Scan | Peak | Mono mass | Mono m/z | Intensity | Charge | Theoretical mass | Ion | Pos | Mass error | PPM error |
| --- | --- | --- | --- | --- | --- | --- | --- | --- | --- | --- |
| 308 | 1 | 3418.7982 | 684.7669 | 44467.42 | 5 |  |  |  |  |  |
| 308 | 2 | 1738.4134 | 580.4784 | 130644.45 | 3 |  |  |  |  |  |
| 308 | 3 | 3474.8214 | 580.1442 | 99675.73 | 6 |  |  |  |  |  |
| 308 | 4 | 2826.5171 | 707.6366 | 30263.69 | 4 | 2826.5360 | C23 | 23 | -0.0188 | -6.66 |
| 308 | 5 | 3025.6482 | 757.4193 | 23688.01 | 4 | 3025.6680 | C25 | 25 | -0.0198 | -6.55 |
| 308 | 6 | 2143.1257 | 715.3825 | 20719.07 | 3 | 2143.1394 | C17 | 17 | -0.0137 | -6.39 |
| 308 | 7 | 2272.1672 | 758.3964 | 19050.80 | 3 | 2272.1820 | C18 | 18 | -0.0147 | -6.48 |
| 308 | 8 | 3418.8002 | 855.7073 | 14978.15 | 4 |  |  |  |  |  |
| 308 | 9 | 3140.6736 | 786.1757 | 18286.16 | 4 | 3140.6950 | C26 | 26 | -0.0214 | -6.80 |
| 308 | 10 | 1866.9803 | 623.3340 | 20441.39 | 3 | 1866.9920 | C15 | 15 | -0.0117 | -6.25 |
| 308 | 11 | 3303.7358 | 661.7544 | 14506.67 | 5 | 3303.7583 | C27 | 27 | -0.0226 | -6.83 |
| 308 | 12 | 579.6381 | 580.6454 | 76008.65 | 1 |  |  |  |  |  |
| 308 | 13 | 2116.1786 | 706.4001 | 14799.66 | 3 | 2116.1839 | Z\_DOT19 | 11 | -5.34e-03 | -2.52 |
| 308 | 14 | 2698.4236 | 900.4818 | 10867.12 | 3 | 2698.4410 | C22 | 22 | -0.0174 | -6.46 |
| 308 | 15 | 2897.5545 | 725.3959 | 13380.13 | 4 | 2897.5731 | C24 | 24 | -0.0186 | -6.42 |
| 308 | 16 | 2800.4505 | 701.1199 | 12508.91 | 4 |  |  |  |  |  |
| 308 | 17 | 1609.8487 | 805.9316 | 16175.12 | 2 | 1609.8511 | Z\_DOT15 | 15 | -2.39e-03 | -1.48 |
| 308 | 18 | 1360.6505 | 681.3325 | 11860.59 | 2 | 1360.6591 | C11 | 11 | -8.54e-03 | -6.28 |
| 308 | 19 | 3388.7634 | 678.7600 | 13409.84 | 5 | 3388.7794 | Z\_DOT29 | 1 | -0.0160 | -4.72 |
| 308 | 20 | 3303.7364 | 826.9414 | 9947.87 | 4 | 3303.7583 | C27 | 27 | -0.0219 | -6.64 |
| 308 | 21 | 3458.7978 | 577.4736 | 11735.92 | 6 |  |  |  |  |  |
| 308 | 22 | 1333.7033 | 667.8589 | 9558.49 | 2 | 1333.7037 | Z\_DOT13 | 17 | -3.80e-04 | -0.28 |
| 308 | 23 | 2030.0427 | 677.6882 | 8589.97 | 3 | 2030.0553 | C16 | 16 | -0.0126 | -6.22 |
| 308 | 24 | 2698.4241 | 675.6133 | 9135.10 | 4 | 2698.4410 | C22 | 22 | -0.0169 | -6.26 |
| 308 | 25 | 3459.8025 | 692.9678 | 7916.38 | 5 |  |  |  |  |  |
| 308 | 26 | 2539.3748 | 635.8510 | 11697.15 | 4 |  |  |  |  |  |
| 308 | 27 | 3374.7760 | 675.9625 | 14273.69 | 5 | 3374.7954 | C28 | 28 | -0.0194 | -5.76 |
| 308 | 28 | 3025.6475 | 1009.5564 | 7551.72 | 3 | 3025.6680 | C25 | 25 | -0.0205 | -6.79 |
| 308 | 29 | 1204.6620 | 603.3383 | 9496.35 | 2 | 1204.6611 | Z\_DOT12 | 18 | 9.49e-04 | 0.79 |
| 308 | 30 | 695.5660 | 696.5732 | 52205.99 | 1 |  |  |  |  |  |
| 308 | 31 | 1390.7304 | 696.3725 | 60613.56 | 2 |  |  |  |  |  |
| 308 | 32 | 3260.6673 | 816.1741 | 7447.59 | 4 | 3260.6845 | Z\_DOT28 | 2 | -0.0172 | -5.27 |
| 308 | 33 | 650.3113 | 651.3186 | 8269.94 | 1 | 650.3071 | Z\_DOT7 | 23 | 4.28e-03 | 6.57 |
| 308 | 34 | 2341.2888 | 781.4369 | 7747.50 | 3 | 2341.2953 | Z\_DOT21 | 9 | -6.45e-03 | -2.76 |
| 308 | 35 | 1738.9197 | 870.4671 | 11424.41 | 2 |  |  |  |  |  |
| 308 | 36 | 564.0603 | 565.0676 | 12970.73 | 1 |  |  |  |  |  |
| 308 | 37 | 2400.2618 | 801.0945 | 4942.69 | 3 | 2400.2769 | C19 | 19 | -0.0151 | -6.31 |
| 308 | 38 | 3374.7777 | 844.7017 | 4812.05 | 4 | 3374.7954 | C28 | 28 | -0.0177 | -5.25 |
| 308 | 39 | 3431.8117 | 858.9602 | 5008.71 | 4 |  |  |  |  |  |
| 308 | 40 | 3082.6468 | 771.6690 | 6845.26 | 4 |  |  |  |  |  |
| 308 | 41 | 1488.7444 | 745.3795 | 5251.07 | 2 | 1488.7540 | C12 | 12 | -9.63e-03 | -6.47 |
| 308 | 42 | 778.4053 | 779.4126 | 3540.35 | 1 | 778.4020 | Z\_DOT8 | 22 | 3.31e-03 | 4.25 |
| 308 | 43 | 847.4538 | 848.4611 | 2833.59 | 1 | 847.4585 | C7 | 7 | -4.67e-03 | -5.51 |
| 308 | 44 | 1135.5409 | 568.7777 | 4846.57 | 2 | 1135.5477 | C9 | 9 | -6.82e-03 | -6.01 |
| 308 | 45 | 356.0579 | 357.0652 | 3766.67 | 1 |  |  |  |  |  |
| 308 | 46 | 1135.5410 | 1136.5483 | 2637.73 | 1 | 1135.5477 | C9 | 9 | -6.75e-03 | -5.94 |
| 308 | 47 | 1007.4831 | 1008.4904 | 2327.13 | 1 | 1007.4892 | C8 | 8 | -6.09e-03 | -6.04 |
| 308 | 48 | 1274.6902 | 638.3524 | 2549.00 | 2 |  |  |  |  |  |
| 308 | 49 | 220.0764 | 221.0837 | 2164.66 | 1 |  |  |  |  |  |
| 308 | 50 | 869.9421 | 870.9494 | 2280.38 | 1 |  |  |  |  |  |
| 308 | 51 | 473.2937 | 474.3010 | 3054.72 | 1 | 473.2961 | C4 | 4 | -2.43e-03 | -5.14 |
| 308 | 52 | 602.6575 | 603.6648 | 2023.35 | 1 |  |  |  |  |  |
| 308 | 53 | 976.4920 | 489.2533 | 1130.39 | 2 |  |  |  |  |  |
| 308 | 54 | 1007.4831 | 504.7488 | 978.38 | 2 | 1007.4892 | C8 | 8 | -6.10e-03 | -6.06 |
| 308 | 55 | 1220.6800 | 611.3473 | 1992.24 | 2 |  |  |  |  |  |
| 308 | 56 | 1098.6213 | 550.3179 | 715.91 | 2 |  |  |  |  |  |
| 308 | 57 | 1076.5684 | 539.2915 | 471.72 | 2 | 1076.5661 | Z\_DOT11 | 19 | 2.28e-03 | 2.12 |

  

All proteins /
CsTx-13a Cupiennius salei toxin 13 isoform a /
Proteoform #40
