# Supplementary material for: The Dual Prey-Inactivation Strategy of Spiders—In-Depth Venomic Analysis of Cupiennius salei
Source: Toxins (Basel). 2019 Mar 19;11(3):167. doi: 10.3390/toxins11030167 (PMC6468893; doi:10.3390/toxins11030167)
Supplement: Supplementary file 1 [file toxins-11-00167-s001.zip › Supplementary Dataset EV1/20180328_f2_topdown_OTMS2_EThcD_NL_i02_ms2_proteoform_cutoff_html/prsms/prsm170.html]

Protein-Spectrum-Match for Spectrum #409


All proteins /
CsTx-1a\_S1 Cupiennius salei toxin 1 isoform a S1^ACsTx-1a\_S2 Cupiennius salei toxin 1 isoform a S2 /
Proteoform #15

## Protein-Spectrum-Match #170 for Spectrum #409

|  |  |  |  |  |  |
| --- | --- | --- | --- | --- | --- |
| PrSM ID: | 170 | Scan(s): | 548 | Precursor charge: | 12 |
| Precursor m/z: | 736.5272 | Precursor mass: | 8826.2393 | Proteoform mass: | 8826.2093 |
| # matched peaks: | 47 | # matched fragment ions: | 37 | # unexpected modifications: | 1 |
| E-value: | 2.08e-31 | P-value: | 2.08e-31 | Q-value (Spectral FDR): | 0 |

  

|  |  |  |  |  |  |  |  |  |  |  |  |  |  |  |  |  |  |  |  |  |  |  |  |  |  |  |  |  |  |  |  |  |  |  |  |  |  |  |  |  |  |  |  |  |  |  |  |  |  |  |  |  |  |  |  |  |  |  |  |  |  |  |  |  |  |  |  |  |  |
| --- | --- | --- | --- | --- | --- | --- | --- | --- | --- | --- | --- | --- | --- | --- | --- | --- | --- | --- | --- | --- | --- | --- | --- | --- | --- | --- | --- | --- | --- | --- | --- | --- | --- | --- | --- | --- | --- | --- | --- | --- | --- | --- | --- | --- | --- | --- | --- | --- | --- | --- | --- | --- | --- | --- | --- | --- | --- | --- | --- | --- | --- | --- | --- | --- | --- | --- | --- | --- | --- |
|  | |  | | | | | | | | | | | | | | | | | | | | | | | | | | | | | | | | | | | | | | | | | | | | | | | | | | | | | | | | | | | | | | | | | | | |
| 1 |  |  | M |  | K |  | V |  | L |  | I |  | I |  | S |  | A |  | V |  | L |  |  | F |  | I |  | T |  | I |  | F |  | S |  | N |  | I |  | S |  | A |  |  | E |  | I |  | E |  | D |  | D |  | F |  | L |  | E |  | D |  | E |  | 30 |  |
|  | |  | | | | | | | | | | | | | | | | | | | | | | | | | | | | | | | | | | | | | | | | | | | | | | | | | | | | | | | | | | | | | | | | | | | |
| 31 |  |  | S |  | F |  | E |  | A |  | E |  | D |  | I |  | I |  | P |  | F |  |  | F |  | E |  | N |  | E |  | Q |  | A |  | R | ] | S | ⎩ | C |  | I |  |  | P |  | K |  | H |  | E | ⎫ | E | ⎫ | C | ⎫ | T | ⎫ | N | ⎱ | D |  | K |  | 60 |  |
|  | |  | | | | | | | | | | | | | | | | | | | | | | | | | | | | | | | | | | | | | | | | | | | | | | | | | | | | | | | | | | | | | | | | | | | |
| 61 |  |  | H | ⎱ | N | ⎫ | C | ⎫ | C |  | R |  | K | ⎫ | G |  | L | ⎫ | F | ⎫ | K |  |  | L |  | K | ⎫ | C |  | Q | ⎫ | C |  | S |  | T |  | F | ⎫ | D | ⎫ | D |  | ⎫ | E | ⎱ | S | ⎫ | G | ⎫ | Q |  | P |  | T | ⎫ | E | ⎫ | R |  | C |  | A |  | 90 |  |
|  | |  | | | | | -42.05 | | | | | | | | | | | | | | | | | | | | | | | | | | | | | | | | | | | | | | | | | | | | | | | | | | | | | | | | | | | |
| 91 |  |  | C | ⎩ | G | ⎱ | R |  | P |  | M |  | G |  | H |  | Q |  | A |  | I |  |  | E |  | T |  | G | ⎫ | L |  | N |  | I |  | F |  | R | ⎫ | G | ⎫ | L |  |  | F |  | K |  | G | ⎫ | K |  | K | ⎫ | K | ⎫ | N | ⎫ | K |  | K |  | T |  | 120 |  |
|  | |  | | | | | | | | | | | | | | | | | | | | | | | | | | | | | | | | | | | | | | | | | | | | | | | | | | | | | | | | | | | | | | | | | | | |
| 121 |  | ⎫ | K | ⎫ | G |  | | | | 122 |  | | | | | | | | | | | | | | | | | | | | | | | | | | | | | | | | | | | | | | | | | | | | | | | | | | | | | | | |

Fixed PTMs: Carbamidomethylation [C49 C56 C63 C64 C73 C75 C89 C91 ]   
  
     Unexpected modifications:   Unknown [-42.05]

  

All peaks (147)  Matched peaks (47)  Not matched peaks (100)

  

| Scan | Peak | Mono mass | Mono m/z | Intensity | Charge | Theoretical mass | Ion | Pos | Mass error | PPM error |
| --- | --- | --- | --- | --- | --- | --- | --- | --- | --- | --- |
| 548 | 1 | 8769.1827 | 877.9255 | 129695.47 | 10 | 8768.2038 | C74 | 74 | -0.0234 | -2.67 |
| 548 | 2 | 8769.1884 | 975.3615 | 99730.01 | 9 | 8768.2038 | C74 | 74 | -0.0177 | -2.02 |
| 548 | 3 | 8769.1790 | 798.2054 | 67023.92 | 11 | 8768.2038 | C74 | 74 | -0.0271 | -3.09 |
| 548 | 4 | 4414.1047 | 883.8282 | 71537.65 | 5 |  |  |  |  |  |
| 548 | 5 | 8783.1884 | 879.3261 | 49491.58 | 10 |  |  |  |  |  |
| 548 | 6 | 8811.1871 | 882.1260 | 51054.49 | 10 |  |  |  |  |  |
| 548 | 7 | 8784.1936 | 977.0288 | 37373.68 | 9 |  |  |  |  |  |
| 548 | 8 | 8725.1728 | 970.4709 | 36695.27 | 9 |  |  |  |  |  |
| 548 | 9 | 8770.1917 | 1097.2812 | 42391.26 | 8 |  |  |  |  |  |
| 548 | 10 | 8712.1535 | 969.0243 | 41220.83 | 9 |  |  |  |  |  |
| 548 | 11 | 8754.1690 | 973.6927 | 36727.00 | 9 |  |  |  |  |  |
| 548 | 12 | 8811.1930 | 980.0287 | 34936.09 | 9 |  |  |  |  |  |
| 548 | 13 | 8753.1762 | 876.3249 | 26417.57 | 10 |  |  |  |  |  |
| 548 | 14 | 4443.9081 | 889.7889 | 34023.34 | 5 | 4443.9333 | C36 | 36 | -0.0252 | -5.67 |
| 548 | 15 | 8727.1781 | 1091.9045 | 19839.29 | 8 |  |  |  |  |  |
| 548 | 16 | 8712.1410 | 1090.0249 | 26124.54 | 8 |  |  |  |  |  |
| 548 | 17 | 8711.1523 | 872.1225 | 28230.88 | 10 |  |  |  |  |  |
| 548 | 18 | 8783.1882 | 799.4789 | 20065.76 | 11 |  |  |  |  |  |
| 548 | 19 | 8697.1265 | 967.3547 | 22109.84 | 9 |  |  |  |  |  |
| 548 | 20 | 4770.0677 | 955.0208 | 19210.63 | 5 | 4770.0923 | C39 | 39 | -0.0246 | -5.15 |
| 548 | 21 | 8810.1873 | 801.9334 | 22146.42 | 11 |  |  |  |  |  |
| 548 | 22 | 2203.3694 | 735.4637 | 19905.28 | 3 |  |  |  |  |  |
| 548 | 23 | 8753.1761 | 1095.1543 | 24254.49 | 8 |  |  |  |  |  |
| 548 | 24 | 3157.5027 | 790.3830 | 16452.63 | 4 | 3157.5153 | C25 | 25 | -0.0126 | -3.99 |
| 548 | 25 | 8698.1429 | 1088.2751 | 16599.08 | 8 |  |  |  |  |  |
| 548 | 26 | 3323.8866 | 831.9789 | 24263.60 | 4 | 3323.8612 | Z\_DOT30 | 45 | 0.0254 | 7.65 |
| 548 | 27 | 4325.2719 | 721.8859 | 17059.62 | 6 |  |  |  |  |  |
| 548 | 28 | 8784.2031 | 1099.0327 | 16673.06 | 8 |  |  |  |  |  |
| 548 | 29 | 2943.0752 | 982.0323 | 21270.09 | 3 |  |  |  |  |  |
| 548 | 30 | 8697.1167 | 870.7189 | 20825.98 | 10 |  |  |  |  |  |
| 548 | 31 | 8813.1976 | 1102.6570 | 13734.90 | 8 |  |  |  |  |  |
| 548 | 32 | 5579.7126 | 930.9594 | 15416.33 | 6 |  |  |  |  |  |
| 548 | 33 | 3445.5856 | 862.4037 | 13694.41 | 4 | 3445.6046 | C27 | 27 | -0.0190 | -5.51 |
| 548 | 34 | 5926.9028 | 847.7077 | 13023.98 | 7 |  |  |  |  |  |
| 548 | 35 | 8099.7301 | 900.9773 | 12869.03 | 9 |  |  |  |  |  |
| 548 | 36 | 2788.2289 | 930.4169 | 16911.89 | 3 | 2788.2414 | C22 | 22 | -0.0124 | -4.46 |
| 548 | 37 | 5503.3302 | 918.2290 | 14453.76 | 6 | 5503.3559 | C45 | 45 | -0.0257 | -4.68 |
| 548 | 38 | 2288.3981 | 763.8066 | 15352.44 | 3 |  |  |  |  |  |
| 548 | 39 | 3998.1291 | 667.3621 | 12614.58 | 6 |  |  |  |  |  |
| 548 | 40 | 8341.8802 | 927.8829 | 16012.60 | 9 |  |  |  |  |  |
| 548 | 41 | 4058.1490 | 812.6371 | 12277.99 | 5 |  |  |  |  |  |
| 548 | 42 | 4384.3047 | 877.8682 | 18355.69 | 5 |  |  |  |  |  |
| 548 | 43 | 3323.8849 | 665.7843 | 11106.41 | 5 | 3323.8612 | Z\_DOT30 | 45 | 0.0237 | 7.13 |
| 548 | 44 | 7969.6230 | 886.5209 | 13099.03 | 9 |  |  |  |  |  |
| 548 | 45 | 8738.1819 | 971.9164 | 11058.01 | 9 |  |  |  |  |  |
| 548 | 46 | 1752.7599 | 877.3872 | 25426.42 | 2 | 1752.7671 | C14 | 14 | -7.21e-03 | -4.11 |
| 548 | 47 | 6225.6520 | 890.3861 | 10285.17 | 7 |  |  |  |  |  |
| 548 | 48 | 8341.8813 | 1043.7424 | 8305.17 | 8 |  |  |  |  |  |
| 548 | 49 | 1169.7793 | 585.8969 | 16552.33 | 2 |  |  |  |  |  |
| 548 | 50 | 1470.5840 | 736.2993 | 19692.74 | 2 |  |  |  |  |  |
| 548 | 51 | 4299.8562 | 860.9785 | 12121.09 | 5 | 4299.8798 | C34 | 34 | -0.0237 | -5.50 |
| 548 | 52 | 8682.1371 | 1086.2744 | 9671.29 | 8 |  |  |  |  |  |
| 548 | 53 | 4443.9124 | 1111.9854 | 9583.02 | 4 | 4443.9333 | C36 | 36 | -0.0209 | -4.71 |
| 548 | 54 | 8724.1697 | 873.4242 | 18798.71 | 10 | 8723.1586 | Z\_DOT74 | 1 | 8.79e-03 | 1.01 |
| 548 | 55 | 8738.1909 | 874.8264 | 10173.91 | 10 |  |  |  |  |  |
| 548 | 56 | 2943.0708 | 736.7750 | 19010.95 | 4 |  |  |  |  |  |
| 548 | 57 | 5927.9136 | 988.9929 | 12646.08 | 6 |  |  |  |  |  |
| 548 | 58 | 8283.8432 | 1036.4877 | 9802.13 | 8 | 8282.8712 | C70 | 70 | -0.0303 | -3.66 |
| 548 | 59 | 8041.7002 | 1006.2198 | 10553.18 | 8 | 8040.7333 | C68 | 68 | -0.0355 | -4.41 |
| 548 | 60 | 3683.8398 | 737.7752 | 21928.96 | 5 |  |  |  |  |  |
| 548 | 61 | 3450.9332 | 691.1939 | 7625.72 | 5 |  |  |  |  |  |
| 548 | 62 | 7076.4704 | 1011.9316 | 10729.29 | 7 |  |  |  |  |  |
| 548 | 63 | 2872.3086 | 958.4435 | 9729.14 | 3 |  |  |  |  |  |
| 548 | 64 | 8681.1599 | 965.5806 | 12401.47 | 9 |  |  |  |  |  |
| 548 | 65 | 8753.1604 | 796.7491 | 12152.44 | 11 |  |  |  |  |  |
| 548 | 66 | 7283.2387 | 1041.4699 | 7328.61 | 7 | 7282.2530 | C61 | 61 | -0.0167 | -2.29 |
| 548 | 67 | 2729.6189 | 910.8802 | 9998.81 | 3 |  |  |  |  |  |
| 548 | 68 | 8099.7379 | 1013.4745 | 8954.65 | 8 |  |  |  |  |  |
| 548 | 69 | 3157.4965 | 1053.5061 | 8347.96 | 3 | 3157.5153 | C25 | 25 | -0.0189 | -5.98 |
| 548 | 70 | 2026.8309 | 1014.4227 | 8758.08 | 2 | 2026.8407 | C16 | 16 | -9.81e-03 | -4.84 |
| 548 | 71 | 2641.1584 | 881.3934 | 10387.40 | 3 | 2641.1730 | C21 | 21 | -0.0145 | -5.50 |
| 548 | 72 | 8641.1027 | 961.1298 | 11083.22 | 9 | 8640.1088 | C73 | 73 | -8.44e-03 | -0.98 |
| 548 | 73 | 6538.8099 | 935.1230 | 10852.28 | 7 |  |  |  |  |  |
| 548 | 74 | 8653.1170 | 962.4647 | 9433.42 | 9 |  |  |  |  |  |
| 548 | 75 | 2617.5787 | 655.4019 | 11477.38 | 4 |  |  |  |  |  |
| 548 | 76 | 8226.8523 | 915.1020 | 9252.17 | 9 |  |  |  |  |  |
| 548 | 77 | 8740.1888 | 1093.5309 | 10429.73 | 8 |  |  |  |  |  |
| 548 | 78 | 7340.2546 | 918.5391 | 11575.65 | 8 | 7339.2745 | C62 | 62 | -0.0222 | -3.03 |
| 548 | 79 | 2671.5892 | 668.9046 | 10325.10 | 4 |  |  |  |  |  |
| 548 | 80 | 7845.7619 | 872.7586 | 10937.86 | 9 |  |  |  |  |  |
| 548 | 81 | 6870.4042 | 859.8078 | 12006.87 | 8 |  |  |  |  |  |
| 548 | 82 | 8598.0473 | 956.3459 | 15526.76 | 9 |  |  |  |  |  |
| 548 | 83 | 8340.8698 | 835.0943 | 9737.92 | 10 |  |  |  |  |  |
| 548 | 84 | 7340.2695 | 1049.6172 | 8366.83 | 7 | 7339.2745 | C62 | 62 | -7.36e-03 | -1.00 |
| 548 | 85 | 1866.8005 | 934.4075 | 18285.50 | 2 | 1866.8101 | C15 | 15 | -9.55e-03 | -5.12 |
| 548 | 86 | 6225.6514 | 1038.6159 | 7936.51 | 6 |  |  |  |  |  |
| 548 | 87 | 4554.9390 | 760.1638 | 8998.00 | 6 |  |  |  |  |  |
| 548 | 88 | 4170.8129 | 835.1699 | 7535.87 | 5 | 4170.8372 | C33 | 33 | -0.0243 | -5.84 |
| 548 | 89 | 2471.0558 | 824.6926 | 8377.43 | 3 | 2471.0674 | C19 | 19 | -0.0116 | -4.69 |
| 548 | 90 | 4527.3501 | 755.5656 | 6487.59 | 6 | 4527.3373 | Z\_DOT41 | 34 | 0.0128 | 2.83 |
| 548 | 91 | 1486.9513 | 744.4829 | 8708.90 | 2 |  |  |  |  |  |
| 548 | 92 | 4055.7862 | 1014.9538 | 7790.65 | 4 | 4055.8103 | C32 | 32 | -0.0241 | -5.94 |
| 548 | 93 | 4271.2594 | 712.8838 | 8402.30 | 6 |  |  |  |  |  |
| 548 | 94 | 4386.8931 | 1097.7305 | 6716.76 | 4 | 4386.9119 | C35 | 35 | -0.0188 | -4.28 |
| 548 | 95 | 8226.8354 | 823.6908 | 9334.01 | 10 |  |  |  |  |  |
| 548 | 96 | 8467.9657 | 847.8038 | 6716.53 | 10 |  |  |  |  |  |
| 548 | 97 | 4384.3029 | 731.7244 | 7403.60 | 6 |  |  |  |  |  |
| 548 | 98 | 3380.9059 | 846.2337 | 8492.95 | 4 | 3380.8827 | Z\_DOT31 | 44 | 0.0232 | 6.87 |
| 548 | 99 | 6639.8626 | 949.5591 | 7627.36 | 7 | 6638.8725 | C56 | 56 | -0.0122 | -1.84 |
| 548 | 100 | 4657.4014 | 932.4876 | 7623.81 | 5 |  |  |  |  |  |
| 548 | 101 | 8654.1127 | 1082.7714 | 7865.86 | 8 |  |  |  |  |  |
| 548 | 102 | 7075.4682 | 885.4408 | 9934.75 | 8 | 7074.4500 | Z\_DOT61 | 14 | 0.0158 | 2.24 |
| 548 | 103 | 6869.4009 | 764.2740 | 6641.21 | 9 |  |  |  |  |  |
| 548 | 104 | 4527.3578 | 906.4788 | 7044.81 | 5 | 4527.3373 | Z\_DOT41 | 34 | 0.0205 | 4.53 |
| 548 | 105 | 2187.3530 | 730.1249 | 7948.12 | 3 |  |  |  |  |  |
| 548 | 106 | 728.4753 | 729.4825 | 9771.63 | 1 |  |  |  |  |  |
| 548 | 107 | 7971.6416 | 997.4625 | 9666.08 | 8 |  |  |  |  |  |
| 548 | 108 | 6097.5960 | 1017.2733 | 10145.12 | 6 |  |  |  |  |  |
| 548 | 109 | 8796.1694 | 978.3594 | 9706.56 | 9 |  |  |  |  |  |
| 548 | 110 | 3929.1088 | 786.8290 | 6414.65 | 5 |  |  |  |  |  |
| 548 | 111 | 2527.0687 | 843.3635 | 8479.80 | 3 |  |  |  |  |  |
| 548 | 112 | 868.4196 | 869.4269 | 13102.48 | 1 | 868.4225 | C7 | 7 | -2.86e-03 | -3.29 |
| 548 | 113 | 8726.1811 | 1247.6046 | 6249.54 | 7 |  |  |  |  |  |
| 548 | 114 | 8168.8262 | 1022.1106 | 7997.40 | 8 | 8168.8283 | C69 | 69 | -2.02e-03 | -0.25 |
| 548 | 115 | 1372.5800 | 687.2973 | 10258.48 | 2 | 1372.5863 | C11 | 11 | -6.33e-03 | -4.61 |
| 548 | 116 | 7682.7295 | 961.3485 | 5409.53 | 8 |  |  |  |  |  |
| 548 | 117 | 7455.6397 | 932.9622 | 9958.66 | 8 | 7454.6308 | Z\_DOT64 | 11 | 6.60e-03 | 0.88 |
| 548 | 118 | 6188.0591 | 885.0157 | 7121.10 | 7 |  |  |  |  |  |
| 548 | 119 | 3940.7541 | 986.1958 | 8049.94 | 4 | 3940.7834 | C31 | 31 | -0.0293 | -7.43 |
| 548 | 120 | 7739.7495 | 860.9794 | 9501.62 | 9 |  |  |  |  |  |
| 548 | 121 | 8712.1580 | 1245.6013 | 6028.74 | 7 |  |  |  |  |  |
| 548 | 122 | 7783.5260 | 865.8435 | 5922.74 | 9 | 7784.5434 | C66 | 66 | -0.0151 | -1.94 |
| 548 | 123 | 6810.9614 | 852.3774 | 6079.81 | 8 |  |  |  |  |  |
| 548 | 124 | 6639.8545 | 830.9891 | 5450.57 | 8 | 6638.8725 | C56 | 56 | -0.0203 | -3.06 |
| 548 | 125 | 4299.8594 | 1075.9721 | 5043.67 | 4 | 4299.8798 | C34 | 34 | -0.0204 | -4.75 |
| 548 | 126 | 4899.1036 | 980.8280 | 6725.42 | 5 | 4899.1349 | C40 | 40 | -0.0313 | -6.39 |
| 548 | 127 | 8598.0648 | 1075.7654 | 7357.82 | 8 |  |  |  |  |  |
| 548 | 128 | 4901.4663 | 817.9183 | 5940.19 | 6 |  |  |  |  |  |
| 548 | 129 | 997.4585 | 998.4658 | 8388.38 | 1 | 997.4651 | C8 | 8 | -6.51e-03 | -6.53 |
| 548 | 130 | 1428.8864 | 477.3027 | 4068.72 | 3 |  |  |  |  |  |
| 548 | 131 | 600.3810 | 601.3883 | 5793.03 | 1 |  |  |  |  |  |
| 548 | 132 | 1316.8505 | 659.4325 | 4128.03 | 2 |  |  |  |  |  |
| 548 | 133 | 1372.5798 | 1373.5871 | 3224.67 | 1 | 1372.5863 | C11 | 11 | -6.50e-03 | -4.73 |
| 548 | 134 | 802.6562 | 803.6635 | 18072.93 | 1 |  |  |  |  |  |
| 548 | 135 | 502.2428 | 503.2501 | 2701.79 | 1 |  |  |  |  |  |
| 548 | 136 | 1185.7981 | 593.9063 | 4036.03 | 2 |  |  |  |  |  |
| 548 | 137 | 834.1636 | 835.1708 | 1541.66 | 1 |  |  |  |  |  |
| 548 | 138 | 486.3387 | 487.3460 | 3531.89 | 1 |  |  |  |  |  |
| 548 | 139 | 1157.4923 | 1158.4996 | 1763.74 | 1 | 1157.4957 | C9 | 9 | -3.41e-03 | -2.95 |
| 548 | 140 | 1386.8768 | 463.2995 | 1634.16 | 3 |  |  |  |  |  |
| 548 | 141 | 895.4370 | 896.4443 | 1733.70 | 1 |  |  |  |  |  |
| 548 | 142 | 428.2733 | 429.2805 | 2702.33 | 1 |  |  |  |  |  |
| 548 | 143 | 983.6203 | 492.8174 | 1527.84 | 2 |  |  |  |  |  |
| 548 | 144 | 1258.5386 | 1259.5458 | 3434.07 | 1 | 1258.5434 | C10 | 10 | -4.83e-03 | -3.84 |
| 548 | 145 | 1029.4831 | 1030.4904 | 1408.38 | 1 |  |  |  |  |  |
| 548 | 146 | 1057.7034 | 529.8590 | 1716.78 | 2 |  |  |  |  |  |
| 548 | 147 | 1258.7821 | 630.3983 | 910.48 | 2 |  |  |  |  |  |

  

All proteins /
CsTx-1a\_S1 Cupiennius salei toxin 1 isoform a S1^ACsTx-1a\_S2 Cupiennius salei toxin 1 isoform a S2 /
Proteoform #15
