# Supplementary material for: The Dual Prey-Inactivation Strategy of Spiders—In-Depth Venomic Analysis of Cupiennius salei
Source: Toxins (Basel). 2019 Mar 19;11(3):167. doi: 10.3390/toxins11030167 (PMC6468893; doi:10.3390/toxins11030167)
Supplement: Supplementary file 1 [file toxins-11-00167-s001.zip › Supplementary Dataset EV1/20180328_f2_topdown_OTMS2_EThcD_NL_i02_ms2_proteoform_cutoff_html/prsms/prsm171.html]

Protein-Spectrum-Match for Spectrum #410


All proteins /
CsTx-1a\_S1 Cupiennius salei toxin 1 isoform a S1^ACsTx-1a\_S2 Cupiennius salei toxin 1 isoform a S2 /
Proteoform #15

## Protein-Spectrum-Match #171 for Spectrum #410

|  |  |  |  |  |  |
| --- | --- | --- | --- | --- | --- |
| PrSM ID: | 171 | Scan(s): | 549 | Precursor charge: | 13 |
| Precursor m/z: | 679.9492 | Precursor mass: | 8826.2456 | Proteoform mass: | 8826.2056 |
| # matched peaks: | 59 | # matched fragment ions: | 40 | # unexpected modifications: | 1 |
| E-value: | 7.56e-33 | P-value: | 7.56e-33 | Q-value (Spectral FDR): | 0 |

  

|  |  |  |  |  |  |  |  |  |  |  |  |  |  |  |  |  |  |  |  |  |  |  |  |  |  |  |  |  |  |  |  |  |  |  |  |  |  |  |  |  |  |  |  |  |  |  |  |  |  |  |  |  |  |  |  |  |  |  |  |  |  |  |  |  |  |  |  |  |  |
| --- | --- | --- | --- | --- | --- | --- | --- | --- | --- | --- | --- | --- | --- | --- | --- | --- | --- | --- | --- | --- | --- | --- | --- | --- | --- | --- | --- | --- | --- | --- | --- | --- | --- | --- | --- | --- | --- | --- | --- | --- | --- | --- | --- | --- | --- | --- | --- | --- | --- | --- | --- | --- | --- | --- | --- | --- | --- | --- | --- | --- | --- | --- | --- | --- | --- | --- | --- | --- | --- |
|  | |  | | | | | | | | | | | | | | | | | | | | | | | | | | | | | | | | | | | | | | | | | | | | | | | | | | | | | | | | | | | | | | | | | | | |
| 1 |  |  | M |  | K |  | V |  | L |  | I |  | I |  | S |  | A |  | V |  | L |  |  | F |  | I |  | T |  | I |  | F |  | S |  | N |  | I |  | S |  | A |  |  | E |  | I |  | E |  | D |  | D |  | F |  | L |  | E |  | D |  | E |  | 30 |  |
|  | |  | | | | | | | | | | | | | | | | | | | | | | | | | | | | | | | | | | | | | | | | | | | | | | | | | | | | | | | | | | | | | | | | | | | |
| 31 |  |  | S |  | F |  | E |  | A |  | E |  | D |  | I |  | I |  | P |  | F |  |  | F |  | E |  | N |  | E |  | Q |  | A |  | R | ] | S |  | C |  | I |  |  | P |  | K |  | H |  | E | ⎫ | E | ⎫ | C |  | T | ⎫ | N | ⎱ | D |  | K |  | 60 |  |
|  | |  | | | | | | | | | | | | | | | | | | | | | | | | | | | | | | | | | | | | | | | | | | | | | | | | | | | | | | | | | | | | | | | | | | | |
| 61 |  | ⎫ | H | ⎫ | N | ⎫ | C | ⎫ | C |  | R | ⎫ | K | ⎫ | G | ⎱ | L | ⎱ | F | ⎫ | K |  | ⎫ | L | ⎫ | K | ⎫ | C |  | Q | ⎫ | C |  | S |  | T |  | F | ⎫ | D | ⎫ | D |  |  | E | ⎫ | S | ⎫ | G | ⎱ | Q |  | P |  | T | ⎫ | E | ⎫ | R |  | C |  | A |  | 90 |  |
|  | |  | | | | | -42.05 | | | | | | | | | | | | | | | | | | | | | | | | | | | | | | | | | | | | | | | | | | | | | | | | | | | | | | | | | | | |
| 91 |  |  | C | ⎫ | G | ⎱ | R |  | P |  | M |  | G |  | H | ⎫ | Q | ⎫ | A |  | I |  |  | E |  | T |  | G | ⎫ | L |  | N |  | I |  | F |  | R | ⎫ | G | ⎫ | L |  |  | F |  | K |  | G |  | K | ⎫ | K | ⎫ | K |  | N |  | K |  | K |  | T |  | 120 |  |
|  | |  | | | | | | | | | | | | | | | | | | | | | | | | | | | | | | | | | | | | | | | | | | | | | | | | | | | | | | | | | | | | | | | | | | | |
| 121 |  | ⎫ | K | ⎫ | G |  | | | | 122 |  | | | | | | | | | | | | | | | | | | | | | | | | | | | | | | | | | | | | | | | | | | | | | | | | | | | | | | | |

Fixed PTMs: Carbamidomethylation [C49 C56 C63 C64 C73 C75 C89 C91 ]   
  
     Unexpected modifications:   Unknown [-42.05]

  

All peaks (147)  Matched peaks (59)  Not matched peaks (88)

  

| Scan | Peak | Mono mass | Mono m/z | Intensity | Charge | Theoretical mass | Ion | Pos | Mass error | PPM error |
| --- | --- | --- | --- | --- | --- | --- | --- | --- | --- | --- |
| 549 | 1 | 8770.1824 | 878.0255 | 79421.92 | 10 |  |  |  |  |  |
| 549 | 2 | 8769.1703 | 798.2046 | 70999.12 | 11 | 8768.2000 | C74 | 74 | -0.0321 | -3.66 |
| 549 | 3 | 8770.1882 | 975.4726 | 43884.06 | 9 |  |  |  |  |  |
| 549 | 4 | 8712.1590 | 969.0249 | 28796.21 | 9 |  |  |  |  |  |
| 549 | 5 | 8783.1860 | 879.3259 | 28050.93 | 10 |  |  |  |  |  |
| 549 | 6 | 8726.1731 | 970.5821 | 23363.31 | 9 |  |  |  |  |  |
| 549 | 7 | 8811.1883 | 802.0244 | 25476.30 | 11 |  |  |  |  |  |
| 549 | 8 | 8754.1790 | 876.4252 | 24213.84 | 10 |  |  |  |  |  |
| 549 | 9 | 8754.1773 | 973.6936 | 26803.32 | 9 |  |  |  |  |  |
| 549 | 10 | 8725.1678 | 873.5241 | 24285.36 | 10 |  |  |  |  |  |
| 549 | 11 | 8712.1405 | 872.2213 | 27724.92 | 10 |  |  |  |  |  |
| 549 | 12 | 4443.9112 | 889.7895 | 27578.43 | 5 | 4443.9333 | C36 | 36 | -0.0221 | -4.98 |
| 549 | 13 | 8769.1792 | 731.7722 | 25895.52 | 12 | 8768.2000 | C74 | 74 | -0.0231 | -2.64 |
| 549 | 14 | 8812.1974 | 882.2270 | 27297.21 | 10 |  |  |  |  |  |
| 549 | 15 | 8783.1888 | 799.4790 | 25665.56 | 11 |  |  |  |  |  |
| 549 | 16 | 2528.0765 | 633.0264 | 22071.33 | 4 | 2528.0889 | C20 | 20 | -0.0124 | -4.92 |
| 549 | 17 | 8784.1985 | 977.0293 | 15840.97 | 9 |  |  |  |  |  |
| 549 | 18 | 4413.1011 | 736.5241 | 28715.10 | 6 |  |  |  |  |  |
| 549 | 19 | 8812.1988 | 980.1405 | 15854.53 | 9 |  |  |  |  |  |
| 549 | 20 | 8770.1956 | 1097.2817 | 16090.21 | 8 |  |  |  |  |  |
| 549 | 21 | 4383.3042 | 731.5580 | 14150.79 | 6 | 4383.2801 | Z\_DOT39 | 36 | 0.0241 | 5.50 |
| 549 | 22 | 4443.9119 | 741.6593 | 21487.24 | 6 | 4443.9333 | C36 | 36 | -0.0214 | -4.82 |
| 549 | 23 | 2471.0550 | 618.7710 | 18552.92 | 4 | 2471.0674 | C19 | 19 | -0.0125 | -5.04 |
| 549 | 24 | 4770.0702 | 955.0213 | 15705.21 | 5 | 4770.0923 | C39 | 39 | -0.0221 | -4.64 |
| 549 | 25 | 3157.5005 | 790.3824 | 15680.92 | 4 | 3157.5153 | C25 | 25 | -0.0148 | -4.69 |
| 549 | 26 | 1752.7599 | 877.3872 | 24483.48 | 2 | 1752.7671 | C14 | 14 | -7.21e-03 | -4.11 |
| 549 | 27 | 8752.1593 | 796.6581 | 18341.68 | 11 |  |  |  |  |  |
| 549 | 28 | 3157.5010 | 632.5075 | 13452.30 | 5 | 3157.5153 | C25 | 25 | -0.0143 | -4.54 |
| 549 | 29 | 1372.5800 | 687.2973 | 17469.53 | 2 | 1372.5863 | C11 | 11 | -6.33e-03 | -4.61 |
| 549 | 30 | 4414.6109 | 883.9295 | 28402.86 | 5 |  |  |  |  |  |
| 549 | 31 | 8698.1245 | 967.4656 | 14233.29 | 9 |  |  |  |  |  |
| 549 | 32 | 8697.1179 | 870.7191 | 15657.83 | 10 |  |  |  |  |  |
| 549 | 33 | 8809.1826 | 735.1058 | 11008.86 | 12 |  |  |  |  |  |
| 549 | 34 | 4384.3090 | 877.8691 | 18770.06 | 5 |  |  |  |  |  |
| 549 | 35 | 2528.0747 | 843.6988 | 17195.72 | 3 | 2528.0889 | C20 | 20 | -0.0142 | -5.61 |
| 549 | 36 | 8228.8200 | 915.3206 | 8753.53 | 9 |  |  |  |  |  |
| 549 | 37 | 4299.8598 | 860.9792 | 11212.81 | 5 | 4299.8798 | C34 | 34 | -0.0200 | -4.65 |
| 549 | 38 | 5579.7144 | 930.9597 | 11238.49 | 6 |  |  |  |  |  |
| 549 | 39 | 3323.8852 | 665.7843 | 14318.65 | 5 | 3323.8575 | Z\_DOT30 | 45 | 0.0277 | 8.34 |
| 549 | 40 | 2916.3233 | 730.0881 | 12995.65 | 4 | 2916.3363 | C23 | 23 | -0.0130 | -4.47 |
| 549 | 41 | 8641.1022 | 961.1297 | 11348.61 | 9 | 8640.1051 | C73 | 73 | -5.26e-03 | -0.61 |
| 549 | 42 | 8726.1830 | 1091.7801 | 10964.61 | 8 |  |  |  |  |  |
| 549 | 43 | 8725.1656 | 794.2042 | 11491.03 | 11 |  |  |  |  |  |
| 549 | 44 | 4055.7894 | 812.1652 | 12073.91 | 5 | 4055.8103 | C32 | 32 | -0.0209 | -5.15 |
| 549 | 45 | 3445.5868 | 690.1246 | 12076.34 | 5 | 3445.6046 | C27 | 27 | -0.0177 | -5.15 |
| 549 | 46 | 2471.0553 | 824.6924 | 11522.05 | 3 | 2471.0674 | C19 | 19 | -0.0121 | -4.91 |
| 549 | 47 | 4554.9448 | 760.1648 | 13035.60 | 6 |  |  |  |  |  |
| 549 | 48 | 8712.1528 | 793.0212 | 12849.92 | 11 |  |  |  |  |  |
| 549 | 49 | 6299.1353 | 900.8838 | 14207.14 | 7 | 6299.1245 | Z\_DOT55 | 20 | 0.0108 | 1.72 |
| 549 | 50 | 5503.3316 | 918.2292 | 11927.60 | 6 | 5503.3559 | C45 | 45 | -0.0243 | -4.41 |
| 549 | 51 | 8713.1640 | 1090.1528 | 11948.80 | 8 |  |  |  |  |  |
| 549 | 52 | 8226.8360 | 823.6909 | 9291.14 | 10 |  |  |  |  |  |
| 549 | 53 | 2788.2300 | 930.4173 | 12619.56 | 3 | 2788.2414 | C22 | 22 | -0.0113 | -4.06 |
| 549 | 54 | 2033.2639 | 678.7619 | 22300.04 | 3 |  |  |  |  |  |
| 549 | 55 | 3445.5844 | 862.4034 | 10216.30 | 4 | 3445.6046 | C27 | 27 | -0.0202 | -5.87 |
| 549 | 56 | 3323.8871 | 831.9791 | 16429.52 | 4 | 3323.8575 | Z\_DOT30 | 45 | 0.0296 | 8.92 |
| 549 | 57 | 2203.3688 | 735.4636 | 9895.95 | 3 |  |  |  |  |  |
| 549 | 58 | 2641.1607 | 661.2974 | 11413.96 | 4 | 2641.1730 | C21 | 21 | -0.0123 | -4.65 |
| 549 | 59 | 7283.2415 | 911.4125 | 7629.07 | 8 | 7282.2493 | C61 | 61 | -0.0101 | -1.39 |
| 549 | 60 | 8098.7330 | 810.8806 | 9189.88 | 10 |  |  |  |  |  |
| 549 | 61 | 6040.5740 | 1007.7696 | 8306.90 | 6 | 6039.5773 | C50 | 50 | -5.62e-03 | -0.93 |
| 549 | 62 | 1169.7796 | 585.8971 | 12888.69 | 2 |  |  |  |  |  |
| 549 | 63 | 2288.3975 | 763.8065 | 10854.68 | 3 |  |  |  |  |  |
| 549 | 64 | 4058.1466 | 677.3650 | 9694.94 | 6 |  |  |  |  |  |
| 549 | 65 | 3029.4063 | 758.3588 | 9410.07 | 4 | 3029.4204 | C24 | 24 | -0.0141 | -4.65 |
| 549 | 66 | 4325.2719 | 721.8859 | 14373.19 | 6 |  |  |  |  |  |
| 549 | 67 | 8699.1185 | 791.8362 | 10285.06 | 11 |  |  |  |  |  |
| 549 | 68 | 3998.1298 | 667.3622 | 10996.10 | 6 |  |  |  |  |  |
| 549 | 69 | 8754.1654 | 1095.2779 | 10083.93 | 8 |  |  |  |  |  |
| 549 | 70 | 8654.1286 | 962.5771 | 10399.74 | 9 |  |  |  |  |  |
| 549 | 71 | 6097.5914 | 872.0918 | 18332.88 | 7 |  |  |  |  |  |
| 549 | 72 | 1866.8014 | 934.4080 | 15561.32 | 2 | 1866.8101 | C15 | 15 | -8.70e-03 | -4.66 |
| 549 | 73 | 2788.2283 | 698.0644 | 9375.46 | 4 | 2788.2414 | C22 | 22 | -0.0130 | -4.68 |
| 549 | 74 | 7341.2638 | 918.6653 | 8073.16 | 8 |  |  |  |  |  |
| 549 | 75 | 3681.9967 | 737.4066 | 6598.47 | 5 |  |  |  |  |  |
| 549 | 76 | 4171.8162 | 835.3705 | 8817.45 | 5 |  |  |  |  |  |
| 549 | 77 | 6256.0788 | 783.0171 | 10429.15 | 8 |  |  |  |  |  |
| 549 | 78 | 7454.6338 | 829.2999 | 7482.34 | 9 | 7454.6271 | Z\_DOT64 | 11 | 6.74e-03 | 0.90 |
| 549 | 79 | 2617.5789 | 655.4020 | 11879.78 | 4 |  |  |  |  |  |
| 549 | 80 | 6300.1064 | 788.5206 | 11020.67 | 8 | 6299.1245 | Z\_DOT55 | 20 | -0.0204 | -3.24 |
| 549 | 81 | 2671.5889 | 668.9045 | 9577.62 | 4 |  |  |  |  |  |
| 549 | 82 | 7339.2556 | 816.4801 | 7047.74 | 9 | 7339.2708 | C62 | 62 | -0.0152 | -2.07 |
| 549 | 83 | 8640.1037 | 865.0176 | 9413.65 | 10 | 8640.1051 | C73 | 73 | -1.34e-03 | -0.16 |
| 549 | 84 | 8041.7315 | 1006.2237 | 6695.11 | 8 | 8040.7296 | C68 | 68 | -4.77e-04 | -0.06 |
| 549 | 85 | 8738.1530 | 874.8226 | 9084.46 | 10 |  |  |  |  |  |
| 549 | 86 | 2026.8319 | 1014.4232 | 7204.98 | 2 | 2026.8407 | C16 | 16 | -8.84e-03 | -4.36 |
| 549 | 87 | 4386.8921 | 1097.7303 | 7937.80 | 4 | 4386.9119 | C35 | 35 | -0.0197 | -4.50 |
| 549 | 88 | 4769.0661 | 795.8516 | 7067.84 | 6 |  |  |  |  |  |
| 549 | 89 | 8696.1361 | 1088.0243 | 7797.27 | 8 |  |  |  |  |  |
| 549 | 90 | 8666.1390 | 867.6212 | 6087.51 | 10 |  |  |  |  |  |
| 549 | 91 | 5926.9079 | 847.7084 | 7672.32 | 7 |  |  |  |  |  |
| 549 | 92 | 1615.7007 | 808.8576 | 12001.07 | 2 | 1615.7082 | C13 | 13 | -7.50e-03 | -4.64 |
| 549 | 93 | 6226.6534 | 890.5292 | 6952.10 | 7 |  |  |  |  |  |
| 549 | 94 | 7913.6094 | 990.2084 | 7829.11 | 8 | 7912.6346 | C67 | 67 | -0.0276 | -3.49 |
| 549 | 95 | 7913.6089 | 880.2971 | 8988.10 | 9 | 7912.6346 | C67 | 67 | -0.0281 | -3.55 |
| 549 | 96 | 8342.8849 | 927.9945 | 7346.82 | 9 |  |  |  |  |  |
| 549 | 97 | 4528.3561 | 755.7333 | 6866.85 | 6 |  |  |  |  |  |
| 549 | 98 | 1486.9521 | 744.4833 | 8984.94 | 2 |  |  |  |  |  |
| 549 | 99 | 6040.9967 | 864.0068 | 6524.05 | 7 |  |  |  |  |  |
| 549 | 100 | 3940.7626 | 789.1598 | 9057.95 | 5 | 3940.7834 | C31 | 31 | -0.0208 | -5.27 |
| 549 | 101 | 6870.4149 | 764.3867 | 6267.00 | 9 |  |  |  |  |  |
| 549 | 102 | 2203.3697 | 551.8497 | 8477.64 | 4 |  |  |  |  |  |
| 549 | 103 | 6097.5938 | 1017.2729 | 6785.33 | 6 |  |  |  |  |  |
| 549 | 104 | 2641.1592 | 881.3937 | 7477.52 | 3 | 2641.1730 | C21 | 21 | -0.0138 | -5.22 |
| 549 | 105 | 6225.6491 | 779.2134 | 6320.52 | 8 |  |  |  |  |  |
| 549 | 106 | 3940.7650 | 986.1985 | 6165.32 | 4 | 3940.7834 | C31 | 31 | -0.0184 | -4.67 |
| 549 | 107 | 728.4756 | 729.4828 | 8452.78 | 1 |  |  |  |  |  |
| 549 | 108 | 7970.6386 | 886.6338 | 8943.95 | 9 |  |  |  |  |  |
| 549 | 109 | 8667.1568 | 964.0247 | 8449.95 | 9 |  |  |  |  |  |
| 549 | 110 | 7455.6426 | 932.9626 | 8642.65 | 8 | 7454.6271 | Z\_DOT64 | 11 | 0.0132 | 1.77 |
| 549 | 111 | 6809.9741 | 852.2540 | 5338.11 | 8 |  |  |  |  |  |
| 549 | 112 | 997.4599 | 998.4672 | 8359.23 | 1 | 997.4651 | C8 | 8 | -5.17e-03 | -5.18 |
| 549 | 113 | 6168.6372 | 1029.1135 | 5442.02 | 6 | 6167.6358 | C51 | 51 | -9.75e-04 | -0.16 |
| 549 | 114 | 6870.4067 | 859.8081 | 7417.65 | 8 |  |  |  |  |  |
| 549 | 115 | 2601.5599 | 651.3972 | 7136.56 | 4 |  |  |  |  |  |
| 549 | 116 | 4444.9186 | 1112.2369 | 6344.27 | 4 |  |  |  |  |  |
| 549 | 117 | 5446.3090 | 908.7254 | 7542.92 | 6 | 5446.3345 | C44 | 44 | -0.0255 | -4.68 |
| 549 | 118 | 8284.8387 | 921.5449 | 7644.67 | 9 |  |  |  |  |  |
| 549 | 119 | 8170.8198 | 1022.3598 | 4811.84 | 8 |  |  |  |  |  |
| 549 | 120 | 2342.9631 | 781.9950 | 6600.23 | 3 | 2342.9725 | C18 | 18 | -9.33e-03 | -3.98 |
| 549 | 121 | 2017.2473 | 673.4230 | 7097.82 | 3 |  |  |  |  |  |
| 549 | 122 | 6639.8583 | 949.5585 | 6534.35 | 7 | 6638.8687 | C56 | 56 | -0.0128 | -1.93 |
| 549 | 123 | 6300.1487 | 1051.0321 | 5270.15 | 6 | 6299.1245 | Z\_DOT55 | 20 | 0.0218 | 3.46 |
| 549 | 124 | 8641.0922 | 1081.1438 | 6659.76 | 8 | 8640.1051 | C73 | 73 | -0.0153 | -1.77 |
| 549 | 125 | 8340.8747 | 835.0947 | 8133.39 | 10 |  |  |  |  |  |
| 549 | 126 | 4899.1062 | 980.8285 | 6340.10 | 5 | 4899.1349 | C40 | 40 | -0.0287 | -5.86 |
| 549 | 127 | 6187.0538 | 884.8721 | 8345.59 | 7 | 6186.0404 | Z\_DOT54 | 21 | 0.0110 | 1.77 |
| 549 | 128 | 868.4189 | 869.4261 | 10536.54 | 1 | 868.4225 | C7 | 7 | -3.59e-03 | -4.14 |
| 549 | 129 | 1428.8864 | 477.3027 | 5425.71 | 3 |  |  |  |  |  |
| 549 | 130 | 1258.5381 | 1259.5453 | 4428.87 | 1 | 1258.5434 | C10 | 10 | -5.32e-03 | -4.22 |
| 549 | 131 | 1258.5375 | 630.2760 | 4803.73 | 2 | 1258.5434 | C10 | 10 | -5.88e-03 | -4.67 |
| 549 | 132 | 600.3810 | 601.3883 | 8243.36 | 1 |  |  |  |  |  |
| 549 | 133 | 1185.7979 | 593.9062 | 3222.14 | 2 |  |  |  |  |  |
| 549 | 134 | 1386.8764 | 463.2994 | 4444.79 | 3 |  |  |  |  |  |
| 549 | 135 | 1472.2039 | 737.1092 | 15885.83 | 2 |  |  |  |  |  |
| 549 | 136 | 1316.8501 | 659.4323 | 4863.43 | 2 |  |  |  |  |  |
| 549 | 137 | 1372.5802 | 1373.5875 | 2806.73 | 1 | 1372.5863 | C11 | 11 | -6.13e-03 | -4.47 |
| 549 | 138 | 678.0312 | 679.0385 | 3521.09 | 1 |  |  |  |  |  |
| 549 | 139 | 802.8389 | 803.8462 | 16797.52 | 1 |  |  |  |  |  |
| 549 | 140 | 486.3386 | 487.3459 | 4423.44 | 1 |  |  |  |  |  |
| 549 | 141 | 1486.9514 | 496.6577 | 2569.02 | 3 |  |  |  |  |  |
| 549 | 142 | 1430.9358 | 716.4752 | 2196.03 | 2 |  |  |  |  |  |
| 549 | 143 | 798.5047 | 400.2596 | 3283.86 | 2 |  |  |  |  |  |
| 549 | 144 | 1357.5796 | 679.7971 | 2410.35 | 2 |  |  |  |  |  |
| 549 | 145 | 786.8301 | 787.8374 | 2355.42 | 1 |  |  |  |  |  |
| 549 | 146 | 502.2428 | 503.2501 | 3271.20 | 1 |  |  |  |  |  |
| 549 | 147 | 997.4611 | 499.7378 | 2194.97 | 2 | 997.4651 | C8 | 8 | -3.96e-03 | -3.97 |

  

All proteins /
CsTx-1a\_S1 Cupiennius salei toxin 1 isoform a S1^ACsTx-1a\_S2 Cupiennius salei toxin 1 isoform a S2 /
Proteoform #15
