# Supplementary material for: The Dual Prey-Inactivation Strategy of Spiders—In-Depth Venomic Analysis of Cupiennius salei
Source: Toxins (Basel). 2019 Mar 19;11(3):167. doi: 10.3390/toxins11030167 (PMC6468893; doi:10.3390/toxins11030167)
Supplement: Supplementary file 1 [file toxins-11-00167-s001.zip › Supplementary Dataset EV1/20180328_f2_topdown_OTMS2_EThcD_NL_i02_ms2_proteoform_cutoff_html/prsms/prsm172.html]

Protein-Spectrum-Match for Spectrum #411


All proteins /
CsTx-1a\_S1 Cupiennius salei toxin 1 isoform a S1^ACsTx-1a\_S2 Cupiennius salei toxin 1 isoform a S2 /
Proteoform #15

## Protein-Spectrum-Match #172 for Spectrum #411

|  |  |  |  |  |  |
| --- | --- | --- | --- | --- | --- |
| PrSM ID: | 172 | Scan(s): | 551 | Precursor charge: | 14 |
| Precursor m/z: | 631.5242 | Precursor mass: | 8827.2371 | Proteoform mass: | 8827.2021 |
| # matched peaks: | 53 | # matched fragment ions: | 36 | # unexpected modifications: | 1 |
| E-value: | 6.28e-31 | P-value: | 6.28e-31 | Q-value (Spectral FDR): | 0 |

  

|  |  |  |  |  |  |  |  |  |  |  |  |  |  |  |  |  |  |  |  |  |  |  |  |  |  |  |  |  |  |  |  |  |  |  |  |  |  |  |  |  |  |  |  |  |  |  |  |  |  |  |  |  |  |  |  |  |  |  |  |  |  |  |  |  |  |  |  |  |  |
| --- | --- | --- | --- | --- | --- | --- | --- | --- | --- | --- | --- | --- | --- | --- | --- | --- | --- | --- | --- | --- | --- | --- | --- | --- | --- | --- | --- | --- | --- | --- | --- | --- | --- | --- | --- | --- | --- | --- | --- | --- | --- | --- | --- | --- | --- | --- | --- | --- | --- | --- | --- | --- | --- | --- | --- | --- | --- | --- | --- | --- | --- | --- | --- | --- | --- | --- | --- | --- | --- |
|  | |  | | | | | | | | | | | | | | | | | | | | | | | | | | | | | | | | | | | | | | | | | | | | | | | | | | | | | | | | | | | | | | | | | | | |
| 1 |  |  | M |  | K |  | V |  | L |  | I |  | I |  | S |  | A |  | V |  | L |  |  | F |  | I |  | T |  | I |  | F |  | S |  | N |  | I |  | S |  | A |  |  | E |  | I |  | E |  | D |  | D |  | F |  | L |  | E |  | D |  | E |  | 30 |  |
|  | |  | | | | | | | | | | | | | | | | | | | | | | | | | | | | | | | | | | | | | | | | | | | | | | | | | | | | | | | | | | | | | | | | | | | |
| 31 |  |  | S |  | F |  | E |  | A |  | E |  | D |  | I |  | I |  | P |  | F |  |  | F |  | E |  | N |  | E |  | Q |  | A |  | R | ] | S | ⎩ | C |  | I |  |  | P |  | K |  | H |  | E | ⎫ | E | ⎫ | C |  | T | ⎫ | N | ⎱ | D |  | K |  | 60 |  |
|  | |  | | | | | | | | | | | | | | | | | | | | | | | | | | | | | | | | | | | | | | | | | | | | | | | | | | | | | | | | | | | | | | | | | | | |
| 61 |  | ⎫ | H | ⎫ | N | ⎫ | C | ⎫ | C |  | R | ⎫ | K | ⎱ | G | ⎱ | L | ⎱ | F | ⎫ | K |  | ⎫ | L | ⎫ | K | ⎫ | C |  | Q | ⎫ | C |  | S |  | T |  | F | ⎫ | D | ⎫ | D |  |  | E | ⎫ | S |  | G | ⎫ | Q |  | P |  | T | ⎱ | E |  | R |  | C |  | A |  | 90 |  |
|  | |  | | | | | -41.06 | | | | | | | | | | | | | | | | | | | | | | | | | | | | | | | | | | | | | | | | | | | | | | | | | | | | | | | | | | | |
| 91 |  |  | C | ⎫ | G | ⎫ | R |  | P |  | M |  | G |  | H | ⎫ | Q |  | A |  | I |  |  | E |  | T |  | G | ⎫ | L |  | N |  | I |  | F |  | R |  | G | ⎫ | L |  |  | F |  | K |  | G |  | K | ⎫ | K |  | K |  | N |  | K |  | K |  | T |  | 120 |  |
|  | |  | | | | | | | | | | | | | | | | | | | | | | | | | | | | | | | | | | | | | | | | | | | | | | | | | | | | | | | | | | | | | | | | | | | |
| 121 |  | ⎫ | K | ⎫ | G |  | | | | 122 |  | | | | | | | | | | | | | | | | | | | | | | | | | | | | | | | | | | | | | | | | | | | | | | | | | | | | | | | |

Fixed PTMs: Carbamidomethylation [C49 C56 C63 C64 C73 C75 C89 C91 ]   
  
     Unexpected modifications:   Unknown [-41.06]

  

All peaks (147)  Matched peaks (53)  Not matched peaks (94)

  

| Scan | Peak | Mono mass | Mono m/z | Intensity | Charge | Theoretical mass | Ion | Pos | Mass error | PPM error |
| --- | --- | --- | --- | --- | --- | --- | --- | --- | --- | --- |
| 551 | 1 | 8769.1669 | 798.2043 | 26730.57 | 11 | 8769.1965 | C74 | 74 | -0.0296 | -3.38 |
| 551 | 2 | 4443.9064 | 741.6583 | 21880.31 | 6 | 4443.9333 | C36 | 36 | -0.0269 | -6.06 |
| 551 | 3 | 2528.0752 | 633.0261 | 27791.41 | 4 | 2528.0889 | C20 | 20 | -0.0136 | -5.40 |
| 551 | 4 | 8770.1849 | 878.0258 | 22300.21 | 10 | 8769.1965 | C74 | 74 | -0.0140 | -1.60 |
| 551 | 5 | 8754.1692 | 876.4242 | 13525.27 | 10 |  |  |  |  |  |
| 551 | 6 | 2471.0545 | 618.7709 | 24106.75 | 4 | 2471.0674 | C19 | 19 | -0.0129 | -5.24 |
| 551 | 7 | 8713.1586 | 969.1360 | 12194.03 | 9 |  |  |  |  |  |
| 551 | 8 | 8769.1719 | 731.7716 | 16846.90 | 12 | 8769.1965 | C74 | 74 | -0.0246 | -2.81 |
| 551 | 9 | 1372.5791 | 687.2968 | 18767.69 | 2 | 1372.5863 | C11 | 11 | -7.18e-03 | -5.23 |
| 551 | 10 | 3445.5844 | 690.1242 | 13477.18 | 5 | 3445.6046 | C27 | 27 | -0.0202 | -5.85 |
| 551 | 11 | 4383.3009 | 731.5574 | 14135.73 | 6 |  |  |  |  |  |
| 551 | 12 | 3157.5019 | 632.5077 | 14022.82 | 5 | 3157.5153 | C25 | 25 | -0.0134 | -4.25 |
| 551 | 13 | 8784.1926 | 799.5702 | 11805.37 | 11 |  |  |  |  |  |
| 551 | 14 | 2528.0745 | 843.6988 | 15849.00 | 3 | 2528.0889 | C20 | 20 | -0.0144 | -5.68 |
| 551 | 15 | 2916.3206 | 730.0874 | 12344.38 | 4 | 2916.3363 | C23 | 23 | -0.0157 | -5.39 |
| 551 | 16 | 8725.1663 | 794.2042 | 9898.00 | 11 | 8724.1514 | Z\_DOT74 | 1 | 0.0126 | 1.44 |
| 551 | 17 | 4443.9069 | 889.7887 | 12527.04 | 5 | 4443.9333 | C36 | 36 | -0.0264 | -5.94 |
| 551 | 18 | 1752.7578 | 877.3862 | 17133.72 | 2 | 1752.7671 | C14 | 14 | -9.28e-03 | -5.30 |
| 551 | 19 | 3323.8846 | 665.7842 | 13503.98 | 5 |  |  |  |  |  |
| 551 | 20 | 4554.9361 | 760.1633 | 12386.95 | 6 |  |  |  |  |  |
| 551 | 21 | 8712.1301 | 872.2203 | 13856.32 | 10 |  |  |  |  |  |
| 551 | 22 | 2203.3680 | 551.8493 | 10896.66 | 4 |  |  |  |  |  |
| 551 | 23 | 3157.4974 | 790.3816 | 9963.08 | 4 | 3157.5153 | C25 | 25 | -0.0180 | -5.69 |
| 551 | 24 | 8726.1653 | 873.6238 | 10071.36 | 10 |  |  |  |  |  |
| 551 | 25 | 4770.0630 | 796.0178 | 10441.68 | 6 | 4770.0923 | C39 | 39 | -0.0294 | -6.16 |
| 551 | 26 | 1866.8000 | 934.4073 | 10905.40 | 2 | 1866.8101 | C15 | 15 | -0.0100 | -5.38 |
| 551 | 27 | 1615.6996 | 808.8571 | 13023.84 | 2 | 1615.7082 | C13 | 13 | -8.60e-03 | -5.32 |
| 551 | 28 | 2641.1590 | 661.2970 | 12452.64 | 4 | 2641.1730 | C21 | 21 | -0.0140 | -5.29 |
| 551 | 29 | 8810.1866 | 801.9333 | 11100.57 | 11 |  |  |  |  |  |
| 551 | 30 | 4271.2577 | 611.1870 | 8479.76 | 7 |  |  |  |  |  |
| 551 | 31 | 8769.1989 | 975.3627 | 9739.38 | 9 | 8769.1965 | C74 | 74 | 2.33e-03 | 0.27 |
| 551 | 32 | 4055.7876 | 812.1648 | 8099.51 | 5 | 4055.8103 | C32 | 32 | -0.0227 | -5.60 |
| 551 | 33 | 6097.5886 | 763.2058 | 8539.78 | 8 |  |  |  |  |  |
| 551 | 34 | 8712.1521 | 793.0211 | 14838.23 | 11 |  |  |  |  |  |
| 551 | 35 | 8753.1651 | 796.7496 | 11659.07 | 11 |  |  |  |  |  |
| 551 | 36 | 8784.1845 | 879.4257 | 8766.64 | 10 |  |  |  |  |  |
| 551 | 37 | 4325.2676 | 618.9027 | 8312.81 | 7 |  |  |  |  |  |
| 551 | 38 | 8726.1678 | 970.5815 | 8882.44 | 9 |  |  |  |  |  |
| 551 | 39 | 2471.0540 | 824.6919 | 8526.68 | 3 | 2471.0674 | C19 | 19 | -0.0134 | -5.43 |
| 551 | 40 | 8697.1150 | 791.6541 | 6888.26 | 11 |  |  |  |  |  |
| 551 | 41 | 4543.3654 | 650.0595 | 6444.07 | 7 |  |  |  |  |  |
| 551 | 42 | 6300.1428 | 901.0277 | 7727.29 | 7 | 6300.1210 | Z\_DOT55 | 20 | 0.0218 | 3.46 |
| 551 | 43 | 4387.8887 | 878.5850 | 9194.90 | 5 |  |  |  |  |  |
| 551 | 44 | 8697.1279 | 967.3548 | 8777.14 | 9 |  |  |  |  |  |
| 551 | 45 | 4299.8540 | 860.9781 | 6252.60 | 5 | 4299.8798 | C34 | 34 | -0.0258 | -6.00 |
| 551 | 46 | 6040.5686 | 863.9456 | 6152.10 | 7 | 6040.5738 | C50 | 50 | -5.16e-03 | -0.85 |
| 551 | 47 | 3029.4065 | 758.3589 | 7976.82 | 4 | 3029.4204 | C24 | 24 | -0.0139 | -4.57 |
| 551 | 48 | 4299.8521 | 717.6493 | 7114.95 | 6 | 4299.8798 | C34 | 34 | -0.0278 | -6.46 |
| 551 | 49 | 8754.1875 | 973.6948 | 9146.18 | 9 |  |  |  |  |  |
| 551 | 50 | 4414.1016 | 736.6909 | 9898.43 | 6 |  |  |  |  |  |
| 551 | 51 | 1169.7786 | 585.8966 | 8989.05 | 2 |  |  |  |  |  |
| 551 | 52 | 4282.8299 | 714.8123 | 6707.68 | 6 |  |  |  |  |  |
| 551 | 53 | 8783.1974 | 732.9404 | 7675.73 | 12 |  |  |  |  |  |
| 551 | 54 | 8811.1756 | 735.2719 | 6067.36 | 12 |  |  |  |  |  |
| 551 | 55 | 2358.4258 | 590.6137 | 7542.22 | 4 |  |  |  |  |  |
| 551 | 56 | 2288.3962 | 763.8060 | 5764.48 | 3 |  |  |  |  |  |
| 551 | 57 | 2617.5757 | 655.4012 | 7487.00 | 4 |  |  |  |  |  |
| 551 | 58 | 2601.5577 | 651.3967 | 5477.55 | 4 |  |  |  |  |  |
| 551 | 59 | 3681.9909 | 737.4055 | 3930.64 | 5 |  |  |  |  |  |
| 551 | 60 | 2342.9602 | 781.9940 | 6192.60 | 3 | 2342.9725 | C18 | 18 | -0.0123 | -5.23 |
| 551 | 61 | 2788.2269 | 698.0640 | 10009.86 | 4 | 2788.2414 | C22 | 22 | -0.0145 | -5.21 |
| 551 | 62 | 2087.2732 | 522.8256 | 4280.37 | 4 |  |  |  |  |  |
| 551 | 63 | 7340.2537 | 816.5910 | 6169.82 | 9 | 7340.2673 | C62 | 62 | -0.0136 | -1.85 |
| 551 | 64 | 8752.1603 | 730.3540 | 5184.83 | 12 |  |  |  |  |  |
| 551 | 65 | 2026.8289 | 1014.4217 | 4974.03 | 2 | 2026.8407 | C16 | 16 | -0.0118 | -5.80 |
| 551 | 66 | 3324.8925 | 832.2304 | 6567.95 | 4 |  |  |  |  |  |
| 551 | 67 | 4171.8093 | 835.3691 | 4665.78 | 5 |  |  |  |  |  |
| 551 | 68 | 997.4593 | 998.4666 | 6378.55 | 1 | 997.4651 | C8 | 8 | -5.78e-03 | -5.79 |
| 551 | 69 | 6040.5784 | 1007.7703 | 4339.65 | 6 | 6040.5738 | C50 | 50 | 4.62e-03 | 0.76 |
| 551 | 70 | 8672.1455 | 964.5790 | 3316.73 | 9 |  |  |  |  |  |
| 551 | 71 | 2671.5860 | 668.9038 | 4543.63 | 4 |  |  |  |  |  |
| 551 | 72 | 2788.2258 | 930.4159 | 5474.65 | 3 | 2788.2414 | C22 | 22 | -0.0155 | -5.57 |
| 551 | 73 | 4527.3501 | 755.5656 | 5069.23 | 6 |  |  |  |  |  |
| 551 | 74 | 2288.3956 | 573.1062 | 4286.73 | 4 |  |  |  |  |  |
| 551 | 75 | 2546.5391 | 637.6421 | 5451.80 | 4 |  |  |  |  |  |
| 551 | 76 | 8772.1921 | 1097.5313 | 3360.37 | 8 |  |  |  |  |  |
| 551 | 77 | 6300.1216 | 788.5225 | 6688.22 | 8 | 6300.1210 | Z\_DOT55 | 20 | 5.62e-04 | 0.09 |
| 551 | 78 | 2018.2511 | 673.7576 | 4937.41 | 3 |  |  |  |  |  |
| 551 | 79 | 3998.1253 | 572.1680 | 4862.60 | 7 |  |  |  |  |  |
| 551 | 80 | 2730.6223 | 683.6628 | 6516.61 | 4 |  |  |  |  |  |
| 551 | 81 | 1486.9500 | 744.4823 | 6776.77 | 2 |  |  |  |  |  |
| 551 | 82 | 5048.5397 | 722.2272 | 4234.33 | 7 |  |  |  |  |  |
| 551 | 83 | 5447.3029 | 908.8911 | 6445.08 | 6 | 5446.3345 | C44 | 44 | -0.0339 | -6.23 |
| 551 | 84 | 3998.1294 | 667.3622 | 3998.12 | 6 |  |  |  |  |  |
| 551 | 85 | 6357.1553 | 795.6517 | 3435.24 | 8 | 6357.1425 | Z\_DOT56 | 19 | 0.0129 | 2.03 |
| 551 | 86 | 3741.6467 | 624.6151 | 3140.58 | 6 |  |  |  |  |  |
| 551 | 87 | 3940.7583 | 789.1589 | 4783.86 | 5 | 3940.7834 | C31 | 31 | -0.0251 | -6.36 |
| 551 | 88 | 4325.2704 | 721.8857 | 5596.33 | 6 |  |  |  |  |  |
| 551 | 89 | 868.4175 | 869.4247 | 6410.35 | 1 | 868.4225 | C7 | 7 | -5.00e-03 | -5.75 |
| 551 | 90 | 1874.1634 | 469.5481 | 3858.65 | 4 |  |  |  |  |  |
| 551 | 91 | 8682.1595 | 869.2232 | 4965.78 | 10 |  |  |  |  |  |
| 551 | 92 | 4348.5727 | 870.7218 | 4906.47 | 5 |  |  |  |  |  |
| 551 | 93 | 600.3807 | 601.3880 | 7518.34 | 1 |  |  |  |  |  |
| 551 | 94 | 7913.6039 | 880.2966 | 5949.62 | 9 | 7913.6311 | C67 | 67 | -0.0272 | -3.44 |
| 551 | 95 | 6098.5843 | 872.2336 | 5680.85 | 7 |  |  |  |  |  |
| 551 | 96 | 2432.4545 | 609.1209 | 4775.42 | 4 |  |  |  |  |  |
| 551 | 97 | 8641.0939 | 961.1288 | 6242.04 | 9 | 8641.1016 | C73 | 73 | -7.66e-03 | -0.89 |
| 551 | 98 | 1386.8754 | 463.2991 | 7296.19 | 3 |  |  |  |  |  |
| 551 | 99 | 8641.0809 | 865.1154 | 7686.92 | 10 | 8641.1016 | C73 | 73 | -0.0207 | -2.39 |
| 551 | 100 | 8668.1475 | 867.8220 | 5150.06 | 10 |  |  |  |  |  |
| 551 | 101 | 4058.1404 | 677.3640 | 6004.99 | 6 | 4058.1176 | Z\_DOT36 | 39 | 0.0229 | 5.63 |
| 551 | 102 | 8228.8253 | 823.8898 | 4228.45 | 10 |  |  |  |  |  |
| 551 | 103 | 4271.2580 | 712.8836 | 5612.01 | 6 |  |  |  |  |  |
| 551 | 104 | 6258.1095 | 895.0229 | 3252.82 | 7 |  |  |  |  |  |
| 551 | 105 | 4039.7596 | 674.3005 | 3679.35 | 6 |  |  |  |  |  |
| 551 | 106 | 5504.3312 | 787.3403 | 4111.64 | 7 | 5503.3559 | C45 | 45 | -0.0270 | -4.91 |
| 551 | 107 | 5579.7126 | 930.9594 | 4927.26 | 6 |  |  |  |  |  |
| 551 | 108 | 4368.8425 | 729.1477 | 9718.03 | 6 |  |  |  |  |  |
| 551 | 109 | 8652.1049 | 866.2178 | 3643.60 | 10 |  |  |  |  |  |
| 551 | 110 | 4426.8999 | 738.8239 | 3209.02 | 6 |  |  |  |  |  |
| 551 | 111 | 7342.2622 | 918.7901 | 5593.67 | 8 |  |  |  |  |  |
| 551 | 112 | 8738.1401 | 874.8213 | 4313.07 | 10 |  |  |  |  |  |
| 551 | 113 | 5049.5266 | 632.1981 | 4592.75 | 8 |  |  |  |  |  |
| 551 | 114 | 4406.5949 | 882.3263 | 4136.24 | 5 |  |  |  |  |  |
| 551 | 115 | 6186.0504 | 774.2636 | 5481.48 | 8 | 6187.0369 | Z\_DOT54 | 21 | 0.0158 | 2.55 |
| 551 | 116 | 4771.0730 | 955.2219 | 5948.47 | 5 |  |  |  |  |  |
| 551 | 117 | 3777.6635 | 630.6179 | 4977.79 | 6 |  |  |  |  |  |
| 551 | 118 | 6639.8574 | 830.9895 | 2700.13 | 8 | 6639.8652 | C56 | 56 | -7.83e-03 | -1.18 |
| 551 | 119 | 5503.3115 | 918.2259 | 3986.27 | 6 | 5503.3559 | C45 | 45 | -0.0444 | -8.08 |
| 551 | 120 | 4153.7925 | 693.3060 | 3565.19 | 6 |  |  |  |  |  |
| 551 | 121 | 1486.9510 | 496.6576 | 4106.56 | 3 |  |  |  |  |  |
| 551 | 122 | 1258.5377 | 1259.5450 | 3896.88 | 1 | 1258.5434 | C10 | 10 | -5.68e-03 | -4.52 |
| 551 | 123 | 6539.8119 | 935.2661 | 4201.72 | 7 |  |  |  |  |  |
| 551 | 124 | 7456.6306 | 829.5218 | 2954.59 | 9 | 7455.6236 | Z\_DOT64 | 11 | 4.65e-03 | 0.62 |
| 551 | 125 | 1643.0509 | 548.6909 | 3773.50 | 3 |  |  |  |  |  |
| 551 | 126 | 6241.1149 | 892.5951 | 3420.61 | 7 |  |  |  |  |  |
| 551 | 127 | 6257.0668 | 696.2369 | 2689.14 | 9 |  |  |  |  |  |
| 551 | 128 | 6539.8057 | 818.4830 | 3975.61 | 8 |  |  |  |  |  |
| 551 | 129 | 1723.2967 | 862.6556 | 2675.05 | 2 |  |  |  |  |  |
| 551 | 130 | 5447.3120 | 1090.4697 | 3738.27 | 5 | 5446.3345 | C44 | 44 | -0.0248 | -4.56 |
| 551 | 131 | 6523.7982 | 932.9784 | 2217.83 | 7 |  |  |  |  |  |
| 551 | 132 | 1428.8857 | 477.3025 | 3545.01 | 3 |  |  |  |  |  |
| 551 | 133 | 798.5040 | 400.2593 | 4299.25 | 2 |  |  |  |  |  |
| 551 | 134 | 486.3384 | 487.3456 | 4579.85 | 1 |  |  |  |  |  |
| 551 | 135 | 1041.6845 | 521.8495 | 3591.23 | 2 |  |  |  |  |  |
| 551 | 136 | 1430.9347 | 716.4746 | 3055.85 | 2 |  |  |  |  |  |
| 551 | 137 | 1316.8457 | 439.9558 | 2479.57 | 3 |  |  |  |  |  |
| 551 | 138 | 1258.5364 | 630.2755 | 5512.46 | 2 | 1258.5434 | C10 | 10 | -6.98e-03 | -5.54 |
| 551 | 139 | 997.4603 | 499.7374 | 3335.01 | 2 | 997.4651 | C8 | 8 | -4.75e-03 | -4.77 |
| 551 | 140 | 1057.7030 | 529.8588 | 2600.63 | 2 |  |  |  |  |  |
| 551 | 141 | 984.6629 | 493.3387 | 1586.64 | 2 |  |  |  |  |  |
| 551 | 142 | 502.2428 | 503.2501 | 2305.59 | 1 |  |  |  |  |  |
| 551 | 143 | 1169.7783 | 390.9334 | 1776.79 | 3 |  |  |  |  |  |
| 551 | 144 | 1185.7984 | 593.9065 | 1969.33 | 2 |  |  |  |  |  |
| 551 | 145 | 1258.7814 | 420.6011 | 1707.48 | 3 |  |  |  |  |  |
| 551 | 146 | 868.4188 | 435.2167 | 1373.75 | 2 | 868.4225 | C7 | 7 | -3.67e-03 | -4.22 |
| 551 | 147 | 1415.8778 | 472.9666 | 1858.55 | 3 |  |  |  |  |  |

  

All proteins /
CsTx-1a\_S1 Cupiennius salei toxin 1 isoform a S1^ACsTx-1a\_S2 Cupiennius salei toxin 1 isoform a S2 /
Proteoform #15
