# Supplementary material for: The Dual Prey-Inactivation Strategy of Spiders—In-Depth Venomic Analysis of Cupiennius salei
Source: Toxins (Basel). 2019 Mar 19;11(3):167. doi: 10.3390/toxins11030167 (PMC6468893; doi:10.3390/toxins11030167)
Supplement: Supplementary file 1 [file toxins-11-00167-s001.zip › Supplementary Dataset EV1/20180328_f2_topdown_OTMS2_EThcD_NL_i02_ms2_proteoform_cutoff_html/prsms/prsm173.html]

Protein-Spectrum-Match for Spectrum #412


All proteins /
CsTx-1a\_S1 Cupiennius salei toxin 1 isoform a S1^ACsTx-1a\_S2 Cupiennius salei toxin 1 isoform a S2 /
Proteoform #15

## Protein-Spectrum-Match #173 for Spectrum #412

|  |  |  |  |  |  |
| --- | --- | --- | --- | --- | --- |
| PrSM ID: | 173 | Scan(s): | 552 | Precursor charge: | 13 |
| Precursor m/z: | 679.9492 | Precursor mass: | 8826.2448 | Proteoform mass: | 8826.2248 |
| # matched peaks: | 50 | # matched fragment ions: | 35 | # unexpected modifications: | 1 |
| E-value: | 1.48e-28 | P-value: | 1.48e-28 | Q-value (Spectral FDR): | 0 |

  

|  |  |  |  |  |  |  |  |  |  |  |  |  |  |  |  |  |  |  |  |  |  |  |  |  |  |  |  |  |  |  |  |  |  |  |  |  |  |  |  |  |  |  |  |  |  |  |  |  |  |  |  |  |  |  |  |  |  |  |  |  |  |  |  |  |  |  |  |  |  |
| --- | --- | --- | --- | --- | --- | --- | --- | --- | --- | --- | --- | --- | --- | --- | --- | --- | --- | --- | --- | --- | --- | --- | --- | --- | --- | --- | --- | --- | --- | --- | --- | --- | --- | --- | --- | --- | --- | --- | --- | --- | --- | --- | --- | --- | --- | --- | --- | --- | --- | --- | --- | --- | --- | --- | --- | --- | --- | --- | --- | --- | --- | --- | --- | --- | --- | --- | --- | --- | --- |
|  | |  | | | | | | | | | | | | | | | | | | | | | | | | | | | | | | | | | | | | | | | | | | | | | | | | | | | | | | | | | | | | | | | | | | | |
| 1 |  |  | M |  | K |  | V |  | L |  | I |  | I |  | S |  | A |  | V |  | L |  |  | F |  | I |  | T |  | I |  | F |  | S |  | N |  | I |  | S |  | A |  |  | E |  | I |  | E |  | D |  | D |  | F |  | L |  | E |  | D |  | E |  | 30 |  |
|  | |  | | | | | | | | | | | | | | | | | | | | | | | | | | | | | | | | | | | | | | | | | | | | | | | | | | | | | | | | | | | | | | | | | | | |
| 31 |  |  | S |  | F |  | E |  | A |  | E |  | D |  | I |  | I |  | P |  | F |  |  | F |  | E |  | N |  | E |  | Q |  | A |  | R | ] | S | ⎩ | C |  | I |  |  | P |  | K |  | H |  | E |  | E | ⎫ | C | ⎫ | T | ⎫ | N | ⎫ | D |  | K |  | 60 |  |
|  | |  | | | | | | | | | | | | | | | | | | | | | | | | | | | | | | | | | | | | | | | | | | | | | | | | | | | | | | | | | | | | | | | | | | | |
| 61 |  | ⎫ | H | ⎱ | N | ⎫ | C |  | C |  | R | ⎫ | K | ⎫ | G | ⎱ | L | ⎱ | F | ⎱ | K |  | ⎫ | L | ⎫ | K | ⎫ | C |  | Q | ⎫ | C |  | S |  | T |  | F | ⎫ | D | ⎫ | D |  | ⎫ | E | ⎱ | S | ⎫ | G | ⎫ | Q |  | P |  | T | ⎫ | E |  | R |  | C |  | A |  | 90 |  |
|  | |  | | | | | 14.99 | | | | | | | | | | | | | | | | | | | | | | | | | | | | | | | | | | | | | | | | | | | | | | | | | | | | | | | | | | | |
| 91 |  |  | C |  | G | ⎱ | R |  | P |  | M |  | G |  | H |  | Q |  | A |  | I |  |  | E |  | T |  | G |  | L |  | N |  | I |  | F |  | R |  | G |  | L |  |  | F |  | K |  | G |  | K | ⎫ | K | ⎫ | K |  | N |  | K |  | K | ⎫ | T |  | 120 |  |
|  | |  | | | | | | | | | | | | | | | | | | | | | | | | | | | | | | | | | | | | | | | | | | | | | | | | | | | | | | | | | | | | | | | | | | | |
| 121 |  | ⎫ | K | [ | G |  | | | | 122 |  | | | | | | | | | | | | | | | | | | | | | | | | | | | | | | | | | | | | | | | | | | | | | | | | | | | | | | | |

Fixed PTMs: Carbamidomethylation [C49 C56 C63 C64 C73 C75 C89 C91 ]   
  
     Unexpected modifications:   Unknown [14.99]

  

All peaks (147)  Matched peaks (50)  Not matched peaks (97)

  

| Scan | Peak | Mono mass | Mono m/z | Intensity | Charge | Theoretical mass | Ion | Pos | Mass error | PPM error |
| --- | --- | --- | --- | --- | --- | --- | --- | --- | --- | --- |
| 552 | 1 | 8770.1812 | 878.0254 | 68691.54 | 10 |  |  |  |  |  |
| 552 | 2 | 8769.1810 | 798.2056 | 54653.64 | 11 |  |  |  |  |  |
| 552 | 3 | 8770.1843 | 975.4722 | 37706.24 | 9 |  |  |  |  |  |
| 552 | 4 | 8783.1864 | 879.3259 | 27913.22 | 10 |  |  |  |  |  |
| 552 | 5 | 8712.1573 | 969.0248 | 24245.09 | 9 |  |  |  |  |  |
| 552 | 6 | 8811.1810 | 882.1254 | 26031.98 | 10 |  |  |  |  |  |
| 552 | 7 | 4443.9081 | 889.7889 | 25958.71 | 5 | 4443.9333 | C36 | 36 | -0.0252 | -5.67 |
| 552 | 8 | 8753.1663 | 876.3239 | 22647.37 | 10 |  |  |  |  |  |
| 552 | 9 | 8754.1696 | 973.6928 | 23087.82 | 9 |  |  |  |  |  |
| 552 | 10 | 8711.1473 | 872.1220 | 25258.66 | 10 |  |  |  |  |  |
| 552 | 11 | 8810.1826 | 801.9330 | 19854.07 | 11 |  |  |  |  |  |
| 552 | 12 | 8784.1930 | 977.0287 | 14558.22 | 9 |  |  |  |  |  |
| 552 | 13 | 8726.1760 | 970.5824 | 18547.95 | 9 |  |  |  |  |  |
| 552 | 14 | 8769.1770 | 731.7720 | 19150.53 | 12 |  |  |  |  |  |
| 552 | 15 | 8783.1768 | 799.4779 | 19876.61 | 11 |  |  |  |  |  |
| 552 | 16 | 8725.1654 | 873.5238 | 23950.87 | 10 |  |  |  |  |  |
| 552 | 17 | 4443.9082 | 741.6586 | 18264.63 | 6 | 4443.9333 | C36 | 36 | -0.0251 | -5.65 |
| 552 | 18 | 5503.3265 | 918.2284 | 13406.83 | 6 | 5503.3559 | C45 | 45 | -0.0294 | -5.34 |
| 552 | 19 | 2528.0752 | 633.0261 | 18856.39 | 4 | 2528.0889 | C20 | 20 | -0.0136 | -5.40 |
| 552 | 20 | 1752.7585 | 877.3865 | 23357.29 | 2 | 1752.7671 | C14 | 14 | -8.67e-03 | -4.95 |
| 552 | 21 | 8698.1097 | 870.8182 | 14951.72 | 10 | 8697.1457 | C73 | 73 | -0.0384 | -4.41 |
| 552 | 22 | 4414.1033 | 883.8279 | 25659.59 | 5 |  |  |  |  |  |
| 552 | 23 | 4413.1011 | 736.5241 | 21470.66 | 6 |  |  |  |  |  |
| 552 | 24 | 4384.3032 | 877.8679 | 16282.24 | 5 |  |  |  |  |  |
| 552 | 25 | 2471.0542 | 618.7708 | 17692.59 | 4 | 2471.0674 | C19 | 19 | -0.0132 | -5.34 |
| 552 | 26 | 8711.1546 | 1089.9016 | 13896.47 | 8 |  |  |  |  |  |
| 552 | 27 | 3323.8840 | 665.7841 | 13240.25 | 5 | 3323.8767 | Z\_DOT29 | 45 | 7.31e-03 | 2.20 |
| 552 | 28 | 8711.1585 | 792.9308 | 12513.20 | 11 |  |  |  |  |  |
| 552 | 29 | 8812.1917 | 980.1397 | 13347.08 | 9 |  |  |  |  |  |
| 552 | 30 | 2033.2633 | 678.7617 | 27668.00 | 3 |  |  |  |  |  |
| 552 | 31 | 3445.5835 | 690.1240 | 11958.90 | 5 | 3445.6046 | C27 | 27 | -0.0211 | -6.12 |
| 552 | 32 | 8753.1678 | 796.7498 | 12982.20 | 11 |  |  |  |  |  |
| 552 | 33 | 1372.5794 | 687.2970 | 16829.78 | 2 | 1372.5863 | C11 | 11 | -6.94e-03 | -5.05 |
| 552 | 34 | 2528.0732 | 843.6984 | 16258.38 | 3 | 2528.0889 | C20 | 20 | -0.0157 | -6.19 |
| 552 | 35 | 3157.5004 | 632.5074 | 11253.45 | 5 | 3157.5153 | C25 | 25 | -0.0149 | -4.73 |
| 552 | 36 | 4384.3014 | 731.7242 | 13823.94 | 6 |  |  |  |  |  |
| 552 | 37 | 4325.2708 | 721.8857 | 13072.76 | 6 |  |  |  |  |  |
| 552 | 38 | 3157.4998 | 790.3822 | 16460.02 | 4 | 3157.5153 | C25 | 25 | -0.0155 | -4.92 |
| 552 | 39 | 4055.7900 | 812.1653 | 11226.48 | 5 | 4055.8103 | C32 | 32 | -0.0203 | -5.00 |
| 552 | 40 | 1866.8010 | 934.4078 | 16859.78 | 2 | 1866.8101 | C15 | 15 | -9.06e-03 | -4.86 |
| 552 | 41 | 2916.3218 | 730.0877 | 11262.41 | 4 | 2916.3363 | C23 | 23 | -0.0145 | -4.97 |
| 552 | 42 | 8770.1829 | 1097.2801 | 10324.10 | 8 |  |  |  |  |  |
| 552 | 43 | 2788.2295 | 930.4171 | 11785.12 | 3 | 2788.2414 | C22 | 22 | -0.0119 | -4.26 |
| 552 | 44 | 5579.7053 | 930.9582 | 10737.61 | 6 |  |  |  |  |  |
| 552 | 45 | 1615.7002 | 808.8574 | 11488.79 | 2 | 1615.7082 | C13 | 13 | -7.99e-03 | -4.94 |
| 552 | 46 | 8698.1208 | 791.7455 | 9099.70 | 11 | 8697.1457 | C73 | 73 | -0.0273 | -3.14 |
| 552 | 47 | 4770.0732 | 955.0219 | 11455.16 | 5 | 4770.0923 | C39 | 39 | -0.0191 | -4.00 |
| 552 | 48 | 2617.5777 | 655.4017 | 11083.52 | 4 |  |  |  |  |  |
| 552 | 49 | 3029.4038 | 758.3582 | 8943.66 | 4 | 3029.4204 | C24 | 24 | -0.0165 | -5.46 |
| 552 | 50 | 2788.2273 | 698.0641 | 9639.82 | 4 | 2788.2414 | C22 | 22 | -0.0140 | -5.03 |
| 552 | 51 | 2641.1595 | 661.2971 | 10759.94 | 4 | 2641.1730 | C21 | 21 | -0.0135 | -5.11 |
| 552 | 52 | 4299.8552 | 860.9783 | 10539.59 | 5 | 4299.8798 | C34 | 34 | -0.0246 | -5.72 |
| 552 | 53 | 3445.5866 | 862.4039 | 8918.79 | 4 | 3445.6046 | C27 | 27 | -0.0180 | -5.23 |
| 552 | 54 | 3323.8849 | 831.9785 | 13343.48 | 4 | 3323.8767 | Z\_DOT29 | 45 | 8.24e-03 | 2.48 |
| 552 | 55 | 8697.1478 | 967.3570 | 17094.91 | 9 | 8697.1457 | C73 | 73 | 2.07e-03 | 0.24 |
| 552 | 56 | 7970.6338 | 886.6333 | 10325.71 | 9 | 7969.6753 | C67 | 67 | -0.0439 | -5.50 |
| 552 | 57 | 2288.3990 | 763.8069 | 9977.97 | 3 |  |  |  |  |  |
| 552 | 58 | 4900.1029 | 981.0279 | 6358.48 | 5 |  |  |  |  |  |
| 552 | 59 | 2203.3677 | 735.4632 | 8898.88 | 3 |  |  |  |  |  |
| 552 | 60 | 8754.1728 | 1095.2789 | 12419.25 | 8 |  |  |  |  |  |
| 552 | 61 | 3394.8657 | 679.9804 | 6359.61 | 5 |  |  |  |  |  |
| 552 | 62 | 6257.0865 | 783.1431 | 7787.54 | 8 |  |  |  |  |  |
| 552 | 63 | 4170.8144 | 835.1702 | 8055.83 | 5 | 4170.8372 | C33 | 33 | -0.0228 | -5.47 |
| 552 | 64 | 6300.1050 | 788.5204 | 9171.02 | 8 | 6299.1437 | Z\_DOT54 | 20 | -0.0410 | -6.51 |
| 552 | 65 | 1169.7791 | 585.8968 | 12097.35 | 2 |  |  |  |  |  |
| 552 | 66 | 6300.1253 | 901.0252 | 11006.81 | 7 | 6299.1437 | Z\_DOT54 | 20 | -0.0208 | -3.29 |
| 552 | 67 | 4554.9361 | 760.1633 | 11119.57 | 6 |  |  |  |  |  |
| 552 | 68 | 2671.5875 | 668.9041 | 8261.46 | 4 |  |  |  |  |  |
| 552 | 69 | 8227.8416 | 915.2119 | 8238.14 | 9 |  |  |  |  |  |
| 552 | 70 | 8341.8742 | 927.8822 | 7419.09 | 9 |  |  |  |  |  |
| 552 | 71 | 3998.1283 | 667.3620 | 8450.24 | 6 |  |  |  |  |  |
| 552 | 72 | 2471.0531 | 824.6916 | 9380.61 | 3 | 2471.0674 | C19 | 19 | -0.0143 | -5.80 |
| 552 | 73 | 8641.0978 | 961.1292 | 12828.56 | 9 |  |  |  |  |  |
| 552 | 74 | 8680.1404 | 965.4673 | 7922.98 | 9 |  |  |  |  |  |
| 552 | 75 | 8726.1752 | 1091.7792 | 8535.56 | 8 |  |  |  |  |  |
| 552 | 76 | 6039.9824 | 863.8619 | 7733.47 | 7 | 6038.9912 | Z\_DOT52 | 22 | -0.0112 | -1.85 |
| 552 | 77 | 3940.7556 | 789.1584 | 8393.30 | 5 | 3940.7834 | C31 | 31 | -0.0278 | -7.05 |
| 552 | 78 | 6186.0396 | 774.2622 | 6603.66 | 8 | 6186.0596 | Z\_DOT53 | 21 | -0.0200 | -3.23 |
| 552 | 79 | 8738.1579 | 874.8231 | 7754.10 | 10 |  |  |  |  |  |
| 552 | 80 | 2943.0681 | 736.7743 | 9257.63 | 4 |  |  |  |  |  |
| 552 | 81 | 8808.1807 | 735.0223 | 8311.03 | 12 |  |  |  |  |  |
| 552 | 82 | 8724.1773 | 794.1143 | 8756.63 | 11 | 8723.1741 | Z\_DOT73 | 1 | 8.97e-04 | 0.10 |
| 552 | 83 | 1486.9505 | 744.4825 | 8318.00 | 2 |  |  |  |  |  |
| 552 | 84 | 2721.3697 | 681.3497 | 13692.80 | 4 |  |  |  |  |  |
| 552 | 85 | 4058.1455 | 677.3649 | 6858.63 | 6 |  |  |  |  |  |
| 552 | 86 | 8655.1107 | 962.6862 | 9397.32 | 9 |  |  |  |  |  |
| 552 | 87 | 2342.9611 | 781.9943 | 6118.93 | 3 | 2342.9725 | C18 | 18 | -0.0113 | -4.84 |
| 552 | 88 | 8642.0892 | 1081.2684 | 5790.68 | 8 |  |  |  |  |  |
| 552 | 89 | 6809.9603 | 852.2523 | 5776.43 | 8 |  |  |  |  |  |
| 552 | 90 | 4456.3312 | 743.7291 | 4477.85 | 6 |  |  |  |  |  |
| 552 | 91 | 3941.7603 | 986.4474 | 5894.39 | 4 |  |  |  |  |  |
| 552 | 92 | 7075.4551 | 885.4392 | 6240.78 | 8 | 7074.4655 | Z\_DOT60 | 14 | -0.0128 | -1.80 |
| 552 | 93 | 8341.8679 | 835.1941 | 5762.26 | 10 |  |  |  |  |  |
| 552 | 94 | 2729.6180 | 910.8799 | 7980.99 | 3 |  |  |  |  |  |
| 552 | 95 | 2601.5582 | 651.3968 | 5840.99 | 4 |  |  |  |  |  |
| 552 | 96 | 7283.2332 | 911.4114 | 6005.53 | 8 |  |  |  |  |  |
| 552 | 97 | 7914.6245 | 990.3353 | 6755.16 | 8 |  |  |  |  |  |
| 552 | 98 | 997.4603 | 998.4676 | 9267.31 | 1 | 997.4651 | C8 | 8 | -4.74e-03 | -4.75 |
| 552 | 99 | 728.4751 | 729.4824 | 8065.27 | 1 |  |  |  |  |  |
| 552 | 100 | 856.5694 | 857.5767 | 8747.47 | 1 |  |  |  |  |  |
| 552 | 101 | 6539.8221 | 935.2676 | 7146.61 | 7 |  |  |  |  |  |
| 552 | 102 | 2017.2456 | 673.4225 | 7163.74 | 3 |  |  |  |  |  |
| 552 | 103 | 8597.0503 | 956.2351 | 6567.10 | 9 | 8596.0981 | C72 | 72 | -0.0501 | -5.83 |
| 552 | 104 | 4055.7901 | 1014.9548 | 5897.84 | 4 | 4055.8103 | C32 | 32 | -0.0202 | -4.98 |
| 552 | 105 | 2203.3682 | 551.8493 | 7551.49 | 4 |  |  |  |  |  |
| 552 | 106 | 3682.0004 | 737.4074 | 6411.78 | 5 |  |  |  |  |  |
| 552 | 107 | 6098.6049 | 1017.4414 | 6086.12 | 6 |  |  |  |  |  |
| 552 | 108 | 5925.8910 | 847.5631 | 6492.09 | 7 |  |  |  |  |  |
| 552 | 109 | 6040.5773 | 1007.7702 | 7562.44 | 6 |  |  |  |  |  |
| 552 | 110 | 7456.6197 | 933.0847 | 6645.34 | 8 |  |  |  |  |  |
| 552 | 111 | 4386.8902 | 1097.7298 | 4793.98 | 4 | 4386.9119 | C35 | 35 | -0.0217 | -4.94 |
| 552 | 112 | 6097.5884 | 872.0913 | 13729.11 | 7 |  |  |  |  |  |
| 552 | 113 | 4444.9127 | 1112.2354 | 6397.26 | 4 |  |  |  |  |  |
| 552 | 114 | 8098.7365 | 810.8809 | 7869.96 | 10 | 8097.7702 | C68 | 68 | -0.0361 | -4.46 |
| 552 | 115 | 6241.0974 | 892.5926 | 4743.63 | 7 |  |  |  |  |  |
| 552 | 116 | 5926.9091 | 988.8255 | 5381.47 | 6 |  |  |  |  |  |
| 552 | 117 | 8740.1546 | 1093.5266 | 4716.50 | 8 |  |  |  |  |  |
| 552 | 118 | 4770.0677 | 796.0186 | 5761.64 | 6 | 4770.0923 | C39 | 39 | -0.0246 | -5.16 |
| 552 | 119 | 8641.0919 | 865.1165 | 4522.99 | 10 |  |  |  |  |  |
| 552 | 120 | 8696.1019 | 1088.0200 | 8011.57 | 8 | 8697.1457 | C73 | 73 | -0.0415 | -4.77 |
| 552 | 121 | 6871.4074 | 764.4970 | 6279.49 | 9 |  |  |  |  |  |
| 552 | 122 | 4527.3596 | 755.5672 | 5731.12 | 6 | 4527.3528 | Z\_DOT40 | 34 | 6.86e-03 | 1.52 |
| 552 | 123 | 5525.7101 | 921.9590 | 3832.31 | 6 |  |  |  |  |  |
| 552 | 124 | 4657.4026 | 777.2410 | 4194.34 | 6 |  |  |  |  |  |
| 552 | 125 | 8042.7295 | 1006.3485 | 3066.48 | 8 |  |  |  |  |  |
| 552 | 126 | 2943.0702 | 982.0307 | 6793.00 | 3 |  |  |  |  |  |
| 552 | 127 | 600.3809 | 601.3882 | 6757.26 | 1 |  |  |  |  |  |
| 552 | 128 | 2087.2737 | 696.7652 | 3855.90 | 3 |  |  |  |  |  |
| 552 | 129 | 4528.3676 | 906.6808 | 5297.00 | 5 |  |  |  |  |  |
| 552 | 130 | 1428.8860 | 477.3026 | 4191.18 | 3 |  |  |  |  |  |
| 552 | 131 | 1486.9500 | 496.6573 | 2910.64 | 3 |  |  |  |  |  |
| 552 | 132 | 1258.5381 | 1259.5453 | 3985.10 | 1 | 1258.5434 | C10 | 10 | -5.32e-03 | -4.22 |
| 552 | 133 | 1386.8759 | 463.2993 | 3875.70 | 3 |  |  |  |  |  |
| 552 | 134 | 486.3385 | 487.3458 | 3761.14 | 1 |  |  |  |  |  |
| 552 | 135 | 1372.5797 | 1373.5870 | 2848.42 | 1 | 1372.5863 | C11 | 11 | -6.62e-03 | -4.82 |
| 552 | 136 | 1185.7972 | 593.9059 | 3568.49 | 2 |  |  |  |  |  |
| 552 | 137 | 803.0203 | 804.0276 | 12842.14 | 1 |  |  |  |  |  |
| 552 | 138 | 959.3423 | 960.3495 | 2408.51 | 1 |  |  |  |  |  |
| 552 | 139 | 798.5042 | 400.2594 | 3060.34 | 2 |  |  |  |  |  |
| 552 | 140 | 1157.4895 | 1158.4968 | 2019.41 | 1 | 1157.4957 | C9 | 9 | -6.22e-03 | -5.37 |
| 552 | 141 | 997.4602 | 499.7374 | 1781.81 | 2 | 997.4651 | C8 | 8 | -4.81e-03 | -4.83 |
| 552 | 142 | 971.6871 | 972.6944 | 2775.70 | 1 |  |  |  |  |  |
| 552 | 143 | 1041.6848 | 521.8497 | 1934.87 | 2 |  |  |  |  |  |
| 552 | 144 | 1057.7036 | 529.8591 | 1784.89 | 2 |  |  |  |  |  |
| 552 | 145 | 670.4102 | 336.2124 | 1514.96 | 2 |  |  |  |  |  |
| 552 | 146 | 894.1895 | 895.1968 | 1437.89 | 1 |  |  |  |  |  |
| 552 | 147 | 1053.5007 | 1054.5080 | 1043.32 | 1 |  |  |  |  |  |

  

All proteins /
CsTx-1a\_S1 Cupiennius salei toxin 1 isoform a S1^ACsTx-1a\_S2 Cupiennius salei toxin 1 isoform a S2 /
Proteoform #15
