# Supplementary material for: The Dual Prey-Inactivation Strategy of Spiders—In-Depth Venomic Analysis of Cupiennius salei
Source: Toxins (Basel). 2019 Mar 19;11(3):167. doi: 10.3390/toxins11030167 (PMC6468893; doi:10.3390/toxins11030167)
Supplement: Supplementary file 1 [file toxins-11-00167-s001.zip › Supplementary Dataset EV1/20180328_f2_topdown_OTMS2_EThcD_NL_i02_ms2_proteoform_cutoff_html/prsms/prsm174.html]

Protein-Spectrum-Match for Spectrum #413


All proteins /
CsTx-12a\_S1 Cupiennius salei toxin 12 isoform a S1^ACsTx-12a\_S2 Cupiennius salei toxin 12 isoform a S2 /
Proteoform #53

## Protein-Spectrum-Match #174 for Spectrum #413

|  |  |  |  |  |  |
| --- | --- | --- | --- | --- | --- |
| PrSM ID: | 174 | Scan(s): | 553 | Precursor charge: | 6 |
| Precursor m/z: | 569.3245 | Precursor mass: | 3409.9032 | Proteoform mass: | 3409.8982 |
| # matched peaks: | 32 | # matched fragment ions: | 27 | # unexpected modifications: | 1 |
| E-value: | 1.14e-20 | P-value: | 1.14e-20 | Q-value (Spectral FDR): | 0 |

  

|  |  |  |  |  |  |  |  |  |  |  |  |  |  |  |  |  |  |  |  |  |  |  |  |  |  |  |  |  |  |  |  |  |  |  |  |  |  |  |  |  |  |  |  |  |  |  |  |  |  |  |  |  |  |  |  |  |  |  |  |  |  |  |  |  |  |  |
| --- | --- | --- | --- | --- | --- | --- | --- | --- | --- | --- | --- | --- | --- | --- | --- | --- | --- | --- | --- | --- | --- | --- | --- | --- | --- | --- | --- | --- | --- | --- | --- | --- | --- | --- | --- | --- | --- | --- | --- | --- | --- | --- | --- | --- | --- | --- | --- | --- | --- | --- | --- | --- | --- | --- | --- | --- | --- | --- | --- | --- | --- | --- | --- | --- | --- | --- |
|  | | ... 30 amino acid residues are skipped at the N-terminus ... | | | | | | | | | | | | | | | | | | | | | | | | | | | | | | | | | | | | | | | | | | | | | | | | | | | | | | | | | | | | | |  | | |
|  | |  | | | | | | | | | | | | | | | | | | | | | | | | | | | | | | | | | | | | | | | | | | | | | | | | | | | | | | | | | | | | | | | | | | | |
| 31 |  |  | S |  | F |  | E |  | A |  | D |  | D |  | V |  | I |  | P |  | F |  |  | L |  | A |  | R |  | E |  | Q |  | V |  | R |  | S |  | D |  | C |  |  | T |  | L |  | R |  | N |  | H |  | D |  | C |  | T |  | D |  | D |  | 60 |  |
|  | |  | | | | | | | | | | | | | | | | | | | | | | | | | | | | | | | | | | | | | | | | | | | | | | | | | | | | | | | | | | | | | | | | | | | |
| 61 |  |  | R |  | H |  | S |  | C |  | C |  | R |  | S |  | K |  | M |  | F |  |  | K |  | D |  | V |  | C |  | K |  | C |  | F |  | Y |  | P |  | S |  |  | Q |  | R |  | S |  | D |  | T |  | A |  | R | ] | A | ⎩ | K | ⎩ | K |  | 90 |  |
|  | |  | | | | | | | | | | | | | | | | | | | | | | | | | | | | | | | | | | | | | | | | | | | | | | | | | | | | | -58.01 | | | | | | | | | | | |
| 91 |  | ⎫ | E | ⎫ | L |  | C | ⎫ | T |  | C | ⎫ | Q | ⎫ | Q | ⎫ | D |  | K |  | H |  |  | L | ⎫ | K | ⎱ | F | ⎱ | I | ⎱ | E | ⎫ | K |  | G | ⎫ | L |  | Q | ⎱ | K |  | ⎱ | A | ⎱ | K | ⎫ | V | ⎫ | L | ⎫ | V | ⎫ | A |  | G |  | | 117 |  | | | | | |

Fixed PTMs: Carbamidomethylation [C93 C95 ]   
  
     Unexpected modifications:   Unknown [-58.01]

  

All peaks (56)  Matched peaks (32)  Not matched peaks (24)

  

| Scan | Peak | Mono mass | Mono m/z | Intensity | Charge | Theoretical mass | Ion | Pos | Mass error | PPM error |
| --- | --- | --- | --- | --- | --- | --- | --- | --- | --- | --- |
| 553 | 1 | 3352.8613 | 671.5795 | 183847.48 | 5 |  |  |  |  |  |
| 553 | 2 | 1705.4470 | 569.4896 | 364270.48 | 3 |  |  |  |  |  |
| 553 | 3 | 3126.6971 | 782.6815 | 77434.55 | 4 | 3126.7157 | C26 | 26 | -0.0187 | -5.97 |
| 553 | 4 | 3408.8932 | 569.1561 | 282701.38 | 6 |  |  |  |  |  |
| 553 | 5 | 3352.8637 | 839.2232 | 64977.99 | 4 |  |  |  |  |  |
| 553 | 6 | 3338.8486 | 668.7770 | 69862.01 | 5 | 3338.8682 | C28 | 28 | -0.0196 | -5.87 |
| 553 | 7 | 2274.1479 | 759.0566 | 55126.77 | 3 | 2274.1612 | C18 | 18 | -0.0133 | -5.86 |
| 553 | 8 | 2899.5352 | 725.8911 | 47026.23 | 4 | 2899.5523 | C24 | 24 | -0.0171 | -5.91 |
| 553 | 9 | 2145.1060 | 716.0426 | 49465.75 | 3 | 2145.1186 | C17 | 17 | -0.0127 | -5.91 |
| 553 | 10 | 2828.4986 | 708.1319 | 46712.62 | 4 | 2828.5152 | C23 | 23 | -0.0167 | -5.89 |
| 553 | 11 | 1884.9552 | 629.3257 | 57337.37 | 3 | 1884.9662 | C15 | 15 | -0.0109 | -5.81 |
| 553 | 12 | 1525.9381 | 763.9763 | 53685.58 | 2 | 1525.9399 | Z\_DOT15 | 15 | -1.72e-03 | -1.13 |
| 553 | 13 | 3393.8666 | 679.7806 | 31083.25 | 5 |  |  |  |  |  |
| 553 | 14 | 568.6486 | 569.6559 | 223434.14 | 1 |  |  |  |  |  |
| 553 | 15 | 3322.8278 | 665.5728 | 35713.84 | 5 | 3322.8424 | Z\_DOT29 | 1 | -0.0146 | -4.40 |
| 553 | 16 | 3194.7356 | 799.6912 | 27846.91 | 4 | 3194.7475 | Z\_DOT28 | 2 | -0.0118 | -3.70 |
| 553 | 17 | 2459.2633 | 820.7617 | 29462.93 | 3 | 2459.2776 | C20 | 20 | -0.0143 | -5.83 |
| 553 | 18 | 3392.8634 | 566.4845 | 27447.85 | 6 |  |  |  |  |  |
| 553 | 19 | 2700.4053 | 901.1424 | 27431.54 | 3 | 2700.4203 | C22 | 22 | -0.0149 | -5.53 |
| 553 | 20 | 2032.0226 | 678.3481 | 33076.40 | 3 | 2032.0346 | C16 | 16 | -0.0120 | -5.89 |
| 553 | 21 | 3365.8719 | 674.1817 | 18596.82 | 5 |  |  |  |  |  |
| 553 | 22 | 3322.8300 | 831.7148 | 37113.49 | 4 | 3322.8424 | Z\_DOT29 | 1 | -0.0124 | -3.74 |
| 553 | 23 | 3239.7793 | 810.9521 | 18556.09 | 4 | 3239.7998 | C27 | 27 | -0.0204 | -6.31 |
| 553 | 24 | 3366.8768 | 842.7265 | 20618.02 | 4 |  |  |  |  |  |
| 553 | 25 | 3408.8835 | 682.7840 | 78619.48 | 5 |  |  |  |  |  |
| 553 | 26 | 3027.6294 | 757.9146 | 23199.51 | 4 | 3027.6473 | C25 | 25 | -0.0179 | -5.90 |
| 553 | 27 | 1884.9555 | 943.4850 | 24254.01 | 2 | 1884.9662 | C15 | 15 | -0.0106 | -5.64 |
| 553 | 28 | 2345.3814 | 587.3526 | 18860.57 | 4 |  |  |  |  |  |
| 553 | 29 | 2700.4039 | 676.1082 | 20188.63 | 4 | 2700.4203 | C22 | 22 | -0.0164 | -6.08 |
| 553 | 30 | 1756.8612 | 879.4379 | 22773.28 | 2 | 1756.8712 | C14 | 14 | -9.96e-03 | -5.67 |
| 553 | 31 | 1265.7873 | 633.9009 | 25597.63 | 2 | 1265.7874 | Z\_DOT13 | 17 | -1.31e-04 | -0.10 |
| 553 | 32 | 1378.8705 | 690.4425 | 18526.36 | 2 | 1378.8715 | Z\_DOT14 | 16 | -9.38e-04 | -0.68 |
| 553 | 33 | 3210.7535 | 803.6956 | 15057.73 | 4 |  |  |  |  |  |
| 553 | 34 | 3338.8483 | 835.7193 | 15893.93 | 4 | 3338.8682 | C28 | 28 | -0.0199 | -5.96 |
| 553 | 35 | 682.1783 | 683.1855 | 61164.29 | 1 |  |  |  |  |  |
| 553 | 36 | 1364.7566 | 683.3856 | 115885.75 | 2 |  |  |  |  |  |
| 553 | 37 | 582.3949 | 583.4022 | 14035.56 | 1 | 582.3908 | Z\_DOT7 | 23 | 4.16e-03 | 7.14 |
| 553 | 38 | 1007.4839 | 1008.4911 | 13592.64 | 1 | 1007.4892 | C8 | 8 | -5.30e-03 | -5.26 |
| 553 | 39 | 710.4891 | 711.4963 | 11154.80 | 1 | 710.4857 | Z\_DOT8 | 22 | 3.32e-03 | 4.67 |
| 553 | 40 | 908.5758 | 455.2952 | 13838.50 | 2 |  |  |  |  |  |
| 553 | 41 | 1206.7743 | 604.3944 | 6360.74 | 2 |  |  |  |  |  |
| 553 | 42 | 1135.5420 | 1136.5493 | 6254.04 | 1 | 1135.5477 | C9 | 9 | -5.77e-03 | -5.08 |
| 553 | 43 | 1152.7640 | 577.3893 | 3809.82 | 2 |  |  |  |  |  |
| 553 | 44 | 511.3584 | 512.3657 | 7455.64 | 1 | 511.3537 | Z\_DOT6 | 24 | 4.71e-03 | 9.21 |
| 553 | 45 | 853.2254 | 854.2327 | 20664.05 | 1 |  |  |  |  |  |
| 553 | 46 | 1496.8989 | 499.9736 | 3429.80 | 3 |  |  |  |  |  |
| 553 | 47 | 1263.5997 | 1264.6070 | 2954.54 | 1 | 1263.6063 | C10 | 10 | -6.61e-03 | -5.23 |
| 553 | 48 | 473.2939 | 474.3012 | 7096.46 | 1 | 473.2961 | C4 | 4 | -2.19e-03 | -4.62 |
| 553 | 49 | 1349.8315 | 675.9230 | 5440.31 | 2 |  |  |  |  |  |
| 553 | 50 | 873.4696 | 874.4769 | 3772.25 | 1 |  |  |  |  |  |
| 553 | 51 | 967.6490 | 484.8318 | 2556.53 | 2 |  |  |  |  |  |
| 553 | 52 | 746.4063 | 747.4136 | 3469.88 | 1 | 746.4108 | C6 | 6 | -4.54e-03 | -6.08 |
| 553 | 53 | 550.0765 | 551.0837 | 4039.64 | 1 |  |  |  |  |  |
| 553 | 54 | 1007.4839 | 504.7492 | 3684.34 | 2 | 1007.4892 | C8 | 8 | -5.31e-03 | -5.27 |
| 553 | 55 | 780.5180 | 391.2663 | 2455.94 | 2 |  |  |  |  |  |
| 553 | 56 | 344.2522 | 345.2595 | 2944.47 | 1 | 344.2535 | C3 | 3 | -1.35e-03 | -3.91 |

  

All proteins /
CsTx-12a\_S1 Cupiennius salei toxin 12 isoform a S1^ACsTx-12a\_S2 Cupiennius salei toxin 12 isoform a S2 /
Proteoform #53
