# Supplementary material for: The Dual Prey-Inactivation Strategy of Spiders—In-Depth Venomic Analysis of Cupiennius salei
Source: Toxins (Basel). 2019 Mar 19;11(3):167. doi: 10.3390/toxins11030167 (PMC6468893; doi:10.3390/toxins11030167)
Supplement: Supplementary file 1 [file toxins-11-00167-s001.zip › Supplementary Dataset EV1/20180328_f2_topdown_OTMS2_EThcD_NL_i02_ms2_proteoform_cutoff_html/prsms/prsm175.html]

Protein-Spectrum-Match for Spectrum #414


All proteins /
CsTx-1a\_S1 Cupiennius salei toxin 1 isoform a S1^ACsTx-1a\_S2 Cupiennius salei toxin 1 isoform a S2 /
Proteoform #15

## Protein-Spectrum-Match #175 for Spectrum #414

|  |  |  |  |  |  |
| --- | --- | --- | --- | --- | --- |
| PrSM ID: | 175 | Scan(s): | 555 | Precursor charge: | 12 |
| Precursor m/z: | 736.5268 | Precursor mass: | 8826.2342 | Proteoform mass: | 8826.1942 |
| # matched peaks: | 44 | # matched fragment ions: | 35 | # unexpected modifications: | 1 |
| E-value: | 1.48e-28 | P-value: | 1.48e-28 | Q-value (Spectral FDR): | 0 |

  

|  |  |  |  |  |  |  |  |  |  |  |  |  |  |  |  |  |  |  |  |  |  |  |  |  |  |  |  |  |  |  |  |  |  |  |  |  |  |  |  |  |  |  |  |  |  |  |  |  |  |  |  |  |  |  |  |  |  |  |  |  |  |  |  |  |  |  |  |  |  |
| --- | --- | --- | --- | --- | --- | --- | --- | --- | --- | --- | --- | --- | --- | --- | --- | --- | --- | --- | --- | --- | --- | --- | --- | --- | --- | --- | --- | --- | --- | --- | --- | --- | --- | --- | --- | --- | --- | --- | --- | --- | --- | --- | --- | --- | --- | --- | --- | --- | --- | --- | --- | --- | --- | --- | --- | --- | --- | --- | --- | --- | --- | --- | --- | --- | --- | --- | --- | --- | --- |
|  | |  | | | | | | | | | | | | | | | | | | | | | | | | | | | | | | | | | | | | | | | | | | | | | | | | | | | | | | | | | | | | | | | | | | | |
| 1 |  |  | M |  | K |  | V |  | L |  | I |  | I |  | S |  | A |  | V |  | L |  |  | F |  | I |  | T |  | I |  | F |  | S |  | N |  | I |  | S |  | A |  |  | E |  | I |  | E |  | D |  | D |  | F |  | L |  | E |  | D |  | E |  | 30 |  |
|  | |  | | | | | | | | | | | | | | | | | | | | | | | | | | | | | | | | | | | | | | | | | | | | | | | | | | | | | | | | | | | | | | | | | | | |
| 31 |  |  | S |  | F |  | E |  | A |  | E |  | D |  | I |  | I |  | P |  | F |  |  | F |  | E |  | N |  | E |  | Q |  | A |  | R | ] | S |  | C |  | I |  |  | P |  | K |  | H |  | E |  | E | ⎫ | C | ⎫ | T | ⎱ | N | ⎱ | D |  | K |  | 60 |  |
|  | |  | | | | | | | | | | | | | | | | | | | | | | | | | | | | | | | | | | | | | | | | | | | | | | | | | | | | | | | | | | | | | | | | | | | |
| 61 |  |  | H | ⎱ | N | ⎫ | C | ⎫ | C |  | R |  | K | ⎫ | G | ⎫ | L | ⎫ | F | ⎱ | K |  |  | L |  | K | ⎫ | C |  | Q | ⎫ | C |  | S |  | T |  | F | ⎫ | D |  | D |  | ⎫ | E | ⎫ | S | ⎫ | G | ⎱ | Q |  | P |  | T | ⎫ | E |  | R |  | C |  | A |  | 90 |  |
|  | |  | | | | | 14.96 | | | | | | | | | | | | | | | | | | | | | | | | | | | | | | | | | | | | | | | | | | | | | | | | | | | | | | | | | | | |
| 91 |  |  | C | ⎫ | G | ⎫ | R |  | P |  | M |  | G |  | H | ⎫ | Q | ⎫ | A |  | I |  |  | E | ⎫ | T |  | G |  | L |  | N |  | I |  | F |  | R | ⎫ | G |  | L |  |  | F |  | K |  | G |  | K | ⎫ | K |  | K | ⎫ | N | ⎫ | K |  | K | ⎫ | T |  | 120 |  |
|  | |  | | | | | | | | | | | | | | | | | | | | | | | | | | | | | | | | | | | | | | | | | | | | | | | | | | | | | | | | | | | | | | | | | | | |
| 121 |  | ⎫ | K | [ | G |  | | | | 122 |  | | | | | | | | | | | | | | | | | | | | | | | | | | | | | | | | | | | | | | | | | | | | | | | | | | | | | | | |

Fixed PTMs: Carbamidomethylation [C49 C56 C63 C64 C73 C75 C89 C91 ]   
  
     Unexpected modifications:   Unknown [14.96]

  

All peaks (147)  Matched peaks (44)  Not matched peaks (103)

  

| Scan | Peak | Mono mass | Mono m/z | Intensity | Charge | Theoretical mass | Ion | Pos | Mass error | PPM error |
| --- | --- | --- | --- | --- | --- | --- | --- | --- | --- | --- |
| 555 | 1 | 8769.1760 | 877.9249 | 97747.24 | 10 |  |  |  |  |  |
| 555 | 2 | 8770.1783 | 975.4715 | 74157.15 | 9 |  |  |  |  |  |
| 555 | 3 | 8769.1730 | 798.2048 | 45212.47 | 11 |  |  |  |  |  |
| 555 | 4 | 8783.1915 | 879.3264 | 34201.36 | 10 |  |  |  |  |  |
| 555 | 5 | 8770.1898 | 1097.2810 | 32431.47 | 8 |  |  |  |  |  |
| 555 | 6 | 8754.1619 | 973.6919 | 29219.35 | 9 |  |  |  |  |  |
| 555 | 7 | 8811.1828 | 882.1256 | 32591.90 | 10 |  |  |  |  |  |
| 555 | 8 | 8712.1579 | 969.0248 | 29683.55 | 9 |  |  |  |  |  |
| 555 | 9 | 4443.9069 | 889.7887 | 25333.24 | 5 | 4443.9333 | C36 | 36 | -0.0264 | -5.94 |
| 555 | 10 | 8725.1514 | 970.4685 | 22809.02 | 9 |  |  |  |  |  |
| 555 | 11 | 4414.6053 | 883.9283 | 47436.07 | 5 |  |  |  |  |  |
| 555 | 12 | 8753.1646 | 876.3237 | 21802.98 | 10 |  |  |  |  |  |
| 555 | 13 | 8784.1881 | 977.0282 | 23443.49 | 9 |  |  |  |  |  |
| 555 | 14 | 8811.1864 | 980.0280 | 25246.23 | 9 |  |  |  |  |  |
| 555 | 15 | 2203.3674 | 735.4631 | 33142.53 | 3 |  |  |  |  |  |
| 555 | 16 | 8697.1134 | 967.3532 | 16702.08 | 9 | 8697.1151 | C73 | 73 | -1.78e-03 | -0.20 |
| 555 | 17 | 8726.1673 | 1091.7782 | 17150.66 | 8 |  |  |  |  |  |
| 555 | 18 | 8712.1472 | 1090.0257 | 18072.06 | 8 |  |  |  |  |  |
| 555 | 19 | 8753.1644 | 1095.1528 | 18318.39 | 8 |  |  |  |  |  |
| 555 | 20 | 8710.1512 | 872.0224 | 20497.93 | 10 |  |  |  |  |  |
| 555 | 21 | 4325.2697 | 721.8856 | 13746.26 | 6 |  |  |  |  |  |
| 555 | 22 | 8725.1605 | 873.5233 | 13582.48 | 10 |  |  |  |  |  |
| 555 | 23 | 4770.0638 | 955.0200 | 14628.99 | 5 | 4770.0923 | C39 | 39 | -0.0286 | -5.99 |
| 555 | 24 | 1470.5789 | 736.2967 | 20338.16 | 2 |  |  |  |  |  |
| 555 | 25 | 8810.1813 | 801.9328 | 14107.48 | 11 |  |  |  |  |  |
| 555 | 26 | 4413.0905 | 736.5224 | 26728.75 | 6 |  |  |  |  |  |
| 555 | 27 | 1752.7582 | 877.3864 | 20123.04 | 2 | 1752.7671 | C14 | 14 | -8.92e-03 | -5.09 |
| 555 | 28 | 8782.1737 | 799.3867 | 13068.71 | 11 |  |  |  |  |  |
| 555 | 29 | 4384.2989 | 877.8671 | 14588.37 | 5 |  |  |  |  |  |
| 555 | 30 | 3157.4964 | 790.3814 | 14028.80 | 4 | 3157.5153 | C25 | 25 | -0.0190 | -6.00 |
| 555 | 31 | 6538.8065 | 935.1225 | 8035.74 | 7 | 6537.8097 | C54 | 54 | -5.49e-03 | -0.84 |
| 555 | 32 | 2788.2251 | 930.4156 | 13366.75 | 3 | 2788.2414 | C22 | 22 | -0.0163 | -5.84 |
| 555 | 33 | 3323.8842 | 831.9783 | 17307.49 | 4 |  |  |  |  |  |
| 555 | 34 | 5927.9176 | 988.9935 | 9017.69 | 6 |  |  |  |  |  |
| 555 | 35 | 3445.5839 | 862.4032 | 9720.72 | 4 | 3445.6046 | C27 | 27 | -0.0207 | -6.01 |
| 555 | 36 | 6097.5861 | 1017.2716 | 8205.14 | 6 | 6096.5873 | C50 | 50 | -3.59e-03 | -0.59 |
| 555 | 37 | 8696.1113 | 1088.0212 | 12475.09 | 8 | 8697.1151 | C73 | 73 | -1.49e-03 | -0.17 |
| 555 | 38 | 4299.8559 | 860.9784 | 8036.62 | 5 | 4299.8798 | C34 | 34 | -0.0240 | -5.57 |
| 555 | 39 | 1866.7994 | 934.4070 | 12327.25 | 2 | 1866.8101 | C15 | 15 | -0.0107 | -5.71 |
| 555 | 40 | 6225.6430 | 890.3849 | 8623.30 | 7 | 6224.6459 | C51 | 51 | -5.21e-03 | -0.84 |
| 555 | 41 | 5579.7064 | 930.9583 | 11989.43 | 6 |  |  |  |  |  |
| 555 | 42 | 8341.8725 | 927.8820 | 8706.34 | 9 |  |  |  |  |  |
| 555 | 43 | 8641.0863 | 1081.1431 | 7135.58 | 8 |  |  |  |  |  |
| 555 | 44 | 8785.1898 | 1099.1560 | 10948.60 | 8 |  |  |  |  |  |
| 555 | 45 | 4058.1417 | 812.6356 | 9020.15 | 5 |  |  |  |  |  |
| 555 | 46 | 3683.8336 | 737.7740 | 18738.03 | 5 |  |  |  |  |  |
| 555 | 47 | 2288.3964 | 763.8061 | 10426.36 | 3 |  |  |  |  |  |
| 555 | 48 | 5503.3240 | 918.2279 | 10032.05 | 6 | 5503.3559 | C45 | 45 | -0.0320 | -5.81 |
| 555 | 49 | 1169.7782 | 585.8964 | 12178.71 | 2 |  |  |  |  |  |
| 555 | 50 | 2471.0535 | 824.6918 | 8447.81 | 3 | 2471.0674 | C19 | 19 | -0.0140 | -5.65 |
| 555 | 51 | 4443.9128 | 1111.9855 | 8420.07 | 4 | 4443.9333 | C36 | 36 | -0.0205 | -4.62 |
| 555 | 52 | 6871.4111 | 859.9337 | 7444.70 | 8 |  |  |  |  |  |
| 555 | 53 | 2729.6187 | 910.8802 | 7759.19 | 3 |  |  |  |  |  |
| 555 | 54 | 2617.5755 | 655.4011 | 7629.88 | 4 |  |  |  |  |  |
| 555 | 55 | 8681.1436 | 965.5788 | 8808.96 | 9 |  |  |  |  |  |
| 555 | 56 | 7340.2506 | 918.5386 | 7043.83 | 8 | 7339.2594 | C61 | 61 | -0.0111 | -1.51 |
| 555 | 57 | 8681.1433 | 1086.1502 | 7104.86 | 8 |  |  |  |  |  |
| 555 | 58 | 2671.5867 | 668.9040 | 7610.14 | 4 |  |  |  |  |  |
| 555 | 59 | 8812.1982 | 1102.5320 | 9866.58 | 8 |  |  |  |  |  |
| 555 | 60 | 8640.0806 | 961.0162 | 8134.19 | 9 |  |  |  |  |  |
| 555 | 61 | 7970.6386 | 886.6338 | 8201.50 | 9 | 7969.6447 | C67 | 67 | -8.45e-03 | -1.06 |
| 555 | 62 | 2943.0712 | 982.0310 | 20680.99 | 3 |  |  |  |  |  |
| 555 | 63 | 3323.8809 | 665.7835 | 8957.26 | 5 |  |  |  |  |  |
| 555 | 64 | 2528.0725 | 843.6981 | 7034.25 | 3 | 2528.0889 | C20 | 20 | -0.0164 | -6.48 |
| 555 | 65 | 2026.8293 | 1014.4219 | 6896.71 | 2 | 2026.8407 | C16 | 16 | -0.0114 | -5.62 |
| 555 | 66 | 3998.1250 | 667.3614 | 8465.44 | 6 |  |  |  |  |  |
| 555 | 67 | 4271.2598 | 712.8839 | 6448.63 | 6 |  |  |  |  |  |
| 555 | 68 | 8283.8501 | 1036.4885 | 6361.90 | 8 |  |  |  |  |  |
| 555 | 69 | 3157.4976 | 1053.5065 | 6072.92 | 3 | 3157.5153 | C25 | 25 | -0.0178 | -5.63 |
| 555 | 70 | 8597.0312 | 956.2330 | 10088.47 | 9 | 8596.0674 | C72 | 72 | -0.0386 | -4.49 |
| 555 | 71 | 8469.9654 | 942.1145 | 6817.16 | 9 |  |  |  |  |  |
| 555 | 72 | 8240.9121 | 916.6642 | 8438.13 | 9 |  |  |  |  |  |
| 555 | 73 | 856.5690 | 857.5763 | 9391.91 | 1 |  |  |  |  |  |
| 555 | 74 | 4170.8123 | 835.1697 | 6453.26 | 5 | 4170.8372 | C33 | 33 | -0.0249 | -5.98 |
| 555 | 75 | 8099.7224 | 900.9764 | 6878.79 | 9 |  |  |  |  |  |
| 555 | 76 | 8226.8274 | 915.0992 | 8283.79 | 9 | 8225.8346 | C69 | 69 | -9.50e-03 | -1.16 |
| 555 | 77 | 8655.1152 | 962.6867 | 6208.10 | 9 |  |  |  |  |  |
| 555 | 78 | 7454.6363 | 932.8368 | 7161.87 | 8 | 7454.6157 | Z\_DOT63 | 11 | 0.0207 | 2.77 |
| 555 | 79 | 8738.1660 | 874.8239 | 6900.63 | 10 |  |  |  |  |  |
| 555 | 80 | 8226.8354 | 823.6908 | 6591.65 | 10 | 8225.8346 | C69 | 69 | -1.55e-03 | -0.19 |
| 555 | 81 | 8099.7455 | 1013.4755 | 7733.61 | 8 |  |  |  |  |  |
| 555 | 82 | 3940.7518 | 986.1952 | 6879.02 | 4 | 3940.7834 | C31 | 31 | -0.0316 | -8.01 |
| 555 | 83 | 6870.3981 | 982.4927 | 5638.58 | 7 |  |  |  |  |  |
| 555 | 84 | 8340.8638 | 835.0937 | 5800.36 | 10 | 8339.8775 | C70 | 70 | -0.0160 | -1.92 |
| 555 | 85 | 5503.3233 | 1101.6719 | 7799.38 | 5 | 5503.3559 | C45 | 45 | -0.0326 | -5.93 |
| 555 | 86 | 1372.5791 | 687.2968 | 6551.13 | 2 | 1372.5863 | C11 | 11 | -7.18e-03 | -5.23 |
| 555 | 87 | 2872.3077 | 958.4432 | 6442.36 | 3 |  |  |  |  |  |
| 555 | 88 | 2641.1568 | 881.3929 | 7481.85 | 3 | 2641.1730 | C21 | 21 | -0.0162 | -6.12 |
| 555 | 89 | 8666.1244 | 1084.2728 | 5370.09 | 8 |  |  |  |  |  |
| 555 | 90 | 7074.4600 | 885.3148 | 7564.42 | 8 | 7074.4349 | Z\_DOT60 | 14 | 0.0251 | 3.55 |
| 555 | 91 | 8753.1604 | 796.7491 | 8852.95 | 11 |  |  |  |  |  |
| 555 | 92 | 4383.2976 | 731.5569 | 7273.08 | 6 | 4383.2687 | Z\_DOT38 | 36 | 0.0289 | 6.59 |
| 555 | 93 | 5926.9037 | 847.7078 | 9450.19 | 7 |  |  |  |  |  |
| 555 | 94 | 7971.6420 | 997.4625 | 6805.71 | 8 |  |  |  |  |  |
| 555 | 95 | 4386.8892 | 1097.7296 | 7432.33 | 4 | 4386.9119 | C35 | 35 | -0.0227 | -5.17 |
| 555 | 96 | 997.4584 | 998.4657 | 7540.39 | 1 | 997.4651 | C8 | 8 | -6.63e-03 | -6.65 |
| 555 | 97 | 2017.2445 | 673.4221 | 5098.68 | 3 |  |  |  |  |  |
| 555 | 98 | 6038.9805 | 863.7188 | 7086.15 | 7 | 6038.9606 | Z\_DOT52 | 22 | 0.0199 | 3.30 |
| 555 | 99 | 8283.8410 | 921.4341 | 5689.60 | 9 |  |  |  |  |  |
| 555 | 100 | 1486.9506 | 744.4826 | 5855.79 | 2 |  |  |  |  |  |
| 555 | 101 | 2187.3495 | 730.1238 | 6410.47 | 3 |  |  |  |  |  |
| 555 | 102 | 8597.0605 | 1075.6398 | 4106.09 | 8 | 8596.0674 | C72 | 72 | -9.29e-03 | -1.08 |
| 555 | 103 | 8182.8673 | 1023.8657 | 5415.62 | 8 |  |  |  |  |  |
| 555 | 104 | 8239.9030 | 824.9976 | 3756.13 | 10 |  |  |  |  |  |
| 555 | 105 | 8342.8729 | 1043.8664 | 4635.73 | 8 |  |  |  |  |  |
| 555 | 106 | 4415.1051 | 1104.7835 | 6510.68 | 4 |  |  |  |  |  |
| 555 | 107 | 8041.7129 | 1006.2214 | 5589.71 | 8 |  |  |  |  |  |
| 555 | 108 | 4554.9379 | 760.1636 | 5537.82 | 6 |  |  |  |  |  |
| 555 | 109 | 5447.3197 | 1090.4712 | 6853.31 | 5 | 5446.3345 | C44 | 44 | -0.0171 | -3.13 |
| 555 | 110 | 8227.8632 | 1029.4902 | 7048.91 | 8 |  |  |  |  |  |
| 555 | 111 | 3929.1061 | 786.8285 | 4638.08 | 5 |  |  |  |  |  |
| 555 | 112 | 5962.5432 | 994.7645 | 3621.27 | 6 |  |  |  |  |  |
| 555 | 113 | 8182.8728 | 910.2154 | 4802.80 | 9 |  |  |  |  |  |
| 555 | 114 | 5580.7232 | 1117.1519 | 5100.20 | 5 |  |  |  |  |  |
| 555 | 115 | 8713.1492 | 1245.7429 | 4480.46 | 7 |  |  |  |  |  |
| 555 | 116 | 4902.4522 | 818.0826 | 4966.59 | 6 |  |  |  |  |  |
| 555 | 117 | 3870.0677 | 646.0186 | 4198.70 | 6 |  |  |  |  |  |
| 555 | 118 | 6098.5822 | 872.2333 | 5769.59 | 7 |  |  |  |  |  |
| 555 | 119 | 4657.3966 | 932.4866 | 6036.25 | 5 |  |  |  |  |  |
| 555 | 120 | 8770.2071 | 1253.8940 | 4785.77 | 7 |  |  |  |  |  |
| 555 | 121 | 3450.9311 | 691.1935 | 6244.57 | 5 |  |  |  |  |  |
| 555 | 122 | 5525.7170 | 921.9601 | 4633.57 | 6 |  |  |  |  |  |
| 555 | 123 | 7846.7648 | 872.8700 | 8814.37 | 9 |  |  |  |  |  |
| 555 | 124 | 6225.6470 | 1038.6151 | 4287.54 | 6 | 6224.6459 | C51 | 51 | -1.20e-03 | -0.19 |
| 555 | 125 | 7455.6456 | 1066.0995 | 5717.33 | 7 | 7454.6157 | Z\_DOT63 | 11 | 0.0275 | 3.69 |
| 555 | 126 | 8227.8499 | 1176.4144 | 4322.71 | 7 |  |  |  |  |  |
| 555 | 127 | 7568.6744 | 947.0916 | 2685.69 | 8 | 7568.6586 | Z\_DOT64 | 10 | 0.0158 | 2.09 |
| 555 | 128 | 4528.3631 | 906.6799 | 5907.56 | 5 |  |  |  |  |  |
| 555 | 129 | 600.3804 | 601.3877 | 4551.37 | 1 |  |  |  |  |  |
| 555 | 130 | 1185.7973 | 593.9059 | 2987.19 | 2 |  |  |  |  |  |
| 555 | 131 | 1428.8845 | 477.3021 | 2537.71 | 3 |  |  |  |  |  |
| 555 | 132 | 802.6555 | 803.6628 | 11229.45 | 1 |  |  |  |  |  |
| 555 | 133 | 1372.5796 | 1373.5869 | 2699.88 | 1 | 1372.5863 | C11 | 11 | -6.74e-03 | -4.91 |
| 555 | 134 | 1258.5365 | 1259.5438 | 2245.83 | 1 | 1258.5434 | C10 | 10 | -6.90e-03 | -5.49 |
| 555 | 135 | 1317.8507 | 659.9326 | 2181.79 | 2 |  |  |  |  |  |
| 555 | 136 | 502.2424 | 503.2497 | 2589.82 | 1 |  |  |  |  |  |
| 555 | 137 | 486.3383 | 487.3456 | 2381.73 | 1 |  |  |  |  |  |
| 555 | 138 | 428.2731 | 429.2804 | 1711.42 | 1 |  |  |  |  |  |
| 555 | 139 | 953.4481 | 954.4554 | 2111.78 | 1 |  |  |  |  |  |
| 555 | 140 | 894.4330 | 895.4403 | 2088.87 | 1 |  |  |  |  |  |
| 555 | 141 | 788.3531 | 789.3604 | 1371.26 | 1 |  |  |  |  |  |
| 555 | 142 | 542.3150 | 543.3223 | 1614.12 | 1 |  |  |  |  |  |
| 555 | 143 | 1157.4890 | 1158.4963 | 1218.14 | 1 | 1157.4957 | C9 | 9 | -6.71e-03 | -5.80 |
| 555 | 144 | 1086.1405 | 1087.1478 | 1168.01 | 1 |  |  |  |  |  |
| 555 | 145 | 1098.7057 | 550.3601 | 1103.83 | 2 |  |  |  |  |  |
| 555 | 146 | 1041.6848 | 1042.6921 | 1416.93 | 1 |  |  |  |  |  |
| 555 | 147 | 1237.1637 | 1238.1710 | 872.63 | 1 |  |  |  |  |  |

  

All proteins /
CsTx-1a\_S1 Cupiennius salei toxin 1 isoform a S1^ACsTx-1a\_S2 Cupiennius salei toxin 1 isoform a S2 /
Proteoform #15
