# Supplementary material for: The Dual Prey-Inactivation Strategy of Spiders—In-Depth Venomic Analysis of Cupiennius salei
Source: Toxins (Basel). 2019 Mar 19;11(3):167. doi: 10.3390/toxins11030167 (PMC6468893; doi:10.3390/toxins11030167)
Supplement: Supplementary file 1 [file toxins-11-00167-s001.zip › Supplementary Dataset EV1/20180328_f2_topdown_OTMS2_EThcD_NL_i02_ms2_proteoform_cutoff_html/prsms/prsm176.html]

Protein-Spectrum-Match for Spectrum #415


All proteins /
CsTx-1a\_S1 Cupiennius salei toxin 1 isoform a S1^ACsTx-1a\_S2 Cupiennius salei toxin 1 isoform a S2 /
Proteoform #15

## Protein-Spectrum-Match #176 for Spectrum #415

|  |  |  |  |  |  |
| --- | --- | --- | --- | --- | --- |
| PrSM ID: | 176 | Scan(s): | 556 | Precursor charge: | 11 |
| Precursor m/z: | 803.3923 | Precursor mass: | 8826.2354 | Proteoform mass: | 8826.2054 |
| # matched peaks: | 43 | # matched fragment ions: | 35 | # unexpected modifications: | 1 |
| E-value: | 9.97e-29 | P-value: | 9.97e-29 | Q-value (Spectral FDR): | 0 |

  

|  |  |  |  |  |  |  |  |  |  |  |  |  |  |  |  |  |  |  |  |  |  |  |  |  |  |  |  |  |  |  |  |  |  |  |  |  |  |  |  |  |  |  |  |  |  |  |  |  |  |  |  |  |  |  |  |  |  |  |  |  |  |  |  |  |  |  |  |  |  |
| --- | --- | --- | --- | --- | --- | --- | --- | --- | --- | --- | --- | --- | --- | --- | --- | --- | --- | --- | --- | --- | --- | --- | --- | --- | --- | --- | --- | --- | --- | --- | --- | --- | --- | --- | --- | --- | --- | --- | --- | --- | --- | --- | --- | --- | --- | --- | --- | --- | --- | --- | --- | --- | --- | --- | --- | --- | --- | --- | --- | --- | --- | --- | --- | --- | --- | --- | --- | --- | --- |
|  | |  | | | | | | | | | | | | | | | | | | | | | | | | | | | | | | | | | | | | | | | | | | | | | | | | | | | | | | | | | | | | | | | | | | | |
| 1 |  |  | M |  | K |  | V |  | L |  | I |  | I |  | S |  | A |  | V |  | L |  |  | F |  | I |  | T |  | I |  | F |  | S |  | N |  | I |  | S |  | A |  |  | E |  | I |  | E |  | D |  | D |  | F |  | L |  | E |  | D |  | E |  | 30 |  |
|  | |  | | | | | | | | | | | | | | | | | | | | | | | | | | | | | | | | | | | | | | | | | | | | | | | | | | | | | | | | | | | | | | | | | | | |
| 31 |  |  | S |  | F |  | E |  | A |  | E |  | D |  | I |  | I |  | P |  | F |  |  | F |  | E |  | N |  | E |  | Q |  | A |  | R | ] | S |  | C |  | I |  |  | P |  | K | ⎱ | H |  | E |  | E | ⎫ | C |  | T |  | N | ⎫ | D |  | K |  | 60 |  |
|  | |  | | | | | | | | | | | | | | | | | | | | | | | | | | | | | | | | | | | | | | | | | | | | | | | | | | | | | | | | | | | | | | | | | | | |
| 61 |  |  | H | ⎱ | N | ⎫ | C | ⎫ | C |  | R |  | K |  | G | ⎫ | L | ⎫ | F | ⎱ | K |  | ⎫ | L |  | K | ⎫ | C |  | Q | ⎫ | C |  | S |  | T |  | F | ⎫ | D | ⎫ | D |  | ⎱ | E | ⎫ | S |  | G | ⎫ | Q |  | P |  | T | ⎩ | E | ⎩ | R |  | C |  | A |  | 90 |  |
|  | |  | | | | | | | | | 14.97 | | | | | | | | | | | | | | | | | | | | | | | | | | | | | | | | | | | | | | | | | | | | | | | | | | | | | | | |
| 91 |  |  | C | ⎫ | G | ⎱ | R |  | P | ⎫ | M |  | G |  | H |  | Q |  | A |  | I |  |  | E | ⎫ | T | ⎩ | G |  | L |  | N |  | I |  | F |  | R |  | G |  | L |  | ⎩ | F |  | K |  | G |  | K | ⎫ | K | ⎫ | K | ⎫ | N | ⎫ | K |  | K |  | T |  | 120 |  |
|  | |  | | | | | | | | | | | | | | | | | | | | | | | | | | | | | | | | | | | | | | | | | | | | | | | | | | | | | | | | | | | | | | | | | | | |
| 121 |  | ⎫ | K | [ | G |  | | | | 122 |  | | | | | | | | | | | | | | | | | | | | | | | | | | | | | | | | | | | | | | | | | | | | | | | | | | | | | | | |

Fixed PTMs: Carbamidomethylation [C49 C56 C63 C64 C73 C75 C89 C91 ]   
  
     Unexpected modifications:   Unknown [14.97]

  

All peaks (145)  Matched peaks (43)  Not matched peaks (102)

  

| Scan | Peak | Mono mass | Mono m/z | Intensity | Charge | Theoretical mass | Ion | Pos | Mass error | PPM error |
| --- | --- | --- | --- | --- | --- | --- | --- | --- | --- | --- |
| 556 | 1 | 8769.1714 | 975.3597 | 45551.01 | 9 |  |  |  |  |  |
| 556 | 2 | 2404.3445 | 802.4554 | 79993.25 | 3 |  |  |  |  |  |
| 556 | 3 | 8770.1722 | 1097.2788 | 33752.15 | 8 |  |  |  |  |  |
| 556 | 4 | 8769.1631 | 877.9236 | 25400.44 | 10 |  |  |  |  |  |
| 556 | 5 | 8810.1720 | 979.9153 | 20228.85 | 9 |  |  |  |  |  |
| 556 | 6 | 8783.1735 | 976.9154 | 15239.68 | 9 |  |  |  |  |  |
| 556 | 7 | 8784.1781 | 1099.0295 | 15234.03 | 8 |  |  |  |  |  |
| 556 | 8 | 8770.1746 | 1253.8894 | 13527.32 | 7 |  |  |  |  |  |
| 556 | 9 | 8725.1615 | 1091.6525 | 14193.91 | 8 |  |  |  |  |  |
| 556 | 10 | 4413.5938 | 883.7260 | 27173.39 | 5 |  |  |  |  |  |
| 556 | 11 | 8753.1485 | 973.5793 | 12071.43 | 9 |  |  |  |  |  |
| 556 | 12 | 8811.1800 | 1102.4048 | 12647.54 | 8 |  |  |  |  |  |
| 556 | 13 | 8712.1214 | 1090.0225 | 16920.51 | 8 |  |  |  |  |  |
| 556 | 14 | 5926.8985 | 988.8237 | 9513.10 | 6 |  |  |  |  |  |
| 556 | 15 | 8754.1492 | 1095.2759 | 15294.36 | 8 |  |  |  |  |  |
| 556 | 16 | 3323.8810 | 831.9775 | 13500.69 | 4 | 3323.8574 | Z\_DOT29 | 45 | 0.0236 | 7.11 |
| 556 | 17 | 8712.1310 | 969.0218 | 9293.82 | 9 |  |  |  |  |  |
| 556 | 18 | 8810.1660 | 882.0239 | 9063.68 | 10 |  |  |  |  |  |
| 556 | 19 | 8782.1672 | 879.2240 | 8348.43 | 10 |  |  |  |  |  |
| 556 | 20 | 4443.9035 | 1111.9831 | 7276.19 | 4 | 4443.9333 | C36 | 36 | -0.0298 | -6.71 |
| 556 | 21 | 2872.3049 | 958.4423 | 8499.75 | 3 |  |  |  |  |  |
| 556 | 22 | 8712.1332 | 1245.5977 | 9440.44 | 7 |  |  |  |  |  |
| 556 | 23 | 2788.2236 | 930.4152 | 8659.23 | 3 | 2788.2414 | C22 | 22 | -0.0177 | -6.36 |
| 556 | 24 | 4414.0993 | 1104.5321 | 11107.50 | 4 |  |  |  |  |  |
| 556 | 25 | 2943.3977 | 982.1398 | 17216.40 | 3 |  |  |  |  |  |
| 556 | 26 | 3210.7527 | 803.6955 | 13500.24 | 4 |  |  |  |  |  |
| 556 | 27 | 5579.6976 | 930.9569 | 7133.23 | 6 |  |  |  |  |  |
| 556 | 28 | 8753.1394 | 1251.4558 | 8036.23 | 7 |  |  |  |  |  |
| 556 | 29 | 2288.3944 | 763.8054 | 8215.50 | 3 |  |  |  |  |  |
| 556 | 30 | 8726.1424 | 970.5786 | 8497.66 | 9 |  |  |  |  |  |
| 556 | 31 | 3157.4939 | 1053.5052 | 7044.05 | 3 | 3157.5153 | C25 | 25 | -0.0214 | -6.79 |
| 556 | 32 | 4443.9048 | 889.7882 | 6633.16 | 5 | 4443.9333 | C36 | 36 | -0.0286 | -6.42 |
| 556 | 33 | 1866.7965 | 934.4055 | 7001.76 | 2 | 1866.8101 | C15 | 15 | -0.0136 | -7.27 |
| 556 | 34 | 1752.7560 | 877.3853 | 10921.76 | 2 | 1752.7671 | C14 | 14 | -0.0111 | -6.34 |
| 556 | 35 | 4384.2952 | 877.8663 | 7211.93 | 5 |  |  |  |  |  |
| 556 | 36 | 7075.4506 | 1011.7859 | 5108.76 | 7 | 7074.4462 | Z\_DOT60 | 14 | 2.12e-03 | 0.30 |
| 556 | 37 | 2729.6127 | 910.8782 | 6299.24 | 3 |  |  |  |  |  |
| 556 | 38 | 8697.0934 | 967.3510 | 4712.78 | 9 | 8697.1264 | C73 | 73 | -0.0330 | -3.79 |
| 556 | 39 | 4055.7811 | 1014.9525 | 5244.80 | 4 | 4055.8103 | C32 | 32 | -0.0292 | -7.21 |
| 556 | 40 | 1169.7771 | 585.8958 | 9295.10 | 2 |  |  |  |  |  |
| 556 | 41 | 856.5679 | 857.5752 | 6442.86 | 1 |  |  |  |  |  |
| 556 | 42 | 5503.3172 | 1101.6707 | 7865.82 | 5 | 5503.3559 | C45 | 45 | -0.0387 | -7.04 |
| 556 | 43 | 8098.7281 | 1013.3483 | 4381.97 | 8 | 8097.7509 | C68 | 68 | -0.0252 | -3.11 |
| 556 | 44 | 4299.8496 | 1075.9697 | 4049.56 | 4 | 4299.8798 | C34 | 34 | -0.0302 | -7.02 |
| 556 | 45 | 4012.9067 | 803.5886 | 18359.18 | 5 |  |  |  |  |  |
| 556 | 46 | 8725.1582 | 1247.4585 | 6687.67 | 7 |  |  |  |  |  |
| 556 | 47 | 3157.4939 | 790.3808 | 5993.56 | 4 | 3157.5153 | C25 | 25 | -0.0214 | -6.78 |
| 556 | 48 | 5291.6153 | 882.9432 | 5919.75 | 6 |  |  |  |  |  |
| 556 | 49 | 4325.2575 | 866.0588 | 4841.76 | 5 |  |  |  |  |  |
| 556 | 50 | 3323.8859 | 1108.9693 | 5579.50 | 3 | 3323.8574 | Z\_DOT29 | 45 | 0.0286 | 8.60 |
| 556 | 51 | 2671.5823 | 891.5347 | 5023.87 | 3 |  |  |  |  |  |
| 556 | 52 | 8340.8669 | 927.7703 | 8665.02 | 9 | 8339.8888 | C70 | 70 | -0.0243 | -2.91 |
| 556 | 53 | 6098.5829 | 1017.4378 | 4179.24 | 6 |  |  |  |  |  |
| 556 | 54 | 3381.9025 | 846.4829 | 4812.66 | 4 |  |  |  |  |  |
| 556 | 55 | 4384.2939 | 1097.0807 | 5285.79 | 4 |  |  |  |  |  |
| 556 | 56 | 3940.7576 | 986.1967 | 3468.28 | 4 | 3940.7834 | C31 | 31 | -0.0257 | -6.52 |
| 556 | 57 | 2026.8293 | 1014.4219 | 3580.66 | 2 | 2026.8407 | C16 | 16 | -0.0114 | -5.62 |
| 556 | 58 | 8695.1146 | 1087.8966 | 6885.00 | 8 |  |  |  |  |  |
| 556 | 59 | 1502.9673 | 752.4909 | 4203.55 | 2 |  |  |  |  |  |
| 556 | 60 | 4057.1427 | 812.4358 | 3997.75 | 5 | 4057.1209 | Z\_DOT35 | 39 | 0.0218 | 5.37 |
| 556 | 61 | 5525.6826 | 921.9544 | 4117.36 | 6 |  |  |  |  |  |
| 556 | 62 | 2232.3323 | 745.1180 | 3632.41 | 3 |  |  |  |  |  |
| 556 | 63 | 8785.2043 | 1256.0365 | 5701.24 | 7 |  |  |  |  |  |
| 556 | 64 | 4170.8029 | 1043.7080 | 3377.82 | 4 | 4170.8372 | C33 | 33 | -0.0344 | -8.24 |
| 556 | 65 | 6039.9745 | 1007.6697 | 3522.95 | 6 | 6038.9719 | Z\_DOT52 | 22 | 2.34e-04 | 0.04 |
| 556 | 66 | 6227.6462 | 1038.9483 | 2467.08 | 6 |  |  |  |  |  |
| 556 | 67 | 2601.5555 | 868.1924 | 4377.19 | 3 |  |  |  |  |  |
| 556 | 68 | 2405.3456 | 1203.6801 | 3423.68 | 2 |  |  |  |  |  |
| 556 | 69 | 5292.6080 | 1059.5289 | 3297.13 | 5 |  |  |  |  |  |
| 556 | 70 | 4325.2664 | 721.8850 | 3500.48 | 6 |  |  |  |  |  |
| 556 | 71 | 7912.6117 | 1131.3804 | 2062.19 | 7 |  |  |  |  |  |
| 556 | 72 | 728.4742 | 729.4814 | 4790.78 | 1 |  |  |  |  |  |
| 556 | 73 | 8225.8222 | 1029.2350 | 4681.07 | 8 | 8224.8923 | Z\_DOT69 | 5 | -0.0725 | -8.82 |
| 556 | 73 | 8225.8222 | 1029.2350 | 4681.07 | 8 | 8225.8459 | C69 | 69 | -0.0237 | -2.88 |
| 556 | 74 | 5927.8902 | 847.8487 | 3517.00 | 7 |  |  |  |  |  |
| 556 | 75 | 4528.3516 | 906.6776 | 3045.29 | 5 |  |  |  |  |  |
| 556 | 76 | 2617.5737 | 873.5319 | 3757.68 | 3 |  |  |  |  |  |
| 556 | 77 | 7075.4454 | 1180.2482 | 3818.13 | 6 | 7074.4462 | Z\_DOT60 | 14 | -3.06e-03 | -0.43 |
| 556 | 78 | 4769.0649 | 954.8203 | 2847.29 | 5 |  |  |  |  |  |
| 556 | 79 | 8679.1531 | 1085.9014 | 3568.33 | 8 |  |  |  |  |  |
| 556 | 80 | 3450.9296 | 863.7397 | 3022.81 | 4 |  |  |  |  |  |
| 556 | 81 | 6538.8001 | 935.1216 | 2390.93 | 7 | 6537.8210 | C54 | 54 | -0.0232 | -3.55 |
| 556 | 82 | 2470.0480 | 824.3566 | 2814.42 | 3 |  |  |  |  |  |
| 556 | 83 | 974.4625 | 975.4698 | 3336.60 | 1 |  |  |  |  |  |
| 556 | 84 | 3173.6753 | 794.4261 | 2166.30 | 4 |  |  |  |  |  |
| 556 | 85 | 7970.6271 | 997.3357 | 3538.17 | 8 | 7969.6560 | C67 | 67 | -0.0312 | -3.92 |
| 556 | 86 | 2641.1544 | 881.3921 | 2594.64 | 3 | 2641.1730 | C21 | 21 | -0.0185 | -7.02 |
| 556 | 87 | 8737.1494 | 1093.1509 | 4591.94 | 8 |  |  |  |  |  |
| 556 | 88 | 2528.0707 | 843.6975 | 2346.44 | 3 | 2528.0889 | C20 | 20 | -0.0182 | -7.20 |
| 556 | 89 | 5580.6922 | 1117.1457 | 3701.72 | 5 |  |  |  |  |  |
| 556 | 90 | 5926.8938 | 1186.3860 | 4402.97 | 5 |  |  |  |  |  |
| 556 | 91 | 3445.5826 | 1149.5348 | 2507.80 | 3 | 3445.6046 | C27 | 27 | -0.0220 | -6.38 |
| 556 | 92 | 2188.3527 | 730.4582 | 2385.92 | 3 | 2188.3447 | Z\_DOT19 | 55 | 8.07e-03 | 3.69 |
| 556 | 93 | 2203.3657 | 735.4625 | 2346.14 | 3 |  |  |  |  |  |
| 556 | 94 | 4656.3901 | 932.2853 | 3894.68 | 5 | 4656.3760 | Z\_DOT41 | 33 | 0.0140 | 3.02 |
| 556 | 95 | 8339.8409 | 1043.4874 | 4304.45 | 8 | 8339.8888 | C70 | 70 | -0.0480 | -5.75 |
| 556 | 96 | 3007.7352 | 752.9411 | 2658.81 | 4 |  |  |  |  |  |
| 556 | 97 | 2916.3171 | 973.1130 | 4100.03 | 3 | 2916.3363 | C23 | 23 | -0.0192 | -6.60 |
| 556 | 98 | 5447.2963 | 1090.4665 | 3836.91 | 5 | 5446.3345 | C44 | 44 | -0.0406 | -7.45 |
| 556 | 99 | 1095.2698 | 1096.2770 | 2453.97 | 1 |  |  |  |  |  |
| 556 | 100 | 997.4580 | 998.4653 | 3806.44 | 1 | 997.4651 | C8 | 8 | -7.06e-03 | -7.08 |
| 556 | 101 | 8668.1303 | 1239.3116 | 2664.12 | 7 |  |  |  |  |  |
| 556 | 102 | 5502.3117 | 918.0592 | 4077.93 | 6 | 5503.3559 | C45 | 45 | -0.0418 | -7.60 |
| 556 | 103 | 1722.7921 | 862.4034 | 2664.88 | 2 |  |  |  |  |  |
| 556 | 104 | 8655.1054 | 1082.8955 | 2268.40 | 8 |  |  |  |  |  |
| 556 | 105 | 8639.0716 | 1235.1604 | 2223.00 | 7 |  |  |  |  |  |
| 556 | 106 | 7913.6181 | 990.2095 | 1604.91 | 8 |  |  |  |  |  |
| 556 | 107 | 8285.8368 | 1036.7369 | 1968.88 | 8 |  |  |  |  |  |
| 556 | 108 | 5757.4637 | 960.5846 | 2317.12 | 6 | 5756.5098 | C47 | 47 | -0.0484 | -8.41 |
| 556 | 109 | 3184.5130 | 1062.5116 | 1705.96 | 3 |  |  |  |  |  |
| 556 | 110 | 1606.9676 | 804.4911 | 1050.48 | 2 |  |  |  |  |  |
| 556 | 111 | 8239.8881 | 1030.9933 | 3071.19 | 8 |  |  |  |  |  |
| 556 | 112 | 8738.1730 | 1249.3177 | 2122.83 | 7 |  |  |  |  |  |
| 556 | 113 | 8678.1424 | 965.2453 | 3107.05 | 9 |  |  |  |  |  |
| 556 | 114 | 602.3176 | 603.3248 | 4017.29 | 1 | 602.3210 | C5 | 5 | -3.41e-03 | -5.66 |
| 556 | 115 | 3008.7456 | 1003.9225 | 2293.66 | 3 |  |  |  |  |  |
| 556 | 116 | 2207.3019 | 1104.6582 | 4737.57 | 2 |  |  |  |  |  |
| 556 | 117 | 3927.0994 | 982.7821 | 3321.93 | 4 |  |  |  |  |  |
| 556 | 118 | 882.8194 | 883.8267 | 2549.18 | 1 |  |  |  |  |  |
| 556 | 119 | 2616.5690 | 655.1495 | 1674.28 | 4 |  |  |  |  |  |
| 556 | 120 | 8698.1341 | 1243.5979 | 4380.58 | 7 | 8697.1264 | C73 | 73 | 5.36e-03 | 0.62 |
| 556 | 121 | 3680.9896 | 921.2547 | 1290.29 | 4 |  |  |  |  |  |
| 556 | 122 | 8470.9783 | 1059.8796 | 2299.77 | 8 |  |  |  |  |  |
| 556 | 123 | 2288.3957 | 1145.2051 | 1504.11 | 2 |  |  |  |  |  |
| 556 | 124 | 1185.7969 | 593.9057 | 1598.96 | 2 |  |  |  |  |  |
| 556 | 125 | 8226.8664 | 1176.2739 | 4178.48 | 7 | 8225.8459 | C69 | 69 | 0.0182 | 2.21 |
| 556 | 126 | 4769.0530 | 1193.2705 | 2144.38 | 4 |  |  |  |  |  |
| 556 | 127 | 3236.8038 | 1079.9419 | 1613.75 | 3 |  |  |  |  |  |
| 556 | 128 | 3928.0964 | 786.6266 | 1740.39 | 5 | 3928.0784 | Z\_DOT34 | 40 | 0.0180 | 4.59 |
| 556 | 129 | 1486.9486 | 744.4816 | 3833.55 | 2 |  |  |  |  |  |
| 556 | 130 | 7845.7454 | 872.7568 | 1829.90 | 9 |  |  |  |  |  |
| 556 | 131 | 8039.7121 | 1149.5376 | 2022.04 | 7 |  |  |  |  |  |
| 556 | 132 | 8169.7925 | 1022.2313 | 1508.94 | 8 |  |  |  |  |  |
| 556 | 133 | 1372.5770 | 1373.5843 | 1270.05 | 1 | 1372.5863 | C11 | 11 | -9.30e-03 | -6.78 |
| 556 | 134 | 1317.8481 | 659.9313 | 1462.53 | 2 | 1317.8371 | Z\_DOT11 | 63 | 0.0110 | 8.31 |
| 556 | 135 | 802.8385 | 803.8458 | 4564.31 | 1 |  |  |  |  |  |
| 556 | 136 | 1111.7127 | 556.8636 | 1025.84 | 2 |  |  |  |  |  |
| 556 | 137 | 1372.5790 | 687.2968 | 1281.61 | 2 | 1372.5863 | C11 | 11 | -7.30e-03 | -5.32 |
| 556 | 138 | 486.3380 | 487.3452 | 1489.91 | 1 |  |  |  |  |  |
| 556 | 139 | 1065.5187 | 1066.5259 | 825.18 | 1 |  |  |  |  |  |
| 556 | 140 | 1151.7426 | 576.8786 | 987.38 | 2 |  |  |  |  |  |
| 556 | 141 | 1041.6843 | 1042.6916 | 1025.34 | 1 |  |  |  |  |  |
| 556 | 142 | 1428.8843 | 477.3020 | 959.22 | 3 |  |  |  |  |  |
| 556 | 143 | 1192.7008 | 1193.7081 | 880.26 | 1 |  |  |  |  |  |
| 556 | 144 | 1222.8655 | 1223.8727 | 960.19 | 1 |  |  |  |  |  |
| 556 | 145 | 918.9489 | 919.9562 | 576.32 | 1 |  |  |  |  |  |

  

All proteins /
CsTx-1a\_S1 Cupiennius salei toxin 1 isoform a S1^ACsTx-1a\_S2 Cupiennius salei toxin 1 isoform a S2 /
Proteoform #15
