# Supplementary material for: The Dual Prey-Inactivation Strategy of Spiders—In-Depth Venomic Analysis of Cupiennius salei
Source: Toxins (Basel). 2019 Mar 19;11(3):167. doi: 10.3390/toxins11030167 (PMC6468893; doi:10.3390/toxins11030167)
Supplement: Supplementary file 1 [file toxins-11-00167-s001.zip › Supplementary Dataset EV1/20180328_f2_topdown_OTMS2_EThcD_NL_i02_ms2_proteoform_cutoff_html/prsms/prsm177.html]

Protein-Spectrum-Match for Spectrum #416


All proteins /
CsTx-12b Cupiennius salei toxin 12 isoform b /
Proteoform #47

## Protein-Spectrum-Match #177 for Spectrum #416

|  |  |  |  |  |  |
| --- | --- | --- | --- | --- | --- |
| PrSM ID: | 177 | Scan(s): | 557 | Precursor charge: | 6 |
| Precursor m/z: | 571.9899 | Precursor mass: | 3425.8960 | Proteoform mass: | 3425.8910 |
| # matched peaks: | 27 | # matched fragment ions: | 25 | # unexpected modifications: | 1 |
| E-value: | 5.31e-20 | P-value: | 5.31e-20 | Q-value (Spectral FDR): | 0 |

  

|  |  |  |  |  |  |  |  |  |  |  |  |  |  |  |  |  |  |  |  |  |  |  |  |  |  |  |  |  |  |  |  |  |  |  |  |  |  |  |  |  |  |  |  |  |  |  |  |  |  |  |  |  |  |  |  |  |  |  |  |  |  |  |  |  |  |  |
| --- | --- | --- | --- | --- | --- | --- | --- | --- | --- | --- | --- | --- | --- | --- | --- | --- | --- | --- | --- | --- | --- | --- | --- | --- | --- | --- | --- | --- | --- | --- | --- | --- | --- | --- | --- | --- | --- | --- | --- | --- | --- | --- | --- | --- | --- | --- | --- | --- | --- | --- | --- | --- | --- | --- | --- | --- | --- | --- | --- | --- | --- | --- | --- | --- | --- | --- |
|  | | ... 30 amino acid residues are skipped at the N-terminus ... | | | | | | | | | | | | | | | | | | | | | | | | | | | | | | | | | | | | | | | | | | | | | | | | | | | | | | | | | | | | | |  | | |
|  | |  | | | | | | | | | | | | | | | | | | | | | | | | | | | | | | | | | | | | | | | | | | | | | | | | | | | | | | | | | | | | | | | | | | | |
| 31 |  |  | S |  | F |  | E |  | A |  | D |  | D |  | V |  | I |  | P |  | F |  |  | L |  | A |  | R |  | E |  | Q |  | V |  | R |  | S |  | D |  | C |  |  | T |  | L |  | R |  | N |  | H |  | D |  | C |  | T |  | D |  | D |  | 60 |  |
|  | |  | | | | | | | | | | | | | | | | | | | | | | | | | | | | | | | | | | | | | | | | | | | | | | | | | | | | | | | | | | | | | | | | | | | |
| 61 |  |  | R |  | H |  | S |  | C |  | C |  | R |  | S |  | K |  | M |  | F |  |  | K |  | D |  | V |  | C |  | K |  | C |  | F |  | Y |  | P |  | S |  |  | Q |  | R |  | S |  | D |  | T |  | A |  | R | ] | A | ⎩ | K | ⎩ | K |  | 90 |  |
|  | |  | | | | | | | | | | | | | | | | | | | | | | | | | | | | | | | | | | | | | | | | | | | | | | | | | | | | | -58.01 | | | | | | | | | | | |
| 91 |  |  | E | ⎫ | L |  | C |  | T | ⎫ | C | ⎫ | Q | ⎫ | Q |  | D | ⎱ | K |  | H |  | ⎫ | L | ⎫ | K | ⎱ | Y |  | I | ⎱ | E | ⎫ | K |  | G | ⎫ | L |  | Q | ⎱ | K |  | ⎫ | A | ⎱ | K | ⎫ | V | ⎫ | L | ⎫ | V | ⎫ | A |  | G |  | | 117 |  | | | | | |

Fixed PTMs: Carbamidomethylation [C93 C95 ]   
  
     Unexpected modifications:   Unknown [-58.01]

  

All peaks (57)  Matched peaks (27)  Not matched peaks (30)

  

| Scan | Peak | Mono mass | Mono m/z | Intensity | Charge | Theoretical mass | Ion | Pos | Mass error | PPM error |
| --- | --- | --- | --- | --- | --- | --- | --- | --- | --- | --- |
| 557 | 1 | 3368.8553 | 674.7783 | 119376.58 | 5 |  |  |  |  |  |
| 557 | 2 | 1713.4423 | 572.1547 | 217650.93 | 3 |  |  |  |  |  |
| 557 | 3 | 3424.8838 | 571.8212 | 181763.66 | 6 |  |  |  |  |  |
| 557 | 4 | 3142.6895 | 786.6796 | 44365.36 | 4 | 3142.7106 | C26 | 26 | -0.0211 | -6.73 |
| 557 | 5 | 3368.8566 | 843.2214 | 36382.89 | 4 |  |  |  |  |  |
| 557 | 6 | 2161.1000 | 721.3740 | 35164.08 | 3 | 2161.1135 | C17 | 17 | -0.0135 | -6.25 |
| 557 | 7 | 2048.2624 | 683.7614 | 37381.77 | 3 | 2048.2655 | Z\_DOT19 | 11 | -3.14e-03 | -1.53 |
| 557 | 8 | 3354.8419 | 671.9757 | 35088.19 | 5 | 3354.8631 | C28 | 28 | -0.0212 | -6.31 |
| 557 | 9 | 2475.2570 | 826.0929 | 25461.79 | 3 | 2475.2726 | C20 | 20 | -0.0155 | -6.28 |
| 557 | 10 | 1884.9545 | 629.3254 | 32392.01 | 3 | 1884.9662 | C15 | 15 | -0.0117 | -6.20 |
| 557 | 11 | 3210.7288 | 803.6895 | 19865.29 | 4 | 3210.7402 | Z\_DOT28 | 2 | -0.0114 | -3.56 |
| 557 | 12 | 1541.9322 | 771.9734 | 27967.16 | 2 | 1541.9327 | Z\_DOT15 | 15 | -4.91e-04 | -0.32 |
| 557 | 13 | 571.3140 | 572.3212 | 130847.77 | 1 |  |  |  |  |  |
| 557 | 14 | 3408.8555 | 569.1499 | 17000.44 | 6 |  |  |  |  |  |
| 557 | 15 | 2290.1416 | 764.3878 | 24022.21 | 3 | 2290.1561 | C18 | 18 | -0.0145 | -6.35 |
| 557 | 16 | 1378.6245 | 690.3195 | 30076.89 | 2 | 1378.6333 | C11 | 11 | -8.72e-03 | -6.33 |
| 557 | 17 | 2716.3981 | 906.4733 | 17194.19 | 3 | 2716.4152 | C22 | 22 | -0.0171 | -6.28 |
| 557 | 18 | 3338.8236 | 835.7132 | 22617.91 | 4 | 3338.8352 | Z\_DOT29 | 1 | -0.0116 | -3.47 |
| 557 | 19 | 2915.5291 | 729.8896 | 18810.06 | 4 | 2915.5473 | C24 | 24 | -0.0182 | -6.23 |
| 557 | 20 | 3382.8702 | 846.7248 | 16473.73 | 4 |  |  |  |  |  |
| 557 | 21 | 2844.4918 | 712.1302 | 15347.51 | 4 | 2844.5102 | C23 | 23 | -0.0184 | -6.47 |
| 557 | 22 | 3338.8202 | 668.7713 | 19067.83 | 5 | 3338.8352 | Z\_DOT29 | 1 | -0.0150 | -4.49 |
| 557 | 23 | 3381.8640 | 677.3801 | 13547.76 | 5 |  |  |  |  |  |
| 557 | 24 | 3409.8590 | 682.9791 | 15202.07 | 5 |  |  |  |  |  |
| 557 | 25 | 1265.7869 | 633.9007 | 17297.07 | 2 | 1265.7853 | Z\_DOT13 | 17 | 1.63e-03 | 1.29 |
| 557 | 26 | 3043.6233 | 761.9131 | 12751.77 | 4 | 3043.6422 | C25 | 25 | -0.0189 | -6.21 |
| 557 | 27 | 2489.4327 | 623.3654 | 10178.78 | 4 |  |  |  |  |  |
| 557 | 28 | 2716.3980 | 680.1068 | 10484.55 | 4 | 2716.4152 | C22 | 22 | -0.0172 | -6.33 |
| 557 | 29 | 3255.7732 | 814.9506 | 8575.37 | 4 | 3255.7947 | C27 | 27 | -0.0215 | -6.59 |
| 557 | 30 | 3226.7474 | 807.6941 | 7977.67 | 4 |  |  |  |  |  |
| 557 | 31 | 1557.9506 | 779.9826 | 13811.96 | 2 |  |  |  |  |  |
| 557 | 32 | 1643.7771 | 822.8958 | 11945.56 | 2 | 1643.7871 | C13 | 13 | -0.0100 | -6.09 |
| 557 | 33 | 1756.8609 | 879.4377 | 13170.93 | 2 | 1756.8712 | C14 | 14 | -0.0103 | -5.88 |
| 557 | 34 | 2435.4212 | 609.8626 | 10783.12 | 4 |  |  |  |  |  |
| 557 | 35 | 908.5756 | 455.2951 | 13277.24 | 2 |  |  |  |  |  |
| 557 | 36 | 2361.3746 | 591.3509 | 12896.12 | 4 |  |  |  |  |  |
| 557 | 37 | 685.5774 | 686.5847 | 61792.41 | 1 |  |  |  |  |  |
| 557 | 38 | 1007.4833 | 1008.4905 | 9050.19 | 1 | 1007.4892 | C8 | 8 | -5.91e-03 | -5.86 |
| 557 | 39 | 1364.3455 | 683.1800 | 17076.90 | 2 |  |  |  |  |  |
| 557 | 40 | 553.0759 | 554.0831 | 21029.32 | 1 |  |  |  |  |  |
| 557 | 41 | 710.4889 | 711.4962 | 6021.53 | 1 | 710.4836 | Z\_DOT8 | 22 | 5.32e-03 | 7.49 |
| 557 | 42 | 1206.7740 | 604.3943 | 4742.35 | 2 |  |  |  |  |  |
| 557 | 43 | 417.0752 | 418.0824 | 11907.82 | 1 |  |  |  |  |  |
| 557 | 44 | 1135.5415 | 1136.5488 | 4471.95 | 1 | 1135.5477 | C9 | 9 | -6.26e-03 | -5.51 |
| 557 | 45 | 847.4535 | 848.4608 | 4676.47 | 1 | 847.4585 | C7 | 7 | -4.97e-03 | -5.87 |
| 557 | 46 | 360.0541 | 361.0613 | 7385.03 | 1 |  |  |  |  |  |
| 557 | 47 | 1121.4823 | 561.7484 | 2132.69 | 2 |  |  |  |  |  |
| 557 | 48 | 535.0654 | 536.0726 | 3813.22 | 1 |  |  |  |  |  |
| 557 | 49 | 473.2937 | 474.3010 | 4082.86 | 1 | 473.2961 | C4 | 4 | -2.43e-03 | -5.14 |
| 557 | 50 | 342.0436 | 343.0509 | 3963.16 | 1 |  |  |  |  |  |
| 557 | 51 | 967.6485 | 484.8315 | 2414.73 | 2 |  |  |  |  |  |
| 557 | 52 | 392.0260 | 393.0333 | 4417.30 | 1 |  |  |  |  |  |
| 557 | 53 | 726.5071 | 727.5144 | 1600.01 | 1 |  |  |  |  |  |
| 557 | 54 | 1024.0067 | 1025.0140 | 1584.28 | 1 |  |  |  |  |  |
| 557 | 55 | 511.3581 | 512.3654 | 3285.61 | 1 | 511.3515 | Z\_DOT6 | 24 | 6.60e-03 | 12.90 |
| 557 | 56 | 1431.7957 | 716.9051 | 1270.68 | 2 |  |  |  |  |  |
| 557 | 57 | 1209.1181 | 1210.1254 | 1146.04 | 1 |  |  |  |  |  |

  

All proteins /
CsTx-12b Cupiennius salei toxin 12 isoform b /
Proteoform #47
