# Supplementary material for: The Dual Prey-Inactivation Strategy of Spiders—In-Depth Venomic Analysis of Cupiennius salei
Source: Toxins (Basel). 2019 Mar 19;11(3):167. doi: 10.3390/toxins11030167 (PMC6468893; doi:10.3390/toxins11030167)
Supplement: Supplementary file 1 [file toxins-11-00167-s001.zip › Supplementary Dataset EV1/20180328_f2_topdown_OTMS2_EThcD_NL_i02_ms2_proteoform_cutoff_html/prsms/prsm178.html]

Protein-Spectrum-Match for Spectrum #417


All proteins /
CsTx-12a\_S1 Cupiennius salei toxin 12 isoform a S1^ACsTx-12a\_S2 Cupiennius salei toxin 12 isoform a S2 /
Proteoform #53

## Protein-Spectrum-Match #178 for Spectrum #417

|  |  |  |  |  |  |
| --- | --- | --- | --- | --- | --- |
| PrSM ID: | 178 | Scan(s): | 559 | Precursor charge: | 6 |
| Precursor m/z: | 569.3239 | Precursor mass: | 3409.8999 | Proteoform mass: | 3409.8999 |
| # matched peaks: | 32 | # matched fragment ions: | 27 | # unexpected modifications: | 1 |
| E-value: | 1.14e-20 | P-value: | 1.14e-20 | Q-value (Spectral FDR): | 0 |

  

|  |  |  |  |  |  |  |  |  |  |  |  |  |  |  |  |  |  |  |  |  |  |  |  |  |  |  |  |  |  |  |  |  |  |  |  |  |  |  |  |  |  |  |  |  |  |  |  |  |  |  |  |  |  |  |  |  |  |  |  |  |  |  |  |  |  |  |
| --- | --- | --- | --- | --- | --- | --- | --- | --- | --- | --- | --- | --- | --- | --- | --- | --- | --- | --- | --- | --- | --- | --- | --- | --- | --- | --- | --- | --- | --- | --- | --- | --- | --- | --- | --- | --- | --- | --- | --- | --- | --- | --- | --- | --- | --- | --- | --- | --- | --- | --- | --- | --- | --- | --- | --- | --- | --- | --- | --- | --- | --- | --- | --- | --- | --- | --- |
|  | | ... 30 amino acid residues are skipped at the N-terminus ... | | | | | | | | | | | | | | | | | | | | | | | | | | | | | | | | | | | | | | | | | | | | | | | | | | | | | | | | | | | | | |  | | |
|  | |  | | | | | | | | | | | | | | | | | | | | | | | | | | | | | | | | | | | | | | | | | | | | | | | | | | | | | | | | | | | | | | | | | | | |
| 31 |  |  | S |  | F |  | E |  | A |  | D |  | D |  | V |  | I |  | P |  | F |  |  | L |  | A |  | R |  | E |  | Q |  | V |  | R |  | S |  | D |  | C |  |  | T |  | L |  | R |  | N |  | H |  | D |  | C |  | T |  | D |  | D |  | 60 |  |
|  | |  | | | | | | | | | | | | | | | | | | | | | | | | | | | | | | | | | | | | | | | | | | | | | | | | | | | | | | | | | | | | | | | | | | | |
| 61 |  |  | R |  | H |  | S |  | C |  | C |  | R |  | S |  | K |  | M |  | F |  |  | K |  | D |  | V |  | C |  | K |  | C |  | F |  | Y |  | P |  | S |  |  | Q |  | R |  | S |  | D |  | T |  | A |  | R | ] | A | ⎩ | K | ⎩ | K |  | 90 |  |
|  | |  | | | | | | | | | | | | | | | | | | | | | | | | | | | | | | | | | | | | | | | | | | | | | | | | | | | | | -58.01 | | | | | | | | | | | |
| 91 |  | ⎫ | E | ⎫ | L |  | C |  | T |  | C | ⎫ | Q | ⎫ | Q | ⎫ | D |  | K |  | H |  |  | L | ⎫ | K | ⎱ | F | ⎱ | I | ⎱ | E | ⎫ | K |  | G | ⎫ | L | ⎩ | Q | ⎱ | K |  | ⎱ | A | ⎱ | K | ⎫ | V | ⎫ | L | ⎫ | V | ⎫ | A |  | G |  | | 117 |  | | | | | |

Fixed PTMs: Carbamidomethylation [C93 C95 ]   
  
     Unexpected modifications:   Unknown [-58.01]

  

All peaks (56)  Matched peaks (32)  Not matched peaks (24)

  

| Scan | Peak | Mono mass | Mono m/z | Intensity | Charge | Theoretical mass | Ion | Pos | Mass error | PPM error |
| --- | --- | --- | --- | --- | --- | --- | --- | --- | --- | --- |
| 559 | 1 | 3352.8616 | 671.5796 | 179772.40 | 5 |  |  |  |  |  |
| 559 | 2 | 3126.6968 | 782.6815 | 68813.20 | 4 | 3126.7157 | C26 | 26 | -0.0189 | -6.04 |
| 559 | 3 | 3408.8925 | 569.1560 | 263852.71 | 6 |  |  |  |  |  |
| 559 | 4 | 3352.8634 | 839.2231 | 61236.88 | 4 |  |  |  |  |  |
| 559 | 5 | 3338.8492 | 668.7771 | 59962.32 | 5 | 3338.8682 | C28 | 28 | -0.0190 | -5.69 |
| 559 | 6 | 2274.1477 | 759.0565 | 51640.93 | 3 | 2274.1612 | C18 | 18 | -0.0135 | -5.94 |
| 559 | 7 | 2145.1061 | 716.0427 | 45393.50 | 3 | 2145.1186 | C17 | 17 | -0.0125 | -5.82 |
| 559 | 8 | 1136.9648 | 569.4897 | 230686.47 | 2 |  |  |  |  |  |
| 559 | 9 | 2899.5355 | 725.8911 | 41969.50 | 4 | 2899.5523 | C24 | 24 | -0.0169 | -5.83 |
| 559 | 10 | 1884.9552 | 629.3257 | 52540.07 | 3 | 1884.9662 | C15 | 15 | -0.0109 | -5.81 |
| 559 | 11 | 3393.8660 | 679.7805 | 27405.02 | 5 |  |  |  |  |  |
| 559 | 12 | 3194.7344 | 799.6909 | 28211.61 | 4 | 3194.7492 | Z\_DOT28 | 2 | -0.0147 | -4.61 |
| 559 | 13 | 568.6486 | 569.6559 | 202379.93 | 1 |  |  |  |  |  |
| 559 | 14 | 2828.4983 | 708.1319 | 37032.10 | 4 | 2828.5152 | C23 | 23 | -0.0169 | -5.97 |
| 559 | 15 | 2032.0226 | 678.3481 | 30022.62 | 3 | 2032.0346 | C16 | 16 | -0.0120 | -5.89 |
| 559 | 16 | 1525.9383 | 763.9764 | 47905.76 | 2 | 1525.9416 | Z\_DOT15 | 15 | -3.30e-03 | -2.16 |
| 559 | 17 | 2459.2633 | 820.7617 | 28685.25 | 3 | 2459.2776 | C20 | 20 | -0.0143 | -5.83 |
| 559 | 18 | 3322.8284 | 665.5730 | 32331.54 | 5 | 3322.8441 | Z\_DOT29 | 1 | -0.0157 | -4.73 |
| 559 | 19 | 2700.4053 | 901.1424 | 27015.84 | 3 | 2700.4203 | C22 | 22 | -0.0149 | -5.53 |
| 559 | 20 | 3392.8619 | 566.4843 | 23968.69 | 6 |  |  |  |  |  |
| 559 | 21 | 3322.8312 | 831.7151 | 36697.77 | 4 | 3322.8441 | Z\_DOT29 | 1 | -0.0129 | -3.88 |
| 559 | 22 | 1884.9558 | 943.4852 | 27334.53 | 2 | 1884.9662 | C15 | 15 | -0.0104 | -5.51 |
| 559 | 23 | 3027.6297 | 757.9147 | 21234.44 | 4 | 3027.6473 | C25 | 25 | -0.0176 | -5.82 |
| 559 | 24 | 3239.7801 | 810.9523 | 18201.66 | 4 | 3239.7998 | C27 | 27 | -0.0197 | -6.08 |
| 559 | 25 | 2345.3812 | 587.3526 | 20696.57 | 4 |  |  |  |  |  |
| 559 | 26 | 3366.8781 | 842.7268 | 18330.41 | 4 |  |  |  |  |  |
| 559 | 27 | 3210.7532 | 803.6956 | 15705.77 | 4 |  |  |  |  |  |
| 559 | 28 | 1265.7873 | 633.9009 | 22468.89 | 2 | 1265.7891 | Z\_DOT13 | 17 | -1.83e-03 | -1.45 |
| 559 | 29 | 3365.8698 | 674.1812 | 16472.31 | 5 |  |  |  |  |  |
| 559 | 30 | 2700.4041 | 676.1083 | 18356.82 | 4 | 2700.4203 | C22 | 22 | -0.0162 | -5.99 |
| 559 | 31 | 3338.8480 | 835.7193 | 16925.17 | 4 | 3338.8682 | C28 | 28 | -0.0201 | -6.03 |
| 559 | 32 | 1378.8704 | 690.4425 | 17182.34 | 2 | 1378.8732 | Z\_DOT14 | 16 | -2.76e-03 | -2.00 |
| 559 | 33 | 3408.8826 | 682.7838 | 70195.99 | 5 |  |  |  |  |  |
| 559 | 34 | 1756.8612 | 879.4379 | 18600.92 | 2 | 1756.8712 | C14 | 14 | -9.96e-03 | -5.67 |
| 559 | 35 | 3393.8730 | 849.4755 | 19458.36 | 4 |  |  |  |  |  |
| 559 | 36 | 682.1783 | 683.1856 | 56905.84 | 1 |  |  |  |  |  |
| 559 | 37 | 1364.7567 | 683.3857 | 112252.95 | 2 |  |  |  |  |  |
| 559 | 38 | 1007.4840 | 1008.4913 | 13456.47 | 1 | 1007.4892 | C8 | 8 | -5.17e-03 | -5.13 |
| 559 | 39 | 908.5759 | 455.2952 | 14348.83 | 2 |  |  |  |  |  |
| 559 | 40 | 710.4891 | 711.4964 | 9649.59 | 1 | 710.4875 | Z\_DOT8 | 22 | 1.67e-03 | 2.35 |
| 559 | 41 | 582.3949 | 583.4022 | 12621.75 | 1 | 582.3925 | Z\_DOT7 | 23 | 2.45e-03 | 4.21 |
| 559 | 42 | 1349.8319 | 675.9232 | 4920.54 | 2 |  |  |  |  |  |
| 559 | 43 | 511.3584 | 512.3657 | 7242.99 | 1 | 511.3554 | Z\_DOT6 | 24 | 3.00e-03 | 5.87 |
| 559 | 44 | 1135.5416 | 1136.5489 | 6356.40 | 1 | 1135.5477 | C9 | 9 | -6.14e-03 | -5.40 |
| 559 | 45 | 1206.7744 | 604.3945 | 6316.90 | 2 |  |  |  |  |  |
| 559 | 46 | 1496.8995 | 499.9738 | 3864.63 | 3 |  |  |  |  |  |
| 559 | 47 | 853.2253 | 854.2325 | 18098.67 | 1 |  |  |  |  |  |
| 559 | 48 | 473.2940 | 474.3013 | 5428.86 | 1 | 473.2961 | C4 | 4 | -2.13e-03 | -4.49 |
| 559 | 49 | 873.4696 | 874.4769 | 3643.68 | 1 |  |  |  |  |  |
| 559 | 50 | 1152.7637 | 577.3891 | 3122.68 | 2 |  |  |  |  |  |
| 559 | 51 | 780.5181 | 391.2663 | 3040.61 | 2 |  |  |  |  |  |
| 559 | 52 | 1007.4835 | 504.7490 | 3371.37 | 2 | 1007.4892 | C8 | 8 | -5.67e-03 | -5.63 |
| 559 | 53 | 921.4402 | 922.4475 | 2876.43 | 1 |  |  |  |  |  |
| 559 | 54 | 1263.5997 | 1264.6070 | 2555.73 | 1 | 1263.6063 | C10 | 10 | -6.61e-03 | -5.23 |
| 559 | 55 | 344.2521 | 345.2594 | 3053.33 | 1 | 344.2535 | C3 | 3 | -1.41e-03 | -4.09 |
| 559 | 56 | 838.5459 | 839.5532 | 2404.32 | 1 | 838.5460 | Z\_DOT9 | 21 | -8.50e-05 | -0.10 |

  

All proteins /
CsTx-12a\_S1 Cupiennius salei toxin 12 isoform a S1^ACsTx-12a\_S2 Cupiennius salei toxin 12 isoform a S2 /
Proteoform #53
