# Supplementary material for: The Dual Prey-Inactivation Strategy of Spiders—In-Depth Venomic Analysis of Cupiennius salei
Source: Toxins (Basel). 2019 Mar 19;11(3):167. doi: 10.3390/toxins11030167 (PMC6468893; doi:10.3390/toxins11030167)
Supplement: Supplementary file 1 [file toxins-11-00167-s001.zip › Supplementary Dataset EV1/20180328_f2_topdown_OTMS2_EThcD_NL_i02_ms2_proteoform_cutoff_html/prsms/prsm179.html]

Protein-Spectrum-Match for Spectrum #418


All proteins /
CsTx-1a\_S1 Cupiennius salei toxin 1 isoform a S1^ACsTx-1a\_S2 Cupiennius salei toxin 1 isoform a S2 /
Proteoform #15

## Protein-Spectrum-Match #179 for Spectrum #418

|  |  |  |  |  |  |
| --- | --- | --- | --- | --- | --- |
| PrSM ID: | 179 | Scan(s): | 560 | Precursor charge: | 13 |
| Precursor m/z: | 680.0252 | Precursor mass: | 8827.2326 | Proteoform mass: | 8827.1926 |
| # matched peaks: | 54 | # matched fragment ions: | 39 | # unexpected modifications: | 1 |
| E-value: | 2.28e-32 | P-value: | 2.28e-32 | Q-value (Spectral FDR): | 0 |

  

|  |  |  |  |  |  |  |  |  |  |  |  |  |  |  |  |  |  |  |  |  |  |  |  |  |  |  |  |  |  |  |  |  |  |  |  |  |  |  |  |  |  |  |  |  |  |  |  |  |  |  |  |  |  |  |  |  |  |  |  |  |  |  |  |  |  |  |  |  |  |
| --- | --- | --- | --- | --- | --- | --- | --- | --- | --- | --- | --- | --- | --- | --- | --- | --- | --- | --- | --- | --- | --- | --- | --- | --- | --- | --- | --- | --- | --- | --- | --- | --- | --- | --- | --- | --- | --- | --- | --- | --- | --- | --- | --- | --- | --- | --- | --- | --- | --- | --- | --- | --- | --- | --- | --- | --- | --- | --- | --- | --- | --- | --- | --- | --- | --- | --- | --- | --- | --- |
|  | |  | | | | | | | | | | | | | | | | | | | | | | | | | | | | | | | | | | | | | | | | | | | | | | | | | | | | | | | | | | | | | | | | | | | |
| 1 |  |  | M |  | K |  | V |  | L |  | I |  | I |  | S |  | A |  | V |  | L |  |  | F |  | I |  | T |  | I |  | F |  | S |  | N |  | I |  | S |  | A |  |  | E |  | I |  | E |  | D |  | D |  | F |  | L |  | E |  | D |  | E |  | 30 |  |
|  | |  | | | | | | | | | | | | | | | | | | | | | | | | | | | | | | | | | | | | | | | | | | | | | | | | | | | | | | | | | | | | | | | | | | | |
| 31 |  |  | S |  | F |  | E |  | A |  | E |  | D |  | I |  | I |  | P |  | F |  |  | F |  | E |  | N |  | E |  | Q |  | A |  | R | ] | S | ⎩ | C |  | I |  |  | P |  | K | ⎩ | H |  | E |  | E | ⎫ | C |  | T | ⎫ | N | ⎱ | D |  | K |  | 60 |  |
|  | |  | | | | | | | | | | | | | | | | | | | | | | | | | | | | | | | | | | | | | | | | | | | | | | | | | | | | | | | | | | | | | | | | | | | |
| 61 |  | ⎫ | H | ⎫ | N | ⎫ | C |  | C |  | R | ⎫ | K | ⎱ | G | ⎱ | L | ⎫ | F | ⎱ | K |  | ⎫ | L | ⎫ | K | ⎫ | C |  | Q | ⎫ | C |  | S |  | T |  | F | ⎫ | D | ⎫ | D |  | ⎫ | E | ⎫ | S | ⎫ | G | ⎱ | Q |  | P |  | T | ⎫ | E |  | R |  | C |  | A |  | 90 |  |
|  | |  | | | | | -41.07 | | | | | | | | | | | | | | | | | | | | | | | | | | | | | | | | | | | | | | | | | | | | | | | | | | | | | | | | | | | |
| 91 |  |  | C | ⎫ | G | ⎫ | R |  | P |  | M |  | G |  | H | ⎫ | Q |  | A |  | I |  |  | E |  | T |  | G |  | L |  | N |  | I |  | F |  | R |  | G | ⎫ | L |  |  | F |  | K |  | G |  | K | ⎫ | K | ⎫ | K | ⎫ | N | ⎫ | K |  | K |  | T |  | 120 |  |
|  | |  | | | | | | | | | | | | | | | | | | | | | | | | | | | | | | | | | | | | | | | | | | | | | | | | | | | | | | | | | | | | | | | | | | | |
| 121 |  | ⎫ | K | ⎫ | G |  | | | | 122 |  | | | | | | | | | | | | | | | | | | | | | | | | | | | | | | | | | | | | | | | | | | | | | | | | | | | | | | | |

Fixed PTMs: Carbamidomethylation [C49 C56 C63 C64 C73 C75 C89 C91 ]   
  
     Unexpected modifications:   Unknown [-41.07]

  

All peaks (147)  Matched peaks (54)  Not matched peaks (93)

  

| Scan | Peak | Mono mass | Mono m/z | Intensity | Charge | Theoretical mass | Ion | Pos | Mass error | PPM error |
| --- | --- | --- | --- | --- | --- | --- | --- | --- | --- | --- |
| 560 | 1 | 8770.1800 | 878.0253 | 55879.57 | 10 | 8769.1871 | C74 | 74 | -9.45e-03 | -1.08 |
| 560 | 2 | 8769.1709 | 798.2046 | 50908.58 | 11 | 8769.1871 | C74 | 74 | -0.0161 | -1.84 |
| 560 | 3 | 8769.1879 | 975.3615 | 29881.14 | 9 | 8769.1871 | C74 | 74 | 7.88e-04 | 0.09 |
| 560 | 4 | 2033.2630 | 678.7616 | 66771.27 | 3 |  |  |  |  |  |
| 560 | 5 | 8811.1999 | 882.1273 | 20839.43 | 10 |  |  |  |  |  |
| 560 | 6 | 8784.1821 | 879.4255 | 21125.39 | 10 |  |  |  |  |  |
| 560 | 7 | 8711.1602 | 872.1233 | 23144.90 | 10 |  |  |  |  |  |
| 560 | 8 | 4443.9075 | 889.7888 | 20007.54 | 5 | 4443.9333 | C36 | 36 | -0.0258 | -5.81 |
| 560 | 9 | 8753.1591 | 876.3232 | 22264.78 | 10 |  |  |  |  |  |
| 560 | 10 | 8753.1656 | 973.5812 | 17122.49 | 9 |  |  |  |  |  |
| 560 | 11 | 8784.1898 | 799.5700 | 19366.52 | 11 |  |  |  |  |  |
| 560 | 12 | 8712.1643 | 969.0255 | 20682.23 | 9 |  |  |  |  |  |
| 560 | 13 | 8726.1731 | 873.6246 | 14946.26 | 10 |  |  |  |  |  |
| 560 | 14 | 8810.1853 | 801.9332 | 19852.18 | 11 |  |  |  |  |  |
| 560 | 15 | 8726.1660 | 970.5813 | 15744.77 | 9 |  |  |  |  |  |
| 560 | 16 | 4414.1031 | 883.8279 | 19870.59 | 5 |  |  |  |  |  |
| 560 | 17 | 8769.1712 | 731.7715 | 18526.96 | 12 | 8769.1871 | C74 | 74 | -0.0159 | -1.81 |
| 560 | 18 | 2528.0752 | 633.0261 | 16227.31 | 4 | 2528.0889 | C20 | 20 | -0.0136 | -5.40 |
| 560 | 19 | 8752.1755 | 796.6596 | 12219.87 | 11 |  |  |  |  |  |
| 560 | 20 | 8710.1386 | 792.8381 | 10180.08 | 11 |  |  |  |  |  |
| 560 | 21 | 1752.7581 | 877.3863 | 19169.29 | 2 | 1752.7671 | C14 | 14 | -9.04e-03 | -5.16 |
| 560 | 22 | 3323.8837 | 665.7840 | 10951.17 | 5 |  |  |  |  |  |
| 560 | 23 | 8696.1556 | 967.2468 | 13091.19 | 9 |  |  |  |  |  |
| 560 | 24 | 4770.0671 | 955.0207 | 10994.26 | 5 | 4770.0923 | C39 | 39 | -0.0252 | -5.28 |
| 560 | 25 | 8784.1941 | 977.0288 | 11661.55 | 9 |  |  |  |  |  |
| 560 | 26 | 3157.4980 | 632.5069 | 10309.64 | 5 | 3157.5153 | C25 | 25 | -0.0174 | -5.51 |
| 560 | 27 | 4443.9093 | 741.6588 | 14193.52 | 6 | 4443.9333 | C36 | 36 | -0.0240 | -5.40 |
| 560 | 28 | 4383.2994 | 731.5572 | 9879.05 | 6 |  |  |  |  |  |
| 560 | 29 | 8813.1881 | 980.2504 | 12558.09 | 9 |  |  |  |  |  |
| 560 | 30 | 3445.5835 | 690.1240 | 9180.42 | 5 | 3445.6046 | C27 | 27 | -0.0211 | -6.12 |
| 560 | 31 | 4325.2708 | 721.8857 | 10705.95 | 6 |  |  |  |  |  |
| 560 | 32 | 3157.4981 | 790.3818 | 12290.83 | 4 | 3157.5153 | C25 | 25 | -0.0172 | -5.46 |
| 560 | 33 | 2471.0545 | 618.7709 | 13586.56 | 4 | 2471.0674 | C19 | 19 | -0.0129 | -5.24 |
| 560 | 34 | 5580.7117 | 931.1259 | 9220.08 | 6 |  |  |  |  |  |
| 560 | 35 | 4055.7873 | 812.1647 | 9078.58 | 5 | 4055.8103 | C32 | 32 | -0.0230 | -5.67 |
| 560 | 36 | 1372.5791 | 687.2968 | 14140.58 | 2 | 1372.5863 | C11 | 11 | -7.18e-03 | -5.23 |
| 560 | 37 | 2528.0734 | 843.6984 | 13081.50 | 3 | 2528.0889 | C20 | 20 | -0.0155 | -6.12 |
| 560 | 38 | 8640.0854 | 961.0168 | 8508.75 | 9 | 8641.0921 | C73 | 73 | -4.41e-03 | -0.51 |
| 560 | 39 | 4384.3007 | 877.8674 | 11039.18 | 5 | 4384.2671 | Z\_DOT39 | 36 | 0.0336 | 7.66 |
| 560 | 40 | 8771.1865 | 1097.4056 | 10261.42 | 8 |  |  |  |  |  |
| 560 | 41 | 8809.1797 | 735.1056 | 7730.82 | 12 |  |  |  |  |  |
| 560 | 42 | 8696.1323 | 870.6205 | 9323.49 | 10 |  |  |  |  |  |
| 560 | 43 | 3445.5851 | 862.4035 | 7953.74 | 4 | 3445.6046 | C27 | 27 | -0.0195 | -5.66 |
| 560 | 44 | 2017.2454 | 673.4224 | 8362.60 | 3 |  |  |  |  |  |
| 560 | 45 | 4554.9361 | 760.1633 | 9191.67 | 6 |  |  |  |  |  |
| 560 | 46 | 4299.8562 | 860.9785 | 10228.93 | 5 | 4299.8798 | C34 | 34 | -0.0237 | -5.50 |
| 560 | 47 | 3323.8877 | 831.9792 | 10734.01 | 4 |  |  |  |  |  |
| 560 | 48 | 1866.7989 | 934.4067 | 13152.62 | 2 | 1866.8101 | C15 | 15 | -0.0111 | -5.97 |
| 560 | 49 | 2788.2280 | 930.4166 | 9364.86 | 3 | 2788.2414 | C22 | 22 | -0.0133 | -4.78 |
| 560 | 50 | 7971.6416 | 886.7452 | 8352.32 | 9 |  |  |  |  |  |
| 560 | 51 | 4170.8123 | 835.1697 | 7553.56 | 5 | 4170.8372 | C33 | 33 | -0.0249 | -5.98 |
| 560 | 52 | 8724.1672 | 794.1134 | 7848.35 | 11 | 8724.1419 | Z\_DOT74 | 1 | 0.0253 | 2.90 |
| 560 | 53 | 4769.0614 | 795.8508 | 7482.60 | 6 |  |  |  |  |  |
| 560 | 54 | 8697.1251 | 791.6550 | 8896.16 | 11 |  |  |  |  |  |
| 560 | 55 | 2788.2254 | 698.0636 | 7528.07 | 4 | 2788.2414 | C22 | 22 | -0.0160 | -5.73 |
| 560 | 56 | 6300.1313 | 901.0260 | 12532.34 | 7 | 6300.1116 | Z\_DOT55 | 20 | 0.0197 | 3.13 |
| 560 | 57 | 6040.5711 | 1007.7691 | 6939.48 | 6 | 6040.5643 | C50 | 50 | 6.74e-03 | 1.12 |
| 560 | 58 | 2943.0682 | 736.7743 | 17181.30 | 4 |  |  |  |  |  |
| 560 | 59 | 1169.7790 | 585.8968 | 9754.66 | 2 |  |  |  |  |  |
| 560 | 60 | 8227.8332 | 915.2110 | 7646.02 | 9 |  |  |  |  |  |
| 560 | 61 | 3029.4051 | 758.3585 | 7120.58 | 4 | 3029.4204 | C24 | 24 | -0.0153 | -5.06 |
| 560 | 62 | 5503.3287 | 918.2287 | 9084.92 | 6 | 5503.3559 | C45 | 45 | -0.0272 | -4.94 |
| 560 | 63 | 2916.3199 | 730.0872 | 9430.36 | 4 | 2916.3363 | C23 | 23 | -0.0164 | -5.64 |
| 560 | 64 | 8698.1367 | 1088.2744 | 6233.40 | 8 |  |  |  |  |  |
| 560 | 65 | 8755.1619 | 1095.4025 | 8417.26 | 8 |  |  |  |  |  |
| 560 | 66 | 1615.6999 | 808.8572 | 8992.91 | 2 | 1615.7082 | C13 | 13 | -8.35e-03 | -5.17 |
| 560 | 67 | 2203.3668 | 735.4629 | 7017.41 | 3 |  |  |  |  |  |
| 560 | 68 | 8739.1546 | 874.9227 | 7357.16 | 10 |  |  |  |  |  |
| 560 | 69 | 2641.1564 | 881.3928 | 6980.31 | 3 | 2641.1730 | C21 | 21 | -0.0165 | -6.26 |
| 560 | 70 | 8040.7024 | 894.4186 | 5599.62 | 9 | 8041.7166 | C68 | 68 | -0.0119 | -1.48 |
| 560 | 71 | 3940.7574 | 789.1588 | 7818.52 | 5 | 3940.7834 | C31 | 31 | -0.0260 | -6.59 |
| 560 | 72 | 4386.8853 | 1097.7286 | 5281.17 | 4 | 4386.9119 | C35 | 35 | -0.0266 | -6.06 |
| 560 | 73 | 7341.2496 | 918.6635 | 5226.56 | 8 | 7340.2578 | C62 | 62 | -0.0105 | -1.44 |
| 560 | 74 | 8640.0917 | 865.0164 | 5900.54 | 10 | 8641.0921 | C73 | 73 | 1.89e-03 | 0.22 |
| 560 | 75 | 997.4598 | 998.4670 | 7594.24 | 1 | 997.4651 | C8 | 8 | -5.29e-03 | -5.30 |
| 560 | 76 | 2641.1582 | 661.2968 | 9832.74 | 4 | 2641.1730 | C21 | 21 | -0.0147 | -5.57 |
| 560 | 77 | 2601.5569 | 651.3965 | 5524.34 | 4 |  |  |  |  |  |
| 560 | 78 | 4900.1084 | 981.0290 | 4920.87 | 5 |  |  |  |  |  |
| 560 | 79 | 8339.8589 | 834.9932 | 5226.31 | 10 |  |  |  |  |  |
| 560 | 80 | 3998.1265 | 667.3617 | 6530.40 | 6 |  |  |  |  |  |
| 560 | 81 | 2288.3972 | 763.8063 | 8180.11 | 3 |  |  |  |  |  |
| 560 | 82 | 5420.1911 | 678.5312 | 8323.37 | 8 |  |  |  |  |  |
| 560 | 83 | 2187.3499 | 730.1239 | 6490.51 | 3 |  |  |  |  |  |
| 560 | 84 | 8668.1428 | 964.1343 | 7954.89 | 9 |  |  |  |  |  |
| 560 | 85 | 3400.8489 | 681.1770 | 10502.99 | 5 |  |  |  |  |  |
| 560 | 86 | 1486.9505 | 744.4825 | 7462.56 | 2 |  |  |  |  |  |
| 560 | 87 | 6039.9773 | 863.8612 | 4609.83 | 7 | 6039.9591 | Z\_DOT53 | 22 | 0.0182 | 3.02 |
| 560 | 88 | 6097.5905 | 872.0916 | 10908.94 | 7 |  |  |  |  |  |
| 560 | 89 | 8170.8177 | 908.8759 | 5365.17 | 9 | 8169.8116 | C69 | 69 | 3.70e-03 | 0.45 |
| 560 | 90 | 4886.4542 | 815.4163 | 4262.27 | 6 |  |  |  |  |  |
| 560 | 91 | 8680.1244 | 1086.0228 | 4768.16 | 8 |  |  |  |  |  |
| 560 | 92 | 2617.5772 | 655.4016 | 8315.18 | 4 |  |  |  |  |  |
| 560 | 93 | 6300.0958 | 788.5192 | 7092.48 | 8 | 6300.1116 | Z\_DOT55 | 20 | -0.0158 | -2.51 |
| 560 | 94 | 8712.1463 | 1090.0256 | 8032.09 | 8 |  |  |  |  |  |
| 560 | 95 | 6256.0860 | 783.0180 | 6440.50 | 8 |  |  |  |  |  |
| 560 | 96 | 6098.5866 | 1017.4384 | 6463.61 | 6 |  |  |  |  |  |
| 560 | 97 | 2671.5862 | 668.9038 | 7173.51 | 4 |  |  |  |  |  |
| 560 | 98 | 6357.1618 | 909.1732 | 4636.01 | 7 | 6357.1330 | Z\_DOT56 | 19 | 0.0288 | 4.53 |
| 560 | 99 | 8753.1557 | 730.4369 | 5017.46 | 12 |  |  |  |  |  |
| 560 | 100 | 8285.8456 | 1036.7380 | 4244.92 | 8 |  |  |  |  |  |
| 560 | 101 | 2342.9593 | 781.9937 | 5150.77 | 3 | 2342.9725 | C18 | 18 | -0.0132 | -5.62 |
| 560 | 102 | 6225.6473 | 890.3855 | 4281.21 | 7 |  |  |  |  |  |
| 560 | 103 | 6539.8097 | 935.2658 | 4548.49 | 7 |  |  |  |  |  |
| 560 | 104 | 8782.1705 | 732.8548 | 7349.16 | 12 |  |  |  |  |  |
| 560 | 105 | 4325.2642 | 618.9022 | 3773.72 | 7 |  |  |  |  |  |
| 560 | 106 | 4443.9064 | 1111.9839 | 5422.01 | 4 | 4443.9333 | C36 | 36 | -0.0269 | -6.05 |
| 560 | 107 | 2470.0507 | 824.3575 | 7953.13 | 3 |  |  |  |  |  |
| 560 | 108 | 8343.8819 | 928.1053 | 5099.92 | 9 |  |  |  |  |  |
| 560 | 109 | 2729.6171 | 910.8796 | 5295.89 | 3 |  |  |  |  |  |
| 560 | 110 | 2721.3679 | 681.3492 | 7670.37 | 4 |  |  |  |  |  |
| 560 | 111 | 8284.8480 | 921.5459 | 6447.68 | 9 | 8283.8545 | C70 | 70 | -8.85e-03 | -1.07 |
| 560 | 112 | 6537.7900 | 818.2310 | 3539.36 | 8 |  |  |  |  |  |
| 560 | 113 | 5447.3265 | 1090.4726 | 4986.93 | 5 | 5446.3345 | C44 | 44 | -0.0103 | -1.90 |
| 560 | 114 | 2203.3660 | 551.8488 | 6634.60 | 4 |  |  |  |  |  |
| 560 | 115 | 2018.2507 | 1010.1326 | 3852.82 | 2 |  |  |  |  |  |
| 560 | 116 | 7912.6032 | 880.1854 | 5987.83 | 9 | 7913.6217 | C67 | 67 | -0.0161 | -2.04 |
| 560 | 117 | 4058.1496 | 677.3655 | 4344.41 | 6 |  |  |  |  |  |
| 560 | 118 | 8099.7364 | 810.9809 | 4826.86 | 10 |  |  |  |  |  |
| 560 | 119 | 5884.4846 | 736.5679 | 25512.35 | 8 |  |  |  |  |  |
| 560 | 120 | 7456.6490 | 933.0884 | 5521.36 | 8 | 7455.6141 | Z\_DOT64 | 11 | 0.0325 | 4.36 |
| 560 | 121 | 4555.9388 | 912.1950 | 4428.47 | 5 |  |  |  |  |  |
| 560 | 122 | 3941.7672 | 986.4491 | 4040.86 | 4 |  |  |  |  |  |
| 560 | 123 | 8226.8270 | 823.6900 | 6474.09 | 10 | 8225.8795 | Z\_DOT70 | 5 | -0.0548 | -6.67 |
| 560 | 124 | 8796.1485 | 880.6221 | 5597.24 | 10 |  |  |  |  |  |
| 560 | 125 | 8226.8149 | 1029.3591 | 6355.44 | 8 | 8225.8795 | Z\_DOT70 | 5 | -0.0669 | -8.14 |
| 560 | 126 | 3682.0031 | 737.4079 | 4570.05 | 5 |  |  |  |  |  |
| 560 | 127 | 600.3807 | 601.3879 | 6595.90 | 1 |  |  |  |  |  |
| 560 | 128 | 856.5692 | 857.5765 | 6726.11 | 1 |  |  |  |  |  |
| 560 | 129 | 1386.8755 | 463.2991 | 4023.06 | 3 |  |  |  |  |  |
| 560 | 130 | 728.4749 | 729.4822 | 7015.22 | 1 |  |  |  |  |  |
| 560 | 131 | 1428.8845 | 477.3021 | 3843.40 | 3 |  |  |  |  |  |
| 560 | 132 | 1486.9510 | 496.6576 | 2064.60 | 3 |  |  |  |  |  |
| 560 | 133 | 486.3384 | 487.3457 | 3504.57 | 1 |  |  |  |  |  |
| 560 | 134 | 1258.5372 | 1259.5445 | 3275.74 | 1 | 1258.5434 | C10 | 10 | -6.17e-03 | -4.90 |
| 560 | 135 | 1372.5791 | 1373.5864 | 2053.28 | 1 | 1372.5863 | C11 | 11 | -7.23e-03 | -5.27 |
| 560 | 136 | 1258.5365 | 630.2755 | 3069.73 | 2 | 1258.5434 | C10 | 10 | -6.85e-03 | -5.45 |
| 560 | 137 | 1185.7973 | 593.9059 | 3027.92 | 2 |  |  |  |  |  |
| 560 | 138 | 1041.6847 | 521.8496 | 2381.59 | 2 |  |  |  |  |  |
| 560 | 139 | 802.7472 | 803.7545 | 9341.73 | 1 |  |  |  |  |  |
| 560 | 140 | 1472.2040 | 737.1093 | 11164.92 | 2 |  |  |  |  |  |
| 560 | 141 | 881.3201 | 882.3273 | 1013.35 | 1 |  |  |  |  |  |
| 560 | 142 | 658.9261 | 659.9334 | 1842.77 | 1 |  |  |  |  |  |
| 560 | 143 | 1429.9272 | 715.9709 | 1866.32 | 2 |  |  |  |  |  |
| 560 | 144 | 984.6640 | 985.6713 | 1748.01 | 1 |  |  |  |  |  |
| 560 | 145 | 1057.7029 | 529.8587 | 1730.58 | 2 |  |  |  |  |  |
| 560 | 146 | 1097.6488 | 1098.6561 | 1113.03 | 1 |  |  |  |  |  |
| 560 | 147 | 1207.7229 | 604.8687 | 1149.12 | 2 |  |  |  |  |  |

  

All proteins /
CsTx-1a\_S1 Cupiennius salei toxin 1 isoform a S1^ACsTx-1a\_S2 Cupiennius salei toxin 1 isoform a S2 /
Proteoform #15
