# Supplementary material for: The Dual Prey-Inactivation Strategy of Spiders—In-Depth Venomic Analysis of Cupiennius salei
Source: Toxins (Basel). 2019 Mar 19;11(3):167. doi: 10.3390/toxins11030167 (PMC6468893; doi:10.3390/toxins11030167)
Supplement: Supplementary file 1 [file toxins-11-00167-s001.zip › Supplementary Dataset EV1/20180328_f2_topdown_OTMS2_EThcD_NL_i02_ms2_proteoform_cutoff_html/prsms/prsm18.html]

Protein-Spectrum-Match for Spectrum #230


All proteins /
CsTx-12a\_S1 Cupiennius salei toxin 12 isoform a S1^ACsTx-12a\_S2 Cupiennius salei toxin 12 isoform a S2 /
Proteoform #25

## Protein-Spectrum-Match #18 for Spectrum #230

|  |  |  |  |  |  |
| --- | --- | --- | --- | --- | --- |
| PrSM ID: | 18 | Scan(s): | 309 | Precursor charge: | 6 |
| Precursor m/z: | 731.9745 | Precursor mass: | 4385.8032 | Proteoform mass: | 4385.7882 |
| # matched peaks: | 31 | # matched fragment ions: | 30 | # unexpected modifications: | 1 |
| E-value: | 2.32e-23 | P-value: | 2.32e-23 | Q-value (Spectral FDR): | 0 |

  

|  |  |  |  |  |  |  |  |  |  |  |  |  |  |  |  |  |  |  |  |  |  |  |  |  |  |  |  |  |  |  |  |  |  |  |  |  |  |  |  |  |  |  |  |  |  |  |  |  |  |  |  |  |  |  |  |  |  |  |  |  |  |  |  |  |  |  |  |  |  |
| --- | --- | --- | --- | --- | --- | --- | --- | --- | --- | --- | --- | --- | --- | --- | --- | --- | --- | --- | --- | --- | --- | --- | --- | --- | --- | --- | --- | --- | --- | --- | --- | --- | --- | --- | --- | --- | --- | --- | --- | --- | --- | --- | --- | --- | --- | --- | --- | --- | --- | --- | --- | --- | --- | --- | --- | --- | --- | --- | --- | --- | --- | --- | --- | --- | --- | --- | --- | --- | --- |
|  | |  | | | | | | | | | | | | | | | | | | | | | | | | | | | | | | | | | | | | | | | | | | | | | | | | | | | | | | | | | | | | | | | | | | | |
| 1 |  |  | M |  | K |  | V |  | L |  | V |  | I |  | C |  | A |  | V |  | L |  |  | F |  | L |  | T |  | I |  | F |  | S |  | N |  | S |  | S |  | A |  |  | E |  | T |  | E |  | D |  | D |  | F |  | L |  | E |  | D |  | E |  | 30 |  |
|  | |  | | | | | | | | | | | | | | | | | | | | | | | | | | | | | | | | | | | | | | | | | | | | | | | | | | | | | | | | | | | | | | | | | | | |
| 31 |  |  | S |  | F |  | E |  | A |  | D |  | D |  | V |  | I |  | P |  | F |  |  | L |  | A |  | R |  | E |  | Q |  | V |  | R | ] | S |  | D |  | C |  |  | T |  | L | ⎱ | R | ⎱ | N |  | H | ⎫ | D | ⎫ | C | ⎫ | T | ⎫ | D | ⎱ | D |  | 60 |  |
|  | |  | | | | | | | | | | | | | | | | | 15.98 | | | | | | | | | | | | | | | | | | | | | | | | | | | | | | | | | | | | | | | | | | | | | | | |
| 61 |  | ⎱ | R |  | H |  | S | ⎫ | C |  | C | ⎫ | R | ⎱ | S | ⎱ | K | ⎫ | M |  | F |  |  | K | ⎱ | D | ⎱ | V | ⎫ | C | ⎫ | K | ⎫ | C | ⎫ | F | ⎫ | Y |  | P | ⎫ | S |  | ⎫ | Q | [ | R |  | S |  | D |  | T |  | A |  | R |  | A |  | K |  | K |  | 90 |  |
|  | |  | | | | | | | | | | | | | | | | | | | | | | | | | | | | | | | | | | | | | | | | | | | | | | | | | | | | | | | | | | | | | | | | | | | |
| 91 |  |  | E |  | L |  | C |  | T |  | C |  | Q |  | Q |  | D |  | K |  | H |  |  | L |  | K |  | F |  | I |  | E |  | K |  | G |  | L |  | Q |  | K |  |  | A |  | K |  | V |  | L |  | V |  | A |  | G |  | | 117 |  | | | | | |

Fixed PTMs: Carbamidomethylation [C50 C57 C64 C65 C74 C76 ]   
  
     Unexpected modifications:   Unknown [15.98]

  

All peaks (73)  Matched peaks (31)  Not matched peaks (42)

  

| Scan | Peak | Mono mass | Mono m/z | Intensity | Charge | Theoretical mass | Ion | Pos | Mass error | PPM error |
| --- | --- | --- | --- | --- | --- | --- | --- | --- | --- | --- |
| 309 | 1 | 4328.7667 | 866.7606 | 1226529.58 | 5 |  |  |  |  |  |
| 309 | 2 | 2193.3894 | 732.1371 | 4018922.45 | 3 |  |  |  |  |  |
| 309 | 3 | 4239.7210 | 848.9515 | 298749.70 | 5 |  |  |  |  |  |
| 309 | 4 | 4055.6326 | 812.1338 | 187501.45 | 5 |  |  |  |  |  |
| 309 | 5 | 4369.7704 | 874.9614 | 141546.98 | 5 |  |  |  |  |  |
| 309 | 6 | 4256.7431 | 852.3559 | 146398.65 | 5 | 4256.7456 | C33 | 33 | -2.52e-03 | -0.59 |
| 309 | 7 | 4368.7872 | 729.1385 | 159772.53 | 6 |  |  |  |  |  |
| 309 | 8 | 3602.4974 | 901.6316 | 132783.12 | 4 | 3602.4984 | C28 | 28 | -9.81e-04 | -0.27 |
| 309 | 9 | 2309.9976 | 771.0065 | 150648.96 | 3 |  |  |  |  |  |
| 309 | 10 | 3100.2793 | 776.0771 | 120719.61 | 4 | 3100.2774 | C24 | 24 | 1.91e-03 | 0.62 |
| 309 | 11 | 3909.5972 | 978.4066 | 89462.79 | 4 | 3909.5975 | C30 | 30 | -2.26e-04 | -0.06 |
| 309 | 12 | 3762.5284 | 941.6394 | 66279.34 | 4 | 3762.5291 | C29 | 29 | -6.48e-04 | -0.17 |
| 309 | 13 | 2549.9890 | 851.0036 | 74662.43 | 3 | 2549.9965 | C20 | 20 | -7.51e-03 | -2.95 |
| 309 | 14 | 4077.7071 | 816.5487 | 63157.34 | 5 |  |  |  |  |  |
| 309 | 15 | 2462.9553 | 821.9924 | 75721.62 | 3 | 2462.9644 | C19 | 19 | -9.17e-03 | -3.72 |
| 309 | 16 | 1986.7963 | 994.4054 | 76414.48 | 2 | 1986.8020 | C16 | 16 | -5.68e-03 | -2.86 |
| 309 | 17 | 4296.7937 | 860.3660 | 53195.33 | 5 |  |  |  |  |  |
| 309 | 18 | 4077.7105 | 1020.4349 | 54278.27 | 4 |  |  |  |  |  |
| 309 | 19 | 877.3583 | 878.3656 | 174659.17 | 1 |  |  |  |  |  |
| 309 | 20 | 2911.2457 | 728.8187 | 108303.44 | 4 |  |  |  |  |  |
| 309 | 21 | 4279.7701 | 856.9613 | 64813.73 | 5 |  |  |  |  |  |
| 309 | 22 | 3215.3059 | 804.8338 | 52018.94 | 4 | 3215.3044 | C25 | 25 | 1.58e-03 | 0.49 |
| 309 | 23 | 3314.3736 | 829.6007 | 41679.28 | 4 | 3314.3728 | C26 | 26 | 8.03e-04 | 0.24 |
| 309 | 24 | 4342.7672 | 869.5607 | 51945.48 | 5 |  |  |  |  |  |
| 309 | 25 | 4321.7987 | 721.3071 | 34092.67 | 6 |  |  |  |  |  |
| 309 | 26 | 731.3012 | 732.3084 | 1953219.08 | 1 |  |  |  |  |  |
| 309 | 27 | 4222.7053 | 845.5483 | 36606.82 | 5 |  |  |  |  |  |
| 309 | 28 | 3474.4033 | 869.6081 | 60613.62 | 4 | 3474.4034 | C27 | 27 | -1.36e-04 | -0.04 |
| 309 | 29 | 4169.7085 | 834.9490 | 34611.72 | 5 | 4169.7136 | C32 | 32 | -5.09e-03 | -1.22 |
| 309 | 30 | 2895.2171 | 966.0796 | 29252.36 | 3 | 2895.2130 | Z\_DOT22 | 12 | 4.07e-03 | 1.41 |
| 309 | 31 | 4311.7516 | 863.3576 | 34937.72 | 5 |  |  |  |  |  |
| 309 | 32 | 1491.5773 | 746.7959 | 35912.45 | 2 | 1491.5830 | C12 | 12 | -5.72e-03 | -3.84 |
| 309 | 33 | 4183.7266 | 698.2950 | 23964.29 | 6 |  |  |  |  |  |
| 309 | 34 | 2678.0826 | 893.7015 | 25548.86 | 3 | 2678.0914 | C21 | 21 | -8.84e-03 | -3.30 |
| 309 | 35 | 4152.6845 | 831.5442 | 20407.32 | 5 |  |  |  |  |  |
| 309 | 36 | 4383.7902 | 731.6390 | 1640566.01 | 6 |  |  |  |  |  |
| 309 | 37 | 1923.8398 | 962.9272 | 30341.41 | 2 | 1923.8316 | Z\_DOT15 | 19 | 8.17e-03 | 4.25 |
| 309 | 38 | 3637.4462 | 910.3688 | 23695.29 | 4 | 3637.4471 | Z\_DOT28 | 6 | -9.30e-04 | -0.26 |
| 309 | 39 | 3793.5467 | 949.3940 | 19900.28 | 4 | 3793.5482 | Z\_DOT29 | 5 | -1.47e-03 | -0.39 |
| 309 | 40 | 1836.8074 | 919.4110 | 26087.83 | 2 | 1836.7996 | Z\_DOT14 | 20 | 7.85e-03 | 4.27 |
| 309 | 41 | 3892.5683 | 779.5209 | 18102.57 | 5 |  |  |  |  |  |
| 309 | 42 | 2780.1917 | 927.7378 | 20202.32 | 3 | 2780.1861 | Z\_DOT21 | 13 | 5.62e-03 | 2.02 |
| 309 | 43 | 1606.6054 | 804.3100 | 33677.12 | 2 | 1606.6100 | C13 | 13 | -4.59e-03 | -2.86 |
| 309 | 44 | 2149.9718 | 717.6645 | 19281.12 | 3 |  |  |  |  |  |
| 309 | 45 | 2306.8562 | 1154.4354 | 19378.88 | 2 | 2306.8633 | C18 | 18 | -7.17e-03 | -3.11 |
| 309 | 46 | 3922.6507 | 981.6700 | 17587.77 | 4 |  |  |  |  |  |
| 309 | 47 | 4328.7588 | 1083.1970 | 18257.55 | 4 |  |  |  |  |  |
| 309 | 48 | 3110.2834 | 1037.7684 | 14649.49 | 3 |  |  |  |  |  |
| 309 | 49 | 3864.5782 | 967.1518 | 13960.71 | 4 |  |  |  |  |  |
| 309 | 50 | 4183.7291 | 837.7531 | 13863.12 | 5 |  |  |  |  |  |
| 309 | 51 | 4351.7606 | 726.3007 | 13381.74 | 6 |  |  |  |  |  |
| 309 | 52 | 1376.5520 | 689.2833 | 20392.29 | 2 | 1376.5561 | C11 | 11 | -4.05e-03 | -2.94 |
| 309 | 53 | 694.2943 | 695.3016 | 9170.60 | 1 |  |  |  |  |  |
| 309 | 54 | 1275.5047 | 638.7596 | 8110.21 | 2 | 1275.5084 | C10 | 10 | -3.74e-03 | -2.93 |
| 309 | 55 | 330.1534 | 331.1607 | 13505.16 | 1 |  |  |  |  |  |
| 309 | 56 | 1115.4748 | 558.7447 | 8934.43 | 2 | 1115.4778 | C9 | 9 | -2.99e-03 | -2.68 |
| 309 | 57 | 849.3455 | 850.3528 | 23637.51 | 1 |  |  |  |  |  |
| 309 | 58 | 1475.5524 | 738.7835 | 46240.57 | 2 |  |  |  |  |  |
| 309 | 59 | 1000.4482 | 501.2314 | 9273.68 | 2 | 1000.4508 | C8 | 8 | -2.66e-03 | -2.66 |
| 309 | 60 | 749.3465 | 750.3538 | 13760.42 | 1 | 749.3490 | C6 | 6 | -2.43e-03 | -3.24 |
| 309 | 61 | 822.3890 | 823.3963 | 4712.43 | 1 |  |  |  |  |  |
| 309 | 62 | 1286.5149 | 1287.5222 | 4555.28 | 1 | 1286.5186 | Z\_DOT10 | 24 | -3.75e-03 | -2.91 |
| 309 | 63 | 1171.4889 | 1172.4962 | 3632.16 | 1 | 1171.4917 | Z\_DOT9 | 25 | -2.81e-03 | -2.40 |
| 309 | 64 | 493.2163 | 494.2235 | 4386.75 | 1 |  |  |  |  |  |
| 309 | 65 | 593.2464 | 594.2537 | 3425.13 | 1 | 593.2479 | C5 | 5 | -1.48e-03 | -2.49 |
| 309 | 66 | 361.1268 | 362.1341 | 3433.46 | 1 |  |  |  |  |  |
| 309 | 67 | 459.1872 | 460.1944 | 2782.93 | 1 |  |  |  |  |  |
| 309 | 68 | 576.2201 | 577.2274 | 3039.96 | 1 |  |  |  |  |  |
| 309 | 69 | 1098.4478 | 550.2312 | 2510.59 | 2 |  |  |  |  |  |
| 309 | 70 | 1242.5259 | 1243.5331 | 1941.66 | 1 |  |  |  |  |  |
| 309 | 71 | 1324.6078 | 663.3112 | 1754.51 | 2 |  |  |  |  |  |
| 309 | 72 | 1115.4759 | 1116.4832 | 1445.73 | 1 | 1115.4778 | C9 | 9 | -1.82e-03 | -1.63 |
| 309 | 73 | 1356.5804 | 679.2975 | 1252.15 | 2 |  |  |  |  |  |

  

All proteins /
CsTx-12a\_S1 Cupiennius salei toxin 12 isoform a S1^ACsTx-12a\_S2 Cupiennius salei toxin 12 isoform a S2 /
Proteoform #25
