# Supplementary material for: The Dual Prey-Inactivation Strategy of Spiders—In-Depth Venomic Analysis of Cupiennius salei
Source: Toxins (Basel). 2019 Mar 19;11(3):167. doi: 10.3390/toxins11030167 (PMC6468893; doi:10.3390/toxins11030167)
Supplement: Supplementary file 1 [file toxins-11-00167-s001.zip › Supplementary Dataset EV1/20180328_f2_topdown_OTMS2_EThcD_NL_i02_ms2_proteoform_cutoff_html/prsms/prsm180.html]

Protein-Spectrum-Match for Spectrum #419


All proteins /
CsTx-1a\_S1 Cupiennius salei toxin 1 isoform a S1^ACsTx-1a\_S2 Cupiennius salei toxin 1 isoform a S2 /
Proteoform #15

## Protein-Spectrum-Match #180 for Spectrum #419

|  |  |  |  |  |  |
| --- | --- | --- | --- | --- | --- |
| PrSM ID: | 180 | Scan(s): | 561 | Precursor charge: | 12 |
| Precursor m/z: | 736.5263 | Precursor mass: | 8826.2283 | Proteoform mass: | 8826.1983 |
| # matched peaks: | 47 | # matched fragment ions: | 36 | # unexpected modifications: | 1 |
| E-value: | 4.90e-29 | P-value: | 4.90e-29 | Q-value (Spectral FDR): | 0 |

  

|  |  |  |  |  |  |  |  |  |  |  |  |  |  |  |  |  |  |  |  |  |  |  |  |  |  |  |  |  |  |  |  |  |  |  |  |  |  |  |  |  |  |  |  |  |  |  |  |  |  |  |  |  |  |  |  |  |  |  |  |  |  |  |  |  |  |  |  |  |  |
| --- | --- | --- | --- | --- | --- | --- | --- | --- | --- | --- | --- | --- | --- | --- | --- | --- | --- | --- | --- | --- | --- | --- | --- | --- | --- | --- | --- | --- | --- | --- | --- | --- | --- | --- | --- | --- | --- | --- | --- | --- | --- | --- | --- | --- | --- | --- | --- | --- | --- | --- | --- | --- | --- | --- | --- | --- | --- | --- | --- | --- | --- | --- | --- | --- | --- | --- | --- | --- | --- |
|  | |  | | | | | | | | | | | | | | | | | | | | | | | | | | | | | | | | | | | | | | | | | | | | | | | | | | | | | | | | | | | | | | | | | | | |
| 1 |  |  | M |  | K |  | V |  | L |  | I |  | I |  | S |  | A |  | V |  | L |  |  | F |  | I |  | T |  | I |  | F |  | S |  | N |  | I |  | S |  | A |  |  | E |  | I |  | E |  | D |  | D |  | F |  | L |  | E |  | D |  | E |  | 30 |  |
|  | |  | | | | | | | | | | | | | | | | | | | | | | | | | | | | | | | | | | | | | | | | | | | | | | | | | | | | | | | | | | | | | | | | | | | |
| 31 |  |  | S |  | F |  | E |  | A |  | E |  | D |  | I |  | I |  | P |  | F |  |  | F |  | E |  | N |  | E |  | Q |  | A |  | R | ] | S | ⎩ | C |  | I |  |  | P |  | K | ⎩ | H |  | E |  | E | ⎫ | C | ⎫ | T | ⎫ | N | ⎱ | D |  | K |  | 60 |  |
|  | |  | | | | | | | | | | | | | | | | | | | | | | | | | | | | | | | | | | | | | | | | | | | | | | | | | | | | | | | | | | | | | | | | | | | |
| 61 |  |  | H | ⎫ | N | ⎫ | C | ⎫ | C |  | R |  | K | ⎫ | G | ⎫ | L |  | F | ⎱ | K |  |  | L |  | K | ⎫ | C |  | Q | ⎫ | C |  | S |  | T |  | F | ⎫ | D |  | D |  | ⎫ | E | ⎫ | S | ⎫ | G | ⎱ | Q |  | P |  | T | ⎫ | E | ⎫ | R |  | C |  | A |  | 90 |  |
|  | |  | | | | | 14.96 | | | | | | | | | | | | | | | | | | | | | | | | | | | | | | | | | | | | | | | | | | | | | | | | | | | | | | | | | | | |
| 91 |  |  | C |  | G | ⎱ | R |  | P |  | M | ⎫ | G |  | H | ⎫ | Q | ⎫ | A |  | I |  |  | E | ⎫ | T | ⎫ | G |  | L |  | N |  | I |  | F |  | R |  | G |  | L |  |  | F |  | K |  | G |  | K |  | K | ⎫ | K | ⎫ | N | ⎫ | K | ⎫ | K |  | T |  | 120 |  |
|  | |  | | | | | | | | | | | | | | | | | | | | | | | | | | | | | | | | | | | | | | | | | | | | | | | | | | | | | | | | | | | | | | | | | | | |
| 121 |  | ⎫ | K | [ | G |  | | | | 122 |  | | | | | | | | | | | | | | | | | | | | | | | | | | | | | | | | | | | | | | | | | | | | | | | | | | | | | | | |

Fixed PTMs: Carbamidomethylation [C49 C56 C63 C64 C73 C75 C89 C91 ]   
  
     Unexpected modifications:   Unknown [14.96]

  

All peaks (147)  Matched peaks (47)  Not matched peaks (100)

  

| Scan | Peak | Mono mass | Mono m/z | Intensity | Charge | Theoretical mass | Ion | Pos | Mass error | PPM error |
| --- | --- | --- | --- | --- | --- | --- | --- | --- | --- | --- |
| 561 | 1 | 8769.1772 | 877.9250 | 78954.30 | 10 |  |  |  |  |  |
| 561 | 2 | 8769.1814 | 975.3608 | 62332.48 | 9 |  |  |  |  |  |
| 561 | 3 | 8769.1743 | 798.2049 | 39999.48 | 11 |  |  |  |  |  |
| 561 | 4 | 8811.1950 | 882.1268 | 38281.73 | 10 |  |  |  |  |  |
| 561 | 5 | 8770.1917 | 1097.2812 | 29556.92 | 8 |  |  |  |  |  |
| 561 | 6 | 2203.3687 | 735.4635 | 52924.81 | 3 |  |  |  |  |  |
| 561 | 7 | 8712.1579 | 969.0248 | 29621.42 | 9 |  |  |  |  |  |
| 561 | 8 | 8754.1696 | 973.6928 | 33095.83 | 9 |  |  |  |  |  |
| 561 | 9 | 8753.1750 | 876.3248 | 26076.70 | 10 |  |  |  |  |  |
| 561 | 10 | 8812.1922 | 980.1397 | 27121.56 | 9 |  |  |  |  |  |
| 561 | 11 | 4443.9087 | 889.7890 | 23204.82 | 5 | 4443.9333 | C36 | 36 | -0.0246 | -5.53 |
| 561 | 12 | 8725.1563 | 970.4691 | 23062.40 | 9 |  |  |  |  |  |
| 561 | 13 | 4413.6026 | 883.7278 | 43958.17 | 5 |  |  |  |  |  |
| 561 | 14 | 8784.1914 | 977.0285 | 21471.09 | 9 |  |  |  |  |  |
| 561 | 15 | 8711.1498 | 1089.9010 | 19063.77 | 8 |  |  |  |  |  |
| 561 | 16 | 8811.1930 | 802.0248 | 22323.18 | 11 |  |  |  |  |  |
| 561 | 17 | 8784.1882 | 879.4261 | 26700.22 | 10 |  |  |  |  |  |
| 561 | 18 | 8711.1518 | 872.1225 | 19664.21 | 10 |  |  |  |  |  |
| 561 | 19 | 8754.1615 | 1095.2775 | 20404.73 | 8 |  |  |  |  |  |
| 561 | 20 | 8726.1707 | 1091.7786 | 16838.62 | 8 |  |  |  |  |  |
| 561 | 21 | 8696.1240 | 967.2433 | 16505.85 | 9 | 8697.1193 | C73 | 73 | 7.11e-03 | 0.82 |
| 561 | 22 | 735.4220 | 736.4293 | 43804.95 | 1 |  |  |  |  |  |
| 561 | 23 | 3157.4974 | 790.3816 | 13984.57 | 4 | 3157.5153 | C25 | 25 | -0.0180 | -5.69 |
| 561 | 24 | 8782.1771 | 799.3870 | 13264.62 | 11 |  |  |  |  |  |
| 561 | 25 | 4770.0647 | 955.0202 | 14285.38 | 5 | 4770.0923 | C39 | 39 | -0.0276 | -5.79 |
| 561 | 26 | 1752.7588 | 877.3867 | 20919.86 | 2 | 1752.7671 | C14 | 14 | -8.30e-03 | -4.74 |
| 561 | 27 | 8724.1571 | 873.4230 | 12558.57 | 10 | 8723.1476 | Z\_DOT73 | 1 | 7.11e-03 | 0.82 |
| 561 | 28 | 8695.1380 | 870.5211 | 12798.21 | 10 |  |  |  |  |  |
| 561 | 29 | 8737.1783 | 874.7251 | 10427.48 | 10 |  |  |  |  |  |
| 561 | 30 | 5579.7089 | 930.9588 | 10554.72 | 6 |  |  |  |  |  |
| 561 | 31 | 8812.1927 | 1102.5314 | 13982.02 | 8 |  |  |  |  |  |
| 561 | 32 | 8784.1937 | 1099.0315 | 11137.41 | 8 |  |  |  |  |  |
| 561 | 33 | 2788.2271 | 930.4163 | 12155.66 | 3 | 2788.2414 | C22 | 22 | -0.0143 | -5.11 |
| 561 | 34 | 8752.1755 | 796.6596 | 13457.68 | 11 |  |  |  |  |  |
| 561 | 35 | 7076.4530 | 885.5639 | 8186.84 | 8 |  |  |  |  |  |
| 561 | 36 | 8340.8657 | 927.7701 | 10078.51 | 9 | 8339.8817 | C70 | 70 | -0.0184 | -2.20 |
| 561 | 37 | 3323.8854 | 831.9786 | 14206.71 | 4 |  |  |  |  |  |
| 561 | 38 | 8738.1630 | 971.9143 | 12640.41 | 9 |  |  |  |  |  |
| 561 | 39 | 4325.2711 | 721.8858 | 11760.49 | 6 |  |  |  |  |  |
| 561 | 40 | 2288.3968 | 763.8062 | 11600.24 | 3 |  |  |  |  |  |
| 561 | 41 | 3445.5819 | 862.4028 | 9316.93 | 4 | 3445.6046 | C27 | 27 | -0.0227 | -6.58 |
| 561 | 42 | 2617.5777 | 655.4017 | 8523.78 | 4 |  |  |  |  |  |
| 561 | 43 | 1866.8001 | 934.4073 | 13090.73 | 2 | 1866.8101 | C15 | 15 | -9.92e-03 | -5.31 |
| 561 | 44 | 8696.1371 | 1088.0244 | 11143.62 | 8 | 8697.1193 | C73 | 73 | 0.0202 | 2.32 |
| 561 | 45 | 4170.8120 | 835.1697 | 7751.36 | 5 | 4170.8372 | C33 | 33 | -0.0253 | -6.05 |
| 561 | 46 | 8739.1648 | 1093.4029 | 9572.58 | 8 |  |  |  |  |  |
| 561 | 47 | 4443.9113 | 1111.9851 | 8125.19 | 4 | 4443.9333 | C36 | 36 | -0.0220 | -4.95 |
| 561 | 48 | 8640.0965 | 1081.0193 | 7741.84 | 8 |  |  |  |  |  |
| 561 | 49 | 2528.0734 | 843.6984 | 8184.49 | 3 | 2528.0889 | C20 | 20 | -0.0155 | -6.12 |
| 561 | 50 | 2943.4016 | 736.8577 | 12433.17 | 4 |  |  |  |  |  |
| 561 | 51 | 2471.0547 | 824.6922 | 8099.43 | 3 | 2471.0674 | C19 | 19 | -0.0127 | -5.14 |
| 561 | 52 | 5926.9011 | 847.7074 | 7541.64 | 7 |  |  |  |  |  |
| 561 | 53 | 4299.8574 | 860.9788 | 7047.38 | 5 | 4299.8798 | C34 | 34 | -0.0224 | -5.22 |
| 561 | 54 | 2943.0735 | 982.0318 | 12437.37 | 3 |  |  |  |  |  |
| 561 | 55 | 3683.8345 | 737.7742 | 17159.48 | 5 |  |  |  |  |  |
| 561 | 56 | 3323.8828 | 665.7838 | 6655.87 | 5 | 3323.8502 | Z\_DOT29 | 45 | 0.0326 | 9.79 |
| 561 | 57 | 7341.2544 | 918.6641 | 7517.84 | 8 |  |  |  |  |  |
| 561 | 58 | 8669.1429 | 1084.6501 | 7287.94 | 8 |  |  |  |  |  |
| 561 | 59 | 4384.3049 | 877.8682 | 12308.58 | 5 |  |  |  |  |  |
| 561 | 60 | 2187.3504 | 730.1241 | 7528.51 | 3 |  |  |  |  |  |
| 561 | 61 | 6097.5857 | 1017.2716 | 7271.11 | 6 | 6096.5915 | C50 | 50 | -8.10e-03 | -1.33 |
| 561 | 62 | 8098.7463 | 900.8680 | 8161.40 | 9 | 8097.7438 | C68 | 68 | 1.28e-04 | 0.02 |
| 561 | 63 | 5503.3298 | 918.2289 | 10900.55 | 6 | 5503.3559 | C45 | 45 | -0.0261 | -4.74 |
| 561 | 64 | 4058.1496 | 812.6372 | 7578.23 | 5 |  |  |  |  |  |
| 561 | 65 | 2729.6200 | 910.8806 | 7927.69 | 3 |  |  |  |  |  |
| 561 | 66 | 3998.1265 | 667.3617 | 7206.62 | 6 |  |  |  |  |  |
| 561 | 67 | 5926.9058 | 988.8249 | 6796.53 | 6 |  |  |  |  |  |
| 561 | 68 | 8099.7313 | 1013.4737 | 5648.71 | 8 |  |  |  |  |  |
| 561 | 69 | 1766.0426 | 884.0286 | 16426.84 | 2 |  |  |  |  |  |
| 561 | 70 | 8342.8700 | 1043.8660 | 6689.21 | 8 |  |  |  |  |  |
| 561 | 71 | 1372.5794 | 687.2970 | 7791.59 | 2 | 1372.5863 | C11 | 11 | -6.94e-03 | -5.05 |
| 561 | 72 | 2026.8298 | 1014.4222 | 7064.07 | 2 | 2026.8407 | C16 | 16 | -0.0109 | -5.38 |
| 561 | 73 | 5504.3269 | 1101.8726 | 6784.33 | 5 | 5503.3559 | C45 | 45 | -0.0314 | -5.71 |
| 561 | 74 | 1169.7790 | 585.8968 | 12499.03 | 2 |  |  |  |  |  |
| 561 | 75 | 8681.1183 | 965.5760 | 9670.15 | 9 |  |  |  |  |  |
| 561 | 76 | 8340.8660 | 835.0939 | 6256.28 | 10 | 8339.8817 | C70 | 70 | -0.0180 | -2.16 |
| 561 | 77 | 8641.0725 | 961.1264 | 7072.01 | 9 |  |  |  |  |  |
| 561 | 78 | 718.3954 | 719.4027 | 13829.14 | 1 |  |  |  |  |  |
| 561 | 79 | 3157.4994 | 1053.5071 | 6430.79 | 3 | 3157.5153 | C25 | 25 | -0.0160 | -5.05 |
| 561 | 80 | 4414.1004 | 1104.5324 | 6966.60 | 4 |  |  |  |  |  |
| 561 | 81 | 8668.1481 | 964.1348 | 6767.87 | 9 |  |  |  |  |  |
| 561 | 82 | 8795.2163 | 978.2535 | 8424.35 | 9 |  |  |  |  |  |
| 561 | 83 | 2671.5870 | 668.9040 | 7845.53 | 4 |  |  |  |  |  |
| 561 | 84 | 6225.6438 | 1038.6146 | 5684.82 | 6 | 6224.6500 | C51 | 51 | -8.60e-03 | -1.38 |
| 561 | 85 | 8225.8389 | 1029.2371 | 7889.27 | 8 | 8224.8852 | Z\_DOT69 | 5 | -0.0486 | -5.91 |
| 561 | 85 | 8225.8389 | 1029.2371 | 7889.27 | 8 | 8225.8387 | C69 | 69 | 1.95e-04 | 0.02 |
| 561 | 86 | 6870.4032 | 982.4935 | 12732.98 | 7 |  |  |  |  |  |
| 561 | 87 | 856.5692 | 857.5765 | 8811.27 | 1 |  |  |  |  |  |
| 561 | 88 | 1486.9510 | 744.4828 | 6932.48 | 2 |  |  |  |  |  |
| 561 | 89 | 4383.3024 | 731.5577 | 6199.47 | 6 | 4383.2728 | Z\_DOT38 | 36 | 0.0296 | 6.74 |
| 561 | 90 | 4386.8882 | 1097.7293 | 5390.70 | 4 | 4386.9119 | C35 | 35 | -0.0236 | -5.39 |
| 561 | 91 | 7456.6372 | 933.0869 | 6434.49 | 8 |  |  |  |  |  |
| 561 | 92 | 6097.5901 | 872.0916 | 8307.37 | 7 | 6096.5915 | C50 | 50 | -3.69e-03 | -0.61 |
| 561 | 93 | 4554.9387 | 911.9950 | 4371.22 | 5 |  |  |  |  |  |
| 561 | 94 | 8041.7080 | 1006.2208 | 5879.44 | 8 |  |  |  |  |  |
| 561 | 95 | 3940.7623 | 986.1978 | 7403.72 | 4 | 3940.7834 | C31 | 31 | -0.0211 | -5.35 |
| 561 | 96 | 8754.1751 | 1251.6037 | 5469.98 | 7 |  |  |  |  |  |
| 561 | 97 | 8282.8325 | 1036.3613 | 7169.00 | 8 |  |  |  |  |  |
| 561 | 98 | 997.4593 | 998.4666 | 7957.75 | 1 | 997.4651 | C8 | 8 | -5.72e-03 | -5.73 |
| 561 | 99 | 2872.3082 | 958.4434 | 6524.76 | 3 |  |  |  |  |  |
| 561 | 100 | 7740.7371 | 861.0892 | 4701.63 | 9 |  |  |  |  |  |
| 561 | 101 | 8226.8261 | 823.6899 | 6947.85 | 10 | 8225.8387 | C69 | 69 | -0.0150 | -1.82 |
| 561 | 102 | 8655.1058 | 1082.8955 | 7461.65 | 8 |  |  |  |  |  |
| 561 | 103 | 4528.3609 | 906.6795 | 4295.88 | 5 |  |  |  |  |  |
| 561 | 104 | 2601.5594 | 651.3971 | 3970.19 | 4 |  |  |  |  |  |
| 561 | 105 | 3940.7617 | 789.1596 | 4669.06 | 5 | 3940.7834 | C31 | 31 | -0.0217 | -5.50 |
| 561 | 106 | 8226.8194 | 915.0983 | 5249.49 | 9 | 8225.8387 | C69 | 69 | -0.0217 | -2.64 |
| 561 | 107 | 5902.5063 | 984.7583 | 4085.91 | 6 | 5902.5111 | C48 | 48 | -4.84e-03 | -0.82 |
| 561 | 108 | 2601.5621 | 868.1946 | 6174.46 | 3 |  |  |  |  |  |
| 561 | 109 | 3450.9341 | 691.1941 | 3541.08 | 5 |  |  |  |  |  |
| 561 | 110 | 8241.9241 | 916.7766 | 7113.16 | 9 |  |  |  |  |  |
| 561 | 111 | 6640.8549 | 831.1141 | 4130.09 | 8 |  |  |  |  |  |
| 561 | 112 | 8467.9578 | 941.8915 | 5771.73 | 9 | 8467.9766 | C71 | 71 | -0.0188 | -2.22 |
| 561 | 113 | 4325.2663 | 866.0605 | 6009.91 | 5 |  |  |  |  |  |
| 561 | 114 | 6224.6488 | 890.2428 | 6232.39 | 7 | 6224.6500 | C51 | 51 | -1.20e-03 | -0.19 |
| 561 | 115 | 6039.9939 | 1007.6729 | 4136.71 | 6 | 6038.9648 | Z\_DOT52 | 22 | 0.0268 | 4.43 |
| 561 | 116 | 6871.4090 | 859.9334 | 7201.44 | 8 |  |  |  |  |  |
| 561 | 117 | 4899.1084 | 980.8289 | 5616.99 | 5 | 4899.1349 | C40 | 40 | -0.0266 | -5.42 |
| 561 | 118 | 7455.6498 | 1066.1001 | 4933.98 | 7 | 7454.6198 | Z\_DOT63 | 11 | 0.0277 | 3.71 |
| 561 | 119 | 8712.1546 | 1245.6008 | 3614.34 | 7 |  |  |  |  |  |
| 561 | 120 | 6622.6499 | 736.8573 | 15593.33 | 9 |  |  |  |  |  |
| 561 | 121 | 7912.6082 | 1131.3799 | 3497.82 | 7 |  |  |  |  |  |
| 561 | 122 | 8073.9776 | 898.1159 | 5729.29 | 9 |  |  |  |  |  |
| 561 | 123 | 7683.7335 | 854.7554 | 3351.58 | 9 |  |  |  |  |  |
| 561 | 124 | 8227.8405 | 1176.4131 | 4124.90 | 7 |  |  |  |  |  |
| 561 | 125 | 6870.4069 | 764.3858 | 3631.00 | 9 |  |  |  |  |  |
| 561 | 126 | 6638.8505 | 949.4145 | 4282.20 | 7 | 6638.8615 | C55 | 55 | -0.0110 | -1.66 |
| 561 | 127 | 2017.2460 | 673.4226 | 4621.19 | 3 |  |  |  |  |  |
| 561 | 128 | 6039.9837 | 863.8621 | 5602.43 | 7 | 6038.9648 | Z\_DOT52 | 22 | 0.0166 | 2.75 |
| 561 | 129 | 6538.8129 | 935.1234 | 5989.55 | 7 | 6537.8138 | C54 | 54 | -3.22e-03 | -0.49 |
| 561 | 130 | 1428.8854 | 477.3024 | 4069.97 | 3 |  |  |  |  |  |
| 561 | 131 | 600.3808 | 601.3881 | 4650.77 | 1 |  |  |  |  |  |
| 561 | 132 | 802.5643 | 803.5716 | 4633.25 | 1 |  |  |  |  |  |
| 561 | 133 | 1185.7980 | 593.9063 | 2968.00 | 2 |  |  |  |  |  |
| 561 | 134 | 1258.5370 | 1259.5442 | 3120.36 | 1 | 1258.5434 | C10 | 10 | -6.41e-03 | -5.10 |
| 561 | 135 | 1372.5787 | 1373.5860 | 2424.62 | 1 | 1372.5863 | C11 | 11 | -7.60e-03 | -5.53 |
| 561 | 136 | 486.3385 | 487.3457 | 2892.81 | 1 |  |  |  |  |  |
| 561 | 137 | 894.4344 | 895.4417 | 2168.70 | 1 |  |  |  |  |  |
| 561 | 138 | 1386.8751 | 463.2990 | 2282.72 | 3 |  |  |  |  |  |
| 561 | 139 | 953.4477 | 954.4550 | 1956.57 | 1 |  |  |  |  |  |
| 561 | 140 | 1042.9541 | 1043.9614 | 1527.31 | 1 |  |  |  |  |  |
| 561 | 141 | 1264.5396 | 633.2771 | 1531.21 | 2 |  |  |  |  |  |
| 561 | 142 | 428.2730 | 429.2803 | 2060.91 | 1 |  |  |  |  |  |
| 561 | 143 | 1317.8522 | 659.9334 | 2778.09 | 2 |  |  |  |  |  |
| 561 | 144 | 917.3876 | 918.3949 | 1386.97 | 1 |  |  |  |  |  |
| 561 | 145 | 542.3153 | 543.3226 | 1652.78 | 1 |  |  |  |  |  |
| 561 | 146 | 1157.4903 | 1158.4976 | 1240.86 | 1 | 1157.4957 | C9 | 9 | -5.37e-03 | -4.64 |
| 561 | 147 | 1456.4347 | 729.2246 | 1389.20 | 2 |  |  |  |  |  |

  

All proteins /
CsTx-1a\_S1 Cupiennius salei toxin 1 isoform a S1^ACsTx-1a\_S2 Cupiennius salei toxin 1 isoform a S2 /
Proteoform #15
