# Supplementary material for: The Dual Prey-Inactivation Strategy of Spiders—In-Depth Venomic Analysis of Cupiennius salei
Source: Toxins (Basel). 2019 Mar 19;11(3):167. doi: 10.3390/toxins11030167 (PMC6468893; doi:10.3390/toxins11030167)
Supplement: Supplementary file 1 [file toxins-11-00167-s001.zip › Supplementary Dataset EV1/20180328_f2_topdown_OTMS2_EThcD_NL_i02_ms2_proteoform_cutoff_html/prsms/prsm181.html]

Protein-Spectrum-Match for Spectrum #420


All proteins /
CsTx-1a\_S1 Cupiennius salei toxin 1 isoform a S1^ACsTx-1a\_S2 Cupiennius salei toxin 1 isoform a S2 /
Proteoform #6

## Protein-Spectrum-Match #181 for Spectrum #420

|  |  |  |  |  |  |
| --- | --- | --- | --- | --- | --- |
| PrSM ID: | 181 | Scan(s): | 563 | Precursor charge: | 14 |
| Precursor m/z: | 630.3105 | Precursor mass: | 8810.2446 | Proteoform mass: | 8810.2496 |
| # matched peaks: | 65 | # matched fragment ions: | 46 | # unexpected modifications: | 1 |
| E-value: | 7.67e-38 | P-value: | 7.67e-38 | Q-value (Spectral FDR): | 0 |

  

|  |  |  |  |  |  |  |  |  |  |  |  |  |  |  |  |  |  |  |  |  |  |  |  |  |  |  |  |  |  |  |  |  |  |  |  |  |  |  |  |  |  |  |  |  |  |  |  |  |  |  |  |  |  |  |  |  |  |  |  |  |  |  |  |  |  |  |  |  |  |
| --- | --- | --- | --- | --- | --- | --- | --- | --- | --- | --- | --- | --- | --- | --- | --- | --- | --- | --- | --- | --- | --- | --- | --- | --- | --- | --- | --- | --- | --- | --- | --- | --- | --- | --- | --- | --- | --- | --- | --- | --- | --- | --- | --- | --- | --- | --- | --- | --- | --- | --- | --- | --- | --- | --- | --- | --- | --- | --- | --- | --- | --- | --- | --- | --- | --- | --- | --- | --- | --- |
|  | |  | | | | | | | | | | | | | | | | | | | | | | | | | | | | | | | | | | | | | | | | | | | | | | | | | | | | | | | | | | | | | | | | | | | |
| 1 |  |  | M |  | K |  | V |  | L |  | I |  | I |  | S |  | A |  | V |  | L |  |  | F |  | I |  | T |  | I |  | F |  | S |  | N |  | I |  | S |  | A |  |  | E |  | I |  | E |  | D |  | D |  | F |  | L |  | E |  | D |  | E |  | 30 |  |
|  | |  | | | | | | | | | | | | | | | | | | | | | | | | | | | | | | | | | | | | | | | | | | | | | | | | | | | | | | | | | | | | | | | | | | | |
| 31 |  |  | S |  | F |  | E |  | A |  | E |  | D |  | I |  | I |  | P |  | F |  |  | F |  | E |  | N |  | E |  | Q |  | A |  | R | ] | S |  | C |  | I |  |  | P |  | K |  | H |  | E |  | E | ⎫ | C |  | T | ⎫ | N | ⎫ | D | ⎫ | K |  | 60 |  |
|  | |  | | | | | | | | | | | | | | | | | | | | | | | | | | | | | | | | | | | | | | | | | | | | | | | | | | | | | | | | | | | | | | | | | | | |
| 61 |  | ⎫ | H | ⎫ | N | ⎫ | C |  | C |  | R | ⎫ | K | ⎫ | G | ⎱ | L | ⎱ | F | ⎫ | K |  | ⎫ | L | ⎫ | K | ⎫ | C | ⎫ | Q | ⎫ | C |  | S |  | T |  | F | ⎫ | D | ⎫ | D |  | ⎫ | E | ⎫ | S | ⎫ | G | ⎫ | Q |  | P |  | T | ⎫ | E | ⎫ | R |  | C |  | A |  | 90 |  |
|  | |  | | | | | | | | | | | | | | | | | | | | | | | | | | | | | | | | | | | | | | | | | | | | | | | | | | | | | | | | | -58.01 | | | | | | | |
| 91 |  |  | C | ⎫ | G | ⎫ | R |  | P |  | M |  | G |  | H | ⎫ | Q |  | A |  | I |  |  | E | ⎫ | T |  | G |  | L | ⎫ | N |  | I |  | F |  | R | ⎱ | G |  | L |  | ⎩ | F | ⎩ | K | ⎩ | G |  | K | ⎱ | K | ⎫ | K | ⎱ | N | ⎱ | K |  | K |  | T |  | 120 |  |
|  | |  | | | | | | | | | | | | | | | | | | | | | | | | | | | | | | | | | | | | | | | | | | | | | | | | | | | | | | | | | | | | | | | | | | | |
| 121 |  | ⎫ | K | ⎫ | G |  | | | | 122 |  | | | | | | | | | | | | | | | | | | | | | | | | | | | | | | | | | | | | | | | | | | | | | | | | | | | | | | | |

Fixed PTMs: Carbamidomethylation [C49 C56 C63 C64 C73 C75 C89 C91 ]   
  
     Unexpected modifications:   Unknown [-58.01]

  

All peaks (146)  Matched peaks (65)  Not matched peaks (81)

  

| Scan | Peak | Mono mass | Mono m/z | Intensity | Charge | Theoretical mass | Ion | Pos | Mass error | PPM error |
| --- | --- | --- | --- | --- | --- | --- | --- | --- | --- | --- |
| 563 | 1 | 8754.1889 | 796.8426 | 72467.90 | 11 |  |  |  |  |  |
| 563 | 2 | 8753.1912 | 730.4399 | 60693.58 | 12 | 8752.2441 | C74 | 74 | -0.0553 | -6.31 |
| 563 | 3 | 4443.9130 | 741.6594 | 49974.67 | 6 | 4443.9333 | C36 | 36 | -0.0203 | -4.57 |
| 563 | 4 | 8768.1934 | 731.6901 | 34648.81 | 12 |  |  |  |  |  |
| 563 | 5 | 8754.1949 | 876.4268 | 43277.91 | 10 |  |  |  |  |  |
| 563 | 6 | 2528.0777 | 633.0267 | 53376.04 | 4 | 2528.0889 | C20 | 20 | -0.0112 | -4.43 |
| 563 | 7 | 8696.1671 | 791.5679 | 36142.70 | 11 |  |  |  |  |  |
| 563 | 8 | 8769.1944 | 798.2068 | 37618.23 | 11 |  |  |  |  |  |
| 563 | 9 | 2471.0567 | 618.7714 | 45493.19 | 4 | 2471.0674 | C19 | 19 | -0.0108 | -4.35 |
| 563 | 10 | 8696.1651 | 870.6238 | 29414.46 | 10 |  |  |  |  |  |
| 563 | 11 | 8795.1963 | 733.9403 | 27470.10 | 12 |  |  |  |  |  |
| 563 | 12 | 8737.1753 | 795.2959 | 31209.90 | 11 |  |  |  |  |  |
| 563 | 13 | 8710.1641 | 792.8404 | 28102.66 | 11 |  |  |  |  |  |
| 563 | 14 | 8738.1780 | 874.8251 | 22095.60 | 10 |  |  |  |  |  |
| 563 | 15 | 3445.5881 | 690.1249 | 27547.63 | 5 | 3445.6046 | C27 | 27 | -0.0165 | -4.79 |
| 563 | 16 | 8682.1317 | 790.2920 | 21257.97 | 11 |  |  |  |  |  |
| 563 | 17 | 4554.9452 | 760.1648 | 30046.75 | 6 |  |  |  |  |  |
| 563 | 18 | 2641.1617 | 661.2977 | 26543.54 | 4 | 2641.1730 | C21 | 21 | -0.0113 | -4.28 |
| 563 | 19 | 8711.1714 | 872.1244 | 29330.95 | 10 |  |  |  |  |  |
| 563 | 20 | 4527.3795 | 647.7758 | 18772.22 | 7 |  |  |  |  |  |
| 563 | 21 | 8769.1821 | 877.9255 | 23913.21 | 10 |  |  |  |  |  |
| 563 | 22 | 1372.5808 | 687.2977 | 31355.28 | 2 | 1372.5863 | C11 | 11 | -5.47e-03 | -3.99 |
| 563 | 23 | 4405.6064 | 735.2750 | 29489.41 | 6 |  |  |  |  |  |
| 563 | 24 | 4443.9167 | 889.7906 | 18309.28 | 5 | 4443.9333 | C36 | 36 | -0.0167 | -3.75 |
| 563 | 25 | 3157.5032 | 632.5079 | 31230.64 | 5 | 3157.5153 | C25 | 25 | -0.0122 | -3.86 |
| 563 | 26 | 8724.1706 | 794.1137 | 17126.23 | 11 |  |  |  |  |  |
| 563 | 27 | 3317.5315 | 664.5136 | 20953.64 | 5 | 3317.5460 | C26 | 26 | -0.0145 | -4.36 |
| 563 | 28 | 8696.1695 | 967.2483 | 15503.59 | 9 |  |  |  |  |  |
| 563 | 29 | 6284.1334 | 786.5239 | 19384.78 | 8 | 6283.1686 | Z\_DOT55 | 20 | -0.0375 | -5.98 |
| 563 | 30 | 8796.1956 | 800.6614 | 23460.78 | 11 |  |  |  |  |  |
| 563 | 31 | 4255.2689 | 608.9028 | 16250.86 | 7 |  |  |  |  |  |
| 563 | 32 | 4309.2767 | 616.6182 | 15341.89 | 7 |  |  |  |  |  |
| 563 | 33 | 4282.8320 | 714.8126 | 17691.48 | 6 |  |  |  |  |  |
| 563 | 34 | 2916.3248 | 730.0885 | 20646.55 | 4 | 2916.3363 | C23 | 23 | -0.0116 | -3.97 |
| 563 | 35 | 4368.3159 | 729.0599 | 18017.88 | 6 |  |  |  |  |  |
| 563 | 36 | 2528.0789 | 843.7002 | 23669.43 | 3 | 2528.0889 | C20 | 20 | -9.97e-03 | -3.95 |
| 563 | 37 | 8680.1322 | 869.0205 | 29506.92 | 10 |  |  |  |  |  |
| 563 | 38 | 2788.2293 | 698.0646 | 17863.02 | 4 | 2788.2414 | C22 | 22 | -0.0121 | -4.33 |
| 563 | 39 | 6082.6065 | 761.3331 | 13985.49 | 8 | 6081.6306 | C50 | 50 | -0.0264 | -4.35 |
| 563 | 40 | 4770.0747 | 796.0197 | 19949.46 | 6 | 4770.0923 | C39 | 39 | -0.0176 | -3.70 |
| 563 | 41 | 8736.1756 | 729.0219 | 16914.71 | 12 |  |  |  |  |  |
| 563 | 42 | 7325.2798 | 814.9273 | 17148.11 | 9 | 7324.3027 | C61 | 61 | -0.0252 | -3.44 |
| 563 | 43 | 3788.0200 | 632.3439 | 22458.97 | 6 |  |  |  |  |  |
| 563 | 44 | 1752.7602 | 877.3874 | 25000.57 | 2 | 1752.7671 | C14 | 14 | -6.96e-03 | -3.97 |
| 563 | 45 | 8736.1858 | 673.0216 | 9751.45 | 13 |  |  |  |  |  |
| 563 | 46 | 8752.1779 | 674.2517 | 19748.31 | 13 | 8752.2441 | C74 | 74 | -0.0662 | -7.56 |
| 563 | 47 | 8793.1957 | 677.4069 | 14881.34 | 13 |  |  |  |  |  |
| 563 | 48 | 7326.2767 | 916.7919 | 9963.70 | 8 |  |  |  |  |  |
| 563 | 49 | 4055.7909 | 812.1655 | 14453.88 | 5 | 4055.8103 | C32 | 32 | -0.0194 | -4.77 |
| 563 | 50 | 3029.4078 | 758.3592 | 16108.82 | 4 | 3029.4204 | C24 | 24 | -0.0126 | -4.17 |
| 563 | 51 | 4299.8590 | 717.6504 | 13272.88 | 6 | 4299.8798 | C34 | 34 | -0.0208 | -4.84 |
| 563 | 52 | 5034.5466 | 630.3256 | 23214.21 | 8 |  |  |  |  |  |
| 563 | 53 | 7955.6595 | 796.5732 | 14361.38 | 10 | 7954.6880 | C67 | 67 | -0.0308 | -3.88 |
| 563 | 54 | 7897.6173 | 878.5203 | 11123.60 | 9 |  |  |  |  |  |
| 563 | 55 | 6241.0927 | 694.4620 | 13082.48 | 9 |  |  |  |  |  |
| 563 | 56 | 8767.2013 | 675.4074 | 10283.48 | 13 |  |  |  |  |  |
| 563 | 57 | 5034.5470 | 720.2283 | 14925.63 | 7 |  |  |  |  |  |
| 563 | 58 | 1615.7018 | 808.8582 | 17937.56 | 2 | 1615.7082 | C13 | 13 | -6.40e-03 | -3.96 |
| 563 | 59 | 7267.2513 | 909.4137 | 11503.64 | 8 |  |  |  |  |  |
| 563 | 60 | 8653.1451 | 866.3218 | 11445.13 | 10 |  |  |  |  |  |
| 563 | 61 | 4386.9002 | 878.3873 | 15066.60 | 5 | 4386.9119 | C35 | 35 | -0.0116 | -2.65 |
| 563 | 62 | 2471.0562 | 824.6927 | 12817.83 | 3 | 2471.0674 | C19 | 19 | -0.0112 | -4.54 |
| 563 | 63 | 6284.1433 | 898.7420 | 14029.71 | 7 | 6283.1686 | Z\_DOT55 | 20 | -0.0276 | -4.40 |
| 563 | 64 | 2203.3707 | 551.8499 | 21448.39 | 4 |  |  |  |  |  |
| 563 | 65 | 8709.1598 | 726.7706 | 14512.81 | 12 |  |  |  |  |  |
| 563 | 66 | 8211.8378 | 822.1911 | 10701.39 | 10 | 8210.8779 | C69 | 69 | -0.0424 | -5.17 |
| 563 | 67 | 4153.7910 | 693.3058 | 12672.61 | 6 |  |  |  |  |  |
| 563 | 68 | 1866.8017 | 934.4081 | 15883.18 | 2 | 1866.8101 | C15 | 15 | -8.33e-03 | -4.46 |
| 563 | 69 | 2937.0684 | 735.2744 | 17374.56 | 4 |  |  |  |  |  |
| 563 | 70 | 8653.1388 | 962.4671 | 8443.76 | 9 |  |  |  |  |  |
| 563 | 71 | 8696.1754 | 725.6886 | 11634.68 | 12 |  |  |  |  |  |
| 563 | 72 | 2342.9626 | 781.9948 | 11308.42 | 3 | 2342.9725 | C18 | 18 | -9.88e-03 | -4.22 |
| 563 | 73 | 3940.7641 | 789.1601 | 12590.91 | 5 | 3940.7834 | C31 | 31 | -0.0193 | -4.89 |
| 563 | 74 | 8754.1981 | 973.6960 | 12278.31 | 9 |  |  |  |  |  |
| 563 | 75 | 5503.3289 | 787.1971 | 8692.01 | 7 | 5503.3559 | C45 | 45 | -0.0270 | -4.90 |
| 563 | 76 | 4299.8610 | 860.9795 | 10978.28 | 5 | 4299.8798 | C34 | 34 | -0.0188 | -4.37 |
| 563 | 77 | 4440.3465 | 635.3425 | 9122.87 | 7 |  |  |  |  |  |
| 563 | 78 | 4255.2721 | 710.2193 | 11580.64 | 6 |  |  |  |  |  |
| 563 | 79 | 4170.8195 | 696.1439 | 9968.37 | 6 | 4170.8372 | C33 | 33 | -0.0178 | -4.26 |
| 563 | 80 | 5503.3342 | 918.2296 | 7069.44 | 6 | 5503.3559 | C45 | 45 | -0.0217 | -3.94 |
| 563 | 81 | 8211.8429 | 747.5384 | 9989.36 | 11 | 8210.8779 | C69 | 69 | -0.0374 | -4.56 |
| 563 | 82 | 8667.1264 | 867.7199 | 10647.11 | 10 |  |  |  |  |  |
| 563 | 83 | 1386.8772 | 463.2997 | 11062.29 | 3 |  |  |  |  |  |
| 563 | 84 | 4512.3648 | 753.0681 | 9914.03 | 6 |  |  |  |  |  |
| 563 | 85 | 8682.1591 | 965.6916 | 8913.04 | 9 |  |  |  |  |  |
| 563 | 86 | 3982.1348 | 569.8837 | 11244.89 | 7 |  |  |  |  |  |
| 563 | 87 | 7897.6377 | 790.7710 | 15064.15 | 10 |  |  |  |  |  |
| 563 | 88 | 8211.8495 | 913.4350 | 8826.25 | 9 | 8210.8779 | C69 | 69 | -0.0307 | -3.74 |
| 563 | 89 | 5381.6467 | 769.8140 | 6648.18 | 7 |  |  |  |  |  |
| 563 | 90 | 1487.6067 | 744.8106 | 9778.71 | 2 | 1487.6133 | C12 | 12 | -6.53e-03 | -4.39 |
| 563 | 91 | 2617.5784 | 655.4019 | 9936.07 | 4 |  |  |  |  |  |
| 563 | 92 | 7324.2751 | 733.4348 | 7743.46 | 10 | 7324.3027 | C61 | 61 | -0.0276 | -3.77 |
| 563 | 93 | 8082.7577 | 809.2830 | 11113.86 | 10 | 8082.7830 | C68 | 68 | -0.0253 | -3.13 |
| 563 | 94 | 6284.1240 | 699.2433 | 12131.05 | 9 | 6283.1686 | Z\_DOT55 | 20 | -0.0470 | -7.47 |
| 563 | 95 | 8712.1879 | 969.0282 | 11610.06 | 9 |  |  |  |  |  |
| 563 | 96 | 4528.3700 | 755.7356 | 7616.64 | 6 |  |  |  |  |  |
| 563 | 97 | 4171.8172 | 835.3707 | 9584.37 | 5 |  |  |  |  |  |
| 563 | 98 | 6171.0748 | 772.3916 | 11132.17 | 8 | 6170.0845 | Z\_DOT54 | 21 | -0.0121 | -1.95 |
| 563 | 99 | 8664.1708 | 788.6592 | 9383.04 | 11 |  |  |  |  |  |
| 563 | 100 | 5446.3178 | 908.7269 | 8906.05 | 6 | 5446.3345 | C44 | 44 | -0.0167 | -3.07 |
| 563 | 101 | 8639.1057 | 864.9178 | 12276.35 | 10 |  |  |  |  |  |
| 563 | 102 | 6241.0919 | 781.1438 | 9434.12 | 8 |  |  |  |  |  |
| 563 | 103 | 8626.1092 | 785.1990 | 9152.97 | 11 |  |  |  |  |  |
| 563 | 104 | 1169.7803 | 585.8974 | 10714.87 | 2 | 1169.7808 | Z\_DOT11 | 64 | -5.41e-04 | -0.46 |
| 563 | 105 | 8325.9009 | 833.5974 | 8447.72 | 10 | 8324.9209 | C70 | 70 | -0.0223 | -2.68 |
| 563 | 106 | 8152.7920 | 816.2865 | 13952.46 | 10 |  |  |  |  |  |
| 563 | 107 | 4713.0487 | 943.6170 | 7774.03 | 5 |  |  |  |  |  |
| 563 | 108 | 8797.2070 | 880.7280 | 11441.17 | 10 |  |  |  |  |  |
| 563 | 109 | 6523.8375 | 932.9841 | 5928.27 | 7 | 6522.8530 | C54 | 54 | -0.0178 | -2.73 |
| 563 | 110 | 3157.5027 | 790.3830 | 17499.33 | 4 | 3157.5153 | C25 | 25 | -0.0126 | -3.99 |
| 563 | 111 | 1428.8873 | 477.3030 | 8709.71 | 3 |  |  |  |  |  |
| 563 | 112 | 3759.6566 | 627.6167 | 5547.11 | 6 |  |  |  |  |  |
| 563 | 113 | 4368.3172 | 874.6707 | 8562.45 | 5 |  |  |  |  |  |
| 563 | 114 | 7954.6428 | 724.1566 | 10632.74 | 11 | 7954.6880 | C67 | 67 | -0.0452 | -5.69 |
| 563 | 115 | 1486.9529 | 496.6582 | 7424.81 | 3 | 1486.9548 | Z\_DOT14 | 61 | -1.91e-03 | -1.28 |
| 563 | 116 | 4055.7912 | 676.9725 | 8033.38 | 6 | 4055.8103 | C32 | 32 | -0.0191 | -4.71 |
| 563 | 117 | 2358.4283 | 590.6143 | 8626.03 | 4 |  |  |  |  |  |
| 563 | 118 | 4406.6100 | 882.3293 | 11613.42 | 5 |  |  |  |  |  |
| 563 | 119 | 8667.1667 | 964.0258 | 8755.93 | 9 |  |  |  |  |  |
| 563 | 120 | 6793.9824 | 850.2551 | 6617.52 | 8 | 6794.0062 | C57 | 57 | -0.0238 | -3.51 |
| 563 | 121 | 6082.6036 | 869.9507 | 8118.22 | 7 | 6081.6306 | C50 | 50 | -0.0294 | -4.83 |
| 563 | 122 | 7669.7550 | 767.9828 | 5858.78 | 10 |  |  |  |  |  |
| 563 | 123 | 5565.7241 | 928.6280 | 6303.63 | 6 |  |  |  |  |  |
| 563 | 124 | 4771.0696 | 955.2212 | 6335.29 | 5 |  |  |  |  |  |
| 563 | 125 | 7955.6625 | 884.9698 | 9025.20 | 9 | 7954.6880 | C67 | 67 | -0.0279 | -3.51 |
| 563 | 126 | 4899.1116 | 817.5259 | 9628.76 | 6 | 4899.1349 | C40 | 40 | -0.0233 | -4.77 |
| 563 | 127 | 8625.1074 | 863.5180 | 12309.99 | 10 | 8624.1492 | C73 | 73 | -0.0441 | -5.11 |
| 563 | 128 | 997.4611 | 998.4684 | 7959.10 | 1 | 997.4651 | C8 | 8 | -3.95e-03 | -3.96 |
| 563 | 129 | 798.5049 | 400.2597 | 6238.60 | 2 |  |  |  |  |  |
| 563 | 130 | 600.3813 | 601.3886 | 11070.82 | 1 | 600.3795 | Z\_DOT6 | 69 | 1.79e-03 | 2.97 |
| 563 | 131 | 1258.5386 | 1259.5458 | 4684.23 | 1 | 1258.5434 | C10 | 10 | -4.83e-03 | -3.84 |
| 563 | 132 | 997.4615 | 499.7380 | 5428.57 | 2 | 997.4651 | C8 | 8 | -3.53e-03 | -3.54 |
| 563 | 133 | 1316.8475 | 439.9565 | 3299.42 | 3 | 1316.8493 | Z\_DOT12 | 63 | -1.73e-03 | -1.32 |
| 563 | 134 | 678.3254 | 679.3327 | 6107.79 | 1 |  |  |  |  |  |
| 563 | 135 | 1111.7147 | 371.5789 | 3316.37 | 3 |  |  |  |  |  |
| 563 | 136 | 1316.8500 | 659.4323 | 4381.74 | 2 | 1316.8493 | Z\_DOT12 | 63 | 7.51e-04 | 0.57 |
| 563 | 137 | 1041.6861 | 521.8503 | 5279.76 | 2 | 1041.6859 | Z\_DOT10 | 65 | 1.81e-04 | 0.17 |
| 563 | 138 | 486.3389 | 487.3462 | 6267.38 | 1 | 486.3366 | Z\_DOT5 | 70 | 2.27e-03 | 4.66 |
| 563 | 139 | 1469.3731 | 735.6938 | 22679.17 | 2 |  |  |  |  |  |
| 563 | 140 | 856.5700 | 857.5773 | 5922.31 | 1 | 856.5695 | Z\_DOT8 | 67 | 5.25e-04 | 0.61 |
| 563 | 141 | 1415.8795 | 472.9671 | 5039.04 | 3 |  |  |  |  |  |
| 563 | 142 | 1169.7805 | 390.9341 | 3683.75 | 3 | 1169.7808 | Z\_DOT11 | 64 | -3.41e-04 | -0.29 |
| 563 | 143 | 629.2695 | 630.2768 | 8112.65 | 1 |  |  |  |  |  |
| 563 | 144 | 1258.7825 | 420.6014 | 3184.33 | 3 |  |  |  |  |  |
| 563 | 145 | 801.5670 | 802.5743 | 13424.95 | 1 |  |  |  |  |  |
| 563 | 146 | 1023.4771 | 512.7458 | 3248.37 | 2 |  |  |  |  |  |

  

All proteins /
CsTx-1a\_S1 Cupiennius salei toxin 1 isoform a S1^ACsTx-1a\_S2 Cupiennius salei toxin 1 isoform a S2 /
Proteoform #6
