# Supplementary material for: The Dual Prey-Inactivation Strategy of Spiders—In-Depth Venomic Analysis of Cupiennius salei
Source: Toxins (Basel). 2019 Mar 19;11(3):167. doi: 10.3390/toxins11030167 (PMC6468893; doi:10.3390/toxins11030167)
Supplement: Supplementary file 1 [file toxins-11-00167-s001.zip › Supplementary Dataset EV1/20180328_f2_topdown_OTMS2_EThcD_NL_i02_ms2_proteoform_cutoff_html/prsms/prsm182.html]

Protein-Spectrum-Match for Spectrum #421


All proteins /
CsTx-1a\_S1 Cupiennius salei toxin 1 isoform a S1^ACsTx-1a\_S2 Cupiennius salei toxin 1 isoform a S2 /
Proteoform #6

## Protein-Spectrum-Match #182 for Spectrum #421

|  |  |  |  |  |  |
| --- | --- | --- | --- | --- | --- |
| PrSM ID: | 182 | Scan(s): | 564 | Precursor charge: | 13 |
| Precursor m/z: | 678.7187 | Precursor mass: | 8810.2479 | Proteoform mass: | 8811.2375 |
| # matched peaks: | 57 | # matched fragment ions: | 46 | # unexpected modifications: | 0 |
| E-value: | 4.42e-42 | P-value: | 4.42e-42 | Q-value (Spectral FDR): | 0 |

  

|  |  |  |  |  |  |  |  |  |  |  |  |  |  |  |  |  |  |  |  |  |  |  |  |  |  |  |  |  |  |  |  |  |  |  |  |  |  |  |  |  |  |  |  |  |  |  |  |  |  |  |  |  |  |  |  |  |  |  |  |  |  |  |  |  |  |  |  |  |  |
| --- | --- | --- | --- | --- | --- | --- | --- | --- | --- | --- | --- | --- | --- | --- | --- | --- | --- | --- | --- | --- | --- | --- | --- | --- | --- | --- | --- | --- | --- | --- | --- | --- | --- | --- | --- | --- | --- | --- | --- | --- | --- | --- | --- | --- | --- | --- | --- | --- | --- | --- | --- | --- | --- | --- | --- | --- | --- | --- | --- | --- | --- | --- | --- | --- | --- | --- | --- | --- | --- |
|  | |  | | | | | | | | | | | | | | | | | | | | | | | | | | | | | | | | | | | | | | | | | | | | | | | | | | | | | | | | | | | | | | | | | | | |
| 1 |  |  | M |  | K |  | V |  | L |  | I |  | I |  | S |  | A |  | V |  | L |  |  | F |  | I |  | T |  | I |  | F |  | S |  | N |  | I |  | S |  | A |  |  | E |  | I |  | E |  | D |  | D |  | F |  | L |  | E |  | D |  | E |  | 30 |  |
|  | |  | | | | | | | | | | | | | | | | | | | | | | | | | | | | | | | | | | | | | | | | | | | | | | | | | | | | | | | | | | | | | | | | | | | |
| 31 |  |  | S |  | F |  | E |  | A |  | E |  | D |  | I |  | I |  | P |  | F |  |  | F |  | E |  | N |  | E |  | Q |  | A |  | R | ] | S | ⎩ | C |  | I |  |  | P |  | K | ⎩ | H |  | E |  | E | ⎫ | C | ⎩ | T | ⎱ | N | ⎱ | D | ⎫ | K |  | 60 |  |
|  | |  | | | | | | | | | | | | | | | | | | | | | | | | | | | | | | | | | | | | | | | | | | | | | | | | | | | | | | | | | | | | | | | | | | | |
| 61 |  |  | H | ⎫ | N |  | C |  | C |  | R |  | K | ⎫ | G | ⎱ | L | ⎱ | F |  | K |  |  | L |  | K | ⎫ | C | ⎫ | Q | ⎫ | C |  | S |  | T |  | F | ⎫ | D | ⎫ | D |  | ⎫ | E | ⎫ | S | ⎫ | G | ⎱ | Q |  | P |  | T | ⎫ | E | ⎫ | R |  | C |  | A |  | 90 |  |
|  | |  | | | | | | | | | | | | | | | | | | | | | | | | | | | | | | | | | | | | | | | | | | | | | | | | | | | | | | | | | | | | | | | | | | | |
| 91 |  |  | C |  | G | ⎫ | R |  | P | ⎫ | M |  | G |  | H | ⎫ | Q | ⎫ | A |  | I |  |  | E | ⎫ | T | ⎫ | G | ⎫ | L | ⎫ | N |  | I |  | F |  | R | ⎫ | G | ⎫ | L |  |  | F |  | K | ⎫ | G | ⎫ | K | ⎫ | K | ⎫ | K | ⎫ | N | ⎫ | K | ⎫ | K | ⎫ | T |  | 120 |  |
|  | |  | | | | | | | | | | | | | | | | | | | | | | | | | | | | | | | | | | | | | | | | | | | | | | | | | | | | | | | | | | | | | | | | | | | |
| 121 |  | ⎫ | K | [ | G |  | | | | 122 |  | | | | | | | | | | | | | | | | | | | | | | | | | | | | | | | | | | | | | | | | | | | | | | | | | | | | | | | |

Fixed PTMs: Carbamidomethylation [C49 C56 C63 C64 C73 C75 C89 C91 ]

  

All peaks (146)  Matched peaks (57)  Not matched peaks (89)

  

| Scan | Peak | Mono mass | Mono m/z | Intensity | Charge | Theoretical mass | Ion | Pos | Mass error | PPM error |
| --- | --- | --- | --- | --- | --- | --- | --- | --- | --- | --- |
| 564 | 1 | 8754.2004 | 796.8437 | 410566.45 | 11 |  |  |  |  |  |
| 564 | 2 | 8753.2176 | 730.4421 | 356703.19 | 12 |  |  |  |  |  |
| 564 | 3 | 8768.2008 | 798.1164 | 190198.92 | 11 |  |  |  |  |  |
| 564 | 4 | 8754.2034 | 876.4276 | 148560.72 | 10 |  |  |  |  |  |
| 564 | 5 | 8768.2066 | 731.6912 | 132730.43 | 12 |  |  |  |  |  |
| 564 | 6 | 8795.2040 | 800.5713 | 131115.96 | 11 |  |  |  |  |  |
| 564 | 7 | 8682.1458 | 790.2933 | 127831.55 | 11 | 8682.1585 | C73 | 73 | -0.0126 | -1.46 |
| 564 | 8 | 8737.2142 | 729.1085 | 106851.05 | 12 |  |  |  |  |  |
| 564 | 9 | 8738.2053 | 795.3896 | 104555.99 | 11 |  |  |  |  |  |
| 564 | 10 | 8696.1771 | 791.5688 | 104780.00 | 11 |  |  |  |  |  |
| 564 | 11 | 8794.2193 | 733.8589 | 96878.07 | 12 |  |  |  |  |  |
| 564 | 12 | 4405.6193 | 735.2772 | 373691.47 | 6 |  |  |  |  |  |
| 564 | 13 | 8793.2251 | 677.4092 | 112563.81 | 13 |  |  |  |  |  |
| 564 | 14 | 8709.1933 | 792.7521 | 79786.98 | 11 | 8708.1868 | Z\_DOT73 | 1 | 4.20e-03 | 0.48 |
| 564 | 15 | 8768.2159 | 877.8289 | 66436.04 | 10 |  |  |  |  |  |
| 564 | 16 | 8083.7519 | 809.3825 | 60426.73 | 10 | 8082.7830 | C68 | 68 | -0.0334 | -4.13 |
| 564 | 17 | 8682.1350 | 869.2208 | 71578.28 | 10 | 8682.1585 | C73 | 73 | -0.0235 | -2.71 |
| 564 | 18 | 8710.1837 | 872.0256 | 56815.77 | 10 |  |  |  |  |  |
| 564 | 19 | 8696.1779 | 870.6251 | 65246.46 | 10 |  |  |  |  |  |
| 564 | 20 | 7955.6896 | 796.5762 | 71240.62 | 10 | 7954.6880 | C67 | 67 | -7.82e-04 | -0.10 |
| 564 | 21 | 8738.1872 | 874.8260 | 50066.81 | 10 |  |  |  |  |  |
| 564 | 22 | 4443.9145 | 741.6597 | 47280.36 | 6 | 4443.9333 | C36 | 36 | -0.0189 | -4.24 |
| 564 | 23 | 8453.9798 | 769.5509 | 44564.11 | 11 | 8453.0158 | C71 | 71 | -0.0384 | -4.54 |
| 564 | 24 | 4554.9507 | 760.1657 | 61860.23 | 6 |  |  |  |  |  |
| 564 | 25 | 8210.8582 | 822.0931 | 46412.62 | 10 | 8209.9243 | Z\_DOT69 | 5 | -0.0685 | -8.34 |
| 564 | 25 | 8210.8582 | 822.0931 | 46412.62 | 10 | 8210.8779 | C69 | 69 | -0.0197 | -2.40 |
| 564 | 26 | 7324.2758 | 814.8157 | 45953.90 | 9 | 7324.3027 | C61 | 61 | -0.0268 | -3.67 |
| 564 | 27 | 6284.1304 | 786.5236 | 44324.87 | 8 | 6284.1564 | Z\_DOT54 | 20 | -0.0260 | -4.13 |
| 564 | 28 | 8324.8930 | 757.8157 | 44006.82 | 11 | 8324.9209 | C70 | 70 | -0.0279 | -3.35 |
| 564 | 29 | 6240.1055 | 781.0205 | 43765.18 | 8 |  |  |  |  |  |
| 564 | 30 | 8796.2365 | 880.6309 | 43442.01 | 10 |  |  |  |  |  |
| 564 | 31 | 678.4020 | 679.4092 | 88088.46 | 1 |  |  |  |  |  |
| 564 | 32 | 8211.8496 | 747.5391 | 39515.21 | 11 | 8210.8779 | C69 | 69 | -0.0307 | -3.74 |
| 564 | 33 | 8720.2125 | 727.6916 | 35715.90 | 12 |  |  |  |  |  |
| 564 | 34 | 8625.1123 | 863.5185 | 32940.38 | 10 |  |  |  |  |  |
| 564 | 35 | 8722.2068 | 793.9352 | 41729.74 | 11 |  |  |  |  |  |
| 564 | 36 | 8705.2202 | 726.4423 | 37470.60 | 12 |  |  |  |  |  |
| 564 | 37 | 8663.1926 | 788.5702 | 31305.81 | 11 |  |  |  |  |  |
| 564 | 38 | 8325.8960 | 833.5969 | 33042.23 | 10 | 8324.9209 | C70 | 70 | -0.0272 | -3.26 |
| 564 | 39 | 4444.9210 | 889.9915 | 34819.68 | 5 |  |  |  |  |  |
| 564 | 40 | 2528.0821 | 633.0278 | 40651.81 | 4 | 2528.0889 | C20 | 20 | -6.81e-03 | -2.70 |
| 564 | 41 | 8453.9868 | 846.4060 | 26861.30 | 10 | 8453.0158 | C71 | 71 | -0.0313 | -3.71 |
| 564 | 42 | 4309.2886 | 719.2220 | 36954.22 | 6 |  |  |  |  |  |
| 564 | 43 | 4405.6153 | 882.1303 | 49801.83 | 5 |  |  |  |  |  |
| 564 | 44 | 7955.6716 | 884.9708 | 29721.13 | 9 | 7954.6880 | C67 | 67 | -0.0187 | -2.35 |
| 564 | 45 | 3157.5068 | 632.5086 | 27409.19 | 5 | 3157.5153 | C25 | 25 | -8.53e-03 | -2.70 |
| 564 | 46 | 4282.8419 | 714.8143 | 28542.34 | 6 |  |  |  |  |  |
| 564 | 47 | 6855.4206 | 762.7207 | 25617.60 | 9 |  |  |  |  |  |
| 564 | 48 | 3445.5930 | 690.1259 | 24733.63 | 5 | 3445.6046 | C27 | 27 | -0.0116 | -3.37 |
| 564 | 49 | 7826.5562 | 783.6629 | 28787.74 | 10 | 7826.5930 | C66 | 66 | -0.0369 | -4.71 |
| 564 | 50 | 7723.7693 | 773.3842 | 26937.80 | 10 |  |  |  |  |  |
| 564 | 51 | 4255.2743 | 710.2197 | 25862.32 | 6 |  |  |  |  |  |
| 564 | 52 | 8662.2047 | 722.8577 | 23220.62 | 12 |  |  |  |  |  |
| 564 | 53 | 2471.0608 | 618.7725 | 33512.24 | 4 | 2471.0674 | C19 | 19 | -6.60e-03 | -2.67 |
| 564 | 54 | 6284.1565 | 898.7439 | 24967.25 | 7 | 6284.1564 | Z\_DOT54 | 20 | 1.05e-04 | 0.02 |
| 564 | 55 | 8639.1205 | 864.9193 | 16972.44 | 10 |  |  |  |  |  |
| 564 | 56 | 8083.7763 | 899.2046 | 23439.73 | 9 | 8082.7830 | C68 | 68 | -9.00e-03 | -1.11 |
| 564 | 57 | 4886.4810 | 699.0760 | 28002.69 | 7 |  |  |  |  |  |
| 564 | 58 | 5033.5519 | 720.0861 | 19939.19 | 7 |  |  |  |  |  |
| 564 | 59 | 4770.0778 | 955.0228 | 19213.11 | 5 | 4770.0923 | C39 | 39 | -0.0145 | -3.04 |
| 564 | 60 | 4299.8675 | 860.9808 | 20007.31 | 5 | 4299.8798 | C34 | 34 | -0.0124 | -2.88 |
| 564 | 61 | 3317.5376 | 664.5148 | 21121.13 | 5 | 3317.5460 | C26 | 26 | -8.37e-03 | -2.52 |
| 564 | 62 | 8665.1440 | 867.5217 | 21075.85 | 10 |  |  |  |  |  |
| 564 | 63 | 6793.9844 | 850.2553 | 23522.46 | 8 | 6794.0062 | C57 | 57 | -0.0218 | -3.21 |
| 564 | 64 | 6240.0968 | 694.3514 | 19068.71 | 9 |  |  |  |  |  |
| 564 | 65 | 4527.3868 | 647.7768 | 21550.37 | 7 |  |  |  |  |  |
| 564 | 66 | 3982.1447 | 664.6981 | 25371.96 | 6 |  |  |  |  |  |
| 564 | 67 | 735.0165 | 736.0238 | 55242.79 | 1 |  |  |  |  |  |
| 564 | 68 | 2641.1665 | 661.2989 | 21842.49 | 4 | 2641.1730 | C21 | 21 | -6.41e-03 | -2.43 |
| 564 | 69 | 6082.6152 | 869.9523 | 38396.05 | 7 | 6081.6306 | C50 | 50 | -0.0178 | -2.93 |
| 564 | 70 | 7325.2859 | 916.6680 | 19523.60 | 8 | 7324.3027 | C61 | 61 | -0.0191 | -2.61 |
| 564 | 71 | 4038.7726 | 674.1360 | 18206.74 | 6 |  |  |  |  |  |
| 564 | 72 | 8618.1768 | 784.4779 | 18108.63 | 11 |  |  |  |  |  |
| 564 | 73 | 7669.7537 | 767.9826 | 17373.43 | 10 |  |  |  |  |  |
| 564 | 74 | 4309.2818 | 616.6190 | 17004.23 | 7 |  |  |  |  |  |
| 564 | 75 | 4554.9472 | 911.9967 | 19051.90 | 5 |  |  |  |  |  |
| 564 | 76 | 4368.3276 | 729.0619 | 20659.92 | 6 | 4368.3120 | Z\_DOT38 | 36 | 0.0156 | 3.58 |
| 564 | 77 | 6080.5988 | 761.0821 | 18212.48 | 8 | 6081.6306 | C50 | 50 | -0.0295 | -4.84 |
| 564 | 78 | 2528.0764 | 843.6994 | 21189.26 | 3 | 2528.0889 | C20 | 20 | -0.0125 | -4.96 |
| 564 | 79 | 8582.0868 | 859.2160 | 18385.65 | 10 | 8581.1108 | C72 | 72 | -0.0263 | -3.07 |
| 564 | 80 | 8025.7328 | 892.7554 | 18039.34 | 9 |  |  |  |  |  |
| 564 | 81 | 6854.4192 | 686.4492 | 15204.34 | 10 |  |  |  |  |  |
| 564 | 82 | 7768.5413 | 864.1785 | 17474.11 | 9 | 7769.5716 | C65 | 65 | -0.0279 | -3.59 |
| 564 | 83 | 1372.5826 | 687.2986 | 27532.79 | 2 | 1372.5863 | C11 | 11 | -3.76e-03 | -2.74 |
| 564 | 84 | 7724.7627 | 703.2584 | 18701.76 | 11 |  |  |  |  |  |
| 564 | 85 | 8774.2067 | 675.9463 | 21750.76 | 13 |  |  |  |  |  |
| 564 | 86 | 8224.9309 | 748.7283 | 27638.77 | 11 |  |  |  |  |  |
| 564 | 87 | 7381.2975 | 821.1514 | 20177.82 | 9 | 7381.3242 | C62 | 62 | -0.0267 | -3.61 |
| 564 | 88 | 4055.7977 | 812.1668 | 19273.14 | 5 | 4055.8103 | C32 | 32 | -0.0126 | -3.12 |
| 564 | 89 | 8395.9632 | 840.6036 | 15341.57 | 10 |  |  |  |  |  |
| 564 | 90 | 7552.6882 | 756.2761 | 17280.60 | 10 | 7553.7019 | Z\_DOT64 | 10 | -0.0113 | -1.50 |
| 564 | 91 | 4771.4468 | 682.6425 | 12102.69 | 7 |  |  |  |  |  |
| 564 | 92 | 5503.3456 | 918.2315 | 17574.33 | 6 | 5503.3559 | C45 | 45 | -0.0104 | -1.88 |
| 564 | 93 | 6523.8328 | 932.9834 | 11912.34 | 7 | 6522.8530 | C54 | 54 | -0.0225 | -3.45 |
| 564 | 94 | 6663.8808 | 833.9924 | 20445.11 | 8 |  |  |  |  |  |
| 564 | 95 | 6170.0675 | 772.2657 | 17757.02 | 8 | 6171.0724 | Z\_DOT53 | 21 | -2.52e-03 | -0.41 |
| 564 | 96 | 8225.9205 | 823.5993 | 19641.50 | 10 |  |  |  |  |  |
| 564 | 97 | 6680.9062 | 836.1206 | 16017.00 | 8 | 6680.9221 | C56 | 56 | -0.0159 | -2.38 |
| 564 | 98 | 1752.7636 | 877.3891 | 22003.58 | 2 | 1752.7671 | C14 | 14 | -3.54e-03 | -2.02 |
| 564 | 99 | 7508.6955 | 751.8768 | 11017.80 | 10 |  |  |  |  |  |
| 564 | 100 | 7267.2578 | 909.4145 | 12727.73 | 8 |  |  |  |  |  |
| 564 | 101 | 7770.5541 | 778.0627 | 18519.15 | 10 | 7769.5716 | C65 | 65 | -0.0198 | -2.55 |
| 564 | 102 | 5757.4886 | 823.5057 | 17771.70 | 7 | 5756.5098 | C47 | 47 | -0.0235 | -4.08 |
| 564 | 103 | 4527.3897 | 755.5722 | 14612.72 | 6 |  |  |  |  |  |
| 564 | 104 | 6209.6683 | 888.1027 | 13268.56 | 7 | 6209.6892 | C51 | 51 | -0.0209 | -3.37 |
| 564 | 105 | 8755.2105 | 973.8084 | 11515.09 | 9 |  |  |  |  |  |
| 564 | 106 | 7898.6266 | 878.6324 | 20359.31 | 9 |  |  |  |  |  |
| 564 | 107 | 8648.1804 | 787.2055 | 20930.82 | 11 |  |  |  |  |  |
| 564 | 108 | 7653.7365 | 766.3809 | 13423.83 | 10 | 7654.7496 | Z\_DOT65 | 9 | -0.0107 | -1.40 |
| 564 | 109 | 8696.1656 | 967.2479 | 10802.07 | 9 |  |  |  |  |  |
| 564 | 110 | 4170.8231 | 835.1719 | 12271.22 | 5 | 4170.8372 | C33 | 33 | -0.0141 | -3.38 |
| 564 | 111 | 6623.8774 | 828.9920 | 15003.09 | 8 | 6623.9007 | C55 | 55 | -0.0232 | -3.51 |
| 564 | 112 | 6645.8652 | 831.7404 | 15532.92 | 8 |  |  |  |  |  |
| 564 | 113 | 3940.7751 | 789.1623 | 16686.18 | 5 | 3940.7834 | C31 | 31 | -8.27e-03 | -2.10 |
| 564 | 114 | 4153.7965 | 693.3067 | 13454.75 | 6 |  |  |  |  |  |
| 564 | 115 | 4899.1233 | 980.8319 | 12020.66 | 5 | 4899.1349 | C40 | 40 | -0.0116 | -2.37 |
| 564 | 116 | 4638.4139 | 663.6378 | 12141.84 | 7 |  |  |  |  |  |
| 564 | 117 | 4511.3654 | 752.9015 | 11835.70 | 6 |  |  |  |  |  |
| 564 | 118 | 7439.6560 | 827.6357 | 21998.33 | 9 | 7439.6590 | Z\_DOT63 | 11 | -3.00e-03 | -0.40 |
| 564 | 119 | 2206.2973 | 736.4397 | 38160.80 | 3 |  |  |  |  |  |
| 564 | 120 | 801.3857 | 802.3929 | 88973.01 | 1 |  |  |  |  |  |
| 564 | 121 | 4440.3486 | 635.3428 | 9522.01 | 7 |  |  |  |  |  |
| 564 | 122 | 5911.9133 | 845.5663 | 10572.85 | 7 |  |  |  |  |  |
| 564 | 123 | 4386.9008 | 878.3874 | 12931.93 | 5 | 4386.9119 | C35 | 35 | -0.0110 | -2.51 |
| 564 | 124 | 5381.6643 | 897.9513 | 9306.33 | 6 |  |  |  |  |  |
| 564 | 125 | 7830.7874 | 712.8970 | 13277.47 | 11 |  |  |  |  |  |
| 564 | 126 | 1356.6505 | 679.3325 | 70542.87 | 2 |  |  |  |  |  |
| 564 | 127 | 1487.6075 | 744.8110 | 7892.73 | 2 | 1487.6133 | C12 | 12 | -5.80e-03 | -3.90 |
| 564 | 128 | 1169.7823 | 585.8984 | 7720.32 | 2 |  |  |  |  |  |
| 564 | 129 | 856.5714 | 857.5787 | 7086.23 | 1 |  |  |  |  |  |
| 564 | 130 | 997.4633 | 998.4706 | 5632.16 | 1 | 997.4651 | C8 | 8 | -1.75e-03 | -1.75 |
| 564 | 131 | 600.3823 | 601.3896 | 6272.38 | 1 |  |  |  |  |  |
| 564 | 132 | 1428.8885 | 477.3034 | 6718.49 | 3 |  |  |  |  |  |
| 564 | 133 | 1386.8782 | 463.3000 | 4426.75 | 3 |  |  |  |  |  |
| 564 | 134 | 1258.5406 | 630.2776 | 7299.13 | 2 | 1258.5434 | C10 | 10 | -2.83e-03 | -2.25 |
| 564 | 135 | 1390.6089 | 696.3117 | 3294.93 | 2 |  |  |  |  |  |
| 564 | 136 | 798.5064 | 400.2605 | 3704.34 | 2 |  |  |  |  |  |
| 564 | 137 | 486.3395 | 487.3468 | 3360.00 | 1 |  |  |  |  |  |
| 564 | 138 | 1415.8819 | 472.9679 | 2436.09 | 3 |  |  |  |  |  |
| 564 | 139 | 1316.8528 | 659.4337 | 3172.84 | 2 |  |  |  |  |  |
| 564 | 140 | 997.4627 | 499.7386 | 2887.18 | 2 | 997.4651 | C8 | 8 | -2.31e-03 | -2.32 |
| 564 | 141 | 953.4521 | 954.4594 | 2745.83 | 1 |  |  |  |  |  |
| 564 | 142 | 502.2438 | 503.2511 | 2758.19 | 1 |  |  |  |  |  |
| 564 | 143 | 1486.9542 | 496.6587 | 2589.30 | 3 |  |  |  |  |  |
| 564 | 144 | 1185.8008 | 593.9077 | 2358.14 | 2 |  |  |  |  |  |
| 564 | 145 | 879.7773 | 880.7846 | 2873.81 | 1 |  |  |  |  |  |
| 564 | 146 | 768.6355 | 769.6428 | 5541.23 | 1 |  |  |  |  |  |

  

All proteins /
CsTx-1a\_S1 Cupiennius salei toxin 1 isoform a S1^ACsTx-1a\_S2 Cupiennius salei toxin 1 isoform a S2 /
Proteoform #6
