# Supplementary material for: The Dual Prey-Inactivation Strategy of Spiders—In-Depth Venomic Analysis of Cupiennius salei
Source: Toxins (Basel). 2019 Mar 19;11(3):167. doi: 10.3390/toxins11030167 (PMC6468893; doi:10.3390/toxins11030167)
Supplement: Supplementary file 1 [file toxins-11-00167-s001.zip › Supplementary Dataset EV1/20180328_f2_topdown_OTMS2_EThcD_NL_i02_ms2_proteoform_cutoff_html/prsms/prsm183.html]

Protein-Spectrum-Match for Spectrum #422


All proteins /
CsTx-12b Cupiennius salei toxin 12 isoform b /
Proteoform #47

## Protein-Spectrum-Match #183 for Spectrum #422

|  |  |  |  |  |  |
| --- | --- | --- | --- | --- | --- |
| PrSM ID: | 183 | Scan(s): | 565 | Precursor charge: | 6 |
| Precursor m/z: | 571.9905 | Precursor mass: | 3425.8993 | Proteoform mass: | 3426.8843 |
| # matched peaks: | 18 | # matched fragment ions: | 18 | # unexpected modifications: | 0 |
| E-value: | 2.14e-19 | P-value: | 2.14e-19 | Q-value (Spectral FDR): | 0 |

  

|  |  |  |  |  |  |  |  |  |  |  |  |  |  |  |  |  |  |  |  |  |  |  |  |  |  |  |  |  |  |  |  |  |  |  |  |  |  |  |  |  |  |  |  |  |  |  |  |  |  |  |  |  |  |  |  |  |  |  |  |  |  |  |  |  |  |  |
| --- | --- | --- | --- | --- | --- | --- | --- | --- | --- | --- | --- | --- | --- | --- | --- | --- | --- | --- | --- | --- | --- | --- | --- | --- | --- | --- | --- | --- | --- | --- | --- | --- | --- | --- | --- | --- | --- | --- | --- | --- | --- | --- | --- | --- | --- | --- | --- | --- | --- | --- | --- | --- | --- | --- | --- | --- | --- | --- | --- | --- | --- | --- | --- | --- | --- | --- |
|  | | ... 30 amino acid residues are skipped at the N-terminus ... | | | | | | | | | | | | | | | | | | | | | | | | | | | | | | | | | | | | | | | | | | | | | | | | | | | | | | | | | | | | | |  | | |
|  | |  | | | | | | | | | | | | | | | | | | | | | | | | | | | | | | | | | | | | | | | | | | | | | | | | | | | | | | | | | | | | | | | | | | | |
| 31 |  |  | S |  | F |  | E |  | A |  | D |  | D |  | V |  | I |  | P |  | F |  |  | L |  | A |  | R |  | E |  | Q |  | V |  | R |  | S |  | D |  | C |  |  | T |  | L |  | R |  | N |  | H |  | D |  | C |  | T |  | D |  | D |  | 60 |  |
|  | |  | | | | | | | | | | | | | | | | | | | | | | | | | | | | | | | | | | | | | | | | | | | | | | | | | | | | | | | | | | | | | | | | | | | |
| 61 |  |  | R |  | H |  | S |  | C |  | C |  | R |  | S |  | K |  | M |  | F |  |  | K |  | D |  | V |  | C |  | K |  | C |  | F |  | Y |  | P |  | S |  |  | Q |  | R |  | S |  | D |  | T |  | A |  | R | ] | A |  | K | ⎩ | K |  | 90 |  |
|  | |  | | | | | | | | | | | | | | | | | | | | | | | | | | | | | | | | | | | | | | | | | | | | | | | | | | | | | | | | | | | | | | | | | | | |
| 91 |  |  | E | ⎫ | L | ⎫ | C |  | T | ⎫ | C | ⎫ | Q | ⎫ | Q |  | D | ⎫ | K |  | H |  |  | L | ⎫ | K | ⎫ | Y |  | I | ⎫ | E | ⎫ | K |  | G | ⎫ | L |  | Q | ⎫ | K |  |  | A | ⎫ | K | ⎫ | V | ⎫ | L | ⎫ | V | ⎫ | A | [ | G |  | | 117 |  | | | | | |

Fixed PTMs: Carbamidomethylation [C93 C95 ]

  

All peaks (57)  Matched peaks (18)  Not matched peaks (39)

  

| Scan | Peak | Mono mass | Mono m/z | Intensity | Charge | Theoretical mass | Ion | Pos | Mass error | PPM error |
| --- | --- | --- | --- | --- | --- | --- | --- | --- | --- | --- |
| 565 | 1 | 3368.8571 | 674.7787 | 91384.14 | 5 |  |  |  |  |  |
| 565 | 2 | 1713.4455 | 572.1558 | 167968.58 | 3 |  |  |  |  |  |
| 565 | 3 | 3424.8860 | 571.8216 | 145775.22 | 6 |  |  |  |  |  |
| 565 | 4 | 2856.6772 | 572.3427 | 110912.82 | 5 |  |  |  |  |  |
| 565 | 5 | 3142.6927 | 786.6804 | 37686.07 | 4 | 3142.7106 | C26 | 26 | -0.0180 | -5.72 |
| 565 | 6 | 2855.6873 | 714.9291 | 36287.48 | 4 |  |  |  |  |  |
| 565 | 7 | 3354.8441 | 671.9761 | 31023.70 | 5 | 3354.8631 | C28 | 28 | -0.0190 | -5.68 |
| 565 | 8 | 3368.8585 | 843.2219 | 29795.50 | 4 |  |  |  |  |  |
| 565 | 9 | 2161.1008 | 721.3742 | 31162.66 | 3 | 2161.1135 | C17 | 17 | -0.0128 | -5.91 |
| 565 | 10 | 2048.2633 | 683.7617 | 28150.97 | 3 |  |  |  |  |  |
| 565 | 11 | 3210.7295 | 803.6897 | 17784.18 | 4 |  |  |  |  |  |
| 565 | 12 | 2475.2588 | 826.0936 | 22298.63 | 3 | 2475.2726 | C20 | 20 | -0.0137 | -5.54 |
| 565 | 13 | 1378.6253 | 690.3199 | 23099.88 | 2 | 1378.6333 | C11 | 11 | -7.99e-03 | -5.79 |
| 565 | 14 | 571.3152 | 572.3225 | 112923.67 | 1 |  |  |  |  |  |
| 565 | 15 | 1884.9552 | 629.3257 | 26114.69 | 3 | 1884.9662 | C15 | 15 | -0.0109 | -5.81 |
| 565 | 16 | 1541.9331 | 771.9738 | 21277.92 | 2 |  |  |  |  |  |
| 565 | 17 | 2290.1427 | 764.3882 | 17521.75 | 3 | 2290.1561 | C18 | 18 | -0.0134 | -5.87 |
| 565 | 18 | 3409.8618 | 682.9796 | 14616.56 | 5 |  |  |  |  |  |
| 565 | 19 | 3408.8558 | 569.1499 | 12539.91 | 6 |  |  |  |  |  |
| 565 | 20 | 3338.8220 | 668.7717 | 15465.17 | 5 |  |  |  |  |  |
| 565 | 21 | 3382.8734 | 846.7256 | 14645.12 | 4 |  |  |  |  |  |
| 565 | 22 | 2716.4007 | 906.4742 | 12016.43 | 3 | 2716.4152 | C22 | 22 | -0.0145 | -5.34 |
| 565 | 23 | 1265.7875 | 633.9010 | 13794.30 | 2 |  |  |  |  |  |
| 565 | 24 | 3338.8256 | 835.7137 | 15836.66 | 4 |  |  |  |  |  |
| 565 | 25 | 2361.3758 | 591.3512 | 10578.33 | 4 |  |  |  |  |  |
| 565 | 26 | 2915.5301 | 729.8898 | 13851.82 | 4 | 2915.5473 | C24 | 24 | -0.0172 | -5.89 |
| 565 | 27 | 3381.8664 | 677.3806 | 11059.68 | 5 |  |  |  |  |  |
| 565 | 28 | 2456.4162 | 819.8127 | 9835.43 | 3 |  |  |  |  |  |
| 565 | 29 | 3043.6241 | 761.9133 | 10029.26 | 4 | 3043.6422 | C25 | 25 | -0.0182 | -5.97 |
| 565 | 30 | 1756.8620 | 879.4383 | 10010.40 | 2 | 1756.8712 | C14 | 14 | -9.23e-03 | -5.25 |
| 565 | 31 | 908.5761 | 455.2953 | 10715.30 | 2 |  |  |  |  |  |
| 565 | 32 | 3255.7754 | 814.9511 | 8702.99 | 4 | 3255.7947 | C27 | 27 | -0.0193 | -5.92 |
| 565 | 33 | 3410.8712 | 853.7251 | 10752.24 | 4 |  |  |  |  |  |
| 565 | 34 | 1557.9516 | 779.9831 | 11614.35 | 2 |  |  |  |  |  |
| 565 | 35 | 2498.5363 | 833.8527 | 10957.36 | 3 |  |  |  |  |  |
| 565 | 36 | 3211.7382 | 1071.5867 | 6711.67 | 3 | 3211.7335 | Z\_DOT27 | 2 | 4.60e-03 | 1.43 |
| 565 | 37 | 1336.9209 | 669.4677 | 6582.79 | 2 |  |  |  |  |  |
| 565 | 38 | 685.5777 | 686.5850 | 39660.46 | 1 |  |  |  |  |  |
| 565 | 39 | 1223.8376 | 612.9261 | 10437.35 | 2 |  |  |  |  |  |
| 565 | 40 | 1095.7433 | 548.8789 | 8029.35 | 2 |  |  |  |  |  |
| 565 | 41 | 1007.4841 | 1008.4913 | 7834.75 | 1 | 1007.4892 | C8 | 8 | -5.11e-03 | -5.07 |
| 565 | 42 | 1179.8243 | 590.9194 | 3902.34 | 2 |  |  |  |  |  |
| 565 | 43 | 1206.7741 | 604.3943 | 3468.56 | 2 |  |  |  |  |  |
| 565 | 44 | 673.9729 | 674.9801 | 9336.77 | 1 |  |  |  |  |  |
| 565 | 45 | 710.4891 | 711.4963 | 4837.01 | 1 |  |  |  |  |  |
| 565 | 46 | 847.4538 | 848.4611 | 3769.62 | 1 | 847.4585 | C7 | 7 | -4.67e-03 | -5.51 |
| 565 | 47 | 641.4189 | 642.4261 | 3415.27 | 1 |  |  |  |  |  |
| 565 | 48 | 1419.8338 | 710.9242 | 3041.69 | 2 |  |  |  |  |  |
| 565 | 49 | 1135.5416 | 1136.5489 | 3229.19 | 1 | 1135.5477 | C9 | 9 | -6.14e-03 | -5.40 |
| 565 | 50 | 400.2782 | 401.2855 | 2713.00 | 1 |  |  |  |  |  |
| 565 | 51 | 517.2836 | 518.2908 | 2852.62 | 1 |  |  |  |  |  |
| 565 | 52 | 553.0760 | 554.0833 | 3402.08 | 1 |  |  |  |  |  |
| 565 | 53 | 1349.8340 | 675.9243 | 2445.80 | 2 |  |  |  |  |  |
| 565 | 54 | 1065.5227 | 1066.5300 | 2207.18 | 1 |  |  |  |  |  |
| 565 | 55 | 473.2939 | 474.3012 | 3070.71 | 1 | 473.2961 | C4 | 4 | -2.19e-03 | -4.62 |
| 565 | 56 | 586.3773 | 587.3846 | 2672.11 | 1 | 586.3802 | C5 | 5 | -2.87e-03 | -4.90 |
| 565 | 57 | 780.5183 | 391.2664 | 1785.39 | 2 |  |  |  |  |  |

  

All proteins /
CsTx-12b Cupiennius salei toxin 12 isoform b /
Proteoform #47
