# Supplementary material for: The Dual Prey-Inactivation Strategy of Spiders—In-Depth Venomic Analysis of Cupiennius salei
Source: Toxins (Basel). 2019 Mar 19;11(3):167. doi: 10.3390/toxins11030167 (PMC6468893; doi:10.3390/toxins11030167)
Supplement: Supplementary file 1 [file toxins-11-00167-s001.zip › Supplementary Dataset EV1/20180328_f2_topdown_OTMS2_EThcD_NL_i02_ms2_proteoform_cutoff_html/prsms/prsm184.html]

Protein-Spectrum-Match for Spectrum #423


All proteins /
CsTx-1a\_S1 Cupiennius salei toxin 1 isoform a S1^ACsTx-1a\_S2 Cupiennius salei toxin 1 isoform a S2 /
Proteoform #6

## Protein-Spectrum-Match #184 for Spectrum #423

|  |  |  |  |  |  |
| --- | --- | --- | --- | --- | --- |
| PrSM ID: | 184 | Scan(s): | 567 | Precursor charge: | 12 |
| Precursor m/z: | 735.1946 | Precursor mass: | 8810.2476 | Proteoform mass: | 8810.2426 |
| # matched peaks: | 57 | # matched fragment ions: | 45 | # unexpected modifications: | 1 |
| E-value: | 4.49e-37 | P-value: | 4.49e-37 | Q-value (Spectral FDR): | 0 |

  

|  |  |  |  |  |  |  |  |  |  |  |  |  |  |  |  |  |  |  |  |  |  |  |  |  |  |  |  |  |  |  |  |  |  |  |  |  |  |  |  |  |  |  |  |  |  |  |  |  |  |  |  |  |  |  |  |  |  |  |  |  |  |  |  |  |  |  |  |  |  |
| --- | --- | --- | --- | --- | --- | --- | --- | --- | --- | --- | --- | --- | --- | --- | --- | --- | --- | --- | --- | --- | --- | --- | --- | --- | --- | --- | --- | --- | --- | --- | --- | --- | --- | --- | --- | --- | --- | --- | --- | --- | --- | --- | --- | --- | --- | --- | --- | --- | --- | --- | --- | --- | --- | --- | --- | --- | --- | --- | --- | --- | --- | --- | --- | --- | --- | --- | --- | --- | --- |
|  | |  | | | | | | | | | | | | | | | | | | | | | | | | | | | | | | | | | | | | | | | | | | | | | | | | | | | | | | | | | | | | | | | | | | | |
| 1 |  |  | M |  | K |  | V |  | L |  | I |  | I |  | S |  | A |  | V |  | L |  |  | F |  | I |  | T |  | I |  | F |  | S |  | N |  | I |  | S |  | A |  |  | E |  | I |  | E |  | D |  | D |  | F |  | L |  | E |  | D |  | E |  | 30 |  |
|  | |  | | | | | | | | | | | | | | | | | | | | | | | | | | | | | | | | | | | | | | | | | | | | | | | | | | | | | | | | | | | | | | | | | | | |
| 31 |  |  | S |  | F |  | E |  | A |  | E |  | D |  | I |  | I |  | P |  | F |  |  | F |  | E |  | N |  | E |  | Q |  | A |  | R | ] | S | ⎩ | C |  | I |  |  | P |  | K |  | H |  | E |  | E | ⎫ | C | ⎩ | T | ⎱ | N | ⎱ | D | ⎫ | K |  | 60 |  |
|  | |  | | | | | | | | | | | | | | | | | | | | | | | | | | | | | | | | | | | | | | | | | | | | | | | | | | | | | | | | | | | | | | | | | | | |
| 61 |  |  | H | ⎩ | N |  | C |  | C |  | R |  | K |  | G | ⎩ | L | ⎩ | F | ⎱ | K |  |  | L |  | K |  | C | ⎫ | Q |  | C |  | S |  | T |  | F | ⎫ | D | ⎫ | D |  | ⎱ | E | ⎫ | S |  | G | ⎱ | Q |  | P |  | T | ⎫ | E |  | R |  | C |  | A |  | 90 |  |
|  | |  | | | | | | | | | | | | | | | | | | | | | | | | | | | | | | | | | | | | | | | | | | | | | | | | | | | | | | | | | | | | | -58.02 | | | |
| 91 |  |  | C |  | G | ⎫ | R |  | P | ⎫ | M |  | G |  | H | ⎫ | Q | ⎫ | A |  | I |  |  | E | ⎫ | T | ⎫ | G | ⎫ | L | ⎫ | N |  | I |  | F |  | R | ⎫ | G | ⎫ | L |  | ⎩ | F | ⎩ | K | ⎫ | G | ⎫ | K | ⎱ | K | ⎫ | K | ⎫ | N | ⎫ | K | ⎫ | K | ⎫ | T |  | 120 |  |
|  | |  | | | | | | | | | | | | | | | | | | | | | | | | | | | | | | | | | | | | | | | | | | | | | | | | | | | | | | | | | | | | | | | | | | | |
| 121 |  | ⎫ | K | ⎫ | G |  | | | | 122 |  | | | | | | | | | | | | | | | | | | | | | | | | | | | | | | | | | | | | | | | | | | | | | | | | | | | | | | | |

Fixed PTMs: Carbamidomethylation [C49 C56 C63 C64 C73 C75 C89 C91 ]   
  
     Unexpected modifications:   Unknown [-58.02]

  

All peaks (146)  Matched peaks (57)  Not matched peaks (89)

  

| Scan | Peak | Mono mass | Mono m/z | Intensity | Charge | Theoretical mass | Ion | Pos | Mass error | PPM error |
| --- | --- | --- | --- | --- | --- | --- | --- | --- | --- | --- |
| 567 | 1 | 8753.2235 | 796.7549 | 826980.67 | 11 | 8752.2371 | C74 | 74 | -0.0159 | -1.82 |
| 567 | 2 | 8753.2140 | 876.3287 | 442454.00 | 10 | 8752.2371 | C74 | 74 | -0.0254 | -2.90 |
| 567 | 3 | 8767.2071 | 798.0261 | 273774.07 | 11 |  |  |  |  |  |
| 567 | 4 | 8737.2277 | 795.3007 | 226426.93 | 11 |  |  |  |  |  |
| 567 | 5 | 8794.2251 | 800.4823 | 224086.74 | 11 |  |  |  |  |  |
| 567 | 6 | 8682.1484 | 869.2221 | 204512.51 | 10 |  |  |  |  |  |
| 567 | 7 | 8767.2106 | 877.7283 | 194839.63 | 10 |  |  |  |  |  |
| 567 | 8 | 8795.2081 | 880.5281 | 152228.25 | 10 |  |  |  |  |  |
| 567 | 9 | 8737.1978 | 874.7271 | 109546.36 | 10 |  |  |  |  |  |
| 567 | 10 | 8324.8938 | 833.4967 | 113053.19 | 10 | 8324.9209 | C70 | 70 | -0.0270 | -3.24 |
| 567 | 11 | 4404.6202 | 735.1106 | 827455.61 | 6 |  |  |  |  |  |
| 567 | 12 | 4406.1147 | 882.2302 | 233382.58 | 5 |  |  |  |  |  |
| 567 | 13 | 8210.8546 | 822.0927 | 89275.43 | 10 | 8210.8779 | C69 | 69 | -0.0234 | -2.85 |
| 567 | 14 | 8452.9889 | 846.3062 | 90995.74 | 10 | 8453.0158 | C71 | 71 | -0.0269 | -3.18 |
| 567 | 15 | 8696.1859 | 870.6259 | 110160.48 | 10 |  |  |  |  |  |
| 567 | 16 | 8082.7601 | 899.0917 | 89520.01 | 9 | 8082.7830 | C68 | 68 | -0.0229 | -2.83 |
| 567 | 17 | 8709.1862 | 871.9259 | 79716.50 | 10 |  |  |  |  |  |
| 567 | 18 | 7954.6578 | 884.8581 | 70919.28 | 9 | 7954.6880 | C67 | 67 | -0.0302 | -3.80 |
| 567 | 19 | 8720.2343 | 793.7558 | 70375.18 | 11 |  |  |  |  |  |
| 567 | 20 | 8581.0913 | 859.1164 | 68598.74 | 10 | 8581.1108 | C72 | 72 | -0.0195 | -2.27 |
| 567 | 21 | 7723.7718 | 773.3845 | 65948.63 | 10 |  |  |  |  |  |
| 567 | 22 | 8706.2285 | 792.4826 | 69954.97 | 11 | 8707.1919 | Z\_DOT74 | 1 | 0.0389 | 4.47 |
| 567 | 23 | 7324.2845 | 916.5428 | 56799.18 | 8 | 7324.3027 | C61 | 61 | -0.0182 | -2.48 |
| 567 | 24 | 6854.4258 | 762.6101 | 49407.70 | 9 |  |  |  |  |  |
| 567 | 25 | 4443.9194 | 889.7912 | 57591.14 | 5 | 4443.9333 | C36 | 36 | -0.0139 | -3.13 |
| 567 | 26 | 8224.9378 | 823.5011 | 65261.43 | 10 |  |  |  |  |  |
| 567 | 27 | 8662.2265 | 788.4824 | 48737.16 | 11 |  |  |  |  |  |
| 567 | 28 | 8754.2168 | 973.6980 | 39133.08 | 9 |  |  |  |  |  |
| 567 | 29 | 8082.7381 | 809.2811 | 40565.48 | 10 | 8082.7830 | C68 | 68 | -0.0448 | -5.54 |
| 567 | 30 | 8722.2045 | 873.2277 | 50075.96 | 10 |  |  |  |  |  |
| 567 | 31 | 8792.2685 | 733.6963 | 70930.98 | 12 |  |  |  |  |  |
| 567 | 32 | 4554.9540 | 760.1663 | 40517.60 | 6 |  |  |  |  |  |
| 567 | 33 | 7829.8026 | 783.9875 | 47880.80 | 10 |  |  |  |  |  |
| 567 | 34 | 4255.2765 | 710.2200 | 44224.20 | 6 |  |  |  |  |  |
| 567 | 35 | 8059.9879 | 807.0061 | 54823.80 | 10 |  |  |  |  |  |
| 567 | 36 | 8060.0592 | 733.7399 | 147329.86 | 11 |  |  |  |  |  |
| 567 | 37 | 7669.7648 | 767.9838 | 31771.45 | 10 |  |  |  |  |  |
| 567 | 38 | 8682.1438 | 965.6899 | 30693.83 | 9 |  |  |  |  |  |
| 567 | 39 | 4309.2897 | 719.2222 | 45743.90 | 6 |  |  |  |  |  |
| 567 | 40 | 7769.5503 | 864.2906 | 32430.27 | 9 | 7769.5716 | C65 | 65 | -0.0213 | -2.74 |
| 567 | 41 | 8324.9025 | 925.9964 | 28451.62 | 9 | 8324.9209 | C70 | 70 | -0.0183 | -2.20 |
| 567 | 42 | 6623.8760 | 828.9918 | 28907.22 | 8 | 6623.9007 | C55 | 55 | -0.0247 | -3.73 |
| 567 | 43 | 8662.1962 | 867.2269 | 33352.50 | 10 |  |  |  |  |  |
| 567 | 44 | 6209.6636 | 888.1021 | 27753.90 | 7 | 6209.6892 | C51 | 51 | -0.0256 | -4.12 |
| 567 | 45 | 4770.0836 | 955.0240 | 28385.88 | 5 | 4770.0923 | C39 | 39 | -8.72e-03 | -1.83 |
| 567 | 46 | 6793.9902 | 850.2560 | 28844.73 | 8 | 6794.0062 | C57 | 57 | -0.0160 | -2.36 |
| 567 | 47 | 801.3865 | 802.3937 | 368425.76 | 1 |  |  |  |  |  |
| 567 | 48 | 6968.4892 | 775.2838 | 29154.96 | 9 |  |  |  |  |  |
| 567 | 49 | 8211.8472 | 913.4347 | 30314.04 | 9 | 8210.8779 | C69 | 69 | -0.0331 | -4.03 |
| 567 | 50 | 5503.3492 | 918.2321 | 25679.94 | 6 | 5503.3559 | C45 | 45 | -6.69e-03 | -1.22 |
| 567 | 51 | 7439.6611 | 827.6363 | 24342.11 | 9 | 7438.6641 | Z\_DOT64 | 11 | -5.36e-03 | -0.72 |
| 567 | 52 | 8224.9308 | 914.8885 | 21999.78 | 9 |  |  |  |  |  |
| 567 | 53 | 3982.1436 | 664.6979 | 30410.11 | 6 |  |  |  |  |  |
| 567 | 54 | 7724.7803 | 859.3162 | 25672.07 | 9 |  |  |  |  |  |
| 567 | 55 | 4527.3867 | 755.5717 | 21054.61 | 6 |  |  |  |  |  |
| 567 | 56 | 6522.8374 | 932.8412 | 24187.57 | 7 | 6522.8530 | C54 | 54 | -0.0156 | -2.39 |
| 567 | 57 | 5910.9255 | 845.4252 | 25995.45 | 7 |  |  |  |  |  |
| 567 | 58 | 7058.4848 | 785.2834 | 21872.25 | 9 | 7058.4833 | Z\_DOT61 | 14 | 1.46e-03 | 0.21 |
| 567 | 59 | 8696.1788 | 967.2494 | 20269.74 | 9 |  |  |  |  |  |
| 567 | 60 | 8618.1950 | 862.8268 | 29985.92 | 10 |  |  |  |  |  |
| 567 | 61 | 8625.1072 | 959.3525 | 19951.34 | 9 | 8624.1421 | C73 | 73 | -0.0373 | -4.32 |
| 567 | 62 | 6854.4360 | 857.8118 | 25361.74 | 8 |  |  |  |  |  |
| 567 | 63 | 7348.6504 | 817.5240 | 20035.11 | 9 |  |  |  |  |  |
| 567 | 64 | 4299.8711 | 860.9815 | 21468.89 | 5 | 4299.8798 | C34 | 34 | -8.71e-03 | -2.03 |
| 567 | 65 | 6680.8901 | 836.1185 | 17762.43 | 8 | 6680.9221 | C56 | 56 | -0.0320 | -4.79 |
| 567 | 66 | 8676.2188 | 789.7544 | 29428.56 | 11 |  |  |  |  |  |
| 567 | 67 | 4554.9576 | 911.9988 | 19585.66 | 5 |  |  |  |  |  |
| 567 | 68 | 734.8443 | 735.8516 | 139744.59 | 1 |  |  |  |  |  |
| 567 | 69 | 6694.3902 | 744.8284 | 22556.16 | 9 |  |  |  |  |  |
| 567 | 70 | 7826.5940 | 870.6288 | 23964.57 | 9 | 7826.5930 | C66 | 66 | 9.65e-04 | 0.12 |
| 567 | 71 | 3317.5367 | 830.3914 | 16210.69 | 4 | 3317.5460 | C26 | 26 | -9.33e-03 | -2.81 |
| 567 | 72 | 4170.8242 | 835.1721 | 16301.73 | 5 | 4170.8372 | C33 | 33 | -0.0130 | -3.13 |
| 567 | 73 | 6082.6306 | 869.9545 | 36165.02 | 7 | 6081.6306 | C50 | 50 | -2.41e-03 | -0.40 |
| 567 | 74 | 7883.8109 | 789.3884 | 18285.29 | 10 |  |  |  |  |  |
| 567 | 75 | 6240.0997 | 781.0197 | 14563.89 | 8 |  |  |  |  |  |
| 567 | 76 | 8452.9838 | 940.2277 | 15395.05 | 9 | 8453.0158 | C71 | 71 | -0.0320 | -3.78 |
| 567 | 77 | 7669.7664 | 853.2035 | 21436.13 | 9 |  |  |  |  |  |
| 567 | 78 | 5756.5115 | 823.3661 | 28023.30 | 7 | 5756.5098 | C47 | 47 | 1.73e-03 | 0.30 |
| 567 | 79 | 6283.1504 | 898.6002 | 19242.92 | 7 | 6283.1615 | Z\_DOT55 | 20 | -0.0112 | -1.78 |
| 567 | 80 | 8003.0044 | 801.3077 | 44343.67 | 10 |  |  |  |  |  |
| 567 | 81 | 1603.1274 | 802.5710 | 461332.12 | 2 |  |  |  |  |  |
| 567 | 82 | 8450.0791 | 769.1963 | 14059.03 | 11 |  |  |  |  |  |
| 567 | 83 | 4886.4780 | 815.4203 | 20766.64 | 6 |  |  |  |  |  |
| 567 | 84 | 7325.2948 | 814.9289 | 17413.28 | 9 | 7324.3027 | C61 | 61 | -0.0102 | -1.39 |
| 567 | 85 | 7653.7385 | 851.4227 | 13510.67 | 9 | 7653.7547 | Z\_DOT66 | 9 | -0.0163 | -2.13 |
| 567 | 86 | 4055.7943 | 812.1661 | 15653.23 | 5 | 4055.8103 | C32 | 32 | -0.0160 | -3.94 |
| 567 | 87 | 8648.1941 | 865.8267 | 18231.28 | 10 |  |  |  |  |  |
| 567 | 88 | 8152.8435 | 906.8788 | 13168.94 | 9 |  |  |  |  |  |
| 567 | 89 | 8710.1875 | 968.8059 | 14140.23 | 9 |  |  |  |  |  |
| 567 | 90 | 5563.7451 | 928.2981 | 14755.02 | 6 |  |  |  |  |  |
| 567 | 91 | 4640.4141 | 774.4096 | 11757.15 | 6 | 4640.4132 | Z\_DOT42 | 33 | 8.78e-04 | 0.19 |
| 567 | 92 | 7394.6566 | 822.6358 | 17175.00 | 9 |  |  |  |  |  |
| 567 | 93 | 8267.8725 | 919.6598 | 17734.62 | 9 |  |  |  |  |  |
| 567 | 94 | 8718.2184 | 727.5255 | 15524.39 | 12 |  |  |  |  |  |
| 567 | 95 | 4367.3264 | 728.8950 | 14706.97 | 6 | 4367.3171 | Z\_DOT39 | 36 | 9.31e-03 | 2.13 |
| 567 | 96 | 2788.2374 | 930.4197 | 14466.62 | 3 | 2788.2414 | C22 | 22 | -4.00e-03 | -1.44 |
| 567 | 97 | 6170.0665 | 772.2656 | 10563.13 | 8 | 6170.0775 | Z\_DOT54 | 21 | -0.0110 | -1.78 |
| 567 | 98 | 8767.2228 | 975.1431 | 15977.51 | 9 |  |  |  |  |  |
| 567 | 99 | 7307.2489 | 914.4134 | 11329.74 | 8 |  |  |  |  |  |
| 567 | 100 | 3434.9475 | 687.9968 | 12698.79 | 5 |  |  |  |  |  |
| 567 | 101 | 8795.2244 | 978.2544 | 13164.83 | 9 |  |  |  |  |  |
| 567 | 102 | 4898.1185 | 980.6310 | 15428.59 | 5 |  |  |  |  |  |
| 567 | 103 | 8775.2173 | 732.2754 | 10528.26 | 12 |  |  |  |  |  |
| 567 | 104 | 7553.7019 | 840.3075 | 12758.20 | 9 | 7552.7070 | Z\_DOT65 | 10 | -7.49e-03 | -0.99 |
| 567 | 105 | 4581.3977 | 764.5736 | 9601.01 | 6 |  |  |  |  |  |
| 567 | 106 | 7265.2572 | 909.1644 | 13656.41 | 8 |  |  |  |  |  |
| 567 | 107 | 7477.3643 | 935.6778 | 11872.75 | 8 |  |  |  |  |  |
| 567 | 108 | 5032.5504 | 719.9430 | 11908.72 | 7 |  |  |  |  |  |
| 567 | 109 | 6693.3960 | 837.6818 | 15438.19 | 8 |  |  |  |  |  |
| 567 | 110 | 8353.0335 | 836.3106 | 22555.48 | 10 |  |  |  |  |  |
| 567 | 111 | 6023.0101 | 861.4373 | 19177.31 | 7 | 6023.0091 | Z\_DOT53 | 22 | 1.02e-03 | 0.17 |
| 567 | 112 | 3940.7824 | 789.1638 | 11870.19 | 5 | 3940.7834 | C31 | 31 | -9.41e-04 | -0.24 |
| 567 | 113 | 4440.3446 | 741.0647 | 15425.59 | 6 |  |  |  |  |  |
| 567 | 114 | 8581.0890 | 954.4616 | 19798.08 | 9 | 8581.1108 | C72 | 72 | -0.0218 | -2.54 |
| 567 | 115 | 7850.8262 | 786.0899 | 11151.53 | 10 |  |  |  |  |  |
| 567 | 116 | 6623.8832 | 947.2763 | 9977.36 | 7 | 6623.9007 | C55 | 55 | -0.0175 | -2.64 |
| 567 | 117 | 3854.0862 | 643.3550 | 13801.91 | 6 |  |  |  |  |  |
| 567 | 118 | 4367.3250 | 874.4723 | 13351.92 | 5 | 4367.3171 | Z\_DOT39 | 36 | 7.92e-03 | 1.81 |
| 567 | 119 | 8724.1961 | 970.3624 | 7361.43 | 9 |  |  |  |  |  |
| 567 | 120 | 6793.9916 | 971.5775 | 12178.80 | 7 | 6794.0062 | C57 | 57 | -0.0146 | -2.15 |
| 567 | 121 | 7382.3054 | 923.7954 | 10758.13 | 8 | 7381.3242 | C62 | 62 | -0.0211 | -2.86 |
| 567 | 122 | 7233.5914 | 804.7397 | 8272.11 | 9 |  |  |  |  |  |
| 567 | 123 | 7058.4702 | 883.3161 | 14931.94 | 8 | 7058.4833 | Z\_DOT61 | 14 | -0.0131 | -1.85 |
| 567 | 124 | 1372.5835 | 687.2990 | 15436.79 | 2 | 1372.5863 | C11 | 11 | -2.79e-03 | -2.03 |
| 567 | 125 | 882.2242 | 883.2315 | 14319.86 | 1 |  |  |  |  |  |
| 567 | 126 | 1169.7824 | 585.8985 | 6856.42 | 2 | 1169.7738 | Z\_DOT11 | 64 | 8.58e-03 | 7.33 |
| 567 | 127 | 1428.8892 | 477.3037 | 4939.82 | 3 |  |  |  |  |  |
| 567 | 128 | 856.5722 | 857.5795 | 6802.46 | 1 | 856.5624 | Z\_DOT8 | 67 | 9.77e-03 | 11.40 |
| 567 | 129 | 898.1968 | 899.2041 | 7966.78 | 1 |  |  |  |  |  |
| 567 | 130 | 997.4635 | 998.4708 | 6265.86 | 1 | 997.4651 | C8 | 8 | -1.51e-03 | -1.51 |
| 567 | 131 | 600.3826 | 601.3899 | 3670.10 | 1 |  |  |  |  |  |
| 567 | 132 | 934.2082 | 935.2155 | 2536.40 | 1 |  |  |  |  |  |
| 567 | 133 | 961.9057 | 962.9130 | 1923.82 | 1 |  |  |  |  |  |
| 567 | 134 | 1057.7069 | 529.8607 | 1757.20 | 2 |  |  |  |  |  |
| 567 | 135 | 946.4104 | 947.4177 | 1469.17 | 1 |  |  |  |  |  |
| 567 | 136 | 428.2743 | 429.2816 | 1571.89 | 1 |  |  |  |  |  |
| 567 | 137 | 1386.8798 | 463.3005 | 1411.17 | 3 |  |  |  |  |  |
| 567 | 138 | 1487.6081 | 744.8113 | 2750.92 | 2 | 1487.6133 | C12 | 12 | -5.19e-03 | -3.49 |
| 567 | 139 | 542.3173 | 543.3246 | 1814.82 | 1 |  |  |  |  |  |
| 567 | 140 | 1316.8503 | 439.9574 | 1111.03 | 3 | 1316.8422 | Z\_DOT12 | 63 | 8.06e-03 | 6.12 |
| 567 | 141 | 983.6225 | 492.8185 | 1123.54 | 2 |  |  |  |  |  |
| 567 | 142 | 1185.8006 | 593.9076 | 1785.08 | 2 |  |  |  |  |  |
| 567 | 143 | 486.3399 | 487.3472 | 1999.65 | 1 |  |  |  |  |  |
| 567 | 144 | 1258.5414 | 630.2780 | 1425.88 | 2 | 1258.5434 | C10 | 10 | -1.97e-03 | -1.57 |
| 567 | 145 | 1061.1754 | 1062.1827 | 1054.53 | 1 |  |  |  |  |  |
| 567 | 146 | 1258.5409 | 1259.5482 | 971.98 | 1 | 1258.5434 | C10 | 10 | -2.51e-03 | -1.99 |

  

All proteins /
CsTx-1a\_S1 Cupiennius salei toxin 1 isoform a S1^ACsTx-1a\_S2 Cupiennius salei toxin 1 isoform a S2 /
Proteoform #6
