# Supplementary material for: The Dual Prey-Inactivation Strategy of Spiders—In-Depth Venomic Analysis of Cupiennius salei
Source: Toxins (Basel). 2019 Mar 19;11(3):167. doi: 10.3390/toxins11030167 (PMC6468893; doi:10.3390/toxins11030167)
Supplement: Supplementary file 1 [file toxins-11-00167-s001.zip › Supplementary Dataset EV1/20180328_f2_topdown_OTMS2_EThcD_NL_i02_ms2_proteoform_cutoff_html/prsms/prsm185.html]

Protein-Spectrum-Match for Spectrum #424


All proteins /
CsTx-1b Cupiennius salei toxin 1 isoform b /
Proteoform #9

## Protein-Spectrum-Match #185 for Spectrum #424

|  |  |  |  |  |  |
| --- | --- | --- | --- | --- | --- |
| PrSM ID: | 185 | Scan(s): | 568 | Precursor charge: | 14 |
| Precursor m/z: | 630.3105 | Precursor mass: | 8810.2455 | Proteoform mass: | 8811.2375 |
| # matched peaks: | 51 | # matched fragment ions: | 38 | # unexpected modifications: | 0 |
| E-value: | 2.69e-34 | P-value: | 2.69e-34 | Q-value (Spectral FDR): | 0 |

  

|  |  |  |  |  |  |  |  |  |  |  |  |  |  |  |  |  |  |  |  |  |  |  |  |  |  |  |  |  |  |  |  |  |  |  |  |  |  |  |  |  |  |  |  |  |  |  |  |  |  |  |  |  |  |  |  |  |  |  |  |  |  |  |  |  |  |  |  |  |  |
| --- | --- | --- | --- | --- | --- | --- | --- | --- | --- | --- | --- | --- | --- | --- | --- | --- | --- | --- | --- | --- | --- | --- | --- | --- | --- | --- | --- | --- | --- | --- | --- | --- | --- | --- | --- | --- | --- | --- | --- | --- | --- | --- | --- | --- | --- | --- | --- | --- | --- | --- | --- | --- | --- | --- | --- | --- | --- | --- | --- | --- | --- | --- | --- | --- | --- | --- | --- | --- | --- |
|  | |  | | | | | | | | | | | | | | | | | | | | | | | | | | | | | | | | | | | | | | | | | | | | | | | | | | | | | | | | | | | | | | | | | | | |
| 1 |  |  | M |  | K |  | V |  | L |  | I |  | I |  | S |  | A |  | V |  | L |  |  | F |  | I |  | T |  | I |  | F |  | S |  | N |  | I |  | S |  | A |  |  | E |  | I |  | E |  | D |  | D |  | F |  | L |  | E |  | D |  | E |  | 30 |  |
|  | |  | | | | | | | | | | | | | | | | | | | | | | | | | | | | | | | | | | | | | | | | | | | | | | | | | | | | | | | | | | | | | | | | | | | |
| 31 |  |  | S |  | F |  | E |  | A |  | E |  | D |  | I |  | I |  | P |  | F |  |  | L |  | E |  | N |  | E |  | Q |  | A |  | R | ] | S | ⎩ | C |  | I |  |  | P |  | K | ⎩ | H |  | E |  | E | ⎫ | C | ⎫ | T | ⎩ | N | ⎫ | D | ⎫ | K |  | 60 |  |
|  | |  | | | | | | | | | | | | | | | | | | | | | | | | | | | | | | | | | | | | | | | | | | | | | | | | | | | | | | | | | | | | | | | | | | | |
| 61 |  | ⎫ | H | ⎱ | N |  | C |  | C |  | R |  | K | ⎱ | G | ⎱ | L | ⎫ | F | ⎫ | K |  |  | L |  | K | ⎫ | C | ⎫ | Q | ⎫ | C |  | S |  | T |  | F |  | D | ⎫ | D |  | ⎫ | E | ⎫ | S |  | G | ⎫ | Q |  | P |  | T | ⎫ | E |  | R |  | C |  | A |  | 90 |  |
|  | |  | | | | | | | | | | | | | | | | | | | | | | | | | | | | | | | | | | | | | | | | | | | | | | | | | | | | | | | | | | | | | | | | | | | |
| 91 |  |  | C |  | G |  | R |  | P |  | M |  | G |  | H | ⎫ | Q | ⎫ | A |  | I |  |  | E | ⎫ | T | ⎫ | G |  | L |  | N |  | I |  | F |  | R | ⎫ | G | ⎫ | L |  |  | F |  | K | ⎫ | G | ⎫ | K | ⎫ | K | ⎫ | K | ⎫ | N | ⎫ | K | ⎫ | K |  | T |  | 120 |  |
|  | |  | | | | | | | | | | | | | | | | | | | | | | | | | | | | | | | | | | | | | | | | | | | | | | | | | | | | | | | | | | | | | | | | | | | |
| 121 |  | ⎫ | K | [ | G |  | | | | 122 |  | | | | | | | | | | | | | | | | | | | | | | | | | | | | | | | | | | | | | | | | | | | | | | | | | | | | | | | |

Fixed PTMs: Carbamidomethylation [C49 C56 C63 C64 C73 C75 C89 C91 ]

  

All peaks (146)  Matched peaks (51)  Not matched peaks (95)

  

| Scan | Peak | Mono mass | Mono m/z | Intensity | Charge | Theoretical mass | Ion | Pos | Mass error | PPM error |
| --- | --- | --- | --- | --- | --- | --- | --- | --- | --- | --- |
| 568 | 1 | 8753.2044 | 730.4410 | 283051.80 | 12 |  |  |  |  |  |
| 568 | 2 | 8753.1980 | 796.7525 | 166149.75 | 11 |  |  |  |  |  |
| 568 | 3 | 8753.2078 | 674.3310 | 162622.28 | 13 |  |  |  |  |  |
| 568 | 4 | 8767.1973 | 731.6071 | 114238.69 | 12 |  |  |  |  |  |
| 568 | 5 | 8793.2140 | 677.4083 | 109853.40 | 13 |  |  |  |  |  |
| 568 | 6 | 8794.2068 | 733.8578 | 119800.44 | 12 |  |  |  |  |  |
| 568 | 7 | 8737.1980 | 729.1071 | 99627.26 | 12 |  |  |  |  |  |
| 568 | 8 | 4554.9511 | 760.1658 | 95007.94 | 6 |  |  |  |  |  |
| 568 | 9 | 4443.9174 | 741.6602 | 91004.55 | 6 | 4443.9333 | C36 | 36 | -0.0159 | -3.58 |
| 568 | 10 | 8737.1995 | 795.2981 | 76273.01 | 11 |  |  |  |  |  |
| 568 | 11 | 8682.1438 | 790.2931 | 71978.44 | 11 | 8682.1585 | C73 | 73 | -0.0147 | -1.69 |
| 568 | 12 | 8695.1841 | 725.6060 | 73506.84 | 12 |  |  |  |  |  |
| 568 | 13 | 4527.3855 | 647.7766 | 78029.98 | 7 |  |  |  |  |  |
| 568 | 14 | 8696.1765 | 791.5688 | 74493.86 | 11 |  |  |  |  |  |
| 568 | 15 | 8736.2009 | 673.0227 | 69535.46 | 13 |  |  |  |  |  |
| 568 | 16 | 4406.1122 | 735.3593 | 167640.36 | 6 |  |  |  |  |  |
| 568 | 17 | 8709.1932 | 726.7734 | 55269.20 | 12 | 8708.1868 | Z\_DOT73 | 1 | 4.04e-03 | 0.46 |
| 568 | 18 | 8710.1856 | 792.8423 | 59355.22 | 11 |  |  |  |  |  |
| 568 | 19 | 8681.1461 | 724.4361 | 70515.76 | 12 | 8682.1585 | C73 | 73 | -9.97e-03 | -1.15 |
| 568 | 20 | 8768.2041 | 798.1167 | 71057.96 | 11 |  |  |  |  |  |
| 568 | 21 | 8767.2028 | 675.4075 | 58009.47 | 13 |  |  |  |  |  |
| 568 | 22 | 8792.2217 | 629.0231 | 56392.20 | 14 |  |  |  |  |  |
| 568 | 23 | 2528.0806 | 633.0274 | 76116.30 | 4 | 2528.0889 | C20 | 20 | -8.28e-03 | -3.27 |
| 568 | 24 | 8795.2120 | 800.5720 | 54430.69 | 11 |  |  |  |  |  |
| 568 | 25 | 6240.0979 | 694.3515 | 55186.33 | 9 |  |  |  |  |  |
| 568 | 26 | 4282.8397 | 714.8139 | 57616.38 | 6 |  |  |  |  |  |
| 568 | 27 | 2471.0606 | 618.7724 | 64927.02 | 4 | 2471.0674 | C19 | 19 | -6.84e-03 | -2.77 |
| 568 | 28 | 8210.8539 | 747.4485 | 45446.38 | 11 | 8209.9243 | Z\_DOT69 | 5 | -0.0728 | -8.86 |
| 568 | 28 | 8210.8539 | 747.4485 | 45446.38 | 11 | 8210.8779 | C69 | 69 | -0.0240 | -2.92 |
| 568 | 29 | 8721.1998 | 727.7739 | 34222.21 | 12 |  |  |  |  |  |
| 568 | 30 | 6081.6080 | 761.2083 | 45918.71 | 8 | 6081.6306 | C50 | 50 | -0.0226 | -3.72 |
| 568 | 31 | 4255.2745 | 608.9036 | 57348.23 | 7 |  |  |  |  |  |
| 568 | 32 | 6284.1163 | 699.2424 | 51437.68 | 9 | 6284.1564 | Z\_DOT54 | 20 | -0.0401 | -6.39 |
| 568 | 33 | 4309.2844 | 616.6193 | 48682.94 | 7 |  |  |  |  |  |
| 568 | 34 | 7324.2819 | 814.8164 | 39479.74 | 9 | 7324.3027 | C61 | 61 | -0.0208 | -2.84 |
| 568 | 35 | 3445.5933 | 690.1259 | 34345.39 | 5 | 3445.6046 | C27 | 27 | -0.0113 | -3.29 |
| 568 | 36 | 3157.5047 | 632.5082 | 41545.74 | 5 | 3157.5153 | C25 | 25 | -0.0107 | -3.38 |
| 568 | 37 | 6284.1431 | 786.5252 | 35215.34 | 8 | 6284.1564 | Z\_DOT54 | 20 | -0.0133 | -2.11 |
| 568 | 38 | 2641.1661 | 661.2988 | 36148.63 | 4 | 2641.1730 | C21 | 21 | -6.90e-03 | -2.61 |
| 568 | 39 | 4153.7962 | 693.3066 | 36115.87 | 6 |  |  |  |  |  |
| 568 | 40 | 7954.6656 | 724.1587 | 60854.74 | 11 | 7954.6880 | C67 | 67 | -0.0224 | -2.82 |
| 568 | 41 | 7954.6639 | 796.4737 | 41266.95 | 10 | 7954.6880 | C67 | 67 | -0.0241 | -3.03 |
| 568 | 42 | 5033.5498 | 720.0858 | 25696.62 | 7 |  |  |  |  |  |
| 568 | 43 | 8719.1892 | 671.7141 | 26454.31 | 13 |  |  |  |  |  |
| 568 | 44 | 7898.6467 | 790.8720 | 34837.50 | 10 |  |  |  |  |  |
| 568 | 45 | 8754.2095 | 876.4282 | 27287.15 | 10 |  |  |  |  |  |
| 568 | 46 | 4440.3508 | 635.3431 | 30218.12 | 7 |  |  |  |  |  |
| 568 | 47 | 7770.5411 | 778.0614 | 22834.77 | 10 | 7769.5716 | C65 | 65 | -0.0328 | -4.22 |
| 568 | 48 | 4770.0798 | 796.0206 | 36608.63 | 6 | 4770.0923 | C39 | 39 | -0.0125 | -2.62 |
| 568 | 49 | 8083.7605 | 809.3833 | 26510.89 | 10 | 8082.7830 | C68 | 68 | -0.0248 | -3.07 |
| 568 | 50 | 3317.5364 | 664.5146 | 29014.46 | 5 | 3317.5460 | C26 | 26 | -9.59e-03 | -2.89 |
| 568 | 51 | 4405.1127 | 630.3091 | 47430.95 | 7 |  |  |  |  |  |
| 568 | 52 | 8625.1176 | 785.1089 | 31836.82 | 11 |  |  |  |  |  |
| 568 | 53 | 4369.8778 | 729.3202 | 27656.27 | 6 |  |  |  |  |  |
| 568 | 54 | 1372.5823 | 687.2984 | 32479.36 | 2 | 1372.5863 | C11 | 11 | -4.01e-03 | -2.92 |
| 568 | 55 | 8663.1802 | 722.9390 | 26219.87 | 12 |  |  |  |  |  |
| 568 | 56 | 8324.8896 | 694.7481 | 22404.01 | 12 | 8324.9209 | C70 | 70 | -0.0312 | -3.75 |
| 568 | 57 | 8664.1379 | 788.6562 | 29054.03 | 11 |  |  |  |  |  |
| 568 | 58 | 8325.8792 | 757.9054 | 29378.02 | 11 | 8324.9209 | C70 | 70 | -0.0440 | -5.28 |
| 568 | 59 | 8696.1769 | 870.6250 | 20913.49 | 10 |  |  |  |  |  |
| 568 | 60 | 8681.1491 | 869.1222 | 29046.00 | 10 | 8682.1585 | C73 | 73 | -7.01e-03 | -0.81 |
| 568 | 61 | 7266.2537 | 808.3688 | 18839.87 | 9 |  |  |  |  |  |
| 568 | 62 | 8723.1863 | 794.0242 | 29288.77 | 11 |  |  |  |  |  |
| 568 | 63 | 3923.7454 | 654.9648 | 22509.07 | 6 |  |  |  |  |  |
| 568 | 64 | 8779.1998 | 732.6073 | 25312.38 | 12 |  |  |  |  |  |
| 568 | 65 | 8211.8507 | 822.1923 | 22726.15 | 10 | 8210.8779 | C69 | 69 | -0.0296 | -3.61 |
| 568 | 66 | 6240.1027 | 781.0201 | 19614.72 | 8 |  |  |  |  |  |
| 568 | 67 | 4299.8718 | 717.6526 | 19112.66 | 6 | 4299.8798 | C34 | 34 | -7.99e-03 | -1.86 |
| 568 | 68 | 4554.9482 | 911.9969 | 19415.36 | 5 |  |  |  |  |  |
| 568 | 69 | 8210.8381 | 685.2438 | 16892.66 | 12 | 8210.8779 | C69 | 69 | -0.0399 | -4.86 |
| 568 | 70 | 6209.6724 | 777.2163 | 18481.30 | 8 | 6209.6892 | C51 | 51 | -0.0168 | -2.70 |
| 568 | 71 | 4255.2758 | 710.2199 | 19907.08 | 6 |  |  |  |  |  |
| 568 | 72 | 3982.1416 | 569.8847 | 23150.79 | 7 |  |  |  |  |  |
| 568 | 73 | 2528.0808 | 843.7009 | 24413.06 | 3 | 2528.0889 | C20 | 20 | -8.14e-03 | -3.22 |
| 568 | 74 | 8738.1843 | 874.8257 | 16172.73 | 10 |  |  |  |  |  |
| 568 | 75 | 8267.8634 | 752.6312 | 17451.45 | 11 |  |  |  |  |  |
| 568 | 76 | 8704.2127 | 670.5621 | 21448.37 | 13 |  |  |  |  |  |
| 568 | 77 | 2203.3736 | 551.8507 | 30898.13 | 4 |  |  |  |  |  |
| 568 | 78 | 8624.1105 | 863.4183 | 17651.06 | 10 |  |  |  |  |  |
| 568 | 79 | 3740.6550 | 624.4498 | 19030.88 | 6 |  |  |  |  |  |
| 568 | 80 | 2202.8017 | 735.2745 | 95261.23 | 3 |  |  |  |  |  |
| 568 | 81 | 4055.7958 | 812.1664 | 15380.00 | 5 | 4055.8103 | C32 | 32 | -0.0145 | -3.57 |
| 568 | 82 | 7323.2789 | 733.3352 | 29473.25 | 10 | 7324.3027 | C61 | 61 | -0.0215 | -2.93 |
| 568 | 83 | 7723.7697 | 703.1682 | 14959.69 | 11 |  |  |  |  |  |
| 568 | 84 | 4638.4169 | 663.6383 | 17845.98 | 7 |  |  |  |  |  |
| 568 | 85 | 3666.0142 | 612.0096 | 14261.18 | 6 |  |  |  |  |  |
| 568 | 86 | 7723.7687 | 773.3841 | 13508.71 | 10 |  |  |  |  |  |
| 568 | 87 | 6522.8360 | 816.3618 | 17496.24 | 8 | 6522.8530 | C54 | 54 | -0.0169 | -2.60 |
| 568 | 88 | 5381.6587 | 769.8157 | 16721.70 | 7 |  |  |  |  |  |
| 568 | 89 | 2788.2325 | 698.0654 | 21865.23 | 4 | 2788.2414 | C22 | 22 | -8.90e-03 | -3.19 |
| 568 | 90 | 4170.8198 | 696.1439 | 19460.20 | 6 | 4170.8372 | C33 | 33 | -0.0174 | -4.17 |
| 568 | 91 | 4536.9382 | 757.1637 | 18438.95 | 6 |  |  |  |  |  |
| 568 | 92 | 8082.7346 | 735.8013 | 30444.41 | 11 | 8082.7830 | C68 | 68 | -0.0484 | -5.98 |
| 568 | 93 | 8452.9996 | 769.4618 | 21939.40 | 11 | 8453.0158 | C71 | 71 | -0.0162 | -1.92 |
| 568 | 94 | 8651.1795 | 787.4781 | 15127.50 | 11 |  |  |  |  |  |
| 568 | 95 | 6854.4296 | 686.4502 | 13898.88 | 10 |  |  |  |  |  |
| 568 | 96 | 3776.6738 | 756.3420 | 15964.04 | 5 |  |  |  |  |  |
| 568 | 97 | 8224.9229 | 748.7275 | 12694.08 | 11 |  |  |  |  |  |
| 568 | 98 | 7826.5637 | 712.5131 | 19669.70 | 11 | 7826.5930 | C66 | 66 | -0.0293 | -3.75 |
| 568 | 99 | 7936.6510 | 722.5210 | 15954.03 | 11 |  |  |  |  |  |
| 568 | 100 | 4510.3674 | 645.3455 | 15090.87 | 7 |  |  |  |  |  |
| 568 | 101 | 5239.6155 | 655.9592 | 17638.06 | 8 |  |  |  |  |  |
| 568 | 102 | 8710.1898 | 872.0263 | 17001.74 | 10 |  |  |  |  |  |
| 568 | 103 | 6855.4201 | 762.7206 | 14382.77 | 9 |  |  |  |  |  |
| 568 | 104 | 6340.1751 | 705.4712 | 13672.73 | 9 | 6341.1779 | Z\_DOT55 | 19 | -4.14e-04 | -0.07 |
| 568 | 105 | 6505.8193 | 814.2347 | 11661.28 | 8 |  |  |  |  |  |
| 568 | 106 | 7058.4712 | 706.8544 | 12931.91 | 10 | 7059.4782 | Z\_DOT60 | 14 | -4.67e-03 | -0.66 |
| 568 | 107 | 4387.8965 | 732.3234 | 12228.19 | 6 |  |  |  |  |  |
| 568 | 108 | 7827.5687 | 783.7641 | 18219.16 | 10 | 7826.5930 | C66 | 66 | -0.0267 | -3.41 |
| 568 | 109 | 4443.9225 | 889.7918 | 14275.27 | 5 | 4443.9333 | C36 | 36 | -0.0109 | -2.44 |
| 568 | 110 | 1615.7038 | 808.8592 | 20846.71 | 2 | 1615.7082 | C13 | 13 | -4.45e-03 | -2.75 |
| 568 | 111 | 7552.6937 | 687.6158 | 10473.63 | 11 | 7553.7019 | Z\_DOT64 | 10 | -5.88e-03 | -0.78 |
| 568 | 112 | 7382.2964 | 739.2369 | 12887.00 | 10 | 7381.3242 | C62 | 62 | -0.0301 | -4.07 |
| 568 | 113 | 8769.2230 | 877.9296 | 13843.42 | 10 |  |  |  |  |  |
| 568 | 114 | 8796.2121 | 880.6285 | 8535.20 | 10 |  |  |  |  |  |
| 568 | 115 | 8025.7276 | 803.5800 | 18088.47 | 10 |  |  |  |  |  |
| 568 | 116 | 4771.4528 | 682.6434 | 11753.78 | 7 |  |  |  |  |  |
| 568 | 117 | 2915.3285 | 729.8394 | 17902.18 | 4 |  |  |  |  |  |
| 568 | 118 | 7552.6878 | 756.2761 | 13754.87 | 10 | 7553.7019 | Z\_DOT64 | 10 | -0.0118 | -1.56 |
| 568 | 119 | 7308.2615 | 813.0363 | 14421.27 | 9 |  |  |  |  |  |
| 568 | 120 | 1752.7632 | 877.3889 | 19666.48 | 2 | 1752.7671 | C14 | 14 | -3.91e-03 | -2.23 |
| 568 | 121 | 8774.2073 | 627.7364 | 18259.31 | 14 |  |  |  |  |  |
| 568 | 122 | 6623.8818 | 828.9925 | 17859.12 | 8 | 6623.9007 | C55 | 55 | -0.0188 | -2.85 |
| 568 | 123 | 1386.8790 | 463.3003 | 13805.64 | 3 |  |  |  |  |  |
| 568 | 124 | 1428.8880 | 477.3033 | 10992.25 | 3 |  |  |  |  |  |
| 568 | 125 | 678.1717 | 679.1790 | 65651.39 | 1 |  |  |  |  |  |
| 568 | 126 | 1216.7310 | 609.3728 | 7605.45 | 2 |  |  |  |  |  |
| 568 | 127 | 1487.6082 | 744.8114 | 10071.98 | 2 | 1487.6133 | C12 | 12 | -5.07e-03 | -3.41 |
| 568 | 128 | 735.1861 | 736.1934 | 10752.22 | 1 |  |  |  |  |  |
| 568 | 129 | 1415.8814 | 472.9677 | 4875.02 | 3 |  |  |  |  |  |
| 568 | 130 | 1169.7820 | 585.8983 | 7126.58 | 2 |  |  |  |  |  |
| 568 | 131 | 798.5059 | 400.2602 | 7475.34 | 2 |  |  |  |  |  |
| 568 | 132 | 786.1052 | 787.1124 | 5694.36 | 1 |  |  |  |  |  |
| 568 | 133 | 801.5682 | 802.5754 | 18850.84 | 1 |  |  |  |  |  |
| 568 | 134 | 600.3824 | 601.3896 | 10086.56 | 1 |  |  |  |  |  |
| 568 | 135 | 1486.9553 | 496.6591 | 7019.55 | 3 |  |  |  |  |  |
| 568 | 136 | 997.4630 | 499.7388 | 5287.10 | 2 | 997.4651 | C8 | 8 | -2.07e-03 | -2.07 |
| 568 | 137 | 486.3397 | 487.3470 | 5247.86 | 1 |  |  |  |  |  |
| 568 | 138 | 1169.7820 | 390.9346 | 3866.11 | 3 |  |  |  |  |  |
| 568 | 139 | 997.4648 | 998.4721 | 4091.14 | 1 | 997.4651 | C8 | 8 | -2.24e-04 | -0.22 |
| 568 | 140 | 856.5710 | 857.5782 | 4800.15 | 1 |  |  |  |  |  |
| 568 | 141 | 1023.4779 | 512.7462 | 4157.62 | 2 |  |  |  |  |  |
| 568 | 142 | 1111.7165 | 371.5794 | 3734.22 | 3 |  |  |  |  |  |
| 568 | 143 | 1157.4936 | 579.7541 | 4565.58 | 2 | 1157.4957 | C9 | 9 | -2.14e-03 | -1.85 |
| 568 | 144 | 1430.9388 | 716.4767 | 2069.78 | 2 |  |  |  |  |  |
| 568 | 145 | 1057.7057 | 529.8601 | 3172.83 | 2 |  |  |  |  |  |
| 568 | 146 | 618.7668 | 619.7741 | 6161.26 | 1 |  |  |  |  |  |

  

All proteins /
CsTx-1b Cupiennius salei toxin 1 isoform b /
Proteoform #9
