# Supplementary material for: The Dual Prey-Inactivation Strategy of Spiders—In-Depth Venomic Analysis of Cupiennius salei
Source: Toxins (Basel). 2019 Mar 19;11(3):167. doi: 10.3390/toxins11030167 (PMC6468893; doi:10.3390/toxins11030167)
Supplement: Supplementary file 1 [file toxins-11-00167-s001.zip › Supplementary Dataset EV1/20180328_f2_topdown_OTMS2_EThcD_NL_i02_ms2_proteoform_cutoff_html/prsms/prsm186.html]

Protein-Spectrum-Match for Spectrum #426


All proteins /
CsTx-1a\_S1 Cupiennius salei toxin 1 isoform a S1^ACsTx-1a\_S2 Cupiennius salei toxin 1 isoform a S2 /
Proteoform #6

## Protein-Spectrum-Match #186 for Spectrum #426

|  |  |  |  |  |  |
| --- | --- | --- | --- | --- | --- |
| PrSM ID: | 186 | Scan(s): | 571 | Precursor charge: | 13 |
| Precursor m/z: | 678.7183 | Precursor mass: | 8810.2439 | Proteoform mass: | 8811.2375 |
| # matched peaks: | 52 | # matched fragment ions: | 40 | # unexpected modifications: | 0 |
| E-value: | 1.54e-35 | P-value: | 1.54e-35 | Q-value (Spectral FDR): | 0 |

  

|  |  |  |  |  |  |  |  |  |  |  |  |  |  |  |  |  |  |  |  |  |  |  |  |  |  |  |  |  |  |  |  |  |  |  |  |  |  |  |  |  |  |  |  |  |  |  |  |  |  |  |  |  |  |  |  |  |  |  |  |  |  |  |  |  |  |  |  |  |  |
| --- | --- | --- | --- | --- | --- | --- | --- | --- | --- | --- | --- | --- | --- | --- | --- | --- | --- | --- | --- | --- | --- | --- | --- | --- | --- | --- | --- | --- | --- | --- | --- | --- | --- | --- | --- | --- | --- | --- | --- | --- | --- | --- | --- | --- | --- | --- | --- | --- | --- | --- | --- | --- | --- | --- | --- | --- | --- | --- | --- | --- | --- | --- | --- | --- | --- | --- | --- | --- | --- |
|  | |  | | | | | | | | | | | | | | | | | | | | | | | | | | | | | | | | | | | | | | | | | | | | | | | | | | | | | | | | | | | | | | | | | | | |
| 1 |  |  | M |  | K |  | V |  | L |  | I |  | I |  | S |  | A |  | V |  | L |  |  | F |  | I |  | T |  | I |  | F |  | S |  | N |  | I |  | S |  | A |  |  | E |  | I |  | E |  | D |  | D |  | F |  | L |  | E |  | D |  | E |  | 30 |  |
|  | |  | | | | | | | | | | | | | | | | | | | | | | | | | | | | | | | | | | | | | | | | | | | | | | | | | | | | | | | | | | | | | | | | | | | |
| 31 |  |  | S |  | F |  | E |  | A |  | E |  | D |  | I |  | I |  | P |  | F |  |  | F |  | E |  | N |  | E |  | Q |  | A |  | R | ] | S | ⎩ | C |  | I |  |  | P |  | K | ⎩ | H |  | E |  | E | ⎫ | C | ⎩ | T | ⎱ | N | ⎱ | D | ⎫ | K |  | 60 |  |
|  | |  | | | | | | | | | | | | | | | | | | | | | | | | | | | | | | | | | | | | | | | | | | | | | | | | | | | | | | | | | | | | | | | | | | | |
| 61 |  |  | H | ⎩ | N |  | C |  | C |  | R |  | K | ⎫ | G | ⎱ | L | ⎱ | F |  | K |  |  | L |  | K | ⎫ | C | ⎫ | Q | ⎫ | C |  | S |  | T |  | F |  | D | ⎫ | D |  |  | E | ⎫ | S |  | G | ⎫ | Q |  | P |  | T | ⎫ | E |  | R |  | C |  | A |  | 90 |  |
|  | |  | | | | | | | | | | | | | | | | | | | | | | | | | | | | | | | | | | | | | | | | | | | | | | | | | | | | | | | | | | | | | | | | | | | |
| 91 |  |  | C |  | G |  | R |  | P | ⎫ | M |  | G |  | H | ⎫ | Q | ⎫ | A |  | I |  |  | E | ⎫ | T | ⎫ | G | ⎫ | L | ⎫ | N |  | I |  | F |  | R | ⎫ | G | ⎫ | L |  |  | F |  | K | ⎫ | G | ⎫ | K | ⎫ | K | ⎫ | K | ⎫ | N | ⎫ | K | ⎫ | K | ⎫ | T |  | 120 |  |
|  | |  | | | | | | | | | | | | | | | | | | | | | | | | | | | | | | | | | | | | | | | | | | | | | | | | | | | | | | | | | | | | | | | | | | | |
| 121 |  | ⎫ | K | [ | G |  | | | | 122 |  | | | | | | | | | | | | | | | | | | | | | | | | | | | | | | | | | | | | | | | | | | | | | | | | | | | | | | | |

Fixed PTMs: Carbamidomethylation [C49 C56 C63 C64 C73 C75 C89 C91 ]

  

All peaks (146)  Matched peaks (52)  Not matched peaks (94)

  

| Scan | Peak | Mono mass | Mono m/z | Intensity | Charge | Theoretical mass | Ion | Pos | Mass error | PPM error |
| --- | --- | --- | --- | --- | --- | --- | --- | --- | --- | --- |
| 571 | 1 | 8753.2227 | 730.4425 | 655936.68 | 12 |  |  |  |  |  |
| 571 | 2 | 8753.2060 | 796.7533 | 594265.51 | 11 |  |  |  |  |  |
| 571 | 3 | 8682.1498 | 790.2936 | 227039.66 | 11 | 8682.1585 | C73 | 73 | -8.61e-03 | -0.99 |
| 571 | 4 | 8767.1924 | 798.0248 | 232207.62 | 11 |  |  |  |  |  |
| 571 | 5 | 8792.2372 | 677.3332 | 243521.71 | 13 |  |  |  |  |  |
| 571 | 6 | 8793.2283 | 733.7763 | 215462.08 | 12 |  |  |  |  |  |
| 571 | 7 | 8737.2244 | 729.1093 | 207910.83 | 12 |  |  |  |  |  |
| 571 | 8 | 8794.2083 | 800.4808 | 202806.37 | 11 |  |  |  |  |  |
| 571 | 9 | 8767.2119 | 731.6083 | 193570.13 | 12 |  |  |  |  |  |
| 571 | 10 | 8696.1879 | 791.5698 | 156559.74 | 11 |  |  |  |  |  |
| 571 | 11 | 8737.2062 | 795.2987 | 162745.03 | 11 |  |  |  |  |  |
| 571 | 12 | 4404.6140 | 735.1096 | 559465.96 | 6 |  |  |  |  |  |
| 571 | 13 | 8753.2079 | 876.3281 | 122035.81 | 10 |  |  |  |  |  |
| 571 | 14 | 8082.7504 | 809.2823 | 110453.19 | 10 | 8082.7830 | C68 | 68 | -0.0326 | -4.03 |
| 571 | 15 | 8709.2007 | 792.7528 | 112312.18 | 11 | 8708.1868 | Z\_DOT73 | 1 | 0.0116 | 1.33 |
| 571 | 16 | 7954.6909 | 796.4764 | 121141.22 | 10 | 7954.6880 | C67 | 67 | 2.88e-03 | 0.36 |
| 571 | 17 | 8324.8916 | 757.8156 | 92457.13 | 11 | 8324.9209 | C70 | 70 | -0.0292 | -3.51 |
| 571 | 18 | 8210.8452 | 747.4477 | 73340.88 | 11 | 8209.9243 | Z\_DOT69 | 5 | -0.0815 | -9.93 |
| 571 | 18 | 8210.8452 | 747.4477 | 73340.88 | 11 | 8210.8779 | C69 | 69 | -0.0327 | -3.98 |
| 571 | 19 | 7324.2775 | 814.8159 | 75751.95 | 9 | 7324.3027 | C61 | 61 | -0.0252 | -3.44 |
| 571 | 20 | 4554.9518 | 760.1659 | 89469.99 | 6 |  |  |  |  |  |
| 571 | 21 | 8452.9908 | 769.4610 | 74390.24 | 11 | 8453.0158 | C71 | 71 | -0.0250 | -2.95 |
| 571 | 22 | 8682.1411 | 869.2214 | 75242.09 | 10 | 8682.1585 | C73 | 73 | -0.0174 | -2.00 |
| 571 | 23 | 4886.4810 | 699.0760 | 68784.06 | 7 |  |  |  |  |  |
| 571 | 24 | 8720.2271 | 727.6929 | 61649.47 | 12 |  |  |  |  |  |
| 571 | 25 | 4443.9134 | 741.6595 | 67169.06 | 6 | 4443.9333 | C36 | 36 | -0.0200 | -4.49 |
| 571 | 26 | 8705.2212 | 726.4424 | 72046.05 | 12 |  |  |  |  |  |
| 571 | 27 | 8210.8562 | 822.0929 | 62898.42 | 10 | 8209.9243 | Z\_DOT69 | 5 | -0.0705 | -8.58 |
| 571 | 27 | 8210.8562 | 822.0929 | 62898.42 | 10 | 8210.8779 | C69 | 69 | -0.0217 | -2.64 |
| 571 | 28 | 4282.8430 | 714.8144 | 62822.16 | 6 |  |  |  |  |  |
| 571 | 29 | 6284.1290 | 786.5234 | 73897.18 | 8 | 6284.1564 | Z\_DOT54 | 20 | -0.0274 | -4.37 |
| 571 | 30 | 6240.1090 | 781.0209 | 56140.04 | 8 |  |  |  |  |  |
| 571 | 31 | 2937.0853 | 735.2786 | 460898.16 | 4 |  |  |  |  |  |
| 571 | 32 | 8721.2098 | 793.8445 | 58821.51 | 11 |  |  |  |  |  |
| 571 | 33 | 8774.2241 | 675.9476 | 43909.31 | 13 |  |  |  |  |  |
| 571 | 34 | 8696.1743 | 870.6247 | 51517.02 | 10 |  |  |  |  |  |
| 571 | 35 | 8663.2013 | 788.5710 | 49410.46 | 11 |  |  |  |  |  |
| 571 | 36 | 4527.3881 | 647.7770 | 54984.04 | 7 |  |  |  |  |  |
| 571 | 37 | 8710.1825 | 872.0255 | 49070.04 | 10 |  |  |  |  |  |
| 571 | 38 | 8324.8938 | 833.4967 | 53114.44 | 10 | 8324.9209 | C70 | 70 | -0.0270 | -3.24 |
| 571 | 39 | 4255.2772 | 710.2201 | 50595.52 | 6 |  |  |  |  |  |
| 571 | 40 | 8768.2202 | 877.8293 | 44199.99 | 10 |  |  |  |  |  |
| 571 | 41 | 8737.1936 | 874.7266 | 44326.65 | 10 |  |  |  |  |  |
| 571 | 42 | 7826.5757 | 783.6648 | 42031.51 | 10 | 7826.5930 | C66 | 66 | -0.0174 | -2.22 |
| 571 | 43 | 7723.7754 | 773.3848 | 38909.19 | 10 |  |  |  |  |  |
| 571 | 44 | 4443.9188 | 889.7910 | 42036.74 | 5 | 4443.9333 | C36 | 36 | -0.0145 | -3.27 |
| 571 | 45 | 4309.2893 | 719.2222 | 47986.42 | 6 |  |  |  |  |  |
| 571 | 46 | 2528.0828 | 633.0280 | 54713.76 | 4 | 2528.0889 | C20 | 20 | -6.08e-03 | -2.41 |
| 571 | 47 | 5033.5549 | 720.0865 | 40758.44 | 7 |  |  |  |  |  |
| 571 | 48 | 4038.7752 | 674.1365 | 44017.45 | 6 |  |  |  |  |  |
| 571 | 49 | 6854.4269 | 762.6103 | 41208.73 | 9 |  |  |  |  |  |
| 571 | 50 | 7669.7556 | 767.9828 | 36444.89 | 10 |  |  |  |  |  |
| 571 | 51 | 8625.1117 | 863.5184 | 41691.22 | 10 |  |  |  |  |  |
| 571 | 52 | 6081.6308 | 869.8117 | 46926.71 | 7 | 6081.6306 | C50 | 50 | 1.50e-04 | 0.02 |
| 571 | 53 | 8224.9296 | 748.7281 | 44500.77 | 11 |  |  |  |  |  |
| 571 | 54 | 4527.3860 | 755.5716 | 39749.72 | 6 |  |  |  |  |  |
| 571 | 55 | 6663.8813 | 833.9924 | 44496.71 | 8 |  |  |  |  |  |
| 571 | 56 | 6854.4259 | 686.4499 | 26687.82 | 10 |  |  |  |  |  |
| 571 | 57 | 4153.7995 | 693.3072 | 33519.08 | 6 |  |  |  |  |  |
| 571 | 58 | 8453.9862 | 846.4059 | 32863.33 | 10 | 8453.0158 | C71 | 71 | -0.0320 | -3.78 |
| 571 | 59 | 2471.0613 | 618.7726 | 42580.81 | 4 | 2471.0674 | C19 | 19 | -6.11e-03 | -2.47 |
| 571 | 60 | 8662.2112 | 722.8582 | 38048.56 | 12 |  |  |  |  |  |
| 571 | 61 | 7954.6720 | 884.8597 | 30013.90 | 9 | 7954.6880 | C67 | 67 | -0.0160 | -2.01 |
| 571 | 62 | 3982.1469 | 664.6984 | 32370.77 | 6 |  |  |  |  |  |
| 571 | 63 | 7769.5481 | 777.9621 | 31179.21 | 10 | 7769.5716 | C65 | 65 | -0.0235 | -3.02 |
| 571 | 64 | 7724.7735 | 703.2594 | 27420.08 | 11 |  |  |  |  |  |
| 571 | 65 | 8796.2165 | 880.6289 | 32188.52 | 10 |  |  |  |  |  |
| 571 | 66 | 3445.5945 | 690.1262 | 31378.43 | 5 | 3445.6046 | C27 | 27 | -0.0101 | -2.93 |
| 571 | 67 | 3157.5080 | 632.5089 | 34104.33 | 5 | 3157.5153 | C25 | 25 | -7.31e-03 | -2.32 |
| 571 | 68 | 6794.9926 | 850.3814 | 32399.97 | 8 | 6794.0062 | C57 | 57 | -0.0159 | -2.34 |
| 571 | 69 | 4554.9543 | 911.9981 | 31263.96 | 5 |  |  |  |  |  |
| 571 | 70 | 7829.7991 | 712.8072 | 25081.92 | 11 |  |  |  |  |  |
| 571 | 71 | 6170.0665 | 772.2656 | 28920.38 | 8 | 6171.0724 | Z\_DOT53 | 21 | -3.50e-03 | -0.57 |
| 571 | 72 | 4367.3312 | 728.8958 | 28836.52 | 6 |  |  |  |  |  |
| 571 | 73 | 8619.1718 | 784.5683 | 26291.95 | 11 |  |  |  |  |  |
| 571 | 74 | 3776.6778 | 756.3428 | 24921.70 | 5 |  |  |  |  |  |
| 571 | 75 | 6646.8705 | 831.8661 | 31115.35 | 8 |  |  |  |  |  |
| 571 | 76 | 5756.4927 | 823.3634 | 26019.49 | 7 | 5756.5098 | C47 | 47 | -0.0171 | -2.96 |
| 571 | 77 | 6505.8042 | 814.2328 | 22370.30 | 8 |  |  |  |  |  |
| 571 | 78 | 8082.7551 | 899.0912 | 22695.99 | 9 | 8082.7830 | C68 | 68 | -0.0278 | -3.44 |
| 571 | 79 | 4638.4182 | 663.6384 | 22549.55 | 7 |  |  |  |  |  |
| 571 | 80 | 6240.1006 | 694.3518 | 26451.11 | 9 |  |  |  |  |  |
| 571 | 81 | 4867.4673 | 696.3597 | 26594.37 | 7 |  |  |  |  |  |
| 571 | 82 | 2641.1665 | 661.2989 | 27957.05 | 4 | 2641.1730 | C21 | 21 | -6.41e-03 | -2.43 |
| 571 | 83 | 6081.6062 | 761.2080 | 26260.91 | 8 | 6081.6306 | C50 | 50 | -0.0245 | -4.03 |
| 571 | 84 | 8581.0998 | 859.1173 | 25363.94 | 10 | 8581.1108 | C72 | 72 | -0.0109 | -1.27 |
| 571 | 85 | 7552.6963 | 756.2769 | 28858.41 | 10 | 7553.7019 | Z\_DOT64 | 10 | -3.24e-03 | -0.43 |
| 571 | 86 | 4406.1220 | 882.2317 | 35259.61 | 5 |  |  |  |  |  |
| 571 | 87 | 6680.9155 | 836.1217 | 25809.71 | 8 | 6680.9221 | C56 | 56 | -6.63e-03 | -0.99 |
| 571 | 88 | 8395.9723 | 840.6045 | 18355.65 | 10 |  |  |  |  |  |
| 571 | 89 | 7653.7437 | 766.3816 | 24030.20 | 10 | 7654.7496 | Z\_DOT65 | 9 | -3.56e-03 | -0.47 |
| 571 | 90 | 4770.0757 | 955.0224 | 22347.59 | 5 | 4770.0923 | C39 | 39 | -0.0167 | -3.49 |
| 571 | 91 | 3923.7461 | 654.9650 | 22809.74 | 6 |  |  |  |  |  |
| 571 | 92 | 7381.2968 | 821.1514 | 26835.05 | 9 | 7381.3242 | C62 | 62 | -0.0274 | -3.71 |
| 571 | 93 | 3317.5382 | 664.5149 | 20100.26 | 5 | 3317.5460 | C26 | 26 | -7.75e-03 | -2.34 |
| 571 | 94 | 8582.0897 | 781.1973 | 46832.81 | 11 | 8581.1108 | C72 | 72 | -0.0234 | -2.73 |
| 571 | 95 | 7325.2966 | 916.6694 | 20334.13 | 8 | 7324.3027 | C61 | 61 | -8.40e-03 | -1.15 |
| 571 | 96 | 4309.2869 | 616.6197 | 24303.18 | 7 |  |  |  |  |  |
| 571 | 97 | 4055.7973 | 812.1667 | 20877.52 | 5 | 4055.8103 | C32 | 32 | -0.0129 | -3.19 |
| 571 | 98 | 1372.5828 | 687.2987 | 31301.82 | 2 | 1372.5863 | C11 | 11 | -3.52e-03 | -2.56 |
| 571 | 99 | 6968.4749 | 775.2823 | 15841.91 | 9 |  |  |  |  |  |
| 571 | 100 | 4771.4477 | 682.6427 | 24251.02 | 7 |  |  |  |  |  |
| 571 | 101 | 8666.1560 | 867.6229 | 21579.61 | 10 |  |  |  |  |  |
| 571 | 102 | 7307.2641 | 812.9255 | 18312.31 | 9 |  |  |  |  |  |
| 571 | 103 | 7897.6405 | 878.5229 | 25873.95 | 9 |  |  |  |  |  |
| 571 | 104 | 8225.9333 | 823.6006 | 28723.01 | 10 |  |  |  |  |  |
| 571 | 105 | 8678.1937 | 724.1901 | 22979.39 | 12 |  |  |  |  |  |
| 571 | 106 | 6284.1531 | 898.7434 | 20075.24 | 7 | 6284.1564 | Z\_DOT54 | 20 | -3.31e-03 | -0.53 |
| 571 | 107 | 4299.8675 | 860.9808 | 23913.07 | 5 | 4299.8798 | C34 | 34 | -0.0124 | -2.88 |
| 571 | 108 | 8722.2070 | 873.2280 | 18821.45 | 10 |  |  |  |  |  |
| 571 | 109 | 8648.1998 | 787.2073 | 22731.51 | 11 |  |  |  |  |  |
| 571 | 110 | 2203.3743 | 551.8509 | 20999.66 | 4 |  |  |  |  |  |
| 571 | 111 | 3982.1442 | 569.8850 | 15272.69 | 7 |  |  |  |  |  |
| 571 | 112 | 678.0950 | 679.1022 | 98623.79 | 1 |  |  |  |  |  |
| 571 | 113 | 2528.0784 | 843.7001 | 22138.54 | 3 | 2528.0889 | C20 | 20 | -0.0105 | -4.16 |
| 571 | 114 | 7267.2743 | 909.4166 | 15138.01 | 8 |  |  |  |  |  |
| 571 | 115 | 6209.6720 | 777.2163 | 26967.42 | 8 | 6209.6892 | C51 | 51 | -0.0173 | -2.78 |
| 571 | 116 | 7440.6499 | 745.0723 | 17342.77 | 10 | 7439.6590 | Z\_DOT63 | 11 | -0.0115 | -1.54 |
| 571 | 117 | 6623.8794 | 828.9922 | 24538.83 | 8 | 6623.9007 | C55 | 55 | -0.0213 | -3.21 |
| 571 | 118 | 6522.8331 | 932.8406 | 15824.89 | 7 | 6522.8530 | C54 | 54 | -0.0199 | -3.05 |
| 571 | 119 | 8639.1314 | 864.9204 | 18887.40 | 10 |  |  |  |  |  |
| 571 | 120 | 7625.4349 | 848.2778 | 17768.34 | 9 |  |  |  |  |  |
| 571 | 121 | 7769.5309 | 864.2885 | 24487.11 | 9 | 7769.5716 | C65 | 65 | -0.0407 | -5.24 |
| 571 | 122 | 6968.4732 | 697.8546 | 22054.93 | 10 |  |  |  |  |  |
| 571 | 123 | 7059.4851 | 785.3945 | 27044.00 | 9 | 7059.4782 | Z\_DOT60 | 14 | 6.88e-03 | 0.97 |
| 571 | 124 | 801.4764 | 802.4837 | 155047.89 | 1 |  |  |  |  |  |
| 571 | 125 | 1356.8018 | 679.4082 | 51060.86 | 2 |  |  |  |  |  |
| 571 | 126 | 1428.8885 | 477.3034 | 7596.52 | 3 |  |  |  |  |  |
| 571 | 127 | 1487.6079 | 744.8112 | 7162.13 | 2 | 1487.6133 | C12 | 12 | -5.31e-03 | -3.57 |
| 571 | 128 | 1386.8791 | 463.3003 | 6922.06 | 3 |  |  |  |  |  |
| 571 | 129 | 1169.7825 | 585.8985 | 7981.35 | 2 |  |  |  |  |  |
| 571 | 130 | 997.4642 | 998.4714 | 5657.55 | 1 | 997.4651 | C8 | 8 | -8.96e-04 | -0.90 |
| 571 | 131 | 600.3824 | 601.3896 | 7515.90 | 1 |  |  |  |  |  |
| 571 | 132 | 1396.1431 | 699.0788 | 9350.93 | 2 |  |  |  |  |  |
| 571 | 133 | 1023.4788 | 512.7467 | 3827.33 | 2 |  |  |  |  |  |
| 571 | 134 | 1169.7808 | 390.9342 | 3224.99 | 3 |  |  |  |  |  |
| 571 | 135 | 798.5063 | 400.2604 | 4850.38 | 2 |  |  |  |  |  |
| 571 | 136 | 820.0310 | 821.0383 | 5236.80 | 1 |  |  |  |  |  |
| 571 | 137 | 1057.7065 | 529.8605 | 3236.99 | 2 |  |  |  |  |  |
| 571 | 138 | 849.3671 | 850.3744 | 3392.44 | 1 |  |  |  |  |  |
| 571 | 139 | 1486.9556 | 496.6591 | 3503.34 | 3 |  |  |  |  |  |
| 571 | 140 | 1316.8493 | 439.9570 | 2050.55 | 3 |  |  |  |  |  |
| 571 | 141 | 1185.8003 | 593.9074 | 2339.48 | 2 |  |  |  |  |  |
| 571 | 142 | 1013.4187 | 1014.4260 | 3936.69 | 1 |  |  |  |  |  |
| 571 | 143 | 756.9003 | 757.9076 | 5796.97 | 1 |  |  |  |  |  |
| 571 | 144 | 1258.5413 | 630.2779 | 9681.60 | 2 | 1258.5434 | C10 | 10 | -2.09e-03 | -1.66 |
| 571 | 145 | 1185.8010 | 396.2743 | 2582.28 | 3 |  |  |  |  |  |
| 571 | 146 | 910.5397 | 911.5470 | 2871.13 | 1 |  |  |  |  |  |

  

All proteins /
CsTx-1a\_S1 Cupiennius salei toxin 1 isoform a S1^ACsTx-1a\_S2 Cupiennius salei toxin 1 isoform a S2 /
Proteoform #6
