# Supplementary material for: The Dual Prey-Inactivation Strategy of Spiders—In-Depth Venomic Analysis of Cupiennius salei
Source: Toxins (Basel). 2019 Mar 19;11(3):167. doi: 10.3390/toxins11030167 (PMC6468893; doi:10.3390/toxins11030167)
Supplement: Supplementary file 1 [file toxins-11-00167-s001.zip › Supplementary Dataset EV1/20180328_f2_topdown_OTMS2_EThcD_NL_i02_ms2_proteoform_cutoff_html/prsms/prsm187.html]

Protein-Spectrum-Match for Spectrum #427


All proteins /
CsTx-1a\_S1 Cupiennius salei toxin 1 isoform a S1^ACsTx-1a\_S2 Cupiennius salei toxin 1 isoform a S2 /
Proteoform #6

## Protein-Spectrum-Match #187 for Spectrum #427

|  |  |  |  |  |  |
| --- | --- | --- | --- | --- | --- |
| PrSM ID: | 187 | Scan(s): | 572 | Precursor charge: | 12 |
| Precursor m/z: | 735.1946 | Precursor mass: | 8810.2483 | Proteoform mass: | 8810.2383 |
| # matched peaks: | 53 | # matched fragment ions: | 42 | # unexpected modifications: | 1 |
| E-value: | 9.36e-35 | P-value: | 9.36e-35 | Q-value (Spectral FDR): | 0 |

  

|  |  |  |  |  |  |  |  |  |  |  |  |  |  |  |  |  |  |  |  |  |  |  |  |  |  |  |  |  |  |  |  |  |  |  |  |  |  |  |  |  |  |  |  |  |  |  |  |  |  |  |  |  |  |  |  |  |  |  |  |  |  |  |  |  |  |  |  |  |  |
| --- | --- | --- | --- | --- | --- | --- | --- | --- | --- | --- | --- | --- | --- | --- | --- | --- | --- | --- | --- | --- | --- | --- | --- | --- | --- | --- | --- | --- | --- | --- | --- | --- | --- | --- | --- | --- | --- | --- | --- | --- | --- | --- | --- | --- | --- | --- | --- | --- | --- | --- | --- | --- | --- | --- | --- | --- | --- | --- | --- | --- | --- | --- | --- | --- | --- | --- | --- | --- | --- |
|  | |  | | | | | | | | | | | | | | | | | | | | | | | | | | | | | | | | | | | | | | | | | | | | | | | | | | | | | | | | | | | | | | | | | | | |
| 1 |  |  | M |  | K |  | V |  | L |  | I |  | I |  | S |  | A |  | V |  | L |  |  | F |  | I |  | T |  | I |  | F |  | S |  | N |  | I |  | S |  | A |  |  | E |  | I |  | E |  | D |  | D |  | F |  | L |  | E |  | D |  | E |  | 30 |  |
|  | |  | | | | | | | | | | | | | | | | | | | | | | | | | | | | | | | | | | | | | | | | | | | | | | | | | | | | | | | | | | | | | | | | | | | |
| 31 |  |  | S |  | F |  | E |  | A |  | E |  | D |  | I |  | I |  | P |  | F |  |  | F |  | E |  | N |  | E |  | Q |  | A |  | R | ] | S | ⎩ | C |  | I |  |  | P |  | K | ⎫ | H |  | E |  | E | ⎫ | C | ⎩ | T | ⎩ | N | ⎱ | D |  | K |  | 60 |  |
|  | |  | | | | | | | | | | | | | | | | | | | | | | | | | | | | | | | | | | | | | | | | | | | | | | | | | | | | | | | | | | | | | | | | | | | |
| 61 |  |  | H | ⎩ | N |  | C |  | C |  | R |  | K |  | G | ⎩ | L | ⎩ | F | ⎩ | K |  |  | L |  | K |  | C | ⎫ | Q |  | C |  | S |  | T |  | F |  | D | ⎫ | D |  | ⎱ | E | ⎱ | S |  | G | ⎱ | Q |  | P |  | T | ⎫ | E |  | R |  | C |  | A |  | 90 |  |
|  | |  | | | | | | | | | | | | | | | | | | | | | | | | | | | | | | | | | | | | | | | | | | | | | | | | | | | | | | | | | | | | | -58.02 | | | |
| 91 |  |  | C |  | G | ⎫ | R |  | P | ⎫ | M |  | G | ⎫ | H | ⎫ | Q | ⎫ | A |  | I |  |  | E | ⎫ | T | ⎫ | G | ⎫ | L | ⎫ | N |  | I |  | F |  | R | ⎱ | G | ⎫ | L |  |  | F |  | K | ⎫ | G | ⎫ | K | ⎫ | K | ⎫ | K | ⎫ | N | ⎫ | K | ⎫ | K | ⎫ | T |  | 120 |  |
|  | |  | | | | | | | | | | | | | | | | | | | | | | | | | | | | | | | | | | | | | | | | | | | | | | | | | | | | | | | | | | | | | | | | | | | |
| 121 |  | ⎫ | K | ⎫ | G |  | | | | 122 |  | | | | | | | | | | | | | | | | | | | | | | | | | | | | | | | | | | | | | | | | | | | | | | | | | | | | | | | |

Fixed PTMs: Carbamidomethylation [C49 C56 C63 C64 C73 C75 C89 C91 ]   
  
     Unexpected modifications:   Unknown [-58.02]

  

All peaks (146)  Matched peaks (53)  Not matched peaks (93)

  

| Scan | Peak | Mono mass | Mono m/z | Intensity | Charge | Theoretical mass | Ion | Pos | Mass error | PPM error |
| --- | --- | --- | --- | --- | --- | --- | --- | --- | --- | --- |
| 572 | 1 | 8753.2275 | 796.7552 | 954005.92 | 11 | 8752.2328 | C74 | 74 | -7.62e-03 | -0.87 |
| 572 | 2 | 8753.2134 | 876.3286 | 427854.16 | 10 | 8752.2328 | C74 | 74 | -0.0217 | -2.48 |
| 572 | 3 | 8766.2135 | 797.9358 | 290983.74 | 11 |  |  |  |  |  |
| 572 | 4 | 8737.2338 | 795.3013 | 264011.34 | 11 |  |  |  |  |  |
| 572 | 5 | 8794.2278 | 800.4825 | 250835.64 | 11 |  |  |  |  |  |
| 572 | 6 | 8682.1484 | 869.2221 | 212149.02 | 10 |  |  |  |  |  |
| 572 | 7 | 8767.2088 | 877.7282 | 163760.36 | 10 |  |  |  |  |  |
| 572 | 8 | 8795.2105 | 880.5283 | 142570.78 | 10 |  |  |  |  |  |
| 572 | 9 | 8324.8975 | 833.4970 | 127128.30 | 10 | 8324.9209 | C70 | 70 | -0.0233 | -2.80 |
| 572 | 10 | 4404.6235 | 735.1112 | 960104.29 | 6 |  |  |  |  |  |
| 572 | 11 | 8737.2007 | 874.7273 | 98268.63 | 10 |  |  |  |  |  |
| 572 | 12 | 4406.1162 | 882.2305 | 213566.92 | 5 |  |  |  |  |  |
| 572 | 13 | 8210.8539 | 822.0927 | 91892.99 | 10 | 8210.8779 | C69 | 69 | -0.0240 | -2.92 |
| 572 | 14 | 8452.9852 | 846.3058 | 96831.81 | 10 | 8453.0158 | C71 | 71 | -0.0306 | -3.62 |
| 572 | 15 | 8720.2457 | 793.7569 | 80812.30 | 11 |  |  |  |  |  |
| 572 | 16 | 8082.7590 | 899.0916 | 84532.29 | 9 | 8082.7830 | C68 | 68 | -0.0240 | -2.97 |
| 572 | 17 | 8696.1761 | 870.6249 | 96038.70 | 10 |  |  |  |  |  |
| 572 | 18 | 8224.9342 | 823.5007 | 75484.86 | 10 |  |  |  |  |  |
| 572 | 19 | 7723.7779 | 773.3851 | 72780.19 | 10 |  |  |  |  |  |
| 572 | 20 | 7954.6589 | 884.8583 | 81852.13 | 9 | 7954.6880 | C67 | 67 | -0.0292 | -3.66 |
| 572 | 21 | 8581.0925 | 859.1165 | 76585.13 | 10 | 8581.1108 | C72 | 72 | -0.0183 | -2.13 |
| 572 | 22 | 8709.1839 | 871.9257 | 73831.94 | 10 |  |  |  |  |  |
| 572 | 23 | 6854.4291 | 762.6105 | 53341.08 | 9 |  |  |  |  |  |
| 572 | 24 | 7324.2826 | 916.5426 | 61247.88 | 8 | 7324.3027 | C61 | 61 | -0.0201 | -2.75 |
| 572 | 25 | 8706.2258 | 792.4823 | 80044.66 | 11 | 8707.1876 | Z\_DOT74 | 1 | 0.0405 | 4.65 |
| 572 | 26 | 8792.2795 | 733.6972 | 174182.39 | 12 |  |  |  |  |  |
| 572 | 27 | 7829.8038 | 783.9877 | 58234.26 | 10 |  |  |  |  |  |
| 572 | 28 | 8082.7430 | 809.2816 | 47445.39 | 10 | 8082.7830 | C68 | 68 | -0.0399 | -4.94 |
| 572 | 29 | 4255.2772 | 710.2201 | 53442.85 | 6 |  |  |  |  |  |
| 572 | 30 | 4443.9197 | 889.7912 | 51126.39 | 5 | 4443.9333 | C36 | 36 | -0.0136 | -3.06 |
| 572 | 31 | 8721.2036 | 873.1276 | 42554.91 | 10 |  |  |  |  |  |
| 572 | 32 | 4554.9544 | 760.1663 | 47132.29 | 6 |  |  |  |  |  |
| 572 | 33 | 8662.2218 | 788.4820 | 61326.32 | 11 |  |  |  |  |  |
| 572 | 34 | 4309.2908 | 719.2224 | 46910.11 | 6 |  |  |  |  |  |
| 572 | 35 | 6968.4848 | 775.2834 | 37440.45 | 9 |  |  |  |  |  |
| 572 | 36 | 7669.7672 | 767.9840 | 36966.91 | 10 |  |  |  |  |  |
| 572 | 37 | 7770.5510 | 864.4018 | 34561.89 | 9 | 7769.5716 | C65 | 65 | -0.0230 | -2.96 |
| 572 | 38 | 801.3868 | 802.3941 | 431934.38 | 1 |  |  |  |  |  |
| 572 | 39 | 6209.6666 | 888.1025 | 27323.37 | 7 | 6209.6892 | C51 | 51 | -0.0226 | -3.64 |
| 572 | 40 | 8677.2332 | 789.8467 | 37400.16 | 11 |  |  |  |  |  |
| 572 | 41 | 6522.8335 | 932.8406 | 28321.55 | 7 | 6522.8530 | C54 | 54 | -0.0195 | -2.98 |
| 572 | 42 | 3982.1451 | 664.6981 | 30129.63 | 6 |  |  |  |  |  |
| 572 | 43 | 4886.4824 | 815.4210 | 26012.73 | 6 |  |  |  |  |  |
| 572 | 44 | 8210.8656 | 913.3257 | 30487.07 | 9 | 8210.8779 | C69 | 69 | -0.0124 | -1.51 |
| 572 | 45 | 4770.0842 | 955.0241 | 29636.24 | 5 | 4770.0923 | C39 | 39 | -8.11e-03 | -1.70 |
| 572 | 46 | 6623.8862 | 828.9931 | 31779.97 | 8 | 6623.9007 | C55 | 55 | -0.0145 | -2.18 |
| 572 | 47 | 5503.3478 | 918.2319 | 23527.50 | 6 | 5503.3559 | C45 | 45 | -8.16e-03 | -1.48 |
| 572 | 48 | 8059.9801 | 807.0053 | 52506.99 | 10 |  |  |  |  |  |
| 572 | 49 | 2202.0494 | 735.0237 | 764342.39 | 3 |  |  |  |  |  |
| 572 | 50 | 6793.9838 | 850.2553 | 31930.39 | 8 | 6794.0062 | C57 | 57 | -0.0224 | -3.29 |
| 572 | 51 | 7668.7646 | 853.0922 | 24428.62 | 9 |  |  |  |  |  |
| 572 | 52 | 8753.2079 | 973.5859 | 23499.74 | 9 | 8752.2328 | C74 | 74 | -0.0273 | -3.12 |
| 572 | 53 | 8682.1443 | 965.6900 | 20758.59 | 9 |  |  |  |  |  |
| 572 | 54 | 8618.1872 | 862.8260 | 26415.09 | 10 |  |  |  |  |  |
| 572 | 55 | 6694.3875 | 744.8281 | 26824.09 | 9 |  |  |  |  |  |
| 572 | 56 | 8324.8987 | 925.9960 | 24413.89 | 9 | 8324.9209 | C70 | 70 | -0.0222 | -2.66 |
| 572 | 57 | 8662.1971 | 867.2270 | 32839.84 | 10 |  |  |  |  |  |
| 572 | 58 | 7058.4881 | 785.2837 | 23778.99 | 9 | 7058.4790 | Z\_DOT61 | 14 | 9.03e-03 | 1.28 |
| 572 | 59 | 8450.0817 | 769.1965 | 32386.37 | 11 |  |  |  |  |  |
| 572 | 60 | 4527.3871 | 755.5718 | 32639.82 | 6 |  |  |  |  |  |
| 572 | 61 | 7438.6432 | 827.5232 | 24605.39 | 9 | 7438.6598 | Z\_DOT64 | 11 | -0.0166 | -2.24 |
| 572 | 62 | 5910.9285 | 845.4256 | 21027.61 | 7 |  |  |  |  |  |
| 572 | 63 | 7884.8186 | 789.4891 | 22965.82 | 10 |  |  |  |  |  |
| 572 | 64 | 6855.4393 | 857.9372 | 28352.82 | 8 |  |  |  |  |  |
| 572 | 65 | 7339.6449 | 816.5234 | 24788.71 | 9 |  |  |  |  |  |
| 572 | 66 | 7324.2819 | 814.8164 | 20286.69 | 9 | 7324.3027 | C61 | 61 | -0.0208 | -2.84 |
| 572 | 67 | 6694.4008 | 837.8074 | 17203.39 | 8 |  |  |  |  |  |
| 572 | 68 | 734.8404 | 735.8477 | 184355.33 | 1 |  |  |  |  |  |
| 572 | 69 | 8649.1717 | 865.9244 | 19420.12 | 10 |  |  |  |  |  |
| 572 | 70 | 8267.8681 | 919.6593 | 16072.97 | 9 |  |  |  |  |  |
| 572 | 71 | 1603.1260 | 802.5703 | 520726.07 | 2 |  |  |  |  |  |
| 572 | 72 | 7724.7759 | 859.3157 | 22868.53 | 9 |  |  |  |  |  |
| 572 | 73 | 4367.3275 | 728.8952 | 15919.59 | 6 | 4367.3128 | Z\_DOT39 | 36 | 0.0147 | 3.36 |
| 572 | 74 | 7826.5821 | 870.6275 | 34772.53 | 9 | 7826.5930 | C66 | 66 | -0.0110 | -1.40 |
| 572 | 75 | 4554.9549 | 911.9982 | 21913.04 | 5 |  |  |  |  |  |
| 572 | 76 | 4299.8726 | 860.9818 | 18428.44 | 5 | 4299.8798 | C34 | 34 | -7.18e-03 | -1.67 |
| 572 | 77 | 8779.1899 | 878.9263 | 20949.99 | 10 |  |  |  |  |  |
| 572 | 78 | 7477.3683 | 935.6783 | 15030.61 | 8 |  |  |  |  |  |
| 572 | 79 | 8003.0044 | 801.3077 | 42105.58 | 10 |  |  |  |  |  |
| 572 | 80 | 6240.1046 | 781.0203 | 17872.08 | 8 |  |  |  |  |  |
| 572 | 81 | 8353.0219 | 836.3095 | 25416.28 | 10 |  |  |  |  |  |
| 572 | 82 | 4440.3472 | 741.0651 | 15243.99 | 6 |  |  |  |  |  |
| 572 | 83 | 6284.1261 | 786.5230 | 14913.15 | 8 | 6283.1573 | Z\_DOT55 | 20 | -0.0336 | -5.34 |
| 572 | 84 | 6082.6220 | 869.9533 | 37246.66 | 7 | 6081.6306 | C50 | 50 | -0.0110 | -1.81 |
| 572 | 85 | 8625.1200 | 959.3539 | 14798.23 | 9 | 8624.1378 | C73 | 73 | -0.0202 | -2.35 |
| 572 | 86 | 6065.6001 | 867.5216 | 15492.80 | 7 |  |  |  |  |  |
| 572 | 87 | 3317.5376 | 830.3917 | 13836.62 | 4 | 3317.5460 | C26 | 26 | -8.35e-03 | -2.52 |
| 572 | 88 | 3854.0881 | 643.3553 | 15158.73 | 6 |  |  |  |  |  |
| 572 | 89 | 4640.4188 | 774.4104 | 15152.63 | 6 | 4640.4089 | Z\_DOT42 | 33 | 9.91e-03 | 2.13 |
| 572 | 90 | 7553.6981 | 840.3071 | 14169.18 | 9 | 7552.7028 | Z\_DOT65 | 10 | -7.07e-03 | -0.94 |
| 572 | 91 | 3434.9484 | 687.9970 | 12540.26 | 5 |  |  |  |  |  |
| 572 | 92 | 8776.2434 | 732.3609 | 15780.96 | 12 |  |  |  |  |  |
| 572 | 93 | 6680.8940 | 836.1190 | 21697.46 | 8 | 6680.9221 | C56 | 56 | -0.0281 | -4.21 |
| 572 | 94 | 8718.2287 | 727.5263 | 17725.60 | 12 |  |  |  |  |  |
| 572 | 95 | 8563.1708 | 779.4773 | 21223.68 | 11 |  |  |  |  |  |
| 572 | 96 | 7057.4468 | 883.1881 | 16529.24 | 8 | 7058.4790 | Z\_DOT61 | 14 | -0.0299 | -4.24 |
| 572 | 97 | 8453.0097 | 940.2306 | 14142.62 | 9 | 8453.0158 | C71 | 71 | -6.15e-03 | -0.73 |
| 572 | 98 | 7307.2596 | 914.4147 | 14480.65 | 8 |  |  |  |  |  |
| 572 | 99 | 5757.4993 | 823.5072 | 32794.04 | 7 | 5756.5098 | C47 | 47 | -0.0128 | -2.23 |
| 572 | 100 | 4055.7922 | 812.1657 | 14517.03 | 5 | 4055.8103 | C32 | 32 | -0.0181 | -4.47 |
| 572 | 101 | 4511.3676 | 752.9019 | 16100.15 | 6 | 4511.3663 | Z\_DOT41 | 34 | 1.24e-03 | 0.27 |
| 572 | 102 | 5563.7378 | 928.2969 | 12039.17 | 6 |  |  |  |  |  |
| 572 | 103 | 8225.9580 | 915.0026 | 16998.12 | 9 |  |  |  |  |  |
| 572 | 104 | 2671.5960 | 668.9063 | 16260.43 | 4 |  |  |  |  |  |
| 572 | 105 | 8581.1002 | 954.4629 | 17599.61 | 9 | 8581.1108 | C72 | 72 | -0.0106 | -1.23 |
| 572 | 106 | 8695.1736 | 967.1377 | 12801.99 | 9 |  |  |  |  |  |
| 572 | 107 | 7265.2543 | 909.1641 | 15015.95 | 8 |  |  |  |  |  |
| 572 | 108 | 6023.0092 | 861.4372 | 15234.84 | 7 | 6023.0048 | Z\_DOT53 | 22 | 4.43e-03 | 0.74 |
| 572 | 109 | 6506.8062 | 814.3580 | 11443.09 | 8 |  |  |  |  |  |
| 572 | 110 | 7280.2704 | 911.0411 | 10812.45 | 8 |  |  |  |  |  |
| 572 | 111 | 5032.5517 | 719.9432 | 11760.93 | 7 |  |  |  |  |  |
| 572 | 112 | 4170.8364 | 835.1746 | 15486.99 | 5 | 4170.8372 | C33 | 33 | -8.39e-04 | -0.20 |
| 572 | 113 | 6663.8837 | 952.9907 | 9160.63 | 7 |  |  |  |  |  |
| 572 | 114 | 6794.9815 | 971.7189 | 11142.64 | 7 | 6794.0062 | C57 | 57 | -0.0270 | -3.97 |
| 572 | 115 | 6283.1457 | 898.5995 | 16927.67 | 7 | 6283.1573 | Z\_DOT55 | 20 | -0.0116 | -1.85 |
| 572 | 116 | 7381.3028 | 923.6701 | 11524.14 | 8 | 7381.3242 | C62 | 62 | -0.0214 | -2.90 |
| 572 | 117 | 6170.0714 | 772.2662 | 11289.71 | 8 | 6170.0732 | Z\_DOT54 | 21 | -1.81e-03 | -0.29 |
| 572 | 118 | 7393.6575 | 822.5248 | 13202.34 | 9 |  |  |  |  |  |
| 572 | 119 | 8166.9203 | 908.4429 | 11228.86 | 9 |  |  |  |  |  |
| 572 | 120 | 7652.7365 | 766.2809 | 11521.78 | 10 | 7653.7505 | Z\_DOT66 | 9 | -0.0116 | -1.52 |
| 572 | 121 | 5945.5555 | 850.3723 | 14241.34 | 7 | 5944.5717 | C49 | 49 | -0.0186 | -3.12 |
| 572 | 122 | 6191.6408 | 774.9624 | 11502.15 | 8 |  |  |  |  |  |
| 572 | 123 | 8563.1628 | 857.3236 | 12179.14 | 10 |  |  |  |  |  |
| 572 | 124 | 1372.5838 | 687.2992 | 17028.24 | 2 | 1372.5863 | C11 | 11 | -2.54e-03 | -1.85 |
| 572 | 125 | 832.5896 | 833.5969 | 6744.72 | 1 |  |  |  |  |  |
| 572 | 126 | 1169.7826 | 585.8986 | 6940.10 | 2 |  |  |  |  |  |
| 572 | 127 | 997.4633 | 998.4706 | 5636.87 | 1 | 997.4651 | C8 | 8 | -1.75e-03 | -1.75 |
| 572 | 128 | 1428.8893 | 477.3037 | 4476.96 | 3 |  |  |  |  |  |
| 572 | 129 | 1308.7943 | 655.4044 | 3773.02 | 2 |  |  |  |  |  |
| 572 | 130 | 860.9192 | 861.9265 | 3418.68 | 1 |  |  |  |  |  |
| 572 | 131 | 896.2292 | 897.2365 | 3161.41 | 1 |  |  |  |  |  |
| 572 | 132 | 952.1261 | 953.1334 | 1925.83 | 1 |  |  |  |  |  |
| 572 | 133 | 1386.8797 | 463.3005 | 2117.70 | 3 |  |  |  |  |  |
| 572 | 134 | 933.5508 | 934.5580 | 1146.04 | 1 |  |  |  |  |  |
| 572 | 135 | 542.3174 | 543.3246 | 1800.49 | 1 |  |  |  |  |  |
| 572 | 136 | 1486.9545 | 496.6588 | 2277.82 | 3 | 1486.9435 | Z\_DOT14 | 61 | 0.0111 | 7.43 |
| 572 | 137 | 602.3202 | 603.3275 | 3028.67 | 1 | 602.3210 | C5 | 5 | -7.26e-04 | -1.20 |
| 572 | 138 | 916.6630 | 917.6702 | 4571.22 | 1 |  |  |  |  |  |
| 572 | 139 | 1185.8018 | 593.9082 | 1574.90 | 2 |  |  |  |  |  |
| 572 | 140 | 814.8091 | 815.8163 | 1417.13 | 1 |  |  |  |  |  |
| 572 | 141 | 798.5064 | 400.2605 | 1441.48 | 2 |  |  |  |  |  |
| 572 | 142 | 1013.9491 | 1014.9564 | 1497.52 | 1 |  |  |  |  |  |
| 572 | 143 | 1057.7067 | 529.8606 | 1980.72 | 2 |  |  |  |  |  |
| 572 | 144 | 1415.8826 | 472.9681 | 1091.69 | 3 |  |  |  |  |  |
| 572 | 145 | 983.6214 | 492.8180 | 1449.31 | 2 |  |  |  |  |  |
| 572 | 146 | 1169.7816 | 390.9345 | 989.52 | 3 |  |  |  |  |  |

  

All proteins /
CsTx-1a\_S1 Cupiennius salei toxin 1 isoform a S1^ACsTx-1a\_S2 Cupiennius salei toxin 1 isoform a S2 /
Proteoform #6
