# Supplementary material for: The Dual Prey-Inactivation Strategy of Spiders—In-Depth Venomic Analysis of Cupiennius salei
Source: Toxins (Basel). 2019 Mar 19;11(3):167. doi: 10.3390/toxins11030167 (PMC6468893; doi:10.3390/toxins11030167)
Supplement: Supplementary file 1 [file toxins-11-00167-s001.zip › Supplementary Dataset EV1/20180328_f2_topdown_OTMS2_EThcD_NL_i02_ms2_proteoform_cutoff_html/prsms/prsm188.html]

Protein-Spectrum-Match for Spectrum #428


All proteins /
CsTx-1a\_S1 Cupiennius salei toxin 1 isoform a S1^ACsTx-1a\_S2 Cupiennius salei toxin 1 isoform a S2 /
Proteoform #6

## Protein-Spectrum-Match #188 for Spectrum #428

|  |  |  |  |  |  |
| --- | --- | --- | --- | --- | --- |
| PrSM ID: | 188 | Scan(s): | 573 | Precursor charge: | 11 |
| Precursor m/z: | 801.9385 | Precursor mass: | 8810.2437 | Proteoform mass: | 8810.2337 |
| # matched peaks: | 51 | # matched fragment ions: | 37 | # unexpected modifications: | 1 |
| E-value: | 1.32e-31 | P-value: | 1.32e-31 | Q-value (Spectral FDR): | 0 |

  

|  |  |  |  |  |  |  |  |  |  |  |  |  |  |  |  |  |  |  |  |  |  |  |  |  |  |  |  |  |  |  |  |  |  |  |  |  |  |  |  |  |  |  |  |  |  |  |  |  |  |  |  |  |  |  |  |  |  |  |  |  |  |  |  |  |  |  |  |  |  |
| --- | --- | --- | --- | --- | --- | --- | --- | --- | --- | --- | --- | --- | --- | --- | --- | --- | --- | --- | --- | --- | --- | --- | --- | --- | --- | --- | --- | --- | --- | --- | --- | --- | --- | --- | --- | --- | --- | --- | --- | --- | --- | --- | --- | --- | --- | --- | --- | --- | --- | --- | --- | --- | --- | --- | --- | --- | --- | --- | --- | --- | --- | --- | --- | --- | --- | --- | --- | --- | --- |
|  | |  | | | | | | | | | | | | | | | | | | | | | | | | | | | | | | | | | | | | | | | | | | | | | | | | | | | | | | | | | | | | | | | | | | | |
| 1 |  |  | M |  | K |  | V |  | L |  | I |  | I |  | S |  | A |  | V |  | L |  |  | F |  | I |  | T |  | I |  | F |  | S |  | N |  | I |  | S |  | A |  |  | E |  | I |  | E |  | D |  | D |  | F |  | L |  | E |  | D |  | E |  | 30 |  |
|  | |  | | | | | | | | | | | | | | | | | | | | | | | | | | | | | | | | | | | | | | | | | | | | | | | | | | | | | | | | | | | | | | | | | | | |
| 31 |  |  | S |  | F |  | E |  | A |  | E |  | D |  | I |  | I |  | P |  | F |  |  | F |  | E |  | N |  | E |  | Q |  | A |  | R | ] | S |  | C |  | I |  |  | P | ⎫ | K | ⎫ | H |  | E |  | E | ⎫ | C |  | T |  | N | ⎱ | D |  | K |  | 60 |  |
|  | |  | | | | | | | | | | | | | | | | | | | | | | | | | | | | | | | | | | | | | | | | | | | | | | | | | | | | | | | | | | | | | | | | | | | |
| 61 |  |  | H | ⎩ | N | ⎫ | C |  | C |  | R |  | K |  | G |  | L |  | F | ⎱ | K |  |  | L |  | K | ⎫ | C |  | Q | ⎫ | C |  | S |  | T |  | F | ⎫ | D |  | D |  | ⎩ | E | ⎩ | S |  | G | ⎱ | Q |  | P |  | T | ⎩ | E |  | R |  | C |  | A |  | 90 |  |
|  | |  | | | | | | | | | | | | | | | | | | | | | | | | | | | | | | | | | | | | | | | | | | | | | | | | | | | | | -58.03 | | | | | | | | | | | |
| 91 |  |  | C |  | G | ⎱ | R |  | P | ⎫ | M | ⎫ | G | ⎫ | H | ⎱ | Q | ⎫ | A |  | I |  |  | E | ⎱ | T | ⎫ | G |  | L |  | N |  | I |  | F |  | R | ⎫ | G |  | L |  |  | F |  | K |  | G |  | K | ⎫ | K | ⎫ | K | ⎫ | N | ⎫ | K | ⎫ | K | ⎫ | T |  | 120 |  |
|  | |  | | | | | | | | | | | | | | | | | | | | | | | | | | | | | | | | | | | | | | | | | | | | | | | | | | | | | | | | | | | | | | | | | | | |
| 121 |  | ⎫ | K | ⎫ | G |  | | | | 122 |  | | | | | | | | | | | | | | | | | | | | | | | | | | | | | | | | | | | | | | | | | | | | | | | | | | | | | | | |

Fixed PTMs: Carbamidomethylation [C49 C56 C63 C64 C73 C75 C89 C91 ]   
  
     Unexpected modifications:   Unknown [-58.03]

  

All peaks (146)  Matched peaks (51)  Not matched peaks (95)

  

| Scan | Peak | Mono mass | Mono m/z | Intensity | Charge | Theoretical mass | Ion | Pos | Mass error | PPM error |
| --- | --- | --- | --- | --- | --- | --- | --- | --- | --- | --- |
| 573 | 1 | 8753.2079 | 876.3281 | 545928.94 | 10 | 8752.2282 | C74 | 74 | -0.0226 | -2.58 |
| 573 | 2 | 8753.2022 | 973.5853 | 427313.93 | 9 | 8752.2282 | C74 | 74 | -0.0283 | -3.23 |
| 573 | 3 | 8795.1991 | 978.2516 | 163329.40 | 9 |  |  |  |  |  |
| 573 | 4 | 8767.2021 | 975.1408 | 164920.18 | 9 |  |  |  |  |  |
| 573 | 5 | 8794.2028 | 880.4276 | 160052.90 | 10 |  |  |  |  |  |
| 573 | 6 | 8766.1981 | 877.6271 | 155730.59 | 10 |  |  |  |  |  |
| 573 | 7 | 4405.6099 | 882.1293 | 494564.82 | 5 |  |  |  |  |  |
| 573 | 8 | 8737.1978 | 874.7271 | 116855.36 | 10 |  |  |  |  |  |
| 573 | 9 | 8695.1802 | 967.1384 | 104698.49 | 9 |  |  |  |  |  |
| 573 | 10 | 8753.2001 | 1095.1573 | 87402.95 | 8 | 8752.2282 | C74 | 74 | -0.0304 | -3.47 |
| 573 | 11 | 8737.1847 | 971.8056 | 104793.73 | 9 |  |  |  |  |  |
| 573 | 12 | 8682.1372 | 965.6892 | 91890.18 | 9 |  |  |  |  |  |
| 573 | 13 | 8709.1839 | 968.6944 | 85192.99 | 9 |  |  |  |  |  |
| 573 | 14 | 8324.8855 | 925.9946 | 88460.99 | 9 |  |  |  |  |  |
| 573 | 15 | 8210.8474 | 913.3237 | 42575.78 | 9 |  |  |  |  |  |
| 573 | 16 | 2938.0764 | 980.3661 | 139299.14 | 3 |  |  |  |  |  |
| 573 | 17 | 8452.9767 | 940.2269 | 40313.63 | 9 |  |  |  |  |  |
| 573 | 18 | 8224.9242 | 914.8877 | 44195.58 | 9 |  |  |  |  |  |
| 573 | 19 | 5910.9174 | 845.4240 | 46062.24 | 7 |  |  |  |  |  |
| 573 | 20 | 8721.2085 | 873.1281 | 46884.40 | 10 |  |  |  |  |  |
| 573 | 21 | 8082.7520 | 1011.3513 | 41308.05 | 8 | 8082.7830 | C68 | 68 | -0.0310 | -3.83 |
| 573 | 22 | 5910.9185 | 986.1604 | 38314.73 | 6 |  |  |  |  |  |
| 573 | 23 | 8721.2062 | 970.0302 | 43900.86 | 9 |  |  |  |  |  |
| 573 | 24 | 8737.1937 | 1093.1565 | 42615.39 | 8 |  |  |  |  |  |
| 573 | 25 | 8082.7436 | 899.0899 | 36569.57 | 9 | 8082.7830 | C68 | 68 | -0.0394 | -4.87 |
| 573 | 26 | 6854.4159 | 857.8093 | 33903.34 | 8 |  |  |  |  |  |
| 573 | 27 | 8581.0866 | 954.4613 | 41438.11 | 9 |  |  |  |  |  |
| 573 | 28 | 8709.1810 | 1089.6549 | 33595.43 | 8 |  |  |  |  |  |
| 573 | 29 | 8662.1954 | 867.2268 | 34378.57 | 10 |  |  |  |  |  |
| 573 | 30 | 5503.3401 | 918.2306 | 35483.97 | 6 | 5503.3559 | C45 | 45 | -0.0158 | -2.88 |
| 573 | 31 | 8795.2089 | 1100.4084 | 29965.08 | 8 |  |  |  |  |  |
| 573 | 32 | 7723.7620 | 859.2031 | 37412.17 | 9 |  |  |  |  |  |
| 573 | 33 | 7059.4733 | 1009.5035 | 30320.29 | 7 | 7058.4744 | Z\_DOT61 | 14 | -3.44e-03 | -0.49 |
| 573 | 34 | 8325.8950 | 1041.7441 | 38697.11 | 8 |  |  |  |  |  |
| 573 | 35 | 4443.9115 | 889.7896 | 29042.63 | 5 | 4443.9333 | C36 | 36 | -0.0218 | -4.91 |
| 573 | 36 | 8768.2093 | 1097.0334 | 30338.34 | 8 |  |  |  |  |  |
| 573 | 37 | 8663.1847 | 963.5834 | 31666.61 | 9 |  |  |  |  |  |
| 573 | 38 | 8696.1746 | 1088.0291 | 35442.15 | 8 |  |  |  |  |  |
| 573 | 39 | 7954.6567 | 995.3394 | 37048.33 | 8 | 7954.6880 | C67 | 67 | -0.0313 | -3.93 |
| 573 | 40 | 8266.8618 | 1034.3650 | 28539.91 | 8 | 8266.8956 | C70 | 70 | -0.0338 | -4.09 |
| 573 | 41 | 7954.6314 | 884.8552 | 33691.05 | 9 | 7954.6880 | C67 | 67 | -0.0566 | -7.12 |
| 573 | 42 | 7829.7956 | 870.9846 | 32381.18 | 9 |  |  |  |  |  |
| 573 | 43 | 8793.2328 | 800.3921 | 24817.14 | 11 |  |  |  |  |  |
| 573 | 44 | 8681.1400 | 1086.1498 | 25732.37 | 8 |  |  |  |  |  |
| 573 | 45 | 8210.8530 | 1027.3639 | 28916.92 | 8 |  |  |  |  |  |
| 573 | 46 | 6694.3881 | 837.8058 | 23901.08 | 8 |  |  |  |  |  |
| 573 | 47 | 8694.1577 | 870.4230 | 29447.42 | 10 |  |  |  |  |  |
| 573 | 48 | 8618.1752 | 958.5823 | 27041.48 | 9 |  |  |  |  |  |
| 573 | 49 | 8705.2140 | 871.5287 | 40714.03 | 10 |  |  |  |  |  |
| 573 | 50 | 7438.6476 | 930.8382 | 23592.14 | 8 | 7438.6552 | Z\_DOT64 | 11 | -7.66e-03 | -1.03 |
| 573 | 51 | 4309.2816 | 719.2209 | 26210.59 | 6 |  |  |  |  |  |
| 573 | 52 | 801.2970 | 802.3043 | 291796.74 | 1 |  |  |  |  |  |
| 573 | 53 | 4309.2824 | 862.8638 | 25643.42 | 5 |  |  |  |  |  |
| 573 | 54 | 5944.5473 | 991.7652 | 25670.61 | 6 | 5944.5717 | C49 | 49 | -0.0245 | -4.11 |
| 573 | 55 | 8582.0726 | 1073.7664 | 19897.31 | 8 |  |  |  |  |  |
| 573 | 56 | 6522.8288 | 932.8400 | 21683.57 | 7 | 6522.8530 | C54 | 54 | -0.0242 | -3.70 |
| 573 | 57 | 5503.3379 | 1101.6749 | 22498.16 | 5 | 5503.3559 | C45 | 45 | -0.0180 | -3.27 |
| 573 | 58 | 4443.9172 | 1111.9866 | 17905.32 | 4 | 4443.9333 | C36 | 36 | -0.0161 | -3.63 |
| 573 | 59 | 6082.6186 | 869.9528 | 30165.79 | 7 | 6081.6306 | C50 | 50 | -0.0144 | -2.37 |
| 573 | 60 | 3157.5049 | 790.3835 | 19893.78 | 4 | 3157.5153 | C25 | 25 | -0.0104 | -3.30 |
| 573 | 61 | 3307.8938 | 827.9807 | 27711.07 | 4 | 3307.8856 | Z\_DOT30 | 45 | 8.23e-03 | 2.49 |
| 573 | 62 | 8452.9897 | 1057.6310 | 17129.14 | 8 |  |  |  |  |  |
| 573 | 63 | 8649.1760 | 962.0268 | 19636.66 | 9 |  |  |  |  |  |
| 573 | 64 | 8223.9233 | 1028.9977 | 21397.13 | 8 |  |  |  |  |  |
| 573 | 65 | 4367.3217 | 874.4716 | 25790.31 | 5 | 4367.3082 | Z\_DOT39 | 36 | 0.0135 | 3.08 |
| 573 | 66 | 5563.7433 | 928.2978 | 31039.45 | 6 |  |  |  |  |  |
| 573 | 67 | 2288.3994 | 763.8071 | 19247.06 | 3 | 2288.3886 | Z\_DOT21 | 54 | 0.0108 | 4.72 |
| 573 | 68 | 6968.4798 | 872.0673 | 22673.63 | 8 |  |  |  |  |  |
| 573 | 69 | 4406.1143 | 1102.5358 | 26512.26 | 4 |  |  |  |  |  |
| 573 | 70 | 7324.2774 | 1047.3326 | 21478.46 | 7 | 7324.3027 | C61 | 61 | -0.0253 | -3.45 |
| 573 | 71 | 7668.7427 | 959.6001 | 18870.72 | 8 |  |  |  |  |  |
| 573 | 72 | 7669.7543 | 853.2022 | 20067.68 | 9 |  |  |  |  |  |
| 573 | 73 | 5944.5485 | 850.2285 | 15149.80 | 7 | 5944.5717 | C49 | 49 | -0.0232 | -3.91 |
| 573 | 74 | 8677.2045 | 868.7277 | 21934.01 | 10 |  |  |  |  |  |
| 573 | 75 | 1169.7807 | 585.8976 | 25652.17 | 2 |  |  |  |  |  |
| 573 | 76 | 8153.8408 | 1020.2374 | 13952.20 | 8 | 8152.8527 | C69 | 69 | -0.0142 | -1.74 |
| 573 | 77 | 7324.2743 | 916.5416 | 20254.24 | 8 | 7324.3027 | C61 | 61 | -0.0284 | -3.88 |
| 573 | 78 | 2872.3139 | 958.4452 | 19178.46 | 3 |  |  |  |  |  |
| 573 | 79 | 6081.6061 | 1014.6083 | 23355.18 | 6 | 6081.6306 | C50 | 50 | -0.0246 | -4.04 |
| 573 | 80 | 2788.2304 | 930.4174 | 21195.53 | 3 | 2788.2414 | C22 | 22 | -0.0110 | -3.93 |
| 573 | 81 | 8625.1127 | 1079.1464 | 16193.95 | 8 | 8624.1332 | C73 | 73 | -0.0229 | -2.65 |
| 573 | 82 | 3445.5878 | 862.4042 | 15658.17 | 4 | 3445.6046 | C27 | 27 | -0.0168 | -4.88 |
| 573 | 83 | 6021.9962 | 861.2924 | 15288.51 | 7 | 6023.0002 | Z\_DOT53 | 22 | -1.62e-03 | -0.27 |
| 573 | 84 | 8167.9033 | 1021.9952 | 15612.09 | 8 |  |  |  |  |  |
| 573 | 85 | 8722.1933 | 1091.2814 | 14606.83 | 8 |  |  |  |  |  |
| 573 | 86 | 2729.6218 | 910.8812 | 14097.94 | 3 | 2729.6109 | Z\_DOT25 | 50 | 0.0109 | 4.00 |
| 573 | 87 | 3982.1465 | 797.4366 | 15061.84 | 5 |  |  |  |  |  |
| 573 | 88 | 3233.8702 | 809.4748 | 18626.53 | 4 |  |  |  |  |  |
| 573 | 89 | 4771.0768 | 955.2226 | 14749.23 | 5 |  |  |  |  |  |
| 573 | 90 | 5756.4830 | 960.4211 | 18931.93 | 6 | 5756.5098 | C47 | 47 | -0.0268 | -4.66 |
| 573 | 91 | 1866.8028 | 934.4087 | 17279.84 | 2 | 1866.8101 | C15 | 15 | -7.23e-03 | -3.87 |
| 573 | 92 | 3157.5060 | 1053.5093 | 13098.61 | 3 | 3157.5153 | C25 | 25 | -9.36e-03 | -2.96 |
| 573 | 93 | 6022.9988 | 1004.8404 | 15899.94 | 6 | 6023.0002 | Z\_DOT53 | 22 | -1.40e-03 | -0.23 |
| 573 | 94 | 3246.8887 | 812.7295 | 15873.04 | 4 |  |  |  |  |  |
| 573 | 95 | 3854.0804 | 643.3540 | 10790.93 | 6 |  |  |  |  |  |
| 573 | 96 | 3982.1392 | 664.6971 | 12210.77 | 6 |  |  |  |  |  |
| 573 | 97 | 8753.2035 | 1251.4649 | 9070.28 | 7 | 8752.2282 | C74 | 74 | -0.0270 | -3.09 |
| 573 | 98 | 7897.6142 | 988.2090 | 13332.13 | 8 |  |  |  |  |  |
| 573 | 99 | 8617.1936 | 862.7266 | 14743.49 | 10 |  |  |  |  |  |
| 573 | 100 | 6209.6499 | 888.1001 | 10282.63 | 7 | 6209.6892 | C51 | 51 | -0.0393 | -6.33 |
| 573 | 101 | 3940.7664 | 986.1989 | 13299.32 | 4 | 3940.7834 | C31 | 31 | -0.0169 | -4.29 |
| 573 | 102 | 1603.1269 | 802.5707 | 297477.64 | 2 |  |  |  |  |  |
| 573 | 103 | 6209.6631 | 1035.9511 | 17064.96 | 6 | 6209.6892 | C51 | 51 | -0.0262 | -4.21 |
| 573 | 104 | 6695.4179 | 957.4955 | 18347.44 | 7 |  |  |  |  |  |
| 573 | 105 | 7440.6603 | 1063.9588 | 7545.01 | 7 |  |  |  |  |  |
| 573 | 106 | 5887.5103 | 982.2590 | 10082.69 | 6 | 5887.5503 | C48 | 48 | -0.0400 | -6.79 |
| 573 | 107 | 6622.8671 | 947.1312 | 14190.19 | 7 | 6623.9007 | C55 | 55 | -0.0312 | -4.71 |
| 573 | 108 | 7910.6480 | 989.8383 | 13100.12 | 8 |  |  |  |  |  |
| 573 | 109 | 8522.0570 | 1066.2644 | 8207.31 | 8 | 8523.0855 | C72 | 72 | -0.0262 | -3.07 |
| 573 | 110 | 4511.3606 | 752.9007 | 7480.29 | 6 | 4511.3617 | Z\_DOT41 | 34 | -1.09e-03 | -0.24 |
| 573 | 111 | 4041.1514 | 809.2375 | 12521.48 | 5 | 4041.1492 | Z\_DOT36 | 39 | 2.16e-03 | 0.53 |
| 573 | 112 | 5275.6378 | 1056.1348 | 7428.29 | 5 |  |  |  |  |  |
| 573 | 113 | 8024.7178 | 1004.0970 | 11617.56 | 8 |  |  |  |  |  |
| 573 | 114 | 4255.2715 | 852.0616 | 9186.99 | 5 |  |  |  |  |  |
| 573 | 115 | 3506.8802 | 877.7273 | 35190.62 | 4 |  |  |  |  |  |
| 573 | 116 | 4886.4733 | 815.4195 | 11432.09 | 6 |  |  |  |  |  |
| 573 | 117 | 8395.9526 | 1050.5013 | 9849.74 | 8 | 8394.9906 | C71 | 71 | -0.0404 | -4.81 |
| 573 | 118 | 4554.9497 | 911.9972 | 10120.76 | 5 |  |  |  |  |  |
| 573 | 119 | 2671.5915 | 891.5378 | 13501.04 | 3 |  |  |  |  |  |
| 573 | 120 | 8282.8905 | 1036.3686 | 11493.94 | 8 |  |  |  |  |  |
| 573 | 121 | 2671.5923 | 668.9054 | 12642.55 | 4 |  |  |  |  |  |
| 573 | 122 | 4640.4056 | 774.4082 | 7793.28 | 6 | 4640.4043 | Z\_DOT42 | 33 | 1.35e-03 | 0.29 |
| 573 | 123 | 882.2194 | 883.2267 | 21062.04 | 1 |  |  |  |  |  |
| 573 | 124 | 7265.2448 | 1038.8994 | 9087.06 | 7 |  |  |  |  |  |
| 573 | 125 | 980.2480 | 981.2553 | 14513.13 | 1 |  |  |  |  |  |
| 573 | 126 | 1486.9541 | 744.4843 | 7978.85 | 2 |  |  |  |  |  |
| 573 | 127 | 728.4761 | 729.4833 | 8545.97 | 1 |  |  |  |  |  |
| 573 | 128 | 1372.5817 | 687.2981 | 7422.84 | 2 | 1372.5863 | C11 | 11 | -4.62e-03 | -3.36 |
| 573 | 129 | 856.5709 | 857.5782 | 12287.74 | 1 |  |  |  |  |  |
| 573 | 130 | 997.4583 | 998.4656 | 6543.86 | 1 | 997.4651 | C8 | 8 | -6.76e-03 | -6.77 |
| 573 | 131 | 602.3194 | 603.3267 | 7683.85 | 1 | 602.3210 | C5 | 5 | -1.58e-03 | -2.62 |
| 573 | 132 | 1372.5820 | 1373.5893 | 2415.83 | 1 | 1372.5863 | C11 | 11 | -4.30e-03 | -3.13 |
| 573 | 133 | 1316.8521 | 659.4333 | 3623.40 | 2 |  |  |  |  |  |
| 573 | 134 | 1033.6068 | 1034.6141 | 2217.21 | 1 |  |  |  |  |  |
| 573 | 135 | 1185.7991 | 593.9068 | 3745.69 | 2 |  |  |  |  |  |
| 573 | 136 | 916.3299 | 917.3372 | 2694.99 | 1 |  |  |  |  |  |
| 573 | 137 | 894.4354 | 895.4427 | 4174.54 | 1 |  |  |  |  |  |
| 573 | 138 | 1098.7073 | 550.3609 | 2266.97 | 2 |  |  |  |  |  |
| 573 | 139 | 1111.7150 | 556.8648 | 2371.30 | 2 |  |  |  |  |  |
| 573 | 140 | 1046.4698 | 1047.4771 | 2413.87 | 1 |  |  |  |  |  |
| 573 | 141 | 1428.8866 | 477.3028 | 2096.20 | 3 |  |  |  |  |  |
| 573 | 142 | 1151.7464 | 576.8805 | 2160.33 | 2 |  |  |  |  |  |
| 573 | 143 | 474.2248 | 475.2321 | 2367.45 | 1 | 474.2260 | C4 | 4 | -1.22e-03 | -2.58 |
| 573 | 144 | 502.2434 | 503.2507 | 2908.45 | 1 |  |  |  |  |  |
| 573 | 145 | 428.2736 | 429.2809 | 3924.79 | 1 |  |  |  |  |  |
| 573 | 146 | 1060.7602 | 1061.7675 | 1845.08 | 1 |  |  |  |  |  |

  

All proteins /
CsTx-1a\_S1 Cupiennius salei toxin 1 isoform a S1^ACsTx-1a\_S2 Cupiennius salei toxin 1 isoform a S2 /
Proteoform #6
